# A Trypanosoma brucei ORFeome-Based Gain-of-Function Library Identifies Genes That Promote Survival during Melarsoprol Treatment

**DOI:** 10.1128/mSphere.00769-20

**Published:** 2020-10-07

**Authors:** McKenzie Carter, Stephanie Gomez, Sam Gritz, Stephen Larson, Eugenia Silva-Herzog, Hee-Sook Kim, Danae Schulz, Galadriel Hovel-Miner

**Affiliations:** a The George Washington University, Department of Microbiology, Immunology, and Tropical Medicine, Washington, DC, USA; b Instituto Nacional de Medicina Genomica, Mexico City, Mexico; c The Public Health Research Institute at the International Center for Public Health, New Jersey Medical School—Rutgers, The State University of New Jersey, Newark, New Jersey, USA; d Harvey Mudd College, F.W.Olin Science Center, Claremont, California, USA; University of Texas Southwestern

**Keywords:** forward genetics, ORFeome, *Trypanosoma*, drug resistance mechanisms, parasitology, redox signaling

## Abstract

Trypanosoma brucei is an early branching protozoan parasite that causes human and animal African trypanosomiasis. Forward genetics approaches are powerful tools for uncovering novel aspects of trypanosomatid biology, pathogenesis, and therapeutic approaches against trypanosomiasis. Here, we have generated a T. brucei cloned ORFeome consisting of >90% of the targeted 7,245 genes and used it to make an inducible gain-of-function parasite library broadly applicable to large-scale forward genetic screens. We conducted a proof-of-principle genetic screen to identify genes whose expression promotes survival in melarsoprol, a critical drug of last resort. The 57 genes identified as overrepresented in melarsoprol survivor populations included the gene encoding the rate-limiting enzyme for the biosynthesis of an established drug target (trypanothione), validating the tool. In addition, novel genes associated with gene expression, flagellum localization, and mitochondrion localization were identified, and a subset of those genes increased melarsoprol resistance upon overexpression in culture. These findings offer new insights into trypanosomatid basic biology, implications for drug targets, and direct or indirect drug resistance mechanisms. This study generated a T. brucei ORFeome and gain-of-function parasite library, demonstrated the library’s usefulness in forward genetic screening, and identified novel aspects of melarsoprol resistance that will be the subject of future investigations. These powerful genetic tools can be used to broadly advance trypanosomatid research.

**IMPORTANCE** Trypanosomatid parasites threaten the health of more than 1 billion people worldwide. Because their genomes are highly diverged from those of well-established eukaryotes, conservation is not always useful in assigning gene functions. However, it is precisely among the trypanosomatid-specific genes that ideal therapeutic targets might be found. Forward genetics approaches are an effective way to identify novel gene functions. We used an ORFeome approach to clone a large percentage of Trypanosoma brucei genes and generate a gain-of-function parasite library. This library was used in a genetic screen to identify genes that promote resistance to the clinically significant yet highly toxic drug melarsoprol. Hits arising from the screen demonstrated the library’s usefulness in identifying known pathways and uncovered novel aspects of resistance mediated by proteins localized to the flagellum and mitochondrion. The powerful new genetic tools generated herein are expected to promote advances in trypanosomatid biology and therapeutic development in the years to come.

## Abstract (precis)

Trypanosomatid parasites threaten the health of more than 1 billion people worldwide. Because their genomes are highly diverged from those of well-established eukaryotes, conservation is not always useful in assigning gene functions. However, it is precisely among the trypanosomatid-specific genes that ideal therapeutic targets might be found. Forward genetics approaches are an effective way to identify novel gene functions. We used an ORFeome approach to clone a large percentage of Trypanosoma brucei genes and generate a gain-of-function parasite library. This library was used in a genetic screen to identify genes that promote resistance to the clinically significant yet highly toxic drug melarsoprol. Hits arising from the screen demonstrated the library’s usefulness in identifying known pathways and uncovered novel aspects of resistance mediated by proteins localized to the flagellum and mitochondrion. The powerful new genetic tools generated herein are expected to promote advances in trypanosomatid biology and therapeutic development in the years to come.

## INTRODUCTION

Trypanosomatids are a major parasitic lineage that include the African trypanosomes, American trypanosomes, and *Leishmania* spp. (family Trypanosomatidae, order Kinetoplastida), which collectively cause death and disease in millions of people living in tropical and subtropical regions (1). There are no vaccines against this family of parasites, and the limited number of antitrypanosomatid drugs present ongoing challenges of host toxicity, complex treatment regimens, and burgeoning drug resistance (2).

Trypanosomatid parasites appear to have diverged from a shared ancestor around 100 million years ago. These early branching eukaryotes have highly divergent genomes from those of well-established model organisms, with more than 35% of open reading frames (ORFs) annotated as hypothetical proteins (3). Of the 9,068 genes in the Trypanosoma brucei (African trypanosome) genome, 6,158 are orthologous with both Trypanosoma cruzi (American trypanosome) and Leishmania major (3). While reverse genetics based on well-established models can promote discrete advances, forward genetics approaches have the potential to uncover important aspects of trypanosomatid biology shared among orthologous genes.

T. brucei, the causative agent of human African trypanosomiasis (HAT), has historically been the most genetically tractable of the trypanosomatid parasites. For the past decade, a whole-genome RNA interference (RNAi) knockdown library has been the primary forward genetics tool in T. brucei, resulting in the identification of essential genes, genes associated with drug resistance and pathogenesis, and signaling factors critical to life cycle progression, to name a few (4–9). A strength of the RNAi library and associated RNA interference targeted sequencing (RIT-seq) approaches is the identification of genes that result in a loss-of-function phenotype (10). However, the RNAi library has some limitations. First, if the target of a genetic screen happens to be essential, it is difficult to identify using an RNAi screen. Second, while the RNAi library has been used to identify proteins involved in drug uptake (11) and activation (12), it cannot be used to identify the molecular target of drugs that selectively kill the parasite and not the host, since the molecular target is, by definition, essential in the parasite.

A gain-of-function library approach may be more effective in the identification of drug targets and resistance mechanisms (13–15). For example, overexpression of the molecular target can act as a sink, effectively mopping up the drug and promoting survival during drug treatment. This could be especially useful for identifying targets of inhibitors that are still in development (16) and has recently been used to identify a target of the antimalarial drug risedronate (17) and another antimalarial proteasome inhibitor (18). In basic biology, overexpression screens have been critical for discoveries in the areas of chromosome segregation, cell cycle, signal transduction, transcriptional regulation, cell polarity, and stem cell biology (19).

Traditional methods of overexpression library formation by cDNA synthesis and cloning are not viable for T. brucei, as most gene expression regulation in trypanosomatids occurs posttranscriptionally, with 5′ and 3′ untranslated regions (UTRs) playing a major role in determining steady-state levels of their associated transcripts (20). Existing T. brucei overexpression libraries generated by physical or enzymatic whole-genome fragmentation have generated useful results but lack the ability to ensure complete ORF integration and can include unwanted regulatory elements (21–24). In addition, random shotgun libraries can be used to identify a protein region required for a particular phenotype, but they are limited by the fact that partial proteins are not always folded properly and that the entire protein may be required for function, which can produce false positives and false negatives (25). In trypanosomatids, increased gene expression has been linked to drug resistance in *Leishmania* spp. through episomal cosmid amplification (25) and in T. brucei *in vitro* when enzymes of trypanothione biosynthesis are overexpressed (21, 26). ORFeome-based approaches, in which all ORFs in the genome are cloned for downstream applications, are powerful tools for the specific evaluation of gene effects whose proximal regulatory elements are excluded (27, 28). In addition, generation of an ORFeome can be applied to the downstream generation of multiple whole-genome methodologies, including yeast 2-hybrid libraries, tagging libraries, and inducible expression libraries for gain-of-function studies (29–32).

In this study, we have taken an ORFeome-based approach to generate a T. brucei gain-of-function library for forward genetic screens. Melarsoprol was selected for a proof-of-principle genetic screen for its clinical significance, the probability that it affects multiple intracellular targets, and because its mode of cell killing is not completely understood (33, 34). Melarsoprol, an arsenical compound, has long been used for the treatment of second-stage (central nervous system) T. brucei infection (33). Second-stage HAT infections caused by T. brucei subsp. gambiense can now be treated by nifurtimox/eflornithine combination therapy (NECT) and the recently approved drug fexinidazole (2, 35). However, melarsoprol remains the only treatment for second-stage T. brucei subsp. *rhodesiense* infection, which rapidly progresses toward host death if left untreated. Melarsoprol treatment is burdened with high levels of host toxicity, challenging treatment regimens, and increasing reports of drug resistance and treatment failures (33). Melarsoprol is taken up into the cell by the P2 adenosine transporter (AT1) and aquaglyceroporin transporter (AQP2), which are mutated in most drug-resistant isolates (33). Redox metabolism in trypanosomatids is based predominantly on their unique dithiol molecule trypanothione and the trypanothione reductase (36). *In vivo*, melarsoprol is rapidly metabolized to trypanocidal metabolites including melarsen oxide, which binds trypanothione forming the stable adduct MelT (37); MelT is expected to have diverse effects on redox metabolism, ROS stress management, and the formation of deoxynucleoside triphosphates (dNTPs) by ribonucleotide reductase (33, 36). Despite the established relationship between melarsoprol and trypanothione, which aspect of trypanothione pathway inhibition results in parasite killing remains undetermined (33). Because the biosynthetic and redox utilization pathways contain enzymes unique to trypanosomatids, they have been broadly explored as drug targets against American trypanosomes and *Leishmania* species (20, 38–42).

Here, we present a description of the newly generated gain-of-function parasite library and describe its use in a screen for factors that increase parasite survival in the presence of melarsoprol. Library induction in the presence of melarsoprol resulted in the isolation of a specific survivor population consisting of 57 significantly overrepresented genes. Among these genes, we identified the gene encoding the rate-limiting enzyme of trypanothione biosynthesis (γ-glutamylcysteine synthetase, *Tb927.10.12370*), whose established relationship with melarsoprol validates the gain-of-function library’s usefulness (26). In addition, we identified subsets of overrepresented genes encoding proteins associated with gene expression, the mitochondrion, and the flagellum whose association with melarsoprol had not been reported previously. Thus, the T. brucei ORFeome and resulting gain-of-function library that we generated are now positioned to provide new insights into trypanosomatid biology, pathogenesis, and drug resistance, which will promote the development of novel therapeutics.

## RESULTS

### Generation of a Trypanosoma brucei ORFeome.

**FIG 1:**
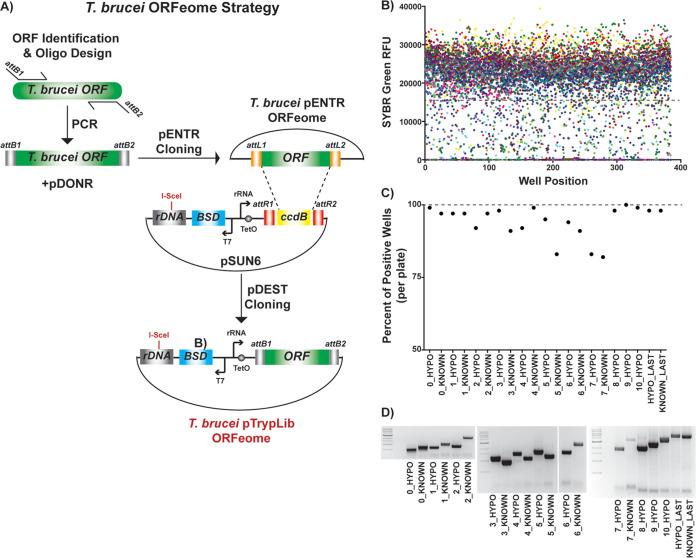
Generating a T. brucei ORFeome. (A) ORFeome cloning strategy: *attB* site addition to T. brucei ORFs during PCR amplification, BP Gateway cloning into pDONR221 to generate the pENTR ORFeome, and LR Gateway cloning into T. brucei*-*specific pDEST (pSUN6) (see Fig. S1 in the supplemental material) to generate the complete pTrypLib ORFeome. (B) Assessment of PCR amplification by SYBR green relative fluorescence units (RFU). Each color represents one of the 21 384-well plates, and each dot represents a PCR in a single well as measured by SYBR green RFU. (C) Percentages of PCR-positive wells (SYBR assessment) for each of the 21 384-well plates, from the first time amplified. (D) Agarose gel bands from each of the original 21 384-well PCR plates pooled prior to gel extraction and cloning, from the first time amplified, compared to 1-kb DNA ladder.

To generate a library consisting of all relevant ORFs from the T. brucei genome, start and stop sites for all T. brucei ORFs were obtained from available *TREU927* ribosomal profiling data for 9,200 genes (Fig. 1A) (43). We filtered out 1,956 ORFs unsuitable in size (<100 bp or >4,500 bp), coding for an undesired product (ribosomal genes, *VSGs*, *ESAGS*, pseudogenes), or annotated as “hypothetical unlikely.” Known multidrug-resistant channels (including MRPA, whose overexpression causes melarsoprol resistance) were also excluded (26). PCR primers for the resulting 7,245 targeted ORFs were designed *in silico* with *attB1* and *attB2* Gateway cloning sites with matched melting temperatures, synthesized, and resuspended in 21 separate 384-well plates that were organized by their anticipated ORF product size and gene annotations as either “known” or “hypothetical” (Table 1; also see Table S1 in the supplemental material for oligonucleotide sequences).

**TABLE 1:** 384-well oligo plates

Plate name	Min length (bp)	Max length (bp)	No. of ORFs per plate
0_hypothetical	102	375	373
0_known	147	591	315
1_hypothetical	375	522	374
1_known	594	849	365
2_hypothetical	522	666	372
2_known	849	1056	363
3_hypothetical	669	822	382
3_known	1056	1287	367
4_hypothetical	822	993	372
4_known	1287	1524	348
5_hypothetical	993	1155	365
5_known	1524	1857	342
6_hypothetical	1158	1365	375
6_known	1857	2337	362
7_hypothetical	1368	1635	378
7_known	2340	3504	376
8_hypothetical	1635	1953	381
9_hypothetical	1953	2508	381
10_hypothetical	2508	3501	381
hypothetical_last	3504	4488	135
known_last	3507	4497	138
Total			7245

10.1128/mSphere.00769-20.1 TABLE S1 Table of oligonucleotide pairs from all 21 384-well plates. The sequences of all 7,245 oligonucleotide pairs used to generate the T. brucei ORFeome are included with respect to their plate “name” and well position in each plate. Download Table S1, PDF file, 1.2 MB. Copyright © 2020 Carter et al. 2020 Carter et al. This content is distributed under the terms of the Creative Commons Attribution 4.0 International license.

10.1128/mSphere.00769-20.2 FIG S1 Map of T. brucei-specific pDEST cloning vector, pSUN6. Critical features of plasmid map are indicated, including rDNA spacer homology, T7 terminators, blasticidin resistance cassette, rRNA promoter, two tetracycline operators, attR site for LR recombination with pENTR library, and the 3′ GPEET and 5′ aldolase (long) UTRs. Download FIG S1, PDF file, 0.5 MB. Copyright © 2020 Carter et al. 2020 Carter et al. This content is distributed under the terms of the Creative Commons Attribution 4.0 International license.

Each ORF was PCR amplified in 384-well format from Lister 427 genomic DNA. The general quality of the PCRs was assessed by the addition of SYBR green and measurement of the resulting relative fluorescence units (RFU) (Fig. 1B). Based on the SYBR green assessment, initial PCRs resulted in the successful amplification of 94% of the ORFeome (6,820/7,245 ORFs) (Fig. 1C). To increase ORFeome coverage, we reamplified 429 failed PCRs and succeeded in producing 228 products, resulting in a final total of 7,039 PCR products amplified (97.2% of the targeted genes).

PCRs from each 384-well plate were pooled (10 μl from each well) into 21 corresponding PCR product pools, irrespective of the SYBR result, which maintained the product size range associated with each plate (Table 1). Each resulting size-sorted PCR pool was run on agarose gels and gel purified prior to Gateway cloning (Fig. 1D). Each size-sorted pool of gel-extracted PCR products was cloned into a standard pDONR Gateway cloning vector (pDONR221), as described (27, 44), to generate the pENTR ORFeome library. The resulting pENTR libraries were then transferred into a T. brucei-specific pDEST type vector with ribosomal DNA (rDNA) spacer targeting homology regions and a tetracycline-inducible system for ORF expression (Fig. 1A; see also Fig. S1). The resulting library of ORFs cloned for T. brucei genomic integration was termed the pTrypLib ORFeome.

10.1128/mSphere.00769-20.3 TABLE S2 Table of all final ORF pools used to make the pENTR ORFeome. Following the first pENTR_1 and pTrypLib _1 ORFeome assessments, ORFs missing from libraries were identified and reselected from original 21 PCR plates to form an additional 8 size-sorted pools. The original 21 pools, 2 pools of PCR reamplifications (NEG_PICKS) and 8 “missing” ORF pools (1-8_MISS) collectively result in 31 pools for pENTR cloning. The size ranges and total number of ORFs per pool are indicated. Download Table S2, PDF file, 0.4 MB. Copyright © 2020 Carter et al. 2020 Carter et al. This content is distributed under the terms of the Creative Commons Attribution 4.0 International license.

### Sequencing, assessment, and final coverage of the T. brucei ORFeome.

The T. brucei pENTR and pTrypLib ORFeome-harboring plasmids were each pooled and prepared for Illumina sequencing by tagmentation, in which a modified transposition reaction is used to cleave DNA and insert adaptors for high-throughput sequencing (45). To assess which of the 7,245 targeted ORFs were not present in the pENTR and pTrypLib ORFeomes, we aligned the sequencing reads to the *TREU927* genome, removed PCR duplicates, and counted the number of reads corresponding to each targeted ORF. Because we knew that some of the targeted genes were highly similar or duplicated, we aligned the reads under two modes, one that required unique alignments and one that allowed multiple alignments. Both data sets were then assessed to determine how many genes were “missing” from each library, defined as any targeted gene with zero aligned reads.

**FIG 2:**
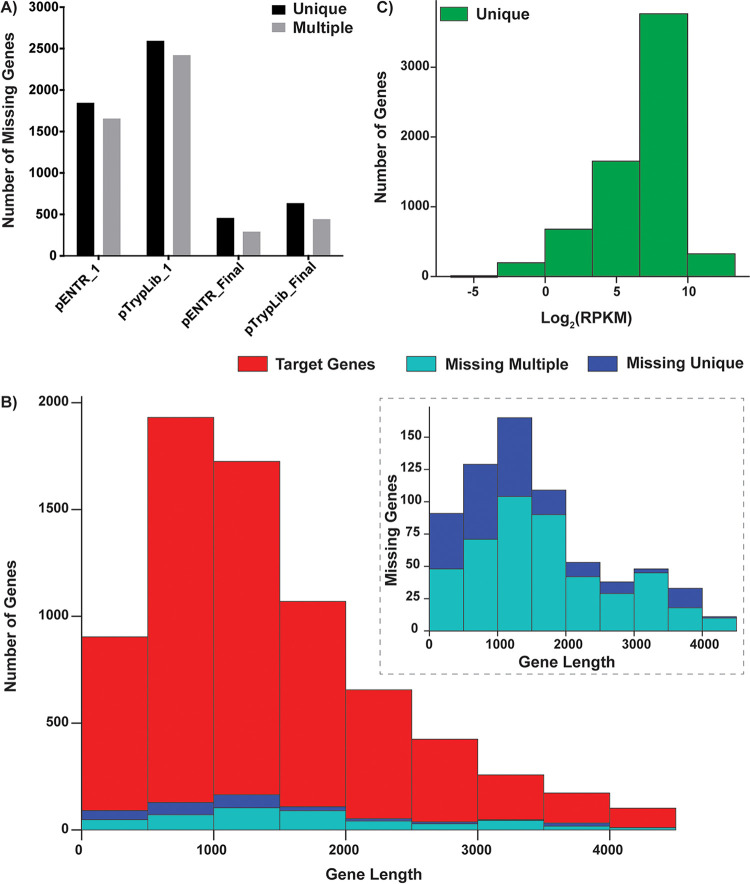
Assessment of pENTR and pTrypLib plasmid libraries. (A) Bar graph showing the number of targeted ORFs with zero detectable aligned reads from the first round of cloning (pENTR_1 and pTrypLib_1) and after both rounds of cloning (pENTR_Final and pTrypLib_Final) using analyses generated from both uniquely and multiply aligned reads. (B) Histograms showing the distribution of ORF lengths for the target gene list (red) and the set of ORFs with zero detectable aligned reads after both rounds of cloning (labeled as missing). Analyses from unique (dark blue) and multiply (light blue) aligned reads are shown. (Inset graph) Target ORF lengths have been left out to better visualize the lengths of the missing ORFs. (C) Histogram showing the distribution of normalized read counts for each ORF in the pooled pTrypLib plasmid libraries (uniquely aligned reads shown) (see Fig. S2 for both uniquely and multiply aligned reads).

Initial analysis showed 1,845 missing ORFs from the pENTR library and 2,593 missing ORFs from pTrypLib (Fig. 2A, pENTR_1, pTrypLib_1, unique alignments). To increase the number of ORFs in the final library, PCR products corresponding to each missing ORF were isolated from the original PCR plates. The resulting eight additional size-sorted ORF pools were gel purified, Gateway cloned (see Table S2 for cloning pools including “MISS_1-8”), sequenced by tagmentation, and analyzed as described above. The final ORFeomes were missing 457 ORFs from the pENTR library and 636 ORFs from pTrypLib (Fig. 2A, pENTR_Final, pTrypLib_Final, uniquely aligned reads, and see Data Set S1 for tables of all genes present). The final pTrypLib ORFeome contains 6,609 uniquely aligned and 6,803 multiply aligned T. brucei ORFs, resulting in 91% to 94% inclusion of the targeted ORFeome.

10.1128/mSphere.00769-20.4 DATA SET S1 Tables of all genes present in sequenced libraries, including pENTR, pTrypLib, GoF_Lib (BSD recovered), GoF_L1, and GoF_L2. Download Data Set S1, XLSX file, 0.9 MB. Copyright © 2020 Carter et al. 2020 Carter et al. This content is distributed under the terms of the Creative Commons Attribution 4.0 International license.

10.1128/mSphere.00769-20.5 FIG S2 Assessment of the pTrypLib ORFeome. (Top) Histograms showing the distributions of normalized read counts in the pTrypLib ORFeome in analyses using uniquely aligning reads (left) and allowing multiple alignments (right). (Bottom) The length of each ORF in the pTrypLib Orfeome was plotted against the normalized read count of the ORF. A best fit line was calculated using linear regression (shown in white). For uniquely aligned reads, this was *y* = 7.6 − 0.00067*x* (*R*^2^ = 0.048), and for multiply aligned reads, this was *y* = 7.3 − 0.00078*x* (*R*^2^ = 0.051). Download FIG S2, PDF file, 2.1 MB. Copyright © 2020 Carter et al. 2020 Carter et al. This content is distributed under the terms of the Creative Commons Attribution 4.0 International license.

To analyze whether large or small genes were overrepresented in the set of missing genes (unsuccessfully cloned ORFs), we compared the distributions of gene lengths between the target set of ORFs (Fig. 2B, red bars) and missing genes (Fig. 2B, blue and teal bars). The distributions of gene lengths were similar, indicating that cloning failure was likely independent of gene size.

Coverage of each ORF in pTrypLib was analyzed by count distribution based on the number of reads aligned. Most ORFs resulted in log_2_ reads per kilobase per million (RPKM) values between 0 and 10 (Fig. 2C and Fig. S2, top right). Thus, the numbers of poorly represented ORFs (RPKM < 1) were 195 for uniquely aligned reads and 369 for multiply aligned reads, representing 3% and 5% of all ORFs in the library, respectively. We then determined if ORF length affected representation in the library by plotting the log_2_ RPKM value against ORF length (Fig. S2). No strong correlation was observed between ORF length and coverage in the pTrypLib ORFeome, with a best fit line showing a small negative slope for both unique and multiply aligned reads (−0.00067 and −0.00077, respectively). Thus, in general, shorter ORFs are not significantly more highly represented than longer ORFs (Fig. S2).

### A T. brucei gain-of-function parasite library.

**FIG 3:**
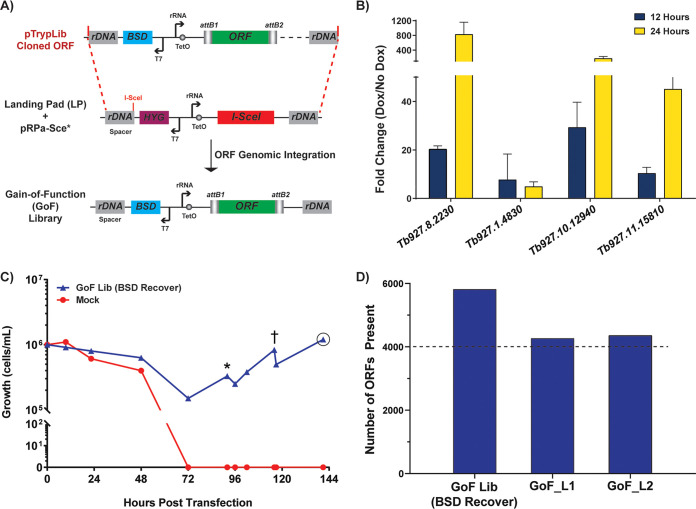
Generation and validation of the T. brucei GoF library. (A) Transfection of pTrypLib ORFeome into parental landing pad (LP) cell line harboring pRPaSce* plasmid for I-SceI-induced enzymatic cleavage of a single rDNA spacer site to increase transfection efficiency, as previously reported (6). (B) Inducible expression of a low-complexity GoF library measured by RT-qPCR following 12 and 24 h of doxycycline induction compared to that in uninduced cells (no Dox). (C) Generation of the pTrypLib ORFeome-based GoF parasite library. Graph shows the recovery of GoF library-harboring cells (blue line) compared to that from mock transfection (red line) in blasticidin (BSD) (“BSD recover” indicates recovery of the selected GoF library) added at time 0, 12 h posttransfection. *, cells spun and resuspended in 300 ml HMI-9; †, addition of 500 ml HMI-9; ○, time of GoF library harvest. (D) Assessment of the number of ORFeome genes present in the GoF library following initial transfection [GoF Lib (BSD recover), blasticidin-recovered population] and following freeze-thaw and 3 days of growth to generate GoF_L, which was then used to generate NGS libraries using two alternative protocols (see Materials and Methods) resulting in GoF_L1 and GoF_L2.

The pTrypLib ORFeome contains more than 6,500 tetracycline-inducible ORFs ready for T. brucei genomic integration at an rDNA spacer site. The landing pad (LP) system, developed for RIT-Seq library screens, was employed to ensure faithful integration into a single rDNA spacer site and high transfection efficiency (Fig. 3A), which is promoted by the site-specific induction of an I-SceI DNA break, as described previously (6).

Prior to transfection of the full pTrypLib ORFeome, we sought to verify inducible expression of this system using a low-complexity library. The low-complexity library was generated by transfecting a small number of equimolar pooled ORFs and recovering a single population of parasites. Thus, we generated an ORF library with 1,000 times less complexity than the complete pTrypLib. The low-complexity library was then grown with or without doxycycline (Dox) induction for 12 or 24 h prior to RNA extraction and reverse transcription-quantitative PCR (RT-qPCR) analysis to measure inducible expression of the transfected ORFs. ORFs showed increased transcript levels following Dox induction at 12 and 24 h; 3 of the 4 ORFs analyzed resulted in approximately 10- to 30-fold increased transcript levels after 12 h and 50- to 600-fold increases in transcript levels after 24 h (Fig. 3B). Thus, the overall strategy of ORFeome exogenous transcription induction from pTrypLib cloned ORFs was deemed viable.

The full pTrypLib ORFeome was then used to generate an inducible T. brucei gain-of-function (GoF) library by transfecting 360 million LP cells and selecting with blasticidin (BSD) (6). Sixty million cells survived transfection, which were then propagated to 3 billion cells over 3 days to generate the T. brucei GoF library (Fig. 3C, blue line). Illumina sequencing libraries were prepared using a custom P5 forward oligonucleotide containing *attB1* site complementarity and a universal P7 reverse oligonucleotide. Indexed products were Illumina sequenced using a custom oligonucleotide complementary to the *attB1* site upstream of the introduced ORF. Thus, the resulting sequencing reads primarily correspond to the 5′ ends of the introduced ORF (see Fig. S3). Immediately following transfection and recovery in blasticidin, the T. brucei GoF library consisted of 5,819 ORFs [Fig. 3D, GoF Lib (BSD recover)] and then approximately 4,300 ORFs following freeze thaw (Fig. 3D, GoF_L1 and GoF_L2) (alternative sequencing conditions described in Materials and Methods). It is unclear if the apparent loss of approximately 1,500 ORFs arose through an artifact associated with a relatively low number of next-generation sequencing (NGS) reads returned from those samples or a true loss of content between library transfection and the subsequent thawing of frozen library.

10.1128/mSphere.00769-20.6 FIG S3 Sequencing strategy and results from transfected libraries. (A) Library preps from pTrypLib-transfected parasites proceed by first fragmenting genomic DNA, ligating Illumina adaptors, and amplifying library-introduced ORFs using a primer complementary to the *attB1* Gateway cloning sequence and the standard Illumina barcoded reverse primer. Sequencing proceeds using a custom forward primer complementary to the *attB1* Gateway cloning sequence. (B) Red rectangles in the top row represent annotated genes from a section of T. brucei chromosome 5. Bars in subsequent rows represent reads that align to the genes in the top row. Most reads align to the first 100 bp of the gene, as expected from the library prep and sequencing strategy. Download FIG S3, PDF file, 3.0 MB. Copyright © 2020 Carter et al. 2020 Carter et al. This content is distributed under the terms of the Creative Commons Attribution 4.0 International license.

### Isolation of melarsoprol survivors by gain-of-function genetic screening.

**FIG 4:**
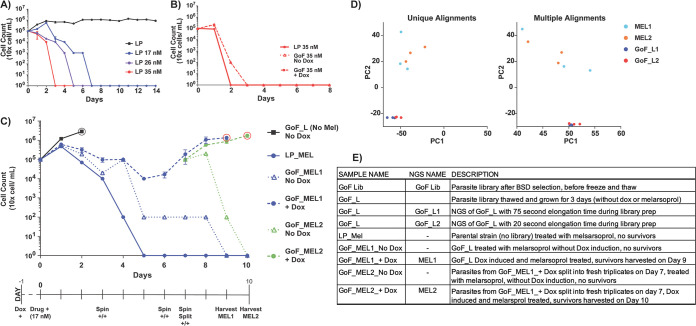
Isolation of melarsoprol survivor populations from a GoF screen. (A) Growth of landing pad (LP) parental cell line in 17 nM (blue line), 26 nM (purple line), 35 nM (red line), or no (black line) melarsoprol (black line). (B) GoF library screen in 35 nM melarsoprol treatment: LP cell line, solid red line; uninduced GoF library, red dotted line; induced GoF library, red dashed line. Dotted and dashed lines overlap. (C) GoF genetic screen in 17 nM melarsoprol. Timeline at the bottom of the graph indicates days on which either Dox (+Dox), melarsoprol (+Drug), or both (+/+) were added. All cultures (other than GoF_L) were continuously grown in the presence of 17 nM melarsoprol. On days 3, 6, and 7, the triplicate cultures were centrifuged and resuspended in fresh medium with melarsoprol and Dox for induction (noted as spin +/+). Biological triplicate cultures are as follows: GoF_L, untreated GoF library-harboring cells grown for 3 days (black line); LP parental cell line, solid blue line; uninduced GoF library (no Dox), blue triangles on dotted line; induced GoF library (+ Dox), blue circles on dashed line, harvested on day 9 (red circle on blue line) to produce MEL1. On day 7, biological triplicates from GoF_MEL1 +Dox (blue circles on dashed line) were split into two sets of triplicate samples, both in 17 nM melarsoprol, one of which was not further induced (no Dox, green triangles on dotted line). The other continued to be induced (+Dox, green squares on dashed green line) and was harvested on day 10 to produce MEL2 (red circle indicates harvest). (D) Principal-component analysis (PCA) comparing GoF_L libraries (L1 and L2) (see Materials and Methods) with libraries arising following continuous melarsoprol selection (MEL1 and MEL2). (E) Table of sample names and NGS sequencing samples with full description.

To identify ORFs whose induced expression promoted survival in the presence of lethal doses of melarsoprol, we tested three concentrations of drug on the LP cell line. Similar to previous reports, we observed that T. brucei LP cells died after 3 days in 35 nM, 5 days in 26 nM, and 7 days in 17 nM melarsoprol (17 nM is approximately two times the standard 50% effective concentration [EC_50_] in culture and significantly less than concentrations used in clinical treatments) (Fig. 4A) (10). In a GoF genetic screen using 35 nM melarsoprol, no survivor population emerged (Fig. 4B, red dashed and dotted lines overlap). Thus, 17 nM melarsoprol was selected for a GoF genetic screen to allow more time for induced ORF expression that might confer resistance.

A GoF survivor screen was conducted in 17 nm melarsoprol for 10 days. As a control, GoF library-harboring parasites were grown in triplicate for 3 days (day −1 through day 2) without melarsoprol or Dox treatment to generate NGS libraries representative of all ORFs present prior to selection [Fig. 4C, GoF_L (no Mel), harvested on day 2, black circle). All other cultures were under continuous 17 nM melarsoprol (Mel) selection in triplicate for the following conditions: (i) landing pad (LP_MEL), (ii) GoF library parasites without Dox induction (GoF_MEL1 no Dox), and (iii) GoF library parasites with Dox induction (GoF_MEL1 +Dox). The timeline at the bottom of Fig. 4C shows when Dox was added, when melarsoprol [“drug (17 nM)”] was added, and when cells were spun and resuspended in fresh medium (“spin”), which was always replenished with the appropriate treatment (Dox/drug [+/+]).

Following 4 days of melarsoprol treatment, LP had cell counts below the limit of detection (10,000 cells/ml) and, from day 5 on, showed no signs of life (Fig. 4C, LP_MEL, solid blue line). On day 5, uninduced GoF library counts were below the limit of detection (Fig. 4C, GoF_MEL1 no Dox, dotted blue line), whereas induced GoF library resulted in a survivor population (Fig. 4C, GoF_MEL1 +Dox, dashed blue line). While a survivor population did not arise from uninduced GoF Library, parasite death was delayed by at least 1 day compared with that for LP (Fig. 4C, dotted blue line). Persistence of uninduced GoF library parasites in the presence of melarsoprol is probably the result of leaky gene expression from the rDNA spacer, an established caveat of this approach (46). The population of melarsoprol survivors arising from the induced GoF library (GoF_MEL1 +Dox) began to replicate efficiently in the presence of drug following day 5. On day 7, the triplicate samples were split into an additional 3 flasks that did not receive Dox induction (GoF_MEL2 no Dox, green dotted line) and 3 with Dox added (GoF_MEL2 +Dox, green dashed line); all continued to undergo 17 nM melarsoprol treatment. Only Dox-induced GoF library cultures were able to grow in the presence of melarsoprol (Fig. 4C, blue and green dashed lines), suggesting that library induction promoted survival in these populations.

The resulting Dox-induced populations of survivors, termed GoF_MEL1 (MEL1) and GoF_MEL2 (MEL2) (Fig. 4E summarizes sample nomenclature), were harvested for genomic DNA extraction at days 9 and day 10, respectively (Fig. 4C, red circles). Genomic DNAs from biological triplicate cultures of GoF_L (no melarsoprol treatment), MEL1 (initial population of survivors), and MEL2 (secondary population of survivors) (9 cultures total grown to ∼1 million cells per ml, 200 ml each) were prepared for NGS analysis. The genomic DNA (gDNA) arising from GoF_L was prepared for NGS analysis using two elongations times to determine if this parameter biased the results, generating GoF_L1 and GoF_L2 (described in Materials and Methods).

We performed principal-component analysis (PCA) on the resulting sequencing data using both unique and multiple alignments (Fig. 4D). The PCA analysis shows two clearly separated clusters for untreated and melarsoprol-treated samples, with most biological replicates clustering together. DNAs arising from melarsoprol survivor populations (MEL1 and MEL2) were distinct from those of untreated GoF_L and showed more variation between samples (Fig. 4D, GoF_L1 and GoF_L2 versus MEL1 and MEL2). We observed, at best, a weak negative association between gene length and normalized read count (slopes of −0.00042 and −0.00045 for unique and multiple alignment analyses, respectively), indicating that ORF representation in the library is largely independent of ORF length (see Fig. S4)

10.1128/mSphere.00769-20.7 FIG S4 Assessment of coverage in the GoF_L library. (Top) Histograms showing the distributions of normalized read counts in the sequencing libraries from GoF_L2 in analyses using uniquely aligning reads (left) and allowing multiple alignments (right). (Bottom) The length of each ORF in the pTrypLib Orfeome was plotted against the normalized read count for the GoF_L2 sequencing library. A best fit line was calculated using linear regression (shown in white). For uniquely aligned reads, this was *y* = 6.1 − 0.00042*x* (*R*^2^ = 0.050), and for multiply aligned reads, this was *y* = 6.1 − 0.00045*x* (*R*^2^ = 0.056). Download FIG S4, PDF file, 3.4 MB. Copyright © 2020 Carter et al. 2020 Carter et al. This content is distributed under the terms of the Creative Commons Attribution 4.0 International license.

### Identification of overrepresented gain-of-function ORFs in melarsoprol survivors.

**FIG 5:**
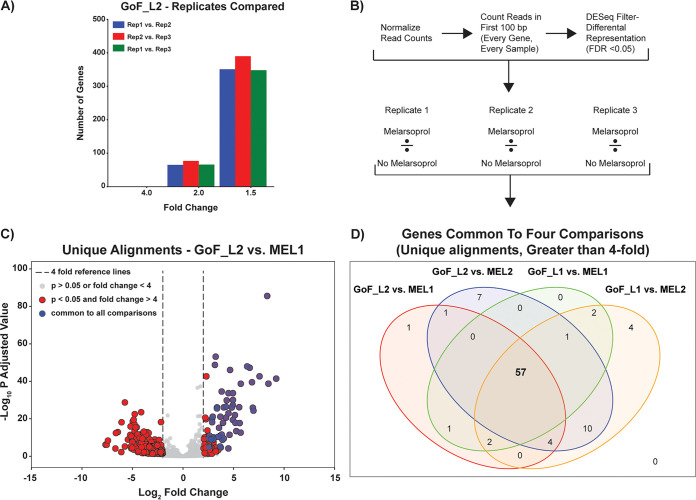
Identification of significantly overrepresented ORFs in melarsoprol GoF survivor populations. (A) The numbers of genes with changes of >1.5-, 2-, or 4-fold for comparisons among all three replicates (Rep1, Rep2, and Rep3) of GoF_L2. (B) Hit-calling pipeline to identify genes overrepresented in melarsoprol survivor populations. (C) Volcano plot showing the −log_10_ *P* adjusted values versus log_2_ fold change in normalized counts for the comparison of melarsoprol-selected MEL1/GoF_L2 for each ORF in the targeted library. Blue dots represent overrepresented ORFs common to all four comparisons described in panel D. (D) Venn diagram illustrates the significantly overrepresented genes common to each comparison between GoF_L (GoF_L1 and GoF_L2) and melarsoprol treated (MEL1 and MEL2) and shared among all comparisons between replicates, resulting in 57 overrepresented hits identified in melarsoprol survivor populations compared to those in GoF_L populations.

We reasoned that any gene whose induction contributed to melarsoprol resistance should be overrepresented in induced libraries generated from melarsoprol survivor populations. To determine the fold change that represents a valid difference between melarsoprol-treated and untreated conditions, we compared each of the three biological replicates of GoF_L2 to one another and counted the number of ORFs with a 1.5-, 2.0-, or 4.0-fold change in normalized read count (Fig. 5A). By evaluating the biological variation between similarly treated replicates, we found that while many ORFs varied in normalized read count by greater than 1.5-fold between replicates (more than 300), very few ORFs varied by greater than 4-fold (Fig. 5A) (similar results obtained from GoF_L1, data not shown). Thus, we used a 4-fold change in normalized read count between melarsoprol-treated and untreated samples as the minimum threshold for identifying an ORF as overrepresented (a “hit”) in this study.

To identify ORFs that were overrepresented in the melarsoprol-selected population, we analyzed the aligned reads using DESeq2 and selected genes that were ≥4-fold overrepresented with an adjusted *P* value of less than 0.05 (Fig. 5B). We used reads exclusively within the first 100 bp of each ORF (Fig. S3B). Four different comparisons were analyzed using this pipeline: GoF_L1 versus MEL1, GoF_L1 versus MEL2, GoF_L2 versus MEL1, and GoF_L2 versus MEL2 (Fig. 5B; see also Data Set S2 for raw and DESeq2 normalized reads). Figure 5C shows a volcano plot of DESeq2-generated significance values versus fold change for the comparison between GoF_L2 and MEL1. After hits had been called for each individual comparison, we identified the hits common among all 4 comparisons for both uniquely and multiply aligned reads (Fig. 5D; see also Data Set S3 for tables of all comparisons). These analyses resulted in the identification of 57 overrepresented ORFs (uniquely aligned) in the GoF melarsoprol survivor populations compared to those in GoF_L populations. In the comparison of GoF_L2 versus MEL1 depicted in the volcano plot, we observe that these 57 ORFs common to all the comparisons were among the most highly overrepresented genes and with some of the lowest *P* adjusted values determined by DESeq2 (Fig. 5C, blue dots). Similar results were obtained for all comparisons between melarsoprol-selected and untreated GoF_L samples. An important caveat is that genes whose overexpression confers a significant survival advantage could very well show up as false positives within the set of genes identified to promote survival in melarsoprol. This is further explored in the Discussion section.

10.1128/mSphere.00769-20.8 DATA SET S2 Melarsoprol screen tables of raw reads and DESeq2 normalized reads for all survivor conditions. Download Data Set S2, XLSX file, 3.4 MB. Copyright © 2020 Carter et al. 2020 Carter et al. This content is distributed under the terms of the Creative Commons Attribution 4.0 International license.

10.1128/mSphere.00769-20.9 DATA SET S3 Tables of all data pertaining to genes overrepresented in melarsoprol survivor populations. Download Data Set S3, XLSX file, 3.4 MB. Copyright © 2020 Carter et al. 2020 Carter et al. This content is distributed under the terms of the Creative Commons Attribution 4.0 International license.

### Melarsoprol resistance resulting from GoF hit overexpression.

The 57 genes overrepresented in melarsoprol survivor populations are predominantly annotated as conserved hypothetical proteins or have putative functional assignments. To categorize all 57 genes, we utilized microscopic and proteomic localization data (47) (curated through TriTrypDB) and available publications (listed in Table 2) that addressed protein functionality. Based on this analysis, we organized the hits into specific categories and found that the top three groups were associated with gene expression (16 genes), the mitochondrion (10 genes), and the flagellum (10 genes) (Table 2). The gene expression category was further divided into those associated with splicing (5 genes), posttranscriptional regulation (5 genes), and translation (3 genes). It is important to note that categories based on localization were predominantly derived from data generated in insect stage (procyclic form) parasites, though some can also be confirmed from specific bloodstream-form data (47–49). Based on these categories and the fold overrepresentation of each ORF in melarsoprol survivors, we selected a subset of genes to analyze their effects on melarsoprol resistance.

**TABLE 2:** Hits overrepresented in melarsoprol survivors (comparison of GoF_L2 vs. MEL1)^*a*^

Gene ID	Description (Tryptag 062920)	Localization (TrypTag or proteome)	PubMed	Category	Fold change overrepresented	DESeq *P*-adjusted value
*Tb927.4.4810*	Hypothetical protein, conserved	Cytoplasm (points, weak), endocytic		Endocytic+	597	3.77E-42
*Tb927.11.590*	Hypothetical protein, conserved	Strong mitochondrial signal		Mitochondrial*	350	1.81E-39
*Tb927.7.2780*	Hypothetical protein, conserved, XAC1	Cytoplasm	26784394	Gene Expression#	322	2.83E-86
*Tb927.10.12370*	Gamma-glutamylcysteine synthetase, GSH1	Nucleoplasm	8663359	Biosynthetic#	191	2.19E-43
*Tb927.5.3450*	Eukaryotic translation initiation factor eIF2Â	Strong cytoplasm signal	24945722	Gene Expression#	126	4.50E-25
*Tb927.11.11435*	Dynein light chain lc6	Strong flagellum axoneme signal		Flagellar+	120	1.30E-26
*Tb927.7.6190*	Ring finger domain containing protein, ubiqutin ligasê	Strong endocytic system signal		Endocytic+	113	3.13E-40
*Tb927.11.12380*	Hypothetical protein, conserved	Nuclear localization (mass spectrometry)		Nuclear+	97	5.89E-48
*Tb927.6.1280*	Translation initiation factor EIF-2b alpha subunit̂	Cytoplasm		Gene Expression#	81	1.14E-48
*Tb927.8.3820*	Stress granule protein	Localization to starvation stress granules	26187993	Stress Granule#	53	2.04E-39
*Tb927.3.5250*	Zinc finger CCCH domain-containing protein 8, ZC3H8	Cytoplasm (points)	26784394	Gene Expression#	53	2.45E-13
*Tb927.9.10930*	Mediator of RNA polymerase II transcription subunit 7, MED-T7	Nucleoplasm (points)	20876299	Gene Expression#	49	1.98E-18
*Tb927.7.770*	Ring finger domain containing protein̂	Moderate nuclear lumen signal, weak cytoplasm signal		Nuclear+	47	7.14E-27
*Tb927.9.7820*	Hypothetical protein, conserved	Nuclear, mass spec		Nuclear○	39	3.88E-14
*Tb927.11.15280*	tRNA-specific adenosine deaminase, ADAT3	Cytoplasm (reticulated, weak)	17483465	Gene Expression#	37	1.59E-18
*Tb927.4.1910*	Hypothetical protein, conserved	Cytoplasm (points, reticulated)	26784394	Gene Expression#	35	1.10E-22
*Tb927.9.15020*	Hypothetical protein, conserved	Weak axoneme signal		Flagellar+	31	4.45E-20
*Tb927.10.2830*	Hypothetical protein, conserved	Endocytic, cytoplasm		Endocytic+	31	9.17E-31
*Tb927.10.12050*	LSU ribosomal protein, mitochondrial̂	Kinetoplast, mitochondrion	18364347	Mitochondrial*	31	2.16E-34
*Tb927.9.7200*	Hypothetical protein, conserved	Kinetoplast, mitochondrion		Mitochondrial*	31	5.61E-28
*Tb927.7.5460*	Exosome-associated protein 3,3' exoribonuclease, putative, EAP3	Nucleoplasm		Nuclear+	28	9.55E-27
*Tb927.4.4540*	Zinc finger domain, LSD1 subclasŝ	Cytoplasm (reticulated)		Zinc finger	25	8.84E-47
*Tb927.5.4370*	Hypothetical protein, conserved	Endocytic, cytoplasm		Endocytic+	24	3.59E-12
*Tb927.9.7080*	Hypothetical protein, conserved	Cytoplasm (patchy, points)		Mitochondrial & ER○	23	1.14E-23
*Tb927.2.5210*	3-Oxoacyl-ACP reductasê	Mitochondrion, kinetoplast (strong)	17166831	Mitochondrial#	23	2.16E-34
*Tb927.11.2910*	Phosphoglycerate mutase, iPGAM̂	Mitochondrion (75%), kinetoplast (75%)		Mitochondrial*	23	6.79E-05
*Tb927.11.7475*	AN1-like zinc finger-containing protein̂	Cytoplasm		Zinc finger	20	1.50E-30
*Tb927.9.5220*	Conserved protein	Endocytic, cytoplasm (weak)		Endocytic+	19	2.60E-21
*Tb927.3.4930*	Hypothetical protein, conserved	Cytoplasm, flagellar cytoplasm,		Flagellar +	19	3.79E-27
*Tb927.8.1930*	Isy1-like splicing family	Nucleoplasm	9250687	Gene Expression#	16	8.36E-27
*Tb927.10.6850*	Mitochondrial ribosomal protein S18̂	Cytoplasm (reticulated)	18951088	Mitochondrial#	16	3.68E-10
*Tb927.11.1810*	Ring finger domain-containing protein̂	Flagellar pocket (ring)		Flagellar +	16	1.50E-18
*Tb927.5.2620*	Hypothetical protein, conserved	Cytoplasm (points)	24945722	Gene Expression	15	1.78E-09
*Tb927.10.390*	DUF2407 ubiquitin-like domain-containing protein̂	Flagellum matrix proteome (BSF)	24741115	Flagellar*	14	2.78E-12
*Tb927.3.1610*	CMGC/CLK family protein kinasê	Nucleoplasm, cytoplasm	24453978	Kinase	14	2.82E-35
*Tb927.11.2350*	Hypothetical protein, conserved	Nucleus, cytoplasm (reticulated)		Nuclear+	13	2.19E-05
*Tb927.8.7790*	Zinc finger domain, LSD1 subclasŝ	Tagging not successful, ND		Mitochondrial & ER○	13	7.88E-22
*Tb927.5.4150*	Hypothetical protein, conserved	Paraflagellar rod		Flagellar*	13	6.29E-06
*Tb927.8.3340*	Hypothetical protein, conserved	Nucleoplasm		Nuclear+	13	1.28E-11
*Tb927.10.1490*	Temperature-dependent protein affecting M2 dsRNA replication̂	Cytoplasm (weak)	20592024	Gene Expression#	12	6.62E-16
*Tb927.4.890*	Small nuclear ribonucleoprotein SmD3, putative, SmD3	Nucleoplasm	10900267	Gene Expression#	11	4.78E-11
*Tb927.7.5360*	Haemolysin-III related̂	Cytoplasm		Pathogenesis	10	8.31E-27
*Tb927.7.710*	Heat shock 70-kDa protein, HSP70̂	Cell tip (anterior), cytoplasm, flagellar cytoplasm	30506377	Flagellar *	10	3.15E-26
*Tb927.9.4930*	Divalent cation transporter̂	Cytoplasm (reticulated)		Trafficking	9	7.16E-54
*Tb927.9.10850*	Splicing factor 3B subunit 10, SF3b10̂	Nucleoplasm		Gene Expression	9	1.80E-49
*Tb927.11.5600*	Archaic translocase of outer membrane 14-kDa subunit, ATOM14	Mitochondrion	22267727	Mitochondrial#	8	9.21E-11
*Tb927.10.9060*	Hypothetical protein, conserved	Basal body		Flagellar +	8	5.86E-10
*Tb927.1.1020*	Leucine-rich repeat-containing protein	Hook complex		Flagellar +	8	1.61E-10
*Tb927.2.2130*	Small GTP-binding protein RAB6̂	Golgi apparatus		Golgi+	8	7.70E-22
*Tb927.1.3310*	Hypothetical protein, conserved	Plasma membrane (posterior)	18242729	Glycosomal#	8	2.28E-09
*Tb927.8.4200*	Hypothetical protein, conserved	Cytoplasm	24945722	Gene Expression#	7	7.02E-19
*Tb927.3.5190*	Hypothetical protein, conserved	Mitochondrion		Mitochondrial+	7	8.48E-21
*Tb927.9.3480*	U5Cwc21 small nuclear ribonucleoprotein	Nucleoplasm	19429779	Gene Expression#	7	8.83E-10
*Tb927.8.2391*	Hypothetical protein, conserved	Not in the genome version used for tagging		ND	7	9.18E-10
*Tb927.2.3780*	Translation initiation factor IF-2̂	Cytoplasm		Gene Expression	6	1.12E-11
*Tb927.1.1500*	Conserved protein, unknown function	Cytoplasm (reticulated)	26784394	Gene Expression#	6	1.34E-05
*Tb927.6.3980*	Hypothetical protein, conserved	Axoneme [50%], cytoplasm		Flagellar +	6	3.88E-10

a Tryptag descriptions containing the word “putative” have been replaced with ^. Category data sources: +, TrypTag; ○, proteome; *, both; #, published.

**FIG 6:**
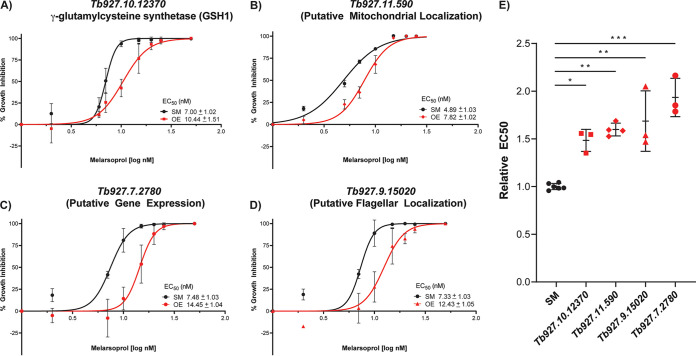
Melarsoprol resistance following gene overexpression. Induced expression of four hits (red lines), in comparison to those in parental cells (SM, black lines), during melarsoprol treatment with cell viability measured by alamarBlue assay to measure the resulting EC_50_: (A) *Tb927.10.12370*. (B) *Tb927.11.590*. (C) *Tb927.7.2780*. (D) *Tb927.9.15020*. (E) Relative EC_50_s following overexpression of each gene for at least 3 biological replicates. *P* values were derived from one-way analysis of variance (ANOVA) with Dennett’s multiple-comparison test. *, *P* < 0.05; **, *P* < 0.01; ***, *P* < 0.001.

We cloned a subset of overrepresented genes into a standard overexpression vector, transfected bloodstream-form T. brucei, and analyzed the effect of overexpression on melarsoprol resistance in cell viability assays (Fig. 6). The essential gene encoding γ-glutamylcysteine synthetase (GSH1; *Tb927.10.12370*) (50), which is the rate-limiting step of trypanothione biosynthesis (26, 38, 42, 51), was 191-fold overrepresented in melarsoprol survivors (Table 2). Trypanothione is the primary intracellular target of melarsoprol, and overexpression of GSH1 in T. brucei and other trypanosomatids increases the concentration of intracellular trypanothione, resulting in melarsoprol resistance under laboratory conditions (52). In our hands, overexpression of GSH1 resulted in an approximately 1.5-fold increase in the relative EC_50_ of melarsoprol (Fig. 6A and E). The occurrence of GSH1 among the most overrepresented melarsoprol GoF survivors supports the usefulness of this tool in identifying drug targets.

We then evaluated the overexpression of three genes not previously linked to melarsoprol resistance, which were categorized as mitochondrial (*Tb927.11.590*, 350-fold overrepresented), gene expression (*Tb927.7.2780*, 322-fold overrepresented), and flagellar (*Tb927.9.15020*, 31-fold overrepresented). The most pronounced effect was a 2-fold increase in relative EC_50_ of melarsoprol following the overexpression of *Tb927.7.2780*, which encodes the putative posttranscriptional activator XAC1 (expression activator 1) (Fig. 6C and E) (24). Overexpression of the mitochondrion-localized protein encoded by *Tb927.11.590* resulted in a >1.5-fold shift in the relative EC_50_ of melarsoprol. Similarly, overexpression of the flagellar protein encoded by *Tb927.9.15020* resulted in an approximately 1.5-fold increase relative EC_50_ of melarsoprol (Fig. 6D and E). Together, these results show that genes identified in melarsoprol GoF screening can promote drug resistance upon overexpression. Our results further support trypanothione as a major target of intracellular melarsoprol and implicate novel genes and mechanisms of melarsoprol resistance in trypanosomatids.

## DISCUSSION

The forward genetics tools generated here address an urgent need to extend genomic functional characterization in T. brucei and its trypanosomatid relatives. More than 30 years of genetic and biochemical studies in trypanosomatids, 10 of which included the extensive use of an RNAi-based loss-of-function library, have produced key discoveries in parasitology and basic biology (53). Yet, with the functions of more than 35% of trypanosomatid encoded genes largely unknown, many mysteries remain unsolved and more functional pathways must be delineated. Here, we have generated two powerful tools for forward genetic approaches: an ORFeome consisting of more than 6,500 T. brucei ORFs and an inducible gain-of-function library harbored in T. brucei parasites, whose functionality was validated in a melarsoprol proof-of-principle screen.

**FIG 7:**
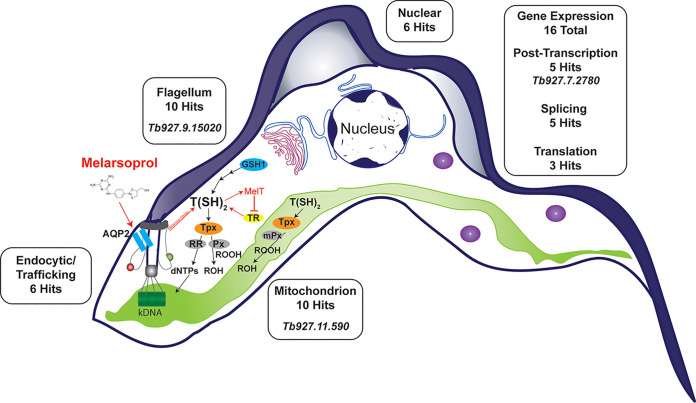
Categorization of GoF Hits. Hits arising from melarsoprol survival screening are shown proximal to bloodstream-form T. brucei cell cartoon. Rectangular boxes indicate the number of hits occurring in each major GoF screen category (see Table 2 for details). Italicized gene names in boxes are shown for genes whose induced expression promoted melarsoprol resistance (Fig. 6). The cell diagram also highlights the flagellum and flagellar pocket with the melarsoprol transporter AQP2 localized as seen in bloodstream form (34, 64). Trypanothione (T[SH]_2_) biosynthesis and redox pathways are loosely depicted as follows: T(SH)_2_ biosynthesis is highly simplified showing the rate-limiting enzyme GSH1, which was identified in the melarsoprol GoF screen; T(SH)_2_ provides reducing equivalents to tryparedoxin (Tpx), which is used to reduce disulfides (not shown), peroxidases (Px), and ribonucleotide reductase (RR) for the reduction of hydroperoxides and generation of dNTPs, respectively. T(SH)_2_ and Tpx are also utilized in the mitochondrion for redox reactions that include reduction of peroxidases (mPx). Melarsoprol uptake, conversion to melarsen oxide, binding with T(SH)_2_ to from the stable adduct MelT, and its inhibition of trypanothione reductase (TR), which prevents the conversion of trypanothione disulfide back to T(SH)_2_, are all indicated in red. Green and red spheres at the flagellar pocket indicate import and export pathways, respectively.

Once in the cell, melarsoprol is metabolized into multiple forms, including melarsen oxide, which complicates the identification of drug targets and determination of its mode of cell killing. In this study, we identified γ-glutamylcysteine synthetase (GSH1, *Tb927.10.12370*) among our top hits, whose overexpression increases the intracellular concentration of trypanothione, the primary intracellular target of melarsen oxide (26, 50, 54). It is likely that GSH1 overexpression generates sufficient levels of trypanothione [T(SH)_2_] to partially overcome melarsoprol inhibition (26). Identification of GSH1 in the melarsoprol GoF screen demonstrates the ability of this tool to identify drug targets [Fig. 7, T(SH)_2_ pathway] (26, 36).

Trypanothione biosynthesis and redox reactions primarily occur in the cytosol (36). Recently it was demonstrated that trypanothione and trypanothione reductase function in the mitochondrion (Fig. 7, mitochondrion in green), but these studies strongly suggested the requirement for unidentified oxidoreductases functioning in the organelle (55). Genes identified in the melarsoprol GoF screen suggest a previously uninvestigated connection between the drug and mitochondrion, though not entirely unanticipated based on trypanothione functions (36). The 10 melarsoprol GoF hits categorized as mitochondrial included β-ketoacyl-acyl carrier protein (ACP)-reductase (*Tb927.2.5210*, 23-fold overrepresented), which is required for fatty acid chain elongation in the mitochondrion as well as the production of the secondary redox carrier lipoic acid (56, 57). Here, we have also shown that overexpression of *Tb927.11.590*, which encodes a mitochondrial protein with predicted oxidoreductase and catalytic domains, can increase the EC_50_ of melarsoprol (Fig. 6). It is intriguing to speculate that melarsoprol treatment may cause reactive oxygen species (ROS) or redox stress in the organelle, which might be alleviated by the overexpression of the mitochondrial proteins identified herein.

One drawback of the melarsoprol screen reported here is that we did not include a doxycycline-induced condition without melarsoprol treatment (+Dox, −Mel). Thus, any gene whose overexpression promotes survival independent of melarsoprol would be included in the hit list as a false positive. We did sequence a number of +Dox, −Mel samples over the course of the development of the library, but the results were not reproducible enough to publish, as they were performed with slightly different conditions each time. That said, we did not identify any genes as being overrepresented by >4-fold in parasites treated with Dox compared to that in untreated parasites in three separate experiments. Another caveat is that genes that code for protein products that are part of large complexes may be unstable when overexpressed individually and thus cannot be easily identified with this genetic screen.

It is unclear at this time if overrepresented genes identified in melarsoprol survivors are direct targets of melarsoprol or if they cause indirect effects that can promote resistance. Hits categorized as gene expression represent a complex list including genes associated with splicing, posttranscriptional activation, and repression. XAC1 is an established posttranscriptional activator that does not bind mRNA directly but forms complexes with other poly(A)-binding proteins (e.g., MKT1 and PBP-1) (52, 58). The gene encoding XAC1 was among the top hits, and its overexpression increased the EC_50_ of melarsoprol (Fig. 6). While this may arise from a general increase in fitness, alternatively, the overexpression of XAC1 might have a secondary effect associated with increasing the translation of enzymes required for trypanothione biosynthesis (such as GSH1 itself) or other unidentified aspects of melarsoprol cell killing.

The AQP2 transporter of melarsoprol and pentamidine is localized to the flagellar pocket in bloodstream-form parasites (Fig. 7, turquoise rectangles) (34). The large number of proteins localizing to the flagellum (10 genes) identified in melarsoprol survivors presents the intriguing possibility that they function in aspects of drug transport. For example, overexpression of accessory proteins may result in reduced drug uptake that promotes resistance. It would be useful to determine if any of these proteins affect the transport of trypanocidal drugs in a manner that might contribute to resistance. Flagellum proteins, mitochondrial proteins, and other categories of hits identified here present new testable hypotheses for future investigations that will likely uncover novel trypanosomatid biology, drug targets, and alternative mechanisms of drug resistance (Fig. 6).

The functionality of the T. brucei ORFeome can be extended to generate additional genetic tools, such as yeast two-hybrid libraries, tagging libraries, and dominant negative genetic screening approaches (27, 30, 31, 59). Based on the conservation of orthologous gene clusters among kinetoplastida (3), we expect the ORFeome could be used in other trypanosomatids to generate orthologous gain-of-function libraries and other tools. The vast majority of genes overrepresented in melarsoprol survivor populations (∼80%) are conserved among sequenced trypanosomatid genomes. This supports the use of these tools to broadly expand our understanding of gene functions in this family of parasites. We see the GoF library as a powerful new tool that can complement existing RNAi knockdown approaches and expand our understanding of drug targets and pathways of resistance. The tools and discoveries arising from this study are expected to support broad advances in basic biology, pathogenesis, pathways of drug resistance, and the identification of the targets for compounds that selectively kill trypanosomatids.

## MATERIALS AND METHODS

Methods for ORFeome generation and assessment, gain-of-function library assessment, and bioinformatic analysis of melarsoprol survivor populations are located in Text S1 in the supplemental material.

10.1128/mSphere.00769-20.10 TEXT S1 ORFeome generation, NGS, and bioinformatics methods. Download Text S1, PDF file, 0.04 MB. Copyright © 2020 Carter et al. 2020 Carter et al. This content is distributed under the terms of the Creative Commons Attribution 4.0 International license.

### Gateway cloning and plasmids.

The pENTR library was generated by cloning each size-sorted PCR product pool into pDONR221 Gateway Entry vector according to the manufacturer’s specifications (Thermo Fisher Scientific, Waltham, MA) and transformed into ElectroMAX DH10B cells by electroporation (44). The resulting transformants were plated on large LB plates containing kanamycin and assessed for efficiency of transformation. Bacterial colonies were isolated from plates and grown in LB liquid cultures, which were split for maxi preps of plasmid and storage at −80°C in glycerol stocks. A T. brucei-specific pDEST Gateway vector, pSUN6 (Fig. S1), was generated by introducing a ccdB Gateway cassette into a pLEW type vector (46) for incorporation into the T. brucei genome based on rDNA spacer homology, blasticidin selection, and ORF transcription from an rRNA promoter repressed by two tetracycline operators. Pools of pENTR plasmids harboring size-sorted ORF populations were combined with pSUN6 in LR Clonase reactions and transformed into ElectroMAX DH10B cells by electroporation. The resulting transformants were plated on large LB plates containing ampicillin and assessed for efficiency of transformation; then, bacteria and DNA were isolated as described above for pENTR steps. The resulting plasmid libraries of pENTR and pTrypLib ORFeome Gateway cloning steps were assessed by NGS (Text S1). Following the initial assessment of “missing” ORFs from both pENTR and pTrypLib cloning libraries, “missing” PCR products were isolated from original plates, using a Perkin-Emer Janus Automated Workstation, to generate 8 new pools of size-sorted PCRs (Table S2), which underwent the same series of Gateway cloning reactions described above and subjected to NGS analysis. The final NGS-validated pTrypLib library plasmids were pooled to generate a single pTrypLib ORFeome for introduction into the T. brucei genome.

### T. brucei cell lines, transfections, and GoF parasite library generation.

Cell lines were generated from Lister 427 bloodstream-form trypanosomes derived from the “single marker” (SM) line (60) and maintained in HMI-9 medium (61) under appropriate drug selection when indicated. A landing pad (LP) cell line was generated using plasmids gifted to us by the Alsford Lab and validated for inducible gene expression, prior to transfection with pRPaSce* as described previously (6, 62). LP parasites harboring the I-SceI cut site and I-SceI endonuclease gene targeted at an rDNA spacer were doxycycline induced to permit I-SceI cutting prior to pTrypLib ORFeome transfection by AMAXA Nucleofector (63). To generate the T. brucei GoF library described here, four 100-ml flask cultures grown to ∼1 million cells/ml were AMAXA transfected with 10 μg pTrypLib DNA in four separate transfection reaction mixtures, which were then pooled into a single cell population in 500 ml of HMI-9 and recovered in a large roller flask, to which blasticidin was added 12 h posttransfection (Fig. 3C). An additional four transfections were completed in parallel with Tris-EDTA (TE; mock) to compare outgrowth with GoF library transfection. The resulting blasticidin-recovered GoF library population was expanded to an 800-ml culture at ∼1 million cells per ml and saved in aliquots of ∼25 million cells per vial for future genetic screens. Cells were also sampled prior to freezing for NGS analysis (GoF library, described below) and after freeze-thaw (GoF_L1).

Single-gene overexpression cell lines were generated by cloning ORFs of interest into pLEW100v5-BSD (plasmid 27658; Addgene, Watertown, MA), which, following validation, were digested with NotI and transfected into SM cells by AMAXA.

### Quantitative PCR assessment of ORF induction.

Individual cloned ORFs were selected randomly from pTrypLib colonies plated on LB originating from the pool “2_known,” ORFs confirmed by traditional DNA sequencing and DNAs arising from 4 individual ORF-harboring pTrypLib vectors were transfected into LP-harboring pRPaSce* by AMAXA as described above. This generated a “low-complexity library” following transfection and recovery, which was split into no Dox and +Dox conditions for 24 h; RNA was extracted and cDNA was prepared with Superscript III (18080044; Thermo Fisher) prior to qPCR analysis. Quantitative PCR data were produced on a Bio-Rad CFX96 real-time PCR detection system with iTaq Universal SYBR green Supermix (1725121; Bio-Rad). The forward primer anneals to the *attb1* site (5′-GGGGACAAGTTTGTACAAAAAAGCAGGCT) and reverse primers were unique to each ORF: *Tb927.8.2230* (primer, 5′-CACGGTTTTTGCCCATTCGT), *Tb927.1.4830* (primer, 5′-ATTTTTGCCGAAGCGCTTGA), *Tb927.10.12940* (primer, 5′-CCGTGATTCCCTGTCGACAT), and *Tb927.11.15810* (primer, 5′-CACCACCCGATGTACGGTAG). Because the forward primer anneals to the *attB1* site present only in the pTrypLib backbone, only those mRNAs arising from the exogenous ORFs integrated at the rDNA spacer, rather than the endogenous ORF, can be detected. Fold changes in transcripts level with Dox and without Dox were plotted (Fig. 3B).

### Melarsoprol GoF library screening.

GoF library cells were seeded for each condition at 1 × 10^5^ cell/ml, induced with doxycycline (1 μg/ml) for 24 h (for induced cultures, +Dox) (Fig. 4), and grown in HMI-9 medium containing Dox (when appropriate) plus melarsoprol at 17 nM or 35 nM (BoC Sciences, CAS 494-79-1). Melarsoprol stocks were diluted in dimethyl sulfoxide (DMSO), and cultures were treated for the duration indicated in the figures and text (Fig. 4C, bottom, time bar indicates time points of replenishment of melarsoprol and/or Dox and time points of sample harvest). GoF library-harboring cells were thawed from a single starting vial of approximately 25 million cells, propagated for 3 days prior to day −1 Dox induction, and on day 0, were split into 100-ml biological triplicates for untreated GoF_L (GoF_L, no Dox), uninduced (no Dox), and induced (+Dox) samples. Two elongation times were employed during PCR enrichment, GoF_L1 for 75 s and GoF_L2 for 20 s, to determine if amplification time resulted in a sequencing bias. Sequencing data were obtained in biological triplicates from GoF_L1 and GoF_L2 libraries (no melarsoprol treatment) and the two sets of melarsoprol-selected parasites (MEL1 and MEL2, NGS libraries were generated using 20-s elongation time). GoF library-harboring cells were recovered from each replicate and condition (GoF_L, MEL, and MEL), and genomic DNA was fragmented and prepared for ORFeome-specific Illumina sequencing (Fig. S3).

### EC_50_ determination by alamarBlue.

For EC_50_ determination, induced and uninduced cells were plated across a melarsoprol dilution series, and viability was assessed after 72 h using alamarBlue (Thermo Fisher) as previously described (11). All experiments were performed in biological triplicates.
