# Supplementary material for: A Trypanosoma brucei ORFeome-Based Gain-of-Function Library Identifies Genes That Promote Survival during Melarsoprol Treatment
Source: mSphere. 2020 Oct 7;5(5):e00769-20. doi: 10.1128/mSphere.00769-20 (PMC7568655; doi:10.1128/mSphere.00769-20)
Supplement: TABLE S1 [file mSphere.00769-20-st001.pdf]

Plate Name	Well Postion	Forward primer name	Forward primer sequence	Reverse primer name	Reverse primer sequence
0_known	A1	Tb927.9.4961_for	GGGGACAAGTTTGTACAAAAAAGCAGGCTATGCCACCGAAGAGCAACG	Tb927.9.4961_rev	GGGGACCACTTTGTACAAGAAAGCTGGGTTCAACGCACAGACTTGGGTTTG
0_known	A2	Tb927.10.14615_for	GGGGACAAGTTTGTACAAAAAAGCAGGCTATGCAGCAGGCGGACTATTATG	Tb927.10.14615_rev	GGGGACCACTTTGTACAAGAAAGCTGGGTTTACCATGAATTAACGACCAACTTAAATAT
0_known	A3	Tb927.10.4740_for	GGGGACAAGTTTGTACAAAAAAGCAGGCTATGCACCTTCAAGTTTATCTCGTGG	Tb927.10.4740_rev	GGGGACCACTTTGTACAAGAAAGCTGGGTTTAAAGTGGCTTTGGGCGTTTTTC
0_known	A4	Tb927.3.960_for	GGGGACAAGTTTGTACAAAAAAGCAGGCTATGGATTTCCTGGATGAAACCGTG	Tb927.3.960_rev	GGGGACCACTTTGTACAAGAAAGCTGGGTTCAAGCGCCGAGTACAACATTATT
0_known	A5	Tb927.11.1870_for	GGGGACAAGTTTGTACAAAAAAGCAGGCTATGGCGAAGGCATCTGCTG	Tb927.11.1870_rev	GGGGACCACTTTGTACAAGAAAGCTGGGTTCACGCCTTCTTAGCGGCG
0_known	A6	Tb927.8.7430_for	GGGGACAAGTTTGTACAAAAAAGCAGGCTATGGCCGACGAGGAACCA	Tb927.8.7430_rev	GGGGACCACTTTGTACAAGAAAGCTGGGTTTACACCAACGTTGGCCACAG
0_known	A8	Tb927.11.3705_for	GGGGACAAGTTTGTACAAAAAAGCAGGCTATGATCATTCCTGTTCGGTGTTTCA	Tb927.11.3705_rev	GGGGACCACTTTGTACAAGAAAGCTGGGTTTACACCGCAACACTGGAGTCA
0_known	A9	Tb927.10.13700_for	GGGGACAAGTTTGTACAAAAAAGCAGGCTATGCCGAGACCGTGCATTC	Tb927.10.13700_rev	GGGGACCACTTTGTACAAGAAAGCTGGGTTCATTTAGTTATATCAGACACCACCTGC
0_known	A10	Tb927.3.2650_for	GGGGACAAGTTTGTACAAAAAAGCAGGCTATGTCGAGTGACGAAGTTGCTG	Tb927.3.2650_rev	GGGGACCACTTTGTACAAGAAAGCTGGGTCTAAAAACTCCGGACACTCCCTCG
0_known	A11	Tb927.9.10250_for	GGGGACAAGTTTGTACAAAAAAGCAGGCTATGGATGCAAATGTACCGGCG	Tb927.9.10250_rev	GGGGACCACTTTGTACAAGAAAGCTGGGTCTATTCCTTTGGTACTTCCCGAATGTAC
0_known	A12	Tb927.11.1880_for	GGGGACAAGTTTGTACAAAAAAGCAGGCTATGGCGAAGACAACTGCTGC	Tb927.11.1880_rev	GGGGACCACTTTGTACAAGAAAGCTGGGTTCACGCCTTCTTAGCGGCG
0_known	A15	Tb927.4.2540_for	GGGGACAAGTTTGTACAAAAAAGCAGGCTATGCTTCTTAAGGTGAAGACTGTAAGC	Tb927.4.2540_rev	GGGGACCACTTTGTACAAGAAAGCTGGGTTCAGCAACCCGCGCGCAG
0_known	A16	Tb927.11.1800_for	GGGGACAAGTTTGTACAAAAAAGCAGGCTATGAACAACACAACCGCTACTGT	Tb927.11.1800_rev	GGGGACCACTTTGTACAAGAAAGCTGGGTTCACGCCTTCTTAGCGGCG
0_known	A17	Tb927.7.7380_for	GGGGACAAGTTTGTACAAAAAAGCAGGCTATGGATCCCTTCGTGTTTCTGC	Tb927.7.7380_rev	GGGGACCACTTTGTACAAGAAAGCTGGGTTCAGTTAACCTGATACGATTGGGGA
0_known	A18	Tb927.9.10170_for	GGGGACAAGTTTGTACAAAAAAGCAGGCTATGGATATAAAAGTGAAGAGTGCCCAC	Tb927.9.10170_rev	GGGGACCACTTTGTACAAGAAAGCTGGGTTTAGAATTCCCATGTCTGTCGGCA
0_known	A19	Tb927.11.14310_for	GGGGACAAGTTTGTACAAAAAAGCAGGCTATGCCCGCCAAGAAGCGC	Tb927.11.14310_rev	GGGGACCACTTTGTACAAGAAAGCTGGGTTTACACTTCAAGACCGACAATATCCAC
0_known	A20	Tb927.9.13070_for	GGGGACAAGTTTGTACAAAAAAGCAGGCTATGGACTACGAAGAACCAGGACA	Tb927.9.13070_rev	GGGGACCACTTTGTACAAGAAAGCTGGGTTTATTTCCCCTTACTCTTTTTCTTATCTTT
0_known	A21	Tb927.1.1170_for	GGGGACAAGTTTGTACAAAAAAGCAGGCTATGCTTTCGTATACAGTGAAGGAGGA	Tb927.1.1170_rev	GGGGACCACTTTGTACAAGAAAGCTGGGTTCAAATAGTGTCATATGTTGTGGGTTGC
0_known	A22	Tb927.3.1250_for	GGGGACAAGTTTGTACAAAAAAGCAGGCTATGCTGATTCCAGTCCGTTGC	Tb927.3.1250_rev	GGGGACCACTTTGTACAAGAAAGCTGGGTTTACTGCCGGCGTGGCAC
0_known	A23	Tb927.9.3530_for	GGGGACAAGTTTGTACAAAAAAGCAGGCTATGGCTTCGGTGAATATGGAGGA	Tb927.9.3530_rev	GGGGACCACTTTGTACAAGAAAGCTGGGTCTAAGAAGTGGTGGAAGCCACAC
0_known	A24	Tb927.6.2700_for	GGGGACAAGTTTGTACAAAAAAGCAGGCTATGAGCGTCACAACAAAGCAGA	Tb927.6.2700_rev	GGGGACCACTTTGTACAAGAAAGCTGGGTCTATATGCCAATAGGGTGAATGACACC
0_known	B1	Tb927.10.8700_for	GGGGACAAGTTTGTACAAAAAAGCAGGCTATGGTGTACATTGAGAACGGCAG	Tb927.10.8700_rev	GGGGACCACTTTGTACAAGAAAGCTGGGTTCATCCACTCGCGCAACCG
0_known	B2	Tb927.11.11435_for	GGGGACAAGTTTGTACAAAAAAGCAGGCTATGACGCAGCTCCCCTCAG	Tb927.11.11435_rev	GGGGACCACTTTGTACAAGAAAGCTGGGTTCATGACGACTTGAAAAGAAGCACA
0_known	B3	Tb927.11.711_for	GGGGACAAGTTTGTACAAAAAAGCAGGCTATGTCTACTGATCGGAAAGCTATTATTAAG	Tb927.11.711_rev	GGGGACCACTTTGTACAAGAAAGCTGGGTCTATCCTGACTTGAACAGAAGAATTGCA
0_known	B4	Tb927.5.4440_for	GGGGACAAGTTTGTACAAAAAAGCAGGCTATGTCTAAGCACCATGAATTCAACATTAAC	Tb927.5.4440_rev	GGGGACCACTTTGTACAAGAAAGCTGGGTTTACGTCACTGTCTTCCACAAAAGG
0_known	B5	Tb927.11.320_for	GGGGACAAGTTTGTACAAAAAAGCAGGCTATGATGAGTGATCGCAAGACCAATG	Tb927.11.320_rev	GGGGACCACTTTGTACAAGAAAGCTGGGTTCAACCTGTTTTCCACAGGAGAATG
0_known	B6	Tb927.9.13820_for	GGGGACAAGTTTGTACAAAAAAGCAGGCTATGGCCACCACATACGAAGAATTT	Tb927.9.13820_rev	GGGGACCACTTTGTACAAGAAAGCTGGGTTCATTTTCCGGGGAACTGGGC
0_known	B8	Tb927.9.13775_for	GGGGACAAGTTTGTACAAAAAAGCAGGCTATGGCCACTACATACGAAGAATTTGC	Tb927.9.13775_rev	GGGGACCACTTTGTACAAGAAAGCTGGGTTCATTTTCCGGGGAACTGGGC
0_known	B9	Tb927.9.13785_for	GGGGACAAGTTTGTACAAAAAAGCAGGCTATGGCCACCACATACGAAGAATTT	Tb927.9.13785_rev	GGGGACCACTTTGTACAAGAAAGCTGGGTTCACTTTCCGGGGAACTGGG
0_known	B11	Tb927.5.4030_for	GGGGACAAGTTTGTACAAAAAAGCAGGCTATGTCGGCACAGCAGGCA	Tb927.5.4030_rev	GGGGACCACTTTGTACAAGAAAGCTGGGTTTACTGGAATGGGTTCTGTTGAAGGA
0_known	B12	Tb927.4.2010_for	GGGGACAAGTTTGTACAAAAAAGCAGGCTATGGTGGAGGAAACGCTTGATG	Tb927.4.2010_rev	GGGGACCACTTTGTACAAGAAAGCTGGGTCTACAGGTTCGACGGGGGC
0_known	B13	Tb927.11.1370_for	GGGGACAAGTTTGTACAAAAAAGCAGGCTATGCCCTCTATCGCTTCGATGA	Tb927.11.1370_rev	GGGGACCACTTTGTACAAGAAAGCTGGGTTTAGCTCAGCAAACCATCGAGTTT
0_known	B14	Tb927.8.5380_for	GGGGACAAGTTTGTACAAAAAAGCAGGCTATGTCTCAAAATGAAGAAGCCCCTG	Tb927.8.5380_rev	GGGGACCACTTTGTACAAGAAAGCTGGGTTTAAACCCTGCCCACACGGT
0_known	B15	Tb927.11.16390_for	GGGGACAAGTTTGTACAAAAAAGCAGGCTATGTCTGGCCGGGACTACTTC	Tb927.11.16390_rev	GGGGACCACTTTGTACAAGAAAGCTGGGTCTATTGTGTCTTGGGCCGCTT
0_known	B16	Tb927.9.14480_for	GGGGACAAGTTTGTACAAAAAAGCAGGCTATGTCTGAGATAGAGGAAAGCTTCCA	Tb927.9.14480_rev	GGGGACCACTTTGTACAAGAAAGCTGGGTTCAAACATCTGAAAATCGTTGAATTACAAT
0_known	B17	Tb927.10.12000_for	GGGGACAAGTTTGTACAAAAAAGCAGGCTATGACAAACACCGTCAAACACTCA	Tb927.10.12000_rev	GGGGACCACTTTGTACAAGAAAGCTGGGTTTACTCTTCTTCTTCTTGCGTCACG
0_known	B18	Tb927.7.1340_for	GGGGACAAGTTTGTACAAAAAAGCAGGCTATGCTCCGCGTTTCCATTCC	Tb927.7.1340_rev	GGGGACCACTTTGTACAAGAAAGCTGGGTTCAATTCTGGATAACACCAAGCAACG
0_known	B19	Tb927.5.4260_for	GGGGACAAGTTTGTACAAAAAAGCAGGCTATGGCGAAGGGTAAGAAGAGTGG	Tb927.5.4260_rev	GGGGACCACTTTGTACAAGAAAGCTGGGTTTATGCATAACCGTACAGAATCTTGCC
0_known	B24	Tb927.2.2670_for	GGGGACAAGTTTGTACAAAAAAGCAGGCTATGGCAAAGGGCAAGAGAGTCG	Tb927.2.2670_rev	GGGGACCACTTTGTACAAGAAAGCTGGGTTCAGTCGTACCCGTAAAGAACCT
0_known	C7	Tb927.11.9700_for	GGGGACAAGTTTGTACAAAAAAGCAGGCTATGCCCTCTATTACTCAGGAGACG	Tb927.11.9700_rev	GGGGACCACTTTGTACAAGAAAGCTGGGTCTATGAAGGCACTTCGCTCGC
0_known	C8	Tb927.6.3490_for	GGGGACAAGTTTGTACAAAAAAGCAGGCTATGCACATTTCAACACCCACTCT	Tb927.6.3490_rev	GGGGACCACTTTGTACAAGAAAGCTGGGTTCAATCCTCCAACCTTACGCTGT
0_known	C9	Tb927.7.3120_for	GGGGACAAGTTTGTACAAAAAAGCAGGCTATGCCCGCGGCGGAGTCC	Tb927.7.3120_rev	GGGGACCACTTTGTACAAGAAAGCTGGGTTCACTCATTTGATCGCTCCGTCC
0_known	C10	Tb927.5.3745_for	GGGGACAAGTTTGTACAAAAAAGCAGGCTATGAGTAATACAAATTCGTCTGGGTGC	Tb927.5.3745_rev	GGGGACCACTTTGTACAAGAAAGCTGGGTTTACGAACGTGCAATACGTTCAGC
0_known	C11	Tb927.9.3370_for	GGGGACAAGTTTGTACAAAAAAGCAGGCTATGTCGGTAGTGGATGTTTATAGCGT	Tb927.9.3370_rev	GGGGACCACTTTGTACAAGAAAGCTGGGTTTAGTTGTCTTTGATGATATCTCTTAATTT
0_known	C12	Tb927.1.1770_for	GGGGACAAGTTTGTACAAAAAAGCAGGCTATGAATAACGCATTGGATCCGGC	Tb927.1.1770_rev	GGGGACCACTTTGTACAAGAAAGCTGGGTTCAGCCCCTTTCAATTTCGGC
0_known	C13	Tb927.10.4620_for	GGGGACAAGTTTGTACAAAAAAGCAGGCTATGCCGCTTCAGTACGACATC	Tb927.10.4620_rev	GGGGACCACTTTGTACAAGAAAGCTGGGTTCAGTTAATTGCGACGAGTTCCATT
0_known	C14	Tb927.11.5840_for	GGGGACAAGTTTGTACAAAAAAGCAGGCTATGAATGCAAACGACGAGATGAGC	Tb927.11.5840_rev	GGGGACCACTTTGTACAAGAAAGCTGGGTTCAGTTCAGCGAGTGGATCTCTAA
0_known	C15	Tb927.10.10320_for	GGGGACAAGTTTGTACAAAAAAGCAGGCTATGAAAGAGAAGTTGACTCTAGCTGATG	Tb927.10.10320_rev	GGGGACCACTTTGTACAAGAAAGCTGGGTCTATGCCACAACACCGTACACA
0_known	C16	Tb927.9.5220_for	GGGGACAAGTTTGTACAAAAAAGCAGGCTATGGGCGGCACCGGAGAG	Tb927.9.5220_rev	GGGGACCACTTTGTACAAGAAAGCTGGGTCTAAGTTCGCATAATGTGCTTGTTTTCG
0_known	C17	Tb927.8.2310_for	GGGGACAAGTTTGTACAAAAAAGCAGGCTATGCCTCCCAAGTATGACAATGTCC	Tb927.8.2310_rev	GGGGACCACTTTGTACAAGAAAGCTGGGTTCAGTTGTCCTGACATCGCAGT
0_known	C18	Tb927.2.4540_for	GGGGACAAGTTTGTACAAAAAAGCAGGCTATGGGCCACCAAAATATGCTTCA	Tb927.2.4540_rev	GGGGACCACTTTGTACAAGAAAGCTGGGTTCAATCGCGTTTCCGCTTGG
0_known	C19	Tb927.3.2030_for	GGGGACAAGTTTGTACAAAAAAGCAGGCTATGCCACAAACTGTAGATCAGGTTG	Tb927.3.2030_rev	GGGGACCACTTTGTACAAGAAAGCTGGGTTCATCGCAGTATCTCGAATGTATCAAAA
0_known	C20	Tb927.10.9620_for	GGGGACAAGTTTGTACAAAAAAGCAGGCTATGCGGCTGCCGCATCCG	Tb927.10.9620_rev	GGGGACCACTTTGTACAAGAAAGCTGGGTTTACACGAGGGGTAACAAAAAAAAAAAAGA
0_known	C21	Tb927.1.4570_for	GGGGACAAGTTTGTACAAAAAAGCAGGCTATGTTTGTTTTTATATGCCGTGACTTTGT	Tb927.1.4570_rev	GGGGACCACTTTGTACAAGAAAGCTGGGTTTAGTGTGTAATGATGTCATGCAACTGG
0_known	C22	Tb927.2.5850_for	GGGGACAAGTTTGTACAAAAAAGCAGGCTATGTCTGGTGAGGAACCCACTAAA	Tb927.2.5850_rev	GGGGACCACTTTGTACAAGAAAGCTGGGTCTATACAGTGCTCTGCGGCAAC
0_known	C23	Tb927.10.10600_for	GGGGACAAGTTTGTACAAAAAAGCAGGCTATGAAAGAATTAAAAGAACTTCTGGATCAG	Tb927.10.10600_rev	GGGGACCACTTTGTACAAGAAAGCTGGGTTTATTTTTTTTCGTTTGATTTGTTTTGCGC
0_known	C24	Tb927.10.10570_for	GGGGACAAGTTTGTACAAAAAAGCAGGCTATGGCCACTCCTAAGAGCACA	Tb927.10.10570_rev	GGGGACCACTTTGTACAAGAAAGCTGGGTTTAGCTGGAAGCGTGTGACAC
0_known	D1	Tb927.7.3420_for	GGGGACAAGTTTGTACAAAAAAGCAGGCTATGTCGCGAAATGACTGCGT	Tb927.7.3420_rev	GGGGACCACTTTGTACAAGAAAGCTGGGTTCAAACAACATCCAACAGCGTGA
0_known	D7	Tb927.10.4690_for	GGGGACAAGTTTGTACAAAAAAGCAGGCTATGACGTCCAACGTGAAGGAAC	Tb927.10.4690_rev	GGGGACCACTTTGTACAAGAAAGCTGGGTCTACAACCCCTTGAAGACGACAATT
0_known	D10	Tb927.10.10451_for	GGGGACAAGTTTGTACAAAAAAGCAGGCTATGGCCACTCCTAGGAGCAC	Tb927.10.10451_rev	GGGGACCACTTTGTACAAGAAAGCTGGGTTTAGCTGGAAGCGTGTGACAC
0_known	D18	Tb927.11.9040_for	GGGGACAAGTTTGTACAAAAAAGCAGGCTATGTCCCGTGTCCCTATTAGCG	Tb927.11.9040_rev	GGGGACCACTTTGTACAAGAAAGCTGGGTTCACCAATCGTCATCCATGCG
0_known	D19	Tb927.11.7040_for	GGGGACAAGTTTGTACAAAAAAGCAGGCTATGCGGCGGTGTGTTTATTATTGC	Tb927.11.7040_rev	GGGGACCACTTTGTACAAGAAAGCTGGGTTCAGTTGTTCGTCAAAGTTCGCT
0_known	D20	Tb927.5.3210_for	GGGGACAAGTTTGTACAAAAAAGCAGGCTATGGACGAACCCACTCATAACTCC	Tb927.5.3210_rev	GGGGACCACTTTGTACAAGAAAGCTGGGTTCACGCCATGCACCAAAGAC
0_known	D22	Tb927.8.5120_for	GGGGACAAGTTTGTACAAAAAAGCAGGCTATGCCACCAAAGGAGCGTG	Tb927.8.5120_rev	GGGGACCACTTTGTACAAGAAAGCTGGGTTTAGTCCTTTAATGTCTCGAGGTATGCG
0_known	D23	Tb927.6.4140_for	GGGGACAAGTTTGTACAAAAAAGCAGGCTATGCCAAACCTCGTTGTCACTG	Tb927.6.4140_rev	GGGGACCACTTTGTACAAGAAAGCTGGGTCTAAGAGCGAGTGAAATAAAACTTAATGCA
0_known	D24	Tb927.7.4060_for	GGGGACAAGTTTGTACAAAAAAGCAGGCTATGTCCGATATCACGTACGAGAACG	Tb927.7.4060_rev	GGGGACCACTTTGTACAAGAAAGCTGGGTTCATGACTGAGGAATAGGATTGGCT
0_known	E1	Tb927.11.7740_for	GGGGACAAGTTTGTACAAAAAAGCAGGCTATGGTGAACGAGTACGATGAAGC	Tb927.11.7740_rev	GGGGACCACTTTGTACAAGAAAGCTGGGTCTAAATAAACATAGTTTCTGTAGAGAAAGT
0_known	E2	Tb927.4.890_for	GGGGACAAGTTTGTACAAAAAAGCAGGCTATGAACACGGAGGGGCTCC	Tb927.4.890_rev	GGGGACCACTTTGTACAAGAAAGCTGGGTTTACTTCTTTGGCTTCTTACGGGAAC
0_known	E3	Tb927.8.690_for	GGGGACAAGTTTGTACAAAAAAGCAGGCTATGTCCGAAAAGCTGAGAGCAG	Tb927.8.690_rev	GGGGACCACTTTGTACAAGAAAGCTGGGTTCACGCGAGCCTCTTGATAATG
0_known	E4	Tb927.4.4320_for	GGGGACAAGTTTGTACAAAAAAGCAGGCTATGTTTTCTGTGTGTTACGTGACGA	Tb927.4.4320_rev	GGGGACCACTTTGTACAAGAAAGCTGGGTTTAGCGGCAACTGCAACCG
0_known	E5	Tb927.10.12090_for	GGGGACAAGTTTGTACAAAAAAGCAGGCTATGCCACCGCGGGCTCGA	Tb927.10.12090_rev	GGGGACCACTTTGTACAAGAAAGCTGGGTTCACCGTTGAACACGTTGTGG
0_known	E6	Tb927.11.13140_for	GGGGACAAGTTTGTACAAAAAAGCAGGCTATGCTGCGTCGCGCTGGC	Tb927.11.13140_rev	GGGGACCACTTTGTACAAGAAAGCTGGGTCTACCAGCCGCGATGGTAGA
0_known	E8	Tb927.8.6950_for	GGGGACAAGTTTGTACAAAAAAGCAGGCTATGGAATTTAACGCATCAACTACCAACG	Tb927.8.6950_rev	GGGGACCACTTTGTACAAGAAAGCTGGGTTCAAGATCTGATCTCTTGCACGACA
0_known	E10	Tb927.11.3660_for	GGGGACAAGTTTGTACAAAAAAGCAGGCTATGGCAGATAACCACGAAGAACC	Tb927.11.3660_rev	GGGGACCACTTTGTACAAGAAAGCTGGGTCTAATCCAGTTTAACGAAGAAGACAGTCA
0_known	E12	Tb927.7.5900_for	GGGGACAAGTTTGTACAAAAAAGCAGGCTATGAAGTATAACTTCAAGGATTCACATTCT	Tb927.7.5900_rev	GGGGACCACTTTGTACAAGAAAGCTGGGTTTATCCAAATGCCGCCTCACC
0_known	E14	Tb927.10.12100_for	GGGGACAAGTTTGTACAAAAAAGCAGGCTATGCCACCGCGGGCTCGA	Tb927.10.12100_rev	GGGGACCACTTTGTACAAGAAAGCTGGGTTCAACGTTGCACACGTTGTGG
0_known	E17	Tb927.6.4810_for	GGGGACAAGTTTGTACAAAAAAGCAGGCTATGGTTCAACGCATCCGCG	Tb927.6.4810_rev	GGGGACCACTTTGTACAAGAAAGCTGGGTTCACGAGCCGGCAGCGGG
0_known	E18	Tb927.11.4450_for	GGGGACAAGTTTGTACAAAAAAGCAGGCTATGTCAGAAAAAGGTGGTGACGAC	Tb927.11.4450_rev	GGGGACCACTTTGTACAAGAAAGCTGGGTCTACTCCTTTTTATCAGCGTTTTCCAAC
0_known	E19	Tb927.3.3480_for	GGGGACAAGTTTGTACAAAAAAGCAGGCTATGGGTGAGCCAAAACAGACG	Tb927.3.3480_rev	GGGGACCACTTTGTACAAGAAAGCTGGGTCTACCCCGCTTGCGTGTC
0_known	E20	Tb927.10.7470_for	GGGGACAAGTTTGTACAAAAAAGCAGGCTATGTCCGATGAGCGAATTCTCCT	Tb927.10.7470_rev	GGGGACCACTTTGTACAAGAAAGCTGGGTTCAATCCTCGGCCTTGACCTC
0_known	E21	Tb927.7.1470_for	GGGGACAAGTTTGTACAAAAAAGCAGGCTATGATGCGCCGCCTTGCC	Tb927.7.1470_rev	GGGGACCACTTTGTACAAGAAAGCTGGGTTTACGAGAAGAGCATCAAGAACGC
0_known	E22	Tb927.6.4340_for	GGGGACAAGTTTGTACAAAAAAGCAGGCTATGTCTGTCAAATCCGAGCGGC	Tb927.6.4340_rev	GGGGACCACTTTGTACAAGAAAGCTGGGTCTATCGGAGGGGAGTGTCCAAA
0_known	E23	Tb927.10.13110_for	GGGGACAAGTTTGTACAAAAAAGCAGGCTATGGCTGCAATTGGAGGCG	Tb927.10.13110_rev	GGGGACCACTTTGTACAAGAAAGCTGGGTCTACGCACTAACACCATTTGCATTG
0_known	E24	Tb927.10.1570_for	GGGGACAAGTTTGTACAAAAAAGCAGGCTATGATGCGCCGTCTCGCT	Tb927.10.1570_rev	GGGGACCACTTTGTACAAGAAAGCTGGGTCTACGAGAAGAGCATCAGAAACGC
0_known	F1	Tb927.11.5280_for	GGGGACAAGTTTGTACAAAAAAGCAGGCTATGATGCGCCGCCTTGCC	Tb927.11.5280_rev	GGGGACCACTTTGTACAAGAAAGCTGGGTCTACGAGAAGAGCATCAGAAACGC
0_known	F2	Tb927.11.13650_for	GGGGACAAGTTTGTACAAAAAAGCAGGCTATGGAGGGGTTTGTATCGCTGA	Tb927.11.13650_rev	GGGGACCACTTTGTACAAGAAAGCTGGGTTCAGCTTGAGAATAGAAAGTATATAATAAT
0_known	F3	Tb927.10.14110_for	GGGGACAAGTTTGTACAAAAAAGCAGGCTATGGCCTCGTTCGGCTCT	Tb927.10.14110_rev	GGGGACCACTTTGTACAAGAAAGCTGGGTTTACGACACTGCGCTTTCACG
0_known	F4	Tb927.3.3720_for	GGGGACAAGTTTGTACAAAAAAGCAGGCTATGCAGCCGCAGAGCTCC	Tb927.3.3720_rev	GGGGACCACTTTGTACAAGAAAGCTGGGTTCACCGGGACTTCAGCATTATATATATCA
0_known	F5	Tb927.7.5910_for	GGGGACAAGTTTGTACAAAAAAGCAGGCTATGAGTAAAAAAGATAGCAAGTACAAAATG	Tb927.7.5910_rev	GGGGACCACTTTGTACAAGAAAGCTGGGTTTAGCATCCAAATGTCGCCTCAC
0_known	F6	Tb927.7.4070_for	GGGGACAAGTTTGTACAAAAAAGCAGGCTATGTCCGATATCACGTACGAGAACG	Tb927.7.4070_rev	GGGGACCACTTTGTACAAGAAAGCTGGGTTTAGGTGGGAACGGATTTCCACT
0_known	F7	Tb927.11.11975_for	GGGGACAAGTTTGTACAAAAAAGCAGGCTATGGACCGGAGACTCATCGAAA	Tb927.11.11975_rev	GGGGACCACTTTGTACAAGAAAGCTGGGTTTACATATCCACAGATGGCTCCTTAGAG
0_known	F8	Tb927.8.6450_for	GGGGACAAGTTTGTACAAAAAAGCAGGCTATGTCCCACAACCTATTTACTGAGGA	Tb927.8.6450_rev	GGGGACCACTTTGTACAAGAAAGCTGGGTTTATGCGGTGGCCTCGACG
0_known	F11	Tb927.3.3100_for	GGGGACAAGTTTGTACAAAAAAGCAGGCTATGGGCAAAGAAAACATGAAGGGC	Tb927.3.3100_rev	GGGGACCACTTTGTACAAGAAAGCTGGGTTCACTGCTTATCCTCCACAAAAATAATGT
0_known	F12	Tb927.6.480_for	GGGGACAAGTTTGTACAAAAAAGCAGGCTATGGCACCTCGTTCCCTTTATCT	Tb927.6.480_rev	GGGGACCACTTTGTACAAGAAAGCTGGGTTTAGAATGCGGCAACGAGACC
0_known	F13	Tb927.1.2230_for	GGGGACAAGTTTGTACAAAAAAGCAGGCTATGGGTTGTGGTGGCTCAAAG	Tb927.1.2230_rev	GGGGACCACTTTGTACAAGAAAGCTGGGTCTATTGCCTGTCTCCATCAGTAATGG
0_known	F14	Tb927.10.8320_for	GGGGACAAGTTTGTACAAAAAAGCAGGCTATGTTCTCCTGTGCGCTGAGA	Tb927.10.8320_rev	GGGGACCACTTTGTACAAGAAAGCTGGGTCTAGTGGTGACCCAGATCCACA
0_known	F15	Tb927.8.4280_for	GGGGACAAGTTTGTACAAAAAAGCAGGCTATGTCCTTTCAAGAGATTGGAACGG	Tb927.8.4280_rev	GGGGACCACTTTGTACAAGAAAGCTGGGTTTAGTGGCCACCGCCGCC
0_known	F16	Tb927.9.12820_for	GGGGACAAGTTTGTACAAAAAAGCAGGCTATGCAAACAGATTTTGGAGATGCCA	Tb927.9.12820_rev	GGGGACCACTTTGTACAAGAAAGCTGGGTTTAATATTGATAGAGGCCGAATACCGTCA
0_known	F17	Tb927.10.6670_for	GGGGACAAGTTTGTACAAAAAAGCAGGCTATGATGGAGGACGATGAGGGTTT	Tb927.10.6670_rev	GGGGACCACTTTGTACAAGAAAGCTGGGTTTACTCCTTTGCATCATTCGCATTTAGC
0_known	F18	Tb927.9.10280_for	GGGGACAAGTTTGTACAAAAAAGCAGGCTATGATCTTCCCCAGTGCAGCA	Tb927.9.10280_rev	GGGGACCACTTTGTACAAGAAAGCTGGGTTCACTTGGCTACCCACTCATTAGG
0_known	F19	Tb927.11.7707_for	GGGGACAAGTTTGTACAAAAAAGCAGGCTATGGTGAACGAGTACCATGAAGC	Tb927.11.7707_rev	GGGGACCACTTTGTACAAGAAAGCTGGGTCTACTCCCAGTACAGCGCATACA
0_known	F20	Tb927.11.4460_for	GGGGACAAGTTTGTACAAAAAAGCAGGCTATGACGACAGGAAAGAGCGACC	Tb927.11.4460_rev	GGGGACCACTTTGTACAAGAAAGCTGGGTTTACGCTGTAGCCTTCTCCTTCT
0_known	F21	Tb927.8.5180_for	GGGGACAAGTTTGTACAAAAAAGCAGGCTATGGATGCGTTGACGACCAG	Tb927.8.5180_rev	GGGGACCACTTTGTACAAGAAAGCTGGGTTCAATCCTGAGGTACGGAAGGTT
0_known	F22	Tb927.10.11300_for	GGGGACAAGTTTGTACAAAAAAGCAGGCTATGTCTCACTACCTTTTGGTCACTTTC	Tb927.10.11300_rev	GGGGACCACTTTGTACAAGAAAGCTGGGTTCAGCTATCCTCAGCAAGAAGTTTATTTAG
0_known	F23	Tb927.10.8950_for	GGGGACAAGTTTGTACAAAAAAGCAGGCTATGCTTCGTGTTTCTCGATTATCCC	Tb927.10.8950_rev	GGGGACCACTTTGTACAAGAAAGCTGGGTCTACTTTCGCATCTGTTTCTGCTGT
0_known	F24	Tb927.10.15150_for	GGGGACAAGTTTGTACAAAAAAGCAGGCTATGTTTTTCTGTCCATTCTGTGGAACG	Tb927.10.15150_rev	GGGGACCACTTTGTACAAGAAAGCTGGGTCTAATCCTGCTTCCACTGGAAGC
0_known	G1	Tb927.10.13650_for	GGGGACAAGTTTGTACAAAAAAGCAGGCTATGGATAGTGAGGAAGGTGAGTTCG	Tb927.10.13650_rev	GGGGACCACTTTGTACAAGAAAGCTGGGTTTATTGATCTCGCTTTTCCCATGCT
0_known	G2	Tb927.11.13960_for	GGGGACAAGTTTGTACAAAAAAGCAGGCTATGAGTGTTAGGAAGCCTATAACGCC	Tb927.11.13960_rev	GGGGACCACTTTGTACAAGAAAGCTGGGTTTACTTCCCGCGGTTACGCT
0_known	G4	Tb927.1.4550_for	GGGGACAAGTTTGTACAAAAAAGCAGGCTATGTTTGTTTTTATATGCCGTGACTTTGT	Tb927.1.4550_rev	GGGGACCACTTTGTACAAGAAAGCTGGGTTCATGCAACTGGAAGAGCAGGG
0_known	G6	Tb927.10.8970_for	GGGGACAAGTTTGTACAAAAAAGCAGGCTATGCTTCGTGTTTCTCGATTATCCC	Tb927.10.8970_rev	GGGGACCACTTTGTACAAGAAAGCTGGGTTCACTTACTTCGCATCTGTTTCTGC
0_known	G7	Tb927.11.16150_for	GGGGACAAGTTTGTACAAAAAAGCAGGCTATGTCGACAAGTGAGGGCAATG	Tb927.11.16150_rev	GGGGACCACTTTGTACAAGAAAGCTGGGTTTATTGTTTAGACAAAGTTAGCTGTGCAC
0_known	G10	Tb927.3.1780_for	GGGGACAAGTTTGTACAAAAAAGCAGGCTATGCTTTCCCAATATCTCAGGCG	Tb927.3.1780_rev	GGGGACCACTTTGTACAAGAAAGCTGGGTCTATCCACCTTTAACTGAAGCTGTTGC
0_known	G11	Tb927.1.2260_for	GGGGACAAGTTTGTACAAAAAAGCAGGCTATGGGTTGCGGTGGCTCA	Tb927.1.2260_rev	GGGGACCACTTTGTACAAGAAAGCTGGGTTTATTTCCTGCCAGATCCCCTGC
0_known	G12	Tb927.11.12100_for	GGGGACAAGTTTGTACAAAAAAGCAGGCTATGTTCAACCAGTTTTCGAAGATGCC	Tb927.11.12100_rev	GGGGACCACTTTGTACAAGAAAGCTGGGTTTAAGCAGTAATACACGTCGGTGG
0_known	G13	Tb927.3.720_for	GGGGACAAGTTTGTACAAAAAAGCAGGCTATGCAGGGCTATTTTGCAGTAAATCC	Tb927.3.720_rev	GGGGACCACTTTGTACAAGAAAGCTGGGTTTATGCCATGGGCGGAACATC
0_known	G14	Tb927.6.3290_for	GGGGACAAGTTTGTACAAAAAAGCAGGCTATGGATGATGATAAACTTGTGATGTTTGAC	Tb927.6.3290_rev	GGGGACCACTTTGTACAAGAAAGCTGGGTCTACTCACGCGAGGCGTG
0_known	G15	Tb927.1.2160_for	GGGGACAAGTTTGTACAAAAAAGCAGGCTATGGGTTGCATTCAGTCGACAG	Tb927.1.2160_rev	GGGGACCACTTTGTACAAGAAAGCTGGGTTTATAGACGATATTGTTCTTCAGATCCTGC
0_known	G16	Tb927.10.15580_for	GGGGACAAGTTTGTACAAAAAAGCAGGCTATGGCGGGCACCACCGCT	Tb927.10.15580_rev	GGGGACCACTTTGTACAAGAAAGCTGGGTTTACATTGACGTTTGTCCCAGCTT
0_known	G17	Tb927.10.4950_for	GGGGACAAGTTTGTACAAAAAAGCAGGCTATGGAAGGTGTAGCTGATATTCCCT	Tb927.10.4950_rev	GGGGACCACTTTGTACAAGAAAGCTGGGTTCATAGGAGTAGCTTTTTCTTTCGATGC
0_known	G18	Tb927.10.10420_for	GGGGACAAGTTTGTACAAAAAAGCAGGCTATGCTGCGCCGCATTTTTG	Tb927.10.10420_rev	GGGGACCACTTTGTACAAGAAAGCTGGGTTCAGAGAAGATCCTTCTCCTTTAGAAGC
0_known	G19	Tb927.11.200_for	GGGGACAAGTTTGTACAAAAAAGCAGGCTATGATACAGTCGGATATTGTTTCGTATGTT	Tb927.11.200_rev	GGGGACCACTTTGTACAAGAAAGCTGGGTTCAGTGCTGAACGTCCAGCA
0_known	G20	Tb927.9.15420_for	GGGGACAAGTTTGTACAAAAAAGCAGGCTATGGTCAAGCCGTTTGTGCC	Tb927.9.15420_rev	GGGGACCACTTTGTACAAGAAAGCTGGGTTTACACCTGGACCTTCCTCAACTT
0_known	G21	Tb927.11.16200_for	GGGGACAAGTTTGTACAAAAAAGCAGGCTATGGATCGCAAGCTTGTTGAGATG	Tb927.11.16200_rev	GGGGACCACTTTGTACAAGAAAGCTGGGTTCATGATGTTTGAGCGGGCC
0_known	G22	Tb927.11.2200_for	GGGGACAAGTTTGTACAAAAAAGCAGGCTATGTCCACCTCACATGGCATGC	Tb927.11.2200_rev	GGGGACCACTTTGTACAAGAAAGCTGGGTCTATTCAACATCCAAACCTTTGCTCTG
0_known	G23	Tb927.11.7280_for	GGGGACAAGTTTGTACAAAAAAGCAGGCTATGGAGTCCACTTTGACGCAGT	Tb927.11.7280_rev	GGGGACCACTTTGTACAAGAAAGCTGGGTTTAATTGTCTTGATGGTCGCTGCT
0_known	G24	Tb927.1.2450_for	GGGGACAAGTTTGTACAAAAAAGCAGGCTATGTCGAGGACCAAGGAAACCG	Tb927.1.2450_rev	GGGGACCACTTTGTACAAGAAAGCTGGGTTTATGCACGTTCACCGCGT
0_known	H2	Tb927.11.6640_for	GGGGACAAGTTTGTACAAAAAAGCAGGCTATGACAACGTCACAACTCCCAAA	Tb927.11.6640_rev	GGGGACCACTTTGTACAAGAAAGCTGGGTCTAGGTAGCAAAAATGTAGCACAATAAAAG
0_known	H4	Tb927.1.3820_for	GGGGACAAGTTTGTACAAAAAAGCAGGCTATGGCGACTGGTCGACTAAAAAAC	Tb927.1.3820_rev	GGGGACCACTTTGTACAAGAAAGCTGGGTTTACTGGCAGCCGAGTGCT
0_known	H9	Tb927.7.2830_for	GGGGACAAGTTTGTACAAAAAAGCAGGCTATGGCAACACCCAAACAGGC	Tb927.7.2830_rev	GGGGACCACTTTGTACAAGAAAGCTGGGTCTAGACGCTTGGCGTCGC

0_known	H14	Tb927.7.3520_for	GGGGACAAGTTTGTACAAAAAAGCAGGCTATGTCTGCAACCACAAAACAAGC	Tb927.7.3520_rev	GGGGACCACTTTGTACAAGAAAGCTGGGTCTAATGCAGAGCTTTCGACCTCTT
0_known	H16	Tb927.7.3320_for	GGGGACAAGTTTGTACAAAAAAGCAGGCTATGCCTTCACACTACCGGTACC	Tb927.7.3320_rev	GGGGACCACTTTGTACAAGAAAGCTGGGTTCAACCAAATGTATTTTCCACACCATACG
0_known	H18	Tb927.7.2820_for	GGGGACAAGTTTGTACAAAAAAGCAGGCTATGGCAACACCCAAGCAGG	Tb927.7.2820_rev	GGGGACCACTTTGTACAAGAAAGCTGGGTCTAGACGCTTGGCGTCGC
0_known	H19	Tb927.11.8890_for	GGGGACAAGTTTGTACAAAAAAGCAGGCTATGAGTTTTCACCCTCGGGATGC	Tb927.11.8890_rev	GGGGACCACTTTGTACAAGAAAGCTGGGTCTAGTGCCCCTCAGGGTCC
0_known	I1	Tb927.5.580_for	GGGGACAAGTTTGTACAAAAAAGCAGGCTATGCAGCAGGTTCCACCCG	Tb927.5.580_rev	GGGGACCACTTTGTACAAGAAAGCTGGGTTTAAGATTGCTGCTGACGTCTTTGG
0_known	I2	Tb927.7.5760_for	GGGGACAAGTTTGTACAAAAAAGCAGGCTATGTCGGAATACGCAGATTGCAC	Tb927.7.5760_rev	GGGGACCACTTTGTACAAGAAAGCTGGGTTTACTCCTTTTCACTTAACCAGCGATAAT
0_known	I3	Tb927.7.5790_for	GGGGACAAGTTTGTACAAAAAAGCAGGCTATGAGGAACTCATTTGCACTTCTGC	Tb927.7.5790_rev	GGGGACCACTTTGTACAAGAAAGCTGGGTTCACATGTTGCTTTTAACAAATTCCTTCA
0_known	I4	Tb927.3.5180_for	GGGGACAAGTTTGTACAAAAAAGCAGGCTATGGCCATGTCTGGTGTTTCTG	Tb927.3.5180_rev	GGGGACCACTTTGTACAAGAAAGCTGGGTTTACCGGTTCGACTTCACTTTGC
0_known	I5	Tb927.11.3310_for	GGGGACAAGTTTGTACAAAAAAGCAGGCTATGGTTGAGGTACCGCGTAATTTC	Tb927.11.3310_rev	GGGGACCACTTTGTACAAGAAAGCTGGGTTCAATACGTTGAACCCTCCTCCG
0_known	I6	Tb927.9.9840_for	GGGGACAAGTTTGTACAAAAAAGCAGGCTATGCGTCGCGCACTGTTT	Tb927.9.9840_rev	GGGGACCACTTTGTACAAGAAAGCTGGGTCTATTCTATAAATTTGTTGTATTCATCGGC
0_known	I7	Tb927.11.1495_for	GGGGACAAGTTTGTACAAAAAAGCAGGCTATGTCCTTTTTCCAGTTGGATTGGG	Tb927.11.1495_rev	GGGGACCACTTTGTACAAGAAAGCTGGGTTTAGCTGTTTCCCCCAGATACGT
0_known	I8	Tb927.11.14950_for	GGGGACAAGTTTGTACAAAAAAGCAGGCTATGGCCTTCAACCAACGCTTT	Tb927.11.14950_rev	GGGGACCACTTTGTACAAGAAAGCTGGGTCTACTGCTGCAGATGGTTATTATCTAACC
0_known	I9	Tb927.9.11380_for	GGGGACAAGTTTGTACAAAAAAGCAGGCTATGGGAAAGGACAAGGCCAACG	Tb927.9.11380_rev	GGGGACCACTTTGTACAAGAAAGCTGGGTCTACACGATAGCTGGAGCGTGG
0_known	I10	Tb927.10.15350_for	GGGGACAAGTTTGTACAAAAAAGCAGGCTATGGCGCAAATGAAGAAAATAACACC	Tb927.10.15350_rev	GGGGACCACTTTGTACAAGAAAGCTGGGTTTAGTTACGCTCGCCTCGGA
0_known	I11	Tb927.9.11410_for	GGGGACAAGTTTGTACAAAAAAGCAGGCTATGGGAAAGGACAAGGCCAACG	Tb927.9.11410_rev	GGGGACCACTTTGTACAAGAAAGCTGGGTCTAGACGATGGCTGGAGCGT
0_known	I12	Tb927.1.2150_for	GGGGACAAGTTTGTACAAAAAAGCAGGCTATGGGTCTATTTCTGAGCAAACAAGG	Tb927.1.2150_rev	GGGGACCACTTTGTACAAGAAAGCTGGGTTTAAAACGATGTCGCCTTGAAGGG
0_known	I13	Tb927.10.6070_for	GGGGACAAGTTTGTACAAAAAAGCAGGCTATGGCTGATAACATGCAGATGTCAA	Tb927.10.6070_rev	GGGGACCACTTTGTACAAGAAAGCTGGGTTTATTTCACAGGGCAGGCACG
0_known	I14	Tb927.10.10260_for	GGGGACAAGTTTGTACAAAAAAGCAGGCTATGGCACCTCGTTCCCTTTATCT	Tb927.10.10260_rev	GGGGACCACTTTGTACAAGAAAGCTGGGTTTAGAATGCGGCAACGAGAGC
0_known	I15	Tb927.9.11230_for	GGGGACAAGTTTGTACAAAAAAGCAGGCTATGTCACGAGCATCTATAATGAAGGAAG	Tb927.9.11230_rev	GGGGACCACTTTGTACAAGAAAGCTGGGTCTACGCACTCTCATGCTCTGTAAAG
0_known	I16	Tb927.3.3330_for	GGGGACAAGTTTGTACAAAAAAGCAGGCTATGTGGGACCCTTTTCGTGATATTG	Tb927.3.3330_rev	GGGGACCACTTTGTACAAGAAAGCTGGGTTTACTGAATCGTTACGGATGTTGCA
0_known	I17	Tb927.7.4480_for	GGGGACAAGTTTGTACAAAAAAGCAGGCTATGTCCAACTGCATTTTCTGTAAGATTG	Tb927.7.4480_rev	GGGGACCACTTTGTACAAGAAAGCTGGGTTCACAACTTCTCGAGAGCCTCA
0_known	I18	Tb927.11.7900_for	GGGGACAAGTTTGTACAAAAAAGCAGGCTATGATTCGCGCTTCCATAGTTAAGC	Tb927.11.7900_rev	GGGGACCACTTTGTACAAGAAAGCTGGGTCTAAAAGTCATCGCTGAAGCTCTGG
0_known	I19	Tb927.11.1000_for	GGGGACAAGTTTGTACAAAAAAGCAGGCTATGGATTTATTAGGAAGCCAAATCGCT	Tb927.11.1000_rev	GGGGACCACTTTGTACAAGAAAGCTGGGTCTACAGCTGCATACTCGCCTCT
0_known	I20	Tb927.10.12420_for	GGGGACAAGTTTGTACAAAAAAGCAGGCTATGGACTCTTTATACAGCTGGAATGATTC	Tb927.10.12420_rev	GGGGACCACTTTGTACAAGAAAGCTGGGTTTACACATTTCGGTTACCCCGGG
0_known	I21	Tb927.11.7350_for	GGGGACAAGTTTGTACAAAAAAGCAGGCTATGCCGCCAACTAAAGGAGGA	Tb927.11.7350_rev	GGGGACCACTTTGTACAAGAAAGCTGGGTCTACAGTACCTTGGAGTACGCCT
0_known	I22	Tb927.3.3760_for	GGGGACAAGTTTGTACAAAAAAGCAGGCTATGTCTGGCCTCACTAAGTATCTTCC	Tb927.3.3760_rev	GGGGACCACTTTGTACAAGAAAGCTGGGTTCAGTTGGGCCACGGAAAGT
0_known	I23	Tb927.3.3780_for	GGGGACAAGTTTGTACAAAAAAGCAGGCTATGTCTGGCCTCGCTAAGTATCT	Tb927.3.3780_rev	GGGGACCACTTTGTACAAGAAAGCTGGGTTCAGTTGGGCCACGGAAAGT
0_known	I24	Tb927.4.3510_for	GGGGACAAGTTTGTACAAAAAAGCAGGCTATGTCTGAGCGTGATGAGGATGA	Tb927.4.3510_rev	GGGGACCACTTTGTACAAGAAAGCTGGGTTCAGATGTTTGTGTAGCGCTCG
0_known	J2	Tb927.1.1210_for	GGGGACAAGTTTGTACAAAAAAGCAGGCTATGGGTTACACACGGGAGCG	Tb927.1.1210_rev	GGGGACCACTTTGTACAAGAAAGCTGGGTTTACTTCATTCTTGCACGCCCC
0_known	J3	Tb927.11.12680_for	GGGGACAAGTTTGTACAAAAAAGCAGGCTATGGCCGCAACAAACTCACC	Tb927.11.12680_rev	GGGGACCACTTTGTACAAGAAAGCTGGGTTCATACCAGAACACCCCTGCTG
0_known	J4	Tb927.10.8280_for	GGGGACAAGTTTGTACAAAAAAGCAGGCTATGGAGGCGCTTCGCGTT	Tb927.10.8280_rev	GGGGACCACTTTGTACAAGAAAGCTGGGTCTATCCGTCAAACATCCTTTTTATTTTGGT
0_known	J5	Tb927.11.11100_for	GGGGACAAGTTTGTACAAAAAAGCAGGCTATGACCGATATAGTGGACCTGGAAC	Tb927.11.11100_rev	GGGGACCACTTTGTACAAGAAAGCTGGGTCTACAAATCCAGCCTCATTTGTCTCT
0_known	J6	Tb927.9.6870_for	GGGGACAAGTTTGTACAAAAAAGCAGGCTATGTCTCGAGTGTACGTTGGTAACA	Tb927.9.6870_rev	GGGGACCACTTTGTACAAGAAAGCTGGGTTCACGGGGAACAAGGCCC
0_known	J7	Tb927.3.860_for	GGGGACAAGTTTGTACAAAAAAGCAGGCTATGCAGCGGTCTCAACTTGC	Tb927.3.860_rev	GGGGACCACTTTGTACAAGAAAGCTGGGTTTACTTGGCCATTGGGTTCTGAGA
0_known	J8	Tb927.5.1000_for	GGGGACAAGTTTGTACAAAAAAGCAGGCTATGGCTCTTCGTCGCATTCAAA	Tb927.5.1000_rev	GGGGACCACTTTGTACAAGAAAGCTGGGTTCACATAGCATACTGCCGCGT
0_known	J9	Tb927.11.13040_for	GGGGACAAGTTTGTACAAAAAAGCAGGCTATGGCCGATCAACTCTCCAACG	Tb927.11.13040_rev	GGGGACCACTTTGTACAAGAAAGCTGGGTCTATTTGCTCATCATCATTTTCACGAACT
0_known	J11	Tb927.11.7930_for	GGGGACAAGTTTGTACAAAAAAGCAGGCTATGGCTTCCTCGAAAGTAATATTGGAAG	Tb927.11.7930_rev	GGGGACCACTTTGTACAAGAAAGCTGGGTCTAACTTCCGGGGCGTTTCATC
0_known	J13	Tb927.7.3410_for	GGGGACAAGTTTGTACAAAAAAGCAGGCTATGGCGGCGCTTACTGATG	Tb927.7.3410_rev	GGGGACCACTTTGTACAAGAAAGCTGGGTCTATTTGCCACGCATCTGCATCA
0_known	J15	Tb927.11.13780_for	GGGGACAAGTTTGTACAAAAAAGCAGGCTATGTCGTGGCAGTCCTACGT	Tb927.11.13780_rev	GGGGACCACTTTGTACAAGAAAGCTGGGTTCAGTAGTCCAAGCTAATCAAATACTCCG
0_known	J16	Tb927.9.8000_for	GGGGACAAGTTTGTACAAAAAAGCAGGCTATGACGCTGACACCTCGTATTATTAAAG	Tb927.9.8000_rev	GGGGACCACTTTGTACAAGAAAGCTGGGTTCACGCCTTGTTGGCGTATTT
0_known	J17	Tb927.11.10420_for	GGGGACAAGTTTGTACAAAAAAGCAGGCTATGAGTGCACCAATGGCTCG	Tb927.11.10420_rev	GGGGACCACTTTGTACAAGAAAGCTGGGTTTATGAGTTGAGCTGCCACTCCG
0_known	J18	Tb927.11.3090_for	GGGGACAAGTTTGTACAAAAAAGCAGGCTATGGAGGAAAGGGAGGAGGGA	Tb927.11.3090_rev	GGGGACCACTTTGTACAAGAAAGCTGGGTTTACGAATTTTCGCTGCAGATACGA
0_known	J19	Tb927.10.7190_for	GGGGACAAGTTTGTACAAAAAAGCAGGCTATGAGAGTTGTCGTTCAGCGTG	Tb927.10.7190_rev	GGGGACCACTTTGTACAAGAAAGCTGGGTTCACTTATTTCGGCTGTCCAGC
0_known	J20	Tb927.11.13290_for	GGGGACAAGTTTGTACAAAAAAGCAGGCTATGACAACACTTCTCGACCCCC	Tb927.11.13290_rev	GGGGACCACTTTGTACAAGAAAGCTGGGTTTAGCGTTGAGCCAACCCCA
0_known	J21	Tb927.11.16430_for	GGGGACAAGTTTGTACAAAAAAGCAGGCTATGAGTTCTTCATGGCTGTCCTTCG	Tb927.11.16430_rev	GGGGACCACTTTGTACAAGAAAGCTGGGTTCATAGCTGCTCATCTGTCGCA
0_known	J22	Tb927.5.1360_for	GGGGACAAGTTTGTACAAAAAAGCAGGCTATGAGAAAGATATATATTGCGGGTCCTGC	Tb927.5.1360_rev	GGGGACCACTTTGTACAAGAAAGCTGGGTTTACTTACTGGGGAAATTTGCCAGG
0_known	J23	Tb927.6.4530_for	GGGGACAAGTTTGTACAAAAAAGCAGGCTATGTCCTCTTCTGCTCTGTTGTTCG	Tb927.6.4530_rev	GGGGACCACTTTGTACAAGAAAGCTGGGTCTAATACGAATGCAACCGCCCC
0_known	J24	Tb927.11.16130_for	GGGGACAAGTTTGTACAAAAAAGCAGGCTATGCCCAGCGAGCGGACC	Tb927.11.16130_rev	GGGGACCACTTTGTACAAGAAAGCTGGGTTTATGCCCTTTCATAAATTTGCTTGACG
0_known	K1	Tb927.5.1880_for	GGGGACAAGTTTGTACAAAAAAGCAGGCTATGACAGACCGACCTCCGAC	Tb927.5.1880_rev	GGGGACCACTTTGTACAAGAAAGCTGGGTCTAACCCGCCCTCTCCTCG
0_known	K2	Tb927.11.7940_for	GGGGACAAGTTTGTACAAAAAAGCAGGCTATGTCCAGCGCATTTGACGAT	Tb927.11.7940_rev	GGGGACCACTTTGTACAAGAAAGCTGGGTTCAGCGAAAAATCTCTGGGGTCA
0_known	K3	Tb927.8.6090_for	GGGGACAAGTTTGTACAAAAAAGCAGGCTATGTCTACAAACGGTAATTCCGTAACA	Tb927.8.6090_rev	GGGGACCACTTTGTACAAGAAAGCTGGGTTCAACACGAGGCGTTTGCC
0_known	K4	Tb927.11.8970_for	GGGGACAAGTTTGTACAAAAAAGCAGGCTATGACGCGCAAGGTGGCT	Tb927.11.8970_rev	GGGGACCACTTTGTACAAGAAAGCTGGGTCTAACAACCATTCGTGTTAGATTCGGC
0_known	K5	Tb927.8.2560_for	GGGGACAAGTTTGTACAAAAAAGCAGGCTATGACGGAGTTGACACGCCT	Tb927.8.2560_rev	GGGGACCACTTTGTACAAGAAAGCTGGGTCTAACGGCGTACAGCAGCTG
0_known	K6	Tb927.10.3420_for	GGGGACAAGTTTGTACAAAAAAGCAGGCTATGAACTTAAATTGTGAAGGACGTGACG	Tb927.10.3420_rev	GGGGACCACTTTGTACAAGAAAGCTGGGTTTATAAATATATATAAATATTTTTTTTAA
0_known	K7	Tb927.11.15920_for	GGGGACAAGTTTGTACAAAAAAGCAGGCTATGAATGGCGGCGCCATT	Tb927.11.15920_rev	GGGGACCACTTTGTACAAGAAAGCTGGGTTCACTCATCGCACAACTGTGAAA
0_known	K8	Tb927.11.3680_for	GGGGACAAGTTTGTACAAAAAAGCAGGCTATGGCAAATGCTGTTAGCGAAAAG	Tb927.11.3680_rev	GGGGACCACTTTGTACAAGAAAGCTGGGTCTACATAACACCGCCGCCC
0_known	K9	Tb927.11.3380_for	GGGGACAAGTTTGTACAAAAAAGCAGGCTATGGCAGACGGTAAGGCAGA	Tb927.11.3380_rev	GGGGACCACTTTGTACAAGAAAGCTGGGTCTATGTGGTGGTTTTGGAAAGGGC
0_known	K10	Tb927.9.6130_for	GGGGACAAGTTTGTACAAAAAAGCAGGCTATGTCATTTCCAAAGGAATGGACCG	Tb927.9.6130_rev	GGGGACCACTTTGTACAAGAAAGCTGGGTTTAAGAGATGCCCGCCAGTTGC
0_known	K11	Tb927.11.15000_for	GGGGACAAGTTTGTACAAAAAAGCAGGCTATGGTCCGGCGGAATAATAAGTCG	Tb927.11.15000_rev	GGGGACCACTTTGTACAAGAAAGCTGGGTTTACTCTCCACGAGCACGCT
0_known	K12	Tb927.4.4620_for	GGGGACAAGTTTGTACAAAAAAGCAGGCTATGATCCGTCGCACGGCA	Tb927.4.4620_rev	GGGGACCACTTTGTACAAGAAAGCTGGGTTCACAGATCTTCAGTCGTCATTGTTAG
0_known	K13	Tb927.10.280_for	GGGGACAAGTTTGTACAAAAAAGCAGGCTATGCCCTTTGTCGATCACAATAAATACA	Tb927.10.280_rev	GGGGACCACTTTGTACAAGAAAGCTGGGTCTACTCATCATATACGGGTTTCTTCCCC
0_known	K14	Tb927.11.12360_for	GGGGACAAGTTTGTACAAAAAAGCAGGCTATGCCGTACCCAGACGTCC	Tb927.11.12360_rev	GGGGACCACTTTGTACAAGAAAGCTGGGTTCAACCCTGTGGCAGTGTTGG
0_known	K15	Tb927.10.14210_for	GGGGACAAGTTTGTACAAAAAAGCAGGCTATGAGGGCGCAGTCAGACC	Tb927.10.14210_rev	GGGGACCACTTTGTACAAGAAAGCTGGGTTTAGACTGACCGGAAAGAATCGGC
0_known	K16	Tb927.3.4000_for	GGGGACAAGTTTGTACAAAAAAGCAGGCTATGATAAGATATCTTTTGCTTATAAGTCGC	Tb927.3.4000_rev	GGGGACCACTTTGTACAAGAAAGCTGGGTTTAGCGTGCCTTTGAGGAATTTGT
0_known	K17	Tb927.7.1170_for	GGGGACAAGTTTGTACAAAAAAGCAGGCTATGATTACAGAGGGCGCCGC	Tb927.7.1170_rev	GGGGACCACTTTGTACAAGAAAGCTGGGTCTACTCACGCTCCCGTTTTCC
0_known	K18	Tb927.10.14810_for	GGGGACAAGTTTGTACAAAAAAGCAGGCTATGGCTCGCGGTCAAATCATG	Tb927.10.14810_rev	GGGGACCACTTTGTACAAGAAAGCTGGGTTCACTTCTTCGGCTGGGGTT
0_known	K19	Tb927.8.920_for	GGGGACAAGTTTGTACAAAAAAGCAGGCTATGCCGTGCCCCATCAGC	Tb927.8.920_rev	GGGGACCACTTTGTACAAGAAAGCTGGGTCTACTCCCCATCATTGAAAGGTGGT
0_known	K20	Tb927.1.1630_for	GGGGACAAGTTTGTACAAAAAAGCAGGCTATGGGTGGAACAAAGAAAAAGGCA	Tb927.1.1630_rev	GGGGACCACTTTGTACAAGAAAGCTGGGTTCAAAACAGCGGCTCATCGTAC
0_known	K21	Tb927.8.1440_for	GGGGACAAGTTTGTACAAAAAAGCAGGCTATGTTAAAGGTAGGCCAGAGACACA	Tb927.8.1440_rev	GGGGACCACTTTGTACAAGAAAGCTGGGTCTACTCGACACGGGGCACA
0_known	K22	Tb927.3.5270_for	GGGGACAAGTTTGTACAAAAAAGCAGGCTATGAGTAGTGAGAGATATGTGGATGGTG	Tb927.3.5270_rev	GGGGACCACTTTGTACAAGAAAGCTGGGTTCATAAAGAGTCGAGCATTTCTAAAACCT
0_known	K23	Tb927.4.2710_for	GGGGACAAGTTTGTACAAAAAAGCAGGCTATGACGGCAATTGCGATGAAACG	Tb927.4.2710_rev	GGGGACCACTTTGTACAAGAAAGCTGGGTCTAAAACGGACATTGAGCTCGCT
0_known	K24	Tb927.10.8300_for	GGGGACAAGTTTGTACAAAAAAGCAGGCTATGGAGGCGCTTCGCGTT	Tb927.10.8300_rev	GGGGACCACTTTGTACAAGAAAGCTGGGTTTAAAGGCGTCTAAGAAAAAGCACAGT
0_known	L1	Tb927.11.9790_for	GGGGACAAGTTTGTACAAAAAAGCAGGCTATGGCGGACCTCTTAACGGT	Tb927.11.9790_rev	GGGGACCACTTTGTACAAGAAAGCTGGGTTTAATCGGTTGAAAACGCTTCCTTCT
0_known	L2	Tb927.10.5120_for	GGGGACAAGTTTGTACAAAAAAGCAGGCTATGCTAGCTCTTACATCACGCCG	Tb927.10.5120_rev	GGGGACCACTTTGTACAAGAAAGCTGGGTTTACGATGGCACACCACTTTGG
0_known	L3	Tb927.11.6010_for	GGGGACAAGTTTGTACAAAAAAGCAGGCTATGCGCGGTGCCGATCAG	Tb927.11.6010_rev	GGGGACCACTTTGTACAAGAAAGCTGGGTTTATACGAGCTCCCTTTTTTCAAGCA
0_known	L4	Tb927.8.6680_for	GGGGACAAGTTTGTACAAAAAAGCAGGCTATGATTAAGGCTGTTATAATAATTAATATG	Tb927.8.6680_rev	GGGGACCACTTTGTACAAGAAAGCTGGGTTTAGTAATGATAGCCACTAGATGATGATCC
0_known	L5	Tb927.5.1730_for	GGGGACAAGTTTGTACAAAAAAGCAGGCTATGTTTGGTTCCCGGAAGGC	Tb927.5.1730_rev	GGGGACCACTTTGTACAAGAAAGCTGGGTTTATTCTTCGGCTTTCTTGGCCAC
0_known	L6	Tb927.8.2100_for	GGGGACAAGTTTGTACAAAAAAGCAGGCTATGCTAAGTGACGATACCTGTCAAC	Tb927.8.2100_rev	GGGGACCACTTTGTACAAGAAAGCTGGGTTCAAGAGCAACCACCCGTATAGG
0_known	L7	Tb927.11.16870_for	GGGGACAAGTTTGTACAAAAAAGCAGGCTATGTCATGGCGAGCGCGT	Tb927.11.16870_rev	GGGGACCACTTTGTACAAGAAAGCTGGGTTCACGGCATTATTTTAAAACCGACATTT
0_known	L8	Tb927.9.5650_for	GGGGACAAGTTTGTACAAAAAAGCAGGCTATGGATCCGGCGGTTAGGG	Tb927.9.5650_rev	GGGGACCACTTTGTACAAGAAAGCTGGGTCTAACTTTCCACTTTTTTGGGTTTAAGAAC
0_known	L9	Tb927.3.1410_for	GGGGACAAGTTTGTACAAAAAAGCAGGCTATGCCGCGTCCCTTTGGT	Tb927.3.1410_rev	GGGGACCACTTTGTACAAGAAAGCTGGGTCTAGGCAGGAATATAGAACTTACGTCTGC
0_known	L10	Tb927.10.8710_for	GGGGACAAGTTTGTACAAAAAAGCAGGCTATGGACGGAGATGATGGTTCGC	Tb927.10.8710_rev	GGGGACCACTTTGTACAAGAAAGCTGGGTTTACTTCTTAGGGGTGAGAAGCATACG
0_known	L11	Tb927.11.740_for	GGGGACAAGTTTGTACAAAAAAGCAGGCTATGTCTGACGATGAGGGACAGTT	Tb927.11.740_rev	GGGGACCACTTTGTACAAGAAAGCTGGGTTTATCGCTCAGCTGCATTCTTGAA
0_known	L12	Tb927.10.12530_for	GGGGACAAGTTTGTACAAAAAAGCAGGCTATGGACATAGATTCCCCCTATAAAATTTTG	Tb927.10.12530_rev	GGGGACCACTTTGTACAAGAAAGCTGGGTTCACTGCTGGGTTTGTCGC
0_known	L13	Tb927.7.1120_for	GGGGACAAGTTTGTACAAAAAAGCAGGCTATGTCTACAATCTTTGACTTTGAGGTGC	Tb927.7.1120_rev	GGGGACCACTTTGTACAAGAAAGCTGGGTTCACGCGCTCTGCGTGCT
0_known	L14	Tb927.4.4980_for	GGGGACAAGTTTGTACAAAAAAGCAGGCTATGCTTTCCGCAACTTTCGGC	Tb927.4.4980_rev	GGGGACCACTTTGTACAAGAAAGCTGGGTTCACCGCATTTCATTCGTTGTTTC
0_known	L15	Tb927.5.1570_for	GGGGACAAGTTTGTACAAAAAAGCAGGCTATGGCAAAGAAGAACAAGCACGT	Tb927.5.1570_rev	GGGGACCACTTTGTACAAGAAAGCTGGGTCTAGAAAACATAAAGGCCGTAGGGG
0_known	L16	Tb927.6.4950_for	GGGGACAAGTTTGTACAAAAAAGCAGGCTATGCAAGCGGAAGAGACCACA	Tb927.6.4950_rev	GGGGACCACTTTGTACAAGAAAGCTGGGTCTACCACGCCTTGATTTTAAAATGCA
0_known	L17	Tb927.4.3460_for	GGGGACAAGTTTGTACAAAAAAGCAGGCTATGCGTCGCTTGCAGAAGG	Tb927.4.3460_rev	GGGGACCACTTTGTACAAGAAAGCTGGGTTCACGAGGTATCTTTGTTTTTTTTATTCAA
0_known	L18	Tb927.7.1130_for	GGGGACAAGTTTGTACAAAAAAGCAGGCTATGTCAGCTGCTTCGTCAATCTTT	Tb927.7.1130_rev	GGGGACCACTTTGTACAAGAAAGCTGGGTTCACGCGCTCTGCGTGCT
0_known	L19	Tb927.8.680_for	GGGGACAAGTTTGTACAAAAAAGCAGGCTATGTTCTCTTCGACATTTAGGCGTT	Tb927.8.680_rev	GGGGACCACTTTGTACAAGAAAGCTGGGTTCACTCCAACGTAGCGACTGA
0_known	L20	Tb927.11.13690.1_for	GGGGACAAGTTTGTACAAAAAAGCAGGCTATGGAGTCGATTTTTTGCGTGGA	Tb927.11.13690.1_rev	GGGGACCACTTTGTACAAGAAAGCTGGGTCTATTGCTCATATCCTGAGCTGAAAAACA
0_known	L21	Tb927.4.3650_for	GGGGACAAGTTTGTACAAAAAAGCAGGCTATGTCCGTCTTGGTCAAGGGG	Tb927.4.3650_rev	GGGGACCACTTTGTACAAGAAAGCTGGGTTTATACAACATCAGTTTCTTGTGCCCT
0_known	L22	Tb927.5.4350_for	GGGGACAAGTTTGTACAAAAAAGCAGGCTATGTACCGAAAAAATGTATGTGTGGTAATA	Tb927.5.4350_rev	GGGGACCACTTTGTACAAGAAAGCTGGGTTTATATGGAAGACCTTGTTTTCAAAAATGC
0_known	L23	Tb927.8.6760_for	GGGGACAAGTTTGTACAAAAAAGCAGGCTATGAAGATCTTCAGGGATATCTTAACAAAT	Tb927.8.6760_rev	GGGGACCACTTTGTACAAGAAAGCTGGGTTCACACGCGTTCGCCCTT
0_known	L24	Tb927.7.2590_for	GGGGACAAGTTTGTACAAAAAAGCAGGCTATGAGTGGTCAGCAAGGTGAGG	Tb927.7.2590_rev	GGGGACCACTTTGTACAAGAAAGCTGGGTCTAAGCCACCGTCTGCGC
0_known	M2	Tb927.7.1180_for	GGGGACAAGTTTGTACAAAAAAGCAGGCTATGCAACGATACAAGGAGGGCA	Tb927.7.1180_rev	GGGGACCACTTTGTACAAGAAAGCTGGGTTCACCTCGCTTCCAACACAAC
0_known	M3	Tb927.1.630_for	GGGGACAAGTTTGTACAAAAAAGCAGGCTATGAGCATACAACCAATTGTACAACTTTTG	Tb927.1.630_rev	GGGGACCACTTTGTACAAGAAAGCTGGGTTCATCTGGGTTCAAGTGCTTCTCT
0_known	M4	Tb927.8.5880_for	GGGGACAAGTTTGTACAAAAAAGCAGGCTATGCCCAAGAATATGGGAAAGGGC	Tb927.8.5880_rev	GGGGACCACTTTGTACAAGAAAGCTGGGTTCAGAGGTCGTCAAAGTTTGCC
0_known	M5	Tb927.1.4490_for	GGGGACAAGTTTGTACAAAAAAGCAGGCTATGACTTCCGCGTACCGCA	Tb927.1.4490_rev	GGGGACCACTTTGTACAAGAAAGCTGGGTTCAATATTTGCTTTTCATGCGACCC
0_known	M6	Tb927.5.1030_for	GGGGACAAGTTTGTACAAAAAAGCAGGCTATGCTGCGCACTTCCCTG	Tb927.5.1030_rev	GGGGACCACTTTGTACAAGAAAGCTGGGTTCACACCTTTTGCTGCTGCTT
0_known	M7	Tb927.9.12410_for	GGGGACAAGTTTGTACAAAAAAGCAGGCTATGCGACAAACTGGAAGTTTTCAGC	Tb927.9.12410_rev	GGGGACCACTTTGTACAAGAAAGCTGGGTTCACTCATCGGGTAGATCCTCGA
0_known	M8	Tb927.11.8910_for	GGGGACAAGTTTGTACAAAAAAGCAGGCTATGATTCGCAAGACTCCACCAC	Tb927.11.8910_rev	GGGGACCACTTTGTACAAGAAAGCTGGGTTCAAAAGTATCCTATGGTCCAATTGTAGC
0_known	M9	Tb927.11.9140_for	GGGGACAAGTTTGTACAAAAAAGCAGGCTATGAGGAGTAGCCGGGGTATTTT	Tb927.11.9140_rev	GGGGACCACTTTGTACAAGAAAGCTGGGTTTACTGTGTACCGTGTTCACCCG
0_known	M10	Tb927.11.510_for	GGGGACAAGTTTGTACAAAAAAGCAGGCTATGTCCCAGCAACCGCAGT	Tb927.11.510_rev	GGGGACCACTTTGTACAAGAAAGCTGGGTTTACTGACGGGATATCGGAGGC
0_known	M11	Tb927.6.2130_for	GGGGACAAGTTTGTACAAAAAAGCAGGCTATGTCTTTAGTTGAAAGTCAGAAGGACATG	Tb927.6.2130_rev	GGGGACCACTTTGTACAAGAAAGCTGGGTTTAGTTAGAGGTACCACCACGCTG
0_known	M12	Tb927.7.1140_for	GGGGACAAGTTTGTACAAAAAAGCAGGCTATGCTGCGTTCATCTCGGAAAA	Tb927.7.1140_rev	GGGGACCACTTTGTACAAGAAAGCTGGGTTCAAAGTCGCGCACTCCCG
0_known	M13	Tb927.8.5780_for	GGGGACAAGTTTGTACAAAAAAGCAGGCTATGGACGTCAACTGTACATTGATTGA	Tb927.8.5780_rev	GGGGACCACTTTGTACAAGAAAGCTGGGTTTACATGATGGCGCAACCTCC
0_known	M14	Tb927.3.3750_for	GGGGACAAGTTTGTACAAAAAAGCAGGCTATGTCGACTCAACTTATTTCCGGTG	Tb927.3.3750_rev	GGGGACCACTTTGTACAAGAAAGCTGGGTTTACCGACGCAGCCACGA
0_known	M15	Tb927.10.14260_for	GGGGACAAGTTTGTACAAAAAAGCAGGCTATGACGACAATTGAGTTCATATTTCTCGT	Tb927.10.14260_rev	GGGGACCACTTTGTACAAGAAAGCTGGGTCTACAGTCCTTTTGATTCTTTGTCCATCA
0_known	M16	Tb927.11.880_for	GGGGACAAGTTTGTACAAAAAAGCAGGCTATGTCATACAGGCCCCACCAC	Tb927.11.880_rev	GGGGACCACTTTGTACAAGAAAGCTGGGTTTATAGTTGGCCGCACGAGTCA
0_known	M17	Tb927.8.550_for	GGGGACAAGTTTGTACAAAAAAGCAGGCTATGAACCCAAATGCTGTTGCTACT	Tb927.8.550_rev	GGGGACCACTTTGTACAAGAAAGCTGGGTTCACCAGTAGAGACGGTGTGC
0_known	M18	Tb927.4.4680_for	GGGGACAAGTTTGTACAAAAAAGCAGGCTATGATCAAGAATTATTTTGATCTCTCTAGC	Tb927.4.4680_rev	GGGGACCACTTTGTACAAGAAAGCTGGGTCTAGAAGGCGCTACGGTACCA
0_known	M19	Tb927.7.890_for	GGGGACAAGTTTGTACAAAAAAGCAGGCTATGGCTGCTTTACGCTACTTGC	Tb927.7.890_rev	GGGGACCACTTTGTACAAGAAAGCTGGGTTCAGTGATGAGGGGTTATGGTGTG
0_known	M20	Tb927.3.3450_for	GGGGACAAGTTTGTACAAAAAAGCAGGCTATGGGCTTGCTTACGCTTCTG	Tb927.3.3450_rev	GGGGACCACTTTGTACAAGAAAGCTGGGTCTATTTCTTTATGCTTTTAATGGCCCACAT
0_known	M21	Tb927.10.370_for	GGGGACAAGTTTGTACAAAAAAGCAGGCTATGGAAGTTGCTGCGGTTGC	Tb927.10.370_rev	GGGGACCACTTTGTACAAGAAAGCTGGGTTTAAAACTGCAGAACATGGGCATTATCC
0_known	M22	Tb927.9.13100_for	GGGGACAAGTTTGTACAAAAAAGCAGGCTATGACAACAGGTGGAAAGCAAGAA	Tb927.9.13100_rev	GGGGACCACTTTGTACAAGAAAGCTGGGTTTAACACACCTCTGCATGCTGTC
0_known	M23	Tb927.7.6360_for	GGGGACAAGTTTGTACAAAAAAGCAGGCTATGTCTCTTACAGGTGATGATGCGG	Tb927.7.6360_rev	GGGGACCACTTTGTACAAGAAAGCTGGGTCTACGAGCCCCTCTTCGATTTCT
0_known	M24	Tb927.9.11720_for	GGGGACAAGTTTGTACAAAAAAGCAGGCTATGCGGCGACTGATATCATCAC	Tb927.9.11720_rev	GGGGACCACTTTGTACAAGAAAGCTGGGTTCAGCTTGACACCTCACCAACA
0_known	N1	Tb927.10.4840_for	GGGGACAAGTTTGTACAAAAAAGCAGGCTATGTACCGTGAGAGCCTGCT	Tb927.10.4840_rev	GGGGACCACTTTGTACAAGAAAGCTGGGTTTAGGGCCGCGAGCGCTT
0_known	N2	Tb927.3.3470_for	GGGGACAAGTTTGTACAAAAAAGCAGGCTATGTTCGACTACCTTCTTTCCCTCT	Tb927.3.3470_rev	GGGGACCACTTTGTACAAGAAAGCTGGGTTTAGGATGACTTAGCCCCACTGC
0_known	N3	Tb927.7.3900_for	GGGGACAAGTTTGTACAAAAAAGCAGGCTATGGCAAGCGTAATTGAAAAGGGG	Tb927.7.3900_rev	GGGGACCACTTTGTACAAGAAAGCTGGGTTCAAGAAAATCGTGGTCCATCATTGA
0_known	N4	Tb927.2.2900_for	GGGGACAAGTTTGTACAAAAAAGCAGGCTATGAGTGAAGAAGAACTCTTGTATTACACT	Tb927.2.2900_rev	GGGGACCACTTTGTACAAGAAAGCTGGGTTCAGTTAGTCGATAAGGCTTGAAAGAAC
0_known	N5	Tb927.3.1000_for	GGGGACAAGTTTGTACAAAAAAGCAGGCTATGCGGCGCACATGTTGC	Tb927.3.1000_rev	GGGGACCACTTTGTACAAGAAAGCTGGGTCTATTCGGTTTCTCCAGCCCCT
0_known	N6	Tb927.9.3000_for	GGGGACAAGTTTGTACAAAAAAGCAGGCTATGCAATGCTCATCATTGAAGACCG	Tb927.9.3000_rev	GGGGACCACTTTGTACAAGAAAGCTGGGTTCAGAAAGTTCCTGGACACCTTGA
0_known	N7	Tb927.9.13680_for	GGGGACAAGTTTGTACAAAAAAGCAGGCTATGGGTCAATGGTTGGCTTCCG	Tb927.9.13680_rev	GGGGACCACTTTGTACAAGAAAGCTGGGTCTACTTCATACTCTTCTTAATGTTTGCTGA
0_known	N8	Tb927.9.13710_for	GGGGACAAGTTTGTACAAAAAAGCAGGCTATGGGTCAGTGGTTGGCTTCC	Tb927.9.13710_rev	GGGGACCACTTTGTACAAGAAAGCTGGGTCTACTTCATACTCTTCTTAATGTTTGCTGA
0_known	N9	Tb927.7.2510_for	GGGGACAAGTTTGTACAAAAAAGCAGGCTATGTCGTCGAAGGACATTATTGGAC	Tb927.7.2510_rev	GGGGACCACTTTGTACAAGAAAGCTGGGTTCAACTCTTGGGCACAAACTTTGG
0_known	N11	Tb927.6.4990_for	GGGGACAAGTTTGTACAAAAAAGCAGGCTATGTTTCGCACGTTCGGTCG	Tb927.6.4990_rev	GGGGACCACTTTGTACAAGAAAGCTGGGTCTAGTGGTGTTTTAGGGCAGCAA
0_known	N12	Tb927.9.13650_for	GGGGACAAGTTTGTACAAAAAAGCAGGCTATGGGTCAATGGTTGGCTTCCG	Tb927.9.13650_rev	GGGGACCACTTTGTACAAGAAAGCTGGGTCTACTTCATACTCTTCTTAATGTTCACTGA

0_known	N13	Tb927.11.6130_for	GGGGACAAGTTTGTACAAAAAAGCAGGCTATGGCGGAAGAGGTGTCGG	Tb927.11.6130_rev	GGGGACCACTTTGTACAAGAAAGCTGGGTTTACGCCTCTTCGCACCAGC
0_known	N15	Tb927.11.580_for	GGGGACAAGTTTGTACAAAAAAGCAGGCTATGATAGTACCGTCAAATTTTGGGTACG	Tb927.11.580_rev	GGGGACCACTTTGTACAAGAAAGCTGGGTTTAGTCGTTTTCATAGAAAAGGATAGTTGG
0_known	N16	Tb927.4.2260_for	GGGGACAAGTTTGTACAAAAAAGCAGGCTATGTCCTCATCTCGTCCTGCC	Tb927.4.2260_rev	GGGGACCACTTTGTACAAGAAAGCTGGGTTCAGTAGTCATCATCTTCCAGCATGA
0_known	N17	Tb927.6.3650_for	GGGGACAAGTTTGTACAAAAAAGCAGGCTATGCTTAAGGGGATCCGTAGCC	Tb927.6.3650_rev	GGGGACCACTTTGTACAAGAAAGCTGGGTTTACGGGGCACAAGTGCTAAGT
0_known	N18	Tb927.9.11740_for	GGGGACAAGTTTGTACAAAAAAGCAGGCTATGTCGGTTGTGATCTCAACATCAC	Tb927.9.11740_rev	GGGGACCACTTTGTACAAGAAAGCTGGGTTCATACATTTAGCTTGATGTGGCCC
0_known	N19	Tb927.10.4540_for	GGGGACAAGTTTGTACAAAAAAGCAGGCTATGGAGAGTCAGTGGAACGGTT	Tb927.10.4540_rev	GGGGACCACTTTGTACAAGAAAGCTGGGTTTACCCACCAAGCAAAAACGGC
0_known	N20	Tb927.9.3480_for	GGGGACAAGTTTGTACAAAAAAGCAGGCTATGTATAATGGCATCGCTCCGATC	Tb927.9.3480_rev	GGGGACCACTTTGTACAAGAAAGCTGGGTTTACTCTCCCTTGATACGCTTCACC
0_known	N21	Tb927.10.4270_for	GGGGACAAGTTTGTACAAAAAAGCAGGCTATGGAGTACATGCATCATATTCAGGGC	Tb927.10.4270_rev	GGGGACCACTTTGTACAAGAAAGCTGGGTTTAAAGATTCTCCTTAAGGTATTCGTTGAC
0_known	N22	Tb927.3.5550_for	GGGGACAAGTTTGTACAAAAAAGCAGGCTATGGTAAACTTGCGTCTTCAGGTG	Tb927.3.5550_rev	GGGGACCACTTTGTACAAGAAAGCTGGGTTTACCTCATTTGGGTGAGTTGTTCAA
0_known	N23	Tb927.8.6440_for	GGGGACAAGTTTGTACAAAAAAGCAGGCTATGGAAGACAATCTTGCGGCAG	Tb927.8.6440_rev	GGGGACCACTTTGTACAAGAAAGCTGGGTTCAACATACAATTAAAGTACTACCCTTGAA
0_known	N24	Tb927.7.1390_for	GGGGACAAGTTTGTACAAAAAAGCAGGCTATGACTGTGAACATCGTTGCGC	Tb927.7.1390_rev	GGGGACCACTTTGTACAAGAAAGCTGGGTTTAGCTCCAACTTGGGTACGGG
0_known	O1	Tb927.9.3590_for	GGGGACAAGTTTGTACAAAAAAGCAGGCTATGCGGCGACTGAGCGGT	Tb927.9.3590_rev	GGGGACCACTTTGTACAAGAAAGCTGGGTTTACAGGTCCCGACAAGTGATTCC
0_known	O2	Tb927.11.380_for	GGGGACAAGTTTGTACAAAAAAGCAGGCTATGGCAGAGCTTCATGTTATTGGAG	Tb927.11.380_rev	GGGGACCACTTTGTACAAGAAAGCTGGGTTCAAGCACAACGGACTCCTAATCG
0_known	O3	Tb927.10.6980_for	GGGGACAAGTTTGTACAAAAAAGCAGGCTATGTCGTCTTTAGACTCTGTTAGAGAAGA	Tb927.10.6980_rev	GGGGACCACTTTGTACAAGAAAGCTGGGTCTAAATCGACTTTCGTAGTGTTTCCATCA
0_known	O4	Tb927.5.3580_for	GGGGACAAGTTTGTACAAAAAAGCAGGCTATGGCAGAGGTGCATGTATGTAGG	Tb927.5.3580_rev	GGGGACCACTTTGTACAAGAAAGCTGGGTTCACAGTTCCACAATGTCAGTGTTT
0_known	O5	Tb927.6.3840_for	GGGGACAAGTTTGTACAAAAAAGCAGGCTATGTCCTGTTTCCTTTGCAACCA	Tb927.6.3840_rev	GGGGACCACTTTGTACAAGAAAGCTGGGTCTAATTGTTCTTCTTTGTCAACTGGTCCA
0_known	O6	Tb927.11.13900_for	GGGGACAAGTTTGTACAAAAAAGCAGGCTATGGAAGGGCCCAAGTTAAAGGG	Tb927.11.13900_rev	GGGGACCACTTTGTACAAGAAAGCTGGGTTTAGTACACATGGGCTCGATTCATTAGC
0_known	O7	Tb927.3.610_for	GGGGACAAGTTTGTACAAAAAAGCAGGCTATGACCACTTTTAGGGATTTCAACTTAGTG	Tb927.3.610_rev	GGGGACCACTTTGTACAAGAAAGCTGGGTTTAATTCCACTCCAATTCATCAGGACG
0_known	O8	Tb927.7.1670_for	GGGGACAAGTTTGTACAAAAAAGCAGGCTATGGCCGCTGAGGCTACTG	Tb927.7.1670_rev	GGGGACCACTTTGTACAAGAAAGCTGGGTCTACTTCGCCTCCTCCCCTTC
0_known	O9	Tb927.10.4250_for	GGGGACAAGTTTGTACAAAAAAGCAGGCTATGGGTTTACTATCGATCATCCGCA	Tb927.10.4250_rev	GGGGACCACTTTGTACAAGAAAGCTGGGTCTACCCCGCAAAATACATTCTACTGG
0_known	O10	Tb927.10.3980_for	GGGGACAAGTTTGTACAAAAAAGCAGGCTATGAAAAGGTGGAGGAGAAGGCG	Tb927.10.3980_rev	GGGGACCACTTTGTACAAGAAAGCTGGGTTCATTCCCTTCTTCCCTCATAAAAATAAAA
0_known	O11	Tb927.6.1970_for	GGGGACAAGTTTGTACAAAAAAGCAGGCTATGGCAGAATACCTCATTGATCTGACG	Tb927.6.1970_rev	GGGGACCACTTTGTACAAGAAAGCTGGGTTTACGAACGGCCGCGCTT
0_known	O12	Tb927.8.7000_for	GGGGACAAGTTTGTACAAAAAAGCAGGCTATGAACACGCCCTCTGTTCAATG	Tb927.8.7000_rev	GGGGACCACTTTGTACAAGAAAGCTGGGTCTATCCGCTGTCGCATGGAC
0_known	O13	Tb927.1.840_for	GGGGACAAGTTTGTACAAAAAAGCAGGCTATGCGCCGGATGTGTGTG	Tb927.1.840_rev	GGGGACCACTTTGTACAAGAAAGCTGGGTTTAAGCGTCAATTTCACTTTTGATTTTGGC
0_known	O14	Tb927.9.12200_for	GGGGACAAGTTTGTACAAAAAAGCAGGCTATGACTCGCGCTGGATTTAAGG	Tb927.9.12200_rev	GGGGACCACTTTGTACAAGAAAGCTGGGTTTACTGCTCAATCACCTTAGTTGTCAG
0_known	O16	Tb927.5.1710_for	GGGGACAAGTTTGTACAAAAAAGCAGGCTATGATGCGCCGTGTTTATGCC	Tb927.5.1710_rev	GGGGACCACTTTGTACAAGAAAGCTGGGTTTACTCTACCTTTACATCAAACAATCCTGC
0_known	O17	Tb927.5.2880_for	GGGGACAAGTTTGTACAAAAAAGCAGGCTATGGGGTCAAAGGAGGACCAAG	Tb927.5.2880_rev	GGGGACCACTTTGTACAAGAAAGCTGGGTTCAGAAAAGGTTTAAATCTTTCCACTCTTT
0_known	O18	Tb927.9.8100_for	GGGGACAAGTTTGTACAAAAAAGCAGGCTATGAGCGCTAACGATGTTCAAAACC	Tb927.9.8100_rev	GGGGACCACTTTGTACAAGAAAGCTGGGTTCAGTTGGGATCCATCGTCAGT
0_known	O19	Tb927.9.5960_for	GGGGACAAGTTTGTACAAAAAAGCAGGCTATGCCTGCTGCACCGCTT	Tb927.9.5960_rev	GGGGACCACTTTGTACAAGAAAGCTGGGTTTAATACTTGGAGGCGACTTTTGCA
0_known	O21	Tb927.9.13200_for	GGGGACAAGTTTGTACAAAAAAGCAGGCTATGAGGCTAACGGCTCTTTTGG	Tb927.9.13200_rev	GGGGACCACTTTGTACAAGAAAGCTGGGTTTATGACATCATTAACATCGCGGAAACT
0_known	O22	Tb927.8.2200_for	GGGGACAAGTTTGTACAAAAAAGCAGGCTATGTCACAGCAGGTTGGAGAGG	Tb927.8.2200_rev	GGGGACCACTTTGTACAAGAAAGCTGGGTCTAAGATCCAACCATCACCATCCAGG
0_known	O23	Tb927.9.7650_for	GGGGACAAGTTTGTACAAAAAAGCAGGCTATGGGTAACGTGCTGAGTTGGT	Tb927.9.7650_rev	GGGGACCACTTTGTACAAGAAAGCTGGGTTCAATGCGTATCGGGTAAGTGACT
0_known	O24	Tb927.11.16160_for	GGGGACAAGTTTGTACAAAAAAGCAGGCTATGACAAATCCAGCAGAACAGGC	Tb927.11.16160_rev	GGGGACCACTTTGTACAAGAAAGCTGGGTTTAGTCATTACCCTGATCCGCCG
0_known	P1	Tb927.8.5060_for	GGGGACAAGTTTGTACAAAAAAGCAGGCTATGGGACAATCCAAAACGAAGCT	Tb927.8.5060_rev	GGGGACCACTTTGTACAAGAAAGCTGGGTTCAGCCCCTCTTCGTCCCA
0_known	P2	Tb927.5.970_for	GGGGACAAGTTTGTACAAAAAAGCAGGCTATGGCTGTACAACCGGGAAAGG	Tb927.5.970_rev	GGGGACCACTTTGTACAAGAAAGCTGGGTCTACATGTTCAGCTGGGAGAATGC
0_known	P3	Tb927.10.8580_for	GGGGACAAGTTTGTACAAAAAAGCAGGCTATGGGCCTGTTGGAGTTCCT	Tb927.10.8580_rev	GGGGACCACTTTGTACAAGAAAGCTGGGTTTATACTTGGCCGTCTGGCCT
0_known	P4	Tb927.9.9060_for	GGGGACAAGTTTGTACAAAAAAGCAGGCTATGCCTGTCTGCAACAACGAC	Tb927.9.9060_rev	GGGGACCACTTTGTACAAGAAAGCTGGGTTCACTTCTTGGAAAGAGTCTTTTGAACT
0_known	P5	Tb927.7.6230_for	GGGGACAAGTTTGTACAAAAAAGCAGGCTATGGGGGCTTTGGTTTCGC	Tb927.7.6230_rev	GGGGACCACTTTGTACAAGAAAGCTGGGTTCACGAGTCTGCAGTGACGA
0_known	P6	Tb927.11.14490_for	GGGGACAAGTTTGTACAAAAAAGCAGGCTATGTTCTACAAAATGAAATTGGAGAGGAAT	Tb927.11.14490_rev	GGGGACCACTTTGTACAAGAAAGCTGGGTCTACAGTGGTCCTAAATAATTCCCTACCA
0_known	P7	Tb927.4.2040_for	GGGGACAAGTTTGTACAAAAAAGCAGGCTATGCCTTCATATCCGCCACGT	Tb927.4.2040_rev	GGGGACCACTTTGTACAAGAAAGCTGGGTTTACTCCTCATTGCCACCACCC
0_known	P8	Tb927.11.9450_for	GGGGACAAGTTTGTACAAAAAAGCAGGCTATGCGCATTTCTGAGCGAGC	Tb927.11.9450_rev	GGGGACCACTTTGTACAAGAAAGCTGGGTTCATTCCAATACCCCACAATCCACA
0_known	P9	Tb927.10.200_for	GGGGACAAGTTTGTACAAAAAAGCAGGCTATGAAGACACAAAACTTGCTTTTTGCT	Tb927.10.200_rev	GGGGACCACTTTGTACAAGAAAGCTGGGTTCACTGACACGGTCCGTAGTT
0_known	P10	Tb927.9.7230_for	GGGGACAAGTTTGTACAAAAAAGCAGGCTATGGGACAACTTATTAGTGGCCTTTG	Tb927.9.7230_rev	GGGGACCACTTTGTACAAGAAAGCTGGGTTCACGCACTGCTCGCAGG
0_known	P11	Tb927.1.1400_for	GGGGACAAGTTTGTACAAAAAAGCAGGCTATGTGTGACACAGGCGTTCAG	Tb927.1.1400_rev	GGGGACCACTTTGTACAAGAAAGCTGGGTTCAACTCCAAAAGGCCAGTTCC
0_known	P12	Tb927.8.5030_for	GGGGACAAGTTTGTACAAAAAAGCAGGCTATGTCCCAACAGCGGCAGG	Tb927.8.5030_rev	GGGGACCACTTTGTACAAGAAAGCTGGGTCTATTGTTCAACCTTAAAGGTTTCCTTCTC
0_known	P13	Tb927.10.2120_for	GGGGACAAGTTTGTACAAAAAAGCAGGCTATGAGTGATAGTTTTGCTTTGGATGATGC	Tb927.10.2120_rev	GGGGACCACTTTGTACAAGAAAGCTGGGTTCATTCACGTTTCGTGACCGAA
0_known	P14	Tb927.5.4500_for	GGGGACAAGTTTGTACAAAAAAGCAGGCTATGGGGCTGTTTTCGTGGTTT	Tb927.5.4500_rev	GGGGACCACTTTGTACAAGAAAGCTGGGTCTAAGAATTTTGAAGGTACTGTGATATCCA
0_known	P15	Tb927.8.2090_for	GGGGACAAGTTTGTACAAAAAAGCAGGCTATGAAGGTGGTGGTCAATGAAATGT	Tb927.8.2090_rev	GGGGACCACTTTGTACAAGAAAGCTGGGTTCACTCCAAATCATTCAAGATACTACTCTG
0_known	P16	Tb927.8.6910_for	GGGGACAAGTTTGTACAAAAAAGCAGGCTATGCCAGTCAAGAGGTCCAACA	Tb927.8.6910_rev	GGGGACCACTTTGTACAAGAAAGCTGGGTTTATTGCTTCGCCTTGGGGAC
0_known	P17	Tb927.7.3450_for	GGGGACAAGTTTGTACAAAAAAGCAGGCTATGCTCTGCCCTCCAGATGTT	Tb927.7.3450_rev	GGGGACCACTTTGTACAAGAAAGCTGGGTTCACAACTCTGCCTCTTCATCTGC
0_known	P18	Tb927.10.5020_for	GGGGACAAGTTTGTACAAAAAAGCAGGCTATGGAGGAGGAGAGTCACGC	Tb927.10.5020_rev	GGGGACCACTTTGTACAAGAAAGCTGGGTTTATTTTCCCTTTTTTTTCTTCTCCTTTTC
0_known	P19	Tb927.10.15590_for	GGGGACAAGTTTGTACAAAAAAGCAGGCTATGTATGGTGTTGAGTGTTTTCGGC	Tb927.10.15590_rev	GGGGACCACTTTGTACAAGAAAGCTGGGTTTACAAGTCTCCCTTCCCCGC
0_known	P20	Tb927.8.1080_for	GGGGACAAGTTTGTACAAAAAAGCAGGCTATGAGCACCACCAACCGTGG	Tb927.8.1080_rev	GGGGACCACTTTGTACAAGAAAGCTGGGTCTAATAGAGCGAAGTTTTCTTCATGATGC
0_known	P21	Tb927.2.1680_for	GGGGACAAGTTTGTACAAAAAAGCAGGCTATGTTGTCGCGCACCTTTTTTG	Tb927.2.1680_rev	GGGGACCACTTTGTACAAGAAAGCTGGGTTTATCCCTGTTCAATTTGGCCACAA
0_known	P22	Tb927.9.3170_for	GGGGACAAGTTTGTACAAAAAAGCAGGCTATGAAGCGCTTTGTCACACCA	Tb927.9.3170_rev	GGGGACCACTTTGTACAAGAAAGCTGGGTCTAGTTACTGATTTTGTTCCCATCCTTGA
0_known	P24	Tb927.2.4580_for	GGGGACAAGTTTGTACAAAAAAGCAGGCTATGGCTGCGACGACCGAG	Tb927.2.4580_rev	GGGGACCACTTTGTACAAGAAAGCTGGGTTTATTTAGTGAATTCCTCCTCTGGTGC
0_hypothetical	A1	Tb927.8.1305_for	GGGGACAAGTTTGTACAAAAAAGCAGGCTATGTTGTGGCAAGCCAAAAGAAC	Tb927.8.1305_rev	GGGGACCACTTTGTACAAGAAAGCTGGGTCTAGACTACAACGGCGGGCA
0_hypothetical	A2	Tb927.3.515_for	GGGGACAAGTTTGTACAAAAAAGCAGGCTATGAAGCACTGGTGGAAGAGTGG	Tb927.3.515_rev	GGGGACCACTTTGTACAAGAAAGCTGGGTTTATAGCATGTCGCCCACTGGC
0_hypothetical	A3	Tb927.10.7675_for	GGGGACAAGTTTGTACAAAAAAGCAGGCTATGCTCCCGCTTTTCGCATG	Tb927.10.7675_rev	GGGGACCACTTTGTACAAGAAAGCTGGGTTTAATACGCCACGGCGACTTC
0_hypothetical	A4	Tb927.9.15905_for	GGGGACAAGTTTGTACAAAAAAGCAGGCTATGATTCATTTCTGCTGTTGTGATAGCA	Tb927.9.15905_rev	GGGGACCACTTTGTACAAGAAAGCTGGGTTTACATTCCTTCCTTCATAACCTTACCG
0_hypothetical	A5	Tb927.10.16121_for	GGGGACAAGTTTGTACAAAAAAGCAGGCTATGCCGGAAGTGAAGATGATGTTG	Tb927.10.16121_rev	GGGGACCACTTTGTACAAGAAAGCTGGGTTTACTGCATCGATGCGTTGTGC
0_hypothetical	A6	Tb927.8.6035_for	GGGGACAAGTTTGTACAAAAAAGCAGGCTATGGGGACCATCAGCCGC	Tb927.8.6035_rev	GGGGACCACTTTGTACAAGAAAGCTGGGTTTACTTGGAGCGCTGGCGC
0_hypothetical	A9	Tb927.11.5855_for	GGGGACAAGTTTGTACAAAAAAGCAGGCTATGAAGGAAATAATAACCTTAAGGGAATTT	Tb927.11.5855_rev	GGGGACCACTTTGTACAAGAAAGCTGGGTTTAAATGGTCAAGACGCTAAGTGTGT
0_hypothetical	A10	Tb927.9.19145_for	GGGGACAAGTTTGTACAAAAAAGCAGGCTATGATTCATTTCTGCTGTTGTGATAGCA	Tb927.9.19145_rev	GGGGACCACTTTGTACAAGAAAGCTGGGTTTACATTCTTTCCTTCATAACCTTACCGAA
0_hypothetical	A11	Tb927.9.4755_for	GGGGACAAGTTTGTACAAAAAAGCAGGCTATGGTGCCACGCCGAGAG	Tb927.9.4755_rev	GGGGACCACTTTGTACAAGAAAGCTGGGTTTACTGCGGCAAATGATGGAAGG
0_hypothetical	A12	Tb927.7.6855_for	GGGGACAAGTTTGTACAAAAAAGCAGGCTATGAAAGGCACGTTTTGCTTTGG	Tb927.7.6855_rev	GGGGACCACTTTGTACAAGAAAGCTGGGTTCAACCCTTCGTATACATTTTCCCCA
0_hypothetical	A13	Tb927.2.725_for	GGGGACAAGTTTGTACAAAAAAGCAGGCTATGGAGTCGTTTCTCCACACCT	Tb927.2.725_rev	GGGGACCACTTTGTACAAGAAAGCTGGGTTCAAGAGTGAGCTCTGGCCTT
0_hypothetical	A14	Tb927.2.1045_for	GGGGACAAGTTTGTACAAAAAAGCAGGCTATGGAGACGTTTCTCCACATCTGC	Tb927.2.1045_rev	GGGGACCACTTTGTACAAGAAAGCTGGGTTCAAGAGTGAGCTCTGGCCTT
0_hypothetical	A15	Tb927.2.4925_for	GGGGACAAGTTTGTACAAAAAAGCAGGCTATGGGCACGACCGAGATAAAATG	Tb927.2.4925_rev	GGGGACCACTTTGTACAAGAAAGCTGGGTTTATCTTTTGTCCACGTTACGTGCA
0_hypothetical	A16	Tb927.7.6425_for	GGGGACAAGTTTGTACAAAAAAGCAGGCTATGTTGCAGTTCCGTAGTTGTTCT	Tb927.7.6425_rev	GGGGACCACTTTGTACAAGAAAGCTGGGTTCAAGAATGACACGCAGAAATAATAAAAGG
0_hypothetical	A17	Tb927.11.6565_for	GGGGACAAGTTTGTACAAAAAAGCAGGCTATGCTTCAGTTGAGCATACCATGTAG	Tb927.11.6565_rev	GGGGACCACTTTGTACAAGAAAGCTGGGTTTACTGCTCATAGAAAACAGCTCCCC
0_hypothetical	A18	Tb927.1.2760_for	GGGGACAAGTTTGTACAAAAAAGCAGGCTATGTGGGTTTCCATCTACCGATGT	Tb927.1.2760_rev	GGGGACCACTTTGTACAAGAAAGCTGGGTTTAATCCTCTTTAACTTTTACCACCTCCG
0_hypothetical	A19	Tb927.8.2685_for	GGGGACAAGTTTGTACAAAAAAGCAGGCTATGGAATTAGACAGGGTTGTTTGGATT	Tb927.8.2685_rev	GGGGACCACTTTGTACAAGAAAGCTGGGTCTAGGCGTTTAAATTCACATCAGGTAAAAA
0_hypothetical	A20	Tb927.11.16915_for	GGGGACAAGTTTGTACAAAAAAGCAGGCTATGCTTCATTGTTTAAGCTGTATCGAATTG	Tb927.11.16915_rev	GGGGACCACTTTGTACAAGAAAGCTGGGTTCACATTACTTCTACCGTTGTTTCCTTC
0_hypothetical	A21	Tb927.11.8775_for	GGGGACAAGTTTGTACAAAAAAGCAGGCTATGCGTGAACTGCCATTTTCCA	Tb927.11.8775_rev	GGGGACCACTTTGTACAAGAAAGCTGGGTTCACTCGTCATAAAGTTTCGTATCGC
0_hypothetical	A22	Tb927.10.9465_for	GGGGACAAGTTTGTACAAAAAAGCAGGCTATGCACAATCTTAACACCATCCAACA	Tb927.10.9465_rev	GGGGACCACTTTGTACAAGAAAGCTGGGTCTACTTATTGCCGGCACGAAGAC
0_hypothetical	A23	Tb927.10.1991_for	GGGGACAAGTTTGTACAAAAAAGCAGGCTATGTCTGTGCTACATGAAGTTGTGAT	Tb927.10.1991_rev	GGGGACCACTTTGTACAAGAAAGCTGGGTCTATTTGTTGTCACCACTCTCACATATGG
0_hypothetical	A24	Tb927.8.8215_for	GGGGACAAGTTTGTACAAAAAAGCAGGCTATGGCAAACACGGCTCTGC	Tb927.8.8215_rev	GGGGACCACTTTGTACAAGAAAGCTGGGTTTACAGACTTTCACCGACACTATGAGC
0_hypothetical	B1	Tb927.5.1145_for	GGGGACAAGTTTGTACAAAAAAGCAGGCTATGGGAGAAAAGGATTCTGTGGCG	Tb927.5.1145_rev	GGGGACCACTTTGTACAAGAAAGCTGGGTTCACATCCACAGTAAGTTATGCACTTG
0_hypothetical	B2	Tb927.7.5945_for	GGGGACAAGTTTGTACAAAAAAGCAGGCTATGAGAGCATGGCATTCATTGTTCA	Tb927.7.5945_rev	GGGGACCACTTTGTACAAGAAAGCTGGGTCTACTGCCTGTCAAATGCCGAG
0_hypothetical	B3	Tb927.1.5180_for	GGGGACAAGTTTGTACAAAAAAGCAGGCTATGCGAGCAGGAGAAACGAC	Tb927.1.5180_rev	GGGGACCACTTTGTACAAGAAAGCTGGGTTTACGTAACCCGCACAATATGTGG
0_hypothetical	B4	Tb927.10.9466_for	GGGGACAAGTTTGTACAAAAAAGCAGGCTATGTGTGTAGTACATTGGAGGTGTACT	Tb927.10.9466_rev	GGGGACCACTTTGTACAAGAAAGCTGGGTTTACCCTACAGCAACGAAAATAAACGA
0_hypothetical	B5	Tb927.11.4105_for	GGGGACAAGTTTGTACAAAAAAGCAGGCTATGGATCCCATCATATGGGAGGATAAC	Tb927.11.4105_rev	GGGGACCACTTTGTACAAGAAAGCTGGGTCTATTTGCTCCGCTGGTATTCATGC
0_hypothetical	B6	Tb927.10.8550_for	GGGGACAAGTTTGTACAAAAAAGCAGGCTATGCACGTTGGCCTCGCG	Tb927.10.8550_rev	GGGGACCACTTTGTACAAGAAAGCTGGGTTCACAACAGAAGACACGTCCGG
0_hypothetical	B7	Tb927.11.101_for	GGGGACAAGTTTGTACAAAAAAGCAGGCTATGAAGGGTAAGTGTACCTCTTCTGG	Tb927.11.101_rev	GGGGACCACTTTGTACAAGAAAGCTGGGTTTATATGGTTTTGACCAGCCTCATCG
0_hypothetical	B8	Tb927.10.9025_for	GGGGACAAGTTTGTACAAAAAAGCAGGCTATGATTTACGACATTACCACTCCGC	Tb927.10.9025_rev	GGGGACCACTTTGTACAAGAAAGCTGGGTCTACAGCCCAGTACTTGCAGC
0_hypothetical	B9	Tb927.9.4895_for	GGGGACAAGTTTGTACAAAAAAGCAGGCTATGAAGCAGGTCTGTGCGC	Tb927.9.4895_rev	GGGGACCACTTTGTACAAGAAAGCTGGGTTCATCCTCTGAAGATTGAACAAATTGTGC
0_hypothetical	B10	Tb927.11.14705_for	GGGGACAAGTTTGTACAAAAAAGCAGGCTATGGCAGGGGCGACTCTAG	Tb927.11.14705_rev	GGGGACCACTTTGTACAAGAAAGCTGGGTTCACTGTATCATTGACTGTGGTCGA
0_hypothetical	B11	Tb927.6.4010_for	GGGGACAAGTTTGTACAAAAAAGCAGGCTATGTCCACACGTCAAGGTGGT	Tb927.6.4010_rev	GGGGACCACTTTGTACAAGAAAGCTGGGTTTATTTCTTTCCGCCCTTCATTTTCGC
0_hypothetical	B12	Tb927.1.3850_for	GGGGACAAGTTTGTACAAAAAAGCAGGCTATGAGATCTGGGAGGAAGGCTG	Tb927.1.3850_rev	GGGGACCACTTTGTACAAGAAAGCTGGGTTTAAGCACTAATGGAGCTGAGCTTG
0_hypothetical	B13	Tb927.2.4785_for	GGGGACAAGTTTGTACAAAAAAGCAGGCTATGGCCTCTTGGACAACCTACC	Tb927.2.4785_rev	GGGGACCACTTTGTACAAGAAAGCTGGGTTTACGACCACTTTTTGCCCAACT
0_hypothetical	B14	Tb927.11.2235_for	GGGGACAAGTTTGTACAAAAAAGCAGGCTATGTTCCCCGGCATGCAGC	Tb927.11.2235_rev	GGGGACCACTTTGTACAAGAAAGCTGGGTTTAAATGCCGTTAAGTAATACAGCGCT
0_hypothetical	B15	Tb927.11.161_for	GGGGACAAGTTTGTACAAAAAAGCAGGCTATGTTTACTCCATCGCTGGCG	Tb927.11.161_rev	GGGGACCACTTTGTACAAGAAAGCTGGGTTTACGATTTCATCATGCGCCGG
0_hypothetical	B16	Tb927.9.3290_for	GGGGACAAGTTTGTACAAAAAAGCAGGCTATGTCGCGCGGCAACCAA	Tb927.9.3290_rev	GGGGACCACTTTGTACAAGAAAGCTGGGTTCAACAACCAGTCTTCCGCG
0_hypothetical	B17	Tb927.2.960_for	GGGGACAAGTTTGTACAAAAAAGCAGGCTATGGTTAGAGAAACTTTGATTTGCCTTGA	Tb927.2.960_rev	GGGGACCACTTTGTACAAGAAAGCTGGGTTCAAAGCGCTGCCCAATTGG
0_hypothetical	B18	Tb927.9.770_for	GGGGACAAGTTTGTACAAAAAAGCAGGCTATGTTCTTTGGTGGGGAAGGAAAG	Tb927.9.770_rev	GGGGACCACTTTGTACAAGAAAGCTGGGTTTACCCTTCTGTAACTTTCCCTTCCC
0_hypothetical	B19	Tb927.7.7475_for	GGGGACAAGTTTGTACAAAAAAGCAGGCTATGTGCAAGCACCTCGGC	Tb927.7.7475_rev	GGGGACCACTTTGTACAAGAAAGCTGGGTTCACCCAATCACACTCGCTAATTC
0_hypothetical	B20	Tb927.9.9115_for	GGGGACAAGTTTGTACAAAAAAGCAGGCTATGCACGCGGAGTGGGAC	Tb927.9.9115_rev	GGGGACCACTTTGTACAAGAAAGCTGGGTCTAGTCTTCCTTTCGGCCAATTGC
0_hypothetical	B21	Tb927.4.170_for	GGGGACAAGTTTGTACAAAAAAGCAGGCTATGGTTAGAGAAACTTTGATTTGCTTTGAC	Tb927.4.170_rev	GGGGACCACTTTGTACAAGAAAGCTGGGTTCAAAGCGCTGTCCAAATGGT
0_hypothetical	B22	Tb927.7.6852_for	GGGGACAAGTTTGTACAAAAAAGCAGGCTATGGAAATCCAAAACTTTCCTTTCTCCC	Tb927.7.6852_rev	GGGGACCACTTTGTACAAGAAAGCTGGGTCTATACTTTGTGAATCATAAATTTGGGTGA
0_hypothetical	B23	Tb927.11.13785_for	GGGGACAAGTTTGTACAAAAAAGCAGGCTATGCACTTATGCCGGGGCG	Tb927.11.13785_rev	GGGGACCACTTTGTACAAGAAAGCTGGGTTCACATCTGGCAACGAGTATTACG
0_hypothetical	B24	Tb927.10.4340_for	GGGGACAAGTTTGTACAAAAAAGCAGGCTATGGGGCACTTCGGGGGA	Tb927.10.4340_rev	GGGGACCACTTTGTACAAGAAAGCTGGGTTCATGTTTTTTGATGGCGATGTATAGATGA
0_hypothetical	C1	Tb927.10.8585_for	GGGGACAAGTTTGTACAAAAAAGCAGGCTATGGAATATAGGATTAAAGTGCGGAACG	Tb927.10.8585_rev	GGGGACCACTTTGTACAAGAAAGCTGGGTTCAATTACCAACCGAATTGCTGTACT
0_hypothetical	C2	Tb927.11.15255_for	GGGGACAAGTTTGTACAAAAAAGCAGGCTATGAGCATGCGTATGACCCCT	Tb927.11.15255_rev	GGGGACCACTTTGTACAAGAAAGCTGGGTTTAAATATTGTTGTTACTTGCTTCCCTAAT
0_hypothetical	C3	Tb927.11.12045_for	GGGGACAAGTTTGTACAAAAAAGCAGGCTATGGATCCTCGACGAGGGGG	Tb927.11.12045_rev	GGGGACCACTTTGTACAAGAAAGCTGGGTTTACTTTGTTCCTGAAGGCGCTG
0_hypothetical	C4	Tb927.10.4350_for	GGGGACAAGTTTGTACAAAAAAGCAGGCTATGGTAGACAAAGCAAAAACTCGAGA	Tb927.10.4350_rev	GGGGACCACTTTGTACAAGAAAGCTGGGTTCAGTCGTGCTTTTTTGAGCATGT
0_hypothetical	C5	Tb927.10.4125_for	GGGGACAAGTTTGTACAAAAAAGCAGGCTATGGTAATGTTTTTGGCCACTGCC	Tb927.10.4125_rev	GGGGACCACTTTGTACAAGAAAGCTGGGTTCACGTCCAACCAGCCGA
0_hypothetical	C6	Tb927.7.1025_for	GGGGACAAGTTTGTACAAAAAAGCAGGCTATGGATTATCAGTTCTTGTGGGATGTG	Tb927.7.1025_rev	GGGGACCACTTTGTACAAGAAAGCTGGGTTTAGTCTCCCTTGGTACGGGTTTT
0_hypothetical	C7	Tb927.7.2985_for	GGGGACAAGTTTGTACAAAAAAGCAGGCTATGATGTACGTGCTTGTAGCCG	Tb927.7.2985_rev	GGGGACCACTTTGTACAAGAAAGCTGGGTTTATTCCTCCTTCCGAGCACGA
0_hypothetical	C9	Tb927.11.2245_for	GGGGACAAGTTTGTACAAAAAAGCAGGCTATGCTTCTAGGAGGTTTTGTTCCCC	Tb927.11.2245_rev	GGGGACCACTTTGTACAAGAAAGCTGGGTTTAGTGATGGTCGTCATGGTGGG
0_hypothetical	C10	Tb927.3.4005_for	GGGGACAAGTTTGTACAAAAAAGCAGGCTATGGAAGGGAAACGGGACTGC	Tb927.3.4005_rev	GGGGACCACTTTGTACAAGAAAGCTGGGTCTAACGACCGCGCTTCCC
0_hypothetical	C11	Tb927.10.1615_for	GGGGACAAGTTTGTACAAAAAAGCAGGCTATGCCGACGGGACACGTG	Tb927.10.1615_rev	GGGGACCACTTTGTACAAGAAAGCTGGGTTCACGCTGTCTCCGCCAA
0_hypothetical	C12	Tb927.3.650_for	GGGGACAAGTTTGTACAAAAAAGCAGGCTATGATGAACCACCAGCGGCC	Tb927.3.650_rev	GGGGACCACTTTGTACAAGAAAGCTGGGTTTACAAGTCGTCATCGTAGTTGGGT
0_hypothetical	C13	Tb927.8.4175_for	GGGGACAAGTTTGTACAAAAAAGCAGGCTATGGGTTTGGAGAATGTGCCTACG	Tb927.8.4175_rev	GGGGACCACTTTGTACAAGAAAGCTGGGTCTAATAGCGGACCCTACACGTATCC
0_hypothetical	C14	Tb927.10.7985_for	GGGGACAAGTTTGTACAAAAAAGCAGGCTATGTTTTCTCTGACCCAAACCTGG	Tb927.10.7985_rev	GGGGACCACTTTGTACAAGAAAGCTGGGTTCACGGCATGTCACGGTACG
0_hypothetical	C15	Tb927.10.9445_for	GGGGACAAGTTTGTACAAAAAAGCAGGCTATGAAAAAAGGCAACCGTCGCG	Tb927.10.9445_rev	GGGGACCACTTTGTACAAGAAAGCTGGGTCTATAATCCGCATGCCTTCTCGG
0_hypothetical	C16	Tb927.10.8350_for	GGGGACAAGTTTGTACAAAAAAGCAGGCTATGACATCCTTGGGGCTGCT	Tb927.10.8350_rev	GGGGACCACTTTGTACAAGAAAGCTGGGTCTACCAGTCGGGGGCGCC
0_hypothetical	C17	Tb927.11.15665_for	GGGGACAAGTTTGTACAAAAAAGCAGGCTATGCTCAGGATCAAAATCGCCG	Tb927.11.15665_rev	GGGGACCACTTTGTACAAGAAAGCTGGGTTTATGTTGAAAACTTTTGCGGGTCG
0_hypothetical	C18	Tb927.3.2476_for	GGGGACAAGTTTGTACAAAAAAGCAGGCTATGCCTGAGACGCCACAGC	Tb927.3.2476_rev	GGGGACCACTTTGTACAAGAAAGCTGGGTTTACGAGAGCGCTGCCTCA
0_hypothetical	C19	Tb927.7.4641_for	GGGGACAAGTTTGTACAAAAAAGCAGGCTATGAAAAGAGCCGCACCGTC	Tb927.7.4641_rev	GGGGACCACTTTGTACAAGAAAGCTGGGTTCATTTGCATCGAAGCTCCTCTTG
0_hypothetical	C20	Tb927.10.8115_for	GGGGACAAGTTTGTACAAAAAAGCAGGCTATGTCAGAAGCCGTCGATAGCT	Tb927.10.8115_rev	GGGGACCACTTTGTACAAGAAAGCTGGGTCTATGATAGAGGGTGCCTGATTGATATGG
0_hypothetical	C21	Tb927.8.4695_for	GGGGACAAGTTTGTACAAAAAAGCAGGCTATGCGGTTTTCTCGGAGGTTATTT	Tb927.8.4695_rev	GGGGACCACTTTGTACAAGAAAGCTGGGTTCACTTCGCCGGCCCGCC
0_hypothetical	C22	Tb927.11.16575_for	GGGGACAAGTTTGTACAAAAAAGCAGGCTATGCCCGCCCTTACTATCGC	Tb927.11.16575_rev	GGGGACCACTTTGTACAAGAAAGCTGGGTTTACTCCTCGTCGGCTTCCG
0_hypothetical	C23	Tb927.2.3035_for	GGGGACAAGTTTGTACAAAAAAGCAGGCTATGTTGGAGTCTGACACGCAGT	Tb927.2.3035_rev	GGGGACCACTTTGTACAAGAAAGCTGGGTTTAAGAAGTGGAAACCCACATAAATGCC
0_hypothetical	C24	Tb927.9.6890_for	GGGGACAAGTTTGTACAAAAAAGCAGGCTATGGGCCCACGCATGGCC	Tb927.9.6890_rev	GGGGACCACTTTGTACAAGAAAGCTGGGTTTATTCATCACCTCCCTGACATTCCA
0_hypothetical	D1	Tb927.11.8185_for	GGGGACAAGTTTGTACAAAAAAGCAGGCTATGTCATCAGTAGGAAGTAACCGTAATTC	Tb927.11.8185_rev	GGGGACCACTTTGTACAAGAAAGCTGGGTCTAGCTAATGCGCTTCACGCC
0_hypothetical	D2	Tb927.7.7350_for	GGGGACAAGTTTGTACAAAAAAGCAGGCTATGATGTCCCTCATGCTTGGACC	Tb927.7.7350_rev	GGGGACCACTTTGTACAAGAAAGCTGGGTTCATCTGACTGCTCCGCAGC
0_hypothetical	D3	Tb927.11.15200_for	GGGGACAAGTTTGTACAAAAAAGCAGGCTATGTCTGACAATGCTTCACCTCCT	Tb927.11.15200_rev	GGGGACCACTTTGTACAAGAAAGCTGGGTTCATCTCATCTCCTCTCGTGGATTG
0_hypothetical	D4	Tb927.11.8130_for	GGGGACAAGTTTGTACAAAAAAGCAGGCTATGGACTTTTTATTCGGCACCATGT	Tb927.11.8130_rev	GGGGACCACTTTGTACAAGAAAGCTGGGTCTATTCTTTTTTGATGTTTCGTGTTGGTAT
0_hypothetical	D5	Tb927.2.1895_for	GGGGACAAGTTTGTACAAAAAAGCAGGCTATGTTCAACCCCAAGAAGATGTTGG	Tb927.2.1895_rev	GGGGACCACTTTGTACAAGAAAGCTGGGTTTACTTCGCTCCACCGGCA

0_hypothetical	D6	Tb927.11.12425_for	GGGGACAAGTTTGTACAAAAAAGCAGGCTATGGCAGGCAGTTCTGAATATTCC	Tb927.11.12425_rev	GGGGACCACTTTGTACAAGAAAGCTGGGTCTAGACAACTTTAAAGGCGTTTGTCCC
0_hypothetical	D7	Tb927.11.485_for	GGGGACAAGTTTGTACAAAAAAGCAGGCTATGGAAAATATTACAAATGTCTCCATTGCC	Tb927.11.485_rev	GGGGACCACTTTGTACAAGAAAGCTGGGTCTACAGATTTGATACGCCGCTGT
0_hypothetical	D8	Tb927.10.8375_for	GGGGACAAGTTTGTACAAAAAAGCAGGCTATGAGGGCTCTTTTGCTCTTTTTGC	Tb927.10.8375_rev	GGGGACCACTTTGTACAAGAAAGCTGGGTTCACCTGTCAGGATGACCCAG
0_hypothetical	D9	Tb927.6.5095_for	GGGGACAAGTTTGTACAAAAAAGCAGGCTATGATTCGACGGACAGTAGTGACG	Tb927.6.5095_rev	GGGGACCACTTTGTACAAGAAAGCTGGGTTTAGTGGTGTGATTGGACAGCG
0_hypothetical	D10	Tb927.11.17015_for	GGGGACAAGTTTGTACAAAAAAGCAGGCTATGCAAAACGCACACAAGAGAGA	Tb927.11.17015_rev	GGGGACCACTTTGTACAAGAAAGCTGGGTCTACTCCGAAGTATTGTTATGGCCC
0_hypothetical	D11	Tb927.11.14595_for	GGGGACAAGTTTGTACAAAAAAGCAGGCTATGGTGCTTGCGTTAACATCACC	Tb927.11.14595_rev	GGGGACCACTTTGTACAAGAAAGCTGGGTTCAGCGTGCCCACCACCG
0_hypothetical	D12	Tb927.8.7255_for	GGGGACAAGTTTGTACAAAAAAGCAGGCTATGACGCATAAGGTCCGCCT	Tb927.8.7255_rev	GGGGACCACTTTGTACAAGAAAGCTGGGTTCAATCAGCGAAAAACATCAATGCTT
0_hypothetical	D13	Tb927.10.1780_for	GGGGACAAGTTTGTACAAAAAAGCAGGCTATGCATCCACTTGCTCGCC	Tb927.10.1780_rev	GGGGACCACTTTGTACAAGAAAGCTGGGTTCAGTAGACCCGCGTTGTTCC
0_hypothetical	D14	Tb927.10.4140_for	GGGGACAAGTTTGTACAAAAAAGCAGGCTATGAGCGTATTCTTCGACATCGAG	Tb927.10.4140_rev	GGGGACCACTTTGTACAAGAAAGCTGGGTTCAATACACAAACAGCATTCGCAC
0_hypothetical	D15	Tb927.5.2560_for	GGGGACAAGTTTGTACAAAAAAGCAGGCTATGTCGAATCCTAACTTCTGGACAAC	Tb927.5.2560_rev	GGGGACCACTTTGTACAAGAAAGCTGGGTTTATACCGCTTCCTCTTCCAGCA
0_hypothetical	D16	Tb927.5.2325_for	GGGGACAAGTTTGTACAAAAAAGCAGGCTATGAGTGGCATTCGAGTGGCG	Tb927.5.2325_rev	GGGGACCACTTTGTACAAGAAAGCTGGGTTCATCCGTTCCTGGCATCCC
0_hypothetical	D17	Tb927.6.250_for	GGGGACAAGTTTGTACAAAAAAGCAGGCTATGAAAAGAATGAAATACACATTTCGATGT	Tb927.6.250_rev	GGGGACCACTTTGTACAAGAAAGCTGGGTTTAGTTACGGCAAGGAATTGCTGAA
0_hypothetical	D18	Tb927.10.6415_for	GGGGACAAGTTTGTACAAAAAAGCAGGCTATGCTGGGTCGTCGGTTCT	Tb927.10.6415_rev	GGGGACCACTTTGTACAAGAAAGCTGGGTTCAGCCATTTAGACAGTGGGGA
0_hypothetical	D19	Tb927.1.2780_for	GGGGACAAGTTTGTACAAAAAAGCAGGCTATGATACCAGTTCATGCGCTGAG	Tb927.1.2780_rev	GGGGACCACTTTGTACAAGAAAGCTGGGTCTAGCGACCCACACTGATTTCC
0_hypothetical	D20	Tb927.3.3010_for	GGGGACAAGTTTGTACAAAAAAGCAGGCTATGCGGCTTCTTGCACGAC	Tb927.3.3010_rev	GGGGACCACTTTGTACAAGAAAGCTGGGTTCAGTGTGAACTCTCCTTGTCACG
0_hypothetical	D21	Tb927.1.1175_for	GGGGACAAGTTTGTACAAAAAAGCAGGCTATGATGAACAGGCCGCCGC	Tb927.1.1175_rev	GGGGACCACTTTGTACAAGAAAGCTGGGTTCAGTTTGTACTATGCTCTGTTGGGA
0_hypothetical	D22	Tb927.3.3240_for	GGGGACAAGTTTGTACAAAAAAGCAGGCTATGAAAAAAAGCGTTTCCTTGAAGGAG	Tb927.3.3240_rev	GGGGACCACTTTGTACAAGAAAGCTGGGTTCACTCCATGTCATACATGACATCCA
0_hypothetical	D23	Tb927.11.7045_for	GGGGACAAGTTTGTACAAAAAAGCAGGCTATGCATGTGGAACCCAGAGAAAC	Tb927.11.7045_rev	GGGGACCACTTTGTACAAGAAAGCTGGGTTCATCGCCGTCTGCCTTTACT
0_hypothetical	D24	Tb927.3.630_for	GGGGACAAGTTTGTACAAAAAAGCAGGCTATGTCGATTTCACCCGCAGAGA	Tb927.3.630_rev	GGGGACCACTTTGTACAAGAAAGCTGGGTCTACGCATCCTTTTTTCCCCGC
0_hypothetical	E1	Tb927.10.5050_for	GGGGACAAGTTTGTACAAAAAAGCAGGCTATGATCCGCCGTAGTTGTGC	Tb927.10.5050_rev	GGGGACCACTTTGTACAAGAAAGCTGGGTTCAGTAATCCTTGGTGTGGATGGG
0_hypothetical	E2	Tb927.9.1365_for	GGGGACAAGTTTGTACAAAAAAGCAGGCTATGGAAGCTGATGCCAATACATTTAACC	Tb927.9.1365_rev	GGGGACCACTTTGTACAAGAAAGCTGGGTTCAAACGCCTGTCCGTCGC
0_hypothetical	E3	Tb927.9.8655_for	GGGGACAAGTTTGTACAAAAAAGCAGGCTATGTGTACGTGTGACGCAGAGG	Tb927.9.8655_rev	GGGGACCACTTTGTACAAGAAAGCTGGGTTTACTCCCCTACGGGGGCT
0_hypothetical	E4	Tb927.11.165_for	GGGGACAAGTTTGTACAAAAAAGCAGGCTATGCCTTTAAGCCCTACAAGGATGA	Tb927.11.165_rev	GGGGACCACTTTGTACAAGAAAGCTGGGTTCACTCAACCACACCGCCC
0_hypothetical	E5	Tb927.1.2000_for	GGGGACAAGTTTGTACAAAAAAGCAGGCTATGCGAATGTTTTGCGTCGTC	Tb927.1.2000_rev	GGGGACCACTTTGTACAAGAAAGCTGGGTTCAGGTGGTGATACCACCATACG
0_hypothetical	E6	Tb927.9.3010_for	GGGGACAAGTTTGTACAAAAAAGCAGGCTATGAGTTCTAAGAGCAGTGTGGGA	Tb927.9.3010_rev	GGGGACCACTTTGTACAAGAAAGCTGGGTCTATGATCCAGCGGCCGAAGT
0_hypothetical	E7	Tb927.11.3040_for	GGGGACAAGTTTGTACAAAAAAGCAGGCTATGGGACGTAGGCTGCGG	Tb927.11.3040_rev	GGGGACCACTTTGTACAAGAAAGCTGGGTTTAATGCAGAAACCGCATGCGA
0_hypothetical	E8	Tb927.3.5655_for	GGGGACAAGTTTGTACAAAAAAGCAGGCTATGCTAGTTAGCGCCAGCGA	Tb927.3.5655_rev	GGGGACCACTTTGTACAAGAAAGCTGGGTTCACCTGAAGATGACGGTACATTTTTT
0_hypothetical	E9	Tb927.10.260_for	GGGGACAAGTTTGTACAAAAAAGCAGGCTATGGTCACTCAAACTGAAATGGAGG	Tb927.10.260_rev	GGGGACCACTTTGTACAAGAAAGCTGGGTCTACGGTAATGGCTTCGCCG
0_hypothetical	E10	Tb927.10.11585_for	GGGGACAAGTTTGTACAAAAAAGCAGGCTATGGAGTGTGTATTTGTCTGTAGAAGGA	Tb927.10.11585_rev	GGGGACCACTTTGTACAAGAAAGCTGGGTTCACATGTGTGTTTGTTTGTCTGTTTG
0_hypothetical	E11	Tb927.11.7260_for	GGGGACAAGTTTGTACAAAAAAGCAGGCTATGAGACAACCCTTTCCGACTACT	Tb927.11.7260_rev	GGGGACCACTTTGTACAAGAAAGCTGGGTTTATGACGAGTCTGCTTTTGCGT
0_hypothetical	E12	Tb927.7.4010_for	GGGGACAAGTTTGTACAAAAAAGCAGGCTATGAGTAACACACGCCAGCG	Tb927.7.4010_rev	GGGGACCACTTTGTACAAGAAAGCTGGGTTCATTCACTCCGTTGACGCTTC
0_hypothetical	E13	Tb927.2.1290_for	GGGGACAAGTTTGTACAAAAAAGCAGGCTATGTTCCCTTTCGTGTTCCCTTTT	Tb927.2.1290_rev	GGGGACCACTTTGTACAAGAAAGCTGGGTTTAGAATGTATTGCCATTTTTTTCGTTGTT
0_hypothetical	E14	Tb927.4.1770_for	GGGGACAAGTTTGTACAAAAAAGCAGGCTATGTTTCGGGTTTCCAGACGG	Tb927.4.1770_rev	GGGGACCACTTTGTACAAGAAAGCTGGGTTTAGGTCTTCCATCCGGCATTCT
0_hypothetical	E15	Tb927.7.2200_for	GGGGACAAGTTTGTACAAAAAAGCAGGCTATGCGCCTGGCTGTAAAGC	Tb927.7.2200_rev	GGGGACCACTTTGTACAAGAAAGCTGGGTCTACAACTTCAACATTTGCTTCACAGC
0_hypothetical	E16	Tb927.10.4090_for	GGGGACAAGTTTGTACAAAAAAGCAGGCTATGTTTTTGCGACCTATCGCGC	Tb927.10.4090_rev	GGGGACCACTTTGTACAAGAAAGCTGGGTTCAATCGTATACTTTGAGCGCAACT
0_hypothetical	E17	Tb927.10.7205_for	GGGGACAAGTTTGTACAAAAAAGCAGGCTATGTCTAATGCAACTCCCGTTACTG	Tb927.10.7205_rev	GGGGACCACTTTGTACAAGAAAGCTGGGTCTAAACTGGACCTCTGGGATGATTCC
0_hypothetical	E18	Tb927.1.305_for	GGGGACAAGTTTGTACAAAAAAGCAGGCTATGTATGAAGGTTTCTGTGCAAGAAGC	Tb927.1.305_rev	GGGGACCACTTTGTACAAGAAAGCTGGGTTCAAACATAAATATTACCCATCAACAGTTG
0_hypothetical	E19	Tb927.10.4960_for	GGGGACAAGTTTGTACAAAAAAGCAGGCTATGGGGTACGACAGACGATCG	Tb927.10.4960_rev	GGGGACCACTTTGTACAAGAAAGCTGGGTTTACGGCGGCGCCCGCCG
0_hypothetical	E21	Tb927.11.10075_for	GGGGACAAGTTTGTACAAAAAAGCAGGCTATGACTGACGTTAACAGATATCTTCAAAGT	Tb927.11.10075_rev	GGGGACCACTTTGTACAAGAAAGCTGGGTTTACTGAAAGGGCAAACCACACG
0_hypothetical	E22	Tb927.10.7375_for	GGGGACAAGTTTGTACAAAAAAGCAGGCTATGCTAGGTATTACGCCGCAAC	Tb927.10.7375_rev	GGGGACCACTTTGTACAAGAAAGCTGGGTCTATAGGGGTGTAATACCCCACTCTG
0_hypothetical	E23	Tb927.4.2330_for	GGGGACAAGTTTGTACAAAAAAGCAGGCTATGTTTAGGAGGACATTCTTTACACCAAT	Tb927.4.2330_rev	GGGGACCACTTTGTACAAGAAAGCTGGGTCTACGGTTTAATTTGACGGCAGTCC
0_hypothetical	E24	Tb927.8.2391_for	GGGGACAAGTTTGTACAAAAAAGCAGGCTATGAAGTACCGACAATGTTCTCTGAC	Tb927.8.2391_rev	GGGGACCACTTTGTACAAGAAAGCTGGGTTTATAGGCCCACATTATGAAGGGAAGG
0_hypothetical	F1	Tb927.8.595_for	GGGGACAAGTTTGTACAAAAAAGCAGGCTATGGGACATTCAGCAAAAATTACTAGAGG	Tb927.8.595_rev	GGGGACCACTTTGTACAAGAAAGCTGGGTCTACGCCTCTGGTCCCTTTACC
0_hypothetical	F2	Tb927.9.2980_for	GGGGACAAGTTTGTACAAAAAAGCAGGCTATGAGTTTTGGCCTTGAATACAGCG	Tb927.9.2980_rev	GGGGACCACTTTGTACAAGAAAGCTGGGTTTAGGCAGTTGCGGTTAACCG
0_hypothetical	F3	Tb927.7.2105_for	GGGGACAAGTTTGTACAAAAAAGCAGGCTATGATGTCTAGGCGGATTTGTATTCG	Tb927.7.2105_rev	GGGGACCACTTTGTACAAGAAAGCTGGGTTCATCGCTTAACAAAGACGCGG
0_hypothetical	F4	Tb927.1.490_for	GGGGACAAGTTTGTACAAAAAAGCAGGCTATGCAATGTGCTTTGAGTGGGG	Tb927.1.490_rev	GGGGACCACTTTGTACAAGAAAGCTGGGTTTAATGTTGGACGCTGATGTGAGG
0_hypothetical	F5	Tb927.4.180_for	GGGGACAAGTTTGTACAAAAAAGCAGGCTATGCAAGGTGCTTTGAGTGGG	Tb927.4.180_rev	GGGGACCACTTTGTACAAGAAAGCTGGGTTTAATGTTGGACGCTGATGTGAGG
0_hypothetical	F6	Tb927.11.12365_for	GGGGACAAGTTTGTACAAAAAAGCAGGCTATGGAGGGTAATTTCTACGTTTCTGAC	Tb927.11.12365_rev	GGGGACCACTTTGTACAAGAAAGCTGGGTCTACTGTTTCGACCCCGAATCAC
0_hypothetical	F7	Tb927.9.1390_for	GGGGACAAGTTTGTACAAAAAAGCAGGCTATGCACGCCGCACCTTATG	Tb927.9.1390_rev	GGGGACCACTTTGTACAAGAAAGCTGGGTTCAAGCAATCACAACATCACGCA
0_hypothetical	F8	Tb927.11.5390_for	GGGGACAAGTTTGTACAAAAAAGCAGGCTATGAATCAGTCTAGTTCATTATGGGGCG	Tb927.11.5390_rev	GGGGACCACTTTGTACAAGAAAGCTGGGTCTACTTTCCTTTGCTTCCTGTCTTACC
0_hypothetical	F9	Tb927.10.1290_for	GGGGACAAGTTTGTACAAAAAAGCAGGCTATGTGGCGCGAAAAGTGGG	Tb927.10.1290_rev	GGGGACCACTTTGTACAAGAAAGCTGGGTTTAGCGCCTCAACAAACGTTTTG
0_hypothetical	F10	Tb927.7.4685_for	GGGGACAAGTTTGTACAAAAAAGCAGGCTATGAAGGGACGAGGCGGAG	Tb927.7.4685_rev	GGGGACCACTTTGTACAAGAAAGCTGGGTTCACTTTGTCGCTTCGTTTCGG
0_hypothetical	F11	Tb927.7.7495_for	GGGGACAAGTTTGTACAAAAAAGCAGGCTATGCTGCAGAACCAGCAGTC	Tb927.7.7495_rev	GGGGACCACTTTGTACAAGAAAGCTGGGTTTACGCAAACCGCCGCAG
0_hypothetical	F12	Tb927.1.1025_for	GGGGACAAGTTTGTACAAAAAAGCAGGCTATGTTTCGCTCGTCGATGGTAG	Tb927.1.1025_rev	GGGGACCACTTTGTACAAGAAAGCTGGGTCTAAAGAACAAGTGATCGACCATCGT
0_hypothetical	F13	Tb927.9.6440_for	GGGGACAAGTTTGTACAAAAAAGCAGGCTATGTTATACGACCGCAGCATAGC	Tb927.9.6440_rev	GGGGACCACTTTGTACAAGAAAGCTGGGTTCACCCGCTTTCCTCCGG
0_hypothetical	F14	Tb927.8.2185_for	GGGGACAAGTTTGTACAAAAAAGCAGGCTATGACTCGAATACCTGGGGTCATC	Tb927.8.2185_rev	GGGGACCACTTTGTACAAGAAAGCTGGGTTCACCGAAACATCCTGTCGTCG
0_hypothetical	F15	Tb927.9.9100_for	GGGGACAAGTTTGTACAAAAAAGCAGGCTATGTCATACCAATCCAGCGAAGC	Tb927.9.9100_rev	GGGGACCACTTTGTACAAGAAAGCTGGGTTTAGTGCTCTTCATTCAGCCGTTT
0_hypothetical	F16	Tb927.4.5210_for	GGGGACAAGTTTGTACAAAAAAGCAGGCTATGAGTGACTCTGTTCGCAAGAAG	Tb927.4.5210_rev	GGGGACCACTTTGTACAAGAAAGCTGGGTTCACTCTTCCTTTTTCAGAAGCTGC
0_hypothetical	F17	Tb927.3.700_for	GGGGACAAGTTTGTACAAAAAAGCAGGCTATGTTACGCCGTAGCGTACCG	Tb927.3.700_rev	GGGGACCACTTTGTACAAGAAAGCTGGGTTTATGGATGAAGCGAATGCGGATC
0_hypothetical	F18	Tb927.7.6465_for	GGGGACAAGTTTGTACAAAAAAGCAGGCTATGTGGAGAATATCCAATGTGCTCCG	Tb927.7.6465_rev	GGGGACCACTTTGTACAAGAAAGCTGGGTTCATCCTTTGTCGCACATGTTTAGC
0_hypothetical	F19	Tb927.9.1520_for	GGGGACAAGTTTGTACAAAAAAGCAGGCTATGGCTCCCGCAGTAATTGAGA	Tb927.9.1520_rev	GGGGACCACTTTGTACAAGAAAGCTGGGTTTACTCATCAAAACAGATCTGGAAATGTGT
0_hypothetical	F20	Tb927.9.15829_for	GGGGACAAGTTTGTACAAAAAAGCAGGCTATGAATTCCTCTTGTGTAAATTCAGTTATG	Tb927.9.15829_rev	GGGGACCACTTTGTACAAGAAAGCTGGGTTTATATATTTGTTACTTGGCGTTTTAACAG
0_hypothetical	F21	Tb927.3.1985_for	GGGGACAAGTTTGTACAAAAAAGCAGGCTATGAGCTCACTATTGTCTGCAGC	Tb927.3.1985_rev	GGGGACCACTTTGTACAAGAAAGCTGGGTCTAGGAAATAAAGACAAGGTTGAGCAACC
0_hypothetical	F22	Tb927.4.780_for	GGGGACAAGTTTGTACAAAAAAGCAGGCTATGTATGCCCTGGACTACAGGAAT	Tb927.4.780_rev	GGGGACCACTTTGTACAAGAAAGCTGGGTTCAGTTATGCGTAAATATTTTCCAACCGA
0_hypothetical	F23	Tb927.1.2200_for	GGGGACAAGTTTGTACAAAAAAGCAGGCTATGCGTTTGTATCTACAGGGGAGA	Tb927.1.2200_rev	GGGGACCACTTTGTACAAGAAAGCTGGGTCTATTTCTCCGCCTCGAGGTTCA
0_hypothetical	F24	Tb927.9.3780_for	GGGGACAAGTTTGTACAAAAAAGCAGGCTATGGTGTCTGCAACGGCAG	Tb927.9.3780_rev	GGGGACCACTTTGTACAAGAAAGCTGGGTTCACACTGCCTTTCGGACAAG
0_hypothetical	G1	Tb927.9.3550_for	GGGGACAAGTTTGTACAAAAAAGCAGGCTATGCGCATTTTCGTACACGTACG	Tb927.9.3550_rev	GGGGACCACTTTGTACAAGAAAGCTGGGTCTACGCATCTGTGACAACCTCC
0_hypothetical	G2	Tb927.9.8815_for	GGGGACAAGTTTGTACAAAAAAGCAGGCTATGCTTCGTCGGTCGTACTCC	Tb927.9.8815_rev	GGGGACCACTTTGTACAAGAAAGCTGGGTTTAGTACTTCCACCACAGGAACGG
0_hypothetical	G3	Tb927.8.6190_for	GGGGACAAGTTTGTACAAAAAAGCAGGCTATGTTTCGCATGACTCGGCTG	Tb927.8.6190_rev	GGGGACCACTTTGTACAAGAAAGCTGGGTTCATTCCACCTTAGTCGTGAGTGA
0_hypothetical	G4	Tb927.11.155_for	GGGGACAAGTTTGTACAAAAAAGCAGGCTATGTTACGGAGGTTACACGTCCC	Tb927.11.155_rev	GGGGACCACTTTGTACAAGAAAGCTGGGTTTAGCGGCCGTCTCCACC
0_hypothetical	G5	Tb927.7.1171_for	GGGGACAAGTTTGTACAAAAAAGCAGGCTATGGCCACTACTCGGGCG	Tb927.7.1171_rev	GGGGACCACTTTGTACAAGAAAGCTGGGTTCAGATCACTAATCTGGTTGAACCGC
0_hypothetical	G6	Tb927.7.4680_for	GGGGACAAGTTTGTACAAAAAAGCAGGCTATGGGAAAGCAAAAGAAGCGGC	Tb927.7.4680_rev	GGGGACCACTTTGTACAAGAAAGCTGGGTTCAACGATTCGTCACTCGTGC
0_hypothetical	G7	Tb927.8.6890_for	GGGGACAAGTTTGTACAAAAAAGCAGGCTATGATTCGTTCACTTGGATATCGCC	Tb927.8.6890_rev	GGGGACCACTTTGTACAAGAAAGCTGGGTCTACGCCTTGAAGAATAAGGCAACA
0_hypothetical	G8	Tb927.7.7560_for	GGGGACAAGTTTGTACAAAAAAGCAGGCTATGTTCCCTTTCGTGTTCCCTTTT	Tb927.7.7560_rev	GGGGACCACTTTGTACAAGAAAGCTGGGTTCATGAGTTCTGCAATGATGAGGGA
0_hypothetical	G9	Tb927.9.10850_for	GGGGACAAGTTTGTACAAAAAAGCAGGCTATGGGCGATCCTTTTGTGCG	Tb927.9.10850_rev	GGGGACCACTTTGTACAAGAAAGCTGGGTTCAAGTATTATGCAAATCCCTCTTCCG
0_hypothetical	G10	Tb927.2.4955_for	GGGGACAAGTTTGTACAAAAAAGCAGGCTATGGACTTTATGAATACAAGCGAGACAG	Tb927.2.4955_rev	GGGGACCACTTTGTACAAGAAAGCTGGGTTCACACCTCGGTGCCCCG
0_hypothetical	G11	Tb927.10.5410_for	GGGGACAAGTTTGTACAAAAAAGCAGGCTATGAGATTGCCAAATGTAAGTTATTGCG	Tb927.10.5410_rev	GGGGACCACTTTGTACAAGAAAGCTGGGTCTAATAAGCAGGGAGAGTGACGCC
0_hypothetical	G12	Tb927.9.17760_for	GGGGACAAGTTTGTACAAAAAAGCAGGCTATGAGAAACTTTGAGGACAGGGGG	Tb927.9.17760_rev	GGGGACCACTTTGTACAAGAAAGCTGGGTTTATAAAAACACCAACGTGTGCCGA
0_hypothetical	G13	Tb927.10.7150_for	GGGGACAAGTTTGTACAAAAAAGCAGGCTATGTTTGGGCGTATGTTTGATATGTGT	Tb927.10.7150_rev	GGGGACCACTTTGTACAAGAAAGCTGGGTCTACATGCATGAGCGCCGG
0_hypothetical	G14	Tb927.2.3420_for	GGGGACAAGTTTGTACAAAAAAGCAGGCTATGGTTTCCAGGGACTCCCG	Tb927.2.3420_rev	GGGGACCACTTTGTACAAGAAAGCTGGGTTCATTCTTCTGTAAAGGGCGGGA
0_hypothetical	G15	Tb927.11.600_for	GGGGACAAGTTTGTACAAAAAAGCAGGCTATGTCGGCTAAGGCAGCTCC	Tb927.11.600_rev	GGGGACCACTTTGTACAAGAAAGCTGGGTCTACTTAGTGAAAGCTTCAGCAAGCT
0_hypothetical	G16	Tb927.11.435_for	GGGGACAAGTTTGTACAAAAAAGCAGGCTATGAATTTTGTTAAGCGTTTGGTTTACTCA	Tb927.11.435_rev	GGGGACCACTTTGTACAAGAAAGCTGGGTTTAGTTACGCGGGACGCCA
0_hypothetical	G17	Tb927.11.12960_for	GGGGACAAGTTTGTACAAAAAAGCAGGCTATGAATTCCTACTCGCGGCTCT	Tb927.11.12960_rev	GGGGACCACTTTGTACAAGAAAGCTGGGTTTAGGTTCTCGTTTGCCCAATGTATT
0_hypothetical	G18	Tb927.11.12200_for	GGGGACAAGTTTGTACAAAAAAGCAGGCTATGAGCAGTCATTTCTCCGGTAGG	Tb927.11.12200_rev	GGGGACCACTTTGTACAAGAAAGCTGGGTTCACTCCACTGGTGGTCTCTTTT
0_hypothetical	G19	Tb927.11.365_for	GGGGACAAGTTTGTACAAAAAAGCAGGCTATGACCCTTAGCAGCGATGCG	Tb927.11.365_rev	GGGGACCACTTTGTACAAGAAAGCTGGGTTTATGGCTGACAACTGCGATACC
0_hypothetical	G20	Tb927.9.8905_for	GGGGACAAGTTTGTACAAAAAAGCAGGCTATGGCGTTGTTTTCGTGCTTTC	Tb927.9.8905_rev	GGGGACCACTTTGTACAAGAAAGCTGGGTTCACCAACGATCGCTCTTGC
0_hypothetical	G21	Tb927.10.7925_for	GGGGACAAGTTTGTACAAAAAAGCAGGCTATGGGAACGAAGGAAAATGCAACA	Tb927.10.7925_rev	GGGGACCACTTTGTACAAGAAAGCTGGGTCTATAGCCGATTTCCTTTCTGCACA
0_hypothetical	G22	Tb927.11.2461_for	GGGGACAAGTTTGTACAAAAAAGCAGGCTATGGCCGTGCCTCGTTGG	Tb927.11.2461_rev	GGGGACCACTTTGTACAAGAAAGCTGGGTTCAGTCGCTGCCGGTGTT
0_hypothetical	G23	Tb927.3.1720_for	GGGGACAAGTTTGTACAAAAAAGCAGGCTATGGCTGACGTTTTGGATCGTG	Tb927.3.1720_rev	GGGGACCACTTTGTACAAGAAAGCTGGGTCTACCAGAATTTCCACCAGAATTTAGTCT
0_hypothetical	G24	Tb927.6.4580_for	GGGGACAAGTTTGTACAAAAAAGCAGGCTATGCTTCGACGGAGCATCCT	Tb927.6.4580_rev	GGGGACCACTTTGTACAAGAAAGCTGGGTTCAACTATCTTCATCAACCCCAAAAATACC
0_hypothetical	H1	Tb927.9.7282_for	GGGGACAAGTTTGTACAAAAAAGCAGGCTATGTGTGGAGGCGACGCT	Tb927.9.7282_rev	GGGGACCACTTTGTACAAGAAAGCTGGGTCTATAGATTCCTGGCTGCCTTGAAAC
0_hypothetical	H2	Tb927.11.5365_for	GGGGACAAGTTTGTACAAAAAAGCAGGCTATGTTCCGGCAGCGGCTT	Tb927.11.5365_rev	GGGGACCACTTTGTACAAGAAAGCTGGGTTTAAGCCGATATTCGCGTTTTTCCA
0_hypothetical	H3	Tb927.1.5160_for	GGGGACAAGTTTGTACAAAAAAGCAGGCTATGGCGAGTCACAACGAATATTTGG	Tb927.1.5160_rev	GGGGACCACTTTGTACAAGAAAGCTGGGTTCAAGTCAGCCCTTGTCTAACATACA
0_hypothetical	H4	Tb927.8.2470_for	GGGGACAAGTTTGTACAAAAAAGCAGGCTATGCCCATGCTTCAACCGTAC	Tb927.8.2470_rev	GGGGACCACTTTGTACAAGAAAGCTGGGTCTATGCAAAGATTTTCCCCCAGAACT
0_hypothetical	H5	Tb927.11.7475_for	GGGGACAAGTTTGTACAAAAAAGCAGGCTATGTCCGTTGTTTGTGCATTCAAAG	Tb927.11.7475_rev	GGGGACCACTTTGTACAAGAAAGCTGGGTCTACTTTTTCTTGATTGGCTTCTTTTGCC
0_hypothetical	H6	Tb927.10.7300_for	GGGGACAAGTTTGTACAAAAAAGCAGGCTATGTCGACTGTGGCGGAGC	Tb927.10.7300_rev	GGGGACCACTTTGTACAAGAAAGCTGGGTTCATCGCTTTTTGCATTTCCCATC
0_hypothetical	H7	Tb927.7.1860_for	GGGGACAAGTTTGTACAAAAAAGCAGGCTATGCCTGGGCGAAGAGGG	Tb927.7.1860_rev	GGGGACCACTTTGTACAAGAAAGCTGGGTTCAGCATCCTACCACAGTACCG
0_hypothetical	H9	Tb927.6.3140_for	GGGGACAAGTTTGTACAAAAAAGCAGGCTATGTCGGAGACAATACCCGTTTCA	Tb927.6.3140_rev	GGGGACCACTTTGTACAAGAAAGCTGGGTTTACATATCGTCCTCATCCTTTTCTGGA
0_hypothetical	H10	Tb927.11.16615_for	GGGGACAAGTTTGTACAAAAAAGCAGGCTATGTTTCTCCCCACGCATTTGT	Tb927.11.16615_rev	GGGGACCACTTTGTACAAGAAAGCTGGGTTTACTTCGCCTTCCTCGCAAGA
0_hypothetical	H11	Tb927.8.5150_for	GGGGACAAGTTTGTACAAAAAAGCAGGCTATGGCGTTTCAAACACCGTGG	Tb927.8.5150_rev	GGGGACCACTTTGTACAAGAAAGCTGGGTTTACCACAGTTTCCAAATCGAGTGG
0_hypothetical	H12	Tb927.5.3340_for	GGGGACAAGTTTGTACAAAAAAGCAGGCTATGCAGAGCCAAATGATGCTCA	Tb927.5.3340_rev	GGGGACCACTTTGTACAAGAAAGCTGGGTCTACTCCATACCGAGGGATCCCG
0_hypothetical	H13	Tb927.1.800_for	GGGGACAAGTTTGTACAAAAAAGCAGGCTATGAATTCACACATCTTCCATTTCCCA	Tb927.1.800_rev	GGGGACCACTTTGTACAAGAAAGCTGGGTCTACGGCCTCAAGTGTGTAGTGG
0_hypothetical	H14	Tb927.10.13341_for	GGGGACAAGTTTGTACAAAAAAGCAGGCTATGCTTCAGGCGCAAGGTTC	Tb927.10.13341_rev	GGGGACCACTTTGTACAAGAAAGCTGGGTTTAATGAGTGTTAACATCCATTTTCTCCTG
0_hypothetical	H15	Tb927.6.2610_for	GGGGACAAGTTTGTACAAAAAAGCAGGCTATGACATCTCCCATCCTACGAGTTG	Tb927.6.2610_rev	GGGGACCACTTTGTACAAGAAAGCTGGGTTTACAAGCGAGCCCCCATATCA
0_hypothetical	H16	Tb927.11.665_for	GGGGACAAGTTTGTACAAAAAAGCAGGCTATGCTGCGTCGATCTCTCATCG	Tb927.11.665_rev	GGGGACCACTTTGTACAAGAAAGCTGGGTCTAATACCACCGCCACCGTTC
0_hypothetical	H17	Tb927.7.1520_for	GGGGACAAGTTTGTACAAAAAAGCAGGCTATGGGTGGGGATGACCAAATATTTC	Tb927.7.1520_rev	GGGGACCACTTTGTACAAGAAAGCTGGGTCTAACGCTGGCACTTCGTGC
0_hypothetical	H18	Tb927.6.3910_for	GGGGACAAGTTTGTACAAAAAAGCAGGCTATGTCGTTTGATATGGAAGTACTGCG	Tb927.6.3910_rev	GGGGACCACTTTGTACAAGAAAGCTGGGTTCAAAGAAGGTAAGGTGCATTTGATAATAT
0_hypothetical	H19	Tb927.4.830_for	GGGGACAAGTTTGTACAAAAAAGCAGGCTATGGAAAAGGAGAACAAAAGCATCTGT	Tb927.4.830_rev	GGGGACCACTTTGTACAAGAAAGCTGGGTTTACGACACAATGCCTTCCTCCT
0_hypothetical	H20	Tb927.11.9295_for	GGGGACAAGTTTGTACAAAAAAGCAGGCTATGGGAGATTCTGAAGCCACTGC	Tb927.11.9295_rev	GGGGACCACTTTGTACAAGAAAGCTGGGTTCATGTGTGGACGTCAAAAGACAG
0_hypothetical	H21	Tb927.11.14495_for	GGGGACAAGTTTGTACAAAAAAGCAGGCTATGCAACATGACAATGTTGGAGACC	Tb927.11.14495_rev	GGGGACCACTTTGTACAAGAAAGCTGGGTTCATGTGATTAGTGCGCTCTTACG
0_hypothetical	H22	Tb927.5.3895_for	GGGGACAAGTTTGTACAAAAAAGCAGGCTATGCAAAGCAGTTGTATGCGC	Tb927.5.3895_rev	GGGGACCACTTTGTACAAGAAAGCTGGGTTCAAAATGTCACAACTTCATCAGAATCTTC
0_hypothetical	H23	Tb927.7.4515_for	GGGGACAAGTTTGTACAAAAAAGCAGGCTATGGAACAGCCGCAGCAAC	Tb927.7.4515_rev	GGGGACCACTTTGTACAAGAAAGCTGGGTCTATGGTTGAACGGGTTTTGGCA
0_hypothetical	H24	Tb927.10.10640_for	GGGGACAAGTTTGTACAAAAAAGCAGGCTATGCCTCGTGCATATTACAAACCC	Tb927.10.10640_rev	GGGGACCACTTTGTACAAGAAAGCTGGGTTCAAGCAGGAGGGGCCCG
0_hypothetical	I1	Tb927.10.8291_for	GGGGACAAGTTTGTACAAAAAAGCAGGCTATGCATCTTGATGAAATGTTGAAAGAGATT	Tb927.10.8291_rev	GGGGACCACTTTGTACAAGAAAGCTGGGTTCAGTCATCAACTTCAGTTTCAAAAGGC
0_hypothetical	I2	Tb927.11.11385_for	GGGGACAAGTTTGTACAAAAAAGCAGGCTATGGCTGAAAAAATAGCAAGCGAC	Tb927.11.11385_rev	GGGGACCACTTTGTACAAGAAAGCTGGGTTTATCCGTGAAATATCGCCGTCG
0_hypothetical	I3	Tb927.8.6990_for	GGGGACAAGTTTGTACAAAAAAGCAGGCTATGCAGGGTTCAACAGAGAAAACTAC	Tb927.8.6990_rev	GGGGACCACTTTGTACAAGAAAGCTGGGTCTAAAAAAAAAGACCGCAGCACGT
0_hypothetical	I4	Tb927.10.2340_for	GGGGACAAGTTTGTACAAAAAAGCAGGCTATGTCTAAACAAGAACTTCAACTCACTGA	Tb927.10.2340_rev	GGGGACCACTTTGTACAAGAAAGCTGGGTCTAAAGTATTAAAAGCACCACCAGAATAAG
0_hypothetical	I5	Tb927.10.14350_for	GGGGACAAGTTTGTACAAAAAAGCAGGCTATGTGGCGCATTTCTCCGC	Tb927.10.14350_rev	GGGGACCACTTTGTACAAGAAAGCTGGGTTCACGCGGTACCGCTTTTTG
0_hypothetical	I6	Tb927.4.5335_for	GGGGACAAGTTTGTACAAAAAAGCAGGCTATGCAGGTTGCAACGGAGAAA	Tb927.4.5335_rev	GGGGACCACTTTGTACAAGAAAGCTGGGTCTAAAAAAATAGACCGCAGCACGTC
0_hypothetical	I7	Tb927.8.2735_for	GGGGACAAGTTTGTACAAAAAAGCAGGCTATGAAGCGCTTCATACAAAGCCG	Tb927.8.2735_rev	GGGGACCACTTTGTACAAGAAAGCTGGGTTCACGAGTCCCTGTACCGTC
0_hypothetical	I9	Tb927.7.6770_for	GGGGACAAGTTTGTACAAAAAAGCAGGCTATGAGTTCTGCCCAGTTTGAAGC	Tb927.7.6770_rev	GGGGACCACTTTGTACAAGAAAGCTGGGTCTACTGAGGTGGCTTCCACGG
0_hypothetical	I10	Tb927.11.8165_for	GGGGACAAGTTTGTACAAAAAAGCAGGCTATGTCGCAAAAAGGTTTGAAGGGT	Tb927.11.8165_rev	GGGGACCACTTTGTACAAGAAAGCTGGGTTCATCCCTTCTTGTCGCCCTT
0_hypothetical	I11	Tb927.1.2985_for	GGGGACAAGTTTGTACAAAAAAGCAGGCTATGGCCGGGGAAGCGGGA	Tb927.1.2985_rev	GGGGACCACTTTGTACAAGAAAGCTGGGTTCATTGGTTCCTTTTCTCACGAACTT
0_hypothetical	I12	Tb927.6.590_for	GGGGACAAGTTTGTACAAAAAAGCAGGCTATGACTGAAAACATCGAAGCGGT	Tb927.6.590_rev	GGGGACCACTTTGTACAAGAAAGCTGGGTTTAATACGGATTGTTGCGGTCCTC
0_hypothetical	I13	Tb927.11.15780_for	GGGGACAAGTTTGTACAAAAAAGCAGGCTATGACAACAAGTGGAGTTGAATCGG	Tb927.11.15780_rev	GGGGACCACTTTGTACAAGAAAGCTGGGTCTACAGACCTAACTCCTTTAGGCACG
0_hypothetical	I14	Tb927.11.16540_for	GGGGACAAGTTTGTACAAAAAAGCAGGCTATGACGAGTGAGCGCGTGG	Tb927.11.16540_rev	GGGGACCACTTTGTACAAGAAAGCTGGGTTCACTTCGTGAGCTCCTGTATCA
0_hypothetical	I15	Tb927.11.2190_for	GGGGACAAGTTTGTACAAAAAAGCAGGCTATGAGCGAGCAACGAGTTTCAC	Tb927.11.2190_rev	GGGGACCACTTTGTACAAGAAAGCTGGGTTTAATTGATTTTCGACTCCTTGGGTTTCA
0_hypothetical	I16	Tb927.8.3521_for	GGGGACAAGTTTGTACAAAAAAGCAGGCTATGACCAATGAGCTCATGCGTC	Tb927.8.3521_rev	GGGGACCACTTTGTACAAGAAAGCTGGGTCTACACGCTACCTCTATTTATACCCTGA
0_hypothetical	I17	Tb927.9.2280_for	GGGGACAAGTTTGTACAAAAAAGCAGGCTATGGGACGCAGCAACAACC	Tb927.9.2280_rev	GGGGACCACTTTGTACAAGAAAGCTGGGTTCAACGCTTACCGCCTTTCTTC
0_hypothetical	I18	Tb927.9.13310_for	GGGGACAAGTTTGTACAAAAAAGCAGGCTATGGGGTTCTCTCTTGCTGGG	Tb927.9.13310_rev	GGGGACCACTTTGTACAAGAAAGCTGGGTTTATCCAAACAAAAGTGTGAACACAATCAC

0_hypothetical	I19	Tb927.9.17690_for	GGGGACAAGTTTGTACAAAAAAGCAGGCTATGCCGAGGACATTCGAAGTTC	Tb927.9.17690_rev	GGGGACCACTTTGTACAAGAAAGCTGGGTTCAATTCAAAAGGATTTGGTTCTTTCCTTT
0_hypothetical	I20	Tb927.7.7390_for	GGGGACAAGTTTGTACAAAAAAGCAGGCTATGAACATGCAAATCCTCCACGAA	Tb927.7.7390_rev	GGGGACCACTTTGTACAAGAAAGCTGGGTTCATCTAGTGCCCTTCATCTGCG
0_hypothetical	I21	Tb927.10.10030_for	GGGGACAAGTTTGTACAAAAAAGCAGGCTATGTCCGCCGAGGCCGTA	Tb927.10.10030_rev	GGGGACCACTTTGTACAAGAAAGCTGGGTCTACGAGTTCTCCTCCGGAGAG
0_hypothetical	I22	Tb927.8.8235_for	GGGGACAAGTTTGTACAAAAAAGCAGGCTATGTTTTATGATATCAAAGATGCTGCCTCA	Tb927.8.8235_rev	GGGGACCACTTTGTACAAGAAAGCTGGGTTCAAAAAGCCTTTAGGAAATCTGAGTTTCT
0_hypothetical	I23	Tb927.10.11030_for	GGGGACAAGTTTGTACAAAAAAGCAGGCTATGATGTTCGGTCGCCCCG	Tb927.10.11030_rev	GGGGACCACTTTGTACAAGAAAGCTGGGTCTAGAATTCCTCTTCGTCCTCCTCC
0_hypothetical	I24	Tb927.10.4190_for	GGGGACAAGTTTGTACAAAAAAGCAGGCTATGCGGGAGAACGGTGGG	Tb927.10.4190_rev	GGGGACCACTTTGTACAAGAAAGCTGGGTTCACACAACGGTTGGACTCCT
0_hypothetical	J1	Tb927.9.8650_for	GGGGACAAGTTTGTACAAAAAAGCAGGCTATGGACCCAGCACTGCAGC	Tb927.9.8650_rev	GGGGACCACTTTGTACAAGAAAGCTGGGTTTAGTACCTCTGGCCACCCTTTT
0_hypothetical	J2	Tb927.5.3090_for	GGGGACAAGTTTGTACAAAAAAGCAGGCTATGTCCTCCGGTTTCCATTTTCATG	Tb927.5.3090_rev	GGGGACCACTTTGTACAAGAAAGCTGGGTTTATTTGAATGGATTCTTGTCGTCCTCTT
0_hypothetical	J3	Tb927.9.16060_for	GGGGACAAGTTTGTACAAAAAAGCAGGCTATGCTATACGTAAGCGCTGGAGA	Tb927.9.16060_rev	GGGGACCACTTTGTACAAGAAAGCTGGGTCTAGGAGCGACACCGTAGTTTATCT
0_hypothetical	J4	Tb927.10.11530_for	GGGGACAAGTTTGTACAAAAAAGCAGGCTATGTACCTTGCGGTTTTCCAGG	Tb927.10.11530_rev	GGGGACCACTTTGTACAAGAAAGCTGGGTTCATCCGGTGTATGTTAATATGACACG
0_hypothetical	J5	Tb927.3.5740_for	GGGGACAAGTTTGTACAAAAAAGCAGGCTATGTGTGATGGCAGGAACTCGC	Tb927.3.5740_rev	GGGGACCACTTTGTACAAGAAAGCTGGGTCTAGGATAGCGTGACGGTTTTTACC
0_hypothetical	J6	Tb927.3.1600_for	GGGGACAAGTTTGTACAAAAAAGCAGGCTATGCAGCCACCTCAAACGAATC	Tb927.3.1600_rev	GGGGACCACTTTGTACAAGAAAGCTGGGTCTATTCGTTCTCCGACAAAACTTCTTTC
0_hypothetical	J7	Tb927.5.4575_for	GGGGACAAGTTTGTACAAAAAAGCAGGCTATGACGGACACGTTTGAAGGTATTT	Tb927.5.4575_rev	GGGGACCACTTTGTACAAGAAAGCTGGGTTTAGGGGGTTTTCCCCTGGTC
0_hypothetical	J8	Tb927.4.1830_for	GGGGACAAGTTTGTACAAAAAAGCAGGCTATGTCGAACCACAACCACATAACA	Tb927.4.1830_rev	GGGGACCACTTTGTACAAGAAAGCTGGGTCATCCGCCATGCAGTGTG
0_hypothetical	J9	Tb927.9.7920_for	GGGGACAAGTTTGTACAAAAAAGCAGGCTATGTGTACGTGCTGCGTGG	Tb927.9.7920_rev	GGGGACCACTTTGTACAAGAAAGCTGGGTCTATACTGATAATTTATTGAGTTGGTTGCT
0_hypothetical	J10	Tb927.4.2660_for	GGGGACAAGTTTGTACAAAAAAGCAGGCTATGTTTATTGCCCGAACCCTTCC	Tb927.4.2660_rev	GGGGACCACTTTGTACAAGAAAGCTGGGTTTAATTCTTAAAGGGTGACCAGGGCT
0_hypothetical	J11	Tb927.11.10050_for	GGGGACAAGTTTGTACAAAAAAGCAGGCTATGGCATTTCGCCGCTTGG	Tb927.11.10050_rev	GGGGACCACTTTGTACAAGAAAGCTGGGTCTAACGAAATGGTGTAGGGAATCCC
0_hypothetical	J12	Tb927.8.4895_for	GGGGACAAGTTTGTACAAAAAAGCAGGCTATGACGTTTAAATTCTTTTACAATGTGCCG	Tb927.8.4895_rev	GGGGACCACTTTGTACAAGAAAGCTGGGTCTAAGGTCGAAGTAAATACTTATCATCGCC
0_hypothetical	J13	Tb927.11.9860_for	GGGGACAAGTTTGTACAAAAAAGCAGGCTATGCAACAAAGTCTCATTCCTGGAC	Tb927.11.9860_rev	GGGGACCACTTTGTACAAGAAAGCTGGGTTTATCGCTGAGCAAAGCTGAGC
0_hypothetical	J14	Tb927.5.4050_for	GGGGACAAGTTTGTACAAAAAAGCAGGCTATGAAAGCAACAACTAAATCAGGAGATAGC	Tb927.5.4050_rev	GGGGACCACTTTGTACAAGAAAGCTGGGTTCACGAACCCCCACGCGC
0_hypothetical	J15	Tb927.4.3600_for	GGGGACAAGTTTGTACAAAAAAGCAGGCTATGAGGCGTATGGATCCCGATG	Tb927.4.3600_rev	GGGGACCACTTTGTACAAGAAAGCTGGGTCTAGACATTGGCAGGTGCATCG
0_hypothetical	J16	Tb927.8.1490_for	GGGGACAAGTTTGTACAAAAAAGCAGGCTATGCGTCGTTCATTCTCACGC	Tb927.8.1490_rev	GGGGACCACTTTGTACAAGAAAGCTGGGTTCAGAAATCGTGGCAGCGTC
0_hypothetical	J17	Tb927.6.240_for	GGGGACAAGTTTGTACAAAAAAGCAGGCTATGATAAGGGAGCATATGGTACAATACAGT	Tb927.6.240_rev	GGGGACCACTTTGTACAAGAAAGCTGGGTTCATGTATTGAGCTTTAAAGGTCGTCG
0_hypothetical	J19	Tb927.7.4800_for	GGGGACAAGTTTGTACAAAAAAGCAGGCTATGTTGCAACGTACGACCTCG	Tb927.7.4800_rev	GGGGACCACTTTGTACAAGAAAGCTGGGTCTACATGGGCCTCCCGCT
0_hypothetical	J20	Tb927.11.15080_for	GGGGACAAGTTTGTACAAAAAAGCAGGCTATGGCGAACGTAATTGCCTCAG	Tb927.11.15080_rev	GGGGACCACTTTGTACAAGAAAGCTGGGTTTAAAATGAATAAGGTGCAAATCCGAAGAG
0_hypothetical	J21	Tb927.10.6200_for	GGGGACAAGTTTGTACAAAAAAGCAGGCTATGCTCCGACGCGTTGCT	Tb927.10.6200_rev	GGGGACCACTTTGTACAAGAAAGCTGGGTTTACACTGCCCTGTCAGGAACA
0_hypothetical	J22	Tb927.6.4130_for	GGGGACAAGTTTGTACAAAAAAGCAGGCTATGCCACCTGTGGTTAAGCG	Tb927.6.4130_rev	GGGGACCACTTTGTACAAGAAAGCTGGGTTTACGGCAATGGGTTCATCATGTC
0_hypothetical	J23	Tb927.3.2880_for	GGGGACAAGTTTGTACAAAAAAGCAGGCTATGGTTTACACTCGTTGGAAGTGTG	Tb927.3.2880_rev	GGGGACCACTTTGTACAAGAAAGCTGGGTCTACACGACTCGACTTAACTCCTCC
0_hypothetical	J24	Tb927.8.2170_for	GGGGACAAGTTTGTACAAAAAAGCAGGCTATGAGGCGATGTGCTTGTAAGATG	Tb927.8.2170_rev	GGGGACCACTTTGTACAAGAAAGCTGGGTTCACACCTTTTCTTCAGCACCAC
0_hypothetical	K1	Tb927.10.15320_for	GGGGACAAGTTTGTACAAAAAAGCAGGCTATGAAACGACTGGCGTCTTTTGG	Tb927.10.15320_rev	GGGGACCACTTTGTACAAGAAAGCTGGGTTCACATGTTTGCCACAGGTTCTG
0_hypothetical	K2	Tb927.7.7550_for	GGGGACAAGTTTGTACAAAAAAGCAGGCTATGACATTAATTGAAATTCTAGGCCACTAC	Tb927.7.7550_rev	GGGGACCACTTTGTACAAGAAAGCTGGGTTTACTGACCCAGTGCCTGGAG
0_hypothetical	K3	Tb927.2.2590_for	GGGGACAAGTTTGTACAAAAAAGCAGGCTATGTACCAGAGGACACGATTTTTGTG	Tb927.2.2590_rev	GGGGACCACTTTGTACAAGAAAGCTGGGTTCACTCAGTAGTTAGGCCGCG
0_hypothetical	K4	Tb927.11.15855_for	GGGGACAAGTTTGTACAAAAAAGCAGGCTATGTTTTGCTCCACGCCACTG	Tb927.11.15855_rev	GGGGACCACTTTGTACAAGAAAGCTGGGTTTATCCAGGTGGTTCGGGGAG
0_hypothetical	K5	Tb927.11.2825_for	GGGGACAAGTTTGTACAAAAAAGCAGGCTATGCTTACGACTGGACCGTTGA	Tb927.11.2825_rev	GGGGACCACTTTGTACAAGAAAGCTGGGTTCATTCATCTACCAATTCTGTAAAGAAACC
0_hypothetical	K6	Tb927.4.510_for	GGGGACAAGTTTGTACAAAAAAGCAGGCTATGGAGGTGGCTGCAATGAGT	Tb927.4.510_rev	GGGGACCACTTTGTACAAGAAAGCTGGGTCTAGCTTTGCTCCAGAGCATCAG
0_hypothetical	K7	Tb927.4.720_for	GGGGACAAGTTTGTACAAAAAAGCAGGCTATGCTGCGGCGACTGGTC	Tb927.4.720_rev	GGGGACCACTTTGTACAAGAAAGCTGGGTTTAAATGCTTTTTGGGAACACCAACTC
0_hypothetical	K8	Tb927.3.5350_for	GGGGACAAGTTTGTACAAAAAAGCAGGCTATGCCCGCACGTGCATCC	Tb927.3.5350_rev	GGGGACCACTTTGTACAAGAAAGCTGGGTTTAAGGACGAGCGCCACGG
0_hypothetical	K9	Tb927.8.4380_for	GGGGACAAGTTTGTACAAAAAAGCAGGCTATGTTTATGATGCTCAACGGTACCG	Tb927.8.4380_rev	GGGGACCACTTTGTACAAGAAAGCTGGGTTCAAGCAGTGGAGGAAACATACTCC
0_hypothetical	K10	Tb927.8.6430_for	GGGGACAAGTTTGTACAAAAAAGCAGGCTATGGTAAAGTTGACTCATGAAGATACGC	Tb927.8.6430_rev	GGGGACCACTTTGTACAAGAAAGCTGGGTTTAGAAACCCTTTGCAGTTTTCAATTCC
0_hypothetical	K11	Tb927.9.740_for	GGGGACAAGTTTGTACAAAAAAGCAGGCTATGCAAGGTGCTTTGAGTGGG	Tb927.9.740_rev	GGGGACCACTTTGTACAAGAAAGCTGGGTTCATAAAATATGTATCATCTCACAAGACAC
0_hypothetical	K12	Tb927.6.1240_for	GGGGACAAGTTTGTACAAAAAAGCAGGCTATGACTCGAAAACAAAAGCAGCG	Tb927.6.1240_rev	GGGGACCACTTTGTACAAGAAAGCTGGGTTTATGCCTTTGGCGTCTGGTG
0_hypothetical	K13	Tb927.3.2380_for	GGGGACAAGTTTGTACAAAAAAGCAGGCTATGGAAAAAAATGCGCATGGGC	Tb927.3.2380_rev	GGGGACCACTTTGTACAAGAAAGCTGGGTTCATTGCCGTGTTGCCCC
0_hypothetical	K14	Tb927.11.605_for	GGGGACAAGTTTGTACAAAAAAGCAGGCTATGAGGAGGCTGAGCGCA	Tb927.11.605_rev	GGGGACCACTTTGTACAAGAAAGCTGGGTTTACTTCACGCTTACTCCCTCGA
0_hypothetical	K15	Tb927.6.4020_for	GGGGACAAGTTTGTACAAAAAAGCAGGCTATGGTGCGCGCCCCTCCG	Tb927.6.4020_rev	GGGGACCACTTTGTACAAGAAAGCTGGGTTTACAAACTTCCCTTCAAATGGCAAGC
0_hypothetical	K16	Tb927.10.1810_for	GGGGACAAGTTTGTACAAAAAAGCAGGCTATGGAGGAAGCGGCTGCT	Tb927.10.1810_rev	GGGGACCACTTTGTACAAGAAAGCTGGGTCTACACTCCTAACTTCTGGTACGACC
0_hypothetical	K17	Tb927.7.5830_for	GGGGACAAGTTTGTACAAAAAAGCAGGCTATGAAGCCCGCCACTATTCCA	Tb927.7.5830_rev	GGGGACCACTTTGTACAAGAAAGCTGGGTTCAAACATCTGTTGTGGTGCACA
0_hypothetical	K18	Tb927.10.90_for	GGGGACAAGTTTGTACAAAAAAGCAGGCTATGTGCCTACTGGGTCGTTCA	Tb927.10.90_rev	GGGGACCACTTTGTACAAGAAAGCTGGGTTCATTCCAGTGGTGGAGAAACGG
0_hypothetical	K19	Tb927.10.3410_for	GGGGACAAGTTTGTACAAAAAAGCAGGCTATGCAACACTTTGTTGGTGGTACA	Tb927.10.3410_rev	GGGGACCACTTTGTACAAGAAAGCTGGGTTTAAGTGGCAAATACATTTACACTTAACTC
0_hypothetical	K20	Tb927.11.17010_for	GGGGACAAGTTTGTACAAAAAAGCAGGCTATGAGCTTAGGGAAAACTATTTATGTTGAC	Tb927.11.17010_rev	GGGGACCACTTTGTACAAGAAAGCTGGGTCTAGAATATATAAATTGCGTCATATGCTAC
0_hypothetical	K21	Tb927.11.12040_for	GGGGACAAGTTTGTACAAAAAAGCAGGCTATGGCTTCTTGCAGAACTGGGC	Tb927.11.12040_rev	GGGGACCACTTTGTACAAGAAAGCTGGGTCTAATAAGTGTACTCTTTACCAGTGTACCC
0_hypothetical	K22	Tb927.11.5950_for	GGGGACAAGTTTGTACAAAAAAGCAGGCTATGTTCTCGAAATCCGTCGCAC	Tb927.11.5950_rev	GGGGACCACTTTGTACAAGAAAGCTGGGTTCAAACGGTTGCCTGCTCC
0_hypothetical	K23	Tb927.10.8160_for	GGGGACAAGTTTGTACAAAAAAGCAGGCTATGCCCGTCCTAGTTGCGG	Tb927.10.8160_rev	GGGGACCACTTTGTACAAGAAAGCTGGGTCTACAGGCTGATGTACAAGAAGAATGC
0_hypothetical	L1	Tb927.2.2140_for	GGGGACAAGTTTGTACAAAAAAGCAGGCTATGTCATATGTTGAAAATGTGATGGCAC	Tb927.2.2140_rev	GGGGACCACTTTGTACAAGAAAGCTGGGTTTACTTCCCCTGCGCATCTGC
0_hypothetical	L2	Tb927.11.13800_for	GGGGACAAGTTTGTACAAAAAAGCAGGCTATGGATTCTGAAGTCTGCGATGAC	Tb927.11.13800_rev	GGGGACCACTTTGTACAAGAAAGCTGGGTCTACGTGACGTATTCGGAGCCA
0_hypothetical	L3	Tb927.11.7702_for	GGGGACAAGTTTGTACAAAAAAGCAGGCTATGAGTGGAAATGTGAAGTTCAGCA	Tb927.11.7702_rev	GGGGACCACTTTGTACAAGAAAGCTGGGTTCATGCGGGAGAAATCATTTCAACA
0_hypothetical	L4	Tb927.1.110_for	GGGGACAAGTTTGTACAAAAAAGCAGGCTATGAAAACAAAACGTTACAATGTTTTCCTA	Tb927.1.110_rev	GGGGACCACTTTGTACAAGAAAGCTGGGTTTAGGGGGAGCGGGTTCG
0_hypothetical	L5	Tb927.11.2960_for	GGGGACAAGTTTGTACAAAAAAGCAGGCTATGAGTGCTCTTCCTGATGCTAGC	Tb927.11.2960_rev	GGGGACCACTTTGTACAAGAAAGCTGGGTTTAAAGTGCTTTCATTTCAGATTCTTCCCC
0_hypothetical	L6	Tb927.9.9610_for	GGGGACAAGTTTGTACAAAAAAGCAGGCTATGCAAAAGGTCGGCGTCAA	Tb927.9.9610_rev	GGGGACCACTTTGTACAAGAAAGCTGGGTCTACGGGCTGCACAGGTGA
0_hypothetical	L7	Tb927.1.40_for	GGGGACAAGTTTGTACAAAAAAGCAGGCTATGAAACTGAAAGAGCTAATAGGTTGGC	Tb927.1.40_rev	GGGGACCACTTTGTACAAGAAAGCTGGGTTTAAAGTGGATTACAAATTTCATTGAAAAT
0_hypothetical	L8	Tb927.9.2055_for	GGGGACAAGTTTGTACAAAAAAGCAGGCTATGTTCAGTTGGAAGGATGTTCTCTC	Tb927.9.2055_rev	GGGGACCACTTTGTACAAGAAAGCTGGGTTTAATCCAACTGCGTTTCACCGC
0_hypothetical	L9	Tb927.6.811_for	GGGGACAAGTTTGTACAAAAAAGCAGGCTATGACTGTGTACTACTATTACGGGGAG	Tb927.6.811_rev	GGGGACCACTTTGTACAAGAAAGCTGGGTTTACATGACGTCTTCTTCATTTGGCC
0_hypothetical	L10	Tb927.4.3430_for	GGGGACAAGTTTGTACAAAAAAGCAGGCTATGGGTCAGGACCAATCCATGG	Tb927.4.3430_rev	GGGGACCACTTTGTACAAGAAAGCTGGGTTTAACGACTAAGTTCCTTTCTCGCC
0_hypothetical	L11	Tb927.6.1710_for	GGGGACAAGTTTGTACAAAAAAGCAGGCTATGTTACGACGGCTAGCTGCG	Tb927.6.1710_rev	GGGGACCACTTTGTACAAGAAAGCTGGGTTTAAACCTTAAGAAATTCCTGCGAGAGC
0_hypothetical	L12	Tb927.11.4670_for	GGGGACAAGTTTGTACAAAAAAGCAGGCTATGCCGGGCATTGAGCGT	Tb927.11.4670_rev	GGGGACCACTTTGTACAAGAAAGCTGGGTTTACGCTGAACATCCGTCAAGAATATC
0_hypothetical	L13	Tb927.9.1920_for	GGGGACAAGTTTGTACAAAAAAGCAGGCTATGCACAGTCGACGCATTTTTG	Tb927.9.1920_rev	GGGGACCACTTTGTACAAGAAAGCTGGGTTTACCGCATCTTCGGAAAAAAACAAG
0_hypothetical	L14	Tb927.9.7890_for	GGGGACAAGTTTGTACAAAAAAGCAGGCTATGTGTACGTGCAGCGTGG	Tb927.9.7890_rev	GGGGACCACTTTGTACAAGAAAGCTGGGTCTACAAACATTTGCTCCTTTCCCATACT
0_hypothetical	L15	Tb927.1.10_for	GGGGACAAGTTTGTACAAAAAAGCAGGCTATGATCGCGGGTTTCTGTTCG	Tb927.1.10_rev	GGGGACCACTTTGTACAAGAAAGCTGGGTCTATTGACTTCCAGCACTTTGTATAGAGTT
0_hypothetical	L16	Tb927.5.460_for	GGGGACAAGTTTGTACAAAAAAGCAGGCTATGGTTGTATTGAAGGGTGGCG	Tb927.5.460_rev	GGGGACCACTTTGTACAAGAAAGCTGGGTTCATCCTCCTTCATTACCATCACTGC
0_hypothetical	L17	Tb927.3.5560_for	GGGGACAAGTTTGTACAAAAAAGCAGGCTATGATTGACGATGTAAGAATTTGTCATCTC	Tb927.3.5560_rev	GGGGACCACTTTGTACAAGAAAGCTGGGTTCAGGAGGAACCGCTGCG
0_hypothetical	L18	Tb927.10.7390_for	GGGGACAAGTTTGTACAAAAAAGCAGGCTATGCTTCGCCACTCCAAGC	Tb927.10.7390_rev	GGGGACCACTTTGTACAAGAAAGCTGGGTTTAGCGTGTGCGTACCGTCG
0_hypothetical	L19	Tb927.10.15900_for	GGGGACAAGTTTGTACAAAAAAGCAGGCTATGTTTGGACGACGCGTTTACG	Tb927.10.15900_rev	GGGGACCACTTTGTACAAGAAAGCTGGGTTCAGTGAAGCACTTTCTTTACCCC
0_hypothetical	L20	Tb927.10.12610_for	GGGGACAAGTTTGTACAAAAAAGCAGGCTATGGCGCGGTGCATCATT	Tb927.10.12610_rev	GGGGACCACTTTGTACAAGAAAGCTGGGTTCACCCCTCGCGTGCTGA
0_hypothetical	L21	Tb927.9.1120_for	GGGGACAAGTTTGTACAAAAAAGCAGGCTATGGGGATTAGTTTTTGTGTGTCACT	Tb927.9.1120_rev	GGGGACCACTTTGTACAAGAAAGCTGGGTTTATATATCCTCTTCTCCAACAATTTCTCT
0_hypothetical	L22	Tb927.8.3440_for	GGGGACAAGTTTGTACAAAAAAGCAGGCTATGAGTGTTGGGAAATTTAAACGGTCA	Tb927.8.3440_rev	GGGGACCACTTTGTACAAGAAAGCTGGGTTTACCAGCGGCACATTTGACG
0_hypothetical	L23	Tb927.6.3200_for	GGGGACAAGTTTGTACAAAAAAGCAGGCTATGCTTCGTAGGAAAGGATACATTGG	Tb927.6.3200_rev	GGGGACCACTTTGTACAAGAAAGCTGGGTTCAGTGTGGAGAGGAATACCGATC
0_hypothetical	L24	Tb927.6.1600_for	GGGGACAAGTTTGTACAAAAAAGCAGGCTATGCTGCGTCGCCGGATA	Tb927.6.1600_rev	GGGGACCACTTTGTACAAGAAAGCTGGGTTCATTTGCGCCAAGGTTCCTT
0_hypothetical	M1	Tb927.11.1330_for	GGGGACAAGTTTGTACAAAAAAGCAGGCTATGTCCGCGAAGATGTGCC	Tb927.11.1330_rev	GGGGACCACTTTGTACAAGAAAGCTGGGTTCACTCCTTCAGCTCAAAGTCCA
0_hypothetical	M2	Tb927.11.15180_for	GGGGACAAGTTTGTACAAAAAAGCAGGCTATGGCGAAGAAACATCGTGTGA	Tb927.11.15180_rev	GGGGACCACTTTGTACAAGAAAGCTGGGTTCAGTACTTCTTCCTCAAATGTTTGACC
0_hypothetical	M3	Tb927.9.10000_for	GGGGACAAGTTTGTACAAAAAAGCAGGCTATGTCGAAGTTAGAGCAGCGC	Tb927.9.10000_rev	GGGGACCACTTTGTACAAGAAAGCTGGGTTTACAGTTCGTGCATCATGTATATCCG
0_hypothetical	M4	Tb927.7.4250_for	GGGGACAAGTTTGTACAAAAAAGCAGGCTATGACTCGGCAATCCGATGTG	Tb927.7.4250_rev	GGGGACCACTTTGTACAAGAAAGCTGGGTTCAGGAGGTTCCACTCGATGC
0_hypothetical	M6	Tb927.8.3600_for	GGGGACAAGTTTGTACAAAAAAGCAGGCTATGCGCGGGAGAGGACGC	Tb927.8.3600_rev	GGGGACCACTTTGTACAAGAAAGCTGGGTTTAGCCGCAGATCGGTGCC
0_hypothetical	M7	Tb927.9.10930_for	GGGGACAAGTTTGTACAAAAAAGCAGGCTATGGAACATATTCCTGCTGAAGCATC	Tb927.9.10930_rev	GGGGACCACTTTGTACAAGAAAGCTGGGTTCACAAACTCTCAGAGAACGACGG
0_hypothetical	M8	Tb927.10.7170_for	GGGGACAAGTTTGTACAAAAAAGCAGGCTATGTTCCCCATTCCGCCAGC	Tb927.10.7170_rev	GGGGACCACTTTGTACAAGAAAGCTGGGTCTAAACGCGCAAACTGACTGTG
0_hypothetical	M9	Tb927.4.440_for	GGGGACAAGTTTGTACAAAAAAGCAGGCTATGACTGGTTGGAACCAAAAAATGGC	Tb927.4.440_rev	GGGGACCACTTTGTACAAGAAAGCTGGGTTTATCGTCTATCCGGCTGAATATCGC
0_hypothetical	M10	Tb927.9.140_for	GGGGACAAGTTTGTACAAAAAAGCAGGCTATGCCGCCAGGGTCCAGT	Tb927.9.140_rev	GGGGACCACTTTGTACAAGAAAGCTGGGTCTAAAACCCCCTGTTTTTTTTTGATGAATC
0_hypothetical	M11	Tb927.11.11135_for	GGGGACAAGTTTGTACAAAAAAGCAGGCTATGGGTGGGCCACCAAGG	Tb927.11.11135_rev	GGGGACCACTTTGTACAAGAAAGCTGGGTTCAATTCATTAGATCCAGATCACCGACA
0_hypothetical	M12	Tb927.10.13770_for	GGGGACAAGTTTGTACAAAAAAGCAGGCTATGGTCCTGCGAGGCGTG	Tb927.10.13770_rev	GGGGACCACTTTGTACAAGAAAGCTGGGTTCACTCCCGAAGCGAGAAGT
0_hypothetical	M13	Tb927.10.10270_for	GGGGACAAGTTTGTACAAAAAAGCAGGCTATGCAACACCCGGGTCGC	Tb927.10.10270_rev	GGGGACCACTTTGTACAAGAAAGCTGGGTCTATGATTGAATCAAATATTGCCATGCCTC
0_hypothetical	M14	Tb927.9.2110_for	GGGGACAAGTTTGTACAAAAAAGCAGGCTATGTCACGCAATACAAAGGAATTTAACC	Tb927.9.2110_rev	GGGGACCACTTTGTACAAGAAAGCTGGGTTCAGTTCGCTTCGGGATTCTTCT
0_hypothetical	M15	Tb927.8.640_for	GGGGACAAGTTTGTACAAAAAAGCAGGCTATGGAGGGGAAGAGTGAAGCC	Tb927.8.640_rev	GGGGACCACTTTGTACAAGAAAGCTGGGTTCACATATCGGATTTCGGTGCCT
0_hypothetical	M16	Tb927.10.13810_for	GGGGACAAGTTTGTACAAAAAAGCAGGCTATGTCTAGTCGCTTTTTCCAAAAGTATTTT	Tb927.10.13810_rev	GGGGACCACTTTGTACAAGAAAGCTGGGTTTAATTCGATGTTCCGGACGACG
0_hypothetical	M17	Tb927.9.670_for	GGGGACAAGTTTGTACAAAAAAGCAGGCTATGTGGTGTGCCGCGCCA	Tb927.9.670_rev	GGGGACCACTTTGTACAAGAAAGCTGGGTTCAGGAATTGTTTCTGTTCCCTCTCA
0_hypothetical	M18	Tb927.8.4910_for	GGGGACAAGTTTGTACAAAAAAGCAGGCTATGCCCGCCAGACCCAAT	Tb927.8.4910_rev	GGGGACCACTTTGTACAAGAAAGCTGGGTCTAACGGAGGGTTAAACGAGCC
0_hypothetical	M19	Tb927.10.1580_for	GGGGACAAGTTTGTACAAAAAAGCAGGCTATGACAGCGCCCGTCTCT	Tb927.10.1580_rev	GGGGACCACTTTGTACAAGAAAGCTGGGTCTAACTCCCGGAAGATTCTCTATCGC
0_hypothetical	M20	Tb927.4.1810_for	GGGGACAAGTTTGTACAAAAAAGCAGGCTATGCTCTCCTCCACCGCC	Tb927.4.1810_rev	GGGGACCACTTTGTACAAGAAAGCTGGGTTTATTTCTTCAACATCTTTTCCGCACC
0_hypothetical	M21	Tb927.9.16410_for	GGGGACAAGTTTGTACAAAAAAGCAGGCTATGTCAGTTTGGAACGACATTCTTTGG	Tb927.9.16410_rev	GGGGACCACTTTGTACAAGAAAGCTGGGTTTACCCCCACATCTCTCTGGTTG
0_hypothetical	M22	Tb927.4.3450_for	GGGGACAAGTTTGTACAAAAAAGCAGGCTATGCTACGGCGACTTGGTGC	Tb927.4.3450_rev	GGGGACCACTTTGTACAAGAAAGCTGGGTTTAAATGTTGAGTGAGCGAGTAATGGC
0_hypothetical	M23	Tb927.9.10430_for	GGGGACAAGTTTGTACAAAAAAGCAGGCTATGCTCACTATCACCGCTCTTCT	Tb927.9.10430_rev	GGGGACCACTTTGTACAAGAAAGCTGGGTTTATTTCCGTCCCCTTTGAACCCT
0_hypothetical	M24	Tb927.8.2530_for	GGGGACAAGTTTGTACAAAAAAGCAGGCTATGGGCATAGAAACGTGGAAGGT	Tb927.8.2530_rev	GGGGACCACTTTGTACAAGAAAGCTGGGTTTACGCCGTGCCTTCCCA
0_hypothetical	N1	Tb927.8.1665_for	GGGGACAAGTTTGTACAAAAAAGCAGGCTATGCTGACTTATTGCCTTTTGGGA	Tb927.8.1665_rev	GGGGACCACTTTGTACAAGAAAGCTGGGTCTATACATCTCGCGTGTTGAAAAAAGC
0_hypothetical	N2	Tb927.1.4970_for	GGGGACAAGTTTGTACAAAAAAGCAGGCTATGGCAGACCGAGCTTTAACG	Tb927.1.4970_rev	GGGGACCACTTTGTACAAGAAAGCTGGGTTTAAACTTGAACGTGTTCCACAGAGC
0_hypothetical	N3	Tb927.9.15080_for	GGGGACAAGTTTGTACAAAAAAGCAGGCTATGAGGCCCCTCCGCGCG	Tb927.9.15080_rev	GGGGACCACTTTGTACAAGAAAGCTGGGTTCATGTTCTTTTTAATCCTTCAATGTCCAC
0_hypothetical	N4	Tb927.10.2970_for	GGGGACAAGTTTGTACAAAAAAGCAGGCTATGCTGCCCCTCCGTGTAC	Tb927.10.2970_rev	GGGGACCACTTTGTACAAGAAAGCTGGGTCTATTGCTTCTCGTTCGTTAACTGCT
0_hypothetical	N5	Tb927.10.16060_for	GGGGACAAGTTTGTACAAAAAAGCAGGCTATGTCCGAGGACACGTTTTTTCG	Tb927.10.16060_rev	GGGGACCACTTTGTACAAGAAAGCTGGGTTCATTTCTGTTTGTCTATATCCGAAGACG
0_hypothetical	N6	Tb927.2.1790_for	GGGGACAAGTTTGTACAAAAAAGCAGGCTATGAAGCACATGCCATTCAGAGAG	Tb927.2.1790_rev	GGGGACCACTTTGTACAAGAAAGCTGGGTTTAGCTCCGCCTGTGAAAACAAC
0_hypothetical	N7	Tb927.5.2200_for	GGGGACAAGTTTGTACAAAAAAGCAGGCTATGGCCACCTGCCCTAAACA	Tb927.5.2200_rev	GGGGACCACTTTGTACAAGAAAGCTGGGTTTACTCCACGGCCTCAAACACC
0_hypothetical	N8	Tb927.9.5800_for	GGGGACAAGTTTGTACAAAAAAGCAGGCTATGTTAGTCCACTTGGATAAATGGTGG	Tb927.9.5800_rev	GGGGACCACTTTGTACAAGAAAGCTGGGTTTAAACAAACCTCCCTTCGCTTCG
0_hypothetical	N9	Tb927.4.390_for	GGGGACAAGTTTGTACAAAAAAGCAGGCTATGCGAGACGAACAAAAGGAATATGC	Tb927.4.390_rev	GGGGACCACTTTGTACAAGAAAGCTGGGTTCAAAGGGCATGGAGAATAGTTTTCTT
0_hypothetical	N10	Tb927.7.4210_for	GGGGACAAGTTTGTACAAAAAAGCAGGCTATGTTAAGAAGCACTGCTGCGT	Tb927.7.4210_rev	GGGGACCACTTTGTACAAGAAAGCTGGGTTCAGACAATACCACCGAGTTTATTCTCC
0_hypothetical	N11	Tb927.3.585_for	GGGGACAAGTTTGTACAAAAAAGCAGGCTATGGCCGCAGCCGTCAAA	Tb927.3.585_rev	GGGGACCACTTTGTACAAGAAAGCTGGGTTTAATCCCGAGCGTCACCGA
0_hypothetical	N12	Tb927.4.1240_for	GGGGACAAGTTTGTACAAAAAAGCAGGCTATGTCCGTACGTTCCGATACTTTTTT	Tb927.4.1240_rev	GGGGACCACTTTGTACAAGAAAGCTGGGTTTAATTGAGCTCATTGACGGGGAATATAAC
0_hypothetical	N13	Tb927.10.11520_for	GGGGACAAGTTTGTACAAAAAAGCAGGCTATGCAACCCCCAACCCCA	Tb927.10.11520_rev	GGGGACCACTTTGTACAAGAAAGCTGGGTCTAGACCCCTCCCCCCTGC
0_hypothetical	N14	Tb927.8.2340_for	GGGGACAAGTTTGTACAAAAAAGCAGGCTATGAACCGGTTCTGCCGGC	Tb927.8.2340_rev	GGGGACCACTTTGTACAAGAAAGCTGGGTTCACTTGCACGGCCACCA
0_hypothetical	N15	Tb927.7.2780_for	GGGGACAAGTTTGTACAAAAAAGCAGGCTATGTCTAAAGCTCCTTCGCAACC	Tb927.7.2780_rev	GGGGACCACTTTGTACAAGAAAGCTGGGTCTATGCCGCCGCTGCCGG
0_hypothetical	N16	Tb927.10.12110_for	GGGGACAAGTTTGTACAAAAAAGCAGGCTATGTCGCAACGGGGTTCCG	Tb927.10.12110_rev	GGGGACCACTTTGTACAAGAAAGCTGGGTTCAAGCAGATGAAGCTGCAGC
0_hypothetical	N17	Tb927.9.13225_for	GGGGACAAGTTTGTACAAAAAAGCAGGCTATGGCAATGAGCGGGGATGG	Tb927.9.13225_rev	GGGGACCACTTTGTACAAGAAAGCTGGGTCTATGCACTCTCTCCGGAGCG
0_hypothetical	N18	Tb927.9.3540_for	GGGGACAAGTTTGTACAAAAAAGCAGGCTATGTCTTCGCTTCCCGGGG	Tb927.9.3540_rev	GGGGACCACTTTGTACAAGAAAGCTGGGTCTAATACCCGTTGTGGCTCCG
0_hypothetical	N19	Tb927.9.3840_for	GGGGACAAGTTTGTACAAAAAAGCAGGCTATGCAACGCCGAGTGCAG	Tb927.9.3840_rev	GGGGACCACTTTGTACAAGAAAGCTGGGTTCATACGCTCTTCTCCACAGCC
0_hypothetical	N20	Tb927.10.13515_for	GGGGACAAGTTTGTACAAAAAAGCAGGCTATGAACCCCCCAGAAGAATCAGAG	Tb927.10.13515_rev	GGGGACCACTTTGTACAAGAAAGCTGGGTCTATTTTGGTTGCGCTGGGGG
0_hypothetical	N21	Tb927.11.16950_for	GGGGACAAGTTTGTACAAAAAAGCAGGCTATGGTGTGGTACATTCCCTTTGTTT	Tb927.11.16950_rev	GGGGACCACTTTGTACAAGAAAGCTGGGTTCAATCATGCCGAAGGGTATAGGC
0_hypothetical	N22	Tb927.7.5010_for	GGGGACAAGTTTGTACAAAAAAGCAGGCTATGTCTCATGTCACAGCTGACCT	Tb927.7.5010_rev	GGGGACCACTTTGTACAAGAAAGCTGGGTTCACTCCGCAAGAAACTTAGCG
0_hypothetical	N23	Tb927.11.9425_for	GGGGACAAGTTTGTACAAAAAAGCAGGCTATGGACATTCTGCAGAACATTGCA	Tb927.11.9425_rev	GGGGACCACTTTGTACAAGAAAGCTGGGTTTAGTTCCACATTTGTTCTGCAACCC
0_hypothetical	N24	Tb927.11.16710_for	GGGGACAAGTTTGTACAAAAAAGCAGGCTATGGGGGTTCGCATGTTCTGC	Tb927.11.16710_rev	GGGGACCACTTTGTACAAGAAAGCTGGGTCTACAGTGGGTTGTTGGATCCAC
0_hypothetical	O1	Tb927.10.11200_for	GGGGACAAGTTTGTACAAAAAAGCAGGCTATGGGCACGATTTTTAGCAACGT	Tb927.10.11200_rev	GGGGACCACTTTGTACAAGAAAGCTGGGTCTAATTGGCAACATGGACCTCTGC
0_hypothetical	O2	Tb927.11.10220_for	GGGGACAAGTTTGTACAAAAAAGCAGGCTATGCCCCGTGGCGGCACT	Tb927.11.10220_rev	GGGGACCACTTTGTACAAGAAAGCTGGGTCTATACCCCCAGATAGCCCCG
0_hypothetical	O3	Tb927.10.500_for	GGGGACAAGTTTGTACAAAAAAGCAGGCTATGCATTTATGTTTTTCCTTTCTTTCTTTC	Tb927.10.500_rev	GGGGACCACTTTGTACAAGAAAGCTGGGTTTACACATTGCGTATATATAATAGAAAGAA
0_hypothetical	O4	Tb927.11.9665_for	GGGGACAAGTTTGTACAAAAAAGCAGGCTATGGAACCTCGCGAGATGAGT	Tb927.11.9665_rev	GGGGACCACTTTGTACAAGAAAGCTGGGTCTAAAAAGTCCTCCCTGCGCC
0_hypothetical	O5	Tb927.11.5600_for	GGGGACAAGTTTGTACAAAAAAGCAGGCTATGACTGAAGCACTCTACAACTTCATC	Tb927.11.5600_rev	GGGGACCACTTTGTACAAGAAAGCTGGGTTTAAATGCCATGACGTTCACGATACT
0_hypothetical	O6	Tb927.11.160_for	GGGGACAAGTTTGTACAAAAAAGCAGGCTATGAGATCCACCGTCTTTGGTGG	Tb927.11.160_rev	GGGGACCACTTTGTACAAGAAAGCTGGGTCTATCGCAGTAGAACGCACCAAA
0_hypothetical	O7	Tb927.2.160_for	GGGGACAAGTTTGTACAAAAAAGCAGGCTATGTTTCAATTGGTTTCTACGGCCG	Tb927.2.160_rev	GGGGACCACTTTGTACAAGAAAGCTGGGTCTACATTTCCTTAAACCGGAAGCTAACC

0_hypothetical	O8	Tb927.10.4240_for	GGGGACAAGTTTGTACAAAAAAGCAGGCTATGATGAGAAACTCCCGGTTTCTGT	Tb927.10.4240_rev	GGGGACCACTTTGTACAAGAAAGCTGGGTTTACCACCAAGCTGCGTGC
0_hypothetical	O9	Tb927.11.9850_for	GGGGACAAGTTTGTACAAAAAAGCAGGCTATGTCACTTGTGTTGGCAGATGAA	Tb927.11.9850_rev	GGGGACCACTTTGTACAAGAAAGCTGGGTTTAACCCTCTGGCAGAAGCGC
0_hypothetical	O10	Tb927.1.400_for	GGGGACAAGTTTGTACAAAAAAGCAGGCTATGCCTCCCCTTGCTCGC	Tb927.1.400_rev	GGGGACCACTTTGTACAAGAAAGCTGGGTCTACAGGATGGTGGAAACGAAGC
0_hypothetical	O11	Tb927.8.3120_for	GGGGACAAGTTTGTACAAAAAAGCAGGCTATGTACTTTCTTTTGCTAGCTGGGATC	Tb927.8.3120_rev	GGGGACCACTTTGTACAAGAAAGCTGGGTTCATGATGATGATGCGATCCCACA
0_hypothetical	O12	Tb927.10.14370_for	GGGGACAAGTTTGTACAAAAAAGCAGGCTATGAGCGAGGAGGTGCAGC	Tb927.10.14370_rev	GGGGACCACTTTGTACAAGAAAGCTGGGTCTAAGATTCGTGCGAAGTCTTAGCC
0_hypothetical	O13	Tb927.11.16520_for	GGGGACAAGTTTGTACAAAAAAGCAGGCTATGAATTATTCAACCGGGTACTCTGC	Tb927.11.16520_rev	GGGGACCACTTTGTACAAGAAAGCTGGGTTTAAGTGCTACTTTCCCCTGTCCA
0_hypothetical	O14	Tb927.7.2690_for	GGGGACAAGTTTGTACAAAAAAGCAGGCTATGCACCGAAGGACATTCTTTCTT	Tb927.7.2690_rev	GGGGACCACTTTGTACAAGAAAGCTGGGTTCACCGTACCAGCGTATTGTGA
0_hypothetical	O15	Tb927.1.280_for	GGGGACAAGTTTGTACAAAAAAGCAGGCTATGTTTCAATTGGTTTCTGCGGC	Tb927.1.280_rev	GGGGACCACTTTGTACAAGAAAGCTGGGTCTACACTTCCTTAAACCGGAAGCTAAC
0_hypothetical	O16	Tb927.8.6310_for	GGGGACAAGTTTGTACAAAAAAGCAGGCTATGGCGGCACCACTAGCA	Tb927.8.6310_rev	GGGGACCACTTTGTACAAGAAAGCTGGGTTTACTTCTTCCCCAGTATGTCCCG
0_hypothetical	O17	Tb927.1.470_for	GGGGACAAGTTTGTACAAAAAAGCAGGCTATGTTTCAATTGGTTTCTGTGGCCG	Tb927.1.470_rev	GGGGACCACTTTGTACAAGAAAGCTGGGTCTACACTTCCTTAAACCGGAAGCTAAC
0_hypothetical	O18	Tb927.1.3810_for	GGGGACAAGTTTGTACAAAAAAGCAGGCTATGGCTCGTACTTTCGTCGTTTT	Tb927.1.3810_rev	GGGGACCACTTTGTACAAGAAAGCTGGGTCTAACCTGCGTAAACAGATGGTGC
0_hypothetical	O19	Tb927.10.2670_for	GGGGACAAGTTTGTACAAAAAAGCAGGCTATGGAGCAAAAGGAAAATGAGATCACT	Tb927.10.2670_rev	GGGGACCACTTTGTACAAGAAAGCTGGGTCTAGGCCGGAAGTACAATGGTTAATC
0_hypothetical	O20	Tb927.2.5720_for	GGGGACAAGTTTGTACAAAAAAGCAGGCTATGTTGCTGTCGCTGGCG	Tb927.2.5720_rev	GGGGACCACTTTGTACAAGAAAGCTGGGTTCATTCCCCCTGAAAGAAGCGG
0_hypothetical	O21	Tb927.9.15750_for	GGGGACAAGTTTGTACAAAAAAGCAGGCTATGAGCAACCTGACAGTGAGCA	Tb927.9.15750_rev	GGGGACCACTTTGTACAAGAAAGCTGGGTTCACAATCTCACCGTTGACCAGC
0_hypothetical	O22	Tb927.6.570_for	GGGGACAAGTTTGTACAAAAAAGCAGGCTATGGAACTGAACGAGCTGCAAC	Tb927.6.570_rev	GGGGACCACTTTGTACAAGAAAGCTGGGTTCATGTCCAAAGACACCTTTCATTTACT
0_hypothetical	O23	Tb927.4.2960_for	GGGGACAAGTTTGTACAAAAAAGCAGGCTATGAATGCGCGCGATGAACT	Tb927.4.2960_rev	GGGGACCACTTTGTACAAGAAAGCTGGGTCTAATGTCTGGCATACCGTTCTTTCA
0_hypothetical	P1	Tb927.8.2150_for	GGGGACAAGTTTGTACAAAAAAGCAGGCTATGACATGTAATGTGACGCCAGAG	Tb927.8.2150_rev	GGGGACCACTTTGTACAAGAAAGCTGGGTCTATGTTTCCACCTCGACGTCCT
0_hypothetical	P3	Tb927.8.7790_for	GGGGACAAGTTTGTACAAAAAAGCAGGCTATGCCTCCTGCCCCGCGC	Tb927.8.7790_rev	GGGGACCACTTTGTACAAGAAAGCTGGGTTTACAGATCATCATCATCGTCGAGTTTC
0_hypothetical	P4	Tb927.11.2930_for	GGGGACAAGTTTGTACAAAAAAGCAGGCTATGCTCCGCCGTTGCGTT	Tb927.11.2930_rev	GGGGACCACTTTGTACAAGAAAGCTGGGTCTAAGCGAGGTCGGTATCCGC
0_hypothetical	P5	Tb927.4.4630_for	GGGGACAAGTTTGTACAAAAAAGCAGGCTATGAGCGCTGATCCTGAAGTACC	Tb927.4.4630_rev	GGGGACCACTTTGTACAAGAAAGCTGGGTTTAAAAGAACTTGTACCATGCTGAGGC
0_hypothetical	P6	Tb927.4.4540_for	GGGGACAAGTTTGTACAAAAAAGCAGGCTATGCCGTCCATAACGCGC	Tb927.4.4540_rev	GGGGACCACTTTGTACAAGAAAGCTGGGTCTAGGGGTCTTCATCATCATCGAGT
0_hypothetical	P7	Tb927.11.10040_for	GGGGACAAGTTTGTACAAAAAAGCAGGCTATGATGCGCCGAACGCCG	Tb927.11.10040_rev	GGGGACCACTTTGTACAAGAAAGCTGGGTTTAACTATCTCCTTCGCCAGGGAG
0_hypothetical	P8	Tb927.2.1720_for	GGGGACAAGTTTGTACAAAAAAGCAGGCTATGGACAGCAACGTCACTTACG	Tb927.2.1720_rev	GGGGACCACTTTGTACAAGAAAGCTGGGTCTAATTCACTGGGGTCAGTTCAAAGC
0_hypothetical	P9	Tb927.7.2380_for	GGGGACAAGTTTGTACAAAAAAGCAGGCTATGGACAAGGACCTCGCGG	Tb927.7.2380_rev	GGGGACCACTTTGTACAAGAAAGCTGGGTTTACTCATCTGGAGAACCCTCCG
0_hypothetical	P10	Tb927.11.11840_for	GGGGACAAGTTTGTACAAAAAAGCAGGCTATGAACGAGCCTGATGCCATG	Tb927.11.11840_rev	GGGGACCACTTTGTACAAGAAAGCTGGGTTCACACCCATGCAGCTGTTG
0_hypothetical	P11	Tb927.10.13920_for	GGGGACAAGTTTGTACAAAAAAGCAGGCTATGTGGTGCTTTTCGTCGCC	Tb927.10.13920_rev	GGGGACCACTTTGTACAAGAAAGCTGGGTTCATCCTTTTCTCATTGCATGATTGCC
0_hypothetical	P12	Tb927.11.410_for	GGGGACAAGTTTGTACAAAAAAGCAGGCTATGTCGCGCAGTACAGTTTTTGG	Tb927.11.410_rev	GGGGACCACTTTGTACAAGAAAGCTGGGTTCACCGCTCGTAGCGCCA
0_hypothetical	P13	Tb927.4.120_for	GGGGACAAGTTTGTACAAAAAAGCAGGCTATGTTGTGGTGGAAACAGTGTGC	Tb927.4.120_rev	GGGGACCACTTTGTACAAGAAAGCTGGGTCTAAGTGGAACGCCAGGACG
0_hypothetical	P14	Tb927.5.1800_for	GGGGACAAGTTTGTACAAAAAAGCAGGCTATGGTGAACCTGCATGAGCTG	Tb927.5.1800_rev	GGGGACCACTTTGTACAAGAAAGCTGGGTTCAATCGAGTTTTGCTCCCCATTG
0_hypothetical	P15	Tb927.8.1120_for	GGGGACAAGTTTGTACAAAAAAGCAGGCTATGAGCGGGGACGGCGTT	Tb927.8.1120_rev	GGGGACCACTTTGTACAAGAAAGCTGGGTCTAAACTTTCCCCAGAAACTTCCTGT
0_hypothetical	P16	Tb927.2.900_for	GGGGACAAGTTTGTACAAAAAAGCAGGCTATGCTGTGGCGGAAGCAG	Tb927.2.900_rev	GGGGACCACTTTGTACAAGAAAGCTGGGTCTAAGTGGAACGCCAGGACG
0_hypothetical	P17	Tb927.2.1250_for	GGGGACAAGTTTGTACAAAAAAGCAGGCTATGCTGTGGCGGAAGCAG	Tb927.2.1250_rev	GGGGACCACTTTGTACAAGAAAGCTGGGTCTAAGTGGAACATCGGGACGTG
0_hypothetical	P18	Tb927.10.490_for	GGGGACAAGTTTGTACAAAAAAGCAGGCTATGAAACAAATGTTTTATTGCAGCAAAGG	Tb927.10.490_rev	GGGGACCACTTTGTACAAGAAAGCTGGGTTCATTCACTCACATGGAAACAAAAGTACA
0_hypothetical	P19	Tb927.8.4690_for	GGGGACAAGTTTGTACAAAAAAGCAGGCTATGCTCGATAAAATCAAGACTATTGTCACT	Tb927.8.4690_rev	GGGGACCACTTTGTACAAGAAAGCTGGGTTCATTTAAACCGCGACGAAACGC
0_hypothetical	P20	Tb927.5.4370_for	GGGGACAAGTTTGTACAAAAAAGCAGGCTATGTCGCCTCTCGTATCGTCC	Tb927.5.4370_rev	GGGGACCACTTTGTACAAGAAAGCTGGGTTTAACATGGCTTCATCGGGTTGC
0_hypothetical	P21	Tb927.7.2210_for	GGGGACAAGTTTGTACAAAAAAGCAGGCTATGCGTCGCTCAGTGTTTTTCA	Tb927.7.2210_rev	GGGGACCACTTTGTACAAGAAAGCTGGGTTCACGCACAGGTTACTCCGT
0_hypothetical	P22	Tb927.11.800_for	GGGGACAAGTTTGTACAAAAAAGCAGGCTATGTCGCTGCTGAAGGGAAAG	Tb927.11.800_rev	GGGGACCACTTTGTACAAGAAAGCTGGGTCTATTCGTCTTCAAGATACACTTGACTACC
0_hypothetical	P23	Tb927.2.5260_for	GGGGACAAGTTTGTACAAAAAAGCAGGCTATGTCTGGTGGTGTTAATCCCACT	Tb927.2.5260_rev	GGGGACCACTTTGTACAAGAAAGCTGGGTTTACTCACGCCGACTCTTCTTTCG
0_hypothetical	P24	Tb927.3.945_for	GGGGACAAGTTTGTACAAAAAAGCAGGCTATGATGAGGCCATCGACGGG	Tb927.3.945_rev	GGGGACCACTTTGTACAAGAAAGCTGGGTCTAGAGGAATTTGGGTGGCTTCTG
1_hypothetical	A1	Tb927.10.120_for	GGGGACAAGTTTGTACAAAAAAGCAGGCTATGTTTTCCAAACGCTGTTTTGCA	Tb927.10.120_rev	GGGGACCACTTTGTACAAGAAAGCTGGGTTCATCGATATCCTGTATTTCTCCAAGAATC
1_hypothetical	A2	Tb927.8.1750_for	GGGGACAAGTTTGTACAAAAAAGCAGGCTATGTCATCCCCCGCTCCATC	Tb927.8.1750_rev	GGGGACCACTTTGTACAAGAAAGCTGGGTTTAGATATCATCAGTAAGATCCAACTTCAC
1_hypothetical	A3	Tb927.10.14650_for	GGGGACAAGTTTGTACAAAAAAGCAGGCTATGACTGGACTTACGGTGCCG	Tb927.10.14650_rev	GGGGACCACTTTGTACAAGAAAGCTGGGTTCATTCACCAAGACGAATATTCAATAACGG
1_hypothetical	A4	Tb927.10.15140_for	GGGGACAAGTTTGTACAAAAAAGCAGGCTATGTCATTGAGGGAGATTGAACTTGC	Tb927.10.15140_rev	GGGGACCACTTTGTACAAGAAAGCTGGGTCTACATCCCCATGTTCTTCATCGTC
1_hypothetical	A6	Tb927.8.5870_for	GGGGACAAGTTTGTACAAAAAAGCAGGCTATGTCACCATGGAAAAAGGAGGAGG	Tb927.8.5870_rev	GGGGACCACTTTGTACAAGAAAGCTGGGTCTACACTCCACGCAGGTACGA
1_hypothetical	A7	Tb927.7.840_for	GGGGACAAGTTTGTACAAAAAAGCAGGCTATGCTTCGCCGTAGCAGTG	Tb927.7.840_rev	GGGGACCACTTTGTACAAGAAAGCTGGGTTCACAACGGAGCGGGCGT
1_hypothetical	A8	Tb927.2.3315_for	GGGGACAAGTTTGTACAAAAAAGCAGGCTATGGCTAGCATGCTTCATGAAACT	Tb927.2.3315_rev	GGGGACCACTTTGTACAAGAAAGCTGGGTCTAGTAACTCTTACCCTTTCCGATTTCC
1_hypothetical	A9	Tb927.10.6430_for	GGGGACAAGTTTGTACAAAAAAGCAGGCTATGTTTCTAACGAGGCAGCACC	Tb927.10.6430_rev	GGGGACCACTTTGTACAAGAAAGCTGGGTCTAAAACCTGTCACCGGAGATGG
1_hypothetical	A10	Tb927.2.3275_for	GGGGACAAGTTTGTACAAAAAAGCAGGCTATGGCTGGCGTGCTTCATG	Tb927.2.3275_rev	GGGGACCACTTTGTACAAGAAAGCTGGGTCTAGTAACTCTTACCCTTTCCGATTTCC
1_hypothetical	A11	Tb927.8.5070_for	GGGGACAAGTTTGTACAAAAAAGCAGGCTATGGATAAGAGCAGCAAAAACTCCA	Tb927.8.5070_rev	GGGGACCACTTTGTACAAGAAAGCTGGGTTCAAGCCCCGACCTGCGC
1_hypothetical	A15	Tb927.10.16080_for	GGGGACAAGTTTGTACAAAAAAGCAGGCTATGTTCAGTCGCCGCGTG	Tb927.10.16080_rev	GGGGACCACTTTGTACAAGAAAGCTGGGTCTACATTTCGCTCGTCCGTCC
1_hypothetical	A16	Tb927.10.1670_for	GGGGACAAGTTTGTACAAAAAAGCAGGCTATGGGAAGTGGGGGTTCCC	Tb927.10.1670_rev	GGGGACCACTTTGTACAAGAAAGCTGGGTCTAGAAAATTGGGTCCCAGCGG
1_hypothetical	A17	Tb927.2.2770_for	GGGGACAAGTTTGTACAAAAAAGCAGGCTATGCCCGTTATTGTTGTAAACCTCG	Tb927.2.2770_rev	GGGGACCACTTTGTACAAGAAAGCTGGGTTTAAGCCCTACGTGAAAAGAAGAAGC
1_hypothetical	A19	Tb927.9.3410_for	GGGGACAAGTTTGTACAAAAAAGCAGGCTATGTTGCGCATCCGCCGA	Tb927.9.3410_rev	GGGGACCACTTTGTACAAGAAAGCTGGGTCTATCCATTCAGCCAACGCTTCA
1_hypothetical	A20	Tb927.10.750_for	GGGGACAAGTTTGTACAAAAAAGCAGGCTATGTTAAATAGCTGTTTGTGCAAAATCTTC	Tb927.10.750_rev	GGGGACCACTTTGTACAAGAAAGCTGGGTTCATGCACAACTCACTGAGCTG
1_hypothetical	A21	Tb927.3.2420_for	GGGGACAAGTTTGTACAAAAAAGCAGGCTATGGCTGGTGCAAAGGAAGTG	Tb927.3.2420_rev	GGGGACCACTTTGTACAAGAAAGCTGGGTTCAATTTCGCTTTTTCTCTCCTTTTGAC
1_hypothetical	A22	Tb927.9.17640_for	GGGGACAAGTTTGTACAAAAAAGCAGGCTATGCCACCATTGCAGTCCTG	Tb927.9.17640_rev	GGGGACCACTTTGTACAAGAAAGCTGGGTTCAGCCACATTTTGAGAAATCATTAAATCC
1_hypothetical	A23	Tb927.9.4550_for	GGGGACAAGTTTGTACAAAAAAGCAGGCTATGTTTGTAATGAAGTTACTGGATGCACT	Tb927.9.4550_rev	GGGGACCACTTTGTACAAGAAAGCTGGGTTCAAGAGTTTACCACATTGACCGATTC
1_hypothetical	A24	Tb927.9.15690_for	GGGGACAAGTTTGTACAAAAAAGCAGGCTATGCGTTCTACCGTCATTTGGAAT	Tb927.9.15690_rev	GGGGACCACTTTGTACAAGAAAGCTGGGTTCACCGTGTGTAACAAGTGAAGTTC
1_hypothetical	B1	Tb927.3.2475_for	GGGGACAAGTTTGTACAAAAAAGCAGGCTATGCCACCAAAGAAGAGCAGC	Tb927.3.2475_rev	GGGGACCACTTTGTACAAGAAAGCTGGGTCTATCGCTTGGAAGGCGCC
1_hypothetical	B2	Tb927.9.7270_for	GGGGACAAGTTTGTACAAAAAAGCAGGCTATGAGTAATAAAACATATTTGTGGTCCAAA	Tb927.9.7270_rev	GGGGACCACTTTGTACAAGAAAGCTGGGTTCACCTTCTCCGTGTGTACCG
1_hypothetical	B3	Tb927.11.15060.2_for	GGGGACAAGTTTGTACAAAAAAGCAGGCTATGGATTTAGCTGGTGACTTAGTGAAG	Tb927.11.15060.2_rev	GGGGACCACTTTGTACAAGAAAGCTGGGTTCATCGACCACGCTTCACCT
1_hypothetical	B4	Tb927.9.7080_for	GGGGACAAGTTTGTACAAAAAAGCAGGCTATGCCGGATTACACGCAGTTC	Tb927.9.7080_rev	GGGGACCACTTTGTACAAGAAAGCTGGGTTCACACGGGCCGACCGCG
1_hypothetical	B5	Tb927.9.11940_for	GGGGACAAGTTTGTACAAAAAAGCAGGCTATGCGTTCAGCTGTTAATCTCGG	Tb927.9.11940_rev	GGGGACCACTTTGTACAAGAAAGCTGGGTCTAAAAGAGTGCTGGAAACTTTGGGC
1_hypothetical	B6	Tb927.6.4060_for	GGGGACAAGTTTGTACAAAAAAGCAGGCTATGAGCGGTATCCGGTACCG	Tb927.6.4060_rev	GGGGACCACTTTGTACAAGAAAGCTGGGTCTAGTTGGGCATCAACTGCTCATAG
1_hypothetical	B7	Tb927.10.1640_for	GGGGACAAGTTTGTACAAAAAAGCAGGCTATGCACTTCTTTTCAAGTTACCGCT	Tb927.10.1640_rev	GGGGACCACTTTGTACAAGAAAGCTGGGTTTACCCTTCAAAGCACTCAGAAACG
1_hypothetical	B8	Tb927.5.510_for	GGGGACAAGTTTGTACAAAAAAGCAGGCTATGCGGCGCTCAATCATTCG	Tb927.5.510_rev	GGGGACCACTTTGTACAAGAAAGCTGGGTTCACTTCAATGCTTCAGGCCTTG
1_hypothetical	B9	Tb927.3.1525_for	GGGGACAAGTTTGTACAAAAAAGCAGGCTATGAGTTATATAATTGGCCATGTAATGAAA	Tb927.3.1525_rev	GGGGACCACTTTGTACAAGAAAGCTGGGTTTATTGCGATTCCCGTTCCTCCG
1_hypothetical	B10	Tb927.5.600_for	GGGGACAAGTTTGTACAAAAAAGCAGGCTATGGGCCGTGGATTCGGG	Tb927.5.600_rev	GGGGACCACTTTGTACAAGAAAGCTGGGTCTATGAACTCGCATGTCCCCG
1_hypothetical	B11	Tb927.10.15920_for	GGGGACAAGTTTGTACAAAAAAGCAGGCTATGTTTGGACGACGCGTTTACG	Tb927.10.15920_rev	GGGGACCACTTTGTACAAGAAAGCTGGGTTTATTCTCGGGTAGTTTGAAGGCG
1_hypothetical	B12	Tb927.8.1730_for	GGGGACAAGTTTGTACAAAAAAGCAGGCTATGGAACCGGCAGAGCATCG	Tb927.8.1730_rev	GGGGACCACTTTGTACAAGAAAGCTGGGTTCACGAGTCGCTGACATCCG
1_hypothetical	B13	Tb927.5.2670_for	GGGGACAAGTTTGTACAAAAAAGCAGGCTATGCCTCTATGGTCCTTTGTGGA	Tb927.5.2670_rev	GGGGACCACTTTGTACAAGAAAGCTGGGTTTACCCACACTTCAACCTTCCCT
1_hypothetical	B14	Tb927.7.4820_for	GGGGACAAGTTTGTACAAAAAAGCAGGCTATGAGGCTGCTCACTCATAATTTTCTATG	Tb927.7.4820_rev	GGGGACCACTTTGTACAAGAAAGCTGGGTCTAGTGTTCAGCAGCAATCATATCAGG
1_hypothetical	B15	Tb927.8.3730_for	GGGGACAAGTTTGTACAAAAAAGCAGGCTATGAGTGACTTGCAACCTATGAGC	Tb927.8.3730_rev	GGGGACCACTTTGTACAAGAAAGCTGGGTTTAACTCTTATTAGCTATTACCTGTCCAAC
1_hypothetical	B16	Tb927.7.5615_for	GGGGACAAGTTTGTACAAAAAAGCAGGCTATGTTTCTACATAATCGCGGCCTT	Tb927.7.5615_rev	GGGGACCACTTTGTACAAGAAAGCTGGGTTCACATGACCGCTAAAAGAATCATGA
1_hypothetical	B17	Tb927.11.8740_for	GGGGACAAGTTTGTACAAAAAAGCAGGCTATGGGAAGGATAGGGGACACTCC	Tb927.11.8740_rev	GGGGACCACTTTGTACAAGAAAGCTGGGTTTAGAAACCTGCAATTGCAACAGACA
1_hypothetical	B18	Tb927.4.5080_for	GGGGACAAGTTTGTACAAAAAAGCAGGCTATGGTCAGCCTTTTGGCATTGG	Tb927.4.5080_rev	GGGGACCACTTTGTACAAGAAAGCTGGGTCTACCTTTCTTTCTTCTCAAGGGCG
1_hypothetical	B19	Tb927.6.2220_for	GGGGACAAGTTTGTACAAAAAAGCAGGCTATGAGCGTACCTGAGTGGGC	Tb927.6.2220_rev	GGGGACCACTTTGTACAAGAAAGCTGGGTTCAGTTGACCGAAGTACATTGGGA
1_hypothetical	B20	Tb927.10.11770_for	GGGGACAAGTTTGTACAAAAAAGCAGGCTATGCTTTCAAGACAATTAGTAACGAGGT	Tb927.10.11770_rev	GGGGACCACTTTGTACAAGAAAGCTGGGTTCACACCTCCACAAAAAGCACC
1_hypothetical	B21	Tb927.10.8220_for	GGGGACAAGTTTGTACAAAAAAGCAGGCTATGCTTTCAAAACCAGTTGCACC	Tb927.10.8220_rev	GGGGACCACTTTGTACAAGAAAGCTGGGTTCATAAGTGCCCCAAGAGAACCA
1_hypothetical	B22	Tb927.11.7500_for	GGGGACAAGTTTGTACAAAAAAGCAGGCTATGGGCAAAGTAAGAGATACTCCGA	Tb927.11.7500_rev	GGGGACCACTTTGTACAAGAAAGCTGGGTTTAGAAACCTGCAATTGCAACAGACA
1_hypothetical	B23	Tb927.11.15680_for	GGGGACAAGTTTGTACAAAAAAGCAGGCTATGAGTGAGGTAGCCGATTTCAGT	Tb927.11.15680_rev	GGGGACCACTTTGTACAAGAAAGCTGGGTTCAGTTGGAAAGGCGTACGGT
1_hypothetical	B24	Tb927.3.1945_for	GGGGACAAGTTTGTACAAAAAAGCAGGCTATGGCTGCGTTGTTACCATTAGTT	Tb927.3.1945_rev	GGGGACCACTTTGTACAAGAAAGCTGGGTCTACAAATCATAATTACCTTCTTCATCTTG
1_hypothetical	C2	Tb927.7.1160_for	GGGGACAAGTTTGTACAAAAAAGCAGGCTATGCTGTGCCGTAGCCCG	Tb927.7.1160_rev	GGGGACCACTTTGTACAAGAAAGCTGGGTTCATAAGTCCTCCCCGTTCCATT
1_hypothetical	C3	Tb927.7.6420_for	GGGGACAAGTTTGTACAAAAAAGCAGGCTATGAGGTACATGGTTTTTGCTGCC	Tb927.7.6420_rev	GGGGACCACTTTGTACAAGAAAGCTGGGTCTAGGGGCAAGGGGAAAGGT
1_hypothetical	C4	Tb927.10.3160_for	GGGGACAAGTTTGTACAAAAAAGCAGGCTATGCGTTACTATCCCACTGGTCT	Tb927.10.3160_rev	GGGGACCACTTTGTACAAGAAAGCTGGGTCTACATCTCTATGCTCAACCCCCG
1_hypothetical	C5	Tb927.11.14165_for	GGGGACAAGTTTGTACAAAAAAGCAGGCTATGTCCATAAGTCAAGTGGATCTCGA	Tb927.11.14165_rev	GGGGACCACTTTGTACAAGAAAGCTGGGTTCAAAGGTTTGACGGATCATCTGAATG
1_hypothetical	C6	Tb927.11.13570_for	GGGGACAAGTTTGTACAAAAAAGCAGGCTATGAGTGAAGGGCGCTTGGA	Tb927.11.13570_rev	GGGGACCACTTTGTACAAGAAAGCTGGGTTTAAACGTGCTCGGTGTCCATG
1_hypothetical	C7	Tb927.10.14050_for	GGGGACAAGTTTGTACAAAAAAGCAGGCTATGCTGTGGGGTTCTAGAAAGGG	Tb927.10.14050_rev	GGGGACCACTTTGTACAAGAAAGCTGGGTCTACAAACAAGCTGACAGACAAAAAAATGG
1_hypothetical	C8	Tb11.02.5105_for	GGGGACAAGTTTGTACAAAAAAGCAGGCTATGGACGTGGTTGAAAAATTTCTTTCTC	Tb11.02.5105_rev	GGGGACCACTTTGTACAAGAAAGCTGGGTTCATGAAACCCCAAGCACGTG
1_hypothetical	C9	Tb927.11.11078_for	GGGGACAAGTTTGTACAAAAAAGCAGGCTATGCCTCACCGCTCCACT	Tb927.11.11078_rev	GGGGACCACTTTGTACAAGAAAGCTGGGTCTACCGTCCCTCTGATTGTTCTTCA
1_hypothetical	C10	Tb927.9.8580_for	GGGGACAAGTTTGTACAAAAAAGCAGGCTATGGTGTCATGGTTTTGGACCAC	Tb927.9.8580_rev	GGGGACCACTTTGTACAAGAAAGCTGGGTTTACTCACTAACGCCCTCAAACAGC
1_hypothetical	C11	Tb927.6.2590_for	GGGGACAAGTTTGTACAAAAAAGCAGGCTATGATTCGCCGATTAGGCAGTG	Tb927.6.2590_rev	GGGGACCACTTTGTACAAGAAAGCTGGGTCTAAAAAGGCCCAAAGCGCTTATTT
1_hypothetical	C12	Tb927.9.1640_for	GGGGACAAGTTTGTACAAAAAAGCAGGCTATGTGGCGTACTGCTGTCTTTTG	Tb927.9.1640_rev	GGGGACCACTTTGTACAAGAAAGCTGGGTCTACATGTCATCCATTCGTTCCTCTTC
1_hypothetical	C13	Tb927.11.15375_for	GGGGACAAGTTTGTACAAAAAAGCAGGCTATGGAGGTATCGGCGGATACAC	Tb927.11.15375_rev	GGGGACCACTTTGTACAAGAAAGCTGGGTTCACGATTCTTTCGTTCTTGTTCCT
1_hypothetical	C14	Tb927.10.320_for	GGGGACAAGTTTGTACAAAAAAGCAGGCTATGGACCCTGCCGATCCC	Tb927.10.320_rev	GGGGACCACTTTGTACAAGAAAGCTGGGTCTATGCAACCGCAGGTCTCTG
1_hypothetical	C15	Tb927.3.3140_for	GGGGACAAGTTTGTACAAAAAAGCAGGCTATGTGCCACGGGGGTCGC	Tb927.3.3140_rev	GGGGACCACTTTGTACAAGAAAGCTGGGTTCACCTCTCAACTCTGCGGTT
1_hypothetical	C16	Tb927.9.10650_for	GGGGACAAGTTTGTACAAAAAAGCAGGCTATGGAACAACAACAACAACAACCAG	Tb927.9.10650_rev	GGGGACCACTTTGTACAAGAAAGCTGGGTTCACATCCTGTAATCTCTTCTGTTTTCG
1_hypothetical	C17	Tb927.8.5900_for	GGGGACAAGTTTGTACAAAAAAGCAGGCTATGTTGATGGTTGTCGGACCTGA	Tb927.8.5900_rev	GGGGACCACTTTGTACAAGAAAGCTGGGTCTAACAGGGGGAGAAGCGTTGG
1_hypothetical	C18	Tb927.4.2170_for	GGGGACAAGTTTGTACAAAAAAGCAGGCTATGTCTCCCAAACTGTATGTTTGCA	Tb927.4.2170_rev	GGGGACCACTTTGTACAAGAAAGCTGGGTTCAATTGCTACTGTCTGCTCCATAAC
1_hypothetical	C19	Tb927.11.15360_for	GGGGACAAGTTTGTACAAAAAAGCAGGCTATGGGGGGTAGTTCGAGTCG	Tb927.11.15360_rev	GGGGACCACTTTGTACAAGAAAGCTGGGTTTATTCGGCCACCTCGTCTTCC
1_hypothetical	C20	Tb927.10.7720_for	GGGGACAAGTTTGTACAAAAAAGCAGGCTATGTGGCTCCGCTCCAGAA	Tb927.10.7720_rev	GGGGACCACTTTGTACAAGAAAGCTGGGTTTACTTCCCCCCTAGAACAGCAC
1_hypothetical	C21	Tb927.11.9580.1_for	GGGGACAAGTTTGTACAAAAAAGCAGGCTATGGGCGAGGAGGTACTGTC	Tb927.11.9580.1_rev	GGGGACCACTTTGTACAAGAAAGCTGGGTCTACCGCTGGACAATTTCCGC
1_hypothetical	C22	Tb927.8.3360_for	GGGGACAAGTTTGTACAAAAAAGCAGGCTATGCTGTGGGGTGCGGTT	Tb927.8.3360_rev	GGGGACCACTTTGTACAAGAAAGCTGGGTCTACATTACCAACCGCCGTCG
1_hypothetical	C23	Tb927.4.4120_for	GGGGACAAGTTTGTACAAAAAAGCAGGCTATGTACTTCGAAGAGGAAGAGAATAATGAT	Tb927.4.4120_rev	GGGGACCACTTTGTACAAGAAAGCTGGGTCTATACCCAACTGCTATACCCCGT
1_hypothetical	C24	Tb927.9.12280_for	GGGGACAAGTTTGTACAAAAAAGCAGGCTATGGGTAGAAATGTAACTGCGGTTAC	Tb927.9.12280_rev	GGGGACCACTTTGTACAAGAAAGCTGGGTTTATTTTTTGCTCCTTGATTTTTTCCTGCC
1_hypothetical	D2	Tb927.11.6700_for	GGGGACAAGTTTGTACAAAAAAGCAGGCTATGGTCAGTCGTTCGGGGT	Tb927.11.6700_rev	GGGGACCACTTTGTACAAGAAAGCTGGGTTCAGTCAACCCATCGCCGC
1_hypothetical	D3	Tb927.11.8420_for	GGGGACAAGTTTGTACAAAAAAGCAGGCTATGGCTGGCATAAACACGGTTG	Tb927.11.8420_rev	GGGGACCACTTTGTACAAGAAAGCTGGGTTTAGGGTGTGTATTCTTCGTCATCTTCA
1_hypothetical	D4	Tb927.11.8750_for	GGGGACAAGTTTGTACAAAAAAGCAGGCTATGAGCGCACAAGGGGATCC	Tb927.11.8750_rev	GGGGACCACTTTGTACAAGAAAGCTGGGTTCAATCGTCATTGTCATCACTAGAATCAG
1_hypothetical	D5	Tb927.11.13590_for	GGGGACAAGTTTGTACAAAAAAGCAGGCTATGATGAAAAATACCGTGCGGCA	Tb927.11.13590_rev	GGGGACCACTTTGTACAAGAAAGCTGGGTTTACGTGGTGCTTTTTTCCCTTTGA
1_hypothetical	D6	Tb927.8.5320_for	GGGGACAAGTTTGTACAAAAAAGCAGGCTATGCCGTTGGGCACTGATG	Tb927.8.5320_rev	GGGGACCACTTTGTACAAGAAAGCTGGGTTCAGTCAGTGGCCCGTTCA
1_hypothetical	D7	Tb927.11.16450_for	GGGGACAAGTTTGTACAAAAAAGCAGGCTATGGTTTCCAAAACGCGGCG	Tb927.11.16450_rev	GGGGACCACTTTGTACAAGAAAGCTGGGTCTACTTCACTTTCTTTTTCAACTTTAGTTT
1_hypothetical	D8	Tb927.10.12300_for	GGGGACAAGTTTGTACAAAAAAGCAGGCTATGGCTAACTTTGTGCGCAAAGG	Tb927.10.12300_rev	GGGGACCACTTTGTACAAGAAAGCTGGGTCTAGTACCCCGGCGGACG
1_hypothetical	D9	Tb927.2.4445_for	GGGGACAAGTTTGTACAAAAAAGCAGGCTATGTCGACTGAGGCTACCAATGA	Tb927.2.4445_rev	GGGGACCACTTTGTACAAGAAAGCTGGGTCTAGCGGCGCTGTTCCTG
1_hypothetical	D10	Tb927.4.2160_for	GGGGACAAGTTTGTACAAAAAAGCAGGCTATGACCGAGTCGTACCGATCG	Tb927.4.2160_rev	GGGGACCACTTTGTACAAGAAAGCTGGGTCTACACTTCAAGCATCTCGCAATCA
1_hypothetical	D11	Tb927.10.440_for	GGGGACAAGTTTGTACAAAAAAGCAGGCTATGTCCAACCGAGGCTTTACCC	Tb927.10.440_rev	GGGGACCACTTTGTACAAGAAAGCTGGGTTCAAAGAGTTGCATCCGTAGCAG
1_hypothetical	D12	Tb927.4.1420_for	GGGGACAAGTTTGTACAAAAAAGCAGGCTATGTTGTGCCGGTCATCACTG	Tb927.4.1420_rev	GGGGACCACTTTGTACAAGAAAGCTGGGTTCAACTAAGTTTCGCGCCCAC
1_hypothetical	D13	Tb927.3.2370_for	GGGGACAAGTTTGTACAAAAAAGCAGGCTATGAACAACAAGGCACCGGG	Tb927.3.2370_rev	GGGGACCACTTTGTACAAGAAAGCTGGGTTTATTTGCTTCGGTATGTCTGGATCTC
1_hypothetical	D14	Tb927.11.2070_for	GGGGACAAGTTTGTACAAAAAAGCAGGCTATGGTGCGCTCGCTTGTTG	Tb927.11.2070_rev	GGGGACCACTTTGTACAAGAAAGCTGGGTTCATACGTCACGGGACATCTCA
1_hypothetical	D15	Tb927.9.10510_for	GGGGACAAGTTTGTACAAAAAAGCAGGCTATGCACACACTGCTGGTTACC	Tb927.9.10510_rev	GGGGACCACTTTGTACAAGAAAGCTGGGTTCAACCTTTGGCATCTTCCCTATAAGC
1_hypothetical	D16	Tb927.6.4360_for	GGGGACAAGTTTGTACAAAAAAGCAGGCTATGCGGGAGAGTAATATACACATGTCG	Tb927.6.4360_rev	GGGGACCACTTTGTACAAGAAAGCTGGGTTTACCCCTCTGTGTTTGGAATGGG
1_hypothetical	D17	Tb927.10.3040_for	GGGGACAAGTTTGTACAAAAAAGCAGGCTATGTTTTCACGAAGCTTTGTCGGA	Tb927.10.3040_rev	GGGGACCACTTTGTACAAGAAAGCTGGGTCTAGTACTTGAAGATGATGCTCTCAATAGG
1_hypothetical	D18	Tb927.11.1650_for	GGGGACAAGTTTGTACAAAAAAGCAGGCTATGCTCCGGTGTTCGACTTTTT	Tb927.11.1650_rev	GGGGACCACTTTGTACAAGAAAGCTGGGTTCATTCCTCACACTCTTCCTCTAAAAGA
1_hypothetical	D19	Tb927.5.4390_for	GGGGACAAGTTTGTACAAAAAAGCAGGCTATGTCTGGAGTTGTTATAAAGAAGCCC	Tb927.5.4390_rev	GGGGACCACTTTGTACAAGAAAGCTGGGTCTACTCTCTGGAGGTAATCATTCGGT
1_hypothetical	D20	Tb927.8.3090_for	GGGGACAAGTTTGTACAAAAAAGCAGGCTATGCAAGTCTCTCCACGTGTGC	Tb927.8.3090_rev	GGGGACCACTTTGTACAAGAAAGCTGGGTCTAACTTGAAAACGGCTTGTTTTTATCCC
1_hypothetical	D21	Tb927.4.3780_for	GGGGACAAGTTTGTACAAAAAAGCAGGCTATGTTTAGAAACCGTTTTTTCATTAGGCTT	Tb927.4.3780_rev	GGGGACCACTTTGTACAAGAAAGCTGGGTTCAACTTTCATCGATACCTTTTGAAAGGC
1_hypothetical	D22	Tb927.11.13660_for	GGGGACAAGTTTGTACAAAAAAGCAGGCTATGAGCCACCCTAAGATGAAGCT	Tb927.11.13660_rev	GGGGACCACTTTGTACAAGAAAGCTGGGTTTAGATCCCAACTCTTGCGGGA
1_hypothetical	D23	Tb927.10.4790_for	GGGGACAAGTTTGTACAAAAAAGCAGGCTATGTCCCACGATGGGGATGATG	Tb927.10.4790_rev	GGGGACCACTTTGTACAAGAAAGCTGGGTTTACTTCCCCTTCTGCAACTTTAACAG
1_hypothetical	D24	Tb927.11.7430_for	GGGGACAAGTTTGTACAAAAAAGCAGGCTATGAGCCGTGCCAACGTG	Tb927.11.7430_rev	GGGGACCACTTTGTACAAGAAAGCTGGGTTTAACATTGGAGTACCTGCGCG
1_hypothetical	E1	Tb927.10.7660_for	GGGGACAAGTTTGTACAAAAAAGCAGGCTATGTACAACTGCAGTTCCAAGTTTAGG	Tb927.10.7660_rev	GGGGACCACTTTGTACAAGAAAGCTGGGTTCACCGAAACGGGAAAAAGGC
1_hypothetical	E2	Tb927.7.2600_for	GGGGACAAGTTTGTACAAAAAAGCAGGCTATGCCTACGGTGCAGCCA	Tb927.7.2600_rev	GGGGACCACTTTGTACAAGAAAGCTGGGTTTAGTCCTCACCACCAAACTCCTG

1_hypothetical	E3	Tb927.10.2830_for	GGGGACAAGTTTGTACAAAAAAGCAGGCTATGCATCCCCGTGTTGCTAAAC	Tb927.10.2830_rev	GGGGACCACTTTGTACAAGAAAGCTGGGTCTATTTTTCAGAAAAATCACCCTCACCCC
1_hypothetical	E4	Tb927.3.2910_for	GGGGACAAGTTTGTACAAAAAAGCAGGCTATGGGAAGGCAGTCCCAGAAG	Tb927.3.2910_rev	GGGGACCACTTTGTACAAGAAAGCTGGGTTTATCTGACGGAGGACATCATTTTCTCC
1_hypothetical	E5	Tb927.9.720_for	GGGGACAAGTTTGTACAAAAAAGCAGGCTATGCTTAAAATTGCAGCAGTCATTTTGT	Tb927.9.720_rev	GGGGACCACTTTGTACAAGAAAGCTGGGTCTATTGTTTTTTTGTTCTTTTTGACTTTTG
1_hypothetical	E6	Tb927.3.3880_for	GGGGACAAGTTTGTACAAAAAAGCAGGCTATGAGCAACCGGGAGCCA	Tb927.3.3880_rev	GGGGACCACTTTGTACAAGAAAGCTGGGTCTACTTCTGTGACGAAGCGGC
1_hypothetical	E7	Tb927.10.4810_for	GGGGACAAGTTTGTACAAAAAAGCAGGCTATGCAGGTAATTGACATGTTTGCTCT	Tb927.10.4810_rev	GGGGACCACTTTGTACAAGAAAGCTGGGTTTATTTACGGAATGACACTACACGATCAA
1_hypothetical	E8	Tb927.11.13610_for	GGGGACAAGTTTGTACAAAAAAGCAGGCTATGGGTCAGAGCACATCTTACGA	Tb927.11.13610_rev	GGGGACCACTTTGTACAAGAAAGCTGGGTTTAGAGGTCCTCCTCTTGCTCTCC
1_hypothetical	E9	Tb927.10.5800_for	GGGGACAAGTTTGTACAAAAAAGCAGGCTATGGCGGGACCTTCCAATGC	Tb927.10.5800_rev	GGGGACCACTTTGTACAAGAAAGCTGGGTCTAAAGTTGGTTTTCACACCACGGA
1_hypothetical	E10	Tb927.10.140_for	GGGGACAAGTTTGTACAAAAAAGCAGGCTATGAATGAGAAGCAACATTTTCTTCCTATG	Tb927.10.140_rev	GGGGACCACTTTGTACAAGAAAGCTGGGTTCAGCCCCACGTATTCCAGAG
1_hypothetical	E11	Tb927.11.9500_for	GGGGACAAGTTTGTACAAAAAAGCAGGCTATGGTCTCCGCAAATAAATCCACAA	Tb927.11.9500_rev	GGGGACCACTTTGTACAAGAAAGCTGGGTCTACGATGCCATGTCTTTGTTGTCC
1_hypothetical	E12	Tb927.3.4170_for	GGGGACAAGTTTGTACAAAAAAGCAGGCTATGTCCAGCAATAATCCCTCCGA	Tb927.3.4170_rev	GGGGACCACTTTGTACAAGAAAGCTGGGTCTAACGAATGCGTGCAAAATCCG
1_hypothetical	E13	Tb927.3.3680_for	GGGGACAAGTTTGTACAAAAAAGCAGGCTATGCCGCCGGTTACGGGC	Tb927.3.3680_rev	GGGGACCACTTTGTACAAGAAAGCTGGGTTTATGTCGATTTGGGGTCGCG
1_hypothetical	E14	Tb927.9.2290_for	GGGGACAAGTTTGTACAAAAAAGCAGGCTATGCCCTTAAATAATGTACAGGTGGAG	Tb927.9.2290_rev	GGGGACCACTTTGTACAAGAAAGCTGGGTTTATTCTACCGTATGCAAGGGAAGATCG
1_hypothetical	E15	Tb927.8.5360_for	GGGGACAAGTTTGTACAAAAAAGCAGGCTATGGTTGTGGTCGTGGGCG	Tb927.8.5360_rev	GGGGACCACTTTGTACAAGAAAGCTGGGTTCACTGTGGTTGCTCATGCC
1_hypothetical	E16	Tb927.1.3750_for	GGGGACAAGTTTGTACAAAAAAGCAGGCTATGTTGATTGGCATGAAATATTCTGCTC	Tb927.1.3750_rev	GGGGACCACTTTGTACAAGAAAGCTGGGTCTACCATCGTACATATGTTTTTTTTTTGG
1_hypothetical	E17	Tb927.10.8670_for	GGGGACAAGTTTGTACAAAAAAGCAGGCTATGCTGCGCCTCATTGCA	Tb927.10.8670_rev	GGGGACCACTTTGTACAAGAAAGCTGGGTTCACCTTTTCTGCAAACGCCA
1_hypothetical	E18	Tb927.5.2840_for	GGGGACAAGTTTGTACAAAAAAGCAGGCTATGCTTCGGCGGTTCGTTG	Tb927.5.2840_rev	GGGGACCACTTTGTACAAGAAAGCTGGGTTCAACCACGCCACTTCTTACTGT
1_hypothetical	E19	Tb927.7.7440_for	GGGGACAAGTTTGTACAAAAAAGCAGGCTATGTCACGAAAAGCTCTTACAAGACC	Tb927.7.7440_rev	GGGGACCACTTTGTACAAGAAAGCTGGGTTTACAAAACACCACGTCTGTGGTT
1_hypothetical	E20	Tb927.10.14100_for	GGGGACAAGTTTGTACAAAAAAGCAGGCTATGCTCACGCAAGCGGGT	Tb927.10.14100_rev	GGGGACCACTTTGTACAAGAAAGCTGGGTTCAGCAGCCTGTGATCCCTC
1_hypothetical	E21	Tb927.7.4925_for	GGGGACAAGTTTGTACAAAAAAGCAGGCTATGTTATTACTGCAGACACTGATGTTAATG	Tb927.7.4925_rev	GGGGACCACTTTGTACAAGAAAGCTGGGTCTAAGACACCTTCAAGCCAACAATCA
1_hypothetical	E22	Tb927.5.4510_for	GGGGACAAGTTTGTACAAAAAAGCAGGCTATGCTCAGGTTAAATAAATTTTTTTCTCAT	Tb927.5.4510_rev	GGGGACCACTTTGTACAAGAAAGCTGGGTTCACTTTTTAAACTTCGATCGACCCTT
1_hypothetical	E23	Tb927.11.650_for	GGGGACAAGTTTGTACAAAAAAGCAGGCTATGTCAGGCCCGGTTGTGG	Tb927.11.650_rev	GGGGACCACTTTGTACAAGAAAGCTGGGTCTATATTGCAGGAACTTTTCGTGTTTCAA
1_hypothetical	E24	Tb927.6.2280_for	GGGGACAAGTTTGTACAAAAAAGCAGGCTATGTCTGCACTTGCGGAGGC	Tb927.6.2280_rev	GGGGACCACTTTGTACAAGAAAGCTGGGTTTACTGTTTCTGCTGCTGCTGTT
1_hypothetical	F2	Tb927.11.8850_for	GGGGACAAGTTTGTACAAAAAAGCAGGCTATGCGGGCACTATACCAGTTCG	Tb927.11.8850_rev	GGGGACCACTTTGTACAAGAAAGCTGGGTTCACAAGAATGAATTGCTATCTTGTGTTTT
1_hypothetical	F3	Tb927.10.5550_for	GGGGACAAGTTTGTACAAAAAAGCAGGCTATGCGGCGCTCGTTCGTC	Tb927.10.5550_rev	GGGGACCACTTTGTACAAGAAAGCTGGGTCTACTTCGGATGTATTTTGCACACATC
1_hypothetical	F4	Tb927.10.4420_for	GGGGACAAGTTTGTACAAAAAAGCAGGCTATGAGAAAAAAGAGGGAAACAAAAAAGAAA	Tb927.10.4420_rev	GGGGACCACTTTGTACAAGAAAGCTGGGTTTAAAAGAAAGAAAGGAAAGAAAGAAAAAA
1_hypothetical	F5	Tb927.10.7820_for	GGGGACAAGTTTGTACAAAAAAGCAGGCTATGAGGCTAACCAACACGAAACC	Tb927.10.7820_rev	GGGGACCACTTTGTACAAGAAAGCTGGGTTTACTCTTTCTCCTTCCGAGAAAATGAC
1_hypothetical	F6	Tb927.10.5100_for	GGGGACAAGTTTGTACAAAAAAGCAGGCTATGTTTGTATTTGTGGTGTGTCCACG	Tb927.10.5100_rev	GGGGACCACTTTGTACAAGAAAGCTGGGTTTATTTAAAAAAAAATAAAAGAGAAGAGAA
1_hypothetical	F7	Tb927.5.4490_for	GGGGACAAGTTTGTACAAAAAAGCAGGCTATGGTACTGATGTCAAAACGAGATGC	Tb927.5.4490_rev	GGGGACCACTTTGTACAAGAAAGCTGGGTCTAACCAAATACCCTTTGCGCAGA
1_hypothetical	F8	Tb927.9.12870_for	GGGGACAAGTTTGTACAAAAAAGCAGGCTATGACGTTGTATTCTATTTACATCTTCAAT	Tb927.9.12870_rev	GGGGACCACTTTGTACAAGAAAGCTGGGTCTAAAGGATCTTCTGAGAGTGGAAAAACT
1_hypothetical	F9	Tb927.9.12890_for	GGGGACAAGTTTGTACAAAAAAGCAGGCTATGACGAAACTGGTGCGCAA	Tb927.9.12890_rev	GGGGACCACTTTGTACAAGAAAGCTGGGTTTAATCATGCAGATCCTCATTCCGG
1_hypothetical	F10	Tb927.8.4535_for	GGGGACAAGTTTGTACAAAAAAGCAGGCTATGGAGGGGCTTGGATTAGAAACG	Tb927.8.4535_rev	GGGGACCACTTTGTACAAGAAAGCTGGGTTTAACACAACTCAAGCTCAAAAGCTGG
1_hypothetical	F11	Tb927.8.3450_for	GGGGACAAGTTTGTACAAAAAAGCAGGCTATGCGGGGCTCTACGGATC	Tb927.8.3450_rev	GGGGACCACTTTGTACAAGAAAGCTGGGTCTAAGAGGTCCCTACCACCTCCC
1_hypothetical	F12	Tb927.9.14990_for	GGGGACAAGTTTGTACAAAAAAGCAGGCTATGTGGGCTTCTGGCTCTCG	Tb927.9.14990_rev	GGGGACCACTTTGTACAAGAAAGCTGGGTCTAGTACGATGCTTGCTTAACACAACC
1_hypothetical	F13	Tb927.9.1590_for	GGGGACAAGTTTGTACAAAAAAGCAGGCTATGCCAGTACCGGTTGTGGA	Tb927.9.1590_rev	GGGGACCACTTTGTACAAGAAAGCTGGGTTTACAGTTTTCCAATTGCATGGTACCA
1_hypothetical	F14	Tb927.4.2420_for	GGGGACAAGTTTGTACAAAAAAGCAGGCTATGACTCTTGCGGATACGCTACA	Tb927.4.2420_rev	GGGGACCACTTTGTACAAGAAAGCTGGGTTCATGAAACAGTCGGCTTCATACG
1_hypothetical	F15	Tb927.1.380_for	GGGGACAAGTTTGTACAAAAAAGCAGGCTATGTTTCAATTGGTTTCTGTGGCCG	Tb927.1.380_rev	GGGGACCACTTTGTACAAGAAAGCTGGGTTTACTGACCCAGTGCCTGAAGC
1_hypothetical	F16	Tb927.6.120_for	GGGGACAAGTTTGTACAAAAAAGCAGGCTATGTTTCAATTGGTTTCTGCGGC	Tb927.6.120_rev	GGGGACCACTTTGTACAAGAAAGCTGGGTTTACTGACCCAGTGCCTGAAGC
1_hypothetical	F18	Tb927.6.4570_for	GGGGACAAGTTTGTACAAAAAAGCAGGCTATGCTGAGATTATGTAGAAGACTATCATAC	Tb927.6.4570_rev	GGGGACCACTTTGTACAAGAAAGCTGGGTTCAGTACGACTCCTTTGTGTGAATG
1_hypothetical	F19	Tb927.2.680_for	GGGGACAAGTTTGTACAAAAAAGCAGGCTATGTTTCAATTGGTTTCTGTGGCCG	Tb927.2.680_rev	GGGGACCACTTTGTACAAGAAAGCTGGGTTTACTGACCCAGTGCCTGGAG
1_hypothetical	F20	Tb927.6.2310_for	GGGGACAAGTTTGTACAAAAAAGCAGGCTATGCTTTTCATGGTTTACACGTCGC	Tb927.6.2310_rev	GGGGACCACTTTGTACAAGAAAGCTGGGTCTACAGCAGCAAATTAGAAATACACAAACC
1_hypothetical	F21	Tb927.8.4630_for	GGGGACAAGTTTGTACAAAAAAGCAGGCTATGATTCTCGGTCTCGGTGATGG	Tb927.8.4630_rev	GGGGACCACTTTGTACAAGAAAGCTGGGTTTAGCGATACCATGTTGGGTGC
1_hypothetical	F22	Tb927.11.13790_for	GGGGACAAGTTTGTACAAAAAAGCAGGCTATGTTATGGAGAAATATCTCTCGATGCC	Tb927.11.13790_rev	GGGGACCACTTTGTACAAGAAAGCTGGGTTCACAACAGCTTCCGACGTCC
1_hypothetical	F23	Tb927.10.5860_for	GGGGACAAGTTTGTACAAAAAAGCAGGCTATGGAGTTGAATGAGCAGGAACTG	Tb927.10.5860_rev	GGGGACCACTTTGTACAAGAAAGCTGGGTTCAATTTTCCTTGGCAACACTTGC
1_hypothetical	F24	Tb927.10.7460_for	GGGGACAAGTTTGTACAAAAAAGCAGGCTATGGCGACATTGAATGTTAGGTGT	Tb927.10.7460_rev	GGGGACCACTTTGTACAAGAAAGCTGGGTCTAGGCCCCCACGCTAACG
1_hypothetical	G1	Tb927.6.2600_for	GGGGACAAGTTTGTACAAAAAAGCAGGCTATGAGGAAACAATTAAATATGCAGGAGGA	Tb927.6.2600_rev	GGGGACCACTTTGTACAAGAAAGCTGGGTTTAACTAAAAAATGTGCCCATGCTCTG
1_hypothetical	G2	Tb927.10.12010_for	GGGGACAAGTTTGTACAAAAAAGCAGGCTATGTTACTCGATTTCATTTTCAGTCACCT	Tb927.10.12010_rev	GGGGACCACTTTGTACAAGAAAGCTGGGTTTATTCCACCTTTTCATCAACCTTCTCTG
1_hypothetical	G3	Tb927.5.645_for	GGGGACAAGTTTGTACAAAAAAGCAGGCTATGTTAGTGTCCAAACAAATAAGCCCT	Tb927.5.645_rev	GGGGACCACTTTGTACAAGAAAGCTGGGTTCAGCTGAAGGTTCGACACAAC
1_hypothetical	G4	Tb927.4.1470_for	GGGGACAAGTTTGTACAAAAAAGCAGGCTATGCGGCACTTTGGACCTTC	Tb927.4.1470_rev	GGGGACCACTTTGTACAAGAAAGCTGGGTTCAGTACTTTGTACGCATGTTGAACA
1_hypothetical	G5	Tb927.6.1420_for	GGGGACAAGTTTGTACAAAAAAGCAGGCTATGAGCAAACTAACACCGCGC	Tb927.6.1420_rev	GGGGACCACTTTGTACAAGAAAGCTGGGTTTACAAACCAGTAACAGCAAACTGTTTTG
1_hypothetical	G6	Tb927.11.13620_for	GGGGACAAGTTTGTACAAAAAAGCAGGCTATGTTTTTCGTAACGCCGCGT	Tb927.11.13620_rev	GGGGACCACTTTGTACAAGAAAGCTGGGTTCACCCTTTGCAACCATCCG
1_hypothetical	G7	Tb927.11.7090_for	GGGGACAAGTTTGTACAAAAAAGCAGGCTATGTCCGAAGAGTTGCGGTGG	Tb927.11.7090_rev	GGGGACCACTTTGTACAAGAAAGCTGGGTTCAAATGCTTTGCTTAGCGATGC
1_hypothetical	G8	Tb927.8.1040_for	GGGGACAAGTTTGTACAAAAAAGCAGGCTATGTCTTCGTCTCAGCAATTGGG	Tb927.8.1040_rev	GGGGACCACTTTGTACAAGAAAGCTGGGTTCAGTGAAACCGAGTTCCACAATAAC
1_hypothetical	G9	Tb927.11.14660_for	GGGGACAAGTTTGTACAAAAAAGCAGGCTATGAACGATATTTACGCACGGCG	Tb927.11.14660_rev	GGGGACCACTTTGTACAAGAAAGCTGGGTTTACTCACCTTCGATAAGAGGTTCTTCC
1_hypothetical	G10	Tb927.4.550_for	GGGGACAAGTTTGTACAAAAAAGCAGGCTATGTACGGCAACAAAGGACCTC	Tb927.4.550_rev	GGGGACCACTTTGTACAAGAAAGCTGGGTTCACTCCTCAGTTTTAAACGTTTTCTTCG
1_hypothetical	G11	Tb927.4.2320_for	GGGGACAAGTTTGTACAAAAAAGCAGGCTATGGTTGTGTTTCACCAATTGATACTTCC	Tb927.4.2320_rev	GGGGACCACTTTGTACAAGAAAGCTGGGTTTACGCATGTTTCTTCCCTGTTGA
1_hypothetical	G12	Tb927.2.3610_for	GGGGACAAGTTTGTACAAAAAAGCAGGCTATGTCTTCCACGAAGTGCGC	Tb927.2.3610_rev	GGGGACCACTTTGTACAAGAAAGCTGGGTCTAGTAGTTGGGATTTACCTTCGTTACC
1_hypothetical	G13	Tb927.8.1945_for	GGGGACAAGTTTGTACAAAAAAGCAGGCTATGTTTTCGTTACGGGAGATACTTTCG	Tb927.8.1945_rev	GGGGACCACTTTGTACAAGAAAGCTGGGTCTAGTAGGTATCTACCCAGGCGTTG
1_hypothetical	G14	Tb927.9.11370_for	GGGGACAAGTTTGTACAAAAAAGCAGGCTATGTCGCAATTATGGGTAAAGCGC	Tb927.9.11370_rev	GGGGACCACTTTGTACAAGAAAGCTGGGTTCAATATGAATCTCTCCTGTCGATATTGAT
1_hypothetical	G15	Tb927.3.1690_for	GGGGACAAGTTTGTACAAAAAAGCAGGCTATGGTACTTTTTTCTACATACCGCAGC	Tb927.3.1690_rev	GGGGACCACTTTGTACAAGAAAGCTGGGTTCAATGATGTGCCTGTGCTCC
1_hypothetical	G16	Tb927.8.3070_for	GGGGACAAGTTTGTACAAAAAAGCAGGCTATGGCCTATGTTTCTCCAGCACG	Tb927.8.3070_rev	GGGGACCACTTTGTACAAGAAAGCTGGGTCTACTTCGGTAACCGCTGCTG
1_hypothetical	G17	Tb927.9.8380_for	GGGGACAAGTTTGTACAAAAAAGCAGGCTATGTCAATGTTCGGTGCCCG	Tb927.9.8380_rev	GGGGACCACTTTGTACAAGAAAGCTGGGTTCATTTTCCCACGGAATTATTATTACGCG
1_hypothetical	G18	Tb927.11.6190_for	GGGGACAAGTTTGTACAAAAAAGCAGGCTATGACACCCGTACTTCATGATGC	Tb927.11.6190_rev	GGGGACCACTTTGTACAAGAAAGCTGGGTCTAAGCAATACCCACGGAAACACC
1_hypothetical	G19	Tb927.3.1960_for	GGGGACAAGTTTGTACAAAAAAGCAGGCTATGGAAGGACCACCTCCCG	Tb927.3.1960_rev	GGGGACCACTTTGTACAAGAAAGCTGGGTTCAACACGTCTCTCCTTTAGCCA
1_hypothetical	G20	Tb927.11.6470_for	GGGGACAAGTTTGTACAAAAAAGCAGGCTATGAGCCTCAAGGACTGTGGT	Tb927.11.6470_rev	GGGGACCACTTTGTACAAGAAAGCTGGGTTCAATCATCCTCGATGTCTGCCT
1_hypothetical	G21	Tb927.10.15790_for	GGGGACAAGTTTGTACAAAAAAGCAGGCTATGGCAAATGGCGTAACGGC	Tb927.10.15790_rev	GGGGACCACTTTGTACAAGAAAGCTGGGTTCATGTTTTGTTCGTTTCGCCG
1_hypothetical	G22	Tb927.10.14120_for	GGGGACAAGTTTGTACAAAAAAGCAGGCTATGCAAGTAAGTGACTTTGCGTGT	Tb927.10.14120_rev	GGGGACCACTTTGTACAAGAAAGCTGGGTCTATGACAGGCCGCGCTTT
1_hypothetical	G23	Tb927.1.1760_for	GGGGACAAGTTTGTACAAAAAAGCAGGCTATGGTGTGGTTACACTGGACAAC	Tb927.1.1760_rev	GGGGACCACTTTGTACAAGAAAGCTGGGTTTACTTCAGGGAAAGAAAAGTGATGCT
1_hypothetical	G24	Tb927.10.12070_for	GGGGACAAGTTTGTACAAAAAAGCAGGCTATGGACGGGGGTTTAAAATCCCT	Tb927.10.12070_rev	GGGGACCACTTTGTACAAGAAAGCTGGGTTTAAAAGGTAGGAAAAACAAAAGAAACAAA
1_hypothetical	H1	Tb927.11.16510_for	GGGGACAAGTTTGTACAAAAAAGCAGGCTATGGCACCTCCAAAGGCTTCA	Tb927.11.16510_rev	GGGGACCACTTTGTACAAGAAAGCTGGGTTTACTCCGTGATGAGCTTTTCACTTC
1_hypothetical	H2	Tb927.10.1220_for	GGGGACAAGTTTGTACAAAAAAGCAGGCTATGACATCTCATCCCGCTGTTCT	Tb927.10.1220_rev	GGGGACCACTTTGTACAAGAAAGCTGGGTTTAATCCAGGGCTTTCTGGGGG
1_hypothetical	H3	Tb927.2.4840_for	GGGGACAAGTTTGTACAAAAAAGCAGGCTATGCTACTTCTTCCGCCTTTCCT	Tb927.2.4840_rev	GGGGACCACTTTGTACAAGAAAGCTGGGTTCAATCGACATGAACCCGACCA
1_hypothetical	H4	Tb927.10.10080_for	GGGGACAAGTTTGTACAAAAAAGCAGGCTATGCCACCTAAGAAGGACGAGG	Tb927.10.10080_rev	GGGGACCACTTTGTACAAGAAAGCTGGGTTCACGAAGATCTGTCTGCCCTC
1_hypothetical	H5	Tb927.6.2510_for	GGGGACAAGTTTGTACAAAAAAGCAGGCTATGTTAAGACGTTTGTTTACGCGC	Tb927.6.2510_rev	GGGGACCACTTTGTACAAGAAAGCTGGGTTTATGATGCACATTTCAGTAACTTCTCCC
1_hypothetical	H6	Tb927.10.11370_for	GGGGACAAGTTTGTACAAAAAAGCAGGCTATGTTTCGTCCCACCACCGC	Tb927.10.11370_rev	GGGGACCACTTTGTACAAGAAAGCTGGGTCTAGTACCACGTCCGCGGT
1_hypothetical	H7	Tb927.10.40_for	GGGGACAAGTTTGTACAAAAAAGCAGGCTATGTATCGACGGGCAAGTTCTCG	Tb927.10.40_rev	GGGGACCACTTTGTACAAGAAAGCTGGGTCTATTTGAAACTCCTCAGACAAGCCA
1_hypothetical	H8	Tb927.10.11280_for	GGGGACAAGTTTGTACAAAAAAGCAGGCTATGGTTGCCCGTTTCCTCTGG	Tb927.10.11280_rev	GGGGACCACTTTGTACAAGAAAGCTGGGTCTAACAAAGCAGCTGGCGATCA
1_hypothetical	H9	Tb927.10.1610_for	GGGGACAAGTTTGTACAAAAAAGCAGGCTATGTCATATAGTCTCCGTAAACTGAGGC	Tb927.10.1610_rev	GGGGACCACTTTGTACAAGAAAGCTGGGTTTAGTGAACACCAAGTCCCCACT
1_hypothetical	H10	Tb927.9.10060_for	GGGGACAAGTTTGTACAAAAAAGCAGGCTATGTCTCGGAAAGCCGGAGA	Tb927.9.10060_rev	GGGGACCACTTTGTACAAGAAAGCTGGGTTCACTTGTATGCTACGAGTGCTGG
1_hypothetical	H11	Tb927.10.10040_for	GGGGACAAGTTTGTACAAAAAAGCAGGCTATGTATAAATTAGAATTGTTGTGTACTTGG	Tb927.10.10040_rev	GGGGACCACTTTGTACAAGAAAGCTGGGTTTATGCACCTGGGAGCGGA
1_hypothetical	H12	Tb927.10.12460_for	GGGGACAAGTTTGTACAAAAAAGCAGGCTATGGACACATTATTGATGATTGGGGC	Tb927.10.12460_rev	GGGGACCACTTTGTACAAGAAAGCTGGGTTCACGAATTGAATGTATGTATCCGCG
1_hypothetical	H13	Tb927.11.12330_for	GGGGACAAGTTTGTACAAAAAAGCAGGCTATGGTGCGTTGTCAGTCGTC	Tb927.11.12330_rev	GGGGACCACTTTGTACAAGAAAGCTGGGTTTATTGCAACTTGTCCAATTTGACGAC
1_hypothetical	H14	Tb927.4.3060_for	GGGGACAAGTTTGTACAAAAAAGCAGGCTATGGAAATCATTGATCGGCCCC	Tb927.4.3060_rev	GGGGACCACTTTGTACAAGAAAGCTGGGTTCAATCACGTGAGAAGCGGGA
1_hypothetical	H15	Tb927.9.7980_for	GGGGACAAGTTTGTACAAAAAAGCAGGCTATGGCTCGCACTCGTCGT	Tb927.9.7980_rev	GGGGACCACTTTGTACAAGAAAGCTGGGTTTATTGAGTTGGTTGCTGCTCACT
1_hypothetical	H16	Tb927.10.6990_for	GGGGACAAGTTTGTACAAAAAAGCAGGCTATGACTGGCAATTCGCATTGTGT	Tb927.10.6990_rev	GGGGACCACTTTGTACAAGAAAGCTGGGTCTATGATTGTTTAACAGCCGTAATCCCG
1_hypothetical	H17	Tb927.10.10110_for	GGGGACAAGTTTGTACAAAAAAGCAGGCTATGCCCCTAAGATGCACTAAACCA	Tb927.10.10110_rev	GGGGACCACTTTGTACAAGAAAGCTGGGTTCAATTCCACGGTTGAAACAACAAC
1_hypothetical	H18	Tb927.9.7810_for	GGGGACAAGTTTGTACAAAAAAGCAGGCTATGTCGAATCTCCCACCCTTAACT	Tb927.9.7810_rev	GGGGACCACTTTGTACAAGAAAGCTGGGTCTATGTTTTATTCTCCCCTTCATCCACG
1_hypothetical	H19	Tb927.10.1160_for	GGGGACAAGTTTGTACAAAAAAGCAGGCTATGGTGCGGTACGCCAAAC	Tb927.10.1160_rev	GGGGACCACTTTGTACAAGAAAGCTGGGTCTAAGCTTCTGCTAATTGTTCCATCTGA
1_hypothetical	H20	Tb927.3.4480_for	GGGGACAAGTTTGTACAAAAAAGCAGGCTATGGCATTAGGGGGTTCACTCC	Tb927.3.4480_rev	GGGGACCACTTTGTACAAGAAAGCTGGGTTCAAATCGCGATCCACTTCAATGA
1_hypothetical	H21	Tb927.8.7210_for	GGGGACAAGTTTGTACAAAAAAGCAGGCTATGTCGAAGAAGCAAGGCGC	Tb927.8.7210_rev	GGGGACCACTTTGTACAAGAAAGCTGGGTTTATTTTAGAACACGATCCACTACCCATC
1_hypothetical	H22	Tb927.10.390_for	GGGGACAAGTTTGTACAAAAAAGCAGGCTATGTCCGTGCGCATTCGC	Tb927.10.390_rev	GGGGACCACTTTGTACAAGAAAGCTGGGTCTACAGCATGGTGCAACAACCA
1_hypothetical	H23	Tb927.4.5190_for	GGGGACAAGTTTGTACAAAAAAGCAGGCTATGACAAAAAGACATAGCGGACGC	Tb927.4.5190_rev	GGGGACCACTTTGTACAAGAAAGCTGGGTTTACTTTAGCACACGCTCCACC
1_hypothetical	H24	Tb927.11.14995_for	GGGGACAAGTTTGTACAAAAAAGCAGGCTATGCGCGGCAATCGTCTG	Tb927.11.14995_rev	GGGGACCACTTTGTACAAGAAAGCTGGGTTTAGTACAGCGACTGCGTCCT
1_hypothetical	I1	Tb927.9.10360_for	GGGGACAAGTTTGTACAAAAAAGCAGGCTATGACGGACCGTTTAACGGC	Tb927.9.10360_rev	GGGGACCACTTTGTACAAGAAAGCTGGGTTCACTCACAATTAATATGCCTCTCAACTT
1_hypothetical	I2	Tb927.2.5140_for	GGGGACAAGTTTGTACAAAAAAGCAGGCTATGAAGCGTGTAGCTCAGAGTGC	Tb927.2.5140_rev	GGGGACCACTTTGTACAAGAAAGCTGGGTTCACAATTCTTTGAGAGCCTTTTCTTGA
1_hypothetical	I3	Tb927.6.4180_for	GGGGACAAGTTTGTACAAAAAAGCAGGCTATGAGTGCGCCGCGTGTC	Tb927.6.4180_rev	GGGGACCACTTTGTACAAGAAAGCTGGGTTTAGGCCAAGTACCGAGCACC
1_hypothetical	I4	Tb927.4.2740_for	GGGGACAAGTTTGTACAAAAAAGCAGGCTATGGAAGCTGTCTTCTACGCATTTG	Tb927.4.2740_rev	GGGGACCACTTTGTACAAGAAAGCTGGGTTTACTTTTGCGAGGAGGTTGTGC
1_hypothetical	I5	Tb927.9.16070_for	GGGGACAAGTTTGTACAAAAAAGCAGGCTATGTTCGTCCGTCATTTCAAAGAAGT	Tb927.9.16070_rev	GGGGACCACTTTGTACAAGAAAGCTGGGTTCATGTGAGCATATTGGCAATGTCA
1_hypothetical	I6	Tb927.11.12730_for	GGGGACAAGTTTGTACAAAAAAGCAGGCTATGAGCTGCCTTCATGGTGGC	Tb927.11.12730_rev	GGGGACCACTTTGTACAAGAAAGCTGGGTTTAACCCGGTTTGTGGCAACA
1_hypothetical	I7	Tb927.11.12700_for	GGGGACAAGTTTGTACAAAAAAGCAGGCTATGCACTTAACCTTAGGTGTGTTAACG	Tb927.11.12700_rev	GGGGACCACTTTGTACAAGAAAGCTGGGTTCATTTTTCTGTGAAGCGCATGTAC
1_hypothetical	I8	Tb927.4.1370_for	GGGGACAAGTTTGTACAAAAAAGCAGGCTATGAACTGCAGCTCCACATTGG	Tb927.4.1370_rev	GGGGACCACTTTGTACAAGAAAGCTGGGTCTAGAAAAACGGGTAAGGCGGTG
1_hypothetical	I9	Tb927.2.4700_for	GGGGACAAGTTTGTACAAAAAAGCAGGCTATGACAGGAAAAGATGGAAAAGTACTGG	Tb927.2.4700_rev	GGGGACCACTTTGTACAAGAAAGCTGGGTTCAAGCGGTAGCGCATTCC
1_hypothetical	I10	Tb927.5.690_for	GGGGACAAGTTTGTACAAAAAAGCAGGCTATGGCGTTTGGAAATGCGTCT	Tb927.5.690_rev	GGGGACCACTTTGTACAAGAAAGCTGGGTTCATATATCACCACCCGCTGCA
1_hypothetical	I11	Tb927.7.5780_for	GGGGACAAGTTTGTACAAAAAAGCAGGCTATGATATCACTACATTTGTCCTCGCAC	Tb927.7.5780_rev	GGGGACCACTTTGTACAAGAAAGCTGGGTTTATGAGTTTTGAAAGACAAAACTGTCCAG
1_hypothetical	I12	Tb927.6.5060_for	GGGGACAAGTTTGTACAAAAAAGCAGGCTATGGCTGCCTCACAGCTTATCG	Tb927.6.5060_rev	GGGGACCACTTTGTACAAGAAAGCTGGGTTCATGCTCGCTTCGACACATTAA
1_hypothetical	I13	Tb04.3I12.100_for	GGGGACAAGTTTGTACAAAAAAGCAGGCTATGCTTGCGAATCCATCTTCCTG	Tb04.3I12.100_rev	GGGGACCACTTTGTACAAGAAAGCTGGGTTTAAAAGGACTCAAAACGTAAAAGAAAAAA
1_hypothetical	I14	Tb927.10.1870_for	GGGGACAAGTTTGTACAAAAAAGCAGGCTATGCTGCGCTACAGCCGA	Tb927.10.1870_rev	GGGGACCACTTTGTACAAGAAAGCTGGGTCTAAACAACACGGAATTCAAAGTCTTGT
1_hypothetical	I15	Tb927.7.1380_for	GGGGACAAGTTTGTACAAAAAAGCAGGCTATGAGTACGATGGATGTCCCGG	Tb927.7.1380_rev	GGGGACCACTTTGTACAAGAAAGCTGGGTTCACCTAAAGCATAGTTGAGTTTGTGT
1_hypothetical	I16	Tb927.7.3590_for	GGGGACAAGTTTGTACAAAAAAGCAGGCTATGCTTCGCCGCGCAGCT	Tb927.7.3590_rev	GGGGACCACTTTGTACAAGAAAGCTGGGTCTAATTCACTATCAAATGCAGGACAGCA
1_hypothetical	I17	Tb927.9.9810_for	GGGGACAAGTTTGTACAAAAAAGCAGGCTATGTCGCTTCAAACAACCCTAATACA	Tb927.9.9810_rev	GGGGACCACTTTGTACAAGAAAGCTGGGTCTAAAATACGTTAACTGTCATAATGGCATA
1_hypothetical	I18	Tb927.7.7090_for	GGGGACAAGTTTGTACAAAAAAGCAGGCTATGCTCGCACGTACGCTTC	Tb927.7.7090_rev	GGGGACCACTTTGTACAAGAAAGCTGGGTTTACTCCATAGCGAACACCGCT
1_hypothetical	I19	Tb927.9.13460_for	GGGGACAAGTTTGTACAAAAAAGCAGGCTATGAGTGGTGACAGCGAGCC	Tb927.9.13460_rev	GGGGACCACTTTGTACAAGAAAGCTGGGTTCATGCACCACTCATCGCCA
1_hypothetical	I20	Tb927.10.14130_for	GGGGACAAGTTTGTACAAAAAAGCAGGCTATGAAACCGTCTCAACCGTCG	Tb927.10.14130_rev	GGGGACCACTTTGTACAAGAAAGCTGGGTCTACTTTTTCCGTGTGTTGGCAGT
1_hypothetical	I21	Tb927.11.730_for	GGGGACAAGTTTGTACAAAAAAGCAGGCTATGTCGGGGTTTTCAGGGACA	Tb927.11.730_rev	GGGGACCACTTTGTACAAGAAAGCTGGGTTTAAGTCCCCGAGCCACGT
1_hypothetical	I22	Tb927.9.17670_for	GGGGACAAGTTTGTACAAAAAAGCAGGCTATGGGGATTAATTTATGTGTGTCACTCTAC	Tb927.9.17670_rev	GGGGACCACTTTGTACAAGAAAGCTGGGTTCACAAATATGTTTTTCTACTCATCGTGAG
1_hypothetical	I23	Tb927.8.1370_for	GGGGACAAGTTTGTACAAAAAAGCAGGCTATGTCGTCCAAGGTTGGTCAGG	Tb927.8.1370_rev	GGGGACCACTTTGTACAAGAAAGCTGGGTTTAGCTCCGTAGCTTGTTGAAGTCG
1_hypothetical	I24	Tb927.5.1840_for	GGGGACAAGTTTGTACAAAAAAGCAGGCTATGCTTTTTTCCCTTGACGGGC	Tb927.5.1840_rev	GGGGACCACTTTGTACAAGAAAGCTGGGTTCATACCTCCCGCCTCATTGTATC
1_hypothetical	J1	Tb927.10.4410_for	GGGGACAAGTTTGTACAAAAAAGCAGGCTATGATGGAGGTGGGCGGTT	Tb927.10.4410_rev	GGGGACCACTTTGTACAAGAAAGCTGGGTTCACGCAAAAGCCACACGC
1_hypothetical	J2	Tb927.4.1670_for	GGGGACAAGTTTGTACAAAAAAGCAGGCTATGGATAAGGCAGTTGGTAAACTAGTTAAG	Tb927.4.1670_rev	GGGGACCACTTTGTACAAGAAAGCTGGGTCTATTCCCATATAAACCACCCAACAACG
1_hypothetical	J3	Tb927.4.2100_for	GGGGACAAGTTTGTACAAAAAAGCAGGCTATGACCGTAATTCATTTATTTCTTTATGTG	Tb927.4.2100_rev	GGGGACCACTTTGTACAAGAAAGCTGGGTTTAATACCGTCCTTTTTCTTTCTGGCT
1_hypothetical	J4	Tb927.10.11350_for	GGGGACAAGTTTGTACAAAAAAGCAGGCTATGTTTCGAGTGACTGGCTTGC	Tb927.10.11350_rev	GGGGACCACTTTGTACAAGAAAGCTGGGTCTAACTTCGCATATCCTTTGCGTTCT
1_hypothetical	J5	Tb927.11.2160_for	GGGGACAAGTTTGTACAAAAAAGCAGGCTATGCAGTCGCGGCGTAAC	Tb927.11.2160_rev	GGGGACCACTTTGTACAAGAAAGCTGGGTTTAGTGATGTGCATGTACCTCACG
1_hypothetical	J6	Tb927.11.16320_for	GGGGACAAGTTTGTACAAAAAAGCAGGCTATGAATTCACAGCGGTCGGAGT	Tb927.11.16320_rev	GGGGACCACTTTGTACAAGAAAGCTGGGTTCAGGTGTTTTTATAAATCTTCCCCTTGAA
1_hypothetical	J7	Tb927.7.7010_for	GGGGACAAGTTTGTACAAAAAAGCAGGCTATGCTTCGGCACTGTACCGC	Tb927.7.7010_rev	GGGGACCACTTTGTACAAGAAAGCTGGGTCTACAACAACTCCCGTGATGGC
1_hypothetical	J8	Tb927.4.4815_for	GGGGACAAGTTTGTACAAAAAAGCAGGCTATGTCGGAAACGAACTTCACGT	Tb927.4.4815_rev	GGGGACCACTTTGTACAAGAAAGCTGGGTTTACAGTGTCTTCTCCACCTCCC
1_hypothetical	J9	Tb927.9.8570_for	GGGGACAAGTTTGTACAAAAAAGCAGGCTATGAAAGGAGGTGAAACAGCAGC	Tb927.9.8570_rev	GGGGACCACTTTGTACAAGAAAGCTGGGTTCACTGATCTTTATATACGGGAAGGTTTAC
1_hypothetical	J10	Tb927.3.940_for	GGGGACAAGTTTGTACAAAAAAGCAGGCTATGAGGATCAGTTGCTCATGCCT	Tb927.3.940_rev	GGGGACCACTTTGTACAAGAAAGCTGGGTCTACCATCGCCGCATAATGTAGG
1_hypothetical	J11	Tb927.5.296b_for	GGGGACAAGTTTGTACAAAAAAGCAGGCTATGGAATATCTCCAGGGAAGGAAGC	Tb927.5.296b_rev	GGGGACCACTTTGTACAAGAAAGCTGGGTTCATTGCTTCTGGGGCCGA
1_hypothetical	J12	Tb927.7.6020_for	GGGGACAAGTTTGTACAAAAAAGCAGGCTATGAACAATGATAAGTCAGAATTTTATATC	Tb927.7.6020_rev	GGGGACCACTTTGTACAAGAAAGCTGGGTTCATATTCCTTCTGCGGACAACTTTT
1_hypothetical	J13	Tb927.5.1170_for	GGGGACAAGTTTGTACAAAAAAGCAGGCTATGGAAAACAAAATGTCGGCTGAGC	Tb927.5.1170_rev	GGGGACCACTTTGTACAAGAAAGCTGGGTCTATGCACTCCCTGACAAAAGGG
1_hypothetical	J14	Tb927.4.2610_for	GGGGACAAGTTTGTACAAAAAAGCAGGCTATGTGTTTTACACATGTCTCTCTTGTGT	Tb927.4.2610_rev	GGGGACCACTTTGTACAAGAAAGCTGGGTTCAATACGGTTGAAAGTTGCTAGCC

1_hypothetical	J15	Tb927.10.3270_for	GGGGACAAGTTTGTACAAAAAAGCAGGCTATGTTGAGGCGTGTCCGTCC	Tb927.10.3270_rev	GGGGACCACTTTGTACAAGAAAGCTGGGTTCAGTTTCGGCGTCGCTCA
1_hypothetical	J16	Tb927.9.12480_for	GGGGACAAGTTTGTACAAAAAAGCAGGCTATGTCGAGTGGAAGCCATCGT	Tb927.9.12480_rev	GGGGACCACTTTGTACAAGAAAGCTGGGTCTAACCGCCATGGCGCCA
1_hypothetical	J17	Tb927.6.4620_for	GGGGACAAGTTTGTACAAAAAAGCAGGCTATGGCTGCTCAAGGGCTGT	Tb927.6.4620_rev	GGGGACCACTTTGTACAAGAAAGCTGGGTTCAGGTCTGACTCGTATAGTCGGG
1_hypothetical	J18	Tb927.2.3190_for	GGGGACAAGTTTGTACAAAAAAGCAGGCTATGGAAATTCCCATAAATGTGTCGGA	Tb927.2.3190_rev	GGGGACCACTTTGTACAAGAAAGCTGGGTTCAAAGACCTGTGACGCGTCG
1_hypothetical	J19	Tb927.10.11920_for	GGGGACAAGTTTGTACAAAAAAGCAGGCTATGCAGGCAGTGGGTGAAAC	Tb927.10.11920_rev	GGGGACCACTTTGTACAAGAAAGCTGGGTTTACCCCCCCCCCACTCC
1_hypothetical	J20	Tb927.10.7960_for	GGGGACAAGTTTGTACAAAAAAGCAGGCTATGGCTCCCAAGAAGAAGGTCC	Tb927.10.7960_rev	GGGGACCACTTTGTACAAGAAAGCTGGGTTTACTTTGTTAGGAGCACCTCTCTCA
1_hypothetical	J21	Tb927.6.260_for	GGGGACAAGTTTGTACAAAAAAGCAGGCTATGTGGGGCAAGAGTTTGGATTG	Tb927.6.260_rev	GGGGACCACTTTGTACAAGAAAGCTGGGTCTAACCGGATGAGTGAGTGACGA
1_hypothetical	J22	Tb927.10.3960_for	GGGGACAAGTTTGTACAAAAAAGCAGGCTATGCTGCCGCTGTCGCGT	Tb927.10.3960_rev	GGGGACCACTTTGTACAAGAAAGCTGGGTCTAAGTACATTTTTTCGGATCTCCATTGC
1_hypothetical	J23	Tb927.11.2240_for	GGGGACAAGTTTGTACAAAAAAGCAGGCTATGGTGAGCGAAGTGATGGTTG	Tb927.11.2240_rev	GGGGACCACTTTGTACAAGAAAGCTGGGTTTACACCATAGGGCTTAGAACTCGT
1_hypothetical	J24	Tb927.10.8090_for	GGGGACAAGTTTGTACAAAAAAGCAGGCTATGCAGCGCGGATTTTGTAGG	Tb927.10.8090_rev	GGGGACCACTTTGTACAAGAAAGCTGGGTCTATTTTGTACACGAAAGCACATGTGT
1_hypothetical	K1	Tb927.10.4550_for	GGGGACAAGTTTGTACAAAAAAGCAGGCTATGGGTAGCACACCTCATTATTCTAGG	Tb927.10.4550_rev	GGGGACCACTTTGTACAAGAAAGCTGGGTTCAAGGCTTCTTAACGGTATTCATCAC
1_hypothetical	K2	Tb927.7.370_for	GGGGACAAGTTTGTACAAAAAAGCAGGCTATGCATGATTTGATCGCTGTTGTAGA	Tb927.7.370_rev	GGGGACCACTTTGTACAAGAAAGCTGGGTCTACTGCAAGGGCCGCAG
1_hypothetical	K3	Tb927.1.3350_for	GGGGACAAGTTTGTACAAAAAAGCAGGCTATGACAGATGGCCACGCAAC	Tb927.1.3350_rev	GGGGACCACTTTGTACAAGAAAGCTGGGTTCAGATATTCGCCTTATGGAAGCATAC
1_hypothetical	K4	Tb927.10.12900_for	GGGGACAAGTTTGTACAAAAAAGCAGGCTATGCTGCACTGTTCACAGTGG	Tb927.10.12900_rev	GGGGACCACTTTGTACAAGAAAGCTGGGTCTAGTGTACCAATGAGAACCTCATGTC
1_hypothetical	K5	Tb927.3.2180_for	GGGGACAAGTTTGTACAAAAAAGCAGGCTATGTTGCGGAAAACACCATTGTTC	Tb927.3.2180_rev	GGGGACCACTTTGTACAAGAAAGCTGGGTCTAGAAGAGCTCTGGCCGGC
1_hypothetical	K6	Tb927.8.1790_for	GGGGACAAGTTTGTACAAAAAAGCAGGCTATGTCTCTTTGCGCTGAGATTAACC	Tb927.8.1790_rev	GGGGACCACTTTGTACAAGAAAGCTGGGTTCAGCGGAGCAGGCGACA
1_hypothetical	K7	Tb927.9.1470_for	GGGGACAAGTTTGTACAAAAAAGCAGGCTATGGCACTGCGTGATATTGTAGG	Tb927.9.1470_rev	GGGGACCACTTTGTACAAGAAAGCTGGGTTCAAAGTCTACCTGCACGCTTTG
1_hypothetical	K8	Tb927.9.16950_for	GGGGACAAGTTTGTACAAAAAAGCAGGCTATGTGTTACTCTGATTCGTATCAAGTGTT	Tb927.9.16950_rev	GGGGACCACTTTGTACAAGAAAGCTGGGTTTATAATAAAAGAAGTAGTAATATTAATAG
1_hypothetical	K9	Tb927.3.3620_for	GGGGACAAGTTTGTACAAAAAAGCAGGCTATGTCGACATTCCAACGCCG	Tb927.3.3620_rev	GGGGACCACTTTGTACAAGAAAGCTGGGTTCAGGAGTCTGGCTCTGCG
1_hypothetical	K10	Tb927.11.13300_for	GGGGACAAGTTTGTACAAAAAAGCAGGCTATGGCAAAGGTGAGCATAGACCC	Tb927.11.13300_rev	GGGGACCACTTTGTACAAGAAAGCTGGGTTTACATCACCACTTCAGGATTGGACT
1_hypothetical	K11	Tb927.10.15860_for	GGGGACAAGTTTGTACAAAAAAGCAGGCTATGCCAAACATAAAGGGAGGGGT	Tb927.10.15860_rev	GGGGACCACTTTGTACAAGAAAGCTGGGTCTACGTATCGTTTTCCATTCCACGG
1_hypothetical	K12	Tb927.10.5160_for	GGGGACAAGTTTGTACAAAAAAGCAGGCTATGTGCGCGTGTATGTCAAGG	Tb927.10.5160_rev	GGGGACCACTTTGTACAAGAAAGCTGGGTTCACAAGCAAATATATCCCCCAATGG
1_hypothetical	K13	Tb927.11.490_for	GGGGACAAGTTTGTACAAAAAAGCAGGCTATGGCTCCACCTTCAGATATTTCGG	Tb927.11.490_rev	GGGGACCACTTTGTACAAGAAAGCTGGGTTTACGTTTCAGCACCACATCGG
1_hypothetical	K14	Tb927.10.1020_for	GGGGACAAGTTTGTACAAAAAAGCAGGCTATGCATAGGATGCCGTTCAACTC	Tb927.10.1020_rev	GGGGACCACTTTGTACAAGAAAGCTGGGTTCAGGGAGATTGAAATTGGTGTATGC
1_hypothetical	K15	Tb927.4.1760_for	GGGGACAAGTTTGTACAAAAAAGCAGGCTATGTTGCGGTTGACACGGC	Tb927.4.1760_rev	GGGGACCACTTTGTACAAGAAAGCTGGGTTCACTCACGAGAGAAAATGTTACGTC
1_hypothetical	K16	Tb927.10.130_for	GGGGACAAGTTTGTACAAAAAAGCAGGCTATGGTAATTTTCCAACGGGTTGAAGC	Tb927.10.130_rev	GGGGACCACTTTGTACAAGAAAGCTGGGTTTATGTTAGAAACCGAATGTACTGACCG
1_hypothetical	K17	Tb927.2.2070_for	GGGGACAAGTTTGTACAAAAAAGCAGGCTATGCGGTTGTACAAGTCCTCTTTT	Tb927.2.2070_rev	GGGGACCACTTTGTACAAGAAAGCTGGGTTTATTCATTGAATTGAGTTCGCATAAGGTC
1_hypothetical	K18	Tb927.7.1940_for	GGGGACAAGTTTGTACAAAAAAGCAGGCTATGGACAATATACACCACCGCCC	Tb927.7.1940_rev	GGGGACCACTTTGTACAAGAAAGCTGGGTTCAACAAGTTGCAGTTGGACCTC
1_hypothetical	K19	Tb927.11.15930_for	GGGGACAAGTTTGTACAAAAAAGCAGGCTATGACAGTTCACTTGCTCTGGATCA	Tb927.11.15930_rev	GGGGACCACTTTGTACAAGAAAGCTGGGTTCACCTCTTCACCGCGGG
1_hypothetical	K20	Tb927.10.13420_for	GGGGACAAGTTTGTACAAAAAAGCAGGCTATGAGCGTCGTCACTTCTGAAC	Tb927.10.13420_rev	GGGGACCACTTTGTACAAGAAAGCTGGGTTCATTCCCTTCCGGACACGG
1_hypothetical	K21	Tb927.4.4190_for	GGGGACAAGTTTGTACAAAAAAGCAGGCTATGGGCAAGCTCCAGGTGT	Tb927.4.4190_rev	GGGGACCACTTTGTACAAGAAAGCTGGGTTTAAGCCATGAGAGCAGGAGCT
1_hypothetical	K22	Tb927.10.9830_for	GGGGACAAGTTTGTACAAAAAAGCAGGCTATGCGCCGCACCTTCATT	Tb927.10.9830_rev	GGGGACCACTTTGTACAAGAAAGCTGGGTCTACTTAGCCTTTGCTTTCACATCTTCG
1_hypothetical	K23	Tb927.9.10200_for	GGGGACAAGTTTGTACAAAAAAGCAGGCTATGCAGGGAGAGGGAAATGAATTG	Tb927.9.10200_rev	GGGGACCACTTTGTACAAGAAAGCTGGGTTTACGCATCTTCTTCCTCGTTATCATC
1_hypothetical	K24	Tb927.8.5800_for	GGGGACAAGTTTGTACAAAAAAGCAGGCTATGAAACACAAAGATGCTCGCGG	Tb927.8.5800_rev	GGGGACCACTTTGTACAAGAAAGCTGGGTTCACTTCTTCACTCTTTTTGGCGG
1_hypothetical	L1	Tb927.4.2530_for	GGGGACAAGTTTGTACAAAAAAGCAGGCTATGGGCTTCGGTGCAGTCG	Tb927.4.2530_rev	GGGGACCACTTTGTACAAGAAAGCTGGGTTTAATTCTTCTTTTTTTCTTTCCCCGTCG
1_hypothetical	L2	Tb927.7.3690_for	GGGGACAAGTTTGTACAAAAAAGCAGGCTATGCAGAAACGAAGGGAATGTTTGG	Tb927.7.3690_rev	GGGGACCACTTTGTACAAGAAAGCTGGGTTTAAAGGAAAGGAAAAAAAAAAAAAAGAGAT
1_hypothetical	L3	Tb927.1.4730_for	GGGGACAAGTTTGTACAAAAAAGCAGGCTATGGAAGGACAAAACTCAGTTTCTCTTC	Tb927.1.4730_rev	GGGGACCACTTTGTACAAGAAAGCTGGGTCTACTCATAACAGTGCAGATCATTTGACA
1_hypothetical	L4	Tb927.9.8370_for	GGGGACAAGTTTGTACAAAAAAGCAGGCTATGCAACGCAATTGGGTATTGC	Tb927.9.8370_rev	GGGGACCACTTTGTACAAGAAAGCTGGGTTCAACAATCCAGGTAATCCGACGC
1_hypothetical	L5	Tb927.10.1480_for	GGGGACAAGTTTGTACAAAAAAGCAGGCTATGTTTGTTTTGCAGCTCATGAAGAG	Tb927.10.1480_rev	GGGGACCACTTTGTACAAGAAAGCTGGGTTTACGTGCAATCAACCTGCAGC
1_hypothetical	L6	Tb927.10.13600_for	GGGGACAAGTTTGTACAAAAAAGCAGGCTATGTTTAGTCGGTGCCAGCG	Tb927.10.13600_rev	GGGGACCACTTTGTACAAGAAAGCTGGGTCTACAATGTCTTCTGAGCGGCG
1_hypothetical	L7	Tb927.4.3830_for	GGGGACAAGTTTGTACAAAAAAGCAGGCTATGGAACCGCGTACAGTCCG	Tb927.4.3830_rev	GGGGACCACTTTGTACAAGAAAGCTGGGTTTACGCCGGTTGCGAATAGAG
1_hypothetical	L8	Tb927.5.1630_for	GGGGACAAGTTTGTACAAAAAAGCAGGCTATGTCCCTCATTACTTCACCGGG	Tb927.5.1630_rev	GGGGACCACTTTGTACAAGAAAGCTGGGTTTAGGCTAAATTTTCATCCTTCTTAAAGAA
1_hypothetical	L9	Tb927.8.5550_for	GGGGACAAGTTTGTACAAAAAAGCAGGCTATGGTCTTCTTTGTGAGAGTCACAGG	Tb927.8.5550_rev	GGGGACCACTTTGTACAAGAAAGCTGGGTCTACATTGCATTGTTGTAGTGAACAACA
1_hypothetical	L10	Tb927.11.15540_for	GGGGACAAGTTTGTACAAAAAAGCAGGCTATGTCAAAGCAGAGTAGGAAAGAGGG	Tb927.11.15540_rev	GGGGACCACTTTGTACAAGAAAGCTGGGTCTACTCATGGGAAACATCGGTGTC
1_hypothetical	L11	Tb927.4.2210_for	GGGGACAAGTTTGTACAAAAAAGCAGGCTATGAAGGCGACAATTATAACACCAGG	Tb927.4.2210_rev	GGGGACCACTTTGTACAAGAAAGCTGGGTTCAGCGCCGCCGTGCACC
1_hypothetical	L12	Tb927.10.400_for	GGGGACAAGTTTGTACAAAAAAGCAGGCTATGTTGGAGTCTTTTGAATTTGATCAGGA	Tb927.10.400_rev	GGGGACCACTTTGTACAAGAAAGCTGGGTCTAAATATCTTCACACACTGGGCAAAATC
1_hypothetical	L13	Tb927.5.3740_for	GGGGACAAGTTTGTACAAAAAAGCAGGCTATGGAGGATTCCTCAGTGAGTTCTT	Tb927.5.3740_rev	GGGGACCACTTTGTACAAGAAAGCTGGGTTTAGTAATCATTCTTTGCGGTTTTGGAC
1_hypothetical	L14	Tb927.10.3050_for	GGGGACAAGTTTGTACAAAAAAGCAGGCTATGTTGAAGGGAGATTTATTATTAGTGTGT	Tb927.10.3050_rev	GGGGACCACTTTGTACAAGAAAGCTGGGTTTATACGAAGTTGTGCTTTCGTTTTTTGG
1_hypothetical	L15	Tb927.4.1730_for	GGGGACAAGTTTGTACAAAAAAGCAGGCTATGGACACCGAGTGCCTCC	Tb927.4.1730_rev	GGGGACCACTTTGTACAAGAAAGCTGGGTCTACTTGGTGACCTTCTTGAGCATT
1_hypothetical	L16	Tb927.7.6100_for	GGGGACAAGTTTGTACAAAAAAGCAGGCTATGAGATCCGTGCATCAATCCAAG	Tb927.7.6100_rev	GGGGACCACTTTGTACAAGAAAGCTGGGTTTATTCCTCCTCCACCTCAAGCTC
1_hypothetical	L17	Tb927.7.4350_for	GGGGACAAGTTTGTACAAAAAAGCAGGCTATGAACCCGAGTCTCTCTGACAG	Tb927.7.4350_rev	GGGGACCACTTTGTACAAGAAAGCTGGGTCTAAGTTACTGGAACTGTAGCGGC
1_hypothetical	L18	Tb927.6.3920_for	GGGGACAAGTTTGTACAAAAAAGCAGGCTATGTCGGCCATTCCGCCG	Tb927.6.3920_rev	GGGGACCACTTTGTACAAGAAAGCTGGGTTTAGACGCCTACAAACCCCACG
1_hypothetical	L19	Tb927.11.7880_for	GGGGACAAGTTTGTACAAAAAAGCAGGCTATGGAACTAAACCAACTCGAATTGTTTG	Tb927.11.7880_rev	GGGGACCACTTTGTACAAGAAAGCTGGGTTCAATCACTGATGCTAAAGGTGGC
1_hypothetical	L20	Tb927.7.4780_for	GGGGACAAGTTTGTACAAAAAAGCAGGCTATGAGTAAGGGAGTTGTGCGGT	Tb927.7.4780_rev	GGGGACCACTTTGTACAAGAAAGCTGGGTCTAAGCCCTTGCACGTGCC
1_hypothetical	L21	Tb927.10.2800_for	GGGGACAAGTTTGTACAAAAAAGCAGGCTATGGTTTTTTATTCTTTTGTACTGGTGATG	Tb927.10.2800_rev	GGGGACCACTTTGTACAAGAAAGCTGGGTTCAGTTTCTCAGAAAACTGGACCAAC
1_hypothetical	L22	Tb927.11.10100_for	GGGGACAAGTTTGTACAAAAAAGCAGGCTATGCTTCGACGCACTTCACTG	Tb927.11.10100_rev	GGGGACCACTTTGTACAAGAAAGCTGGGTTTAATCACTCCGCGGTGCAAA
1_hypothetical	L23	Tb927.10.1000_for	GGGGACAAGTTTGTACAAAAAAGCAGGCTATGAAAGCCCGTTCCTCCCG	Tb927.10.1000_rev	GGGGACCACTTTGTACAAGAAAGCTGGGTTCAAAGGAGTGTTGCCGCG
1_hypothetical	L24	Tb927.5.1480_for	GGGGACAAGTTTGTACAAAAAAGCAGGCTATGCAAATGGATGAAGCGAATGAAG	Tb927.5.1480_rev	GGGGACCACTTTGTACAAGAAAGCTGGGTTTACACAGCAGAGGGAAACAGACA
1_hypothetical	M1	Tb927.7.6710_for	GGGGACAAGTTTGTACAAAAAAGCAGGCTATGCGTGTTTGTTTACGGCTTTTC	Tb927.7.6710_rev	GGGGACCACTTTGTACAAGAAAGCTGGGTCTAGGAAAAGAGGGAGAAAAGGAAGGA
1_hypothetical	M2	Tb927.8.720_for	GGGGACAAGTTTGTACAAAAAAGCAGGCTATGGAGGAACTCGTCAATCCCA	Tb927.8.720_rev	GGGGACCACTTTGTACAAGAAAGCTGGGTTCATTCAAAGGCATTTACGCATGATTC
1_hypothetical	M3	Tb927.10.6490_for	GGGGACAAGTTTGTACAAAAAAGCAGGCTATGTCTGTCGCACCAAAACGT	Tb927.10.6490_rev	GGGGACCACTTTGTACAAGAAAGCTGGGTCTACTCACCCACGCCCTTCC
1_hypothetical	M4	Tb927.2.2390_for	GGGGACAAGTTTGTACAAAAAAGCAGGCTATGGTTAATCTGCCGCTGAAGG	Tb927.2.2390_rev	GGGGACCACTTTGTACAAGAAAGCTGGGTCTATCGACGGCCACCCTTACG
1_hypothetical	M5	Tb927.11.7050_for	GGGGACAAGTTTGTACAAAAAAGCAGGCTATGGCGCTGTGGTTGAGAGT	Tb927.11.7050_rev	GGGGACCACTTTGTACAAGAAAGCTGGGTTCAAACGATATACCGAAGCACATCC
1_hypothetical	M6	Tb927.2.3920_for	GGGGACAAGTTTGTACAAAAAAGCAGGCTATGTTTTCTTGTTGCCTGCGTAGC	Tb927.2.3920_rev	GGGGACCACTTTGTACAAGAAAGCTGGGTCTACTTGCGCCTAGAATAAACAGCC
1_hypothetical	M7	Tb927.10.8140_for	GGGGACAAGTTTGTACAAAAAAGCAGGCTATGAATCGTAAAAGGCCGCGG	Tb927.10.8140_rev	GGGGACCACTTTGTACAAGAAAGCTGGGTTTATTTTTTTCGCCCAAGCGATACAAC
1_hypothetical	M8	Tb927.10.9940_for	GGGGACAAGTTTGTACAAAAAAGCAGGCTATGCGGGTACTCACGCACC	Tb927.10.9940_rev	GGGGACCACTTTGTACAAGAAAGCTGGGTCTAGTAGAGATCCCGGACATGGTATG
1_hypothetical	M9	Tb927.11.15700_for	GGGGACAAGTTTGTACAAAAAAGCAGGCTATGTCGACAAGCATCACAAATTTTGT	Tb927.11.15700_rev	GGGGACCACTTTGTACAAGAAAGCTGGGTTTACTGAGGTCGTAAGTCTTCCTCG
1_hypothetical	M10	Tb927.9.3750_for	GGGGACAAGTTTGTACAAAAAAGCAGGCTATGCCTACTCCTCGGGAAACTG	Tb927.9.3750_rev	GGGGACCACTTTGTACAAGAAAGCTGGGTTCACGAAATTTCAAAGAAATTTTGCGGT
1_hypothetical	M11	Tb927.3.3600_for	GGGGACAAGTTTGTACAAAAAAGCAGGCTATGCCCATGCTGGTCCCT	Tb927.3.3600_rev	GGGGACCACTTTGTACAAGAAAGCTGGGTTTATTTCCGGATGAGAAAACTTTCTATCCC
1_hypothetical	M12	Tb927.10.11650_for	GGGGACAAGTTTGTACAAAAAAGCAGGCTATGGGTTGTGGAGACTCGAAAGT	Tb927.10.11650_rev	GGGGACCACTTTGTACAAGAAAGCTGGGTCTACTCAGCGGGGTCATCGT
1_hypothetical	M13	Tb927.6.4790_for	GGGGACAAGTTTGTACAAAAAAGCAGGCTATGGGTGTAGCACTGCGGG	Tb927.6.4790_rev	GGGGACCACTTTGTACAAGAAAGCTGGGTTCAAAGGGTTGCACGGTACG
1_hypothetical	M14	Tb927.3.3570_for	GGGGACAAGTTTGTACAAAAAAGCAGGCTATGACTACTAGTAACGGCTTCGAATCG	Tb927.3.3570_rev	GGGGACCACTTTGTACAAGAAAGCTGGGTTCACTGCGATGACCCCTGC
1_hypothetical	M15	Tb927.1.3390_for	GGGGACAAGTTTGTACAAAAAAGCAGGCTATGCCACTTTACCGCCTTTCG	Tb927.1.3390_rev	GGGGACCACTTTGTACAAGAAAGCTGGGTCTATTTGGATTTCTTCAGACGACTGAATTC
1_hypothetical	M16	Tb927.9.6910_for	GGGGACAAGTTTGTACAAAAAAGCAGGCTATGGCTCAGTGGGTTCCTAAAACG	Tb927.9.6910_rev	GGGGACCACTTTGTACAAGAAAGCTGGGTTTAGTCCTCACCAGAAACCGCT
1_hypothetical	M17	Tb04.24M18.150_for	GGGGACAAGTTTGTACAAAAAAGCAGGCTATGTGGCATGCTGATAATGCCA	Tb04.24M18.150_rev	GGGGACCACTTTGTACAAGAAAGCTGGGTTTACTTAGGCAATTGAGAGAACGCG
1_hypothetical	M18	Tb927.11.14670_for	GGGGACAAGTTTGTACAAAAAAGCAGGCTATGAAATGGCTCCTTAGGCTCGT	Tb927.11.14670_rev	GGGGACCACTTTGTACAAGAAAGCTGGGTCTATTTTTTCCTTTCCTGGGCATCGG
1_hypothetical	M19	Tb927.10.4400_for	GGGGACAAGTTTGTACAAAAAAGCAGGCTATGAGTTTCTTCGGCAAGTTTATGAAAAA	Tb927.10.4400_rev	GGGGACCACTTTGTACAAGAAAGCTGGGTTCAAAGTGGAATGTTGTGTAGATTTGGG
1_hypothetical	M20	Tb927.2.3330_for	GGGGACAAGTTTGTACAAAAAAGCAGGCTATGTGGAGTCGCCTTTTGGGT	Tb927.2.3330_rev	GGGGACCACTTTGTACAAGAAAGCTGGGTTCAAGGAGTAGTAACGATTTGGCTTC
1_hypothetical	M21	Tb927.6.3950_for	GGGGACAAGTTTGTACAAAAAAGCAGGCTATGGAGGATCCACGGGACAATC	Tb927.6.3950_rev	GGGGACCACTTTGTACAAGAAAGCTGGGTCTAACGGCGATATTCCTCGTCAC
1_hypothetical	M22	Tb927.7.3240_for	GGGGACAAGTTTGTACAAAAAAGCAGGCTATGCTGCGCTCAACTCTGAC	Tb927.7.3240_rev	GGGGACCACTTTGTACAAGAAAGCTGGGTCTATTTCCTCCGCTCGTAAAGTTCC
1_hypothetical	M23	Tb927.11.4020_for	GGGGACAAGTTTGTACAAAAAAGCAGGCTATGCGAGCGAGGCCCTCT	Tb927.11.4020_rev	GGGGACCACTTTGTACAAGAAAGCTGGGTTTACAAGTGAAACCCCGGTTCG
1_hypothetical	M24	Tb927.11.9410_for	GGGGACAAGTTTGTACAAAAAAGCAGGCTATGCATTTACAAATAAATAAATACATATAT	Tb927.11.9410_rev	GGGGACCACTTTGTACAAGAAAGCTGGGTTCATTCTTCTTTCTGTTTGTATGTTTTGTT
1_hypothetical	N1	Tb927.6.3360_for	GGGGACAAGTTTGTACAAAAAAGCAGGCTATGATATCATTGCGGGGAAACATAGG	Tb927.6.3360_rev	GGGGACCACTTTGTACAAGAAAGCTGGGTTCAAAAGTCCAGCACTTCATCTAGTG
1_hypothetical	N2	Tb927.9.17730_for	GGGGACAAGTTTGTACAAAAAAGCAGGCTATGACAACGCTGCATGCGG	Tb927.9.17730_rev	GGGGACCACTTTGTACAAGAAAGCTGGGTTCAGTGCTGCGGGTCGCT
1_hypothetical	N3	Tb927.6.2680_for	GGGGACAAGTTTGTACAAAAAAGCAGGCTATGTACCGCACTCTCACACCG	Tb927.6.2680_rev	GGGGACCACTTTGTACAAGAAAGCTGGGTTCACAAATAACTATCCAAACCACGATAATA
1_hypothetical	N4	Tb927.8.2180_for	GGGGACAAGTTTGTACAAAAAAGCAGGCTATGCGACGCCGTGGGTCC	Tb927.8.2180_rev	GGGGACCACTTTGTACAAGAAAGCTGGGTTTAATGTCCATATCTCACCATCAGCAAAA
1_hypothetical	N5	Tb927.10.1760_for	GGGGACAAGTTTGTACAAAAAAGCAGGCTATGAACCGAAGAGTAGATTTCATATGCC	Tb927.10.1760_rev	GGGGACCACTTTGTACAAGAAAGCTGGGTCTACAGGGTGCAGAGGCGG
1_hypothetical	N6	Tb927.9.15820_for	GGGGACAAGTTTGTACAAAAAAGCAGGCTATGAATTGCATCCAGCCATCTGG	Tb927.9.15820_rev	GGGGACCACTTTGTACAAGAAAGCTGGGTTTAAAAAACCGACTTTCTGACGGCA
1_hypothetical	N7	Tb927.9.9260_for	GGGGACAAGTTTGTACAAAAAAGCAGGCTATGTCCGATGACGATGATGATGTG	Tb927.9.9260_rev	GGGGACCACTTTGTACAAGAAAGCTGGGTTTACTCGAGAACTGCTGTGTTGATAAG
1_hypothetical	N8	Tb927.11.4790_for	GGGGACAAGTTTGTACAAAAAAGCAGGCTATGCGCTTGAAAACTGCCGT	Tb927.11.4790_rev	GGGGACCACTTTGTACAAGAAAGCTGGGTTCAAGTGACCTCGAATAACACCCT
1_hypothetical	N9	Tb927.10.1010_for	GGGGACAAGTTTGTACAAAAAAGCAGGCTATGTATGAAACAGGAAGAGTAGCCACC	Tb927.10.1010_rev	GGGGACCACTTTGTACAAGAAAGCTGGGTTTAAGGGCCCTGCTTTCGCA
1_hypothetical	N10	Tb927.3.4050_for	GGGGACAAGTTTGTACAAAAAAGCAGGCTATGTTTTCGAGCTATGCAGGTTGC	Tb927.3.4050_rev	GGGGACCACTTTGTACAAGAAAGCTGGGTTCAACCACTGCCGACCGA
1_hypothetical	N11	Tb927.10.9610_for	GGGGACAAGTTTGTACAAAAAAGCAGGCTATGCAGGACGAAAGGGAAACAAG	Tb927.10.9610_rev	GGGGACCACTTTGTACAAGAAAGCTGGGTCTATTTAAATGCAAGTCGTGCTGTGTT
1_hypothetical	N12	Tb927.9.13480_for	GGGGACAAGTTTGTACAAAAAAGCAGGCTATGAAGAAGGCAAAAAAACAAGAGCC	Tb927.9.13480_rev	GGGGACCACTTTGTACAAGAAAGCTGGGTTTAAAAACAGCAGTCACAATCAAAGGG
1_hypothetical	N13	Tb927.4.3080_for	GGGGACAAGTTTGTACAAAAAAGCAGGCTATGAAGGTATCGTCGTTTATTGTTCAGC	Tb927.4.3080_rev	GGGGACCACTTTGTACAAGAAAGCTGGGTTCATGAAAAATCGGCAGACTTGAGC
1_hypothetical	N14	Tb927.11.8020_for	GGGGACAAGTTTGTACAAAAAAGCAGGCTATGGCACTTGAAGGGTCAACAC	Tb927.11.8020_rev	GGGGACCACTTTGTACAAGAAAGCTGGGTCTACACGTGCCTCAGTTCATTCA
1_hypothetical	N15	Tb927.8.5960_for	GGGGACAAGTTTGTACAAAAAAGCAGGCTATGCCCCCAAAGGATAACAGAAAAG	Tb927.8.5960_rev	GGGGACCACTTTGTACAAGAAAGCTGGGTCTACTGGGTCGAATCTCCAGATTTTG
1_hypothetical	N16	Tb927.9.13240_for	GGGGACAAGTTTGTACAAAAAAGCAGGCTATGAGTTCTGATGAGTTAGATGCTTTCC	Tb927.9.13240_rev	GGGGACCACTTTGTACAAGAAAGCTGGGTTTAACTTCGAAGCCTTGGTCGC
1_hypothetical	N17	Tb927.4.1130_for	GGGGACAAGTTTGTACAAAAAAGCAGGCTATGTATAGCAAGACACCGTGTGTTT	Tb927.4.1130_rev	GGGGACCACTTTGTACAAGAAAGCTGGGTTCATTTACTAAAGTCCTCAGCGTCAGC
1_hypothetical	N18	Tb927.11.14050_for	GGGGACAAGTTTGTACAAAAAAGCAGGCTATGGGTTTAGAAATATTTGAGATTGACACC	Tb927.11.14050_rev	GGGGACCACTTTGTACAAGAAAGCTGGGTTCAGACAAATGCCAACTTCTGATTCA
1_hypothetical	N19	Tb927.7.7060_for	GGGGACAAGTTTGTACAAAAAAGCAGGCTATGAGCGCTTTTGTAGATGTAGTTAAGA	Tb927.7.7060_rev	GGGGACCACTTTGTACAAGAAAGCTGGGTTTACACCACACCTCTCAAGCAAC
1_hypothetical	N20	Tb927.4.1040_for	GGGGACAAGTTTGTACAAAAAAGCAGGCTATGGAACAAGATTCACATCCCAGGA	Tb927.4.1040_rev	GGGGACCACTTTGTACAAGAAAGCTGGGTTTAGACAAGGTCGTATACCTTGTTCACT
1_hypothetical	N21	Tb927.8.6010_for	GGGGACAAGTTTGTACAAAAAAGCAGGCTATGGCTCTTTCAGAGAAAGCGTG	Tb927.8.6010_rev	GGGGACCACTTTGTACAAGAAAGCTGGGTCTACAACATGCTCGTCTGAGGGC
1_hypothetical	N22	Tb927.8.1430_for	GGGGACAAGTTTGTACAAAAAAGCAGGCTATGTTCTGCTTCAGTAGAGGATTGTG	Tb927.8.1430_rev	GGGGACCACTTTGTACAAGAAAGCTGGGTCTAAATCCGACTATACTGAAGAACGTTGC
1_hypothetical	N23	Tb927.8.3780_for	GGGGACAAGTTTGTACAAAAAAGCAGGCTATGCCACCCAAAAAGAAGGTGG	Tb927.8.3780_rev	GGGGACCACTTTGTACAAGAAAGCTGGGTCTAACCCTCTTTTTCATCCTTCAGTACAT
1_hypothetical	N24	Tb927.1.4160_for	GGGGACAAGTTTGTACAAAAAAGCAGGCTATGCAGATTTATGTGAAGCATCGCG	Tb927.1.4160_rev	GGGGACCACTTTGTACAAGAAAGCTGGGTTTATTTATTAACTTCCACCGCTTTCTGTTG
1_hypothetical	O1	Tb927.2.2570_for	GGGGACAAGTTTGTACAAAAAAGCAGGCTATGTCTCCGCGTACCTCTGAA	Tb927.2.2570_rev	GGGGACCACTTTGTACAAGAAAGCTGGGTTTATTCGTCATCGTCATCAGCAGAG
1_hypothetical	O2	Tb927.9.17940_for	GGGGACAAGTTTGTACAAAAAAGCAGGCTATGACATGTAGGAGTTCTCACACCT	Tb927.9.17940_rev	GGGGACCACTTTGTACAAGAAAGCTGGGTTCATTCAGATCCAAAGCCGATAAATATAAG
1_hypothetical	O3	Tb927.11.13390_for	GGGGACAAGTTTGTACAAAAAAGCAGGCTATGAGTCAGGAGGCAGGATGC	Tb927.11.13390_rev	GGGGACCACTTTGTACAAGAAAGCTGGGTCTATTGCGATTGGAGTTTATCGACTGC
1_hypothetical	O4	Tb927.10.11900_for	GGGGACAAGTTTGTACAAAAAAGCAGGCTATGACCCAAAACATTATTCGTCGCA	Tb927.10.11900_rev	GGGGACCACTTTGTACAAGAAAGCTGGGTCTAACTCTCTGTATTCAGAGCAGCCT
1_hypothetical	O5	Tb927.9.13530_for	GGGGACAAGTTTGTACAAAAAAGCAGGCTATGAGGCGGATCATGTCCCC	Tb927.9.13530_rev	GGGGACCACTTTGTACAAGAAAGCTGGGTTTACCACATCGCGTCATTTCCC
1_hypothetical	O6	Tb927.6.4490_for	GGGGACAAGTTTGTACAAAAAAGCAGGCTATGGCGCCCAGCAGTGAA	Tb927.6.4490_rev	GGGGACCACTTTGTACAAGAAAGCTGGGTTTAGTTCAGAAATAGCTTTCTTGACTCACT
1_hypothetical	O7	Tb927.8.4650_for	GGGGACAAGTTTGTACAAAAAAGCAGGCTATGCAAAGGTGTGTCCACATACTC	Tb927.8.4650_rev	GGGGACCACTTTGTACAAGAAAGCTGGGTTTACACAGCGGGCTTGAACG
1_hypothetical	O8	Tb927.9.10560_for	GGGGACAAGTTTGTACAAAAAAGCAGGCTATGGATAGCCTTGCACACAGTG	Tb927.9.10560_rev	GGGGACCACTTTGTACAAGAAAGCTGGGTTTAGTTGGAAGTACGGCTTTCATGTAAG
1_hypothetical	O9	Tb927.11.1440_for	GGGGACAAGTTTGTACAAAAAAGCAGGCTATGATGTGCAATTCCTTTGTTGCC	Tb927.11.1440_rev	GGGGACCACTTTGTACAAGAAAGCTGGGTTTACAAAGTTCCTCCGTCTCCATATTG
1_hypothetical	O10	Tb927.10.5720_for	GGGGACAAGTTTGTACAAAAAAGCAGGCTATGGGGAATTTTGGGGGTACACC	Tb927.10.5720_rev	GGGGACCACTTTGTACAAGAAAGCTGGGTCTAGGGTGTATTTGCATATTTCCTGCT
1_hypothetical	O11	Tb927.9.10230_for	GGGGACAAGTTTGTACAAAAAAGCAGGCTATGCACATTCCAACCAAGTGGG	Tb927.9.10230_rev	GGGGACCACTTTGTACAAGAAAGCTGGGTTTATTCCTCCAAATCCTTCAAGTCCG
1_hypothetical	O12	Tb927.11.15390_for	GGGGACAAGTTTGTACAAAAAAGCAGGCTATGAAGGCGTTAAAGACAAATATTACCGT	Tb927.11.15390_rev	GGGGACCACTTTGTACAAGAAAGCTGGGTTTAAGCATAAATTTCACGACAAAATTTCAT
1_hypothetical	O13	Tb927.1.2750_for	GGGGACAAGTTTGTACAAAAAAGCAGGCTATGGAGTCAGGGAAGAATGCTCA	Tb927.1.2750_rev	GGGGACCACTTTGTACAAGAAAGCTGGGTTTAGCCCCCAAAGCGGCT
1_hypothetical	O14	Tb927.9.3340_for	GGGGACAAGTTTGTACAAAAAAGCAGGCTATGTCGTACTACGACAAGTCTGACA	Tb927.9.3340_rev	GGGGACCACTTTGTACAAGAAAGCTGGGTTTAGCTGAGCTTTCGCTCGTC
1_hypothetical	O15	Tb927.11.11130_for	GGGGACAAGTTTGTACAAAAAAGCAGGCTATGTTCCACAGGGCATTCGTC	Tb927.11.11130_rev	GGGGACCACTTTGTACAAGAAAGCTGGGTTTAATCGTTGTGCCCATGTGTGT
1_hypothetical	O16	Tb927.10.8980_for	GGGGACAAGTTTGTACAAAAAAGCAGGCTATGTTGCGCTTTACACGTTTTGTT	Tb927.10.8980_rev	GGGGACCACTTTGTACAAGAAAGCTGGGTTTAAGCACTCGCCTTACCCATCG
1_hypothetical	O17	Tb927.9.9230_for	GGGGACAAGTTTGTACAAAAAAGCAGGCTATGCAGGAACTTGCGGTTTTTAAAC	Tb927.9.9230_rev	GGGGACCACTTTGTACAAGAAAGCTGGGTTCAGTTTAGGAGAACGCGGAGC
1_hypothetical	O18	Tb05.30F7.410_for	GGGGACAAGTTTGTACAAAAAAGCAGGCTATGCGGGAAAATGCAACTGCA	Tb05.30F7.410_rev	GGGGACCACTTTGTACAAGAAAGCTGGGTTCATAATACACAAATGCCTACGGTACTTC
1_hypothetical	O19	Tb927.1.1650_for	GGGGACAAGTTTGTACAAAAAAGCAGGCTATGTGGCGGGTGAAACCCT	Tb927.1.1650_rev	GGGGACCACTTTGTACAAGAAAGCTGGGTTCACTCCAAAGCGATGACTCGA
1_hypothetical	O20	Tb927.9.8710_for	GGGGACAAGTTTGTACAAAAAAGCAGGCTATGTCAGTGCTGAGGTTCTGGC	Tb927.9.8710_rev	GGGGACCACTTTGTACAAGAAAGCTGGGTCTAAGAATCATTCCTCTGATATCTGGGACT
1_hypothetical	O21	Tb927.11.1270_for	GGGGACAAGTTTGTACAAAAAAGCAGGCTATGACAAAATATGAGCTGAAGATGCAGT	Tb927.11.1270_rev	GGGGACCACTTTGTACAAGAAAGCTGGGTCTAATAATTGTAGATGGCCTGCTGAGAA
1_hypothetical	O22	Tb927.4.3710_for	GGGGACAAGTTTGTACAAAAAAGCAGGCTATGAGAGTGAACAGGACGCAGC	Tb927.4.3710_rev	GGGGACCACTTTGTACAAGAAAGCTGGGTTCAGTCACCTTTTCCATGTTTTATGTCG
1_hypothetical	O23	Tb927.5.4450_for	GGGGACAAGTTTGTACAAAAAAGCAGGCTATGTCAATGAGCAACGGAAGTAGTG	Tb927.5.4450_rev	GGGGACCACTTTGTACAAGAAAGCTGGGTTCACTTTACAGCAGTTTTTTCATACAGC
1_hypothetical	O24	Tb927.7.6400_for	GGGGACAAGTTTGTACAAAAAAGCAGGCTATGCCAGTTGCTAATGAAGCTTCC	Tb927.7.6400_rev	GGGGACCACTTTGTACAAGAAAGCTGGGTTTATATGAAAATTTGCGGGTAAGCACCT

1_hypothetical	P1	Tb927.10.12360_for	GGGGACAAGTTTGTACAAAAAAGCAGGCTATGAAAGCAACAAAAGCAAAATCCGG	Tb927.10.12360_rev	GGGGACCACTTTGTACAAGAAAGCTGGGTTCAAATGTTGTTGGTATCGGAAAGCA
1_hypothetical	P2	Tb927.8.5350_for	GGGGACAAGTTTGTACAAAAAAGCAGGCTATGGAAAAGCCACTGGATAACGG	Tb927.8.5350_rev	GGGGACCACTTTGTACAAGAAAGCTGGGTTCAGCCCCCGTGCGCATA
1_hypothetical	P3	Tb927.8.3160_for	GGGGACAAGTTTGTACAAAAAAGCAGGCTATGTTCCGCATCTCATTCACTTCC	Tb927.8.3160_rev	GGGGACCACTTTGTACAAGAAAGCTGGGTCTACAACAGACCTTTCAGTGGGTTG
1_hypothetical	P4	Tb927.7.5795_for	GGGGACAAGTTTGTACAAAAAAGCAGGCTATGGCATATAGCGACTATAACAAACAGG	Tb927.7.5795_rev	GGGGACCACTTTGTACAAGAAAGCTGGGTTCATCTTTTTCGCATTGTAATGACTGGA
1_hypothetical	P5	Tb927.4.3730_for	GGGGACAAGTTTGTACAAAAAAGCAGGCTATGTTTACAGGACGTTGGGTTTACAC	Tb927.4.3730_rev	GGGGACCACTTTGTACAAGAAAGCTGGGTCTAAGTGAGACACTTGCCTCCCA
1_hypothetical	P6	Tb927.11.11170_for	GGGGACAAGTTTGTACAAAAAAGCAGGCTATGAACCTAGAGCTGTATGTAACACTCG	Tb927.11.11170_rev	GGGGACCACTTTGTACAAGAAAGCTGGGTTCAGTCGTTGTTTGTGCGTGG
1_hypothetical	P7	Tb927.10.900_for	GGGGACAAGTTTGTACAAAAAAGCAGGCTATGCGTCGAGGGCACCAC	Tb927.10.900_rev	GGGGACCACTTTGTACAAGAAAGCTGGGTTCATCCACCTTTGATCGTGTTGC
1_hypothetical	P8	Tb927.10.9030_for	GGGGACAAGTTTGTACAAAAAAGCAGGCTATGAAGCGATTTTTTGACAAGCTATACC	Tb927.10.9030_rev	GGGGACCACTTTGTACAAGAAAGCTGGGTTTACTTCGTTCCAGAAGCAGAATCATT
1_hypothetical	P9	Tb927.7.6330_for	GGGGACAAGTTTGTACAAAAAAGCAGGCTATGGTGCCCTTATTTAAGACACCAAA	Tb927.7.6330_rev	GGGGACCACTTTGTACAAGAAAGCTGGGTTCAACGTAAGCATAGTGGAAATAATAACAA
1_hypothetical	P10	Tb927.10.2160_for	GGGGACAAGTTTGTACAAAAAAGCAGGCTATGGATGCGGAAGAGTACCTGC	Tb927.10.2160_rev	GGGGACCACTTTGTACAAGAAAGCTGGGTTCAATGAACTTGATCCGCACAGATG
1_hypothetical	P11	Tb927.9.12330_for	GGGGACAAGTTTGTACAAAAAAGCAGGCTATGCCACCAAAGCGTGCTG	Tb927.9.12330_rev	GGGGACCACTTTGTACAAGAAAGCTGGGTTCATTCAACTGCTGCCGGTG
1_hypothetical	P12	Tb927.7.1010_for	GGGGACAAGTTTGTACAAAAAAGCAGGCTATGCCACCGAATTCGGCTAC	Tb927.7.1010_rev	GGGGACCACTTTGTACAAGAAAGCTGGGTTCACTCCTCCGATCGGAGGG
1_hypothetical	P13	Tb927.4.2090_for	GGGGACAAGTTTGTACAAAAAAGCAGGCTATGTTACTGCCACACCCCTCG	Tb927.4.2090_rev	GGGGACCACTTTGTACAAGAAAGCTGGGTTCAAATACTGGAGGTCCCCCCC
1_hypothetical	P14	Tb927.6.2180_for	GGGGACAAGTTTGTACAAAAAAGCAGGCTATGTTACGTCGCTGCCCCT	Tb927.6.2180_rev	GGGGACCACTTTGTACAAGAAAGCTGGGTTTACTTGGGCAGCAAACCGG
1_hypothetical	P15	Tb927.8.3210_for	GGGGACAAGTTTGTACAAAAAAGCAGGCTATGACGGACACCACCAAAGGT	Tb927.8.3210_rev	GGGGACCACTTTGTACAAGAAAGCTGGGTCTACAAACGTGGGCGTTTCCT
1_hypothetical	P16	Tb927.3.5300_for	GGGGACAAGTTTGTACAAAAAAGCAGGCTATGTTATTCACCCGCTGTCTGC	Tb927.3.5300_rev	GGGGACCACTTTGTACAAGAAAGCTGGGTCTACCATTTCGGCGATGATGGC
1_hypothetical	P17	Tb927.9.910_for	GGGGACAAGTTTGTACAAAAAAGCAGGCTATGAAGCTTGCAATAGCGTTAGCA	Tb927.9.910_rev	GGGGACCACTTTGTACAAGAAAGCTGGGTTTATAGGCCGCCCGCCGT
1_hypothetical	P18	Tb927.2.5070_for	GGGGACAAGTTTGTACAAAAAAGCAGGCTATGTCGGGTGACGGTAGTGAT	Tb927.2.5070_rev	GGGGACCACTTTGTACAAGAAAGCTGGGTTCATAGACTGAATCGAGCAATCGACA
1_hypothetical	P19	Tb927.7.430_for	GGGGACAAGTTTGTACAAAAAAGCAGGCTATGCATGATTTGGTCGCTGTTGT	Tb927.7.430_rev	GGGGACCACTTTGTACAAGAAAGCTGGGTCTAACTGCAGCCAGAATGCTTTTC
1_hypothetical	P21	Tb927.10.1650_for	GGGGACAAGTTTGTACAAAAAAGCAGGCTATGCTATCGAAAATGAAATGCACTCTTAC	Tb927.10.1650_rev	GGGGACCACTTTGTACAAGAAAGCTGGGTTCAAACGAAGAAGTGAGAAAAGGGATT
1_hypothetical	P22	Tb927.7.6870_for	GGGGACAAGTTTGTACAAAAAAGCAGGCTATGTTGATTGGCATGAAATATTCTGCTC	Tb927.7.6870_rev	GGGGACCACTTTGTACAAGAAAGCTGGGTTCAAAAGGGATTTGGTTCTTTCTTTTCG
1_hypothetical	P23	Tb927.11.10980_for	GGGGACAAGTTTGTACAAAAAAGCAGGCTATGTCGAAGTACGTGAATTTAGACTCTC	Tb927.11.10980_rev	GGGGACCACTTTGTACAAGAAAGCTGGGTTCACGTAAAACGTGAAAGCACCT
1_hypothetical	P24	Tb927.10.12730_for	GGGGACAAGTTTGTACAAAAAAGCAGGCTATGACGAGTAACCGGATTTACTGTTTG	Tb927.10.12730_rev	GGGGACCACTTTGTACAAGAAAGCTGGGTTTAATGTCGACTGTAACAAATAAAATAAAA
1_known	A1	Tb927.7.570_for	GGGGACAAGTTTGTACAAAAAAGCAGGCTATGGAAAAGTACGAATTTCCCGAGG	Tb927.7.570_rev	GGGGACCACTTTGTACAAGAAAGCTGGGTTTATTTCTCTGAGGTTTCCTTCTTGTTATC
1_known	A2	Tb927.5.3040_for	GGGGACAAGTTTGTACAAAAAAGCAGGCTATGCTACGTCGAACGCGTTT	Tb927.5.3040_rev	GGGGACCACTTTGTACAAGAAAGCTGGGTTTAGGTGTGCGTCGGCACT
1_known	A3	Tb927.3.5090_for	GGGGACAAGTTTGTACAAAAAAGCAGGCTATGCCCTCCGCTGAAACACT	Tb927.3.5090_rev	GGGGACCACTTTGTACAAGAAAGCTGGGTTCAACTCCACGACTGCCAAAATT
1_known	A4	Tb927.2.2460_for	GGGGACAAGTTTGTACAAAAAAGCAGGCTATGTCCGGGCTATCTTTAGCTCA	Tb927.2.2460_rev	GGGGACCACTTTGTACAAGAAAGCTGGGTTTATACCCGCTTCCGGTGGT
1_known	A5	Tb927.8.6510_for	GGGGACAAGTTTGTACAAAAAAGCAGGCTATGCTCTCACGCATCAAGAGGC	Tb927.8.6510_rev	GGGGACCACTTTGTACAAGAAAGCTGGGTTCAGTCGATATAATGCCCACTCATCC
1_known	A6	Tb927.11.13750_for	GGGGACAAGTTTGTACAAAAAAGCAGGCTATGTCAGAGAGATATCAACAGTTAATGAAG	Tb927.11.13750_rev	GGGGACCACTTTGTACAAGAAAGCTGGGTCTAACAAGCACACGAAAACCGTTT
1_known	A7	Tb927.11.15910_for	GGGGACAAGTTTGTACAAAAAAGCAGGCTATGACCTTCAGCATTCCACCACT	Tb927.11.15910_rev	GGGGACCACTTTGTACAAGAAAGCTGGGTCTAGCTTTTCAGCAGCTGCTCC
1_known	A8	Tb927.7.4770_for	GGGGACAAGTTTGTACAAAAAAGCAGGCTATGCTTTTTTTTGACATTTCAGCGAAAAC	Tb927.7.4770_rev	GGGGACCACTTTGTACAAGAAAGCTGGGTTTACAAGACACCGCAGTCGGA
1_known	A9	Tb927.7.3750_for	GGGGACAAGTTTGTACAAAAAAGCAGGCTATGAGACCATTGCTGGTTTTTGGA	Tb927.7.3750_rev	GGGGACCACTTTGTACAAGAAAGCTGGGTTCACTGTTGGCCCCATGGG
1_known	A10	Tb927.1.1990_for	GGGGACAAGTTTGTACAAAAAAGCAGGCTATGAATAATGAGCGACCGGCG	Tb927.1.1990_rev	GGGGACCACTTTGTACAAGAAAGCTGGGTTCACAGGAGCGGAGAAAAGTTAAC
1_known	A11	Tb927.11.15030_for	GGGGACAAGTTTGTACAAAAAAGCAGGCTATGTCCTTAAAAGTGAAGAAACTAATTGAC	Tb927.11.15030_rev	GGGGACCACTTTGTACAAGAAAGCTGGGTTTATTCTTTGATGAAGCCAAGTACCTGC
1_known	A12	Tb927.9.5840_for	GGGGACAAGTTTGTACAAAAAAGCAGGCTATGTCCTGCGGTGATGCGA	Tb927.9.5840_rev	GGGGACCACTTTGTACAAGAAAGCTGGGTTTAGTTCATGCTGCTGAAGTAATCTTGG
1_known	A13	Tb927.10.13370_for	GGGGACAAGTTTGTACAAAAAAGCAGGCTATGTCTGATGAGACGCCTCAGG	Tb927.10.13370_rev	GGGGACCACTTTGTACAAGAAAGCTGGGTTTAAATCACGCTCCAATAAACACTTACCA
1_known	A14	Tb927.10.10670_for	GGGGACAAGTTTGTACAAAAAAGCAGGCTATGCTGAAGTTTCGCGAGCG	Tb927.10.10670_rev	GGGGACCACTTTGTACAAGAAAGCTGGGTTCAGTTGGTGCGAGCTGC
1_known	A15	Tb927.11.7480_for	GGGGACAAGTTTGTACAAAAAAGCAGGCTATGAAATACTCTTTTATAGGTTTACTCTCC	Tb927.11.7480_rev	GGGGACCACTTTGTACAAGAAAGCTGGGTTCATGAAGAGTCACAGAGTCCCG
1_known	A17	Tb927.1.650_for	GGGGACAAGTTTGTACAAAAAAGCAGGCTATGGACGAGATCAAGTGGCCC	Tb927.1.650_rev	GGGGACCACTTTGTACAAGAAAGCTGGGTTTATCGGAGGGGTATGTATTCCGATG
1_known	A18	Tb927.6.1820_for	GGGGACAAGTTTGTACAAAAAAGCAGGCTATGCACGCCAACTGGATTACTTG	Tb927.6.1820_rev	GGGGACCACTTTGTACAAGAAAGCTGGGTTCATAATCGGACATACGTCACCCA
1_known	A19	Tb927.9.8450_for	GGGGACAAGTTTGTACAAAAAAGCAGGCTATGACATCCACCGAGAAGCCG	Tb927.9.8450_rev	GGGGACCACTTTGTACAAGAAAGCTGGGTTCATTTCTTCAGTAGTTCCTCCCCC
1_known	A20	Tb927.11.530_for	GGGGACAAGTTTGTACAAAAAAGCAGGCTATGGAACCGGAGTGTGAAAGC	Tb927.11.530_rev	GGGGACCACTTTGTACAAGAAAGCTGGGTTTAATTCTGCTGCTGCTGCTGC
1_known	A21	Tb927.3.4240_for	GGGGACAAGTTTGTACAAAAAAGCAGGCTATGTCTGCTACCGTGGGCC	Tb927.3.4240_rev	GGGGACCACTTTGTACAAGAAAGCTGGGTTCAATTTGTCGCCCCACCCT
1_known	A22	Tb927.8.1680_for	GGGGACAAGTTTGTACAAAAAAGCAGGCTATGATGGCGAGTGCCCCC	Tb927.8.1680_rev	GGGGACCACTTTGTACAAGAAAGCTGGGTTCAACTGGGGTCACCAATGAGT
1_known	A23	Tb927.10.5840_for	GGGGACAAGTTTGTACAAAAAAGCAGGCTATGTCATCGCTAAAGGAAATCAACGG	Tb927.10.5840_rev	GGGGACCACTTTGTACAAGAAAGCTGGGTTTAGATCTTGTTCCAAGCAACAATATCCA
1_known	A24	Tb927.3.1750_for	GGGGACAAGTTTGTACAAAAAAGCAGGCTATGGCAGAGAGGTTACAAACCATTAC	Tb927.3.1750_rev	GGGGACCACTTTGTACAAGAAAGCTGGGTCTATGAACCATCTCTATAAGCCACGGA
1_known	B1	Tb927.11.1320_for	GGGGACAAGTTTGTACAAAAAAGCAGGCTATGCTTCGTCGCACGTCG	Tb927.11.1320_rev	GGGGACCACTTTGTACAAGAAAGCTGGGTTTAATCTCGAACAGAATACTTTTGAATTTC
1_known	B2	Tb927.8.7010_for	GGGGACAAGTTTGTACAAAAAAGCAGGCTATGATGCGCCGTGCCTGT	Tb927.8.7010_rev	GGGGACCACTTTGTACAAGAAAGCTGGGTCTAAGACGACGAACCATAACTATAAAAAGA
1_known	B3	Tb927.6.490_for	GGGGACAAGTTTGTACAAAAAAGCAGGCTATGTTCCGGAAGTCTTCGGAGT	Tb927.6.490_rev	GGGGACCACTTTGTACAAGAAAGCTGGGTTTACACAAATCTAAAGGCCAAGCCA
1_known	B5	Tb927.7.5460_for	GGGGACAAGTTTGTACAAAAAAGCAGGCTATGCAATTCGATGAAGATATTGTGCCC	Tb927.7.5460_rev	GGGGACCACTTTGTACAAGAAAGCTGGGTTCAGGAGTTGTTAGTTCCCGTAACG
1_known	B6	Tb927.9.12150_for	GGGGACAAGTTTGTACAAAAAAGCAGGCTATGCTCAACAGGGACGGTACC	Tb927.9.12150_rev	GGGGACCACTTTGTACAAGAAAGCTGGGTTCAGTCACGTATTCTCCTTTCTCGG
1_known	B8	Tb927.10.12330_for	GGGGACAAGTTTGTACAAAAAAGCAGGCTATGCAACCATTTGGTCAAACGC	Tb927.10.12330_rev	GGGGACCACTTTGTACAAGAAAGCTGGGTTTAGGGGGATTCGGCAGCC
1_known	B9	Tb927.11.4570_for	GGGGACAAGTTTGTACAAAAAAGCAGGCTATGTCTGTGAAGACGCTTGCC	Tb927.11.4570_rev	GGGGACCACTTTGTACAAGAAAGCTGGGTTCAACCACAGCAACCGGAAC
1_known	B10	Tb927.9.16190_for	GGGGACAAGTTTGTACAAAAAAGCAGGCTATGATTAGTAGAAAAACATATTTGTGGTTC	Tb927.9.16190_rev	GGGGACCACTTTGTACAAGAAAGCTGGGTCTAGCATGTAAATTCATGCCAAAACGC
1_known	B11	Tb927.11.14280_for	GGGGACAAGTTTGTACAAAAAAGCAGGCTATGGCTGAAGAGATTCGTACCATGA	Tb927.11.14280_rev	GGGGACCACTTTGTACAAGAAAGCTGGGTTCATAGTTGGCTAAGTAGTTTTTCAACCA
1_known	B12	Tb927.11.7100_for	GGGGACAAGTTTGTACAAAAAAGCAGGCTATGCAACAGGAGAACCGCCA	Tb927.11.7100_rev	GGGGACCACTTTGTACAAGAAAGCTGGGTTCACCGAGCGCCGATGTG
1_known	B13	Tb927.11.3080_for	GGGGACAAGTTTGTACAAAAAAGCAGGCTATGAGCACTACGAACCCACTCG	Tb927.11.3080_rev	GGGGACCACTTTGTACAAGAAAGCTGGGTTTACCGTGCACCGATGCTAATAC
1_known	B14	Tb927.9.4040_for	GGGGACAAGTTTGTACAAAAAAGCAGGCTATGTCTACATCCCGCATTACGTTG	Tb927.9.4040_rev	GGGGACCACTTTGTACAAGAAAGCTGGGTTCAGAAGCTGGAAAGGTCTGTGC
1_known	B16	Tb927.6.1380_for	GGGGACAAGTTTGTACAAAAAAGCAGGCTATGAAGGAGGAACAGAACTCCTGC	Tb927.6.1380_rev	GGGGACCACTTTGTACAAGAAAGCTGGGTCTATTCCTCTCCTGCACAACTCAC
1_known	B20	Tb927.3.4890_for	GGGGACAAGTTTGTACAAAAAAGCAGGCTATGTTACGCAGATCGGTGGTTAAC	Tb927.3.4890_rev	GGGGACCACTTTGTACAAGAAAGCTGGGTTCAAATCCTTTTCGCTAAAGCCACA
1_known	B21	Tb927.6.4720_for	GGGGACAAGTTTGTACAAAAAAGCAGGCTATGCGACCGGAGCAGTGT	Tb927.6.4720_rev	GGGGACCACTTTGTACAAGAAAGCTGGGTCTAATTCGCACGGGTTATGTTCACG
1_known	B22	Tb927.6.1210_for	GGGGACAAGTTTGTACAAAAAAGCAGGCTATGCATTCCATTCTACCATACACATTAGAT	Tb927.6.1210_rev	GGGGACCACTTTGTACAAGAAAGCTGGGTTTACCTAGCTCCCAAGACAGAAGC
1_known	B23	Tb927.10.12450_for	GGGGACAAGTTTGTACAAAAAAGCAGGCTATGCTGTATTTTACCTTGATTGTCCGA	Tb927.10.12450_rev	GGGGACCACTTTGTACAAGAAAGCTGGGTTCAAGCGGAGAGGTAAAATACGTATACC
1_known	B24	Tb927.10.3150_for	GGGGACAAGTTTGTACAAAAAAGCAGGCTATGGTATCCAACGGTAAAACCAACG	Tb927.10.3150_rev	GGGGACCACTTTGTACAAGAAAGCTGGGTTCACTTCGCCTTCCCACCC
1_known	C1	Tb927.9.14080_for	GGGGACAAGTTTGTACAAAAAAGCAGGCTATGAAGCTTTACTCCCTGGCAATTG	Tb927.9.14080_rev	GGGGACCACTTTGTACAAGAAAGCTGGGTTCACATGACGGTGCAACACC
1_known	C2	Tb927.11.7270_for	GGGGACAAGTTTGTACAAAAAAGCAGGCTATGTCCATTCTCACGTACAGCGG	Tb927.11.7270_rev	GGGGACCACTTTGTACAAGAAAGCTGGGTCTAGTCTTTGCGACCCTTGATGAG
1_known	C3	Tb927.10.7160_for	GGGGACAAGTTTGTACAAAAAAGCAGGCTATGGTGGAGTTGGTGAGGTTGA	Tb927.10.7160_rev	GGGGACCACTTTGTACAAGAAAGCTGGGTTTAGAACCTCTGCACGGGTTTTTC
1_known	C4	Tb927.11.1710_for	GGGGACAAGTTTGTACAAAAAAGCAGGCTATGATTCGACTCGCATGCCTG	Tb927.11.1710_rev	GGGGACCACTTTGTACAAGAAAGCTGGGTTTAATGGTATCGCGATGTGTCACTTAC
1_known	C5	Tb927.10.11700_for	GGGGACAAGTTTGTACAAAAAAGCAGGCTATGAGCTCTGGAAGCACGAAGC	Tb927.10.11700_rev	GGGGACCACTTTGTACAAGAAAGCTGGGTTCACTTATTTAACCCCTCCATACCATATTT
1_known	C6	Tb927.10.4710_for	GGGGACAAGTTTGTACAAAAAAGCAGGCTATGGCAGAGACGACTATCGGG	Tb927.10.4710_rev	GGGGACCACTTTGTACAAGAAAGCTGGGTCTAGCTTACAGATTGCACTCGCTC
1_known	C7	Tb927.8.3370_for	GGGGACAAGTTTGTACAAAAAAGCAGGCTATGCAGGAGAAGGAGGTGCA	Tb927.8.3370_rev	GGGGACCACTTTGTACAAGAAAGCTGGGTTCATTCTTCCGGTACAAAAAGACCC
1_known	C8	Tb927.7.6380_for	GGGGACAAGTTTGTACAAAAAAGCAGGCTATGTCGGTAGAGGCGAGTAGAC	Tb927.7.6380_rev	GGGGACCACTTTGTACAAGAAAGCTGGGTTCACTCTTTCTTTGCTTTGGTCCG
1_known	C9	Tb927.10.4590_for	GGGGACAAGTTTGTACAAAAAAGCAGGCTATGGTGATGCGTTGGGGATTTT	Tb927.10.4590_rev	GGGGACCACTTTGTACAAGAAAGCTGGGTTTATGGTCCCTCACCCGCTC
1_known	C10	Tb927.11.8470_for	GGGGACAAGTTTGTACAAAAAAGCAGGCTATGAGCGTGGGCAAACCTATCA	Tb927.11.8470_rev	GGGGACCACTTTGTACAAGAAAGCTGGGTTTACCTTTGCTGCGCAGTAAGC
1_known	C11	Tb927.5.3220_for	GGGGACAAGTTTGTACAAAAAAGCAGGCTATGTCATTGCTGCATCGGGC	Tb927.5.3220_rev	GGGGACCACTTTGTACAAGAAAGCTGGGTTTACATTCCGGTGTACCAGCACC
1_known	C12	Tb927.11.15480_for	GGGGACAAGTTTGTACAAAAAAGCAGGCTATGGCCGATACGGAGGCA	Tb927.11.15480_rev	GGGGACCACTTTGTACAAGAAAGCTGGGTTTAAGCTTTTTCTTCGGCAGCACC
1_known	C13	Tb927.11.15020_for	GGGGACAAGTTTGTACAAAAAAGCAGGCTATGGCTTTCAGCATTCCACCAC	Tb927.11.15020_rev	GGGGACCACTTTGTACAAGAAAGCTGGGTTTACAAGTCACTATGCGGTGCC
1_known	C14	Tb927.7.5570_for	GGGGACAAGTTTGTACAAAAAAGCAGGCTATGGAAAAGCATAAGGAAGGAACTGAA	Tb927.7.5570_rev	GGGGACCACTTTGTACAAGAAAGCTGGGTTTACGGAACCTCCAACTTTTCCTTC
1_known	C15	Tb927.8.890_for	GGGGACAAGTTTGTACAAAAAAGCAGGCTATGTCTACAGAGTACGATCACCTTTTTAAG	Tb927.8.890_rev	GGGGACCACTTTGTACAAGAAAGCTGGGTTTAGCAACATCCACTCTTTTGACCTC
1_known	C16	Tb927.4.3610_for	GGGGACAAGTTTGTACAAAAAAGCAGGCTATGGCCCAACCCATGCTGG	Tb927.4.3610_rev	GGGGACCACTTTGTACAAGAAAGCTGGGTTCAGCAAGAGCCAAAATACTGAAAGC
1_known	C17	Tb927.11.10240_for	GGGGACAAGTTTGTACAAAAAAGCAGGCTATGCTTCGCCGTGTCGGC	Tb927.11.10240_rev	GGGGACCACTTTGTACAAGAAAGCTGGGTTTACTCGCTAGTTTTCGCCGAC
1_known	C18	Tb927.10.8890_for	GGGGACAAGTTTGTACAAAAAAGCAGGCTATGTTCCGCGCTTGTTTGCG	Tb927.10.8890_rev	GGGGACCACTTTGTACAAGAAAGCTGGGTTCACTTTTTTTTCGTTACCTTCTTTTCTTT
1_known	C19	Tb927.10.1470_for	GGGGACAAGTTTGTACAAAAAAGCAGGCTATGGAACCAGCTTGCAAATACGA	Tb927.10.1470_rev	GGGGACCACTTTGTACAAGAAAGCTGGGTTTACAGTTTTGCCTTCACAGCGG
1_known	C20	Tb927.11.5680_for	GGGGACAAGTTTGTACAAAAAAGCAGGCTATGTCAGGAACAACTTCCATTCGAG	Tb927.11.5680_rev	GGGGACCACTTTGTACAAGAAAGCTGGGTTTACCGGCCGCGCTCCGC
1_known	C21	Tb927.10.1400_for	GGGGACAAGTTTGTACAAAAAAGCAGGCTATGGAACCAGCTTGCAAATACGA	Tb927.10.1400_rev	GGGGACCACTTTGTACAAGAAAGCTGGGTTTACCGCTTGGCTTCTCCAGC
1_known	C22	Tb927.11.14080_for	GGGGACAAGTTTGTACAAAAAAGCAGGCTATGACCATTATGATGGACGGCCC	Tb927.11.14080_rev	GGGGACCACTTTGTACAAGAAAGCTGGGTTTACTGCTCCTGATTCGCCGA
1_known	C24	Tb927.8.4030_for	GGGGACAAGTTTGTACAAAAAAGCAGGCTATGACACGATTGGCGGGTCA	Tb927.8.4030_rev	GGGGACCACTTTGTACAAGAAAGCTGGGTTTAGTCTTCCTCATCCTCTTTCCCTTC
1_known	D1	Tb927.11.15240_for	GGGGACAAGTTTGTACAAAAAAGCAGGCTATGCAGCAGCACCATTATGTTTTC	Tb927.11.15240_rev	GGGGACCACTTTGTACAAGAAAGCTGGGTTCAGCAGAAGCAGCCACTTTC
1_known	D2	Tb927.10.12190_for	GGGGACAAGTTTGTACAAAAAAGCAGGCTATGACAGATAAGGATGGTGGCACT	Tb927.10.12190_rev	GGGGACCACTTTGTACAAGAAAGCTGGGTCTACACAAAATGGGCCAATTCATTCTC
1_known	D3	Tb927.3.1090_for	GGGGACAAGTTTGTACAAAAAAGCAGGCTATGACGTACTGCCCCATTACGC	Tb927.3.1090_rev	GGGGACCACTTTGTACAAGAAAGCTGGGTTCACTCTCTGGTTGATGAGGGAG
1_known	D4	Tb927.11.4960_for	GGGGACAAGTTTGTACAAAAAAGCAGGCTATGGGTAAGGATTCCAATGATGGGA	Tb927.11.4960_rev	GGGGACCACTTTGTACAAGAAAGCTGGGTCTAATAGCGACGCCATTTGTGCT
1_known	D5	Tb927.6.5020_for	GGGGACAAGTTTGTACAAAAAAGCAGGCTATGTCGGAAAGGGACAAGCAGC	Tb927.6.5020_rev	GGGGACCACTTTGTACAAGAAAGCTGGGTTTACCAATATAAACAACACTCCGACATCC
1_known	D6	Tb927.6.2050_for	GGGGACAAGTTTGTACAAAAAAGCAGGCTATGAGTGAGTATCACATGGATCTGGG	Tb927.6.2050_rev	GGGGACCACTTTGTACAAGAAAGCTGGGTTTACTTTTTACCCTTCTTCCTGAGCG
1_known	D7	Tb927.10.6060_for	GGGGACAAGTTTGTACAAAAAAGCAGGCTATGGCGGACGAAGCTTCCA	Tb927.10.6060_rev	GGGGACCACTTTGTACAAGAAAGCTGGGTTTATTTCACAGGGCAGGCACG
1_known	D8	Tb927.6.3870_for	GGGGACAAGTTTGTACAAAAAAGCAGGCTATGGCATCAGACCCGTCGG	Tb927.6.3870_rev	GGGGACCACTTTGTACAAGAAAGCTGGGTTCAATATGGCCCACCGCGT
1_known	D9	Tb927.10.11990_for	GGGGACAAGTTTGTACAAAAAAGCAGGCTATGCGTCCGTTAACGGAGGA	Tb927.10.11990_rev	GGGGACCACTTTGTACAAGAAAGCTGGGTCTACGTCAAGCGTGCCTCG
1_known	D10	Tb927.8.4770_for	GGGGACAAGTTTGTACAAAAAAGCAGGCTATGGCGCAAGGTGACAACAG	Tb927.8.4770_rev	GGGGACCACTTTGTACAAGAAAGCTGGGTCTAACAGCACACCGCGCC
1_known	D11	Tb927.10.5760_for	GGGGACAAGTTTGTACAAAAAAGCAGGCTATGGGTCTGCATATCCTTTTATTTGGAG	Tb927.10.5760_rev	GGGGACCACTTTGTACAAGAAAGCTGGGTTTACAACTTGCTCTTTTCGATACGAAGA
1_known	D12	Tb927.11.12950_for	GGGGACAAGTTTGTACAAAAAAGCAGGCTATGCCATCTCATACCACTTCCGA	Tb927.11.12950_rev	GGGGACCACTTTGTACAAGAAAGCTGGGTTTAAAAGAAAGCTGCCACCACATCC
1_known	D13	Tb927.8.4330_for	GGGGACAAGTTTGTACAAAAAAGCAGGCTATGGAAGACATGAACCTTACGTTCAAA	Tb927.8.4330_rev	GGGGACCACTTTGTACAAGAAAGCTGGGTTTAACAGCACCCGCCACTCG
1_known	D14	Tb927.11.13940_for	GGGGACAAGTTTGTACAAAAAAGCAGGCTATGTCCACTGACCGTTCATCTCG	Tb927.11.13940_rev	GGGGACCACTTTGTACAAGAAAGCTGGGTTTAGATGTCAATTGCGTCAGGTTCA
1_known	D15	Tb927.11.9420_for	GGGGACAAGTTTGTACAAAAAAGCAGGCTATGAGCGAGGCACGCCAG	Tb927.11.9420_rev	GGGGACCACTTTGTACAAGAAAGCTGGGTTCAGATAGATGACTCTGGATTGAAGAGG
1_known	D16	Tb927.5.3560_for	GGGGACAAGTTTGTACAAAAAAGCAGGCTATGCCCATTAAATATAGTTGTGTCAATGAA	Tb927.5.3560_rev	GGGGACCACTTTGTACAAGAAAGCTGGGTTTATGACTTGCAGTTGGAAAAGTTCGG
1_known	D17	Tb927.4.2030_for	GGGGACAAGTTTGTACAAAAAAGCAGGCTATGCCTTCATATCCGTCGCAC	Tb927.4.2030_rev	GGGGACCACTTTGTACAAGAAAGCTGGGTTCAGTTGCCTTCACCCAAATCG
1_known	D18	Tb927.3.1120_for	GGGGACAAGTTTGTACAAAAAAGCAGGCTATGCAGGCTTCCTCGACGG	Tb927.3.1120_rev	GGGGACCACTTTGTACAAGAAAGCTGGGTCTAGTCGTCCTCGCCCATCG
1_known	D19	Tb927.9.1610_for	GGGGACAAGTTTGTACAAAAAAGCAGGCTATGCCGAAAGCTAAAACTACCACA	Tb927.9.1610_rev	GGGGACCACTTTGTACAAGAAAGCTGGGTTCACTGGCGGCTTCCCGC
1_known	D20	Tb927.9.11840_for	GGGGACAAGTTTGTACAAAAAAGCAGGCTATGCTCTCAGCAGCTGCTGC	Tb927.9.11840_rev	GGGGACCACTTTGTACAAGAAAGCTGGGTTTAAAAGGTATCGTTAACACGATTCATCAC
1_known	D21	Tb927.3.2000_for	GGGGACAAGTTTGTACAAAAAAGCAGGCTATGGATGAGAAGCCGCGGT	Tb927.3.2000_rev	GGGGACCACTTTGTACAAGAAAGCTGGGTTCATTGGCGAGCAGCAGAC
1_known	D22	Tb927.11.12120_for	GGGGACAAGTTTGTACAAAAAAGCAGGCTATGGCAGCTGTCACCCAGC	Tb927.11.12120_rev	GGGGACCACTTTGTACAAGAAAGCTGGGTTTACGACAGCGTAGGAGGAACG
1_known	D23	Tb927.11.11520_for	GGGGACAAGTTTGTACAAAAAAGCAGGCTATGTCTGAGTTCCAAAGGTTTGTTAAGC	Tb927.11.11520_rev	GGGGACCACTTTGTACAAGAAAGCTGGGTCTATTTGATCTTGTTCCAGTTCAAATACGT
1_known	D24	Tb927.8.4610_for	GGGGACAAGTTTGTACAAAAAAGCAGGCTATGATCACAGCAGCTTCCCCT	Tb927.8.4610_rev	GGGGACCACTTTGTACAAGAAAGCTGGGTTTAACAGCAACCACCACGCTT
1_known	E1	Tb927.10.5110_for	GGGGACAAGTTTGTACAAAAAAGCAGGCTATGCGGGTGCGTTCACTC	Tb927.10.5110_rev	GGGGACCACTTTGTACAAGAAAGCTGGGTTTAACACTCCAACTCCTGCAAAGAAA
1_known	E2	Tb927.11.10740_for	GGGGACAAGTTTGTACAAAAAAGCAGGCTATGGAAGTGGCATTCACCATTTCG	Tb927.11.10740_rev	GGGGACCACTTTGTACAAGAAAGCTGGGTTTACGCCTTTGGCCGTCG
1_known	E3	Tb927.8.5465_for	GGGGACAAGTTTGTACAAAAAAGCAGGCTATGGGTTGCTCAGGATCGAAGA	Tb927.8.5465_rev	GGGGACCACTTTGTACAAGAAAGCTGGGTTCAATTACCTTCATTTGCTCCGTTCT
1_known	E4	Tb927.8.5440_for	GGGGACAAGTTTGTACAAAAAAGCAGGCTATGGGTTGCTCAGGATCGAAGA	Tb927.8.5440_rev	GGGGACCACTTTGTACAAGAAAGCTGGGTTCAATTACCTTCATTTGCTCCGTTCT
1_known	E5	Tb927.4.430_for	GGGGACAAGTTTGTACAAAAAAGCAGGCTATGGCATCTGGAGGATCTGTTATTGG	Tb927.4.430_rev	GGGGACCACTTTGTACAAGAAAGCTGGGTTCATCGAATAATGGCTGTTTTCTCAAAGC
1_known	E6	Tb927.10.6380_for	GGGGACAAGTTTGTACAAAAAAGCAGGCTATGTCTCGTACTGACTTTTCATGTGC	Tb927.10.6380_rev	GGGGACCACTTTGTACAAGAAAGCTGGGTTCACACCATAAAAAAAGATGTGGCTAACA
1_known	E7	Tb927.11.16830_for	GGGGACAAGTTTGTACAAAAAAGCAGGCTATGTCGGACGACTTGGTAAAGATACT	Tb927.11.16830_rev	GGGGACCACTTTGTACAAGAAAGCTGGGTTCAACGCTTCAGCCGCGG
1_known	E8	Tb927.11.10000_for	GGGGACAAGTTTGTACAAAAAAGCAGGCTATGTTTTCCGGGTGGTTCAAAAGG	Tb927.11.10000_rev	GGGGACCACTTTGTACAAGAAAGCTGGGTTCAACTAACAGTCCCACGAAGCA
1_known	E9	Tb927.11.8140_for	GGGGACAAGTTTGTACAAAAAAGCAGGCTATGATGTACATTCGTACCTTCGGTG	Tb927.11.8140_rev	GGGGACCACTTTGTACAAGAAAGCTGGGTTCATCGCGCCAAATCATATCGC
1_known	E10	Tb927.9.15090_for	GGGGACAAGTTTGTACAAAAAAGCAGGCTATGGAACGGATAGCCTCCGG	Tb927.9.15090_rev	GGGGACCACTTTGTACAAGAAAGCTGGGTTTAGTCAATAAGTCGCTTCTTTCGGAA
1_known	E11	Tb927.8.4620_for	GGGGACAAGTTTGTACAAAAAAGCAGGCTATGAAAGAGGAACCCGCTTACAAG	Tb927.8.4620_rev	GGGGACCACTTTGTACAAGAAAGCTGGGTTCAGCACCTCCACTTCCTCTTT
1_known	E12	Tb927.11.500_for	GGGGACAAGTTTGTACAAAAAAGCAGGCTATGTCTCAGGTTCCACTGGCT	Tb927.11.500_rev	GGGGACCACTTTGTACAAGAAAGCTGGGTTCATTTACGGGCAGGCCGA
1_known	E13	Tb927.10.13320_for	GGGGACAAGTTTGTACAAAAAAGCAGGCTATGAGCAGTGAAAGCTACGCC	Tb927.10.13320_rev	GGGGACCACTTTGTACAAGAAAGCTGGGTCTACACTACCTGTCGGTACGTAACG
1_known	E14	Tb927.6.5140_for	GGGGACAAGTTTGTACAAAAAAGCAGGCTATGGCGCTGCAGTTGAATAAAGA	Tb927.6.5140_rev	GGGGACCACTTTGTACAAGAAAGCTGGGTTCACCACCTTGGTGTTGTACCT
1_known	E15	Tb927.9.11000_for	GGGGACAAGTTTGTACAAAAAAGCAGGCTATGGCTCCAAACCGACAGTTG	Tb927.9.11000_rev	GGGGACCACTTTGTACAAGAAAGCTGGGTCTAGCACGCACAATCGCTTTTC
1_known	E16	Tb927.4.3190_for	GGGGACAAGTTTGTACAAAAAAGCAGGCTATGCCGGCGCCGCCAGCG	Tb927.4.3190_rev	GGGGACCACTTTGTACAAGAAAGCTGGGTTCAATTCCTAAGCACTAAGAGCCCC
1_known	E17	Tb927.10.3860_for	GGGGACAAGTTTGTACAAAAAAGCAGGCTATGAACCGTCTATTCGGTCGGA	Tb927.10.3860_rev	GGGGACCACTTTGTACAAGAAAGCTGGGTTTACAGTCCCAACTGTGTTTCCAGT
1_known	E18	Tb927.10.650_for	GGGGACAAGTTTGTACAAAAAAGCAGGCTATGCCCCGTTATTACACCCCA	Tb927.10.650_rev	GGGGACCACTTTGTACAAGAAAGCTGGGTTTACGCCTCCGTGAGATCATCA
1_known	E19	Tb927.3.4650_for	GGGGACAAGTTTGTACAAAAAAGCAGGCTATGGGTCTCTGCAAAACACTTTTTGT	Tb927.3.4650_rev	GGGGACCACTTTGTACAAGAAAGCTGGGTTTATATCTTTCCTCGAAGGGCATTAGGT

1_known	E20	Tb927.1.3200_for	GGGGACAAGTTTGTACAAAAAAGCAGGCTATGGACGCATCCAGTGCCG	Tb927.1.3200_rev	GGGGACCACTTTGTACAAGAAAGCTGGGTTTAGCCTTCGTACTTCTTTTCTTCCATT
1_known	E21	Tb927.2.5120_for	GGGGACAAGTTTGTACAAAAAAGCAGGCTATGCTTATATCTGCCTCCTTTGTGTC	Tb927.2.5120_rev	GGGGACCACTTTGTACAAGAAAGCTGGGTCTACTTTTTGCACTTGAGGTTAGTGC
1_known	E22	Tb927.10.2530_for	GGGGACAAGTTTGTACAAAAAAGCAGGCTATGAAGGTAATTTTTCTTGGACCTCCAG	Tb927.10.2530_rev	GGGGACCACTTTGTACAAGAAAGCTGGGTTTAATTCCCATGTATCTTTGCACCGA
1_known	E23	Tb927.5.950_for	GGGGACAAGTTTGTACAAAAAAGCAGGCTATGAAACGCGTTGGGACTCTTG	Tb927.5.950_rev	GGGGACCACTTTGTACAAGAAAGCTGGGTTCATAGCTTCAGAGCAGATTTCAACTC
1_known	E24	Tb927.7.6440_for	GGGGACAAGTTTGTACAAAAAAGCAGGCTATGTCTAATGCCGAGGCCTACG	Tb927.7.6440_rev	GGGGACCACTTTGTACAAGAAAGCTGGGTTTATTTTTGCAAAAACTTTAACGTAGCAAA
1_known	F1	Tb927.6.2170_for	GGGGACAAGTTTGTACAAAAAAGCAGGCTATGCGTGCGCTTACGTTTCG	Tb927.6.2170_rev	GGGGACCACTTTGTACAAGAAAGCTGGGTCTAATCATCCGAAGCTACTCCAACTTG
1_known	F2	Tb927.8.5740_for	GGGGACAAGTTTGTACAAAAAAGCAGGCTATGTCTGGAACTTTACTCAAGGAAGAGC	Tb927.8.5740_rev	GGGGACCACTTTGTACAAGAAAGCTGGGTCTAATTCAATTTCATTGGCTCAAATGTGC
1_known	F3	Tb927.4.2670_for	GGGGACAAGTTTGTACAAAAAAGCAGGCTATGAAGCCTACAAAACCACTACTTTACA	Tb927.4.2670_rev	GGGGACCACTTTGTACAAGAAAGCTGGGTTCATTCAACTGCTTCCATGTGTTTCA
1_known	F4	Tb927.9.1720_for	GGGGACAAGTTTGTACAAAAAAGCAGGCTATGGCGGCTGAGCCCGCG	Tb927.9.1720_rev	GGGGACCACTTTGTACAAGAAAGCTGGGTTTAAAAGGAAGTATCACTGTTAAAGCAAAG
1_known	F5	Tb927.8.3790_for	GGGGACAAGTTTGTACAAAAAAGCAGGCTATGGATGCAGAAGAGAATTTCCTGAATG	Tb927.8.3790_rev	GGGGACCACTTTGTACAAGAAAGCTGGGTCTACGCACTGTTCGCAGCG
1_known	F6	Tb927.8.5330_for	GGGGACAAGTTTGTACAAAAAAGCAGGCTATGGAAGGTCCTTTCCTCAGCC	Tb927.8.5330_rev	GGGGACCACTTTGTACAAGAAAGCTGGGTCTATGAAACGCTACCGTTGGGG
1_known	F7	Tb927.11.13280_for	GGGGACAAGTTTGTACAAAAAAGCAGGCTATGCTCCGACGTATCATCAGCC	Tb927.11.13280_rev	GGGGACCACTTTGTACAAGAAAGCTGGGTCTACACAGATGTGCGAGCGAA
1_known	F8	Tb927.6.5050_for	GGGGACAAGTTTGTACAAAAAAGCAGGCTATGGCGGTTGAATCCGATGTG	Tb927.6.5050_rev	GGGGACCACTTTGTACAAGAAAGCTGGGTTTATACCATCACCGCCTTTTGGGC
1_known	F9	Tb927.10.7420_for	GGGGACAAGTTTGTACAAAAAAGCAGGCTATGAGCAAGAACGAGCGAGATAC	Tb927.10.7420_rev	GGGGACCACTTTGTACAAGAAAGCTGGGTCTACGAGCTGTCGTAATCGCTG
1_known	F10	Tb927.7.280_for	GGGGACAAGTTTGTACAAAAAAGCAGGCTATGCCCCTTCGGTTCACCT	Tb927.7.280_rev	GGGGACCACTTTGTACAAGAAAGCTGGGTCTACGCCGGTTCCTTCAGC
1_known	F11	Tb927.7.5580_for	GGGGACAAGTTTGTACAAAAAAGCAGGCTATGAAGCGTTTTAAGGCAAAGCTC	Tb927.7.5580_rev	GGGGACCACTTTGTACAAGAAAGCTGGGTTTACGGAACCTCCAACTTTTCCTTC
1_known	F12	Tb927.8.4180_for	GGGGACAAGTTTGTACAAAAAAGCAGGCTATGGTGCAAGATACAGGCAAGG	Tb927.8.4180_rev	GGGGACCACTTTGTACAAGAAAGCTGGGTTCAAACAACTGACAAATCCTTCCGT
1_known	F13	Tb927.4.3570_for	GGGGACAAGTTTGTACAAAAAAGCAGGCTATGTCGATCAAGGACGTCAATGTG	Tb927.4.3570_rev	GGGGACCACTTTGTACAAGAAAGCTGGGTCTAGATTTTATTCCAGGCCACAATGTCC
1_known	F14	Tb927.11.14290_for	GGGGACAAGTTTGTACAAAAAAGCAGGCTATGGTTCTTGTGCTTGTTGTTGGT	Tb927.11.14290_rev	GGGGACCACTTTGTACAAGAAAGCTGGGTTCATTCAGGAGGTGGAGGAGGA
1_known	F16	Tb927.10.9010_for	GGGGACAAGTTTGTACAAAAAAGCAGGCTATGATGCCTGCACCGAACG	Tb927.10.9010_rev	GGGGACCACTTTGTACAAGAAAGCTGGGTTCACCGTAATGCCAGCGC
1_known	F17	Tb927.10.8560_for	GGGGACAAGTTTGTACAAAAAAGCAGGCTATGTTGAAGAGAGGTGGGTTAAACG	Tb927.10.8560_rev	GGGGACCACTTTGTACAAGAAAGCTGGGTCTACATAACACTGCACTTTTTCTTCTTTGA
1_known	F18	Tb927.11.6030_for	GGGGACAAGTTTGTACAAAAAAGCAGGCTATGAGGAATATTAACCTCGTCGTTTTGG	Tb927.11.6030_rev	GGGGACCACTTTGTACAAGAAAGCTGGGTTCAGAGCATAGTGCATCCCGA
1_known	F19	Tb927.8.1990_for	GGGGACAAGTTTGTACAAAAAAGCAGGCTATGCTTCGCCGTTTCTCCATG	Tb927.8.1990_rev	GGGGACCACTTTGTACAAGAAAGCTGGGTCTATAGATTCTTCTCAAAGTATTCCTTCGC
1_known	F20	Tb927.7.2540_for	GGGGACAAGTTTGTACAAAAAAGCAGGCTATGGATAATGAATCTACCAATGAAGCAGC	Tb927.7.2540_rev	GGGGACCACTTTGTACAAGAAAGCTGGGTTCACTCCGCGCCTTCCGT
1_known	F21	Tb927.11.13930_for	GGGGACAAGTTTGTACAAAAAAGCAGGCTATGGTGCGGGACACGAGT	Tb927.11.13930_rev	GGGGACCACTTTGTACAAGAAAGCTGGGTTTACTCGTAATCGTCCCGCTGT
1_known	F22	Tb927.9.9750_for	GGGGACAAGTTTGTACAAAAAAGCAGGCTATGCCAGCGGACTCAGTTGT	Tb927.9.9750_rev	GGGGACCACTTTGTACAAGAAAGCTGGGTCTATGGGGCTGTGTCAGCAGG
1_known	F23	Tb927.10.12170_for	GGGGACAAGTTTGTACAAAAAAGCAGGCTATGCGATCAGCCACTGAATATACAG	Tb927.10.12170_rev	GGGGACCACTTTGTACAAGAAAGCTGGGTTCACTTGTTAGGGCTCTCCATGT
1_known	F24	Tb927.6.2420_for	GGGGACAAGTTTGTACAAAAAAGCAGGCTATGCGTCGTGCACTTGTATTCA	Tb927.6.2420_rev	GGGGACCACTTTGTACAAGAAAGCTGGGTTTACGAAACAAATTTGTTAATGCTGCTCA
1_known	G1	Tb927.1.1220_for	GGGGACAAGTTTGTACAAAAAAGCAGGCTATGCCGGCCACTGAGATGC	Tb927.1.1220_rev	GGGGACCACTTTGTACAAGAAAGCTGGGTTCATTCATCCAAATCAAACTCCTGCT
1_known	G2	Tb927.7.4850_for	GGGGACAAGTTTGTACAAAAAAGCAGGCTATGGTAGCAAGACTTAGGCAATATGAAC	Tb927.7.4850_rev	GGGGACCACTTTGTACAAGAAAGCTGGGTCTAAGCATGGTGAGGTGGGGG
1_known	G3	Tb927.10.14630_for	GGGGACAAGTTTGTACAAAAAAGCAGGCTATGCGTGGCTGCTACCTCC	Tb927.10.14630_rev	GGGGACCACTTTGTACAAGAAAGCTGGGTCTATTGCGGGGGGGATTTGTAGT
1_known	G4	Tb927.11.15230_for	GGGGACAAGTTTGTACAAAAAAGCAGGCTATGACCATGCGCGCGATG	Tb927.11.15230_rev	GGGGACCACTTTGTACAAGAAAGCTGGGTCTATACAACTCTTCTTTTGTTGAAAATACG
1_known	G5	Tb927.4.470_for	GGGGACAAGTTTGTACAAAAAAGCAGGCTATGGCACGGGGAGGAGGC	Tb927.4.470_rev	GGGGACCACTTTGTACAAGAAAGCTGGGTTCAAAAACCACGCCCGCG
1_known	G6	Tb927.10.2540_for	GGGGACAAGTTTGTACAAAAAAGCAGGCTATGCACGTGTTTCTCACAGGTC	Tb927.10.2540_rev	GGGGACCACTTTGTACAAGAAAGCTGGGTTCAACTTGACCACCAAAACAACTTGG
1_known	G7	Tb927.10.10610_for	GGGGACAAGTTTGTACAAAAAAGCAGGCTATGCTCTCCAAAGTATATTTACTTGCCTAT	Tb927.10.10610_rev	GGGGACCACTTTGTACAAGAAAGCTGGGTCTCAGCTTGCCTTCTTCTTGTTGG
1_known	G8	Tb927.10.11610_for	GGGGACAAGTTTGTACAAAAAAGCAGGCTATGGTGAATCATTTAGTTGTATTGATCGGC	Tb927.10.11610_rev	GGGGACCACTTTGTACAAGAAAGCTGGGTCTACTTCTCCTCTGACATTCTTCCGT
1_known	G9	Tb927.2.3960_for	GGGGACAAGTTTGTACAAAAAAGCAGGCTATGAATAGGTCCTCCTCTACCAGTGT	Tb927.2.3960_rev	GGGGACCACTTTGTACAAGAAAGCTGGGTTTACGCCGTAATGTTGACACCAC
1_known	G10	Tb927.10.12960_for	GGGGACAAGTTTGTACAAAAAAGCAGGCTATGTCGGTGTCAGCGACACC	Tb927.10.12960_rev	GGGGACCACTTTGTACAAGAAAGCTGGGTTCAGCAGGCACACCCGCC
1_known	G11	Tb927.2.2130_for	GGGGACAAGTTTGTACAAAAAAGCAGGCTATGGAAACAAACTCAGCGCCA	Tb927.2.2130_rev	GGGGACCACTTTGTACAAGAAAGCTGGGTTTAGCAGCAACCGCCTTCCC
1_known	G12	Tb927.10.1830_for	GGGGACAAGTTTGTACAAAAAAGCAGGCTATGTCGTCTCTGCAAGATCCTTTC	Tb927.10.1830_rev	GGGGACCACTTTGTACAAGAAAGCTGGGTTCAACTAAAGACACAATAGAAGAGGAGCA
1_known	G13	Tb927.7.1790_for	GGGGACAAGTTTGTACAAAAAAGCAGGCTATGTCACAGTATGATGCGATTCTGAC	Tb927.7.1790_rev	GGGGACCACTTTGTACAAGAAAGCTGGGTTCACAGTCGTGACCTGGTAATGT
1_known	G14	Tb927.10.290_for	GGGGACAAGTTTGTACAAAAAAGCAGGCTATGTCTGAGTCTTCGTACGGTCTT	Tb927.10.290_rev	GGGGACCACTTTGTACAAGAAAGCTGGGTTTAGATTTGGTCCAGGTAATCCTTCAAC
1_known	G15	Tb927.7.3430_for	GGGGACAAGTTTGTACAAAAAAGCAGGCTATGCTACATCGTACGCGTTTAGC	Tb927.7.3430_rev	GGGGACCACTTTGTACAAGAAAGCTGGGTCTAATAACGAGGAGACAGAATGTCAAGAA
1_known	G16	Tb927.10.3710_for	GGGGACAAGTTTGTACAAAAAAGCAGGCTATGCCACCGAAACGCGCC	Tb927.10.3710_rev	GGGGACCACTTTGTACAAGAAAGCTGGGTTCAACTCACCATATGATCGCTTCCC
1_known	G17	Tb927.10.3120_for	GGGGACAAGTTTGTACAAAAAAGCAGGCTATGCTCCGTTCCCCGTGG	Tb927.10.3120_rev	GGGGACCACTTTGTACAAGAAAGCTGGGTCTACGCTAAATTCGACCGAAAAATTATGT
1_known	G18	Tb927.10.13950_for	GGGGACAAGTTTGTACAAAAAAGCAGGCTATGTCCGTTGTTAAAGTATCGCTTACG	Tb927.10.13950_rev	GGGGACCACTTTGTACAAGAAAGCTGGGTTTAAAACACCTCCGGGGGAAAGT
1_known	G19	Tb927.11.11030_for	GGGGACAAGTTTGTACAAAAAAGCAGGCTATGGGGGTGCCAACAATGGA	Tb927.11.11030_rev	GGGGACCACTTTGTACAAGAAAGCTGGGTCTAAAATGCAAACTGTTCGCACAACT
1_known	G20	Tb927.11.2260_for	GGGGACAAGTTTGTACAAAAAAGCAGGCTATGATGGCTGAATCAAGCGCG	Tb927.11.2260_rev	GGGGACCACTTTGTACAAGAAAGCTGGGTTTAGGCCTTGCTAGCGCCA
1_known	G21	Tb927.8.5470_for	GGGGACAAGTTTGTACAAAAAAGCAGGCTATGGGTTGCTCAGGATCGAAGA	Tb927.8.5470_rev	GGGGACCACTTTGTACAAGAAAGCTGGGTCTAGGCAGAACCCTCGGCC
1_known	G22	Tb927.10.1390_for	GGGGACAAGTTTGTACAAAAAAGCAGGCTATGCACTCGGGCCATCCT	Tb927.10.1390_rev	GGGGACCACTTTGTACAAGAAAGCTGGGTTTACAATTTGCTCGGGTACCGTTT
1_known	G23	Tb927.10.4700_for	GGGGACAAGTTTGTACAAAAAAGCAGGCTATGTCAAAGGAGCCGGGTCA	Tb927.10.4700_rev	GGGGACCACTTTGTACAAGAAAGCTGGGTTCATATAGCTGCGCCACCAAAC
1_known	G24	Tb927.10.7070_for	GGGGACAAGTTTGTACAAAAAAGCAGGCTATGCAGCAGCGTCTTGTTCTC	Tb927.10.7070_rev	GGGGACCACTTTGTACAAGAAAGCTGGGTTCATTTACGCTTCAGCAGACTTTCA
1_known	H1	Tb927.11.12860.2_for	GGGGACAAGTTTGTACAAAAAAGCAGGCTATGAGCAACGCAGCTCCAG	Tb927.11.12860.2_rev	GGGGACCACTTTGTACAAGAAAGCTGGGTCTAGTTCTTACGGCGCGTAACA
1_known	H2	Tb927.10.15690_for	GGGGACAAGTTTGTACAAAAAAGCAGGCTATGCAATTTGATGGACGTAGTGGT	Tb927.10.15690_rev	GGGGACCACTTTGTACAAGAAAGCTGGGTTCAGCGAAGATGAAGGTTCTGTG
1_known	H3	Tb927.7.1780_for	GGGGACAAGTTTGTACAAAAAAGCAGGCTATGTCACTTGTGGAGGTTTTGCC	Tb927.7.1780_rev	GGGGACCACTTTGTACAAGAAAGCTGGGTTTAAATCAATTTTTTTGCCTCGGCGC
1_known	H4	Tb927.10.8330_for	GGGGACAAGTTTGTACAAAAAAGCAGGCTATGGCCAGTTTACTTGCAGATGTT	Tb927.10.8330_rev	GGGGACCACTTTGTACAAGAAAGCTGGGTCTAGTTATAAGTCCTTACTTTTTCCATCCA
1_known	H5	Tb927.11.5510_for	GGGGACAAGTTTGTACAAAAAAGCAGGCTATGCCCACACGACCCCTG	Tb927.11.5510_rev	GGGGACCACTTTGTACAAGAAAGCTGGGTTCAAGCTTTGGTAGCATTACATAATGTCT
1_known	H6	Tb927.11.250_for	GGGGACAAGTTTGTACAAAAAAGCAGGCTATGACCAAAACGTGGCTACCG	Tb927.11.250_rev	GGGGACCACTTTGTACAAGAAAGCTGGGTTCACTCGCGAGACTCACACA
1_known	H7	Tb927.8.4470_for	GGGGACAAGTTTGTACAAAAAAGCAGGCTATGTTCACTTACTCTACGTTTTTACGGAG	Tb927.8.4470_rev	GGGGACCACTTTGTACAAGAAAGCTGGGTTCATCGGCCCATCACAACGT
1_known	H8	Tb927.1.1680_for	GGGGACAAGTTTGTACAAAAAAGCAGGCTATGTCGTCAAGCAAAGGTGGTG	Tb927.1.1680_rev	GGGGACCACTTTGTACAAGAAAGCTGGGTCTAGTCACTTTCGTCAAAGAATGCATTAAC
1_known	H9	Tb927.5.1080_for	GGGGACAAGTTTGTACAAAAAAGCAGGCTATGCTCGACACGAGTGGCA	Tb927.5.1080_rev	GGGGACCACTTTGTACAAGAAAGCTGGGTTTAATTTTTTTTTACTTTCTTCTTTTTTAA
1_known	H10	Tb927.3.780_for	GGGGACAAGTTTGTACAAAAAAGCAGGCTATGTCTGGCTCTGGTCATGACC	Tb927.3.780_rev	GGGGACCACTTTGTACAAGAAAGCTGGGTCTATGATGCGGGGGTTGGATG
1_known	H11	Tb927.5.1370_for	GGGGACAAGTTTGTACAAAAAAGCAGGCTATGATAGCCATTCTGTTTGGGTTCAC	Tb927.5.1370_rev	GGGGACCACTTTGTACAAGAAAGCTGGGTTCAAAACCGAAGGAACGCCG
1_known	H12	Tb927.8.2410_for	GGGGACAAGTTTGTACAAAAAAGCAGGCTATGGTTGTGCGGGAGGTGG	Tb927.8.2410_rev	GGGGACCACTTTGTACAAGAAAGCTGGGTTTAGGTTCGAGCAAACGTGATGC
1_known	H13	Tb927.10.15500_for	GGGGACAAGTTTGTACAAAAAAGCAGGCTATGCATGGAGTTGAGGTATCAGCG	Tb927.10.15500_rev	GGGGACCACTTTGTACAAGAAAGCTGGGTCTACCATATCAGATAGATAAACAGCAACGT
1_known	H14	Tb927.5.3350_for	GGGGACAAGTTTGTACAAAAAAGCAGGCTATGAGGTCTGTCATGATGCGTTG	Tb927.5.3350_rev	GGGGACCACTTTGTACAAGAAAGCTGGGTTTACTTCATAGCCTGTTCATACGTCCT
1_known	H15	Tb927.10.790_for	GGGGACAAGTTTGTACAAAAAAGCAGGCTATGCAGGGAGGAACAAAAATATATGGC	Tb927.10.790_rev	GGGGACCACTTTGTACAAGAAAGCTGGGTTTACTTCTTTTCCTCTTTTTTACCACCTAC
1_known	H16	Tb927.11.4530_for	GGGGACAAGTTTGTACAAAAAAGCAGGCTATGCAGGTGCGTCGCGCC	Tb927.11.4530_rev	GGGGACCACTTTGTACAAGAAAGCTGGGTTTACTTTTTACCTTTTCCGTGCTTACCG
1_known	H17	Tb927.3.2930_for	GGGGACAAGTTTGTACAAAAAAGCAGGCTATGTTCTACCCCAACAGCCCG	Tb927.3.2930_rev	GGGGACCACTTTGTACAAGAAAGCTGGGTTCAACCAGCGGCACCGCG
1_known	H18	Tb927.6.3320_for	GGGGACAAGTTTGTACAAAAAAGCAGGCTATGAGCACCGAAGACATTCTGATTG	Tb927.6.3320_rev	GGGGACCACTTTGTACAAGAAAGCTGGGTTTACCAGGCCACAAAGGGGC
1_known	H19	Tb927.10.13830_for	GGGGACAAGTTTGTACAAAAAAGCAGGCTATGTGGCGTAGTCAGTGCTGT	Tb927.10.13830_rev	GGGGACCACTTTGTACAAGAAAGCTGGGTTCATGCAACAAGTGAGTTGTTCATGA
1_known	H20	Tb927.6.3040_for	GGGGACAAGTTTGTACAAAAAAGCAGGCTATGAGTAGCGACAGTTCAGACTCA	Tb927.6.3040_rev	GGGGACCACTTTGTACAAGAAAGCTGGGTCTACATCACTGCGCATTTACCCT
1_known	H21	Tb927.4.3320_for	GGGGACAAGTTTGTACAAAAAAGCAGGCTATGGCTCTACACGACAACGC	Tb927.4.3320_rev	GGGGACCACTTTGTACAAGAAAGCTGGGTTCATTCGCATGTGCCGAAGTATC
1_known	H22	Tb927.2.5210_for	GGGGACAAGTTTGTACAAAAAAGCAGGCTATGCCATGGAACCTTCAAGGC	Tb927.2.5210_rev	GGGGACCACTTTGTACAAGAAAGCTGGGTTCACCACATTCCCCCATCTACAT
1_known	H23	Tb927.6.710_for	GGGGACAAGTTTGTACAAAAAAGCAGGCTATGCTACTCGTCGGTCTCACC	Tb927.6.710_rev	GGGGACCACTTTGTACAAGAAAGCTGGGTTTAGAATACGATGCGGAGGCAAC
1_known	H24	Tb927.10.6650_for	GGGGACAAGTTTGTACAAAAAAGCAGGCTATGCCGAAAATCCGCCCAG	Tb927.10.6650_rev	GGGGACCACTTTGTACAAGAAAGCTGGGTCTATTTACCCGATTTCACCTCGTAGTC
1_known	I1	Tb927.10.15870_for	GGGGACAAGTTTGTACAAAAAAGCAGGCTATGAACTACGCCGACGGTAACT	Tb927.10.15870_rev	GGGGACCACTTTGTACAAGAAAGCTGGGTTCAAGATCTCCTTGGGACAGCC
1_known	I2	Tb927.10.7850_for	GGGGACAAGTTTGTACAAAAAAGCAGGCTATGGCATGGACATGTTCCGGT	Tb927.10.7850_rev	GGGGACCACTTTGTACAAGAAAGCTGGGTCTAAATACCTCCCCTTTGGTGCTTC
1_known	I4	Tb927.11.3800_for	GGGGACAAGTTTGTACAAAAAAGCAGGCTATGCCCTGTAATGAACAGACCTCT	Tb927.11.3800_rev	GGGGACCACTTTGTACAAGAAAGCTGGGTTTACTCGTACTTCATATGCGCCAAG
1_known	I5	Tb927.8.4070_for	GGGGACAAGTTTGTACAAAAAAGCAGGCTATGACCATCTCGTCTGCGGA	Tb927.8.4070_rev	GGGGACCACTTTGTACAAGAAAGCTGGGTCTAAGGCAACGCAGACCCG
1_known	I6	Tb927.8.3380_for	GGGGACAAGTTTGTACAAAAAAGCAGGCTATGCTTCGTAAAGTCACTAGCCCA	Tb927.8.3380_rev	GGGGACCACTTTGTACAAGAAAGCTGGGTTCATGCGCCTAGTTTCCGC
1_known	I7	Tb927.11.10870_for	GGGGACAAGTTTGTACAAAAAAGCAGGCTATGCGTTACCCGGGGCGA	Tb927.11.10870_rev	GGGGACCACTTTGTACAAGAAAGCTGGGTTCACAACTCTTTTTCTCGAACTAAAATACG
1_known	I10	Tb927.10.11270_for	GGGGACAAGTTTGTACAAAAAAGCAGGCTATGGTGGATCCTTCTGTGCTGC	Tb927.10.11270_rev	GGGGACCACTTTGTACAAGAAAGCTGGGTTCAGTTGTGGACTTGCACCCC
1_known	I11	Tb927.9.11580_for	GGGGACAAGTTTGTACAAAAAAGCAGGCTATGTCTGCTCAAGCCCATACATACC	Tb927.9.11580_rev	GGGGACCACTTTGTACAAGAAAGCTGGGTTCAATAGAATTCACACACAGACGCA
1_known	I12	Tb927.4.2520_for	GGGGACAAGTTTGTACAAAAAAGCAGGCTATGAGGCGGCCCAATGGT	Tb927.4.2520_rev	GGGGACCACTTTGTACAAGAAAGCTGGGTTCATGCCGCGGGACCCTT
1_known	I13	Tb927.9.9120_for	GGGGACAAGTTTGTACAAAAAAGCAGGCTATGACTTCCACAACACAACCGAA	Tb927.9.9120_rev	GGGGACCACTTTGTACAAGAAAGCTGGGTTTATGGCACCCGCACGTG
1_known	I14	Tb927.2.4930_for	GGGGACAAGTTTGTACAAAAAAGCAGGCTATGTCTCACATTCTTTTGTTGGGTGA	Tb927.2.4930_rev	GGGGACCACTTTGTACAAGAAAGCTGGGTTCATTTCATCAACGTCATGAAGTCTGG
1_known	I15	Tb927.9.12690_for	GGGGACAAGTTTGTACAAAAAAGCAGGCTATGCATTGGGGGTATCGGCA	Tb927.9.12690_rev	GGGGACCACTTTGTACAAGAAAGCTGGGTCTAACGAATGAAAGGAATCCCCACC
1_known	I16	Tb927.9.11130_for	GGGGACAAGTTTGTACAAAAAAGCAGGCTATGGCACAAGGAGGGTTCACG	Tb927.9.11130_rev	GGGGACCACTTTGTACAAGAAAGCTGGGTCTAACTGCACCATGTCTGACAAAGG
1_known	I17	Tb927.10.6440_for	GGGGACAAGTTTGTACAAAAAAGCAGGCTATGAAAAGAGTTCTTTTACTCTTTGACGTT	Tb927.10.6440_rev	GGGGACCACTTTGTACAAGAAAGCTGGGTTTACTTCATGGCAATTATTTTTTCCACCTC
1_known	I18	Tb927.4.650_for	GGGGACAAGTTTGTACAAAAAAGCAGGCTATGCGCCGCAGTGCTGTA	Tb927.4.650_rev	GGGGACCACTTTGTACAAGAAAGCTGGGTCTATTGTCCATCAGCATCTTCGAGC
1_known	I19	Tb927.4.4080_for	GGGGACAAGTTTGTACAAAAAAGCAGGCTATGACCACAGTGGTGATGAGCG	Tb927.4.4080_rev	GGGGACCACTTTGTACAAGAAAGCTGGGTTCATGAACCATGGACGCGGG
1_known	I20	Tb927.7.3440_for	GGGGACAAGTTTGTACAAAAAAGCAGGCTATGCTCTGCCCTCCAGATGTT	Tb927.7.3440_rev	GGGGACCACTTTGTACAAGAAAGCTGGGTTTAGCACCCGCAACCTGCC
1_known	I21	Tb927.10.230_for	GGGGACAAGTTTGTACAAAAAAGCAGGCTATGTTCTCCTCCAAAACCGAGTATGA	Tb927.10.230_rev	GGGGACCACTTTGTACAAGAAAGCTGGGTTTAATTTTCCTCGGCCTGCCG
1_known	I22	Tb927.11.9810_for	GGGGACAAGTTTGTACAAAAAAGCAGGCTATGAGGGACCGCTATATCAGAGTTG	Tb927.11.9810_rev	GGGGACCACTTTGTACAAGAAAGCTGGGTTCAGAGAGTTGCTATTTTCAGACTCGT
1_known	I23	Tb927.11.850_for	GGGGACAAGTTTGTACAAAAAAGCAGGCTATGAATACGCACGCGGCC	Tb927.11.850_rev	GGGGACCACTTTGTACAAGAAAGCTGGGTCTATCCCACCATACTACGCTTGC
1_known	I24	Tb927.10.12210_for	GGGGACAAGTTTGTACAAAAAAGCAGGCTATGGTTCACCGTCAAGATGGAGC	Tb927.10.12210_rev	GGGGACCACTTTGTACAAGAAAGCTGGGTCTAACTCGTTGTGGATCCCAGATAAC
1_known	J1	Tb927.3.740_for	GGGGACAAGTTTGTACAAAAAAGCAGGCTATGATGCAACGGGATACCGC	Tb927.3.740_rev	GGGGACCACTTTGTACAAGAAAGCTGGGTTCAATGTAACCCAGTTTGTAGAATGGC
1_known	J2	Tb927.5.288b_for	GGGGACAAGTTTGTACAAAAAAGCAGGCTATGTACAGGTTCGATCATCAAGACAAG	Tb927.5.288b_rev	GGGGACCACTTTGTACAAGAAAGCTGGGTCTATAAGCGAGACTTTAAGTAGTGCTCAA
1_known	J3	Tb927.11.10060_for	GGGGACAAGTTTGTACAAAAAAGCAGGCTATGTCAGCCTTTTTAACACCCAAGA	Tb927.11.10060_rev	GGGGACCACTTTGTACAAGAAAGCTGGGTTTAAAAAAGGTACTCCGCCACAAGC
1_known	J4	Tb927.6.4930_for	GGGGACAAGTTTGTACAAAAAAGCAGGCTATGCTGGTTTCGTACTTGCGT	Tb927.6.4930_rev	GGGGACCACTTTGTACAAGAAAGCTGGGTTCACTGAAGACCGAAAGGCATG
1_known	J5	Tb927.10.4180_for	GGGGACAAGTTTGTACAAAAAAGCAGGCTATGTCTACTGTCCCTCGCCAAA	Tb927.10.4180_rev	GGGGACCACTTTGTACAAGAAAGCTGGGTTTAGCATTGCAATGACGCTAAGTCC
1_known	J6	Tb927.10.3200_for	GGGGACAAGTTTGTACAAAAAAGCAGGCTATGTATCAAGACCGTTGCATATTTTTCTCC	Tb927.10.3200_rev	GGGGACCACTTTGTACAAGAAAGCTGGGTTTATTTTAAGGGGCATTCGCGAGAG
1_known	J7	Tb927.11.14200_for	GGGGACAAGTTTGTACAAAAAAGCAGGCTATGTCTGCAATCCCTTACTTGCAA	Tb927.11.14200_rev	GGGGACCACTTTGTACAAGAAAGCTGGGTCTACATCAATGCCTCCATTAGAGTTGC
1_known	J8	Tb927.11.7020_for	GGGGACAAGTTTGTACAAAAAAGCAGGCTATGAGCTACGATCGCGCAATT	Tb927.11.7020_rev	GGGGACCACTTTGTACAAGAAAGCTGGGTTCAATCCTCTTCGGCAAGGCG
1_known	J9	Tb927.11.13240_for	GGGGACAAGTTTGTACAAAAAAGCAGGCTATGAGCCTTTTCCACAGTTACGC	Tb927.11.13240_rev	GGGGACCACTTTGTACAAGAAAGCTGGGTTCAAAACCACACTACCGGGCC
1_known	J10	Tb927.10.5300_for	GGGGACAAGTTTGTACAAAAAAGCAGGCTATGACACTGCGTACGCGC	Tb927.10.5300_rev	GGGGACCACTTTGTACAAGAAAGCTGGGTTTATGCCAGTTCATCCACCAACG
1_known	J11	Tb927.11.13580_for	GGGGACAAGTTTGTACAAAAAAGCAGGCTATGCAGAGTAGGGTGGTTTCGT	Tb927.11.13580_rev	GGGGACCACTTTGTACAAGAAAGCTGGGTTCATGAATCGTGTAATCCGCCAC
1_known	J12	Tb927.11.14840_for	GGGGACAAGTTTGTACAAAAAAGCAGGCTATGCAGTGGAAGAAAGATGACTTATTCG	Tb927.11.14840_rev	GGGGACCACTTTGTACAAGAAAGCTGGGTCTAGAAATGCAGAGGTAAACTTTCGGT
1_known	J13	Tb927.6.2330_for	GGGGACAAGTTTGTACAAAAAAGCAGGCTATGCCCGCACGTGGCTGT	Tb927.6.2330_rev	GGGGACCACTTTGTACAAGAAAGCTGGGTTTAGCGTAGTGCGTACATCTTCAC
1_known	J14	Tb927.9.9670_for	GGGGACAAGTTTGTACAAAAAAGCAGGCTATGAGCCGAGCTGGGTTTGA	Tb927.9.9670_rev	GGGGACCACTTTGTACAAGAAAGCTGGGTCTAATCAGCCTCCGCAAGTAGAGT
1_known	J15	Tb927.11.3340_for	GGGGACAAGTTTGTACAAAAAAGCAGGCTATGTCCAAGTTATTTATATCTAAACTTCCA	Tb927.11.3340_rev	GGGGACCACTTTGTACAAGAAAGCTGGGTTTACTCCAGCGCAGAAATATACGC
1_known	J16	Tb927.4.4330_for	GGGGACAAGTTTGTACAAAAAAGCAGGCTATGAAGGCGCAACGCTACG	Tb927.4.4330_rev	GGGGACCACTTTGTACAAGAAAGCTGGGTTCATAGCTTTGCCTCTCTCCTCC
1_known	J17	Tb927.11.5520_for	GGGGACAAGTTTGTACAAAAAAGCAGGCTATGTCCAAGCCACAACCCATCG	Tb927.11.5520_rev	GGGGACCACTTTGTACAAGAAAGCTGGGTTCACTGAGTGGCTTTGATGATGTC
1_known	J18	Tb927.7.2180_for	GGGGACAAGTTTGTACAAAAAAGCAGGCTATGTCACAAGTGCCAATTACCGC	Tb927.7.2180_rev	GGGGACCACTTTGTACAAGAAAGCTGGGTTTATTCAACGCTAGACAGCATTTGCT
1_known	J19	Tb927.9.15060_for	GGGGACAAGTTTGTACAAAAAAGCAGGCTATGCCAGTGTACGATGTGGCC	Tb927.9.15060_rev	GGGGACCACTTTGTACAAGAAAGCTGGGTTTAGCGCCTCCGTCGTGC
1_known	J20	Tb927.10.16070_for	GGGGACAAGTTTGTACAAAAAAGCAGGCTATGCAGACCTTACTGAGACCCC	Tb927.10.16070_rev	GGGGACCACTTTGTACAAGAAAGCTGGGTTCACAATTGATACAAGGGTGGGACA
1_known	J21	Tb927.7.2110_for	GGGGACAAGTTTGTACAAAAAAGCAGGCTATGACCAGGCCACGACTTACT	Tb927.7.2110_rev	GGGGACCACTTTGTACAAGAAAGCTGGGTTTAACAGCATAATAAATCATCGAGAGAGGT
1_known	J22	Tb927.2.3720_for	GGGGACAAGTTTGTACAAAAAAGCAGGCTATGCCCGTTTCACCGGCT	Tb927.2.3720_rev	GGGGACCACTTTGTACAAGAAAGCTGGGTTCAAATACGTCTGAGTGCTGCC
1_known	J23	Tb927.1.3280_for	GGGGACAAGTTTGTACAAAAAAGCAGGCTATGGTGCTACAGCATCTTGAACTTC	Tb927.1.3280_rev	GGGGACCACTTTGTACAAGAAAGCTGGGTCTAATTGTGTGAAATTATCCCCTCATCGA
1_known	J24	Tb927.6.2940_for	GGGGACAAGTTTGTACAAAAAAGCAGGCTATGGAAAAAGAAAAAAAAACAGTGAATATT	Tb927.6.2940_rev	GGGGACCACTTTGTACAAGAAAGCTGGGTTTATTGCGGTTCATCCGCGC
1_known	K1	Tb927.11.12080_for	GGGGACAAGTTTGTACAAAAAAGCAGGCTATGCATGGTGCTTTGGTGTTTGG	Tb927.11.12080_rev	GGGGACCACTTTGTACAAGAAAGCTGGGTTCATGCGACCCCCAATTCCT
1_known	K2	Tb927.5.4530_for	GGGGACAAGTTTGTACAAAAAAGCAGGCTATGATGGACTGCAGAGATGCTTCG	Tb927.5.4530_rev	GGGGACCACTTTGTACAAGAAAGCTGGGTTCATTGTGGTTTTTTTTCTATAGCACAACC
1_known	K3	Tb927.8.8040_for	GGGGACAAGTTTGTACAAAAAAGCAGGCTATGAAAGTGCAGGGGTACGCC	Tb927.8.8040_rev	GGGGACCACTTTGTACAAGAAAGCTGGGTTCAGGCCGCCGCCGACGC
1_known	K4	Tb927.10.7450_for	GGGGACAAGTTTGTACAAAAAAGCAGGCTATGCCTGTGATTAAGGAGTACATCAATC	Tb927.10.7450_rev	GGGGACCACTTTGTACAAGAAAGCTGGGTTTACTCGATGTTACCTAAATTCGCTTGC
1_known	K5	Tb927.11.6870_for	GGGGACAAGTTTGTACAAAAAAGCAGGCTATGGCGGGCTTTCAAATACCTG	Tb927.11.6870_rev	GGGGACCACTTTGTACAAGAAAGCTGGGTTCACTCCAATTCCTCCATCGCA
1_known	K6	Tb927.3.2190_for	GGGGACAAGTTTGTACAAAAAAGCAGGCTATGATGGAAGCGACGGAACG	Tb927.3.2190_rev	GGGGACCACTTTGTACAAGAAAGCTGGGTTCAACCCTCAGATGCGGTGA
1_known	K7	Tb927.8.730_for	GGGGACAAGTTTGTACAAAAAAGCAGGCTATGGCGCCCATGTTTCATGG	Tb927.8.730_rev	GGGGACCACTTTGTACAAGAAAGCTGGGTTTAGAACCGCCCCCGCTT
1_known	K8	Tb927.10.6640_for	GGGGACAAGTTTGTACAAAAAAGCAGGCTATGAATACTATGCAGGGAATTATGAGAGAT	Tb927.10.6640_rev	GGGGACCACTTTGTACAAGAAAGCTGGGTTCACACCAACTTCTTCTCCCGC
1_known	K9	Tb927.9.15620_for	GGGGACAAGTTTGTACAAAAAAGCAGGCTATGAGCATCACTTTTCATAACTTATGGCT	Tb927.9.15620_rev	GGGGACCACTTTGTACAAGAAAGCTGGGTTTACAGTACAGAACCGAGAAACAATCC

1_known	K10	Tb927.9.11310_for	GGGGACAAGTTTGTACAAAAAAGCAGGCTATGACAGGGTTCTCATTTGAAAACGT	Tb927.9.11310_rev	GGGGACCACTTTGTACAAGAAAGCTGGGTTCACTCCAATTCGGTGATTGTCAC
1_known	K11	Tb927.8.2500_for	GGGGACAAGTTTGTACAAAAAAGCAGGCTATGCACTTTCCCAGTATCGTGTTT	Tb927.8.2500_rev	GGGGACCACTTTGTACAAGAAAGCTGGGTCTACGGCTTGCGTCGCCG
1_known	K13	Tb927.5.1700_for	GGGGACAAGTTTGTACAAAAAAGCAGGCTATGGAAGGAAGTGGAAGTAACTTCTTTAC	Tb927.5.1700_rev	GGGGACCACTTTGTACAAGAAAGCTGGGTTTAAATGCCAAACTTACAATCATGTGTTTG
1_known	K14	Tb927.9.15560_for	GGGGACAAGTTTGTACAAAAAAGCAGGCTATGAGCATCACTTTTCATAACTTATGGCT	Tb927.9.15560_rev	GGGGACCACTTTGTACAAGAAAGCTGGGTTTACAGTACAGAACTGAGAAACAATCCAG
1_known	K15	Tb927.10.15130_for	GGGGACAAGTTTGTACAAAAAAGCAGGCTATGGCGTCAAAGGGTTGTTTCA	Tb927.10.15130_rev	GGGGACCACTTTGTACAAGAAAGCTGGGTTTAGAACAGGTAGAGGTAGAAGAAGAGAGC
1_known	K16	Tb927.11.9930_for	GGGGACAAGTTTGTACAAAAAAGCAGGCTATGACCGTCGCGCGCGAC	Tb927.11.9930_rev	GGGGACCACTTTGTACAAGAAAGCTGGGTCTATGCGACATCCCCAGGTTTC
1_known	K17	Tb927.2.2180_for	GGGGACAAGTTTGTACAAAAAAGCAGGCTATGGCAACAATATCGAGGCGC	Tb927.2.2180_rev	GGGGACCACTTTGTACAAGAAAGCTGGGTTCATTCTAGGGCCATGCTGCA
1_known	K18	Tb927.10.4450_for	GGGGACAAGTTTGTACAAAAAAGCAGGCTATGTCTGTAGCTGACTTAAAGGCAAAG	Tb927.10.4450_rev	GGGGACCACTTTGTACAAGAAAGCTGGGTTTAACCCCTAACAGCCCGTTGG
1_known	K19	Tb927.5.1280_for	GGGGACAAGTTTGTACAAAAAAGCAGGCTATGTCACGCTACGATATCGGCA	Tb927.5.1280_rev	GGGGACCACTTTGTACAAGAAAGCTGGGTCTACTTTGCTGCTCCATCCTTTGG
1_known	K20	Tb927.7.6660_for	GGGGACAAGTTTGTACAAAAAAGCAGGCTATGTGGCGCGTGGCATTAC	Tb927.7.6660_rev	GGGGACCACTTTGTACAAGAAAGCTGGGTCTAATAACTTAACTCACGTTTGAGGAAGGA
1_known	K21	Tb927.7.4790_for	GGGGACAAGTTTGTACAAAAAAGCAGGCTATGATTGAAGACTTCAGTGAGCACC	Tb927.7.4790_rev	GGGGACCACTTTGTACAAGAAAGCTGGGTTTAGTCTTTGCGGAGCTCAAACAT
1_known	K22	Tb927.8.1890_for	GGGGACAAGTTTGTACAAAAAAGCAGGCTATGGCAGGTAAGAAAGCTCACCC	Tb927.8.1890_rev	GGGGACCACTTTGTACAAGAAAGCTGGGTTTACCATGTCTTCTTCCAGTAGCGG
1_known	K23	Tb927.3.5280_for	GGGGACAAGTTTGTACAAAAAAGCAGGCTATGACCACGTTCAAGCGGGA	Tb927.3.5280_rev	GGGGACCACTTTGTACAAGAAAGCTGGGTCTAACGTACGCGAGCGTAGG
1_known	K24	Tb927.10.4610_for	GGGGACAAGTTTGTACAAAAAAGCAGGCTATGACGGTGAAGTATTCCATTATTGTTCC	Tb927.10.4610_rev	GGGGACCACTTTGTACAAGAAAGCTGGGTCTAAAGAAGAATGTAAAAACAGTAAAAAAT
1_known	L1	Tb927.11.12090_for	GGGGACAAGTTTGTACAAAAAAGCAGGCTATGGGCAAGCTATTTAAACCACCAT	Tb927.11.12090_rev	GGGGACCACTTTGTACAAGAAAGCTGGGTCTATATACGCTTCCGTTTTGGGGC
1_known	L2	Tb927.5.860_for	GGGGACAAGTTTGTACAAAAAAGCAGGCTATGCGCAACCGATTTGTTCGC	Tb927.5.860_rev	GGGGACCACTTTGTACAAGAAAGCTGGGTTTATGCGGTCCTCGTTCCTGC
1_known	L3	Tb927.11.290_for	GGGGACAAGTTTGTACAAAAAAGCAGGCTATGAGTTCGCCTAATTGTTGTCCTG	Tb927.11.290_rev	GGGGACCACTTTGTACAAGAAAGCTGGGTTTACGACCGGGTCAAACACGA
1_known	L4	Tb927.3.1760_for	GGGGACAAGTTTGTACAAAAAAGCAGGCTATGGTGGTGGTGGGGATGAA	Tb927.3.1760_rev	GGGGACCACTTTGTACAAGAAAGCTGGGTTTAAACACCCTTTGGACCGC
1_known	L5	Tb927.2.5660_for	GGGGACAAGTTTGTACAAAAAAGCAGGCTATGGCGGTACTGAGCGAAGA	Tb927.2.5660_rev	GGGGACCACTTTGTACAAGAAAGCTGGGTTTAGCGAAAGCGGTGCTTCTC
1_known	L6	Tb927.9.15610_for	GGGGACAAGTTTGTACAAAAAAGCAGGCTATGAGCATCACTTTTCATAACTTATGGCT	Tb927.9.15610_rev	GGGGACCACTTTGTACAAGAAAGCTGGGTTTACAGTACAGAACCGAGAAACAGTCC
1_known	L7	Tb927.10.2350_for	GGGGACAAGTTTGTACAAAAAAGCAGGCTATGCGTAGGTCGCTTGCG	Tb927.10.2350_rev	GGGGACCACTTTGTACAAGAAAGCTGGGTTTACTTGCCAACCGCTGCG
1_known	L8	Tb927.7.3470_for	GGGGACAAGTTTGTACAAAAAAGCAGGCTATGCGGCGTGTAGTTGCTTC	Tb927.7.3470_rev	GGGGACCACTTTGTACAAGAAAGCTGGGTTCAAGTAGCGAACTGTTTAAGTAAGCG
1_known	L9	Tb927.11.14520_for	GGGGACAAGTTTGTACAAAAAAGCAGGCTATGTTACGGCATTTGTTCCGATGT	Tb927.11.14520_rev	GGGGACCACTTTGTACAAGAAAGCTGGGTTTAGTGGCTCAGGCGAGCG
1_known	L10	Tb927.6.1400_for	GGGGACAAGTTTGTACAAAAAAGCAGGCTATGTCAACGACAAAACTAGAAGGTGT	Tb927.6.1400_rev	GGGGACCACTTTGTACAAGAAAGCTGGGTCTATTCCTCTCCTGCACAACTCAC
1_known	L12	Tb927.4.1220_for	GGGGACAAGTTTGTACAAAAAAGCAGGCTATGGTGGTACCGGTGATGAATATGA	Tb927.4.1220_rev	GGGGACCACTTTGTACAAGAAAGCTGGGTTCACCCATCAGCTACTTTTACCTCG
1_known	L14	Tb927.10.11930_for	GGGGACAAGTTTGTACAAAAAAGCAGGCTATGTTATCAGGAAAGGTGGCGC	Tb927.10.11930_rev	GGGGACCACTTTGTACAAGAAAGCTGGGTCTACTGTGCTGTCCAGCCACC
1_known	L15	Tb927.3.5570_for	GGGGACAAGTTTGTACAAAAAAGCAGGCTATGGACGATCCAAGTTGGCAGC	Tb927.3.5570_rev	GGGGACCACTTTGTACAAGAAAGCTGGGTTCATACTTTATGGTACGCAACGAGC
1_known	L17	Tb927.5.3970_for	GGGGACAAGTTTGTACAAAAAAGCAGGCTATGTCCACCGGCCAACTTTC	Tb927.5.3970_rev	GGGGACCACTTTGTACAAGAAAGCTGGGTTTACACTGAAACAAACCTCGAGTCC
1_known	L18	Tb927.10.830_for	GGGGACAAGTTTGTACAAAAAAGCAGGCTATGGCGGACCTTTCCGATAATAAGT	Tb927.10.830_rev	GGGGACCACTTTGTACAAGAAAGCTGGGTTCAACGCAACAGCCGGCT
1_known	L20	Tb927.10.5290_for	GGGGACAAGTTTGTACAAAAAAGCAGGCTATGCTACGTGGCACACGGC	Tb927.10.5290_rev	GGGGACCACTTTGTACAAGAAAGCTGGGTTCACTCATCCCCATCGGGC
1_known	L21	Tb927.9.15630_for	GGGGACAAGTTTGTACAAAAAAGCAGGCTATGAGCATCACTTTTCATAATTTATGGCTA	Tb927.9.15630_rev	GGGGACCACTTTGTACAAGAAAGCTGGGTTTACAGTACAGAACCGAGAAACAGTCC
1_known	L22	Tb927.3.2840_for	GGGGACAAGTTTGTACAAAAAAGCAGGCTATGCGCCCAACATCAATTATGTTTAAA	Tb927.3.2840_rev	GGGGACCACTTTGTACAAGAAAGCTGGGTTCAGTGAAGCCAGTAACCGGG
1_known	L23	Tb927.6.4960_for	GGGGACAAGTTTGTACAAAAAAGCAGGCTATGTCATTTACGGCTAACTCTGGC	Tb927.6.4960_rev	GGGGACCACTTTGTACAAGAAAGCTGGGTTCACATGTCTTCCGTGCTCTGA
1_known	L24	Tb927.11.9530_for	GGGGACAAGTTTGTACAAAAAAGCAGGCTATGACGGACTGCATCAAGTGGT	Tb927.11.9530_rev	GGGGACCACTTTGTACAAGAAAGCTGGGTTCAGTTCACGGGTTGCTCGT
1_known	M1	Tb927.10.15950_for	GGGGACAAGTTTGTACAAAAAAGCAGGCTATGGACAATGACTTCACTGGAAACTT	Tb927.10.15950_rev	GGGGACCACTTTGTACAAGAAAGCTGGGTTTACCTCTTTGCGTACTGTGCAAT
1_known	M2	Tb927.11.14000_for	GGGGACAAGTTTGTACAAAAAAGCAGGCTATGGCCCCAAAGTCTGCTACA	Tb927.11.14000_rev	GGGGACCACTTTGTACAAGAAAGCTGGGTTCACTGCTTCCTCTTGGCATCC
1_known	M3	Tb927.10.9840_for	GGGGACAAGTTTGTACAAAAAAGCAGGCTATGGTCTCCAAGAAATCACTGTATGATG	Tb927.10.9840_rev	GGGGACCACTTTGTACAAGAAAGCTGGGTTTATTTCTTTGTCTTTTTTCGAGGTGCC
1_known	M4	Tb927.8.2000_for	GGGGACAAGTTTGTACAAAAAAGCAGGCTATGCCCAAGGTAAGCGTCCC	Tb927.8.2000_rev	GGGGACCACTTTGTACAAGAAAGCTGGGTTTATTTCTCTTCCGCCTGGGCG
1_known	M5	Tb927.6.4030_for	GGGGACAAGTTTGTACAAAAAAGCAGGCTATGCGTTTGTGCCGGCGC	Tb927.6.4030_rev	GGGGACCACTTTGTACAAGAAAGCTGGGTTTAAACGACTTGTCCAATAGCCATAAGAT
1_known	M6	Tb927.6.4820_for	GGGGACAAGTTTGTACAAAAAAGCAGGCTATGAGCAACCCTTGGAACGC	Tb927.6.4820_rev	GGGGACCACTTTGTACAAGAAAGCTGGGTTCACCTTTGAATCAAGTTTGAACGGA
1_known	M7	Tb927.9.3470_for	GGGGACAAGTTTGTACAAAAAAGCAGGCTATGTCCACCAGCAGCCGC	Tb927.9.3470_rev	GGGGACCACTTTGTACAAGAAAGCTGGGTCTATGAAATAAATTCCTCCAGTAGCGCT
1_known	M8	Tb927.10.6810_for	GGGGACAAGTTTGTACAAAAAAGCAGGCTATGTTGCCCCCTCCCCAC	Tb927.10.6810_rev	GGGGACCACTTTGTACAAGAAAGCTGGGTTCACAACTTACTGCTCGGTGAGC
1_known	M9	Tb927.11.15170_for	GGGGACAAGTTTGTACAAAAAAGCAGGCTATGGGGAAGTCCAAAGGGCC	Tb927.11.15170_rev	GGGGACCACTTTGTACAAGAAAGCTGGGTTCATACTCCGTTACACTCGCTAACC
1_known	M10	Tb927.11.9390_for	GGGGACAAGTTTGTACAAAAAAGCAGGCTATGAATGGAATGCGGGCGTATAAC	Tb927.11.9390_rev	GGGGACCACTTTGTACAAGAAAGCTGGGTTTATTCAAGTTCGCAAAGGATGCTTCG
1_known	M11	Tb927.10.4130_for	GGGGACAAGTTTGTACAAAAAAGCAGGCTATGCGTTCCTCCCAATTTTTGCT	Tb927.10.4130_rev	GGGGACCACTTTGTACAAGAAAGCTGGGTTCACTTGCCCAGGTCTGACTT
1_known	M12	Tb927.11.10270_for	GGGGACAAGTTTGTACAAAAAAGCAGGCTATGGATAGCAAACAACGAATATGGGAT	Tb927.11.10270_rev	GGGGACCACTTTGTACAAGAAAGCTGGGTTTAGAATAAAAACAACACGAAAATTGTCAT
1_known	M13	Tb927.11.6330_for	GGGGACAAGTTTGTACAAAAAAGCAGGCTATGTCATTCAAGCCAACCATCAGC	Tb927.11.6330_rev	GGGGACCACTTTGTACAAGAAAGCTGGGTTCAAAACTTTGCCAAGTTTTCAGCA
1_known	M14	Tb927.10.16140_for	GGGGACAAGTTTGTACAAAAAAGCAGGCTATGTCACTCCCAATTATGAGTGTGGT	Tb927.10.16140_rev	GGGGACCACTTTGTACAAGAAAGCTGGGTTCAATGGGTTACTTGTTTCATCTGCG
1_known	M15	Tb927.10.8050_for	GGGGACAAGTTTGTACAAAAAAGCAGGCTATGCCGCGGGTTCAGCGA	Tb927.10.8050_rev	GGGGACCACTTTGTACAAGAAAGCTGGGTTCAACGGACTAACAGATTCCACCG
1_known	M16	Tb927.11.7310_for	GGGGACAAGTTTGTACAAAAAAGCAGGCTATGCCTAGTAGGGCAGTTCTTCG	Tb927.11.7310_rev	GGGGACCACTTTGTACAAGAAAGCTGGGTCTAATGCTCGCTGCCAGAAGC
1_known	M17	Tb927.10.6030_for	GGGGACAAGTTTGTACAAAAAAGCAGGCTATGTTTAAAAACCAGTACGACACGGA	Tb927.10.6030_rev	GGGGACCACTTTGTACAAGAAAGCTGGGTTCACTCCTCAACGTCAGGGG
1_known	M18	Tb927.8.6240_for	GGGGACAAGTTTGTACAAAAAAGCAGGCTATGACAACATTGCACACTATTTCCAGC	Tb927.8.6240_rev	GGGGACCACTTTGTACAAGAAAGCTGGGTCTAATCCGCAATTTCTCCTGAGGG
1_known	M19	Tb927.11.15290_for	GGGGACAAGTTTGTACAAAAAAGCAGGCTATGGCAGTTACCCCAGACTACC	Tb927.11.15290_rev	GGGGACCACTTTGTACAAGAAAGCTGGGTCTACTTGGCAGATTGCTGGTCATG
1_known	M20	Tb927.7.2060_for	GGGGACAAGTTTGTACAAAAAAGCAGGCTATGCCTCCCCTCCCACGG	Tb927.7.2060_rev	GGGGACCACTTTGTACAAGAAAGCTGGGTTCATTGGCCACCTTCCTGCA
1_known	M21	Tb927.11.7230_for	GGGGACAAGTTTGTACAAAAAAGCAGGCTATGCCCGTGCTCCCGCCG	Tb927.11.7230_rev	GGGGACCACTTTGTACAAGAAAGCTGGGTTTAGACGAGGGCACATTCATCCT
1_known	M22	Tb927.9.10040_for	GGGGACAAGTTTGTACAAAAAAGCAGGCTATGCTGGACATCAAGATAGAAAAGTATGAC	Tb927.9.10040_rev	GGGGACCACTTTGTACAAGAAAGCTGGGTCTACGGTTCCTCATCGGCTTCC
1_known	M23	Tb927.5.2700_for	GGGGACAAGTTTGTACAAAAAAGCAGGCTATGTCAGAGCATGCAACGACC	Tb927.5.2700_rev	GGGGACCACTTTGTACAAGAAAGCTGGGTCTACTTGTAAAGAAGGTCGTAATGACCC
1_known	M24	Tb927.9.9990_for	GGGGACAAGTTTGTACAAAAAAGCAGGCTATGGCCACTCCCTCCGCC	Tb927.9.9990_rev	GGGGACCACTTTGTACAAGAAAGCTGGGTTCAAGCGTGTAAATTGTTAAGATAAAAGTC
1_known	N1	Tb927.8.2210_for	GGGGACAAGTTTGTACAAAAAAGCAGGCTATGGAAGCTCCCGCTGCA	Tb927.8.2210_rev	GGGGACCACTTTGTACAAGAAAGCTGGGTTTAGGCATGCACAAGGCTTAACC
1_known	N2	Tb927.1.860_for	GGGGACAAGTTTGTACAAAAAAGCAGGCTATGTTGGGGCTGAAGGCGA	Tb927.1.860_rev	GGGGACCACTTTGTACAAGAAAGCTGGGTTCACTGCTTAGGCGCGAAAAT
1_known	N3	Tb927.10.9250_for	GGGGACAAGTTTGTACAAAAAAGCAGGCTATGCCGCCGCCGCCACCA	Tb927.10.9250_rev	GGGGACCACTTTGTACAAGAAAGCTGGGTCTACGCCACCTCATTTGGCTT
1_known	N4	Tb927.11.180_for	GGGGACAAGTTTGTACAAAAAAGCAGGCTATGTTATCTCGCACGGTTCCTGG	Tb927.11.180_rev	GGGGACCACTTTGTACAAGAAAGCTGGGTTCAAATAACTTTAGCTTCATTGTGGAGTTT
1_known	N5	Tb927.3.4810_for	GGGGACAAGTTTGTACAAAAAAGCAGGCTATGTTTTTTGAGGATCAACTCGACGA	Tb927.3.4810_rev	GGGGACCACTTTGTACAAGAAAGCTGGGTTCAAACCAACCTCACGCGTC
1_known	N6	Tb927.10.1520_for	GGGGACAAGTTTGTACAAAAAAGCAGGCTATGCGGTCGTCGGTGCCT	Tb927.10.1520_rev	GGGGACCACTTTGTACAAGAAAGCTGGGTTCAGGAACAACAGCGGTTCCG
1_known	N7	Tb927.4.4650_for	GGGGACAAGTTTGTACAAAAAAGCAGGCTATGTTCACCCTAATAGGTCTGGGAC	Tb927.4.4650_rev	GGGGACCACTTTGTACAAGAAAGCTGGGTTTAATTAAGGTAAAAAAGACGCAAGTGCTC
1_known	N8	Tb927.7.2440_for	GGGGACAAGTTTGTACAAAAAAGCAGGCTATGAAGATATCATTTATTGGATGTGGCAAC	Tb927.7.2440_rev	GGGGACCACTTTGTACAAGAAAGCTGGGTTTAATTCAATCCCTTCTCAAGTTCCTTTGA
1_known	N9	Tb927.10.5230_for	GGGGACAAGTTTGTACAAAAAAGCAGGCTATGAGCAGTTCTGAACACGATAGTC	Tb927.10.5230_rev	GGGGACCACTTTGTACAAGAAAGCTGGGTCTATTCTTCAAGCAACTTCTTCCTCTTTTG
1_known	N10	Tb927.2.2520_for	GGGGACAAGTTTGTACAAAAAAGCAGGCTATGTTTGCACCTGCAAAATCCCT	Tb927.2.2520_rev	GGGGACCACTTTGTACAAGAAAGCTGGGTCTACGAATGGGTAATAAGGACACCG
1_known	N11	Tb927.8.3240_for	GGGGACAAGTTTGTACAAAAAAGCAGGCTATGGAAGTGGCATTCGACGTTT	Tb927.8.3240_rev	GGGGACCACTTTGTACAAGAAAGCTGGGTTTATTCCTGTTTTTGAGCTGAAACTCGA
1_known	N12	Tb927.3.4850_for	GGGGACAAGTTTGTACAAAAAAGCAGGCTATGGATTAAATGTTACGCTCCTGTGC	Tb927.3.4850_rev	GGGGACCACTTTGTACAAGAAAGCTGGGTCTACGAATTGGTGAAGAACGGTTTCC
1_known	N14	Tb927.10.15210_for	GGGGACAAGTTTGTACAAAAAAGCAGGCTATGCGACGGGGAAGTGGA	Tb927.10.15210_rev	GGGGACCACTTTGTACAAGAAAGCTGGGTTTAGTTGAACTCTAACAGTTCATCAATTAA
1_known	N15	Tb927.7.4170_for	GGGGACAAGTTTGTACAAAAAAGCAGGCTATGTTTCCATACGTCACAGACTATAGCG	Tb927.7.4170_rev	GGGGACCACTTTGTACAAGAAAGCTGGGTTCATTTCGACTTTTCGGTCCTCTTT
1_known	N16	Tb927.10.9400_for	GGGGACAAGTTTGTACAAAAAAGCAGGCTATGGGGGAGAACCGTCGAC	Tb927.10.9400_rev	GGGGACCACTTTGTACAAGAAAGCTGGGTCTACAACTCGTCCAGAAACTCCTG
1_known	N17	Tb927.11.15150_for	GGGGACAAGTTTGTACAAAAAAGCAGGCTATGGGAGCCAACTACGCGG	Tb927.11.15150_rev	GGGGACCACTTTGTACAAGAAAGCTGGGTCTAGCGGTCCTTGTGACTATTCTCC
1_known	N18	Tb927.3.3490_for	GGGGACAAGTTTGTACAAAAAAGCAGGCTATGGCAACAGAATTGAAGAAAGGACC	Tb927.3.3490_rev	GGGGACCACTTTGTACAAGAAAGCTGGGTCTACTTGGGTAGTGCTGCCATTTC
1_known	N19	Tb927.9.8680_for	GGGGACAAGTTTGTACAAAAAAGCAGGCTATGCGGCGGGTATCAGGA	Tb927.9.8680_rev	GGGGACCACTTTGTACAAGAAAGCTGGGTTCACCAGTTTGTCCACTCCGG
1_known	N20	Tb927.4.1930_for	GGGGACAAGTTTGTACAAAAAAGCAGGCTATGGCAACATGGGCCGATG	Tb927.4.1930_rev	GGGGACCACTTTGTACAAGAAAGCTGGGTTCAGTTGCGCTGTTTCGCA
1_known	N21	Tb927.8.5540_for	GGGGACAAGTTTGTACAAAAAAGCAGGCTATGATGCGCTTCTTCGTATGGATAAG	Tb927.8.5540_rev	GGGGACCACTTTGTACAAGAAAGCTGGGTTCAAAGCTGCTCGTCAGCG
1_known	N22	Tb927.2.3440_for	GGGGACAAGTTTGTACAAAAAAGCAGGCTATGGCGGCGAGGAAAGCA	Tb927.2.3440_rev	GGGGACCACTTTGTACAAGAAAGCTGGGTTTATTCATACCGCACCCACCCC
1_known	N23	Tb927.6.4050_for	GGGGACAAGTTTGTACAAAAAAGCAGGCTATGGGGTTCCCTTCCGGG	Tb927.6.4050_rev	GGGGACCACTTTGTACAAGAAAGCTGGGTTTAGTGAGAAGCACCGGGTGG
1_known	N24	Tb927.7.6350_for	GGGGACAAGTTTGTACAAAAAAGCAGGCTATGTGGCGTCGAGCTGGG	Tb927.7.6350_rev	GGGGACCACTTTGTACAAGAAAGCTGGGTCTACTTCTTTGCTGCGCCGC
1_known	O1	Tb927.9.1960_for	GGGGACAAGTTTGTACAAAAAAGCAGGCTATGCGCGTTACTTTGTGTCAGA	Tb927.9.1960_rev	GGGGACCACTTTGTACAAGAAAGCTGGGTCTACTTCCAAGTTAAAGAATAAAGATCATG
1_known	O2	Tb927.9.6940_for	GGGGACAAGTTTGTACAAAAAAGCAGGCTATGTCTTCGGCTAACTCGGAGG	Tb927.9.6940_rev	GGGGACCACTTTGTACAAGAAAGCTGGGTTCAAAACGTAAAGTCCCTTGCCTT
1_known	O3	Tb927.9.6060_for	GGGGACAAGTTTGTACAAAAAAGCAGGCTATGTCGAAACAGGAGCCGTTG	Tb927.9.6060_rev	GGGGACCACTTTGTACAAGAAAGCTGGGTTCACGCTGATGATTTCTTATCATCAGG
1_known	O4	Tb927.11.15350_for	GGGGACAAGTTTGTACAAAAAAGCAGGCTATGTCCTCCACACAGACGACG	Tb927.11.15350_rev	GGGGACCACTTTGTACAAGAAAGCTGGGTCTACTCAAGGTCTGCCGCAAG
1_known	O5	Tb927.10.13490_for	GGGGACAAGTTTGTACAAAAAAGCAGGCTATGACACCTCCTGTGCGGC	Tb927.10.13490_rev	GGGGACCACTTTGTACAAGAAAGCTGGGTTCACGCCGACTCGTTCAGA
1_known	O6	Tb927.10.6610_for	GGGGACAAGTTTGTACAAAAAAGCAGGCTATGAACTCTATGGATGATGCGGCA	Tb927.10.6610_rev	GGGGACCACTTTGTACAAGAAAGCTGGGTCTAACGGTGCATGGCTTGACG
1_known	O7	Tb927.6.1860_for	GGGGACAAGTTTGTACAAAAAAGCAGGCTATGCGTCGGATGCTGCTAGC	Tb927.6.1860_rev	GGGGACCACTTTGTACAAGAAAGCTGGGTCTATGCTACAACGTCTGATTCGTCG
1_known	O8	Tb927.11.8820_for	GGGGACAAGTTTGTACAAAAAAGCAGGCTATGGCGAAAGGAAGCGATTATGAT	Tb927.11.8820_rev	GGGGACCACTTTGTACAAGAAAGCTGGGTCTACGTCTTGTACCGCATCCAG
1_known	O9	Tb927.3.3630_for	GGGGACAAGTTTGTACAAAAAAGCAGGCTATGTTGTTTCGTACGATTCGGCA	Tb927.3.3630_rev	GGGGACCACTTTGTACAAGAAAGCTGGGTTTACTTTGTTCCTGCCACCGGC
1_known	O10	Tb927.9.5280_for	GGGGACAAGTTTGTACAAAAAAGCAGGCTATGCTCCGCGGTACCCCA	Tb927.9.5280_rev	GGGGACCACTTTGTACAAGAAAGCTGGGTTCACTTCCCACCTCCTTCCCT
1_known	O11	Tb927.11.14430_for	GGGGACAAGTTTGTACAAAAAAGCAGGCTATGTTGACCACCACCGACGT	Tb927.11.14430_rev	GGGGACCACTTTGTACAAGAAAGCTGGGTCTACACAATGCGCTGCAGATCA
1_known	O12	Tb927.6.3970_for	GGGGACAAGTTTGTACAAAAAAGCAGGCTATGTCCCTCTTTACAGTGCATTGC	Tb927.6.3970_rev	GGGGACCACTTTGTACAAGAAAGCTGGGTCTAGTGATCAATTCCACTGCTAGTCATC
1_known	O13	Tb927.10.1130_for	GGGGACAAGTTTGTACAAAAAAGCAGGCTATGCAACCTTACGGGATGTCAAC	Tb927.10.1130_rev	GGGGACCACTTTGTACAAGAAAGCTGGGTTTACGGAGTTGAAGGCATCGAAATC
1_known	O14	Tb927.11.3900_for	GGGGACAAGTTTGTACAAAAAAGCAGGCTATGTTTTGCCGTTCCGTGAAGT	Tb927.11.3900_rev	GGGGACCACTTTGTACAAGAAAGCTGGGTTCAGCGAACGGACGTCCC
1_known	O15	Tb927.10.13480_for	GGGGACAAGTTTGTACAAAAAAGCAGGCTATGGAAAATTTTGTTCTTCCACCGTACA	Tb927.10.13480_rev	GGGGACCACTTTGTACAAGAAAGCTGGGTTTACGCAGCCTCCTTTAGACACT
1_known	O16	Tb927.10.5130_for	GGGGACAAGTTTGTACAAAAAAGCAGGCTATGATCCTTAAGGAAGAGACCGTACTG	Tb927.10.5130_rev	GGGGACCACTTTGTACAAGAAAGCTGGGTTCATGTTCGGTCCCGCACT
1_known	O17	Tb927.3.5330_for	GGGGACAAGTTTGTACAAAAAAGCAGGCTATGACGGAAAAGGAAAATGGAAACAAAG	Tb927.3.5330_rev	GGGGACCACTTTGTACAAGAAAGCTGGGTTCAAGAAAAGCCAAACTGCTCCA
1_known	O18	Tb927.1.570_for	GGGGACAAGTTTGTACAAAAAAGCAGGCTATGGACGCACGTGTTCATCG	Tb927.1.570_rev	GGGGACCACTTTGTACAAGAAAGCTGGGTTCACGATCGCTTGATGTAAAGATAAAATG
1_known	O19	Tb927.4.3130_for	GGGGACAAGTTTGTACAAAAAAGCAGGCTATGTCGGTTCCACTCACAGAGG	Tb927.4.3130_rev	GGGGACCACTTTGTACAAGAAAGCTGGGTTCACATGTGACCAAATGTGCTCC
1_known	O20	Tb927.11.4700_for	GGGGACAAGTTTGTACAAAAAAGCAGGCTATGGCTCTCACTCAATCCCTAAAACT	Tb927.11.4700_rev	GGGGACCACTTTGTACAAGAAAGCTGGGTTCAAAAGTCGTTCATGAAGACCTCC
1_known	O21	Tb927.6.2890_for	GGGGACAAGTTTGTACAAAAAAGCAGGCTATGAGAGGGCCTGTTACTATATTACTACAG	Tb927.6.2890_rev	GGGGACCACTTTGTACAAGAAAGCTGGGTCTAGAGTATACGCTGCTCTGCAGT
1_known	O22	Tb927.7.880_for	GGGGACAAGTTTGTACAAAAAAGCAGGCTATGAACAAACTTACATTTACGCCACTTAC	Tb927.7.880_rev	GGGGACCACTTTGTACAAGAAAGCTGGGTTTACGACTCTTCATTTCCTGCAACA
1_known	O23	Tb927.10.4000_for	GGGGACAAGTTTGTACAAAAAAGCAGGCTATGCGCCGTTGTCCGGTT	Tb927.10.4000_rev	GGGGACCACTTTGTACAAGAAAGCTGGGTTCATTTACCAACATATCTGGGTGGTCG
1_known	O24	Tb927.11.12750_for	GGGGACAAGTTTGTACAAAAAAGCAGGCTATGTTTACTGACAACGCTGCCC	Tb927.11.12750_rev	GGGGACCACTTTGTACAAGAAAGCTGGGTTTACTGCCTTCCCGTTGCATC
1_known	P1	Tb927.11.5170_for	GGGGACAAGTTTGTACAAAAAAGCAGGCTATGTTGAATGCGGCGCTTTACG	Tb927.11.5170_rev	GGGGACCACTTTGTACAAGAAAGCTGGGTTCAGCATCGTTTCACGCCTAC
1_known	P2	Tb927.9.1680_for	GGGGACAAGTTTGTACAAAAAAGCAGGCTATGGAGGCGAAGCATGCG	Tb927.9.1680_rev	GGGGACCACTTTGTACAAGAAAGCTGGGTTCACTTGGAAATACGCAGCCG
1_known	P3	Tb927.8.4810_for	GGGGACAAGTTTGTACAAAAAAGCAGGCTATGAGCTTCAGATTTGTCCAGCG	Tb927.8.4810_rev	GGGGACCACTTTGTACAAGAAAGCTGGGTTCAATGTGCCTGCTCAGATGC
1_known	P5	Tb927.8.4830_for	GGGGACAAGTTTGTACAAAAAAGCAGGCTATGCAGACCCCTGGTGAGG	Tb927.8.4830_rev	GGGGACCACTTTGTACAAGAAAGCTGGGTTCACGTAATACTCATGATATCATCCAGAAG
1_known	P6	Tb927.11.12340_for	GGGGACAAGTTTGTACAAAAAAGCAGGCTATGCTACGAAATTACGACACACCC	Tb927.11.12340_rev	GGGGACCACTTTGTACAAGAAAGCTGGGTTTAGGAGTCAACTTCTCGCCTGC
1_known	P7	Tb927.11.8440_for	GGGGACAAGTTTGTACAAAAAAGCAGGCTATGACCTACCGTGCAGCTGT	Tb927.11.8440_rev	GGGGACCACTTTGTACAAGAAAGCTGGGTCTAGTCTTCAATATTGAACAGTTCGCGA
1_known	P8	Tb927.11.14020_for	GGGGACAAGTTTGTACAAAAAAGCAGGCTATGGCCCCAAAGTCTGCTGC	Tb927.11.14020_rev	GGGGACCACTTTGTACAAGAAAGCTGGGTTTACTTCCTCTTGGCATCCTTCTTTTC
1_known	P9	Tb927.4.3680_for	GGGGACAAGTTTGTACAAAAAAGCAGGCTATGTTTACACCGTCGACCCGA	Tb927.4.3680_rev	GGGGACCACTTTGTACAAGAAAGCTGGGTTCATGTCTTGTGCACATCGAGAAG
1_known	P10	Tb927.2.5230_for	GGGGACAAGTTTGTACAAAAAAGCAGGCTATGGAGCGGTACAAGCTGCG	Tb927.2.5230_rev	GGGGACCACTTTGTACAAGAAAGCTGGGTTTACACAACTAACCAAGGGTGCC
1_known	P11	Tb927.2.3460_for	GGGGACAAGTTTGTACAAAAAAGCAGGCTATGGAAAGAGAGCCAGCGGT	Tb927.2.3460_rev	GGGGACCACTTTGTACAAGAAAGCTGGGTTTATTCATACCGCACCCACCCC
1_known	P12	Tb927.7.5680_for	GGGGACAAGTTTGTACAAAAAAGCAGGCTATGACCGACCTTCACATGAGAGA	Tb927.7.5680_rev	GGGGACCACTTTGTACAAGAAAGCTGGGTTTAGTATTTACTGCGGGAGCGGT
1_known	P13	Tb927.10.12800_for	GGGGACAAGTTTGTACAAAAAAGCAGGCTATGACCACAGCAACTGATGTGAC	Tb927.10.12800_rev	GGGGACCACTTTGTACAAGAAAGCTGGGTTTACAGCTGCTGGTAAGGATTATGTCG
1_known	P14	Tb927.7.1720_for	GGGGACAAGTTTGTACAAAAAAGCAGGCTATGATAAAGTTCACTCTGCGGTACTTC	Tb927.7.1720_rev	GGGGACCACTTTGTACAAGAAAGCTGGGTCTAGTTTGATTGTGAGGCGCTCA
1_known	P15	Tb927.4.360_for	GGGGACAAGTTTGTACAAAAAAGCAGGCTATGGAACCCGACACGGATGC	Tb927.4.360_rev	GGGGACCACTTTGTACAAGAAAGCTGGGTCTACCTGAAGCCAAGTTCCTTTCC
1_known	P16	Tb927.11.16770_for	GGGGACAAGTTTGTACAAAAAAGCAGGCTATGCGTATCGTCATTTCCCCCG	Tb927.11.16770_rev	GGGGACCACTTTGTACAAGAAAGCTGGGTTCACTTTGGTGGTGGTGCTTC
1_known	P17	Tb927.5.4320_for	GGGGACAAGTTTGTACAAAAAAGCAGGCTATGGAAGAGGGCGGTGAAGG	Tb927.5.4320_rev	GGGGACCACTTTGTACAAGAAAGCTGGGTTTACGTGTCCTCAGAGTGTTGTTG
1_known	P18	Tb927.11.12570_for	GGGGACAAGTTTGTACAAAAAAGCAGGCTATGCAGCTAAAGGAGCGCC	Tb927.11.12570_rev	GGGGACCACTTTGTACAAGAAAGCTGGGTTCAGACCGCCAACAAATCACTTG
1_known	P19	Tb927.8.6390_for	GGGGACAAGTTTGTACAAAAAAGCAGGCTATGTTTGGCACGCCGGTT	Tb927.8.6390_rev	GGGGACCACTTTGTACAAGAAAGCTGGGTTTACGATTTCGATGAAGGTCCGGG
1_known	P20	Tb927.11.7150_for	GGGGACAAGTTTGTACAAAAAAGCAGGCTATGACTTCTCTTATCAGTCGAGTAGCA	Tb927.11.7150_rev	GGGGACCACTTTGTACAAGAAAGCTGGGTTCACCAAACAACAAGTGGATCCTTG
1_known	P21	Tb927.11.14030_for	GGGGACAAGTTTGTACAAAAAAGCAGGCTATGGCAGGTTTCACCGAGACA	Tb927.11.14030_rev	GGGGACCACTTTGTACAAGAAAGCTGGGTTCACTCCACCGATGGTGCA
1_known	P22	Tb927.1.3110_for	GGGGACAAGTTTGTACAAAAAAGCAGGCTATGTCTGCTGAGTCCATATACCGC	Tb927.1.3110_rev	GGGGACCACTTTGTACAAGAAAGCTGGGTCTACAACAGACTACTCATTTTGTTCTTCAC
1_known	P23	Tb927.6.1260_for	GGGGACAAGTTTGTACAAAAAAGCAGGCTATGTTGAGACGCCCCCCC	Tb927.6.1260_rev	GGGGACCACTTTGTACAAGAAAGCTGGGTCTACTGAGAAGTCATTAGGTTCTGGCA
1_known	P24	Tb927.11.5290_for	GGGGACAAGTTTGTACAAAAAAGCAGGCTATGGAAGTTGTTGTAAGTTTTTTAAGTGGA	Tb927.11.5290_rev	GGGGACCACTTTGTACAAGAAAGCTGGGTCTACATCCCACAGAGATTGTTTAGTGTG
2_hypothetical	A1	Tb927.10.780_for	GGGGACAAGTTTGTACAAAAAAGCAGGCTATGTATTTTTGCCACGGCTGCC	Tb927.10.780_rev	GGGGACCACTTTGTACAAGAAAGCTGGGTCTATGCTGTGGTGTTCGTGTCTG
2_hypothetical	A2	Tb927.11.7850_for	GGGGACAAGTTTGTACAAAAAAGCAGGCTATGGTGGTGTCTATGCAGAGGT	Tb927.11.7850_rev	GGGGACCACTTTGTACAAGAAAGCTGGGTCTACCCGAGCCGCCTAATAAGG

2_hypothetical	A3	Tb927.10.1280_for	GGGGACAAGTTTGTACAAAAAAGCAGGCTATGGGAATGAGAAAAAAGGAAACAATAAAG	Tb927.10.1280_rev	GGGGACCACTTTGTACAAGAAAGCTGGGTCTATGCCTTCTTCGATACGTCGTC
2_hypothetical	A4	Tb927.3.2350_for	GGGGACAAGTTTGTACAAAAAAGCAGGCTATGGATCCGGCGGTAGGG	Tb927.3.2350_rev	GGGGACCACTTTGTACAAGAAAGCTGGGTCTACCGTTTCCGCTTCAATTTCTCG
2_hypothetical	A5	Tb927.10.150_for	GGGGACAAGTTTGTACAAAAAAGCAGGCTATGAGCACTAGTGAACGGCGT	Tb927.10.150_rev	GGGGACCACTTTGTACAAGAAAGCTGGGTCTAGAGAGAATCAAAAAAATCATCAATATC
2_hypothetical	A6	Tb927.5.1420_for	GGGGACAAGTTTGTACAAAAAAGCAGGCTATGTTCAGATATCATGAGAGTATAAGGGAA	Tb927.5.1420_rev	GGGGACCACTTTGTACAAGAAAGCTGGGTCTACCTAACAACTTGTTCATTATGAGTTGA
2_hypothetical	A7	Tb927.5.1400_for	GGGGACAAGTTTGTACAAAAAAGCAGGCTATGTTCAGATATCATGAGAGTATAAGGGAA	Tb927.5.1400_rev	GGGGACCACTTTGTACAAGAAAGCTGGGTCTACCTAACAACTTGGTCATTATGAGTAGA
2_hypothetical	A8	Tb927.7.6840_for	GGGGACAAGTTTGTACAAAAAAGCAGGCTATGGAGAAGTTGACACTCTCGGC	Tb927.7.6840_rev	GGGGACCACTTTGTACAAGAAAGCTGGGTTTACCCGCGGGGAAGATTGT
2_hypothetical	A9	Tb927.8.5670_for	GGGGACAAGTTTGTACAAAAAAGCAGGCTATGGTAGAGAAGAAGCAAGCAAAGAAA	Tb927.8.5670_rev	GGGGACCACTTTGTACAAGAAAGCTGGGTTCACTTGTCACTGTCTGTGTCACT
2_hypothetical	A10	Tb927.11.4810_for	GGGGACAAGTTTGTACAAAAAAGCAGGCTATGAGGGCTCTCCGCCTAAC	Tb927.11.4810_rev	GGGGACCACTTTGTACAAGAAAGCTGGGTCTAGTGTGAACGCCGCGG
2_hypothetical	A11	Tb927.4.2150_for	GGGGACAAGTTTGTACAAAAAAGCAGGCTATGATTGTCGGCCAAATGAGAAGG	Tb927.4.2150_rev	GGGGACCACTTTGTACAAGAAAGCTGGGTTTACCTTTTCACACGGGAAAGGAC
2_hypothetical	A12	Tb927.7.260_for	GGGGACAAGTTTGTACAAAAAAGCAGGCTATGACGGAGCGTTCACCGG	Tb927.7.260_rev	GGGGACCACTTTGTACAAGAAAGCTGGGTTCACTTTATGACTGCTTCAATAACCCC
2_hypothetical	A13	Tb927.11.420_for	GGGGACAAGTTTGTACAAAAAAGCAGGCTATGACGTCTAAATCTATTGACTCCATGATG	Tb927.11.420_rev	GGGGACCACTTTGTACAAGAAAGCTGGGTTCAAAGCATCATATCTCTTTTCTTTAAACG
2_hypothetical	A14	Tb927.2.5040_for	GGGGACAAGTTTGTACAAAAAAGCAGGCTATGCAGAGGGACGCTGACA	Tb927.2.5040_rev	GGGGACCACTTTGTACAAGAAAGCTGGGTCTATAAACTAACCTCCGCAAGCAAGC
2_hypothetical	A15	Tb927.9.10660_for	GGGGACAAGTTTGTACAAAAAAGCAGGCTATGAGCGGAGCTGACGATGA	Tb927.9.10660_rev	GGGGACCACTTTGTACAAGAAAGCTGGGTTCAGTCCTGCTTCTTGTCGGG
2_hypothetical	A16	Tb927.8.520_for	GGGGACAAGTTTGTACAAAAAAGCAGGCTATGCGGGTGTGGCGTTTAAG	Tb927.8.520_rev	GGGGACCACTTTGTACAAGAAAGCTGGGTTTAGAAGAAAAGAAAAATAACAATTATCAA
2_hypothetical	A17	Tb927.3.5470_for	GGGGACAAGTTTGTACAAAAAAGCAGGCTATGACTGGGCGGAAGCGT	Tb927.3.5470_rev	GGGGACCACTTTGTACAAGAAAGCTGGGTTTACTTTTCACCTATGCCATTGCTATCC
2_hypothetical	A18	Tb927.4.1380_for	GGGGACAAGTTTGTACAAAAAAGCAGGCTATGACCGTTCGTGTAGCACTCC	Tb927.4.1380_rev	GGGGACCACTTTGTACAAGAAAGCTGGGTTCACAAGTCTAAAACATGTGTGAGAGG
2_hypothetical	A19	Tb927.4.5360_for	GGGGACAAGTTTGTACAAAAAAGCAGGCTATGTACGCTGTGAAACCGTGG	Tb927.4.5360_rev	GGGGACCACTTTGTACAAGAAAGCTGGGTTCACGAGCGAGGCAAGTGA
2_hypothetical	A20	Tb927.9.3850_for	GGGGACAAGTTTGTACAAAAAAGCAGGCTATGGCCAAGGATACGGCGA	Tb927.9.3850_rev	GGGGACCACTTTGTACAAGAAAGCTGGGTTTACACTCTCTTCTTGCGCTGTTG
2_hypothetical	A21	Tb927.4.3500_for	GGGGACAAGTTTGTACAAAAAAGCAGGCTATGTGCTGCGGTTGGTTGAC	Tb927.4.3500_rev	GGGGACCACTTTGTACAAGAAAGCTGGGTCTACAGGCAACAACCCGCG
2_hypothetical	A22	Tb927.1.2420_for	GGGGACAAGTTTGTACAAAAAAGCAGGCTATGAGGTGGCCGGCTGAG	Tb927.1.2420_rev	GGGGACCACTTTGTACAAGAAAGCTGGGTTCACAATTTCACAGTTACGGCTTTGA
2_hypothetical	A23	Tb927.6.2620_for	GGGGACAAGTTTGTACAAAAAAGCAGGCTATGCGCCGTGATTGTGTAAGAT	Tb927.6.2620_rev	GGGGACCACTTTGTACAAGAAAGCTGGGTTCACGAATCAGATGTTAGGTGACCC
2_hypothetical	A24	Tb927.8.4520_for	GGGGACAAGTTTGTACAAAAAAGCAGGCTATGATAGACCTCACTAAGCAGATGACG	Tb927.8.4520_rev	GGGGACCACTTTGTACAAGAAAGCTGGGTTTACACTTTTCCAGCGAGCGG
2_hypothetical	B1	Tb927.11.11180_for	GGGGACAAGTTTGTACAAAAAAGCAGGCTATGTCGGTGAAGGTTTGGGTAAAAT	Tb927.11.11180_rev	GGGGACCACTTTGTACAAGAAAGCTGGGTTCAAAAGATAGTACTCCGGCAGCT
2_hypothetical	B2	Tb927.5.2810_for	GGGGACAAGTTTGTACAAAAAAGCAGGCTATGTCACCTGCCTCACCACT	Tb927.5.2810_rev	GGGGACCACTTTGTACAAGAAAGCTGGGTTTACTTTGTGTTATCACCCTCCTTCTTG
2_hypothetical	B3	Tb927.6.3210_for	GGGGACAAGTTTGTACAAAAAAGCAGGCTATGCAACAGCCGAAAGGAATCA	Tb927.6.3210_rev	GGGGACCACTTTGTACAAGAAAGCTGGGTCTAAACTGCCTGAATGGAACCGAC
2_hypothetical	B4	Tb927.1.4710_for	GGGGACAAGTTTGTACAAAAAAGCAGGCTATGAGCGGATTGCCTGGAAGT	Tb927.1.4710_rev	GGGGACCACTTTGTACAAGAAAGCTGGGTTTACTCCGTCGACTGGTCCTC
2_hypothetical	B5	Tb927.8.4420_for	GGGGACAAGTTTGTACAAAAAAGCAGGCTATGGAAGGAAGTGGTAGCTACCG	Tb927.8.4420_rev	GGGGACCACTTTGTACAAGAAAGCTGGGTTCAGCATACAAACTCCTTCTCACCA
2_hypothetical	B6	Tb927.4.3520_for	GGGGACAAGTTTGTACAAAAAAGCAGGCTATGCTGTGTTGCGGCTGTT	Tb927.4.3520_rev	GGGGACCACTTTGTACAAGAAAGCTGGGTCTAAAGGCAACAGTGCGTGATTG
2_hypothetical	B7	Tb927.11.9405_for	GGGGACAAGTTTGTACAAAAAAGCAGGCTATGGAAGGGGAGGAGAAGGTAGA	Tb927.11.9405_rev	GGGGACCACTTTGTACAAGAAAGCTGGGTTCAGATGCGGGTCCCACAA
2_hypothetical	B8	Tb927.10.6420_for	GGGGACAAGTTTGTACAAAAAAGCAGGCTATGGGGTGCGCACCTCTT	Tb927.10.6420_rev	GGGGACCACTTTGTACAAGAAAGCTGGGTTTACTGAAAGCGCCCCTTATATGTTT
2_hypothetical	B9	Tb927.10.8370_for	GGGGACAAGTTTGTACAAAAAAGCAGGCTATGCGGAGGAAAACGACACTAAAT	Tb927.10.8370_rev	GGGGACCACTTTGTACAAGAAAGCTGGGTTTAGATAGGTTTTTTTCCTGCCTTTATGTT
2_hypothetical	B10	Tb927.1.4020_for	GGGGACAAGTTTGTACAAAAAAGCAGGCTATGCATTTCATTATTTTAATGTGCTCCCG	Tb927.1.4020_rev	GGGGACCACTTTGTACAAGAAAGCTGGGTTTAAACTTCTAAACCTGATAGAAAAAAAAA
2_hypothetical	B11	Tb927.10.6020_for	GGGGACAAGTTTGTACAAAAAAGCAGGCTATGGCGAAAAAGACCACCAAGAA	Tb927.10.6020_rev	GGGGACCACTTTGTACAAGAAAGCTGGGTTCATGAACATAGCAACTCCATCAGTG
2_hypothetical	B12	Tb927.10.16050_for	GGGGACAAGTTTGTACAAAAAAGCAGGCTATGTCAGATGGACTTCCGTGGC	Tb927.10.16050_rev	GGGGACCACTTTGTACAAGAAAGCTGGGTTCACAGTACATCACACAGGTTTTCTC
2_hypothetical	B13	Tb927.5.2910_for	GGGGACAAGTTTGTACAAAAAAGCAGGCTATGTTAGTCGAACGATCGTACAAAACC	Tb927.5.2910_rev	GGGGACCACTTTGTACAAGAAAGCTGGGTTTAAGTACTTAGTCCCTCAACAAGTCGC
2_hypothetical	B14	Tb927.8.6460_for	GGGGACAAGTTTGTACAAAAAAGCAGGCTATGGAACGAATTGGTGAGTTTACCG	Tb927.8.6460_rev	GGGGACCACTTTGTACAAGAAAGCTGGGTTCAAATACCGCTTGTGGCTAACC
2_hypothetical	B15	Tb927.2.2540_for	GGGGACAAGTTTGTACAAAAAAGCAGGCTATGGTGAAGCCTCGTTCTCAGG	Tb927.2.2540_rev	GGGGACCACTTTGTACAAGAAAGCTGGGTTCATTCCCTTTTAACCCTCCCTACA
2_hypothetical	B16	Tb927.10.9550_for	GGGGACAAGTTTGTACAAAAAAGCAGGCTATGCTTGCGCAGCAAGGT	Tb927.10.9550_rev	GGGGACCACTTTGTACAAGAAAGCTGGGTTCACTTTAGGAAGGGTAGCCGG
2_hypothetical	B17	Tb927.11.13330_for	GGGGACAAGTTTGTACAAAAAAGCAGGCTATGGCTGCTTGCGATGTGAC	Tb927.11.13330_rev	GGGGACCACTTTGTACAAGAAAGCTGGGTTTAGTATGCGAACGGGTCAACTTG
2_hypothetical	B18	Tb927.10.12750_for	GGGGACAAGTTTGTACAAAAAAGCAGGCTATGTGCGTATATAATTATATCATTTATATT	Tb927.10.12750_rev	GGGGACCACTTTGTACAAGAAAGCTGGGTTCACAGAAACAAAATAATGTTAAAAAAGAA
2_hypothetical	B19	Tb927.5.3180_for	GGGGACAAGTTTGTACAAAAAAGCAGGCTATGTCAAGCAATGAAAAGGTCAGTAGT	Tb927.5.3180_rev	GGGGACCACTTTGTACAAGAAAGCTGGGTTCATTGTGATTGACGTGCGATTCC
2_hypothetical	B20	Tb927.1.5260_for	GGGGACAAGTTTGTACAAAAAAGCAGGCTATGTGCAGCTCCTCAAGCTTTT	Tb927.1.5260_rev	GGGGACCACTTTGTACAAGAAAGCTGGGTCTACATCTTAATTGCGGGAGGTGC
2_hypothetical	B21	Tb927.11.10400_for	GGGGACAAGTTTGTACAAAAAAGCAGGCTATGCTTCGGTTCACTCATAGGGC	Tb927.11.10400_rev	GGGGACCACTTTGTACAAGAAAGCTGGGTTCACGAAGGGGTGCCTGTT
2_hypothetical	B22	Tb927.9.8810_for	GGGGACAAGTTTGTACAAAAAAGCAGGCTATGCCTCCCCGCAGCGCG	Tb927.9.8810_rev	GGGGACCACTTTGTACAAGAAAGCTGGGTCTAACTTTCTTGCAGGCTCGCT
2_hypothetical	B23	Tb927.9.2910_for	GGGGACAAGTTTGTACAAAAAAGCAGGCTATGTCCGGTAGGGGTCATGG	Tb927.9.2910_rev	GGGGACCACTTTGTACAAGAAAGCTGGGTTTATCCCTTCCCACGTGCCC
2_hypothetical	B24	Tb927.7.320_for	GGGGACAAGTTTGTACAAAAAAGCAGGCTATGTATACCAGTGGCCATCCGA	Tb927.7.320_rev	GGGGACCACTTTGTACAAGAAAGCTGGGTCTAAATCACGCGACTCTCCGG
2_hypothetical	C1	Tb927.10.14540_for	GGGGACAAGTTTGTACAAAAAAGCAGGCTATGTTTGAGGGGTATTTAAAGTGGTGG	Tb927.10.14540_rev	GGGGACCACTTTGTACAAGAAAGCTGGGTTCATTCCTTCCCTTGTTTTAATGTTGCG
2_hypothetical	C2	Tb927.11.13510_for	GGGGACAAGTTTGTACAAAAAAGCAGGCTATGAACCCGTCAAAGGTAGTCCA	Tb927.11.13510_rev	GGGGACCACTTTGTACAAGAAAGCTGGGTTTAGGCTGTCAGGATTCCCTCTTT
2_hypothetical	C3	Tb927.6.4330_for	GGGGACAAGTTTGTACAAAAAAGCAGGCTATGGCAGAGGGCAGTGACA	Tb927.6.4330_rev	GGGGACCACTTTGTACAAGAAAGCTGGGTTCAAGTGGTAGATTCAGTTTCAGACG
2_hypothetical	C4	Tb927.7.2740_for	GGGGACAAGTTTGTACAAAAAAGCAGGCTATGGAATACCAAGGTTCTCCACAGC	Tb927.7.2740_rev	GGGGACCACTTTGTACAAGAAAGCTGGGTCTAAAAAAAAACTTCATTAGCATATCGGCA
2_hypothetical	C5	Tb927.11.3060_for	GGGGACAAGTTTGTACAAAAAAGCAGGCTATGAACATTATACTAATAGGACTCGTTATG	Tb927.11.3060_rev	GGGGACCACTTTGTACAAGAAAGCTGGGTCTACTGGCCTTTCCTGCCCT
2_hypothetical	C6	Tb927.11.3530_for	GGGGACAAGTTTGTACAAAAAAGCAGGCTATGTTCAAACAACTCTACAAACGTGAC	Tb927.11.3530_rev	GGGGACCACTTTGTACAAGAAAGCTGGGTTCACCAACCCTGGGGAAACA
2_hypothetical	C7	Tb927.11.17860_for	GGGGACAAGTTTGTACAAAAAAGCAGGCTATGTTGTACGGTGGCACCTGC	Tb927.11.17860_rev	GGGGACCACTTTGTACAAGAAAGCTGGGTCTAATCCCGGAGGAACGTGGG
2_hypothetical	C8	Tb927.11.6520_for	GGGGACAAGTTTGTACAAAAAAGCAGGCTATGACGACTTTAGAAAAGCGGATCG	Tb927.11.6520_rev	GGGGACCACTTTGTACAAGAAAGCTGGGTTCACTCTTTCTTGACCTCATCGAATG
2_hypothetical	C9	Tb927.2.3090_for	GGGGACAAGTTTGTACAAAAAAGCAGGCTATGAGGGAGGGGGATCGAACC	Tb927.2.3090_rev	GGGGACCACTTTGTACAAGAAAGCTGGGTTCAGACTTTGCTCCACCCGC
2_hypothetical	C10	Tb927.4.4660_for	GGGGACAAGTTTGTACAAAAAAGCAGGCTATGATCATTTGGGCAGTAGGGACA	Tb927.4.4660_rev	GGGGACCACTTTGTACAAGAAAGCTGGGTTTAATTGTCCCAATACGTCCGACTTG
2_hypothetical	C11	Tb927.1.3800_for	GGGGACAAGTTTGTACAAAAAAGCAGGCTATGCGATGCACGCATGTTCG	Tb927.1.3800_rev	GGGGACCACTTTGTACAAGAAAGCTGGGTCTAACGATCGTAACGATCAGGAATGC
2_hypothetical	C12	Tb927.10.9170_for	GGGGACAAGTTTGTACAAAAAAGCAGGCTATGACGGCGCCTCTTTTCAG	Tb927.10.9170_rev	GGGGACCACTTTGTACAAGAAAGCTGGGTTCATAGAAGCCTCTGCACCATTTCC
2_hypothetical	C13	Tb927.10.13140_for	GGGGACAAGTTTGTACAAAAAAGCAGGCTATGGGCAAACGCCGAGTTG	Tb927.10.13140_rev	GGGGACCACTTTGTACAAGAAAGCTGGGTCTATGTGGTCAAGTGTGCAAACATCT
2_hypothetical	C14	Tb927.3.770_for	GGGGACAAGTTTGTACAAAAAAGCAGGCTATGGTGTTGCGCAGGTGG	Tb927.3.770_rev	GGGGACCACTTTGTACAAGAAAGCTGGGTCTAATCCTCCTCCTCCTCCGTTG
2_hypothetical	C15	Tb927.10.9790_for	GGGGACAAGTTTGTACAAAAAAGCAGGCTATGAGACTTATTTCTTTAGCAACGAGAACT	Tb927.10.9790_rev	GGGGACCACTTTGTACAAGAAAGCTGGGTTTATCGCACGCCACTCTCGT
2_hypothetical	C16	Tb927.4.4780_for	GGGGACAAGTTTGTACAAAAAAGCAGGCTATGATATGTGTAGTGCACCACACG	Tb927.4.4780_rev	GGGGACCACTTTGTACAAGAAAGCTGGGTCTACCCGAGCCGCAACAAC
2_hypothetical	C17	Tb927.11.11910_for	GGGGACAAGTTTGTACAAAAAAGCAGGCTATGGATAGTGGGACCAAAAGGGAA	Tb927.11.11910_rev	GGGGACCACTTTGTACAAGAAAGCTGGGTTTACAAGATAAGTGGAATTACTGGTGGTG
2_hypothetical	C18	Tb927.9.10830_for	GGGGACAAGTTTGTACAAAAAAGCAGGCTATGGACACGATCGAATTCCTCCA	Tb927.9.10830_rev	GGGGACCACTTTGTACAAGAAAGCTGGGTTTAACTCTTCCGAGAGGGTCGTG
2_hypothetical	C19	Tb927.8.8150_for	GGGGACAAGTTTGTACAAAAAAGCAGGCTATGGGGAAGCTTCAGGTTTGTATTTG	Tb927.8.8150_rev	GGGGACCACTTTGTACAAGAAAGCTGGGTTTATGCTGAGGCTACAGCTTCAGG
2_hypothetical	C20	Tb927.11.4720_for	GGGGACAAGTTTGTACAAAAAAGCAGGCTATGCCCGACTCGCCGACA	Tb927.11.4720_rev	GGGGACCACTTTGTACAAGAAAGCTGGGTTTATCTAGAGTCGTCTCTTGGGTGC
2_hypothetical	C21	Tb927.3.4880_for	GGGGACAAGTTTGTACAAAAAAGCAGGCTATGAGCTCCATACCGGTGGAC	Tb927.3.4880_rev	GGGGACCACTTTGTACAAGAAAGCTGGGTTTATTCAAAGGCTAGAAGAGGGTTTTGG
2_hypothetical	C22	Tb927.10.10290_for	GGGGACAAGTTTGTACAAAAAAGCAGGCTATGCACACCGGGGACAAGC	Tb927.10.10290_rev	GGGGACCACTTTGTACAAGAAAGCTGGGTTTACATTTGGGTTCTCGCGCC
2_hypothetical	C23	Tb927.5.2110_for	GGGGACAAGTTTGTACAAAAAAGCAGGCTATGTCATCGATTGCCAAACGAATGC	Tb927.5.2110_rev	GGGGACCACTTTGTACAAGAAAGCTGGGTTCAGTGGCCGCGGTGTGA
2_hypothetical	C24	Tb927.10.10370_for	GGGGACAAGTTTGTACAAAAAAGCAGGCTATGCACACCGGGGACAAGC	Tb927.10.10370_rev	GGGGACCACTTTGTACAAGAAAGCTGGGTTTACATTTGGGTTCTCGTGCCAC
2_hypothetical	D1	Tb927.7.520_for	GGGGACAAGTTTGTACAAAAAAGCAGGCTATGTCTTTGCCTGACGGAGGA	Tb927.7.520_rev	GGGGACCACTTTGTACAAGAAAGCTGGGTTTAGCCGGGGCCTTTTTCTTTT
2_hypothetical	D2	Tb927.9.12080_for	GGGGACAAGTTTGTACAAAAAAGCAGGCTATGAGTTACCTACCCGACGCG	Tb927.9.12080_rev	GGGGACCACTTTGTACAAGAAAGCTGGGTCTACTGATTTTCAGTCACACCAGTTGA
2_hypothetical	D3	Tb927.11.1690_for	GGGGACAAGTTTGTACAAAAAAGCAGGCTATGACGACGATGGAGAAACCGC	Tb927.11.1690_rev	GGGGACCACTTTGTACAAGAAAGCTGGGTTTATTTATTTTTTCTCTTTTTTGGCGAGTT
2_hypothetical	D4	Tb927.11.11930.2_for	GGGGACAAGTTTGTACAAAAAAGCAGGCTATGCGCAGCAGGAACCTG	Tb927.11.11930.2_rev	GGGGACCACTTTGTACAAGAAAGCTGGGTTTACTTCTCGGATTGAAAACGAATAGCT
2_hypothetical	D5	Tb927.10.6470_for	GGGGACAAGTTTGTACAAAAAAGCAGGCTATGCACTTGAAGGAGTGTCCACC	Tb927.10.6470_rev	GGGGACCACTTTGTACAAGAAAGCTGGGTTTACAACGTTGTAAGGGCAACAAGA
2_hypothetical	D6	Tb927.4.3480_for	GGGGACAAGTTTGTACAAAAAAGCAGGCTATGATCTTTTTCGGCGTACTCTCTTT	Tb927.4.3480_rev	GGGGACCACTTTGTACAAGAAAGCTGGGTCTACAGTTCCTCTAAATACATACACATCTC
2_hypothetical	D7	Tb927.10.8620_for	GGGGACAAGTTTGTACAAAAAAGCAGGCTATGGTATTGCACAAGTGGGCTG	Tb927.10.8620_rev	GGGGACCACTTTGTACAAGAAAGCTGGGTCTACCCTCCCCCCGAGGT
2_hypothetical	D8	Tb927.7.6030_for	GGGGACAAGTTTGTACAAAAAAGCAGGCTATGGGCCCACCTCCAAACC	Tb927.7.6030_rev	GGGGACCACTTTGTACAAGAAAGCTGGGTTCACAGCACCCCCGCCCC
2_hypothetical	D9	Tb927.8.4550_for	GGGGACAAGTTTGTACAAAAAAGCAGGCTATGCGGCGTGTTAGGGATGG	Tb927.8.4550_rev	GGGGACCACTTTGTACAAGAAAGCTGGGTCTACAGGTACTTATCTAACGTCATCGC
2_hypothetical	D11	Tb927.8.1230_for	GGGGACAAGTTTGTACAAAAAAGCAGGCTATGTCGCTTCACTGCTTCTTTGG	Tb927.8.1230_rev	GGGGACCACTTTGTACAAGAAAGCTGGGTTTAATTTCCCTTCCCTTTCTTCTCCGC
2_hypothetical	D12	Tb927.4.3330_for	GGGGACAAGTTTGTACAAAAAAGCAGGCTATGCCTGCTTCAGCCTCTACG	Tb927.4.3330_rev	GGGGACCACTTTGTACAAGAAAGCTGGGTCTAACGATGATTTGAGAGAAAGTTCATGAA
2_hypothetical	D13	Tb927.11.16350_for	GGGGACAAGTTTGTACAAAAAAGCAGGCTATGGGTATACTTCAGCACCAGGC	Tb927.11.16350_rev	GGGGACCACTTTGTACAAGAAAGCTGGGTTTACGAGTACTTATCCACCATGGCG
2_hypothetical	D14	Tb927.4.5090_for	GGGGACAAGTTTGTACAAAAAAGCAGGCTATGTACTCTTTTTGGGCCTCCCC	Tb927.4.5090_rev	GGGGACCACTTTGTACAAGAAAGCTGGGTTTAGCCGTAGCCGCGCCG
2_hypothetical	D15	Tb927.9.7250_for	GGGGACAAGTTTGTACAAAAAAGCAGGCTATGAAAAACAAAGCGGCATCTCG	Tb927.9.7250_rev	GGGGACCACTTTGTACAAGAAAGCTGGGTTTACAAACCTTTGCCTATCTTTTGCAAC
2_hypothetical	D16	Tb927.6.3080_for	GGGGACAAGTTTGTACAAAAAAGCAGGCTATGCCCGTTACTTCTTTCAATAACCG	Tb927.6.3080_rev	GGGGACCACTTTGTACAAGAAAGCTGGGTTCACTCTCCACTTTCCCTTTTAGAACC
2_hypothetical	D17	Tb927.7.7020_for	GGGGACAAGTTTGTACAAAAAAGCAGGCTATGGAGGGAAGAAGAAGCGTAAGG	Tb927.7.7020_rev	GGGGACCACTTTGTACAAGAAAGCTGGGTTTATACAACCGCGGCCAAAAAAC
2_hypothetical	D18	Tb927.4.4640_for	GGGGACAAGTTTGTACAAAAAAGCAGGCTATGCGTGGAACAACAAAGCCG	Tb927.4.4640_rev	GGGGACCACTTTGTACAAGAAAGCTGGGTTTACGTAGGAAATGGAAATCTAGTTTTCCA
2_hypothetical	D19	Tb927.5.2830_for	GGGGACAAGTTTGTACAAAAAAGCAGGCTATGCGGTTCGTTTCTCGCAG	Tb927.5.2830_rev	GGGGACCACTTTGTACAAGAAAGCTGGGTCTACACAAGTGTATAACCCTTCACCTTG
2_hypothetical	D20	Tb927.4.1210_for	GGGGACAAGTTTGTACAAAAAAGCAGGCTATGCGAGAGCCTCCCTACG	Tb927.4.1210_rev	GGGGACCACTTTGTACAAGAAAGCTGGGTTTAAGAAGTGAGAGTGAATAAAGGAGACCT
2_hypothetical	D21	Tb927.8.5520_for	GGGGACAAGTTTGTACAAAAAAGCAGGCTATGCCGCATATTACTACGCCCT	Tb927.8.5520_rev	GGGGACCACTTTGTACAAGAAAGCTGGGTTCATTCGGTGGTAGACCGCA
2_hypothetical	D22	Tb927.11.8250_for	GGGGACAAGTTTGTACAAAAAAGCAGGCTATGAGCCACCCAGATGGTTCC	Tb927.11.8250_rev	GGGGACCACTTTGTACAAGAAAGCTGGGTTCACACCTCCACCTCGGC
2_hypothetical	D23	Tb927.4.4390_for	GGGGACAAGTTTGTACAAAAAAGCAGGCTATGGAGGACGTAAATAGGCAGAATCA	Tb927.4.4390_rev	GGGGACCACTTTGTACAAGAAAGCTGGGTTCAACGTAACCCAATGATTGTTTCATTC
2_hypothetical	D24	Tb927.11.300_for	GGGGACAAGTTTGTACAAAAAAGCAGGCTATGAAACGTCTACGCCGCTTC	Tb927.11.300_rev	GGGGACCACTTTGTACAAGAAAGCTGGGTTCAATTTGTGGTGCTAACGCCA
2_hypothetical	E1	Tb927.11.8040_for	GGGGACAAGTTTGTACAAAAAAGCAGGCTATGTTGAATCCGACATTTTCTCTGTACA	Tb927.11.8040_rev	GGGGACCACTTTGTACAAGAAAGCTGGGTCTAACCCCATAACTCCGCTGC
2_hypothetical	E2	Tb927.10.5090_for	GGGGACAAGTTTGTACAAAAAAGCAGGCTATGGGTCGCAGGCATGGA	Tb927.10.5090_rev	GGGGACCACTTTGTACAAGAAAGCTGGGTTTAGGCGGATGAGTCTTGTGATACG
2_hypothetical	E3	Tb927.11.13710_for	GGGGACAAGTTTGTACAAAAAAGCAGGCTATGGGTCACCTTCAGAGCACC	Tb927.11.13710_rev	GGGGACCACTTTGTACAAGAAAGCTGGGTTCATCTTTCTTTGTTGAGAAACTCTGCC
2_hypothetical	E4	Tb927.11.3640_for	GGGGACAAGTTTGTACAAAAAAGCAGGCTATGTTAAAGTGCTCCTTGTGCTATCTAG	Tb927.11.3640_rev	GGGGACCACTTTGTACAAGAAAGCTGGGTTCATGTGTAACTGTACCTATCCAAGGG
2_hypothetical	E5	Tb927.10.5630_for	GGGGACAAGTTTGTACAAAAAAGCAGGCTATGGATGATGGTGTAGAAGCCAAGC	Tb927.10.5630_rev	GGGGACCACTTTGTACAAGAAAGCTGGGTTTATTCATGTCGACGTGACAAGCG
2_hypothetical	E6	Tb927.7.3140_for	GGGGACAAGTTTGTACAAAAAAGCAGGCTATGAGCACTTTATCATCGTGGCTC	Tb927.7.3140_rev	GGGGACCACTTTGTACAAGAAAGCTGGGTTTACGCTTCGAGAAGCATCTGGT
2_hypothetical	E7	Tb927.11.9940_for	GGGGACAAGTTTGTACAAAAAAGCAGGCTATGACGGAGGGTGTTGGGC	Tb927.11.9940_rev	GGGGACCACTTTGTACAAGAAAGCTGGGTCTAGGCTATGGCACCCGCA
2_hypothetical	E8	Tb927.8.2360_for	GGGGACAAGTTTGTACAAAAAAGCAGGCTATGACTGACCCCTTCTATAGGTTACG	Tb927.8.2360_rev	GGGGACCACTTTGTACAAGAAAGCTGGGTTTAGAAGGGCAAAAAAACTTGCCG
2_hypothetical	E9	Tb927.8.2010_for	GGGGACAAGTTTGTACAAAAAAGCAGGCTATGCTGAGGGGAAGGTGGG	Tb927.8.2010_rev	GGGGACCACTTTGTACAAGAAAGCTGGGTTCACCGCTTCTGAAGAACAAGGT
2_hypothetical	E10	Tb927.8.2080_for	GGGGACAAGTTTGTACAAAAAAGCAGGCTATGATGGGTTGTGGAGGTTCTAAATTG	Tb927.8.2080_rev	GGGGACCACTTTGTACAAGAAAGCTGGGTTCACCGCACAAACTGAAACACA
2_hypothetical	E11	Tb927.9.700_for	GGGGACAAGTTTGTACAAAAAAGCAGGCTATGTATAATTTGTACATTTACTTTGTGCGA	Tb927.9.700_rev	GGGGACCACTTTGTACAAGAAAGCTGGGTTTATCCCTGTGTGACTAACCGAAAGT
2_hypothetical	E12	Tb927.11.9770_for	GGGGACAAGTTTGTACAAAAAAGCAGGCTATGGCGATCGCTAGGACCAC	Tb927.11.9770_rev	GGGGACCACTTTGTACAAGAAAGCTGGGTTCATCCCAAGCATGACGGGG
2_hypothetical	E13	Tb927.11.16220_for	GGGGACAAGTTTGTACAAAAAAGCAGGCTATGCGCCGTAATCCGTCCC	Tb927.11.16220_rev	GGGGACCACTTTGTACAAGAAAGCTGGGTTTATGCTCCATCATTGTCCGTGC
2_hypothetical	E14	Tb927.11.9970_for	GGGGACAAGTTTGTACAAAAAAGCAGGCTATGAGCTCCCTTAAGTCGGTAGC	Tb927.11.9970_rev	GGGGACCACTTTGTACAAGAAAGCTGGGTTCACGCGCCAAGACCCTT
2_hypothetical	E15	Tb927.11.570_for	GGGGACAAGTTTGTACAAAAAAGCAGGCTATGAATCGGTCGTGTTCCCGT	Tb927.11.570_rev	GGGGACCACTTTGTACAAGAAAGCTGGGTTCAACGTGTCGCAGCGCG
2_hypothetical	E16	Tb927.10.11260_for	GGGGACAAGTTTGTACAAAAAAGCAGGCTATGCTGAAAAGAACGTTAACTGTTTTAGAT	Tb927.10.11260_rev	GGGGACCACTTTGTACAAGAAAGCTGGGTTCACACTGTCTTTTTACTCTGTCGTC
2_hypothetical	E17	Tb927.11.14320_for	GGGGACAAGTTTGTACAAAAAAGCAGGCTATGACCGGTTTTGACACTGTCTG	Tb927.11.14320_rev	GGGGACCACTTTGTACAAGAAAGCTGGGTCTATTGCGCCTGCACCTCC
2_hypothetical	E18	Tb927.11.2480_for	GGGGACAAGTTTGTACAAAAAAGCAGGCTATGTGTGGGGCTCATTCGTGC	Tb927.11.2480_rev	GGGGACCACTTTGTACAAGAAAGCTGGGTTTATGAAAACGTTGCGAGCTCGT
2_hypothetical	E19	Tb927.11.3370_for	GGGGACAAGTTTGTACAAAAAAGCAGGCTATGCTCAACATCACCAGACTGC	Tb927.11.3370_rev	GGGGACCACTTTGTACAAGAAAGCTGGGTCTACCCAATGTTTTCAATACTTTGGCTATC
2_hypothetical	E20	Tb927.6.4240_for	GGGGACAAGTTTGTACAAAAAAGCAGGCTATGGTTTCACTGATGAAGTTGGCC	Tb927.6.4240_rev	GGGGACCACTTTGTACAAGAAAGCTGGGTTCACCTCACGTCCAGAAAATACATATG
2_hypothetical	E21	Tb927.4.2265_for	GGGGACAAGTTTGTACAAAAAAGCAGGCTATGACGCAAAACAAGGTGAGGAA	Tb927.4.2265_rev	GGGGACCACTTTGTACAAGAAAGCTGGGTTCATGAAGCGGCTGGTTTGAC
2_hypothetical	E22	Tb927.4.850_for	GGGGACAAGTTTGTACAAAAAAGCAGGCTATGAGAAATAATATAGAGGCGGCAGGT	Tb927.4.850_rev	GGGGACCACTTTGTACAAGAAAGCTGGGTTTACGGTTTTCCTTTAGACAATTCATATGC
2_hypothetical	E23	Tb927.10.8260_for	GGGGACAAGTTTGTACAAAAAAGCAGGCTATGCGTCAGTCATCTTTGCCTG	Tb927.10.8260_rev	GGGGACCACTTTGTACAAGAAAGCTGGGTTCACCTGTAAAAGGAGAGCACGA
2_hypothetical	E24	Tb927.6.2260_for	GGGGACAAGTTTGTACAAAAAAGCAGGCTATGCGGGCGTGGAGTAACC	Tb927.6.2260_rev	GGGGACCACTTTGTACAAGAAAGCTGGGTTTACCGATTCACTGCCACAGC
2_hypothetical	F1	Tb927.10.2660_for	GGGGACAAGTTTGTACAAAAAAGCAGGCTATGAAAAGGGATACAAACAACCACAATG	Tb927.10.2660_rev	GGGGACCACTTTGTACAAGAAAGCTGGGTTCACCACAGGGACGTGGG
2_hypothetical	F2	Tb927.5.1220_for	GGGGACAAGTTTGTACAAAAAAGCAGGCTATGATGCCCGTCGCGGGT	Tb927.5.1220_rev	GGGGACCACTTTGTACAAGAAAGCTGGGTTCAGATGCTCACCTTCTGTAACAGC
2_hypothetical	F3	Tb927.10.4660_for	GGGGACAAGTTTGTACAAAAAAGCAGGCTATGGCTGCATCAGAGGCAATG	Tb927.10.4660_rev	GGGGACCACTTTGTACAAGAAAGCTGGGTTCAACCCTTCAACGATTTTTCACTGC
2_hypothetical	F4	Tb927.7.4140_for	GGGGACAAGTTTGTACAAAAAAGCAGGCTATGCCCTCACTAGCGAACGG	Tb927.7.4140_rev	GGGGACCACTTTGTACAAGAAAGCTGGGTTCACACACCACCCAAAAGGTCG
2_hypothetical	F6	Tb927.3.820_for	GGGGACAAGTTTGTACAAAAAAGCAGGCTATGTCGGGGGCGTTCGGT	Tb927.3.820_rev	GGGGACCACTTTGTACAAGAAAGCTGGGTCTAGTAGTCGCTCGCTTGTACAGC
2_hypothetical	F7	Tb927.6.1300_for	GGGGACAAGTTTGTACAAAAAAGCAGGCTATGATGTGGAACGTGGCGC	Tb927.6.1300_rev	GGGGACCACTTTGTACAAGAAAGCTGGGTTCACAGAAACATGGAAAAATTCATTTGAGA
2_hypothetical	F8	Tb927.10.5060_for	GGGGACAAGTTTGTACAAAAAAGCAGGCTATGCCGTGGTATTTAAGCACTTTCA	Tb927.10.5060_rev	GGGGACCACTTTGTACAAGAAAGCTGGGTTCAAATGGCCCCTGTTCCTTTTC
2_hypothetical	F9	Tb927.11.7790_for	GGGGACAAGTTTGTACAAAAAAGCAGGCTATGCAAGTGACAGAAACGGTGC	Tb927.11.7790_rev	GGGGACCACTTTGTACAAGAAAGCTGGGTCTAAAGATACGGCGCCACCG
2_hypothetical	F10	Tb927.11.17016_for	GGGGACAAGTTTGTACAAAAAAGCAGGCTATGCCGATAATACGTAAGGTTCAATGT	Tb927.11.17016_rev	GGGGACCACTTTGTACAAGAAAGCTGGGTTTATCCTTTTTTGTTCGCACTGCAC
2_hypothetical	F11	Tb927.11.11300_for	GGGGACAAGTTTGTACAAAAAAGCAGGCTATGGTGTGGAAAGGATCACGTGA	Tb927.11.11300_rev	GGGGACCACTTTGTACAAGAAAGCTGGGTCTAAGCGACACGTTGGCGT
2_hypothetical	F12	Tb927.11.10080_for	GGGGACAAGTTTGTACAAAAAAGCAGGCTATGTTGCGTCTCTGCCGTG	Tb927.11.10080_rev	GGGGACCACTTTGTACAAGAAAGCTGGGTCTATGAGGGCCTTCCGTATATATTCGA
2_hypothetical	F13	Tb927.11.10410_for	GGGGACAAGTTTGTACAAAAAAGCAGGCTATGAGCATGCCATGCAATATTCGT	Tb927.11.10410_rev	GGGGACCACTTTGTACAAGAAAGCTGGGTTTAGTTTGGTAACAGAGCGAGCG
2_hypothetical	F14	Tb927.6.2070_for	GGGGACAAGTTTGTACAAAAAAGCAGGCTATGTGGCGGTTGTCGCGG	Tb927.6.2070_rev	GGGGACCACTTTGTACAAGAAAGCTGGGTTTACCATGGTCCGTCCGC

2_hypothetical	F15	Tb927.11.13870_for	GGGGACAAGTTTGTACAAAAAAGCAGGCTATGGTCAACGCGACTCCCG	Tb927.11.13870_rev	GGGGACCACTTTGTACAAGAAAGCTGGGTTCAGCAAGGTAGTGAACCAAGGT
2_hypothetical	F16	Tb927.10.14760_for	GGGGACAAGTTTGTACAAAAAAGCAGGCTATGGACTTCCTAAACGAGAGTCAAAC	Tb927.10.14760_rev	GGGGACCACTTTGTACAAGAAAGCTGGGTCTATCCACTCCCTGACTGTGACA
2_hypothetical	F17	Tb927.11.6080_for	GGGGACAAGTTTGTACAAAAAAGCAGGCTATGGATAAAGATGTGGATGTGACAAAGT	Tb927.11.6080_rev	GGGGACCACTTTGTACAAGAAAGCTGGGTTCATTGTACACCTCCAGCAGACC
2_hypothetical	F18	Tb927.8.7040_for	GGGGACAAGTTTGTACAAAAAAGCAGGCTATGCGTAAGTCATTTCTTGGAGCA	Tb927.8.7040_rev	GGGGACCACTTTGTACAAGAAAGCTGGGTTCATTTCGCTGCTGCTCGC
2_hypothetical	F19	Tb927.11.4330_for	GGGGACAAGTTTGTACAAAAAAGCAGGCTATGGAGCAACCCTCCCAGATG	Tb927.11.4330_rev	GGGGACCACTTTGTACAAGAAAGCTGGGTCTAAACCATGCCCTCCGCAG
2_hypothetical	F20	Tb927.10.2360_for	GGGGACAAGTTTGTACAAAAAAGCAGGCTATGTATTTTGGTTTCGCTCATTTGCC	Tb927.10.2360_rev	GGGGACCACTTTGTACAAGAAAGCTGGGTTTACTTTATGTGGTGGAGGCGC
2_hypothetical	F21	Tb927.10.7910_for	GGGGACAAGTTTGTACAAAAAAGCAGGCTATGTTTTCCAGCGTATTACTGCGC	Tb927.10.7910_rev	GGGGACCACTTTGTACAAGAAAGCTGGGTTTACTTCCATACAAAAGTTCCCTGCG
2_hypothetical	F22	Tb927.8.2190_for	GGGGACAAGTTTGTACAAAAAAGCAGGCTATGAGGCAATGCAGCAGAACAA	Tb927.8.2190_rev	GGGGACCACTTTGTACAAGAAAGCTGGGTTCATCCTCTTGTACTCCCAACTGTT
2_hypothetical	F23	Tb927.6.1700_for	GGGGACAAGTTTGTACAAAAAAGCAGGCTATGTTGCGGTCACGTATCCCA	Tb927.6.1700_rev	GGGGACCACTTTGTACAAGAAAGCTGGGTCTAACTGTTTGGGTCGAACAAAAACTC
2_hypothetical	F24	Tb927.8.1880_for	GGGGACAAGTTTGTACAAAAAAGCAGGCTATGCTGCAAATAAACGCGTTCAA	Tb927.8.1880_rev	GGGGACCACTTTGTACAAGAAAGCTGGGTTCACTTCTTTCCACCCCCATCC
2_hypothetical	G1	Tb927.2.2200_for	GGGGACAAGTTTGTACAAAAAAGCAGGCTATGCCTGCCCCCAGTTTAGC	Tb927.2.2200_rev	GGGGACCACTTTGTACAAGAAAGCTGGGTTCAGGCTGCTGTGGCTTTTC
2_hypothetical	G2	Tb927.1.1090_for	GGGGACAAGTTTGTACAAAAAAGCAGGCTATGGTTTGGTTCTCCGCGC	Tb927.1.1090_rev	GGGGACCACTTTGTACAAGAAAGCTGGGTTTATATAATTGCATCCCCCTCGTGC
2_hypothetical	G3	Tb927.7.2980_for	GGGGACAAGTTTGTACAAAAAAGCAGGCTATGCGCCGGTCTGTATATCAGC	Tb927.7.2980_rev	GGGGACCACTTTGTACAAGAAAGCTGGGTTTATTTAAACACCTTAAAGCGTTCTTCATC
2_hypothetical	G5	Tb927.7.4630_for	GGGGACAAGTTTGTACAAAAAAGCAGGCTATGTCAGGGCGACTTATCATTTATGTG	Tb927.7.4630_rev	GGGGACCACTTTGTACAAGAAAGCTGGGTCTACGGCGGGAACGCAAA
2_hypothetical	G6	Tb927.11.16440_for	GGGGACAAGTTTGTACAAAAAAGCAGGCTATGGATGCGTTTTTGGAGTCGC	Tb927.11.16440_rev	GGGGACCACTTTGTACAAGAAAGCTGGGTTCATGTTTTTTTCTTGCTTGAAGCTGC
2_hypothetical	G7	Tb927.5.1940_for	GGGGACAAGTTTGTACAAAAAAGCAGGCTATGCCAAAAAAGAAGCCACCATGT	Tb927.5.1940_rev	GGGGACCACTTTGTACAAGAAAGCTGGGTTCACGGTTTCCCACCGCA
2_hypothetical	G8	Tb927.8.4290_for	GGGGACAAGTTTGTACAAAAAAGCAGGCTATGGGGCGAATTAGGGTGACA	Tb927.8.4290_rev	GGGGACCACTTTGTACAAGAAAGCTGGGTTCATTCCTTTCTGTGCCGGTTC
2_hypothetical	G9	Tb927.10.1880_for	GGGGACAAGTTTGTACAAAAAAGCAGGCTATGACCACGCCTGGTACTTCA	Tb927.10.1880_rev	GGGGACCACTTTGTACAAGAAAGCTGGGTTCAGTTGCGCCCCTCCGT
2_hypothetical	G10	Tb927.10.16010_for	GGGGACAAGTTTGTACAAAAAAGCAGGCTATGTGCAGCCGGCGCCCT	Tb927.10.16010_rev	GGGGACCACTTTGTACAAGAAAGCTGGGTCTATTCCGTCAGGCTGACGC
2_hypothetical	G11	Tb927.9.7710_for	GGGGACAAGTTTGTACAAAAAAGCAGGCTATGAGTGCCTTTGAGTCATTCCTCC	Tb927.9.7710_rev	GGGGACCACTTTGTACAAGAAAGCTGGGTCTACACACGAGGGGACACCT
2_hypothetical	G12	Tb927.5.4120_for	GGGGACAAGTTTGTACAAAAAAGCAGGCTATGAGGCGATGTATCCCCGC	Tb927.5.4120_rev	GGGGACCACTTTGTACAAGAAAGCTGGGTTCAAGCTGCTCCGTTCCCC
2_hypothetical	G13	Tb927.10.15620_for	GGGGACAAGTTTGTACAAAAAAGCAGGCTATGGTAAAACACCAAAATGGCGC	Tb927.10.15620_rev	GGGGACCACTTTGTACAAGAAAGCTGGGTTTATATATATATATATATATATATATATAT
2_hypothetical	G14	Tb927.9.10300_for	GGGGACAAGTTTGTACAAAAAAGCAGGCTATGAGGGAACCCCCCACTACG	Tb927.9.10300_rev	GGGGACCACTTTGTACAAGAAAGCTGGGTTTATGATGACGAAGGCACTTGCAT
2_hypothetical	G15	Tb927.10.7870_for	GGGGACAAGTTTGTACAAAAAAGCAGGCTATGCCTGGCATTGCGCAA	Tb927.10.7870_rev	GGGGACCACTTTGTACAAGAAAGCTGGGTCTACGCAAATCTATCGGCAACCT
2_hypothetical	G16	Tb927.11.10620_for	GGGGACAAGTTTGTACAAAAAAGCAGGCTATGGAGGACGAACCGACTCAA	Tb927.11.10620_rev	GGGGACCACTTTGTACAAGAAAGCTGGGTTTAGTCATCGGTAACACTCTTGCCA
2_hypothetical	G17	Tb927.5.750_for	GGGGACAAGTTTGTACAAAAAAGCAGGCTATGGCTTTTCCTCATGTGAGTTATCG	Tb927.5.750_rev	GGGGACCACTTTGTACAAGAAAGCTGGGTCTACTCTAACATGTACTGACACAGGCA
2_hypothetical	G18	Tb927.7.7460_for	GGGGACAAGTTTGTACAAAAAAGCAGGCTATGGTTTTTTCCACACACGGCA	Tb927.7.7460_rev	GGGGACCACTTTGTACAAGAAAGCTGGGTTTACCTCCTCACCGTTGGTTTGA
2_hypothetical	G19	Tb927.10.8380_for	GGGGACAAGTTTGTACAAAAAAGCAGGCTATGGATACGCCCCCGATAAGTG	Tb927.10.8380_rev	GGGGACCACTTTGTACAAGAAAGCTGGGTCTAGCAGCGACTTTGCGCA
2_hypothetical	G20	Tb927.9.14230_for	GGGGACAAGTTTGTACAAAAAAGCAGGCTATGTGGAAGCAGCGTCGGA	Tb927.9.14230_rev	GGGGACCACTTTGTACAAGAAAGCTGGGTCTAACCCCTGAGGCCGCC
2_hypothetical	G21	Tb927.11.1620_for	GGGGACAAGTTTGTACAAAAAAGCAGGCTATGTGGGCGCAACAGCTTAG	Tb927.11.1620_rev	GGGGACCACTTTGTACAAGAAAGCTGGGTTTATTCCTTCCCGACGGCTTGC
2_hypothetical	G22	Tb927.11.7440_for	GGGGACAAGTTTGTACAAAAAAGCAGGCTATGAAAAGGGCAAAGGCACTTGT	Tb927.11.7440_rev	GGGGACCACTTTGTACAAGAAAGCTGGGTTTACATATCTAGGAGGACGCTCAACG
2_hypothetical	G23	Tb927.10.2620_for	GGGGACAAGTTTGTACAAAAAAGCAGGCTATGACGAATCAGTCGGACGCG	Tb927.10.2620_rev	GGGGACCACTTTGTACAAGAAAGCTGGGTTTACGGTTCGAGAGGAGGCAG
2_hypothetical	G24	Tb927.5.2010_for	GGGGACAAGTTTGTACAAAAAAGCAGGCTATGTATGTTGTTTCACTTAAAATATATATT	Tb927.5.2010_rev	GGGGACCACTTTGTACAAGAAAGCTGGGTCTAAAGGAAACGACACACACAAAACC
2_hypothetical	H1	Tb927.11.3180_for	GGGGACAAGTTTGTACAAAAAAGCAGGCTATGGGTGACTTTACCCGAGAAGA	Tb927.11.3180_rev	GGGGACCACTTTGTACAAGAAAGCTGGGTTTACATCTCAAGAACCTTCATTAGCAACA
2_hypothetical	H2	Tb927.10.2300_for	GGGGACAAGTTTGTACAAAAAAGCAGGCTATGTTTTCGAGACACTCCTTGTGG	Tb927.10.2300_rev	GGGGACCACTTTGTACAAGAAAGCTGGGTTCACGATTTTCCAGTCAAAATGCAC
2_hypothetical	H3	Tb927.7.5260_for	GGGGACAAGTTTGTACAAAAAAGCAGGCTATGGGTACCACCGCCAGTAC	Tb927.7.5260_rev	GGGGACCACTTTGTACAAGAAAGCTGGGTCTAAGCGTCCTTTACCCATGGGT
2_hypothetical	H4	Tb927.7.330_for	GGGGACAAGTTTGTACAAAAAAGCAGGCTATGGACTACAACGAAATAAGCCCAAG	Tb927.7.330_rev	GGGGACCACTTTGTACAAGAAAGCTGGGTCTACAGTTTTGAGATGGCCGCA
2_hypothetical	H5	Tb927.11.12300_for	GGGGACAAGTTTGTACAAAAAAGCAGGCTATGACATTTGTGTTCGAGGCTAGAAG	Tb927.11.12300_rev	GGGGACCACTTTGTACAAGAAAGCTGGGTTTATTGACCTGAAGGATGAAGGTGGT
2_hypothetical	H6	Tb927.7.4470_for	GGGGACAAGTTTGTACAAAAAAGCAGGCTATGACACGAGGACAAAACGATACAC	Tb927.7.4470_rev	GGGGACCACTTTGTACAAGAAAGCTGGGTTTATTTGTGCCTCGCCATGCG
2_hypothetical	H7	Tb927.8.4300_for	GGGGACAAGTTTGTACAAAAAAGCAGGCTATGAATATACATATATATATATATATATTA	Tb927.8.4300_rev	GGGGACCACTTTGTACAAGAAAGCTGGGTTTAAAAAAAAATGAATAATTTCATGCAGCA
2_hypothetical	H8	Tb927.9.920_for	GGGGACAAGTTTGTACAAAAAAGCAGGCTATGGCCAGAACCGACAAATGTC	Tb927.9.920_rev	GGGGACCACTTTGTACAAGAAAGCTGGGTTCAGCACGCATTTTGTTCCTTATCT
2_hypothetical	H9	Tb927.3.5010_for	GGGGACAAGTTTGTACAAAAAAGCAGGCTATGGCGGATAATGAAGAAGTGCC	Tb927.3.5010_rev	GGGGACCACTTTGTACAAGAAAGCTGGGTTTATGACCCCCTCCTCGACAGA
2_hypothetical	H10	Tb927.3.4130_for	GGGGACAAGTTTGTACAAAAAAGCAGGCTATGCTCCACACAACCCGCC	Tb927.3.4130_rev	GGGGACCACTTTGTACAAGAAAGCTGGGTTTAATTCACCCTCCACCGATAGTTGA
2_hypothetical	H11	Tb927.3.1260_for	GGGGACAAGTTTGTACAAAAAAGCAGGCTATGGAGGAGAAAGTGGTGACTTCG	Tb927.3.1260_rev	GGGGACCACTTTGTACAAGAAAGCTGGGTCTATGAAGAATTGTTCCTTACATCACAGTT
2_hypothetical	H12	Tb927.11.2840_for	GGGGACAAGTTTGTACAAAAAAGCAGGCTATGAGTACTGAGCCAACCCCTC	Tb927.11.2840_rev	GGGGACCACTTTGTACAAGAAAGCTGGGTTTACAAAGCGCGTTTTTTTTCCTTATCT
2_hypothetical	H13	Tb927.11.2900_for	GGGGACAAGTTTGTACAAAAAAGCAGGCTATGACAGGGATGCTCCGTTGC	Tb927.11.2900_rev	GGGGACCACTTTGTACAAGAAAGCTGGGTTCAGGAGTTAAAGTTTACATCAACCCTAC
2_hypothetical	H14	Tb927.10.15220_for	GGGGACAAGTTTGTACAAAAAAGCAGGCTATGTATCGGCTCATGGTTCCGC	Tb927.10.15220_rev	GGGGACCACTTTGTACAAGAAAGCTGGGTTTAGTAATGTTGCAAATCAAGCATGTGTT
2_hypothetical	H15	Tb927.7.5270_for	GGGGACAAGTTTGTACAAAAAAGCAGGCTATGATTCAGATTATTGCGCCGAGG	Tb927.7.5270_rev	GGGGACCACTTTGTACAAGAAAGCTGGGTTCACACGAACCTACGGAGGG
2_hypothetical	H16	Tb927.9.2400_for	GGGGACAAGTTTGTACAAAAAAGCAGGCTATGGGGTCCACAGATGGTGAA	Tb927.9.2400_rev	GGGGACCACTTTGTACAAGAAAGCTGGGTTCATCTTAGCAAATAGAGCGATGAAGTT
2_hypothetical	H17	Tb927.8.3260_for	GGGGACAAGTTTGTACAAAAAAGCAGGCTATGGGTTGGGGAAGCAGTGA	Tb927.8.3260_rev	GGGGACCACTTTGTACAAGAAAGCTGGGTTCAACCTTGCAGTTGAACCGC
2_hypothetical	H18	Tb927.1.4920_for	GGGGACAAGTTTGTACAAAAAAGCAGGCTATGGCTCCCCGTGGCGGG	Tb927.1.4920_rev	GGGGACCACTTTGTACAAGAAAGCTGGGTCTAAGTCGCTGCAAAATATATCCCCA
2_hypothetical	H19	Tb927.10.5740_for	GGGGACAAGTTTGTACAAAAAAGCAGGCTATGACAAGGCGGAACGCAAT	Tb927.10.5740_rev	GGGGACCACTTTGTACAAGAAAGCTGGGTTCATTTGCTTCGCGTCAACCC
2_hypothetical	H20	Tb927.3.4010_for	GGGGACAAGTTTGTACAAAAAAGCAGGCTATGCGGGTGTGTCGTCCC	Tb927.3.4010_rev	GGGGACCACTTTGTACAAGAAAGCTGGGTTCAGGAGTGCCTTAAATAAAGTAAGGC
2_hypothetical	H21	Tb927.6.830_for	GGGGACAAGTTTGTACAAAAAAGCAGGCTATGACCGCAACGACAACGC	Tb927.6.830_rev	GGGGACCACTTTGTACAAGAAAGCTGGGTTCACACGTTCGGATCAATCGC
2_hypothetical	H22	Tb927.5.990_for	GGGGACAAGTTTGTACAAAAAAGCAGGCTATGGATGAAAGCAACAGGGTGC	Tb927.5.990_rev	GGGGACCACTTTGTACAAGAAAGCTGGGTTTATCTTTTTCGCACAGCACGCG
2_hypothetical	H23	Tb927.10.7480_for	GGGGACAAGTTTGTACAAAAAAGCAGGCTATGCATCGCAAACCTCTGTTTGA	Tb927.10.7480_rev	GGGGACCACTTTGTACAAGAAAGCTGGGTTCAGAACAACAGTTCGGATAAAGAAGAG
2_hypothetical	H24	Tb927.11.13010_for	GGGGACAAGTTTGTACAAAAAAGCAGGCTATGGATAAAGGTATGAAGGATAAGGCTCC	Tb927.11.13010_rev	GGGGACCACTTTGTACAAGAAAGCTGGGTCTACATGAAGTTCAGGCGATGAACC
2_hypothetical	I1	Tb927.9.4990_for	GGGGACAAGTTTGTACAAAAAAGCAGGCTATGGCGGACGTTGCTGAC	Tb927.9.4990_rev	GGGGACCACTTTGTACAAGAAAGCTGGGTCTACTCCACAAGCTCTACCACCC
2_hypothetical	I2	Tb927.2.4480_for	GGGGACAAGTTTGTACAAAAAAGCAGGCTATGATATTTGGGTCTGAACCACCAC	Tb927.2.4480_rev	GGGGACCACTTTGTACAAGAAAGCTGGGTTCATTGGTGTGAATGGGAATCCTCC
2_hypothetical	I3	Tb927.10.11060_for	GGGGACAAGTTTGTACAAAAAAGCAGGCTATGCGTGAGAAGGTGGTGCC	Tb927.10.11060_rev	GGGGACCACTTTGTACAAGAAAGCTGGGTTCAACCAGTGTTACCCGAGTACG
2_hypothetical	I4	Tb927.3.2970_for	GGGGACAAGTTTGTACAAAAAAGCAGGCTATGAGCGACTCGCACGCC	Tb927.3.2970_rev	GGGGACCACTTTGTACAAGAAAGCTGGGTTCAGTCTCGGCGCCTAGAAA
2_hypothetical	I5	Tb927.10.7760_for	GGGGACAAGTTTGTACAAAAAAGCAGGCTATGGGCCAAGGCCTCACG	Tb927.10.7760_rev	GGGGACCACTTTGTACAAGAAAGCTGGGTTTAGAAGTCCATCAGCGCGTAAC
2_hypothetical	I6	Tb927.4.1520_for	GGGGACAAGTTTGTACAAAAAAGCAGGCTATGAACCCCGTCGGAGTGG	Tb927.4.1520_rev	GGGGACCACTTTGTACAAGAAAGCTGGGTTTACTTGTGAAGTCCTTCGGGTTCC
2_hypothetical	I7	Tb927.4.940_for	GGGGACAAGTTTGTACAAAAAAGCAGGCTATGTCCTTTGCTTGGACGCG	Tb927.4.940_rev	GGGGACCACTTTGTACAAGAAAGCTGGGTCTATGTCATATCTGTCCCAATCTTTCGTT
2_hypothetical	I8	Tb927.8.5840_for	GGGGACAAGTTTGTACAAAAAAGCAGGCTATGGGGGAACAGGATGGGC	Tb927.8.5840_rev	GGGGACCACTTTGTACAAGAAAGCTGGGTTTATGTATCCGCGCCGTCGC
2_hypothetical	I9	Tb927.6.2200_for	GGGGACAAGTTTGTACAAAAAAGCAGGCTATGCCCAAGGTTCTCGTTCCC	Tb927.6.2200_rev	GGGGACCACTTTGTACAAGAAAGCTGGGTCTAAAACTTACTGAACATTTGATTAATCAC
2_hypothetical	I10	Tb927.11.9830_for	GGGGACAAGTTTGTACAAAAAAGCAGGCTATGCTACGCTGTAGTTGTGCG	Tb927.11.9830_rev	GGGGACCACTTTGTACAAGAAAGCTGGGTTCAGCGCCAAAAGAAAAGCCT
2_hypothetical	I11	Tb927.2.5710_for	GGGGACAAGTTTGTACAAAAAAGCAGGCTATGCCTTGTTCGTTCCAAGAGG	Tb927.2.5710_rev	GGGGACCACTTTGTACAAGAAAGCTGGGTTTAAGGCGACAGGCGATTTACC
2_hypothetical	I12	Tb927.11.5200_for	GGGGACAAGTTTGTACAAAAAAGCAGGCTATGGACAAGAGTAAAACGATGGAGATC	Tb927.11.5200_rev	GGGGACCACTTTGTACAAGAAAGCTGGGTTCACTCGCCCCGGCCTCC
2_hypothetical	I13	Tb927.7.4190_for	GGGGACAAGTTTGTACAAAAAAGCAGGCTATGAGTCAGTTTGGTCAACTTGTGT	Tb927.7.4190_rev	GGGGACCACTTTGTACAAGAAAGCTGGGTCTATAATATGCGTGTTCCTACTACCATAAT
2_hypothetical	I14	Tb927.5.1040_for	GGGGACAAGTTTGTACAAAAAAGCAGGCTATGTCTATGGCCTACGTAATGGGAA	Tb927.5.1040_rev	GGGGACCACTTTGTACAAGAAAGCTGGGTTCACCACAGGGGACCACTG
2_hypothetical	I15	Tb927.9.3640_for	GGGGACAAGTTTGTACAAAAAAGCAGGCTATGCGACGCCTGGGCGTG	Tb927.9.3640_rev	GGGGACCACTTTGTACAAGAAAGCTGGGTTTACTCTTCAGCCCCCTCTTTCC
2_hypothetical	I16	Tb927.10.8340_for	GGGGACAAGTTTGTACAAAAAAGCAGGCTATGGGATGCTCGGCAAGCT	Tb927.10.8340_rev	GGGGACCACTTTGTACAAGAAAGCTGGGTTCAGTTGTAACGTTGCCCGC
2_hypothetical	I17	Tb927.11.11120_for	GGGGACAAGTTTGTACAAAAAAGCAGGCTATGCGTTGTGTGAGACGATCTG	Tb927.11.11120_rev	GGGGACCACTTTGTACAAGAAAGCTGGGTCTACAAATTATCATCGTGGTTCGCTGC
2_hypothetical	I18	Tb927.5.2015_for	GGGGACAAGTTTGTACAAAAAAGCAGGCTATGGCGGGTTTCAATATCGACG	Tb927.5.2015_rev	GGGGACCACTTTGTACAAGAAAGCTGGGTTCAGTGACGAGGCACCTCG
2_hypothetical	I21	Tb927.9.2390_for	GGGGACAAGTTTGTACAAAAAAGCAGGCTATGACAACACAGAACGGTGTTAACT	Tb927.9.2390_rev	GGGGACCACTTTGTACAAGAAAGCTGGGTTTATTTCCGTGGATAAAGGGCAACG
2_hypothetical	I22	Tb927.7.1630_for	GGGGACAAGTTTGTACAAAAAAGCAGGCTATGGGTCAGAAGCCGTCCAT	Tb927.7.1630_rev	GGGGACCACTTTGTACAAGAAAGCTGGGTCTATTGGTGGAAACCTACCAAGCAC
2_hypothetical	I23	Tb927.8.2260_for	GGGGACAAGTTTGTACAAAAAAGCAGGCTATGGGTTGTGGAGGTTCAGCT	Tb927.8.2260_rev	GGGGACCACTTTGTACAAGAAAGCTGGGTTCACCAGTGCATGAAACGAAACT
2_hypothetical	I24	Tb927.10.2070_for	GGGGACAAGTTTGTACAAAAAAGCAGGCTATGTCCAGAAAATCTTATCTAACCGCAG	Tb927.10.2070_rev	GGGGACCACTTTGTACAAGAAAGCTGGGTTTACCTTCGGTAGTTTCTTCGTTTTCC
2_hypothetical	J2	Tb927.10.7920_for	GGGGACAAGTTTGTACAAAAAAGCAGGCTATGAGCGACCCCCTCGTTG	Tb927.10.7920_rev	GGGGACCACTTTGTACAAGAAAGCTGGGTTTATCGCGTTTTTCCAAGTGTGACA
2_hypothetical	J5	Tb927.11.590_for	GGGGACAAGTTTGTACAAAAAAGCAGGCTATGCGGCGCACGTTAGGA	Tb927.11.590_rev	GGGGACCACTTTGTACAAGAAAGCTGGGTTTAGAAAGAAGGAAGCTTGGCACC
2_hypothetical	J6	Tb927.11.12160_for	GGGGACAAGTTTGTACAAAAAAGCAGGCTATGCGGCGTAGTCCTTGTGC	Tb927.11.12160_rev	GGGGACCACTTTGTACAAGAAAGCTGGGTCTAATTCCTTTGCCGAACTATATCCCC
2_hypothetical	J7	Tb927.8.4340_for	GGGGACAAGTTTGTACAAAAAAGCAGGCTATGGCTTCCGTCGGTAATTGC	Tb927.8.4340_rev	GGGGACCACTTTGTACAAGAAAGCTGGGTTCATTTAACGCAAGGCACTCCAC
2_hypothetical	J8	Tb927.10.9510_for	GGGGACAAGTTTGTACAAAAAAGCAGGCTATGAGAACGAATAGTCTCAACATCGG	Tb927.10.9510_rev	GGGGACCACTTTGTACAAGAAAGCTGGGTCTAATTGGAGGTTTGTGCCACAGA
2_hypothetical	J9	Tb927.10.13910_for	GGGGACAAGTTTGTACAAAAAAGCAGGCTATGCGTAGGACATTTCTCCGC	Tb927.10.13910_rev	GGGGACCACTTTGTACAAGAAAGCTGGGTTTACCACTGCTTCTTAATTTCGGGAG
2_hypothetical	J10	Tb927.9.7830_for	GGGGACAAGTTTGTACAAAAAAGCAGGCTATGACTATGAACTTTGGTAACATGACCC	Tb927.9.7830_rev	GGGGACCACTTTGTACAAGAAAGCTGGGTTTACTTCATCTCCGCATATTGAGGAGC
2_hypothetical	J11	Tb927.11.16170_for	GGGGACAAGTTTGTACAAAAAAGCAGGCTATGCGGCGCCTGTATCGC	Tb927.11.16170_rev	GGGGACCACTTTGTACAAGAAAGCTGGGTCTACAAATGGGTATTGTAACTAGGATCTGT
2_hypothetical	J12	Tb927.8.2440_for	GGGGACAAGTTTGTACAAAAAAGCAGGCTATGAGCCTTTTAAAAGCAGATCAATTGC	Tb927.8.2440_rev	GGGGACCACTTTGTACAAGAAAGCTGGGTCTACTCGCATGTATGAAGTTTTCCATCC
2_hypothetical	J13	Tb927.3.2300_for	GGGGACAAGTTTGTACAAAAAAGCAGGCTATGCTCCACACAGTGAGCGA	Tb927.3.2300_rev	GGGGACCACTTTGTACAAGAAAGCTGGGTTCAGTTATTATTACCGTCGCGCTC
2_hypothetical	J14	Tb927.10.4830_for	GGGGACAAGTTTGTACAAAAAAGCAGGCTATGTATAGTGATGGGACACGAAAGACC	Tb927.10.4830_rev	GGGGACCACTTTGTACAAGAAAGCTGGGTTTAAAAAATAATAACAGATTTGCGTTGTAG
2_hypothetical	J15	Tb927.10.4280_for	GGGGACAAGTTTGTACAAAAAAGCAGGCTATGCGGTCAACAGCTCCTCT	Tb927.10.4280_rev	GGGGACCACTTTGTACAAGAAAGCTGGGTTCAGTACTCTACCTCCTGCCCC
2_hypothetical	J16	Tb927.7.6430_for	GGGGACAAGTTTGTACAAAAAAGCAGGCTATGTCTGGGGGCCTTCCG	Tb927.7.6430_rev	GGGGACCACTTTGTACAAGAAAGCTGGGTCTATTGGTTCTCACAGCCGAGATC
2_hypothetical	J17	Tb927.9.12030_for	GGGGACAAGTTTGTACAAAAAAGCAGGCTATGGATTTAGATATCGTTGACGATGCG	Tb927.9.12030_rev	GGGGACCACTTTGTACAAGAAAGCTGGGTCTACCAGTCCAGTGAATGCGGT
2_hypothetical	J18	Tb927.8.4590_for	GGGGACAAGTTTGTACAAAAAAGCAGGCTATGGAACGTGTGAGGTACAAAGTC	Tb927.8.4590_rev	GGGGACCACTTTGTACAAGAAAGCTGGGTTCATGACCCCTTGTTCAAAGCTAC
2_hypothetical	J19	Tb927.7.4520_for	GGGGACAAGTTTGTACAAAAAAGCAGGCTATGACCGATACAATTGACGTGACG	Tb927.7.4520_rev	GGGGACCACTTTGTACAAGAAAGCTGGGTTTAAAATAGCGACATTTCAGCGTCCA
2_hypothetical	J20	Tb927.6.3270_for	GGGGACAAGTTTGTACAAAAAAGCAGGCTATGCATTTCACGTCGTCACGT	Tb927.6.3270_rev	GGGGACCACTTTGTACAAGAAAGCTGGGTCTAAGGCATTACTACTTGGTCCGC
2_hypothetical	J21	Tb927.10.9990_for	GGGGACAAGTTTGTACAAAAAAGCAGGCTATGACTGACGCCAGTAAAACAACC	Tb927.10.9990_rev	GGGGACCACTTTGTACAAGAAAGCTGGGTCTATTTGCTATTTTCAAAATGCTTCCTTAC
2_hypothetical	J22	Tb927.8.3270_for	GGGGACAAGTTTGTACAAAAAAGCAGGCTATGGAGTCCCTTGTACGTGACA	Tb927.8.3270_rev	GGGGACCACTTTGTACAAGAAAGCTGGGTTTACAATGAACATATTCGCTTCTGCTGT
2_hypothetical	J23	Tb927.3.4450_for	GGGGACAAGTTTGTACAAAAAAGCAGGCTATGCGGTGCTCTCCCCAA	Tb927.3.4450_rev	GGGGACCACTTTGTACAAGAAAGCTGGGTTTACGAATTGCGACTTATTTCTTCCGT
2_hypothetical	J24	Tb927.11.8070_for	GGGGACAAGTTTGTACAAAAAAGCAGGCTATGGATTTCCAGGGCTTACTACAACA	Tb927.11.8070_rev	GGGGACCACTTTGTACAAGAAAGCTGGGTTTATAAATCATCGGCAAGTTGTAGGCT
2_hypothetical	K1	Tb927.8.3040_for	GGGGACAAGTTTGTACAAAAAAGCAGGCTATGTTTCGCATATCCTCATGTTTTCACA	Tb927.8.3040_rev	GGGGACCACTTTGTACAAGAAAGCTGGGTTCAACGCAGTGGCGGGGT
2_hypothetical	K2	Tb927.11.14710_for	GGGGACAAGTTTGTACAAAAAAGCAGGCTATGGTCAAGAAAATGGGTAGAGACGA	Tb927.11.14710_rev	GGGGACCACTTTGTACAAGAAAGCTGGGTTCATTGTTGCTGATTTCGTTGAATTTTGT
2_hypothetical	K3	Tb927.3.2990_for	GGGGACAAGTTTGTACAAAAAAGCAGGCTATGATTCGTCATGGACTCACTGGA	Tb927.3.2990_rev	GGGGACCACTTTGTACAAGAAAGCTGGGTTCACCTCTCCACCTCACGAC
2_hypothetical	K4	Tb927.10.11950_for	GGGGACAAGTTTGTACAAAAAAGCAGGCTATGAAAAGATATATTCAACACAAGGAAAGT	Tb927.10.11950_rev	GGGGACCACTTTGTACAAGAAAGCTGGGTTCACTTAAAGTAACTTTTCATAAAGTGCTG
2_hypothetical	K5	Tb927.10.4970_for	GGGGACAAGTTTGTACAAAAAAGCAGGCTATGGAGGATATGGGCGACTTGG	Tb927.10.4970_rev	GGGGACCACTTTGTACAAGAAAGCTGGGTCTACCATTGAATCTCTTTGAGAATTGAATT
2_hypothetical	K6	Tb927.6.4500_for	GGGGACAAGTTTGTACAAAAAAGCAGGCTATGCGGCTCCTCTATGTCATCC	Tb927.6.4500_rev	GGGGACCACTTTGTACAAGAAAGCTGGGTTCATTTAACTGCCTTGGAGCGAC
2_hypothetical	K7	Tb927.4.4350_for	GGGGACAAGTTTGTACAAAAAAGCAGGCTATGTACGCACTACTGCAGCTTC	Tb927.4.4350_rev	GGGGACCACTTTGTACAAGAAAGCTGGGTTTATACAAACGACTTGCGCTCCA
2_hypothetical	K8	Tb927.11.430_for	GGGGACAAGTTTGTACAAAAAAGCAGGCTATGACACAAGGAATTTCTGAGGCTTT	Tb927.11.430_rev	GGGGACCACTTTGTACAAGAAAGCTGGGTTCATTGCCACTCTCCTTCACCT
2_hypothetical	K9	Tb927.8.3420_for	GGGGACAAGTTTGTACAAAAAAGCAGGCTATGCACGAGTTGGAGTTGCC	Tb927.8.3420_rev	GGGGACCACTTTGTACAAGAAAGCTGGGTTCACCTACTGCACTTTGACAAGTTAAT
2_hypothetical	K10	Tb927.9.11700_for	GGGGACAAGTTTGTACAAAAAAGCAGGCTATGCAGAACTCTGCCTTCAGAGT	Tb927.9.11700_rev	GGGGACCACTTTGTACAAGAAAGCTGGGTTCAATTTAATTCAATGATGCGTGCCG
2_hypothetical	K11	Tb927.9.5370_for	GGGGACAAGTTTGTACAAAAAAGCAGGCTATGCGCTCTAAGATGGAACTGTCA	Tb927.9.5370_rev	GGGGACCACTTTGTACAAGAAAGCTGGGTTTAGCCGCATCCTCTAAGAGAAACG
2_hypothetical	K12	Tb927.10.7320_for	GGGGACAAGTTTGTACAAAAAAGCAGGCTATGGACTCAATCAATAGCGTTACGTT	Tb927.10.7320_rev	GGGGACCACTTTGTACAAGAAAGCTGGGTTCAAGCTGTACTTGCGATAATTTTCCT
2_hypothetical	K13	Tb927.7.6550_for	GGGGACAAGTTTGTACAAAAAAGCAGGCTATGAATCTAGCGAAGAAAATCATCGTGT	Tb927.7.6550_rev	GGGGACCACTTTGTACAAGAAAGCTGGGTTCAAGGTCTCGGTGATGTTGCA
2_hypothetical	K14	Tb927.11.8900_for	GGGGACAAGTTTGTACAAAAAAGCAGGCTATGAGTGTAACCCTTTCAGAGCCT	Tb927.11.8900_rev	GGGGACCACTTTGTACAAGAAAGCTGGGTCTATTGCTGATTGCAACTGGTCCT
2_hypothetical	K15	Tb927.9.12390_for	GGGGACAAGTTTGTACAAAAAAGCAGGCTATGCAAACCAATGAACGTGTCGT	Tb927.9.12390_rev	GGGGACCACTTTGTACAAGAAAGCTGGGTTTACTCTTCGTGTTGCTCACACAC
2_hypothetical	K16	Tb927.3.5410_for	GGGGACAAGTTTGTACAAAAAAGCAGGCTATGTTACGACTCTCCAGTCGGTG	Tb927.3.5410_rev	GGGGACCACTTTGTACAAGAAAGCTGGGTTCAGGAACCGCGTGTTCCT
2_hypothetical	K17	Tb927.4.520_for	GGGGACAAGTTTGTACAAAAAAGCAGGCTATGAACAAACAACTGCTTCAGACTCT	Tb927.4.520_rev	GGGGACCACTTTGTACAAGAAAGCTGGGTCTAATAATGTTTCTTGGGTGTCTTTTTCAT
2_hypothetical	K18	Tb927.4.1190_for	GGGGACAAGTTTGTACAAAAAAGCAGGCTATGGTGGATGTTCATCGAAAGGAAG	Tb927.4.1190_rev	GGGGACCACTTTGTACAAGAAAGCTGGGTCTAAAAAAATTCGAGCAGCGATGCT
2_hypothetical	K19	Tb927.6.2410_for	GGGGACAAGTTTGTACAAAAAAGCAGGCTATGAAGAGACCATGGGGTGAGTC	Tb927.6.2410_rev	GGGGACCACTTTGTACAAGAAAGCTGGGTCTAATCCGCATGCTTCGTAGATCC
2_hypothetical	K20	Tb927.9.15010_for	GGGGACAAGTTTGTACAAAAAAGCAGGCTATGCGCCGTACTGCCTTCT	Tb927.9.15010_rev	GGGGACCACTTTGTACAAGAAAGCTGGGTCTACGTACACGCCTTCAGCAAC
2_hypothetical	K21	Tb927.4.3010_for	GGGGACAAGTTTGTACAAAAAAGCAGGCTATGCTAAGAAGATGGTCTGAATGTGG	Tb927.4.3010_rev	GGGGACCACTTTGTACAAGAAAGCTGGGTTCATGCAGCAAATCGTCGTTCC
2_hypothetical	K22	Tb927.4.1290_for	GGGGACAAGTTTGTACAAAAAAGCAGGCTATGTCGACAACCCCTTACGATGA	Tb927.4.1290_rev	GGGGACCACTTTGTACAAGAAAGCTGGGTTCATTTGACTTCATCTGATTCTGCTGC
2_hypothetical	K23	Tb927.3.2850_for	GGGGACAAGTTTGTACAAAAAAGCAGGCTATGATGCTGAGCAATGCGCA	Tb927.3.2850_rev	GGGGACCACTTTGTACAAGAAAGCTGGGTTCACCCCCTAAAGAACCCAATGA
2_hypothetical	K24	Tb927.11.3430_for	GGGGACAAGTTTGTACAAAAAAGCAGGCTATGAAACCGGAAGGTGCAAAAGG	Tb927.11.3430_rev	GGGGACCACTTTGTACAAGAAAGCTGGGTCTACTTCGTTGCTTTCGGGGG
2_hypothetical	L1	Tb927.4.3040_for	GGGGACAAGTTTGTACAAAAAAGCAGGCTATGCGGCGGAGCGTCTGT	Tb927.4.3040_rev	GGGGACCACTTTGTACAAGAAAGCTGGGTTTATGGTTCATCATCTACGATTTGATCATT
2_hypothetical	L2	Tb927.9.7160_for	GGGGACAAGTTTGTACAAAAAAGCAGGCTATGGAGGACGTGAGGCCTG	Tb927.9.7160_rev	GGGGACCACTTTGTACAAGAAAGCTGGGTCTAAGACCCGTGTTCAAACATTTTCTTAC
2_hypothetical	L3	Tb927.11.3170_for	GGGGACAAGTTTGTACAAAAAAGCAGGCTATGGACTTTAACTTTCTATGGGAGATACCG	Tb927.11.3170_rev	GGGGACCACTTTGTACAAGAAAGCTGGGTCTACCGTATGAAGTAGCGCTGTAC
2_hypothetical	L4	Tb927.2.1740_for	GGGGACAAGTTTGTACAAAAAAGCAGGCTATGTCTCTAGGGAGTGCGCTT	Tb927.2.1740_rev	GGGGACCACTTTGTACAAGAAAGCTGGGTTCATCTGTCCACTTGCAGTTGTTT
2_hypothetical	L5	Tb927.4.1440_for	GGGGACAAGTTTGTACAAAAAAGCAGGCTATGCCACGCTCCCTGTTTCG	Tb927.4.1440_rev	GGGGACCACTTTGTACAAGAAAGCTGGGTTCACCGCGTCACCGGGGA
2_hypothetical	L6	Tb927.6.4080_for	GGGGACAAGTTTGTACAAAAAAGCAGGCTATGTTGCGATTTACTCGTCTTTTTCG	Tb927.6.4080_rev	GGGGACCACTTTGTACAAGAAAGCTGGGTCTAATAAAGTGCGAACATGGAGTTCATTT

2_hypothetical	L7	Tb927.8.8030_for	GGGGACAAGTTTGTACAAAAAAGCAGGCTATGAATAAGATGCACATATTGCCACCA	Tb927.8.8030_rev	GGGGACCACTTTGTACAAGAAAGCTGGGTTTACACAAATGATTTACGTTCCAAGAAACG
2_hypothetical	L8	Tb927.11.15420_for	GGGGACAAGTTTGTACAAAAAAGCAGGCTATGAACCAAGATTTGGACAAGGAGC	Tb927.11.15420_rev	GGGGACCACTTTGTACAAGAAAGCTGGGTCTACAACGACACAATGGTGACAGT
2_hypothetical	L9	Tb927.11.5810_for	GGGGACAAGTTTGTACAAAAAAGCAGGCTATGAGTGCACGGGGTTCCC	Tb927.11.5810_rev	GGGGACCACTTTGTACAAGAAAGCTGGGTCTATTTTGATCCTTTGGGGCGCC
2_hypothetical	L10	Tb927.7.3220_for	GGGGACAAGTTTGTACAAAAAAGCAGGCTATGCGAGCTGAAAAGGTGACAG	Tb927.7.3220_rev	GGGGACCACTTTGTACAAGAAAGCTGGGTTCACTCGGGAACATTTTGCGC
2_hypothetical	L11	Tb927.8.7500_for	GGGGACAAGTTTGTACAAAAAAGCAGGCTATGGAGAGAGTGGGTGTGGC	Tb927.8.7500_rev	GGGGACCACTTTGTACAAGAAAGCTGGGTTCAATCGATAGACATCCACGCAGA
2_hypothetical	L12	Tb927.7.5520_for	GGGGACAAGTTTGTACAAAAAAGCAGGCTATGACACCTCTTGGTTTTGATTTTACTTCA	Tb927.7.5520_rev	GGGGACCACTTTGTACAAGAAAGCTGGGTTTAAAGGAGTTTGAAATGACCTGTTACACG
2_hypothetical	L13	Tb927.11.15440_for	GGGGACAAGTTTGTACAAAAAAGCAGGCTATGTCAGCCCTTCTTCCAAGGG	Tb927.11.15440_rev	GGGGACCACTTTGTACAAGAAAGCTGGGTTCAACCCTCATTAGAAACTATGCCATAGG
2_hypothetical	L14	Tb927.9.6390_for	GGGGACAAGTTTGTACAAAAAAGCAGGCTATGTCATGTGGGAAGCCCACG	Tb927.9.6390_rev	GGGGACCACTTTGTACAAGAAAGCTGGGTTCACCGATGTTTCCTGATTATCGAC
2_hypothetical	L15	Tb927.9.10350_for	GGGGACAAGTTTGTACAAAAAAGCAGGCTATGTCTGTTAATCAGCTGCAGGAC	Tb927.9.10350_rev	GGGGACCACTTTGTACAAGAAAGCTGGGTTTATTTACCCCGTAACATGTCAAGTCG
2_hypothetical	L16	Tb927.11.9740_for	GGGGACAAGTTTGTACAAAAAAGCAGGCTATGGCTTCTGGTAGTGGTAGAGG	Tb927.11.9740_rev	GGGGACCACTTTGTACAAGAAAGCTGGGTTCACAATTGAACAATGGGAGTATAACTTAA
2_hypothetical	L17	Tb927.10.15810_for	GGGGACAAGTTTGTACAAAAAAGCAGGCTATGGGTAAGAAAAAGAACACGGCG	Tb927.10.15810_rev	GGGGACCACTTTGTACAAGAAAGCTGGGTTTAAGCTGGAGCTCCATAGACCTC
2_hypothetical	L18	Tb927.10.12930_for	GGGGACAAGTTTGTACAAAAAAGCAGGCTATGGACCAATTCACAAAACCACTGG	Tb927.10.12930_rev	GGGGACCACTTTGTACAAGAAAGCTGGGTTTAGTTGACATTTGTGCTTATCCCTACG
2_hypothetical	L19	Tb927.7.5870_for	GGGGACAAGTTTGTACAAAAAAGCAGGCTATGAAACGATGGGATGAAAAGTTAACGT	Tb927.7.5870_rev	GGGGACCACTTTGTACAAGAAAGCTGGGTTCAAGTCTTTGATGTGAGAACGGG
2_hypothetical	L20	Tb927.10.940_for	GGGGACAAGTTTGTACAAAAAAGCAGGCTATGACATTAGGGCCCACAACCA	Tb927.10.940_rev	GGGGACCACTTTGTACAAGAAAGCTGGGTTCACTCCAGTTGTCTTTCACTCCC
2_hypothetical	L21	Tb927.11.8100_for	GGGGACAAGTTTGTACAAAAAAGCAGGCTATGGCAGATGATGAACCCGAATAC	Tb927.11.8100_rev	GGGGACCACTTTGTACAAGAAAGCTGGGTTCATTCGATTTCATCAACACCCACG
2_hypothetical	L22	Tb927.3.4930_for	GGGGACAAGTTTGTACAAAAAAGCAGGCTATGAGAGAAACTGCTATTATGAACAACAGA	Tb927.3.4930_rev	GGGGACCACTTTGTACAAGAAAGCTGGGTTCAGGAAAAAAATATGTGAAGTAAAGCGTC
2_hypothetical	L23	Tb927.5.3190_for	GGGGACAAGTTTGTACAAAAAAGCAGGCTATGGGGCCGAAAAAACAACTTCG	Tb927.5.3190_rev	GGGGACCACTTTGTACAAGAAAGCTGGGTTCACGGTGGAAGCATCAATGTC
2_hypothetical	L24	Tb927.10.13980_for	GGGGACAAGTTTGTACAAAAAAGCAGGCTATGTCGATGATCGCCATGAGTTG	Tb927.10.13980_rev	GGGGACCACTTTGTACAAGAAAGCTGGGTCTACGATGGTCCGAGACTTAGGT
2_hypothetical	M1	Tb927.6.3770_for	GGGGACAAGTTTGTACAAAAAAGCAGGCTATGCTCATAGTTAAGACTGAAGGCG	Tb927.6.3770_rev	GGGGACCACTTTGTACAAGAAAGCTGGGTTTAACAGTGTTCTTTGTCTAATGGGGC
2_hypothetical	M2	Tb927.11.10250_for	GGGGACAAGTTTGTACAAAAAAGCAGGCTATGGTGGAAAAGCACATGTACGAC	Tb927.11.10250_rev	GGGGACCACTTTGTACAAGAAAGCTGGGTTTACTCAAGCCCTCTGGCCTT
2_hypothetical	M3	Tb927.6.3340_for	GGGGACAAGTTTGTACAAAAAAGCAGGCTATGGTTTACTACTTCACTCTTGCAGC	Tb927.6.3340_rev	GGGGACCACTTTGTACAAGAAAGCTGGGTTCACATGAAGTCATCTTCATCTGGAATATG
2_hypothetical	M4	Tb927.4.1580_for	GGGGACAAGTTTGTACAAAAAAGCAGGCTATGTCAAGAGGAGAACGCGGT	Tb927.4.1580_rev	GGGGACCACTTTGTACAAGAAAGCTGGGTCTAGTTATCCGAGGCGGACTCA
2_hypothetical	M5	Tb927.8.7130_for	GGGGACAAGTTTGTACAAAAAAGCAGGCTATGAACGCACTAAGCGGGAAG	Tb927.8.7130_rev	GGGGACCACTTTGTACAAGAAAGCTGGGTCTACGAGCCGTATTTCGGTAAACA
2_hypothetical	M6	Tb927.9.6620_for	GGGGACAAGTTTGTACAAAAAAGCAGGCTATGGACGCCGGAAGCGAT	Tb927.9.6620_rev	GGGGACCACTTTGTACAAGAAAGCTGGGTTTAATCCTGTAGCTTCTTTCGCTTCTC
2_hypothetical	M7	Tb927.5.740_for	GGGGACAAGTTTGTACAAAAAAGCAGGCTATGCCCTCCACCATCTGTCAC	Tb927.5.740_rev	GGGGACCACTTTGTACAAGAAAGCTGGGTTTAATGAAAGAAGAAAAAAAAGAAGAACAA
2_hypothetical	M8	Tb927.9.2160_for	GGGGACAAGTTTGTACAAAAAAGCAGGCTATGGGTCAGCGAGGCTCC	Tb927.9.2160_rev	GGGGACCACTTTGTACAAGAAAGCTGGGTTTACATACGCCGCACCGC
2_hypothetical	M9	Tb927.7.6190_for	GGGGACAAGTTTGTACAAAAAAGCAGGCTATGAGCAAAGACGGGAATTGCG	Tb927.7.6190_rev	GGGGACCACTTTGTACAAGAAAGCTGGGTTCACAAATCATCTTCACATGTGCAAC
2_hypothetical	M10	Tb927.9.8550_for	GGGGACAAGTTTGTACAAAAAAGCAGGCTATGCCTCATAACGATCCGGATGG	Tb927.9.8550_rev	GGGGACCACTTTGTACAAGAAAGCTGGGTTTATCGGCGCTTTTTCGTCTTACG
2_hypothetical	M11	Tb927.11.7330_for	GGGGACAAGTTTGTACAAAAAAGCAGGCTATGTCAACGCAGCTGGTTCG	Tb927.11.7330_rev	GGGGACCACTTTGTACAAGAAAGCTGGGTTCACAATAACATCGTGCTCTGCAC
2_hypothetical	M12	Tb927.9.2420_for	GGGGACAAGTTTGTACAAAAAAGCAGGCTATGCCGTATTACGCGGAGGA	Tb927.9.2420_rev	GGGGACCACTTTGTACAAGAAAGCTGGGTTTAGCTGTCATGTTGCTTGGTAATAACT
2_hypothetical	M13	Tb927.11.16380_for	GGGGACAAGTTTGTACAAAAAAGCAGGCTATGCTTCCAGGCAAACGTAGC	Tb927.11.16380_rev	GGGGACCACTTTGTACAAGAAAGCTGGGTTCAGCTACTCCTCTTCATGAGTTCC
2_hypothetical	M14	Tb927.7.2290_for	GGGGACAAGTTTGTACAAAAAAGCAGGCTATGAAAACCGCAAAGGTGAACAGT	Tb927.7.2290_rev	GGGGACCACTTTGTACAAGAAAGCTGGGTTCAGACTGCGAGTAAGGGAAGC
2_hypothetical	M15	Tb927.9.14140_for	GGGGACAAGTTTGTACAAAAAAGCAGGCTATGGCAACTGGAAGTCAAATCCC	Tb927.9.14140_rev	GGGGACCACTTTGTACAAGAAAGCTGGGTCTACTGCAACCCTGAAGTGCG
2_hypothetical	M16	Tb927.11.9320_for	GGGGACAAGTTTGTACAAAAAAGCAGGCTATGTATCGCTCAGTGGGAGAAGC	Tb927.11.9320_rev	GGGGACCACTTTGTACAAGAAAGCTGGGTTCATCCACCGTATGAGGGACG
2_hypothetical	M17	Tb927.11.10530_for	GGGGACAAGTTTGTACAAAAAAGCAGGCTATGCCACGCCACCACACG	Tb927.11.10530_rev	GGGGACCACTTTGTACAAGAAAGCTGGGTCTAAAATTCGCGAGTAGCTGCGA
2_hypothetical	M18	Tb927.7.4280_for	GGGGACAAGTTTGTACAAAAAAGCAGGCTATGGTGATGAAGGCTTTCCCCC	Tb927.7.4280_rev	GGGGACCACTTTGTACAAGAAAGCTGGGTTTAATCCCTTTCTTCAAGTTTCGGACG
2_hypothetical	M19	Tb927.11.970_for	GGGGACAAGTTTGTACAAAAAAGCAGGCTATGGATTGGACTGATCGTGTCCT	Tb927.11.970_rev	GGGGACCACTTTGTACAAGAAAGCTGGGTTCACCCACTGTTGTAAGGTGTTATGG
2_hypothetical	M20	Tb927.8.6900_for	GGGGACAAGTTTGTACAAAAAAGCAGGCTATGAGTCAACATGGCTTGGTTAGTG	Tb927.8.6900_rev	GGGGACCACTTTGTACAAGAAAGCTGGGTTCATGTGGCAACGGATCTGAAATC
2_hypothetical	M21	Tb927.11.11240_for	GGGGACAAGTTTGTACAAAAAAGCAGGCTATGTACAAGATAGATGGCGACGGC	Tb927.11.11240_rev	GGGGACCACTTTGTACAAGAAAGCTGGGTCTACGAAGTGCACAACGGCA
2_hypothetical	M22	Tb927.8.6630_for	GGGGACAAGTTTGTACAAAAAAGCAGGCTATGTCTCGGAATATTCACGTGAATTCA	Tb927.8.6630_rev	GGGGACCACTTTGTACAAGAAAGCTGGGTTTATGACAAATGCTTTCCAGGCGT
2_hypothetical	M23	Tb927.2.4620_for	GGGGACAAGTTTGTACAAAAAAGCAGGCTATGCCTTATCCCGATGTTTATGAACG	Tb927.2.4620_rev	GGGGACCACTTTGTACAAGAAAGCTGGGTTTATGATTTCGAGAAAAAGTATTTCCTCAG
2_hypothetical	N1	Tb927.9.9550_for	GGGGACAAGTTTGTACAAAAAAGCAGGCTATGTTGGATCACACAATTGCTATTGGT	Tb927.9.9550_rev	GGGGACCACTTTGTACAAGAAAGCTGGGTTCACTGTTGTTGCTGTTGCCA
2_hypothetical	N2	Tb927.5.3720_for	GGGGACAAGTTTGTACAAAAAAGCAGGCTATGCCAAAGCGGTCCAGGT	Tb927.5.3720_rev	GGGGACCACTTTGTACAAGAAAGCTGGGTTCACAAGACAAAGCGATCCGAAC
2_hypothetical	N3	Tb927.10.4630_for	GGGGACAAGTTTGTACAAAAAAGCAGGCTATGTTTGTTAGTGACAACGATGATTATCTC	Tb927.10.4630_rev	GGGGACCACTTTGTACAAGAAAGCTGGGTTTAACGACGCATGCAAGAGTGA
2_hypothetical	N4	Tb927.11.4140_for	GGGGACAAGTTTGTACAAAAAAGCAGGCTATGCTTTCCACTGCGTGCA	Tb927.11.4140_rev	GGGGACCACTTTGTACAAGAAAGCTGGGTTCAATCGAGGTCATATTCAAATTTAATCTT
2_hypothetical	N5	Tb927.6.4150_for	GGGGACAAGTTTGTACAAAAAAGCAGGCTATGTCTCGCCTTGGAGGAGATG	Tb927.6.4150_rev	GGGGACCACTTTGTACAAGAAAGCTGGGTCTACAGGACAAAGAATCCCCCAATTAC
2_hypothetical	N6	Tb927.11.10170_for	GGGGACAAGTTTGTACAAAAAAGCAGGCTATGTTGCGGCGTACTGTATGTG	Tb927.11.10170_rev	GGGGACCACTTTGTACAAGAAAGCTGGGTCTACTTGTTCACCTCCCCCTTCC
2_hypothetical	N7	Tb927.11.12910_for	GGGGACAAGTTTGTACAAAAAAGCAGGCTATGTCTGGTGTGTCAAGGGGG	Tb927.11.12910_rev	GGGGACCACTTTGTACAAGAAAGCTGGGTCTAGCCCTTCCACGTTGCTTT
2_hypothetical	N8	Tb927.10.2150_for	GGGGACAAGTTTGTACAAAAAAGCAGGCTATGGTTGGAACAGTATTACGCGTATATAC	Tb927.10.2150_rev	GGGGACCACTTTGTACAAGAAAGCTGGGTTCAAACAGTCTCCAGCCCGC
2_hypothetical	N9	Tb927.7.6110_for	GGGGACAAGTTTGTACAAAAAAGCAGGCTATGGAGGTGGAACTGGAGCC	Tb927.7.6110_rev	GGGGACCACTTTGTACAAGAAAGCTGGGTTTACACATGAATGCTCTTTCCCATTACA
2_hypothetical	N10	Tb927.11.14150_for	GGGGACAAGTTTGTACAAAAAAGCAGGCTATGCAGCCCATGGTGCTTC	Tb927.11.14150_rev	GGGGACCACTTTGTACAAGAAAGCTGGGTCTATTCAAGAGTCGCCAGAAGCG
2_hypothetical	N11	Tb927.11.2980_for	GGGGACAAGTTTGTACAAAAAAGCAGGCTATGGGTGATTTCGCGCTCATC	Tb927.11.2980_rev	GGGGACCACTTTGTACAAGAAAGCTGGGTTTAATCCTCAATCTCAATGTATTTCCTCAT
2_hypothetical	N13	Tb927.2.5500_for	GGGGACAAGTTTGTACAAAAAAGCAGGCTATGCTTCCGCACTCTCAGCC	Tb927.2.5500_rev	GGGGACCACTTTGTACAAGAAAGCTGGGTTTATGGCTTCCCATCGGTCGC
2_hypothetical	N14	Tb927.11.1720_for	GGGGACAAGTTTGTACAAAAAAGCAGGCTATGCAGCAGCAACAACAAGTACC	Tb927.11.1720_rev	GGGGACCACTTTGTACAAGAAAGCTGGGTTCAATTCCGCACAACACACCA
2_hypothetical	N15	Tb927.11.2110_for	GGGGACAAGTTTGTACAAAAAAGCAGGCTATGTCATTCATGTCATTGTTTCAGCG	Tb927.11.2110_rev	GGGGACCACTTTGTACAAGAAAGCTGGGTTTACTTCATAGCCGCCCGAAGA
2_hypothetical	N17	Tb927.10.10780_for	GGGGACAAGTTTGTACAAAAAAGCAGGCTATGCGCAATTCGTTGCGGT	Tb927.10.10780_rev	GGGGACCACTTTGTACAAGAAAGCTGGGTTCACTTTTCCCGCGGAACCT
2_hypothetical	N18	Tb927.9.1910_for	GGGGACAAGTTTGTACAAAAAAGCAGGCTATGCCTAACCCGTACACCGG	Tb927.9.1910_rev	GGGGACCACTTTGTACAAGAAAGCTGGGTTCAACATTTGTTAACCCTTTCGGTCG
2_hypothetical	N19	Tb927.9.2800_for	GGGGACAAGTTTGTACAAAAAAGCAGGCTATGGTTTTCTGCGTGTTTTCTTGTG	Tb927.9.2800_rev	GGGGACCACTTTGTACAAGAAAGCTGGGTTTAAATAGCAACCATGATACCTCTTTCGT
2_hypothetical	N20	Tb927.5.1270_for	GGGGACAAGTTTGTACAAAAAAGCAGGCTATGGAGCTTCTCAAGAAAACGGC	Tb927.5.1270_rev	GGGGACCACTTTGTACAAGAAAGCTGGGTCTATTCCGTTCGCACAACCTTAGA
2_hypothetical	N21	Tb927.8.5430_for	GGGGACAAGTTTGTACAAAAAAGCAGGCTATGGGCAACAACGGAAGTAGC	Tb927.8.5430_rev	GGGGACCACTTTGTACAAGAAAGCTGGGTCTATGCCGCCAGTCGAACAC
2_hypothetical	N22	Tb927.4.3180_for	GGGGACAAGTTTGTACAAAAAAGCAGGCTATGAGTACCACAGGAAGCTGGAC	Tb927.4.3180_rev	GGGGACCACTTTGTACAAGAAAGCTGGGTTTAACTGTCACTGCCTTTATTGGATTGA
2_hypothetical	N23	Tb927.5.1930_for	GGGGACAAGTTTGTACAAAAAAGCAGGCTATGCATTCCCTTTGGGTGCG	Tb927.5.1930_rev	GGGGACCACTTTGTACAAGAAAGCTGGGTCTACGCGTATCCTCTGCTGC
2_hypothetical	N24	Tb927.6.1270_for	GGGGACAAGTTTGTACAAAAAAGCAGGCTATGCCTTGCAACTGCCGTC	Tb927.6.1270_rev	GGGGACCACTTTGTACAAGAAAGCTGGGTCTAGAATATAGTGAATGTTTTCTTGAGCTC
2_hypothetical	O1	Tb927.10.1440_for	GGGGACAAGTTTGTACAAAAAAGCAGGCTATGAAGTCGTCCGACATATTCTTTGC	Tb927.10.1440_rev	GGGGACCACTTTGTACAAGAAAGCTGGGTTTACTTGTTGTACGGCAGACGG
2_hypothetical	O2	Tb927.2.3340_for	GGGGACAAGTTTGTACAAAAAAGCAGGCTATGTCTGAAAACCCCGCCCA	Tb927.2.3340_rev	GGGGACCACTTTGTACAAGAAAGCTGGGTTTACACCATTTCCACTCTCCTTGGA
2_hypothetical	O3	Tb927.5.3300_for	GGGGACAAGTTTGTACAAAAAAGCAGGCTATGTTACGCAGAGCTGTTGCA	Tb927.5.3300_rev	GGGGACCACTTTGTACAAGAAAGCTGGGTTTAGTTCGCGCTCGTCATATGC
2_hypothetical	O4	Tb927.11.13230_for	GGGGACAAGTTTGTACAAAAAAGCAGGCTATGAGAAGTAAAACGGCGGCAC	Tb927.11.13230_rev	GGGGACCACTTTGTACAAGAAAGCTGGGTCTAGTAGGCAAACCACCACCAGC
2_hypothetical	O5	Tb927.10.7860_for	GGGGACAAGTTTGTACAAAAAAGCAGGCTATGACCGTGTCAGAAGCCTCC	Tb927.10.7860_rev	GGGGACCACTTTGTACAAGAAAGCTGGGTCTACCGCGATCTGTTGGTTGC
2_hypothetical	O6	Tb927.5.3140_for	GGGGACAAGTTTGTACAAAAAAGCAGGCTATGTTGCCCATAACGCGGC	Tb927.5.3140_rev	GGGGACCACTTTGTACAAGAAAGCTGGGTTTACTCCTCTCCTGAAGGTGGGG
2_hypothetical	O7	Tb927.7.3480_for	GGGGACAAGTTTGTACAAAAAAGCAGGCTATGCTAACCGACACCCTCACA	Tb927.7.3480_rev	GGGGACCACTTTGTACAAGAAAGCTGGGTTTAAAAAAAAATGAAAAGTATATATATCAA
2_hypothetical	O8	Tb927.4.3960_for	GGGGACAAGTTTGTACAAAAAAGCAGGCTATGTCACTAGATATCTCACGATTCCTCC	Tb927.4.3960_rev	GGGGACCACTTTGTACAAGAAAGCTGGGTCTACAAACTCCACAACGATCGCA
2_hypothetical	O9	Tb927.10.6770_for	GGGGACAAGTTTGTACAAAAAAGCAGGCTATGCGTGCGCTGTTTGGC	Tb927.10.6770_rev	GGGGACCACTTTGTACAAGAAAGCTGGGTTCACAACCCCCCAGTCACG
2_hypothetical	O10	Tb927.10.13760_for	GGGGACAAGTTTGTACAAAAAAGCAGGCTATGCGGTGTCACCGAGTTTTT	Tb927.10.13760_rev	GGGGACCACTTTGTACAAGAAAGCTGGGTTTAGTTCAAGTGATCATCGGGGGA
2_hypothetical	O11	Tb927.9.8290_for	GGGGACAAGTTTGTACAAAAAAGCAGGCTATGATGCGGCGCATGTGG	Tb927.9.8290_rev	GGGGACCACTTTGTACAAGAAAGCTGGGTTCAACACTTCTCTCCCTCCTTATTATCTG
2_hypothetical	O12	Tb927.9.5390_for	GGGGACAAGTTTGTACAAAAAAGCAGGCTATGTCTATTGAGAGGCTTCGGCG	Tb927.9.5390_rev	GGGGACCACTTTGTACAAGAAAGCTGGGTTTAATCATCGCTAAGTTCATCGAAATCATC
2_hypothetical	O13	Tb927.10.12630_for	GGGGACAAGTTTGTACAAAAAAGCAGGCTATGGGCTATACGTTCTCGCGT	Tb927.10.12630_rev	GGGGACCACTTTGTACAAGAAAGCTGGGTCTACATCAAATTCTTCATCTTCTCCAGCA
2_hypothetical	O14	Tb927.7.6150_for	GGGGACAAGTTTGTACAAAAAAGCAGGCTATGTACTCTGTTCATTGGGAGGAAGA	Tb927.7.6150_rev	GGGGACCACTTTGTACAAGAAAGCTGGGTTTACACATGAATGCTCTTTCCCATTACA
2_hypothetical	O15	Tb927.9.2990_for	GGGGACAAGTTTGTACAAAAAAGCAGGCTATGGAGGAAGCGAAAGAGAATGTCC	Tb927.9.2990_rev	GGGGACCACTTTGTACAAGAAAGCTGGGTCTAATTCTCCCACGAAATTCTACTTCCC
2_hypothetical	O16	Tb927.3.2560_for	GGGGACAAGTTTGTACAAAAAAGCAGGCTATGCTGTGCCTACCAATAATACTGTTC	Tb927.3.2560_rev	GGGGACCACTTTGTACAAGAAAGCTGGGTTCAGAGAAATAGGAGCAACACTGAGG
2_hypothetical	O17	Tb927.10.6280_for	GGGGACAAGTTTGTACAAAAAAGCAGGCTATGGCTGATGCTTATCGAGAACG	Tb927.10.6280_rev	GGGGACCACTTTGTACAAGAAAGCTGGGTCTATGGACTCACAAAACTAATTAACTCTGC
2_hypothetical	O18	Tb927.5.4410_for	GGGGACAAGTTTGTACAAAAAAGCAGGCTATGGCAGGCCGTTTCCGT	Tb927.5.4410_rev	GGGGACCACTTTGTACAAGAAAGCTGGGTCTACCAGTACCTATCCACTTTCTTTGGT
2_hypothetical	O19	Tb927.11.9460_for	GGGGACAAGTTTGTACAAAAAAGCAGGCTATGACCAAAGTTTGGATGCCGT	Tb927.11.9460_rev	GGGGACCACTTTGTACAAGAAAGCTGGGTCTATGGTGTGTCGGTGGCTG
2_hypothetical	O20	Tb927.3.2550_for	GGGGACAAGTTTGTACAAAAAAGCAGGCTATGCTGTGCCTACCAATAATACTGTTC	Tb927.3.2550_rev	GGGGACCACTTTGTACAAGAAAGCTGGGTTCAGAGAAATAGGAGCAACACTGGA
2_hypothetical	O21	Tb927.8.5660_for	GGGGACAAGTTTGTACAAAAAAGCAGGCTATGATATCGCCCAGTACATTGCTG	Tb927.8.5660_rev	GGGGACCACTTTGTACAAGAAAGCTGGGTTCAGGAGTTCAAGTATAACACCAGCA
2_hypothetical	O23	Tb927.11.2400_for	GGGGACAAGTTTGTACAAAAAAGCAGGCTATGGGTTGTGGCGGTTCCA	Tb927.11.2400_rev	GGGGACCACTTTGTACAAGAAAGCTGGGTTTACTCGGATTTGTACTTCTGGAGACT
2_hypothetical	O24	Tb927.5.3110_for	GGGGACAAGTTTGTACAAAAAAGCAGGCTATGCTGAACCGGTCACGTTTG	Tb927.5.3110_rev	GGGGACCACTTTGTACAAGAAAGCTGGGTTCAGAAACCCAACCCCAGCA
2_hypothetical	P1	Tb927.9.14440_for	GGGGACAAGTTTGTACAAAAAAGCAGGCTATGTTCAGGGGAGGACAGTCC	Tb927.9.14440_rev	GGGGACCACTTTGTACAAGAAAGCTGGGTTTACCCCATATGTTCCACGGTTAGA
2_hypothetical	P2	Tb927.11.7190_for	GGGGACAAGTTTGTACAAAAAAGCAGGCTATGGGGAAGCGTGATTTGGAGA	Tb927.11.7190_rev	GGGGACCACTTTGTACAAGAAAGCTGGGTTTATCCCGCCGACGGAACG
2_hypothetical	P3	Tb927.7.6130_for	GGGGACAAGTTTGTACAAAAAAGCAGGCTATGTCAAATTCGCCGGCTAGTC	Tb927.7.6130_rev	GGGGACCACTTTGTACAAGAAAGCTGGGTTTACACATGAATGCTCTTTCCCATTACA
2_hypothetical	P4	Tb927.11.5890_for	GGGGACAAGTTTGTACAAAAAAGCAGGCTATGGCCACTGCGCGGTCG	Tb927.11.5890_rev	GGGGACCACTTTGTACAAGAAAGCTGGGTTCACAGTTCGTCAAAATACGGAATGC
2_hypothetical	P5	Tb927.8.7710_for	GGGGACAAGTTTGTACAAAAAAGCAGGCTATGGAAACCACGCACATAATCAACG	Tb927.8.7710_rev	GGGGACCACTTTGTACAAGAAAGCTGGGTTCACCAACAGTTGTTCCCAACG
2_hypothetical	P6	Tb927.9.4570_for	GGGGACAAGTTTGTACAAAAAAGCAGGCTATGACGGATTATGAGGAATTGCGG	Tb927.9.4570_rev	GGGGACCACTTTGTACAAGAAAGCTGGGTCTACCCCCGCCGTCCGCG
2_hypothetical	P7	Tb927.4.2980_for	GGGGACAAGTTTGTACAAAAAAGCAGGCTATGGGGCTCGTGCACAGT	Tb927.4.2980_rev	GGGGACCACTTTGTACAAGAAAGCTGGGTTCAGATTAATGACGCCAGCGTG
2_hypothetical	P8	Tb927.8.2290_for	GGGGACAAGTTTGTACAAAAAAGCAGGCTATGTTGATGTTTCATCTTAACGCCAATAG	Tb927.8.2290_rev	GGGGACCACTTTGTACAAGAAAGCTGGGTTCAACGCAGCCGTTTGCA
2_hypothetical	P9	Tb927.3.4410_for	GGGGACAAGTTTGTACAAAAAAGCAGGCTATGTCTTATAGTGACATTCAGAGCCTCG	Tb927.3.4410_rev	GGGGACCACTTTGTACAAGAAAGCTGGGTTCAAACCAGCCTATCGGAATCGT
2_hypothetical	P10	Tb927.4.790_for	GGGGACAAGTTTGTACAAAAAAGCAGGCTATGGCGAACATATCTCCTAATTCAGATG	Tb927.4.790_rev	GGGGACCACTTTGTACAAGAAAGCTGGGTTTAAAATAACTGGGCTCGCCTTGAT
2_hypothetical	P11	Tb927.10.14640_for	GGGGACAAGTTTGTACAAAAAAGCAGGCTATGGGCGGCGATGGCCAA	Tb927.10.14640_rev	GGGGACCACTTTGTACAAGAAAGCTGGGTTCACGTGTTTGAGCGCCC
2_hypothetical	P12	Tb927.7.5800_for	GGGGACAAGTTTGTACAAAAAAGCAGGCTATGGGCACACACATTATAGCGTATG	Tb927.7.5800_rev	GGGGACCACTTTGTACAAGAAAGCTGGGTCTACCACGGAAACATATCGCTACC
2_hypothetical	P13	Tb927.11.4870_for	GGGGACAAGTTTGTACAAAAAAGCAGGCTATGTCCGGTAGAATTTACAAGCTTTTCA	Tb927.11.4870_rev	GGGGACCACTTTGTACAAGAAAGCTGGGTCTACGATTTAGGGATAGCTGAGATGTGA
2_hypothetical	P14	Tb927.11.5460_for	GGGGACAAGTTTGTACAAAAAAGCAGGCTATGACAAATTCCCCAGATGACGATG	Tb927.11.5460_rev	GGGGACCACTTTGTACAAGAAAGCTGGGTTCAATCCCGCGTTCGCTTAAA
2_hypothetical	P15	Tb927.9.3770_for	GGGGACAAGTTTGTACAAAAAAGCAGGCTATGAGTGAGTCTAGTGCGACCAA	Tb927.9.3770_rev	GGGGACCACTTTGTACAAGAAAGCTGGGTTTAGGATTGCCGTTTCTTTTTTGAGGT
2_hypothetical	P16	Tb927.3.2220_for	GGGGACAAGTTTGTACAAAAAAGCAGGCTATGGAGGAGGACTACCATGAGTATGA	Tb927.3.2220_rev	GGGGACCACTTTGTACAAGAAAGCTGGGTTCACATAAAGTTGTCATCACCATCGG
2_hypothetical	P17	Tb927.10.9220_for	GGGGACAAGTTTGTACAAAAAAGCAGGCTATGTCTGAGGAGTCCTTGACGTT	Tb927.10.9220_rev	GGGGACCACTTTGTACAAGAAAGCTGGGTCTACCGAAATCCACTGAACCTCCC
2_hypothetical	P18	Tb927.11.13530_for	GGGGACAAGTTTGTACAAAAAAGCAGGCTATGAGTGAAGAAGATGGTGAAGGTCT	Tb927.11.13530_rev	GGGGACCACTTTGTACAAGAAAGCTGGGTCTATGTAGAGTGCGCTTCGCC
2_hypothetical	P19	Tb927.8.2300_for	GGGGACAAGTTTGTACAAAAAAGCAGGCTATGGATGATGTGAAGAGGAAGTCCG	Tb927.8.2300_rev	GGGGACCACTTTGTACAAGAAAGCTGGGTCTAAAAGCCGAACAACATTCTGTATAAGG
2_hypothetical	P20	Tb927.4.1000_for	GGGGACAAGTTTGTACAAAAAAGCAGGCTATGATGGAGCTCCCTATGGAAATGA	Tb927.4.1000_rev	GGGGACCACTTTGTACAAGAAAGCTGGGTCTACAGCTCCTGAGATCTTTCAGATGA
2_hypothetical	P21	Tb927.11.10070_for	GGGGACAAGTTTGTACAAAAAAGCAGGCTATGGTCCTCACAGGGGTCAAAC	Tb927.11.10070_rev	GGGGACCACTTTGTACAAGAAAGCTGGGTTCACTCCAACTGCCCGTTTG
2_hypothetical	P22	Tb927.3.4200_for	GGGGACAAGTTTGTACAAAAAAGCAGGCTATGGCGATCCCTTTGGGGG	Tb927.3.4200_rev	GGGGACCACTTTGTACAAGAAAGCTGGGTTCACACCAAATCATTGCGCCA
2_hypothetical	P23	Tb927.8.7310_for	GGGGACAAGTTTGTACAAAAAAGCAGGCTATGAGGAGAGTGCTGCTATTCCTG	Tb927.8.7310_rev	GGGGACCACTTTGTACAAGAAAGCTGGGTTCAAAGTAACGTGCTGTACAAAAAAGG
2_hypothetical	P24	Tb927.11.7550_for	GGGGACAAGTTTGTACAAAAAAGCAGGCTATGCTTCAAAACTGTGAGGTTGCT	Tb927.11.7550_rev	GGGGACCACTTTGTACAAGAAAGCTGGGTTTATTCAGACAAACGATCTGAGTTTGTGT
2_known	A1	Tb927.8.5230_for	GGGGACAAGTTTGTACAAAAAAGCAGGCTATGTCCTTGTCATCCACCACAATC	Tb927.8.5230_rev	GGGGACCACTTTGTACAAGAAAGCTGGGTCTACCCGCCGGAAGGGGA
2_known	A2	Tb927.9.13150_for	GGGGACAAGTTTGTACAAAAAAGCAGGCTATGGGATACAGGTTGTTGCGTG	Tb927.9.13150_rev	GGGGACCACTTTGTACAAGAAAGCTGGGTTTATGACGTGCAGGTGGCAC
2_known	A3	Tb927.10.3760_for	GGGGACAAGTTTGTACAAAAAAGCAGGCTATGTCATCGAACCGGTACACTGC	Tb927.10.3760_rev	GGGGACCACTTTGTACAAGAAAGCTGGGTCTACACAACAAGATCCGCCTCG
2_known	A4	Tb927.10.8310_for	GGGGACAAGTTTGTACAAAAAAGCAGGCTATGAAACGACAGAGGTCGGGA	Tb927.10.8310_rev	GGGGACCACTTTGTACAAGAAAGCTGGGTTTATGATAGCGGCACCCATAGTAACA
2_known	A5	Tb927.5.2690_for	GGGGACAAGTTTGTACAAAAAAGCAGGCTATGCCAATAAATAACTGTGAATTGGACG	Tb927.5.2690_rev	GGGGACCACTTTGTACAAGAAAGCTGGGTCTAGCATCCTAACACCGATTTTTTGTAGC
2_known	A6	Tb927.11.11730_for	GGGGACAAGTTTGTACAAAAAAGCAGGCTATGAAGTTTTCACGCGTTGCCA	Tb927.11.11730_rev	GGGGACCACTTTGTACAAGAAAGCTGGGTTTACTGAAACGTTATGGTAGGTGTTTCTAC
2_known	A7	Tb927.7.6200_for	GGGGACAAGTTTGTACAAAAAAGCAGGCTATGAAACCCATACCAGTAGCCATGA	Tb927.7.6200_rev	GGGGACCACTTTGTACAAGAAAGCTGGGTTCAATCGGCAGGACTGTTACCA
2_known	A8	Tb927.10.14780_for	GGGGACAAGTTTGTACAAAAAAGCAGGCTATGCCCGCAGGCATGTGT	Tb927.10.14780_rev	GGGGACCACTTTGTACAAGAAAGCTGGGTCTAGACCTTTAAGAAGGGATGTTGCAA
2_known	A9	Tb927.4.4170_for	GGGGACAAGTTTGTACAAAAAAGCAGGCTATGTACAGGTGCCGTTCCGC	Tb927.4.4170_rev	GGGGACCACTTTGTACAAGAAAGCTGGGTCTAGTCCACGCGACTGCAGT
2_known	A10	Tb927.9.3820_for	GGGGACAAGTTTGTACAAAAAAGCAGGCTATGTCGGATGTAAAAGGGGCGC	Tb927.9.3820_rev	GGGGACCACTTTGTACAAGAAAGCTGGGTTCACCTAGACATGTTGTATATCGCCC
2_known	A11	Tb927.3.1890_for	GGGGACAAGTTTGTACAAAAAAGCAGGCTATGGCCGAAACCCGAAAAGAAG	Tb927.3.1890_rev	GGGGACCACTTTGTACAAGAAAGCTGGGTCTAGAATTCTTCCATTTGTGGCTCAGG
2_known	A12	Tb927.9.11870_for	GGGGACAAGTTTGTACAAAAAAGCAGGCTATGGGCGCTAGTGAAGCAAAG	Tb927.9.11870_rev	GGGGACCACTTTGTACAAGAAAGCTGGGTTCAATAATTCGGTAGACGGCTCAGC
2_known	A13	Tb927.2.6070_for	GGGGACAAGTTTGTACAAAAAAGCAGGCTATGTTCAGCGGGCTTCAGC	Tb927.2.6070_rev	GGGGACCACTTTGTACAAGAAAGCTGGGTCTAAGAATCTGATTCTTTAGAGGCTCCAC
2_known	A14	Tb927.10.14930_for	GGGGACAAGTTTGTACAAAAAAGCAGGCTATGAACGATGCCGGTATGGGA	Tb927.10.14930_rev	GGGGACCACTTTGTACAAGAAAGCTGGGTTTAACCACCACCTCGAGTGTCA
2_known	A15	Tb927.10.11980_for	GGGGACAAGTTTGTACAAAAAAGCAGGCTATGGATACTTCCTCGGAGCTGTT	Tb927.10.11980_rev	GGGGACCACTTTGTACAAGAAAGCTGGGTTTACATTAGTGTACAACTGCTCGAATTGT
2_known	A16	Tb927.9.12290_for	GGGGACAAGTTTGTACAAAAAAGCAGGCTATGTCTCATCCCGACAATGACGC	Tb927.9.12290_rev	GGGGACCACTTTGTACAAGAAAGCTGGGTCTACACTGATGGTTGCACATCGG
2_known	A17	Tb927.8.5810_for	GGGGACAAGTTTGTACAAAAAAGCAGGCTATGACTGAGGAGTATGTCCACACG	Tb927.8.5810_rev	GGGGACCACTTTGTACAAGAAAGCTGGGTCTAATCCCTGGTGAGGGCGT
2_known	A18	Tb927.3.3770_for	GGGGACAAGTTTGTACAAAAAAGCAGGCTATGAGTGCAACAGAGGCGGT	Tb927.3.3770_rev	GGGGACCACTTTGTACAAGAAAGCTGGGTTTAGTACCTAGAGAACCGTGTCACC
2_known	A19	Tb927.7.4420_for	GGGGACAAGTTTGTACAAAAAAGCAGGCTATGTCCTCGCGTTACGACTCC	Tb927.7.4420_rev	GGGGACCACTTTGTACAAGAAAGCTGGGTTTACGGCGAGGCCAAGCG
2_known	A20	Tb927.10.12780_for	GGGGACAAGTTTGTACAAAAAAGCAGGCTATGCCCTCAAAGAAATCTATGGCAA	Tb927.10.12780_rev	GGGGACCACTTTGTACAAGAAAGCTGGGTTTATAACCCGTAAGGATTATGTCGGTAGA

2_known	A21	Tb927.10.14440_for	GGGGACAAGTTTGTACAAAAAAGCAGGCTATGGAGGAAAGTGTATATAAACCACTTGG	Tb927.10.14440_rev	GGGGACCACTTTGTACAAGAAAGCTGGGTTCATGTTCCTATCCCACACTCTCTG
2_known	A22	Tb927.10.4440_for	GGGGACAAGTTTGTACAAAAAAGCAGGCTATGAGGAAACCCGGGCGG	Tb927.10.4440_rev	GGGGACCACTTTGTACAAGAAAGCTGGGTCTAAACGCTCCTGCGGCG
2_known	A23	Tb927.7.5160_for	GGGGACAAGTTTGTACAAAAAAGCAGGCTATGAAGAACGCCCGCAGAGT	Tb927.7.5160_rev	GGGGACCACTTTGTACAAGAAAGCTGGGTTTACGACTTTAGCCAGTGCCCA
2_known	A24	Tb927.7.2700_for	GGGGACAAGTTTGTACAAAAAAGCAGGCTATGAAGGTATTTGCAGCTGCTGC	Tb927.7.2700_rev	GGGGACCACTTTGTACAAGAAAGCTGGGTTCAAAACTTGTAAACCATAGCCTCTGG
2_known	B1	Tb927.6.2780_for	GGGGACAAGTTTGTACAAAAAAGCAGGCTATGCGACGTAGCGTTATTCGG	Tb927.6.2780_rev	GGGGACCACTTTGTACAAGAAAGCTGGGTTTACATCAGTCTCCGCTTCTTCGC
2_known	B2	Tb927.6.4350_for	GGGGACAAGTTTGTACAAAAAAGCAGGCTATGACGTCTTCGTCAAAATCCAAGT	Tb927.6.4350_rev	GGGGACCACTTTGTACAAGAAAGCTGGGTCTACTCCATCTTGAAATCATCGCGT
2_known	B3	Tb927.7.2710_for	GGGGACAAGTTTGTACAAAAAAGCAGGCTATGAAAGCTCTGTTTGCCTCCG	Tb927.7.2710_rev	GGGGACCACTTTGTACAAGAAAGCTGGGTCTAAATTTTAAACACCATTTTCTGTATGTA
2_known	B4	Tb927.9.10950_for	GGGGACAAGTTTGTACAAAAAAGCAGGCTATGTTCGTGGATTACTACGCTGTAC	Tb927.9.10950_rev	GGGGACCACTTTGTACAAGAAAGCTGGGTTTAGATGTTAAAGAAAACCCCCCACG
2_known	B5	Tb927.9.10580_for	GGGGACAAGTTTGTACAAAAAAGCAGGCTATGTCCTCAGCAATGGCTCGC	Tb927.9.10580_rev	GGGGACCACTTTGTACAAGAAAGCTGGGTTTATTTGGCATCTTTTTCACTTTTCATATT
2_known	B6	Tb927.11.10510_for	GGGGACAAGTTTGTACAAAAAAGCAGGCTATGCTCCGTCGCAGTGGT	Tb927.11.10510_rev	GGGGACCACTTTGTACAAGAAAGCTGGGTCTATGCAGCCCCCACTTTACTG
2_known	B7	Tb927.7.1500_for	GGGGACAAGTTTGTACAAAAAAGCAGGCTATGGCCACCTCCCTTTCGG	Tb927.7.1500_rev	GGGGACCACTTTGTACAAGAAAGCTGGGTTTAATGACGTTGTTGCAGTTGCG
2_known	B8	Tb927.3.2980_for	GGGGACAAGTTTGTACAAAAAAGCAGGCTATGGTTTCCGAGGGCACTTCC	Tb927.3.2980_rev	GGGGACCACTTTGTACAAGAAAGCTGGGTTTACCGTTCCTCCATGAACTTCTTG
2_known	B9	Tb927.3.1820_for	GGGGACAAGTTTGTACAAAAAAGCAGGCTATGTCTGCCGCATTTGGTATCG	Tb927.3.1820_rev	GGGGACCACTTTGTACAAGAAAGCTGGGTCTAGCGGCGGTATTGCCC
2_known	B10	Tb927.6.3850_for	GGGGACAAGTTTGTACAAAAAAGCAGGCTATGCCATTGTTTTGTAGTTCTACCACC	Tb927.6.3850_rev	GGGGACCACTTTGTACAAGAAAGCTGGGTTCACGTGGATGGATATGGACAAACA
2_known	B11	Tb927.4.5790_for	GGGGACAAGTTTGTACAAAAAAGCAGGCTATGAGATTTGTTTCCAAGGGTGTAAGG	Tb927.4.5790_rev	GGGGACCACTTTGTACAAGAAAGCTGGGTTCACGCGTATTCCTCCTCTGC
2_known	B12	Tb927.9.15550_for	GGGGACAAGTTTGTACAAAAAAGCAGGCTATGAGCATCACTTTTAATAATTTATGGCTA	Tb927.9.15550_rev	GGGGACCACTTTGTACAAGAAAGCTGGGTTTACAGTACAGAACCGAGAAACAGTCC
2_known	B14	Tb927.10.5040_for	GGGGACAAGTTTGTACAAAAAAGCAGGCTATGGGTTTGGAGGATATTGATGTAGATGA	Tb927.10.5040_rev	GGGGACCACTTTGTACAAGAAAGCTGGGTTCAACTCGGTGGAAAGAACCAATC
2_known	B15	Tb927.11.3550_for	GGGGACAAGTTTGTACAAAAAAGCAGGCTATGTCGACAAAGCCAAAACCTTTAGT	Tb927.11.3550_rev	GGGGACCACTTTGTACAAGAAAGCTGGGTTTAATCCTTCTTCCGGTCCTCGC
2_known	B16	Tb927.11.2920_for	GGGGACAAGTTTGTACAAAAAAGCAGGCTATGTCACGATGTGGGCGC	Tb927.11.2920_rev	GGGGACCACTTTGTACAAGAAAGCTGGGTTTACCGGTCCGTTCCCTCG
2_known	B17	Tb927.9.12170_for	GGGGACAAGTTTGTACAAAAAAGCAGGCTATGGCGGGAGTTCTCACTGAA	Tb927.9.12170_rev	GGGGACCACTTTGTACAAGAAAGCTGGGTTTACTGCAAAGCATCAATGAAGCG
2_known	B18	Tb927.11.9180_for	GGGGACAAGTTTGTACAAAAAAGCAGGCTATGAGTAACGCTAACGCTGCTC	Tb927.11.9180_rev	GGGGACCACTTTGTACAAGAAAGCTGGGTTTACTGAGGAGAAAAGCATTTGGAGG
2_known	B19	Tb927.9.5190_for	GGGGACAAGTTTGTACAAAAAAGCAGGCTATGCTTGAGGCTCAGGTTCTGC	Tb927.9.5190_rev	GGGGACCACTTTGTACAAGAAAGCTGGGTTTACTCGGCGTCGTCCACC
2_known	B20	Tb927.7.1900_for	GGGGACAAGTTTGTACAAAAAAGCAGGCTATGGAGAAGTACCTCCTTGGCC	Tb927.7.1900_rev	GGGGACCACTTTGTACAAGAAAGCTGGGTTCAGTACTCGTTGAAATATGTATGACAAAG
2_known	B21	Tb927.2.1890_for	GGGGACAAGTTTGTACAAAAAAGCAGGCTATGAACAAACAAAGTTTGTATGAAGGTTTC	Tb927.2.1890_rev	GGGGACCACTTTGTACAAGAAAGCTGGGTTTAAATGTCGATACCTGTCGACAAATCA
2_known	B22	Tb927.10.4930_for	GGGGACAAGTTTGTACAAAAAAGCAGGCTATGGGGGAAACGCTGTCCA	Tb927.10.4930_rev	GGGGACCACTTTGTACAAGAAAGCTGGGTTCAACGCTCACTACAAAAGAACGC
2_known	B23	Tb927.7.1880_for	GGGGACAAGTTTGTACAAAAAAGCAGGCTATGGTTGGCGAGAGGACCG	Tb927.7.1880_rev	GGGGACCACTTTGTACAAGAAAGCTGGGTTCATTCCCACTCCCCACACTC
2_known	C1	Tb927.11.11510_for	GGGGACAAGTTTGTACAAAAAAGCAGGCTATGGCAAACTGTACGACTCAGGC	Tb927.11.11510_rev	GGGGACCACTTTGTACAAGAAAGCTGGGTTTACGCGGTTCTCCCAAGCC
2_known	C2	Tb927.11.440_for	GGGGACAAGTTTGTACAAAAAAGCAGGCTATGGGACGCAATAAGAAAAAGAGCA	Tb927.11.440_rev	GGGGACCACTTTGTACAAGAAAGCTGGGTCTAGTTTCCGTAACATTTGACCTTCTTCT
2_known	C3	Tb927.8.1030_for	GGGGACAAGTTTGTACAAAAAAGCAGGCTATGCGGACAGAGGGCCTC	Tb927.8.1030_rev	GGGGACCACTTTGTACAAGAAAGCTGGGTTCACCTCAATTCACGTTTGGAAAAAGA
2_known	C4	Tb927.11.16600_for	GGGGACAAGTTTGTACAAAAAAGCAGGCTATGTCACTTCCACCCGCCA	Tb927.11.16600_rev	GGGGACCACTTTGTACAAGAAAGCTGGGTTTATCCTTCCTTTAGGGGATGTGGG
2_known	C5	Tb927.11.10820_for	GGGGACAAGTTTGTACAAAAAAGCAGGCTATGAACTACCCGATGTATGTTGGC	Tb927.11.10820_rev	GGGGACCACTTTGTACAAGAAAGCTGGGTTTATCTCTTTGGTGTCTCCTCAGGC
2_known	C6	Tb927.10.8400_for	GGGGACAAGTTTGTACAAAAAAGCAGGCTATGCCGCCGACCCCATTA	Tb927.10.8400_rev	GGGGACCACTTTGTACAAGAAAGCTGGGTTTAGTCACTTAACTCCTGTGTAACCTTGT
2_known	C7	Tb927.4.3640_for	GGGGACAAGTTTGTACAAAAAAGCAGGCTATGACCCTAGTACAGACACTGCTG	Tb927.4.3640_rev	GGGGACCACTTTGTACAAGAAAGCTGGGTTTAGCGAGCTGCAATGATAGCAA
2_known	C8	Tb927.4.3620_for	GGGGACAAGTTTGTACAAAAAAGCAGGCTATGACCCTAGTACAGACACTGCTG	Tb927.4.3620_rev	GGGGACCACTTTGTACAAGAAAGCTGGGTTCAGCGAGCAGGTATAATGAGAAAGG
2_known	C9	Tb927.11.14870_for	GGGGACAAGTTTGTACAAAAAAGCAGGCTATGCCGAAGGAGCCCATTCT	Tb927.11.14870_rev	GGGGACCACTTTGTACAAGAAAGCTGGGTCTACAGGTTCACAATACCGTTTAACTTATG
2_known	C10	Tb927.6.2670_for	GGGGACAAGTTTGTACAAAAAAGCAGGCTATGCCAAACGAAACCGCGG	Tb927.6.2670_rev	GGGGACCACTTTGTACAAGAAAGCTGGGTTCACTCGCAGGAGGTGGG
2_known	C11	Tb927.5.1470_for	GGGGACAAGTTTGTACAAAAAAGCAGGCTATGGTGCGACTCGCCTTATCT	Tb927.5.1470_rev	GGGGACCACTTTGTACAAGAAAGCTGGGTTCAGTATTTAAACACCTGCGACTCC
2_known	C13	Tb11.02.5410_for	GGGGACAAGTTTGTACAAAAAAGCAGGCTATGAATGCGGACGAAAAAACGC	Tb11.02.5410_rev	GGGGACCACTTTGTACAAGAAAGCTGGGTCTACGGGTTGCGCAGGTG
2_known	C14	Tb927.1.1660_for	GGGGACAAGTTTGTACAAAAAAGCAGGCTATGCACAATAGTGTTCCAGCATCC	Tb927.1.1660_rev	GGGGACCACTTTGTACAAGAAAGCTGGGTTCAAAATATAAGAAAATCAGGCCGTCCA
2_known	C15	Tb927.8.1920_for	GGGGACAAGTTTGTACAAAAAAGCAGGCTATGCCTGGATTGCTAATATCCCGG	Tb927.8.1920_rev	GGGGACCACTTTGTACAAGAAAGCTGGGTTCATCTCCGTCGGTGAATGAAAAAA
2_known	C16	Tb927.10.4310_for	GGGGACAAGTTTGTACAAAAAAGCAGGCTATGTCCCGGGCACCTCCA	Tb927.10.4310_rev	GGGGACCACTTTGTACAAGAAAGCTGGGTCTAATGTCCAATAGTTTGGGTGTTCAGA
2_known	C17	Tb927.10.340_for	GGGGACAAGTTTGTACAAAAAAGCAGGCTATGTCGGTACGGGAACGAGC	Tb927.10.340_rev	GGGGACCACTTTGTACAAGAAAGCTGGGTTTATAGCTTCAGGAAGAATTCACACACC
2_known	C18	Tb927.6.1080_for	GGGGACAAGTTTGTACAAAAAAGCAGGCTATGGAAGTTGTAGTGAAGAGCATCG	Tb927.6.1080_rev	GGGGACCACTTTGTACAAGAAAGCTGGGTTTACGGACACGTATTATAGAGGAAGTCC
2_known	C19	Tb927.10.12990_for	GGGGACAAGTTTGTACAAAAAAGCAGGCTATGAGTGATCACCTGTCGGAAATATGC	Tb927.10.12990_rev	GGGGACCACTTTGTACAAGAAAGCTGGGTTTAAACCTCCCAGAATTCTTTTCCCTC
2_known	C20	Tb927.6.1640_for	GGGGACAAGTTTGTACAAAAAAGCAGGCTATGAAGAATCCATCACTTTCATCACTTTGC	Tb927.6.1640_rev	GGGGACCACTTTGTACAAGAAAGCTGGGTTTATTTCCCCTTCTTTCGGAGCAC
2_known	C21	Tb927.6.3120_for	GGGGACAAGTTTGTACAAAAAAGCAGGCTATGCAAAAAAATCAGACTGCATATTATCGT	Tb927.6.3120_rev	GGGGACCACTTTGTACAAGAAAGCTGGGTTTATGATCTTCTTGTTTTTGCGGTCAC
2_known	C22	Tb927.8.870_for	GGGGACAAGTTTGTACAAAAAAGCAGGCTATGAGAGGTACGAAACGCATTGG	Tb927.8.870_rev	GGGGACCACTTTGTACAAGAAAGCTGGGTCTAGACGGTTTGTACCGCATTTTGC
2_known	C23	Tb927.9.15510_for	GGGGACAAGTTTGTACAAAAAAGCAGGCTATGAGCATCACTTTTAATAATTTATGGCTA	Tb927.9.15510_rev	GGGGACCACTTTGTACAAGAAAGCTGGGTTTACAGTACAGAACCGAGAAACAATCC
2_known	C24	Tb927.9.14160_for	GGGGACAAGTTTGTACAAAAAAGCAGGCTATGTTCCGCAGGTCCTGCA	Tb927.9.14160_rev	GGGGACCACTTTGTACAAGAAAGCTGGGTCTATAGTTTCCCAAATATATCGTCTTATC
2_known	D1	Tb927.10.13970_for	GGGGACAAGTTTGTACAAAAAAGCAGGCTATGGTGCAACGGACACTGTTT	Tb927.10.13970_rev	GGGGACCACTTTGTACAAGAAAGCTGGGTCTACTTTCCCTTACTGAGGCATACGG
2_known	D2	Tb927.3.3970_for	GGGGACAAGTTTGTACAAAAAAGCAGGCTATGGTTTCCTTCACTTGTAGCGTC	Tb927.3.3970_rev	GGGGACCACTTTGTACAAGAAAGCTGGGTTTATCCCGCCTCTTCATCTCGC
2_known	D3	Tb927.8.3400_for	GGGGACAAGTTTGTACAAAAAAGCAGGCTATGCAACAGAACATTCACTCTTTCACA	Tb927.8.3400_rev	GGGGACCACTTTGTACAAGAAAGCTGGGTTCAAACAAGTGAAGCCGCCTC
2_known	D4	Tb927.9.7770_for	GGGGACAAGTTTGTACAAAAAAGCAGGCTATGCCTGGACCCGGTCTTC	Tb927.9.7770_rev	GGGGACCACTTTGTACAAGAAAGCTGGGTTCAAGAATAGTTTGGTTGAGATGGCG
2_known	D5	Tb927.7.1600_for	GGGGACAAGTTTGTACAAAAAAGCAGGCTATGCCTGAGGCGGTTGTAATTG	Tb927.7.1600_rev	GGGGACCACTTTGTACAAGAAAGCTGGGTTCACAAGGCACGAACGCTTC
2_known	D6	Tb927.3.1840_for	GGGGACAAGTTTGTACAAAAAAGCAGGCTATGAAGGTTACCGTGATATCAGGCT	Tb927.3.1840_rev	GGGGACCACTTTGTACAAGAAAGCTGGGTCTACAGAAGGAATGGTAACATGGCC
2_known	D7	Tb927.10.4680_for	GGGGACAAGTTTGTACAAAAAAGCAGGCTATGGCTATGGGAAGAGTTTCCGT	Tb927.10.4680_rev	GGGGACCACTTTGTACAAGAAAGCTGGGTCTAACGCTTTCCAAGTGGGTGC
2_known	D8	Tb927.3.2410_for	GGGGACAAGTTTGTACAAAAAAGCAGGCTATGCAACCGGCCACTGCA	Tb927.3.2410_rev	GGGGACCACTTTGTACAAGAAAGCTGGGTCTACGACGCACCGCTGTC
2_known	D9	Tb927.10.13310_for	GGGGACAAGTTTGTACAAAAAAGCAGGCTATGGATTCCGTCAAAAGTTTCAAGATGA	Tb927.10.13310_rev	GGGGACCACTTTGTACAAGAAAGCTGGGTCTAAATAACATGTCGATACATTTCGTACGT
2_known	D10	Tb927.10.2250_for	GGGGACAAGTTTGTACAAAAAAGCAGGCTATGTCGATAGATGTGGTTCCTGCT	Tb927.10.2250_rev	GGGGACCACTTTGTACAAGAAAGCTGGGTTCACAACCCTCCAAATTCCTCATTG
2_known	D11	Tb927.6.1280_for	GGGGACAAGTTTGTACAAAAAAGCAGGCTATGGAAGTGCCCCATGATGACT	Tb927.6.1280_rev	GGGGACCACTTTGTACAAGAAAGCTGGGTCTACTGGCATTTCCCCGGC
2_known	D12	Tb927.10.7500_for	GGGGACAAGTTTGTACAAAAAAGCAGGCTATGCGAGGTGGGTTTGGACG	Tb927.10.7500_rev	GGGGACCACTTTGTACAAGAAAGCTGGGTTTATTGTTGTACTGCTTTCTTCATAGAGCG
2_known	D13	Tb927.6.1570_for	GGGGACAAGTTTGTACAAAAAAGCAGGCTATGTCGCTGCGAGTTGGC	Tb927.6.1570_rev	GGGGACCACTTTGTACAAGAAAGCTGGGTTCATGGCTTGGTCTCCTTAATCTTGC
2_known	D14	Tb927.10.4460_for	GGGGACAAGTTTGTACAAAAAAGCAGGCTATGGCGCTTCAGCAAACGG	Tb927.10.4460_rev	GGGGACCACTTTGTACAAGAAAGCTGGGTTCACGTCCACAACGACGTG
2_known	D15	Tb927.1.4830_for	GGGGACAAGTTTGTACAAAAAAGCAGGCTATGTCGGAACCCGTTGATCAGT	Tb927.1.4830_rev	GGGGACCACTTTGTACAAGAAAGCTGGGTTCATGAAGAACCGAACCCTCCG
2_known	D16	Tb927.1.1980_for	GGGGACAAGTTTGTACAAAAAAGCAGGCTATGGACTCCTTTGGAAACCAGAAAC	Tb927.1.1980_rev	GGGGACCACTTTGTACAAGAAAGCTGGGTCTACTGAAAGGGCCGTATCGC
2_known	D17	Tb927.6.2740_for	GGGGACAAGTTTGTACAAAAAAGCAGGCTATGTCAGAGAAGACGGTTTTATCGATTC	Tb927.6.2740_rev	GGGGACCACTTTGTACAAGAAAGCTGGGTTCATGAAATTGGTTTAACGTCAACAACTG
2_known	D18	Tb927.10.16160_for	GGGGACAAGTTTGTACAAAAAAGCAGGCTATGAGCGACAGTCAAATGGACTG	Tb927.10.16160_rev	GGGGACCACTTTGTACAAGAAAGCTGGGTTTAACTTCTCAGACTACAATGCTCAGAAAC
2_known	D19	Tb927.3.2310_for	GGGGACAAGTTTGTACAAAAAAGCAGGCTATGAGTTACGAGATACAACCCATATTGAAG	Tb927.3.2310_rev	GGGGACCACTTTGTACAAGAAAGCTGGGTTCAACTGAAAATACAGCTCTCATACGTC
2_known	D20	Tb927.10.8150_for	GGGGACAAGTTTGTACAAAAAAGCAGGCTATGTCCGATACTACTAGAAGAGTGAAGC	Tb927.10.8150_rev	GGGGACCACTTTGTACAAGAAAGCTGGGTTCAGTTTTCCACCCTCGCCT
2_known	D21	Tb927.1.1580_for	GGGGACAAGTTTGTACAAAAAAGCAGGCTATGTCACTTCGGCGCCAC	Tb927.1.1580_rev	GGGGACCACTTTGTACAAGAAAGCTGGGTTCAACCGCGTGTGTCCTTAAATC
2_known	D22	Tb927.3.2230_for	GGGGACAAGTTTGTACAAAAAAGCAGGCTATGCTCTCAAGGACAGTTCGCC	Tb927.3.2230_rev	GGGGACCACTTTGTACAAGAAAGCTGGGTTTAGATTGCCCTCTTTTTCATTAACTCAGC
2_known	D23	Tb927.7.4930_for	GGGGACAAGTTTGTACAAAAAAGCAGGCTATGGGAAAGAAAAGGTTTTACGCGG	Tb927.7.4930_rev	GGGGACCACTTTGTACAAGAAAGCTGGGTTTAGCCAAAGGGCGATTCACAG
2_known	D24	Tb927.10.1070_for	GGGGACAAGTTTGTACAAAAAAGCAGGCTATGGGGAGTCGTTACGAGCG	Tb927.10.1070_rev	GGGGACCACTTTGTACAAGAAAGCTGGGTTTAGAACTCGACAGAAAAGTATGGGTG
2_known	E1	Tb927.7.4160_for	GGGGACAAGTTTGTACAAAAAAGCAGGCTATGTTGATGAACTTCGGGGGC	Tb927.7.4160_rev	GGGGACCACTTTGTACAAGAAAGCTGGGTTTACTCTTTTTTCTTTATTTCCGCTGCTAC
2_known	E2	Tb927.7.4670_for	GGGGACAAGTTTGTACAAAAAAGCAGGCTATGAGTGTCCCGACGTGCA	Tb927.7.4670_rev	GGGGACCACTTTGTACAAGAAAGCTGGGTTCAATAAATTGTACGCTTACGGGTGT
2_known	E3	Tb927.3.1540_for	GGGGACAAGTTTGTACAAAAAAGCAGGCTATGAGTGATGTCGGCATCTGC	Tb927.3.1540_rev	GGGGACCACTTTGTACAAGAAAGCTGGGTTTATGTTGACCTTCTAGCAACTGTTGAA
2_known	E4	Tb927.2.4870_for	GGGGACAAGTTTGTACAAAAAAGCAGGCTATGGTGAGACGGGGTGCA	Tb927.2.4870_rev	GGGGACCACTTTGTACAAGAAAGCTGGGTTCACGCAACACCCAAATCACG
2_known	E5	Tb927.10.4980_for	GGGGACAAGTTTGTACAAAAAAGCAGGCTATGAGCATCACCATAAAGTTGACCAA	Tb927.10.4980_rev	GGGGACCACTTTGTACAAGAAAGCTGGGTTTACCCATTTAAAAGCCTTTCGATCGC
2_known	E6	Tb927.10.12540_for	GGGGACAAGTTTGTACAAAAAAGCAGGCTATGAGGCGTTTTTCTAACTGCTTGC	Tb927.10.12540_rev	GGGGACCACTTTGTACAAGAAAGCTGGGTCTATTTTCCTAGCGTTACTGTTCCTCTATC
2_known	E7	Tb927.10.14280_for	GGGGACAAGTTTGTACAAAAAAGCAGGCTATGGACTCGTTTGTGGCAGGT	Tb927.10.14280_rev	GGGGACCACTTTGTACAAGAAAGCTGGGTTTATTTTTCATGGAAACGATAACAACTACA
2_known	E8	Tb927.11.16970_for	GGGGACAAGTTTGTACAAAAAAGCAGGCTATGGACACGATAGACCAGTTCCTG	Tb927.11.16970_rev	GGGGACCACTTTGTACAAGAAAGCTGGGTTTACAAGAAATAATCCGGCACAGTAGG
2_known	E9	Tb927.11.8090_for	GGGGACAAGTTTGTACAAAAAAGCAGGCTATGTCTGTAGATGTCGATGCCATTATC	Tb927.11.8090_rev	GGGGACCACTTTGTACAAGAAAGCTGGGTCTACTGAAAGTACTTGGGCTTCTTTACG
2_known	E10	Tb927.10.14750_for	GGGGACAAGTTTGTACAAAAAAGCAGGCTATGCGCGGTGGTAGAGGG	Tb927.10.14750_rev	GGGGACCACTTTGTACAAGAAAGCTGGGTCTATTGCGGGGGGGATTTGTAGT
2_known	E11	Tb927.10.12840_for	GGGGACAAGTTTGTACAAAAAAGCAGGCTATGTCGAAAGAGACAAAGGCGC	Tb927.10.12840_rev	GGGGACCACTTTGTACAAGAAAGCTGGGTCTACTGCTTTGCCTGAAGCTTGG
2_known	E12	Tb927.11.15810_for	GGGGACAAGTTTGTACAAAAAAGCAGGCTATGATGCGCCGTTGCTTTCC	Tb927.11.15810_rev	GGGGACCACTTTGTACAAGAAAGCTGGGTCTATCTCCTGGCAATATTACTACCTGCA
2_known	E13	Tb927.11.10340_for	GGGGACAAGTTTGTACAAAAAAGCAGGCTATGACGGTGTTGTCACGGGT	Tb927.11.10340_rev	GGGGACCACTTTGTACAAGAAAGCTGGGTTTACGGAACGTTATGAGTTGTGACC
2_known	E14	Tb927.9.7070_for	GGGGACAAGTTTGTACAAAAAAGCAGGCTATGAGTGAAACAAACCCAGTAGTGAG	Tb927.9.7070_rev	GGGGACCACTTTGTACAAGAAAGCTGGGTTTACTCCTTGGGAAAATAACCGCG
2_known	E15	Tb927.10.14160_for	GGGGACAAGTTTGTACAAAAAAGCAGGCTATGCAGAGCCAACCAGACAATG	Tb927.10.14160_rev	GGGGACCACTTTGTACAAGAAAGCTGGGTTTAGTGTGGCACAAAATATTTGTACAGAAA
2_known	E16	Tb927.9.13990_for	GGGGACAAGTTTGTACAAAAAAGCAGGCTATGCAAGGAGGAAATATGTTCGACG	Tb927.9.13990_rev	GGGGACCACTTTGTACAAGAAAGCTGGGTTCATGAAGTGGATTTATTGTCAAGACGG
2_known	E17	Tb927.9.13030_for	GGGGACAAGTTTGTACAAAAAAGCAGGCTATGGCGACCCGTGACCGT	Tb927.9.13030_rev	GGGGACCACTTTGTACAAGAAAGCTGGGTTTAAGACAGCATCTTTTGAAACAGAAGAAT
2_known	E18	Tb927.3.4820_for	GGGGACAAGTTTGTACAAAAAAGCAGGCTATGGCGATCGTCGTGACGA	Tb927.3.4820_rev	GGGGACCACTTTGTACAAGAAAGCTGGGTTTAGGTAGCTTTTTCCGTGGAGGC
2_known	E19	Tb927.10.3030_for	GGGGACAAGTTTGTACAAAAAAGCAGGCTATGTTCCATCCCCAATTGGTGGC	Tb927.10.3030_rev	GGGGACCACTTTGTACAAGAAAGCTGGGTTCAGAAGACAACCCCATTAATCATCG
2_known	E20	Tb927.10.180_for	GGGGACAAGTTTGTACAAAAAAGCAGGCTATGTCAGGTAAGCTTCGTCTTTACAAA	Tb927.10.180_rev	GGGGACCACTTTGTACAAGAAAGCTGGGTCTACTTGGTTACTGCCCCTTCCC
2_known	E22	Tb927.11.7360_for	GGGGACAAGTTTGTACAAAAAAGCAGGCTATGTCTAGTCTACCTTCAGATTCAGCG	Tb927.11.7360_rev	GGGGACCACTTTGTACAAGAAAGCTGGGTCTATACCATGTGAACATCGGATGCG
2_known	E23	Tb927.7.4180_for	GGGGACAAGTTTGTACAAAAAAGCAGGCTATGTTTTTTACTCCTCCACAACTTCAGA	Tb927.7.4180_rev	GGGGACCACTTTGTACAAGAAAGCTGGGTTTATTTAACTTTCTTCTCGACAGAACCGT
2_known	E24	Tb927.1.1060_for	GGGGACAAGTTTGTACAAAAAAGCAGGCTATGTCTGTTCAGTGCCAGGTTG	Tb927.1.1060_rev	GGGGACCACTTTGTACAAGAAAGCTGGGTTTACTGATCGTGCAGATTGTATATGGG
2_known	F1	Tb927.7.820_for	GGGGACAAGTTTGTACAAAAAAGCAGGCTATGGCAACCTTGAACGCACC	Tb927.7.820_rev	GGGGACCACTTTGTACAAGAAAGCTGGGTTCACATGCCTGGTGTCGGG
2_known	F2	Tb927.10.6690_for	GGGGACAAGTTTGTACAAAAAAGCAGGCTATGTCCACAGCGAAGAGTTTTCC	Tb927.10.6690_rev	GGGGACCACTTTGTACAAGAAAGCTGGGTCTACGCTTTTAAGTTAAGTGTCGACAC
2_known	F3	Tb927.11.3750_for	GGGGACAAGTTTGTACAAAAAAGCAGGCTATGATGCTCGCAGTTATCGTAGC	Tb927.11.3750_rev	GGGGACCACTTTGTACAAGAAAGCTGGGTTTAAAAGGTAAATATATTGTCTTGTGAGTA
2_known	F4	Tb927.10.7790_for	GGGGACAAGTTTGTACAAAAAAGCAGGCTATGTATCCTGGAAAGGAGAGCCC	Tb927.10.7790_rev	GGGGACCACTTTGTACAAGAAAGCTGGGTTCAGTTTTTTGCTGCGTTGAATCTG
2_known	F5	Tb927.11.11940_for	GGGGACAAGTTTGTACAAAAAAGCAGGCTATGAGCGACTTAAACGCCATGAT	Tb927.11.11940_rev	GGGGACCACTTTGTACAAGAAAGCTGGGTTTATAGGAAATACTCCGGTGGCGT
2_known	F6	Tb927.8.2780_for	GGGGACAAGTTTGTACAAAAAAGCAGGCTATGGGAGACTCGATATCACCTTCG	Tb927.8.2780_rev	GGGGACCACTTTGTACAAGAAAGCTGGGTTCACTCCATTCGAACCGGAGG
2_known	F7	Tb927.8.3140_for	GGGGACAAGTTTGTACAAAAAAGCAGGCTATGGCTGACCGCCTTGCT	Tb927.8.3140_rev	GGGGACCACTTTGTACAAGAAAGCTGGGTTTACTTCGTGCCGCCCAAC
2_known	F8	Tb927.7.2750_for	GGGGACAAGTTTGTACAAAAAAGCAGGCTATGGGAATAGTAGGAGAGTATGAAATATTA	Tb927.7.2750_rev	GGGGACCACTTTGTACAAGAAAGCTGGGTTTAGCTGTTTGCAACTTGTTTCTCCT
2_known	F9	Tb927.6.3730_for	GGGGACAAGTTTGTACAAAAAAGCAGGCTATGCGCTCGTCGTTTTTTTTGC	Tb927.6.3730_rev	GGGGACCACTTTGTACAAGAAAGCTGGGTCTAACCACCAGCCACAATGCTG
2_known	F10	Tb927.9.11040_for	GGGGACAAGTTTGTACAAAAAAGCAGGCTATGGATGTAATATGCTCCGCTCTTG	Tb927.9.11040_rev	GGGGACCACTTTGTACAAGAAAGCTGGGTTTACGCCATATTACCTTTAAGATAGCTGTA
2_known	F11	Tb927.3.2400_for	GGGGACAAGTTTGTACAAAAAAGCAGGCTATGCTCAGCTTTCTTTCCCCCA	Tb927.3.2400_rev	GGGGACCACTTTGTACAAGAAAGCTGGGTTCACACCTCCGCCTCTTCAA
2_known	F12	Tb927.10.14830_for	GGGGACAAGTTTGTACAAAAAAGCAGGCTATGACGGATAAAAAGCGGGAACC	Tb927.10.14830_rev	GGGGACCACTTTGTACAAGAAAGCTGGGTCTAATTCGATCTGCGCCACTCC
2_known	F13	Tb927.11.9990_for	GGGGACAAGTTTGTACAAAAAAGCAGGCTATGTCACTACCAAGAAGAATGTGCATG	Tb927.11.9990_rev	GGGGACCACTTTGTACAAGAAAGCTGGGTCTACGGTACAAGGGTTCCAGTCT
2_known	F14	Tb927.10.14680_for	GGGGACAAGTTTGTACAAAAAAGCAGGCTATGGTTAAGAGGGAGAAACGTCAGC	Tb927.10.14680_rev	GGGGACCACTTTGTACAAGAAAGCTGGGTTCAATCGCGGAATACTGAGTCCA
2_known	F15	Tb927.7.2730_for	GGGGACAAGTTTGTACAAAAAAGCAGGCTATGATAGAAGAAGTTGAATCTGTGTACAAG	Tb927.7.2730_rev	GGGGACCACTTTGTACAAGAAAGCTGGGTCTACGAAGCGTTCTTTTTCCTGGA
2_known	F16	Tb927.9.11280_for	GGGGACAAGTTTGTACAAAAAAGCAGGCTATGCTTCGGCGATCACTCTTAGC	Tb927.9.11280_rev	GGGGACCACTTTGTACAAGAAAGCTGGGTCTAAGGAGACTTCTTAAAGCTCTTGAAGTC
2_known	F17	Tb927.10.9300_for	GGGGACAAGTTTGTACAAAAAAGCAGGCTATGGAATCCACAGTCACCGGT	Tb927.10.9300_rev	GGGGACCACTTTGTACAAGAAAGCTGGGTTTAACAAATGTGTCGGCTCACCA
2_known	F20	Tb927.11.14460_for	GGGGACAAGTTTGTACAAAAAAGCAGGCTATGTCACATACTGCTGAGGATGCC	Tb927.11.14460_rev	GGGGACCACTTTGTACAAGAAAGCTGGGTTTAGTCTTTCTTTGATTGGAAGTCCCC
2_known	F21	Tb927.11.1350_for	GGGGACAAGTTTGTACAAAAAAGCAGGCTATGTGGAGGCCTTATACTTTGTTTGTT	Tb927.11.1350_rev	GGGGACCACTTTGTACAAGAAAGCTGGGTTCAGTGATGTTTAAACCACTTCCGC
2_known	F22	Tb927.8.3330_for	GGGGACAAGTTTGTACAAAAAAGCAGGCTATGTCTCGCACCATAAAGAAGGAAAA	Tb927.8.3330_rev	GGGGACCACTTTGTACAAGAAAGCTGGGTCTACATGCAAGTGGTGAAAAGCTCA
2_known	F23	Tb927.10.16170_for	GGGGACAAGTTTGTACAAAAAAGCAGGCTATGAGGTTCATCAACCAAGCAGC	Tb927.10.16170_rev	GGGGACCACTTTGTACAAGAAAGCTGGGTTCAGTCCACCGTCCAAATATTAACTG
2_known	F24	Tb927.10.3620_for	GGGGACAAGTTTGTACAAAAAAGCAGGCTATGATGTTTCACACATTCACTGCGC	Tb927.10.3620_rev	GGGGACCACTTTGTACAAGAAAGCTGGGTTTATGGGGCCGCGGCACT
2_known	G1	Tb927.7.2530_for	GGGGACAAGTTTGTACAAAAAAGCAGGCTATGAACAATAAAAATGTGTTAGTTTCTGGC	Tb927.7.2530_rev	GGGGACCACTTTGTACAAGAAAGCTGGGTTTACGTCTGCTCGTTTTCAGGAAC
2_known	G2	Tb927.9.15110_for	GGGGACAAGTTTGTACAAAAAAGCAGGCTATGACATTCGTTAAGGTTGTGAAGAACA	Tb927.9.15110_rev	GGGGACCACTTTGTACAAGAAAGCTGGGTTTACTTCGCACGGTCACGGA
2_known	G3	Tb927.7.2360_for	GGGGACAAGTTTGTACAAAAAAGCAGGCTATGCCAAAAAAAGGGAAAAAGAATGCG	Tb927.7.2360_rev	GGGGACCACTTTGTACAAGAAAGCTGGGTTCAGATAGGAGAAGTAGAGGTAGAAGGA
2_known	G4	Tb927.11.16980_for	GGGGACAAGTTTGTACAAAAAAGCAGGCTATGGGGTCAGATGTGTTTGAGCT	Tb927.11.16980_rev	GGGGACCACTTTGTACAAGAAAGCTGGGTCTATCTACTTCGGTCATAGTTTCCCAGG
2_known	G6	Tb927.11.15550_for	GGGGACAAGTTTGTACAAAAAAGCAGGCTATGAAATTCCTTACTTTTACTGCTGCCT	Tb927.11.15550_rev	GGGGACCACTTTGTACAAGAAAGCTGGGTTTAGAAACGATAGACGTGATCTTTTGTATA
2_known	G7	Tb927.11.5140_for	GGGGACAAGTTTGTACAAAAAAGCAGGCTATGCCCAGTTGGTGTCTTATTGAAA	Tb927.11.5140_rev	GGGGACCACTTTGTACAAGAAAGCTGGGTCTACTGCTTAGCCCCTTGACCA
2_known	G8	Tb927.5.1200_for	GGGGACAAGTTTGTACAAAAAAGCAGGCTATGGCAGTCATAGTAAAGACTGGTAAGT	Tb927.5.1200_rev	GGGGACCACTTTGTACAAGAAAGCTGGGTTTAGACTAACCGAATTAGCGGGCA
2_known	G9	Tb927.11.15820_for	GGGGACAAGTTTGTACAAAAAAGCAGGCTATGCGTCGCGTGGCTTCA	Tb927.11.15820_rev	GGGGACCACTTTGTACAAGAAAGCTGGGTTTACCAGAACTTCATCTCGTAGTAGTCTT
2_known	G10	Tb927.11.8220_for	GGGGACAAGTTTGTACAAAAAAGCAGGCTATGAGGTCAACTGAGGTCGGG	Tb927.11.8220_rev	GGGGACCACTTTGTACAAGAAAGCTGGGTTTAATTCTCTTTCCCTGCAGTTGGC
2_known	G11	Tb927.7.7040_for	GGGGACAAGTTTGTACAAAAAAGCAGGCTATGATGTACACGAGTCCCCACG	Tb927.7.7040_rev	GGGGACCACTTTGTACAAGAAAGCTGGGTTTACGGAGCGAATATGGGATATTTTGC
2_known	G12	Tb927.10.6080_for	GGGGACAAGTTTGTACAAAAAAGCAGGCTATGTTGGCAGATTTTGAAAGTGTTCTC	Tb927.10.6080_rev	GGGGACCACTTTGTACAAGAAAGCTGGGTCTATGCACTCTGAGAAGAATAACGGTC
2_known	G13	Tb927.4.4360_for	GGGGACAAGTTTGTACAAAAAAGCAGGCTATGGGTTGCTGTTCCTGCTG	Tb927.4.4360_rev	GGGGACCACTTTGTACAAGAAAGCTGGGTCTACTGGCAATGTGCATTAAGAAATTTTAA

2_known	G14	Tb927.7.6960_for	GGGGACAAGTTTGTACAAAAAAGCAGGCTATGAACGTACAGAAGGCCATTCAG	Tb927.7.6960_rev	GGGGACCACTTTGTACAAGAAAGCTGGGTTTATCCCCAAAGTGCTTTCTTCACG
2_known	G15	Tb927.10.11870_for	GGGGACAAGTTTGTACAAAAAAGCAGGCTATGCTGCGCCACACATCAC	Tb927.10.11870_rev	GGGGACCACTTTGTACAAGAAAGCTGGGTTTATTTCTGCAGTTGATGCGTCTGC
2_known	G16	Tb927.11.270_for	GGGGACAAGTTTGTACAAAAAAGCAGGCTATGCATGATGGAAACTTTCTGAAGGA	Tb927.11.270_rev	GGGGACCACTTTGTACAAGAAAGCTGGGTTTATCCATTCAAACCGCTGCACA
2_known	G17	Tb927.5.1550_for	GGGGACAAGTTTGTACAAAAAAGCAGGCTATGGCACTCCCGACATCGC	Tb927.5.1550_rev	GGGGACCACTTTGTACAAGAAAGCTGGGTTTATGCGGGAGCGCAAGAAAC
2_known	G18	Tb927.5.1210_for	GGGGACAAGTTTGTACAAAAAAGCAGGCTATGTTATTTGACTGCACTTTGCTGTC	Tb927.5.1210_rev	GGGGACCACTTTGTACAAGAAAGCTGGGTTTACTGTTTCGCCTTGCGCT
2_known	G19	Tb927.3.5630_for	GGGGACAAGTTTGTACAAAAAAGCAGGCTATGGCTGCAACACCTTACAGGT	Tb927.3.5630_rev	GGGGACCACTTTGTACAAGAAAGCTGGGTCTACGCACTACCTGCAATGTACG
2_known	G20	Tb927.10.4990_for	GGGGACAAGTTTGTACAAAAAAGCAGGCTATGACAATGCTTGGGGCGTT	Tb927.10.4990_rev	GGGGACCACTTTGTACAAGAAAGCTGGGTCTAAAACATGGCATCACTAAACCATGG
2_known	G21	Tb927.9.9940_for	GGGGACAAGTTTGTACAAAAAAGCAGGCTATGGCGTTCTCACGAAAGGAGC	Tb927.9.9940_rev	GGGGACCACTTTGTACAAGAAAGCTGGGTTCACATGAGGCATGACTCATACGT
2_known	G22	Tb927.11.7370_for	GGGGACAAGTTTGTACAAAAAAGCAGGCTATGCCCCAATGTGGCACG	Tb927.11.7370_rev	GGGGACCACTTTGTACAAGAAAGCTGGGTCTAGCTCAGCAGAAACACTTCACG
2_known	G23	Tb927.2.2970_for	GGGGACAAGTTTGTACAAAAAAGCAGGCTATGTCATCCGAACACGCACC	Tb927.2.2970_rev	GGGGACCACTTTGTACAAGAAAGCTGGGTTTAATGCCGAACCACCCCTCT
2_known	G24	Tb927.7.3500_for	GGGGACAAGTTTGTACAAAAAAGCAGGCTATGTTCACAAAGGGGTCCATGTC	Tb927.7.3500_rev	GGGGACCACTTTGTACAAGAAAGCTGGGTTCACTTTGGGATGTATTTCTCATTACCC
2_known	H1	Tb927.10.14170_for	GGGGACAAGTTTGTACAAAAAAGCAGGCTATGCAGAGCCAACCAGACAATG	Tb927.10.14170_rev	GGGGACCACTTTGTACAAGAAAGCTGGGTTTAGTGTGGAAGAAAATATTTGTACAGAAA
2_known	H2	Tb927.10.1690_for	GGGGACAAGTTTGTACAAAAAAGCAGGCTATGAACTTGGCAAATTCTCATTGTATCG	Tb927.10.1690_rev	GGGGACCACTTTGTACAAGAAAGCTGGGTCTACTCCATCGCTAAACTTTCAATGAGT
2_known	H3	Tb927.11.5360_for	GGGGACAAGTTTGTACAAAAAAGCAGGCTATGGACCGTATTCCATATTTGGTTTCAT	Tb927.11.5360_rev	GGGGACCACTTTGTACAAGAAAGCTGGGTTTAATCTATCGTGTTGCTATGCCAACA
2_known	H4	Tb927.5.2380_for	GGGGACAAGTTTGTACAAAAAAGCAGGCTATGTTGACTACGACTGACACTATTGTTAAG	Tb927.5.2380_rev	GGGGACCACTTTGTACAAGAAAGCTGGGTTTAAGCCAGAGTCAGTTCCCGT
2_known	H5	Tb927.10.4500_for	GGGGACAAGTTTGTACAAAAAAGCAGGCTATGGAGAGCCTACGGACTGC	Tb927.10.4500_rev	GGGGACCACTTTGTACAAGAAAGCTGGGTTCAGCTGGCTTTCCGGAAGC
2_known	H6	Tb927.8.1810_for	GGGGACAAGTTTGTACAAAAAAGCAGGCTATGGCTGGTGAGGGTGCA	Tb927.8.1810_rev	GGGGACCACTTTGTACAAGAAAGCTGGGTTCACACTACAGCAGCATCGTGC
2_known	H7	Tb927.11.10020_for	GGGGACAAGTTTGTACAAAAAAGCAGGCTATGCAACTCTTATCGTGGTCTAGCG	Tb927.11.10020_rev	GGGGACCACTTTGTACAAGAAAGCTGGGTTCATTTCTGGAGAGAGCGATGACT
2_known	H8	Tb927.11.7140_for	GGGGACAAGTTTGTACAAAAAAGCAGGCTATGGCCGCAACAATTCTCAAGG	Tb927.11.7140_rev	GGGGACCACTTTGTACAAGAAAGCTGGGTCTACGAAGGTGTTTCTGGTTCCATC
2_known	H9	Tb927.5.3120_for	GGGGACAAGTTTGTACAAAAAAGCAGGCTATGGCTGAACAACCGGAAGTTT	Tb927.5.3120_rev	GGGGACCACTTTGTACAAGAAAGCTGGGTCTAAAGTGTCATGGCTTGGCGT
2_known	H10	Tb927.5.3830_for	GGGGACAAGTTTGTACAAAAAAGCAGGCTATGAGTTTGAAAGTTAATATTCTCGGACAT	Tb927.5.3830_rev	GGGGACCACTTTGTACAAGAAAGCTGGGTTCAATCCATCGTCTTCACGCG
2_known	H11	Tb927.4.2110_for	GGGGACAAGTTTGTACAAAAAAGCAGGCTATGCATCAACACGGCAAGACA	Tb927.4.2110_rev	GGGGACCACTTTGTACAAGAAAGCTGGGTCTACATATTGTGTAGAGGGTCGCCA
2_known	H12	Tb927.8.8020_for	GGGGACAAGTTTGTACAAAAAAGCAGGCTATGGGATGTTTTTGCTGCTGCT	Tb927.8.8020_rev	GGGGACCACTTTGTACAAGAAAGCTGGGTCTATTTACAGTGTGCATTAATAAACTTTAA
2_known	H13	Tb927.10.6860_for	GGGGACAAGTTTGTACAAAAAAGCAGGCTATGGGTGTGGGAGAAATGAGTGA	Tb927.10.6860_rev	GGGGACCACTTTGTACAAGAAAGCTGGGTTCAGCATTCATTTAGTTGAATCCGTCG
2_known	H14	Tb927.4.410_for	GGGGACAAGTTTGTACAAAAAAGCAGGCTATGCACCAAACCCAAGCGTATT	Tb927.4.410_rev	GGGGACCACTTTGTACAAGAAAGCTGGGTTCACGCACTGGCATCACCG
2_known	H15	Tb927.11.13440_for	GGGGACAAGTTTGTACAAAAAAGCAGGCTATGATTGATTGGGCACTCACGTT	Tb927.11.13440_rev	GGGGACCACTTTGTACAAGAAAGCTGGGTCTACCTCCATTCCTTCTGCCACC
2_known	H16	Tb927.6.1650_for	GGGGACAAGTTTGTACAAAAAAGCAGGCTATGAAGAATCCATCACTTTCATCACTTTGC	Tb927.6.1650_rev	GGGGACCACTTTGTACAAGAAAGCTGGGTTCACTGGTCTTCAGCAAACAGATG
2_known	H17	Tb927.10.12760_for	GGGGACAAGTTTGTACAAAAAAGCAGGCTATGTCATCGCAGGACAATCGC	Tb927.10.12760_rev	GGGGACCACTTTGTACAAGAAAGCTGGGTTTATGACCTGCAGAGAACAGGATAGT
2_known	H18	Tb927.9.10310_for	GGGGACAAGTTTGTACAAAAAAGCAGGCTATGAGCGCAAAGAACAAAACTTGG	Tb927.9.10310_rev	GGGGACCACTTTGTACAAGAAAGCTGGGTTTACTTCTTTCCGGAACCACCACT
2_known	H19	Tb927.9.2580_for	GGGGACAAGTTTGTACAAAAAAGCAGGCTATGGATCAGGACCTCTCTGTTTGT	Tb927.9.2580_rev	GGGGACCACTTTGTACAAGAAAGCTGGGTCTAGTGTTGGATTCTCTGATTGTTAAATTG
2_known	H20	Tb927.11.11360_for	GGGGACAAGTTTGTACAAAAAAGCAGGCTATGGCTGTCGCTTACGAAGGA	Tb927.11.11360_rev	GGGGACCACTTTGTACAAGAAAGCTGGGTTCACGCGTTCTCCGATACACC
2_known	H21	Tb927.10.2560_for	GGGGACAAGTTTGTACAAAAAAGCAGGCTATGCGTACCAGTGCAACTCTCC	Tb927.10.2560_rev	GGGGACCACTTTGTACAAGAAAGCTGGGTTCACTGTCTGGAACCGAAGGC
2_known	H23	Tb927.9.3440_for	GGGGACAAGTTTGTACAAAAAAGCAGGCTATGGCAATCGTCGGGGACT	Tb927.9.3440_rev	GGGGACCACTTTGTACAAGAAAGCTGGGTTCACTCTGTAAGGTCAATCACACCG
2_known	H24	Tb927.11.8790_for	GGGGACAAGTTTGTACAAAAAAGCAGGCTATGGACCGCTTGCTTCAGTTC	Tb927.11.8790_rev	GGGGACCACTTTGTACAAGAAAGCTGGGTTTACAATAACGACCCTAGCAAACCAG
2_known	I1	Tb927.9.3880_for	GGGGACAAGTTTGTACAAAAAAGCAGGCTATGGCTCCAGTAGTGTGGCC	Tb927.9.3880_rev	GGGGACCACTTTGTACAAGAAAGCTGGGTTCAAAACAGGTTGTGAACGCGG
2_known	I2	Tb927.8.990_for	GGGGACAAGTTTGTACAAAAAAGCAGGCTATGAGCACGGGTGATCCAACT	Tb927.8.990_rev	GGGGACCACTTTGTACAAGAAAGCTGGGTCTATGAATTGTGACGCATCACGGA
2_known	I3	Tb927.2.6210_for	GGGGACAAGTTTGTACAAAAAAGCAGGCTATGACACGCGCCTCGCTT	Tb927.2.6210_rev	GGGGACCACTTTGTACAAGAAAGCTGGGTCTACATTTTTGAGTAATCTTTCCCATATGC
2_known	I4	Tb927.2.6180_for	GGGGACAAGTTTGTACAAAAAAGCAGGCTATGACACGCGCCTCGCTT	Tb927.2.6180_rev	GGGGACCACTTTGTACAAGAAAGCTGGGTCTACATTTTAGGTTTTGCAAGCGAAAAC
2_known	I6	Tb927.10.13860_for	GGGGACAAGTTTGTACAAAAAAGCAGGCTATGTTGCCCATGTTACTGTGGC	Tb927.10.13860_rev	GGGGACCACTTTGTACAAGAAAGCTGGGTCTAGAACAAATCGTAACGTAACTCTACTGG
2_known	I7	Tb927.6.2540_for	GGGGACAAGTTTGTACAAAAAAGCAGGCTATGATTCCGTCACAACTGAAGAGC	Tb927.6.2540_rev	GGGGACCACTTTGTACAAGAAAGCTGGGTTCAAGTATGGATAGTACTACCATCTGTATC
2_known	I9	Tb927.2.5050_for	GGGGACAAGTTTGTACAAAAAAGCAGGCTATGGACTTATTTGGAGCCTATGCTTC	Tb927.2.5050_rev	GGGGACCACTTTGTACAAGAAAGCTGGGTTCATAATTTTACATTACTCGAATGATCGTC
2_known	I11	Tb927.11.13990_for	GGGGACAAGTTTGTACAAAAAAGCAGGCTATGTTAGGGTCCCTACCGAGTGT	Tb927.11.13990_rev	GGGGACCACTTTGTACAAGAAAGCTGGGTTCACTTCCGTGCGACATCGT
2_known	I12	Tb927.11.15130_for	GGGGACAAGTTTGTACAAAAAAGCAGGCTATGGCCGATCTATACGATGTTCTCG	Tb927.11.15130_rev	GGGGACCACTTTGTACAAGAAAGCTGGGTTTACTTGGCATCTAGAATACGGCTCA
2_known	I13	Tb927.5.300b_for	GGGGACAAGTTTGTACAAAAAAGCAGGCTATGGCTCACGGCTCGATTCC	Tb927.5.300b_rev	GGGGACCACTTTGTACAAGAAAGCTGGGTTCACATCTTTGTTTTGCGATGGGC
2_known	I14	Tb927.10.10830_for	GGGGACAAGTTTGTACAAAAAAGCAGGCTATGAAGCGCACACCTGTTAGG	Tb927.10.10830_rev	GGGGACCACTTTGTACAAGAAAGCTGGGTTTACACCTTCTGACTGGCATCACC
2_known	I15	Tb927.7.7230_for	GGGGACAAGTTTGTACAAAAAAGCAGGCTATGAACGTGAGCCGCTGC	Tb927.7.7230_rev	GGGGACCACTTTGTACAAGAAAGCTGGGTTCAGAAGCGATTCCATCGGACG
2_known	I16	Tb927.4.2350_for	GGGGACAAGTTTGTACAAAAAAGCAGGCTATGGCAAAAGCCCTTAGTGTCAG	Tb927.4.2350_rev	GGGGACCACTTTGTACAAGAAAGCTGGGTTTACGGTTTTCCGTCTCCACTGG
2_known	I18	Tb927.7.7500_for	GGGGACAAGTTTGTACAAAAAAGCAGGCTATGAATCACACTTCGATTCCAGTTATTGA	Tb927.7.7500_rev	GGGGACCACTTTGTACAAGAAAGCTGGGTTCACATATTTGCCTTGCGATGGG
2_known	I19	Tb927.10.6850_for	GGGGACAAGTTTGTACAAAAAAGCAGGCTATGAATCGTACGGGTGGGTCG	Tb927.10.6850_rev	GGGGACCACTTTGTACAAGAAAGCTGGGTTTACACCTTCTTGATACCCAGTGTCG
2_known	I20	Tb927.6.1520_for	GGGGACAAGTTTGTACAAAAAAGCAGGCTATGTCTGACGAAAAAATTAACGTGCAT	Tb927.6.1520_rev	GGGGACCACTTTGTACAAGAAAGCTGGGTTTAGTAGGGAACAAAGTATTTGTAGAGGAA
2_known	I21	Tb927.9.10570_for	GGGGACAAGTTTGTACAAAAAAGCAGGCTATGGCCCTTCTCGAGCATGT	Tb927.9.10570_rev	GGGGACCACTTTGTACAAGAAAGCTGGGTTTATTTCTGAGCGGCACCCTCT
2_known	I22	Tb927.9.7740_for	GGGGACAAGTTTGTACAAAAAAGCAGGCTATGACACTTCCGGGGCAACC	Tb927.9.7740_rev	GGGGACCACTTTGTACAAGAAAGCTGGGTTCACTTCGCACCTCGCCG
2_known	I23	Tb927.3.2890_for	GGGGACAAGTTTGTACAAAAAAGCAGGCTATGTCGAAGCAAAAGAATGTGGAGA	Tb927.3.2890_rev	GGGGACCACTTTGTACAAGAAAGCTGGGTCTAATCGGTCCACGTCATGCC
2_known	I24	Tb927.9.14180_for	GGGGACAAGTTTGTACAAAAAAGCAGGCTATGGATGACACTCGCATCAATTGG	Tb927.9.14180_rev	GGGGACCACTTTGTACAAGAAAGCTGGGTTCAATCAGTTAAAACACAAAGTGGAGAGG
2_known	J1	Tb927.11.7420_for	GGGGACAAGTTTGTACAAAAAAGCAGGCTATGGGAGAGGAGGTAGTTAGGTTAGA	Tb927.11.7420_rev	GGGGACCACTTTGTACAAGAAAGCTGGGTTCATGTGCTTAGCCTTCCACATC
2_known	J2	Tb927.11.1910_for	GGGGACAAGTTTGTACAAAAAAGCAGGCTATGCTTCCCGGGTTTCCCT	Tb927.11.1910_rev	GGGGACCACTTTGTACAAGAAAGCTGGGTTTATTCTACATGAACAATAATTCCGGTATT
2_known	J3	Tb927.9.9400_for	GGGGACAAGTTTGTACAAAAAAGCAGGCTATGGCTGTCCCACCAGTGG	Tb927.9.9400_rev	GGGGACCACTTTGTACAAGAAAGCTGGGTCTATGACTTTCCCTCGAAATTCATCATTTC
2_known	J4	Tb927.10.14690_for	GGGGACAAGTTTGTACAAAAAAGCAGGCTATGCCTAATTACTTACCCAACAGGGA	Tb927.10.14690_rev	GGGGACCACTTTGTACAAGAAAGCTGGGTTCATATGACGTCAAACTGCATGAGAA
2_known	J5	Tb927.3.1240_for	GGGGACAAGTTTGTACAAAAAAGCAGGCTATGCCTGCGACTACCTCAATTCC	Tb927.3.1240_rev	GGGGACCACTTTGTACAAGAAAGCTGGGTTCACAAAAAGTAGTCAGGTGTCTTTTTTG
2_known	J6	Tb927.10.15710_for	GGGGACAAGTTTGTACAAAAAAGCAGGCTATGTCTTCGGCGGCGGAA	Tb927.10.15710_rev	GGGGACCACTTTGTACAAGAAAGCTGGGTTCACGGCTGCGCGTCGCT
2_known	J7	Tb927.4.460_for	GGGGACAAGTTTGTACAAAAAAGCAGGCTATGGAAGACCCCGTGCGC	Tb927.4.460_rev	GGGGACCACTTTGTACAAGAAAGCTGGGTTCATTCGTTAAGGAACTCACACCTG
2_known	J8	Tb927.10.15410_for	GGGGACAAGTTTGTACAAAAAAGCAGGCTATGGTCAGCGTTGCCGTAATT	Tb927.10.15410_rev	GGGGACCACTTTGTACAAGAAAGCTGGGTTTACAACTTTGAGCGCGCGA
2_known	J9	Tb927.11.2060_for	GGGGACAAGTTTGTACAAAAAAGCAGGCTATGCCATCCGTTTCCCAGGA	Tb927.11.2060_rev	GGGGACCACTTTGTACAAGAAAGCTGGGTTTAGAAGAGAGCACCCATGCCA
2_known	J10	Tb927.10.12940_for	GGGGACAAGTTTGTACAAAAAAGCAGGCTATGGGGGGGAGCTCGTCG	Tb927.10.12940_rev	GGGGACCACTTTGTACAAGAAAGCTGGGTTCATACCTGGGAGCTGAGAGGC
2_known	J11	Tb927.11.16400_for	GGGGACAAGTTTGTACAAAAAAGCAGGCTATGTTGTTCCGCACCGTCAA	Tb927.11.16400_rev	GGGGACCACTTTGTACAAGAAAGCTGGGTCTAACGCCTCCTTACGGGTGG
2_known	J12	Tb927.11.14900_for	GGGGACAAGTTTGTACAAAAAAGCAGGCTATGGCGGATCCATTTTACGATGC	Tb927.11.14900_rev	GGGGACCACTTTGTACAAGAAAGCTGGGTCTATGCAGATGCCGTAAATTCTTCAATAG
2_known	J13	Tb927.11.7540_for	GGGGACAAGTTTGTACAAAAAAGCAGGCTATGTTTCGTTACACCGCTGCA	Tb927.11.7540_rev	GGGGACCACTTTGTACAAGAAAGCTGGGTTCACTTCCTTGACTTCGTAACCAGT
2_known	J14	Tb927.11.2050_for	GGGGACAAGTTTGTACAAAAAAGCAGGCTATGCCATCCGTTTCCCAGGA	Tb927.11.2050_rev	GGGGACCACTTTGTACAAGAAAGCTGGGTCTAGAAGAGAGCACCCATGCCA
2_known	J15	Tb927.10.14360_for	GGGGACAAGTTTGTACAAAAAAGCAGGCTATGAGGCTGACTCTTGACACCA	Tb927.10.14360_rev	GGGGACCACTTTGTACAAGAAAGCTGGGTTTATGATGTGCGAGTCTTCTTTGTCC
2_known	J16	Tb927.11.1180_for	GGGGACAAGTTTGTACAAAAAAGCAGGCTATGTCTGATACATTAAGTAGCAAACTAGAC	Tb927.11.1180_rev	GGGGACCACTTTGTACAAGAAAGCTGGGTCTAGTCACCTTGCAGAAAAGGATGT
2_known	J17	Tb927.6.4470_for	GGGGACAAGTTTGTACAAAAAAGCAGGCTATGTCGGTCACGCGGATTAAC	Tb927.6.4470_rev	GGGGACCACTTTGTACAAGAAAGCTGGGTTCAGGCACTGCGTGCGTA
2_known	J18	Tb927.11.1310_for	GGGGACAAGTTTGTACAAAAAAGCAGGCTATGCGCTTTGTTCCACCTTTCC	Tb927.11.1310_rev	GGGGACCACTTTGTACAAGAAAGCTGGGTTTAGAAGCGATAAACTTCATCATTGGAGT
2_known	J19	Tb927.11.11150_for	GGGGACAAGTTTGTACAAAAAAGCAGGCTATGGAAAGGATGTCTCTCGCGG	Tb927.11.11150_rev	GGGGACCACTTTGTACAAGAAAGCTGGGTTTAAATTCGCTGTAATATTACCTCGGTCTC
2_known	J20	Tb927.9.15590_for	GGGGACAAGTTTGTACAAAAAAGCAGGCTATGAGCATCACTTTTCATAATTTATGGCTA	Tb927.9.15590_rev	GGGGACCACTTTGTACAAGAAAGCTGGGTCATAGAAGTGTATAGAAAGTGAATATGGT
2_known	J21	Tb927.4.3560_for	GGGGACAAGTTTGTACAAAAAAGCAGGCTATGTCGACTATTGTGCGGAAACG	Tb927.4.3560_rev	GGGGACCACTTTGTACAAGAAAGCTGGGTTTAATCGTATTGTGGTCTAAATATCTCAAA
2_known	J22	Tb927.10.5070_for	GGGGACAAGTTTGTACAAAAAAGCAGGCTATGGCGGAACCTCGGTCTT	Tb927.10.5070_rev	GGGGACCACTTTGTACAAGAAAGCTGGGTCTAAGGGACTTCCCGCGACA
2_known	J23	Tb927.3.3020_for	GGGGACAAGTTTGTACAAAAAAGCAGGCTATGGACGCTACATGTGTAATTGATTATGG	Tb927.3.3020_rev	GGGGACCACTTTGTACAAGAAAGCTGGGTTCAAATAATCTTGGGCCGTACGC
2_known	J24	Tb927.8.7450_for	GGGGACAAGTTTGTACAAAAAAGCAGGCTATGCAGCCCGCGGGGGGG	Tb927.8.7450_rev	GGGGACCACTTTGTACAAGAAAGCTGGGTTCAGTAAGTGACGCGGGACA
2_known	K1	Tb927.10.12240_for	GGGGACAAGTTTGTACAAAAAAGCAGGCTATGTTCTACATTGACGATTTATTTTGGACA	Tb927.10.12240_rev	GGGGACCACTTTGTACAAGAAAGCTGGGTTCAGGACTGCTTCTTCCCGC
2_known	K2	Tb927.9.4310_for	GGGGACAAGTTTGTACAAAAAAGCAGGCTATGCTTCCATGTCCGTCGTTC	Tb927.9.4310_rev	GGGGACCACTTTGTACAAGAAAGCTGGGTCTACAAACCCTTGTAATACGTGAATATTTC
2_known	K3	Tb927.3.2960_for	GGGGACAAGTTTGTACAAAAAAGCAGGCTATGGCGAAAACAGTGATCCTCG	Tb927.3.2960_rev	GGGGACCACTTTGTACAAGAAAGCTGGGTTCAGCAAACGCGTGTGGAA
2_known	K4	Tb927.5.3820_for	GGGGACAAGTTTGTACAAAAAAGCAGGCTATGGCGGAGCTGCAACCT	Tb927.5.3820_rev	GGGGACCACTTTGTACAAGAAAGCTGGGTTTAAGCGAGAACACTGTAAAGTATGGC
2_known	K5	Tb927.11.8120_for	GGGGACAAGTTTGTACAAAAAAGCAGGCTATGTCTAATGTTGCTTTTGACTCGGG	Tb927.11.8120_rev	GGGGACCACTTTGTACAAGAAAGCTGGGTTTAAGGTAGCTTACACGAACGTTCC
2_known	K6	Tb927.9.14600_for	GGGGACAAGTTTGTACAAAAAAGCAGGCTATGTATTCTGCTACGCCGTTCATG	Tb927.9.14600_rev	GGGGACCACTTTGTACAAGAAAGCTGGGTTTACATTTTCGATTTGTAAGAATAGAGATC
2_known	K7	Tb927.4.4970_for	GGGGACAAGTTTGTACAAAAAAGCAGGCTATGCAGTCAGCAGGAGGAGATT	Tb927.4.4970_rev	GGGGACCACTTTGTACAAGAAAGCTGGGTTCAGTAAGTGACACGGGACAGC
2_known	K8	Tb927.9.8740_for	GGGGACAAGTTTGTACAAAAAAGCAGGCTATGTACAGCCAACCTTTTACTCAAAACA	Tb927.9.8740_rev	GGGGACCACTTTGTACAAGAAAGCTGGGTTTAACTCCACTGCTGTGTAATGCC
2_known	K9	Tb927.6.1690_for	GGGGACAAGTTTGTACAAAAAAGCAGGCTATGGAGCGTCTGAAAAATTTCGTACA	Tb927.6.1690_rev	GGGGACCACTTTGTACAAGAAAGCTGGGTTCATGGTTCAGTTGGCCACTCC
2_known	K10	Tb927.10.14200_for	GGGGACAAGTTTGTACAAAAAAGCAGGCTATGGTTGTAGAGCGCGACCG	Tb927.10.14200_rev	GGGGACCACTTTGTACAAGAAAGCTGGGTCTAGCGCACAACGAGAAACCC
2_known	K11	Tb927.7.2070_for	GGGGACAAGTTTGTACAAAAAAGCAGGCTATGTTTGCCTTTTCGGACCATATGG	Tb927.7.2070_rev	GGGGACCACTTTGTACAAGAAAGCTGGGTTCACTGCGCAGTACACCCA
2_known	K12	Tb927.11.11250_for	GGGGACAAGTTTGTACAAAAAAGCAGGCTATGTCTAACACATGTAAGCGTGTGG	Tb927.11.11250_rev	GGGGACCACTTTGTACAAGAAAGCTGGGTCTAGGCTGTGGAGACCTCTCTAC
2_known	K13	Tb927.8.900_for	GGGGACAAGTTTGTACAAAAAAGCAGGCTATGGATTCCAGAGACGGGAGTG	Tb927.8.900_rev	GGGGACCACTTTGTACAAGAAAGCTGGGTCTAATCTCGGCGTTTGCGGT
2_known	K15	Tb927.9.9390_for	GGGGACAAGTTTGTACAAAAAAGCAGGCTATGGCTGTCCCACCAGTGG	Tb927.9.9390_rev	GGGGACCACTTTGTACAAGAAAGCTGGGTTCAATGATTTCTCTCATCTACGACTTTCC
2_known	K16	Tb927.3.5150_for	GGGGACAAGTTTGTACAAAAAAGCAGGCTATGGTTTCTTGCAAGGCAATACCG	Tb927.3.5150_rev	GGGGACCACTTTGTACAAGAAAGCTGGGTTTACTCGCGAAGGTGTCGTTG
2_known	K17	Tb927.2.2160_for	GGGGACAAGTTTGTACAAAAAAGCAGGCTATGACATCGGAGGAAGCCGT	Tb927.2.2160_rev	GGGGACCACTTTGTACAAGAAAGCTGGGTTTAAATGTATTGCTCCAAATGCTCATACAA
2_known	K18	Tb927.10.7090_for	GGGGACAAGTTTGTACAAAAAAGCAGGCTATGTTTCGTAACCACGCATCGA	Tb927.10.7090_rev	GGGGACCACTTTGTACAAGAAAGCTGGGTTTACACGTGTTTGTTTACATTACTCGC
2_known	K19	Tb927.9.8260_for	GGGGACAAGTTTGTACAAAAAAGCAGGCTATGTTACGGTACTCGCCGATGC	Tb927.9.8260_rev	GGGGACCACTTTGTACAAGAAAGCTGGGTTTACAATGCTGCTGTAAGTTTGTCAAG
2_known	K20	Tb927.4.4070_for	GGGGACAAGTTTGTACAAAAAAGCAGGCTATGCACGTGGCTGTTAAGGAC	Tb927.4.4070_rev	GGGGACCACTTTGTACAAGAAAGCTGGGTCTATAGCTTACTTCCGCCGGGC
2_known	K21	Tb927.5.790_for	GGGGACAAGTTTGTACAAAAAAGCAGGCTATGGGTGGCGACGGGCGC	Tb927.5.790_rev	GGGGACCACTTTGTACAAGAAAGCTGGGTTCATTCGGTGTCATCACTCTCCT
2_known	K22	Tb927.9.12120_for	GGGGACAAGTTTGTACAAAAAAGCAGGCTATGGATGCAACACTTCACGATAAAAGC	Tb927.9.12120_rev	GGGGACCACTTTGTACAAGAAAGCTGGGTTCAGTCTGCCACCAAGGGAA
2_known	K23	Tb927.6.850_for	GGGGACAAGTTTGTACAAAAAAGCAGGCTATGAACAATAGCAACCAGGGAACAC	Tb927.6.850_rev	GGGGACCACTTTGTACAAGAAAGCTGGGTTCAGTCCCCTCCCACCACA
2_known	K24	Tb927.10.13400_for	GGGGACAAGTTTGTACAAAAAAGCAGGCTATGACCACCGAAAGCGGGA	Tb927.10.13400_rev	GGGGACCACTTTGTACAAGAAAGCTGGGTTCATTTAGTTATATCAGACAACACCTGCC
2_known	L1	Tb927.8.2020_for	GGGGACAAGTTTGTACAAAAAAGCAGGCTATGTTCTCCAAGTTCCTGATGAACG	Tb927.8.2020_rev	GGGGACCACTTTGTACAAGAAAGCTGGGTTCAATGATCGGGGTACGGATTTTC
2_known	L2	Tb927.10.6880_for	GGGGACAAGTTTGTACAAAAAAGCAGGCTATGGTAATCAGAGTCGGCATCAATG	Tb927.10.6880_rev	GGGGACCACTTTGTACAAGAAAGCTGGGTTCACTTTGTGATGTGCGCAATGA
2_known	L3	Tb927.8.570_for	GGGGACAAGTTTGTACAAAAAAGCAGGCTATGCCTGAGGCTTGCTTTCTC	Tb927.8.570_rev	GGGGACCACTTTGTACAAGAAAGCTGGGTTTAGTCCTCCTTCTCTTCGTTCTGATC
2_known	L4	Tb927.11.1560_for	GGGGACAAGTTTGTACAAAAAAGCAGGCTATGCCGAGCAGTTGGTTATTGAA	Tb927.11.1560_rev	GGGGACCACTTTGTACAAGAAAGCTGGGTCTACAATTTCTCCACCCACTTCTCATAAC
2_known	L5	Tb927.10.10140_for	GGGGACAAGTTTGTACAAAAAAGCAGGCTATGTACCGGTCCCGGCCC	Tb927.10.10140_rev	GGGGACCACTTTGTACAAGAAAGCTGGGTTTACGAAGCTTTCACTGAGGAAGC
2_known	L6	Tb927.11.1050_for	GGGGACAAGTTTGTACAAAAAAGCAGGCTATGGGAAAAGGTGCGCGC	Tb927.11.1050_rev	GGGGACCACTTTGTACAAGAAAGCTGGGTTTAGAGGTACCACTCCAGCTTGTC
2_known	L7	Tb927.7.3940_for	GGGGACAAGTTTGTACAAAAAAGCAGGCTATGGATCACGATCAACTATACGACTCT	Tb927.7.3940_rev	GGGGACCACTTTGTACAAGAAAGCTGGGTCTAAACGGCCCAGAAATGGCG
2_known	L8	Tb927.4.690_for	GGGGACAAGTTTGTACAAAAAAGCAGGCTATGTTTGGAGCGCCAAACACA	Tb927.4.690_rev	GGGGACCACTTTGTACAAGAAAGCTGGGTTCACGCACAGGTACGCTTCC
2_known	L9	Tb927.3.2340_for	GGGGACAAGTTTGTACAAAAAAGCAGGCTATGGCTTGGATAACTGATGCTGC	Tb927.3.2340_rev	GGGGACCACTTTGTACAAGAAAGCTGGGTTCACTGTGCCCATGGGGTG
2_known	L10	Tb927.11.16070_for	GGGGACAAGTTTGTACAAAAAAGCAGGCTATGCCATCCTATATTTTCATCCTGCATT	Tb927.11.16070_rev	GGGGACCACTTTGTACAAGAAAGCTGGGTTCACTTAGAAGCGGCGCAGT
2_known	L11	Tb927.8.480_for	GGGGACAAGTTTGTACAAAAAAGCAGGCTATGGCAGATGATGGTAAGACAGGG	Tb927.8.480_rev	GGGGACCACTTTGTACAAGAAAGCTGGGTTCACTCAGCGACATCAGCGA
2_known	L12	Tb927.10.13930_for	GGGGACAAGTTTGTACAAAAAAGCAGGCTATGGCCACTGGAGGTCGTT	Tb927.10.13930_rev	GGGGACCACTTTGTACAAGAAAGCTGGGTTCAAACAGTATCATTGTCGTTTGGCG
2_known	L13	Tb927.11.1150_for	GGGGACAAGTTTGTACAAAAAAGCAGGCTATGCAAGGGCAAAACCAAGCA	Tb927.11.1150_rev	GGGGACCACTTTGTACAAGAAAGCTGGGTTCAGCTATTCAACTGTATGGCAACTAC
2_known	L14	Tb927.6.2790_for	GGGGACAAGTTTGTACAAAAAAGCAGGCTATGTTTTGTTTTTCTCGTTTCGCTCT	Tb927.6.2790_rev	GGGGACCACTTTGTACAAGAAAGCTGGGTTCACAAGGATGGAAGTCCATGAAGG
2_known	L15	Tb927.11.1490_for	GGGGACAAGTTTGTACAAAAAAGCAGGCTATGCCGGCAGTTTTGTGCG	Tb927.11.1490_rev	GGGGACCACTTTGTACAAGAAAGCTGGGTCTACAGACAGTGTTTCAGAATAGTTGANNN
2_known	L16	Tb927.8.5600_for	GGGGACAAGTTTGTACAAAAAAGCAGGCTATGAATCAACTGGAGAGCCTCAAGA	Tb927.8.5600_rev	GGGGACCACTTTGTACAAGAAAGCTGGGTTTACAAAAGTGTAGCGATGAGTGCT
2_known	L17	Tb927.2.5800_for	GGGGACAAGTTTGTACAAAAAAGCAGGCTATGATGCGACAAAATCTGACAAGTGA	Tb927.2.5800_rev	GGGGACCACTTTGTACAAGAAAGCTGGGTCTATAGTTTGCTGCACCCGCG
2_known	L18	Tb927.5.800_for	GGGGACAAGTTTGTACAAAAAAGCAGGCTATGAGCGTAGAGCTTCGTGTGG	Tb927.5.800_rev	GGGGACCACTTTGTACAAGAAAGCTGGGTTTAGACGGGATGTTCATCTTCCTTTTC
2_known	L19	Tb927.11.15650_for	GGGGACAAGTTTGTACAAAAAAGCAGGCTATGCCACCATCACCAGCAGT	Tb927.11.15650_rev	GGGGACCACTTTGTACAAGAAAGCTGGGTCTAGACAATAGTGAGTAGCGCCGT
2_known	L20	Tb927.11.1020_for	GGGGACAAGTTTGTACAAAAAAGCAGGCTATGAGTGCTGCCGGCGTT	Tb927.11.1020_rev	GGGGACCACTTTGTACAAGAAAGCTGGGTCTATATTTTGCATTCCTTCACCTCAGCA
2_known	L21	Tb927.10.15600_for	GGGGACAAGTTTGTACAAAAAAGCAGGCTATGAACAATGGCGATAATTCCTCCG	Tb927.10.15600_rev	GGGGACCACTTTGTACAAGAAAGCTGGGTTTACAGTGTGCCTTCACCGTTAC
2_known	L22	Tb927.8.810_for	GGGGACAAGTTTGTACAAAAAAGCAGGCTATGTCATCTGTTTCCGATTGTATATTTGGC	Tb927.8.810_rev	GGGGACCACTTTGTACAAGAAAGCTGGGTTTACGGAAGGAGAAAGCAAACCTC
2_known	L23	Tb927.9.11100_for	GGGGACAAGTTTGTACAAAAAAGCAGGCTATGACGACAACTCCCACTGGT	Tb927.9.11100_rev	GGGGACCACTTTGTACAAGAAAGCTGGGTCTAAAAACCACGGAATGCAACCTG
2_known	L24	Tb927.8.1310_for	GGGGACAAGTTTGTACAAAAAAGCAGGCTATGGTTGGTGGCGATGGTGA	Tb927.8.1310_rev	GGGGACCACTTTGTACAAGAAAGCTGGGTTCAGGCAGCCGGTAAAAACC
2_known	M1	Tb927.11.6090_for	GGGGACAAGTTTGTACAAAAAAGCAGGCTATGTCGTCAGTTACAGCCAAATATTTACC	Tb927.11.6090_rev	GGGGACCACTTTGTACAAGAAAGCTGGGTTTACCCATCTGGGTGACACGG
2_known	M2	Tb927.10.690_for	GGGGACAAGTTTGTACAAAAAAGCAGGCTATGACACGCACATCACGGAG	Tb927.10.690_rev	GGGGACCACTTTGTACAAGAAAGCTGGGTTTAAACGGCATGCACCACGT
2_known	M3	Tb927.11.1780_for	GGGGACAAGTTTGTACAAAAAAGCAGGCTATGCATGTGGTAAAGTTGTTGTTGAG	Tb927.11.1780_rev	GGGGACCACTTTGTACAAGAAAGCTGGGTTCAAGGCCAATCCCTGTAAGGG
2_known	M4	Tb927.11.2130_for	GGGGACAAGTTTGTACAAAAAAGCAGGCTATGCAAACGACCGGTGAGC	Tb927.11.2130_rev	GGGGACCACTTTGTACAAGAAAGCTGGGTTTACGGTAGGCCGTCTTCGAA
2_known	M5	Tb927.11.12610_for	GGGGACAAGTTTGTACAAAAAAGCAGGCTATGATGACAAACTTGAATGTGCGGA	Tb927.11.12610_rev	GGGGACCACTTTGTACAAGAAAGCTGGGTTCACCTGGGTGCACATAGTAGC

2_known	M6	Tb927.9.11260_for	GGGGACAAGTTTGTACAAAAAAGCAGGCTATGACAGCTCTAGAGCGGTACG	Tb927.9.11260_rev	GGGGACCACTTTGTACAAGAAAGCTGGGTCTAAGCGTTCTGCAGAAGCGA
2_known	M7	Tb927.10.9560_for	GGGGACAAGTTTGTACAAAAAAGCAGGCTATGCAGAGGAAAATTGTAGCGAAGA	Tb927.10.9560_rev	GGGGACCACTTTGTACAAGAAAGCTGGGTTCAAATCTCAACGATGACCTTTCCC
2_known	M8	Tb927.3.1400_for	GGGGACAAGTTTGTACAAAAAAGCAGGCTATGACCCACAATGTGATGTGTGATG	Tb927.3.1400_rev	GGGGACCACTTTGTACAAGAAAGCTGGGTCTAAGATCCCGTAGACCCATTGATTATATT
2_known	M9	Tb927.4.2970_for	GGGGACAAGTTTGTACAAAAAAGCAGGCTATGACAACATATGGTCACCGTCCT	Tb927.4.2970_rev	GGGGACCACTTTGTACAAGAAAGCTGGGTCTATGCCACAATTCGCCGCT
2_known	M10	Tb927.8.2480_for	GGGGACAAGTTTGTACAAAAAAGCAGGCTATGATGATTTCCCAAAGTCATTCGCC	Tb927.8.2480_rev	GGGGACCACTTTGTACAAGAAAGCTGGGTTCAGTACACGAATGGTATCACCGC
2_known	M11	Tb927.10.5660_for	GGGGACAAGTTTGTACAAAAAAGCAGGCTATGCGAGGTCTAATAAAATCATTTAGCGG	Tb927.10.5660_rev	GGGGACCACTTTGTACAAGAAAGCTGGGTTCAGATCTCAAATGTACGGTCGACA
2_known	M12	Tb927.10.2370_for	GGGGACAAGTTTGTACAAAAAAGCAGGCTATGCCACTTTCCTCCGAGAACA	Tb927.10.2370_rev	GGGGACCACTTTGTACAAGAAAGCTGGGTTTATTCACGTGACCGCTTGTGTC
2_known	M13	Tb927.6.4430_for	GGGGACAAGTTTGTACAAAAAAGCAGGCTATGTGTGTCCTTCCAAGGAAGGT	Tb927.6.4430_rev	GGGGACCACTTTGTACAAGAAAGCTGGGTCTAAGTCGATACATAAACAAGATTATGCCG
2_known	M14	Tb927.7.7410_for	GGGGACAAGTTTGTACAAAAAAGCAGGCTATGTCGAGGCTGTCTTCGAAGG	Tb927.7.7410_rev	GGGGACCACTTTGTACAAGAAAGCTGGGTTTACTTCTTGACAACAACAATACCCTTTGT
2_known	M15	Tb927.5.3520_for	GGGGACAAGTTTGTACAAAAAAGCAGGCTATGCACGGAGTGTATCCTATTTTAGTTG	Tb927.5.3520_rev	GGGGACCACTTTGTACAAGAAAGCTGGGTTCACAACTGTGCCAGCACC
2_known	M16	Tb927.4.5300_for	GGGGACAAGTTTGTACAAAAAAGCAGGCTATGTTCGTTCCGTCTAATGTTGCT	Tb927.4.5300_rev	GGGGACCACTTTGTACAAGAAAGCTGGGTTCAGGCCCTCTTACGCCAA
2_known	M17	Tb927.10.8540_for	GGGGACAAGTTTGTACAAAAAAGCAGGCTATGGGTATTGACTACTACAAGGTGCT	Tb927.10.8540_rev	GGGGACCACTTTGTACAAGAAAGCTGGGTCTAAAATTCACATTTTTCAACCAATCGCTT
2_known	M18	Tb927.8.6340_for	GGGGACAAGTTTGTACAAAAAAGCAGGCTATGAAGAAGGCTTTGATCTCGGATTT	Tb927.8.6340_rev	GGGGACCACTTTGTACAAGAAAGCTGGGTTCAAACTGCTGCGCCTTCC
2_known	M19	Tb927.8.4410_for	GGGGACAAGTTTGTACAAAAAAGCAGGCTATGCTGTTGCTGTTAGAGAGGGA	Tb927.8.4410_rev	GGGGACCACTTTGTACAAGAAAGCTGGGTTCAGGTTATTGTAATCTTCGTTTCTTCAGC
2_known	M20	Tb927.10.12830_for	GGGGACAAGTTTGTACAAAAAAGCAGGCTATGAGCGCAGTGGAGCTTG	Tb927.10.12830_rev	GGGGACCACTTTGTACAAGAAAGCTGGGTTTAACTTCTGAATAACTCAATATCAGCAAC
2_known	M21	Tb927.9.11030_for	GGGGACAAGTTTGTACAAAAAAGCAGGCTATGCCTGTAGCCACGTCGA	Tb927.9.11030_rev	GGGGACCACTTTGTACAAGAAAGCTGGGTCTAAAACCCACGGAACTCCAATTGT
2_known	M22	Tb927.2.4830_for	GGGGACAAGTTTGTACAAAAAAGCAGGCTATGTATGGCCGCTGTCTGCA	Tb927.2.4830_rev	GGGGACCACTTTGTACAAGAAAGCTGGGTTCAAGAAGCGCTACTGTGCTTG
2_known	M23	Tb927.11.12810_for	GGGGACAAGTTTGTACAAAAAAGCAGGCTATGCCACCCAAATCTCACAAGC	Tb927.11.12810_rev	GGGGACCACTTTGTACAAGAAAGCTGGGTCTAGAAATCTGCGTCCAGAGAGAAAAC
2_known	M24	Tb927.10.13010_for	GGGGACAAGTTTGTACAAAAAAGCAGGCTATGAAGTCGGATGGGTGCCT	Tb927.10.13010_rev	GGGGACCACTTTGTACAAGAAAGCTGGGTTCAGATCCTCGTGTATTCACCATCG
2_known	N2	Tb927.10.15370_for	GGGGACAAGTTTGTACAAAAAAGCAGGCTATGGTCAAAGTGTACAATTTGCAACG	Tb927.10.15370_rev	GGGGACCACTTTGTACAAGAAAGCTGGGTCTATTCCGTAAACGCAGTGGCT
2_known	N3	Tb927.3.5500_for	GGGGACAAGTTTGTACAAAAAAGCAGGCTATGAAGGTGACGATTGTAGACTGC	Tb927.3.5500_rev	GGGGACCACTTTGTACAAGAAAGCTGGGTTCAATTCATCACGAACTTGAGATCGT
2_known	N4	Tb927.9.9410_for	GGGGACAAGTTTGTACAAAAAAGCAGGCTATGGCTGTCCCACCAGTGG	Tb927.9.9410_rev	GGGGACCACTTTGTACAAGAAAGCTGGGTTCATACCTCGTTAGTTGATACCTCACA
2_known	N5	Tb927.10.11160_for	GGGGACAAGTTTGTACAAAAAAGCAGGCTATGCGGGTCGGTTCCTGG	Tb927.10.11160_rev	GGGGACCACTTTGTACAAGAAAGCTGGGTTTAGTCCTTGCCTTGCCGTTG
2_known	N9	Tb927.6.3760_for	GGGGACAAGTTTGTACAAAAAAGCAGGCTATGAAGCTAGAGAGATCGTCCTGC	Tb927.6.3760_rev	GGGGACCACTTTGTACAAGAAAGCTGGGTTCAAATAGGAATGGCTAGTATGTGTGAAAC
2_known	N10	Tb927.8.2740_for	GGGGACAAGTTTGTACAAAAAAGCAGGCTATGTTCCGAAGGCTCACACAGT	Tb927.8.2740_rev	GGGGACCACTTTGTACAAGAAAGCTGGGTTCACTGGACAAAGAGTCCAGCC
2_known	N11	Tb927.7.4860_for	GGGGACAAGTTTGTACAAAAAAGCAGGCTATGACTGAGCATATCTGTGCTTCG	Tb927.7.4860_rev	GGGGACCACTTTGTACAAGAAAGCTGGGTCTATTGTACGGTTTCCCTGGCTAC
2_known	N12	Tb927.10.8920_for	GGGGACAAGTTTGTACAAAAAAGCAGGCTATGACATCCAGTTTCTCGCACAT	Tb927.10.8920_rev	GGGGACCACTTTGTACAAGAAAGCTGGGTCTAGTTGCGTCGGAAAATACTATAAGCA
2_known	N13	Tb927.11.14360_for	GGGGACAAGTTTGTACAAAAAAGCAGGCTATGGCACAAAGTACTGCTCGTC	Tb927.11.14360_rev	GGGGACCACTTTGTACAAGAAAGCTGGGTCTAAAAAAAATTCTGCTGCATTAACTTCTC
2_known	N14	Tb927.9.15930_for	GGGGACAAGTTTGTACAAAAAAGCAGGCTATGCCAGCACCCGCCTCT	Tb927.9.15930_rev	GGGGACCACTTTGTACAAGAAAGCTGGGTTTATTGGCAGCAACAATTATCATTTCTCTG
2_known	N15	Tb927.3.4990_for	GGGGACAAGTTTGTACAAAAAAGCAGGCTATGAAAACTATGCGTGCGGTTACG	Tb927.3.4990_rev	GGGGACCACTTTGTACAAGAAAGCTGGGTTCACTCACCTGTAGAGTCGCAC
2_known	N16	Tb927.4.1170_for	GGGGACAAGTTTGTACAAAAAAGCAGGCTATGGAAGCCGCCTTTGATGC	Tb927.4.1170_rev	GGGGACCACTTTGTACAAGAAAGCTGGGTTTAATCCCTACGTAGAGAAAATTCCATGG
2_known	N17	Tb927.2.5180_for	GGGGACAAGTTTGTACAAAAAAGCAGGCTATGCCTGGGGTTAACGTGATGA	Tb927.2.5180_rev	GGGGACCACTTTGTACAAGAAAGCTGGGTTTACGAAAAGAAACTCTCGCCGG
2_known	N18	Tb927.4.1640_for	GGGGACAAGTTTGTACAAAAAAGCAGGCTATGACGGCGCGTGACGGG	Tb927.4.1640_rev	GGGGACCACTTTGTACAAGAAAGCTGGGTTTAAATAGTTTCTTCCGACCTTGACCTC
2_known	N19	Tb927.10.8750_for	GGGGACAAGTTTGTACAAAAAAGCAGGCTATGGTGGGGTCGAGGACCA	Tb927.10.8750_rev	GGGGACCACTTTGTACAAGAAAGCTGGGTTTAGAGCTGTTGACAAAACTTCCTCTC
2_known	N20	Tb927.8.2230_for	GGGGACAAGTTTGTACAAAAAAGCAGGCTATGAAATTCAAAGCAACAGTACGAGATCG	Tb927.8.2230_rev	GGGGACCACTTTGTACAAGAAAGCTGGGTTCAATTTGTTACAGCCGGTATGCA
2_known	N21	Tb927.7.3730_for	GGGGACAAGTTTGTACAAAAAAGCAGGCTATGGAAACGCACCAGGATCCG	Tb927.7.3730_rev	GGGGACCACTTTGTACAAGAAAGCTGGGTTTAGTTTGGGCTCATGAAGTTGGTG
2_known	N22	Tb927.6.560_for	GGGGACAAGTTTGTACAAAAAAGCAGGCTATGCATCTCATGCGTGCCTG	Tb927.6.560_rev	GGGGACCACTTTGTACAAGAAAGCTGGGTCTACGCCGTGTTGGGTGC
2_known	N23	Tb927.11.9550_for	GGGGACAAGTTTGTACAAAAAAGCAGGCTATGACACAACAGACAGTACCTTGGG	Tb927.11.9550_rev	GGGGACCACTTTGTACAAGAAAGCTGGGTTTACGACTTCGTTGCGACAATGA
2_known	N24	Tb927.10.14080_for	GGGGACAAGTTTGTACAAAAAAGCAGGCTATGGCCGATCGAGGTGACG	Tb927.10.14080_rev	GGGGACCACTTTGTACAAGAAAGCTGGGTCTAGGCCACACTCCGGCA
2_known	O1	Tb927.8.4430_for	GGGGACAAGTTTGTACAAAAAAGCAGGCTATGGCTGCATCCGCTAATGG	Tb927.8.4430_rev	GGGGACCACTTTGTACAAGAAAGCTGGGTCTAAGCGCTGACAAGCGCT
2_known	O3	Tb927.11.5860_for	GGGGACAAGTTTGTACAAAAAAGCAGGCTATGTGTGGGAAGGATTCATCACATG	Tb927.11.5860_rev	GGGGACCACTTTGTACAAGAAAGCTGGGTCTAAAAACCGGCCATAAGCTCTTCA
2_known	O4	Tb927.1.870_for	GGGGACAAGTTTGTACAAAAAAGCAGGCTATGTCAGGTGTACCTTTTCCTTCACG	Tb927.1.870_rev	GGGGACCACTTTGTACAAGAAAGCTGGGTCTAGTTCCCATCCTCCCTCACAC
2_known	O5	Tb927.8.1150_for	GGGGACAAGTTTGTACAAAAAAGCAGGCTATGTCCTCGGTTCTTACGCGG	Tb927.8.1150_rev	GGGGACCACTTTGTACAAGAAAGCTGGGTTCAGATGTTCGTGGAGTCTCGA
2_known	O6	Tb927.10.6140_for	GGGGACAAGTTTGTACAAAAAAGCAGGCTATGCGCAATTGTCAGCCGG	Tb927.10.6140_rev	GGGGACCACTTTGTACAAGAAAGCTGGGTTCACTGAAGCATGCCTGCG
2_known	O7	Tb927.10.380_for	GGGGACAAGTTTGTACAAAAAAGCAGGCTATGCTCCGCCGTAGTGATTTACT	Tb927.10.380_rev	GGGGACCACTTTGTACAAGAAAGCTGGGTCTAATATGCAGTTCCACAAACCTCTGC
2_known	O8	Tb927.11.9610_for	GGGGACAAGTTTGTACAAAAAAGCAGGCTATGAACATTCAAGGCATGGCTCTG	Tb927.11.9610_rev	GGGGACCACTTTGTACAAGAAAGCTGGGTTTATATGCCTTCGGGTGTCCAGA
2_known	O9	Tb927.9.6090_for	GGGGACAAGTTTGTACAAAAAAGCAGGCTATGGTGAGGACGACACGCT	Tb927.9.6090_rev	GGGGACCACTTTGTACAAGAAAGCTGGGTCTAAAGTCTTGAAGGAGTGTGTCGC
2_known	O11	Tb927.6.1610_for	GGGGACAAGTTTGTACAAAAAAGCAGGCTATGCCGAAGGAACAACGCAG	Tb927.6.1610_rev	GGGGACCACTTTGTACAAGAAAGCTGGGTCTACACAAAATGGATATCATTCTTTGTGAA
2_known	O12	Tb927.8.3910_for	GGGGACAAGTTTGTACAAAAAAGCAGGCTATGGTGGCCGTGAACCGA	Tb927.8.3910_rev	GGGGACCACTTTGTACAAGAAAGCTGGGTTTAAATATGTTTTGCGACGGCATTGTATAT
2_known	O13	Tb927.9.9520_for	GGGGACAAGTTTGTACAAAAAAGCAGGCTATGACACAGGTCATCACGGAGA	Tb927.9.9520_rev	GGGGACCACTTTGTACAAGAAAGCTGGGTTCACCACAACGGCACGGA
2_known	O14	Tb927.10.1940_for	GGGGACAAGTTTGTACAAAAAAGCAGGCTATGGTAGACTGCACGGTCGA	Tb927.10.1940_rev	GGGGACCACTTTGTACAAGAAAGCTGGGTTTACGGCATCCTCAGAAAGGGG
2_known	O15	Tb927.11.4060_for	GGGGACAAGTTTGTACAAAAAAGCAGGCTATGACTTCCGCAGGTCGTCC	Tb927.11.4060_rev	GGGGACCACTTTGTACAAGAAAGCTGGGTCTACCGGAGTGGGTTCGTCT
2_known	O16	Tb927.6.2480_for	GGGGACAAGTTTGTACAAAAAAGCAGGCTATGCGACGCGTGACTTCC	Tb927.6.2480_rev	GGGGACCACTTTGTACAAGAAAGCTGGGTTTAGTATGGTCTGTGATATGAGTACAACTT
2_known	O17	Tb927.9.5710_for	GGGGACAAGTTTGTACAAAAAAGCAGGCTATGTCGACTACCACAGATTTTGTTGG	Tb927.9.5710_rev	GGGGACCACTTTGTACAAGAAAGCTGGGTTCATGTTTCCTCCCTCTTCACGC
2_known	O18	Tb927.6.2360_for	GGGGACAAGTTTGTACAAAAAAGCAGGCTATGTCATCCGCTCCTCTGAGG	Tb927.6.2360_rev	GGGGACCACTTTGTACAAGAAAGCTGGGTTCAAGGAGAGAAACTGGGCTTCT
2_known	O20	Tb927.7.7360_for	GGGGACAAGTTTGTACAAAAAAGCAGGCTATGCAGGTGCAGGTGCAGG	Tb927.7.7360_rev	GGGGACCACTTTGTACAAGAAAGCTGGGTTTAAGCTCCGTTGAACCGCATC
2_known	O22	Tb927.1.4690_for	GGGGACAAGTTTGTACAAAAAAGCAGGCTATGACGGTGGACGCAAATGC	Tb927.1.4690_rev	GGGGACCACTTTGTACAAGAAAGCTGGGTCTACCGCAGCCGAAAATCCTG
2_known	O23	Tb927.9.800_for	GGGGACAAGTTTGTACAAAAAAGCAGGCTATGTACTTTTGTCTCTATACAGCTTGTTTG	Tb927.9.800_rev	GGGGACCACTTTGTACAAGAAAGCTGGGTTTAACAGCTAGCTTCATGCCAGAATG
2_known	O24	Tb927.11.14350_for	GGGGACAAGTTTGTACAAAAAAGCAGGCTATGACAACCGCCCTACGAGA	Tb927.11.14350_rev	GGGGACCACTTTGTACAAGAAAGCTGGGTTCAGACGCGGCGGGAGAA
2_known	P1	Tb927.10.15380_for	GGGGACAAGTTTGTACAAAAAAGCAGGCTATGACAAAGGGCAAGGGCCG	Tb927.10.15380_rev	GGGGACCACTTTGTACAAGAAAGCTGGGTTTAACGAGCGCGTCGCTTAG
2_known	P2	Tb927.4.5030_for	GGGGACAAGTTTGTACAAAAAAGCAGGCTATGAACTGTGACGAAATCATAAAGAAACTC	Tb927.4.5030_rev	GGGGACCACTTTGTACAAGAAAGCTGGGTCTAAAAGAAGTACCTCAACAGCGTGC
2_known	P3	Tb927.8.7390_for	GGGGACAAGTTTGTACAAAAAAGCAGGCTATGAATTGTCGTGAGATCATAAGGAAGC	Tb927.8.7390_rev	GGGGACCACTTTGTACAAGAAAGCTGGGTCTAAAAGAAGTACCTCAGCAGCGTAC
2_known	P4	Tb927.6.940_for	GGGGACAAGTTTGTACAAAAAAGCAGGCTATGTGCTCCTTAATTACACAACTCTGTG	Tb927.6.940_rev	GGGGACCACTTTGTACAAGAAAGCTGGGTCTATTGGATAGATCTGTCAACAGAAAACAT
2_known	P5	Tb927.6.3890_for	GGGGACAAGTTTGTACAAAAAAGCAGGCTATGTTGCCAGACCCACCACT	Tb927.6.3890_rev	GGGGACCACTTTGTACAAGAAAGCTGGGTTTACACTGAGCATATGAAACTTCCTAATTC
2_known	P6	Tb927.6.3480_for	GGGGACAAGTTTGTACAAAAAAGCAGGCTATGTGGTACAGTAATTTGTTCGTAGCA	Tb927.6.3480_rev	GGGGACCACTTTGTACAAGAAAGCTGGGTTTACCAGGGTCCATATACAAGTGTTCG
2_known	P7	Tb927.9.9380_for	GGGGACAAGTTTGTACAAAAAAGCAGGCTATGGCTGTCCCACCAGTGG	Tb927.9.9380_rev	GGGGACCACTTTGTACAAGAAAGCTGGGTTCACACATACGCCCCACATTTAAA
2_known	P8	Tb927.10.4040_for	GGGGACAAGTTTGTACAAAAAAGCAGGCTATGGGAGTTATATTTCTTGTGTGTGCG	Tb927.10.4040_rev	GGGGACCACTTTGTACAAGAAAGCTGGGTTTACATGACTTTTTTACCCCTCTCTGATTC
2_known	P9	Tb927.6.3000_for	GGGGACAAGTTTGTACAAAAAAGCAGGCTATGCTGGCTTCTCAACTGAAAACC	Tb927.6.3000_rev	GGGGACCACTTTGTACAAGAAAGCTGGGTTTAATTTTCTTCCTTTCCGATTACCTCAGC
2_known	P10	Tb927.11.16290_for	GGGGACAAGTTTGTACAAAAAAGCAGGCTATGATTGGTTTCGTGTACAATATAGTGTGT	Tb927.11.16290_rev	GGGGACCACTTTGTACAAGAAAGCTGGGTCTAGGTAACCGGTTGGGATGCT
2_known	P11	Tb927.3.1790_for	GGGGACAAGTTTGTACAAAAAAGCAGGCTATGCGCCGTTTTGCTACTACTT	Tb927.3.1790_rev	GGGGACCACTTTGTACAAGAAAGCTGGGTTTAGCTCAGAATGCGACGTGC
2_known	P12	Tb927.11.20730_for	GGGGACAAGTTTGTACAAAAAAGCAGGCTATGGCCTTTTCTGCACTAGCC	Tb927.11.20730_rev	GGGGACCACTTTGTACAAGAAAGCTGGGTTCAGTTCAGCAAATTCATCGTATTAGAAAC
2_known	P13	Tb927.4.1720_for	GGGGACAAGTTTGTACAAAAAAGCAGGCTATGGCGCGTTCACTGCAG	Tb927.4.1720_rev	GGGGACCACTTTGTACAAGAAAGCTGGGTCTATTCATCTACATCCTCCGGTCTAAAGA
2_known	P14	Tb927.1.3320_for	GGGGACAAGTTTGTACAAAAAAGCAGGCTATGTCGACATCAAAATCTGAAAAAGGTG	Tb927.1.3320_rev	GGGGACCACTTTGTACAAGAAAGCTGGGTTTAACGAGGGTACGAGAGAATTTCCT
2_known	P15	Tb927.9.3760_for	GGGGACAAGTTTGTACAAAAAAGCAGGCTATGTTTCCGTTTTACTCATGCGACC	Tb927.9.3760_rev	GGGGACCACTTTGTACAAGAAAGCTGGGTCTATCTTGCTTTCAGCTGTGTAGGATT
2_known	P16	Tb927.9.14430_for	GGGGACAAGTTTGTACAAAAAAGCAGGCTATGTCCGTCTCTTCGCAACCT	Tb927.9.14430_rev	GGGGACCACTTTGTACAAGAAAGCTGGGTCTACTCCGTGACAACATTCCCTCT
2_known	P17	Tb927.9.9620_for	GGGGACAAGTTTGTACAAAAAAGCAGGCTATGCAGCACGTGGGAACG	Tb927.9.9620_rev	GGGGACCACTTTGTACAAGAAAGCTGGGTCTACTCACGTGCGTCAACAATCC
2_known	P18	Tb927.10.7990_for	GGGGACAAGTTTGTACAAAAAAGCAGGCTATGCTGTGGGTCGACAAGTACC	Tb927.10.7990_rev	GGGGACCACTTTGTACAAGAAAGCTGGGTTCAGTTTCGCTTGAGAAGTTGCA
2_known	P19	Tb927.10.7780_for	GGGGACAAGTTTGTACAAAAAAGCAGGCTATGGTGTCGTTCAGCATCGATG	Tb927.10.7780_rev	GGGGACCACTTTGTACAAGAAAGCTGGGTTTACAAGGCTAACTTTTGCTTTCGACG
2_known	P20	Tb927.9.8720_for	GGGGACAAGTTTGTACAAAAAAGCAGGCTATGCCTGACTACAGCCGCG	Tb927.9.8720_rev	GGGGACCACTTTGTACAAGAAAGCTGGGTTTAAAGCTTGCTCCTCTCCCTTTG
2_known	P21	Tb927.10.9760_for	GGGGACAAGTTTGTACAAAAAAGCAGGCTATGCTGCTCACAAAACTTGCGC	Tb927.10.9760_rev	GGGGACCACTTTGTACAAGAAAGCTGGGTTTATTGCACCTCGTGCATTTCGT
2_known	P22	Tb927.7.1110_for	GGGGACAAGTTTGTACAAAAAAGCAGGCTATGGGCGACGACGGTTATTCG	Tb927.7.1110_rev	GGGGACCACTTTGTACAAGAAAGCTGGGTTTACAACAAATTGTGCTTTTTCCGAATCT
2_known	P23	Tb927.6.600_for	GGGGACAAGTTTGTACAAAAAAGCAGGCTATGATGCAGTATGGTGGCGGG	Tb927.6.600_rev	GGGGACCACTTTGTACAAGAAAGCTGGGTTCAGCTATGACCCTTTACCGCT
2_known	P24	Tb927.8.6540_for	GGGGACAAGTTTGTACAAAAAAGCAGGCTATGCTTAAAACCGTGCTACTACTTGA	Tb927.8.6540_rev	GGGGACCACTTTGTACAAGAAAGCTGGGTTCACGAGGTGGCAACGCA
3_hypothetical	A1	Tb927.7.4270_for	GGGGACAAGTTTGTACAAAAAAGCAGGCTATGGTGATGAAGGCTTTCCACCT	Tb927.7.4270_rev	GGGGACCACTTTGTACAAGAAAGCTGGGTTTATTTGTTTGTTTCCTCAGTCCTATAGTG
3_hypothetical	A2	Tb927.10.11750_for	GGGGACAAGTTTGTACAAAAAAGCAGGCTATGCCAGTTGAGCAGTTGGAAG	Tb927.10.11750_rev	GGGGACCACTTTGTACAAGAAAGCTGGGTCTAGTTAGGTTTCCACAGCTTTAACGC
3_hypothetical	A3	Tb927.11.2410_for	GGGGACAAGTTTGTACAAAAAAGCAGGCTATGCCCTGCTGCAGCAAC	Tb927.11.2410_rev	GGGGACCACTTTGTACAAGAAAGCTGGGTTTACGTCGAACTCGGAACAACTTT
3_hypothetical	A4	Tb927.3.4250_for	GGGGACAAGTTTGTACAAAAAAGCAGGCTATGTTGCAACTAACGGTTGAGGAC	Tb927.3.4250_rev	GGGGACCACTTTGTACAAGAAAGCTGGGTCTCACGTTGATGTATCATACGGAGGA
3_hypothetical	A5	Tb927.5.140_for	GGGGACAAGTTTGTACAAAAAAGCAGGCTATGCCCATTATGTGCCATCGG	Tb927.5.140_rev	GGGGACCACTTTGTACAAGAAAGCTGGGTTTACACGTTATTCGTTGGCAGCT
3_hypothetical	A6	Tb927.11.16180_for	GGGGACAAGTTTGTACAAAAAAGCAGGCTATGACTCAGCAGCAACCCCC	Tb927.11.16180_rev	GGGGACCACTTTGTACAAGAAAGCTGGGTTTAAGCTATTTTTTCACCAAGTTGCTTGG
3_hypothetical	A7	Tb927.4.970_for	GGGGACAAGTTTGTACAAAAAAGCAGGCTATGACAAGCCGCCGTGTC	Tb927.4.970_rev	GGGGACCACTTTGTACAAGAAAGCTGGGTTTAGAGGGGATTTAGTCCTCGAATCC
3_hypothetical	A8	Tb927.9.4920_for	GGGGACAAGTTTGTACAAAAAAGCAGGCTATGGCCGCGGATGATGAGT	Tb927.9.4920_rev	GGGGACCACTTTGTACAAGAAAGCTGGGTCTAAATATTTCTATTCACCAGACGAATGCA
3_hypothetical	A9	Tb927.11.1950_for	GGGGACAAGTTTGTACAAAAAAGCAGGCTATGAAGCGTACACGCAATGCT	Tb927.11.1950_rev	GGGGACCACTTTGTACAAGAAAGCTGGGTCTATTCTAGTGCCTCAAGAACGTGTT
3_hypothetical	A10	Tb927.3.5670_for	GGGGACAAGTTTGTACAAAAAAGCAGGCTATGGCTAAGGTTGATACGCTGC	Tb927.3.5670_rev	GGGGACCACTTTGTACAAGAAAGCTGGGTCTAGACAACAACAAAAAAAAGTATTGCATC
3_hypothetical	A11	Tb927.6.1470_for	GGGGACAAGTTTGTACAAAAAAGCAGGCTATGCGCCGAGACGACTTCG	Tb927.6.1470_rev	GGGGACCACTTTGTACAAGAAAGCTGGGTTCAAGCCGCCCCTCGGAA
3_hypothetical	A12	Tb927.11.14830_for	GGGGACAAGTTTGTACAAAAAAGCAGGCTATGAGTTTGCTTTTGGATGACGACG	Tb927.11.14830_rev	GGGGACCACTTTGTACAAGAAAGCTGGGTTTACATCTTCCTTACACATGCCATTTTGG
3_hypothetical	A13	Tb927.5.289b_for	GGGGACAAGTTTGTACAAAAAAGCAGGCTATGCCTCTCGTTCCAACTGCT	Tb927.5.289b_rev	GGGGACCACTTTGTACAAGAAAGCTGGGTTTATCTCGTGGCACCAGAGCA
3_hypothetical	A14	Tb927.11.4240_for	GGGGACAAGTTTGTACAAAAAAGCAGGCTATGCATCACAATGAGTTCAATAGTGTAGA	Tb927.11.4240_rev	GGGGACCACTTTGTACAAGAAAGCTGGGTTCAAGGGGCAATGCCCATACT
3_hypothetical	A15	Tb927.4.1890_for	GGGGACAAGTTTGTACAAAAAAGCAGGCTATGTCTGACCTTGACCGTGATAGT	Tb927.4.1890_rev	GGGGACCACTTTGTACAAGAAAGCTGGGTCTAATAATCCTCCTCTTCCTCTTCATTCTC
3_hypothetical	A16	Tb927.8.6570_for	GGGGACAAGTTTGTACAAAAAAGCAGGCTATGGGAAGTTTTGTGGTGCGT	Tb927.8.6570_rev	GGGGACCACTTTGTACAAGAAAGCTGGGTTTAGCTGGAAATGATGGCAGTAATGG
3_hypothetical	A17	Tb927.3.2580_for	GGGGACAAGTTTGTACAAAAAAGCAGGCTATGACGAGACCTGTGTGTGTCT	Tb927.3.2580_rev	GGGGACCACTTTGTACAAGAAAGCTGGGTTCAGAGAAATAGGAGCAACACTGGA
3_hypothetical	A18	Tb927.2.4410_for	GGGGACAAGTTTGTACAAAAAAGCAGGCTATGCTTGAGCCTTCGCAGC	Tb927.2.4410_rev	GGGGACCACTTTGTACAAGAAAGCTGGGTCTATACACGGCCAACGGCTTG
3_hypothetical	A19	Tb927.10.4880_for	GGGGACAAGTTTGTACAAAAAAGCAGGCTATGTTACGCAAATCTTCGGTTTCGT	Tb927.10.4880_rev	GGGGACCACTTTGTACAAGAAAGCTGGGTCTACTCCGCGTTCAGGTACACC
3_hypothetical	A20	Tb927.11.4090_for	GGGGACAAGTTTGTACAAAAAAGCAGGCTATGAGGAGAGTGCTATTATTCCTGTGT	Tb927.11.4090_rev	GGGGACCACTTTGTACAAGAAAGCTGGGTTCAAAGTAACATGCTGTATAAAAGAGGAAT
3_hypothetical	A21	Tb927.4.4200_for	GGGGACAAGTTTGTACAAAAAAGCAGGCTATGCGATCCAACTGCGCAC	Tb927.4.4200_rev	GGGGACCACTTTGTACAAGAAAGCTGGGTTCAGTGTCTCCATAGAACAGGGAG
3_hypothetical	A22	Tb927.8.5890_for	GGGGACAAGTTTGTACAAAAAAGCAGGCTATGACCACAGCCGGTCAGT	Tb927.8.5890_rev	GGGGACCACTTTGTACAAGAAAGCTGGGTTTAACGGTGGTGCTTTTCTTTCTCT
3_hypothetical	A23	Tb927.3.4830_for	GGGGACAAGTTTGTACAAAAAAGCAGGCTATGGAGATTCCCGGTGTATTTTCAGA	Tb927.3.4830_rev	GGGGACCACTTTGTACAAGAAAGCTGGGTTCAGCACGCCGAAGCCAT
3_hypothetical	A24	Tb927.8.860_for	GGGGACAAGTTTGTACAAAAAAGCAGGCTATGTATTTTTTTTTCTTTCAGGTAATCGAT	Tb927.8.860_rev	GGGGACCACTTTGTACAAGAAAGCTGGGTTCAAAACACCTCTTGAAGGGTTCC
3_hypothetical	B1	Tb927.11.4070_for	GGGGACAAGTTTGTACAAAAAAGCAGGCTATGCTGTCGGAAATGTTGTTGCT	Tb927.11.4070_rev	GGGGACCACTTTGTACAAGAAAGCTGGGTTCAAAGTAACATGCTGTATAAAAGAGGAAT
3_hypothetical	B2	Tb927.8.7720_for	GGGGACAAGTTTGTACAAAAAAGCAGGCTATGGCGACAAATAAGGGAATAACGT	Tb927.8.7720_rev	GGGGACCACTTTGTACAAGAAAGCTGGGTTTATCGGGAAGCGACCAGACT
3_hypothetical	B3	Tb927.9.7020_for	GGGGACAAGTTTGTACAAAAAAGCAGGCTATGGCACTCACGCTGATACCG	Tb927.9.7020_rev	GGGGACCACTTTGTACAAGAAAGCTGGGTTCAAAATATTATTTCCCCGTCATCATCGT
3_hypothetical	B4	Tb927.7.3600_for	GGGGACAAGTTTGTACAAAAAAGCAGGCTATGTACCGCAGTGGTACGACG	Tb927.7.3600_rev	GGGGACCACTTTGTACAAGAAAGCTGGGTCTAGGCCACTGCTCGGGT
3_hypothetical	B5	Tb927.10.12200_for	GGGGACAAGTTTGTACAAAAAAGCAGGCTATGACAGCACTGGTGGTCCA	Tb927.10.12200_rev	GGGGACCACTTTGTACAAGAAAGCTGGGTTCAACTCAGTAGCATCACCCATGC
3_hypothetical	B6	Tb927.8.4220_for	GGGGACAAGTTTGTACAAAAAAGCAGGCTATGGGCAAGAAGGCTGAGCA	Tb927.8.4220_rev	GGGGACCACTTTGTACAAGAAAGCTGGGTTCAAGAAAGATCATACGCGGCTTC
3_hypothetical	B7	Tb927.11.5540_for	GGGGACAAGTTTGTACAAAAAAGCAGGCTATGTTGCAGTGTCTCACATATGCG	Tb927.11.5540_rev	GGGGACCACTTTGTACAAGAAAGCTGGGTTCAAATGTACTTTTTGGTCGTGAAAAAGT
3_hypothetical	B8	Tb927.5.3690_for	GGGGACAAGTTTGTACAAAAAAGCAGGCTATGTTCGTAAGGTTGCCGACC	Tb927.5.3690_rev	GGGGACCACTTTGTACAAGAAAGCTGGGTTTATGGCAGCCTGTTCACCTTATTTT
3_hypothetical	B9	Tb927.9.10160_for	GGGGACAAGTTTGTACAAAAAAGCAGGCTATGTTGCATTCCTCAAGAATTGTCCT	Tb927.9.10160_rev	GGGGACCACTTTGTACAAGAAAGCTGGGTTCACCAGATATGGGTCTTTCCACA
3_hypothetical	B10	Tb927.9.9720_for	GGGGACAAGTTTGTACAAAAAAGCAGGCTATGGCAGAGCCGGTGTTGG	Tb927.9.9720_rev	GGGGACCACTTTGTACAAGAAAGCTGGGTTTACAGTGCAAATATCATATCAGCATCGG
3_hypothetical	B11	Tb927.11.4370_for	GGGGACAAGTTTGTACAAAAAAGCAGGCTATGAATGTTCCCGTAAAGCTTAGCG	Tb927.11.4370_rev	GGGGACCACTTTGTACAAGAAAGCTGGGTTTAACGGTGCAAACGCACTGC
3_hypothetical	B12	Tb927.10.11150_for	GGGGACAAGTTTGTACAAAAAAGCAGGCTATGGAAGAGCGTGATGAAATAACGG	Tb927.10.11150_rev	GGGGACCACTTTGTACAAGAAAGCTGGGTTCAACGAATCAGCCTATACATCTGC
3_hypothetical	B13	Tb927.10.3540_for	GGGGACAAGTTTGTACAAAAAAGCAGGCTATGAAGCTTCTAAAGTTCGTCAACTGT	Tb927.10.3540_rev	GGGGACCACTTTGTACAAGAAAGCTGGGTTCACGTTTCCTTTTTAATGCTTCGC
3_hypothetical	B14	Tb927.10.670_for	GGGGACAAGTTTGTACAAAAAAGCAGGCTATGTCGTATCGATTTAAGGGTCGC	Tb927.10.670_rev	GGGGACCACTTTGTACAAGAAAGCTGGGTTCACATGTCCCGCAGCCG
3_hypothetical	B15	Tb927.11.1770_for	GGGGACAAGTTTGTACAAAAAAGCAGGCTATGAGCGGTTGTGGCAGGA	Tb927.11.1770_rev	GGGGACCACTTTGTACAAGAAAGCTGGGTCTACTCAATACCAGTTTCCTCATCACTTC
3_hypothetical	B16	Tb927.9.11660_for	GGGGACAAGTTTGTACAAAAAAGCAGGCTATGACGGAGTATTTGAAGGATATTGGGC	Tb927.9.11660_rev	GGGGACCACTTTGTACAAGAAAGCTGGGTTTAATATAATGTTTCGGGTCTTTTGTCCTC
3_hypothetical	B17	Tb927.7.4880_for	GGGGACAAGTTTGTACAAAAAAGCAGGCTATGAGTCTCCTCTGCAACATTTCCA	Tb927.7.4880_rev	GGGGACCACTTTGTACAAGAAAGCTGGGTTCAAATTTTCGCACCAGTTTTAGATCC
3_hypothetical	B18	Tb927.10.5710_for	GGGGACAAGTTTGTACAAAAAAGCAGGCTATGGACAAGCAGGAAATGTTGATTCT	Tb927.10.5710_rev	GGGGACCACTTTGTACAAGAAAGCTGGGTTTATAGCAAGAAAAATAACGCTGAAAGCAT
3_hypothetical	B19	Tb927.7.6560_for	GGGGACAAGTTTGTACAAAAAAGCAGGCTATGGCCATGAGGCTTGCG	Tb927.7.6560_rev	GGGGACCACTTTGTACAAGAAAGCTGGGTTCACTGCGAGGTTACATTTTCACC
3_hypothetical	B20	Tb927.5.297b_for	GGGGACAAGTTTGTACAAAAAAGCAGGCTATGGGCCGCCACCATCAA	Tb927.5.297b_rev	GGGGACCACTTTGTACAAGAAAGCTGGGTCTACGAGTTCCCTACTCTCGGTT
3_hypothetical	B21	Tb927.11.12020_for	GGGGACAAGTTTGTACAAAAAAGCAGGCTATGGTGGACGAGCTTCCACC	Tb927.11.12020_rev	GGGGACCACTTTGTACAAGAAAGCTGGGTTCATCGAGCTAATTCAAAGTATTTTTCAAT
3_hypothetical	B22	Tb927.8.7330_for	GGGGACAAGTTTGTACAAAAAAGCAGGCTATGATGGGGAAAGTGCTGTTATTTCTG	Tb927.8.7330_rev	GGGGACCACTTTGTACAAGAAAGCTGGGTTCAAAGTAACGTGCTGTATAAAAAAGGAAT
3_hypothetical	B23	Tb927.1.1790_for	GGGGACAAGTTTGTACAAAAAAGCAGGCTATGGGAATTTATTCCGACACTGATGATG	Tb927.1.1790_rev	GGGGACCACTTTGTACAAGAAAGCTGGGTTCATTCAAATTTCCCACCTCTTAAACCA

3_hypothetical	B24	Tb927.4.4790_for	GGGGACAAGTTTGTACAAAAAAGCAGGCTATGAGTAATGCCCCAACACTGC	Tb927.4.4790_rev	GGGGACCACTTTGTACAAGAAAGCTGGGTTTACCGTGGAGAGAAAATGAGGGG
3_hypothetical	C1	Tb927.6.2500_for	GGGGACAAGTTTGTACAAAAAAGCAGGCTATGAGTTGGTTAACTCCTTCAGGTTCC	Tb927.6.2500_rev	GGGGACCACTTTGTACAAGAAAGCTGGGTTTACCACAGCCCTTTGCGAGC
3_hypothetical	C2	Tb927.5.1160_for	GGGGACAAGTTTGTACAAAAAAGCAGGCTATGTTCTCTATGTCGCCATGCG	Tb927.5.1160_rev	GGGGACCACTTTGTACAAGAAAGCTGGGTTCAAAGATCGCCACGGCCG
3_hypothetical	C3	Tb927.7.6880_for	GGGGACAAGTTTGTACAAAAAAGCAGGCTATGAAATACTGCGAAGTGCAACCG	Tb927.7.6880_rev	GGGGACCACTTTGTACAAGAAAGCTGGGTCTATCTTCTGCATATTTTCGCCCTCA
3_hypothetical	C4	Tb927.2.5190_for	GGGGACAAGTTTGTACAAAAAAGCAGGCTATGGCGTCCAAGAAAGGAGGG	Tb927.2.5190_rev	GGGGACCACTTTGTACAAGAAAGCTGGGTTCAACTACTCACCGTCGCTGG
3_hypothetical	C5	Tb927.9.15850_for	GGGGACAAGTTTGTACAAAAAAGCAGGCTATGGACGGAAACTCGGAGGAT	Tb927.9.15850_rev	GGGGACCACTTTGTACAAGAAAGCTGGGTTCACGATTTTGTGGAACCGAGAAT
3_hypothetical	C6	Tb927.10.7210_for	GGGGACAAGTTTGTACAAAAAAGCAGGCTATGTCCCGCGCCACTTATAGC	Tb927.10.7210_rev	GGGGACCACTTTGTACAAGAAAGCTGGGTTTATCCTTCATCAGAATCATCCATATCATT
3_hypothetical	C7	Tb927.10.12410_for	GGGGACAAGTTTGTACAAAAAAGCAGGCTATGGAGGTTAAGATTGGAAAGGATGTTG	Tb927.10.12410_rev	GGGGACCACTTTGTACAAGAAAGCTGGGTTTATGACTCAAGCAGTAATACAGACGC
3_hypothetical	C8	Tb927.3.510_for	GGGGACAAGTTTGTACAAAAAAGCAGGCTATGAAATACTGCGAAGTACAACCGC	Tb927.3.510_rev	GGGGACCACTTTGTACAAGAAAGCTGGGTCTATCTTCTGCATATTTTCGCCCTCA
3_hypothetical	C9	Tb927.8.7180_for	GGGGACAAGTTTGTACAAAAAAGCAGGCTATGCCCACGCCACAGCAG	Tb927.8.7180_rev	GGGGACCACTTTGTACAAGAAAGCTGGGTTCATAGCTCACCCTCGGAATTGA
3_hypothetical	C10	Tb927.10.11330_for	GGGGACAAGTTTGTACAAAAAAGCAGGCTATGTCCAAAAAGGAAGCAATATCGACA	Tb927.10.11330_rev	GGGGACCACTTTGTACAAGAAAGCTGGGTCTATTCATTGGCTAGGACGTCGAC
3_hypothetical	C11	Tb927.3.2260_for	GGGGACAAGTTTGTACAAAAAAGCAGGCTATGAGGCGCGGCGCCCTT	Tb927.3.2260_rev	GGGGACCACTTTGTACAAGAAAGCTGGGTTCATGAACTCTGCTTCATCCCACT
3_hypothetical	C12	Tb927.11.9630_for	GGGGACAAGTTTGTACAAAAAAGCAGGCTATGTTTGGCCGGTTATTTGGTTTTAAG	Tb927.11.9630_rev	GGGGACCACTTTGTACAAGAAAGCTGGGTTCACGACAGTAGTTCCCTCTCTAGT
3_hypothetical	C13	Tb927.6.1410_for	GGGGACAAGTTTGTACAAAAAAGCAGGCTATGCGATGCGTGAGATCCG	Tb927.6.1410_rev	GGGGACCACTTTGTACAAGAAAGCTGGGTTCATCCCTTGCCGATGGACT
3_hypothetical	C14	Tb927.9.2600_for	GGGGACAAGTTTGTACAAAAAAGCAGGCTATGAGCACCGCTCAGTCGT	Tb927.9.2600_rev	GGGGACCACTTTGTACAAGAAAGCTGGGTTCAAGTGAAGGCGCTCGC
3_hypothetical	C15	Tb927.4.340_for	GGGGACAAGTTTGTACAAAAAAGCAGGCTATGCGTTTGACAAGTAGAGGGAGT	Tb927.4.340_rev	GGGGACCACTTTGTACAAGAAAGCTGGGTCACATTTCACCTAACCTCCCTTCT
3_hypothetical	C16	Tb927.4.3720_for	GGGGACAAGTTTGTACAAAAAAGCAGGCTATGCCTAGTAGGCGAGTCACG	Tb927.4.3720_rev	GGGGACCACTTTGTACAAGAAAGCTGGGTCTATTCCTCATCAATAGAGTCATCATCCTC
3_hypothetical	C17	Tb927.7.5840_for	GGGGACAAGTTTGTACAAAAAAGCAGGCTATGAGCAGTTCCAATGATATCGCC	Tb927.7.5840_rev	GGGGACCACTTTGTACAAGAAAGCTGGGTTCAATGGAAGAATCCAATGATTTGCCT
3_hypothetical	C18	Tb927.7.4430_for	GGGGACAAGTTTGTACAAAAAAGCAGGCTATGCGTTCCGTTATTCGTTCCTG	Tb927.7.4430_rev	GGGGACCACTTTGTACAAGAAAGCTGGGTTCAATCATTCCATGGACTTTCTAATGCT
3_hypothetical	C19	Tb927.2.2270_for	GGGGACAAGTTTGTACAAAAAAGCAGGCTATGTCACATGTGAAGGCATTTGACC	Tb927.2.2270_rev	GGGGACCACTTTGTACAAGAAAGCTGGGTTTACCCAATAATAAAAAAGAGTTCATAAAG
3_hypothetical	C20	Tb927.10.7770_for	GGGGACAAGTTTGTACAAAAAAGCAGGCTATGCCATATAAGTCGGGTGTCGC	Tb927.10.7770_rev	GGGGACCACTTTGTACAAGAAAGCTGGGTCTAATCAGAAGCGGAACTGTAATCACT
3_hypothetical	C22	Tb927.10.15110_for	GGGGACAAGTTTGTACAAAAAAGCAGGCTATGGTGGTCATACATAGTGGACTTGT	Tb927.10.15110_rev	GGGGACCACTTTGTACAAGAAAGCTGGGTTTACATAATATGTGAGGATACACCACAC
3_hypothetical	C23	Tb927.10.15960_for	GGGGACAAGTTTGTACAAAAAAGCAGGCTATGTCTACGTGGGAGCAGGC	Tb927.10.15960_rev	GGGGACCACTTTGTACAAGAAAGCTGGGTTCAACACGAGTTGAGGAGTTGC
3_hypothetical	C24	Tb927.9.3620_for	GGGGACAAGTTTGTACAAAAAAGCAGGCTATGACACCTGCTGTAGTTTCGATG	Tb927.9.3620_rev	GGGGACCACTTTGTACAAGAAAGCTGGGTTCATCTCCCAAAGACCGGTC
3_hypothetical	D1	Tb927.4.900_for	GGGGACAAGTTTGTACAAAAAAGCAGGCTATGCGTACAAGTGCGGACATT	Tb927.4.900_rev	GGGGACCACTTTGTACAAGAAAGCTGGGTTTAACTGTGTACTAAATCAGGACTCCGC
3_hypothetical	D2	Tb927.10.15090_for	GGGGACAAGTTTGTACAAAAAAGCAGGCTATGGTGGTCATACATAGTGGACTTGT	Tb927.10.15090_rev	GGGGACCACTTTGTACAAGAAAGCTGGGTTTACATAACATGTGAGGATACACCACAC
3_hypothetical	D3	Tb927.8.4190_for	GGGGACAAGTTTGTACAAAAAAGCAGGCTATGAGCGGCCATAGGCATTG	Tb927.8.4190_rev	GGGGACCACTTTGTACAAGAAAGCTGGGTTTACTCCCCAGTAAAAATGTTATATGTTTG
3_hypothetical	D4	Tb927.4.4810_for	GGGGACAAGTTTGTACAAAAAAGCAGGCTATGGAGCTTACGCAAGCAATAAACC	Tb927.4.4810_rev	GGGGACCACTTTGTACAAGAAAGCTGGGTCTAATATGTGGTGGAGGGAACATCG
3_hypothetical	D5	Tb927.10.5700_for	GGGGACAAGTTTGTACAAAAAAGCAGGCTATGGACAAGCAGGAAATGTTGATTCT	Tb927.10.5700_rev	GGGGACCACTTTGTACAAGAAAGCTGGGTTTATAGCAAGAAAATAACGTTGAAAGCAT
3_hypothetical	D6	Tb927.3.1730_for	GGGGACAAGTTTGTACAAAAAAGCAGGCTATGCTTCGCTCCCTCTTTGGA	Tb927.3.1730_rev	GGGGACCACTTTGTACAAGAAAGCTGGGTTCACGTCGCCCGTCCGCC
3_hypothetical	D7	Tb927.4.2830_for	GGGGACAAGTTTGTACAAAAAAGCAGGCTATGCGACCAGGTAGTCAGTTCA	Tb927.4.2830_rev	GGGGACCACTTTGTACAAGAAAGCTGGGTCTACACACGTTTCCCCTCCGA
3_hypothetical	D8	Tb927.7.4230_for	GGGGACAAGTTTGTACAAAAAAGCAGGCTATGGTGATGAAGGCTTTCCCCC	Tb927.7.4230_rev	GGGGACCACTTTGTACAAGAAAGCTGGGTCTACACGCGTGTTCTCGACC
3_hypothetical	D9	Tb927.4.5230_for	GGGGACAAGTTTGTACAAAAAAGCAGGCTATGCCGATACCCCCGAATAACG	Tb927.4.5230_rev	GGGGACCACTTTGTACAAGAAAGCTGGGTCTACACGTCATCTTCCGAGAATATGG
3_hypothetical	D10	Tb927.11.6930_for	GGGGACAAGTTTGTACAAAAAAGCAGGCTATGGAAGAGACTTAAATGACAGTAGGGC	Tb927.11.6930_rev	GGGGACCACTTTGTACAAGAAAGCTGGGTTCATCGCCTAACCACAGACGG
3_hypothetical	D11	Tb927.8.2140_for	GGGGACAAGTTTGTACAAAAAAGCAGGCTATGGCTTGTGGTGCGATTGT	Tb927.8.2140_rev	GGGGACCACTTTGTACAAGAAAGCTGGGTTTAGAACGCGTTGTGACGCA
3_hypothetical	D12	Tb927.3.600_for	GGGGACAAGTTTGTACAAAAAAGCAGGCTATGTCAAGGTTTGGGGCTTGG	Tb927.3.600_rev	GGGGACCACTTTGTACAAGAAAGCTGGGTCTACAGGGAGAATAACAATACTGAATATAT
3_hypothetical	D13	Tb927.9.15870_for	GGGGACAAGTTTGTACAAAAAAGCAGGCTATGACAAGCCTCACCGAAGAGC	Tb927.9.15870_rev	GGGGACCACTTTGTACAAGAAAGCTGGGTCTACTCTGCATTGTAACCCGATTCC
3_hypothetical	D14	Tb927.10.9270_for	GGGGACAAGTTTGTACAAAAAAGCAGGCTATGAAGAGTTTCCGCGTAGTTTGC	Tb927.10.9270_rev	GGGGACCACTTTGTACAAGAAAGCTGGGTTTAAGCAATCTTCTGCTTTATAAAGAATCG
3_hypothetical	D15	Tb927.4.480_for	GGGGACAAGTTTGTACAAAAAAGCAGGCTATGGTAACGGGGGATATGTCGAC	Tb927.4.480_rev	GGGGACCACTTTGTACAAGAAAGCTGGGTCATGGTGTTTCACAGGTCTCCC
3_hypothetical	D16	Tb927.10.5570_for	GGGGACAAGTTTGTACAAAAAAGCAGGCTATGGAGTCTCCGCCCTCAAAT	Tb927.10.5570_rev	GGGGACCACTTTGTACAAGAAAGCTGGGTTCAAAGGCGCATACCGCAC
3_hypothetical	D17	Tb927.4.3090_for	GGGGACAAGTTTGTACAAAAAAGCAGGCTATGCACCCGTTCTACACTATACGT	Tb927.4.3090_rev	GGGGACCACTTTGTACAAGAAAGCTGGGTCATCGTGCCTGATCCACAAATAC
3_hypothetical	D18	Tb927.7.7510_for	GGGGACAAGTTTGTACAAAAAAGCAGGCTATGGAAATGGAGCAGGCTCCA	Tb927.7.7510_rev	GGGGACCACTTTGTACAAGAAAGCTGGGTCTAGGAGGAAGGAGTTTGAAGAGTGT
3_hypothetical	D19	Tb927.10.5650_for	GGGGACAAGTTTGTACAAAAAAGCAGGCTATGTACCCACTCCTCGTGTGC	Tb927.10.5650_rev	GGGGACCACTTTGTACAAGAAAGCTGGGTCTAGCGGCCAGGCGAAAC
3_hypothetical	D20	Tb927.9.7000_for	GGGGACAAGTTTGTACAAAAAAGCAGGCTATGGAACCTAAGAGCGTCGAGG	Tb927.9.7000_rev	GGGGACCACTTTGTACAAGAAAGCTGGGTTTATTCCCTGACCGTGTAAACAAACA
3_hypothetical	D21	Tb927.7.2270_for	GGGGACAAGTTTGTACAAAAAAGCAGGCTATGATTGAACGCACCGATGTTGT	Tb927.7.2270_rev	GGGGACCACTTTGTACAAGAAAGCTGGGTCTAAACAAATGGATGTAGCATTAGTTGAAA
3_hypothetical	D22	Tb927.7.1260_for	GGGGACAAGTTTGTACAAAAAAGCAGGCTATGATGGAAAGTATGAACCTTGAACAACC	Tb927.7.1260_rev	GGGGACCACTTTGTACAAGAAAGCTGGGTTCAGAAGAAAATCAACGATATTGCTACAAG
3_hypothetical	D23	Tb927.9.12440_for	GGGGACAAGTTTGTACAAAAAAGCAGGCTATGTACAACTCTGGCGGGGAG	Tb927.9.12440_rev	GGGGACCACTTTGTACAAGAAAGCTGGGTTCACGATGATAATGGTCGCATATAATTTTC
3_hypothetical	D24	Tb927.11.1590_for	GGGGACAAGTTTGTACAAAAAAGCAGGCTATGAAGCGCGGGCGGTCG	Tb927.11.1590_rev	GGGGACCACTTTGTACAAGAAAGCTGGGTCTACTTTACTCGCGCCAACACC
3_hypothetical	E1	Tb927.7.1280_for	GGGGACAAGTTTGTACAAAAAAGCAGGCTATGATGGAAAGTATGAACCTTGAACAACC	Tb927.7.1280_rev	GGGGACCACTTTGTACAAGAAAGCTGGGTTCAAAAGAAAATCACCAGTATAGCCACA
3_hypothetical	E2	Tb927.9.9690_for	GGGGACAAGTTTGTACAAAAAAGCAGGCTATGGGGTGCCAGATTTCTGTGG	Tb927.9.9690_rev	GGGGACCACTTTGTACAAGAAAGCTGGGTTCATTCATTACCTTCACCCTCATCGC
3_hypothetical	E3	Tb927.4.1600_for	GGGGACAAGTTTGTACAAAAAAGCAGGCTATGAAAGAAAATGGTGTTTTAGGCGG	Tb927.4.1600_rev	GGGGACCACTTTGTACAAGAAAGCTGGGTTTAGTGCATTTGGCTCCACCCC
3_hypothetical	E4	Tb927.11.16750_for	GGGGACAAGTTTGTACAAAAAAGCAGGCTATGTCTTCAGGTGACAGCAGCG	Tb927.11.16750_rev	GGGGACCACTTTGTACAAGAAAGCTGGGTTCACGATGACTGACCTGGCA
3_hypothetical	E5	Tb927.8.3470_for	GGGGACAAGTTTGTACAAAAAAGCAGGCTATGTCATCTGATCTTTTGCGAGAGTAC	Tb927.8.3470_rev	GGGGACCACTTTGTACAAGAAAGCTGGGTCTCACTTCCAAAATACAATGATTAGAAAGGT
3_hypothetical	E6	Tb927.9.6470_for	GGGGACAAGTTTGTACAAAAAAGCAGGCTATGACACTGGAGGGCGGC	Tb927.9.6470_rev	GGGGACCACTTTGTACAAGAAAGCTGGGTTTAGGCCCCTTCGCCAATCG
3_hypothetical	E7	Tb927.8.2330_for	GGGGACAAGTTTGTACAAAAAAGCAGGCTATGAGCTCTGCTGAGCGGC	Tb927.8.2330_rev	GGGGACCACTTTGTACAAGAAAGCTGGGTCTAATCAAACTCACCATCTGCGTACC
3_hypothetical	E8	Tb927.11.5080_for	GGGGACAAGTTTGTACAAAAAAGCAGGCTATGCAGCCGCATGGTGGA	Tb927.11.5080_rev	GGGGACCACTTTGTACAAGAAAGCTGGGTCAACCGCGCCTCACATCC
3_hypothetical	E9	Tb927.5.730_for	GGGGACAAGTTTGTACAAAAAAGCAGGCTATGTTCAGTGCAGGAGCTAGTGA	Tb927.5.730_rev	GGGGACCACTTTGTACAAGAAAGCTGGGTTTACGATTTGAGAAACTGGTAAGAGCG
3_hypothetical	E10	Tb927.9.7670_for	GGGGACAAGTTTGTACAAAAAAGCAGGCTATGGCGAAGTCCACCCGC	Tb927.9.7670_rev	GGGGACCACTTTGTACAAGAAAGCTGGGTTTACCTCCACTGCACCTTCTTCT
3_hypothetical	E11	Tb927.1.30_for	GGGGACAAGTTTGTACAAAAAAGCAGGCTATGAGGACCAACAAAAAAACACAGAGG	Tb927.1.30_rev	GGGGACCACTTTGTACAAGAAAGCTGGGTCTACACATATCGTCCCCCTATTACCA
3_hypothetical	E12	Tb927.11.5230_for	GGGGACAAGTTTGTACAAAAAAGCAGGCTATGAGTGCTTCTGGAAGTGGAGT	Tb927.11.5230_rev	GGGGACCACTTTGTACAAGAAAGCTGGGTCAGTAGACATGCTCTGAAGGCA
3_hypothetical	E13	Tb927.7.6570_for	GGGGACAAGTTTGTACAAAAAAGCAGGCTATGAATCTAGCGAAGAAAATCATCGTGT	Tb927.7.6570_rev	GGGGACCACTTTGTACAAGAAAGCTGGGTTCAATTAGCAGATTCTGTGCCCG
3_hypothetical	E14	Tb927.11.2350_for	GGGGACAAGTTTGTACAAAAAAGCAGGCTATGTATGCTTCGCATAAAAAGGATATTAAC	Tb927.11.2350_rev	GGGGACCACTTTGTACAAGAAAGCTGGGTCTCACTTTTTCATATCCAGCAGTTCTTGC
3_hypothetical	E15	Tb927.11.13470_for	GGGGACAAGTTTGTACAAAAAAGCAGGCTATGGAACAAGGTCAACCCGC	Tb927.11.13470_rev	GGGGACCACTTTGTACAAGAAAGCTGGGTTCACCGTACTGCCTTTCTGTCT
3_hypothetical	E16	Tb927.6.4270_for	GGGGACAAGTTTGTACAAAAAAGCAGGCTATGAAGCGGTCTTTTTTGTTTCTGAC	Tb927.6.4270_rev	GGGGACCACTTTGTACAAGAAAGCTGGGTTCAATGAACTGCAGCACTACTATTTCC
3_hypothetical	E17	Tb927.8.3570_for	GGGGACAAGTTTGTACAAAAAAGCAGGCTATGACCACGAATCTTGTCCTTCCA	Tb927.8.3570_rev	GGGGACCACTTTGTACAAGAAAGCTGGGTTTACGGTATAAACACACGAGTTGTTTTTC
3_hypothetical	E18	Tb927.6.4250_for	GGGGACAAGTTTGTACAAAAAAGCAGGCTATGGTGGTTTGCTGAAGTGCTTGT	Tb927.6.4250_rev	GGGGACCACTTTGTACAAGAAAGCTGGGTCTACACTTTTTGTGGCACACCGA
3_hypothetical	E19	Tb927.7.1800_for	GGGGACAAGTTTGTACAAAAAAGCAGGCTATGTCGTCTTTAATGTCGGCAAATAATAGT	Tb927.7.1800_rev	GGGGACCACTTTGTACAAGAAAGCTGGGTTTATTTTTTTCGCGTAGTAATGCCCATG
3_hypothetical	E20	Tb927.8.6400_for	GGGGACAAGTTTGTACAAAAAAGCAGGCTATGCGCCACTCCGTGAAGT	Tb927.8.6400_rev	GGGGACCACTTTGTACAAGAAAGCTGGGTTCAATCACTCTTCTTCCCCTGTAGG
3_hypothetical	E21	Tb927.6.3060_for	GGGGACAAGTTTGTACAAAAAAGCAGGCTATGAAAGGCTTGGAAGAGTGGC	Tb927.6.3060_rev	GGGGACCACTTTGTACAAGAAAGCTGGGTTCAATACCGCGGCATCGGT
3_hypothetical	E22	Tb927.3.5200_for	GGGGACAAGTTTGTACAAAAAAGCAGGCTATGGCATGTGATGATTCGACGAA	Tb927.3.5200_rev	GGGGACCACTTTGTACAAGAAAGCTGGGTTTATCGACACCACCCTCCTGTG
3_hypothetical	E23	Tb927.10.8960_for	GGGGACAAGTTTGTACAAAAAAGCAGGCTATGTTGCGTTTTACGGTTGCG	Tb927.10.8960_rev	GGGGACCACTTTGTACAAGAAAGCTGGGTTTATTTTTTGCCTGCTGCCTTGTTC
3_hypothetical	E24	Tb927.11.6650_for	GGGGACAAGTTTGTACAAAAAAGCAGGCTATGGTTAAGCCTGCCGTGTTTC	Tb927.11.6650_rev	GGGGACCACTTTGTACAAGAAAGCTGGGTCAAAGACAACCAGGCGGTTTT
3_hypothetical	F1	Tb927.2.4530_for	GGGGACAAGTTTGTACAAAAAAGCAGGCTATGCAATCTCCGAAGTTTGGTCG	Tb927.2.4530_rev	GGGGACCACTTTGTACAAGAAAGCTGGGTCTAGGAATGACCACCCGACACA
3_hypothetical	F2	Tb927.8.2830_for	GGGGACAAGTTTGTACAAAAAAGCAGGCTATGGAGGCTGTGGTCGCA	Tb927.8.2830_rev	GGGGACCACTTTGTACAAGAAAGCTGGGTCTAGTATTCGGGGCTGGTCCTC
3_hypothetical	F3	Tb927.10.9120_for	GGGGACAAGTTTGTACAAAAAAGCAGGCTATGTCCCGGTACAGTACAGGC	Tb927.10.9120_rev	GGGGACCACTTTGTACAAGAAAGCTGGGTTTAGCTGCAATAGCCGGCAATG
3_hypothetical	F4	Tb927.7.4720_for	GGGGACAAGTTTGTACAAAAAAGCAGGCTATGACTGTGCGTGGCGCC	Tb927.7.4720_rev	GGGGACCACTTTGTACAAGAAAGCTGGGTTTACTCGTTTGCCCGCTCCT
3_hypothetical	F5	Tb927.10.580_for	GGGGACAAGTTTGTACAAAAAAGCAGGCTATGCTTCTACAGTGTGGTTCTTGTAG	Tb927.10.580_rev	GGGGACCACTTTGTACAAGAAAGCTGGGTCTCATTTCTGCAGCGACACGC
3_hypothetical	F6	Tb927.11.1920_for	GGGGACAAGTTTGTACAAAAAAGCAGGCTATGATGATCTTCATTATGTCAATAGAGCTG	Tb927.11.1920_rev	GGGGACCACTTTGTACAAGAAAGCTGGGTTTAAAGCGCAGCGCACCA
3_hypothetical	F7	Tb927.8.4790_for	GGGGACAAGTTTGTACAAAAAAGCAGGCTATGGCAGAGAACAGTCCTACATCT	Tb927.8.4790_rev	GGGGACCACTTTGTACAAGAAAGCTGGGTCTACAACCGAATTTGCATCAAGTCG
3_hypothetical	F8	Tb927.10.3070_for	GGGGACAAGTTTGTACAAAAAAGCAGGCTATGGACGCCGCAGATGCC	Tb927.10.3070_rev	GGGGACCACTTTGTACAAGAAAGCTGGGTTTACTGTGAAGCAAACCTTCTCCTC
3_hypothetical	F9	Tb927.7.5130_for	GGGGACAAGTTTGTACAAAAAAGCAGGCTATGAGCAACCACGGGGGC	Tb927.7.5130_rev	GGGGACCACTTTGTACAAGAAAGCTGGGTTCACAAGGGACACTTTGAGACCC
3_hypothetical	F10	Tb927.11.11620_for	GGGGACAAGTTTGTACAAAAAAGCAGGCTATGACCATTCTCCACAATCCCGG	Tb927.11.11620_rev	GGGGACCACTTTGTACAAGAAAGCTGGGTCTACAACTTTTTCCCTGCCTGCA
3_hypothetical	F11	Tb927.10.8410_for	GGGGACAAGTTTGTACAAAAAAGCAGGCTATGGACCGGAGCGAGACATT	Tb927.10.8410_rev	GGGGACCACTTTGTACAAGAAAGCTGGGTTTAGTCTGCTTTAATGCTATCGACTCG
3_hypothetical	F12	Tb927.8.2700_for	GGGGACAAGTTTGTACAAAAAAGCAGGCTATGATCCGACTGCACCGCA	Tb927.8.2700_rev	GGGGACCACTTTGTACAAGAAAGCTGGGTTCACCGTTCGGTGGCAAGG
3_hypothetical	F13	Tb927.8.6040_for	GGGGACAAGTTTGTACAAAAAAGCAGGCTATGCACCCGCGATTGTCG	Tb927.8.6040_rev	GGGGACCACTTTGTACAAGAAAGCTGGGTCAACCACGAGGTAAAGTGGGG
3_hypothetical	F14	Tb927.7.350_for	GGGGACAAGTTTGTACAAAAAAGCAGGCTATGTCCTTGGCCTCAGTTCCT	Tb927.7.350_rev	GGGGACCACTTTGTACAAGAAAGCTGGGTCTATTGCATGCAAGCACAGGC
3_hypothetical	F15	Tb927.10.15200_for	GGGGACAAGTTTGTACAAAAAAGCAGGCTATGGCAGGCGGATCTACCTC	Tb927.10.15200_rev	GGGGACCACTTTGTACAAGAAAGCTGGGTCTAATCGGAATCGTACATGTCCCTCT
3_hypothetical	F16	Tb927.10.4390_for	GGGGACAAGTTTGTACAAAAAAGCAGGCTATGAATAGCATAAAAGTGTTGCTCGTTTT	Tb927.10.4390_rev	GGGGACCACTTTGTACAAGAAAGCTGGGTTTACGCTAAAAGAAAAAAAGGGATGAGAAA
3_hypothetical	F17	Tb927.5.2630_for	GGGGACAAGTTTGTACAAAAAAGCAGGCTATGGCCTTTTGGGAAAAGATATGGTG	Tb927.5.2630_rev	GGGGACCACTTTGTACAAGAAAGCTGGGTTTAGTGGAGGGCCTCCGC
3_hypothetical	F18	Tb927.7.3860_for	GGGGACAAGTTTGTACAAAAAAGCAGGCTATGCAACAAAAATATCTGGGCAGAGA	Tb927.7.3860_rev	GGGGACCACTTTGTACAAGAAAGCTGGGTTTAGTTGTTCTTTTTAACGACAGCCTTG
3_hypothetical	F19	Tb927.11.7800_for	GGGGACAAGTTTGTACAAAAAAGCAGGCTATGGCGACGGAACAACACC	Tb927.11.7800_rev	GGGGACCACTTTGTACAAGAAAGCTGGGTCTCATACACGACGCAGTCCCC
3_hypothetical	F20	Tb927.10.6550_for	GGGGACAAGTTTGTACAAAAAAGCAGGCTATGCCGCAGCTGCGGGTG	Tb927.10.6550_rev	GGGGACCACTTTGTACAAGAAAGCTGGGTTTAAACTGAATGCCTTATAACAGCCCG
3_hypothetical	F21	Tb927.11.4250_for	GGGGACAAGTTTGTACAAAAAAGCAGGCTATGCAACAACCGATTGGCTTAACT	Tb927.11.4250_rev	GGGGACCACTTTGTACAAGAAAGCTGGGTCTATGTCAAGTAATTGTTCACATGTAGATT
3_hypothetical	F22	Tb927.11.6850_for	GGGGACAAGTTTGTACAAAAAAGCAGGCTATGCCAGCACCAGTTTACCG	Tb927.11.6850_rev	GGGGACCACTTTGTACAAGAAAGCTGGGTCTATGTTCCAAGTGCCTTTTTTGCA
3_hypothetical	F23	Tb927.8.1320_for	GGGGACAAGTTTGTACAAAAAAGCAGGCTATGACGTCCAAAGTTGAGGAGC	Tb927.8.1320_rev	GGGGACCACTTTGTACAAGAAAGCTGGGTTCATTTTTCAGCGTTTTTTTCGCCT
3_hypothetical	F24	Tb927.11.3760_for	GGGGACAAGTTTGTACAAAAAAGCAGGCTATGGAAACGAAAGAGAGTTGCAGG	Tb927.11.3760_rev	GGGGACCACTTTGTACAAGAAAGCTGGGTTCAGTCATCATCATTGGCATGTAAACC
3_hypothetical	G1	Tb927.6.2490_for	GGGGACAAGTTTGTACAAAAAAGCAGGCTATGCGGCGGCAAATTCTTGG	Tb927.6.2490_rev	GGGGACCACTTTGTACAAGAAAGCTGGGTTTACGACAACTGTGGGAACGC
3_hypothetical	G2	Tb927.10.8660_for	GGGGACAAGTTTGTACAAAAAAGCAGGCTATGGATAGGGCTCGGAACTTATG	Tb927.10.8660_rev	GGGGACCACTTTGTACAAGAAAGCTGGGTTTACGACCGTCGGAAAGCCA
3_hypothetical	G3	Tb927.11.7700_for	GGGGACAAGTTTGTACAAAAAAGCAGGCTATGTCCCGCGCCACCACA	Tb927.11.7700_rev	GGGGACCACTTTGTACAAGAAAGCTGGGTTTAACGAAGTGTAGGTAAGGGACCA
3_hypothetical	G4	Tb927.11.3520_for	GGGGACAAGTTTGTACAAAAAAGCAGGCTATGAAGGGGAATAAAGGTGCGAAC	Tb927.11.3520_rev	GGGGACCACTTTGTACAAGAAAGCTGGGTTTAGATGAGCCCAAACCACTCACC
3_hypothetical	G5	Tb927.5.720_for	GGGGACAAGTTTGTACAAAAAAGCAGGCTATGGCGTCTGTGTCTAGGCG	Tb927.5.720_rev	GGGGACCACTTTGTACAAGAAAGCTGGGTCTCAATCAATCTCAAAAGCCCCTCC
3_hypothetical	G6	Tb927.10.9090_for	GGGGACAAGTTTGTACAAAAAAGCAGGCTATGAGCTGGAGGGTTTCCTGC	Tb927.10.9090_rev	GGGGACCACTTTGTACAAGAAAGCTGGGTCTCACACTTTTGATTTAACGTACTCAACAAA
3_hypothetical	G7	Tb927.11.2250_for	GGGGACAAGTTTGTACAAAAAAGCAGGCTATGTACAATAACGACTCGGCGG	Tb927.11.2250_rev	GGGGACCACTTTGTACAAGAAAGCTGGGTTTAGGAAGGCCCATCTGCCTT
3_hypothetical	G8	Tb927.11.13150_for	GGGGACAAGTTTGTACAAAAAAGCAGGCTATGTTTGACGATGCATCCCGTT	Tb927.11.13150_rev	GGGGACCACTTTGTACAAGAAAGCTGGGTTTAGTTGTACACCCGTTGGCG
3_hypothetical	G9	Tb927.6.4040_for	GGGGACAAGTTTGTACAAAAAAGCAGGCTATGTTACGGAACACAGATGTTCGAC	Tb927.6.4040_rev	GGGGACCACTTTGTACAAGAAAGCTGGGTCAATTAGTCGAGACCTCCACATGA
3_hypothetical	G10	Tb927.5.4090_for	GGGGACAAGTTTGTACAAAAAAGCAGGCTATGGAAGAGGGCCATGCCA	Tb927.5.4090_rev	GGGGACCACTTTGTACAAGAAAGCTGGGTTTACAAATGAATCACGGCGTCGA
3_hypothetical	G11	Tb927.8.3880_for	GGGGACAAGTTTGTACAAAAAAGCAGGCTATGTGCGCTGGGCGGGAT	Tb927.8.3880_rev	GGGGACCACTTTGTACAAGAAAGCTGGGTCTATTTCATAATTCTGCACCACGGATATCC
3_hypothetical	G12	Tb927.11.12370_for	GGGGACAAGTTTGTACAAAAAAGCAGGCTATGTCAGCCGCTGGCATTG	Tb927.11.12370_rev	GGGGACCACTTTGTACAAGAAAGCTGGGTCTAACGTAGCTCGGATATGGGACG
3_hypothetical	G13	Tb927.7.1290_for	GGGGACAAGTTTGTACAAAAAAGCAGGCTATGTTTGGACGTGGTTGCATGC	Tb927.7.1290_rev	GGGGACCACTTTGTACAAGAAAGCTGGGTCACTTGTGCTTCCTCTCGTGT
3_hypothetical	G14	Tb927.11.10440_for	GGGGACAAGTTTGTACAAAAAAGCAGGCTATGAGACAAGCGTCTGCGGG	Tb927.11.10440_rev	GGGGACCACTTTGTACAAGAAAGCTGGGTTCAGTCATCCCAGATATCGGGGT
3_hypothetical	G15	Tb927.11.11630_for	GGGGACAAGTTTGTACAAAAAAGCAGGCTATGTTGCGTGGCACTCGTG	Tb927.11.11630_rev	GGGGACCACTTTGTACAAGAAAGCTGGGTTTATTCCGAAAGAAACGCCTTGATATTTAT
3_hypothetical	G16	Tb927.9.1790_for	GGGGACAAGTTTGTACAAAAAAGCAGGCTATGGGTCGGGGGTTCGTG	Tb927.9.1790_rev	GGGGACCACTTTGTACAAGAAAGCTGGGTCTCACTTGCTTTGTGGAAACGAAATATC
3_hypothetical	G17	Tb927.10.4760_for	GGGGACAAGTTTGTACAAAAAAGCAGGCTATGACGCAGAACATTCTTTTGGACC	Tb927.10.4760_rev	GGGGACCACTTTGTACAAGAAAGCTGGGTCTATTTACCTTCAAGGAGTAAACGGGG
3_hypothetical	G18	Tb927.8.2510_for	GGGGACAAGTTTGTACAAAAAAGCAGGCTATGAGCATTTCATGTTCCCTGGC	Tb927.8.2510_rev	GGGGACCACTTTGTACAAGAAAGCTGGGTCTACCAAAAAAATATGAGGAAGTCGCG
3_hypothetical	G19	Tb927.10.1750_for	GGGGACAAGTTTGTACAAAAAAGCAGGCTATGCGCGCGGTGCGGGCC	Tb927.10.1750_rev	GGGGACCACTTTGTACAAGAAAGCTGGGTTCACTTCGTCCGCTTTGATAAGTAG
3_hypothetical	G20	Tb927.10.7670_for	GGGGACAAGTTTGTACAAAAAAGCAGGCTATGCGCGGGAAAAGGAAATGCC	Tb927.10.7670_rev	GGGGACCACTTTGTACAAGAAAGCTGGGTTTAATTGGCCCTTCGCGTCTTC
3_hypothetical	G21	Tb927.4.1910_for	GGGGACAAGTTTGTACAAAAAAGCAGGCTATGTCTTCCTTTAACATGCAAGCGT	Tb927.4.1910_rev	GGGGACCACTTTGTACAAGAAAGCTGGGTTTACGAAGTCTGTCCCTCTTCATTTACT
3_hypothetical	G22	Tb927.4.2620_for	GGGGACAAGTTTGTACAAAAAAGCAGGCTATGCGCGGCGTGGTGTAC	Tb927.4.2620_rev	GGGGACCACTTTGTACAAGAAAGCTGGGTCTAAGTGGCCGTGGGATAAATGTT
3_hypothetical	G23	Tb927.1.690_for	GGGGACAAGTTTGTACAAAAAAGCAGGCTATGTACCCTTCTTTTGTAAGTGAGTTGTC	Tb927.1.690_rev	GGGGACCACTTTGTACAAGAAAGCTGGGTTCATTTCACAGAGCAGATGAAACAATAGA
3_hypothetical	G24	Tb927.11.5830_for	GGGGACAAGTTTGTACAAAAAAGCAGGCTATGGACGGTGTTGACGACTCC	Tb927.11.5830_rev	GGGGACCACTTTGTACAAGAAAGCTGGGTTCAGTTGGAACCCGCTCCTG
3_hypothetical	H1	Tb927.11.4930_for	GGGGACAAGTTTGTACAAAAAAGCAGGCTATGGTCGAAAGAACAGCGGATC	Tb927.11.4930_rev	GGGGACCACTTTGTACAAGAAAGCTGGGTTCATGCATCATCATCATCATCATCGT
3_hypothetical	H2	Tb927.11.6490_for	GGGGACAAGTTTGTACAAAAAAGCAGGCTATGTCTGCTAATGCTTGGCGG	Tb927.11.6490_rev	GGGGACCACTTTGTACAAGAAAGCTGGGTCTAACCCTTCGTGTCGCCCT
3_hypothetical	H3	Tb927.8.4760_for	GGGGACAAGTTTGTACAAAAAAGCAGGCTATGAAGGACAAAAATAAGTGCACTTTTAGG	Tb927.8.4760_rev	GGGGACCACTTTGTACAAGAAAGCTGGGTCTACCTTTGTCCTCCTTTTAAATACCATTC
3_hypothetical	H4	Tb927.10.1410_for	GGGGACAAGTTTGTACAAAAAAGCAGGCTATGTTTCGGCGATGTGGTTTGC	Tb927.10.1410_rev	GGGGACCACTTTGTACAAGAAAGCTGGGTTCATACGCTTTGGTGTCCTCTTGT
3_hypothetical	H5	Tb927.10.8010_for	GGGGACAAGTTTGTACAAAAAAGCAGGCTATGGCAGCCGAGCAGCCA	Tb927.10.8010_rev	GGGGACCACTTTGTACAAGAAAGCTGGGTCTACAGGTTCGTCGTCCATGGT
3_hypothetical	H6	Tb927.3.880_for	GGGGACAAGTTTGTACAAAAAAGCAGGCTATGTTAGGGTTGACAATCAGTCGGT	Tb927.3.880_rev	GGGGACCACTTTGTACAAGAAAGCTGGGTTCATGATAAAGAAACCAACGATTGAGGA
3_hypothetical	H7	Tb927.6.2750_for	GGGGACAAGTTTGTACAAAAAAGCAGGCTATGGGCGAGACTTTAGGCAGC	Tb927.6.2750_rev	GGGGACCACTTTGTACAAGAAAGCTGGGTTCAACGTCGTTTCCCTGCAC
3_hypothetical	H8	Tb927.9.1660_for	GGGGACAAGTTTGTACAAAAAAGCAGGCTATGACACTTGATCTCGTTGGTAAACTT	Tb927.9.1660_rev	GGGGACCACTTTGTACAAGAAAGCTGGGTCTCACTCAGATCGGGGGCG
3_hypothetical	H9	Tb927.10.1430_for	GGGGACAAGTTTGTACAAAAAAGCAGGCTATGGTGGAATATAAGAGCGCCGC	Tb927.10.1430_rev	GGGGACCACTTTGTACAAGAAAGCTGGGTTCAGGGCGCCTTCTTCTCG
3_hypothetical	H10	Tb927.1.1390_for	GGGGACAAGTTTGTACAAAAAAGCAGGCTATGCTGGTTCGAACATGGATGTT	Tb927.1.1390_rev	GGGGACCACTTTGTACAAGAAAGCTGGGTCTATGCCACTTGCTGCCCATC

3_hypothetical	H11	Tb927.11.770_for	GGGGACAAGTTTGTACAAAAAAGCAGGCTATGGCGTCAGAGAAGTCACTACA	Tb927.11.770_rev	GGGGACCACTTTGTACAAGAAAGCTGGGTCTACAGGCTTAGCCACGGGT
3_hypothetical	H12	Tb927.10.5000_for	GGGGACAAGTTTGTACAAAAAAGCAGGCTATGACAGCACTATCGAATGAAGAAATGT	Tb927.10.5000_rev	GGGGACCACTTTGTACAAGAAAGCTGGGTTCACATACTCGCATCTTGATGCAA
3_hypothetical	H13	Tb927.7.3800_for	GGGGACAAGTTTGTACAAAAAAGCAGGCTATGAAGGCGACTGAAGAAGAAGC	Tb927.7.3800_rev	GGGGACCACTTTGTACAAGAAAGCTGGGTCTACAACTCCCACCACAGGGA
3_hypothetical	H14	Tb927.10.4380_for	GGGGACAAGTTTGTACAAAAAAGCAGGCTATGAATAACCTGAAAGTGTTGCTTGTGT	Tb927.10.4380_rev	GGGGACCACTTTGTACAAGAAAGCTGGGTTTACGCTAAAAGAAAAAAAGGGATGAGAAA
3_hypothetical	H15	Tb927.9.7100_for	GGGGACAAGTTTGTACAAAAAAGCAGGCTATGGCATCCAACGGTTTGGC	Tb927.9.7100_rev	GGGGACCACTTTGTACAAGAAAGCTGGGTCTATGCACGGAATTCACCTACGAA
3_hypothetical	H16	Tb927.7.1490_for	GGGGACAAGTTTGTACAAAAAAGCAGGCTATGAGACCGTGTGCGCCT	Tb927.7.1490_rev	GGGGACCACTTTGTACAAGAAAGCTGGGTTTACAGCTCCTCTTTGATCTCCTTCG
3_hypothetical	H17	Tb927.6.4310_for	GGGGACAAGTTTGTACAAAAAAGCAGGCTATGATGAATCACTCACCCCATGAAC	Tb927.6.4310_rev	GGGGACCACTTTGTACAAGAAAGCTGGGTCTACTGAGGCGGAAGGCTTAGG
3_hypothetical	H18	Tb927.5.292b_for	GGGGACAAGTTTGTACAAAAAAGCAGGCTATGCTACCTGTGATTTTCGTGTACG	Tb927.5.292b_rev	GGGGACCACTTTGTACAAGAAAGCTGGGTCTAAACCATTGACAAATATCCCAAGACTAA
3_hypothetical	H19	Tb927.1.2970_for	GGGGACAAGTTTGTACAAAAAAGCAGGCTATGAAACATGTTTCTGAAATGTTCATCCC	Tb927.1.2970_rev	GGGGACCACTTTGTACAAGAAAGCTGGGTTCAAATACGGTTGGGCTTCTGTTC
3_hypothetical	H20	Tb927.6.3680_for	GGGGACAAGTTTGTACAAAAAAGCAGGCTATGGAGCATCTACCAACTATCGACT	Tb927.6.3680_rev	GGGGACCACTTTGTACAAGAAAGCTGGGTTTATTCTTGCTTTTTATTTGCGTCTTCCC
3_hypothetical	H21	Tb927.11.870_for	GGGGACAAGTTTGTACAAAAAAGCAGGCTATGGTGGTTAGTCTGAGGAGGTTT	Tb927.11.870_rev	GGGGACCACTTTGTACAAGAAAGCTGGGTTCATTGAGTGATTCGTTCCTGGGG
3_hypothetical	H22	Tb927.10.6120_for	GGGGACAAGTTTGTACAAAAAAGCAGGCTATGGCCTCTGTGGAACCTGAT	Tb927.10.6120_rev	GGGGACCACTTTGTACAAGAAAGCTGGGTCTACAACTCGAACTTCTCGTCACC
3_hypothetical	H23	Tb927.9.11640_for	GGGGACAAGTTTGTACAAAAAAGCAGGCTATGAGTTCCCTTCCTCCGGAC	Tb927.9.11640_rev	GGGGACCACTTTGTACAAGAAAGCTGGGTTCATGGTGACATAAAAAAGGACGGTAA
3_hypothetical	H24	Tb927.3.1160_for	GGGGACAAGTTTGTACAAAAAAGCAGGCTATGTTTGCCCCATTCTTTCGTTCA	Tb927.3.1160_rev	GGGGACCACTTTGTACAAGAAAGCTGGGTTTAATCCCCTGTGTAACGCGGC
3_hypothetical	I1	Tb927.4.5440_for	GGGGACAAGTTTGTACAAAAAAGCAGGCTATGTCACATGTGTGCGATGTCTT	Tb927.4.5440_rev	GGGGACCACTTTGTACAAGAAAGCTGGGTTCACTGTCGTGGCGTGCG
3_hypothetical	I2	Tb927.8.7280_for	GGGGACAAGTTTGTACAAAAAAGCAGGCTATGATTGTTCCATCGTTGAACGGC	Tb927.8.7280_rev	GGGGACCACTTTGTACAAGAAAGCTGGGTCTACTGAGGCTTACACCGCAGA
3_hypothetical	I3	Tb927.4.1710_for	GGGGACAAGTTTGTACAAAAAAGCAGGCTATGGCTATGCGAGACGTCATTG	Tb927.4.1710_rev	GGGGACCACTTTGTACAAGAAAGCTGGGTTCATCGCGGACGGGGCTT
3_hypothetical	I4	Tb927.11.7470_for	GGGGACAAGTTTGTACAAAAAAGCAGGCTATGTCATTTGTACCCGCTTCACAA	Tb927.11.7470_rev	GGGGACCACTTTGTACAAGAAAGCTGGGTCTACCACTCACGCGTTGGTAAG
3_hypothetical	I5	Tb927.9.13120_for	GGGGACAAGTTTGTACAAAAAAGCAGGCTATGACAGACTTGGACGCGACC	Tb927.9.13120_rev	GGGGACCACTTTGTACAAGAAAGCTGGGTTTACTCTGAAAGTGAGACATTTGCCTG
3_hypothetical	I6	Tb927.9.15240_for	GGGGACAAGTTTGTACAAAAAAGCAGGCTATGCTTGCTGCTACCCGAAGT	Tb927.9.15240_rev	GGGGACCACTTTGTACAAGAAAGCTGGGTCTACTCTTTCCTATATCCATTGCAGCC
3_hypothetical	I7	Tb927.8.6280_for	GGGGACAAGTTTGTACAAAAAAGCAGGCTATGAGCGGAAAACATCTTTTGCATC	Tb927.8.6280_rev	GGGGACCACTTTGTACAAGAAAGCTGGGTTCACATCTCTCCGCACAGAGTG
3_hypothetical	I8	Tb927.4.2840_for	GGGGACAAGTTTGTACAAAAAAGCAGGCTATGCGTTGCCATGTGATGGG	Tb927.4.2840_rev	GGGGACCACTTTGTACAAGAAAGCTGGGTCTAATCTTCGTAGCACTCACGCAG
3_hypothetical	I9	Tb927.11.4260_for	GGGGACAAGTTTGTACAAAAAAGCAGGCTATGAACCCACTTTTTGCCGGC	Tb927.11.4260_rev	GGGGACCACTTTGTACAAGAAAGCTGGGTCTACACCTTTCCGTAGCGAGC
3_hypothetical	I10	Tb927.10.11120_for	GGGGACAAGTTTGTACAAAAAAGCAGGCTATGCAGGCCCAACACTATCATCT	Tb927.10.11120_rev	GGGGACCACTTTGTACAAGAAAGCTGGGTTCACGATAAAGGTGGAAGACCATTTTC
3_hypothetical	I11	Tb927.6.690_for	GGGGACAAGTTTGTACAAAAAAGCAGGCTATGAACTGTATCGAGTCAAAGTGTGC	Tb927.6.690_rev	GGGGACCACTTTGTACAAGAAAGCTGGGTTTAACAGAAGAGATACGACTCCTTTGGA
3_hypothetical	I12	Tb927.10.2080_for	GGGGACAAGTTTGTACAAAAAAGCAGGCTATGCTGTGGATTCAAATATGTACATTTGTA	Tb927.10.2080_rev	GGGGACCACTTTGTACAAGAAAGCTGGGTCTATTGCTTATGATTTTTACTCTTACTTC
3_hypothetical	I13	Tb927.7.630_for	GGGGACAAGTTTGTACAAAAAAGCAGGCTATGCGCGCAGGCTGCGTT	Tb927.7.630_rev	GGGGACCACTTTGTACAAGAAAGCTGGGTCTACACTTGATACTTAGATTTGGGGCG
3_hypothetical	I14	Tb927.10.9450_for	GGGGACAAGTTTGTACAAAAAAGCAGGCTATGAGCACCTTCGCGGTACT	Tb927.10.9450_rev	GGGGACCACTTTGTACAAGAAAGCTGGGTTTATTTTTGCGTCTCCCCTTCTTTTGA
3_hypothetical	I15	Tb927.10.12030_for	GGGGACAAGTTTGTACAAAAAAGCAGGCTATGTTAACTATCCGTGTCGGTAACCT	Tb927.10.12030_rev	GGGGACCACTTTGTACAAGAAAGCTGGGTCTATGAAGAAATGAGCTCCGTCGC
3_hypothetical	I16	Tb927.6.2120_for	GGGGACAAGTTTGTACAAAAAAGCAGGCTATGACAGGTGCGGATAGGTTTAACA	Tb927.6.2120_rev	GGGGACCACTTTGTACAAGAAAGCTGGGTTCATCGCTTCCTTCTCAGCGT
3_hypothetical	I17	Tb927.9.4290_for	GGGGACAAGTTTGTACAAAAAAGCAGGCTATGGCACACTGTTGCTTTCTCA	Tb927.9.4290_rev	GGGGACCACTTTGTACAAGAAAGCTGGGTTTATTTATCGAACCCCAACTCTCTAATGAG
3_hypothetical	I18	Tb927.4.700_for	GGGGACAAGTTTGTACAAAAAAGCAGGCTATGATGAGGCGGCAAACTTATGTT	Tb927.4.700_rev	GGGGACCACTTTGTACAAGAAAGCTGGGTCTACGTAAACGGGCTAACGGC
3_hypothetical	I19	Tb927.11.5180_for	GGGGACAAGTTTGTACAAAAAAGCAGGCTATGCAAAGGCACAGTTCGCA	Tb927.11.5180_rev	GGGGACCACTTTGTACAAGAAAGCTGGGTCTATAGATCACTAGCAGCTTCTTCAGC
3_hypothetical	I21	Tb927.10.7750_for	GGGGACAAGTTTGTACAAAAAAGCAGGCTATGAACAAGGAAAAGGTGGATCGC	Tb927.10.7750_rev	GGGGACCACTTTGTACAAGAAAGCTGGGTTCATTTCCCCTTTTGGAAGAACGATC
3_hypothetical	I22	Tb927.9.15000_for	GGGGACAAGTTTGTACAAAAAAGCAGGCTATGAAGGGCGTTGGCAGTAATAATT	Tb927.9.15000_rev	GGGGACCACTTTGTACAAGAAAGCTGGGTTCATTTCTTCACTTGCTTCAGTAATCCG
3_hypothetical	I23	Tb927.3.1080_for	GGGGACAAGTTTGTACAAAAAAGCAGGCTATGGCACAGGAGGGATTGAAAGA	Tb927.3.1080_rev	GGGGACCACTTTGTACAAGAAAGCTGGGTTCATTCTACCGTTGTAGTTTTCTGCTT
3_hypothetical	I24	Tb927.8.2040_for	GGGGACAAGTTTGTACAAAAAAGCAGGCTATGGCGGCTCTCCACGAG	Tb927.8.2040_rev	GGGGACCACTTTGTACAAGAAAGCTGGGTCTACTGAGGTCGGTTATAGTGGTGG
3_hypothetical	J1	Tb927.2.4850_for	GGGGACAAGTTTGTACAAAAAAGCAGGCTATGCTCGGCTCAGGAAAAAGC	Tb927.2.4850_rev	GGGGACCACTTTGTACAAGAAAGCTGGGTTTATTCGCATTCCTCCTGCTTACAG
3_hypothetical	J2	Tb927.10.15510_for	GGGGACAAGTTTGTACAAAAAAGCAGGCTATGAACTTGGGTCAAGTGCCCC	Tb927.10.15510_rev	GGGGACCACTTTGTACAAGAAAGCTGGGTTTAAACGCTGCTTGAACGCCA
3_hypothetical	J3	Tb927.1.3310_for	GGGGACAAGTTTGTACAAAAAAGCAGGCTATGGAAGAAGTACTCGATAACAAAGAGAC	Tb927.1.3310_rev	GGGGACCACTTTGTACAAGAAAGCTGGGTTTACGCCATTGGTGTGTTTTCCT
3_hypothetical	J4	Tb927.11.13480_for	GGGGACAAGTTTGTACAAAAAAGCAGGCTATGGACGAGAACACCACATCCT	Tb927.11.13480_rev	GGGGACCACTTTGTACAAGAAAGCTGGGTTCAAGGGGTCTCGTCATCGC
3_hypothetical	J5	Tb927.3.2480_for	GGGGACAAGTTTGTACAAAAAAGCAGGCTATGCTCCGGTGTATACAATGTAATGC	Tb927.3.2480_rev	GGGGACCACTTTGTACAAGAAAGCTGGGTTCACACCAGAAGTTGTGGACTCC
3_hypothetical	J6	Tb927.5.1620_for	GGGGACAAGTTTGTACAAAAAAGCAGGCTATGGAAAATAACGATACGACTCCATCC	Tb927.5.1620_rev	GGGGACCACTTTGTACAAGAAAGCTGGGTTTAGTCCACATGTAGATGAGGATCCAC
3_hypothetical	J7	Tb927.10.1240_for	GGGGACAAGTTTGTACAAAAAAGCAGGCTATGCGCATGTCGTTAACAACACG	Tb927.10.1240_rev	GGGGACCACTTTGTACAAGAAAGCTGGGTTTATTTCCGCAACCTCTTGGACTC
3_hypothetical	J8	Tb927.11.14770_for	GGGGACAAGTTTGTACAAAAAAGCAGGCTATGAGTACTAAATCCTCAATTTCACCTAGT	Tb927.11.14770_rev	GGGGACCACTTTGTACAAGAAAGCTGGGTTTACATAACACCACACGTTTGCGC
3_hypothetical	J9	Tb927.11.14010_for	GGGGACAAGTTTGTACAAAAAAGCAGGCTATGCTGCTGAAAAAGTTGCCGG	Tb927.11.14010_rev	GGGGACCACTTTGTACAAGAAAGCTGGGTTTAAACGGCACTCTTACGGGGG
3_hypothetical	J10	Tb927.9.3390_for	GGGGACAAGTTTGTACAAAAAAGCAGGCTATGCCGACCGGTGCCGCC	Tb927.9.3390_rev	GGGGACCACTTTGTACAAGAAAGCTGGGTCTATATGGGAACGAAGAGCTCCTTTG
3_hypothetical	J11	Tb927.4.920_for	GGGGACAAGTTTGTACAAAAAAGCAGGCTATGACCACAAGCGACGAATTACC	Tb927.4.920_rev	GGGGACCACTTTGTACAAGAAAGCTGGGTTCACTGCTCAAAGCGTACAGAATAG
3_hypothetical	J12	Tb927.8.6050_for	GGGGACAAGTTTGTACAAAAAAGCAGGCTATGCCTCCTAAGAGACCACCCC	Tb927.8.6050_rev	GGGGACCACTTTGTACAAGAAAGCTGGGTTCATTTCTTCGCCGGAAGGTTG
3_hypothetical	J13	Tb927.9.10520_for	GGGGACAAGTTTGTACAAAAAAGCAGGCTATGAAACGAACAGCTTTTCACTCCA	Tb927.9.10520_rev	GGGGACCACTTTGTACAAGAAAGCTGGGTTTAAATAAGTCGCTTTGACTTGAGATGCT
3_hypothetical	J14	Tb927.9.9080_for	GGGGACAAGTTTGTACAAAAAAGCAGGCTATGTCGCTACTCAACACTACTCTTCA	Tb927.9.9080_rev	GGGGACCACTTTGTACAAGAAAGCTGGGTTTAAGCATGAAAAATGTAGTGCATACCTCT
3_hypothetical	J15	Tb927.2.4650_for	GGGGACAAGTTTGTACAAAAAAGCAGGCTATGGCGATTCGGGCAAAGATG	Tb927.2.4650_rev	GGGGACCACTTTGTACAAGAAAGCTGGGTTCAACCACCCCATAAGGAAGATTCC
3_hypothetical	J16	Tb927.11.12590_for	GGGGACAAGTTTGTACAAAAAAGCAGGCTATGGCCCAAAGCTTTGATGACTG	Tb927.11.12590_rev	GGGGACCACTTTGTACAAGAAAGCTGGGTTTAAGTTGTGCCAAGCCGGTG
3_hypothetical	J17	Tb927.10.300_for	GGGGACAAGTTTGTACAAAAAAGCAGGCTATGGAGCACGTAGTAGCCAGG	Tb927.10.300_rev	GGGGACCACTTTGTACAAGAAAGCTGGGTTTAAGCTTTGTCTTTGGTATCGGAAGAA
3_hypothetical	J18	Tb927.6.3470_for	GGGGACAAGTTTGTACAAAAAAGCAGGCTATGTTTCTCAACAATAACATGGTTGACG	Tb927.6.3470_rev	GGGGACCACTTTGTACAAGAAAGCTGGGTCTAAACAGCGAGCCTCTGCG
3_hypothetical	J19	Tb927.5.1590_for	GGGGACAAGTTTGTACAAAAAAGCAGGCTATGGTGATAAAGAAGGATGTTCCTTTCAA	Tb927.5.1590_rev	GGGGACCACTTTGTACAAGAAAGCTGGGTTTAACTCTCATTGTCTCCTCCTGACA
3_hypothetical	J20	Tb927.5.3100_for	GGGGACAAGTTTGTACAAAAAAGCAGGCTATGATTAACAAAGAAAACAGTGGGCG	Tb927.5.3100_rev	GGGGACCACTTTGTACAAGAAAGCTGGGTTTATTCCCCCTAATCTCAAAATCCGG
3_hypothetical	J21	Tb927.5.2990_for	GGGGACAAGTTTGTACAAAAAAGCAGGCTATGTCAAGGTCGCGAAATATCGTT	Tb927.5.2990_rev	GGGGACCACTTTGTACAAGAAAGCTGGGTTCATGCCCTCTGTCGCGC
3_hypothetical	J22	Tb927.9.8190_for	GGGGACAAGTTTGTACAAAAAAGCAGGCTATGTCACATCTCTTGCGCTCTCG	Tb927.9.8190_rev	GGGGACCACTTTGTACAAGAAAGCTGGGTCTACAGGCAATACTTCCGCTCAG
3_hypothetical	J23	Tb927.6.2820_for	GGGGACAAGTTTGTACAAAAAAGCAGGCTATGACTCACAGGACGGCGG	Tb927.6.2820_rev	GGGGACCACTTTGTACAAGAAAGCTGGGTTCATGCTAACTGCCCAGTTAGAAGG
3_hypothetical	J24	Tb927.7.6890_for	GGGGACAAGTTTGTACAAAAAAGCAGGCTATGTCCAAGTACGCTAACCCGG	Tb927.7.6890_rev	GGGGACCACTTTGTACAAGAAAGCTGGGTTCAGTCAAACCTTGCGGCATC
3_hypothetical	K1	Tb927.10.8030_for	GGGGACAAGTTTGTACAAAAAAGCAGGCTATGTTCCGCCGACTTTCTTCAAG	Tb927.10.8030_rev	GGGGACCACTTTGTACAAGAAAGCTGGGTTCAAACGCCATATTTCGCCTCC
3_hypothetical	K2	Tb927.3.4530_for	GGGGACAAGTTTGTACAAAAAAGCAGGCTATGTGTCGCGTCGTTGATGC	Tb927.3.4530_rev	GGGGACCACTTTGTACAAGAAAGCTGGGTTCAACCAGATATAACGACGGTAACGT
3_hypothetical	K3	Tb927.10.8630_for	GGGGACAAGTTTGTACAAAAAAGCAGGCTATGATGAATTCACATAACGCGATCGT	Tb927.10.8630_rev	GGGGACCACTTTGTACAAGAAAGCTGGGTCTACATCACACTGCAATTGAGGCC
3_hypothetical	K4	Tb927.5.480_for	GGGGACAAGTTTGTACAAAAAAGCAGGCTATGCATGCTGCTGGACAACG	Tb927.5.480_rev	GGGGACCACTTTGTACAAGAAAGCTGGGTTCACCTCGTAATACTCCACATGAGT
3_hypothetical	K5	Tb927.5.3680_for	GGGGACAAGTTTGTACAAAAAAGCAGGCTATGGCGCCGCCGATATCG	Tb927.5.3680_rev	GGGGACCACTTTGTACAAGAAAGCTGGGTTCAGTTGAGAACGTTTTGTGTAGCC
3_hypothetical	K6	Tb927.6.3700_for	GGGGACAAGTTTGTACAAAAAAGCAGGCTATGTTGGAGGGAAACGTGTTGG	Tb927.6.3700_rev	GGGGACCACTTTGTACAAGAAAGCTGGGTTCAGGGGAAAAGTTTCAAATTCCCAG
3_hypothetical	K7	Tb927.9.3490_for	GGGGACAAGTTTGTACAAAAAAGCAGGCTATGGGAAGTTGTTGTGCGAAGAG	Tb927.9.3490_rev	GGGGACCACTTTGTACAAGAAAGCTGGGTTCATTTACCACGGTTCCGAAGC
3_hypothetical	K8	Tb927.7.3870_for	GGGGACAAGTTTGTACAAAAAAGCAGGCTATGCTCATTAATTTTTTAGTAATCTTGATG	Tb927.7.3870_rev	GGGGACCACTTTGTACAAGAAAGCTGGGTTCAAAGCTCATCAAAGTCTACTTTCGC
3_hypothetical	K9	Tb927.1.890_for	GGGGACAAGTTTGTACAAAAAAGCAGGCTATGAATCGGACAGTTGCAATCTGT	Tb927.1.890_rev	GGGGACCACTTTGTACAAGAAAGCTGGGTCTACTTCCTTTCGCGTGTACGC
3_hypothetical	K10	Tb927.8.5190_for	GGGGACAAGTTTGTACAAAAAAGCAGGCTATGCCTGTCTGTGTTCTAGTGCC	Tb927.8.5190_rev	GGGGACCACTTTGTACAAGAAAGCTGGGTTTAGCCATTAACCGCAATACGTTCC
3_hypothetical	K11	Tb927.7.5810_for	GGGGACAAGTTTGTACAAAAAAGCAGGCTATGTTGGCGCTTCGTGCTG	Tb927.7.5810_rev	GGGGACCACTTTGTACAAGAAAGCTGGGTTTATGCTCCGTGCCGCAGG
3_hypothetical	K12	Tb927.11.6290_for	GGGGACAAGTTTGTACAAAAAAGCAGGCTATGGTCGGCCCCCGCATC	Tb927.11.6290_rev	GGGGACCACTTTGTACAAGAAAGCTGGGTTCACACTATGAACTTCATACACCTAGTTGC
3_hypothetical	K13	Tb927.9.9370_for	GGGGACAAGTTTGTACAAAAAAGCAGGCTATGGTCGCAGCAGCTGCT	Tb927.9.9370_rev	GGGGACCACTTTGTACAAGAAAGCTGGGTTCATGACCGAATTCGCGCC
3_hypothetical	K14	Tb927.8.1000_for	GGGGACAAGTTTGTACAAAAAAGCAGGCTATGAGGCAACGACAGTTGCAG	Tb927.8.1000_rev	GGGGACCACTTTGTACAAGAAAGCTGGGTCTACCGTCTCCCACGGTTTGG
3_hypothetical	K15	Tb927.11.4360_for	GGGGACAAGTTTGTACAAAAAAGCAGGCTATGCCGAGCGGAGCAAAG	Tb927.11.4360_rev	GGGGACCACTTTGTACAAGAAAGCTGGGTTTACTGACCACTATCCTCTTCTTCGC
3_hypothetical	K16	Tb927.8.5280_for	GGGGACAAGTTTGTACAAAAAAGCAGGCTATGTTACGGTGCGCACGC	Tb927.8.5280_rev	GGGGACCACTTTGTACAAGAAAGCTGGGTCTACTTTCCATCATAATGCACCGACC
3_hypothetical	K17	Tb927.10.12390_for	GGGGACAAGTTTGTACAAAAAAGCAGGCTATGGAAGAACGTCAGCGGC	Tb927.10.12390_rev	GGGGACCACTTTGTACAAGAAAGCTGGGTTTATACTAACGGCATAGCATCGTCCG
3_hypothetical	K18	Tb927.4.4920_for	GGGGACAAGTTTGTACAAAAAAGCAGGCTATGGATTCTTCATCAAGGAGTTTGAACC	Tb927.4.4920_rev	GGGGACCACTTTGTACAAGAAAGCTGGGTTCACCTAAAAGAACTAAACGGTTCAGC
3_hypothetical	K19	Tb927.11.2020_for	GGGGACAAGTTTGTACAAAAAAGCAGGCTATGACTAGTGAGTGCGTTACCGT	Tb927.11.2020_rev	GGGGACCACTTTGTACAAGAAAGCTGGGTCTACTGAAGATTACTGCGGTGGC
3_hypothetical	K20	Tb927.3.3710_for	GGGGACAAGTTTGTACAAAAAAGCAGGCTATGAAGCGGGCGGTTGCA	Tb927.3.3710_rev	GGGGACCACTTTGTACAAGAAAGCTGGGTCTACTGTTGCACTGGAGCCG
3_hypothetical	K21	Tb927.4.910_for	GGGGACAAGTTTGTACAAAAAAGCAGGCTATGTCAGAGGTGGATCAACAGCA	Tb927.4.910_rev	GGGGACCACTTTGTACAAGAAAGCTGGGTCTAATGGGGTGAAGCGCTGAC
3_hypothetical	K22	Tb927.6.1440_for	GGGGACAAGTTTGTACAAAAAAGCAGGCTATGAAGAGGTCCTTGATCGCGC	Tb927.6.1440_rev	GGGGACCACTTTGTACAAGAAAGCTGGGTTCAAGACGTCGACGCTGTTG
3_hypothetical	K23	Tb927.4.4040_for	GGGGACAAGTTTGTACAAAAAAGCAGGCTATGGATAATACGGAAGAAACGAGCAG	Tb927.4.4040_rev	GGGGACCACTTTGTACAAGAAAGCTGGGTTCAAACCCATCCGATTGCCCT
3_hypothetical	K24	Tb927.5.3200_for	GGGGACAAGTTTGTACAAAAAAGCAGGCTATGGCGACGAGGCCAAAC	Tb927.5.3200_rev	GGGGACCACTTTGTACAAGAAAGCTGGGTTCACTGTGCTGTCGGTGGG
3_hypothetical	L1	Tb927.11.4650_for	GGGGACAAGTTTGTACAAAAAAGCAGGCTATGCGCCTACATACGGTCCG	Tb927.11.4650_rev	GGGGACCACTTTGTACAAGAAAGCTGGGTCTACTCGTCCTTGATGGTGTTCTTTG
3_hypothetical	L2	Tb927.11.12760_for	GGGGACAAGTTTGTACAAAAAAGCAGGCTATGACAATTTTGTCTTTGCACGGC	Tb927.11.12760_rev	GGGGACCACTTTGTACAAGAAAGCTGGGTTCATTCCCTTACAGTCGGTTTAGTGG
3_hypothetical	L3	Tb927.4.4600_for	GGGGACAAGTTTGTACAAAAAAGCAGGCTATGAGCCGCAATGGCGAG	Tb927.4.4600_rev	GGGGACCACTTTGTACAAGAAAGCTGGGTCTAGGGGGGCTTTAATTGTTCCTTTC
3_hypothetical	L4	Tb927.10.10630_for	GGGGACAAGTTTGTACAAAAAAGCAGGCTATGCGCCCTAGGGATCTGTG	Tb927.10.10630_rev	GGGGACCACTTTGTACAAGAAAGCTGGGTCTATTCGTCATCACCACCGCTG
3_hypothetical	L5	Tb927.10.12080_for	GGGGACAAGTTTGTACAAAAAAGCAGGCTATGTCTGTTTCCCCCAAGCGG	Tb927.10.12080_rev	GGGGACCACTTTGTACAAGAAAGCTGGGTCTACCATCCGGCCATCGATGG
3_hypothetical	L6	Tb927.11.10970_for	GGGGACAAGTTTGTACAAAAAAGCAGGCTATGGAAAATGATGTGGATGAAGAGGTT	Tb927.11.10970_rev	GGGGACCACTTTGTACAAGAAAGCTGGGTTTACGGCATTGTCAAGAGAATATTGTGC
3_hypothetical	L7	Tb927.3.2080_for	GGGGACAAGTTTGTACAAAAAAGCAGGCTATGTCACTCTTCGCGAAGAAGAAG	Tb927.3.2080_rev	GGGGACCACTTTGTACAAGAAAGCTGGGTCTACAAACCGAGTTCAGTGGGATC
3_hypothetical	L8	Tb927.8.3390_for	GGGGACAAGTTTGTACAAAAAAGCAGGCTATGGTGAAAATGCCGTCGACC	Tb927.8.3390_rev	GGGGACCACTTTGTACAAGAAAGCTGGGTTTAACCGAGGTGCCCATCACC
3_hypothetical	L9	Tb927.11.13760_for	GGGGACAAGTTTGTACAAAAAAGCAGGCTATGATTTCACGGAGGAGCTTTGTAC	Tb927.11.13760_rev	GGGGACCACTTTGTACAAGAAAGCTGGGTTCAGCTTTGCTTTGCCTTTACGT
3_hypothetical	L10	Tb927.11.7600_for	GGGGACAAGTTTGTACAAAAAAGCAGGCTATGCTTGCCAACTACGACTATGAAA	Tb927.11.7600_rev	GGGGACCACTTTGTACAAGAAAGCTGGGTTTAGCCTCTATAAGAGCCCCGC
3_hypothetical	L11	Tb927.8.2400_for	GGGGACAAGTTTGTACAAAAAAGCAGGCTATGAATATCGTCACAAGCTACTTCCTG	Tb927.8.2400_rev	GGGGACCACTTTGTACAAGAAAGCTGGGTTTAATGCCGTTCCAACCTGTCG
3_hypothetical	L12	Tb927.6.4070_for	GGGGACAAGTTTGTACAAAAAAGCAGGCTATGACTGTATCAGGAATGACTGGGA	Tb927.6.4070_rev	GGGGACCACTTTGTACAAGAAAGCTGGGTTCAGTGTGCCGTTGCTATAGGG
3_hypothetical	L13	Tb927.10.1250_for	GGGGACAAGTTTGTACAAAAAAGCAGGCTATGACAAGTGTGGAAACCCGC	Tb927.10.1250_rev	GGGGACCACTTTGTACAAGAAAGCTGGGTTCAGTCCACCAACTGCCCC
3_hypothetical	L14	Tb927.10.13820_for	GGGGACAAGTTTGTACAAAAAAGCAGGCTATGTTCAACCGTAACTTTATTCTCCGA	Tb927.10.13820_rev	GGGGACCACTTTGTACAAGAAAGCTGGGTTTAGCAAGCCAACCTCTTCCTCC
3_hypothetical	L15	Tb927.7.6210_for	GGGGACAAGTTTGTACAAAAAAGCAGGCTATGGACACGTCGGGCACC	Tb927.7.6210_rev	GGGGACCACTTTGTACAAGAAAGCTGGGTCTACAAGTCGTAAAAGCGGCACA
3_hypothetical	L16	Tb927.11.8430_for	GGGGACAAGTTTGTACAAAAAAGCAGGCTATGTTTTGTGGCTCTAGGATGTTCC	Tb927.11.8430_rev	GGGGACCACTTTGTACAAGAAAGCTGGGTTTAAAGGTGATACTGCTCCACTTCATC
3_hypothetical	L17	Tb927.10.700_for	GGGGACAAGTTTGTACAAAAAAGCAGGCTATGCTACCGTGGCTATTATTTATCAACG	Tb927.10.700_rev	GGGGACCACTTTGTACAAGAAAGCTGGGTTCATGACACGACTTTACTAAATCCTTCTAT
3_hypothetical	L18	Tb927.8.1930_for	GGGGACAAGTTTGTACAAAAAAGCAGGCTATGCAAGACTACTTGCGGGAAATC	Tb927.8.1930_rev	GGGGACCACTTTGTACAAGAAAGCTGGGTCTAATTCTTGAGTGCATCAAGGCGC
3_hypothetical	L19	Tb927.8.8280_for	GGGGACAAGTTTGTACAAAAAAGCAGGCTATGGAATATGAGGGGTGTGCATCG	Tb927.8.8280_rev	GGGGACCACTTTGTACAAGAAAGCTGGGTTTAATTCCCCTTTTCGAAATTAAGTTCGC
3_hypothetical	L20	Tb927.11.3770_for	GGGGACAAGTTTGTACAAAAAAGCAGGCTATGATTCCTGCCCTTAATGGTGTTG	Tb927.11.3770_rev	GGGGACCACTTTGTACAAGAAAGCTGGGTCTACTGTTCTGTAACCTCAGTATCGC
3_hypothetical	L21	Tb927.11.10800_for	GGGGACAAGTTTGTACAAAAAAGCAGGCTATGGAGCCAAGTAAAACCAGCAC	Tb927.11.10800_rev	GGGGACCACTTTGTACAAGAAAGCTGGGTTCAAGAAGGTGATAGCGTAAACTGTTT
3_hypothetical	L22	Tb927.9.9430_for	GGGGACAAGTTTGTACAAAAAAGCAGGCTATGTCGGCAGCAGTTCCTGT	Tb927.9.9430_rev	GGGGACCACTTTGTACAAGAAAGCTGGGTCTAACGAGTAATCCTTTTTGCCAGCA
3_hypothetical	L23	Tb927.11.10700_for	GGGGACAAGTTTGTACAAAAAAGCAGGCTATGTATGCGACTCGTGCTAAGGT	Tb927.11.10700_rev	GGGGACCACTTTGTACAAGAAAGCTGGGTCTAACCCTGTTGTTTATTGTTCCAATCTAC
3_hypothetical	L24	Tb927.11.5530_for	GGGGACAAGTTTGTACAAAAAAGCAGGCTATGAAGCGACTATTCCCTTCTGCA	Tb927.11.5530_rev	GGGGACCACTTTGTACAAGAAAGCTGGGTTTACTCACAACGTTCCGCCCA
3_hypothetical	M1	Tb927.5.3410_for	GGGGACAAGTTTGTACAAAAAAGCAGGCTATGCGTCGCACGGTGGTG	Tb927.5.3410_rev	GGGGACCACTTTGTACAAGAAAGCTGGGTTTAGAAGAGCTTGATTTTTCTAGCCTTGC
3_hypothetical	M2	Tb927.8.3890_for	GGGGACAAGTTTGTACAAAAAAGCAGGCTATGTGCGCTGGGCGGGAT	Tb927.8.3890_rev	GGGGACCACTTTGTACAAGAAAGCTGGGTCTATTCCATTGTTTGCTCTCTTGGATATCC
3_hypothetical	M3	Tb927.5.3790_for	GGGGACAAGTTTGTACAAAAAAGCAGGCTATGAAAGTAGAATCACCTCATTCGAACTAT	Tb927.5.3790_rev	GGGGACCACTTTGTACAAGAAAGCTGGGTTCACGACTCTGCATGAGGATATGC
3_hypothetical	M4	Tb927.7.4260_for	GGGGACAAGTTTGTACAAAAAAGCAGGCTATGACAGTGAAGGTTTCATTTTCTCTTACA	Tb927.7.4260_rev	GGGGACCACTTTGTACAAGAAAGCTGGGTTTACTTCGGATTGTTGTCAGCACTATT
3_hypothetical	M5	Tb927.9.7800_for	GGGGACAAGTTTGTACAAAAAAGCAGGCTATGGGTAATCAAAAAGCTGTAGCTGG	Tb927.9.7800_rev	GGGGACCACTTTGTACAAGAAAGCTGGGTTCACGCTTTCGCAAGAAAATTCG
3_hypothetical	M6	Tb927.11.720_for	GGGGACAAGTTTGTACAAAAAAGCAGGCTATGGAACTGATGGAGGAGCGC	Tb927.11.720_rev	GGGGACCACTTTGTACAAGAAAGCTGGGTTTACCTGAATATAATAAGATTCCTTTCACT
3_hypothetical	M7	Tb927.10.1820_for	GGGGACAAGTTTGTACAAAAAAGCAGGCTATGATGAAGAGAACAATATTACAAAGATGC	Tb927.10.1820_rev	GGGGACCACTTTGTACAAGAAAGCTGGGTTCACCTGCCTCCCATCATAGTTT
3_hypothetical	M8	Tb927.7.1540_for	GGGGACAAGTTTGTACAAAAAAGCAGGCTATGCACGAGAAAGGATATGCCG	Tb927.7.1540_rev	GGGGACCACTTTGTACAAGAAAGCTGGGTCTATCTGCTAGCAAACAACCTGCG
3_hypothetical	M9	Tb927.5.3530_for	GGGGACAAGTTTGTACAAAAAAGCAGGCTATGGCCGCTGTCCCTTCA	Tb927.5.3530_rev	GGGGACCACTTTGTACAAGAAAGCTGGGTTTATCCGGCCCAATTATGGCAC
3_hypothetical	M10	Tb927.5.1990_for	GGGGACAAGTTTGTACAAAAAAGCAGGCTATGACGCAATCGCAATCGCA	Tb927.5.1990_rev	GGGGACCACTTTGTACAAGAAAGCTGGGTTTAGCGACTGAAGCGGGCG
3_hypothetical	M11	Tb927.8.4480_for	GGGGACAAGTTTGTACAAAAAAGCAGGCTATGACGGCGAGCCGTTATCA	Tb927.8.4480_rev	GGGGACCACTTTGTACAAGAAAGCTGGGTTTACACCACAGTAAAGAGCACATCC
3_hypothetical	M12	Tb927.10.1770_for	GGGGACAAGTTTGTACAAAAAAGCAGGCTATGAAAACGGAAAATTTTCCAAGAGGAC	Tb927.10.1770_rev	GGGGACCACTTTGTACAAGAAAGCTGGGTCTATGCTCCGGTGTTCTGTTTACC
3_hypothetical	M13	Tb927.8.6920_for	GGGGACAAGTTTGTACAAAAAAGCAGGCTATGACAAAAGGTGGGTCGAGTGT	Tb927.8.6920_rev	GGGGACCACTTTGTACAAGAAAGCTGGGTTTAATACTTCAACGGCTGATCAACAGG
3_hypothetical	M14	Tb927.3.3660_for	GGGGACAAGTTTGTACAAAAAAGCAGGCTATGCTGACTTTACGCAAGCAAAC	Tb927.3.3660_rev	GGGGACCACTTTGTACAAGAAAGCTGGGTCTACCAGTTTCCCGCCTCATGC
3_hypothetical	M15	Tb927.10.15820_for	GGGGACAAGTTTGTACAAAAAAGCAGGCTATGACAGCAGTACCTGTGGCA	Tb927.10.15820_rev	GGGGACCACTTTGTACAAGAAAGCTGGGTTCACTTCGGTACACCAATACCACA
3_hypothetical	M16	Tb927.10.8250_for	GGGGACAAGTTTGTACAAAAAAGCAGGCTATGCAGGGGAAGGTTGTCAGT	Tb927.10.8250_rev	GGGGACCACTTTGTACAAGAAAGCTGGGTTCATGGCTTTCGCTGTAACACG
3_hypothetical	M17	Tb927.7.6490_for	GGGGACAAGTTTGTACAAAAAAGCAGGCTATGAGCTCCACGAGGGCTG	Tb927.7.6490_rev	GGGGACCACTTTGTACAAGAAAGCTGGGTTTATTCGTCGTCGCCGGC
3_hypothetical	M18	Tb927.11.13860_for	GGGGACAAGTTTGTACAAAAAAGCAGGCTATGCATAACAGCAGGTCCACCA	Tb927.11.13860_rev	GGGGACCACTTTGTACAAGAAAGCTGGGTTTAAATGAAGAAAGATGATACGGCATCCC
3_hypothetical	M19	Tb927.8.7250_for	GGGGACAAGTTTGTACAAAAAAGCAGGCTATGGAGTTCGTTCCCCTCGAG	Tb927.8.7250_rev	GGGGACCACTTTGTACAAGAAAGCTGGGTTCACGGGCGATAATAATAATGTACATTCTT
3_hypothetical	M20	Tb927.4.5130_for	GGGGACAAGTTTGTACAAAAAAGCAGGCTATGCATGTCACGGGGTGC	Tb927.4.5130_rev	GGGGACCACTTTGTACAAGAAAGCTGGGTTCAAAAACAATTGCATGTCGCTTTCT
3_hypothetical	M21	Tb927.10.8790_for	GGGGACAAGTTTGTACAAAAAAGCAGGCTATGCTGCGCTTCCGGGTT	Tb927.10.8790_rev	GGGGACCACTTTGTACAAGAAAGCTGGGTTTACCGCAAACGCAACTTTTTAGC

3_hypothetical	M22	Tb927.4.5140_for	GGGGACAAGTTTGTACAAAAAAGCAGGCTATGGAGTTCGTTCCCCTCGAG	Tb927.4.5140_rev	GGGGACCACTTTGTACAAGAAAGCTGGGTTTACGCACGGCAATAATAATGTACATTC
3_hypothetical	M23	Tb927.4.2810_for	GGGGACAAGTTTGTACAAAAAAGCAGGCTATGCATCGTGTGATCACCATTTTATCT	Tb927.4.2810_rev	GGGGACCACTTTGTACAAGAAAGCTGGGTTCATTCGCTCATTAACCAGAATTCCA
3_hypothetical	M24	Tb927.11.13910_for	GGGGACAAGTTTGTACAAAAAAGCAGGCTATGTCCGTTGTTGGTGTCTCTTC	Tb927.11.13910_rev	GGGGACCACTTTGTACAAGAAAGCTGGGTTCACATTTCACACCGCCGG
3_hypothetical	N1	Tb927.10.12350_for	GGGGACAAGTTTGTACAAAAAAGCAGGCTATGCGCGTGGCGCTAACT	Tb927.10.12350_rev	GGGGACCACTTTGTACAAGAAAGCTGGGTTTATTTTTCACTACTCTGTTGGAAAAACTT
3_hypothetical	N2	Tb927.10.9870_for	GGGGACAAGTTTGTACAAAAAAGCAGGCTATGGTTCTTATTGAGTGCAAGCGT	Tb927.10.9870_rev	GGGGACCACTTTGTACAAGAAAGCTGGGTTTACCCAGGTTTGTACTTAACTCCATCG
3_hypothetical	N3	Tb927.11.14390_for	GGGGACAAGTTTGTACAAAAAAGCAGGCTATGGCCACACTTCAAGTGCG	Tb927.11.14390_rev	GGGGACCACTTTGTACAAGAAAGCTGGGTTTACTTTACCACTTCAACGAGTGGAG
3_hypothetical	N4	Tb927.5.2590_for	GGGGACAAGTTTGTACAAAAAAGCAGGCTATGAAACGAGAGCGAGGTGGA	Tb927.5.2590_rev	GGGGACCACTTTGTACAAGAAAGCTGGGTTTAGAGACTGCTCCTCCCAACG
3_hypothetical	N5	Tb927.7.2190_for	GGGGACAAGTTTGTACAAAAAAGCAGGCTATGGCTTGCCGTCGTTCC	Tb927.7.2190_rev	GGGGACCACTTTGTACAAGAAAGCTGGGTTCATTTCTTCTTTTTCGGTGATTGGGA
3_hypothetical	N6	Tb927.9.10630_for	GGGGACAAGTTTGTACAAAAAAGCAGGCTATGTCGTGGCGTACGCGT	Tb927.9.10630_rev	GGGGACCACTTTGTACAAGAAAGCTGGGTTTACAGAATTCCTAACGCAGAACACC
3_hypothetical	N7	Tb927.6.1620_for	GGGGACAAGTTTGTACAAAAAAGCAGGCTATGGAATTCTTCCGGCGGACG	Tb927.6.1620_rev	GGGGACCACTTTGTACAAGAAAGCTGGGTTCACACGTGAGACACCCCAC
3_hypothetical	N8	Tb927.11.11320_for	GGGGACAAGTTTGTACAAAAAAGCAGGCTATGACGAGGAAAGTGGCCGG	Tb927.11.11320_rev	GGGGACCACTTTGTACAAGAAAGCTGGGTTCAACTGCGAGGGATTGTGATG
3_hypothetical	N9	Tb927.10.15830_for	GGGGACAAGTTTGTACAAAAAAGCAGGCTATGCGACGCTGCGCGGCA	Tb927.10.15830_rev	GGGGACCACTTTGTACAAGAAAGCTGGGTTCAGAAGTACCGTTTGGGCCG
3_hypothetical	N10	Tb927.11.2810_for	GGGGACAAGTTTGTACAAAAAAGCAGGCTATGGTGCTCCGCTGTGCT	Tb927.11.2810_rev	GGGGACCACTTTGTACAAGAAAGCTGGGTTTACCGTGAAGGGTAATGTTGCAA
3_hypothetical	N11	Tb927.9.3560_for	GGGGACAAGTTTGTACAAAAAAGCAGGCTATGGAGAGGAGTAATTCCCGGAGG	Tb927.9.3560_rev	GGGGACCACTTTGTACAAGAAAGCTGGGTCTACCGTCGAGCAGCATATGC
3_hypothetical	N12	Tb927.6.2140_for	GGGGACAAGTTTGTACAAAAAAGCAGGCTATGTTCCGCTGGTCGTGGT	Tb927.6.2140_rev	GGGGACCACTTTGTACAAGAAAGCTGGGTTCAGGTTAAGGACGCAGAAACAC
3_hypothetical	N13	Tb927.5.2730_for	GGGGACAAGTTTGTACAAAAAAGCAGGCTATGAGCAGCGATTCAAAAGCCA	Tb927.5.2730_rev	GGGGACCACTTTGTACAAGAAAGCTGGGTTTACGGCAACGTGTCGTCC
3_hypothetical	N14	Tb927.3.3040_for	GGGGACAAGTTTGTACAAAAAAGCAGGCTATGGAAACTACAACACACGTTGTCA	Tb927.3.3040_rev	GGGGACCACTTTGTACAAGAAAGCTGGGTTCATGTCGGTGGATCGACGT
3_hypothetical	N15	Tb927.8.6380_for	GGGGACAAGTTTGTACAAAAAAGCAGGCTATGATAGTGCGGTTGCTTTTCTTTTC	Tb927.8.6380_rev	GGGGACCACTTTGTACAAGAAAGCTGGGTCTACTTCATCTTTTTCCTCTTCTCCGC
3_hypothetical	N16	Tb927.7.6600_for	GGGGACAAGTTTGTACAAAAAAGCAGGCTATGGTTTTCGCAAGGGGCG	Tb927.7.6600_rev	GGGGACCACTTTGTACAAGAAAGCTGGGTCTAATCCACCTCTGCCTGGGC
3_hypothetical	N17	Tb927.9.10890_for	GGGGACAAGTTTGTACAAAAAAGCAGGCTATGGAAACATTCCGTGAGCGC	Tb927.9.10890_rev	GGGGACCACTTTGTACAAGAAAGCTGGGTTCAGTCGTCACTGTCCAACTGA
3_hypothetical	N18	Tb927.11.13890_for	GGGGACAAGTTTGTACAAAAAAGCAGGCTATGCTTAGGCGTGTCCCGA	Tb927.11.13890_rev	GGGGACCACTTTGTACAAGAAAGCTGGGTCTACGATAAGTGCACAGCGCC
3_hypothetical	N19	Tb927.8.8120_for	GGGGACAAGTTTGTACAAAAAAGCAGGCTATGCTATTCATCAGAATATTAAACCCATTA	Tb927.8.8120_rev	GGGGACCACTTTGTACAAGAAAGCTGGGTTCAGTCCTTCTTCACGTCACCT
3_hypothetical	N20	Tb927.10.12270_for	GGGGACAAGTTTGTACAAAAAAGCAGGCTATGAGGCGAGTGGTAGTGGATT	Tb927.10.12270_rev	GGGGACCACTTTGTACAAGAAAGCTGGGTTCAGACTGCCCGCCTGGC
3_hypothetical	N21	Tb927.11.15270_for	GGGGACAAGTTTGTACAAAAAAGCAGGCTATGGATCCGCTTCTGTTAGGCA	Tb927.11.15270_rev	GGGGACCACTTTGTACAAGAAAGCTGGGTTCAGGTACTCAACTCACATAACCGC
3_hypothetical	N22	Tb927.10.7030_for	GGGGACAAGTTTGTACAAAAAAGCAGGCTATGGATCCATCGAAGCGCATATG	Tb927.10.7030_rev	GGGGACCACTTTGTACAAGAAAGCTGGGTTTACTTCATTTTGGCGCGTAACGC
3_hypothetical	N23	Tb927.11.6020_for	GGGGACAAGTTTGTACAAAAAAGCAGGCTATGTTTGATGTGTCACCGCGT	Tb927.11.6020_rev	GGGGACCACTTTGTACAAGAAAGCTGGGTTTAGTACTTCCCATATCTCCTGTAGGTCG
3_hypothetical	N24	Tb927.11.10140_for	GGGGACAAGTTTGTACAAAAAAGCAGGCTATGGTGTCGTACGCCTCTGC	Tb927.11.10140_rev	GGGGACCACTTTGTACAAGAAAGCTGGGTCTACAATTTGGAGGGCTGCGT
3_hypothetical	O1	Tb927.10.5750_for	GGGGACAAGTTTGTACAAAAAAGCAGGCTATGACGGTGTACCCCTCACTATTT	Tb927.10.5750_rev	GGGGACCACTTTGTACAAGAAAGCTGGGTTCATTCAGTAAACAAGTAATTCCGTACATT
3_hypothetical	O2	Tb927.3.2670_for	GGGGACAAGTTTGTACAAAAAAGCAGGCTATGCAGAGACTCTTTTTTAGATCGACAT	Tb927.3.2670_rev	GGGGACCACTTTGTACAAGAAAGCTGGGTTTATTTCTGCACTGTGGGCGTC
3_hypothetical	O3	Tb927.11.1280_for	GGGGACAAGTTTGTACAAAAAAGCAGGCTATGAGGCCAACAGGAGAGTCTG	Tb927.11.1280_rev	GGGGACCACTTTGTACAAGAAAGCTGGGTTTATCTGCCCGTCCGGGC
3_hypothetical	O4	Tb927.10.8200_for	GGGGACAAGTTTGTACAAAAAAGCAGGCTATGGAGACCCCAAGAAGCAGAG	Tb927.10.8200_rev	GGGGACCACTTTGTACAAGAAAGCTGGGTCTACTCTTTCTTTCTTTTCTTCGTCTGACG
3_hypothetical	O5	Tb927.8.3740_for	GGGGACAAGTTTGTACAAAAAAGCAGGCTATGAATGTGCTAACATCTCACACTCAT	Tb927.8.3740_rev	GGGGACCACTTTGTACAAGAAAGCTGGGTTTACGGGGATGTGCAAGAAGAAGG
3_hypothetical	O6	Tb927.4.4700_for	GGGGACAAGTTTGTACAAAAAAGCAGGCTATGGCTCAGACGAGCAACAATG	Tb927.4.4700_rev	GGGGACCACTTTGTACAAGAAAGCTGGGTTCATACCCGCCCGGATGTC
3_hypothetical	O7	Tb927.4.1750_for	GGGGACAAGTTTGTACAAAAAAGCAGGCTATGTTCCGCGGGTCAGGG	Tb927.4.1750_rev	GGGGACCACTTTGTACAAGAAAGCTGGGTTCACCGCGATCCACTCGC
3_hypothetical	O8	Tb927.11.6250_for	GGGGACAAGTTTGTACAAAAAAGCAGGCTATGTCGAAGCAACTAACGTTTATATCAGC	Tb927.11.6250_rev	GGGGACCACTTTGTACAAGAAAGCTGGGTCTAAAGTGCAGAAACCACCGCC
3_hypothetical	O9	Tb927.2.1730_for	GGGGACAAGTTTGTACAAAAAAGCAGGCTATGAACACGGAGGACTCCTCC	Tb927.2.1730_rev	GGGGACCACTTTGTACAAGAAAGCTGGGTTCAGATAGCACAAAAATACAAATACGCAG
3_hypothetical	O10	Tb927.5.3640_for	GGGGACAAGTTTGTACAAAAAAGCAGGCTATGTTTAGCGAATCTCTCTGTATTCTCTCA	Tb927.5.3640_rev	GGGGACCACTTTGTACAAGAAAGCTGGGTTTAGAACCAGTGACCCCACATGA
3_hypothetical	O11	Tb927.11.13270_for	GGGGACAAGTTTGTACAAAAAAGCAGGCTATGAACCCCGGAAATGATTTGGATG	Tb927.11.13270_rev	GGGGACCACTTTGTACAAGAAAGCTGGGTTTAAATTTTGAAGGATGGTCTTACTCCCC
3_hypothetical	O12	Tb927.1.3040_for	GGGGACAAGTTTGTACAAAAAAGCAGGCTATGCGCACGTTCAGTCGTAC	Tb927.1.3040_rev	GGGGACCACTTTGTACAAGAAAGCTGGGTTCACGTGCCTTTGGGCGC
3_hypothetical	O13	Tb927.11.7830_for	GGGGACAAGTTTGTACAAAAAAGCAGGCTATGCGGGCACTTAGAGGGG	Tb927.11.7830_rev	GGGGACCACTTTGTACAAGAAAGCTGGGTCTACCGGCTCCTGCCATATGA
3_hypothetical	O14	Tb927.5.1050_for	GGGGACAAGTTTGTACAAAAAAGCAGGCTATGGCCAGTAGCACGGATTCA	Tb927.5.1050_rev	GGGGACCACTTTGTACAAGAAAGCTGGGTTCATTTTCCCCGATGTCTGTCCG
3_hypothetical	O15	Tb927.9.8490_for	GGGGACAAGTTTGTACAAAAAAGCAGGCTATGAGCACCCACACCAGCT	Tb927.9.8490_rev	GGGGACCACTTTGTACAAGAAAGCTGGGTTTATCCTCGGCGCCTCCG
3_hypothetical	O16	Tb927.1.1610_for	GGGGACAAGTTTGTACAAAAAAGCAGGCTATGCAGCGCGATGTGTACG	Tb927.1.1610_rev	GGGGACCACTTTGTACAAGAAAGCTGGGTTCAGTGAACGAATTGGTACTCGTC
3_hypothetical	O17	Tb927.7.4870_for	GGGGACAAGTTTGTACAAAAAAGCAGGCTATGGACGTTCTTATTGCAAAAGCAGT	Tb927.7.4870_rev	GGGGACCACTTTGTACAAGAAAGCTGGGTTCACATATTCATGGTGTTGGGTCCC
3_hypothetical	O18	Tb927.2.4090_for	GGGGACAAGTTTGTACAAAAAAGCAGGCTATGCTTTTAATTGCCGTCACAATCATT	Tb927.2.4090_rev	GGGGACCACTTTGTACAAGAAAGCTGGGTTTAACTCGTCGCGAACTCACG
3_hypothetical	O19	Tb927.8.5590_for	GGGGACAAGTTTGTACAAAAAAGCAGGCTATGGAAACGAGAAAAGTTACAGAGTGC	Tb927.8.5590_rev	GGGGACCACTTTGTACAAGAAAGCTGGGTTCACCTCGTAACCACACTTGATTG
3_hypothetical	O20	Tb927.11.14690_for	GGGGACAAGTTTGTACAAAAAAGCAGGCTATGGCCACTGAAGGAGAAGTGG	Tb927.11.14690_rev	GGGGACCACTTTGTACAAGAAAGCTGGGTTCAGGTAAGCAGCACCATCAGT
3_hypothetical	O21	Tb927.10.15230_for	GGGGACAAGTTTGTACAAAAAAGCAGGCTATGTCAGATTGGAAGGCCGCT	Tb927.10.15230_rev	GGGGACCACTTTGTACAAGAAAGCTGGGTCTATGGAATGGCCTCAACACTGTC
3_hypothetical	O22	Tb927.11.2770_for	GGGGACAAGTTTGTACAAAAAAGCAGGCTATGTTTTCTCGGCGCCGTAAG	Tb927.11.2770_rev	GGGGACCACTTTGTACAAGAAAGCTGGGTTCAGTCACCATAAAAAGTTGCTATAACTCT
3_hypothetical	O23	Tb927.7.5640_for	GGGGACAAGTTTGTACAAAAAAGCAGGCTATGAGCGAAGACCCAAACGGC	Tb927.7.5640_rev	GGGGACCACTTTGTACAAGAAAGCTGGGTTCACTCGATGAAAGGCGTATCGG
3_hypothetical	O24	Tb927.5.150_for	GGGGACAAGTTTGTACAAAAAAGCAGGCTATGCTTAAAATTGCAGCAGTCATTTTTTTG	Tb927.5.150_rev	GGGGACCACTTTGTACAAGAAAGCTGGGTTCAGGAGAACACAACACTTAATCCTGC
3_hypothetical	P1	Tb927.11.2800_for	GGGGACAAGTTTGTACAAAAAAGCAGGCTATGAGCCCCGTTAAAGCTTTCTG	Tb927.11.2800_rev	GGGGACCACTTTGTACAAGAAAGCTGGGTTCACGACTCCGCAGCATTCC
3_hypothetical	P2	Tb927.8.510_for	GGGGACAAGTTTGTACAAAAAAGCAGGCTATGAAAACCATAGTACTGGCGCTC	Tb927.8.510_rev	GGGGACCACTTTGTACAAGAAAGCTGGGTTTAAAAATAACATAAAATGCTTAGTAAAAA
3_hypothetical	P3	Tb927.7.2090_for	GGGGACAAGTTTGTACAAAAAAGCAGGCTATGGACAACGTTGTGAAGAATTTAATGC	Tb927.7.2090_rev	GGGGACCACTTTGTACAAGAAAGCTGGGTCTACTGCATTGCTGGAACTAAAACG
3_hypothetical	P4	Tb927.10.5540_for	GGGGACAAGTTTGTACAAAAAAGCAGGCTATGCTTAGCCGCTGTTTTGTCG	Tb927.10.5540_rev	GGGGACCACTTTGTACAAGAAAGCTGGGTTCACGAATCCAGTTCAAGTCGC
3_hypothetical	P5	Tb927.7.3540_for	GGGGACAAGTTTGTACAAAAAAGCAGGCTATGGCATGTGGTGACTTTGGC	Tb927.7.3540_rev	GGGGACCACTTTGTACAAGAAAGCTGGGTTCACACACTTTCCTCGTCAGACT
3_hypothetical	P6	Tb927.6.3240_for	GGGGACAAGTTTGTACAAAAAAGCAGGCTATGATGGGAGGAATCTCTATTTGTGTTG	Tb927.6.3240_rev	GGGGACCACTTTGTACAAGAAAGCTGGGTTCATATTCGTTTAATGGGGGTACTAATAGT
3_hypothetical	P7	Tb927.3.5600_for	GGGGACAAGTTTGTACAAAAAAGCAGGCTATGAAGGGTCTGCGGTTATTTTTTACA	Tb927.3.5600_rev	GGGGACCACTTTGTACAAGAAAGCTGGGTCTACACTCCTCTGCCTCCTGC
3_hypothetical	P8	Tb927.3.5430_for	GGGGACAAGTTTGTACAAAAAAGCAGGCTATGCCAAAGGTTCTCATCCCTATGT	Tb927.3.5430_rev	GGGGACCACTTTGTACAAGAAAGCTGGGTTCAGCCCCTTCGTCTTCTCG
3_hypothetical	P9	Tb927.11.13950_for	GGGGACAAGTTTGTACAAAAAAGCAGGCTATGCATTCGTCACCAGCGAC	Tb927.11.13950_rev	GGGGACCACTTTGTACAAGAAAGCTGGGTTCACCGAACCGACTGGGAC
3_hypothetical	P10	Tb927.1.5000_for	GGGGACAAGTTTGTACAAAAAAGCAGGCTATGACAACTGAAGAGGGAAAAGTAGAGA	Tb927.1.5000_rev	GGGGACCACTTTGTACAAGAAAGCTGGGTTTACCGCAGCCGAGGAACA
3_hypothetical	P11	Tb927.9.12680_for	GGGGACAAGTTTGTACAAAAAAGCAGGCTATGAGTAGTATCTTCACCTATGGTGCG	Tb927.9.12680_rev	GGGGACCACTTTGTACAAGAAAGCTGGGTTTAGTTTCTCCACTCATCGTGGTCG
3_hypothetical	P12	Tb927.4.5060_for	GGGGACAAGTTTGTACAAAAAAGCAGGCTATGGGTTCTGCTGCGGGA	Tb927.4.5060_rev	GGGGACCACTTTGTACAAGAAAGCTGGGTTCACGGTGTTGGTGTACAACG
3_hypothetical	P13	Tb927.4.2930_for	GGGGACAAGTTTGTACAAAAAAGCAGGCTATGCGCCGCTCTTCGATG	Tb927.4.2930_rev	GGGGACCACTTTGTACAAGAAAGCTGGGTTCACTTGTCGGGTCCCTTTCC
3_hypothetical	P14	Tb927.11.4920_for	GGGGACAAGTTTGTACAAAAAAGCAGGCTATGTCTACAATCTACAATGCAGTGGATG	Tb927.11.4920_rev	GGGGACCACTTTGTACAAGAAAGCTGGGTCTACTTGCTCGATGTTTCCCCAAA
3_hypothetical	P15	Tb927.11.4760_for	GGGGACAAGTTTGTACAAAAAAGCAGGCTATGTTTATCAGTGGGCTGTTCGTG	Tb927.11.4760_rev	GGGGACCACTTTGTACAAGAAAGCTGGGTTTACAAAACACGCGTAATGGTGGC
3_hypothetical	P16	Tb927.8.1570_for	GGGGACAAGTTTGTACAAAAAAGCAGGCTATGCCCGGGCAGGTTTCT	Tb927.8.1570_rev	GGGGACCACTTTGTACAAGAAAGCTGGGTTCAGGCGGAGACTGTTTGGT
3_hypothetical	P17	Tb927.5.4020_for	GGGGACAAGTTTGTACAAAAAAGCAGGCTATGATGATTTCCCGCGCTTTGT	Tb927.5.4020_rev	GGGGACCACTTTGTACAAGAAAGCTGGGTTTAGAAAGAGAGGGCCGTGGC
3_hypothetical	P18	Tb927.11.11390_for	GGGGACAAGTTTGTACAAAAAAGCAGGCTATGCAAACGTCGCCGGTC	Tb927.11.11390_rev	GGGGACCACTTTGTACAAGAAAGCTGGGTTCATGTGTGGCCCTTTCTTTCC
3_hypothetical	P19	Tb927.8.4530_for	GGGGACAAGTTTGTACAAAAAAGCAGGCTATGATGAATGACAAAATGCTTGGGGG	Tb927.8.4530_rev	GGGGACCACTTTGTACAAGAAAGCTGGGTCTATGGAGTATGCGCAAGAATCCTC
3_hypothetical	P20	Tb927.10.3290_for	GGGGACAAGTTTGTACAAAAAAGCAGGCTATGCGCAGCCTCCGCTCA	Tb927.10.3290_rev	GGGGACCACTTTGTACAAGAAAGCTGGGTTTAACGGCCTCTGGGGTAGC
3_hypothetical	P21	Tb927.8.6550_for	GGGGACAAGTTTGTACAAAAAAGCAGGCTATGTGGCGGCGGACGCCA	Tb927.8.6550_rev	GGGGACCACTTTGTACAAGAAAGCTGGGTTTAGCGGAAGTTGGGGTGGG
3_hypothetical	P22	Tb927.5.3570_for	GGGGACAAGTTTGTACAAAAAAGCAGGCTATGAATTCGTGTGATAACGTCGGC	Tb927.5.3570_rev	GGGGACCACTTTGTACAAGAAAGCTGGGTTCAACTTCTGTCAGACACCATAACCG
3_hypothetical	P23	Tb927.7.6700_for	GGGGACAAGTTTGTACAAAAAAGCAGGCTATGGAAGACATTCCACAAGATCCTGA	Tb927.7.6700_rev	GGGGACCACTTTGTACAAGAAAGCTGGGTTTACGTGATCTCGCAGTCACGC
3_hypothetical	P24	Tb927.11.5790_for	GGGGACAAGTTTGTACAAAAAAGCAGGCTATGGACTACCGTGGAAAGCACG	Tb927.11.5790_rev	GGGGACCACTTTGTACAAGAAAGCTGGGTTCAGGCGCTCACGTCTCTC
3_known	A1	Tb927.5.4430_for	GGGGACAAGTTTGTACAAAAAAGCAGGCTATGTCGCACGTTCCCTTTCG	Tb927.5.4430_rev	GGGGACCACTTTGTACAAGAAAGCTGGGTCTATCCCTCTTCACATTGGCTCGT
3_known	A2	Tb927.8.5950_for	GGGGACAAGTTTGTACAAAAAAGCAGGCTATGCCACCCAAACGACCCA	Tb927.8.5950_rev	GGGGACCACTTTGTACAAGAAAGCTGGGTTCAGGATACTGTGGTGAACCATCTT
3_known	A3	Tb927.7.1690_for	GGGGACAAGTTTGTACAAAAAAGCAGGCTATGACAGAACCGAAGTTAGCAACC	Tb927.7.1690_rev	GGGGACCACTTTGTACAAGAAAGCTGGGTTTAACCCTCAACGACTTTTTCCCTAC
3_known	A4	Tb927.6.4440_for	GGGGACAAGTTTGTACAAAAAAGCAGGCTATGCCAACGCCAACTGAGG	Tb927.6.4440_rev	GGGGACCACTTTGTACAAGAAAGCTGGGTTCAGCTGCGCTGCTTCTG
3_known	A5	Tb927.3.4910_for	GGGGACAAGTTTGTACAAAAAAGCAGGCTATGCCGAGTCCATCTGAAACAATAC	Tb927.3.4910_rev	GGGGACCACTTTGTACAAGAAAGCTGGGTCTAGTCATTCTTTTTTGTTCCCTCACATAC
3_known	A6	Tb927.10.13780_for	GGGGACAAGTTTGTACAAAAAAGCAGGCTATGTCGCTCAACCTTACCGATGC	Tb927.10.13780_rev	GGGGACCACTTTGTACAAGAAAGCTGGGTTTACTTCTTCAGCAGATACTCCCGC
3_known	A7	Tb927.6.1550_for	GGGGACAAGTTTGTACAAAAAAGCAGGCTATGGACATTGATCTTTCATATGGTGACC	Tb927.6.1550_rev	GGGGACCACTTTGTACAAGAAAGCTGGGTTCAGCGCGCTGCGACCCG
3_known	A8	Tb927.10.2440_for	GGGGACAAGTTTGTACAAAAAAGCAGGCTATGGGAGGCTGCGTCAGT	Tb927.10.2440_rev	GGGGACCACTTTGTACAAGAAAGCTGGGTCTACAGGAGCGTCCGTTCCT
3_known	A9	Tb927.1.4100_for	GGGGACAAGTTTGTACAAAAAAGCAGGCTATGTTTGCTCGCCGCTCAC	Tb927.1.4100_rev	GGGGACCACTTTGTACAAGAAAGCTGGGTCTAAATCTTGTTTGAGAGCTTCTTCCATTG
3_known	A10	Tb927.8.2650_for	GGGGACAAGTTTGTACAAAAAAGCAGGCTATGCGCCACTTCAGATTAAACCG	Tb927.8.2650_rev	GGGGACCACTTTGTACAAGAAAGCTGGGTTTAAAGATAGCCTTCATTAAAACCCACTGA
3_known	A11	Tb927.11.9470_for	GGGGACAAGTTTGTACAAAAAAGCAGGCTATGAGTCGTAATAGGGATGAGGTGC	Tb927.11.9470_rev	GGGGACCACTTTGTACAAGAAAGCTGGGTTTAGTGCTTCTTACCAAAAGGATTTAGAGA
3_known	A12	Tb927.8.3530_for	GGGGACAAGTTTGTACAAAAAAGCAGGCTATGGTCTCCGGTGTGACATATCTG	Tb927.8.3530_rev	GGGGACCACTTTGTACAAGAAAGCTGGGTTTACATTTTGGAAGTGCGCGGG
3_known	A13	Tb927.11.1600_for	GGGGACAAGTTTGTACAAAAAAGCAGGCTATGAGGGGATCTTCCGTTCCTTG	Tb927.11.1600_rev	GGGGACCACTTTGTACAAGAAAGCTGGGTTCAGATGTGAAACACCTCAACACATG
3_known	A14	Tb927.9.9780_for	GGGGACAAGTTTGTACAAAAAAGCAGGCTATGGGCAAGTTCTGCTGGC	Tb927.9.9780_rev	GGGGACCACTTTGTACAAGAAAGCTGGGTTCACGAAAACATCTTCTTCAAACCGG
3_known	A15	Tb927.10.10700_for	GGGGACAAGTTTGTACAAAAAAGCAGGCTATGAGCTATATCGAAACCGGATGGG	Tb927.10.10700_rev	GGGGACCACTTTGTACAAGAAAGCTGGGTTTATCTTAAATTCCGTCGCGGCC
3_known	A16	Tb927.11.3320_for	GGGGACAAGTTTGTACAAAAAAGCAGGCTATGCACGAACAAATCCAGAAGCT	Tb927.11.3320_rev	GGGGACCACTTTGTACAAGAAAGCTGGGTCTACAACACTTTACGCATTTCCTCAGC
3_known	A17	Tb927.9.9820_for	GGGGACAAGTTTGTACAAAAAAGCAGGCTATGGAGGCGGAAACGAGCA	Tb927.9.9820_rev	GGGGACCACTTTGTACAAGAAAGCTGGGTCTACACATTATTCTTCTTTGCATCCGC
3_known	A18	Tb927.9.6290_for	GGGGACAAGTTTGTACAAAAAAGCAGGCTATGGCTACCCGCGACGTT	Tb927.9.6290_rev	GGGGACCACTTTGTACAAGAAAGCTGGGTCTACTTCGACTTCTCCAGTTTGATGAG
3_known	A19	Tb927.9.9000_for	GGGGACAAGTTTGTACAAAAAAGCAGGCTATGTCCGAGTCAAGGAATGCAGA	Tb927.9.9000_rev	GGGGACCACTTTGTACAAGAAAGCTGGGTCTAGAGTGGTGCGCGCAAG
3_known	A20	Tb927.3.790_for	GGGGACAAGTTTGTACAAAAAAGCAGGCTATGGCCGAGCGGCGGCAG	Tb927.3.790_rev	GGGGACCACTTTGTACAAGAAAGCTGGGTCTAGCGCTTCCATAACCGCAG
3_known	A21	Tb927.11.3030_for	GGGGACAAGTTTGTACAAAAAAGCAGGCTATGCAGGGGCAAGCCGAC	Tb927.11.3030_rev	GGGGACCACTTTGTACAAGAAAGCTGGGTCTATGTGCGGAACAGAGAGGATAAC
3_known	A22	Tb927.9.3360_for	GGGGACAAGTTTGTACAAAAAAGCAGGCTATGAACCGAGCGGCTCGC	Tb927.9.3360_rev	GGGGACCACTTTGTACAAGAAAGCTGGGTTCAGCCGCTCCCAGCCAC
3_known	A23	Tb927.11.18280_for	GGGGACAAGTTTGTACAAAAAAGCAGGCTATGGTTTCACTAACAGCACTGTTTTTC	Tb927.11.18280_rev	GGGGACCACTTTGTACAAGAAAGCTGGGTTCAACTTCTGCGCTGTCGAATG
3_known	A24	Tb927.9.10080_for	GGGGACAAGTTTGTACAAAAAAGCAGGCTATGGCTTCGCTCACACGAC	Tb927.9.10080_rev	GGGGACCACTTTGTACAAGAAAGCTGGGTCTACTTGCCACTCTCCGACAATC
3_known	B1	Tb927.6.4650_for	GGGGACAAGTTTGTACAAAAAAGCAGGCTATGAGGATAATTCTCAAGAGCGTGC	Tb927.6.4650_rev	GGGGACCACTTTGTACAAGAAAGCTGGGTTCAGTCGGTAATCCCGAGAAAGT
3_known	B2	Tb927.7.3350_for	GGGGACAAGTTTGTACAAAAAAGCAGGCTATGTGCAGCTGTTGCTGTGAG	Tb927.7.3350_rev	GGGGACCACTTTGTACAAGAAAGCTGGGTTCAAGCAATAGGTTCGTTATCATGTGT
3_known	B3	Tb927.9.12300_for	GGGGACAAGTTTGTACAAAAAAGCAGGCTATGACGGGAGAAGGCACACT	Tb927.9.12300_rev	GGGGACCACTTTGTACAAGAAAGCTGGGTTCAGCACAGGCCACCTGC
3_known	B4	Tb927.1.1140_for	GGGGACAAGTTTGTACAAAAAAGCAGGCTATGGCGACTATTTACCACGAGGG	Tb927.1.1140_rev	GGGGACCACTTTGTACAAGAAAGCTGGGTCTAGCGCGGTGCATAACCAC
3_known	B5	Tb927.3.1030_for	GGGGACAAGTTTGTACAAAAAAGCAGGCTATGCCGGCAAAAAAGAAAGGAGG	Tb927.3.1030_rev	GGGGACCACTTTGTACAAGAAAGCTGGGTTCATCTCCGCAGTTCCTCCC
3_known	B6	Tb927.6.930_for	GGGGACAAGTTTGTACAAAAAAGCAGGCTATGGCCGTGGACCCAAGG	Tb927.6.930_rev	GGGGACCACTTTGTACAAGAAAGCTGGGTCTATTGGATAGATCTGTCAACAGAAAACAT
3_known	B7	Tb927.3.4340_for	GGGGACAAGTTTGTACAAAAAAGCAGGCTATGGAGGCGGAGTTGGGTC	Tb927.3.4340_rev	GGGGACCACTTTGTACAAGAAAGCTGGGTTTATAGGGATTTGTTTGTGTAGACAGCC
3_known	B8	Tb927.11.10010_for	GGGGACAAGTTTGTACAAAAAAGCAGGCTATGTTGGAGCGGGAGCGG	Tb927.11.10010_rev	GGGGACCACTTTGTACAAGAAAGCTGGGTTTAATGAGATGTTTCACCCGCCTC
3_known	B9	Tb927.7.4570_for	GGGGACAAGTTTGTACAAAAAAGCAGGCTATGGTTCATCGAAAACTTATCATCGACA	Tb927.7.4570_rev	GGGGACCACTTTGTACAAGAAAGCTGGGTTTACGCGACATATTCTACGATATCCCG
3_known	B10	Tb927.10.2550_for	GGGGACAAGTTTGTACAAAAAAGCAGGCTATGGTAATTTTCTTTGGTCGAGCACA	Tb927.10.2550_rev	GGGGACCACTTTGTACAAGAAAGCTGGGTTCATGAAACTGCATCTACCCCTGT
3_known	B11	Tb927.2.6000_for	GGGGACAAGTTTGTACAAAAAAGCAGGCTATGTTTGGTGGTGTAAAGTGGTCAC	Tb927.2.6000_rev	GGGGACCACTTTGTACAAGAAAGCTGGGTTTATGACCTTGCGGTTTGGTTGG
3_known	B12	Tb927.6.4670_for	GGGGACAAGTTTGTACAAAAAAGCAGGCTATGATATACTCTGGTGAGATAGAAAATGGC	Tb927.6.4670_rev	GGGGACCACTTTGTACAAGAAAGCTGGGTTTAATAATCCGGATTGCTGAGATCACAG
3_known	B13	Tb927.8.7530_for	GGGGACAAGTTTGTACAAAAAAGCAGGCTATGCGACGCAGTGTTGTGAC	Tb927.8.7530_rev	GGGGACCACTTTGTACAAGAAAGCTGGGTTTACTTTTTCGCTGCCACTCCC
3_known	B14	Tb927.6.4300_for	GGGGACAAGTTTGTACAAAAAAGCAGGCTATGACTATTAAAGTTGGCATCAATGGTTTC	Tb927.6.4300_rev	GGGGACCACTTTGTACAAGAAAGCTGGGTTTATAGCTTTGCTGCACGGTCC
3_known	B15	Tb927.10.13900_for	GGGGACAAGTTTGTACAAAAAAGCAGGCTATGCCTTCCGTTAAATGGCTTTCG	Tb927.10.13900_rev	GGGGACCACTTTGTACAAGAAAGCTGGGTCTAATGATGTTCCTCTCCTTTCGAAGTT
3_known	B16	Tb927.9.5890_for	GGGGACAAGTTTGTACAAAAAAGCAGGCTATGCACCGTGCTAATATTATATTACGCA	Tb927.9.5890_rev	GGGGACCACTTTGTACAAGAAAGCTGGGTTCACAATTCCCGTGTCAGGAGG
3_known	B17	Tb927.6.2720_for	GGGGACAAGTTTGTACAAAAAAGCAGGCTATGCCGCACTCCATGGACA	Tb927.6.2720_rev	GGGGACCACTTTGTACAAGAAAGCTGGGTTTACAAAAGTTTCCATTGATTATTGAGAAC
3_known	B18	Tb927.10.6950_for	GGGGACAAGTTTGTACAAAAAAGCAGGCTATGTCGGCCGGATCTCGT	Tb927.10.6950_rev	GGGGACCACTTTGTACAAGAAAGCTGGGTTTAGCACGACAGCTCTTCCCC
3_known	B21	Tb927.11.11210_for	GGGGACAAGTTTGTACAAAAAAGCAGGCTATGAAGACTAAGTTCTATACATATAAGAAC	Tb927.11.11210_rev	GGGGACCACTTTGTACAAGAAAGCTGGGTTCACCTTCTGTTTCTGCGCTC
3_known	B22	Tb927.9.14400_for	GGGGACAAGTTTGTACAAAAAAGCAGGCTATGGAACTGACACTGACTAGACGG	Tb927.9.14400_rev	GGGGACCACTTTGTACAAGAAAGCTGGGTTTAAGCAAAACCAGTGGACTGGTT
3_known	B23	Tb927.11.10230_for	GGGGACAAGTTTGTACAAAAAAGCAGGCTATGAGTCTTCGCGAGGACTGG	Tb927.11.10230_rev	GGGGACCACTTTGTACAAGAAAGCTGGGTCTACGGCTGGTCCGGCGG
3_known	B24	Tb927.3.3610_for	GGGGACAAGTTTGTACAAAAAAGCAGGCTATGCCTTCATTTGTTCTCCACCC	Tb927.3.3610_rev	GGGGACCACTTTGTACAAGAAAGCTGGGTCTACCTGACTGGGGGCACG
3_known	C2	Tb927.11.14260_for	GGGGACAAGTTTGTACAAAAAAGCAGGCTATGTATCAAGGACGTAGTCAACAGC	Tb927.11.14260_rev	GGGGACCACTTTGTACAAGAAAGCTGGGTCTAGTCAAGGTCCTGGACAATTGAC
3_known	C3	Tb927.11.11660_for	GGGGACAAGTTTGTACAAAAAAGCAGGCTATGCTAGCACCTAGAGGCTCTG	Tb927.11.11660_rev	GGGGACCACTTTGTACAAGAAAGCTGGGTTTAGATTGTACCAGTGTTCACACCAC
3_known	C4	Tb927.11.16480_for	GGGGACAAGTTTGTACAAAAAAGCAGGCTATGCGTTGCACGCTTCCG	Tb927.11.16480_rev	GGGGACCACTTTGTACAAGAAAGCTGGGTCTAGTACGGCTCTGTGGGATGC
3_known	C5	Tb927.3.2150_for	GGGGACAAGTTTGTACAAAAAAGCAGGCTATGGGAGGACAAAGCAGTGTAACC	Tb927.3.2150_rev	GGGGACCACTTTGTACAAGAAAGCTGGGTTTACCATGTGCTAGTAGCGTTAAGTTC
3_known	C6	Tb927.10.15490_for	GGGGACAAGTTTGTACAAAAAAGCAGGCTATGTCAGCACGTCGACTGGC	Tb927.10.15490_rev	GGGGACCACTTTGTACAAGAAAGCTGGGTTCAGCTGGTTTCGGCGGT
3_known	C7	Tb927.9.14410_for	GGGGACAAGTTTGTACAAAAAAGCAGGCTATGCTAACGTTCGAGGGAAGCA	Tb927.9.14410_rev	GGGGACCACTTTGTACAAGAAAGCTGGGTCTATCCACTCTTCTTCCAGACATTCAC
3_known	C8	Tb11.02.5400_for	GGGGACAAGTTTGTACAAAAAAGCAGGCTATGAAGGACCAACGAACCACTATTC	Tb11.02.5400_rev	GGGGACCACTTTGTACAAGAAAGCTGGGTTCACTTCCCAATTAAACCGAGTTTGG
3_known	C9	Tb927.9.10370_for	GGGGACAAGTTTGTACAAAAAAGCAGGCTATGCAAGGCGCCAATTTAAAAGTG	Tb927.9.10370_rev	GGGGACCACTTTGTACAAGAAAGCTGGGTCTACTGCTCCGCGGCAAC
3_known	C10	Tb927.9.12670_for	GGGGACAAGTTTGTACAAAAAAGCAGGCTATGGTAGCAACTATTACCTTCAAGTTGC	Tb927.9.12670_rev	GGGGACCACTTTGTACAAGAAAGCTGGGTTCAGTCCCGCGCACGCTT

3_known	C11	Tb927.6.670_for	GGGGACAAGTTTGTACAAAAAAGCAGGCTATGCAGTACCAACGAGCAGC	Tb927.6.670_rev	GGGGACCACTTTGTACAAGAAAGCTGGGTTCACGTTTCTGTTTTTATCTTCTTCACAC
3_known	C12	Tb927.9.9320_for	GGGGACAAGTTTGTACAAAAAAGCAGGCTATGCAAAGTACGCAATATTAGGAAAGAAA	Tb927.9.9320_rev	GGGGACCACTTTGTACAAGAAAGCTGGGTTCACCCTTGAAGTTTGGGCAGA
3_known	C13	Tb927.9.6350_for	GGGGACAAGTTTGTACAAAAAAGCAGGCTATGGCTGAGTATGACATTAATTTGGAGG	Tb927.9.6350_rev	GGGGACCACTTTGTACAAGAAAGCTGGGTCTACAGCCAAAATCTTCCATAGCTCA
3_known	C14	Tb927.5.810_for	GGGGACAAGTTTGTACAAAAAAGCAGGCTATGAGCACTGCAACATCTGCA	Tb927.5.810_rev	GGGGACCACTTTGTACAAGAAAGCTGGGTTCACAAGGAAAGAAACATATGCAGACC
3_known	C15	Tb927.3.3380_for	GGGGACAAGTTTGTACAAAAAAGCAGGCTATGGTCGCAGAGTGCGCT	Tb927.3.3380_rev	GGGGACCACTTTGTACAAGAAAGCTGGGTCTATGGTATAAGTCTCTGCCACTGCT
3_known	C16	Tb927.11.15280_for	GGGGACAAGTTTGTACAAAAAAGCAGGCTATGGAGGAGGTGGTGGTACCG	Tb927.11.15280_rev	GGGGACCACTTTGTACAAGAAAGCTGGGTTCAAGGCACCGTAAGGTCCTC
3_known	C17	Tb927.9.4940_for	GGGGACAAGTTTGTACAAAAAAGCAGGCTATGAGGCGGAGCAGGGTAC	Tb927.9.4940_rev	GGGGACCACTTTGTACAAGAAAGCTGGGTCTACAGTGCAAGCACAGAGAGAG
3_known	C18	Tb927.10.16030_for	GGGGACAAGTTTGTACAAAAAAGCAGGCTATGGACATACCACAAGAACTGGCT	Tb927.10.16030_rev	GGGGACCACTTTGTACAAGAAAGCTGGGTCTAGTCACCCTTTGGACCGGG
3_known	C19	Tb927.10.6870_for	GGGGACAAGTTTGTACAAAAAAGCAGGCTATGATATTTTTACTGTTCATAAGCTTTATT	Tb927.10.6870_rev	GGGGACCACTTTGTACAAGAAAGCTGGGTTCATTTCTCCGAACTACTCCTGCT
3_known	C20	Tb927.10.240_for	GGGGACAAGTTTGTACAAAAAAGCAGGCTATGTCTTTGCTGCTGTCGGG	Tb927.10.240_rev	GGGGACCACTTTGTACAAGAAAGCTGGGTTCAAGCTGCCTCGCCGCC
3_known	C21	Tb927.8.750_for	GGGGACAAGTTTGTACAAAAAAGCAGGCTATGGAGGGTTTCTACGGTGTTGA	Tb927.8.750_rev	GGGGACCACTTTGTACAAGAAAGCTGGGTTTAGTAACGACCCCTCCCAGAGT
3_known	C22	Tb927.6.4630_for	GGGGACAAGTTTGTACAAAAAAGCAGGCTATGCCGAACGCTGATGCTC	Tb927.6.4630_rev	GGGGACCACTTTGTACAAGAAAGCTGGGTCTACACAGGGGAAAAAGTCCCATATC
3_known	C23	Tb927.6.3540_for	GGGGACAAGTTTGTACAAAAAAGCAGGCTATGTCGCTGCCAGCTCCT	Tb927.6.3540_rev	GGGGACCACTTTGTACAAGAAAGCTGGGTCTAATTCACTCTACCCTCACCATCATAAC
3_known	C24	Tb927.10.15180_for	GGGGACAAGTTTGTACAAAAAAGCAGGCTATGCCGCCGAAGCGAAAA	Tb927.10.15180_rev	GGGGACCACTTTGTACAAGAAAGCTGGGTTTACTGGTGTTTGCATTGTTGCTG
3_known	D1	Tb927.11.16000_for	GGGGACAAGTTTGTACAAAAAAGCAGGCTATGAGTGCCAATAAACACGGACAT	Tb927.11.16000_rev	GGGGACCACTTTGTACAAGAAAGCTGGGTCTATACAGTTTCCGTATTATCGGGATCCC
3_known	D2	Tb927.9.8210_for	GGGGACAAGTTTGTACAAAAAAGCAGGCTATGCGTATACATCATGCATTCTTTAGGC	Tb927.9.8210_rev	GGGGACCACTTTGTACAAGAAAGCTGGGTCTACTGAGGGGCGTTATCCTTCA
3_known	D3	Tb927.11.9430_for	GGGGACAAGTTTGTACAAAAAAGCAGGCTATGGAGGTTTTGTGTAATGGTAGACAAT	Tb927.11.9430_rev	GGGGACCACTTTGTACAAGAAAGCTGGGTCTACTTGGCAGGCGTAGCTCC
3_known	D4	Tb927.10.13540_for	GGGGACAAGTTTGTACAAAAAAGCAGGCTATGAATTTTTCACCATTGGCGAATCC	Tb927.10.13540_rev	GGGGACCACTTTGTACAAGAAAGCTGGGTCTACTCGCCCGATCCGCT
3_known	D5	Tb927.2.4590_for	GGGGACAAGTTTGTACAAAAAAGCAGGCTATGACTTTCCTCGCCAAGGACC	Tb927.2.4590_rev	GGGGACCACTTTGTACAAGAAAGCTGGGTTTAAGAAGTGGTACCAACAATGCGT
3_known	D6	Tb927.2.4610_for	GGGGACAAGTTTGTACAAAAAAGCAGGCTATGACTTTCCTCGCCAAAAAAATTTTTGT	Tb927.2.4610_rev	GGGGACCACTTTGTACAAGAAAGCTGGGTTTAAGAAGTGGTACCAACAATGCGT
3_known	D7	Tb927.7.3360_for	GGGGACAAGTTTGTACAAAAAAGCAGGCTATGGTAATGCAAATGTTTATGCAGGTATAT	Tb927.7.3360_rev	GGGGACCACTTTGTACAAGAAAGCTGGGTTCACTTCTGGCGCTTGTAAGTC
3_known	D8	Tb927.8.4440_for	GGGGACAAGTTTGTACAAAAAAGCAGGCTATGTCGGATGTACCGTGGGC	Tb927.8.4440_rev	GGGGACCACTTTGTACAAGAAAGCTGGGTTCAACCACTTTGGTGTGCCC
3_known	D9	Tb927.2.4240_for	GGGGACAAGTTTGTACAAAAAAGCAGGCTATGCACACTTCGTCATTGCAACT	Tb927.2.4240_rev	GGGGACCACTTTGTACAAGAAAGCTGGGTTTATCGCGAGGCTCCATGCT
3_known	D10	Tb927.7.300_for	GGGGACAAGTTTGTACAAAAAAGCAGGCTATGGTGTGGAGTGGGCATAAATACA	Tb927.7.300_rev	GGGGACCACTTTGTACAAGAAAGCTGGGTTCATGTGCACGAGGCGTG
3_known	D11	Tb927.10.6800_for	GGGGACAAGTTTGTACAAAAAAGCAGGCTATGTCCAATTTGCAGAAGATAAATGACATC	Tb927.10.6800_rev	GGGGACCACTTTGTACAAGAAAGCTGGGTCTAAACCTTCTTCACAACCTGCACC
3_known	D12	Tb927.5.3960_for	GGGGACAAGTTTGTACAAAAAAGCAGGCTATGGAGTCCGGGGGGTTTG	Tb927.5.3960_rev	GGGGACCACTTTGTACAAGAAAGCTGGGTTTATTTTAACTCGAGCTCAATGGTGTAGAA
3_known	D13	Tb927.8.5690_for	GGGGACAAGTTTGTACAAAAAAGCAGGCTATGCACCGCGGACCGCCG	Tb927.8.5690_rev	GGGGACCACTTTGTACAAGAAAGCTGGGTCTACAGCAAATTCATTGCTGCAAGT
3_known	D14	Tb927.8.7440_for	GGGGACAAGTTTGTACAAAAAAGCAGGCTATGAGTGAGCGGAAAGGCAAC	Tb927.8.7440_rev	GGGGACCACTTTGTACAAGAAAGCTGGGTTCAGTCCGCAGGCTTGTCG
3_known	D15	Tb927.10.4330_for	GGGGACAAGTTTGTACAAAAAAGCAGGCTATGAGGCGGTGGGCCAGT	Tb927.10.4330_rev	GGGGACCACTTTGTACAAGAAAGCTGGGTCTAGAACGCGATCACTTCCTTCAC
3_known	D16	Tb927.11.11110_for	GGGGACAAGTTTGTACAAAAAAGCAGGCTATGCACCGGGGGAGCCAA	Tb927.11.11110_rev	GGGGACCACTTTGTACAAGAAAGCTGGGTTCACTCTAGGGACGAAATCGGG
3_known	D17	Tb927.1.1120_for	GGGGACAAGTTTGTACAAAAAAGCAGGCTATGTCTCGGGGTGGAATATTTGGT	Tb927.1.1120_rev	GGGGACCACTTTGTACAAGAAAGCTGGGTTCACCGCTTTTTGTACAGTGACG
3_known	D18	Tb927.11.5010_for	GGGGACAAGTTTGTACAAAAAAGCAGGCTATGTCGGATCTCCGTCGAGTT	Tb927.11.5010_rev	GGGGACCACTTTGTACAAGAAAGCTGGGTTTACACCGTCCAGTGCTGCA
3_known	D19	Tb927.9.2670_for	GGGGACAAGTTTGTACAAAAAAGCAGGCTATGGGTTCCAGTATGTCTTGTCTGG	Tb927.9.2670_rev	GGGGACCACTTTGTACAAGAAAGCTGGGTCTACTCGGGGGAGAAAAAGCTCT
3_known	D20	Tb927.8.2050_for	GGGGACAAGTTTGTACAAAAAAGCAGGCTATGAGGGCCGTCATTCTTGTTG	Tb927.8.2050_rev	GGGGACCACTTTGTACAAGAAAGCTGGGTTTACATGACCACTTCTGGTTCGGG
3_known	D21	Tb927.11.14610_for	GGGGACAAGTTTGTACAAAAAAGCAGGCTATGAGGTTCATGCTAAGGTTAGTTATGTTG	Tb927.11.14610_rev	GGGGACCACTTTGTACAAGAAAGCTGGGTTTACCACAATACCACAGAGAATACAAAAAA
3_known	D22	Tb927.6.4460_for	GGGGACAAGTTTGTACAAAAAAGCAGGCTATGTCCTCTTGCAAGGACTCTCT	Tb927.6.4460_rev	GGGGACCACTTTGTACAAGAAAGCTGGGTTCATTCCTTCGCTCCAGAAGCT
3_known	D23	Tb927.9.6230_for	GGGGACAAGTTTGTACAAAAAAGCAGGCTATGGCTACCCGCGACGTT	Tb927.9.6230_rev	GGGGACCACTTTGTACAAGAAAGCTGGGTCTACAAGTTCGAACCAACAAATGGTTT
3_known	D24	Tb927.9.10970_for	GGGGACAAGTTTGTACAAAAAAGCAGGCTATGAGCGCTGAGCGGGTT	Tb927.9.10970_rev	GGGGACCACTTTGTACAAGAAAGCTGGGTCTAAATTAGCTGTTCTCTATGGCGAAGC
3_known	E1	Tb927.7.270_for	GGGGACAAGTTTGTACAAAAAAGCAGGCTATGTCCTCTATCGGTGGCTTCAA	Tb927.7.270_rev	GGGGACCACTTTGTACAAGAAAGCTGGGTTCAAATATCCCTATCGGGGTTTATGTCA
3_known	E3	Tb927.10.7940_for	GGGGACAAGTTTGTACAAAAAAGCAGGCTATGCCTGCCGTTGCAGAC	Tb927.10.7940_rev	GGGGACCACTTTGTACAAGAAAGCTGGGTCTATGACTTGACCTCCTCCATTGC
3_known	E4	Tb927.10.10230_for	GGGGACAAGTTTGTACAAAAAAGCAGGCTATGCGAAGAACAGTGGCATTTTTAGT	Tb927.10.10230_rev	GGGGACCACTTTGTACAAGAAAGCTGGGTTTATACCCCACAATCCTTCAACTCTTCC
3_known	E5	Tb927.11.14880_for	GGGGACAAGTTTGTACAAAAAAGCAGGCTATGTCTGCTGACCCCACATCT	Tb927.11.14880_rev	GGGGACCACTTTGTACAAGAAAGCTGGGTCTACTCCTGAGCCTCCGCAT
3_known	E6	Tb927.8.3550_for	GGGGACAAGTTTGTACAAAAAAGCAGGCTATGCACAAAAGTAATCAAGAGTTGTGC	Tb927.8.3550_rev	GGGGACCACTTTGTACAAGAAAGCTGGGTCTACGCGCCTTTCGGTATTTGG
3_known	E7	Tb927.5.1310_for	GGGGACAAGTTTGTACAAAAAAGCAGGCTATGATGCGCCGCGTACTC	Tb927.5.1310_rev	GGGGACCACTTTGTACAAGAAAGCTGGGTCTATAATGCAACAGCAGTGAAGTGC
3_known	E8	Tb927.11.1010_for	GGGGACAAGTTTGTACAAAAAAGCAGGCTATGCGCCGCTTTGTTTCCC	Tb927.11.1010_rev	GGGGACCACTTTGTACAAGAAAGCTGGGTTCAAAAAAGGCGCATCCGCT
3_known	E9	Tb927.10.12290_for	GGGGACAAGTTTGTACAAAAAAGCAGGCTATGGTTGGACAAATTTTGAGTAGGAGG	Tb927.10.12290_rev	GGGGACCACTTTGTACAAGAAAGCTGGGTTCACCGCTTGCCGCATGT
3_known	E10	Tb927.4.980_for	GGGGACAAGTTTGTACAAAAAAGCAGGCTATGGCAGTAGTGGAGATTGGATGG	Tb927.4.980_rev	GGGGACCACTTTGTACAAGAAAGCTGGGTTCAGTAAAATAACTTGCGGTACAACACC
3_known	E11	Tb927.5.3910_for	GGGGACAAGTTTGTACAAAAAAGCAGGCTATGCGTAGGAACAATCGCACAC	Tb927.5.3910_rev	GGGGACCACTTTGTACAAGAAAGCTGGGTTCAAACTTCTTCATCCAGTTTATGTTTCTT
3_known	E12	Tb927.10.5620_for	GGGGACAAGTTTGTACAAAAAAGCAGGCTATGTCCAAGCGTGTTGAAGTTCT	Tb927.10.5620_rev	GGGGACCACTTTGTACAAGAAAGCTGGGTTTAGTATGTGTTGCCAGCAACGT
3_known	E13	Tb927.11.3220_for	GGGGACAAGTTTGTACAAAAAAGCAGGCTATGTCGTCTGAAAAAGTCGCTGG	Tb927.11.3220_rev	GGGGACCACTTTGTACAAGAAAGCTGGGTTTATTTTCCACCAAGAGGGGTGGT
3_known	E14	Tb927.7.6470_for	GGGGACAAGTTTGTACAAAAAAGCAGGCTATGAGCGCCAACACTCAGC	Tb927.7.6470_rev	GGGGACCACTTTGTACAAGAAAGCTGGGTTTAAGTGCGCCACACTACGTTG
3_known	E15	Tb927.2.2430_for	GGGGACAAGTTTGTACAAAAAAGCAGGCTATGTCCCGCAGCGGTGGT	Tb927.2.2430_rev	GGGGACCACTTTGTACAAGAAAGCTGGGTCTAACCTCTGCGCCTCGGG
3_known	E16	Tb927.1.3010_for	GGGGACAAGTTTGTACAAAAAAGCAGGCTATGCGGACGTTCCTGGCA	Tb927.1.3010_rev	GGGGACCACTTTGTACAAGAAAGCTGGGTTTACAGCGGAACAAACTGTTTCTGA
3_known	E17	Tb927.3.4860_for	GGGGACAAGTTTGTACAAAAAAGCAGGCTATGAAAAGGAAGGGGATTGCGTT	Tb927.3.4860_rev	GGGGACCACTTTGTACAAGAAAGCTGGGTCTACAGAACACCTAGCATTTCCAATTTTAT
3_known	E18	Tb927.7.7080_for	GGGGACAAGTTTGTACAAAAAAGCAGGCTATGCACAGTTCTCTCATAATACTGCG	Tb927.7.7080_rev	GGGGACCACTTTGTACAAGAAAGCTGGGTTCACGCAGCCACGAAGTCC
3_known	E19	Tb927.10.3230_for	GGGGACAAGTTTGTACAAAAAAGCAGGCTATGAACAGTAATGCTCCGCTTATCTTT	Tb927.10.3230_rev	GGGGACCACTTTGTACAAGAAAGCTGGGTTCAATCCTCATCATCGCTAACTAGTAGG
3_known	E20	Tb927.11.8190_for	GGGGACAAGTTTGTACAAAAAAGCAGGCTATGAACACTCGCACCAAAAATAAGAAAC	Tb927.11.8190_rev	GGGGACCACTTTGTACAAGAAAGCTGGGTCTAGTCCCTAACGTCTCCCACAC
3_known	E21	Tb927.9.15380_for	GGGGACAAGTTTGTACAAAAAAGCAGGCTATGTCCCGGCGGGTGTTT	Tb927.9.15380_rev	GGGGACCACTTTGTACAAGAAAGCTGGGTTTACATTCGTGGGGCCAAGTCT
3_known	E22	Tb927.10.11800_for	GGGGACAAGTTTGTACAAAAAAGCAGGCTATGGGGTTGCTTAAGGTGGAGA	Tb927.10.11800_rev	GGGGACCACTTTGTACAAGAAAGCTGGGTCTAGAGCTCTACACCGAGGTCAG
3_known	E23	Tb927.10.10210_for	GGGGACAAGTTTGTACAAAAAAGCAGGCTATGACAACCATGGGGAAGACAATAAAA	Tb927.10.10210_rev	GGGGACCACTTTGTACAAGAAAGCTGGGTTTACACCATCGCAGCGACAAG
3_known	E24	Tb927.3.2290_for	GGGGACAAGTTTGTACAAAAAAGCAGGCTATGTCCGGGCCGCCTAAG	Tb927.3.2290_rev	GGGGACCACTTTGTACAAGAAAGCTGGGTTTACACGCAGGTACTGATATCTTTCTCC
3_known	F1	Tb927.4.1300_for	GGGGACAAGTTTGTACAAAAAAGCAGGCTATGTCGACTGCTCTGTTCGTTC	Tb927.4.1300_rev	GGGGACCACTTTGTACAAGAAAGCTGGGTTCAGCAACCGCCGTCCTT
3_known	F2	Tb927.9.9020_for	GGGGACAAGTTTGTACAAAAAAGCAGGCTATGGGCTTACTTGAACGCCG	Tb927.9.9020_rev	GGGGACCACTTTGTACAAGAAAGCTGGGTTTATTCGTTAGACGTTTTAACCACTATTTG
3_known	F3	Tb927.1.990_for	GGGGACAAGTTTGTACAAAAAAGCAGGCTATGAGTGGCGAAAACATTAAAAATTTGC	Tb927.1.990_rev	GGGGACCACTTTGTACAAGAAAGCTGGGTCTAGTCGGAGGCAAAGAGACGG
3_known	F4	Tb927.7.5920_for	GGGGACAAGTTTGTACAAAAAAGCAGGCTATGCACGGTGACGGTTTGG	Tb927.7.5920_rev	GGGGACCACTTTGTACAAGAAAGCTGGGTTTACGTGTCTATGCGAGGATGAGC
3_known	F5	Tb927.10.14180_for	GGGGACAAGTTTGTACAAAAAAGCAGGCTATGCTTCATTCAACTAGATACCCGTCG	Tb927.10.14180_rev	GGGGACCACTTTGTACAAGAAAGCTGGGTTCAGTGAACCCGCTGATTTTGG
3_known	F6	Tb927.6.750_for	GGGGACAAGTTTGTACAAAAAAGCAGGCTATGGTGGGGAAATACTTGTTAATTTCGC	Tb927.6.750_rev	GGGGACCACTTTGTACAAGAAAGCTGGGTTCACAAGTCGTCTAGAACATCGTTC
3_known	F7	Tb927.11.9670_for	GGGGACAAGTTTGTACAAAAAAGCAGGCTATGTCAACTTCTCTACGGCACAC	Tb927.11.9670_rev	GGGGACCACTTTGTACAAGAAAGCTGGGTTCATCCCCTATAATAGCGCGGG
3_known	F8	Tb927.10.160_for	GGGGACAAGTTTGTACAAAAAAGCAGGCTATGGCTTTAGTTCGTAGGATAGGATCA	Tb927.10.160_rev	GGGGACCACTTTGTACAAGAAAGCTGGGTTCAAACACTAGAAAGCCTTCCTTGTT
3_known	F9	Tb927.11.3150_for	GGGGACAAGTTTGTACAAAAAAGCAGGCTATGACTGAGTACAAGAAACTTTGCGC	Tb927.11.3150_rev	GGGGACCACTTTGTACAAGAAAGCTGGGTCTAAAACTTGCAAGCTTCCAAAGAGTG
3_known	F10	Tb927.8.7150_for	GGGGACAAGTTTGTACAAAAAAGCAGGCTATGACACAAAGGAAAGTGTACCCG	Tb927.8.7150_rev	GGGGACCACTTTGTACAAGAAAGCTGGGTTTAGCACAGAACCATAGCGCG
3_known	F11	Tb927.9.8850_for	GGGGACAAGTTTGTACAAAAAAGCAGGCTATGTCGGACGAGGAACAAACTG	Tb927.9.8850_rev	GGGGACCACTTTGTACAAGAAAGCTGGGTTCAAAAGCATTTGCTGTGTACGATG
3_known	F12	Tb927.9.8880_for	GGGGACAAGTTTGTACAAAAAAGCAGGCTATGTCGGACGAGGAACAAACTG	Tb927.9.8880_rev	GGGGACCACTTTGTACAAGAAAGCTGGGTTTAAAAGCATTTGCTGTGTACGATGC
3_known	F13	Tb927.7.1300_for	GGGGACAAGTTTGTACAAAAAAGCAGGCTATGACCCCGTTTTCATGTCGC	Tb927.7.1300_rev	GGGGACCACTTTGTACAAGAAAGCTGGGTTTACTCACCTTTGATTGCCGAAAGAA
3_known	F14	Tb927.2.3370_for	GGGGACAAGTTTGTACAAAAAAGCAGGCTATGGTGTCGAAGGGTTTACAGATGG	Tb927.2.3370_rev	GGGGACCACTTTGTACAAGAAAGCTGGGTTCAGCATTTCCCACGAGCG
3_known	F15	Tb927.10.9430_for	GGGGACAAGTTTGTACAAAAAAGCAGGCTATGTCTTCTTCAACGTGTGCCTT	Tb927.10.9430_rev	GGGGACCACTTTGTACAAGAAAGCTGGGTTTATGGAGTGGCACCGGTTGC
3_known	F16	Tb927.3.3960_for	GGGGACAAGTTTGTACAAAAAAGCAGGCTATGATGCCGTGCAAGTCATTTCC	Tb927.3.3960_rev	GGGGACCACTTTGTACAAGAAAGCTGGGTTTAATCCACTGGTGAAGGCGGC
3_known	F17	Tb927.10.13720_for	GGGGACAAGTTTGTACAAAAAAGCAGGCTATGGCGCATGTTGGACAGC	Tb927.10.13720_rev	GGGGACCACTTTGTACAAGAAAGCTGGGTTCAACATTTATTTTCAACCTCTTCATCAAC
3_known	F18	Tb927.11.15760_for	GGGGACAAGTTTGTACAAAAAAGCAGGCTATGAGGAGCAGCGGTGATCT	Tb927.11.15760_rev	GGGGACCACTTTGTACAAGAAAGCTGGGTCTACGAGTTTTGAACAACCTGCGC
3_known	F19	Tb927.3.4920_for	GGGGACAAGTTTGTACAAAAAAGCAGGCTATGTTTCCGGACCTGATTCCCT	Tb927.3.4920_rev	GGGGACCACTTTGTACAAGAAAGCTGGGTCTAATTTTTTGCAATCACCTCTGAAGGC
3_known	F20	Tb927.9.4390_for	GGGGACAAGTTTGTACAAAAAAGCAGGCTATGAAAATTACCTGTACGGATGAAAGTGG	Tb927.9.4390_rev	GGGGACCACTTTGTACAAGAAAGCTGGGTTCACTCCTCCATCAGAAGTGACAT
3_known	F21	Tb927.10.5270_for	GGGGACAAGTTTGTACAAAAAAGCAGGCTATGACCGGGTTGAAGATTGACATC	Tb927.10.5270_rev	GGGGACCACTTTGTACAAGAAAGCTGGGTTCACTGCACCAACTTATTTAGTTCCTC
3_known	F22	Tb927.10.2910_for	GGGGACAAGTTTGTACAAAAAAGCAGGCTATGGATGCGATCCCACCTGA	Tb927.10.2910_rev	GGGGACCACTTTGTACAAGAAAGCTGGGTCTACCTCCTGTATCGCCCTTCA
3_known	F23	Tb927.11.12880_for	GGGGACAAGTTTGTACAAAAAAGCAGGCTATGGAGGGTGGTGCCAAGT	Tb927.11.12880_rev	GGGGACCACTTTGTACAAGAAAGCTGGGTCTAACGGAGTTCGTAACGGCC
3_known	F24	Tb927.10.12700_for	GGGGACAAGTTTGTACAAAAAAGCAGGCTATGCTTAAGTGTGTCAGCCGAG	Tb927.10.12700_rev	GGGGACCACTTTGTACAAGAAAGCTGGGTTCACGGCTTGGCATAAACTGTT
3_known	G1	Tb927.10.2390_for	GGGGACAAGTTTGTACAAAAAAGCAGGCTATGACTGATGTCTCGCTTCGACC	Tb927.10.2390_rev	GGGGACCACTTTGTACAAGAAAGCTGGGTTCATCCCTTTCCGGCGGT
3_known	G2	Tb927.6.4220_for	GGGGACAAGTTTGTACAAAAAAGCAGGCTATGGTAACAGCAAATGGCTCCTC	Tb927.6.4220_rev	GGGGACCACTTTGTACAAGAAAGCTGGGTTTACTGATAGTGCTGAATCTCTTTCCATAT
3_known	G3	Tb927.1.780_for	GGGGACAAGTTTGTACAAAAAAGCAGGCTATGCATTCGCTACGGAGCAAC	Tb927.1.780_rev	GGGGACCACTTTGTACAAGAAAGCTGGGTTCAAAGGTGAAGCATCTTCACGG
3_known	G4	Tb927.10.10440_for	GGGGACAAGTTTGTACAAAAAAGCAGGCTATGTTAGGTCGATTTGATTTTTTAGGGAAA	Tb927.10.10440_rev	GGGGACCACTTTGTACAAGAAAGCTGGGTTCACTTATTATCTCCTGGGGGCC
3_known	G5	Tb927.3.3920_for	GGGGACAAGTTTGTACAAAAAAGCAGGCTATGGTTATTTGGTTTTCCGCTGGT	Tb927.3.3920_rev	GGGGACCACTTTGTACAAGAAAGCTGGGTTCACACGCGAGGACGTTTTC
3_known	G6	Tb927.10.14040_for	GGGGACAAGTTTGTACAAAAAAGCAGGCTATGTCGGAGCACTTTCTCCTTTTG	Tb927.10.14040_rev	GGGGACCACTTTGTACAAGAAAGCTGGGTCTAACACCTTTGCAAATTGGTCATCTTAA
3_known	G7	Tb927.7.4970_for	GGGGACAAGTTTGTACAAAAAAGCAGGCTATGACAAACTCTTCAGTGCAGCA	Tb927.7.4970_rev	GGGGACCACTTTGTACAAGAAAGCTGGGTCTACACCTTAGAAGTACTTGCCATCCA
3_known	G8	Tb927.2.3480_for	GGGGACAAGTTTGTACAAAAAAGCAGGCTATGATTCCATCATTTGCTCCAGGG	Tb927.2.3480_rev	GGGGACCACTTTGTACAAGAAAGCTGGGTTCACTGTTCAGCACCATCCACC
3_known	G9	Tb927.10.14530_for	GGGGACAAGTTTGTACAAAAAAGCAGGCTATGGCCAATGATGACCCCAAGG	Tb927.10.14530_rev	GGGGACCACTTTGTACAAGAAAGCTGGGTTTACTTCCCTTCTGAAGTTCCACCG
3_known	G10	Tb927.11.6710_for	GGGGACAAGTTTGTACAAAAAAGCAGGCTATGGCAAAGCGCTCGAAGTC	Tb927.11.6710_rev	GGGGACCACTTTGTACAAGAAAGCTGGGTTTAATGCCTATCTGCAAGGAAGCG
3_known	G11	Tb927.4.820_for	GGGGACAAGTTTGTACAAAAAAGCAGGCTATGAGCAGGAAGTGTAGGAAGAGAC	Tb927.4.820_rev	GGGGACCACTTTGTACAAGAAAGCTGGGTTTAACTTCCAGATGCGGGGAAAGG
3_known	G12	Tb927.6.1800_for	GGGGACAAGTTTGTACAAAAAAGCAGGCTATGGGGGTTATGTTACCGAAACCG	Tb927.6.1800_rev	GGGGACCACTTTGTACAAGAAAGCTGGGTTTATTTGTTGCTTTGCTCTGCTAGCG
3_known	G13	Tb927.10.14070_for	GGGGACAAGTTTGTACAAAAAAGCAGGCTATGTCCGACCAGTGTGCCA	Tb927.10.14070_rev	GGGGACCACTTTGTACAAGAAAGCTGGGTTCATACCCGAGGTGGAATGAGTG
3_known	G14	Tb927.4.5240_for	GGGGACAAGTTTGTACAAAAAAGCAGGCTATGTCACCGCTGAAGATGCG	Tb927.4.5240_rev	GGGGACCACTTTGTACAAGAAAGCTGGGTTCAATGCTGAAAACAAGGTAGCGT
3_known	G15	Tb927.10.13560_for	GGGGACAAGTTTGTACAAAAAAGCAGGCTATGTCCGACCAGTGTGTCACC	Tb927.10.13560_rev	GGGGACCACTTTGTACAAGAAAGCTGGGTTCATACCCGAGGTGGAATGAGTG
3_known	G16	Tb927.11.940_for	GGGGACAAGTTTGTACAAAAAAGCAGGCTATGAGACGGGCTGTGGTACTC	Tb927.11.940_rev	GGGGACCACTTTGTACAAGAAAGCTGGGTTTACCGCCCTCCCAGTGC
3_known	G17	Tb927.10.12850_for	GGGGACAAGTTTGTACAAAAAAGCAGGCTATGTACTGTCACGCTGGCGT	Tb927.10.12850_rev	GGGGACCACTTTGTACAAGAAAGCTGGGTTCACTCGTTATTCTCCATATTGGAGGC
3_known	G18	Tb927.10.2770_for	GGGGACAAGTTTGTACAAAAAAGCAGGCTATGACGTCTTCAATGATCCCTGTGG	Tb927.10.2770_rev	GGGGACCACTTTGTACAAGAAAGCTGGGTTCACGCTGCGACACACTTG
3_known	G19	Tb927.10.14790_for	GGGGACAAGTTTGTACAAAAAAGCAGGCTATGTCTCGGCCAGAAGGTGAG	Tb927.10.14790_rev	GGGGACCACTTTGTACAAGAAAGCTGGGTCTACTCCTTGCGCTTCTTCTCTAGC
3_known	G20	Tb927.7.2480_for	GGGGACAAGTTTGTACAAAAAAGCAGGCTATGGTTACCTCTACTGGTCTTCTCTC	Tb927.7.2480_rev	GGGGACCACTTTGTACAAGAAAGCTGGGTTCACTCAACGCGATATATCAAATGCA
3_known	G21	Tb927.5.550_for	GGGGACAAGTTTGTACAAAAAAGCAGGCTATGGGGCGAGGAATTCTGAACT	Tb927.5.550_rev	GGGGACCACTTTGTACAAGAAAGCTGGGTTTAAAATATGCTCACGTACTCATCTACGC
3_known	G22	Tb927.9.3280_for	GGGGACAAGTTTGTACAAAAAAGCAGGCTATGACGGCAGTGGTGAATGAGT	Tb927.9.3280_rev	GGGGACCACTTTGTACAAGAAAGCTGGGTTTACAAATTGTTCCACACTGACAAAAAACT
3_known	G23	Tb927.3.1430_for	GGGGACAAGTTTGTACAAAAAAGCAGGCTATGCGCTGCACAATGTCTTCG	Tb927.3.1430_rev	GGGGACCACTTTGTACAAGAAAGCTGGGTTCAGTCGAGTGCCCTTTCAATAGC
3_known	G24	Tb927.11.11680_for	GGGGACAAGTTTGTACAAAAAAGCAGGCTATGCTCCGTCGTTTGGCC	Tb927.11.11680_rev	GGGGACCACTTTGTACAAGAAAGCTGGGTTTAGGCGAGGTCGAGCACA
3_known	H1	Tb927.11.2910_for	GGGGACAAGTTTGTACAAAAAAGCAGGCTATGGCAGTTGTACGCAGGTG	Tb927.11.2910_rev	GGGGACCACTTTGTACAAGAAAGCTGGGTTCACATGAAAGGCGCTCCAGT
3_known	H3	Tb927.3.5340_for	GGGGACAAGTTTGTACAAAAAAGCAGGCTATGCATACTCTGTCCTCATGTGATTTT	Tb927.3.5340_rev	GGGGACCACTTTGTACAAGAAAGCTGGGTTTAATCCAACTCGTCAGGGTCATGA
3_known	H4	Tb927.11.14140_for	GGGGACAAGTTTGTACAAAAAAGCAGGCTATGAAACGGTCGGTGTCGAAG	Tb927.11.14140_rev	GGGGACCACTTTGTACAAGAAAGCTGGGTTCACACCTCTCTGACATTTCTGTACA
3_known	H5	Tb927.5.2760_for	GGGGACAAGTTTGTACAAAAAAGCAGGCTATGTTTGCTAGGGTTAAAAGGGTAAGG	Tb927.5.2760_rev	GGGGACCACTTTGTACAAGAAAGCTGGGTTCACCACCTTCCACCCAAATGC
3_known	H6	Tb927.4.4250_for	GGGGACAAGTTTGTACAAAAAAGCAGGCTATGGCCCCCGCGCTTCGG	Tb927.4.4250_rev	GGGGACCACTTTGTACAAGAAAGCTGGGTCTACAAACAACTCATTTCCCGGAGA
3_known	H8	Tb927.9.4910_for	GGGGACAAGTTTGTACAAAAAAGCAGGCTATGTCAGCGTCCCGGATGG	Tb927.9.4910_rev	GGGGACCACTTTGTACAAGAAAGCTGGGTTCAATCCCCCCTAATCTTGCCA
3_known	H9	Tb927.4.400_for	GGGGACAAGTTTGTACAAAAAAGCAGGCTATGGAAAGTATGCCGTCTGAGGA	Tb927.4.400_rev	GGGGACCACTTTGTACAAGAAAGCTGGGTTTAGGCATAAAGCCTTGCCACC
3_known	H10	Tb927.11.16740_for	GGGGACAAGTTTGTACAAAAAAGCAGGCTATGCGGGGTTTGGCGGCA	Tb927.11.16740_rev	GGGGACCACTTTGTACAAGAAAGCTGGGTCTACAGATGCTCCGCCAACTC
3_known	H11	Tb927.9.14420_for	GGGGACAAGTTTGTACAAAAAAGCAGGCTATGAGCGCGCTCGTTCGA	Tb927.9.14420_rev	GGGGACCACTTTGTACAAGAAAGCTGGGTCTACAAGAACCTCCACCAAGGAGT
3_known	H12	Tb927.10.13190_for	GGGGACAAGTTTGTACAAAAAAGCAGGCTATGAGACCTCAAAAAGAAAAAAACACCG	Tb927.10.13190_rev	GGGGACCACTTTGTACAAGAAAGCTGGGTCTAAAGCTTCGTGACGTCTTTTCTTG
3_known	H13	Tb927.4.1660_for	GGGGACAAGTTTGTACAAAAAAGCAGGCTATGGGGGTTGAAGTAGCAGACT	Tb927.4.1660_rev	GGGGACCACTTTGTACAAGAAAGCTGGGTTTAGTCATCACCGACCAAAATGCTC
3_known	H14	Tb927.4.4290_for	GGGGACAAGTTTGTACAAAAAAGCAGGCTATGTGTAGGCAATTTAACAGTCGCG	Tb927.4.4290_rev	GGGGACCACTTTGTACAAGAAAGCTGGGTCTACAAACAACTCATTTCCCGGAGA
3_known	H15	Tb927.8.4640_for	GGGGACAAGTTTGTACAAAAAAGCAGGCTATGGAGCCAGCAGCTGAGA	Tb927.8.4640_rev	GGGGACCACTTTGTACAAGAAAGCTGGGTCTACATACAGAGATCCCAATACTTGAAATA
3_known	H16	Tb927.9.2450_for	GGGGACAAGTTTGTACAAAAAAGCAGGCTATGATACGCGGCTCACACCT	Tb927.9.2450_rev	GGGGACCACTTTGTACAAGAAAGCTGGGTTTAGATTTCACTGGGTTTCTGGACG
3_known	H17	Tb927.5.2960_for	GGGGACAAGTTTGTACAAAAAAGCAGGCTATGAGTAAACCTACCGTTTCGGAGG	Tb927.5.2960_rev	GGGGACCACTTTGTACAAGAAAGCTGGGTTTATGGAGAGTCGACGGATCCC
3_known	H18	Tb927.11.10990_for	GGGGACAAGTTTGTACAAAAAAGCAGGCTATGAGCAGTGCGCACAACA	Tb927.11.10990_rev	GGGGACCACTTTGTACAAGAAAGCTGGGTTCAAGGCTCAACAATACGGGATATG
3_known	H19	Tb927.10.5690_for	GGGGACAAGTTTGTACAAAAAAGCAGGCTATGCTCAAGTTTGTTCTTGTGTTAATACTG	Tb927.10.5690_rev	GGGGACCACTTTGTACAAGAAAGCTGGGTTTACGTACACTCCTTCATTTCCTCCG
3_known	H20	Tb927.10.4160_for	GGGGACAAGTTTGTACAAAAAAGCAGGCTATGTCTTTTTTCAGGGGCTTAAACTTCA	Tb927.10.4160_rev	GGGGACCACTTTGTACAAGAAAGCTGGGTTCACCGTTTTTTCAGTCGTGGG
3_known	H21	Tb927.10.7800_for	GGGGACAAGTTTGTACAAAAAAGCAGGCTATGATAAAGGAGGGGGGAAATGAGG	Tb927.10.7800_rev	GGGGACCACTTTGTACAAGAAAGCTGGGTTCACAATAGGGGACTTTCACATAGCT
3_known	H22	Tb927.9.6040_for	GGGGACAAGTTTGTACAAAAAAGCAGGCTATGAGTCAGGATAGGGGCAAGC	Tb927.9.6040_rev	GGGGACCACTTTGTACAAGAAAGCTGGGTCTACATGGCTCTCATTTTGGCCA
3_known	H23	Tb927.11.9030_for	GGGGACAAGTTTGTACAAAAAAGCAGGCTATGGCTAACGTTAATAACGAGACCAC	Tb927.11.9030_rev	GGGGACCACTTTGTACAAGAAAGCTGGGTTTAGATCCATTTTCCAATAAAGGCCATTAA

3_known	H24	Tb927.10.14800_for	GGGGACAAGTTTGTACAAAAAAGCAGGCTATGAATCGGTATACGATATTGGGTCAGC	Tb927.10.14800_rev	GGGGACCACTTTGTACAAGAAAGCTGGGTTTAAAAGTTGAACGCTTCATCGTCAAG
3_known	I1	Tb927.11.6970_for	GGGGACAAGTTTGTACAAAAAAGCAGGCTATGGATCCCAGCAGTGGTAACG	Tb927.11.6970_rev	GGGGACCACTTTGTACAAGAAAGCTGGGTTCATTGGTAGGCCGTTAAATGTGTC
3_known	I2	Tb927.11.8310_for	GGGGACAAGTTTGTACAAAAAAGCAGGCTATGTCGACGCTAGCTTCTGCT	Tb927.11.8310_rev	GGGGACCACTTTGTACAAGAAAGCTGGGTTCAAAACGCCCTCCTATGAAAATGA
3_known	I3	Tb927.3.5660_for	GGGGACAAGTTTGTACAAAAAAGCAGGCTATGGCAATCAAATCACGAGGAGTT	Tb927.3.5660_rev	GGGGACCACTTTGTACAAGAAAGCTGGGTTTATGCCGTCCATTCCGCATC
3_known	I4	Tb927.10.1680_for	GGGGACAAGTTTGTACAAAAAAGCAGGCTATGAATGAGGATGGTGATTGTCGG	Tb927.10.1680_rev	GGGGACCACTTTGTACAAGAAAGCTGGGTTCAAACAGTCATTATATTTGGACAGTGCT
3_known	I5	Tb927.3.5580_for	GGGGACAAGTTTGTACAAAAAAGCAGGCTATGACTGAAGCCGTTGTAACACC	Tb927.3.5580_rev	GGGGACCACTTTGTACAAGAAAGCTGGGTTCACTGGGTTGCCGCCTT
3_known	I6	Tb927.10.3560_for	GGGGACAAGTTTGTACAAAAAAGCAGGCTATGTCACCGAAGAAAAACTCGGC	Tb927.10.3560_rev	GGGGACCACTTTGTACAAGAAAGCTGGGTTCAATACCTTTGGTAGTTGTACGTGC
3_known	I7	Tb927.8.7510_for	GGGGACAAGTTTGTACAAAAAAGCAGGCTATGGCTAGCGTGTCATCAGGA	Tb927.8.7510_rev	GGGGACCACTTTGTACAAGAAAGCTGGGTTTAGTCGTCACGTGCATCAGGC
3_known	I8	Tb927.7.5490_for	GGGGACAAGTTTGTACAAAAAAGCAGGCTATGCCCCCAAAGCAGCAC	Tb927.7.5490_rev	GGGGACCACTTTGTACAAGAAAGCTGGGTTCAGGTTGTTTTGCCCTCGC
3_known	I9	Tb927.8.7170_for	GGGGACAAGTTTGTACAAAAAAGCAGGCTATGCCTCAGGTGGATCTCGTT	Tb927.8.7170_rev	GGGGACCACTTTGTACAAGAAAGCTGGGTCTACTGCCATCCCAAACGCC
3_known	I10	Tb927.8.5630_for	GGGGACAAGTTTGTACAAAAAAGCAGGCTATGGCGCGCGTTGAACTG	Tb927.8.5630_rev	GGGGACCACTTTGTACAAGAAAGCTGGGTTCACATTACTTTCCCCACAAGCTTC
3_known	I11	Tb927.8.8100_for	GGGGACAAGTTTGTACAAAAAAGCAGGCTATGTCCCTGTCGGAGCCC	Tb927.8.8100_rev	GGGGACCACTTTGTACAAGAAAGCTGGGTCTACGAGCAGTCGAAAGTTTTCACA
3_known	I12	Tb927.8.8090_for	GGGGACAAGTTTGTACAAAAAAGCAGGCTATGTCCCTGTCGGAGCCC	Tb927.8.8090_rev	GGGGACCACTTTGTACAAGAAAGCTGGGTCTACCGGCAACTAAAACCCTTCAT
3_known	I13	Tb927.9.12720_for	GGGGACAAGTTTGTACAAAAAAGCAGGCTATGTTCAGGACTATAGGAATCGTGCG	Tb927.9.12720_rev	GGGGACCACTTTGTACAAGAAAGCTGGGTTCAATGTAAAGAATTTTCCCCCAAACGT
3_known	I14	Tb927.10.6460_for	GGGGACAAGTTTGTACAAAAAAGCAGGCTATGCTGCCTATACTAAAGGCCGG	Tb927.10.6460_rev	GGGGACCACTTTGTACAAGAAAGCTGGGTTCATGCTCCCTCACACGAAATG
3_known	I15	Tb927.10.14720_for	GGGGACAAGTTTGTACAAAAAAGCAGGCTATGTACGGTGGTTATGGTGCAGG	Tb927.10.14720_rev	GGGGACCACTTTGTACAAGAAAGCTGGGTCTAGAGTTTTGTCTCCCTCTCCAGAA
3_known	I16	Tb927.11.8990_for	GGGGACAAGTTTGTACAAAAAAGCAGGCTATGCTTGGAGCGAACCTTGG	Tb927.11.8990_rev	GGGGACCACTTTGTACAAGAAAGCTGGGTCTAGTGCCACTTGGACAGCAG
3_known	I17	Tb927.11.1960_for	GGGGACAAGTTTGTACAAAAAAGCAGGCTATGAACCACCCTGATGAACTTGAAG	Tb927.11.1960_rev	GGGGACCACTTTGTACAAGAAAGCTGGGTTTACATCACTACCTTGGCACAGCA
3_known	I18	Tb927.11.4910_for	GGGGACAAGTTTGTACAAAAAAGCAGGCTATGGACAACGGTAAGCGCATT	Tb927.11.4910_rev	GGGGACCACTTTGTACAAGAAAGCTGGGTTCACTTCGACCTCCGCGT
3_known	I19	Tb927.8.1520_for	GGGGACAAGTTTGTACAAAAAAGCAGGCTATGTCGCTCCCCGCTGTG	Tb927.8.1520_rev	GGGGACCACTTTGTACAAGAAAGCTGGGTCTACACCTCTCCATCATTGTTTGATGC
3_known	I20	Tb927.11.11950_for	GGGGACAAGTTTGTACAAAAAAGCAGGCTATGATTCGCCTTCGTTATCGTGC	Tb927.11.11950_rev	GGGGACCACTTTGTACAAGAAAGCTGGGTTTACACAAGTACCAACCCTCGAGA
3_known	I21	Tb927.2.5250_for	GGGGACAAGTTTGTACAAAAAAGCAGGCTATGAGTGAGTTCCCGTTGGTGT	Tb927.2.5250_rev	GGGGACCACTTTGTACAAGAAAGCTGGGTTCACGTGCTCCCGATAGGC
3_known	I22	Tb927.3.830_for	GGGGACAAGTTTGTACAAAAAAGCAGGCTATGGGTGTTCTTGGCCTTTCG	Tb927.3.830_rev	GGGGACCACTTTGTACAAGAAAGCTGGGTCTACTTTTTGACAGCCTTTTTGTGTCC
3_known	I23	Tb927.10.9410_for	GGGGACAAGTTTGTACAAAAAAGCAGGCTATGTCAGAGAAGGAAGGAAAAGTAATACCC	Tb927.10.9410_rev	GGGGACCACTTTGTACAAGAAAGCTGGGTTTAAATAATCTTATCATTTACCTTCGCTCC
3_known	I24	Tb927.6.4120_for	GGGGACAAGTTTGTACAAAAAAGCAGGCTATGTCTGGCTTCCCGCCT	Tb927.6.4120_rev	GGGGACCACTTTGTACAAGAAAGCTGGGTCTACGATTTCCACATAGAAGAGACCCA
3_known	J1	Tb927.8.620_for	GGGGACAAGTTTGTACAAAAAAGCAGGCTATGAAGCGTGTTACTTCACATATTTCGC	Tb927.8.620_rev	GGGGACCACTTTGTACAAGAAAGCTGGGTTCACACCTTCAACACTGACCCA
3_known	J2	Tb927.11.10450_for	GGGGACAAGTTTGTACAAAAAAGCAGGCTATGCACGCGACAGTCACTG	Tb927.11.10450_rev	GGGGACCACTTTGTACAAGAAAGCTGGGTTCAGTAGACGTGGTCGTACAGG
3_known	J3	Tb927.6.2040_for	GGGGACAAGTTTGTACAAAAAAGCAGGCTATGGGATGTTTGCCCAGTGTG	Tb927.6.2040_rev	GGGGACCACTTTGTACAAGAAAGCTGGGTTTACAAGACGATTACCCGCTCTTCC
3_known	J4	Tb927.2.5060_for	GGGGACAAGTTTGTACAAAAAAGCAGGCTATGCCACCTAAGAAGAAGGATGACA	Tb927.2.5060_rev	GGGGACCACTTTGTACAAGAAAGCTGGGTTTACTTCTTACCTCCTTTAGCTGCATTAAA
3_known	J5	Tb927.10.5680_for	GGGGACAAGTTTGTACAAAAAAGCAGGCTATGAAGGTGAATTGTTTTCTACCTTTGTTG	Tb927.10.5680_rev	GGGGACCACTTTGTACAAGAAAGCTGGGTTCAAACAAAAACAGGAACGAAAACTAAGAA
3_known	J6	Tb927.11.10110_for	GGGGACAAGTTTGTACAAAAAAGCAGGCTATGGTACAACAACAGCCCGTTG	Tb927.11.10110_rev	GGGGACCACTTTGTACAAGAAAGCTGGGTCTAAATCCTCTCGTGAACGACGG
3_known	J7	Tb927.8.1910_for	GGGGACAAGTTTGTACAAAAAAGCAGGCTATGAACTGCAGAGAGTGGTTGGC	Tb927.8.1910_rev	GGGGACCACTTTGTACAAGAAAGCTGGGTCTACAAATGCGGTTTTCTTCCAATATCG
3_known	J8	Tb927.10.3080_for	GGGGACAAGTTTGTACAAAAAAGCAGGCTATGGGGATAGTCCGAGTACAGAGG	Tb927.10.3080_rev	GGGGACCACTTTGTACAAGAAAGCTGGGTTTATTCCGGCTCCTGTAAACTGC
3_known	J9	Tb927.11.9010_for	GGGGACAAGTTTGTACAAAAAAGCAGGCTATGGCTAACGTTAATAACGAGACCAC	Tb927.11.9010_rev	GGGGACCACTTTGTACAAGAAAGCTGGGTTTAGATCCACTTTCCGATAAAGGCCA
3_known	J10	Tb927.11.9000_for	GGGGACAAGTTTGTACAAAAAAGCAGGCTATGGCTAACGTTAATAACGAGACCAC	Tb927.11.9000_rev	GGGGACCACTTTGTACAAGAAAGCTGGGTTTAGACCCATTTTCCTAACACGGC
3_known	J11	Tb927.7.4980_for	GGGGACAAGTTTGTACAAAAAAGCAGGCTATGCCTGGTTGCCTTTCGG	Tb927.7.4980_rev	GGGGACCACTTTGTACAAGAAAGCTGGGTTCAGTAAACCACGATCAGCACAAC
3_known	J12	Tb927.8.5530_for	GGGGACAAGTTTGTACAAAAAAGCAGGCTATGATAGATGATGTGGTAACCGATGATG	Tb927.8.5530_rev	GGGGACCACTTTGTACAAGAAAGCTGGGTTCAAAGTGGATCGACACCGTATTG
3_known	J13	Tb927.8.7410_for	GGGGACAAGTTTGTACAAAAAAGCAGGCTATGCTAATGTGCATGCGCCC	Tb927.8.7410_rev	GGGGACCACTTTGTACAAGAAAGCTGGGTCTACAAGTCTGACTTATCCTCCTTCTTCT
3_known	J14	Tb927.10.15850_for	GGGGACAAGTTTGTACAAAAAAGCAGGCTATGATAGAGAGTAACTTGCTCTCGCA	Tb927.10.15850_rev	GGGGACCACTTTGTACAAGAAAGCTGGGTTCAGCACTCATAAATGCGTCGAA
3_known	J15	Tb927.9.4950_for	GGGGACAAGTTTGTACAAAAAAGCAGGCTATGAACAAGTGCAAGCAAGCCG	Tb927.9.4950_rev	GGGGACCACTTTGTACAAGAAAGCTGGGTTCACGGGGCAGATCCCTCG
3_known	J17	Tb927.11.15640_for	GGGGACAAGTTTGTACAAAAAAGCAGGCTATGCGCAAGCAATTATTTTTCACCC	Tb927.11.15640_rev	GGGGACCACTTTGTACAAGAAAGCTGGGTTCACGATGCATCTTCCCCGT
3_known	J18	Tb927.10.15270_for	GGGGACAAGTTTGTACAAAAAAGCAGGCTATGTCGTGCGAAGGTTGCG	Tb927.10.15270_rev	GGGGACCACTTTGTACAAGAAAGCTGGGTTCAAGCAGCGGTAGCCGA
3_known	J19	Tb927.11.2730_for	GGGGACAAGTTTGTACAAAAAAGCAGGCTATGCGCGTTTTGGTTTGTGG	Tb927.11.2730_rev	GGGGACCACTTTGTACAAGAAAGCTGGGTTTACAACTTAGTGGTCCTTCCCCC
3_known	J20	Tb927.8.5510_for	GGGGACAAGTTTGTACAAAAAAGCAGGCTATGCCACCGAAAAAACTATCCAAGC	Tb927.8.5510_rev	GGGGACCACTTTGTACAAGAAAGCTGGGTTTACTTACGGAGCCACATCTGCA
3_known	J21	Tb927.8.7140_for	GGGGACAAGTTTGTACAAAAAAGCAGGCTATGAGGACAAGGCGTCGTAGG	Tb927.8.7140_rev	GGGGACCACTTTGTACAAGAAAGCTGGGTCTAAGGGTGCCGACGGAAAG
3_known	J22	Tb927.6.4850_for	GGGGACAAGTTTGTACAAAAAAGCAGGCTATGTCCGTGCGCCAGATATTC	Tb927.6.4850_rev	GGGGACCACTTTGTACAAGAAAGCTGGGTCTACTGCACGTCACTAAGACCCT
3_known	K2	Tb927.11.9350_for	GGGGACAAGTTTGTACAAAAAAGCAGGCTATGCAGATGTTGGCCCCTGG	Tb927.11.9350_rev	GGGGACCACTTTGTACAAGAAAGCTGGGTCTAGCCCCTCAAGCAGTTAGTGA
3_known	K3	Tb927.1.2580_for	GGGGACAAGTTTGTACAAAAAAGCAGGCTATGCAGTTTGGCCCTGTTGTC	Tb927.1.2580_rev	GGGGACCACTTTGTACAAGAAAGCTGGGTTTACCCACTACTCCCCGCAAG
3_known	K5	Tb927.8.7730_for	GGGGACAAGTTTGTACAAAAAAGCAGGCTATGGCACGGCACCCTTTAGG	Tb927.8.7730_rev	GGGGACCACTTTGTACAAGAAAGCTGGGTCTACGACGTCTTGGAGTGCTTG
3_known	K10	Tb927.11.7160_for	GGGGACAAGTTTGTACAAAAAAGCAGGCTATGGAGCAGCAGGGAGATAGTG	Tb927.11.7160_rev	GGGGACCACTTTGTACAAGAAAGCTGGGTCTAACGAGTGCGTTTCCGTTCC
3_known	K11	Tb927.11.16030_for	GGGGACAAGTTTGTACAAAAAAGCAGGCTATGCCGCCTCAGCAACCG	Tb927.11.16030_rev	GGGGACCACTTTGTACAAGAAAGCTGGGTTCACACTTCTGCAAGACGAGATAATTT
3_known	K12	Tb927.10.3520_for	GGGGACAAGTTTGTACAAAAAAGCAGGCTATGCAGGAACGTCAGGAGGAG	Tb927.10.3520_rev	GGGGACCACTTTGTACAAGAAAGCTGGGTCTATTGCTCTGAGTATTGATGTGGAGC
3_known	K13	Tb927.4.5110_for	GGGGACAAGTTTGTACAAAAAAGCAGGCTATGCGCGCATCAACGCCA	Tb927.4.5110_rev	GGGGACCACTTTGTACAAGAAAGCTGGGTTCACACGGCAGGCACCAC
3_known	K14	Tb927.4.5260_for	GGGGACAAGTTTGTACAAAAAAGCAGGCTATGTCACCGCTGAAGATGCG	Tb927.4.5260_rev	GGGGACCACTTTGTACAAGAAAGCTGGGTTCAGGCCCTCTTACGCCAA
3_known	K15	Tb927.4.4740_for	GGGGACAAGTTTGTACAAAAAAGCAGGCTATGGCCCGGCAACCTTTG	Tb927.4.4740_rev	GGGGACCACTTTGTACAAGAAAGCTGGGTCTACTCCCTATGAGGTTTTGAATTCTTTGG
3_known	K16	Tb927.3.1630_for	GGGGACAAGTTTGTACAAAAAAGCAGGCTATGGCTGAGGCAACCGAAAG	Tb927.3.1630_rev	GGGGACCACTTTGTACAAGAAAGCTGGGTTTATTGTCGCCTGAAGGAATGACTC
3_known	K17	Tb927.4.4910_for	GGGGACAAGTTTGTACAAAAAAGCAGGCTATGCGTCGCAACGTCCTG	Tb927.4.4910_rev	GGGGACCACTTTGTACAAGAAAGCTGGGTTCACTTCCTTACTGCCTTACCCTT
3_known	K18	Tb927.2.5160_for	GGGGACAAGTTTGTACAAAAAAGCAGGCTATGGTGAAAGAAACAAAATACTACGACGC	Tb927.2.5160_rev	GGGGACCACTTTGTACAAGAAAGCTGGGTCTATTGCTGCGTACACGTTGCA
3_known	K19	Tb927.4.5290_for	GGGGACAAGTTTGTACAAAAAAGCAGGCTATGTCACCGCTGAAGATGCG	Tb927.4.5290_rev	GGGGACCACTTTGTACAAGAAAGCTGGGTTCAGGCCCTCTTACGCCAA
3_known	K20	Tb927.4.5250_for	GGGGACAAGTTTGTACAAAAAAGCAGGCTATGTCACCGCTGAAGATGCG	Tb927.4.5250_rev	GGGGACCACTTTGTACAAGAAAGCTGGGTTCAGGCCCTCTTACGCCAA
3_known	K21	Tb927.4.5270_for	GGGGACAAGTTTGTACAAAAAAGCAGGCTATGTCACCGCTGAAGATGCG	Tb927.4.5270_rev	GGGGACCACTTTGTACAAGAAAGCTGGGTTCAGGCCCTCTTACGCCAA
3_known	K22	Tb927.10.15800_for	GGGGACAAGTTTGTACAAAAAAGCAGGCTATGAGCGAAAAGCTCCCCGT	Tb927.10.15800_rev	GGGGACCACTTTGTACAAGAAAGCTGGGTCTAAACCCCAAATCGGGCCTG
3_known	K23	Tb927.4.5280_for	GGGGACAAGTTTGTACAAAAAAGCAGGCTATGTCACCGCTGAAGATGCG	Tb927.4.5280_rev	GGGGACCACTTTGTACAAGAAAGCTGGGTTCAGGCCCTCTTACGCCAA
3_known	K24	Tb927.5.630_for	GGGGACAAGTTTGTACAAAAAAGCAGGCTATGTCGCGTACTATTGTGCTTGG	Tb927.5.630_rev	GGGGACCACTTTGTACAAGAAAGCTGGGTTTAAAACGGGTCGATGTTTGTCGA
3_known	L1	Tb927.10.13120_for	GGGGACAAGTTTGTACAAAAAAGCAGGCTATGAGTATCACCAACATCGCTGC	Tb927.10.13120_rev	GGGGACCACTTTGTACAAGAAAGCTGGGTTCATATTTGTTTCCAGAGTTCTCGGTC
3_known	L2	Tb927.11.8770_for	GGGGACAAGTTTGTACAAAAAAGCAGGCTATGACAGCAACCGCAAGGAG	Tb927.11.8770_rev	GGGGACCACTTTGTACAAGAAAGCTGGGTTTAGAACTGTTCACCGACGTTTGC
3_known	L3	Tb927.5.2370_for	GGGGACAAGTTTGTACAAAAAAGCAGGCTATGTCTCAAGCTGATAATGCTGAGC	Tb927.5.2370_rev	GGGGACCACTTTGTACAAGAAAGCTGGGTTCAAAGTTTGGCCGCAACGG
3_known	L4	Tb927.1.3000_for	GGGGACAAGTTTGTACAAAAAAGCAGGCTATGACGTTTATCAGCTCCCTAATACAGG	Tb927.1.3000_rev	GGGGACCACTTTGTACAAGAAAGCTGGGTTTACGTTCCTGGACTAGGAGCG
3_known	L5	Tb927.10.10220_for	GGGGACAAGTTTGTACAAAAAAGCAGGCTATGATAAGGGTCGTATTCACAATAGCG	Tb927.10.10220_rev	GGGGACCACTTTGTACAAGAAAGCTGGGTTTAGTCGTCCTTTCCCCAATTTCCG
3_known	L6	Tb927.3.5320_for	GGGGACAAGTTTGTACAAAAAAGCAGGCTATGGATGAAGATGTGGTGCAAATCT	Tb927.3.5320_rev	GGGGACCACTTTGTACAAGAAAGCTGGGTTCAATGGATGTGAAGAACCACTGTATC
3_known	L7	Tb927.10.3660_for	GGGGACAAGTTTGTACAAAAAAGCAGGCTATGTCCAGGCCCTTTAAGGACTT	Tb927.10.3660_rev	GGGGACCACTTTGTACAAGAAAGCTGGGTCTACTTGTTACGCACGTGTCGG
3_known	L8	Tb927.11.14600_for	GGGGACAAGTTTGTACAAAAAAGCAGGCTATGCTTGCAACAGTAGTTTTGTTTGT	Tb927.11.14600_rev	GGGGACCACTTTGTACAAGAAAGCTGGGTCTAACCCTCCGATGTCCAACTTATACG
3_known	L9	Tb927.6.1090_for	GGGGACAAGTTTGTACAAAAAAGCAGGCTATGACTTCACTTTTAGCGCAAACAAC	Tb927.6.1090_rev	GGGGACCACTTTGTACAAGAAAGCTGGGTCTACGAATAAAAATCATATTGTTCATCACC
3_known	L10	Tb927.5.590_for	GGGGACAAGTTTGTACAAAAAAGCAGGCTATGTCACAGGAGGGTGGGAAAT	Tb927.5.590_rev	GGGGACCACTTTGTACAAGAAAGCTGGGTTCAAGCTTTTCGCTTACGATCAGC
3_known	L11	Tb927.10.6240_for	GGGGACAAGTTTGTACAAAAAAGCAGGCTATGAACCTACGTGGTGATGGGA	Tb927.10.6240_rev	GGGGACCACTTTGTACAAGAAAGCTGGGTCTACATGATTACACACGAACCTGTGC
3_known	L12	Tb927.6.440_for	GGGGACAAGTTTGTACAAAAAAGCAGGCTATGGAGAAACCGTCTTGCAGGG	Tb927.6.440_rev	GGGGACCACTTTGTACAAGAAAGCTGGGTCTACACCACCACCTGGAGCA
3_known	L13	Tb927.9.4680_for	GGGGACAAGTTTGTACAAAAAAGCAGGCTATGGCCCAACAAGGAAAGGTCG	Tb927.9.4680_rev	GGGGACCACTTTGTACAAGAAAGCTGGGTTTACTCACCAAGGTAGGCAGCA
3_known	L14	Tb927.7.4950_for	GGGGACAAGTTTGTACAAAAAAGCAGGCTATGGGTAAACTAAACCCCGCCC	Tb927.7.4950_rev	GGGGACCACTTTGTACAAGAAAGCTGGGTTTACCTTGTCATCCTTGCCTGTATGT
3_known	L15	Tb927.8.6060_for	GGGGACAAGTTTGTACAAAAAAGCAGGCTATGTTCCGCACTGTTCCTCGT	Tb927.8.6060_rev	GGGGACCACTTTGTACAAGAAAGCTGGGTCTATACGTTAAGTTCTTTCTTGATTTTTGT
3_known	L16	Tb927.9.6170_for	GGGGACAAGTTTGTACAAAAAAGCAGGCTATGGGCTTCGGATCATCAAAACC	Tb927.9.6170_rev	GGGGACCACTTTGTACAAGAAAGCTGGGTTTATTCATTTTCATTCGTGGCTACACCG
3_known	L17	Tb927.11.13090_for	GGGGACAAGTTTGTACAAAAAAGCAGGCTATGTCTCTCACACTCTGGTTTCAGC	Tb927.11.13090_rev	GGGGACCACTTTGTACAAGAAAGCTGGGTCTATTTAAAGACGCGGCCCTCAA
3_known	L18	Tb927.8.1710_for	GGGGACAAGTTTGTACAAAAAAGCAGGCTATGTCGCAGGTAGCTGGTAATCC	Tb927.8.1710_rev	GGGGACCACTTTGTACAAGAAAGCTGGGTCTACTGTTGCTCAGGCCATGG
3_known	L19	Tb927.10.6530_for	GGGGACAAGTTTGTACAAAAAAGCAGGCTATGGAGTATCGGTGGCTGAATAGG	Tb927.10.6530_rev	GGGGACCACTTTGTACAAGAAAGCTGGGTCTACTTTCCCCTTTTCACAGCTGT
3_known	L21	Tb927.10.10240_for	GGGGACAAGTTTGTACAAAAAAGCAGGCTATGGTCTCTTTGATTATCCTTTTTCGCT	Tb927.10.10240_rev	GGGGACCACTTTGTACAAGAAAGCTGGGTTTAAAACAACAAAAATAACAACGCGAGGG
3_known	L22	Tb927.10.15720_for	GGGGACAAGTTTGTACAAAAAAGCAGGCTATGGAGGAGCGGACGCTG	Tb927.10.15720_rev	GGGGACCACTTTGTACAAGAAAGCTGGGTCTAGTTGGGAGTTTGCTGCGT
3_known	L23	Tb927.7.2660_for	GGGGACAAGTTTGTACAAAAAAGCAGGCTATGCAGCTCGTAGCCTCGC	Tb927.7.2660_rev	GGGGACCACTTTGTACAAGAAAGCTGGGTTTAATTCCTGCCCCTCCCTTCC
3_known	L24	Tb927.7.1710_for	GGGGACAAGTTTGTACAAAAAAGCAGGCTATGCGCTTGTTGAAAAAGTCGG	Tb927.7.1710_rev	GGGGACCACTTTGTACAAGAAAGCTGGGTTTACACAACTACACTTGCCGCAG
3_known	M1	Tb927.3.2440_for	GGGGACAAGTTTGTACAAAAAAGCAGGCTATGCTCAATAGACTATTTGGCCGG	Tb927.3.2440_rev	GGGGACCACTTTGTACAAGAAAGCTGGGTCTAACTACCACCACCCAGATGATTATCA
3_known	M2	Tb927.1.1330_for	GGGGACAAGTTTGTACAAAAAAGCAGGCTATGAATGTAGCCAAACGAGAGTTTATTAGA	Tb927.1.1330_rev	GGGGACCACTTTGTACAAGAAAGCTGGGTTCATTGGGGTCGGAACGTTTTAAC
3_known	M3	Tb927.10.3990_for	GGGGACAAGTTTGTACAAAAAAGCAGGCTATGGTAACCGATGATGATTGGAGGG	Tb927.10.3990_rev	GGGGACCACTTTGTACAAGAAAGCTGGGTTTACGCGGTGTACAGCTCAGG
3_known	M4	Tb927.9.3350_for	GGGGACAAGTTTGTACAAAAAAGCAGGCTATGTTGCATGCCGTTCGCC	Tb927.9.3350_rev	GGGGACCACTTTGTACAAGAAAGCTGGGTTCAGCCCGGTATCAGCGG
3_known	M5	Tb927.7.220_for	GGGGACAAGTTTGTACAAAAAAGCAGGCTATGAAAAGCAAGTCCAGCAAGCA	Tb927.7.220_rev	GGGGACCACTTTGTACAAGAAAGCTGGGTCTATTCTGTGAGAGACCTATTAAGAGTCGA
3_known	M6	Tb927.8.5460_for	GGGGACAAGTTTGTACAAAAAAGCAGGCTATGGGTTGCTCTGCATCGAAG	Tb927.8.5460_rev	GGGGACCACTTTGTACAAGAAAGCTGGGTTCAATTACCTTCATTTGCTCCGTTCT
3_known	M7	Tb927.11.6690_for	GGGGACAAGTTTGTACAAAAAAGCAGGCTATGGAACGAGACGATGGTGCG	Tb927.11.6690_rev	GGGGACCACTTTGTACAAGAAAGCTGGGTCTAAGCATTCGCTCCTGATAGAAAGG
3_known	M8	Tb927.5.2780_for	GGGGACAAGTTTGTACAAAAAAGCAGGCTATGCTCAGACGAACCTTCTTCCG	Tb927.5.2780_rev	GGGGACCACTTTGTACAAGAAAGCTGGGTCTATGGGTCCCGATTCTCCGG
3_known	M9	Tb927.10.9740_for	GGGGACAAGTTTGTACAAAAAAGCAGGCTATGGCAACCAAGATGGTGACAC	Tb927.10.9740_rev	GGGGACCACTTTGTACAAGAAAGCTGGGTCTATTTCCACATCTTCTTGAGTGAGACG
3_known	M10	Tb927.11.760_for	GGGGACAAGTTTGTACAAAAAAGCAGGCTATGGGTGTTTCGCTACCAAAGC	Tb927.11.760_rev	GGGGACCACTTTGTACAAGAAAGCTGGGTTCATTCTTCCGACTGCCCCG
3_known	M11	Tb927.2.3080_for	GGGGACAAGTTTGTACAAAAAAGCAGGCTATGTTGCCTAAGCAACAGATGGG	Tb927.2.3080_rev	GGGGACCACTTTGTACAAGAAAGCTGGGTTTAACTGCGATAAATGCAGATCCCC
3_known	M12	Tb927.1.1720_for	GGGGACAAGTTTGTACAAAAAAGCAGGCTATGCAAGCTACACTGGGGTTTG	Tb927.1.1720_rev	GGGGACCACTTTGTACAAGAAAGCTGGGTTTAGTATCGCCTTCGCAGAACCT
3_known	M13	Tb927.11.14780_for	GGGGACAAGTTTGTACAAAAAAGCAGGCTATGTCGAAGTTAATTAAATTGGATTGTGGC	Tb927.11.14780_rev	GGGGACCACTTTGTACAAGAAAGCTGGGTTCACATATGCGATGCCGAATGG
3_known	M14	Tb927.8.3770_for	GGGGACAAGTTTGTACAAAAAAGCAGGCTATGGGTTCTAAAGGCGAGGAGC	Tb927.8.3770_rev	GGGGACCACTTTGTACAAGAAAGCTGGGTTTAGGGCATAGAGGTGGGAAGATC
3_known	M15	Tb927.9.4930_for	GGGGACAAGTTTGTACAAAAAAGCAGGCTATGACTGTGTCTGAATTCGGCAG	Tb927.9.4930_rev	GGGGACCACTTTGTACAAGAAAGCTGGGTCTAGTGCGAAGTGGGCACC
3_known	M16	Tb927.8.1820_for	GGGGACAAGTTTGTACAAAAAAGCAGGCTATGGCATCTCAGGTTGTGCG	Tb927.8.1820_rev	GGGGACCACTTTGTACAAGAAAGCTGGGTCTACAAGTCACTAATCAAAATGGCAACG
3_known	M17	Tb927.6.3620_for	GGGGACAAGTTTGTACAAAAAAGCAGGCTATGGGTTTTGAATTTAAGTTGCACCAG	Tb927.6.3620_rev	GGGGACCACTTTGTACAAGAAAGCTGGGTCTAGTGAATCACCTTGTCGATACAGC
3_known	M18	Tb927.10.15010_for	GGGGACAAGTTTGTACAAAAAAGCAGGCTATGTTTCAGCGATGGTCGTTTGC	Tb927.10.15010_rev	GGGGACCACTTTGTACAAGAAAGCTGGGTCTACTCGGAATCCTTTGGTTCATTCC
3_known	M19	Tb927.8.1850_for	GGGGACAAGTTTGTACAAAAAAGCAGGCTATGCTCTCAATATGGCGAGCG	Tb927.8.1850_rev	GGGGACCACTTTGTACAAGAAAGCTGGGTCTAACCTACTGAGCCCCTGAGTG
3_known	M20	Tb927.9.12320_for	GGGGACAAGTTTGTACAAAAAAGCAGGCTATGTCTAAGCAGCAGCATTTGGT	Tb927.9.12320_rev	GGGGACCACTTTGTACAAGAAAGCTGGGTCTAATGGCACATGGCATCCAGC
3_known	M21	Tb927.7.930_for	GGGGACAAGTTTGTACAAAAAAGCAGGCTATGTCTCGTCGGCGTCCT	Tb927.7.930_rev	GGGGACCACTTTGTACAAGAAAGCTGGGTTCACCCTATTTCAGCATTGGGATGC
3_known	M22	Tb927.11.4120_for	GGGGACAAGTTTGTACAAAAAAGCAGGCTATGCTAATGAACACCCCGCTAAAG	Tb927.11.4120_rev	GGGGACCACTTTGTACAAGAAAGCTGGGTCTACCACAGCTTCACAGCCAC
3_known	M23	Tb927.10.5080_for	GGGGACAAGTTTGTACAAAAAAGCAGGCTATGTTGGGCAACAAAGTCGTGT	Tb927.10.5080_rev	GGGGACCACTTTGTACAAGAAAGCTGGGTTCAAGCCTGCTTTTCTTTGGTAATGC
3_known	M24	Tb927.10.8940_for	GGGGACAAGTTTGTACAAAAAAGCAGGCTATGTCTGCAACCGTCGCAC	Tb927.10.8940_rev	GGGGACCACTTTGTACAAGAAAGCTGGGTTTAACACAGTGAGATTGAACTTCCCC
3_known	N1	Tb927.6.1780_for	GGGGACAAGTTTGTACAAAAAAGCAGGCTATGTTGACGCATGTGTCCAGTC	Tb927.6.1780_rev	GGGGACCACTTTGTACAAGAAAGCTGGGTTTACCGTCTCGAAGCACTGAAATC
3_known	N2	Tb927.9.5630_for	GGGGACAAGTTTGTACAAAAAAGCAGGCTATGTTTCAAAAAGCACTTCGTCGAC	Tb927.9.5630_rev	GGGGACCACTTTGTACAAGAAAGCTGGGTCTACACACCTTTAGAGAGTGATTGAGTAAC
3_known	N3	Tb927.11.1540_for	GGGGACAAGTTTGTACAAAAAAGCAGGCTATGCGCTCGTATCGTTTAGCG	Tb927.11.1540_rev	GGGGACCACTTTGTACAAGAAAGCTGGGTTTAAAGACTGTTGATCAAATCTTTCGTAAT
3_known	N4	Tb927.10.13290_for	GGGGACAAGTTTGTACAAAAAAGCAGGCTATGGGAGTAGTAGACTTACTCACTTCTACG	Tb927.10.13290_rev	GGGGACCACTTTGTACAAGAAAGCTGGGTTCACTCCACTTTCCTTACGGAGC
3_known	N5	Tb927.11.900_for	GGGGACAAGTTTGTACAAAAAAGCAGGCTATGAACAAGATTAGTGCTACCGGTG	Tb927.11.900_rev	GGGGACCACTTTGTACAAGAAAGCTGGGTCTACACTTTGCTTTTCTGCATTGCC
3_known	N6	Tb927.4.2450_for	GGGGACAAGTTTGTACAAAAAAGCAGGCTATGACGATGAAGCGCCCTTC	Tb927.4.2450_rev	GGGGACCACTTTGTACAAGAAAGCTGGGTTTACTTCGGCTGCTCCTTCGT
3_known	N7	Tb927.9.15490_for	GGGGACAAGTTTGTACAAAAAAGCAGGCTATGCCACCAACTTTACCAAAGGATT	Tb927.9.15490_rev	GGGGACCACTTTGTACAAGAAAGCTGGGTCTAAAGATCATCAAGGAATTCCTTTACTTT
3_known	N8	Tb927.10.12040_for	GGGGACAAGTTTGTACAAAAAAGCAGGCTATGAACCGTAGTTCGAGGAACCT	Tb927.10.12040_rev	GGGGACCACTTTGTACAAGAAAGCTGGGTTCACTCAACGGAACCCACATATTTG
3_known	N9	Tb927.11.11590_for	GGGGACAAGTTTGTACAAAAAAGCAGGCTATGACTGAAATGCTGAGCTGTTTGG	Tb927.11.11590_rev	GGGGACCACTTTGTACAAGAAAGCTGGGTTCAGCGCCGACCGTGGTC
3_known	N10	Tb927.10.11310_for	GGGGACAAGTTTGTACAAAAAAGCAGGCTATGAGCGAAGAGACACAACCCC	Tb927.10.11310_rev	GGGGACCACTTTGTACAAGAAAGCTGGGTTTACATATAATCCGTTTCTTCCCACTCATC
3_known	N11	Tb927.11.2990_for	GGGGACAAGTTTGTACAAAAAAGCAGGCTATGTTACGTGTTGAAAACTTGCGG	Tb927.11.2990_rev	GGGGACCACTTTGTACAAGAAAGCTGGGTCTATTTTTTCTTTGTGGCAAGGCGG
3_known	N12	Tb927.11.7060_for	GGGGACAAGTTTGTACAAAAAAGCAGGCTATGAACAACACACACGGTGTGG	Tb927.11.7060_rev	GGGGACCACTTTGTACAAGAAAGCTGGGTTCACAGTTCTCCCCTCTTGTTCA
3_known	N13	Tb927.6.1460_for	GGGGACAAGTTTGTACAAAAAAGCAGGCTATGAACATACGTACAACTGTGACGC	Tb927.6.1460_rev	GGGGACCACTTTGTACAAGAAAGCTGGGTCTATGAATTTCTTGTCGCAAGGCAC
3_known	N14	Tb927.6.1960_for	GGGGACAAGTTTGTACAAAAAAGCAGGCTATGGCTCTGTTCCTCTGGCTT	Tb927.6.1960_rev	GGGGACCACTTTGTACAAGAAAGCTGGGTTCACAAACTGAAGAGTGGGGTGA
3_known	N16	Tb927.11.7320_for	GGGGACAAGTTTGTACAAAAAAGCAGGCTATGAGCCGGTTGTTGTCTCCA	Tb927.11.7320_rev	GGGGACCACTTTGTACAAGAAAGCTGGGTTTACATATGATGCTTTTGATTGTTTAGTTC
3_known	N17	Tb927.8.3860_for	GGGGACAAGTTTGTACAAAAAAGCAGGCTATGGAGCATGTGGATGCTCGG	Tb927.8.3860_rev	GGGGACCACTTTGTACAAGAAAGCTGGGTTCACAGCGCTGCAGTCAC
3_known	N18	Tb927.9.5350_for	GGGGACAAGTTTGTACAAAAAAGCAGGCTATGGCCTACCCGGTAGTTGTAAT	Tb927.9.5350_rev	GGGGACCACTTTGTACAAGAAAGCTGGGTCTACTCAAAAACAGAGTGGAACATGTTG
3_known	N19	Tb927.8.2980_for	GGGGACAAGTTTGTACAAAAAAGCAGGCTATGAGGAGTGCTCAGTTGCCA	Tb927.8.2980_rev	GGGGACCACTTTGTACAAGAAAGCTGGGTTCAACCGCAAGTTGCGCA
3_known	N20	Tb927.9.2050_for	GGGGACAAGTTTGTACAAAAAAGCAGGCTATGCAACCGCAACCAAACGG	Tb927.9.2050_rev	GGGGACCACTTTGTACAAGAAAGCTGGGTCTAAAATATTGTTAGTGTGGCGAAGACCG

3_known	N21	Tb927.6.1230_for	GGGGACAAGTTTGTACAAAAAAGCAGGCTATGCCATCTGTATCGACAGAAGCA	Tb927.6.1230_rev	GGGGACCACTTTGTACAAGAAAGCTGGGTCTATTTTTGCAGCAAGTGTTTTTTTACTAG
3_known	N22	Tb927.10.2290_for	GGGGACAAGTTTGTACAAAAAAGCAGGCTATGGTCAAGGAGACAGAGTACTACG	Tb927.10.2290_rev	GGGGACCACTTTGTACAAGAAAGCTGGGTCTACTGTTGAGTACACTGATGTGCTG
3_known	N23	Tb927.11.1410_for	GGGGACAAGTTTGTACAAAAAAGCAGGCTATGTCACATCGAATAAGTTCAACTAACAAC	Tb927.11.1410_rev	GGGGACCACTTTGTACAAGAAAGCTGGGTTTATGTTTTTCGGCTCCAAGAACTCG
3_known	N24	Tb927.1.3030_for	GGGGACAAGTTTGTACAAAAAAGCAGGCTATGTTGCGTCGCCTCGGT	Tb927.1.3030_rev	GGGGACCACTTTGTACAAGAAAGCTGGGTTCATTCGCTAAAGTCAGGAGACTGA
3_known	O1	Tb927.10.660_for	GGGGACAAGTTTGTACAAAAAAGCAGGCTATGCTCCGAGTCTCGTGCC	Tb927.10.660_rev	GGGGACCACTTTGTACAAGAAAGCTGGGTTCACTTGCCGTAGAATCCCCTG
3_known	O2	Tb927.6.4000_for	GGGGACAAGTTTGTACAAAAAAGCAGGCTATGAGTGGAGCAGCTGAAGGT	Tb927.6.4000_rev	GGGGACCACTTTGTACAAGAAAGCTGGGTTTAGGAGGAAGCACCACCGTT
3_known	O3	Tb927.11.5090_for	GGGGACAAGTTTGTACAAAAAAGCAGGCTATGCTCAATCCCACAACACAGC	Tb927.11.5090_rev	GGGGACCACTTTGTACAAGAAAGCTGGGTTCATTTAGTAACGTTGTGAATAGCTTGGG
3_known	O4	Tb927.11.16810_for	GGGGACAAGTTTGTACAAAAAAGCAGGCTATGAGTATGCAAGGCGGTGC	Tb927.11.16810_rev	GGGGACCACTTTGTACAAGAAAGCTGGGTTTATTTGTCACCCTTGTCCCGTCT
3_known	O5	Tb927.3.2900_for	GGGGACAAGTTTGTACAAAAAAGCAGGCTATGGCAGCTTACGGTATAGTGGA	Tb927.3.2900_rev	GGGGACCACTTTGTACAAGAAAGCTGGGTTCAGTCCGTGTCTTCGCCT
3_known	O6	Tb927.10.8770_for	GGGGACAAGTTTGTACAAAAAAGCAGGCTATGTCGGATGGGGGGAATGTT	Tb927.10.8770_rev	GGGGACCACTTTGTACAAGAAAGCTGGGTTCACACCTGCCCTTCACGT
3_known	O7	Tb927.10.7410_for	GGGGACAAGTTTGTACAAAAAAGCAGGCTATGTTTACTCGCATTGGTCGTGT	Tb927.10.7410_rev	GGGGACCACTTTGTACAAGAAAGCTGGGTTCACGCGGCGAGTTTGCA
3_known	O8	Tb927.9.13380_for	GGGGACAAGTTTGTACAAAAAAGCAGGCTATGTCTGAAAATGTCTTTGAGTTCCGT	Tb927.9.13380_rev	GGGGACCACTTTGTACAAGAAAGCTGGGTCTAATCGTCATCAATCAAATGGTCACTG
3_known	O9	Tb927.7.5880_for	GGGGACAAGTTTGTACAAAAAAGCAGGCTATGCAGCCTGTGCTGGTTG	Tb927.7.5880_rev	GGGGACCACTTTGTACAAGAAAGCTGGGTTTATTTGCCGTAGCGTTTGTCACG
3_known	O10	Tb927.4.3110_for	GGGGACAAGTTTGTACAAAAAAGCAGGCTATGAGTGGCGTGGAACCCG	Tb927.4.3110_rev	GGGGACCACTTTGTACAAGAAAGCTGGGTTTATTCCACCTGAAACCTTCCCTCC
3_known	O11	Tb927.10.9860_for	GGGGACAAGTTTGTACAAAAAAGCAGGCTATGCGTCGATGGCTGCGT	Tb927.10.9860_rev	GGGGACCACTTTGTACAAGAAAGCTGGGTTTAGTGGGAGGAAGAGGCGG
3_known	O12	Tb927.1.1000_for	GGGGACAAGTTTGTACAAAAAAGCAGGCTATGCGTCGCTTATTGTGTCTCTC	Tb927.1.1000_rev	GGGGACCACTTTGTACAAGAAAGCTGGGTTCAAATTTGGATAAAATAATAGACACTGGT
3_known	O13	Tb927.1.710_for	GGGGACAAGTTTGTACAAAAAAGCAGGCTATGTCACTAAAAGAGAGGAAGAGCATTAAT	Tb927.1.710_rev	GGGGACCACTTTGTACAAGAAAGCTGGGTTTACTCCTTTTCGTCCAATACTGTAACG
3_known	O14	Tb927.7.6270_for	GGGGACAAGTTTGTACAAAAAAGCAGGCTATGCCAGCAGAGACCATTGAGC	Tb927.7.6270_rev	GGGGACCACTTTGTACAAGAAAGCTGGGTTCACCGTTTCCGCAGCTTG
3_known	O15	Tb927.10.7100_for	GGGGACAAGTTTGTACAAAAAAGCAGGCTATGAGTTCGGTAAAGAGTAAAGGTTCAAG	Tb927.10.7100_rev	GGGGACCACTTTGTACAAGAAAGCTGGGTTCACAACCGTTTGTCTTCTATGCC
3_known	O16	Tb927.7.2310_for	GGGGACAAGTTTGTACAAAAAAGCAGGCTATGATAGACGAAACATCGCTACGAG	Tb927.7.2310_rev	GGGGACCACTTTGTACAAGAAAGCTGGGTCTATCTCTCCTCTGCAGGATCTCG
3_known	O17	Tb927.9.5440_for	GGGGACAAGTTTGTACAAAAAAGCAGGCTATGCGTATCCCGGAAGGCA	Tb927.9.5440_rev	GGGGACCACTTTGTACAAGAAAGCTGGGTTCACAAACCTCGCCGCAG
3_known	O18	Tb927.9.12450_for	GGGGACAAGTTTGTACAAAAAAGCAGGCTATGCCGGAGGTCGGCACT	Tb927.9.12450_rev	GGGGACCACTTTGTACAAGAAAGCTGGGTTCAATTAGTTAGAGCGAAAAGTTTATTCCT
3_known	O19	Tb927.11.6620_for	GGGGACAAGTTTGTACAAAAAAGCAGGCTATGCAGGAAGGGAAATCGATAAAACC	Tb927.11.6620_rev	GGGGACCACTTTGTACAAGAAAGCTGGGTCTAGAAGAACTTGTCGTAGTATTCATCATC
3_known	O20	Tb927.9.5730_for	GGGGACAAGTTTGTACAAAAAAGCAGGCTATGCCCCGTGAGAACTTCTCA	Tb927.9.5730_rev	GGGGACCACTTTGTACAAGAAAGCTGGGTTTACTGCTGCTGGCACTCCG
3_known	O21	Tb927.11.8260_for	GGGGACAAGTTTGTACAAAAAAGCAGGCTATGAACACCATTTTCCGCCTTTTTC	Tb927.11.8260_rev	GGGGACCACTTTGTACAAGAAAGCTGGGTTTAGAGGGAGGATGTCCCGTACA
3_known	O22	Tb927.6.3690_for	GGGGACAAGTTTGTACAAAAAAGCAGGCTATGTCTTCTAATTGTCGCACTGAAGAA	Tb927.6.3690_rev	GGGGACCACTTTGTACAAGAAAGCTGGGTTCATGGGGCTTGTAAAGGTGGC
3_known	O23	Tb927.3.2110_for	GGGGACAAGTTTGTACAAAAAAGCAGGCTATGAACCACGATGCCATGTCATT	Tb927.3.2110_rev	GGGGACCACTTTGTACAAGAAAGCTGGGTTCAGTAATGCGGAGAGTTTTGTCTC
3_known	O24	Tb927.11.13730_for	GGGGACAAGTTTGTACAAAAAAGCAGGCTATGGACATTGTCGTGAACGATGAC	Tb927.11.13730_rev	GGGGACCACTTTGTACAAGAAAGCTGGGTTTATGATTTTTGACTTTTCAACTCCCGGA
3_known	P1	Tb927.11.5710_for	GGGGACAAGTTTGTACAAAAAAGCAGGCTATGTTTTTTGTGACGTCAATAGTTCGC	Tb927.11.5710_rev	GGGGACCACTTTGTACAAGAAAGCTGGGTTTATGTGTAGCACCCTGATCTATCGT
3_known	P2	Tb927.10.5490_for	GGGGACAAGTTTGTACAAAAAAGCAGGCTATGACATCCACCGCGACGG	Tb927.10.5490_rev	GGGGACCACTTTGTACAAGAAAGCTGGGTCTATGCACGAACTCGCTTACGC
3_known	P3	Tb927.10.16100_for	GGGGACAAGTTTGTACAAAAAAGCAGGCTATGAGCTCACCAGAGAGCAGTG	Tb927.10.16100_rev	GGGGACCACTTTGTACAAGAAAGCTGGGTCTACGAAAACATCTTCTTATATGCCTCACG
3_known	P4	Tb927.5.1660_for	GGGGACAAGTTTGTACAAAAAAGCAGGCTATGCCACCCAAAAATAGGAATCGG	Tb927.5.1660_rev	GGGGACCACTTTGTACAAGAAAGCTGGGTCTACTTAAGATCCACAATCATTATTGTCAT
3_known	P5	Tb927.10.11220_for	GGGGACAAGTTTGTACAAAAAAGCAGGCTATGTGCATCGAACAGCTCGTG	Tb927.10.11220_rev	GGGGACCACTTTGTACAAGAAAGCTGGGTCTACACTGTAGGATTTGGTTGGGGG
3_known	P6	Tb927.7.2400_for	GGGGACAAGTTTGTACAAAAAAGCAGGCTATGTACAAGTACGTTCTTCAGGAAAAATCA	Tb927.7.2400_rev	GGGGACCACTTTGTACAAGAAAGCTGGGTTCACTTTACAATAGCATTTGTTATTCCCGG
3_known	P7	Tb927.4.1870_for	GGGGACAAGTTTGTACAAAAAAGCAGGCTATGCAAGAAGAGGAAGGGAATTTGG	Tb927.4.1870_rev	GGGGACCACTTTGTACAAGAAAGCTGGGTTCAACTGTCACCTGAAAAGTAATGTGC
3_known	P8	Tb927.9.14040_for	GGGGACAAGTTTGTACAAAAAAGCAGGCTATGTTGCATGTCGAGTCATCTGAG	Tb927.9.14040_rev	GGGGACCACTTTGTACAAGAAAGCTGGGTTCACAAAAGTTTTACTGCAATAAGTCGC
3_known	P9	Tb927.4.2020_for	GGGGACAAGTTTGTACAAAAAAGCAGGCTATGATAACCGGCCTTTTCTTCCTTAATAA	Tb927.4.2020_rev	GGGGACCACTTTGTACAAGAAAGCTGGGTTCAAATTGTCCGCACGGCA
3_known	P10	Tb927.1.1020_for	GGGGACAAGTTTGTACAAAAAAGCAGGCTATGGGTTGTGCAGGGAGTCA	Tb927.1.1020_rev	GGGGACCACTTTGTACAAGAAAGCTGGGTCTATCCCACAACACTCGCAGTATC
3_known	P11	Tb927.1.820_for	GGGGACAAGTTTGTACAAAAAAGCAGGCTATGATGAGCAATACCACCAGTAGCG	Tb927.1.820_rev	GGGGACCACTTTGTACAAGAAAGCTGGGTTCAAACCACACGTGTGCACG
3_known	P12	Tb927.3.3930_for	GGGGACAAGTTTGTACAAAAAAGCAGGCTATGTCCCAGGAAGGGGTTCATC	Tb927.3.3930_rev	GGGGACCACTTTGTACAAGAAAGCTGGGTCTACTTTTCATCCAAGTATTGGCGGG
3_known	P13	Tb927.10.5980_for	GGGGACAAGTTTGTACAAAAAAGCAGGCTATGAGCACATCCAATATAGCGCC	Tb927.10.5980_rev	GGGGACCACTTTGTACAAGAAAGCTGGGTCTAAGTCATGTATTTGCTTCCAGACTTCA
3_known	P14	Tb927.11.16720_for	GGGGACAAGTTTGTACAAAAAAGCAGGCTATGAATCCCACGGCACTTCGT	Tb927.11.16720_rev	GGGGACCACTTTGTACAAGAAAGCTGGGTTCAAAAGTCAGGTACTTCACTAGGCA
3_known	P15	Tb927.9.5320_for	GGGGACAAGTTTGTACAAAAAAGCAGGCTATGGTTTTTGCCTTATTGGAGTTGC	Tb927.9.5320_rev	GGGGACCACTTTGTACAAGAAAGCTGGGTTTACCTGAACCCACCGCCG
3_known	P16	Tb927.10.170_for	GGGGACAAGTTTGTACAAAAAAGCAGGCTATGGTCAGTAAGAAGCTTGGTGAGG	Tb927.10.170_rev	GGGGACCACTTTGTACAAGAAAGCTGGGTTTAGGTGTCATCTGCTTCTGACCT
3_known	P17	Tb927.10.7020_for	GGGGACAAGTTTGTACAAAAAAGCAGGCTATGAGGAAGTACGCCATATTTTCCTTAG	Tb927.10.7020_rev	GGGGACCACTTTGTACAAGAAAGCTGGGTCTAACGAATGTCCGTGTCATGGT
3_known	P18	Tb927.9.8950_for	GGGGACAAGTTTGTACAAAAAAGCAGGCTATGGTTGCGTGGGGTGGG	Tb927.9.8950_rev	GGGGACCACTTTGTACAAGAAAGCTGGGTTCATTTCCGTTTCTTATTTTCCTCCTTAAT
3_known	P19	Tb927.6.1870_for	GGGGACAAGTTTGTACAAAAAAGCAGGCTATGCAAAATTTACGTGCTGATGCG	Tb927.6.1870_rev	GGGGACCACTTTGTACAAGAAAGCTGGGTTCACCAAAGCCGACGATTGC
3_known	P20	Tb927.7.6830_for	GGGGACAAGTTTGTACAAAAAAGCAGGCTATGGAGGAACTCCACCAACAAATG	Tb927.7.6830_rev	GGGGACCACTTTGTACAAGAAAGCTGGGTTTAGTACACCATCACGAGTTGCCA
3_known	P21	Tb927.8.4450_for	GGGGACAAGTTTGTACAAAAAAGCAGGCTATGGGGATCAAGGCGAAGAAGG	Tb927.8.4450_rev	GGGGACCACTTTGTACAAGAAAGCTGGGTTTACGACGGGTCGCGCTT
3_known	P22	Tb927.5.610_for	GGGGACAAGTTTGTACAAAAAAGCAGGCTATGTCGCGTACTATTGTGCTTGG	Tb927.5.610_rev	GGGGACCACTTTGTACAAGAAAGCTGGGTCTAAAGAATATTACCTTCTAAGACGTCAGA
3_known	P23	Tb927.3.4670_for	GGGGACAAGTTTGTACAAAAAAGCAGGCTATGACAGACGCTGATCGTTCG	Tb927.3.4670_rev	GGGGACCACTTTGTACAAGAAAGCTGGGTCTACGAATCTTTTGTGTCTTTCTCAGCT
3_known	P24	Tb927.6.2970_for	GGGGACAAGTTTGTACAAAAAAGCAGGCTATGTTGAGTGGCAACACCGAAG	Tb927.6.2970_rev	GGGGACCACTTTGTACAAGAAAGCTGGGTTTAAGTTGGGTTACCACACCGATG
4_hypothetical	A1	Tb927.1.530_for	GGGGACAAGTTTGTACAAAAAAGCAGGCTATGCGTAGTCGGTACATGCGC	Tb927.1.530_rev	GGGGACCACTTTGTACAAGAAAGCTGGGTTCATAAATTAAGTGGAATATATTCCTTTAC
4_hypothetical	A2	Tb927.7.2040_for	GGGGACAAGTTTGTACAAAAAAGCAGGCTATGTGGCGCCAGATCCTTCA	Tb927.7.2040_rev	GGGGACCACTTTGTACAAGAAAGCTGGGTTTAATCGAACATATGGAAGCGAGAAGC
4_hypothetical	A3	Tb927.8.7230_for	GGGGACAAGTTTGTACAAAAAAGCAGGCTATGGAGCTTTCGTTCCCCTTTCT	Tb927.8.7230_rev	GGGGACCACTTTGTACAAGAAAGCTGGGTTTAAACAACCGCATTGTACCCCTG
4_hypothetical	A4	Tb927.11.4880_for	GGGGACAAGTTTGTACAAAAAAGCAGGCTATGTCGGGTGAGAGTGTCTTCA	Tb927.11.4880_rev	GGGGACCACTTTGTACAAGAAAGCTGGGTCTAATTTCGATCATCGTAAACAAATACATG
4_hypothetical	A5	Tb927.10.8110_for	GGGGACAAGTTTGTACAAAAAAGCAGGCTATGAGACGGATACAGTGTTGTATTCCG	Tb927.10.8110_rev	GGGGACCACTTTGTACAAGAAAGCTGGGTTCAATTGGTTTTAGCAACAGCTAACG
4_hypothetical	A6	Tb927.3.5360_for	GGGGACAAGTTTGTACAAAAAAGCAGGCTATGGAGAGGGTAACATACGCTCG	Tb927.3.5360_rev	GGGGACCACTTTGTACAAGAAAGCTGGGTTTAAGCTCGGACAATCAAGTACTTCC
4_hypothetical	A7	Tb927.4.710_for	GGGGACAAGTTTGTACAAAAAAGCAGGCTATGACTATATTCTACGAGTACCCTCGC	Tb927.4.710_rev	GGGGACCACTTTGTACAAGAAAGCTGGGTTCATCCAAAGGGAGGAAACCCC
4_hypothetical	A8	Tb927.4.5160_for	GGGGACAAGTTTGTACAAAAAAGCAGGCTATGGAACTTTCGTTCGCCTTTCT	Tb927.4.5160_rev	GGGGACCACTTTGTACAAGAAAGCTGGGTTTAGGCAAGTGCATCATACCCATG
4_hypothetical	A9	Tb927.11.1970_for	GGGGACAAGTTTGTACAAAAAAGCAGGCTATGGTCGATCGAATTGAAGCAACA	Tb927.11.1970_rev	GGGGACCACTTTGTACAAGAAAGCTGGGTTCACATCTTTCCTCGCCCAAAC
4_hypothetical	A10	Tb927.9.5000_for	GGGGACAAGTTTGTACAAAAAAGCAGGCTATGCTGATTAGTGCTGCTGATGTG	Tb927.9.5000_rev	GGGGACCACTTTGTACAAGAAAGCTGGGTTCACTCGCCTTGACTTCTACCTT
4_hypothetical	A11	Tb927.11.13450_for	GGGGACAAGTTTGTACAAAAAAGCAGGCTATGATGAGTAGTGCAACGTTAAGTGAT	Tb927.11.13450_rev	GGGGACCACTTTGTACAAGAAAGCTGGGTTTACCTGGTAACGGTAAAAGGCCT
4_hypothetical	A12	Tb927.7.4620_for	GGGGACAAGTTTGTACAAAAAAGCAGGCTATGAGGTACCGACTGCGTTTAGA	Tb927.7.4620_rev	GGGGACCACTTTGTACAAGAAAGCTGGGTTCAGAACCATTTCCTGGATCGGT
4_hypothetical	A13	Tb927.5.1970_for	GGGGACAAGTTTGTACAAAAAAGCAGGCTATGGAGTCATATCCCAACAGCCC	Tb927.5.1970_rev	GGGGACCACTTTGTACAAGAAAGCTGGGTTTAAAAATAGTAAAACTGCCCAATCCCATG
4_hypothetical	A14	Tb927.10.4290_for	GGGGACAAGTTTGTACAAAAAAGCAGGCTATGCAGAAGCTTGCTGATCGC	Tb927.10.4290_rev	GGGGACCACTTTGTACAAGAAAGCTGGGTTCACTGTTCCACCTCCAGGAG
4_hypothetical	A15	Tb927.4.1030_for	GGGGACAAGTTTGTACAAAAAAGCAGGCTATGCAGAGCCCGAGTGCG	Tb927.4.1030_rev	GGGGACCACTTTGTACAAGAAAGCTGGGTTCAGTTAATCCGATGCTTCTTCCG
4_hypothetical	A16	Tb927.10.2330_for	GGGGACAAGTTTGTACAAAAAAGCAGGCTATGGAAAACAAGGACGTTGCAACC	Tb927.10.2330_rev	GGGGACCACTTTGTACAAGAAAGCTGGGTTTACCTGCCACTGTTTTCATTTCCG
4_hypothetical	A17	Tb927.10.16180_for	GGGGACAAGTTTGTACAAAAAAGCAGGCTATGCTCTTCCCGCACCTTAGT	Tb927.10.16180_rev	GGGGACCACTTTGTACAAGAAAGCTGGGTTTACACAGCAAATGGAAGAATTTTCAAATC
4_hypothetical	A18	Tb927.6.3660_for	GGGGACAAGTTTGTACAAAAAAGCAGGCTATGGAGTCGTCTGTTGCTTCAC	Tb927.6.3660_rev	GGGGACCACTTTGTACAAGAAAGCTGGGTTCAAGAATTGCTGCTACACGACAC
4_hypothetical	A19	Tb927.7.3380_for	GGGGACAAGTTTGTACAAAAAAGCAGGCTATGAACCATGATGATGCGATGTGG	Tb927.7.3380_rev	GGGGACCACTTTGTACAAGAAAGCTGGGTTCACCAATCCAAATCAGCCGTG
4_hypothetical	A20	Tb927.11.4040_for	GGGGACAAGTTTGTACAAAAAAGCAGGCTATGCTTCGAGCTTCTGTGTTGC	Tb927.11.4040_rev	GGGGACCACTTTGTACAAGAAAGCTGGGTCTACTTACCTTTCCCCGTTTTGCA
4_hypothetical	A21	Tb927.9.2120_for	GGGGACAAGTTTGTACAAAAAAGCAGGCTATGCCCCGTGGTGAACGA	Tb927.9.2120_rev	GGGGACCACTTTGTACAAGAAAGCTGGGTCTAGCGATGTCTTTCCAAGAACATGC
4_hypothetical	A22	Tb927.2.2490_for	GGGGACAAGTTTGTACAAAAAAGCAGGCTATGTCAAGTCGCTACTTCGCAG	Tb927.2.2490_rev	GGGGACCACTTTGTACAAGAAAGCTGGGTTTAATTGGTATTACGTGCTGCGGA
4_hypothetical	A23	Tb927.8.1580_for	GGGGACAAGTTTGTACAAAAAAGCAGGCTATGTTACGCTGCGCTACCAG	Tb927.8.1580_rev	GGGGACCACTTTGTACAAGAAAGCTGGGTTCACTGTCCATACGCAATCTGGT
4_hypothetical	A24	Tb927.10.2630_for	GGGGACAAGTTTGTACAAAAAAGCAGGCTATGAGCAGTTTTTGGTCCAAGAAGT	Tb927.10.2630_rev	GGGGACCACTTTGTACAAGAAAGCTGGGTTCAACCACGTTTAAGCTGAAGTAAGC
4_hypothetical	B1	Tb927.11.3110_for	GGGGACAAGTTTGTACAAAAAAGCAGGCTATGTTTGTACTACCGGCTATGAAGGA	Tb927.11.3110_rev	GGGGACCACTTTGTACAAGAAAGCTGGGTTCACTGAATGCAACGAAGTTTTCG
4_hypothetical	B2	Tb927.7.4320_for	GGGGACAAGTTTGTACAAAAAAGCAGGCTATGCTGCAACGTATTCCACCAT	Tb927.7.4320_rev	GGGGACCACTTTGTACAAGAAAGCTGGGTTCACTTCACCGTCAGGAACTGT
4_hypothetical	B3	Tb927.5.2640_for	GGGGACAAGTTTGTACAAAAAAGCAGGCTATGTCTTACAGATTTTTCGCGTCGA	Tb927.5.2640_rev	GGGGACCACTTTGTACAAGAAAGCTGGGTTTAGGAGATACTCACCGGCTCTTTC
4_hypothetical	B4	Tb927.11.8800_for	GGGGACAAGTTTGTACAAAAAAGCAGGCTATGATAAATCAAACAGGCTTCGCAAGC	Tb927.11.8800_rev	GGGGACCACTTTGTACAAGAAAGCTGGGTCTACAGGTTCATTAACCCACGATACAC
4_hypothetical	B5	Tb927.11.4590_for	GGGGACAAGTTTGTACAAAAAAGCAGGCTATGCCATCAGTGTTGGTCTTCCT	Tb927.11.4590_rev	GGGGACCACTTTGTACAAGAAAGCTGGGTTCACGATACTTCAGCGAGCAGC
4_hypothetical	B6	Tb927.11.8280_for	GGGGACAAGTTTGTACAAAAAAGCAGGCTATGGATGATGTTGGTGACGAAGAGG	Tb927.11.8280_rev	GGGGACCACTTTGTACAAGAAAGCTGGGTCTACACACTCTCGTCGTCACCC
4_hypothetical	B7	Tb927.11.4270_for	GGGGACAAGTTTGTACAAAAAAGCAGGCTATGTTCGGTTTAGATCGTTTATCCATCTT	Tb927.11.4270_rev	GGGGACCACTTTGTACAAGAAAGCTGGGTTCAAGAATCCAACTTCGCCCCC
4_hypothetical	B8	Tb927.4.4690_for	GGGGACAAGTTTGTACAAAAAAGCAGGCTATGATTGGAGGGGCGGTGT	Tb927.4.4690_rev	GGGGACCACTTTGTACAAGAAAGCTGGGTTTAGGGATGGATAATCGGCGGAA
4_hypothetical	B9	Tb927.7.2760_for	GGGGACAAGTTTGTACAAAAAAGCAGGCTATGCCAAGACCTGCCCCA	Tb927.7.2760_rev	GGGGACCACTTTGTACAAGAAAGCTGGGTCTAAATGCCCTCAGCAGTAATAGCG
4_hypothetical	B10	Tb927.7.6180_for	GGGGACAAGTTTGTACAAAAAAGCAGGCTATGAACGCTGCCAGTTATAGTGG	Tb927.7.6180_rev	GGGGACCACTTTGTACAAGAAAGCTGGGTCTACTCCTGTTGTGTACCCTTTCGG
4_hypothetical	B11	Tb927.7.7490_for	GGGGACAAGTTTGTACAAAAAAGCAGGCTATGAGTTCCATCCCCGCCA	Tb927.7.7490_rev	GGGGACCACTTTGTACAAGAAAGCTGGGTCTATGATTGTCCCATCCCGCG
4_hypothetical	B12	Tb927.3.4640_for	GGGGACAAGTTTGTACAAAAAAGCAGGCTATGGACAGCTTTCGCCTCACG	Tb927.3.4640_rev	GGGGACCACTTTGTACAAGAAAGCTGGGTTCACACACCGCTACGACAAGC
4_hypothetical	B13	Tb927.8.5040_for	GGGGACAAGTTTGTACAAAAAAGCAGGCTATGGATTACCGACAGAAAGCTGC	Tb927.8.5040_rev	GGGGACCACTTTGTACAAGAAAGCTGGGTCTAACACGCCTCGTTATCCTCG
4_hypothetical	B14	Tb927.9.13770_for	GGGGACAAGTTTGTACAAAAAAGCAGGCTATGGCTTCTGCAGACTCCAAAGC	Tb927.9.13770_rev	GGGGACCACTTTGTACAAGAAAGCTGGGTTCAGTTGTCACTCTCTCCCATTTGC
4_hypothetical	B15	Tb927.2.4980_for	GGGGACAAGTTTGTACAAAAAAGCAGGCTATGCCCCCCTCGCACACG	Tb927.2.4980_rev	GGGGACCACTTTGTACAAGAAAGCTGGGTTTAACGATTGCCGGTGGCTG
4_hypothetical	B16	Tb927.10.7590_for	GGGGACAAGTTTGTACAAAAAAGCAGGCTATGGAGCGTTGTCTCTCTCTCG	Tb927.10.7590_rev	GGGGACCACTTTGTACAAGAAAGCTGGGTTCACTTGACCCACGGTGGG
4_hypothetical	B17	Tb927.7.2260_for	GGGGACAAGTTTGTACAAAAAAGCAGGCTATGAGTGGCGGTGGCCCG	Tb927.7.2260_rev	GGGGACCACTTTGTACAAGAAAGCTGGGTTTAAAGGGTTCTCACAATCACGGTAC
4_hypothetical	B18	Tb927.11.10780_for	GGGGACAAGTTTGTACAAAAAAGCAGGCTATGGGTCCCAGTGAGTACGATG	Tb927.11.10780_rev	GGGGACCACTTTGTACAAGAAAGCTGGGTTCACCTTCCTGTGAAAACAATACCAA
4_hypothetical	B19	Tb927.8.5270_for	GGGGACAAGTTTGTACAAAAAAGCAGGCTATGGACGCCACCAATTCGTG	Tb927.8.5270_rev	GGGGACCACTTTGTACAAGAAAGCTGGGTTCATAAATCATGTGAAAAACTACAACAGCC
4_hypothetical	B20	Tb927.6.3530_for	GGGGACAAGTTTGTACAAAAAAGCAGGCTATGGTTCTCACTACTGTTGTGGGT	Tb927.6.3530_rev	GGGGACCACTTTGTACAAGAAAGCTGGGTTCAATATCCAAAGCCCGCGC
4_hypothetical	B21	Tb927.9.15040_for	GGGGACAAGTTTGTACAAAAAAGCAGGCTATGAAGCGCGCGCGCAAA	Tb927.9.15040_rev	GGGGACCACTTTGTACAAGAAAGCTGGGTTCACGAAGTTCTGTGGTTGACCA
4_hypothetical	B22	Tb927.8.8340_for	GGGGACAAGTTTGTACAAAAAAGCAGGCTATGACTTCCTCTTCTTTTCTAGAGGAACT	Tb927.8.8340_rev	GGGGACCACTTTGTACAAGAAAGCTGGGTTTATTCATTAGCAGAAAGGCACTCCAA
4_hypothetical	B23	Tb927.6.1450_for	GGGGACAAGTTTGTACAAAAAAGCAGGCTATGGCACCATACGTGCGC	Tb927.6.1450_rev	GGGGACCACTTTGTACAAGAAAGCTGGGTCTAAGTGAGGTTCTTACTTCGCTCG
4_hypothetical	B24	Tb927.11.550_for	GGGGACAAGTTTGTACAAAAAAGCAGGCTATGGCTGCGACACGTGCT	Tb927.11.550_rev	GGGGACCACTTTGTACAAGAAAGCTGGGTTTAGTTGTAGCGGCCGTTGTAAC
4_hypothetical	C1	Tb927.7.3890_for	GGGGACAAGTTTGTACAAAAAAGCAGGCTATGGAGGGTATGCTTCTGGATGC	Tb927.7.3890_rev	GGGGACCACTTTGTACAAGAAAGCTGGGTTCACAGTATTCCACAAATTATCGACCG
4_hypothetical	C2	Tb927.9.8520_for	GGGGACAAGTTTGTACAAAAAAGCAGGCTATGAGCACCGCTGCGACT	Tb927.9.8520_rev	GGGGACCACTTTGTACAAGAAAGCTGGGTTTACTCATCATCAAGAAATTTGGATGTTTT
4_hypothetical	C3	Tb927.3.1150_for	GGGGACAAGTTTGTACAAAAAAGCAGGCTATGGGCAAATACGCTGTTGTCG	Tb927.3.1150_rev	GGGGACCACTTTGTACAAGAAAGCTGGGTTTACCACATCACATCCTCACCGG
4_hypothetical	C4	Tb927.11.13410_for	GGGGACAAGTTTGTACAAAAAAGCAGGCTATGTGGACACCTCGTTCCGA	Tb927.11.13410_rev	GGGGACCACTTTGTACAAGAAAGCTGGGTTCACACACGTGAAATTATTTGTTGCAC
4_hypothetical	C5	Tb927.2.2500_for	GGGGACAAGTTTGTACAAAAAAGCAGGCTATGATTCCCACATGCATAGCAATCC	Tb927.2.2500_rev	GGGGACCACTTTGTACAAGAAAGCTGGGTTTAAGTGTATAACGTGCGGCGAA
4_hypothetical	C6	Tb927.10.5430_for	GGGGACAAGTTTGTACAAAAAAGCAGGCTATGGCACAGTCTTTGGTGTGG	Tb927.10.5430_rev	GGGGACCACTTTGTACAAGAAAGCTGGGTCTATTTTTTTCGCTTACCACCCTTCCC
4_hypothetical	C7	Tb927.3.800_for	GGGGACAAGTTTGTACAAAAAAGCAGGCTATGTCCGAATCAGAAAAAATTGATGTAAGT	Tb927.3.800_rev	GGGGACCACTTTGTACAAGAAAGCTGGGTTCACACTTCGATGCTAGATCGCA
4_hypothetical	C8	Tb927.9.7880_for	GGGGACAAGTTTGTACAAAAAAGCAGGCTATGAAAGAGTATGGGACACTTATGTTTTTT	Tb927.9.7880_rev	GGGGACCACTTTGTACAAGAAAGCTGGGTTTACATGTTTTCATTATATTTTGAATTACT
4_hypothetical	C9	Tb927.11.13370_for	GGGGACAAGTTTGTACAAAAAAGCAGGCTATGCTGCACACGGACCCA	Tb927.11.13370_rev	GGGGACCACTTTGTACAAGAAAGCTGGGTTCAGTACCGACAATTGTGGTCTGT
4_hypothetical	C10	Tb927.11.11470_for	GGGGACAAGTTTGTACAAAAAAGCAGGCTATGCGTCGTTTCTGCACTGC	Tb927.11.11470_rev	GGGGACCACTTTGTACAAGAAAGCTGGGTTCAATGGGCACCGGCAGG
4_hypothetical	C11	Tb927.11.5380_for	GGGGACAAGTTTGTACAAAAAAGCAGGCTATGAGATGCAGCCGTGTCACA	Tb927.11.5380_rev	GGGGACCACTTTGTACAAGAAAGCTGGGTTCAGTAGGGATACCGGTGAGAAAC
4_hypothetical	C12	Tb927.11.9660_for	GGGGACAAGTTTGTACAAAAAAGCAGGCTATGGGAAACGCGCCCTCA	Tb927.11.9660_rev	GGGGACCACTTTGTACAAGAAAGCTGGGTTCAACAAAAAGACTTCACTGACGACT
4_hypothetical	C13	Tb927.11.8180_for	GGGGACAAGTTTGTACAAAAAAGCAGGCTATGTCACATTTGGGGGGTCACA	Tb927.11.8180_rev	GGGGACCACTTTGTACAAGAAAGCTGGGTTCATGCAAGCGACGCATGA
4_hypothetical	C14	Tb927.10.8840_for	GGGGACAAGTTTGTACAAAAAAGCAGGCTATGGGCATGCATGAAGGGGC	Tb927.10.8840_rev	GGGGACCACTTTGTACAAGAAAGCTGGGTTCACACAAATTCACGGGGGTG
4_hypothetical	C15	Tb927.10.1370_for	GGGGACAAGTTTGTACAAAAAAGCAGGCTATGTCCGCCTCCTGCTTCA	Tb927.10.1370_rev	GGGGACCACTTTGTACAAGAAAGCTGGGTTCACCAAGTGCCTAGAGGCAG
4_hypothetical	C16	Tb927.11.10940_for	GGGGACAAGTTTGTACAAAAAAGCAGGCTATGTCAACCACCTGCACCAAG	Tb927.11.10940_rev	GGGGACCACTTTGTACAAGAAAGCTGGGTCTACTCATACCTCACTGCAGGATAAGT
4_hypothetical	C17	Tb927.11.15500_for	GGGGACAAGTTTGTACAAAAAAGCAGGCTATGCGTCCGACCTTTCCGG	Tb927.11.15500_rev	GGGGACCACTTTGTACAAGAAAGCTGGGTTTAAGCCGGGTACATATTGTCCTTGA
4_hypothetical	C19	Tb927.9.2560_for	GGGGACAAGTTTGTACAAAAAAGCAGGCTATGGGGCTTTATGGTGCATATGAC	Tb927.9.2560_rev	GGGGACCACTTTGTACAAGAAAGCTGGGTTTATTCTTTCTTCTTCCTTTTACTGACGGA
4_hypothetical	C20	Tb927.4.2870_for	GGGGACAAGTTTGTACAAAAAAGCAGGCTATGAACCGCCAGCAACGC	Tb927.4.2870_rev	GGGGACCACTTTGTACAAGAAAGCTGGGTCTATTTTTTGCGTGAGAGGCGC
4_hypothetical	C21	Tb927.11.15090_for	GGGGACAAGTTTGTACAAAAAAGCAGGCTATGCTTCGTTTCCGTGTCGAA	Tb927.11.15090_rev	GGGGACCACTTTGTACAAGAAAGCTGGGTTTACCCAATTGTTGAATGTGAAGGGT
4_hypothetical	C22	Tb927.10.7560_for	GGGGACAAGTTTGTACAAAAAAGCAGGCTATGAGTGCCCCTACTGAAAGGG	Tb927.10.7560_rev	GGGGACCACTTTGTACAAGAAAGCTGGGTTCACTGTTCACCCCTCTTACTCTTC
4_hypothetical	C23	Tb927.11.6310_for	GGGGACAAGTTTGTACAAAAAAGCAGGCTATGCGCACTGGTTTTCGTAGG	Tb927.11.6310_rev	GGGGACCACTTTGTACAAGAAAGCTGGGTTCAAAAAAAGCCATTATCGTCACCGC
4_hypothetical	C24	Tb927.10.3640_for	GGGGACAAGTTTGTACAAAAAAGCAGGCTATGGAGGGCTTTCGATATTTTACACAG	Tb927.10.3640_rev	GGGGACCACTTTGTACAAGAAAGCTGGGTTCACACAAATACAGAGGCGATACG
4_hypothetical	D1	Tb927.6.4680_for	GGGGACAAGTTTGTACAAAAAAGCAGGCTATGGAGTCTTTTAAGGGGTTGGCG	Tb927.6.4680_rev	GGGGACCACTTTGTACAAGAAAGCTGGGTTCAAGCTGCCACATGTGTTGT
4_hypothetical	D2	Tb927.8.2460_for	GGGGACAAGTTTGTACAAAAAAGCAGGCTATGAAAAAGCGAGTGGCGGT	Tb927.8.2460_rev	GGGGACCACTTTGTACAAGAAAGCTGGGTTTAGTTTTCCGCTTCTGCATCCTC
4_hypothetical	D3	Tb927.3.4520_for	GGGGACAAGTTTGTACAAAAAAGCAGGCTATGCAAATCGCAACCGTGCC	Tb927.3.4520_rev	GGGGACCACTTTGTACAAGAAAGCTGGGTTCAAATAATGGCGGTGAGAGTTGTAG
4_hypothetical	D4	Tb927.10.8760_for	GGGGACAAGTTTGTACAAAAAAGCAGGCTATGCACATTCATGAAACGATCCCT	Tb927.10.8760_rev	GGGGACCACTTTGTACAAGAAAGCTGGGTTTAACATCGGAGTGCGAAGTAGC
4_hypothetical	D5	Tb927.9.7940_for	GGGGACAAGTTTGTACAAAAAAGCAGGCTATGCGAGGTGACAATATGAAAGAGTATG	Tb927.9.7940_rev	GGGGACCACTTTGTACAAGAAAGCTGGGTTTACATGTTTTCATTATATTTTGAATGATT
4_hypothetical	D6	Tb927.6.350_for	GGGGACAAGTTTGTACAAAAAAGCAGGCTATGACGATTCAAGCCGTATCGC	Tb927.6.350_rev	GGGGACCACTTTGTACAAGAAAGCTGGGTTTACATAATCGAGCATCCCGCATTT
4_hypothetical	D7	Tb927.10.15440_for	GGGGACAAGTTTGTACAAAAAAGCAGGCTATGAAGAATGCACTGGAGCAGC	Tb927.10.15440_rev	GGGGACCACTTTGTACAAGAAAGCTGGGTTTACAATGCCCCCTGTGTACTGG

4_hypothetical	D8	Tb927.1.2320_for	GGGGACAAGTTTGTACAAAAAAGCAGGCTATGCGTTGCTGCAGCACG	Tb927.1.2320_rev	GGGGACCACTTTGTACAAGAAAGCTGGGTTCACATATAGAACGTTTTGATGTAATCCCA
4_hypothetical	D10	Tb927.5.1380_for	GGGGACAAGTTTGTACAAAAAAGCAGGCTATGGCGATACTTCTGACACTCGT	Tb927.5.1380_rev	GGGGACCACTTTGTACAAGAAAGCTGGGTTCAGCCACTAAGAAAAAGTAACAAGCA
4_hypothetical	D11	Tb927.9.7860_for	GGGGACAAGTTTGTACAAAAAAGCAGGCTATGAAAGGGCGTGGGAAAGC	Tb927.9.7860_rev	GGGGACCACTTTGTACAAGAAAGCTGGGTTTACATGTTTTCATTATATTTTGAATTATT
4_hypothetical	D12	Tb927.11.9100_for	GGGGACAAGTTTGTACAAAAAAGCAGGCTATGGTCTTTTCAATTCCCTCATATCTTTGC	Tb927.11.9100_rev	GGGGACCACTTTGTACAAGAAAGCTGGGTTCACAGGCGCCTAAACACGA
4_hypothetical	D13	Tb927.10.710_for	GGGGACAAGTTTGTACAAAAAAGCAGGCTATGGCTCATTCAAATGTTGTAGTGGC	Tb927.10.710_rev	GGGGACCACTTTGTACAAGAAAGCTGGGTTCAAAGGTCCCCACGGCTT
4_hypothetical	D14	Tb927.11.11040_for	GGGGACAAGTTTGTACAAAAAAGCAGGCTATGGCAACGTCGGAATCTGAC	Tb927.11.11040_rev	GGGGACCACTTTGTACAAGAAAGCTGGGTTTACACACGTCCCACACTGTTTG
4_hypothetical	D15	Tb927.8.6850_for	GGGGACAAGTTTGTACAAAAAAGCAGGCTATGACTTCATTTATGCTGGCAACTAACT	Tb927.8.6850_rev	GGGGACCACTTTGTACAAGAAAGCTGGGTCTATCGGAACATCCTTCCAACACAG
4_hypothetical	D17	Tb927.10.4530_for	GGGGACAAGTTTGTACAAAAAAGCAGGCTATGATGAATGCAAGCTGGAGGG	Tb927.10.4530_rev	GGGGACCACTTTGTACAAGAAAGCTGGGTCTACCGCTTCCCATCCTCCA
4_hypothetical	D18	Tb927.11.15590_for	GGGGACAAGTTTGTACAAAAAAGCAGGCTATGGGTGACCGTGTGGGC	Tb927.11.15590_rev	GGGGACCACTTTGTACAAGAAAGCTGGGTCTAAGGCCTCTGCCCAGCT
4_hypothetical	D19	Tb927.6.2190_for	GGGGACAAGTTTGTACAAAAAAGCAGGCTATGCTCCGTGTTGTGTCAAGG	Tb927.6.2190_rev	GGGGACCACTTTGTACAAGAAAGCTGGGTCTACTCGGCGACACACTCAGT
4_hypothetical	D20	Tb927.11.15510_for	GGGGACAAGTTTGTACAAAAAAGCAGGCTATGGTCAAGCGGTCCCCC	Tb927.11.15510_rev	GGGGACCACTTTGTACAAGAAAGCTGGGTTCAACTCAGCTCGCGTTGC
4_hypothetical	D21	Tb927.6.3980_for	GGGGACAAGTTTGTACAAAAAAGCAGGCTATGATTATTGACCACGAGCGTCG	Tb927.6.3980_rev	GGGGACCACTTTGTACAAGAAAGCTGGGTCTACCGCGTCTGACCGCG
4_hypothetical	D22	Tb927.10.4230_for	GGGGACAAGTTTGTACAAAAAAGCAGGCTATGGACAGCTGTATCGTTGACGG	Tb927.10.4230_rev	GGGGACCACTTTGTACAAGAAAGCTGGGTTCAGATAAATCGACGATGTGGCAC
4_hypothetical	D23	Tb927.10.10160_for	GGGGACAAGTTTGTACAAAAAAGCAGGCTATGGCAATGCTTTCACAGGCT	Tb927.10.10160_rev	GGGGACCACTTTGTACAAGAAAGCTGGGTTTACCCTTTCCCTTCCTCCTCCT
4_hypothetical	D24	Tb927.9.10070_for	GGGGACAAGTTTGTACAAAAAAGCAGGCTATGGCGAGCATTGGCCCA	Tb927.9.10070_rev	GGGGACCACTTTGTACAAGAAAGCTGGGTTCAGTTCTTGAAAAATACAACTGGTTCTTC
4_hypothetical	E1	Tb927.10.8870_for	GGGGACAAGTTTGTACAAAAAAGCAGGCTATGTCATCGCTTGCCGAGAGA	Tb927.10.8870_rev	GGGGACCACTTTGTACAAGAAAGCTGGGTCTATGTTGCCGCATCCGTACTC
4_hypothetical	E2	Tb927.6.3020_for	GGGGACAAGTTTGTACAAAAAAGCAGGCTATGCAACCAAGAAAAACGGTTAAAAAAGT	Tb927.6.3020_rev	GGGGACCACTTTGTACAAGAAAGCTGGGTTCAAGCTCGTTTCTGGCGTATC
4_hypothetical	E3	Tb927.7.1890_for	GGGGACAAGTTTGTACAAAAAAGCAGGCTATGAATGTTTCTGAGTTTCTAAAAGTTTGG	Tb927.7.1890_rev	GGGGACCACTTTGTACAAGAAAGCTGGGTCTATGACGTAAACCTCTTTCCAAAAAAATC
4_hypothetical	E4	Tb927.1.4400_for	GGGGACAAGTTTGTACAAAAAAGCAGGCTATGCCCATCACTGGACCGT	Tb927.1.4400_rev	GGGGACCACTTTGTACAAGAAAGCTGGGTTCATTGTTGCACAAACCGGGC
4_hypothetical	E5	Tb927.10.3680_for	GGGGACAAGTTTGTACAAAAAAGCAGGCTATGGGAGGCAATAAAGTCTCCGT	Tb927.10.3680_rev	GGGGACCACTTTGTACAAGAAAGCTGGGTTTACATAAAAGAGGATTCGTCGTCGC
4_hypothetical	E6	Tb927.3.1870_for	GGGGACAAGTTTGTACAAAAAAGCAGGCTATGGTGTCTCTAAACCGAAAACAGAAG	Tb927.3.1870_rev	GGGGACCACTTTGTACAAGAAAGCTGGGTTCACAAGTCACCGAAGAAACTCG
4_hypothetical	E7	Tb927.9.7200_for	GGGGACAAGTTTGTACAAAAAAGCAGGCTATGCTCGCGACCCAAACC	Tb927.9.7200_rev	GGGGACCACTTTGTACAAGAAAGCTGGGTTCAGTGTGTGTGTACGTCGGC
4_hypothetical	E8	Tb927.10.12050_for	GGGGACAAGTTTGTACAAAAAAGCAGGCTATGGTACTCCCACGCAAGTTGG	Tb927.10.12050_rev	GGGGACCACTTTGTACAAGAAAGCTGGGTCTACATCTTCCTAAAGGGGTGATAGGC
4_hypothetical	E9	Tb927.10.14250_for	GGGGACAAGTTTGTACAAAAAAGCAGGCTATGGTAGTCTCTCAGATAGGGGTGC	Tb927.10.14250_rev	GGGGACCACTTTGTACAAGAAAGCTGGGTCTACTGAACAAAGTCTGGCATCTCTG
4_hypothetical	E10	Tb927.11.4560_for	GGGGACAAGTTTGTACAAAAAAGCAGGCTATGATGACGTACCGGCCGT	Tb927.11.4560_rev	GGGGACCACTTTGTACAAGAAAGCTGGGTTTACGGGCCACGGCACAA
4_hypothetical	E11	Tb927.9.7960_for	GGGGACAAGTTTGTACAAAAAAGCAGGCTATGCGAGGTGACAATATGAAAGAGTATG	Tb927.9.7960_rev	GGGGACCACTTTGTACAAGAAAGCTGGGTTTATTTCCATAACTGTTTACATGCTTCCGT
4_hypothetical	E12	Tb927.8.670_for	GGGGACAAGTTTGTACAAAAAAGCAGGCTATGTCTCTACTAGTGGCGGGTG	Tb927.8.670_rev	GGGGACCACTTTGTACAAGAAAGCTGGGTTTACGGGGTGGAGAGAGCAG
4_hypothetical	E13	Tb927.8.3990_for	GGGGACAAGTTTGTACAAAAAAGCAGGCTATGCCTCCCAAGCAACATAAGGA	Tb927.8.3990_rev	GGGGACCACTTTGTACAAGAAAGCTGGGTCTACACTGCGGTTAGCGCAA
4_hypothetical	E14	Tb927.10.680_for	GGGGACAAGTTTGTACAAAAAAGCAGGCTATGCGTCGTTCATTAGTATTTCCTCC	Tb927.10.680_rev	GGGGACCACTTTGTACAAGAAAGCTGGGTCTATTTTGCAGCAACGCGATGG
4_hypothetical	E15	Tb927.11.1730_for	GGGGACAAGTTTGTACAAAAAAGCAGGCTATGTGCGAATTCCAGTTTCATGACG	Tb927.11.1730_rev	GGGGACCACTTTGTACAAGAAAGCTGGGTTCAATCGCTCTCATCACTTTCACAA
4_hypothetical	E16	Tb927.7.5100_for	GGGGACAAGTTTGTACAAAAAAGCAGGCTATGGCCTTTTCACTTCCAAAAAAATATTAC	Tb927.7.5100_rev	GGGGACCACTTTGTACAAGAAAGCTGGGTCTATTCTGGAACCGGAGGCTCC
4_hypothetical	E17	Tb927.11.12210_for	GGGGACAAGTTTGTACAAAAAAGCAGGCTATGTTCAGTGAACCTGTGTATCTGAC	Tb927.11.12210_rev	GGGGACCACTTTGTACAAGAAAGCTGGGTCTACACCACTGGGGGAGGG
4_hypothetical	E18	Tb927.9.6320_for	GGGGACAAGTTTGTACAAAAAAGCAGGCTATGAGGTCGACATTACCCTCTGC	Tb927.9.6320_rev	GGGGACCACTTTGTACAAGAAAGCTGGGTTTACGGCGGGCAGTAATGC
4_hypothetical	E19	Tb927.11.12070_for	GGGGACAAGTTTGTACAAAAAAGCAGGCTATGAACGCTAGGAAGCCACTCG	Tb927.11.12070_rev	GGGGACCACTTTGTACAAGAAAGCTGGGTCTACAACTGAAGACTTTCTTTAAGGTCTTT
4_hypothetical	E20	Tb927.11.3790_for	GGGGACAAGTTTGTACAAAAAAGCAGGCTATGAAAGCCAGGCGGGCT	Tb927.11.3790_rev	GGGGACCACTTTGTACAAGAAAGCTGGGTTTACAAAAGCACCGAGGCGG
4_hypothetical	E21	Tb927.11.6390_for	GGGGACAAGTTTGTACAAAAAAGCAGGCTATGCCATCAGATACCACCAAAGAGG	Tb927.11.6390_rev	GGGGACCACTTTGTACAAGAAAGCTGGGTTCAACTGCTGCCGCTCTCT
4_hypothetical	E22	Tb927.9.6760_for	GGGGACAAGTTTGTACAAAAAAGCAGGCTATGCACAAGCAAGGGTTGACA	Tb927.9.6760_rev	GGGGACCACTTTGTACAAGAAAGCTGGGTTCAGTGGATTCCCACAGGGAATC
4_hypothetical	E23	Tb927.5.3700_for	GGGGACAAGTTTGTACAAAAAAGCAGGCTATGCCCGCAACTATTGTGTCC	Tb927.5.3700_rev	GGGGACCACTTTGTACAAGAAAGCTGGGTTTAAGCATTAGCAGTTTGATCAGCAAC
4_hypothetical	E24	Tb927.7.4440_for	GGGGACAAGTTTGTACAAAAAAGCAGGCTATGAAGTCGGGTAAGCTATTGATATTTGG	Tb927.7.4440_rev	GGGGACCACTTTGTACAAGAAAGCTGGGTTCATTTATTACTTTGATTGTTATCACCACA
4_hypothetical	F1	Tb927.5.293b_for	GGGGACAAGTTTGTACAAAAAAGCAGGCTATGGGAGGCGATGAATCGTGC	Tb927.5.293b_rev	GGGGACCACTTTGTACAAGAAAGCTGGGTTCAAATGACACAAAGAAACTTAAATTCTGC
4_hypothetical	F2	Tb927.11.16080_for	GGGGACAAGTTTGTACAAAAAAGCAGGCTATGTCTAAACCCCCGGAACAGT	Tb927.11.16080_rev	GGGGACCACTTTGTACAAGAAAGCTGGGTCTACATGATGCTTTCACTGCGGC
4_hypothetical	F3	Tb927.4.1360_for	GGGGACAAGTTTGTACAAAAAAGCAGGCTATGGCGAAATCTCCTATTGTGGC	Tb927.4.1360_rev	GGGGACCACTTTGTACAAGAAAGCTGGGTTTACATTTTAGAACTGGGAAGCACAAGG
4_hypothetical	F4	Tb927.9.13780_for	GGGGACAAGTTTGTACAAAAAAGCAGGCTATGCTGTGCCGTACCTTTTTACG	Tb927.9.13780_rev	GGGGACCACTTTGTACAAGAAAGCTGGGTCTACGGATAAGGTGCCAGGAAGC
4_hypothetical	F5	Tb927.9.15020_for	GGGGACAAGTTTGTACAAAAAAGCAGGCTATGTTCGCTACGTTGGGGTCG	Tb927.9.15020_rev	GGGGACCACTTTGTACAAGAAAGCTGGGTTCATGATCGCCTCTTCGAACTCC
4_hypothetical	F6	Tb927.10.2190_for	GGGGACAAGTTTGTACAAAAAAGCAGGCTATGTTCCACAACAGTTACCAGTCG	Tb927.10.2190_rev	GGGGACCACTTTGTACAAGAAAGCTGGGTCTAAGCGCCCTCTACTGGTGA
4_hypothetical	F7	Tb927.10.2580_for	GGGGACAAGTTTGTACAAAAAAGCAGGCTATGAATAGGAGCACCTCCCTTGAA	Tb927.10.2580_rev	GGGGACCACTTTGTACAAGAAAGCTGGGTTCAACGTCTTCGATTCTTCCTTTTACC
4_hypothetical	F8	Tb927.7.2620_for	GGGGACAAGTTTGTACAAAAAAGCAGGCTATGGAAGTGTACCGTCGTATCACT	Tb927.7.2620_rev	GGGGACCACTTTGTACAAGAAAGCTGGGTTCACCAGCGGTTAATCGCCT
4_hypothetical	F9	Tb927.6.1190_for	GGGGACAAGTTTGTACAAAAAAGCAGGCTATGGCCGCTACACCTCAGAC	Tb927.6.1190_rev	GGGGACCACTTTGTACAAGAAAGCTGGGTCTACAATGTATATGCCTCAAATGTCTCCA
4_hypothetical	F10	Tb927.10.2730_for	GGGGACAAGTTTGTACAAAAAAGCAGGCTATGTGGGACGTCGTAAGCCG	Tb927.10.2730_rev	GGGGACCACTTTGTACAAGAAAGCTGGGTTTAAGAGGCCTCGGGTTGGG
4_hypothetical	F11	Tb927.11.9960_for	GGGGACAAGTTTGTACAAAAAAGCAGGCTATGGTCTTACTGAGTGTTAAGGGTACG	Tb927.11.9960_rev	GGGGACCACTTTGTACAAGAAAGCTGGGTTCAATTCACCTCATGTTCCTCTCGC
4_hypothetical	F12	Tb927.6.2160_for	GGGGACAAGTTTGTACAAAAAAGCAGGCTATGACCATGAGACGCTTCGGA	Tb927.6.2160_rev	GGGGACCACTTTGTACAAGAAAGCTGGGTTCACGGTGGCAACAGGGT
4_hypothetical	F13	Tb927.10.1660_for	GGGGACAAGTTTGTACAAAAAAGCAGGCTATGTTTCGTCAGGAACTACGCC	Tb927.10.1660_rev	GGGGACCACTTTGTACAAGAAAGCTGGGTCTACTGGAACGGTACAGATCCATGT
4_hypothetical	F14	Tb927.10.13750_for	GGGGACAAGTTTGTACAAAAAAGCAGGCTATGCCGGTGCCGTGGCGT	Tb927.10.13750_rev	GGGGACCACTTTGTACAAGAAAGCTGGGTCTAATCGAAGTGGGCAGTTACCC
4_hypothetical	F15	Tb927.10.6840_for	GGGGACAAGTTTGTACAAAAAAGCAGGCTATGGAGCCACTGGAAAAGCTACA	Tb927.10.6840_rev	GGGGACCACTTTGTACAAGAAAGCTGGGTCTACTCGCCCGGCATTTCC
4_hypothetical	F16	Tb927.5.2580_for	GGGGACAAGTTTGTACAAAAAAGCAGGCTATGGCAGTAATGAGGTGTATCTTGC	Tb927.5.2580_rev	GGGGACCACTTTGTACAAGAAAGCTGGGTTTAGCGCTTACGGAAGTCATCAAC
4_hypothetical	F17	Tb927.1.4500_for	GGGGACAAGTTTGTACAAAAAAGCAGGCTATGAAGGGTGCCGTGCCA	Tb927.1.4500_rev	GGGGACCACTTTGTACAAGAAAGCTGGGTTTACGTGTTGCAATCCACTTTCCC
4_hypothetical	F18	Tb927.8.3460_for	GGGGACAAGTTTGTACAAAAAAGCAGGCTATGGACGGACGGAACGGC	Tb927.8.3460_rev	GGGGACCACTTTGTACAAGAAAGCTGGGTTCAAACAACCTCTTGCGTGCA
4_hypothetical	F19	Tb927.11.7390_for	GGGGACAAGTTTGTACAAAAAAGCAGGCTATGGCCACAGCTAAAGAACTTGG	Tb927.11.7390_rev	GGGGACCACTTTGTACAAGAAAGCTGGGTCTACTTTTCATCACTCGGCGGTG
4_hypothetical	F20	Tb927.3.1480_for	GGGGACAAGTTTGTACAAAAAAGCAGGCTATGACGAGTGCTTCAAAAAAATCAACG	Tb927.3.1480_rev	GGGGACCACTTTGTACAAGAAAGCTGGGTTCATTCGTTCTGAACAATCATTGGTAATAC
4_hypothetical	F21	Tb927.9.7110_for	GGGGACAAGTTTGTACAAAAAAGCAGGCTATGGAGGGAAGCACTGCGC	Tb927.9.7110_rev	GGGGACCACTTTGTACAAGAAAGCTGGGTTTACTGAGTGTACTGAACACCCGG
4_hypothetical	F22	Tb927.11.9600_for	GGGGACAAGTTTGTACAAAAAAGCAGGCTATGTGGCGGCGTGGAGTT	Tb927.11.9600_rev	GGGGACCACTTTGTACAAGAAAGCTGGGTCTATATGTAATGCGCCTCCAACCAC
4_hypothetical	F23	Tb927.3.640_for	GGGGACAAGTTTGTACAAAAAAGCAGGCTATGCTAACGATGATGAGGGGGT	Tb927.3.640_rev	GGGGACCACTTTGTACAAGAAAGCTGGGTTCACCTCGCTGCACTTGACT
4_hypothetical	F24	Tb927.10.12550_for	GGGGACAAGTTTGTACAAAAAAGCAGGCTATGGGGCAGGCGTTGACG	Tb927.10.12550_rev	GGGGACCACTTTGTACAAGAAAGCTGGGTTCAGCCCTTTGTATAATCAACTGTGAC
4_hypothetical	G1	Tb927.3.1980_for	GGGGACAAGTTTGTACAAAAAAGCAGGCTATGTCTACGGGTGCCGAGTT	Tb927.3.1980_rev	GGGGACCACTTTGTACAAGAAAGCTGGGTTCACGGAAAAACAGACGAAGCC
4_hypothetical	G2	Tb927.8.6130_for	GGGGACAAGTTTGTACAAAAAAGCAGGCTATGGACACAACTACCTCGTCTGA	Tb927.8.6130_rev	GGGGACCACTTTGTACAAGAAAGCTGGGTTCACTCACCATCATCCCGTGG
4_hypothetical	G3	Tb927.7.4710_for	GGGGACAAGTTTGTACAAAAAAGCAGGCTATGCGTCCGGTAGCACGT	Tb927.7.4710_rev	GGGGACCACTTTGTACAAGAAAGCTGGGTTCAACTCTCAAATGTGCTGTCAGC
4_hypothetical	G4	Tb927.11.6380_for	GGGGACAAGTTTGTACAAAAAAGCAGGCTATGCATAGGAGTGGCACTTCCG	Tb927.11.6380_rev	GGGGACCACTTTGTACAAGAAAGCTGGGTCTACACAGAAAGCCACTGGACTC
4_hypothetical	G5	Tb927.10.10620_for	GGGGACAAGTTTGTACAAAAAAGCAGGCTATGCAGGAGCGAGAGAGTTTGC	Tb927.10.10620_rev	GGGGACCACTTTGTACAAGAAAGCTGGGTTCAACGCCTCTTTTTCGCACA
4_hypothetical	G6	Tb927.9.7060_for	GGGGACAAGTTTGTACAAAAAAGCAGGCTATGATACTCCCTTCTGCCCCTCC	Tb927.9.7060_rev	GGGGACCACTTTGTACAAGAAAGCTGGGTTCACTGCAGGTTGCGCAAC
4_hypothetical	G7	Tb927.11.16420_for	GGGGACAAGTTTGTACAAAAAAGCAGGCTATGGTGATGAGGGCGCATCT	Tb927.11.16420_rev	GGGGACCACTTTGTACAAGAAAGCTGGGTTTACAGAAGTCGCTTCTGAAGTTTCTC
4_hypothetical	G8	Tb927.7.4290_for	GGGGACAAGTTTGTACAAAAAAGCAGGCTATGGCGACGGACGAACGA	Tb927.7.4290_rev	GGGGACCACTTTGTACAAGAAAGCTGGGTTCAGCATATTTTGGCTTGTGAGAAGT
4_hypothetical	G9	Tb927.6.2950_for	GGGGACAAGTTTGTACAAAAAAGCAGGCTATGGATGGCAAGGAAGATGGTTTAAA	Tb927.6.2950_rev	GGGGACCACTTTGTACAAGAAAGCTGGGTCTAACAAAAGGGCCAGCGGT
4_hypothetical	G10	Tb927.10.920_for	GGGGACAAGTTTGTACAAAAAAGCAGGCTATGCCGCAGGATAAACGATTAATTATAACT	Tb927.10.920_rev	GGGGACCACTTTGTACAAGAAAGCTGGGTTTACTTCTGTATGTGAACCTCCATGAAGC
4_hypothetical	G11	Tb927.6.3330_for	GGGGACAAGTTTGTACAAAAAAGCAGGCTATGGGGGGGAAAACAAATGTAAAGG	Tb927.6.3330_rev	GGGGACCACTTTGTACAAGAAAGCTGGGTTTATAGTGGCAATCCCGTAATAATATCATA
4_hypothetical	G12	Tb927.7.470_for	GGGGACAAGTTTGTACAAAAAAGCAGGCTATGACCCGATATTCACTTGCCCT	Tb927.7.470_rev	GGGGACCACTTTGTACAAGAAAGCTGGGTTCATAGTTGCGTTGGTTTTTGCATT
4_hypothetical	G13	Tb927.9.5410_for	GGGGACAAGTTTGTACAAAAAAGCAGGCTATGATTTACAGCGATAACAACAATCCACG	Tb927.9.5410_rev	GGGGACCACTTTGTACAAGAAAGCTGGGTTTACCTCCCGTGCAGTGTATTCG
4_hypothetical	G14	Tb927.7.2280_for	GGGGACAAGTTTGTACAAAAAAGCAGGCTATGGGGCAGAAGGGGAGAATG	Tb927.7.2280_rev	GGGGACCACTTTGTACAAGAAAGCTGGGTTTAGTCCACATCCCGCACACT
4_hypothetical	G15	Tb927.11.2540_for	GGGGACAAGTTTGTACAAAAAAGCAGGCTATGTCACTGGCTGATATTTTTGCATTTG	Tb927.11.2540_rev	GGGGACCACTTTGTACAAGAAAGCTGGGTCTACAGTTCAAAGGCAATGTCATGGT
4_hypothetical	G16	Tb927.10.11010_for	GGGGACAAGTTTGTACAAAAAAGCAGGCTATGAAGGAGCGGAGAAGTGTCT	Tb927.10.11010_rev	GGGGACCACTTTGTACAAGAAAGCTGGGTTTATCTTCCGCGCGACTTTTTCA
4_hypothetical	G17	Tb927.11.9570_for	GGGGACAAGTTTGTACAAAAAAGCAGGCTATGGACGGTCTTTACTTCACCGC	Tb927.11.9570_rev	GGGGACCACTTTGTACAAGAAAGCTGGGTTTATCCCCCTGAGGGCAACTG
4_hypothetical	G18	Tb927.10.7840_for	GGGGACAAGTTTGTACAAAAAAGCAGGCTATGGATTACAGTGACTTCGATTTTCCG	Tb927.10.7840_rev	GGGGACCACTTTGTACAAGAAAGCTGGGTTTACCAATCAATAATGAAATCGTCGGTGT
4_hypothetical	G19	Tb927.3.2250_for	GGGGACAAGTTTGTACAAAAAAGCAGGCTATGACCGTGAAGAGGGAGACC	Tb927.3.2250_rev	GGGGACCACTTTGTACAAGAAAGCTGGGTTCAAAGACATGCGGACGACAAA
4_hypothetical	G20	Tb927.11.3460_for	GGGGACAAGTTTGTACAAAAAAGCAGGCTATGCTGTCAGGTGCAGAGGA	Tb927.11.3460_rev	GGGGACCACTTTGTACAAGAAAGCTGGGTTTAACGATGCGCCCTCGC
4_hypothetical	G21	Tb927.10.6390_for	GGGGACAAGTTTGTACAAAAAAGCAGGCTATGGCACTCCACCTCTGCC	Tb927.10.6390_rev	GGGGACCACTTTGTACAAGAAAGCTGGGTTCAACATTCCACAGGTTCACAGC
4_hypothetical	G22	Tb927.7.5710_for	GGGGACAAGTTTGTACAAAAAAGCAGGCTATGTTTACCTCAACTGCTGCGTAC	Tb927.7.5710_rev	GGGGACCACTTTGTACAAGAAAGCTGGGTTTACAACAATAACAGCGACAAAATAACAAT
4_hypothetical	G23	Tb927.9.4810_for	GGGGACAAGTTTGTACAAAAAAGCAGGCTATGAGACACGTGGGCTTTGC	Tb927.9.4810_rev	GGGGACCACTTTGTACAAGAAAGCTGGGTCTAAACGTCCTCCCGCGTG
4_hypothetical	G24	Tb927.4.2750_for	GGGGACAAGTTTGTACAAAAAAGCAGGCTATGAGCACAACATCGTCACGTAC	Tb927.4.2750_rev	GGGGACCACTTTGTACAAGAAAGCTGGGTCTACTTTTTTTGGGCTGATCCTTCCG
4_hypothetical	H1	Tb927.11.6100_for	GGGGACAAGTTTGTACAAAAAAGCAGGCTATGAACTCTTTGATGTTGCAAAAGGC	Tb927.11.6100_rev	GGGGACCACTTTGTACAAGAAAGCTGGGTTCACTCACAACCGGTGTCGG
4_hypothetical	H2	Tb927.11.4550_for	GGGGACAAGTTTGTACAAAAAAGCAGGCTATGTCGAATGCACTTGAGAGCAT	Tb927.11.4550_rev	GGGGACCACTTTGTACAAGAAAGCTGGGTTCACGCGTCATCATCTCTGGA
4_hypothetical	H3	Tb927.11.8450_for	GGGGACAAGTTTGTACAAAAAAGCAGGCTATGCATGGTGGCGCAAACT	Tb927.11.8450_rev	GGGGACCACTTTGTACAAGAAAGCTGGGTCTATGCCCTAAGTGCGAAAACTACA
4_hypothetical	H4	Tb927.2.2940_for	GGGGACAAGTTTGTACAAAAAAGCAGGCTATGACGGTCGCAACAGCC	Tb927.2.2940_rev	GGGGACCACTTTGTACAAGAAAGCTGGGTTCATAGCTGCACGGCAACATC
4_hypothetical	H5	Tb927.11.640_for	GGGGACAAGTTTGTACAAAAAAGCAGGCTATGCGAGCCAGTGCTATGACG	Tb927.11.640_rev	GGGGACCACTTTGTACAAGAAAGCTGGGTCTAAATACCATAGTTGAACAGGTGGAGG
4_hypothetical	H6	Tb927.8.4850_for	GGGGACAAGTTTGTACAAAAAAGCAGGCTATGCTGCAGTGGGAGTGGC	Tb927.8.4850_rev	GGGGACCACTTTGTACAAGAAAGCTGGGTTCATATGTTGAAGATGATACCCTCTTCTAT
4_hypothetical	H7	Tb927.7.1620_for	GGGGACAAGTTTGTACAAAAAAGCAGGCTATGTCACTTAAAGCGAAGATCGACG	Tb927.7.1620_rev	GGGGACCACTTTGTACAAGAAAGCTGGGTTTACCTGCAATAGTTTATGTTTACACGAGA
4_hypothetical	H8	Tb927.3.2860_for	GGGGACAAGTTTGTACAAAAAAGCAGGCTATGTTTCGCGGAGTTTTGGCT	Tb927.3.2860_rev	GGGGACCACTTTGTACAAGAAAGCTGGGTTTACACCATTTTGTAACTATATAACCACGG
4_hypothetical	H9	Tb927.10.9150_for	GGGGACAAGTTTGTACAAAAAAGCAGGCTATGGGGAAAGCACCAGTCAAAG	Tb927.10.9150_rev	GGGGACCACTTTGTACAAGAAAGCTGGGTCTACAAATCAGGGTTCACCTCCG
4_hypothetical	H10	Tb927.7.6990_for	GGGGACAAGTTTGTACAAAAAAGCAGGCTATGTATCGATACGCGTCGAGTGC	Tb927.7.6990_rev	GGGGACCACTTTGTACAAGAAAGCTGGGTTTACTTTGCTGTTACCAATGCCGC
4_hypothetical	H11	Tb927.4.860_for	GGGGACAAGTTTGTACAAAAAAGCAGGCTATGGGCCACACCGAACACC	Tb927.4.860_rev	GGGGACCACTTTGTACAAGAAAGCTGGGTTCACAAGAAGAACTCGTCGACAATG
4_hypothetical	H12	Tb927.10.10180_for	GGGGACAAGTTTGTACAAAAAAGCAGGCTATGGGCGCTGCGGATTCG	Tb927.10.10180_rev	GGGGACCACTTTGTACAAGAAAGCTGGGTTTACGCGGACCTCAGCGA
4_hypothetical	H13	Tb927.8.4150_for	GGGGACAAGTTTGTACAAAAAAGCAGGCTATGCTGCGGTTGCGGTGT	Tb927.8.4150_rev	GGGGACCACTTTGTACAAGAAAGCTGGGTCTACACGGCACCTGTGGTCA
4_hypothetical	H14	Tb927.10.4890_for	GGGGACAAGTTTGTACAAAAAAGCAGGCTATGCCGCCTCTCTTTCGCA	Tb927.10.4890_rev	GGGGACCACTTTGTACAAGAAAGCTGGGTCTACGCCCTGCCAAATGCC
4_hypothetical	H15	Tb927.11.7695_for	GGGGACAAGTTTGTACAAAAAAGCAGGCTATGAGCATTAGTTCCAGTGATGCG	Tb927.11.7695_rev	GGGGACCACTTTGTACAAGAAAGCTGGGTCTAATCAAAATGAAGTCCAAGTCGCTG
4_hypothetical	H16	Tb927.10.10720_for	GGGGACAAGTTTGTACAAAAAAGCAGGCTATGGCCGGAGGAAAACTGCT	Tb927.10.10720_rev	GGGGACCACTTTGTACAAGAAAGCTGGGTTTAGTACTCGTACTGAAAATATAACGGACT
4_hypothetical	H17	Tb927.11.15220_for	GGGGACAAGTTTGTACAAAAAAGCAGGCTATGCCGAGGAAAGCGCTTTT	Tb927.11.15220_rev	GGGGACCACTTTGTACAAGAAAGCTGGGTTCACAGCTTGTCCCCGTTGG
4_hypothetical	H18	Tb927.11.3200_for	GGGGACAAGTTTGTACAAAAAAGCAGGCTATGACCTTTTTCTACGCTGCAGC	Tb927.11.3200_rev	GGGGACCACTTTGTACAAGAAAGCTGGGTTCAAGTAATTTTTCTGTAGATAAAAAACGG
4_hypothetical	H19	Tb927.10.9810_for	GGGGACAAGTTTGTACAAAAAAGCAGGCTATGGGCAGTAGTAGCAGCAGT	Tb927.10.9810_rev	GGGGACCACTTTGTACAAGAAAGCTGGGTCTACGCGAAAGGAACATTCCAACT
4_hypothetical	H20	Tb927.4.630_for	GGGGACAAGTTTGTACAAAAAAGCAGGCTATGCAGTTCATTTACTCCTTATTTACGCC	Tb927.4.630_rev	GGGGACCACTTTGTACAAGAAAGCTGGGTCTACTGTTCCTTTAACATATTCTCCACACA
4_hypothetical	H21	Tb927.1.610_for	GGGGACAAGTTTGTACAAAAAAGCAGGCTATGTGTGATCCCCTCGTTGAGA	Tb927.1.610_rev	GGGGACCACTTTGTACAAGAAAGCTGGGTTCACTTTCCACAACCGTGCTTT
4_hypothetical	H22	Tb927.3.2940_for	GGGGACAAGTTTGTACAAAAAAGCAGGCTATGCCTCGTACACTTTTTAAACTTTCCA	Tb927.3.2940_rev	GGGGACCACTTTGTACAAGAAAGCTGGGTTCATTTTTCAATGGCTGCGGAGA
4_hypothetical	H23	Tb927.11.13310_for	GGGGACAAGTTTGTACAAAAAAGCAGGCTATGCCACCTCTGAATAGCCGC	Tb927.11.13310_rev	GGGGACCACTTTGTACAAGAAAGCTGGGTTCATCTCTTCCCCCTGCCCT
4_hypothetical	H24	Tb927.11.5820_for	GGGGACAAGTTTGTACAAAAAAGCAGGCTATGCTTCGCCGGCTCAAC	Tb927.11.5820_rev	GGGGACCACTTTGTACAAGAAAGCTGGGTTTAACGGTCACCAGAACCCAGA
4_hypothetical	I1	Tb927.5.1440_for	GGGGACAAGTTTGTACAAAAAAGCAGGCTATGAACATTGTGGCGAAGAGGG	Tb927.5.1440_rev	GGGGACCACTTTGTACAAGAAAGCTGGGTCTACCTAACAACTTGTTCATTATGAGTTGA
4_hypothetical	I2	Tb927.4.2550_for	GGGGACAAGTTTGTACAAAAAAGCAGGCTATGTCACTGTGGCCGTTTCG	Tb927.4.2550_rev	GGGGACCACTTTGTACAAGAAAGCTGGGTCTATAAATCTCTGTATTTTTTTGCCCGATT
4_hypothetical	I3	Tb927.7.420_for	GGGGACAAGTTTGTACAAAAAAGCAGGCTATGACTGCCCGTTTTTTGTGCT	Tb927.7.420_rev	GGGGACCACTTTGTACAAGAAAGCTGGGTTTAAAAATGTGCGGCAGCACTG
4_hypothetical	I5	Tb927.8.7030_for	GGGGACAAGTTTGTACAAAAAAGCAGGCTATGTATGTCGATTCCGATGAGGAGG	Tb927.8.7030_rev	GGGGACCACTTTGTACAAGAAAGCTGGGTTTAGTCGCTTCCCACTATTTGCAC
4_hypothetical	I6	Tb927.3.730_for	GGGGACAAGTTTGTACAAAAAAGCAGGCTATGGAAGAGGAAGAGGAAACACGT	Tb927.3.730_rev	GGGGACCACTTTGTACAAGAAAGCTGGGTTTAAACTTGAAAAGAGTAGATGCCCAGC
4_hypothetical	I7	Tb927.8.600_for	GGGGACAAGTTTGTACAAAAAAGCAGGCTATGAGTTCCAGCTCTTCTTCAACTTC	Tb927.8.600_rev	GGGGACCACTTTGTACAAGAAAGCTGGGTTTAGTTCATCTCACTTTCGCCACG
4_hypothetical	I8	Tb927.11.7212_for	GGGGACAAGTTTGTACAAAAAAGCAGGCTATGAAACGGTGTAGGCTTGCG	Tb927.11.7212_rev	GGGGACCACTTTGTACAAGAAAGCTGGGTTTAATAACCCTGCTGAATACTTTCCGC
4_hypothetical	I9	Tb927.10.12970_for	GGGGACAAGTTTGTACAAAAAAGCAGGCTATGCAAGTTGGCGTTTGTGACG	Tb927.10.12970_rev	GGGGACCACTTTGTACAAGAAAGCTGGGTTCACATACACCAACAGCCACTCT
4_hypothetical	I10	Tb927.2.3970_for	GGGGACAAGTTTGTACAAAAAAGCAGGCTATGAACGAAAGCAACGGCCG	Tb927.2.3970_rev	GGGGACCACTTTGTACAAGAAAGCTGGGTTTACAGTAACTCATCACAATCCAACTGC
4_hypothetical	I11	Tb927.10.360_for	GGGGACAAGTTTGTACAAAAAAGCAGGCTATGTTTAGTCAGAAGCCGCCAC	Tb927.10.360_rev	GGGGACCACTTTGTACAAGAAAGCTGGGTTCACGAAGACACCTTAAGCTTAGAGC
4_hypothetical	I12	Tb927.5.4010_for	GGGGACAAGTTTGTACAAAAAAGCAGGCTATGAGGATATTTGTCGTGTTTTTAGTCTTG	Tb927.5.4010_rev	GGGGACCACTTTGTACAAGAAAGCTGGGTTCAGAATATACTGAGGAGCAAAAAAGAAAC
4_hypothetical	I13	Tb927.5.3490_for	GGGGACAAGTTTGTACAAAAAAGCAGGCTATGACTGATGTGGCAGAGGGAC	Tb927.5.3490_rev	GGGGACCACTTTGTACAAGAAAGCTGGGTTCATCTATCTATATGATTGGTGTAGTACTC
4_hypothetical	I14	Tb927.11.8080_for	GGGGACAAGTTTGTACAAAAAAGCAGGCTATGAGAGAGAGAAAACCCATTAGGAAGA	Tb927.11.8080_rev	GGGGACCACTTTGTACAAGAAAGCTGGGTCTACATATCCTCGTCTTTAACACCTGC
4_hypothetical	I15	Tb927.10.6450_for	GGGGACAAGTTTGTACAAAAAAGCAGGCTATGGATCACCCTCCCGCAG	Tb927.10.6450_rev	GGGGACCACTTTGTACAAGAAAGCTGGGTCTAAATGAATGTCGTAGCGGACATTACG
4_hypothetical	I16	Tb927.8.6590_for	GGGGACAAGTTTGTACAAAAAAGCAGGCTATGGATGTTAGCGCTGGTGTTG	Tb927.8.6590_rev	GGGGACCACTTTGTACAAGAAAGCTGGGTTTATGACACACAAGAGCCCCGG
4_hypothetical	I17	Tb927.8.2970_for	GGGGACAAGTTTGTACAAAAAAGCAGGCTATGGAGTTGCATATTTTCGACTTCGA	Tb927.8.2970_rev	GGGGACCACTTTGTACAAGAAAGCTGGGTTTAGCTTCTCTTCCGCAAGCCC
4_hypothetical	I18	Tb927.10.4670_for	GGGGACAAGTTTGTACAAAAAAGCAGGCTATGTTGCACATACCACGCATGG	Tb927.10.4670_rev	GGGGACCACTTTGTACAAGAAAGCTGGGTTCATGTGTCCTCAGTGTTGGGA
4_hypothetical	I19	Tb927.9.6420_for	GGGGACAAGTTTGTACAAAAAAGCAGGCTATGGTAGGCTCCAGCTTTTCCT	Tb927.9.6420_rev	GGGGACCACTTTGTACAAGAAAGCTGGGTTTACGTTACTTCCTGTGATGTGAACC
4_hypothetical	I20	Tb927.8.4680_for	GGGGACAAGTTTGTACAAAAAAGCAGGCTATGACTGATCACGAGGGTTCTATAAGG	Tb927.8.4680_rev	GGGGACCACTTTGTACAAGAAAGCTGGGTTTATTCTGTGTGCGTCTGCAGC

4_hypothetical	I21	Tb927.8.1970_for	GGGGACAAGTTTGTACAAAAAAGCAGGCTATGATCGGCGAGTCAGAAGCA	Tb927.8.1970_rev	GGGGACCACTTTGTACAAGAAAGCTGGGTCTACAGCGGTAAAACTTGGACACA
4_hypothetical	I22	Tb927.8.5910_for	GGGGACAAGTTTGTACAAAAAAGCAGGCTATGCTTCCCGTGTCCTGCG	Tb927.8.5910_rev	GGGGACCACTTTGTACAAGAAAGCTGGGTTCAGGCGGTTTCCTCTGACTC
4_hypothetical	I23	Tb927.5.3890_for	GGGGACAAGTTTGTACAAAAAAGCAGGCTATGATGACTGGGGGAGTCGTG	Tb927.5.3890_rev	GGGGACCACTTTGTACAAGAAAGCTGGGTCTACGAGGCGAGTAGTGAGAAACT
4_hypothetical	I24	Tb927.3.3060_for	GGGGACAAGTTTGTACAAAAAAGCAGGCTATGGATTTTGATTTTGTTCGTATGCTACAA	Tb927.3.3060_rev	GGGGACCACTTTGTACAAGAAAGCTGGGTCTAAAACGGATCCGCTGAGGTTATG
4_hypothetical	J1	Tb927.8.4930_for	GGGGACAAGTTTGTACAAAAAAGCAGGCTATGTTTCAGAGGGCTAGATTTCTCCT	Tb927.8.4930_rev	GGGGACCACTTTGTACAAGAAAGCTGGGTTCACAAAACCAAAAGATCTCTGTGAATGC
4_hypothetical	J2	Tb927.8.2420_for	GGGGACAAGTTTGTACAAAAAAGCAGGCTATGTACGGCCCGCACCAC	Tb927.8.2420_rev	GGGGACCACTTTGTACAAGAAAGCTGGGTTCACATAGAGAAAATGTCATCTTCGATGTA
4_hypothetical	J3	Tb927.3.890_for	GGGGACAAGTTTGTACAAAAAAGCAGGCTATGAGCGATGATGGAGAGGAAAATAATAG	Tb927.3.890_rev	GGGGACCACTTTGTACAAGAAAGCTGGGTTCAATAGTCGGTGACCCCTGTT
4_hypothetical	J4	Tb927.3.4590_for	GGGGACAAGTTTGTACAAAAAAGCAGGCTATGGGAGGGGCTGTCGTC	Tb927.3.4590_rev	GGGGACCACTTTGTACAAGAAAGCTGGGTTCAAAACTTTGGAAAGCGTGTGGA
4_hypothetical	J5	Tb927.9.10790_for	GGGGACAAGTTTGTACAAAAAAGCAGGCTATGTCCACGCTGGCATCATC	Tb927.9.10790_rev	GGGGACCACTTTGTACAAGAAAGCTGGGTTCAGTAACCCAAAAGCCTCGGG
4_hypothetical	J6	Tb927.7.3700_for	GGGGACAAGTTTGTACAAAAAAGCAGGCTATGACTGAGCAAACCGAAGCTG	Tb927.7.3700_rev	GGGGACCACTTTGTACAAGAAAGCTGGGTCTAAGCAGGGCTTCCATTTTCTGA
4_hypothetical	J7	Tb927.11.3950_for	GGGGACAAGTTTGTACAAAAAAGCAGGCTATGTTAAAGCATCGAGACAACATATCTTAT	Tb927.11.3950_rev	GGGGACCACTTTGTACAAGAAAGCTGGGTTTAAAAAGAATCACTGCTCTCTACCGC
4_hypothetical	J8	Tb927.9.7850_for	GGGGACAAGTTTGTACAAAAAAGCAGGCTATGCTATTTGGAGTTTTGCTACTGATGT	Tb927.9.7850_rev	GGGGACCACTTTGTACAAGAAAGCTGGGTTTAAAGAAAAAAGGAAAAAAATGAAATCAC
4_hypothetical	J9	Tb927.10.9320_for	GGGGACAAGTTTGTACAAAAAAGCAGGCTATGAAATCATTGGAAGATTTGAACGGATG	Tb927.10.9320_rev	GGGGACCACTTTGTACAAGAAAGCTGGGTTTAAAGTTCCCCAATGAACTCGCC
4_hypothetical	J10	Tb927.3.2590_for	GGGGACAAGTTTGTACAAAAAAGCAGGCTATGTTGACATTTACAGCGTTGTTTCTAG	Tb927.3.2590_rev	GGGGACCACTTTGTACAAGAAAGCTGGGTTTATCCGGTGATAAAGCATACAATTGCG
4_hypothetical	J11	Tb927.4.3370_for	GGGGACAAGTTTGTACAAAAAAGCAGGCTATGTCAAGCAACACGTGCATAAAC	Tb927.4.3370_rev	GGGGACCACTTTGTACAAGAAAGCTGGGTTCAGCCCTTCAATTCGTCGC
4_hypothetical	J12	Tb927.5.980_for	GGGGACAAGTTTGTACAAAAAAGCAGGCTATGAAGCGTTTGTTGGACAGCG	Tb927.5.980_rev	GGGGACCACTTTGTACAAGAAAGCTGGGTCTAAACACGCAACATGTCCTCCA
4_hypothetical	J13	Tb927.5.2610_for	GGGGACAAGTTTGTACAAAAAAGCAGGCTATGAACAGTGACCCCAATGCCT	Tb927.5.2610_rev	GGGGACCACTTTGTACAAGAAAGCTGGGTTTATTTGGTGTGATGTGATGCATGATTG
4_hypothetical	J14	Tb927.1.3170_for	GGGGACAAGTTTGTACAAAAAAGCAGGCTATGAAGGTTTATTTCACATACGAGGGC	Tb927.1.3170_rev	GGGGACCACTTTGTACAAGAAAGCTGGGTTTACAGCAGGTGCGGCCC
4_hypothetical	J15	Tb927.1.3150_for	GGGGACAAGTTTGTACAAAAAAGCAGGCTATGCATGTTGTGAGTGAAATTCAGC	Tb927.1.3150_rev	GGGGACCACTTTGTACAAGAAAGCTGGGTTCATGTTCCCCACTGCTGTGC
4_hypothetical	J16	Tb927.8.7810_for	GGGGACAAGTTTGTACAAAAAAGCAGGCTATGCTCAGTTCCTTGGATCACACG	Tb927.8.7810_rev	GGGGACCACTTTGTACAAGAAAGCTGGGTTCAAGCAACAAACGAACCAACAAC
4_hypothetical	J17	Tb927.4.1880_for	GGGGACAAGTTTGTACAAAAAAGCAGGCTATGCTGCGACAGCATAGTCCT	Tb927.4.1880_rev	GGGGACCACTTTGTACAAGAAAGCTGGGTCTAGTCATCCCTTCGACTACCAATAGG
4_hypothetical	J18	Tb927.3.2740_for	GGGGACAAGTTTGTACAAAAAAGCAGGCTATGGTGCCGCTACCGGCC	Tb927.3.2740_rev	GGGGACCACTTTGTACAAGAAAGCTGGGTTTAACGATCTAAGAACCATTTTTCCCAGA
4_hypothetical	J19	Tb927.1.1300_for	GGGGACAAGTTTGTACAAAAAAGCAGGCTATGACAGAGATTGTGTCGGATAATTACTTT	Tb927.1.1300_rev	GGGGACCACTTTGTACAAGAAAGCTGGGTCTACATGGTGCGTGAAAGTGATATTGG
4_hypothetical	J20	Tb927.7.5670_for	GGGGACAAGTTTGTACAAAAAAGCAGGCTATGGCTACTAACGTTGCTTCCG	Tb927.7.5670_rev	GGGGACCACTTTGTACAAGAAAGCTGGGTTCACTGCAATCGATTTTTTTTCTTGTTTTT
4_hypothetical	J21	Tb927.2.5830_for	GGGGACAAGTTTGTACAAAAAAGCAGGCTATGACGACCGCTTCTGTGAGT	Tb927.2.5830_rev	GGGGACCACTTTGTACAAGAAAGCTGGGTTCACTTCCCAATCACTGAGTCCC
4_hypothetical	J22	Tb927.10.3250_for	GGGGACAAGTTTGTACAAAAAAGCAGGCTATGCGATGTAGTTGCGCTTTTCT	Tb927.10.3250_rev	GGGGACCACTTTGTACAAGAAAGCTGGGTTCAATCTGGAGTCATTCCCGGG
4_hypothetical	J23	Tb927.9.3600_for	GGGGACAAGTTTGTACAAAAAAGCAGGCTATGAGCAACAAGGCATTCCTAACA	Tb927.9.3600_rev	GGGGACCACTTTGTACAAGAAAGCTGGGTCTACAGGAGGATCCAGTCGTTACA
4_hypothetical	J24	Tb927.9.1420_for	GGGGACAAGTTTGTACAAAAAAGCAGGCTATGACTCAGCGGTGGATTAAAAGG	Tb927.9.1420_rev	GGGGACCACTTTGTACAAGAAAGCTGGGTTTATGGTGGGGCATGAGACTGA
4_hypothetical	K1	Tb927.11.9480_for	GGGGACAAGTTTGTACAAAAAAGCAGGCTATGGAAGAAAGGTTTCTTAGAACGCG	Tb927.11.9480_rev	GGGGACCACTTTGTACAAGAAAGCTGGGTTCATACCGCAGCAGCATCCT
4_hypothetical	K2	Tb927.2.2830_for	GGGGACAAGTTTGTACAAAAAAGCAGGCTATGTTTACCTCGAGCGGTTGTAGC	Tb927.2.2830_rev	GGGGACCACTTTGTACAAGAAAGCTGGGTTTATATAATTTCATACACAATGATCTCCAT
4_hypothetical	K3	Tb927.8.6120_for	GGGGACAAGTTTGTACAAAAAAGCAGGCTATGTCGTGGTTCGGTTGGGA	Tb927.8.6120_rev	GGGGACCACTTTGTACAAGAAAGCTGGGTTCAGCGGCTGCTGCTTTC
4_hypothetical	K4	Tb927.3.2100_for	GGGGACAAGTTTGTACAAAAAAGCAGGCTATGACGACTATTACCTCACAGCTGC	Tb927.3.2100_rev	GGGGACCACTTTGTACAAGAAAGCTGGGTTTACAACCCACCAGTAAGTAATATCACAGC
4_hypothetical	K6	Tb927.7.2990_for	GGGGACAAGTTTGTACAAAAAAGCAGGCTATGTCAAACCCACCTAAGCACTACA	Tb927.7.2990_rev	GGGGACCACTTTGTACAAGAAAGCTGGGTTTACTTCTTTCTGCCACCACCCT
4_hypothetical	K8	Tb927.6.2560_for	GGGGACAAGTTTGTACAAAAAAGCAGGCTATGCCACGCCATTTACGCTC	Tb927.6.2560_rev	GGGGACCACTTTGTACAAGAAAGCTGGGTTCAGTTGACCTTCTTGCCCTTGG
4_hypothetical	K9	Tb927.10.4220_for	GGGGACAAGTTTGTACAAAAAAGCAGGCTATGTCGTACCGGCGAAAGGT	Tb927.10.4220_rev	GGGGACCACTTTGTACAAGAAAGCTGGGTCTACGTAAACACTGAAGTACGTTTCGA
4_hypothetical	K10	Tb927.9.7870_for	GGGGACAAGTTTGTACAAAAAAGCAGGCTATGCGAATACTGTTTGGATTATTGTTGC	Tb927.9.7870_rev	GGGGACCACTTTGTACAAGAAAGCTGGGTTCAAATTAAAAAGCATAAAAATGAAATTAG
4_hypothetical	K11	Tb927.11.2460_for	GGGGACAAGTTTGTACAAAAAAGCAGGCTATGTTATCGATGCTGGCGGAAG	Tb927.11.2460_rev	GGGGACCACTTTGTACAAGAAAGCTGGGTTCAACTTTGAAACTTCCCTGGTGAAG
4_hypothetical	K12	Tb927.7.4660_for	GGGGACAAGTTTGTACAAAAAAGCAGGCTATGCGAACACGATTTAGAAACAAGC	Tb927.7.4660_rev	GGGGACCACTTTGTACAAGAAAGCTGGGTTTACTTCCATGGAACACGCCG
4_hypothetical	K13	Tb927.9.11350_for	GGGGACAAGTTTGTACAAAAAAGCAGGCTATGAAGGTGCGGGTGGCC	Tb927.9.11350_rev	GGGGACCACTTTGTACAAGAAAGCTGGGTTCACGAATCTACCACGTGATCGC
4_hypothetical	K14	Tb927.8.2490_for	GGGGACAAGTTTGTACAAAAAAGCAGGCTATGCCAGTGTTTGAGAACAAGCA	Tb927.8.2490_rev	GGGGACCACTTTGTACAAGAAAGCTGGGTTCACAAAACGCGTTGCATTCC
4_hypothetical	K15	Tb06.3A7.960_for	GGGGACAAGTTTGTACAAAAAAGCAGGCTATGGCGACATTCACTTGCGG	Tb06.3A7.960_rev	GGGGACCACTTTGTACAAGAAAGCTGGGTCTATGGGTGCTGGTCAATTAACACA
4_hypothetical	K16	Tb927.8.2450_for	GGGGACAAGTTTGTACAAAAAAGCAGGCTATGGGACAACACAGCGCCA	Tb927.8.2450_rev	GGGGACCACTTTGTACAAGAAAGCTGGGTTTATTGGAGGGATGCCGTGGG
4_hypothetical	K17	Tb927.2.1600_for	GGGGACAAGTTTGTACAAAAAAGCAGGCTATGGCATCACCTTCTGATAGCAGA	Tb927.2.1600_rev	GGGGACCACTTTGTACAAGAAAGCTGGGTCTAAATTTGCACAGCGACAAGAATCG
4_hypothetical	K18	Tb927.11.10460_for	GGGGACAAGTTTGTACAAAAAAGCAGGCTATGTCTGCCGGTAGTGCCG	Tb927.11.10460_rev	GGGGACCACTTTGTACAAGAAAGCTGGGTTTACGTCTGTCTTAGCGTCCGC
4_hypothetical	K19	Tb927.6.3410_for	GGGGACAAGTTTGTACAAAAAAGCAGGCTATGACACTAAAGAACAAGAGACCGGG	Tb927.6.3410_rev	GGGGACCACTTTGTACAAGAAAGCTGGGTTTACCCGTGGGGAGGGGT
4_hypothetical	K20	Tb927.7.4540_for	GGGGACAAGTTTGTACAAAAAAGCAGGCTATGTCCATCCACCAAACAAAAGTCT	Tb927.7.4540_rev	GGGGACCACTTTGTACAAGAAAGCTGGGTCTAGGATGACTCCATAACGACGGA
4_hypothetical	K21	Tb927.9.13340_for	GGGGACAAGTTTGTACAAAAAAGCAGGCTATGTCAAAACTGCGTGACTTATGGT	Tb927.9.13340_rev	GGGGACCACTTTGTACAAGAAAGCTGGGTTTAAAAGGATGTTTCGGGAATCACACC
4_hypothetical	K22	Tb927.9.7950_for	GGGGACAAGTTTGTACAAAAAAGCAGGCTATGCGAACGATAGTTGGAGTTTTGT	Tb927.9.7950_rev	GGGGACCACTTTGTACAAGAAAGCTGGGTTTAAAGAAAAAAGCATAAAAATGAAATTAC
4_hypothetical	K23	Tb927.10.14590_for	GGGGACAAGTTTGTACAAAAAAGCAGGCTATGCGGCGGATAGCTTGTG	Tb927.10.14590_rev	GGGGACCACTTTGTACAAGAAAGCTGGGTTTACTTTCCCGCCTTACAACCTGC
4_hypothetical	K24	Tb927.5.1510_for	GGGGACAAGTTTGTACAAAAAAGCAGGCTATGCGACAATGTGTAGTGCGG	Tb927.5.1510_rev	GGGGACCACTTTGTACAAGAAAGCTGGGTTCAGCTCGTTCCACTGTATGTCG
4_hypothetical	L1	Tb927.8.7190_for	GGGGACAAGTTTGTACAAAAAAGCAGGCTATGCCCCCACACAGGAAGC	Tb927.8.7190_rev	GGGGACCACTTTGTACAAGAAAGCTGGGTCTAGTGGGCAGCATCAGAATTCTG
4_hypothetical	L2	Tb927.11.13120_for	GGGGACAAGTTTGTACAAAAAAGCAGGCTATGCGTTTGGAAGAGGTGGATG	Tb927.11.13120_rev	GGGGACCACTTTGTACAAGAAAGCTGGGTTCACAAAAACAGTTGTGAAAACGAGAC
4_hypothetical	L3	Tb927.4.2270_for	GGGGACAAGTTTGTACAAAAAAGCAGGCTATGGACGAATACGGTGATTTTGACA	Tb927.4.2270_rev	GGGGACCACTTTGTACAAGAAAGCTGGGTTTATATGCAGTCGGCCGCATTG
4_hypothetical	L4	Tb927.11.3400_for	GGGGACAAGTTTGTACAAAAAAGCAGGCTATGGAGGGAAGAGGGGGAGA	Tb927.11.3400_rev	GGGGACCACTTTGTACAAGAAAGCTGGGTCTAATATGTATAGCCGTTGGCGCG
4_hypothetical	L5	Tb927.5.1130_for	GGGGACAAGTTTGTACAAAAAAGCAGGCTATGTGCGGAAAGGACAGCG	Tb927.5.1130_rev	GGGGACCACTTTGTACAAGAAAGCTGGGTTTAGTGGTATGCCGGACCTTCC
4_hypothetical	L6	Tb927.9.9420_for	GGGGACAAGTTTGTACAAAAAAGCAGGCTATGTCGCCTAGGTCGTTGTATGT	Tb927.9.9420_rev	GGGGACCACTTTGTACAAGAAAGCTGGGTTTACTTGCTCAACTCAGAACACCTG
4_hypothetical	L7	Tb927.9.7900_for	GGGGACAAGTTTGTACAAAAAAGCAGGCTATGCAAATACTATTTGGAGTGTTGTTGC	Tb927.9.7900_rev	GGGGACCACTTTGTACAAGAAAGCTGGGTTCAAATTAAAAAGCATAAAAATGAAATTAG
4_hypothetical	L8	Tb927.2.2220_for	GGGGACAAGTTTGTACAAAAAAGCAGGCTATGCATTCACATCTACAAAACACGTTTAG	Tb927.2.2220_rev	GGGGACCACTTTGTACAAGAAAGCTGGGTTTAGTTTCGCTGCCGAGAGAGC
4_hypothetical	L9	Tb927.11.3990_for	GGGGACAAGTTTGTACAAAAAAGCAGGCTATGTTGGATGGGAGGGAATTACGA	Tb927.11.3990_rev	GGGGACCACTTTGTACAAGAAAGCTGGGTTCATTTCTTGGCCTCTGTCTTAATCTTT
4_hypothetical	L10	Tb927.11.14810_for	GGGGACAAGTTTGTACAAAAAAGCAGGCTATGTTCGCTGAGTTAGACTTCGATG	Tb927.11.14810_rev	GGGGACCACTTTGTACAAGAAAGCTGGGTTCACTTATGATGAATGATGCGTCTCAC
4_hypothetical	L11	Tb927.3.5370_for	GGGGACAAGTTTGTACAAAAAAGCAGGCTATGCCATTCTGGAAGTATTTCGTAATTACC	Tb927.3.5370_rev	GGGGACCACTTTGTACAAGAAAGCTGGGTTTATAGCGCATCCTTTATCTTGTTAAAGAT
4_hypothetical	L13	Tb927.11.13830_for	GGGGACAAGTTTGTACAAAAAAGCAGGCTATGTGGCGCTTTCGAGCG	Tb927.11.13830_rev	GGGGACCACTTTGTACAAGAAAGCTGGGTTTAATGGCTCAAAATCCCTTGTCGA
4_hypothetical	L14	Tb927.4.1690_for	GGGGACAAGTTTGTACAAAAAAGCAGGCTATGTCGGACGTGGATGATTTAATTGA	Tb927.4.1690_rev	GGGGACCACTTTGTACAAGAAAGCTGGGTTTAATGCTTTGGCACGTGTCCC
4_hypothetical	L15	Tb927.6.1530_for	GGGGACAAGTTTGTACAAAAAAGCAGGCTATGCGGCCCGTGATTGTG	Tb927.6.1530_rev	GGGGACCACTTTGTACAAGAAAGCTGGGTTTAAAAAAGGAAACCAGAACACCACGT
4_hypothetical	L16	Tb927.8.500_for	GGGGACAAGTTTGTACAAAAAAGCAGGCTATGTGTAAACAGATAGGCGCTATTTTAATA	Tb927.8.500_rev	GGGGACCACTTTGTACAAGAAAGCTGGGTTTAGAAGAAAAGAAAAATAACAATTATCAA
4_hypothetical	L17	Tb927.10.11050_for	GGGGACAAGTTTGTACAAAAAAGCAGGCTATGAAGATGGGGGCTTCAGCG	Tb927.10.11050_rev	GGGGACCACTTTGTACAAGAAAGCTGGGTTTACTTCGGCACTGGGATACGA
4_hypothetical	L18	Tb927.8.3190_for	GGGGACAAGTTTGTACAAAAAAGCAGGCTATGCCTCCGCCGCCATTC	Tb927.8.3190_rev	GGGGACCACTTTGTACAAGAAAGCTGGGTTCACTGCATCGAAAATGAAAACCCC
4_hypothetical	L19	Tb927.10.6820_for	GGGGACAAGTTTGTACAAAAAAGCAGGCTATGGGTTCGACGTCGTCCG	Tb927.10.6820_rev	GGGGACCACTTTGTACAAGAAAGCTGGGTTTAAAATTGTTTCACTAGTTCAACCCAATT
4_hypothetical	L20	Tb927.11.5030_for	GGGGACAAGTTTGTACAAAAAAGCAGGCTATGGGGCCCCGCATATATCC	Tb927.11.5030_rev	GGGGACCACTTTGTACAAGAAAGCTGGGTTTATATCTTGAAGGTCGCAAATTTATCACC
4_hypothetical	L21	Tb927.9.14100_for	GGGGACAAGTTTGTACAAAAAAGCAGGCTATGATACCAGCCGCTATGCTCG	Tb927.9.14100_rev	GGGGACCACTTTGTACAAGAAAGCTGGGTTTACGCGGAATTCAACTCCTCCC
4_hypothetical	L22	Tb927.8.6600_for	GGGGACAAGTTTGTACAAAAAAGCAGGCTATGAATGAAGGTTCTAGCAGAGCG	Tb927.8.6600_rev	GGGGACCACTTTGTACAAGAAAGCTGGGTTCAAGAACAGCCTTGTCTACACGT
4_hypothetical	L23	Tb927.10.15760_for	GGGGACAAGTTTGTACAAAAAAGCAGGCTATGAAATCAGAGGGGACGCCG	Tb927.10.15760_rev	GGGGACCACTTTGTACAAGAAAGCTGGGTTTACGATTGCTCAGCAGCGG
4_hypothetical	L24	Tb927.10.5190_for	GGGGACAAGTTTGTACAAAAAAGCAGGCTATGGGCTCAATGGTAATCGGAGG	Tb927.10.5190_rev	GGGGACCACTTTGTACAAGAAAGCTGGGTTCAGTTAGTGGACTCCTCCAATCTAG
4_hypothetical	M1	Tb927.4.1570_for	GGGGACAAGTTTGTACAAAAAAGCAGGCTATGCCTTTAGACTCACCAGTGGG	Tb927.4.1570_rev	GGGGACCACTTTGTACAAGAAAGCTGGGTTCACAGTTCCAAATCCTTGGTTAAGAG
4_hypothetical	M2	Tb927.9.15260_for	GGGGACAAGTTTGTACAAAAAAGCAGGCTATGCCGGACGTTGCCGAA	Tb927.9.15260_rev	GGGGACCACTTTGTACAAGAAAGCTGGGTTCACAGGCGACGCATCATTAC
4_hypothetical	M3	Tb927.8.7070_for	GGGGACAAGTTTGTACAAAAAAGCAGGCTATGTGGGAAGCCGCTTGC	Tb927.8.7070_rev	GGGGACCACTTTGTACAAGAAAGCTGGGTTCAGTTCACACTCTGCGACCG
4_hypothetical	M4	Tb927.11.12060_for	GGGGACAAGTTTGTACAAAAAAGCAGGCTATGAAGAACACTAATAACCTCCGCG	Tb927.11.12060_rev	GGGGACCACTTTGTACAAGAAAGCTGGGTTTAGCGGCCATACGTCCAAAC
4_hypothetical	M5	Tb927.6.370_for	GGGGACAAGTTTGTACAAAAAAGCAGGCTATGGGCACTGAGATAAGCATGACG	Tb927.6.370_rev	GGGGACCACTTTGTACAAGAAAGCTGGGTTCAGCACACAATTGCGAGGG
4_hypothetical	M6	Tb927.7.7140_for	GGGGACAAGTTTGTACAAAAAAGCAGGCTATGTCTTTAGCAGATGAAGTCCCCC	Tb927.7.7140_rev	GGGGACCACTTTGTACAAGAAAGCTGGGTTTAACGTTGAGCGAGCAGGTC
4_hypothetical	M8	Tb927.9.11750_for	GGGGACAAGTTTGTACAAAAAAGCAGGCTATGCCTGATAAGATTGAAAATAATACCCGC	Tb927.9.11750_rev	GGGGACCACTTTGTACAAGAAAGCTGGGTTCACTTTTGGGGCATGTTCCTG
4_hypothetical	M9	Tb927.11.220_for	GGGGACAAGTTTGTACAAAAAAGCAGGCTATGATTGAAGCGGACGTCATACG	Tb927.11.220_rev	GGGGACCACTTTGTACAAGAAAGCTGGGTTCACATCAACGACCTGGTGGG
4_hypothetical	M10	Tb927.10.11890_for	GGGGACAAGTTTGTACAAAAAAGCAGGCTATGGTGGTGGGACACCGG	Tb927.10.11890_rev	GGGGACCACTTTGTACAAGAAAGCTGGGTTTACTCCGACAAGCCCTTTGGC
4_hypothetical	M11	Tb927.11.10920_for	GGGGACAAGTTTGTACAAAAAAGCAGGCTATGTGGCTTTTCTTTGGAACTTTGTG	Tb927.11.10920_rev	GGGGACCACTTTGTACAAGAAAGCTGGGTTCAGTCTTTCCGGTGACTGGAC
4_hypothetical	M12	Tb927.4.600_for	GGGGACAAGTTTGTACAAAAAAGCAGGCTATGAATGTAATCAATCGCATGCTTTCG	Tb927.4.600_rev	GGGGACCACTTTGTACAAGAAAGCTGGGTCTATTCCTCCCCACCTGTTGTCT
4_hypothetical	M13	Tb927.7.1020_for	GGGGACAAGTTTGTACAAAAAAGCAGGCTATGTCCTCCGGCGAACTGC	Tb927.7.1020_rev	GGGGACCACTTTGTACAAGAAAGCTGGGTCTAGTCGGAGTCGCTGTGAGT
4_hypothetical	M14	Tb927.5.3950_for	GGGGACAAGTTTGTACAAAAAAGCAGGCTATGTACAGCTATAACAACATTATTGAAAAA	Tb927.5.3950_rev	GGGGACCACTTTGTACAAGAAAGCTGGGTTCACAGTTGGTATAACTCTGCTACTCC
4_hypothetical	M15	Tb927.11.1810_for	GGGGACAAGTTTGTACAAAAAAGCAGGCTATGATCGCCGATAAGTGTGGC	Tb927.11.1810_rev	GGGGACCACTTTGTACAAGAAAGCTGGGTTTACCGTTTAAAACAGCAAAATACCCTACT
4_hypothetical	M16	Tb927.4.2130_for	GGGGACAAGTTTGTACAAAAAAGCAGGCTATGGGTAGTGACGTTGATTTGAGGA	Tb927.4.2130_rev	GGGGACCACTTTGTACAAGAAAGCTGGGTTCACCGAATCACGCGTGC
4_hypothetical	M17	Tb927.1.2210_for	GGGGACAAGTTTGTACAAAAAAGCAGGCTATGGGTAGTCCTGCTGCGC	Tb927.1.2210_rev	GGGGACCACTTTGTACAAGAAAGCTGGGTTCATTCGTGTTTGTTTGGCTTTTCG
4_hypothetical	M18	Tb927.11.7970_for	GGGGACAAGTTTGTACAAAAAAGCAGGCTATGCTTGCTGGATTGTTTTATAAACAGATT	Tb927.11.7970_rev	GGGGACCACTTTGTACAAGAAAGCTGGGTTCAGTTTTCCGATATTTGTTCAATGTGC
4_hypothetical	M19	Tb927.10.9110_for	GGGGACAAGTTTGTACAAAAAAGCAGGCTATGTGGCAACGCCTCAGGT	Tb927.10.9110_rev	GGGGACCACTTTGTACAAGAAAGCTGGGTCTAAGTTTGATGGGAAGTCCACGAA
4_hypothetical	M20	Tb927.3.5210_for	GGGGACAAGTTTGTACAAAAAAGCAGGCTATGTTGAGGCTTATCAACAGAGGAAAC	Tb927.3.5210_rev	GGGGACCACTTTGTACAAGAAAGCTGGGTCTACGAATCTGCGTCCTTTACATCTT
4_hypothetical	M21	Tb927.11.15210_for	GGGGACAAGTTTGTACAAAAAAGCAGGCTATGTATGCACGACTGAGTAACACTTC	Tb927.11.15210_rev	GGGGACCACTTTGTACAAGAAAGCTGGGTCTACAAGTCGAAATTCATGAACACTGAATC
4_hypothetical	M22	Tb927.1.2640_for	GGGGACAAGTTTGTACAAAAAAGCAGGCTATGAAGTCAGAGAAAGCAGAGAAGGC	Tb927.1.2640_rev	GGGGACCACTTTGTACAAGAAAGCTGGGTTTAATACAACACTTCCGTACGACTGG
4_hypothetical	M23	Tb927.1.4700_for	GGGGACAAGTTTGTACAAAAAAGCAGGCTATGCATACCGATGACAAGCAGG	Tb927.1.4700_rev	GGGGACCACTTTGTACAAGAAAGCTGGGTTCACACGGCATCAGCGGA
4_hypothetical	M24	Tb927.4.4610_for	GGGGACAAGTTTGTACAAAAAAGCAGGCTATGTTTACTGTATCTTGTAATGTTGCCTTC	Tb927.4.4610_rev	GGGGACCACTTTGTACAAGAAAGCTGGGTTTACCTATGAAGACCCTGCATCGT
4_hypothetical	N1	Tb927.2.4150_for	GGGGACAAGTTTGTACAAAAAAGCAGGCTATGAAGTCTGTGGCCTTTCATTTTCT	Tb927.2.4150_rev	GGGGACCACTTTGTACAAGAAAGCTGGGTTTATGATATATGTGACAAATGTTCTTCTGT
4_hypothetical	N2	Tb927.7.4690_for	GGGGACAAGTTTGTACAAAAAAGCAGGCTATGATCTTGATTTATTACGTATATCTTAGC	Tb927.7.4690_rev	GGGGACCACTTTGTACAAGAAAGCTGGGTTCAAGAGTCCGCGAAAGCTTG
4_hypothetical	N3	Tb927.10.6960_for	GGGGACAAGTTTGTACAAAAAAGCAGGCTATGGTAACACCCGAGCAAGCT	Tb927.10.6960_rev	GGGGACCACTTTGTACAAGAAAGCTGGGTTCAAATGTTCATTGCACTAGCCACA
4_hypothetical	N4	Tb927.10.6920_for	GGGGACAAGTTTGTACAAAAAAGCAGGCTATGGTAACACCCGAGCAAGCT	Tb927.10.6920_rev	GGGGACCACTTTGTACAAGAAAGCTGGGTTCAAATGTTCATTGCACTGCCCA
4_hypothetical	N5	Tb927.7.5700_for	GGGGACAAGTTTGTACAAAAAAGCAGGCTATGTCATCAACACTCCTTGCAACA	Tb927.7.5700_rev	GGGGACCACTTTGTACAAGAAAGCTGGGTTCATCGACGCCCCTCCCT
4_hypothetical	N6	Tb927.7.3970_for	GGGGACAAGTTTGTACAAAAAAGCAGGCTATGACGTTGTCGTCCGGGA	Tb927.7.3970_rev	GGGGACCACTTTGTACAAGAAAGCTGGGTTTAAAAAACATAGGGCGGTGGCA
4_hypothetical	N7	Tb927.10.6570_for	GGGGACAAGTTTGTACAAAAAAGCAGGCTATGCGAGCTGCGAAGGAAC	Tb927.10.6570_rev	GGGGACCACTTTGTACAAGAAAGCTGGGTTCACCACATCGTACTCTCATACTTATAAGA
4_hypothetical	N8	Tb927.4.5220_for	GGGGACAAGTTTGTACAAAAAAGCAGGCTATGTTTCCCAATGCACACTACCC	Tb927.4.5220_rev	GGGGACCACTTTGTACAAGAAAGCTGGGTTTACCCTGTGTTGATCGCCGC
4_hypothetical	N9	Tb927.3.3890_for	GGGGACAAGTTTGTACAAAAAAGCAGGCTATGTGGGTGAGGACATTCCTGC	Tb927.3.3890_rev	GGGGACCACTTTGTACAAGAAAGCTGGGTTCACGGTGCCGCATGGCA
4_hypothetical	N10	Tb927.7.3920_for	GGGGACAAGTTTGTACAAAAAAGCAGGCTATGACCGAGGGGCGTTTTGA	Tb927.7.3920_rev	GGGGACCACTTTGTACAAGAAAGCTGGGTTTATTTGAGCCTTGCGGGCC
4_hypothetical	N11	Tb927.4.2360_for	GGGGACAAGTTTGTACAAAAAAGCAGGCTATGTTCCTGCGGCGTCTGA	Tb927.4.2360_rev	GGGGACCACTTTGTACAAGAAAGCTGGGTCTACACTTTATCTCCCTCCTCTTTTTTCTG
4_hypothetical	N12	Tb927.8.2990_for	GGGGACAAGTTTGTACAAAAAAGCAGGCTATGTACCCGCTGAGTGCCA	Tb927.8.2990_rev	GGGGACCACTTTGTACAAGAAAGCTGGGTTCACGAGCACTTAACAAACTCAGTG
4_hypothetical	N13	Tb927.7.1680_for	GGGGACAAGTTTGTACAAAAAAGCAGGCTATGGAAGAGGAGGTAAACACAACGT	Tb927.7.1680_rev	GGGGACCACTTTGTACAAGAAAGCTGGGTTCACATGAACGGTAAAATACCTTTCTCTAT
4_hypothetical	N14	Tb927.3.4160_for	GGGGACAAGTTTGTACAAAAAAGCAGGCTATGAAGCTGATAGCGAAACTCGG	Tb927.3.4160_rev	GGGGACCACTTTGTACAAGAAAGCTGGGTTTATAACTGCGGCGGGTTCTCG
4_hypothetical	N15	Tb927.11.11960_for	GGGGACAAGTTTGTACAAAAAAGCAGGCTATGATGATATGGCTGCAACGCA	Tb927.11.11960_rev	GGGGACCACTTTGTACAAGAAAGCTGGGTTTAATAAGCGGGGGGGCCC
4_hypothetical	N16	Tb927.7.1450_for	GGGGACAAGTTTGTACAAAAAAGCAGGCTATGTTCGGAGAGCTGGTCTGC	Tb927.7.1450_rev	GGGGACCACTTTGTACAAGAAAGCTGGGTTCATAGAAAGTTGCCGATGCAATCA
4_hypothetical	N17	Tb927.11.3940_for	GGGGACAAGTTTGTACAAAAAAGCAGGCTATGCCGCTCCCCACTGAC	Tb927.11.3940_rev	GGGGACCACTTTGTACAAGAAAGCTGGGTCTACAGTTCAAATCTTTCCGCTGCC
4_hypothetical	N18	Tb927.11.5900_for	GGGGACAAGTTTGTACAAAAAAGCAGGCTATGGAAACCAGTGGCCCTAAGA	Tb927.11.5900_rev	GGGGACCACTTTGTACAAGAAAGCTGGGTTTATTTGTAGGAGTGACTCTTCTTAGCAGC
4_hypothetical	N19	Tb927.5.2550_for	GGGGACAAGTTTGTACAAAAAAGCAGGCTATGCTTCGCCGGTTAGCATG	Tb927.5.2550_rev	GGGGACCACTTTGTACAAGAAAGCTGGGTTTACACATACGCCGCCCACA
4_hypothetical	N20	Tb927.7.6260_for	GGGGACAAGTTTGTACAAAAAAGCAGGCTATGAAAGGGCCAACCGACAAC	Tb927.7.6260_rev	GGGGACCACTTTGTACAAGAAAGCTGGGTTTAATCATCACGATGTAGCATCGCC
4_hypothetical	N21	Tb927.3.1970_for	GGGGACAAGTTTGTACAAAAAAGCAGGCTATGAGTGCCGAGGGACTGAG	Tb927.3.1970_rev	GGGGACCACTTTGTACAAGAAAGCTGGGTTCATCTTCTCCTCCCCTCAAGTTTG
4_hypothetical	N22	Tb927.4.3800_for	GGGGACAAGTTTGTACAAAAAAGCAGGCTATGGAGAAACGGGTGATACCAACA	Tb927.4.3800_rev	GGGGACCACTTTGTACAAGAAAGCTGGGTTCATGACAGAAGAAATAGTATTTGCGTCT
4_hypothetical	N23	Tb927.9.11820_for	GGGGACAAGTTTGTACAAAAAAGCAGGCTATGGGTAGCAAGGGCGACG	Tb927.9.11820_rev	GGGGACCACTTTGTACAAGAAAGCTGGGTTTACCGACTCTTCTGTTCACTGCT
4_hypothetical	N24	Tb927.5.4360_for	GGGGACAAGTTTGTACAAAAAAGCAGGCTATGTTTTCATATTCGTTCAAGGCGC	Tb927.5.4360_rev	GGGGACCACTTTGTACAAGAAAGCTGGGTTCACCCCTCCGCTGGAAATG
4_hypothetical	O1	Tb927.3.3260_for	GGGGACAAGTTTGTACAAAAAAGCAGGCTATGAGCTTTCACGATGACAATCATTTCG	Tb927.3.3260_rev	GGGGACCACTTTGTACAAGAAAGCTGGGTTCACGCAGAACCTGCGAGA
4_hypothetical	O2	Tb927.6.3960_for	GGGGACAAGTTTGTACAAAAAAGCAGGCTATGACGAGACATGAAAATGTGGAGC	Tb927.6.3960_rev	GGGGACCACTTTGTACAAGAAAGCTGGGTTTATGTCAATCTTGATTCCTTCGCTGA
4_hypothetical	O3	Tb927.10.5520_for	GGGGACAAGTTTGTACAAAAAAGCAGGCTATGACATACTCACGCCGCGC	Tb927.10.5520_rev	GGGGACCACTTTGTACAAGAAAGCTGGGTTCATGGCGCAGATTTATCACCG
4_hypothetical	O4	Tb927.11.13850_for	GGGGACAAGTTTGTACAAAAAAGCAGGCTATGTACTCATGTAAGACGGCCCA	Tb927.11.13850_rev	GGGGACCACTTTGTACAAGAAAGCTGGGTTTATTCGGTTGGCTTACTTAGCAACT
4_hypothetical	O5	Tb927.6.4700_for	GGGGACAAGTTTGTACAAAAAAGCAGGCTATGAGATCGTCTCCCCGTTTAGC	Tb927.6.4700_rev	GGGGACCACTTTGTACAAGAAAGCTGGGTCTATGCAAGCCCCGATCGC
4_hypothetical	O6	Tb927.3.4870_for	GGGGACAAGTTTGTACAAAAAAGCAGGCTATGGGAGTAGTCAAAGTGTGGTTGC	Tb927.3.4870_rev	GGGGACCACTTTGTACAAGAAAGCTGGGTCTACCATTGATCTTTAGGCGAAGGTATG
4_hypothetical	O7	Tb927.6.3250_for	GGGGACAAGTTTGTACAAAAAAGCAGGCTATGTCGGATTTAGAGAAACTACGTGC	Tb927.6.3250_rev	GGGGACCACTTTGTACAAGAAAGCTGGGTTCATGCCCCGCCCTTTGC
4_hypothetical	O8	Tb927.6.4260_for	GGGGACAAGTTTGTACAAAAAAGCAGGCTATGTTCGTTTTTTTCGTAGCCCTCG	Tb927.6.4260_rev	GGGGACCACTTTGTACAAGAAAGCTGGGTCTATGGAGTCGAGTTTGTTTCATCTCC
4_hypothetical	O9	Tb927.10.610_for	GGGGACAAGTTTGTACAAAAAAGCAGGCTATGGTAGTGAATACACGCGTGTC	Tb927.10.610_rev	GGGGACCACTTTGTACAAGAAAGCTGGGTCTACGTGGCCTTTGGTGCAG
4_hypothetical	O10	Tb927.10.3580_for	GGGGACAAGTTTGTACAAAAAAGCAGGCTATGTCGTTTCGCTATACGAACAACT	Tb927.10.3580_rev	GGGGACCACTTTGTACAAGAAAGCTGGGTCTACTGCGTAAACCGGAGTTGC

4_hypothetical	O11	Tb927.6.1990_for	GGGGACAAGTTTGTACAAAAAAGCAGGCTATGTTGAGAGATGGTGACCGTTATACC	Tb927.6.1990_rev	GGGGACCACTTTGTACAAGAAAGCTGGGTTTAAGCAAAGTCAACCTTGACTTGCG
4_hypothetical	O12	Tb927.11.10130_for	GGGGACAAGTTTGTACAAAAAAGCAGGCTATGACAGACATGGAACTTGTGGAAG	Tb927.11.10130_rev	GGGGACCACTTTGTACAAGAAAGCTGGGTTCAACGGTAAAAATGTCCGCCA
4_hypothetical	O13	Tb927.10.9260_for	GGGGACAAGTTTGTACAAAAAAGCAGGCTATGACTTCGCTCAACACAGAGC	Tb927.10.9260_rev	GGGGACCACTTTGTACAAGAAAGCTGGGTTCACCACCACTTCGACAGCG
4_hypothetical	O14	Tb927.4.950_for	GGGGACAAGTTTGTACAAAAAAGCAGGCTATGCGGGGGCAACGGAGC	Tb927.4.950_rev	GGGGACCACTTTGTACAAGAAAGCTGGGTTCAAAAACGGTACCTCAGTAACCTCC
4_hypothetical	O15	Tb927.10.7120_for	GGGGACAAGTTTGTACAAAAAAGCAGGCTATGCCTTTAAGGACCGATTACGC	Tb927.10.7120_rev	GGGGACCACTTTGTACAAGAAAGCTGGGTCTAGTAGTCATATACGATTCGATGACAAAA
4_hypothetical	O16	Tb927.10.11960_for	GGGGACAAGTTTGTACAAAAAAGCAGGCTATGAAGTACCTGGAGAATACCACTACTATT	Tb927.10.11960_rev	GGGGACCACTTTGTACAAGAAAGCTGGGTTTAACTGTGTTGCCCATTTACTTGAGC
4_hypothetical	O17	Tb927.6.4510_for	GGGGACAAGTTTGTACAAAAAAGCAGGCTATGAACACATCAATTCGTCTGCTTTCG	Tb927.6.4510_rev	GGGGACCACTTTGTACAAGAAAGCTGGGTTCACAACTTGAGTGCTTCCAACC
4_hypothetical	O18	Tb927.11.9690_for	GGGGACAAGTTTGTACAAAAAAGCAGGCTATGCTAGACAAAGTTGTAGGCGATC	Tb927.11.9690_rev	GGGGACCACTTTGTACAAGAAAGCTGGGTTCACCGGACAGTAAGAGGAAAGT
4_hypothetical	O19	Tb927.9.11560_for	GGGGACAAGTTTGTACAAAAAAGCAGGCTATGTGGCTTTTCTCACGAAGTAATGA	Tb927.9.11560_rev	GGGGACCACTTTGTACAAGAAAGCTGGGTTCACGAGTACCTTCCACGCA
4_hypothetical	O20	Tb927.3.1810_for	GGGGACAAGTTTGTACAAAAAAGCAGGCTATGCGAACAACCACGCATGG	Tb927.3.1810_rev	GGGGACCACTTTGTACAAGAAAGCTGGGTTCACTGGGTGTGCTGTGGC
4_hypothetical	O21	Tb927.5.2040_for	GGGGACAAGTTTGTACAAAAAAGCAGGCTATGGCGGCACTACGCTATTTC	Tb927.5.2040_rev	GGGGACCACTTTGTACAAGAAAGCTGGGTTTAAATGCGGTTAAGTAAACAACGGTTG
4_hypothetical	O22	Tb927.5.1460_for	GGGGACAAGTTTGTACAAAAAAGCAGGCTATGCCCGGTACCGACGAA	Tb927.5.1460_rev	GGGGACCACTTTGTACAAGAAAGCTGGGTTTACGATGCTCCATTCTTTACAAAGAAATT
4_hypothetical	O23	Tb927.10.10400_for	GGGGACAAGTTTGTACAAAAAAGCAGGCTATGATGCAGAAATCAGTCACCTTCTTAC	Tb927.10.10400_rev	GGGGACCACTTTGTACAAGAAAGCTGGGTTTACTTGAAGACGCCACGAGGC
4_hypothetical	O24	Tb927.2.4750_for	GGGGACAAGTTTGTACAAAAAAGCAGGCTATGAAGGGTGGTGGGGGC	Tb927.2.4750_rev	GGGGACCACTTTGTACAAGAAAGCTGGGTCTACTCAATTATACCACACAGAAACGACA
4_hypothetical	P2	Tb927.8.660_for	GGGGACAAGTTTGTACAAAAAAGCAGGCTATGTCCGGACATCGTGCCT	Tb927.8.660_rev	GGGGACCACTTTGTACAAGAAAGCTGGGTTTACTTACAGCGGAGAAGCCGT
4_hypothetical	P3	Tb927.9.10680_for	GGGGACAAGTTTGTACAAAAAAGCAGGCTATGCCACCTAAGGTCCCCAAC	Tb927.9.10680_rev	GGGGACCACTTTGTACAAGAAAGCTGGGTTCAGTGCGCGTCGACGTT
4_hypothetical	P4	Tb927.11.1890_for	GGGGACAAGTTTGTACAAAAAAGCAGGCTATGAACAACAGACATTCGGGATCC	Tb927.11.1890_rev	GGGGACCACTTTGTACAAGAAAGCTGGGTTTAGTTCTCGTTATCGTATGTCTCGATTAT
4_hypothetical	P5	Tb927.7.5360_for	GGGGACAAGTTTGTACAAAAAAGCAGGCTATGAGTTCCCTCTTGAAGGTTCATCA	Tb927.7.5360_rev	GGGGACCACTTTGTACAAGAAAGCTGGGTTCAAGTCACGCACCGCGC
4_hypothetical	P6	Tb927.10.9390_for	GGGGACAAGTTTGTACAAAAAAGCAGGCTATGTTTTTTCTAACCCGCTTCAAACTG	Tb927.10.9390_rev	GGGGACCACTTTGTACAAGAAAGCTGGGTCTACGGCTTTGTAAAGTACACAGCA
4_hypothetical	P7	Tb927.7.340_for	GGGGACAAGTTTGTACAAAAAAGCAGGCTATGGGGAATGTGAGGGCGC	Tb927.7.340_rev	GGGGACCACTTTGTACAAGAAAGCTGGGTCTAGGAAGTTTGCAGTGTCTCCAAT
4_hypothetical	P8	Tb927.11.1750_for	GGGGACAAGTTTGTACAAAAAAGCAGGCTATGTCTCTGCAAGCTGTTGGC	Tb927.11.1750_rev	GGGGACCACTTTGTACAAGAAAGCTGGGTTTAGAAGGTTGGGGGCGGC
4_hypothetical	P9	Tb927.4.4770_for	GGGGACAAGTTTGTACAAAAAAGCAGGCTATGCCGATTTCCATTCTTTACGTGA	Tb927.4.4770_rev	GGGGACCACTTTGTACAAGAAAGCTGGGTTTACCCCGCCGCGTATTCT
4_hypothetical	P10	Tb927.10.4210_for	GGGGACAAGTTTGTACAAAAAAGCAGGCTATGGAAAGCAGATGCCTGAGTAGC	Tb927.10.4210_rev	GGGGACCACTTTGTACAAGAAAGCTGGGTTTACTCTTCTAGGGACATGGTCAGAAG
4_hypothetical	P11	Tb927.7.3310_for	GGGGACAAGTTTGTACAAAAAAGCAGGCTATGTTTTACACGGGCTGCAGC	Tb927.7.3310_rev	GGGGACCACTTTGTACAAGAAAGCTGGGTCTACAGAGAATCCTCTACAATATTAAGACA
4_hypothetical	P12	Tb927.11.14160_for	GGGGACAAGTTTGTACAAAAAAGCAGGCTATGCCGCTTGGTCCCATTG	Tb927.11.14160_rev	GGGGACCACTTTGTACAAGAAAGCTGGGTTCATTTGACAGGTTTACGAATGCTGC
4_hypothetical	P13	Tb927.7.1250_for	GGGGACAAGTTTGTACAAAAAAGCAGGCTATGCAACGCCGCTTGCAG	Tb927.7.1250_rev	GGGGACCACTTTGTACAAGAAAGCTGGGTTTATGATGTTCTCACACATGACGAAAATTT
4_hypothetical	P15	Tb927.4.2940_for	GGGGACAAGTTTGTACAAAAAAGCAGGCTATGAAAAAATGTTGGGTATTCGACTGC	Tb927.4.2940_rev	GGGGACCACTTTGTACAAGAAAGCTGGGTTCACAAAAGGCTAACCTCATCGC
4_hypothetical	P16	Tb927.11.2080_for	GGGGACAAGTTTGTACAAAAAAGCAGGCTATGAATGATTGGATTACTAAGGCTTTGGA	Tb927.11.2080_rev	GGGGACCACTTTGTACAAGAAAGCTGGGTTCACGGGGGCGGGTGCTC
4_hypothetical	P17	Tb927.8.6230_for	GGGGACAAGTTTGTACAAAAAAGCAGGCTATGGTCCGCTGGGGTGGT	Tb927.8.6230_rev	GGGGACCACTTTGTACAAGAAAGCTGGGTCTACGGATGTCCACCGCACA
4_hypothetical	P18	Tb927.11.10950_for	GGGGACAAGTTTGTACAAAAAAGCAGGCTATGTTTCGTTTCAACCAAACCCTCC	Tb927.11.10950_rev	GGGGACCACTTTGTACAAGAAAGCTGGGTTCACGTCTTTTTGTAATTCCCAGTGA
4_hypothetical	P19	Tb927.4.4090_for	GGGGACAAGTTTGTACAAAAAAGCAGGCTATGACTCCAGCTGCTTCTCGC	Tb927.4.4090_rev	GGGGACCACTTTGTACAAGAAAGCTGGGTTTACCGTTCGTATACGTACGATTTTGG
4_hypothetical	P21	Tb927.7.4360_for	GGGGACAAGTTTGTACAAAAAAGCAGGCTATGGGTTTCTTTAAAAAGCGTTATGGTG	Tb927.7.4360_rev	GGGGACCACTTTGTACAAGAAAGCTGGGTCTAGGTTGCCAGTCGGTGTTG
4_hypothetical	P22	Tb927.4.4280_for	GGGGACAAGTTTGTACAAAAAAGCAGGCTATGGAAACGGAGAGCAAGACAAC	Tb927.4.4280_rev	GGGGACCACTTTGTACAAGAAAGCTGGGTTCATTGTCCTGCAGCGGC
4_hypothetical	P23	Tb927.11.5420_for	GGGGACAAGTTTGTACAAAAAAGCAGGCTATGACCCAAGCTGTAGAAATGGC	Tb927.11.5420_rev	GGGGACCACTTTGTACAAGAAAGCTGGGTTCAATTCTTTACTTGTGCTTGACCCT
4_known	A1	Tb927.10.11590_for	GGGGACAAGTTTGTACAAAAAAGCAGGCTATGAAGCCAATTATAAAGTCGATCCTTGA	Tb927.10.11590_rev	GGGGACCACTTTGTACAAGAAAGCTGGGTTCATTGTACCTGTGTTGGGTGGT
4_known	A2	Tb927.11.14910_for	GGGGACAAGTTTGTACAAAAAAGCAGGCTATGAAAGGCGCGATGCCTC	Tb927.11.14910_rev	GGGGACCACTTTGTACAAGAAAGCTGGGTTCAACTCTCCTCCCCATCTTCTG
4_known	A3	Tb927.10.2890_for	GGGGACAAGTTTGTACAAAAAAGCAGGCTATGACGATCCAGAAAGTTCACGG	Tb927.10.2890_rev	GGGGACCACTTTGTACAAGAAAGCTGGGTTTACGACCAACCGGGAAAGCC
4_known	A4	Tb927.9.13280_for	GGGGACAAGTTTGTACAAAAAAGCAGGCTATGTCGCTCACCGCTGAGC	Tb927.9.13280_rev	GGGGACCACTTTGTACAAGAAAGCTGGGTCTAGTGTCCCTTCTCCGGGTTC
4_known	A5	Tb927.10.11970_for	GGGGACAAGTTTGTACAAAAAAGCAGGCTATGCAACAGTCAATTCGGCTTGC	Tb927.10.11970_rev	GGGGACCACTTTGTACAAGAAAGCTGGGTCTAATGGGCAGGAAGCAGGTACT
4_known	A6	Tb927.11.7890_for	GGGGACAAGTTTGTACAAAAAAGCAGGCTATGTCAATGCACAGCGTAGAGG	Tb927.11.7890_rev	GGGGACCACTTTGTACAAGAAAGCTGGGTTCAGCTGGATCTCCCGTCCC
4_known	A7	Tb927.6.3300_for	GGGGACAAGTTTGTACAAAAAAGCAGGCTATGGAACTGCAGTCGCTTATAGACA	Tb927.6.3300_rev	GGGGACCACTTTGTACAAGAAAGCTGGGTCTAAGCTACTTTGATCGACTTCGCT
4_known	A8	Tb927.9.8160_for	GGGGACAAGTTTGTACAAAAAAGCAGGCTATGCTGGGTCGAAAATGCAGTG	Tb927.9.8160_rev	GGGGACCACTTTGTACAAGAAAGCTGGGTTCACACCACTTCATCAGCCCC
4_known	A9	Tb927.1.1310_for	GGGGACAAGTTTGTACAAAAAAGCAGGCTATGTCACTTCTAGTTGCTCTCGTACG	Tb927.1.1310_rev	GGGGACCACTTTGTACAAGAAAGCTGGGTTCACAATGCACCGCGCCC
4_known	A10	Tb927.7.4730_for	GGGGACAAGTTTGTACAAAAAAGCAGGCTATGCTTCGTAGGGGTGAACGC	Tb927.7.4730_rev	GGGGACCACTTTGTACAAGAAAGCTGGGTTCACTCACCGACTGCCCC
4_known	A11	Tb927.9.1540_for	GGGGACAAGTTTGTACAAAAAAGCAGGCTATGTCGAGTGGTGCTTCACAC	Tb927.9.1540_rev	GGGGACCACTTTGTACAAGAAAGCTGGGTTCAAGTAACTATATGATGCATCGCACAA
4_known	A12	Tb927.8.6930_for	GGGGACAAGTTTGTACAAAAAAGCAGGCTATGGCCGAACCGTTCTCGA	Tb927.8.6930_rev	GGGGACCACTTTGTACAAGAAAGCTGGGTTTAGGCGACACCTATACCACGT
4_known	A13	Tb927.4.2700_for	GGGGACAAGTTTGTACAAAAAAGCAGGCTATGTTTCGAACGTTACCCTTGAGG	Tb927.4.2700_rev	GGGGACCACTTTGTACAAGAAAGCTGGGTTCAGCAGGAAGATGCTTCAACG
4_known	A15	Tb927.11.2090_for	GGGGACAAGTTTGTACAAAAAAGCAGGCTATGGAGGTGGCTGTGGGG	Tb927.11.2090_rev	GGGGACCACTTTGTACAAGAAAGCTGGGTTTATGAAGATGCACTAAACTCCTTCCTC
4_known	A16	Tb927.9.8250_for	GGGGACAAGTTTGTACAAAAAAGCAGGCTATGAAAAGTCTTCACTCGCCCCC	Tb927.9.8250_rev	GGGGACCACTTTGTACAAGAAAGCTGGGTTCAAACGGAATTCCTGCGCC
4_known	A17	Tb927.8.6000_for	GGGGACAAGTTTGTACAAAAAAGCAGGCTATGAGCGAAGCAATGGAAAGCA	Tb927.8.6000_rev	GGGGACCACTTTGTACAAGAAAGCTGGGTTCACTCATTCAAGTAGCCCACTTGT
4_known	A18	Tb927.10.8900_for	GGGGACAAGTTTGTACAAAAAAGCAGGCTATGAACGCGCCTCACAGAAC	Tb927.10.8900_rev	GGGGACCACTTTGTACAAGAAAGCTGGGTCTACTCCCACTTCGTGTTGGAGT
4_known	A19	Tb927.10.14950_for	GGGGACAAGTTTGTACAAAAAAGCAGGCTATGTACGGTAACCAGTCTAGTGGTG	Tb927.10.14950_rev	GGGGACCACTTTGTACAAGAAAGCTGGGTTTAAGTACTTCCCTCAGAAGGTTCCG
4_known	A20	Tb927.2.2450_for	GGGGACAAGTTTGTACAAAAAAGCAGGCTATGGGTCGTGCTTCAAAGGAC	Tb927.2.2450_rev	GGGGACCACTTTGTACAAGAAAGCTGGGTTTATCCGACACGCTGCCGT
4_known	A21	Tb927.7.3180_for	GGGGACAAGTTTGTACAAAAAAGCAGGCTATGGCTTCCGTGTTCTATATTCTTGAC	Tb927.7.3180_rev	GGGGACCACTTTGTACAAGAAAGCTGGGTTTACGCCGTCCGAATCTGGT
4_known	A22	Tb927.11.3880_for	GGGGACAAGTTTGTACAAAAAAGCAGGCTATGGTTTGCAGCACGGAGC	Tb927.11.3880_rev	GGGGACCACTTTGTACAAGAAAGCTGGGTCTACTGGCACTTGCAGTGCA
4_known	A23	Tb927.8.6810_for	GGGGACAAGTTTGTACAAAAAAGCAGGCTATGAGTTGCACACCTGCAACG	Tb927.8.6810_rev	GGGGACCACTTTGTACAAGAAAGCTGGGTTTATTCCGTTTCCCCCAGTATCCA
4_known	A24	Tb927.1.1270_for	GGGGACAAGTTTGTACAAAAAAGCAGGCTATGAAGGACATTAGTTGCCCTACGG	Tb927.1.1270_rev	GGGGACCACTTTGTACAAGAAAGCTGGGTTCACACTAAACTGCGTATCATAGCAAT
4_known	B1	Tb927.8.3970_for	GGGGACAAGTTTGTACAAAAAAGCAGGCTATGGGCGCAGGGACGTCG	Tb927.8.3970_rev	GGGGACCACTTTGTACAAGAAAGCTGGGTTTATCGCGCGGTGGCATC
4_known	B2	Tb927.11.16020_for	GGGGACAAGTTTGTACAAAAAAGCAGGCTATGGCAGCAGCGTTACTTAAGAG	Tb927.11.16020_rev	GGGGACCACTTTGTACAAGAAAGCTGGGTTTAGTTTGCCTCATTGTTGGAACTCT
4_known	B3	Tb927.6.4540_for	GGGGACAAGTTTGTACAAAAAAGCAGGCTATGCTTCGTAGGACTCTCTCATGT	Tb927.6.4540_rev	GGGGACCACTTTGTACAAGAAAGCTGGGTTTACTTCCTACGATTCAACCGCATG
4_known	B4	Tb927.10.13870_for	GGGGACAAGTTTGTACAAAAAAGCAGGCTATGCCGGCGGTAGGGGAG	Tb927.10.13870_rev	GGGGACCACTTTGTACAAGAAAGCTGGGTCTACGTCAGAAGGCGCTCAC
4_known	B5	Tb927.5.2280_for	GGGGACAAGTTTGTACAAAAAAGCAGGCTATGGCACTCACTGCACGTG	Tb927.5.2280_rev	GGGGACCACTTTGTACAAGAAAGCTGGGTTTAGGGTATTAAATAGTAGCTCAAGTTACC
4_known	B6	Tb927.10.4870_for	GGGGACAAGTTTGTACAAAAAAGCAGGCTATGTCCATTTTCAATGGGAAAACAATTCTT	Tb927.10.4870_rev	GGGGACCACTTTGTACAAGAAAGCTGGGTTTACCGACCCATCTCCGCC
4_known	B7	Tb927.1.740_for	GGGGACAAGTTTGTACAAAAAAGCAGGCTATGTTTGTGCGCCGGTTGG	Tb927.1.740_rev	GGGGACCACTTTGTACAAGAAAGCTGGGTTTATCCCTTTGAGGTAAAGGCCTTCC
4_known	B8	Tb927.11.350_for	GGGGACAAGTTTGTACAAAAAAGCAGGCTATGGAGGAGAAAGAAAGTGATTCTTCG	Tb927.11.350_rev	GGGGACCACTTTGTACAAGAAAGCTGGGTTTAAAACTGCCGACGAGGGTTC
4_known	B9	Tb927.11.3620_for	GGGGACAAGTTTGTACAAAAAAGCAGGCTATGGCTCTTGGATTTTCCTCTGC	Tb927.11.3620_rev	GGGGACCACTTTGTACAAGAAAGCTGGGTTCATGGTAGGAAGACAACCACAATCA
4_known	B11	Tb927.3.2060_for	GGGGACAAGTTTGTACAAAAAAGCAGGCTATGGAGAACATTCGTAGGACGAATCG	Tb927.3.2060_rev	GGGGACCACTTTGTACAAGAAAGCTGGGTCTACTCTGGGTCTTTACTCGACCG
4_known	B12	Tb927.6.1490_for	GGGGACAAGTTTGTACAAAAAAGCAGGCTATGTGTTCCAACCCCGGGG	Tb927.6.1490_rev	GGGGACCACTTTGTACAAGAAAGCTGGGTTTACATGGAGCAAATGTGGCAACG
4_known	B13	Tb927.10.540_for	GGGGACAAGTTTGTACAAAAAAGCAGGCTATGAGTAGTGGTCTCGCTGACTT	Tb927.10.540_rev	GGGGACCACTTTGTACAAGAAAGCTGGGTCTATTGGTTCATATACTGGCTCTGGTTC
4_known	B14	Tb927.11.14110_for	GGGGACAAGTTTGTACAAAAAAGCAGGCTATGGATGTGTGGGATGCCTCT	Tb927.11.14110_rev	GGGGACCACTTTGTACAAGAAAGCTGGGTTCAGATAGAGAATTCCCTGCGGAG
4_known	B15	Tb927.11.7216_for	GGGGACAAGTTTGTACAAAAAAGCAGGCTATGAAACGAAGGAGGGACAGTAACG	Tb927.11.7216_rev	GGGGACCACTTTGTACAAGAAAGCTGGGTCTATATCGAAAACAGTGGGCATATCCC
4_known	B16	Tb927.11.1670_for	GGGGACAAGTTTGTACAAAAAAGCAGGCTATGTTTAGTGGTGTTCGCGTACTT	Tb927.11.1670_rev	GGGGACCACTTTGTACAAGAAAGCTGGGTCTACCGCCACTCCACGTCT
4_known	B17	Tb927.8.2940_for	GGGGACAAGTTTGTACAAAAAAGCAGGCTATGAAAGGCGCTCAGTTTTTGAAGA	Tb927.8.2940_rev	GGGGACCACTTTGTACAAGAAAGCTGGGTTCAAAAAAAAAAACAGCTCAACGCAG
4_known	B18	Tb927.8.3690_for	GGGGACAAGTTTGTACAAAAAAGCAGGCTATGTTCCGCCGTGTAGCCT	Tb927.8.3690_rev	GGGGACCACTTTGTACAAGAAAGCTGGGTTCACCCCTTTACTGAACGCGC
4_known	B19	Tb927.10.4940_for	GGGGACAAGTTTGTACAAAAAAGCAGGCTATGTCAGATGTGCCTAAATCTCAAGTTG	Tb927.10.4940_rev	GGGGACCACTTTGTACAAGAAAGCTGGGTTTAATCATCATCTGAAAAATTCTGCAGCTC
4_known	B20	Tb927.11.9590_for	GGGGACAAGTTTGTACAAAAAAGCAGGCTATGACTGATTACAAGGTACGTGATATCAGC	Tb927.11.9590_rev	GGGGACCACTTTGTACAAGAAAGCTGGGTTTAGTAACGGTAGTGGTCAGGTTTGA
4_known	B21	Tb927.7.2500_for	GGGGACAAGTTTGTACAAAAAAGCAGGCTATGGCAAGCGAGAAGGGAGG	Tb927.7.2500_rev	GGGGACCACTTTGTACAAGAAAGCTGGGTTTAGTTGTACACCATGTATTTGGCTGT
4_known	B22	Tb927.11.3740_for	GGGGACAAGTTTGTACAAAAAAGCAGGCTATGGGGCAAGCAGGTGGG	Tb927.11.3740_rev	GGGGACCACTTTGTACAAGAAAGCTGGGTCTAAAGGTAAAACTTGGAGAAGCGACC
4_known	B23	Tb927.3.3990_for	GGGGACAAGTTTGTACAAAAAAGCAGGCTATGACTCCTGTATTTGGTACCGCT	Tb927.3.3990_rev	GGGGACCACTTTGTACAAGAAAGCTGGGTTCACCTCATCAAAGACAATCTCTTCG
4_known	B24	Tb927.8.2540_for	GGGGACAAGTTTGTACAAAAAAGCAGGCTATGCTCCGTCGTACGTCCTC	Tb927.8.2540_rev	GGGGACCACTTTGTACAAGAAAGCTGGGTTTAGATATTCGTTAGCACCATAGACACTGC
4_known	C1	Tb927.6.3110_for	GGGGACAAGTTTGTACAAAAAAGCAGGCTATGGATTCACCAACTGAGTTTTCTTGT	Tb927.6.3110_rev	GGGGACCACTTTGTACAAGAAAGCTGGGTTCAAAGTGTGGCCATGTTCATGT
4_known	C2	Tb927.5.4330_for	GGGGACAAGTTTGTACAAAAAAGCAGGCTATGAGATCGTTTATGTACATGTGCCG	Tb927.5.4330_rev	GGGGACCACTTTGTACAAGAAAGCTGGGTTCACGCCACCTCGCCCGC
4_known	C3	Tb927.11.3630_for	GGGGACAAGTTTGTACAAAAAAGCAGGCTATGGCTCTTGGATTTTCCTCTGC	Tb927.11.3630_rev	GGGGACCACTTTGTACAAGAAAGCTGGGTTCATGGTAGGAAGATAACCACAATCATCG
4_known	C4	Tb927.3.2320_for	GGGGACAAGTTTGTACAAAAAAGCAGGCTATGGAGGATTATTATTATTACGAAGACGAC	Tb927.3.2320_rev	GGGGACCACTTTGTACAAGAAAGCTGGGTTCAAAGGTTACGTTCTATGTCCTTGTATG
4_known	C5	Tb927.1.700_for	GGGGACAAGTTTGTACAAAAAAGCAGGCTATGACCCTTAACGAGAAGAAGAGCA	Tb927.1.700_rev	GGGGACCACTTTGTACAAGAAAGCTGGGTTTAAAGAGAGCTCCACCGGTTAGA
4_known	C6	Tb927.8.7110_for	GGGGACAAGTTTGTACAAAAAAGCAGGCTATGACCGCGCACAAACGT	Tb927.8.7110_rev	GGGGACCACTTTGTACAAGAAAGCTGGGTTCAATGCGATATGGCTTTCTGAAGG
4_known	C7	Tb927.4.5310_for	GGGGACAAGTTTGTACAAAAAAGCAGGCTATGACGGAACATAAGCGTCCCG	Tb927.4.5310_rev	GGGGACCACTTTGTACAAGAAAGCTGGGTTCAACGAGGCACGGCATTC
4_known	C8	Tb927.11.5040_for	GGGGACAAGTTTGTACAAAAAAGCAGGCTATGAAAGTGGACAGCCTTCTGTCT	Tb927.11.5040_rev	GGGGACCACTTTGTACAAGAAAGCTGGGTCTATCCAGGTGTGGGCTTATTATGATATTC
4_known	C9	Tb927.1.1920_for	GGGGACAAGTTTGTACAAAAAAGCAGGCTATGAAGGGATGTACGCTCCAGAA	Tb927.1.1920_rev	GGGGACCACTTTGTACAAGAAAGCTGGGTCTAACGCTTTTCACGCGATCCA
4_known	C10	Tb927.2.4710_for	GGGGACAAGTTTGTACAAAAAAGCAGGCTATGCAACAATATACCCTTCGGGTG	Tb927.2.4710_rev	GGGGACCACTTTGTACAAGAAAGCTGGGTTCAGTCCCTTACGCGGTCG
4_known	C11	Tb927.11.16560_for	GGGGACAAGTTTGTACAAAAAAGCAGGCTATGTTTCCCTTGAGGAGTGTGAGT	Tb927.11.16560_rev	GGGGACCACTTTGTACAAGAAAGCTGGGTTCAACCGCGACTATAAATTATCAGCG
4_known	C12	Tb927.8.4890_for	GGGGACAAGTTTGTACAAAAAAGCAGGCTATGTTGAAGATGCGATTATTGATAGTAGTT	Tb927.8.4890_rev	GGGGACCACTTTGTACAAGAAAGCTGGGTTCAACTCATAGCCGTGTAATTGAATTTTTC
4_known	C13	Tb927.10.5140_for	GGGGACAAGTTTGTACAAAAAAGCAGGCTATGTCATCAGAAATAGAGCCACATATCCT	Tb927.10.5140_rev	GGGGACCACTTTGTACAAGAAAGCTGGGTTCATTTGTGCAACACACGAGAGG
4_known	C14	Tb927.11.14480_for	GGGGACAAGTTTGTACAAAAAAGCAGGCTATGGCTAGTGTTTTACCATCCGC	Tb927.11.14480_rev	GGGGACCACTTTGTACAAGAAAGCTGGGTTCATGTCTTGCGCTGGTACAC
4_known	C15	Tb927.1.2330_for	GGGGACAAGTTTGTACAAAAAAGCAGGCTATGCGCGAAATCGTCTGCG	Tb927.1.2330_rev	GGGGACCACTTTGTACAAGAAAGCTGGGTCTAGTATTGCTCCTCCTCGTCGA
4_known	C19	Tb927.11.11770_for	GGGGACAAGTTTGTACAAAAAAGCAGGCTATGAATCCAGAAGCGGAGGAATTTG	Tb927.11.11770_rev	GGGGACCACTTTGTACAAGAAAGCTGGGTCTAAAGCGTGAAGTCGGGTGTT
4_known	C20	Tb927.7.1930_for	GGGGACAAGTTTGTACAAAAAAGCAGGCTATGGTTACCCTACGGATGGCC	Tb927.7.1930_rev	GGGGACCACTTTGTACAAGAAAGCTGGGTCTAACGCCGCACCTTCCC
4_known	C21	Tb927.10.14730_for	GGGGACAAGTTTGTACAAAAAAGCAGGCTATGCAGCAGGCGGACTATTATG	Tb927.10.14730_rev	GGGGACCACTTTGTACAAGAAAGCTGGGTTTATAGTTTCCACTCATCCAAGTTAATTGG
4_known	C22	Tb927.9.11250_for	GGGGACAAGTTTGTACAAAAAAGCAGGCTATGGTAAAGGTGAACGTTATCAGCC	Tb927.9.11250_rev	GGGGACCACTTTGTACAAGAAAGCTGGGTCTACTTCAGATACTGATAAACCTTGCGC
4_known	C23	Tb927.3.3280_for	GGGGACAAGTTTGTACAAAAAAGCAGGCTATGTCCGTTGATTTCACGTCCAA	Tb927.3.3280_rev	GGGGACCACTTTGTACAAGAAAGCTGGGTTTATCCCTCCTGCCCAAACTCTT
4_known	C24	Tb927.10.10170_for	GGGGACAAGTTTGTACAAAAAAGCAGGCTATGAAATGTGAGCTGGCATCCC	Tb927.10.10170_rev	GGGGACCACTTTGTACAAGAAAGCTGGGTTTACTCCTTCGTGCGCCACA
4_known	D1	Tb927.3.1070_for	GGGGACAAGTTTGTACAAAAAAGCAGGCTATGAGGAATGCCCCATTGCTCT	Tb927.3.1070_rev	GGGGACCACTTTGTACAAGAAAGCTGGGTTTACGTAACACAGTTGAAGTCGCTC
4_known	D2	Tb927.11.4780_for	GGGGACAAGTTTGTACAAAAAAGCAGGCTATGTTTCGGTGTTATGGACGAAAACT	Tb927.11.4780_rev	GGGGACCACTTTGTACAAGAAAGCTGGGTCTAAAACGAACCCTCTCTATCCCG
4_known	D3	Tb927.5.3620_for	GGGGACAAGTTTGTACAAAAAAGCAGGCTATGCAGTCGTCAAGTTTTGCTGG	Tb927.5.3620_rev	GGGGACCACTTTGTACAAGAAAGCTGGGTTCAGGATTCATTAGCCAACGACTCC
4_known	D4	Tb927.9.7190_for	GGGGACAAGTTTGTACAAAAAAGCAGGCTATGCGACGCTCTGCCACC	Tb927.9.7190_rev	GGGGACCACTTTGTACAAGAAAGCTGGGTTCAATGTGTGCTCTCTGCCGC
4_known	D5	Tb927.3.4680_for	GGGGACAAGTTTGTACAAAAAAGCAGGCTATGGAGGAAACGTACGATGCAATT	Tb927.3.4680_rev	GGGGACCACTTTGTACAAGAAAGCTGGGTCTACCTATCCTCGTTGGAAGCAGT
4_known	D6	Tb927.7.2550_for	GGGGACAAGTTTGTACAAAAAAGCAGGCTATGACGCAGGACACCGCA	Tb927.7.2550_rev	GGGGACCACTTTGTACAAGAAAGCTGGGTCTATGCGTAATAGTTCAAGCTAGATTTTTT
4_known	D7	Tb927.9.13430_for	GGGGACAAGTTTGTACAAAAAAGCAGGCTATGCGTGCGGAGATTCCCG	Tb927.9.13430_rev	GGGGACCACTTTGTACAAGAAAGCTGGGTTCAAAAAAAAGTTGGTTCAACCTGAGG
4_known	D8	Tb927.10.14240_for	GGGGACAAGTTTGTACAAAAAAGCAGGCTATGACTGACAAAGCATTTACGGAACA	Tb927.10.14240_rev	GGGGACCACTTTGTACAAGAAAGCTGGGTTTAAACCATCACAAGACCAACTTCATTAGC
4_known	D9	Tb927.10.4770_for	GGGGACAAGTTTGTACAAAAAAGCAGGCTATGGGTTCATGCCAGGCGG	Tb927.10.4770_rev	GGGGACCACTTTGTACAAGAAAGCTGGGTCTATTTCTCTGGTGGAGGTATTTTGCC
4_known	D10	Tb927.3.910_for	GGGGACAAGTTTGTACAAAAAAGCAGGCTATGCCACGGGAAATCATCACAC	Tb927.3.910_rev	GGGGACCACTTTGTACAAGAAAGCTGGGTTCAAAAGTTTCGTATATAATCGGAGCTCTC
4_known	D11	Tb927.6.3130_for	GGGGACAAGTTTGTACAAAAAAGCAGGCTATGTCCAAGGAGGAGGCGG	Tb927.6.3130_rev	GGGGACCACTTTGTACAAGAAAGCTGGGTTCAGCGGGGGAGCTCAAC
4_known	D12	Tb927.9.12700_for	GGGGACAAGTTTGTACAAAAAAGCAGGCTATGGTAACGTGGGCGCTGA	Tb927.9.12700_rev	GGGGACCACTTTGTACAAGAAAGCTGGGTCTAACACGTTGAACACACTTCGGT
4_known	D13	Tb927.6.2830_for	GGGGACAAGTTTGTACAAAAAAGCAGGCTATGCTCCAACGCGGTTTGG	Tb927.6.2830_rev	GGGGACCACTTTGTACAAGAAAGCTGGGTCTACTGCGCGAGATGTGCAA
4_known	D14	Tb927.9.9970_for	GGGGACAAGTTTGTACAAAAAAGCAGGCTATGCCTCCTAAACAATTGGTGGAC	Tb927.9.9970_rev	GGGGACCACTTTGTACAAGAAAGCTGGGTTTAAGCAGTGTAGTACTTTCCTTGAGGG
4_known	D15	Tb927.7.1430_for	GGGGACAAGTTTGTACAAAAAAGCAGGCTATGCTCGCGGACCGCATA	Tb927.7.1430_rev	GGGGACCACTTTGTACAAGAAAGCTGGGTTCATGCTCGAGAGGATGATGTGC
4_known	D16	Tb927.10.2110_for	GGGGACAAGTTTGTACAAAAAAGCAGGCTATGGGAAAGGAAAAGGTGCACATG	Tb927.10.2110_rev	GGGGACCACTTTGTACAAGAAAGCTGGGTTTATTTCTTCGAAGCCTTCACCGC
4_known	D21	Tb927.11.4220_for	GGGGACAAGTTTGTACAAAAAAGCAGGCTATGAGCGTCGGCCTCTACTG	Tb927.11.4220_rev	GGGGACCACTTTGTACAAGAAAGCTGGGTTCAAATAACACCCACACTACCATCCT
4_known	E2	Tb927.6.960_for	GGGGACAAGTTTGTACAAAAAAGCAGGCTATGCCTCGAACAGAAATGGTGC	Tb927.6.960_rev	GGGGACCACTTTGTACAAGAAAGCTGGGTTCACTGGTGTGGGACCAGC
4_known	E5	Tb927.4.4850_for	GGGGACAAGTTTGTACAAAAAAGCAGGCTATGGAAGTCAGGAACGAACCAATC	Tb927.4.4850_rev	GGGGACCACTTTGTACAAGAAAGCTGGGTTTATATGTTAATAGCTCCCCAGATGGAAGC
4_known	E9	Tb927.9.9150_for	GGGGACAAGTTTGTACAAAAAAGCAGGCTATGCGCCGTGGACTCACT	Tb927.9.9150_rev	GGGGACCACTTTGTACAAGAAAGCTGGGTTCACCGCCCTGGAATAATGCG
4_known	E10	Tb927.9.12730_for	GGGGACAAGTTTGTACAAAAAAGCAGGCTATGTTGCGGTTCACGTCCG	Tb927.9.12730_rev	GGGGACCACTTTGTACAAGAAAGCTGGGTCTACTGATGAACGCACTCGGC
4_known	E11	Tb927.10.7570_for	GGGGACAAGTTTGTACAAAAAAGCAGGCTATGTTCCGCCGTCCCGTC	Tb927.10.7570_rev	GGGGACCACTTTGTACAAGAAAGCTGGGTCTACAGCAATAGCGAGAGTGGGT
4_known	E12	Tb927.9.12910_for	GGGGACAAGTTTGTACAAAAAAGCAGGCTATGTGTAGCATTGAGGGCCCG	Tb927.9.12910_rev	GGGGACCACTTTGTACAAGAAAGCTGGGTCTAAAACTCACCGAACTGTTGCAC
4_known	E13	Tb927.1.2400_for	GGGGACAAGTTTGTACAAAAAAGCAGGCTATGCGTGAGGCTATCTGCATCC	Tb927.1.2400_rev	GGGGACCACTTTGTACAAGAAAGCTGGGTCTAGTACTCCTCCACATCCTCCTCA
4_known	E17	Tb927.4.4870_for	GGGGACAAGTTTGTACAAAAAAGCAGGCTATGGAAGTCAGGAACGAACCAATC	Tb927.4.4870_rev	GGGGACCACTTTGTACAAGAAAGCTGGGTTCAATTTCCCACCACTACGCCA
4_known	E18	Tb927.3.3410_for	GGGGACAAGTTTGTACAAAAAAGCAGGCTATGTTGCCACTTGACTCACCG	Tb927.3.3410_rev	GGGGACCACTTTGTACAAGAAAGCTGGGTTTAGGAGGTCACCATGTCCCTG
4_known	E19	Tb927.11.2300_for	GGGGACAAGTTTGTACAAAAAAGCAGGCTATGGCCGACCACGAGTTGT	Tb927.11.2300_rev	GGGGACCACTTTGTACAAGAAAGCTGGGTTTACATAAAGTCGTCGTCGAAGTCAAA
4_known	E20	Tb927.10.10800_for	GGGGACAAGTTTGTACAAAAAAGCAGGCTATGTGTTGTGGTCGGCGTATC	Tb927.10.10800_rev	GGGGACCACTTTGTACAAGAAAGCTGGGTTCAAACAGGAGTAGGACTTGTCAACG
4_known	E21	Tb927.11.12650_for	GGGGACAAGTTTGTACAAAAAAGCAGGCTATGGAATCGTTCGGCCCTCT	Tb927.11.12650_rev	GGGGACCACTTTGTACAAGAAAGCTGGGTTCAACTGTAGGGAAACATCACCTCC

4_known	E22	Tb927.2.2120_for	GGGGACAAGTTTGTACAAAAAAGCAGGCTATGGCTGATCTCCTTGAGCTTCG	Tb927.2.2120_rev	GGGGACCACTTTGTACAAGAAAGCTGGGTTTAATTACTGCTGCCGCTGCTC
4_known	E23	Tb927.11.4540_for	GGGGACAAGTTTGTACAAAAAAGCAGGCTATGTACCAGCCACTCCTTGAGG	Tb927.11.4540_rev	GGGGACCACTTTGTACAAGAAAGCTGGGTTTATGCATGATACGCAGGAACAAATGA
4_known	E24	Tb927.10.6500_for	GGGGACAAGTTTGTACAAAAAAGCAGGCTATGGTTGTGAATTCTGATGGGGTG	Tb927.10.6500_rev	GGGGACCACTTTGTACAAGAAAGCTGGGTCTAATTGATAATCGCATAAATCGTCGCC
4_known	F1	Tb927.4.3930_for	GGGGACAAGTTTGTACAAAAAAGCAGGCTATGACGGAAGGACTAGGCGAA	Tb927.4.3930_rev	GGGGACCACTTTGTACAAGAAAGCTGGGTTCACACAATCACGTCATAAACCGC
4_known	F2	Tb927.10.6350_for	GGGGACAAGTTTGTACAAAAAAGCAGGCTATGCCACCACGGACCGCT	Tb927.10.6350_rev	GGGGACCACTTTGTACAAGAAAGCTGGGTCTACTCAAAGTTGCTCCGTGGC
4_known	F3	Tb927.6.540_for	GGGGACAAGTTTGTACAAAAAAGCAGGCTATGATGCACGAATTAGTGTTTATCATTGG	Tb927.6.540_rev	GGGGACCACTTTGTACAAGAAAGCTGGGTCTAAGATATACAAATAAAAGATGAAAACAA
4_known	F4	Tb927.8.7050_for	GGGGACAAGTTTGTACAAAAAAGCAGGCTATGCCCATTTCGCAAGTGTTCA	Tb927.8.7050_rev	GGGGACCACTTTGTACAAGAAAGCTGGGTTCAATGTGTTCGGAATACGTAGGAATC
4_known	F5	Tb927.11.11440_for	GGGGACAAGTTTGTACAAAAAAGCAGGCTATGCCAGCGATGAAAAATAATTGTGC	Tb927.11.11440_rev	GGGGACCACTTTGTACAAGAAAGCTGGGTCTATCCGGCCAATCTAGGTCGT
4_known	F6	Tb927.2.1780_for	GGGGACAAGTTTGTACAAAAAAGCAGGCTATGCGTCGTCACCGCGTG	Tb927.2.1780_rev	GGGGACCACTTTGTACAAGAAAGCTGGGTTCAGGATGGGCTTCCCACAA
4_known	F7	Tb927.10.880_for	GGGGACAAGTTTGTACAAAAAAGCAGGCTATGCACGACGGAGATGGCA	Tb927.10.880_rev	GGGGACCACTTTGTACAAGAAAGCTGGGTCTAAGTAGTATCAACGGCCATTTGATTTCT
4_known	F8	Tb927.11.3140_for	GGGGACAAGTTTGTACAAAAAAGCAGGCTATGGATGTCTCTCGCAGGTTGG	Tb927.11.3140_rev	GGGGACCACTTTGTACAAGAAAGCTGGGTCTAGCACCCGCATCTGTCGG
4_known	F9	Tb927.11.9160_for	GGGGACAAGTTTGTACAAAAAAGCAGGCTATGGAGGATTTCGTTGTTGTGCC	Tb927.11.9160_rev	GGGGACCACTTTGTACAAGAAAGCTGGGTCTATGATGACACCATCATTCTCCTCG
4_known	F10	Tb927.4.4000_for	GGGGACAAGTTTGTACAAAAAAGCAGGCTATGAGCAACTCCAAGCAACAACA	Tb927.4.4000_rev	GGGGACCACTTTGTACAAGAAAGCTGGGTTCATCCCCTGACGGTATCCAAAA
4_known	F11	Tb927.9.1400_for	GGGGACAAGTTTGTACAAAAAAGCAGGCTATGCGTGTTGTCATCGTTGGT	Tb927.9.1400_rev	GGGGACCACTTTGTACAAGAAAGCTGGGTTTAGTTCCTGTACGCCGTTTCCA
4_known	F14	Tb927.8.7120_for	GGGGACAAGTTTGTACAAAAAAGCAGGCTATGGATTACGTTAGCGAATTGTACGC	Tb927.8.7120_rev	GGGGACCACTTTGTACAAGAAAGCTGGGTTCACCTGCCCAAATAACCGAAGA
4_known	F15	Tb927.6.2000_for	GGGGACAAGTTTGTACAAAAAAGCAGGCTATGAAGGAAGCAAAAAAGAAAAAGTTACTG	Tb927.6.2000_rev	GGGGACCACTTTGTACAAGAAAGCTGGGTTCAAAATTTTGTTGGTGCACGTGG
4_known	F16	Tb927.11.8460_for	GGGGACAAGTTTGTACAAAAAAGCAGGCTATGGAATCAAAACACCCCGAAGTG	Tb927.11.8460_rev	GGGGACCACTTTGTACAAGAAAGCTGGGTTCAACCCTTTGGAAATACCTTCATGAC
4_known	F17	Tb927.6.500_for	GGGGACAAGTTTGTACAAAAAAGCAGGCTATGAGGCACGAATTAGTGTTTACCAT	Tb927.6.500_rev	GGGGACCACTTTGTACAAGAAAGCTGGGTCTAAGATATACAAATGAAAGATGAAAACAA
4_known	F18	Tb927.1.1030_for	GGGGACAAGTTTGTACAAAAAAGCAGGCTATGAGTCGTTTTGCCGCAAGC	Tb927.1.1030_rev	GGGGACCACTTTGTACAAGAAAGCTGGGTTTACCAACCTGAGGCCTCAGTC
4_known	F19	Tb927.6.470_for	GGGGACAAGTTTGTACAAAAAAGCAGGCTATGAGGCACGAATTAGTGTTTACCAT	Tb927.6.470_rev	GGGGACCACTTTGTACAAGAAAGCTGGGTCTAAGATATACAAATAAAAGATGAAAACAA
4_known	F20	Tb927.11.16590_for	GGGGACAAGTTTGTACAAAAAAGCAGGCTATGAGCACATCAGCGGGGA	Tb927.11.16590_rev	GGGGACCACTTTGTACAAGAAAGCTGGGTCTACACCCACCTCCTCCCATTT
4_known	F21	Tb927.7.540_for	GGGGACAAGTTTGTACAAAAAAGCAGGCTATGTCGTCCGCAGATCCCA	Tb927.7.540_rev	GGGGACCACTTTGTACAAGAAAGCTGGGTTTACAGAAAACCAACATACCGCAATGG
4_known	F22	Tb927.8.4700_for	GGGGACAAGTTTGTACAAAAAAGCAGGCTATGCTGAGCCCTACAGAACCA	Tb927.8.4700_rev	GGGGACCACTTTGTACAAGAAAGCTGGGTTTACACAGCCACATTGTAGATTGAGG
4_known	F24	Tb927.3.4900_for	GGGGACAAGTTTGTACAAAAAAGCAGGCTATGAGGTCTATTTACTTTGTCATAGGCG	Tb927.3.4900_rev	GGGGACCACTTTGTACAAGAAAGCTGGGTTTACGACCGCTTAACTCTCTTAGAAGG
4_known	G1	Tb927.8.4730_for	GGGGACAAGTTTGTACAAAAAAGCAGGCTATGCTGAGCCCTACAGAAGCA	Tb927.8.4730_rev	GGGGACCACTTTGTACAAGAAAGCTGGGTTTACACAGCCACATTGTAGATTGAGG
4_known	G3	Tb927.11.15530_for	GGGGACAAGTTTGTACAAAAAAGCAGGCTATGCCGCCACGGAGCCGC	Tb927.11.15530_rev	GGGGACCACTTTGTACAAGAAAGCTGGGTCTAATAGACGTATGGGATCAAGCGATAAC
4_known	G4	Tb927.5.3810_for	GGGGACAAGTTTGTACAAAAAAGCAGGCTATGTCCTCTACTTTCTTTGATATGCTTAAC	Tb927.5.3810_rev	GGGGACCACTTTGTACAAGAAAGCTGGGTTTACAGCTTGCTAGTGGATTCCAAC
4_known	G5	Tb927.6.2250_for	GGGGACAAGTTTGTACAAAAAAGCAGGCTATGACCATCGATTACTCAGGTTTTCTTG	Tb927.6.2250_rev	GGGGACCACTTTGTACAAGAAAGCTGGGTCTATGTAGGTCTTGATCTCCCATTAAACG
4_known	G7	Tb927.10.5960_for	GGGGACAAGTTTGTACAAAAAAGCAGGCTATGGGAACTTCGTCCACCGA	Tb927.10.5960_rev	GGGGACCACTTTGTACAAGAAAGCTGGGTTCACTCTTCATTATTATCATCATGATCCCC
4_known	G8	Tb927.4.1270_for	GGGGACAAGTTTGTACAAAAAAGCAGGCTATGTCTGGAATCAAAATTGAGGAGGTT	Tb927.4.1270_rev	GGGGACCACTTTGTACAAGAAAGCTGGGTCTATTGGTAGACGTAATCCTCTGCGT
4_known	G9	Tb927.4.1400_for	GGGGACAAGTTTGTACAAAAAAGCAGGCTATGGAAGATTCCTTCAACCTGAAGAAAG	Tb927.4.1400_rev	GGGGACCACTTTGTACAAGAAAGCTGGGTTCATTTTCCTCCTGTGCGCC
4_known	G10	Tb927.8.8220_for	GGGGACAAGTTTGTACAAAAAAGCAGGCTATGCGAGCCACCAAAAAGCA	Tb927.8.8220_rev	GGGGACCACTTTGTACAAGAAAGCTGGGTCTACGGCACCGTGTTGTATATTGT
4_known	G11	Tb927.9.8410_for	GGGGACAAGTTTGTACAAAAAAGCAGGCTATGAGGCAACGGAGTCCTTTGT	Tb927.9.8410_rev	GGGGACCACTTTGTACAAGAAAGCTGGGTCTAGGCGGCAAACCAAGATTTGT
4_known	G13	Tb927.8.8260_for	GGGGACAAGTTTGTACAAAAAAGCAGGCTATGACGAATCAATCCAGCGGGG	Tb927.8.8260_rev	GGGGACCACTTTGTACAAGAAAGCTGGGTCTACACAGCATAATATACTGCCGATCC
4_known	G14	Tb927.10.210_for	GGGGACAAGTTTGTACAAAAAAGCAGGCTATGTTGCAGGCGGGAACAAA	Tb927.10.210_rev	GGGGACCACTTTGTACAAGAAAGCTGGGTCTAAGCACCCAACGTCGACC
4_known	G15	Tb927.8.8250_for	GGGGACAAGTTTGTACAAAAAAGCAGGCTATGACGAATCAATCCAGCGGGG	Tb927.8.8250_rev	GGGGACCACTTTGTACAAGAAAGCTGGGTCTACGGCACCGTGTTGTATATTGT
4_known	G16	Tb927.8.8230_for	GGGGACAAGTTTGTACAAAAAAGCAGGCTATGACGAATCAATCCAGCGGGG	Tb927.8.8230_rev	GGGGACCACTTTGTACAAGAAAGCTGGGTTCAAGACAGTGCACCGGCT
4_known	G17	Tb927.9.15050_for	GGGGACAAGTTTGTACAAAAAAGCAGGCTATGCCACCAAAGAAGGAAGCAG	Tb927.9.15050_rev	GGGGACCACTTTGTACAAGAAAGCTGGGTCTACAGGGTGCTGCTTGATCCC
4_known	G18	Tb927.10.2750_for	GGGGACAAGTTTGTACAAAAAAGCAGGCTATGGCTGAGTTGGCCAAGAGT	Tb927.10.2750_rev	GGGGACCACTTTGTACAAGAAAGCTGGGTTCACGAGCGGATATTCTCCTTTCC
4_known	G19	Tb927.7.2670_for	GGGGACAAGTTTGTACAAAAAAGCAGGCTATGAGCCATCCACAATCTCATCGC	Tb927.7.2670_rev	GGGGACCACTTTGTACAAGAAAGCTGGGTTCAATCGCTGATTCCTCCCGT
4_known	G20	Tb927.2.6150_for	GGGGACAAGTTTGTACAAAAAAGCAGGCTATGGCAATGCTTGGTTTCGAGT	Tb927.2.6150_rev	GGGGACCACTTTGTACAAGAAAGCTGGGTTTATTTAGGAAGTCCCTCCTTAACAGCC
4_known	G21	Tb927.3.590_for	GGGGACAAGTTTGTACAAAAAAGCAGGCTATGATGCTCGGGTTCGAATCG	Tb927.3.590_rev	GGGGACCACTTTGTACAAGAAAGCTGGGTCTATTGAGGAAGTCCCTCCTTGACG
4_known	G22	Tb927.2.6220_for	GGGGACAAGTTTGTACAAAAAAGCAGGCTATGGCAATGCTTGGTTTCGAGT	Tb927.2.6220_rev	GGGGACCACTTTGTACAAGAAAGCTGGGTCTACTCTGTTTTAGGAAGAGCCTCCT
4_known	G23	Tb927.9.7470_for	GGGGACAAGTTTGTACAAAAAAGCAGGCTATGCTCGGGTTTGAGTCGTTTT	Tb927.9.7470_rev	GGGGACCACTTTGTACAAGAAAGCTGGGTTTATGATTCTGGAAGAGCCGTCATAAC
4_known	G24	Tb927.6.3230_for	GGGGACAAGTTTGTACAAAAAAGCAGGCTATGGTACGCAGCATTCGCTAC	Tb927.6.3230_rev	GGGGACCACTTTGTACAAGAAAGCTGGGTTCACTGATAACAGAAGGTAGTGATAACCT
4_known	H1	Tb927.11.15860_for	GGGGACAAGTTTGTACAAAAAAGCAGGCTATGTCACCCAGGGCAACAGA	Tb927.11.15860_rev	GGGGACCACTTTGTACAAGAAAGCTGGGTCTACTTCTTAACCTCGTCGTAAATGGTT
4_known	H4	Tb927.10.14010_for	GGGGACAAGTTTGTACAAAAAAGCAGGCTATGACCTACCAAGCGAAGGAAGT	Tb927.10.14010_rev	GGGGACCACTTTGTACAAGAAAGCTGGGTCTAAAAGTCGTCACTGTCATCGTCG
4_known	H5	Tb927.9.2010_for	GGGGACAAGTTTGTACAAAAAAGCAGGCTATGCGCAATAACGCAGCGG	Tb927.9.2010_rev	GGGGACCACTTTGTACAAGAAAGCTGGGTTTAAAAACACTCGGCTATGGCACG
4_known	H6	Tb927.11.9130_for	GGGGACAAGTTTGTACAAAAAAGCAGGCTATGCAGCAGCCATCACAACA	Tb927.11.9130_rev	GGGGACCACTTTGTACAAGAAAGCTGGGTTTACAAGTAGGCATTAATGCATTTAATCAT
4_known	H7	Tb927.2.6240_for	GGGGACAAGTTTGTACAAAAAAGCAGGCTATGGCAATGCTTGGTTTCGAGT	Tb927.2.6240_rev	GGGGACCACTTTGTACAAGAAAGCTGGGTTTATTCAGGAAGTCCCTCCTTAACAGC
4_known	H8	Tb927.6.220_for	GGGGACAAGTTTGTACAAAAAAGCAGGCTATGCTCGGTTTTTATTCAGTACCTGAA	Tb927.6.220_rev	GGGGACCACTTTGTACAAGAAAGCTGGGTTTAATGCGGAAGAGCTTTTATAACCGC
4_known	H9	Tb927.10.1420_for	GGGGACAAGTTTGTACAAAAAAGCAGGCTATGCCACCTAAAGGCGGGG	Tb927.10.1420_rev	GGGGACCACTTTGTACAAGAAAGCTGGGTTTAGTAGTCGTCGCCCTTAGAAAGG
4_known	H10	Tb927.9.14070_for	GGGGACAAGTTTGTACAAAAAAGCAGGCTATGGGTGTTGGGTTGGGTTTC	Tb927.9.14070_rev	GGGGACCACTTTGTACAAGAAAGCTGGGTCTACGCGGAAAGGTCTTCCTTTG
4_known	H11	Tb927.5.286b_for	GGGGACAAGTTTGTACAAAAAAGCAGGCTATGCTCGGGTTTGACTCAGCT	Tb927.5.286b_rev	GGGGACCACTTTGTACAAGAAAGCTGGGTCTACTTGGGAAGCCCCTCATTGA
4_known	H12	Tb927.10.1040_for	GGGGACAAGTTTGTACAAAAAAGCAGGCTATGATGCTGTGCCACACGTC	Tb927.10.1040_rev	GGGGACCACTTTGTACAAGAAAGCTGGGTTTACGAGAGTGGTTCGTTCCTCA
4_known	H13	Tb927.2.6200_for	GGGGACAAGTTTGTACAAAAAAGCAGGCTATGGCAATGCTTGGTTTCGAGT	Tb927.2.6200_rev	GGGGACCACTTTGTACAAGAAAGCTGGGTTTATTCAGGAAGTCCCTCTTTAACAGCC
4_known	H14	Tb927.7.5770_for	GGGGACAAGTTTGTACAAAAAAGCAGGCTATGTCCACGCGGAAAGAGC	Tb927.7.5770_rev	GGGGACCACTTTGTACAAGAAAGCTGGGTTTACCGTTTGAGGTTGAATTTGTAGTCT
4_known	H15	Tb927.8.5390_for	GGGGACAAGTTTGTACAAAAAAGCAGGCTATGTCAACTGTCAACAGACTGCAA	Tb927.8.5390_rev	GGGGACCACTTTGTACAAGAAAGCTGGGTTTACGGATATACACTGTGTTCTCGAAGT
4_known	H17	Tb927.9.1500_for	GGGGACAAGTTTGTACAAAAAAGCAGGCTATGGATGGCGTGTGCGAAC	Tb927.9.1500_rev	GGGGACCACTTTGTACAAGAAAGCTGGGTTCACTTGACAATCTTCTGCGATACC
4_known	H18	Tb927.11.1390_for	GGGGACAAGTTTGTACAAAAAAGCAGGCTATGTCAAACGAAGTTGAATCTTCTGCA	Tb927.11.1390_rev	GGGGACCACTTTGTACAAGAAAGCTGGGTTTATCTTAGATCCTTATCTGTATTGATAAC
4_known	H19	Tb927.11.12410_for	GGGGACAAGTTTGTACAAAAAAGCAGGCTATGGATAGCATAAGCTCTGGTCTAACC	Tb927.11.12410_rev	GGGGACCACTTTGTACAAGAAAGCTGGGTTTACATGACAGGTGTTGGGGGG
4_known	H20	Tb927.5.2100_for	GGGGACAAGTTTGTACAAAAAAGCAGGCTATGCGTTTTGTTTGTCTTTTCTCACG	Tb927.5.2100_rev	GGGGACCACTTTGTACAAGAAAGCTGGGTCATTTCTTTTCTGATTCCCTCTCCAAC
4_known	H21	Tb927.4.3790_for	GGGGACAAGTTTGTACAAAAAAGCAGGCTATGGTTCCTGTTAAAGTGAAATGGGG	Tb927.4.3790_rev	GGGGACCACTTTGTACAAGAAAGCTGGGTCTACAGCGGAATGACCGACTG
4_known	H22	Tb927.7.4560_for	GGGGACAAGTTTGTACAAAAAAGCAGGCTATGGTAATGTTTGAGGTGCGGC	Tb927.7.4560_rev	GGGGACCACTTTGTACAAGAAAGCTGGGTTTATACTTTCTCAGCTCTACGCAAGTCA
4_known	H23	Tb927.2.6280_for	GGGGACAAGTTTGTACAAAAAAGCAGGCTATGGCAATGCTTGGTTTCGAGT	Tb927.2.6280_rev	GGGGACCACTTTGTACAAGAAAGCTGGGTCTACTTTTTTTCCTTCGGAAGTCCCT
4_known	H24	Tb927.10.4600_for	GGGGACAAGTTTGTACAAAAAAGCAGGCTATGCAAACAGAAGCCCTGCAA	Tb927.10.4600_rev	GGGGACCACTTTGTACAAGAAAGCTGGGTTCAAAGATCATTTAGGTGTGGCGC
4_known	I2	Tb927.8.7650_for	GGGGACAAGTTTGTACAAAAAAGCAGGCTATGACCAGCATCAAGACCAAGTCG	Tb927.8.7650_rev	GGGGACCACTTTGTACAAGAAAGCTGGGTCTAACCAGTGAAAGTTCCCCAGATAGC
4_known	I3	Tb927.10.6830_for	GGGGACAAGTTTGTACAAAAAAGCAGGCTATGGAAAAGAAAGGATCGGGAAGGG	Tb927.10.6830_rev	GGGGACCACTTTGTACAAGAAAGCTGGGTTCATTTGCGTAGCTTTGCCGT
4_known	I4	Tb927.3.3360_for	GGGGACAAGTTTGTACAAAAAAGCAGGCTATGGACGCCGGTAAACTTTTTCA	Tb927.3.3360_rev	GGGGACCACTTTGTACAAGAAAGCTGGGTTTATCCCTTGGTGAACCTCTTAGGTG
4_known	I5	Tb927.10.1050_for	GGGGACAAGTTTGTACAAAAAAGCAGGCTATGCGGCTCATTTCGTATCCG	Tb927.10.1050_rev	GGGGACCACTTTGTACAAGAAAGCTGGGTTTACGAGAGTGGTTCGTTCCTCA
4_known	I6	Tb927.8.7640_for	GGGGACAAGTTTGTACAAAAAAGCAGGCTATGACCAGCATCAAGACCAAGTCG	Tb927.8.7640_rev	GGGGACCACTTTGTACAAGAAAGCTGGGTTCATCCCACAGTAACTGCCCA
4_known	I7	Tb927.8.7620_for	GGGGACAAGTTTGTACAAAAAAGCAGGCTATGACCAGCATCAATGCCCAAC	Tb927.8.7620_rev	GGGGACCACTTTGTACAAGAAAGCTGGGTTCATCCCACAGTAACTGCCCA
4_known	I8	Tb927.8.7600_for	GGGGACAAGTTTGTACAAAAAAGCAGGCTATGACCAGCATCAATGCCCAAC	Tb927.8.7600_rev	GGGGACCACTTTGTACAAGAAAGCTGGGTTTAACCAGTCGCAGTTCCCCA
4_known	I9	Tb927.4.4960_for	GGGGACAAGTTTGTACAAAAAAGCAGGCTATGGTGGAGAAACCCCGTGA	Tb927.4.4960_rev	GGGGACCACTTTGTACAAGAAAGCTGGGTTTAAACTGTTCCACAAGTTGAGTGACA
4_known	I10	Tb927.9.4620_for	GGGGACAAGTTTGTACAAAAAAGCAGGCTATGTCATGCAATCCGCTGCG	Tb927.9.4620_rev	GGGGACCACTTTGTACAAGAAAGCTGGGTTCACGAGATGTGTATATCAGCCCC
4_known	I11	Tb927.10.3690_for	GGGGACAAGTTTGTACAAAAAAGCAGGCTATGTCCCGTCGATTCCTCAACC	Tb927.10.3690_rev	GGGGACCACTTTGTACAAGAAAGCTGGGTTTAGGTCCCTGAGCCATGCG
4_known	I13	Tb927.8.7610_for	GGGGACAAGTTTGTACAAAAAAGCAGGCTATGATCAGCATCAATGCCCAACC	Tb927.8.7610_rev	GGGGACCACTTTGTACAAGAAAGCTGGGTTCATCCCACAGTAACTGCCCA
4_known	I14	Tb927.11.4970_for	GGGGACAAGTTTGTACAAAAAAGCAGGCTATGCGAAAAGCCTTGGTGGAA	Tb927.11.4970_rev	GGGGACCACTTTGTACAAGAAAGCTGGGTTTAAGAAGTATAGAAAATATCCCTTGGTCT
4_known	I15	Tb927.2.3580_for	GGGGACAAGTTTGTACAAAAAAGCAGGCTATGCTTCAAGAAAGAGTATTTCATATTAAC	Tb927.2.3580_rev	GGGGACCACTTTGTACAAGAAAGCTGGGTCTAATTCTCTGACCAATTATGTTTGCACTT
4_known	I16	Tb927.8.7740_for	GGGGACAAGTTTGTACAAAAAAGCAGGCTATGACATCCGGAGCTGCTGC	Tb927.8.7740_rev	GGGGACCACTTTGTACAAGAAAGCTGGGTTTAATAGTACTTCTTAATTGTGTAATAAAT
4_known	I17	Tb927.10.730_for	GGGGACAAGTTTGTACAAAAAAGCAGGCTATGTCGTCCACGCCCGAG	Tb927.10.730_rev	GGGGACCACTTTGTACAAGAAAGCTGGGTTTAAATGCACTCCCACCGTTGAA
4_known	I18	Tb927.11.11270_for	GGGGACAAGTTTGTACAAAAAAGCAGGCTATGATGTCCACTGAATCCCTTGAGT	Tb927.11.11270_rev	GGGGACCACTTTGTACAAGAAAGCTGGGTTCACCGAAAACCCTCCAGCG
4_known	I19	Tb927.10.70_for	GGGGACAAGTTTGTACAAAAAAGCAGGCTATGGCTGATGGCCGCGCC	Tb927.10.70_rev	GGGGACCACTTTGTACAAGAAAGCTGGGTTTAACGCACTTCCCTTTTCCAAAATC
4_known	I20	Tb927.4.1680_for	GGGGACAAGTTTGTACAAAAAAGCAGGCTATGCCTCCCAAGCGAGCG	Tb927.4.1680_rev	GGGGACCACTTTGTACAAGAAAGCTGGGTTCAGTAAACTGGTTGCCAGTTCG
4_known	I21	Tb927.11.9070_for	GGGGACAAGTTTGTACAAAAAAGCAGGCTATGACGGGGTTCCGTCTCC	Tb927.11.9070_rev	GGGGACCACTTTGTACAAGAAAGCTGGGTTTACAGTATGAAATCATCTGCCTTCTCC
4_known	I22	Tb927.7.1590_for	GGGGACAAGTTTGTACAAAAAAGCAGGCTATGAGTTACAATACCAGTAGCAGCAGC	Tb927.7.1590_rev	GGGGACCACTTTGTACAAGAAAGCTGGGTTCAAAGACCGTACAGAAGGTCTGG
4_known	I23	Tb927.2.4110_for	GGGGACAAGTTTGTACAAAAAAGCAGGCTATGCTGCGTCATTGTATTCCACG	Tb927.2.4110_rev	GGGGACCACTTTGTACAAGAAAGCTGGGTTCAGTTTAGTTTCTCATAAGTTTTGCGAAC
4_known	I24	Tb927.9.4360_for	GGGGACAAGTTTGTACAAAAAAGCAGGCTATGCAACTCCAAAGGTTGGGTG	Tb927.9.4360_rev	GGGGACCACTTTGTACAAGAAAGCTGGGTTCATTCGCCCTTTGTGGGGG
4_known	J1	Tb927.9.9200_for	GGGGACAAGTTTGTACAAAAAAGCAGGCTATGTTACTCACCGCACCGTTATTAG	Tb927.9.9200_rev	GGGGACCACTTTGTACAAGAAAGCTGGGTCTATGACGGAAGATGCCGGC
4_known	J2	Tb927.1.1750_for	GGGGACAAGTTTGTACAAAAAAGCAGGCTATGGAGCTCAATCTTACACAACTTCC	Tb927.1.1750_rev	GGGGACCACTTTGTACAAGAAAGCTGGGTTTAATACACCGAGTCTGCAGATGAGG
4_known	J3	Tb927.10.13360_for	GGGGACAAGTTTGTACAAAAAAGCAGGCTATGTTTCGCGCTGCCTTTCG	Tb927.10.13360_rev	GGGGACCACTTTGTACAAGAAAGCTGGGTTTACTTCTTTTTCTTGCTCTTCCCCG
4_known	J4	Tb927.8.8300_for	GGGGACAAGTTTGTACAAAAAAGCAGGCTATGGACAGATCTGGCAGTCAATCC	Tb927.8.8300_rev	GGGGACCACTTTGTACAAGAAAGCTGGGTTCAACCCACAGCAGCGCC
4_known	J5	Tb927.5.2720_for	GGGGACAAGTTTGTACAAAAAAGCAGGCTATGGCTGAAACACCAAAGGAGATTC	Tb927.5.2720_rev	GGGGACCACTTTGTACAAGAAAGCTGGGTCTAGTTGACATTCATCTGATGGAAGCC
4_known	J6	Tb927.4.4020_for	GGGGACAAGTTTGTACAAAAAAGCAGGCTATGTGTATTGCCAGAGAAAATACAAGCA	Tb927.4.4020_rev	GGGGACCACTTTGTACAAGAAAGCTGGGTTTAACCAGTTATAGTGCCCCATATACTTGC
4_known	J7	Tb927.9.5170_for	GGGGACAAGTTTGTACAAAAAAGCAGGCTATGGTCGTGGCATCGTGTCC	Tb927.9.5170_rev	GGGGACCACTTTGTACAAGAAAGCTGGGTTTAATCAAGAACAAGCCCATTTGCATC
4_known	J8	Tb927.10.2010_for	GGGGACAAGTTTGTACAAAAAAGCAGGCTATGTCTAGACGCCTAAACAATATCCTCG	Tb927.10.2010_rev	GGGGACCACTTTGTACAAGAAAGCTGGGTTTACTTGTCGTTCACCACCATTGC
4_known	J9	Tb927.4.1310_for	GGGGACAAGTTTGTACAAAAAAGCAGGCTATGTTCGGTGGTGGCGAAGA	Tb927.4.1310_rev	GGGGACCACTTTGTACAAGAAAGCTGGGTTCAAAACTCTTTTGTCATAGACACGAGA
4_known	J10	Tb927.8.8290_for	GGGGACAAGTTTGTACAAAAAAGCAGGCTATGAGTAATGTCCGTGGAAATATAACCC	Tb927.8.8290_rev	GGGGACCACTTTGTACAAGAAAGCTGGGTTTAGCCAACAATTGTGCCCCAA
4_known	J11	Tb927.8.7460_for	GGGGACAAGTTTGTACAAAAAAGCAGGCTATGGAAGAGGCAAGTAGCGAGC	Tb927.8.7460_rev	GGGGACCACTTTGTACAAGAAAGCTGGGTTTAGGCGATAGTTTGTGCCGAGG
4_known	J12	Tb927.11.5850_for	GGGGACAAGTTTGTACAAAAAAGCAGGCTATGCCTGTGCTTCCGGAGT	Tb927.11.5850_rev	GGGGACCACTTTGTACAAGAAAGCTGGGTTCACAAGGGGCGACCACG
4_known	J13	Tb927.10.2020_for	GGGGACAAGTTTGTACAAAAAAGCAGGCTATGTCTAGACGCCTAAACAATATCCTCG	Tb927.10.2020_rev	GGGGACCACTTTGTACAAGAAAGCTGGGTTCACTTCCCGTCAGCAACGA
4_known	J14	Tb927.10.15940_for	GGGGACAAGTTTGTACAAAAAAGCAGGCTATGCGGCGACGGCTCCAT	Tb927.10.15940_rev	GGGGACCACTTTGTACAAGAAAGCTGGGTTCAACAACAACCCACTGAAGGATG
4_known	J15	Tb927.10.15480_for	GGGGACAAGTTTGTACAAAAAAGCAGGCTATGGCAATCTGGGCATGTATTGC	Tb927.10.15480_rev	GGGGACCACTTTGTACAAGAAAGCTGGGTTTATTCCTCCAAGACGCACAAATAAACC
4_known	J16	Tb927.9.7550_for	GGGGACAAGTTTGTACAAAAAAGCAGGCTATGGAGAAGGGTTCACCATCCG	Tb927.9.7550_rev	GGGGACCACTTTGTACAAGAAAGCTGGGTTTATTTAGCACTGAAGCGAGGAACG
4_known	J17	Tb927.11.950_for	GGGGACAAGTTTGTACAAAAAAGCAGGCTATGCAGGAAGGCCAACTGTTTC	Tb927.11.950_rev	GGGGACCACTTTGTACAAGAAAGCTGGGTTTAGTACCCCTTGTCGCTTGCC
4_known	J18	Tb927.3.3290_for	GGGGACAAGTTTGTACAAAAAAGCAGGCTATGTTCACGACAATGGAGAGCG	Tb927.3.3290_rev	GGGGACCACTTTGTACAAGAAAGCTGGGTTCACTTCTTCTTCAGAAGTAAACTTAACAT
4_known	J19	Tb927.10.13670_for	GGGGACAAGTTTGTACAAAAAAGCAGGCTATGGCCGGGGTAGAGGAAGC	Tb927.10.13670_rev	GGGGACCACTTTGTACAAGAAAGCTGGGTTCAAAACAGGCCAAATCCTGTGG
4_known	J20	Tb927.10.7430_for	GGGGACAAGTTTGTACAAAAAAGCAGGCTATGCTTCAACCCTGCCTGC	Tb927.10.7430_rev	GGGGACCACTTTGTACAAGAAAGCTGGGTTCACTGCCACTTCTCACTGGT
4_known	J21	Tb927.11.6320_for	GGGGACAAGTTTGTACAAAAAAGCAGGCTATGCAGGGGACTGGATCCG	Tb927.11.6320_rev	GGGGACCACTTTGTACAAGAAAGCTGGGTTTATTTACGCCGGTGAGAACGAAC
4_known	J22	Tb927.4.4730_for	GGGGACAAGTTTGTACAAAAAAGCAGGCTATGACATTTGAAGCTGCTGCACA	Tb927.4.4730_rev	GGGGACCACTTTGTACAAGAAAGCTGGGTTTAAGAATACCTCTTAATTGTGTAGTAAAC
4_known	J23	Tb927.4.4840_for	GGGGACAAGTTTGTACAAAAAAGCAGGCTATGGCGTCCGCAGTGGTC	Tb927.4.4840_rev	GGGGACCACTTTGTACAAGAAAGCTGGGTTTACCAGCCCACAGCCACT
4_known	J24	Tb927.8.5730_for	GGGGACAAGTTTGTACAAAAAAGCAGGCTATGAGCGACGAGGGGTTTGA	Tb927.8.5730_rev	GGGGACCACTTTGTACAAGAAAGCTGGGTCTACAGGCGGTAGACACTCTTCA
4_known	K1	Tb927.8.2240_for	GGGGACAAGTTTGTACAAAAAAGCAGGCTATGCGGCGGGCTTCGCAT	Tb927.8.2240_rev	GGGGACCACTTTGTACAAGAAAGCTGGGTCTACGCCATAATGTTGCGCACC
4_known	K2	Tb927.9.15795_for	GGGGACAAGTTTGTACAAAAAAGCAGGCTATGCGGAGAAGTATGTACTGGGT	Tb927.9.15795_rev	GGGGACCACTTTGTACAAGAAAGCTGGGTTTATACCAAAAGCAAAATTAATCCCAGTGG
4_known	K3	Tb927.7.2570_for	GGGGACAAGTTTGTACAAAAAAGCAGGCTATGCTTCGCTTATTGCGGCG	Tb927.7.2570_rev	GGGGACCACTTTGTACAAGAAAGCTGGGTTTACAACTTCGCCTCACAGCC
4_known	K5	Tb927.4.4860_for	GGGGACAAGTTTGTACAAAAAAGCAGGCTATGGCGTCCGCAGTGGTC	Tb927.4.4860_rev	GGGGACCACTTTGTACAAGAAAGCTGGGTTTACCAACCCACAGCCACTCC
4_known	K6	Tb927.8.3130_for	GGGGACAAGTTTGTACAAAAAAGCAGGCTATGAAAAGAATCGTTGTACCCCATCG	Tb927.8.3130_rev	GGGGACCACTTTGTACAAGAAAGCTGGGTTCATTTGTCATCCAGCCCCACG
4_known	K7	Tb927.4.2000_for	GGGGACAAGTTTGTACAAAAAAGCAGGCTATGGCTGAGACAAATATACGTGCAG	Tb927.4.2000_rev	GGGGACCACTTTGTACAAGAAAGCTGGGTTCACTGCTCTTCCTCATTCTGACA
4_known	K8	Tb927.8.6410_for	GGGGACAAGTTTGTACAAAAAAGCAGGCTATGCATCTTGTGAGGAAGGGGA	Tb927.8.6410_rev	GGGGACCACTTTGTACAAGAAAGCTGGGTTCAGTTCGCACTGCTAACCTTAC
4_known	K9	Tb927.10.12740_for	GGGGACAAGTTTGTACAAAAAAGCAGGCTATGACGATGGATATTATCGAGGGGG	Tb927.10.12740_rev	GGGGACCACTTTGTACAAGAAAGCTGGGTTTACCTCGCGTATGGCCAATTTAAA
4_known	K10	Tb927.10.12260_for	GGGGACAAGTTTGTACAAAAAAGCAGGCTATGTCGGTGGATTGGAATACCGT	Tb927.10.12260_rev	GGGGACCACTTTGTACAAGAAAGCTGGGTCTAATCCTTTCGCAACTTTGGCG
4_known	K11	Tb927.8.4390_for	GGGGACAAGTTTGTACAAAAAAGCAGGCTATGGGGAAACGATCGATTCGGA	Tb927.8.4390_rev	GGGGACCACTTTGTACAAGAAAGCTGGGTTCAGTCCTCAGGACTGTAGTTTTCC
4_known	K12	Tb927.10.10650_for	GGGGACAAGTTTGTACAAAAAAGCAGGCTATGTCGGCCAACAATTTCTCCG	Tb927.10.10650_rev	GGGGACCACTTTGTACAAGAAAGCTGGGTTCACCATTTCAAACTATGCACCCC
4_known	K13	Tb927.5.1520_for	GGGGACAAGTTTGTACAAAAAAGCAGGCTATGATGCGGCGAGTAACTTCTTC	Tb927.5.1520_rev	GGGGACCACTTTGTACAAGAAAGCTGGGTCTAGAGGATGAATTTCTTGATGTCCACC
4_known	K14	Tb927.6.400_for	GGGGACAAGTTTGTACAAAAAAGCAGGCTATGCAGGTTGATTGGGGTGC	Tb927.6.400_rev	GGGGACCACTTTGTACAAGAAAGCTGGGTTCACTGCTTATGTATCTTTGGCGTT
4_known	K15	Tb927.6.1900_for	GGGGACAAGTTTGTACAAAAAAGCAGGCTATGCCGGGACGCGAAAAG	Tb927.6.1900_rev	GGGGACCACTTTGTACAAGAAAGCTGGGTTCAACTGTTCCCTCCTTTCGCC
4_known	K16	Tb927.1.4650_for	GGGGACAAGTTTGTACAAAAAAGCAGGCTATGTCCTTGGAGGAGGGAAGC	Tb927.1.4650_rev	GGGGACCACTTTGTACAAGAAAGCTGGGTCTACATCTCAGCGTAATCCACTTCATG
4_known	K17	Tb927.6.1580_for	GGGGACAAGTTTGTACAAAAAAGCAGGCTATGAAGCGCGCAAGGAGC	Tb927.6.1580_rev	GGGGACCACTTTGTACAAGAAAGCTGGGTTCATGAGAGCAGGAAAAACTCCCT
4_known	K18	Tb927.11.13250_for	GGGGACAAGTTTGTACAAAAAAGCAGGCTATGAGCGAGAATGACATTGCCG	Tb927.11.13250_rev	GGGGACCACTTTGTACAAGAAAGCTGGGTTTAATTCAGTTTCACTGGCACTCCAC
4_known	K19	Tb927.4.1350_for	GGGGACAAGTTTGTACAAAAAAGCAGGCTATGATGGAAAGCGTGAAGCAGTT	Tb927.4.1350_rev	GGGGACCACTTTGTACAAGAAAGCTGGGTCTACCCCGTGTCGTTCCGC

4_known	K20	Tb927.3.5230_for	GGGGACAAGTTTGTACAAAAAAGCAGGCTATGGCTAGCGTGAATGCTCCC	Tb927.3.5230_rev	GGGGACCACTTTGTACAAGAAAGCTGGGTTCATTCGTCGTTGCATATACCTTCG
4_known	K21	Tb927.11.16730_for	GGGGACAAGTTTGTACAAAAAAGCAGGCTATGTTCCGTCGCTGCTTTCC	Tb927.11.16730_rev	GGGGACCACTTTGTACAAGAAAGCTGGGTTTAGAAGTTGATTGTTTTGGCAAAGCA
4_known	K22	Tb927.3.1110_for	GGGGACAAGTTTGTACAAAAAAGCAGGCTATGTCTTTTAGAGAGCTTCGTTCCTTTG	Tb927.3.1110_rev	GGGGACCACTTTGTACAAGAAAGCTGGGTCTAGAGGTCGCTGCTGCTTTC
4_known	K23	Tb927.10.4730_for	GGGGACAAGTTTGTACAAAAAAGCAGGCTATGTTGGGTGTGAGGGCGT	Tb927.10.4730_rev	GGGGACCACTTTGTACAAGAAAGCTGGGTCTATGAGTGGCTATCTTTCATTTTGCG
4_known	K24	Tb927.9.4520_for	GGGGACAAGTTTGTACAAAAAAGCAGGCTATGCTTCGTCCATCCTTTTGCA	Tb927.9.4520_rev	GGGGACCACTTTGTACAAGAAAGCTGGGTTCAAAGTATGTGCTTTACTTGTGTGAGG
4_known	L1	Tb927.6.2060_for	GGGGACAAGTTTGTACAAAAAAGCAGGCTATGGCAAAGACAGTAGATAACGCC	Tb927.6.2060_rev	GGGGACCACTTTGTACAAGAAAGCTGGGTCTACACGATTTTATCAACTGGAAGAACG
4_known	L2	Tb927.10.4520_for	GGGGACAAGTTTGTACAAAAAAGCAGGCTATGACTCAGTCAACGATCCCTTTGC	Tb927.10.4520_rev	GGGGACCACTTTGTACAAGAAAGCTGGGTTCACCCGACGGAGGTGCT
4_known	L3	Tb927.3.4380_for	GGGGACAAGTTTGTACAAAAAAGCAGGCTATGACCTTTTCTAGCGAGAGTAGTGT	Tb927.3.4380_rev	GGGGACCACTTTGTACAAGAAAGCTGGGTTCACATGGAAAAAGCGGATGACC
4_known	L4	Tb927.11.7170_for	GGGGACAAGTTTGTACAAAAAAGCAGGCTATGGTGCTTGATATACAGCTGTTTCG	Tb927.11.7170_rev	GGGGACCACTTTGTACAAGAAAGCTGGGTTCACTCCCCCTTGTCGGGT
4_known	L5	Tb927.9.12110_for	GGGGACAAGTTTGTACAAAAAAGCAGGCTATGTCAATGGATGTCGGTGTTGTC	Tb927.9.12110_rev	GGGGACCACTTTGTACAAGAAAGCTGGGTTTATTGCAACTCAGGCCATTGGAA
4_known	L6	Tb927.10.14150_for	GGGGACAAGTTTGTACAAAAAAGCAGGCTATGAGCAAGACAGAGACGGCA	Tb927.10.14150_rev	GGGGACCACTTTGTACAAGAAAGCTGGGTTTACGCAAAATCCTCCCCCTCT
4_known	L7	Tb927.6.3780_for	GGGGACAAGTTTGTACAAAAAAGCAGGCTATGAACGTTAATTCGTGTGGCAATATC	Tb927.6.3780_rev	GGGGACCACTTTGTACAAGAAAGCTGGGTTTAATCCACTTCCTCCATACGCAGC
4_known	L8	Tb927.8.3580_for	GGGGACAAGTTTGTACAAAAAAGCAGGCTATGAATCTTGCTGCTCTCGCC	Tb927.8.3580_rev	GGGGACCACTTTGTACAAGAAAGCTGGGTTCATTGCAAGCTGTGTAACATCGG
4_known	L9	Tb927.1.1150_for	GGGGACAAGTTTGTACAAAAAAGCAGGCTATGGCAATTGTTGTCGTGCAGG	Tb927.1.1150_rev	GGGGACCACTTTGTACAAGAAAGCTGGGTTCAATCCTCCTCCGCATCAGC
4_known	L10	Tb927.1.1230_for	GGGGACAAGTTTGTACAAAAAAGCAGGCTATGCGAGGCATTACATTGGCG	Tb927.1.1230_rev	GGGGACCACTTTGTACAAGAAAGCTGGGTTTACGTACCCGGCAGCCA
4_known	L11	Tb927.4.1620_for	GGGGACAAGTTTGTACAAAAAAGCAGGCTATGCCGTTCAATAGAGAAATACTTGCC	Tb927.4.1620_rev	GGGGACCACTTTGTACAAGAAAGCTGGGTTTACACAATAATGGACGAGACAAAACTGA
4_known	L12	Tb927.7.940_for	GGGGACAAGTTTGTACAAAAAAGCAGGCTATGACCGATATGTTGTTTGCTGTTTTATTG	Tb927.7.940_rev	GGGGACCACTTTGTACAAGAAAGCTGGGTTTACTCGCATATAGCGGGTGTTTG
4_known	L13	Tb927.8.8310_for	GGGGACAAGTTTGTACAAAAAAGCAGGCTATGAAGAAAGAATCCGTTCCTTCCAAA	Tb927.8.8310_rev	GGGGACCACTTTGTACAAGAAAGCTGGGTTTATTGTGCTTCAATTACCACACTGGA
4_known	L14	Tb927.11.6210_for	GGGGACAAGTTTGTACAAAAAAGCAGGCTATGTTGCTTGAAGTTGCCATTTTCTT	Tb927.11.6210_rev	GGGGACCACTTTGTACAAGAAAGCTGGGTCTAAGCAGCTGCCGCCTTC
4_known	L15	Tb927.11.6230_for	GGGGACAAGTTTGTACAAAAAAGCAGGCTATGACCGACATCCTCATGAAGATGA	Tb927.11.6230_rev	GGGGACCACTTTGTACAAGAAAGCTGGGTTCACGCAACCTTCCGCTTG
4_known	L16	Tb927.4.2480_for	GGGGACAAGTTTGTACAAAAAAGCAGGCTATGCGCGATTGGCATGCG	Tb927.4.2480_rev	GGGGACCACTTTGTACAAGAAAGCTGGGTTCATGCTGTTGCTTCCTTGTTCC
4_known	L17	Tb927.10.12400_for	GGGGACAAGTTTGTACAAAAAAGCAGGCTATGACAGCCAAGCCTGCATC	Tb927.10.12400_rev	GGGGACCACTTTGTACAAGAAAGCTGGGTTCAGTTAGTGGAAAGCAGGGCC
4_known	L18	Tb927.9.9870_for	GGGGACAAGTTTGTACAAAAAAGCAGGCTATGCCCAACATACCGTTAGTGGC	Tb927.9.9870_rev	GGGGACCACTTTGTACAAGAAAGCTGGGTTCATCTCCTCTGCGTAGCACA
4_known	L19	Tb927.10.1550_for	GGGGACAAGTTTGTACAAAAAAGCAGGCTATGACCGACGTAGACCAGTGG	Tb927.10.1550_rev	GGGGACCACTTTGTACAAGAAAGCTGGGTCTATGAAGCAGTCGCACCCG
4_known	L20	Tb927.7.610_for	GGGGACAAGTTTGTACAAAAAAGCAGGCTATGCGGTTTAGTCGCCCATG	Tb927.7.610_rev	GGGGACCACTTTGTACAAGAAAGCTGGGTTTAAACCCAATCACTACCAGTCTTTGTG
4_known	L21	Tb927.9.11810_for	GGGGACAAGTTTGTACAAAAAAGCAGGCTATGGTGACCATGGAAGGAACCC	Tb927.9.11810_rev	GGGGACCACTTTGTACAAGAAAGCTGGGTCTATGTGAGTTCGTCCTCATCCACC
4_known	L22	Tb927.11.16860_for	GGGGACAAGTTTGTACAAAAAAGCAGGCTATGAGTAACCCTTTTGAGAAAGTTGCG	Tb927.11.16860_rev	GGGGACCACTTTGTACAAGAAAGCTGGGTCTACTTACCACCATCTTGCGACAAC
4_known	L23	Tb927.9.15980_for	GGGGACAAGTTTGTACAAAAAAGCAGGCTATGCTTGGCTTCGGTTCTGTG	Tb927.9.15980_rev	GGGGACCACTTTGTACAAGAAAGCTGGGTTTACTGTGACTCATCTTTCGGGAGA
4_known	L24	Tb927.11.5350_for	GGGGACAAGTTTGTACAAAAAAGCAGGCTATGAAGTGGCGCGTGAAGATATT	Tb927.11.5350_rev	GGGGACCACTTTGTACAAGAAAGCTGGGTCTAAATGGGCGCACGGGC
4_known	M1	Tb927.8.3750_for	GGGGACAAGTTTGTACAAAAAAGCAGGCTATGTCAAAAACACTTTATCTCTTATATGAG	Tb927.8.3750_rev	GGGGACCACTTTGTACAAGAAAGCTGGGTCTATGCCGATTCTTGTGGCACT
4_known	M2	Tb927.10.13430_for	GGGGACAAGTTTGTACAAAAAAGCAGGCTATGTGCATGCGTGCTCGT	Tb927.10.13430_rev	GGGGACCACTTTGTACAAGAAAGCTGGGTCTACGCTATGTTGTACTTTGTGAGAAGT
4_known	M3	Tb927.11.12420_for	GGGGACAAGTTTGTACAAAAAAGCAGGCTATGGCTTCATACATAGCGTCAAACG	Tb927.11.12420_rev	GGGGACCACTTTGTACAAGAAAGCTGGGTTTACATGACAGGTGTTGGGGGG
4_known	M4	Tb927.10.13130_for	GGGGACAAGTTTGTACAAAAAAGCAGGCTATGCCGCTAAACCCTCCTTCA	Tb927.10.13130_rev	GGGGACCACTTTGTACAAGAAAGCTGGGTCTACTCGACTACCACAACCTCATTG
4_known	M5	Tb927.4.3770_for	GGGGACAAGTTTGTACAAAAAAGCAGGCTATGTCATACACAACAGAAAGTGCCTT	Tb927.4.3770_rev	GGGGACCACTTTGTACAAGAAAGCTGGGTCTATTTACAGCCGGAAACCAAAGCT
4_known	M6	Tb927.10.13620_for	GGGGACAAGTTTGTACAAAAAAGCAGGCTATGTTGCGCCGTGTAGGAGA	Tb927.10.13620_rev	GGGGACCACTTTGTACAAGAAAGCTGGGTCTAATGAATAGCCACAGGTCCGGG
4_known	M7	Tb927.10.15250_for	GGGGACAAGTTTGTACAAAAAAGCAGGCTATGGCAAGTTCTACAGCAACCG	Tb927.10.15250_rev	GGGGACCACTTTGTACAAGAAAGCTGGGTTTACATCTCTGGGTTCTCCTCGC
4_known	M8	Tb927.1.1080_for	GGGGACAAGTTTGTACAAAAAAGCAGGCTATGAGTCAACTCAGCGAGTTGC	Tb927.1.1080_rev	GGGGACCACTTTGTACAAGAAAGCTGGGTTCAAGCATCTTTCGGAAAAGAGCG
4_known	M9	Tb927.11.2640_for	GGGGACAAGTTTGTACAAAAAAGCAGGCTATGGGTGGACAGTCTAAGAAGCA	Tb927.11.2640_rev	GGGGACCACTTTGTACAAGAAAGCTGGGTTTACTGCACACCACTTTTCATCTTACT
4_known	M10	Tb927.8.1270_for	GGGGACAAGTTTGTACAAAAAAGCAGGCTATGGCCGTCACATCAAAGGC	Tb927.8.1270_rev	GGGGACCACTTTGTACAAGAAAGCTGGGTCTACTCCTCAGCAGACCCCTTT
4_known	M11	Tb927.11.8400_for	GGGGACAAGTTTGTACAAAAAAGCAGGCTATGATTCGCTTGAGCGAAGTTGT	Tb927.11.8400_rev	GGGGACCACTTTGTACAAGAAAGCTGGGTTCAGGGCGGCTGCCCACC
4_known	M12	Tb927.3.3270_for	GGGGACAAGTTTGTACAAAAAAGCAGGCTATGGCCGTTGAATCTCGCAG	Tb927.3.3270_rev	GGGGACCACTTTGTACAAGAAAGCTGGGTTTAGAGCTTGGCCAGTTCCTCG
4_known	M13	Tb927.8.7680_for	GGGGACAAGTTTGTACAAAAAAGCAGGCTATGCATCCTGACGGAACCACT	Tb927.8.7680_rev	GGGGACCACTTTGTACAAGAAAGCTGGGTTTACCACTCCACTGCGACACT
4_known	M14	Tb927.10.1140_for	GGGGACAAGTTTGTACAAAAAAGCAGGCTATGCCCCGTGAGGTTGTTACA	Tb927.10.1140_rev	GGGGACCACTTTGTACAAGAAAGCTGGGTTCAAAAGTAAACAAGGTCACGCATGC
4_known	M15	Tb927.11.5400_for	GGGGACAAGTTTGTACAAAAAAGCAGGCTATGGTGTTAGCAGAACTCGGACA	Tb927.11.5400_rev	GGGGACCACTTTGTACAAGAAAGCTGGGTTCACTTTATGCTCTGAAGTTGCTTCA
4_known	M16	Tb927.2.3880_for	GGGGACAAGTTTGTACAAAAAAGCAGGCTATGGAACAGTCTCCTGAGCAGC	Tb927.2.3880_rev	GGGGACCACTTTGTACAAGAAAGCTGGGTTTAACAGAACATCTGTGGTACCATTGG
4_known	M17	Tb927.7.1550_for	GGGGACAAGTTTGTACAAAAAAGCAGGCTATGTTGATGCATACCGCGCC	Tb927.7.1550_rev	GGGGACCACTTTGTACAAGAAAGCTGGGTTCATTCACGGTCTCTGTCACGC
4_known	M18	Tb927.11.8170_for	GGGGACAAGTTTGTACAAAAAAGCAGGCTATGGAAGACTACGAAACCGTGGC	Tb927.11.8170_rev	GGGGACCACTTTGTACAAGAAAGCTGGGTTCACCGCTTCGAGCCATTG
4_known	M20	Tb927.7.690_for	GGGGACAAGTTTGTACAAAAAAGCAGGCTATGCAAAAGAAGAACTGCAGGTGG	Tb927.7.690_rev	GGGGACCACTTTGTACAAGAAAGCTGGGTTTACACATTCTGTTGTAACCAATCACGA
4_known	M21	Tb927.7.1440_for	GGGGACAAGTTTGTACAAAAAAGCAGGCTATGAGCGCAAGCGACGGA	Tb927.7.1440_rev	GGGGACCACTTTGTACAAGAAAGCTGGGTTTACACCCCAATGGGATTTCCTACG
4_known	M22	Tb927.8.5450_for	GGGGACAAGTTTGTACAAAAAAGCAGGCTATGAGAGAGCCGATACAAACTTCAAC	Tb927.8.5450_rev	GGGGACCACTTTGTACAAGAAAGCTGGGTTCAGAGTTCAGCAATGACGCTG
4_known	M23	Tb927.9.5260_for	GGGGACAAGTTTGTACAAAAAAGCAGGCTATGCTTTCCCTATATGCGTACATTTTCC	Tb927.9.5260_rev	GGGGACCACTTTGTACAAGAAAGCTGGGTCTAGGAGGCATCAATGGATTTCAGAC
4_known	M24	Tb927.10.4640_for	GGGGACAAGTTTGTACAAAAAAGCAGGCTATGGCGTCGAACCCTGATTCA	Tb927.10.4640_rev	GGGGACCACTTTGTACAAGAAAGCTGGGTTTAGTTAGTATTGTGGGCATCCGGT
4_known	N1	Tb927.9.1570_for	GGGGACAAGTTTGTACAAAAAAGCAGGCTATGCGCGGGGAGCATGAA	Tb927.9.1570_rev	GGGGACCACTTTGTACAAGAAAGCTGGGTTTAGTCCCAGACAACAGACTTCAAGC
4_known	N2	Tb927.2.5150_for	GGGGACAAGTTTGTACAAAAAAGCAGGCTATGGATTACGCCGCCGCA	Tb927.2.5150_rev	GGGGACCACTTTGTACAAGAAAGCTGGGTCTATATAACCCCAAGTTGCCGCC
4_known	N3	Tb927.10.7510_for	GGGGACAAGTTTGTACAAAAAAGCAGGCTATGGCGCCTTATGCTGTGG	Tb927.10.7510_rev	GGGGACCACTTTGTACAAGAAAGCTGGGTTCAAATAACAGCTAAAAAGTGGTCGGT
4_known	N4	Tb927.11.10930_for	GGGGACAAGTTTGTACAAAAAAGCAGGCTATGGCATGTGTGCATGTCCTG	Tb927.11.10930_rev	GGGGACCACTTTGTACAAGAAAGCTGGGTTCAAGCCGCCCCGTATGC
4_known	N5	Tb927.9.13490_for	GGGGACAAGTTTGTACAAAAAAGCAGGCTATGTCGTCAAATTGTATTCGCAACAC	Tb927.9.13490_rev	GGGGACCACTTTGTACAAGAAAGCTGGGTTCAAGGGGCATTTTTTTGGTTAACG
4_known	N6	Tb927.5.1060_for	GGGGACAAGTTTGTACAAAAAAGCAGGCTATGTCTGTCAGTTTTTCCACTCTGATT	Tb927.5.1060_rev	GGGGACCACTTTGTACAAGAAAGCTGGGTTTAATACCAGTACTTGTAGGACCAGTGT
4_known	N7	Tb927.10.5930_for	GGGGACAAGTTTGTACAAAAAAGCAGGCTATGTCGTCAACGGATGCAATGA	Tb927.10.5930_rev	GGGGACCACTTTGTACAAGAAAGCTGGGTTTACAATCCAAGAACCTCCTGTATTTTCTG
4_known	N8	Tb927.3.1700_for	GGGGACAAGTTTGTACAAAAAAGCAGGCTATGCTTCAAAAGATCAGCGCTGA	Tb927.3.1700_rev	GGGGACCACTTTGTACAAGAAAGCTGGGTCTATCGTATAACTAACCCTTTTGAATCCGC
4_known	N9	Tb927.9.10010_for	GGGGACAAGTTTGTACAAAAAAGCAGGCTATGGTGGAAAGTGGAATTGGTGG	Tb927.9.10010_rev	GGGGACCACTTTGTACAAGAAAGCTGGGTCTAGAGCCGGCGCAGGCT
4_known	N10	Tb927.11.9360_for	GGGGACAAGTTTGTACAAAAAAGCAGGCTATGTTAAGGCGTACAATTTTCCTGGG	Tb927.11.9360_rev	GGGGACCACTTTGTACAAGAAAGCTGGGTCTAAACGGCGATGCTGGAGAAC
4_known	N11	Tb927.4.1020_for	GGGGACAAGTTTGTACAAAAAAGCAGGCTATGGTGGGATTTAAGACTCTTTCATCCA	Tb927.4.1020_rev	GGGGACCACTTTGTACAAGAAAGCTGGGTTCAAACAAAAACACTCTTCACCGC
4_known	N12	Tb927.7.6930_for	GGGGACAAGTTTGTACAAAAAAGCAGGCTATGTTTTGGCGCTTCCCTGC	Tb927.7.6930_rev	GGGGACCACTTTGTACAAGAAAGCTGGGTCTCACGCTCCGGTACTTAATGACA
4_known	N13	Tb927.8.1010_for	GGGGACAAGTTTGTACAAAAAAGCAGGCTATGAGTTATGGCAAGTGGGCTG	Tb927.8.1010_rev	GGGGACCACTTTGTACAAGAAAGCTGGGTTTACAACACCCGGTTCTCAGCT
4_known	N14	Tb927.9.4850_for	GGGGACAAGTTTGTACAAAAAAGCAGGCTATGCTTGAAGGAAGTGATGCGAG	Tb927.9.4850_rev	GGGGACCACTTTGTACAAGAAAGCTGGGTTCATCGAAGCAGATTCACTCCCC
4_known	N15	Tb927.5.2080_for	GGGGACAAGTTTGTACAAAAAAGCAGGCTATGTCCTTCAATGAATCGGCATCC	Tb927.5.2080_rev	GGGGACCACTTTGTACAAGAAAGCTGGGTTTAAAGTTTGGCAACACCGTGACT
4_known	N16	Tb927.10.9440_for	GGGGACAAGTTTGTACAAAAAAGCAGGCTATGATATGCCGCACATCTTTTCTGC	Tb927.10.9440_rev	GGGGACCACTTTGTACAAGAAAGCTGGGTCTACTCGTTCTGTTTCTTTGTCGCG
4_known	N17	Tb927.1.4300_for	GGGGACAAGTTTGTACAAAAAAGCAGGCTATGGCTCATATATCCGTTACTGACACC	Tb927.1.4300_rev	GGGGACCACTTTGTACAAGAAAGCTGGGTTTAACGACGACTCGTAGCCGC
4_known	N18	Tb927.10.8070_for	GGGGACAAGTTTGTACAAAAAAGCAGGCTATGAGAGCTTCGTTGTGTACCCG	Tb927.10.8070_rev	GGGGACCACTTTGTACAAGAAAGCTGGGTCTACTCCAGAAGGGCAGCGA
4_known	N19	Tb927.2.3800_for	GGGGACAAGTTTGTACAAAAAAGCAGGCTATGCTGCGCGCGCGCCTG	Tb927.2.3800_rev	GGGGACCACTTTGTACAAGAAAGCTGGGTCTAGTATGCCGAAACGGCAGTC
4_known	N20	Tb927.9.10340_for	GGGGACAAGTTTGTACAAAAAAGCAGGCTATGCACATGTCTAATGTCACCGAC	Tb927.9.10340_rev	GGGGACCACTTTGTACAAGAAAGCTGGGTTCATTTGAGAGACTCATAAGCTTGCAT
4_known	N21	Tb927.10.10390_for	GGGGACAAGTTTGTACAAAAAAGCAGGCTATGTCCAAGGCCTTCGATTTGG	Tb927.10.10390_rev	GGGGACCACTTTGTACAAGAAAGCTGGGTTCACAGGTTAGAGTCCGGAAGC
4_known	N22	Tb927.11.4800_for	GGGGACAAGTTTGTACAAAAAAGCAGGCTATGAACTCTAAAGACACGAATGAGTTGAA	Tb927.11.4800_rev	GGGGACCACTTTGTACAAGAAAGCTGGGTCTACTGGCCTCCCTTCTGGC
4_known	N23	Tb927.1.1700_for	GGGGACAAGTTTGTACAAAAAAGCAGGCTATGTCTACACGTAGCGGTAAGCA	Tb927.1.1700_rev	GGGGACCACTTTGTACAAGAAAGCTGGGTTTATTGGAATAAAGACTTGAACAACACCTC
4_known	N24	Tb927.11.2690_for	GGGGACAAGTTTGTACAAAAAAGCAGGCTATGCTCCGCCGAACAAATTTTTTC	Tb927.11.2690_rev	GGGGACCACTTTGTACAAGAAAGCTGGGTCTACGTGAGGGGCGCCAA
4_known	O1	Tb927.11.13880_for	GGGGACAAGTTTGTACAAAAAAGCAGGCTATGTCGCGCGATCCGCCA	Tb927.11.13880_rev	GGGGACCACTTTGTACAAGAAAGCTGGGTTCAGAGCACATCTGCAGACTCT
4_known	O2	Tb927.10.5950_for	GGGGACAAGTTTGTACAAAAAAGCAGGCTATGTCGTCAACGGATGCAATGA	Tb927.10.5950_rev	GGGGACCACTTTGTACAAGAAAGCTGGGTTCACTCAGCATCACCAATGCC
4_known	O4	Tb927.5.3760_for	GGGGACAAGTTTGTACAAAAAAGCAGGCTATGCACAGAGCCGCGTATG	Tb927.5.3760_rev	GGGGACCACTTTGTACAAGAAAGCTGGGTCTAAATGTAGTCCCGCCTAAATAATCCC
4_known	O5	Tb927.11.15960_for	GGGGACAAGTTTGTACAAAAAAGCAGGCTATGTCTGACAAAGGCAAGTGTGC	Tb927.11.15960_rev	GGGGACCACTTTGTACAAGAAAGCTGGGTTCAGCAGACAGGCGGGGG
4_known	O6	Tb927.11.11690_for	GGGGACAAGTTTGTACAAAAAAGCAGGCTATGGGCGGACATAATGAAGAAGTG	Tb927.11.11690_rev	GGGGACCACTTTGTACAAGAAAGCTGGGTTCAACGGTTGGCGGGTTG
4_known	O7	Tb927.4.3980_for	GGGGACAAGTTTGTACAAAAAAGCAGGCTATGTTTGTTTTATGTACAGTTGAAAACATG	Tb927.4.3980_rev	GGGGACCACTTTGTACAAGAAAGCTGGGTCTAAAGCCGTTGGGACGTCG
4_known	O8	Tb927.11.11750_for	GGGGACAAGTTTGTACAAAAAAGCAGGCTATGATGCGTCCACTCTCACTTGT	Tb927.11.11750_rev	GGGGACCACTTTGTACAAGAAAGCTGGGTTTAGACAGCCATCTTTTGTTTAAACATCAC
4_known	O9	Tb927.11.15950_for	GGGGACAAGTTTGTACAAAAAAGCAGGCTATGTCTGACAAAGGCAAGTGTGC	Tb927.11.15950_rev	GGGGACCACTTTGTACAAGAAAGCTGGGTTCAGCACTGAGTTTCCGGCG
4_known	O10	Tb927.11.14120_for	GGGGACAAGTTTGTACAAAAAAGCAGGCTATGAGTACCATGGAGAACACCATTCT	Tb927.11.14120_rev	GGGGACCACTTTGTACAAGAAAGCTGGGTCTAAAAACGCATTAGCTTTGAGTTCCTG
4_known	O11	Tb927.11.12230_for	GGGGACAAGTTTGTACAAAAAAGCAGGCTATGATTCGTTTCAGTTGGGTTCGC	Tb927.11.12230_rev	GGGGACCACTTTGTACAAGAAAGCTGGGTTCAGAGAATGTACTTTGCTAAGTCAATGT
4_known	O12	Tb927.5.450_for	GGGGACAAGTTTGTACAAAAAAGCAGGCTATGCTCCGGCGTGTGGGT	Tb927.5.450_rev	GGGGACCACTTTGTACAAGAAAGCTGGGTTTAGTTCCAATTCCGCACGATCTTT
4_known	O13	Tb927.10.8230_for	GGGGACAAGTTTGTACAAAAAAGCAGGCTATGCGCGCTATTTTTCTTGTTGC	Tb927.10.8230_rev	GGGGACCACTTTGTACAAGAAAGCTGGGTCTACAGATCTTGTTTATCAACATTGCTCTC
4_known	O14	Tb927.5.1750_for	GGGGACAAGTTTGTACAAAAAAGCAGGCTATGTACGCAAAGGGTACGCG	Tb927.5.1750_rev	GGGGACCACTTTGTACAAGAAAGCTGGGTTTAGCTGCGAGACACCGCT
4_known	O15	Tb927.8.6420_for	GGGGACAAGTTTGTACAAAAAAGCAGGCTATGCCATTCCAGCTAAGCCGT	Tb927.8.6420_rev	GGGGACCACTTTGTACAAGAAAGCTGGGTTTATCTGTCCCATTGCATATATGGTTGC
4_known	O16	Tb927.8.6110_for	GGGGACAAGTTTGTACAAAAAAGCAGGCTATGCGCCGAACTGTGCGT	Tb927.8.6110_rev	GGGGACCACTTTGTACAAGAAAGCTGGGTCTACTTCACGAACCGGTAATATTCGAT
4_known	O17	Tb927.10.4900_for	GGGGACAAGTTTGTACAAAAAAGCAGGCTATGGAGCCTGATGATGGTGTTGC	Tb927.10.4900_rev	GGGGACCACTTTGTACAAGAAAGCTGGGTTCACCTATAGTGAAATGTTTGCTTTTGC
4_known	O18	Tb927.8.3100_for	GGGGACAAGTTTGTACAAAAAAGCAGGCTATGGACGTATCGCGGTTTCG	Tb927.8.3100_rev	GGGGACCACTTTGTACAAGAAAGCTGGGTTTACGCTTGACTACCAGAGGCA
4_known	O19	Tb927.7.5740_for	GGGGACAAGTTTGTACAAAAAAGCAGGCTATGCTACGGTCCTTTAGAAGTAATGC	Tb927.7.5740_rev	GGGGACCACTTTGTACAAGAAAGCTGGGTTCAATTCATGAGATCAGTATCGCGTAAG
4_known	O20	Tb927.11.4610_for	GGGGACAAGTTTGTACAAAAAAGCAGGCTATGTCTGAAAAGGGAACATCGTTAAACC	Tb927.11.4610_rev	GGGGACCACTTTGTACAAGAAAGCTGGGTCTACTTCCTCCCCTCTGCCC
4_known	O21	Tb927.11.2660_for	GGGGACAAGTTTGTACAAAAAAGCAGGCTATGGGACAGGGGAAAAGCGA	Tb927.11.2660_rev	GGGGACCACTTTGTACAAGAAAGCTGGGTTTAGCGAGATGGTGTGGCTGG
4_known	O22	Tb927.1.3130_for	GGGGACAAGTTTGTACAAAAAAGCAGGCTATGGCCATCGCTCCCGCC	Tb927.1.3130_rev	GGGGACCACTTTGTACAAGAAAGCTGGGTTCAGCCATGTGCGCCAAAC
4_known	O23	Tb927.10.14140_for	GGGGACAAGTTTGTACAAAAAAGCAGGCTATGTCGCAGCTAGAGCACAAC	Tb927.10.14140_rev	GGGGACCACTTTGTACAAGAAAGCTGGGTTTAAGGAAGATAGATGAGGCGAGTTTGG
4_known	O24	Tb927.3.3080_for	GGGGACAAGTTTGTACAAAAAAGCAGGCTATGAAGCCGCGGGATATTTCC	Tb927.3.3080_rev	GGGGACCACTTTGTACAAGAAAGCTGGGTTCAACACAACCCATAATTTGTAACCTTGT
4_known	P1	Tb927.10.7520_for	GGGGACAAGTTTGTACAAAAAAGCAGGCTATGACTGCGTGGCACCGC	Tb927.10.7520_rev	GGGGACCACTTTGTACAAGAAAGCTGGGTTTAAGACCCTTGCGAGTCATACAAATT
4_known	P2	Tb927.10.5210_for	GGGGACAAGTTTGTACAAAAAAGCAGGCTATGAACCCGTCCTATTTGAATCAGATATTG	Tb927.10.5210_rev	GGGGACCACTTTGTACAAGAAAGCTGGGTTTAATCCGGAGTAATGAAATTGGACAGTAC
4_known	P3	Tb927.9.14220_for	GGGGACAAGTTTGTACAAAAAAGCAGGCTATGGACGCAGCTCTCGCT	Tb927.9.14220_rev	GGGGACCACTTTGTACAAGAAAGCTGGGTTCACTTGCGGCCGGGTTT
4_known	P4	Tb927.11.11740_for	GGGGACAAGTTTGTACAAAAAAGCAGGCTATGATGCGTCCACTCTCACTTGT	Tb927.11.11740_rev	GGGGACCACTTTGTACAAGAAAGCTGGGTTTACCTTACGGTGGGATCTTTCGG
4_known	P5	Tb927.2.3910_for	GGGGACAAGTTTGTACAAAAAAGCAGGCTATGGCATTTGGAACGTTCCCC	Tb927.2.3910_rev	GGGGACCACTTTGTACAAGAAAGCTGGGTTCAAAACTGTGTGAAGAGTAGCGC
4_known	P6	Tb927.10.7140_for	GGGGACAAGTTTGTACAAAAAAGCAGGCTATGCCTTACGCCTGCCTCC	Tb927.10.7140_rev	GGGGACCACTTTGTACAAGAAAGCTGGGTCTAGCCCTTCGCCCTCGT
4_known	P7	Tb927.8.3430_for	GGGGACAAGTTTGTACAAAAAAGCAGGCTATGGATTTCCTTCAGTTTTGCTCATCG	Tb927.8.3430_rev	GGGGACCACTTTGTACAAGAAAGCTGGGTCTAGAGTGGTCCCACTTGATGAAAC
4_known	P8	Tb927.7.2420_for	GGGGACAAGTTTGTACAAAAAAGCAGGCTATGAGTGAGCGGATTTTGCCG	Tb927.7.2420_rev	GGGGACCACTTTGTACAAGAAAGCTGGGTTTATATCGCATTCTCCGGCGC
4_known	P9	Tb927.10.3700_for	GGGGACAAGTTTGTACAAAAAAGCAGGCTATGTCTCAGTATGACCATTTATTTGATACC	Tb927.10.3700_rev	GGGGACCACTTTGTACAAGAAAGCTGGGTTCAGGTACCACAGCCGCA
4_known	P10	Tb927.11.2500_for	GGGGACAAGTTTGTACAAAAAAGCAGGCTATGAAGGCATACAAAGAGCTCGAG	Tb927.11.2500_rev	GGGGACCACTTTGTACAAGAAAGCTGGGTTCAGTTGGCATCGTCACGGT
4_known	P11	Tb927.9.11790_for	GGGGACAAGTTTGTACAAAAAAGCAGGCTATGATTGGCACTATGGAAGACGATG	Tb927.9.11790_rev	GGGGACCACTTTGTACAAGAAAGCTGGGTCTAATCCTTACTTCCAAGACTCTCAACAG
4_known	P12	Tb927.3.3190_for	GGGGACAAGTTTGTACAAAAAAGCAGGCTATGGCGCAGGTTGCAGATG	Tb927.3.3190_rev	GGGGACCACTTTGTACAAGAAAGCTGGGTCTACATCTTCTTCCCCTCGTACACG
4_known	P13	Tb927.10.6190_for	GGGGACAAGTTTGTACAAAAAAGCAGGCTATGCGAAAGACGACTCGTCTACT	Tb927.10.6190_rev	GGGGACCACTTTGTACAAGAAAGCTGGGTCTACACACGTGACTCCAGAACATATTT
4_known	P14	Tb927.1.3950_for	GGGGACAAGTTTGTACAAAAAAGCAGGCTATGAAAGGTAACCAGGAATTTTTTCAGAAG	Tb927.1.3950_rev	GGGGACCACTTTGTACAAGAAAGCTGGGTTTATGCATACTTGGCCCAAATGCC
4_known	P15	Tb927.11.14100_for	GGGGACAAGTTTGTACAAAAAAGCAGGCTATGCAAACCTGTCTTTTGAGTAACATTC	Tb927.11.14100_rev	GGGGACCACTTTGTACAAGAAAGCTGGGTTCACTCATTCTGTTCGTCCTGTGG
4_known	P16	Tb927.11.670_for	GGGGACAAGTTTGTACAAAAAAGCAGGCTATGTCATTTCCGACTTCTCTCCACG	Tb927.11.670_rev	GGGGACCACTTTGTACAAGAAAGCTGGGTCTACTGACCTAACCGGCGACC
4_known	P17	Tb927.3.4390_for	GGGGACAAGTTTGTACAAAAAAGCAGGCTATGTTTGCTAAAACCCGGCTCA	Tb927.3.4390_rev	GGGGACCACTTTGTACAAGAAAGCTGGGTCTAATATGCTCTGCCTCGGTCAAG
4_known	P18	Tb927.11.2330_for	GGGGACAAGTTTGTACAAAAAAGCAGGCTATGAATGTCCTTGAAGATCTTGAAGCTC	Tb927.11.2330_rev	GGGGACCACTTTGTACAAGAAAGCTGGGTTTAGTAAAAGGGGAAGTTTCTGTCTACTTC
4_known	P19	Tb927.8.4090_for	GGGGACAAGTTTGTACAAAAAAGCAGGCTATGCATCGCATCACCGTACG	Tb927.8.4090_rev	GGGGACCACTTTGTACAAGAAAGCTGGGTTTAACCGGTGTCGTTGGTCGA
4_known	P20	Tb927.6.4370_for	GGGGACAAGTTTGTACAAAAAAGCAGGCTATGGCCTTTGTGTTACCCCAGA	Tb927.6.4370_rev	GGGGACCACTTTGTACAAGAAAGCTGGGTCTAACCGTCTTCCTCACCATCATCA
4_known	P22	Tb927.10.12810_for	GGGGACAAGTTTGTACAAAAAAGCAGGCTATGGTATCCCACTACCTGAGTGAC	Tb927.10.12810_rev	GGGGACCACTTTGTACAAGAAAGCTGGGTCTACGACCCCTCCATAACAGTTGA
4_known	P23	Tb927.11.7240_for	GGGGACAAGTTTGTACAAAAAAGCAGGCTATGCAAGCCGATGCAAAGGG	Tb927.11.7240_rev	GGGGACCACTTTGTACAAGAAAGCTGGGTCTACCTCGTCATGTCGGAGACG
4_known	P24	Tb927.11.16410_for	GGGGACAAGTTTGTACAAAAAAGCAGGCTATGGAATCCGTCTCATGGAAATTGTATG	Tb927.11.16410_rev	GGGGACCACTTTGTACAAGAAAGCTGGGTTCACTTCTTGCGCTTCTTCTTATCC
5_hypothetical	A1	Tb927.10.4070_for	GGGGACAAGTTTGTACAAAAAAGCAGGCTATGGACGTGAGAGATCAGTTGATGC	Tb927.10.4070_rev	GGGGACCACTTTGTACAAGAAAGCTGGGTTTACCCGCCAGCAACCGA
5_hypothetical	A3	Tb927.3.5690_for	GGGGACAAGTTTGTACAAAAAAGCAGGCTATGGAGAACAAATATTTAGTGTGGTGCA	Tb927.3.5690_rev	GGGGACCACTTTGTACAAGAAAGCTGGGTTTAAAGCAACAATAACGAAAGTGTTAAAAG
5_hypothetical	A4	Tb927.8.1190_for	GGGGACAAGTTTGTACAAAAAAGCAGGCTATGTTTCACGGTTTTCCGCAGG	Tb927.8.1190_rev	GGGGACCACTTTGTACAAGAAAGCTGGGTCTACAGGTCACGTAGGAGTGATGA
5_hypothetical	A5	Tb927.10.13710_for	GGGGACAAGTTTGTACAAAAAAGCAGGCTATGGCGCGTATAGGTGAAGGA	Tb927.10.13710_rev	GGGGACCACTTTGTACAAGAAAGCTGGGTTCACAGATATTCCAACGAGTAACCGA
5_hypothetical	A6	Tb927.7.4740_for	GGGGACAAGTTTGTACAAAAAAGCAGGCTATGAGTGATGGCGTTCCTCCA	Tb927.7.4740_rev	GGGGACCACTTTGTACAAGAAAGCTGGGTTTATATGGGGGCGAGAAACCGA
5_hypothetical	A7	Tb927.1.4680_for	GGGGACAAGTTTGTACAAAAAAGCAGGCTATGTCAACAGAGGAACTCGTGC	Tb927.1.4680_rev	GGGGACCACTTTGTACAAGAAAGCTGGGTTTATCTGTGATTATTCGCATTAGCTTTGAT
5_hypothetical	A8	Tb927.4.1230_for	GGGGACAAGTTTGTACAAAAAAGCAGGCTATGGGAAGAAAGACTGGTGCGC	Tb927.4.1230_rev	GGGGACCACTTTGTACAAGAAAGCTGGGTTCAAGACAATGCAGTTAACAGAAGCA
5_hypothetical	A9	Tb927.11.3540_for	GGGGACAAGTTTGTACAAAAAAGCAGGCTATGAGCGTGCCTCGTTTGC	Tb927.11.3540_rev	GGGGACCACTTTGTACAAGAAAGCTGGGTTCAGTCGAGGGCAAATGCAAG

5_hypothetical	A10	Tb927.11.610_for	GGGGACAAGTTTGTACAAAAAAGCAGGCTATGAGTTTCGACTACGCCGTTC	Tb927.11.610_rev	GGGGACCACTTTGTACAAGAAAGCTGGGTCTACGCCAGTTTGAAGTTGTGTCG
5_hypothetical	A12	Tb927.11.5760_for	GGGGACAAGTTTGTACAAAAAAGCAGGCTATGTTCCCCCCGGTGTTCA	Tb927.11.5760_rev	GGGGACCACTTTGTACAAGAAAGCTGGGTTTATCGCTTTCGGCGTGGAG
5_hypothetical	A13	Tb927.10.7000_for	GGGGACAAGTTTGTACAAAAAAGCAGGCTATGAAGACAGTATACATTGTAGCGATGTG	Tb927.10.7000_rev	GGGGACCACTTTGTACAAGAAAGCTGGGTTCATTTCTCAACAACATCTACTGTGAAAAC
5_hypothetical	A14	Tb927.6.2990_for	GGGGACAAGTTTGTACAAAAAAGCAGGCTATGGCACTCATGTGGCGGT	Tb927.6.2990_rev	GGGGACCACTTTGTACAAGAAAGCTGGGTTCAGCATTGCGTAACGTAACGC
5_hypothetical	A15	Tb927.3.2120_for	GGGGACAAGTTTGTACAAAAAAGCAGGCTATGAGCGCTATTTATAGTCTTCTATTCGAC	Tb927.3.2120_rev	GGGGACCACTTTGTACAAGAAAGCTGGGTTCATCCAATAAACGTTTTCTCGCCT
5_hypothetical	A16	Tb927.11.4730_for	GGGGACAAGTTTGTACAAAAAAGCAGGCTATGGCAGGATTGCAGAGTGC	Tb927.11.4730_rev	GGGGACCACTTTGTACAAGAAAGCTGGGTTTACGTCAGCCCCGCTACG
5_hypothetical	A17	Tb927.10.11430_for	GGGGACAAGTTTGTACAAAAAAGCAGGCTATGTCATTGTTTGATGATGATGATGATGTG	Tb927.10.11430_rev	GGGGACCACTTTGTACAAGAAAGCTGGGTTTACACAAAACGGTCCATAGATCGTC
5_hypothetical	A18	Tb927.9.6730_for	GGGGACAAGTTTGTACAAAAAAGCAGGCTATGGATGATGAGACATTCGCGTG	Tb927.9.6730_rev	GGGGACCACTTTGTACAAGAAAGCTGGGTTTACGGGCGACCCCACTC
5_hypothetical	A19	Tb927.4.380_for	GGGGACAAGTTTGTACAAAAAAGCAGGCTATGTCGGAGGAACATGCGAATG	Tb927.4.380_rev	GGGGACCACTTTGTACAAGAAAGCTGGGTTCACTGCTTTACCGTAGCGAAAAG
5_hypothetical	A20	Tb927.6.1930_for	GGGGACAAGTTTGTACAAAAAAGCAGGCTATGAGCACTCGCAGTGGTGT	Tb927.6.1930_rev	GGGGACCACTTTGTACAAGAAAGCTGGGTTCAGCCACTTTTATCACTCGGC
5_hypothetical	A21	Tb927.4.2250_for	GGGGACAAGTTTGTACAAAAAAGCAGGCTATGATATTACGGCACTCTCGCCG	Tb927.4.2250_rev	GGGGACCACTTTGTACAAGAAAGCTGGGTTCAAAACCATCGTCCGATGCG
5_hypothetical	A22	Tb927.10.10820_for	GGGGACAAGTTTGTACAAAAAAGCAGGCTATGCCAAGTTCGGTGGCCC	Tb927.10.10820_rev	GGGGACCACTTTGTACAAGAAAGCTGGGTTCACTGCCCCGATTGAGGG
5_hypothetical	A23	Tb927.10.9730_for	GGGGACAAGTTTGTACAAAAAAGCAGGCTATGTGGCGTCCAGCTGAAGT	Tb927.10.9730_rev	GGGGACCACTTTGTACAAGAAAGCTGGGTCTACATTCGCACTGAATCGGTTGT
5_hypothetical	A24	Tb927.11.10570_for	GGGGACAAGTTTGTACAAAAAAGCAGGCTATGCTTCGTCTGTCGTCATGGA	Tb927.11.10570_rev	GGGGACCACTTTGTACAAGAAAGCTGGGTCTAGTTACCCGCTGCTGCCG
5_hypothetical	B1	Tb927.8.5640_for	GGGGACAAGTTTGTACAAAAAAGCAGGCTATGCGTCGCTTCCCACCC	Tb927.8.5640_rev	GGGGACCACTTTGTACAAGAAAGCTGGGTTTATTCAGGTGATCGTTGGAGGCA
5_hypothetical	B2	Tb927.8.6080_for	GGGGACAAGTTTGTACAAAAAAGCAGGCTATGAAGAGTGTATTTGTAGCATCTAGTATG	Tb927.8.6080_rev	GGGGACCACTTTGTACAAGAAAGCTGGGTTCATTTGCTGGGAAGTTTTGCTGC
5_hypothetical	B3	Tb927.5.3060_for	GGGGACAAGTTTGTACAAAAAAGCAGGCTATGGCGTCGGTTGTCAGC	Tb927.5.3060_rev	GGGGACCACTTTGTACAAGAAAGCTGGGTTCACACACTCAACCCCGTGT
5_hypothetical	B4	Tb927.10.11820_for	GGGGACAAGTTTGTACAAAAAAGCAGGCTATGACGGATCCTGTGCGCC	Tb927.10.11820_rev	GGGGACCACTTTGTACAAGAAAGCTGGGTTCAAATACGGTAAAACCCCGGAATAC
5_hypothetical	B5	Tb927.3.810_for	GGGGACAAGTTTGTACAAAAAAGCAGGCTATGTTGAATGCTGGTGGAGCC	Tb927.3.810_rev	GGGGACCACTTTGTACAAGAAAGCTGGGTTCAAGAACCGTAGCTGAGAAACAGC
5_hypothetical	B6	Tb927.11.13260.2_for	GGGGACAAGTTTGTACAAAAAAGCAGGCTATGCACCCTTATTTATTTATTTGTAAAGAA	Tb927.11.13260.2_rev	GGGGACCACTTTGTACAAGAAAGCTGGGTTCACATCTTCAAGAGACGAAACATCTG
5_hypothetical	B7	Tb927.4.1260_for	GGGGACAAGTTTGTACAAAAAAGCAGGCTATGAAGTTTGTGTGGTTAGACACGAA	Tb927.4.1260_rev	GGGGACCACTTTGTACAAGAAAGCTGGGTTTATATTCCTGTGGGCACACGC
5_hypothetical	B8	Tb927.5.3080_for	GGGGACAAGTTTGTACAAAAAAGCAGGCTATGTGGGGACCAAACCAGGAG	Tb927.5.3080_rev	GGGGACCACTTTGTACAAGAAAGCTGGGTTCACTGCACTTCGCAATCCTC
5_hypothetical	B9	Tb927.9.2130_for	GGGGACAAGTTTGTACAAAAAAGCAGGCTATGATGGGGGAAAAACAAGCACA	Tb927.9.2130_rev	GGGGACCACTTTGTACAAGAAAGCTGGGTTTACTTCTTTTCATCTCCCCCACTTAGT
5_hypothetical	B10	Tb927.7.850_for	GGGGACAAGTTTGTACAAAAAAGCAGGCTATGATGACATTGCGTTCCTCAACG	Tb927.7.850_rev	GGGGACCACTTTGTACAAGAAAGCTGGGTTCATAAACCCATACTCGCCAAGTTTG
5_hypothetical	B11	Tb927.11.13420_for	GGGGACAAGTTTGTACAAAAAAGCAGGCTATGGGCGGAGGCTGTTGC	Tb927.11.13420_rev	GGGGACCACTTTGTACAAGAAAGCTGGGTTTATTCTGACCGCTGTTGTCTCTGT
5_hypothetical	B12	Tb927.3.750_for	GGGGACAAGTTTGTACAAAAAAGCAGGCTATGCGGCTGCGGGGTCAA	Tb927.3.750_rev	GGGGACCACTTTGTACAAGAAAGCTGGGTTCATACGCCACGCTGCTTG
5_hypothetical	B13	Tb927.7.640_for	GGGGACAAGTTTGTACAAAAAAGCAGGCTATGGGTTGGTGGAGGAGAGGC	Tb927.7.640_rev	GGGGACCACTTTGTACAAGAAAGCTGGGTCTAAAGTAGAAATAGGAGCACAGCCAA
5_hypothetical	B14	Tb927.11.11050_for	GGGGACAAGTTTGTACAAAAAAGCAGGCTATGACGATTGTTGCGGACTGTG	Tb927.11.11050_rev	GGGGACCACTTTGTACAAGAAAGCTGGGTTCATGGCGGCGCTTTGGA
5_hypothetical	B15	Tb927.10.510_for	GGGGACAAGTTTGTACAAAAAAGCAGGCTATGCCTCTCATGTCTGCAGATGG	Tb927.10.510_rev	GGGGACCACTTTGTACAAGAAAGCTGGGTTCACTCTTCATCTTCTAAAAACTCTTCTTC
5_hypothetical	B16	Tb927.8.4570_for	GGGGACAAGTTTGTACAAAAAAGCAGGCTATGGGGCAAAAATCTTCAACGGC	Tb927.8.4570_rev	GGGGACCACTTTGTACAAGAAAGCTGGGTCTAAGCGGTCATTAGGCGTTCAA
5_hypothetical	B17	Tb927.10.11230_for	GGGGACAAGTTTGTACAAAAAAGCAGGCTATGGCAGGGACTGGTGCC	Tb927.10.11230_rev	GGGGACCACTTTGTACAAGAAAGCTGGGTTCACAACTTCTTTACTTCACCCACAG
5_hypothetical	B18	Tb927.2.3400_for	GGGGACAAGTTTGTACAAAAAAGCAGGCTATGAGTGCAACACCACAATTAGGAG	Tb927.2.3400_rev	GGGGACCACTTTGTACAAGAAAGCTGGGTTTACTCACCCTTCTTGCGCTCG
5_hypothetical	B19	Tb927.7.4840_for	GGGGACAAGTTTGTACAAAAAAGCAGGCTATGCCTCCGCGTCGCAAG	Tb927.7.4840_rev	GGGGACCACTTTGTACAAGAAAGCTGGGTTCACTGTGGTTGCACAAACGG
5_hypothetical	B20	Tb927.3.2460_for	GGGGACAAGTTTGTACAAAAAAGCAGGCTATGAAAGAAGTTAAAGGGGAAAATGAGTTT	Tb927.3.2460_rev	GGGGACCACTTTGTACAAGAAAGCTGGGTTCAACAATGCTTTTTGACACGAAGAG
5_hypothetical	B21	Tb927.3.2920_for	GGGGACAAGTTTGTACAAAAAAGCAGGCTATGAATCAAAAGCGTCGTTGCAC	Tb927.3.2920_rev	GGGGACCACTTTGTACAAGAAAGCTGGGTTTAGTTAACAACCCTAATAATGTCATTGGG
5_hypothetical	B22	Tb927.9.9710_for	GGGGACAAGTTTGTACAAAAAAGCAGGCTATGTTTTATCTGATGGTGGTTGCACT	Tb927.9.9710_rev	GGGGACCACTTTGTACAAGAAAGCTGGGTTCAGAAACCCTTGTACAACACTGATAC
5_hypothetical	B23	Tb927.11.12520_for	GGGGACAAGTTTGTACAAAAAAGCAGGCTATGACTGCTGGTGGAGGCA	Tb927.11.12520_rev	GGGGACCACTTTGTACAAGAAAGCTGGGTTCACTTGAACATGTGCTGATGCT
5_hypothetical	B24	Tb927.4.1180_for	GGGGACAAGTTTGTACAAAAAAGCAGGCTATGGCAAAGAAGGAAGTTGTCTGG	Tb927.4.1180_rev	GGGGACCACTTTGTACAAGAAAGCTGGGTCTACACTTCGTCCTCAACGACAC
5_hypothetical	C1	Tb927.5.960_for	GGGGACAAGTTTGTACAAAAAAGCAGGCTATGCGACCTCTGGACGTGT	Tb927.5.960_rev	GGGGACCACTTTGTACAAGAAAGCTGGGTTCAAACGCTTGATGCCATCGA
5_hypothetical	C2	Tb927.7.1850_for	GGGGACAAGTTTGTACAAAAAAGCAGGCTATGGGTTGCCAACGTATGGGG	Tb927.7.1850_rev	GGGGACCACTTTGTACAAGAAAGCTGGGTTTAGCGAAAATCTGGAACCCGC
5_hypothetical	C4	Tb927.9.10870_for	GGGGACAAGTTTGTACAAAAAAGCAGGCTATGAGTATCTTGGTGAACCGTCGT	Tb927.9.10870_rev	GGGGACCACTTTGTACAAGAAAGCTGGGTTCAACGACGCCTTGAAGAAGAC
5_hypothetical	C5	Tb927.11.15630_for	GGGGACAAGTTTGTACAAAAAAGCAGGCTATGCACTCGAGAAGGAAAGGGA	Tb927.11.15630_rev	GGGGACCACTTTGTACAAGAAAGCTGGGTTTATGAACTCGTGCTATCATCGTCATTT
5_hypothetical	C6	Tb927.3.3250_for	GGGGACAAGTTTGTACAAAAAAGCAGGCTATGCCGCACAGGAAACTACTG	Tb927.3.3250_rev	GGGGACCACTTTGTACAAGAAAGCTGGGTTCACCTCCCAAGCGGGCT
5_hypothetical	C7	Tb927.5.1350_for	GGGGACAAGTTTGTACAAAAAAGCAGGCTATGCTGTGGGCATTCAGTAGAGG	Tb927.5.1350_rev	GGGGACCACTTTGTACAAGAAAGCTGGGTTCATACCGCAATCTGTGAAGAGTTG
5_hypothetical	C8	Tb927.10.8690_for	GGGGACAAGTTTGTACAAAAAAGCAGGCTATGCCGCCACAGAGGCAA	Tb927.10.8690_rev	GGGGACCACTTTGTACAAGAAAGCTGGGTTTATTGTCCCTCTACCTCCACATTTTGC
5_hypothetical	C9	Tb927.9.13350_for	GGGGACAAGTTTGTACAAAAAAGCAGGCTATGGCAGCGAAGCGTAGC	Tb927.9.13350_rev	GGGGACCACTTTGTACAAGAAAGCTGGGTCTAGAACTTTTTAAGTGAACGCCGTTG
5_hypothetical	C10	Tb927.10.14500_for	GGGGACAAGTTTGTACAAAAAAGCAGGCTATGTCTGTCAAGTTGCCACACG	Tb927.10.14500_rev	GGGGACCACTTTGTACAAGAAAGCTGGGTCTAATTAGTTGTGGTTGGTTGATTCTTCAG
5_hypothetical	C11	Tb927.10.14310_for	GGGGACAAGTTTGTACAAAAAAGCAGGCTATGGTCATTTTCGATGATGTGGAAAAC	Tb927.10.14310_rev	GGGGACCACTTTGTACAAGAAAGCTGGGTTCAGTTTTTTGCATGGTTGTCGGG
5_hypothetical	C12	Tb927.7.6820_for	GGGGACAAGTTTGTACAAAAAAGCAGGCTATGGGCGGTGATGGCAACG	Tb927.7.6820_rev	GGGGACCACTTTGTACAAGAAAGCTGGGTTCAAGAGTGTATATCCACAGCTCCC
5_hypothetical	C13	Tb927.6.3070_for	GGGGACAAGTTTGTACAAAAAAGCAGGCTATGGACTCCATGGAAGAGTTTTATAACAAC	Tb927.6.3070_rev	GGGGACCACTTTGTACAAGAAAGCTGGGTTTAACGCTCAAGTGCTGACAAGG
5_hypothetical	C14	Tb927.10.11500_for	GGGGACAAGTTTGTACAAAAAAGCAGGCTATGTGGCGCCGGGGGATT	Tb927.10.11500_rev	GGGGACCACTTTGTACAAGAAAGCTGGGTTTATGTAAGTTTACTCAGCGCCTCAC
5_hypothetical	C15	Tb927.10.8650_for	GGGGACAAGTTTGTACAAAAAAGCAGGCTATGTCGGACCGCCTTAGAGG	Tb927.10.8650_rev	GGGGACCACTTTGTACAAGAAAGCTGGGTTCACTTAGACTGCTCCTGTGCG
5_hypothetical	C16	Tb927.9.1360_for	GGGGACAAGTTTGTACAAAAAAGCAGGCTATGAACTCAGCCGATTCTTCAGGT	Tb927.9.1360_rev	GGGGACCACTTTGTACAAGAAAGCTGGGTTTACGGTGTGGGAACCGAGT
5_hypothetical	C17	Tb927.8.7370_for	GGGGACAAGTTTGTACAAAAAAGCAGGCTATGGGTTCCGGGGTAGGAAGA	Tb927.8.7370_rev	GGGGACCACTTTGTACAAGAAAGCTGGGTTTACGGGTAGTAACCCACAGAGG
5_hypothetical	C18	Tb927.9.13290_for	GGGGACAAGTTTGTACAAAAAAGCAGGCTATGCCCGGGATTGATTACTCAAAAT	Tb927.9.13290_rev	GGGGACCACTTTGTACAAGAAAGCTGGGTTCACGTACTTCCCCCACGC
5_hypothetical	C19	Tb927.3.1680_for	GGGGACAAGTTTGTACAAAAAAGCAGGCTATGGAAGGTCTTGTGGTTCCCC	Tb927.3.1680_rev	GGGGACCACTTTGTACAAGAAAGCTGGGTTTAACGGGTATTATTTCCTCTGTTGGG
5_hypothetical	C20	Tb927.10.2200_for	GGGGACAAGTTTGTACAAAAAAGCAGGCTATGAGCCGTGTGCCGCAT	Tb927.10.2200_rev	GGGGACCACTTTGTACAAGAAAGCTGGGTTCATCCTCGCATAACGCTGTG
5_hypothetical	C21	Tb927.10.2810_for	GGGGACAAGTTTGTACAAAAAAGCAGGCTATGTGTAACGACGATGCTCGG	Tb927.10.2810_rev	GGGGACCACTTTGTACAAGAAAGCTGGGTCTACATGGCATCGGGGCATTC
5_hypothetical	C22	Tb927.11.8110_for	GGGGACAAGTTTGTACAAAAAAGCAGGCTATGGAGCCAAGGCGTTGGC	Tb927.11.8110_rev	GGGGACCACTTTGTACAAGAAAGCTGGGTTCAAACCAACATGAGCGACTTGT
5_hypothetical	C23	Tb927.9.6720_for	GGGGACAAGTTTGTACAAAAAAGCAGGCTATGCTGGCTGATGGATTTCGC	Tb927.9.6720_rev	GGGGACCACTTTGTACAAGAAAGCTGGGTTTACCTTTTGCATTCGACTCGTAAAATG
5_hypothetical	C24	Tb927.10.11000_for	GGGGACAAGTTTGTACAAAAAAGCAGGCTATGAACCCCGAAGCGCCG	Tb927.10.11000_rev	GGGGACCACTTTGTACAAGAAAGCTGGGTTCACCTCCTACGTGGCGG
5_hypothetical	D1	Tb927.8.3820_for	GGGGACAAGTTTGTACAAAAAAGCAGGCTATGAATCGATCTTCTGATGCTGAGTG	Tb927.8.3820_rev	GGGGACCACTTTGTACAAGAAAGCTGGGTTTACTTTCCCCTGTTCCTTTGATAATTAGA
5_hypothetical	D2	Tb927.9.13970_for	GGGGACAAGTTTGTACAAAAAAGCAGGCTATGCACTGCCGCCTGCTC	Tb927.9.13970_rev	GGGGACCACTTTGTACAAGAAAGCTGGGTTCATTGTCTAGGAATAAGGACATTACCAAA
5_hypothetical	D3	Tb927.7.5510_for	GGGGACAAGTTTGTACAAAAAAGCAGGCTATGATTTTGAGGGGAAGGGTTGGA	Tb927.7.5510_rev	GGGGACCACTTTGTACAAGAAAGCTGGGTTCACTTTTCCCTGTGAAGAAAAGGAAT
5_hypothetical	D4	Tb927.10.6480_for	GGGGACAAGTTTGTACAAAAAAGCAGGCTATGGTGGATAAACTAACCCCACCA	Tb927.10.6480_rev	GGGGACCACTTTGTACAAGAAAGCTGGGTTCAAGTGAGCCGTTGCCG
5_hypothetical	D5	Tb927.8.6480_for	GGGGACAAGTTTGTACAAAAAAGCAGGCTATGGGGTTCAAGTGTCTCCTCTC	Tb927.8.6480_rev	GGGGACCACTTTGTACAAGAAAGCTGGGTTCATGATTTGGTGAGGACTTTGCG
5_hypothetical	D6	Tb927.11.13680_for	GGGGACAAGTTTGTACAAAAAAGCAGGCTATGAGTAGGAAAAATGCGCCTCC	Tb927.11.13680_rev	GGGGACCACTTTGTACAAGAAAGCTGGGTTCAACCCTTTACCCGCTTCTTCT
5_hypothetical	D7	Tb927.11.10350_for	GGGGACAAGTTTGTACAAAAAAGCAGGCTATGGACGTGGGGTATTACGTTCC	Tb927.11.10350_rev	GGGGACCACTTTGTACAAGAAAGCTGGGTCTATAGAGAAGCGGAGGCTGAGAC
5_hypothetical	D8	Tb927.9.6960_for	GGGGACAAGTTTGTACAAAAAAGCAGGCTATGAACTCCCCTCACGACTCG	Tb927.9.6960_rev	GGGGACCACTTTGTACAAGAAAGCTGGGTCTATCTCGTGTGACGCGGGT
5_hypothetical	D9	Tb927.10.1800_for	GGGGACAAGTTTGTACAAAAAAGCAGGCTATGTCCTTCTCCAGTGACGAGAA	Tb927.10.1800_rev	GGGGACCACTTTGTACAAGAAAGCTGGGTTTAAGATAAACAAAAGCTGCCATTTATGAA
5_hypothetical	D10	Tb927.11.9800_for	GGGGACAAGTTTGTACAAAAAAGCAGGCTATGTTAAACATATTGTCGTACTCAAAGTTA	Tb927.11.9800_rev	GGGGACCACTTTGTACAAGAAAGCTGGGTTCAAGAACGGTCATCACCATCGT
5_hypothetical	D11	Tb927.4.2770_for	GGGGACAAGTTTGTACAAAAAAGCAGGCTATGGCGGTGACACCAATAGAAAAA	Tb927.4.2770_rev	GGGGACCACTTTGTACAAGAAAGCTGGGTTCAGGAGGCAGTGGCTTCC
5_hypothetical	D13	Tb927.10.9980_for	GGGGACAAGTTTGTACAAAAAAGCAGGCTATGAGCTCAAGGAACTGCAGC	Tb927.10.9980_rev	GGGGACCACTTTGTACAAGAAAGCTGGGTTCACCACATGGAAACATGGTAGGA
5_hypothetical	D14	Tb927.8.5480_for	GGGGACAAGTTTGTACAAAAAAGCAGGCTATGCGCAACGGTGTGTCG	Tb927.8.5480_rev	GGGGACCACTTTGTACAAGAAAGCTGGGTTTACAAAACATGTACATGAGGCCCAA
5_hypothetical	D15	Tb927.11.11710_for	GGGGACAAGTTTGTACAAAAAAGCAGGCTATGACAGTCGCAAAGAAGTGTATGG	Tb927.11.11710_rev	GGGGACCACTTTGTACAAGAAAGCTGGGTCTACTCCTGGAAAGTTGCCAGC
5_hypothetical	D16	Tb927.7.2160_for	GGGGACAAGTTTGTACAAAAAAGCAGGCTATGCAAGCATCTGAATCAGGAGATC	Tb927.7.2160_rev	GGGGACCACTTTGTACAAGAAAGCTGGGTTCACGCTGCAACCTTCTGC
5_hypothetical	D17	Tb927.10.4820_for	GGGGACAAGTTTGTACAAAAAAGCAGGCTATGGTGACGGAGGCCCTG	Tb927.10.4820_rev	GGGGACCACTTTGTACAAGAAAGCTGGGTTTACCGCTTGAGGAATTGCTGAAT
5_hypothetical	D18	Tb927.10.9640_for	GGGGACAAGTTTGTACAAAAAAGCAGGCTATGAGTGGAAACTCACAAACTGCTAG	Tb927.10.9640_rev	GGGGACCACTTTGTACAAGAAAGCTGGGTTTACTTCGGCTTTGTCCCAATTTTTG
5_hypothetical	D19	Tb927.11.14400_for	GGGGACAAGTTTGTACAAAAAAGCAGGCTATGAGTTACGAAAATTTCGCTGCAC	Tb927.11.14400_rev	GGGGACCACTTTGTACAAGAAAGCTGGGTTTAGACGAGCACAGAACCAACAATT
5_hypothetical	D20	Tb927.10.14220_for	GGGGACAAGTTTGTACAAAAAAGCAGGCTATGACGGAAGCAGTAGCGGC	Tb927.10.14220_rev	GGGGACCACTTTGTACAAGAAAGCTGGGTTTAGTCGTCAAGTTGCGTATGCTC
5_hypothetical	D21	Tb927.5.2390_for	GGGGACAAGTTTGTACAAAAAAGCAGGCTATGCGATCACCGAACGGATCT	Tb927.5.2390_rev	GGGGACCACTTTGTACAAGAAAGCTGGGTTCATAACATGAACTTGCCAAATTCACG
5_hypothetical	D22	Tb927.9.2780_for	GGGGACAAGTTTGTACAAAAAAGCAGGCTATGGATAACGAAGGCTGCAGC	Tb927.9.2780_rev	GGGGACCACTTTGTACAAGAAAGCTGGGTCTAGCAGGGTGCCAATATACCGA
5_hypothetical	D23	Tb927.10.1230_for	GGGGACAAGTTTGTACAAAAAAGCAGGCTATGGCGGAAGAAAGGTGCCT	Tb927.10.1230_rev	GGGGACCACTTTGTACAAGAAAGCTGGGTTTACACAGAAAGAGTGGTTGCCC
5_hypothetical	D24	Tb927.10.13840_for	GGGGACAAGTTTGTACAAAAAAGCAGGCTATGAGTCTTTTGTCTAATGCGGGTAAC	Tb927.10.13840_rev	GGGGACCACTTTGTACAAGAAAGCTGGGTTCAAACACGTGCTTTGCCACA
5_hypothetical	E1	Tb927.11.15410_for	GGGGACAAGTTTGTACAAAAAAGCAGGCTATGACAAGTATTACAGCGTATATGCGT	Tb927.11.15410_rev	GGGGACCACTTTGTACAAGAAAGCTGGGTTTATGCACCACTCCGTCCGA
5_hypothetical	E2	Tb927.7.310_for	GGGGACAAGTTTGTACAAAAAAGCAGGCTATGCGGTGGCCATCTGCC	Tb927.7.310_rev	GGGGACCACTTTGTACAAGAAAGCTGGGTTTATTTTTTTGTCACATGCGTCCGC
5_hypothetical	E3	Tb927.10.14700_for	GGGGACAAGTTTGTACAAAAAAGCAGGCTATGAACGCGAGAGGTAAAGGACG	Tb927.10.14700_rev	GGGGACCACTTTGTACAAGAAAGCTGGGTTTACCATACACCCTCACCGTTGC
5_hypothetical	E4	Tb927.11.13210_for	GGGGACAAGTTTGTACAAAAAAGCAGGCTATGCCCCTGGGAAGTACTAATCAG	Tb927.11.13210_rev	GGGGACCACTTTGTACAAGAAAGCTGGGTTCAACAACTGAGCCGACTGTTG
5_hypothetical	E5	Tb927.9.12470_for	GGGGACAAGTTTGTACAAAAAAGCAGGCTATGTTAAATATTTGCCAAAACTTGTCTTCC	Tb927.9.12470_rev	GGGGACCACTTTGTACAAGAAAGCTGGGTTCATGAAAGAAGAAAAATAATTTTTTCCAA
5_hypothetical	E6	Tb927.11.9490_for	GGGGACAAGTTTGTACAAAAAAGCAGGCTATGAGCGATGATGAATGCCCG	Tb927.11.9490_rev	GGGGACCACTTTGTACAAGAAAGCTGGGTCTACGCACAACCCCGGAGG
5_hypothetical	E7	Tb927.10.13170_for	GGGGACAAGTTTGTACAAAAAAGCAGGCTATGCCACGCCACCTTTCG	Tb927.10.13170_rev	GGGGACCACTTTGTACAAGAAAGCTGGGTTCAGCCGTGGGTGTGGTG
5_hypothetical	E8	Tb927.9.12750_for	GGGGACAAGTTTGTACAAAAAAGCAGGCTATGCTTCGCCGCACGCTG	Tb927.9.12750_rev	GGGGACCACTTTGTACAAGAAAGCTGGGTTTATGAGGTAATTTTCTTTGTTTCTTCCAC
5_hypothetical	E9	Tb927.9.10470_for	GGGGACAAGTTTGTACAAAAAAGCAGGCTATGTTATGTCGTACGTTTGTCAGACG	Tb927.9.10470_rev	GGGGACCACTTTGTACAAGAAAGCTGGGTTCACTTGTTCAGTGCACGTCC
5_hypothetical	E10	Tb927.9.9180_for	GGGGACAAGTTTGTACAAAAAAGCAGGCTATGGACAATAGGCCGGCGC	Tb927.9.9180_rev	GGGGACCACTTTGTACAAGAAAGCTGGGTTCAAGCCGTTGGAGGTTGATCT
5_hypothetical	E11	Tb927.4.3150_for	GGGGACAAGTTTGTACAAAAAAGCAGGCTATGTGGAACGCATTTAGCGGG	Tb927.4.3150_rev	GGGGACCACTTTGTACAAGAAAGCTGGGTCTACTTCGCATGTGGCATTAGAGG
5_hypothetical	E12	Tb927.9.10880_for	GGGGACAAGTTTGTACAAAAAAGCAGGCTATGGATGTCAAGACCCTCTTAGCT	Tb927.9.10880_rev	GGGGACCACTTTGTACAAGAAAGCTGGGTTTAAGCGATGTCATCATCACCCTCA
5_hypothetical	E13	Tb927.10.9370_for	GGGGACAAGTTTGTACAAAAAAGCAGGCTATGTCGCGCTTTCCTCACTC	Tb927.10.9370_rev	GGGGACCACTTTGTACAAGAAAGCTGGGTCTAAATTGGAATACAGGGAATGGGATTAAC
5_hypothetical	E14	Tb927.5.820_for	GGGGACAAGTTTGTACAAAAAAGCAGGCTATGCCGAGGAATTCTCAAACATTTCG	Tb927.5.820_rev	GGGGACCACTTTGTACAAGAAAGCTGGGTTCAGCCGACCGCCGGTGC
5_hypothetical	E15	Tb927.6.1840_for	GGGGACAAGTTTGTACAAAAAAGCAGGCTATGAGCCGTTTCAAACGCTGC	Tb927.6.1840_rev	GGGGACCACTTTGTACAAGAAAGCTGGGTTCAGCAGGATGCCAGCAGC
5_hypothetical	E16	Tb927.5.2480_for	GGGGACAAGTTTGTACAAAAAAGCAGGCTATGGCACAAACCTCTTTCACACC	Tb927.5.2480_rev	GGGGACCACTTTGTACAAGAAAGCTGGGTTCAAAGCACAAATACCATAATACCGTAAAC
5_hypothetical	E17	Tb927.9.11360_for	GGGGACAAGTTTGTACAAAAAAGCAGGCTATGGCGTCATTGGAAGATAAGGTTG	Tb927.9.11360_rev	GGGGACCACTTTGTACAAGAAAGCTGGGTTCACGATAACGACAACCTCACCC
5_hypothetical	E18	Tb927.7.770_for	GGGGACAAGTTTGTACAAAAAAGCAGGCTATGCTAATGCCTCAGTTTGAGTTTTTG	Tb927.7.770_rev	GGGGACCACTTTGTACAAGAAAGCTGGGTCTATAATCTCGGTTTCTTCTTTGGATGCA
5_hypothetical	E19	Tb927.8.5830_for	GGGGACAAGTTTGTACAAAAAAGCAGGCTATGGAGTTCGTTGCAGAGGCA	Tb927.8.5830_rev	GGGGACCACTTTGTACAAGAAAGCTGGGTTCACTCCGCTTGACCCCC
5_hypothetical	E20	Tb927.10.10430_for	GGGGACAAGTTTGTACAAAAAAGCAGGCTATGCTCGACATTACAGCCATCCC	Tb927.10.10430_rev	GGGGACCACTTTGTACAAGAAAGCTGGGTTTAACCGCTCTTCCCCAGGG
5_hypothetical	E21	Tb927.6.3610_for	GGGGACAAGTTTGTACAAAAAAGCAGGCTATGACGCTGCTTCACATGTTGG	Tb927.6.3610_rev	GGGGACCACTTTGTACAAGAAAGCTGGGTTCATTCGAAGTGGTCGTGCTCA
5_hypothetical	E22	Tb927.3.3440_for	GGGGACAAGTTTGTACAAAAAAGCAGGCTATGAACAATTCACTTAAGAAGGGGTCC	Tb927.3.3440_rev	GGGGACCACTTTGTACAAGAAAGCTGGGTCTATTCCACTTTGCATCTTTTTGAAACCT
5_hypothetical	E23	Tb927.6.1350_for	GGGGACAAGTTTGTACAAAAAAGCAGGCTATGTGGCGATCATTTATGCAAGTGG	Tb927.6.1350_rev	GGGGACCACTTTGTACAAGAAAGCTGGGTTCAGATAAAACAGAGGGATACTGATAGTAA
5_hypothetical	E24	Tb927.10.820_for	GGGGACAAGTTTGTACAAAAAAGCAGGCTATGGAGTCCCCTGTGGAAATGT	Tb927.10.820_rev	GGGGACCACTTTGTACAAGAAAGCTGGGTTTAATCTTCGTCTTCCACAATGCTATCTT
5_hypothetical	F1	Tb927.8.6610_for	GGGGACAAGTTTGTACAAAAAAGCAGGCTATGGATTCACTGCAGTCGGCG	Tb927.8.6610_rev	GGGGACCACTTTGTACAAGAAAGCTGGGTCTACACCCCGTCGACCGC
5_hypothetical	F2	Tb927.9.8990_for	GGGGACAAGTTTGTACAAAAAAGCAGGCTATGGCACAAGGTGGTGTGGT	Tb927.9.8990_rev	GGGGACCACTTTGTACAAGAAAGCTGGGTTTACATTTCCTGCCGCATTTTTTTTGT
5_hypothetical	F3	Tb927.10.1200_for	GGGGACAAGTTTGTACAAAAAAGCAGGCTATGCGAGAGGTATTGCTTGGTG	Tb927.10.1200_rev	GGGGACCACTTTGTACAAGAAAGCTGGGTCTACGTCCTTCCTGTCATAAAGGTG
5_hypothetical	F4	Tb927.8.4600_for	GGGGACAAGTTTGTACAAAAAAGCAGGCTATGTGTACAGGAGGTTCAACAAAGTT	Tb927.8.4600_rev	GGGGACCACTTTGTACAAGAAAGCTGGGTCTAATCGTTGCCTTTGGAGAGGC
5_hypothetical	F5	Tb927.11.11760_for	GGGGACAAGTTTGTACAAAAAAGCAGGCTATGGATTTTGGCACGGATGTAGC	Tb927.11.11760_rev	GGGGACCACTTTGTACAAGAAAGCTGGGTTCAGATGTCCATCACAACTCCATCA
5_hypothetical	F6	Tb927.7.2810_for	GGGGACAAGTTTGTACAAAAAAGCAGGCTATGCGCGACTCGGGTGTG	Tb927.7.2810_rev	GGGGACCACTTTGTACAAGAAAGCTGGGTTTAATGCTTGGTGGTGGTGTCG
5_hypothetical	F7	Tb927.10.5500_for	GGGGACAAGTTTGTACAAAAAAGCAGGCTATGCGGTCGGTAGGTGGTG	Tb927.10.5500_rev	GGGGACCACTTTGTACAAGAAAGCTGGGTCTAAACTGCATTCATTTCTGCTTCCG
5_hypothetical	F8	Tb927.9.15650_for	GGGGACAAGTTTGTACAAAAAAGCAGGCTATGCACTCATGTTGGATTGGTTCG	Tb927.9.15650_rev	GGGGACCACTTTGTACAAGAAAGCTGGGTTTATATGGTAAACGCAATTACGAAAAAAGC
5_hypothetical	F9	Tb927.10.5580_for	GGGGACAAGTTTGTACAAAAAAGCAGGCTATGGTCCTTTATGTAATGGGCCCC	Tb927.10.5580_rev	GGGGACCACTTTGTACAAGAAAGCTGGGTCTAGAGTATTTCAAGAAAATGTCTCTCCTT
5_hypothetical	F10	Tb927.9.7520_for	GGGGACAAGTTTGTACAAAAAAGCAGGCTATGAGACAAGGACGCGTGC	Tb927.9.7520_rev	GGGGACCACTTTGTACAAGAAAGCTGGGTTTACAACGACTCCCGAAGCTCT
5_hypothetical	F11	Tb927.3.4440_for	GGGGACAAGTTTGTACAAAAAAGCAGGCTATGATAGAGAAATGGAAGGATAACCCACT	Tb927.3.4440_rev	GGGGACCACTTTGTACAAGAAAGCTGGGTTTACCGTTTGGCCCTTCCTCT
5_hypothetical	F12	Tb927.11.4680_for	GGGGACAAGTTTGTACAAAAAAGCAGGCTATGAGGCCAGATTACATGGGTTTCA	Tb927.11.4680_rev	GGGGACCACTTTGTACAAGAAAGCTGGGTTTACGACTTCTCCCCTGAGGC
5_hypothetical	F13	Tb927.9.8700_for	GGGGACAAGTTTGTACAAAAAAGCAGGCTATGGAGGGGTCATTTAAGGACTTCG	Tb927.9.8700_rev	GGGGACCACTTTGTACAAGAAAGCTGGGTTCAATCATCTTTATCAATAGAAAGGGCGG
5_hypothetical	F14	Tb927.6.1390_for	GGGGACAAGTTTGTACAAAAAAGCAGGCTATGTGGAGAGTAATTATGAAAGTGGTCATG	Tb927.6.1390_rev	GGGGACCACTTTGTACAAGAAAGCTGGGTTCAGATAAAACAGAGGGATACTGACAGT
5_hypothetical	F15	Tb927.6.1370_for	GGGGACAAGTTTGTACAAAAAAGCAGGCTATGTGGAGAGTAATTATGAAAGTGGTCATG	Tb927.6.1370_rev	GGGGACCACTTTGTACAAGAAAGCTGGGTTCAGATAAAACAGAGGGATACTGATAGTAA
5_hypothetical	F16	Tb927.4.2860_for	GGGGACAAGTTTGTACAAAAAAGCAGGCTATGCGGCGTGGCGTCGGT	Tb927.4.2860_rev	GGGGACCACTTTGTACAAGAAAGCTGGGTTCAGTTACCAGAGGCGACACC
5_hypothetical	F17	Tb927.3.5060_for	GGGGACAAGTTTGTACAAAAAAGCAGGCTATGAGCGACTTCCCCAAACTGA	Tb927.3.5060_rev	GGGGACCACTTTGTACAAGAAAGCTGGGTCTATTTGGAGGCTTTAGGGCGTC
5_hypothetical	F18	Tb927.11.15300_for	GGGGACAAGTTTGTACAAAAAAGCAGGCTATGTGGACCGTTGTGCAGC	Tb927.11.15300_rev	GGGGACCACTTTGTACAAGAAAGCTGGGTCTACACCATATCAAGTCCCACACATG
5_hypothetical	F19	Tb927.4.1960_for	GGGGACAAGTTTGTACAAAAAAGCAGGCTATGGATGGCTTCATTAGCCTTGAAA	Tb927.4.1960_rev	GGGGACCACTTTGTACAAGAAAGCTGGGTTTAAGCCTCCTCCCCTGAGGC
5_hypothetical	F20	Tb927.10.12690_for	GGGGACAAGTTTGTACAAAAAAGCAGGCTATGTTGCGGTGGCGCATT	Tb927.10.12690_rev	GGGGACCACTTTGTACAAGAAAGCTGGGTTTATTTTTTTTTGCTTGGTGGTGGTTCA
5_hypothetical	F21	Tb927.5.3000_for	GGGGACAAGTTTGTACAAAAAAGCAGGCTATGTTGCGGTGCAGCTGG	Tb927.5.3000_rev	GGGGACCACTTTGTACAAGAAAGCTGGGTCTATGTGGACACACCCCCCG
5_hypothetical	F22	Tb927.7.3390_for	GGGGACAAGTTTGTACAAAAAAGCAGGCTATGGCATCGTTAGGCGAATTCTG	Tb927.7.3390_rev	GGGGACCACTTTGTACAAGAAAGCTGGGTTCACGAAGGCACGTCCGT

5_hypothetical	F23	Tb927.11.9340_for	GGGGACAAGTTTGTACAAAAAAGCAGGCTATGGAGTGGGTTCCGTGCA	Tb927.11.9340_rev	GGGGACCACTTTGTACAAGAAAGCTGGGTTTACCGTTGCAAAGTAACACCCG
5_hypothetical	F24	Tb927.10.6600_for	GGGGACAAGTTTGTACAAAAAAGCAGGCTATGTTACTTAGTGATGAGCACCTGGC	Tb927.10.6600_rev	GGGGACCACTTTGTACAAGAAAGCTGGGTCTAGGCATCCATTAAACCCACATGT
5_hypothetical	G1	Tb927.8.5310_for	GGGGACAAGTTTGTACAAAAAAGCAGGCTATGCGATTTGTTTCTCGACGACT	Tb927.8.5310_rev	GGGGACCACTTTGTACAAGAAAGCTGGGTTTAGTTGTGCGGCAGATGCAC
5_hypothetical	G2	Tb927.11.11010_for	GGGGACAAGTTTGTACAAAAAAGCAGGCTATGCCTTTACCGCCTGTTGAG	Tb927.11.11010_rev	GGGGACCACTTTGTACAAGAAAGCTGGGTTCACACACCATCTAAGTGGCTCA
5_hypothetical	G3	Tb927.3.2200_for	GGGGACAAGTTTGTACAAAAAAGCAGGCTATGGCCAATGCAGCGTTAGAG	Tb927.3.2200_rev	GGGGACCACTTTGTACAAGAAAGCTGGGTTCATGAAGTGCGAGAGCGC
5_hypothetical	G4	Tb927.5.1670_for	GGGGACAAGTTTGTACAAAAAAGCAGGCTATGTTAACTTCCACTGCTTATGGCA	Tb927.5.1670_rev	GGGGACCACTTTGTACAAGAAAGCTGGGTTCAACCCTCCTTTCCAATAAATTGCA
5_hypothetical	G5	Tb927.1.3400_for	GGGGACAAGTTTGTACAAAAAAGCAGGCTATGCGACATGTCGATGTGTTACA	Tb927.1.3400_rev	GGGGACCACTTTGTACAAGAAAGCTGGGTCTAAACACCTGCAATACGCTGCG
5_hypothetical	G6	Tb927.10.3330_for	GGGGACAAGTTTGTACAAAAAAGCAGGCTATGCTTCTTATAGTGTTCGTAGTCGGA	Tb927.10.3330_rev	GGGGACCACTTTGTACAAGAAAGCTGGGTTCACAACATGATTATCCTGTTACACTCTC
5_hypothetical	G7	Tb927.7.5720_for	GGGGACAAGTTTGTACAAAAAAGCAGGCTATGGTCCGCGCCCTACGC	Tb927.7.5720_rev	GGGGACCACTTTGTACAAGAAAGCTGGGTTCACTCTCTGTCCGCAGTAACC
5_hypothetical	G8	Tb927.11.11870_for	GGGGACAAGTTTGTACAAAAAAGCAGGCTATGGTAGCGAAGCTGCAGC	Tb927.11.11870_rev	GGGGACCACTTTGTACAAGAAAGCTGGGTTCAGTGGTGCGGTGGATTCC
5_hypothetical	G9	Tb927.10.9310_for	GGGGACAAGTTTGTACAAAAAAGCAGGCTATGATTTCATCGAGATTTCGAGGGG	Tb927.10.9310_rev	GGGGACCACTTTGTACAAGAAAGCTGGGTCTAGCAGTTGAGCGCCATTTTATCA
5_hypothetical	G10	Tb927.11.12740.1_for	GGGGACAAGTTTGTACAAAAAAGCAGGCTATGAGACGCACCTGCTGTGT	Tb927.11.12740.1_rev	GGGGACCACTTTGTACAAGAAAGCTGGGTTCACACACGACACAAACCCG
5_hypothetical	G11	Tb927.6.3420_for	GGGGACAAGTTTGTACAAAAAAGCAGGCTATGCGGCGAATTTTTAGCAAATCA	Tb927.6.3420_rev	GGGGACCACTTTGTACAAGAAAGCTGGGTTCAAACTTGCGTCAGCAGGAC
5_hypothetical	G12	Tb927.10.7380_for	GGGGACAAGTTTGTACAAAAAAGCAGGCTATGCTTGTTCCCGGGTTGTC	Tb927.10.7380_rev	GGGGACCACTTTGTACAAGAAAGCTGGGTTTACTTCCCTACAGCTGCCTGC
5_hypothetical	G13	Tb927.6.1630_for	GGGGACAAGTTTGTACAAAAAAGCAGGCTATGCAAACGTCCACTGGCAC	Tb927.6.1630_rev	GGGGACCACTTTGTACAAGAAAGCTGGGTTCAACCCCGCTTAAACTCGTAGG
5_hypothetical	G14	Tb927.11.8830_for	GGGGACAAGTTTGTACAAAAAAGCAGGCTATGACCTTACTCTCCCATGTAGTGG	Tb927.11.8830_rev	GGGGACCACTTTGTACAAGAAAGCTGGGTTTAAGGAGCGGTAATGCGAGC
5_hypothetical	G15	Tb927.3.3840_for	GGGGACAAGTTTGTACAAAAAAGCAGGCTATGGCCAAAATTAATGCAACTTATGGTT	Tb927.3.3840_rev	GGGGACCACTTTGTACAAGAAAGCTGGGTTCAATCGTCAAGCAAGGAAGCC
5_hypothetical	G16	Tb927.9.4760_for	GGGGACAAGTTTGTACAAAAAAGCAGGCTATGCTCTCCGGTGTGTATGGT	Tb927.9.4760_rev	GGGGACCACTTTGTACAAGAAAGCTGGGTTCATTGGAGGCTGCGGCT
5_hypothetical	G17	Tb927.8.3340_for	GGGGACAAGTTTGTACAAAAAAGCAGGCTATGGAGGGACACGGAATTGATGT	Tb927.8.3340_rev	GGGGACCACTTTGTACAAGAAAGCTGGGTTCACTCGGAACCCACGCA
5_hypothetical	G18	Tb927.7.1350_for	GGGGACAAGTTTGTACAAAAAAGCAGGCTATGTTTCGCTATCGTTGCCGC	Tb927.7.1350_rev	GGGGACCACTTTGTACAAGAAAGCTGGGTCTATAAGATGACAGACTCCGACGCA
5_hypothetical	G19	Tb927.4.990_for	GGGGACAAGTTTGTACAAAAAAGCAGGCTATGGACACCGCTGCTGAAATG	Tb927.4.990_rev	GGGGACCACTTTGTACAAGAAAGCTGGGTTCAAACAAACGATTTTCGAACGGC
5_hypothetical	G20	Tb927.3.3200_for	GGGGACAAGTTTGTACAAAAAAGCAGGCTATGACTCACTTGAGCGCGTTT	Tb927.3.3200_rev	GGGGACCACTTTGTACAAGAAAGCTGGGTTTAATAAAAACGGCGCAAACATGATTGA
5_hypothetical	G21	Tb927.10.12480_for	GGGGACAAGTTTGTACAAAAAAGCAGGCTATGCTCGATGCGGCCCCC	Tb927.10.12480_rev	GGGGACCACTTTGTACAAGAAAGCTGGGTCTAAGCCTTTGGTAGAAGAGGGTACA
5_hypothetical	G22	Tb927.6.840_for	GGGGACAAGTTTGTACAAAAAAGCAGGCTATGGTTCTCACATTGGTTGGGTC	Tb927.6.840_rev	GGGGACCACTTTGTACAAGAAAGCTGGGTCTACATGATGTTTATCTTCAGCGCCG
5_hypothetical	G23	Tb927.11.16360_for	GGGGACAAGTTTGTACAAAAAAGCAGGCTATGAAAAATTATACACCGGTAGAAATCGCA	Tb927.11.16360_rev	GGGGACCACTTTGTACAAGAAAGCTGGGTTTACCGTGGAGGAAAAGACCACC
5_hypothetical	G24	Tb927.7.3640_for	GGGGACAAGTTTGTACAAAAAAGCAGGCTATGTACGACCCCGCGGTAC	Tb927.7.3640_rev	GGGGACCACTTTGTACAAGAAAGCTGGGTTTAGGTTCCACATAAAGGCTCACCA
5_hypothetical	H1	Tb927.6.1310_for	GGGGACAAGTTTGTACAAAAAAGCAGGCTATGTGGCAAGCAATTGTACAAGTGG	Tb927.6.1310_rev	GGGGACCACTTTGTACAAGAAAGCTGGGTTCAGATAAAACAGAGGGATACTGATAGTAA
5_hypothetical	H2	Tb927.11.12500_for	GGGGACAAGTTTGTACAAAAAAGCAGGCTATGGAGAAGTACAGGAAGTTTGGTGA	Tb927.11.12500_rev	GGGGACCACTTTGTACAAGAAAGCTGGGTTTACTGAGCCATGAAAACCTCAACG
5_hypothetical	H3	Tb927.5.2120_for	GGGGACAAGTTTGTACAAAAAAGCAGGCTATGGAGAATCAGGCACCCCC	Tb927.5.2120_rev	GGGGACCACTTTGTACAAGAAAGCTGGGTCTACTTCATCATGGTATTTGTTCCAGCG
5_hypothetical	H4	Tb927.2.5220_for	GGGGACAAGTTTGTACAAAAAAGCAGGCTATGCGGTTGAGTGTTTCGGG	Tb927.2.5220_rev	GGGGACCACTTTGTACAAGAAAGCTGGGTTCAATGTGGGAGATTCTTATCCGAAGC
5_hypothetical	H5	Tb927.11.12690_for	GGGGACAAGTTTGTACAAAAAAGCAGGCTATGCAGGTTAGCGACGGCA	Tb927.11.12690_rev	GGGGACCACTTTGTACAAGAAAGCTGGGTTTATAAAGGCTTCACCGGTCGGT
5_hypothetical	H6	Tb927.2.4920_for	GGGGACAAGTTTGTACAAAAAAGCAGGCTATGAGCATGGACACCCGTATGG	Tb927.2.4920_rev	GGGGACCACTTTGTACAAGAAAGCTGGGTTTAGCCATAATATTGCAGGGCTAAGGG
5_hypothetical	H7	Tb927.8.1140_for	GGGGACAAGTTTGTACAAAAAAGCAGGCTATGTTTCTTCGCGTTCGCGG	Tb927.8.1140_rev	GGGGACCACTTTGTACAAGAAAGCTGGGTTTAGTGCCCACCACAGTGTCC
5_hypothetical	H8	Tb927.9.9660_for	GGGGACAAGTTTGTACAAAAAAGCAGGCTATGCTGAAGGAATGGCTTCGAG	Tb927.9.9660_rev	GGGGACCACTTTGTACAAGAAAGCTGGGTTTAGGCAGTGAATACCACACCATGT
5_hypothetical	H9	Tb927.5.1240_for	GGGGACAAGTTTGTACAAAAAAGCAGGCTATGCCACTAAATGCTCAACAATTGTTATT	Tb927.5.1240_rev	GGGGACCACTTTGTACAAGAAAGCTGGGTTCAAAGCAACTCCAACGGAATTCG
5_hypothetical	H10	Tb927.9.8500_for	GGGGACAAGTTTGTACAAAAAAGCAGGCTATGGTCCGAAATGAACATATACTTCTACAA	Tb927.9.8500_rev	GGGGACCACTTTGTACAAGAAAGCTGGGTCTACTCCACAGGTTGCAAAGACG
5_hypothetical	H11	Tb927.10.760_for	GGGGACAAGTTTGTACAAAAAAGCAGGCTATGCAGAAATGTTTAGTCATACGTCATGG	Tb927.10.760_rev	GGGGACCACTTTGTACAAGAAAGCTGGGTTCATATGGTGGATGGGGCATCTG
5_hypothetical	H12	Tb927.6.4420_for	GGGGACAAGTTTGTACAAAAAAGCAGGCTATGGATGATGAACGGACAACGG	Tb927.6.4420_rev	GGGGACCACTTTGTACAAGAAAGCTGGGTTCAGTCCGCCTGCCCAGA
5_hypothetical	H13	Tb927.6.680_for	GGGGACAAGTTTGTACAAAAAAGCAGGCTATGTGGCAGCTGTCAAGCTG	Tb927.6.680_rev	GGGGACCACTTTGTACAAGAAAGCTGGGTTCACTCATCGTTCCCGATTTCGT
5_hypothetical	H14	Tb927.3.5700_for	GGGGACAAGTTTGTACAAAAAAGCAGGCTATGAGGAAACAACATGTCATATACTACGTA	Tb927.3.5700_rev	GGGGACCACTTTGTACAAGAAAGCTGGGTCTAAAGCAACAATAAAGAAAGTGTTAAAAA
5_hypothetical	H15	Tb927.11.9230_for	GGGGACAAGTTTGTACAAAAAAGCAGGCTATGCTTCTGCAATTCAACTGGGA	Tb927.11.9230_rev	GGGGACCACTTTGTACAAGAAAGCTGGGTCTAAAGGGACAGTGGCACCAC
5_hypothetical	H16	Tb927.6.3440_for	GGGGACAAGTTTGTACAAAAAAGCAGGCTATGAGTAAGAGTGCAATTTACGGGTT	Tb927.6.3440_rev	GGGGACCACTTTGTACAAGAAAGCTGGGTCTACGCAAAGCTATGCGTCTGT
5_hypothetical	H17	Tb927.10.16020_for	GGGGACAAGTTTGTACAAAAAAGCAGGCTATGCTTCAACCAGAGTGTCAACC	Tb927.10.16020_rev	GGGGACCACTTTGTACAAGAAAGCTGGGTCTAAGCTATCGGACACGTCAGAATTAA
5_hypothetical	H20	Tb927.9.2060_for	GGGGACAAGTTTGTACAAAAAAGCAGGCTATGTTTTCGCTTGTCCGGGG	Tb927.9.2060_rev	GGGGACCACTTTGTACAAGAAAGCTGGGTTTATTTACCATCATCTGGTAGTGGCTGC
5_hypothetical	H21	Tb927.1.2730_for	GGGGACAAGTTTGTACAAAAAAGCAGGCTATGTTTAGATTGTCATTTGCCGCATTC	Tb927.1.2730_rev	GGGGACCACTTTGTACAAGAAAGCTGGGTCTATAGAGGCAGAAACCCAAAGCG
5_hypothetical	H23	Tb927.7.3200_for	GGGGACAAGTTTGTACAAAAAAGCAGGCTATGAAAAGCACGGTAACGATGGC	Tb927.7.3200_rev	GGGGACCACTTTGTACAAGAAAGCTGGGTCTAGCGCGGCAATTCCTTTCC
5_hypothetical	H24	Tb927.10.2500_for	GGGGACAAGTTTGTACAAAAAAGCAGGCTATGAAGCCCTTTCCCGCCT	Tb927.10.2500_rev	GGGGACCACTTTGTACAAGAAAGCTGGGTTCATAAGTGTCCATGCGGCG
5_hypothetical	I1	Tb927.7.4200_for	GGGGACAAGTTTGTACAAAAAAGCAGGCTATGTGGTTTACATCGCTTGCCC	Tb927.7.4200_rev	GGGGACCACTTTGTACAAGAAAGCTGGGTTTATGCACCACGCCGACCA
5_hypothetical	I2	Tb927.8.1380_for	GGGGACAAGTTTGTACAAAAAAGCAGGCTATGGAAATGGCGGCGCCA	Tb927.8.1380_rev	GGGGACCACTTTGTACAAGAAAGCTGGGTTTATTCAGGTCTACGTCCACCAAGA
5_hypothetical	I3	Tb927.11.16900_for	GGGGACAAGTTTGTACAAAAAAGCAGGCTATGTCCATCGCAGCGTCTAGT	Tb927.11.16900_rev	GGGGACCACTTTGTACAAGAAAGCTGGGTCTAATTCATTGGTGATAACGACGTTAAACG
5_hypothetical	I4	Tb927.10.8100_for	GGGGACAAGTTTGTACAAAAAAGCAGGCTATGATATGCAAGGTTTTGGAGCGT	Tb927.10.8100_rev	GGGGACCACTTTGTACAAGAAAGCTGGGTCTACGACGCAGACCACGTG
5_hypothetical	I5	Tb927.11.3830_for	GGGGACAAGTTTGTACAAAAAAGCAGGCTATGGGGCACTCGAAGGAAACT	Tb927.11.3830_rev	GGGGACCACTTTGTACAAGAAAGCTGGGTCTATATGGTGTTCTCTTCCGCAGAAAG
5_hypothetical	I6	Tb927.10.2940_for	GGGGACAAGTTTGTACAAAAAAGCAGGCTATGTCAACCGGAGCATTTACTGAG	Tb927.10.2940_rev	GGGGACCACTTTGTACAAGAAAGCTGGGTTTATCGCAAGTCTTCCTCACCGT
5_hypothetical	I7	Tb927.11.13600_for	GGGGACAAGTTTGTACAAAAAAGCAGGCTATGCGCAGTTGGGCTGCA	Tb927.11.13600_rev	GGGGACCACTTTGTACAAGAAAGCTGGGTTTAGCGCTGCCGCCGTCT
5_hypothetical	I8	Tb927.11.700_for	GGGGACAAGTTTGTACAAAAAAGCAGGCTATGGGGGATAAAGTTGAATATGGCAC	Tb927.11.700_rev	GGGGACCACTTTGTACAAGAAAGCTGGGTTCACTTAGCACCCTTAAACGGC
5_hypothetical	I9	Tb927.9.9630_for	GGGGACAAGTTTGTACAAAAAAGCAGGCTATGACAACCGCAACGGAATTGA	Tb927.9.9630_rev	GGGGACCACTTTGTACAAGAAAGCTGGGTTCAAAACGGTTGCTGAGAGAACTG
5_hypothetical	I10	Tb927.7.1330_for	GGGGACAAGTTTGTACAAAAAAGCAGGCTATGTTTTGTGACGCTGAAGATGGG	Tb927.7.1330_rev	GGGGACCACTTTGTACAAGAAAGCTGGGTTCAGCACCGCAAAAGCACC
5_hypothetical	I11	Tb927.3.840_for	GGGGACAAGTTTGTACAAAAAAGCAGGCTATGAATGTTTCTGTGGTGCTTCCA	Tb927.3.840_rev	GGGGACCACTTTGTACAAGAAAGCTGGGTTTAAGGGGTTTGCAACAACCAAGA
5_hypothetical	I12	Tb927.8.580_for	GGGGACAAGTTTGTACAAAAAAGCAGGCTATGAGTTCACACTCTTCTCTTGGCG	Tb927.8.580_rev	GGGGACCACTTTGTACAAGAAAGCTGGGTCTATGACGGATGCCTCGTTTTTGT
5_hypothetical	I13	Tb927.3.3910_for	GGGGACAAGTTTGTACAAAAAAGCAGGCTATGACCTCAGTGCCGGGTG	Tb927.3.3910_rev	GGGGACCACTTTGTACAAGAAAGCTGGGTTCACGACATCCACTCCTTGAGG
5_hypothetical	I14	Tb927.11.14420_for	GGGGACAAGTTTGTACAAAAAAGCAGGCTATGGGTGACTGCGACAGGT	Tb927.11.14420_rev	GGGGACCACTTTGTACAAGAAAGCTGGGTTTACTCGAAAAACAGGGTGGGACC
5_hypothetical	I15	Tb927.11.3050_for	GGGGACAAGTTTGTACAAAAAAGCAGGCTATGGAGTGCAAGACTAACTTGGC	Tb927.11.3050_rev	GGGGACCACTTTGTACAAGAAAGCTGGGTTCATCTAAATACCATACCAATATCGGACGA
5_hypothetical	I16	Tb927.7.510_for	GGGGACAAGTTTGTACAAAAAAGCAGGCTATGGGAAGCGGATGTTCAACC	Tb927.7.510_rev	GGGGACCACTTTGTACAAGAAAGCTGGGTCTACCCGCCACTGTTCAAAGC
5_hypothetical	I17	Tb927.11.2760_for	GGGGACAAGTTTGTACAAAAAAGCAGGCTATGGAAACGGAGGGGGTCG	Tb927.11.2760_rev	GGGGACCACTTTGTACAAGAAAGCTGGGTTCACTTACTTAGGTTCCGCACAAAC
5_hypothetical	I18	Tb927.3.900_for	GGGGACAAGTTTGTACAAAAAAGCAGGCTATGAAAACCTTACACCTGTTGCTTGG	Tb927.3.900_rev	GGGGACCACTTTGTACAAGAAAGCTGGGTTCACTTAAAATAAACAAAGCTATTCCCTTG
5_hypothetical	I19	Tb927.11.12670_for	GGGGACAAGTTTGTACAAAAAAGCAGGCTATGTTCGACTTGATGGCAGACG	Tb927.11.12670_rev	GGGGACCACTTTGTACAAGAAAGCTGGGTCTACGCATCGGGGTGTTTGC
5_hypothetical	I20	Tb927.11.12030_for	GGGGACAAGTTTGTACAAAAAAGCAGGCTATGGCTTGTGTAACCGGTGAC	Tb927.11.12030_rev	GGGGACCACTTTGTACAAGAAAGCTGGGTTTACTGCTGCCAGTTGATATCATCG
5_hypothetical	I21	Tb927.9.11220_for	GGGGACAAGTTTGTACAAAAAAGCAGGCTATGGCGTCGCGTTTGGCT	Tb927.9.11220_rev	GGGGACCACTTTGTACAAGAAAGCTGGGTTTACAGAATCTCCACATCCCCAACG
5_hypothetical	I23	Tb927.9.13540_for	GGGGACAAGTTTGTACAAAAAAGCAGGCTATGCGCCGCTTAGCTACCG	Tb927.9.13540_rev	GGGGACCACTTTGTACAAGAAAGCTGGGTCTAAAGAATCTCTGACAAAAAACGATTCCT
5_hypothetical	I24	Tb927.10.1600_for	GGGGACAAGTTTGTACAAAAAAGCAGGCTATGCGGCATACAACACAACATTACA	Tb927.10.1600_rev	GGGGACCACTTTGTACAAGAAAGCTGGGTTTACTTCAGAACTTGCCTGACGGA
5_hypothetical	J1	Tb927.11.15770_for	GGGGACAAGTTTGTACAAAAAAGCAGGCTATGCAGGTGCATCACAGTGTG	Tb927.11.15770_rev	GGGGACCACTTTGTACAAGAAAGCTGGGTTCAGCTGGCTCCCTTAATATACGG
5_hypothetical	J2	Tb927.5.3310_for	GGGGACAAGTTTGTACAAAAAAGCAGGCTATGACGGATCCACAGGAGGATAAC	Tb927.5.3310_rev	GGGGACCACTTTGTACAAGAAAGCTGGGTTCATGCAAGTGACAGGAAACCC
5_hypothetical	J3	Tb927.2.4800_for	GGGGACAAGTTTGTACAAAAAAGCAGGCTATGTTACGTCCGTCGTCCCT	Tb927.2.4800_rev	GGGGACCACTTTGTACAAGAAAGCTGGGTCTATTCATTATTCAGCAACCGATTCGC
5_hypothetical	J4	Tb927.11.13770_for	GGGGACAAGTTTGTACAAAAAAGCAGGCTATGTCCACAAGTGGCATAGTCCG	Tb927.11.13770_rev	GGGGACCACTTTGTACAAGAAAGCTGGGTCTAAAGAACCCATTGCTGGATGATAACA
5_hypothetical	J5	Tb927.8.2110_for	GGGGACAAGTTTGTACAAAAAAGCAGGCTATGCAGGGCGCGACAGTC	Tb927.8.2110_rev	GGGGACCACTTTGTACAAGAAAGCTGGGTTTAGGCCAACAACTCAGCAAGTG
5_hypothetical	J6	Tb927.10.1730_for	GGGGACAAGTTTGTACAAAAAAGCAGGCTATGTGGCGTTGCTCTACTCTACT	Tb927.10.1730_rev	GGGGACCACTTTGTACAAGAAAGCTGGGTTCAACTTTTCCCCACAGTTTTTTCCC
5_hypothetical	J7	Tb927.11.16300_for	GGGGACAAGTTTGTACAAAAAAGCAGGCTATGGAGGTGGTTGGGCGT	Tb927.11.16300_rev	GGGGACCACTTTGTACAAGAAAGCTGGGTTTATGCAACATTGCACGTCGTCA
5_hypothetical	J8	Tb927.10.4510_for	GGGGACAAGTTTGTACAAAAAAGCAGGCTATGACTGACCGAAATGGGGAACA	Tb927.10.4510_rev	GGGGACCACTTTGTACAAGAAAGCTGGGTTCACGGATAACTGGATAGCGCT
5_hypothetical	J9	Tb927.3.5190_for	GGGGACAAGTTTGTACAAAAAAGCAGGCTATGTTCGTTCGGAAATTTCAGGGA	Tb927.3.5190_rev	GGGGACCACTTTGTACAAGAAAGCTGGGTTTAGATACGAAAATTTGCTTTAGATGTGTT
5_hypothetical	J10	Tb927.3.3810_for	GGGGACAAGTTTGTACAAAAAAGCAGGCTATGTCTTCTGGTGTCCCTGTTTCA	Tb927.3.3810_rev	GGGGACCACTTTGTACAAGAAAGCTGGGTTCATTGCGCCACATGCACA
5_hypothetical	J11	Tb927.4.1610_for	GGGGACAAGTTTGTACAAAAAAGCAGGCTATGATTCCTCCGGCAAAATTAAATGATTTC	Tb927.4.1610_rev	GGGGACCACTTTGTACAAGAAAGCTGGGTTCAACCGGATTCCACGGAAAGA
5_hypothetical	J12	Tb927.8.2350_for	GGGGACAAGTTTGTACAAAAAAGCAGGCTATGGAGGCGGGCACATCC	Tb927.8.2350_rev	GGGGACCACTTTGTACAAGAAAGCTGGGTTTAGCTGCTCAACCTAAGCCAGA
5_hypothetical	J13	Tb927.3.4400_for	GGGGACAAGTTTGTACAAAAAAGCAGGCTATGGACTACACTGACATATATTTGCGG	Tb927.3.4400_rev	GGGGACCACTTTGTACAAGAAAGCTGGGTTCATACGTCCTTCACTGCCCC
5_hypothetical	J14	Tb927.10.1860_for	GGGGACAAGTTTGTACAAAAAAGCAGGCTATGACAGATAATCAAGGTAGCTATATCACT	Tb927.10.1860_rev	GGGGACCACTTTGTACAAGAAAGCTGGGTTCAGAGATCGATCAGCTTCCTTCC
5_hypothetical	J15	Tb927.11.1300_for	GGGGACAAGTTTGTACAAAAAAGCAGGCTATGAATGTTTTTGATTCCAACAGCATGC	Tb927.11.1300_rev	GGGGACCACTTTGTACAAGAAAGCTGGGTTCAAGAGCTATCTTTGTTCACAATGAAGT
5_hypothetical	J16	Tb927.10.4720_for	GGGGACAAGTTTGTACAAAAAAGCAGGCTATGAGCACATCAGCAACAAGCA	Tb927.10.4720_rev	GGGGACCACTTTGTACAAGAAAGCTGGGTTTAACAGAGCTTCAAGTACTCCAATGTG
5_hypothetical	J17	Tb927.7.5030_for	GGGGACAAGTTTGTACAAAAAAGCAGGCTATGATTCTATCCCTCGACATGAAAAATGC	Tb927.7.5030_rev	GGGGACCACTTTGTACAAGAAAGCTGGGTTCATTCCCGTGCGATCGCA
5_hypothetical	J18	Tb927.10.11670_for	GGGGACAAGTTTGTACAAAAAAGCAGGCTATGCTGCCTCGTTACACCCC	Tb927.10.11670_rev	GGGGACCACTTTGTACAAGAAAGCTGGGTTTAATTACAGTATCTGTCGTAGAGTCCCTC
5_hypothetical	J19	Tb927.7.1080_for	GGGGACAAGTTTGTACAAAAAAGCAGGCTATGATGCAACGTTGCGGCA	Tb927.7.1080_rev	GGGGACCACTTTGTACAAGAAAGCTGGGTTTAATCCATCTTCCTCCCGTATTTGTCT
5_hypothetical	J20	Tb927.10.9290_for	GGGGACAAGTTTGTACAAAAAAGCAGGCTATGACAGTTTGTCTTGTTCTACTAACTGG	Tb927.10.9290_rev	GGGGACCACTTTGTACAAGAAAGCTGGGTTCAAAGATCTACTAAGGCATGCTCGA
5_hypothetical	J21	Tb927.5.2000_for	GGGGACAAGTTTGTACAAAAAAGCAGGCTATGCGCCCCACCGGAGTA	Tb927.5.2000_rev	GGGGACCACTTTGTACAAGAAAGCTGGGTTCACCCCCTTCTTCCGTTCAT
5_hypothetical	J22	Tb927.11.13100_for	GGGGACAAGTTTGTACAAAAAAGCAGGCTATGCAGCAGAAGAAGTGCTACAC	Tb927.11.13100_rev	GGGGACCACTTTGTACAAGAAAGCTGGGTTCATGTTGCCGTTGAAACAGATTGC
5_hypothetical	J23	Tb927.5.4290_for	GGGGACAAGTTTGTACAAAAAAGCAGGCTATGACATCGAAGACCCCTTCGC	Tb927.5.4290_rev	GGGGACCACTTTGTACAAGAAAGCTGGGTTCACATGTATTCTGCCTCAGGTGT
5_hypothetical	J24	Tb927.10.13990_for	GGGGACAAGTTTGTACAAAAAAGCAGGCTATGCCACCTCTTACTCTTGAACAGG	Tb927.10.13990_rev	GGGGACCACTTTGTACAAGAAAGCTGGGTCTAAAAGCGCCAGCGGCG
5_hypothetical	K1	Tb927.9.3930_for	GGGGACAAGTTTGTACAAAAAAGCAGGCTATGGTCGTCAAACGCCGTG	Tb927.9.3930_rev	GGGGACCACTTTGTACAAGAAAGCTGGGTTCAAGGCGACAAGAACCCGG
5_hypothetical	K2	Tb927.3.3500_for	GGGGACAAGTTTGTACAAAAAAGCAGGCTATGTCGAGCTGTGAGCGACT	Tb927.3.3500_rev	GGGGACCACTTTGTACAAGAAAGCTGGGTTTAGTTCATGAGTGAGAAGCCGTTG
5_hypothetical	K3	Tb927.6.4160_for	GGGGACAAGTTTGTACAAAAAAGCAGGCTATGAGTACTGTGTTTCCCCTTCTTCG	Tb927.6.4160_rev	GGGGACCACTTTGTACAAGAAAGCTGGGTCTACTTCAAAAGGCAGTGCGACG
5_hypothetical	K4	Tb927.9.9090_for	GGGGACAAGTTTGTACAAAAAAGCAGGCTATGGGAAGCCAGATTCCGCA	Tb927.9.9090_rev	GGGGACCACTTTGTACAAGAAAGCTGGGTTTACACGTGTAGTAGCTGCGC
5_hypothetical	K5	Tb927.8.1350_for	GGGGACAAGTTTGTACAAAAAAGCAGGCTATGCAGAAACTGTGGCACGAC	Tb927.8.1350_rev	GGGGACCACTTTGTACAAGAAAGCTGGGTTTAATCACGACCGTTGGGTCTACT
5_hypothetical	K6	Tb927.11.3100_for	GGGGACAAGTTTGTACAAAAAAGCAGGCTATGACCGCACTGTCAAATGGG	Tb927.11.3100_rev	GGGGACCACTTTGTACAAGAAAGCTGGGTCTAGCGGGAGGAAAACCGC
5_hypothetical	K8	Tb927.9.6920_for	GGGGACAAGTTTGTACAAAAAAGCAGGCTATGACACCAACAGTAACCCCTCT	Tb927.9.6920_rev	GGGGACCACTTTGTACAAGAAAGCTGGGTCTAGCGCTTAGCGTTCCACTG
5_hypothetical	K9	Tb927.11.10600_for	GGGGACAAGTTTGTACAAAAAAGCAGGCTATGCTTTGTCACAATCGACTGCT	Tb927.11.10600_rev	GGGGACCACTTTGTACAAGAAAGCTGGGTTTAGCGAACGAATTGAGGAGGAGG
5_hypothetical	K10	Tb927.7.870_for	GGGGACAAGTTTGTACAAAAAAGCAGGCTATGCTCCTTCCGTTTCCACGG	Tb927.7.870_rev	GGGGACCACTTTGTACAAGAAAGCTGGGTTCATTCCAAGGCACGGCAGG
5_hypothetical	K11	Tb927.1.3550_for	GGGGACAAGTTTGTACAAAAAAGCAGGCTATGGGTGTTGGTTTTCAGAGTAAAAAAC	Tb927.1.3550_rev	GGGGACCACTTTGTACAAGAAAGCTGGGTCTAACAATGCGCGTCAAGCCC
5_hypothetical	K13	Tb927.7.5550_for	GGGGACAAGTTTGTACAAAAAAGCAGGCTATGGTCGGTAAGTGTCCACGA	Tb927.7.5550_rev	GGGGACCACTTTGTACAAGAAAGCTGGGTTCACTGGGCACAGAAGTGCC
5_hypothetical	K14	Tb927.11.7520_for	GGGGACAAGTTTGTACAAAAAAGCAGGCTATGAACGGAGAGGAAGGGAAGG	Tb927.11.7520_rev	GGGGACCACTTTGTACAAGAAAGCTGGGTTCAGTTGGGGGGACGGAGG
5_hypothetical	K15	Tb927.4.3100_for	GGGGACAAGTTTGTACAAAAAAGCAGGCTATGCCGTTATGTGTTCCTCTTCTTC	Tb927.4.3100_rev	GGGGACCACTTTGTACAAGAAAGCTGGGTTTAAATTGCAATGGAAACCCGCTTAC
5_hypothetical	K16	Tb927.8.3720_for	GGGGACAAGTTTGTACAAAAAAGCAGGCTATGAAAGCATGCTGTGGCGG	Tb927.8.3720_rev	GGGGACCACTTTGTACAAGAAAGCTGGGTTCAGATAGTCAGTGGGTTTCGCC
5_hypothetical	K17	Tb927.11.6610_for	GGGGACAAGTTTGTACAAAAAAGCAGGCTATGAGTGAACAGAATAGGGATGCGG	Tb927.11.6610_rev	GGGGACCACTTTGTACAAGAAAGCTGGGTTTAATCCGCCGAGCGGTGT
5_hypothetical	K18	Tb927.10.550_for	GGGGACAAGTTTGTACAAAAAAGCAGGCTATGAACGTTTACGGCAAGGACC	Tb927.10.550_rev	GGGGACCACTTTGTACAAGAAAGCTGGGTCTATCTATGCGTCGAGTCTCCAATAATATC
5_hypothetical	K19	Tb927.3.2830_for	GGGGACAAGTTTGTACAAAAAAGCAGGCTATGGGGAAATCTAAAAAGATGCCGG	Tb927.3.2830_rev	GGGGACCACTTTGTACAAGAAAGCTGGGTTTAGTCCATATCAGCTCCGTAGCC
5_hypothetical	K20	Tb927.6.3940_for	GGGGACAAGTTTGTACAAAAAAGCAGGCTATGCCGGGACACGGAAAGG	Tb927.6.3940_rev	GGGGACCACTTTGTACAAGAAAGCTGGGTTTAAACGTTTCCAGCTGGCGC
5_hypothetical	K21	Tb927.10.9240_for	GGGGACAAGTTTGTACAAAAAAGCAGGCTATGGATGACAAACAGGCGTTCTT	Tb927.10.9240_rev	GGGGACCACTTTGTACAAGAAAGCTGGGTCTAGTTAAGCAGTTCATTTATGAGCGAAC
5_hypothetical	K22	Tb927.2.4760_for	GGGGACAAGTTTGTACAAAAAAGCAGGCTATGAGCATGGACACCCGTATGG	Tb927.2.4760_rev	GGGGACCACTTTGTACAAGAAAGCTGGGTCTAATACGATTGACCGGTCATCATTTGG
5_hypothetical	K23	Tb927.4.4340_for	GGGGACAAGTTTGTACAAAAAAGCAGGCTATGGTGCAACGAGAAGTATTTACTTTTG	Tb927.4.4340_rev	GGGGACCACTTTGTACAAGAAAGCTGGGTTCAAAAACTGTGATACCACACAACAAC
5_hypothetical	L1	Tb927.5.4140_for	GGGGACAAGTTTGTACAAAAAAGCAGGCTATGCTACCCTTTGTTGCCCTGC	Tb927.5.4140_rev	GGGGACCACTTTGTACAAGAAAGCTGGGTTCATATTTCAATTCGCCCAAGGAGC
5_hypothetical	L2	Tb927.10.7600_for	GGGGACAAGTTTGTACAAAAAAGCAGGCTATGCCGGACGGAGCTATTGG	Tb927.10.7600_rev	GGGGACCACTTTGTACAAGAAAGCTGGGTTTAACGAGTAGAACGCGAGCCA
5_hypothetical	L3	Tb927.8.2800_for	GGGGACAAGTTTGTACAAAAAAGCAGGCTATGGGTTGTCTCCCTCCCG	Tb927.8.2800_rev	GGGGACCACTTTGTACAAGAAAGCTGGGTTCACGAACTCAAAACTGAGCGAA
5_hypothetical	L4	Tb927.10.7970_for	GGGGACAAGTTTGTACAAAAAAGCAGGCTATGGCCATACATGTCGCTCGT	Tb927.10.7970_rev	GGGGACCACTTTGTACAAGAAAGCTGGGTCTACTGATTACGCACGATAGCTTTGA
5_hypothetical	L5	Tb927.9.7260_for	GGGGACAAGTTTGTACAAAAAAGCAGGCTATGCGATCCGGTCGCAAAC	Tb927.9.7260_rev	GGGGACCACTTTGTACAAGAAAGCTGGGTTTACGCCTCCTCTTCGGATGG
5_hypothetical	L6	Tb927.7.1360_for	GGGGACAAGTTTGTACAAAAAAGCAGGCTATGCCTGAGCAGTTGGGCG	Tb927.7.1360_rev	GGGGACCACTTTGTACAAGAAAGCTGGGTCTACATGCCGCCATCCTCTTG
5_hypothetical	L7	Tb927.10.8640_for	GGGGACAAGTTTGTACAAAAAAGCAGGCTATGTCGACCGAGGTTGTTCACA	Tb927.10.8640_rev	GGGGACCACTTTGTACAAGAAAGCTGGGTCTATCCACCTTTCTTTCGGGAACC
5_hypothetical	L8	Tb927.8.2120_for	GGGGACAAGTTTGTACAAAAAAGCAGGCTATGTGGGAAGAAACGTACGTACG	Tb927.8.2120_rev	GGGGACCACTTTGTACAAGAAAGCTGGGTCTACTCCCTTGCCACCATAGGAC
5_hypothetical	L9	Tb927.11.9910_for	GGGGACAAGTTTGTACAAAAAAGCAGGCTATGTCTACCGGTGAGAGCGC	Tb927.11.9910_rev	GGGGACCACTTTGTACAAGAAAGCTGGGTTTAAGTTGAGGCGAACATCTTGGC
5_hypothetical	L10	Tb927.7.1870_for	GGGGACAAGTTTGTACAAAAAAGCAGGCTATGAGTAGAGATGCGACACCCTAC	Tb927.7.1870_rev	GGGGACCACTTTGTACAAGAAAGCTGGGTTTACTCGGGGAACTTGGGAAGC
5_hypothetical	L11	Tb927.9.1650_for	GGGGACAAGTTTGTACAAAAAAGCAGGCTATGTGGGGATTGACAAGGAGATGC	Tb927.9.1650_rev	GGGGACCACTTTGTACAAGAAAGCTGGGTTCAACGAACAAAAGCAATATCACAATGAA
5_hypothetical	L13	Tb927.5.3130_for	GGGGACAAGTTTGTACAAAAAAGCAGGCTATGAAAAATTTTGTGAATGCAAACCCCA	Tb927.5.3130_rev	GGGGACCACTTTGTACAAGAAAGCTGGGTTTACGTGGGACCAAATAAGGGAAAG
5_hypothetical	L14	Tb927.2.5290_for	GGGGACAAGTTTGTACAAAAAAGCAGGCTATGAGCATGGACACCCGTATGG	Tb927.2.5290_rev	GGGGACCACTTTGTACAAGAAAGCTGGGTTTATTCACTGGTTCTCATTTGGTTATAATA
5_hypothetical	L15	Tb927.9.2320_for	GGGGACAAGTTTGTACAAAAAAGCAGGCTATGCTGGCGTTGTGCAAATGT	Tb927.9.2320_rev	GGGGACCACTTTGTACAAGAAAGCTGGGTTCAGCGGCGCCGCCACAC
5_hypothetical	L16	Tb927.9.7090_for	GGGGACAAGTTTGTACAAAAAAGCAGGCTATGTACTCTCCAAGTGAAGAAGCAGG	Tb927.9.7090_rev	GGGGACCACTTTGTACAAGAAAGCTGGGTCTATCGTGCGAAACGGCAGT

5_hypothetical	L17	Tb927.8.3280_for	GGGGACAAGTTTGTACAAAAAAGCAGGCTATGAAGGGAGGCGTGGGAAG	Tb927.8.3280_rev	GGGGACCACTTTGTACAAGAAAGCTGGGTTCATAAACGTCCACTAAGGTGCTGC
5_hypothetical	L18	Tb927.3.4260_for	GGGGACAAGTTTGTACAAAAAAGCAGGCTATGTTGCGTCGCTGTTTTACCC	Tb927.3.4260_rev	GGGGACCACTTTGTACAAGAAAGCTGGGTCTACCGCCTGCTATTGCGC
5_hypothetical	L19	Tb927.10.4060_for	GGGGACAAGTTTGTACAAAAAAGCAGGCTATGCAACAAGTTGGTGAACCATTTC	Tb927.10.4060_rev	GGGGACCACTTTGTACAAGAAAGCTGGGTCTAGGCCGCCTTCGGTCG
5_hypothetical	L20	Tb927.5.2050_for	GGGGACAAGTTTGTACAAAAAAGCAGGCTATGCTCCAATGGGTCGTGAAAG	Tb927.5.2050_rev	GGGGACCACTTTGTACAAGAAAGCTGGGTCTATCGAGTCCGCTCTCGCT
5_hypothetical	L21	Tb927.11.16820_for	GGGGACAAGTTTGTACAAAAAAGCAGGCTATGACATTTTTCGGGTTAATGCGGT	Tb927.11.16820_rev	GGGGACCACTTTGTACAAGAAAGCTGGGTTCATCCGCCAGGCCCTGC
5_hypothetical	L22	Tb927.8.3640_for	GGGGACAAGTTTGTACAAAAAAGCAGGCTATGATGGCTGCTCGTCGTGG	Tb927.8.3640_rev	GGGGACCACTTTGTACAAGAAAGCTGGGTTTAAACTATTCTCCGGTCACGCTTC
5_hypothetical	L23	Tb927.2.2860_for	GGGGACAAGTTTGTACAAAAAAGCAGGCTATGGTTCATTTTACTGTTTATTACGGCAAC	Tb927.2.2860_rev	GGGGACCACTTTGTACAAGAAAGCTGGGTCTAATTCTTTTTTGACCGCTTCCGTT
5_hypothetical	L24	Tb927.8.2690_for	GGGGACAAGTTTGTACAAAAAAGCAGGCTATGGACCCCTCTCAAGAGGGA	Tb927.8.2690_rev	GGGGACCACTTTGTACAAGAAAGCTGGGTTCACTCTTCCTCTTCACAAAGAGGA
5_hypothetical	M1	Tb927.10.12220_for	GGGGACAAGTTTGTACAAAAAAGCAGGCTATGAGAAAGGGAATATTTTCTCACATCATC	Tb927.10.12220_rev	GGGGACCACTTTGTACAAGAAAGCTGGGTTCAAGGGATCATAATCTGCCGTAGG
5_hypothetical	M2	Tb927.3.3030_for	GGGGACAAGTTTGTACAAAAAAGCAGGCTATGTCGACAGAAGATGTGTTTGTTGA	Tb927.3.3030_rev	GGGGACCACTTTGTACAAGAAAGCTGGGTTTAATGGGGAGATGTACGCCGG
5_hypothetical	M5	Tb927.1.4140_for	GGGGACAAGTTTGTACAAAAAAGCAGGCTATGCCAGCTGGTGGTGACA	Tb927.1.4140_rev	GGGGACCACTTTGTACAAGAAAGCTGGGTTTATGTCACGACATTCCACAAATCCT
5_hypothetical	M6	Tb927.7.4910_for	GGGGACAAGTTTGTACAAAAAAGCAGGCTATGTTCTCCACTACCACCGGTT	Tb927.7.4910_rev	GGGGACCACTTTGTACAAGAAAGCTGGGTTCAGTCCACCAGCACCTGG
5_hypothetical	M9	Tb927.8.6020_for	GGGGACAAGTTTGTACAAAAAAGCAGGCTATGGGTATGGGTCTCACCGG	Tb927.8.6020_rev	GGGGACCACTTTGTACAAGAAAGCTGGGTTTAGTTCTCGCGCACGGC
5_hypothetical	M10	Tb927.3.970_for	GGGGACAAGTTTGTACAAAAAAGCAGGCTATGTCGGCCATTAAACGCTCC	Tb927.3.970_rev	GGGGACCACTTTGTACAAGAAAGCTGGGTCTAGTAAAAGTCCTCCACCCCAAATTC
5_hypothetical	M11	Tb927.5.2930_for	GGGGACAAGTTTGTACAAAAAAGCAGGCTATGCGCCGTGTATCTTCCCC	Tb927.5.2930_rev	GGGGACCACTTTGTACAAGAAAGCTGGGTTTAGTGATGGGCCTTTTTCTTTTGTTCG
5_hypothetical	M12	Tb927.11.6940_for	GGGGACAAGTTTGTACAAAAAAGCAGGCTATGAGTGGCGCTCCCACG	Tb927.11.6940_rev	GGGGACCACTTTGTACAAGAAAGCTGGGTTCACTCCCAAGGGATAAGTCGTT
5_hypothetical	M13	Tb927.10.12160_for	GGGGACAAGTTTGTACAAAAAAGCAGGCTATGCGTGCAACTGCGGAC	Tb927.10.12160_rev	GGGGACCACTTTGTACAAGAAAGCTGGGTTCATTTTGAGTTAGAATGCGGGGAA
5_hypothetical	M14	Tb927.8.6620_for	GGGGACAAGTTTGTACAAAAAAGCAGGCTATGGCATTAGCACACGCCC	Tb927.8.6620_rev	GGGGACCACTTTGTACAAGAAAGCTGGGTCTAGGCACCAACAAGCACATTTACA
5_hypothetical	M15	Tb927.10.11410_for	GGGGACAAGTTTGTACAAAAAAGCAGGCTATGTTGCAGGGGGAAATGGC	Tb927.10.11410_rev	GGGGACCACTTTGTACAAGAAAGCTGGGTTCACTGCGCCGCGTTCGA
5_hypothetical	M16	Tb927.9.4250_for	GGGGACAAGTTTGTACAAAAAAGCAGGCTATGCTTTCGACGGTTTGTGGT	Tb927.9.4250_rev	GGGGACCACTTTGTACAAGAAAGCTGGGTTTATGATTTCAGTTTTACGCGTATGCC
5_hypothetical	M17	Tb927.11.5670_for	GGGGACAAGTTTGTACAAAAAAGCAGGCTATGAACAAATCCGCAAAAAAGAAGGG	Tb927.11.5670_rev	GGGGACCACTTTGTACAAGAAAGCTGGGTTTACCTTCGGCAACGCCG
5_hypothetical	M18	Tb927.11.13490_for	GGGGACAAGTTTGTACAAAAAAGCAGGCTATGACGGAGTATGGCCCAAGG	Tb927.11.13490_rev	GGGGACCACTTTGTACAAGAAAGCTGGGTCTAGAACAAGCCGCTTCTCCTAAG
5_hypothetical	M19	Tb927.9.13630_for	GGGGACAAGTTTGTACAAAAAAGCAGGCTATGCGGTCTATACCTTACGGGAG	Tb927.9.13630_rev	GGGGACCACTTTGTACAAGAAAGCTGGGTTTACTTCAGGTGTGGTAAGCGC
5_hypothetical	M20	Tb927.8.6530_for	GGGGACAAGTTTGTACAAAAAAGCAGGCTATGAACAGGGGGTCGGTGG	Tb927.8.6530_rev	GGGGACCACTTTGTACAAGAAAGCTGGGTTTACCTCTCCCTCCTGCATCG
5_hypothetical	M21	Tb927.11.14750_for	GGGGACAAGTTTGTACAAAAAAGCAGGCTATGTTCCACGATCTGACTAACGTG	Tb927.11.14750_rev	GGGGACCACTTTGTACAAGAAAGCTGGGTTTATTTGTACGTTGGAGGGCGG
5_hypothetical	M22	Tb927.10.3630_for	GGGGACAAGTTTGTACAAAAAAGCAGGCTATGGCGACACCGCGGACG	Tb927.10.3630_rev	GGGGACCACTTTGTACAAGAAAGCTGGGTTCACACCGATGTGGAGAGTTCC
5_hypothetical	M23	Tb927.5.420_for	GGGGACAAGTTTGTACAAAAAAGCAGGCTATGGGCGCAAGATTGCGT	Tb927.5.420_rev	GGGGACCACTTTGTACAAGAAAGCTGGGTCTAACAATCCTCTTCTTCCTCAGCG
5_hypothetical	M24	Tb927.8.6560_for	GGGGACAAGTTTGTACAAAAAAGCAGGCTATGAACACAGTGAAAGTTCTGTTACGG	Tb927.8.6560_rev	GGGGACCACTTTGTACAAGAAAGCTGGGTTCAACGCAAGAGGAGGGGC
5_hypothetical	N1	Tb927.10.430_for	GGGGACAAGTTTGTACAAAAAAGCAGGCTATGCGGGTAGCATCTTTTAGGGC	Tb927.10.430_rev	GGGGACCACTTTGTACAAGAAAGCTGGGTCTAATCTGTTGATGCGTCGTATGGG
5_hypothetical	N2	Tb927.7.3290_for	GGGGACAAGTTTGTACAAAAAAGCAGGCTATGCTGCTCTCTGTCCCCG	Tb927.7.3290_rev	GGGGACCACTTTGTACAAGAAAGCTGGGTTCAACACCCATCTCCACCGC
5_hypothetical	N3	Tb927.4.5150_for	GGGGACAAGTTTGTACAAAAAAGCAGGCTATGTTTCGGAAGACGTTGTTGTTTC	Tb927.4.5150_rev	GGGGACCACTTTGTACAAGAAAGCTGGGTTCATTTCTTTTTTACTTTCTTTAGTTTCGC
5_hypothetical	N4	Tb927.5.3980_for	GGGGACAAGTTTGTACAAAAAAGCAGGCTATGCTCTCTGTGAGTCCGCC	Tb927.5.3980_rev	GGGGACCACTTTGTACAAGAAAGCTGGGTCTACAAACGACCTGCCGCTC
5_hypothetical	N5	Tb927.10.8240_for	GGGGACAAGTTTGTACAAAAAAGCAGGCTATGGACGCTAAGCAAAGGGAATC	Tb927.10.8240_rev	GGGGACCACTTTGTACAAGAAAGCTGGGTCTACTTAGGTCCCAATGTTGAGGCC
5_hypothetical	N6	Tb927.8.7240_for	GGGGACAAGTTTGTACAAAAAAGCAGGCTATGCTCCGGCGGACGTTG	Tb927.8.7240_rev	GGGGACCACTTTGTACAAGAAAGCTGGGTTCATTTCTTTTTTACTTTCTTTAGTTTCGC
5_hypothetical	N7	Tb927.11.3690_for	GGGGACAAGTTTGTACAAAAAAGCAGGCTATGAAAACTTTAGCCAAGAAAACACTGC	Tb927.11.3690_rev	GGGGACCACTTTGTACAAGAAAGCTGGGTTTACAAATACTCCGTCAAAAACGGCA
5_hypothetical	N8	Tb927.11.2940_for	GGGGACAAGTTTGTACAAAAAAGCAGGCTATGGGCGATGACTGCGAGA	Tb927.11.2940_rev	GGGGACCACTTTGTACAAGAAAGCTGGGTTTAGTCGAGTTCATCACCGCTAGT
5_hypothetical	N9	Tb927.10.10710_for	GGGGACAAGTTTGTACAAAAAAGCAGGCTATGAACGAGGTTATGCGTGTGC	Tb927.10.10710_rev	GGGGACCACTTTGTACAAGAAAGCTGGGTCTAATAACCGTCATCCGTATCATTCCAA
5_hypothetical	N10	Tb927.11.14720_for	GGGGACAAGTTTGTACAAAAAAGCAGGCTATGATTGAGACACCTGATGCGTC	Tb927.11.14720_rev	GGGGACCACTTTGTACAAGAAAGCTGGGTTCACGAAGACGCCGCTTTC
5_hypothetical	N11	Tb927.6.3260_for	GGGGACAAGTTTGTACAAAAAAGCAGGCTATGGACGATGACCTCATCCTCAA	Tb927.6.3260_rev	GGGGACCACTTTGTACAAGAAAGCTGGGTTCAAGGTCGTAATAGCGGAGTATCTAA
5_hypothetical	N12	Tb927.11.15160_for	GGGGACAAGTTTGTACAAAAAAGCAGGCTATGTTTTATGGTACCAACGACGTCG	Tb927.11.15160_rev	GGGGACCACTTTGTACAAGAAAGCTGGGTTTACTCCTCCTGTTCTTCAAGTTCATATAC
5_hypothetical	N13	Tb927.7.2140_for	GGGGACAAGTTTGTACAAAAAAGCAGGCTATGTCCTTCTCAATGAACACAGTGAC	Tb927.7.2140_rev	GGGGACCACTTTGTACAAGAAAGCTGGGTTCACTGCCCTAAAGAAGTTGCG
5_hypothetical	N14	Tb927.9.3030_for	GGGGACAAGTTTGTACAAAAAAGCAGGCTATGTCGGTTTTTCCCCCTGGC	Tb927.9.3030_rev	GGGGACCACTTTGTACAAGAAAGCTGGGTTTAGCGCTTTTTACCTATTGGTGGT
5_hypothetical	N15	Tb927.6.2910_for	GGGGACAAGTTTGTACAAAAAAGCAGGCTATGGTCTCGCCACCAGCT	Tb927.6.2910_rev	GGGGACCACTTTGTACAAGAAAGCTGGGTCTAGGGTTTGTCTTCTATTATTAACGCAAA
5_hypothetical	N16	Tb927.3.2640_for	GGGGACAAGTTTGTACAAAAAAGCAGGCTATGTCCATCACCACAGCCGG	Tb927.3.2640_rev	GGGGACCACTTTGTACAAGAAAGCTGGGTCTACAAAACCTCTTCGTCCCCCA
5_hypothetical	N17	Tb927.11.5610_for	GGGGACAAGTTTGTACAAAAAAGCAGGCTATGAAAGGCGATGATTCTGTTCATGG	Tb927.11.5610_rev	GGGGACCACTTTGTACAAGAAAGCTGGGTCTAAAGCAGTCCTTGTGAAAGTACCG
5_hypothetical	N18	Tb927.2.4420_for	GGGGACAAGTTTGTACAAAAAAGCAGGCTATGCTTCCACCCATTTCCGTG	Tb927.2.4420_rev	GGGGACCACTTTGTACAAGAAAGCTGGGTTCATGTGGTTAGTGGTAGTGAAGGA
5_hypothetical	N19	Tb927.5.430_for	GGGGACAAGTTTGTACAAAAAAGCAGGCTATGGGCGCAAGATTGCGT	Tb927.5.430_rev	GGGGACCACTTTGTACAAGAAAGCTGGGTCTATATCATGAGTCGAGACCCTGACC
5_hypothetical	N20	Tb927.3.5130_for	GGGGACAAGTTTGTACAAAAAAGCAGGCTATGTCGCCCCACAACAAGC	Tb927.3.5130_rev	GGGGACCACTTTGTACAAGAAAGCTGGGTTTAGAGCAGCGTCATCACTTTCC
5_hypothetical	N21	Tb927.3.660_for	GGGGACAAGTTTGTACAAAAAAGCAGGCTATGAATGAAGCGGTGGTAAGCG	Tb927.3.660_rev	GGGGACCACTTTGTACAAGAAAGCTGGGTTTACTCGTCAGGTTTGTGGTTGTAC
5_hypothetical	N22	Tb927.7.2230_for	GGGGACAAGTTTGTACAAAAAAGCAGGCTATGACCACGCAAGATGAAAAGAACC	Tb927.7.2230_rev	GGGGACCACTTTGTACAAGAAAGCTGGGTTCAAACAGCCTTCCAGCAGC
5_hypothetical	N23	Tb927.7.6800_for	GGGGACAAGTTTGTACAAAAAAGCAGGCTATGCGGCTGGCGCGGACC	Tb927.7.6800_rev	GGGGACCACTTTGTACAAGAAAGCTGGGTTCACGCCTTTGATTTCTTTTTCTCCT
5_hypothetical	N24	Tb927.8.5820_for	GGGGACAAGTTTGTACAAAAAAGCAGGCTATGGAGCATGAGAGGGGGAGT	Tb927.8.5820_rev	GGGGACCACTTTGTACAAGAAAGCTGGGTCTAAGAGAGCGTCCAATCACCTACA
5_hypothetical	O1	Tb927.10.2850_for	GGGGACAAGTTTGTACAAAAAAGCAGGCTATGGAGCGCGTTACCCTCA	Tb927.10.2850_rev	GGGGACCACTTTGTACAAGAAAGCTGGGTTTACTGGGTCGTAGAAGGAATGCC
5_hypothetical	O2	Tb927.3.3830_for	GGGGACAAGTTTGTACAAAAAAGCAGGCTATGGGCATTATTAAACATGTGGCAGT	Tb927.3.3830_rev	GGGGACCACTTTGTACAAGAAAGCTGGGTTTAACGGGAAAGCCGTCGC
5_hypothetical	O3	Tb927.3.1710_for	GGGGACAAGTTTGTACAAAAAAGCAGGCTATGTACGCTCGTTTCCGGACT	Tb927.3.1710_rev	GGGGACCACTTTGTACAAGAAAGCTGGGTTCAAACATGCTTCACACTTTCCTCA
5_hypothetical	O4	Tb927.10.4580_for	GGGGACAAGTTTGTACAAAAAAGCAGGCTATGGTTGGGAAGGTTGAAGTAATCCC	Tb927.10.4580_rev	GGGGACCACTTTGTACAAGAAAGCTGGGTTTATGTGACCCACTGTCGCACG
5_hypothetical	O5	Tb927.9.9170_for	GGGGACAAGTTTGTACAAAAAAGCAGGCTATGGCGTTTTGGTCAAAGGCG	Tb927.9.9170_rev	GGGGACCACTTTGTACAAGAAAGCTGGGTTCAAGTTGGGCTCAGCGGC
5_hypothetical	O6	Tb927.11.8880_for	GGGGACAAGTTTGTACAAAAAAGCAGGCTATGGCGAGGATAATTGACGTATCCG	Tb927.11.8880_rev	GGGGACCACTTTGTACAAGAAAGCTGGGTTCATCCGAAGCGAATGTTAGCTATC
5_hypothetical	O7	Tb927.9.11510_for	GGGGACAAGTTTGTACAAAAAAGCAGGCTATGAGACGCCAACCGTTTCC	Tb927.9.11510_rev	GGGGACCACTTTGTACAAGAAAGCTGGGTTCAACTGGTTGCTGCTGTGAC
5_hypothetical	O8	Tb927.4.680_for	GGGGACAAGTTTGTACAAAAAAGCAGGCTATGCGGCGCATTCCAATTGC	Tb927.4.680_rev	GGGGACCACTTTGTACAAGAAAGCTGGGTCTACTTTAAATCGGAAACCAATGGCG
5_hypothetical	O9	Tb927.4.1900_for	GGGGACAAGTTTGTACAAAAAAGCAGGCTATGCCGCCAACACGTCCC	Tb927.4.1900_rev	GGGGACCACTTTGTACAAGAAAGCTGGGTCTAAAAACGGGGTCGCCGC
5_hypothetical	O10	Tb927.11.1640_for	GGGGACAAGTTTGTACAAAAAAGCAGGCTATGAGTGGACAGCGCAACTCT	Tb927.11.1640_rev	GGGGACCACTTTGTACAAGAAAGCTGGGTCTACGACAACCTGCGGTATAATACAA
5_hypothetical	O11	Tb927.4.330_for	GGGGACAAGTTTGTACAAAAAAGCAGGCTATGCCACGAACGTTCTATAGGAGG	Tb927.4.330_rev	GGGGACCACTTTGTACAAGAAAGCTGGGTCTAGAACTTACCCTCACTGTTTAGATCCC
5_hypothetical	O12	Tb927.9.1380_for	GGGGACAAGTTTGTACAAAAAAGCAGGCTATGTTATTCCGGAGGCTCAAGCG	Tb927.9.1380_rev	GGGGACCACTTTGTACAAGAAAGCTGGGTTCATCGAGGAATGTCACCCCTC
5_hypothetical	O13	Tb927.4.3540_for	GGGGACAAGTTTGTACAAAAAAGCAGGCTATGCACTCACGTGGTGTCTACA	Tb927.4.3540_rev	GGGGACCACTTTGTACAAGAAAGCTGGGTTCACTTCTGGAGCCCCTTTAGC
5_hypothetical	O14	Tb927.3.1560_for	GGGGACAAGTTTGTACAAAAAAGCAGGCTATGAAAAAGAATTGTAGCTTAATGTTGAGC	Tb927.3.1560_rev	GGGGACCACTTTGTACAAGAAAGCTGGGTCTATGAAAAGAACCCCCGAAGACAA
5_hypothetical	O15	Tb927.5.1960_for	GGGGACAAGTTTGTACAAAAAAGCAGGCTATGATCAATGAAGCAGCTGCTGAA	Tb927.5.1960_rev	GGGGACCACTTTGTACAAGAAAGCTGGGTTCATAGTTTTAATGTTCTCAACGCACGC
5_hypothetical	O16	Tb927.4.1780_for	GGGGACAAGTTTGTACAAAAAAGCAGGCTATGCATAAGCTGACTCAGCTTCTG	Tb927.4.1780_rev	GGGGACCACTTTGTACAAGAAAGCTGGGTTTACAATACAATTGGTCTGAAGACCCG
5_hypothetical	O17	Tb927.7.5530_for	GGGGACAAGTTTGTACAAAAAAGCAGGCTATGAATCAGAAGATTCTCCTAGCGGA	Tb927.7.5530_rev	GGGGACCACTTTGTACAAGAAAGCTGGGTTCATGTTTCGTCGCCCTCGT
5_hypothetical	O18	Tb927.10.13850_for	GGGGACAAGTTTGTACAAAAAAGCAGGCTATGCGAAAGTTTTTACGGCCTCA	Tb927.10.13850_rev	GGGGACCACTTTGTACAAGAAAGCTGGGTTCAAATAACCTCTTCTAGAAACGCAGAC
5_hypothetical	O19	Tb927.9.5330_for	GGGGACAAGTTTGTACAAAAAAGCAGGCTATGCAGCGCTGTAAGCTTCTG	Tb927.9.5330_rev	GGGGACCACTTTGTACAAGAAAGCTGGGTTCACGCTCCATCACTTGAGTAATCC
5_hypothetical	O20	Tb927.3.3530_for	GGGGACAAGTTTGTACAAAAAAGCAGGCTATGTTGAGGAGGATGGAAGATTGCT	Tb927.3.3530_rev	GGGGACCACTTTGTACAAGAAAGCTGGGTTTATATCGCAGGCCAGAGACCA
5_hypothetical	O21	Tb927.1.1820_for	GGGGACAAGTTTGTACAAAAAAGCAGGCTATGGATAATAGCACGGAACTTGGGC	Tb927.1.1820_rev	GGGGACCACTTTGTACAAGAAAGCTGGGTTTAGCACCCGTAATAATCCAATAAACCTT
5_hypothetical	O22	Tb927.7.3810_for	GGGGACAAGTTTGTACAAAAAAGCAGGCTATGTTCCGACTCGCGGGC	Tb927.7.3810_rev	GGGGACCACTTTGTACAAGAAAGCTGGGTTCACTCAAGTAGATTGAATTCATCGTCG
5_hypothetical	O23	Tb927.5.3770_for	GGGGACAAGTTTGTACAAAAAAGCAGGCTATGAGACGGTTTGCACCACAC	Tb927.5.3770_rev	GGGGACCACTTTGTACAAGAAAGCTGGGTCTAAACTTGAATCACAGAAACCCGGT
5_hypothetical	O24	Tb927.9.9140_for	GGGGACAAGTTTGTACAAAAAAGCAGGCTATGTGTGAGGTTGACACTACCAAAC	Tb927.9.9140_rev	GGGGACCACTTTGTACAAGAAAGCTGGGTTTACTCGATTTTATCTCGGGAGTGTTTC
5_hypothetical	P1	Tb927.11.9060_for	GGGGACAAGTTTGTACAAAAAAGCAGGCTATGGCATCGGCAGTGGTGG	Tb927.11.9060_rev	GGGGACCACTTTGTACAAGAAAGCTGGGTTCACTCACAAAGAAAACATGACCCC
5_hypothetical	P2	Tb927.9.13020_for	GGGGACAAGTTTGTACAAAAAAGCAGGCTATGAAGTTGGTCCGTCATATAGAAACC	Tb927.9.13020_rev	GGGGACCACTTTGTACAAGAAAGCTGGGTTTATTCACTCCGCAACACGAGAG
5_hypothetical	P4	Tb927.10.2320_for	GGGGACAAGTTTGTACAAAAAAGCAGGCTATGATGGCGCTTGAGCTGC	Tb927.10.2320_rev	GGGGACCACTTTGTACAAGAAAGCTGGGTTTACGACATGGAGAAAATGCTGAGAA
5_hypothetical	P5	Tb927.8.2730_for	GGGGACAAGTTTGTACAAAAAAGCAGGCTATGCTTGGTGCTGTTCCTCCT	Tb927.8.2730_rev	GGGGACCACTTTGTACAAGAAAGCTGGGTTCATCGCGACCGCCAAAAC
5_hypothetical	P6	Tb927.10.1740_for	GGGGACAAGTTTGTACAAAAAAGCAGGCTATGGATACTTTCCAGGAGAAACTGGA	Tb927.10.1740_rev	GGGGACCACTTTGTACAAGAAAGCTGGGTCTAACAGTCTCGATCTCGCTTCCT
5_hypothetical	P7	Tb927.5.4160_for	GGGGACAAGTTTGTACAAAAAAGCAGGCTATGAATTCTTCTCTTGTTTTTGATAAGGAT	Tb927.5.4160_rev	GGGGACCACTTTGTACAAGAAAGCTGGGTTCAAAGGAAACGGCCGATGAG
5_hypothetical	P8	Tb927.11.480_for	GGGGACAAGTTTGTACAAAAAAGCAGGCTATGCTGTCTCGTACTTTTTCCCG	Tb927.11.480_rev	GGGGACCACTTTGTACAAGAAAGCTGGGTTTACTTGCAGGACATTCCACCTGG
5_hypothetical	P10	Tb927.10.3530_for	GGGGACAAGTTTGTACAAAAAAGCAGGCTATGGATCCTTTGGACGATTGGGA	Tb927.10.3530_rev	GGGGACCACTTTGTACAAGAAAGCTGGGTTCAGCGGGATGAAGGGGTAC
5_hypothetical	P11	Tb927.4.2280_for	GGGGACAAGTTTGTACAAAAAAGCAGGCTATGTTGCCTCATTCGGTGCC	Tb927.4.2280_rev	GGGGACCACTTTGTACAAGAAAGCTGGGTTTAGACGGATCGTGTATCTTCACCG
5_hypothetical	P12	Tb927.10.7730_for	GGGGACAAGTTTGTACAAAAAAGCAGGCTATGTTGAGCGTACAGGGAGCA	Tb927.10.7730_rev	GGGGACCACTTTGTACAAGAAAGCTGGGTCTATAGGTCTTTCAAAAGTGTGGCCA
5_hypothetical	P13	Tb927.3.4730_for	GGGGACAAGTTTGTACAAAAAAGCAGGCTATGGGCCGCATCACTACCG	Tb927.3.4730_rev	GGGGACCACTTTGTACAAGAAAGCTGGGTTCACCCGGTTCCATCCTCATT
5_hypothetical	P15	Tb927.9.2250_for	GGGGACAAGTTTGTACAAAAAAGCAGGCTATGAACCATGGCAGTCGGGG	Tb927.9.2250_rev	GGGGACCACTTTGTACAAGAAAGCTGGGTTTATCGCTCCCTCACGTCTTCC
5_hypothetical	P16	Tb927.10.10690_for	GGGGACAAGTTTGTACAAAAAAGCAGGCTATGCAGTTTGTGTCTGATCTGGG	Tb927.10.10690_rev	GGGGACCACTTTGTACAAGAAAGCTGGGTCTAGTCCGAGTCGTCCCACTC
5_hypothetical	P17	Tb927.11.13820_for	GGGGACAAGTTTGTACAAAAAAGCAGGCTATGGATTTAGGGGAAGATTCGACACT	Tb927.11.13820_rev	GGGGACCACTTTGTACAAGAAAGCTGGGTTTAAATAGTACTGAACTGCTCCTTAGCCA
5_hypothetical	P18	Tb927.11.15870_for	GGGGACAAGTTTGTACAAAAAAGCAGGCTATGTCTGGGGAGATTCGATATGCT	Tb927.11.15870_rev	GGGGACCACTTTGTACAAGAAAGCTGGGTTTACGAAGGGCTCATAAATAACTTTACTAC
5_hypothetical	P19	Tb927.11.10380_for	GGGGACAAGTTTGTACAAAAAAGCAGGCTATGACTGAGCTATTAAGTGCAGGGG	Tb927.11.10380_rev	GGGGACCACTTTGTACAAGAAAGCTGGGTTCAGGGTTCTTCGTGCAAAAGC
5_hypothetical	P20	Tb927.11.6070_for	GGGGACAAGTTTGTACAAAAAAGCAGGCTATGTGGTCTGTCATTCGTAAACTTCAG	Tb927.11.6070_rev	GGGGACCACTTTGTACAAGAAAGCTGGGTTTAACACTTTCGTTCCAGCTGGC
5_hypothetical	P21	Tb927.11.5980_for	GGGGACAAGTTTGTACAAAAAAGCAGGCTATGTCTATTGCATGCGCACTTGA	Tb927.11.5980_rev	GGGGACCACTTTGTACAAGAAAGCTGGGTTTAGATGCGAGTTGTTCCGTTGG
5_hypothetical	P22	Tb927.4.2440_for	GGGGACAAGTTTGTACAAAAAAGCAGGCTATGTTTTCACAAACGCGCTGTTC	Tb927.4.2440_rev	GGGGACCACTTTGTACAAGAAAGCTGGGTTTACGAATCCGCTGGCACAC
5_hypothetical	P23	Tb927.6.5010_for	GGGGACAAGTTTGTACAAAAAAGCAGGCTATGGAACATTCGCGTCGGAAG	Tb927.6.5010_rev	GGGGACCACTTTGTACAAGAAAGCTGGGTTTAAACACTCTTCCCTCCTGGAACA
5_hypothetical	P24	Tb927.10.990_for	GGGGACAAGTTTGTACAAAAAAGCAGGCTATGGCAGCAGTGATGAAATTAGAAAAGT	Tb927.10.990_rev	GGGGACCACTTTGTACAAGAAAGCTGGGTTCATTGAGTCCATAAACACAGCGAG
5_known	A1	Tb927.11.2550_for	GGGGACAAGTTTGTACAAAAAAGCAGGCTATGTCCGTGGAGCAATGCTCC	Tb927.11.2550_rev	GGGGACCACTTTGTACAAGAAAGCTGGGTTCAAAGAGTGGGTCGGAAAACATC
5_known	A2	Tb927.11.6680_for	GGGGACAAGTTTGTACAAAAAAGCAGGCTATGGCTGGGGATGGATTCGT	Tb927.11.6680_rev	GGGGACCACTTTGTACAAGAAAGCTGGGTCTACTGTTTTTCATCCTCGCTCAGAG
5_known	A3	Tb927.7.3760_for	GGGGACAAGTTTGTACAAAAAAGCAGGCTATGGCCGGTAAGCTCAACAGT	Tb927.7.3760_rev	GGGGACCACTTTGTACAAGAAAGCTGGGTTTATCGCCAAAAGATTATGTAGCGCT
5_known	A4	Tb927.10.8360_for	GGGGACAAGTTTGTACAAAAAAGCAGGCTATGGACGTCGACAAATATAACTGTTCC	Tb927.10.8360_rev	GGGGACCACTTTGTACAAGAAAGCTGGGTCTACCCACTAAGACGGTAAACACTTAGT
5_known	A5	Tb927.1.720_for	GGGGACAAGTTTGTACAAAAAAGCAGGCTATGTCCACTGCACCGAATGC	Tb927.1.720_rev	GGGGACCACTTTGTACAAGAAAGCTGGGTTTACTCCTTATCATCCAATACTGCAACAC
5_known	A6	Tb927.2.5240_for	GGGGACAAGTTTGTACAAAAAAGCAGGCTATGTTTTGTTGCATTTCTAACTGTGTGC	Tb927.2.5240_rev	GGGGACCACTTTGTACAAGAAAGCTGGGTTCATGTTGATCTGTACTCCTTCACCA
5_known	A7	Tb927.10.2000_for	GGGGACAAGTTTGTACAAAAAAGCAGGCTATGACTGCTGGATATCCGGTTCT	Tb927.10.2000_rev	GGGGACCACTTTGTACAAGAAAGCTGGGTTTACAGTAAATTTTGAAGCGCCGAGA
5_known	A8	Tb927.11.4170_for	GGGGACAAGTTTGTACAAAAAAGCAGGCTATGATGGGGGACGATGATCGT	Tb927.11.4170_rev	GGGGACCACTTTGTACAAGAAAGCTGGGTTCAACTATTACGCTCTCCATAATTTAGTCG
5_known	A9	Tb927.4.2230_for	GGGGACAAGTTTGTACAAAAAAGCAGGCTATGATTTACGAACTTGCGCTACTTATATCC	Tb927.4.2230_rev	GGGGACCACTTTGTACAAGAAAGCTGGGTTCAGTCCGCCTTTTTTCCTCCC
5_known	A10	Tb927.7.4590_for	GGGGACAAGTTTGTACAAAAAAGCAGGCTATGTTTCAACTACGGTCGTGTGTT	Tb927.7.4590_rev	GGGGACCACTTTGTACAAGAAAGCTGGGTTTATCCTCCTTCATCGTACTGGATACTAG
5_known	A11	Tb927.11.16550_for	GGGGACAAGTTTGTACAAAAAAGCAGGCTATGAGTGAAGCGAAGGACTCTTTG	Tb927.11.16550_rev	GGGGACCACTTTGTACAAGAAAGCTGGGTTTACACATATGTAAGCAACAGCGGC
5_known	A12	Tb927.3.3540_for	GGGGACAAGTTTGTACAAAAAAGCAGGCTATGATGTCAACTGCCCCAACG	Tb927.3.3540_rev	GGGGACCACTTTGTACAAGAAAGCTGGGTTTATGAGCGACCGGTTTTGTTCA
5_known	A13	Tb927.9.12570_for	GGGGACAAGTTTGTACAAAAAAGCAGGCTATGAAGTACGTCGGATCCATTGACC	Tb927.9.12570_rev	GGGGACCACTTTGTACAAGAAAGCTGGGTCTACAACTTTGCCCACTTCGTCC
5_known	A16	Tb927.8.630_for	GGGGACAAGTTTGTACAAAAAAGCAGGCTATGCGCGCTGCCAGACTG	Tb927.8.630_rev	GGGGACCACTTTGTACAAGAAAGCTGGGTTTAATAACTTATGGGGTAGAAGACGTTTTT
5_known	A19	Tb927.10.16120_for	GGGGACAAGTTTGTACAAAAAAGCAGGCTATGGAAAACACCAACCTACGCAC	Tb927.10.16120_rev	GGGGACCACTTTGTACAAGAAAGCTGGGTTTAGAGCTTCGAGGCAAAGAGTTTC
5_known	A20	Tb927.10.350_for	GGGGACAAGTTTGTACAAAAAAGCAGGCTATGATTTCCGTCTTGTCGCCC	Tb927.10.350_rev	GGGGACCACTTTGTACAAGAAAGCTGGGTTCAAATAGACGTGTCTTTGATGCCA
5_known	A21	Tb927.11.13970_for	GGGGACAAGTTTGTACAAAAAAGCAGGCTATGGATTCAAAGGGACGCGGT	Tb927.11.13970_rev	GGGGACCACTTTGTACAAGAAAGCTGGGTTTACAACGTTGCCGATGTCGT
5_known	A22	Tb927.10.6290_for	GGGGACAAGTTTGTACAAAAAAGCAGGCTATGGTTAAGGAGAAGTTGGCGGG	Tb927.10.6290_rev	GGGGACCACTTTGTACAAGAAAGCTGGGTTCATGACTTCATCTGCTTCCGC
5_known	A23	Tb927.6.2450_for	GGGGACAAGTTTGTACAAAAAAGCAGGCTATGCCCCGTAGTGGTGATGC	Tb927.6.2450_rev	GGGGACCACTTTGTACAAGAAAGCTGGGTTTACTTCATGAAATCTTCATGAACATTATT
5_known	A24	Tb927.1.2670_for	GGGGACAAGTTTGTACAAAAAAGCAGGCTATGCCCAACAGGCAGATCATTC	Tb927.1.2670_rev	GGGGACCACTTTGTACAAGAAAGCTGGGTTTAATGCTGCTGCACTTGTTGGA
5_known	B1	Tb927.2.2440_for	GGGGACAAGTTTGTACAAAAAAGCAGGCTATGGAAAAGGATTTGGACTCACTTTGG	Tb927.2.2440_rev	GGGGACCACTTTGTACAAGAAAGCTGGGTTTATTCCTTCTTCGGCTCACTTTTCG
5_known	B2	Tb927.7.600_for	GGGGACAAGTTTGTACAAAAAAGCAGGCTATGTGGCGGATTTCTAATTTATGGCG	Tb927.7.600_rev	GGGGACCACTTTGTACAAGAAAGCTGGGTTTAGAGCCAGTCCTTTTCGGACT
5_known	B3	Tb927.11.4740_for	GGGGACAAGTTTGTACAAAAAAGCAGGCTATGGCGAAAAAGGGTAAAAAGAATTCG	Tb927.11.4740_rev	GGGGACCACTTTGTACAAGAAAGCTGGGTTCAGTGTGAAGGCAACATATCCCA
5_known	B4	Tb927.10.5150_for	GGGGACAAGTTTGTACAAAAAAGCAGGCTATGTATCGGCCAACTAACAGATGC	Tb927.10.5150_rev	GGGGACCACTTTGTACAAGAAAGCTGGGTTTAGATGGTTTCACGAGGGTGTGG
5_known	B5	Tb927.7.5820_for	GGGGACAAGTTTGTACAAAAAAGCAGGCTATGAATGGTCGTACACGGGTATTG	Tb927.7.5820_rev	GGGGACCACTTTGTACAAGAAAGCTGGGTTTATGCAAGGAGAAATGACTCCCGG
5_known	B6	Tb927.10.9900_for	GGGGACAAGTTTGTACAAAAAAGCAGGCTATGACAGTAAACGCAATTACAGGTTCT	Tb927.10.9900_rev	GGGGACCACTTTGTACAAGAAAGCTGGGTTCACACGCTCGTTAATTCATCGT
5_known	B8	Tb927.11.9510_for	GGGGACAAGTTTGTACAAAAAAGCAGGCTATGTCTGAAGCCATTGAGGGCG	Tb927.11.9510_rev	GGGGACCACTTTGTACAAGAAAGCTGGGTCTACTGAAAGACACTATGGGTGGGA
5_known	B9	Tb927.1.580_for	GGGGACAAGTTTGTACAAAAAAGCAGGCTATGGTGCATCCATACTTATGGGTCA	Tb927.1.580_rev	GGGGACCACTTTGTACAAGAAAGCTGGGTTTACTGCGAGGTGAGACTCGG
5_known	B10	Tb927.7.4830_for	GGGGACAAGTTTGTACAAAAAAGCAGGCTATGGAACACATAGGTGCTTTTCTACG	Tb927.7.4830_rev	GGGGACCACTTTGTACAAGAAAGCTGGGTCTATGAGGCTTGTGGCCCGA
5_known	B11	Tb927.5.3010_for	GGGGACAAGTTTGTACAAAAAAGCAGGCTATGCGGAGCGCGTTGCGT	Tb927.5.3010_rev	GGGGACCACTTTGTACAAGAAAGCTGGGTTTATGCTTGCGGTGTAGACCCA
5_known	B12	Tb927.5.4560_for	GGGGACAAGTTTGTACAAAAAAGCAGGCTATGGAAACCGCCGTGCCT	Tb927.5.4560_rev	GGGGACCACTTTGTACAAGAAAGCTGGGTCTAAAGTTCTACCCTCCCGTGGA
5_known	B13	Tb927.11.1980_for	GGGGACAAGTTTGTACAAAAAAGCAGGCTATGAGCAGCGCGGTAGATTCC	Tb927.11.1980_rev	GGGGACCACTTTGTACAAGAAAGCTGGGTTCACAGGGTCTTGGGCTTCA
5_known	B14	Tb927.10.14910_for	GGGGACAAGTTTGTACAAAAAAGCAGGCTATGAGTTCGGGTAATAGGGACCTTT	Tb927.10.14910_rev	GGGGACCACTTTGTACAAGAAAGCTGGGTCTACCGTGGAGCATAGAACTGGT

5_known	B15	Tb927.5.2850_for	GGGGACAAGTTTGTACAAAAAAGCAGGCTATGACTGAGAGTGCATACCTCAAATG	Tb927.5.2850_rev	GGGGACCACTTTGTACAAGAAAGCTGGGTTTAGTTGCCTTCGTCCCCATCA
5_known	B16	Tb927.11.3980_for	GGGGACAAGTTTGTACAAAAAAGCAGGCTATGTACCGACGCGCCGTT	Tb927.11.3980_rev	GGGGACCACTTTGTACAAGAAAGCTGGGTTTACGCTCCCAACTGACGGA
5_known	B17	Tb927.8.1420_for	GGGGACAAGTTTGTACAAAAAAGCAGGCTATGAGACGTTTCATTTCTGTTTCTAAGCA	Tb927.8.1420_rev	GGGGACCACTTTGTACAAGAAAGCTGGGTCTACTGTAAAGCATCTTCGCAAATATCG
5_known	B18	Tb927.3.1380_for	GGGGACAAGTTTGTACAAAAAAGCAGGCTATGCTGACTCGTTTCCGAAGTG	Tb927.3.1380_rev	GGGGACCACTTTGTACAAGAAAGCTGGGTTTAGCTACTGGCTTGAGCAACAC
5_known	B19	Tb927.8.7570_for	GGGGACAAGTTTGTACAAAAAAGCAGGCTATGACATACAATGAGGCGGCG	Tb927.8.7570_rev	GGGGACCACTTTGTACAAGAAAGCTGGGTTCACATCAAGAAGTCGATGCCAAG
5_known	B20	Tb927.10.10380_for	GGGGACAAGTTTGTACAAAAAAGCAGGCTATGAGCGAGTTCGAAGCACTG	Tb927.10.10380_rev	GGGGACCACTTTGTACAAGAAAGCTGGGTTTATTCCCCTGCCATGCTGGT
5_known	B21	Tb927.5.3750_for	GGGGACAAGTTTGTACAAAAAAGCAGGCTATGCCAAGTTCGTCGTTGTCG	Tb927.5.3750_rev	GGGGACCACTTTGTACAAGAAAGCTGGGTTTATTGGCGCACCCTCTTGC
5_known	B22	Tb927.10.7400_for	GGGGACAAGTTTGTACAAAAAAGCAGGCTATGACACCGCTTCAAGTATTGTTCA	Tb927.10.7400_rev	GGGGACCACTTTGTACAAGAAAGCTGGGTTCAGCTAGAGTCCCTCTGCCG
5_known	B23	Tb927.5.2650_for	GGGGACAAGTTTGTACAAAAAAGCAGGCTATGGGACAGGAGACGATGTCC	Tb927.5.2650_rev	GGGGACCACTTTGTACAAGAAAGCTGGGTCTACAGATGACTAATAGTGCTGTTAAGTAC
5_known	B24	Tb927.3.5460_for	GGGGACAAGTTTGTACAAAAAAGCAGGCTATGGAGGGCCGCAAGGGA	Tb927.3.5460_rev	GGGGACCACTTTGTACAAGAAAGCTGGGTTCAGGAGGAATGCGGTAGCA
5_known	C1	Tb927.11.11200_for	GGGGACAAGTTTGTACAAAAAAGCAGGCTATGGAACTTGAGGTGAGTGAGGAG	Tb927.11.11200_rev	GGGGACCACTTTGTACAAGAAAGCTGGGTTCACGAATGTTTCTCCCTCGTTTC
5_known	C2	Tb927.11.6590_for	GGGGACAAGTTTGTACAAAAAAGCAGGCTATGCCTACTTTACCCAAGGCCG	Tb927.11.6590_rev	GGGGACCACTTTGTACAAGAAAGCTGGGTCTACAATTTTCGGAAGTACTCGGTAAGT
5_known	C3	Tb927.11.4940_for	GGGGACAAGTTTGTACAAAAAAGCAGGCTATGTCCGTCGATGGGGCG	Tb927.11.4940_rev	GGGGACCACTTTGTACAAGAAAGCTGGGTTCAAAGGGCAGCAAATGCATAATCA
5_known	C4	Tb927.5.2430_for	GGGGACAAGTTTGTACAAAAAAGCAGGCTATGCAGACACTTTCAGCCTCTTTT	Tb927.5.2430_rev	GGGGACCACTTTGTACAAGAAAGCTGGGTTTACTGGTGTCGCCGACTCA
5_known	C5	Tb927.6.2810_for	GGGGACAAGTTTGTACAAAAAAGCAGGCTATGGCACTGTCATTTACCGATCC	Tb927.6.2810_rev	GGGGACCACTTTGTACAAGAAAGCTGGGTTCACACGATTACGTTCACGTGC
5_known	C6	Tb927.5.3860_for	GGGGACAAGTTTGTACAAAAAAGCAGGCTATGTTCCCTAATACATCGCTGCG	Tb927.5.3860_rev	GGGGACCACTTTGTACAAGAAAGCTGGGTTCAGCACTCTCCTACTTGCAAAAG
5_known	C7	Tb927.6.1680_for	GGGGACAAGTTTGTACAAAAAAGCAGGCTATGCGGCGCTGGTTGGTG	Tb927.6.1680_rev	GGGGACCACTTTGTACAAGAAAGCTGGGTTTACGACGTCACCTCACTTACCT
5_known	C8	Tb927.11.13130_for	GGGGACAAGTTTGTACAAAAAAGCAGGCTATGAAGGAGAGAAAGGGCTCTTGG	Tb927.11.13130_rev	GGGGACCACTTTGTACAAGAAAGCTGGGTCTATGGGACGGGGCCATCA
5_known	C9	Tb927.2.4210_for	GGGGACAAGTTTGTACAAAAAAGCAGGCTATGGCTCCTATAATTCACAAGAATCTCAC	Tb927.2.4210_rev	GGGGACCACTTTGTACAAGAAAGCTGGGTTTAAAGACGCGAAGCCTCAACAT
5_known	C10	Tb927.10.470_for	GGGGACAAGTTTGTACAAAAAAGCAGGCTATGCGGTGCAACAAAGCATTAC	Tb927.10.470_rev	GGGGACCACTTTGTACAAGAAAGCTGGGTTCAATAAATTATACGCGCCTTCGCA
5_known	C11	Tb927.10.1710_for	GGGGACAAGTTTGTACAAAAAAGCAGGCTATGCGCTGCATCGCCCAC	Tb927.10.1710_rev	GGGGACCACTTTGTACAAGAAAGCTGGGTTCAGTCAATGATGTGTACATCCTTCTCT
5_known	C12	Tb927.11.8720_for	GGGGACAAGTTTGTACAAAAAAGCAGGCTATGGGTCGTAGGAGCAACTCTG	Tb927.11.8720_rev	GGGGACCACTTTGTACAAGAAAGCTGGGTTCATACAACCAAAGAAAGGGCACTG
5_known	C13	Tb927.6.1850_for	GGGGACAAGTTTGTACAAAAAAGCAGGCTATGTTCTCTCGATCATTTGTAGTCTTTGC	Tb927.6.1850_rev	GGGGACCACTTTGTACAAGAAAGCTGGGTCTAGTAACGGTCCCGCCTCTT
5_known	C14	Tb927.8.8070_for	GGGGACAAGTTTGTACAAAAAAGCAGGCTATGCGGACCAGAACAACAATGAC	Tb927.8.8070_rev	GGGGACCACTTTGTACAAGAAAGCTGGGTTTAGCAAATGCGTTTCATCACAGC
5_known	C15	Tb927.9.8780_for	GGGGACAAGTTTGTACAAAAAAGCAGGCTATGTTGCTACCAAAGAAGGTTAGATTGA	Tb927.9.8780_rev	GGGGACCACTTTGTACAAGAAAGCTGGGTTTAGGAGGAACCTCCCTGTCTTAAC
5_known	C16	Tb927.10.14090_for	GGGGACAAGTTTGTACAAAAAAGCAGGCTATGATGGCTTCCGAAGATAAAACGC	Tb927.10.14090_rev	GGGGACCACTTTGTACAAGAAAGCTGGGTCTACAAAGTGTCTTTGCTTCTTTCCG
5_known	C17	Tb927.10.13580_for	GGGGACAAGTTTGTACAAAAAAGCAGGCTATGATGGCTTCCGAAGATAAAACGC	Tb927.10.13580_rev	GGGGACCACTTTGTACAAGAAAGCTGGGTCTACGAATTAGCCTTTTCAGCCTCC
5_known	C18	Tb927.10.8440_for	GGGGACAAGTTTGTACAAAAAAGCAGGCTATGACTGAGCGTCGTGATAACG	Tb927.10.8440_rev	GGGGACCACTTTGTACAAGAAAGCTGGGTTTAGTTCCGCGGAGATGCTTCA
5_known	C20	Tb927.10.8480_for	GGGGACAAGTTTGTACAAAAAAGCAGGCTATGACTGAGCGTCGTGATAACG	Tb927.10.8480_rev	GGGGACCACTTTGTACAAGAAAGCTGGGTTTAGTTACGCGGAGATGCTTCAAC
5_known	C21	Tb927.7.5480_for	GGGGACAAGTTTGTACAAAAAAGCAGGCTATGCTCAGTCTTACGCGTATCCT	Tb927.7.5480_rev	GGGGACCACTTTGTACAAGAAAGCTGGGTCTACACCGCCATCTCCATAGAAATTAC
5_known	C22	Tb927.1.4600_for	GGGGACAAGTTTGTACAAAAAAGCAGGCTATGTTTTTGAAGGAGGGAAACAGTGA	Tb927.1.4600_rev	GGGGACCACTTTGTACAAGAAAGCTGGGTCTAAGATGCCGCTAGTACGCAC
5_known	C24	Tb927.11.7960_for	GGGGACAAGTTTGTACAAAAAAGCAGGCTATGAGAAAGTTTTCAGCTTTTCGGTCA	Tb927.11.7960_rev	GGGGACCACTTTGTACAAGAAAGCTGGGTCTACTGATGATTGTCGCATTTTATCCAGC
5_known	D6	Tb927.7.6450_for	GGGGACAAGTTTGTACAAAAAAGCAGGCTATGAGTGACAAAGATGATGGTGGTAC	Tb927.7.6450_rev	GGGGACCACTTTGTACAAGAAAGCTGGGTTCAACCCTCAACATCCATATCCGC
5_known	D7	Tb927.7.3630_for	GGGGACAAGTTTGTACAAAAAAGCAGGCTATGTACATTACAGGGGAGAACTCTGC	Tb927.7.3630_rev	GGGGACCACTTTGTACAAGAAAGCTGGGTTTAGTCGAAGTGAAAATAAAACTGCTGAAA
5_known	D8	Tb927.10.7110_for	GGGGACAAGTTTGTACAAAAAAGCAGGCTATGCCAGCCGTCCGTACG	Tb927.10.7110_rev	GGGGACCACTTTGTACAAGAAAGCTGGGTTCAACTTCCCACGCCGCG
5_known	D9	Tb927.10.8490_for	GGGGACAAGTTTGTACAAAAAAGCAGGCTATGACTGAGCGTCGTGATAACG	Tb927.10.8490_rev	GGGGACCACTTTGTACAAGAAAGCTGGGTTTAGTTCCGCGGCGTGGG
5_known	D12	Tb927.11.1900_for	GGGGACAAGTTTGTACAAAAAAGCAGGCTATGCTTTTCGCTAACCAAGCTCA	Tb927.11.1900_rev	GGGGACCACTTTGTACAAGAAAGCTGGGTTCATTGAGTCCGTTTGCGTGG
5_known	D15	Tb927.10.7680_for	GGGGACAAGTTTGTACAAAAAAGCAGGCTATGATCAACAACGGTTTAGAGGCC	Tb927.10.7680_rev	GGGGACCACTTTGTACAAGAAAGCTGGGTTTAGTATTTAGTGTAAGTTAACCCTGGCG
5_known	D16	Tb927.10.13160_for	GGGGACAAGTTTGTACAAAAAAGCAGGCTATGTCGCCAACTCGTGCG	Tb927.10.13160_rev	GGGGACCACTTTGTACAAGAAAGCTGGGTTCAGGTCCATGGCAAGCCA
5_known	D17	Tb927.11.6550_for	GGGGACAAGTTTGTACAAAAAAGCAGGCTATGTTGTCCCGAGCCCCA	Tb927.11.6550_rev	GGGGACCACTTTGTACAAGAAAGCTGGGTTTAGCCGTTCTCACTGCTCACG
5_known	D18	Tb927.11.16060_for	GGGGACAAGTTTGTACAAAAAAGCAGGCTATGGCGATGTTGCGCGGT	Tb927.11.16060_rev	GGGGACCACTTTGTACAAGAAAGCTGGGTTTACTTCCACCGCTCCTTCGC
5_known	D19	Tb927.9.12810_for	GGGGACAAGTTTGTACAAAAAAGCAGGCTATGACTTCTCACAACATAGTGATGAGGC	Tb927.9.12810_rev	GGGGACCACTTTGTACAAGAAAGCTGGGTCTAAATTAACACCAGATGTGTGCCCA
5_known	D20	Tb927.5.520_for	GGGGACAAGTTTGTACAAAAAAGCAGGCTATGTTACGTAGTTTGGTGCACTCG	Tb927.5.520_rev	GGGGACCACTTTGTACAAGAAAGCTGGGTCTACCTAAATCCGCTACTATTATTATTATT
5_known	D21	Tb927.8.5250_for	GGGGACAAGTTTGTACAAAAAAGCAGGCTATGACCGTTATTGTTGCTGGGATAG	Tb927.8.5250_rev	GGGGACCACTTTGTACAAGAAAGCTGGGTTTACTGTACGTTGTATCCTTCCGCA
5_known	D22	Tb927.10.9420_for	GGGGACAAGTTTGTACAAAAAAGCAGGCTATGAGGCGGTGTGTAGCTTATGT	Tb927.10.9420_rev	GGGGACCACTTTGTACAAGAAAGCTGGGTTCATCGACGACATTTTCTGTAAATAGCG
5_known	D23	Tb927.6.2640_for	GGGGACAAGTTTGTACAAAAAAGCAGGCTATGTTCAGTGGTCAGAATGAGAAACC	Tb927.6.2640_rev	GGGGACCACTTTGTACAAGAAAGCTGGGTTCAAAGATCGAACTGGCCTTGTG
5_known	D24	Tb927.1.3220_for	GGGGACAAGTTTGTACAAAAAAGCAGGCTATGGTGCTGTCCGCCGCA	Tb927.1.3220_rev	GGGGACCACTTTGTACAAGAAAGCTGGGTTCAAACTGTGAAAACTGAAGATCTTATGCT
5_known	E1	Tb927.3.5540_for	GGGGACAAGTTTGTACAAAAAAGCAGGCTATGCCCATTCCGTGTAATTTCAAGG	Tb927.3.5540_rev	GGGGACCACTTTGTACAAGAAAGCTGGGTTCAGAGAGAGAAACCAAACAGAGATATGA
5_known	E2	Tb927.7.3110_for	GGGGACAAGTTTGTACAAAAAAGCAGGCTATGGCGAAGCGCCGTGAA	Tb927.7.3110_rev	GGGGACCACTTTGTACAAGAAAGCTGGGTCTACTTGGCATCCACAGTGTCATC
5_known	E3	Tb927.9.15030_for	GGGGACAAGTTTGTACAAAAAAGCAGGCTATGGATTACCGTAATGATATCCAACAGTTA	Tb927.9.15030_rev	GGGGACCACTTTGTACAAGAAAGCTGGGTTCATACTTGGTCGTCGACACTGC
5_known	E4	Tb927.6.3510_for	GGGGACAAGTTTGTACAAAAAAGCAGGCTATGAAGAGAAACAGTGCGCGAG	Tb927.6.3510_rev	GGGGACCACTTTGTACAAGAAAGCTGGGTCTATTTGCACCGCCTGCGG
5_known	E5	Tb927.10.1060_for	GGGGACAAGTTTGTACAAAAAAGCAGGCTATGTCTACCAAGAAGGCAAATGAAAACA	Tb927.10.1060_rev	GGGGACCACTTTGTACAAGAAAGCTGGGTTTACGCCCGTGTCATGATGATG
5_known	E6	Tb927.10.8190_for	GGGGACAAGTTTGTACAAAAAAGCAGGCTATGTCGTTCATGCAAACTGGTCC	Tb927.10.8190_rev	GGGGACCACTTTGTACAAGAAAGCTGGGTTTAATCATCATCTCTTGCCTGATCCG
5_known	E7	Tb927.7.290_for	GGGGACAAGTTTGTACAAAAAAGCAGGCTATGCCAAATGCTATTGGCCTGC	Tb927.7.290_rev	GGGGACCACTTTGTACAAGAAAGCTGGGTTTATTTATTAACCCATACGTTCATTGACTT
5_known	E8	Tb927.10.5440_for	GGGGACAAGTTTGTACAAAAAAGCAGGCTATGGAGCGCGGAGGGCGC	Tb927.10.5440_rev	GGGGACCACTTTGTACAAGAAAGCTGGGTCTAAACTAATTCGGCCACTAGTAGTGC
5_known	E9	Tb927.10.9600_for	GGGGACAAGTTTGTACAAAAAAGCAGGCTATGCTAGGTGACCGCTACGC	Tb927.10.9600_rev	GGGGACCACTTTGTACAAGAAAGCTGGGTTCAGGCTTTTGAGGAGGCGT
5_known	E10	Tb927.11.14250_for	GGGGACAAGTTTGTACAAAAAAGCAGGCTATGTCTCTGGCCTTTGATGAGTACG	Tb927.11.14250_rev	GGGGACCACTTTGTACAAGAAAGCTGGGTCTATTCCTCCTCTTCGGGCTTTGT
5_known	E11	Tb927.6.3630_for	GGGGACAAGTTTGTACAAAAAAGCAGGCTATGTCGCTGTCATGCTTTTTGGA	Tb927.6.3630_rev	GGGGACCACTTTGTACAAGAAAGCTGGGTCTAATGTTCGGTGCTGTAGTACTTGT
5_known	E12	Tb927.9.9960_for	GGGGACAAGTTTGTACAAAAAAGCAGGCTATGCCAGTTATCGATTCGATGACAC	Tb927.9.9960_rev	GGGGACCACTTTGTACAAGAAAGCTGGGTTCACGAAGAAAGTGGTATTTCATTTAATAC
5_known	E13	Tb927.11.15560_for	GGGGACAAGTTTGTACAAAAAAGCAGGCTATGAAGGGCTTTGCTGTGCG	Tb927.11.15560_rev	GGGGACCACTTTGTACAAGAAAGCTGGGTCTAAGCGATCGTTGACTTCCCTAGC
5_known	E14	Tb927.6.4660_for	GGGGACAAGTTTGTACAAAAAAGCAGGCTATGGTCTCATGGTCCCGTCC	Tb927.6.4660_rev	GGGGACCACTTTGTACAAGAAAGCTGGGTTCAATTATGGACATAACGCTCAGTCAA
5_known	E15	Tb927.1.2740_for	GGGGACAAGTTTGTACAAAAAAGCAGGCTATGCAAATGATGGGACGGGC	Tb927.1.2740_rev	GGGGACCACTTTGTACAAGAAAGCTGGGTTTATGATAAGGATTTGTAATACCGCTGCC
5_known	E16	Tb927.8.6500_for	GGGGACAAGTTTGTACAAAAAAGCAGGCTATGAACTTCGAGTTGAGCATCAACA	Tb927.8.6500_rev	GGGGACCACTTTGTACAAGAAAGCTGGGTTCAGCGCAAGGGGAGCTC
5_known	E17	Tb927.11.5480_for	GGGGACAAGTTTGTACAAAAAAGCAGGCTATGATGAACATATCACCTCAATTTAAACGG	Tb927.11.5480_rev	GGGGACCACTTTGTACAAGAAAGCTGGGTCTCACCGTGGTAGTAGATCATTTGTCC
5_known	E18	Tb927.11.2440_for	GGGGACAAGTTTGTACAAAAAAGCAGGCTATGACTAACAAATACGCCCCTGGA	Tb927.11.2440_rev	GGGGACCACTTTGTACAAGAAAGCTGGGTCTACGACTGGCCGTTCGC
5_known	E19	Tb927.6.4090_for	GGGGACAAGTTTGTACAAAAAAGCAGGCTATGCTTTATTCAACACGCCGTCT	Tb927.6.4090_rev	GGGGACCACTTTGTACAAGAAAGCTGGGTTCAACCACTATCCCGTTGACGC
5_known	E20	Tb927.10.1260_for	GGGGACAAGTTTGTACAAAAAAGCAGGCTATGTATCGTTTCTGGCCGAGC	Tb927.10.1260_rev	GGGGACCACTTTGTACAAGAAAGCTGGGTTCATGGCGCCAACCGCCT
5_known	E21	Tb927.11.6960_for	GGGGACAAGTTTGTACAAAAAAGCAGGCTATGTCATGGATTGGCATTATTAACCGC	Tb927.11.6960_rev	GGGGACCACTTTGTACAAGAAAGCTGGGTTCAATTCCACAAGACCAACCGC
5_known	E22	Tb927.6.3160_for	GGGGACAAGTTTGTACAAAAAAGCAGGCTATGTTTGGTGGAGTTTTAGAAAAGATTCG	Tb927.6.3160_rev	GGGGACCACTTTGTACAAGAAAGCTGGGTTCAACCTGTTTTCCTCGACATCTG
5_known	E23	Tb927.7.800_for	GGGGACAAGTTTGTACAAAAAAGCAGGCTATGCGACGTCGGGTAGTTTTATG	Tb927.7.800_rev	GGGGACCACTTTGTACAAGAAAGCTGGGTCTAAAGCGATGCTTCGTCAGGT
5_known	E24	Tb927.6.3050_for	GGGGACAAGTTTGTACAAAAAAGCAGGCTATGCCAGCAGGTGTTCCGG	Tb927.6.3050_rev	GGGGACCACTTTGTACAAGAAAGCTGGGTTCATTCATTGCTCCTCGGACCT
5_known	F1	Tb927.7.1920_for	GGGGACAAGTTTGTACAAAAAAGCAGGCTATGTTACAAGCGGCTGAGCAG	Tb927.7.1920_rev	GGGGACCACTTTGTACAAGAAAGCTGGGTTCATCTCTCAATATATAATCGTCGATTAAA
5_known	F2	Tb927.11.6760_for	GGGGACAAGTTTGTACAAAAAAGCAGGCTATGAAGATCGACCTCTCTCACCG	Tb927.11.6760_rev	GGGGACCACTTTGTACAAGAAAGCTGGGTTCAGTTTTCGAAATCCGCGTCG
5_known	F3	Tb927.11.3240_for	GGGGACAAGTTTGTACAAAAAAGCAGGCTATGATGGCATCTAACCTAGCATACATAAAT	Tb927.11.3240_rev	GGGGACCACTTTGTACAAGAAAGCTGGGTTTACTGCGACGGGGGGGC
5_known	F4	Tb927.11.9560_for	GGGGACAAGTTTGTACAAAAAAGCAGGCTATGACATGCAAACGGACATTTGATG	Tb927.11.9560_rev	GGGGACCACTTTGTACAAGAAAGCTGGGTCTACCCAACGGGGTCTTGTAAAAA
5_known	F5	Tb927.11.2520_for	GGGGACAAGTTTGTACAAAAAAGCAGGCTATGAGTGACAGGGACGTGTGC	Tb927.11.2520_rev	GGGGACCACTTTGTACAAGAAAGCTGGGTTTACATGTTCGATGATTCGGAGACC
5_known	F6	Tb927.11.4200_for	GGGGACAAGTTTGTACAAAAAAGCAGGCTATGTCGGCCATCGTGCAC	Tb927.11.4200_rev	GGGGACCACTTTGTACAAGAAAGCTGGGTCTAGTCACGACGATCTCTCGAGC
5_known	F7	Tb927.10.10130_for	GGGGACAAGTTTGTACAAAAAAGCAGGCTATGCTCAACGTTCTCTCGAGC	Tb927.10.10130_rev	GGGGACCACTTTGTACAAGAAAGCTGGGTTCACATAACTCCGGGAGCAGC
5_known	F8	Tb927.3.5640_for	GGGGACAAGTTTGTACAAAAAAGCAGGCTATGGTGGAAATCACTGAACTCGC	Tb927.3.5640_rev	GGGGACCACTTTGTACAAGAAAGCTGGGTTCACTCAAGTGCAGGAAATGGTG
5_known	F9	Tb927.7.6220_for	GGGGACAAGTTTGTACAAAAAAGCAGGCTATGGACTTCCATTCGTTGCAGAG	Tb927.7.6220_rev	GGGGACCACTTTGTACAAGAAAGCTGGGTTCATCTCTCGTTGCGGCGC
5_known	F10	Tb927.9.11050_for	GGGGACAAGTTTGTACAAAAAAGCAGGCTATGAGAACTACAATTCGATACACTCGC	Tb927.9.11050_rev	GGGGACCACTTTGTACAAGAAAGCTGGGTCTAGCGGCGCTGGTTGGG
5_known	F11	Tb927.11.2670_for	GGGGACAAGTTTGTACAAAAAAGCAGGCTATGGTGAACCGGAAGGCAAAG	Tb927.11.2670_rev	GGGGACCACTTTGTACAAGAAAGCTGGGTTTAGGGCTTTGATTGCATCGTCAT
5_known	F12	Tb927.8.7380_for	GGGGACAAGTTTGTACAAAAAAGCAGGCTATGTTACGGCACACGCGTT	Tb927.8.7380_rev	GGGGACCACTTTGTACAAGAAAGCTGGGTCTATTGTTTGCCACCGCCTTGA
5_known	F13	Tb927.8.4010_for	GGGGACAAGTTTGTACAAAAAAGCAGGCTATGGGTGGCAGGACTGAATCA	Tb927.8.4010_rev	GGGGACCACTTTGTACAAGAAAGCTGGGTTCACTCGAACGCCGGCAA
5_known	F14	Tb927.11.8870_for	GGGGACAAGTTTGTACAAAAAAGCAGGCTATGAGGGCCCTGCGTTGT	Tb927.11.8870_rev	GGGGACCACTTTGTACAAGAAAGCTGGGTCTACCAACCACTGTCACGAGGT
5_known	F15	Tb927.7.740_for	GGGGACAAGTTTGTACAAAAAAGCAGGCTATGTCAAGTGGACTAACAGCCAGG	Tb927.7.740_rev	GGGGACCACTTTGTACAAGAAAGCTGGGTTTAACCACCTTCACCTCGTTTCTG
5_known	F16	Tb927.6.2150_for	GGGGACAAGTTTGTACAAAAAAGCAGGCTATGGAGCTGTCAGCTGTACTTGA	Tb927.6.2150_rev	GGGGACCACTTTGTACAAGAAAGCTGGGTTCACGCGGAGAAGAACAGACT
5_known	F17	Tb927.5.470_for	GGGGACAAGTTTGTACAAAAAAGCAGGCTATGCTTCCAAGTTTCACCCGTAAA	Tb927.5.470_rev	GGGGACCACTTTGTACAAGAAAGCTGGGTTCACATAATCTCAACAAGTTTTGTCTTCCG
5_known	F18	Tb927.11.7820_for	GGGGACAAGTTTGTACAAAAAAGCAGGCTATGTATACAACAATGCCTAACTCCCCA	Tb927.11.7820_rev	GGGGACCACTTTGTACAAGAAAGCTGGGTTTAAGAGAGTAAGCACAGCGGAACT
5_known	F19	Tb927.1.1840_for	GGGGACAAGTTTGTACAAAAAAGCAGGCTATGCGGCCCGCTCCCACG	Tb927.1.1840_rev	GGGGACCACTTTGTACAAGAAAGCTGGGTCTAAGAGCGTTTGCAACTCATTGC
5_known	F20	Tb927.3.3690_for	GGGGACAAGTTTGTACAAAAAAGCAGGCTATGGCTAGTCTCACAGATGATGACA	Tb927.3.3690_rev	GGGGACCACTTTGTACAAGAAAGCTGGGTCTAATCATATTCTTCTTCATCTTCCCCAGC
5_known	F21	Tb927.11.7070_for	GGGGACAAGTTTGTACAAAAAAGCAGGCTATGAAAACCTTTGGGGTGCGAT	Tb927.11.7070_rev	GGGGACCACTTTGTACAAGAAAGCTGGGTTCAATATGCACATTTAGCACCTGAAACC
5_known	F22	Tb927.8.4540_for	GGGGACAAGTTTGTACAAAAAAGCAGGCTATGGAGGGGTCTCATAGCAGC	Tb927.8.4540_rev	GGGGACCACTTTGTACAAGAAAGCTGGGTCTATTTCCCAACTCGTTTCTTCGGC
5_known	F23	Tb927.9.11900_for	GGGGACAAGTTTGTACAAAAAAGCAGGCTATGTCGAAATCGCTGCAGCG	Tb927.9.11900_rev	GGGGACCACTTTGTACAAGAAAGCTGGGTCTACAAGAAACGTTCCTCGGATGG
5_known	F24	Tb927.11.3420_for	GGGGACAAGTTTGTACAAAAAAGCAGGCTATGGTGCATAACACCTCCACTGC	Tb927.11.3420_rev	GGGGACCACTTTGTACAAGAAAGCTGGGTCTACTGCGGTGCATTGGCA
5_known	G1	Tb927.5.2940_for	GGGGACAAGTTTGTACAAAAAAGCAGGCTATGGACGCAACGGAGTTAAAGAAT	Tb927.5.2940_rev	GGGGACCACTTTGTACAAGAAAGCTGGGTCTACTGGCCGAAACGGATAATCC
5_known	G2	Tb927.10.7050_for	GGGGACAAGTTTGTACAAAAAAGCAGGCTATGGCAACATCCATTCAGCAAAATC	Tb927.10.7050_rev	GGGGACCACTTTGTACAAGAAAGCTGGGTTTAATCGTTGAGGTTATCCATAAGTTTGGA
5_known	G3	Tb927.10.7930_for	GGGGACAAGTTTGTACAAAAAAGCAGGCTATGGCACTCACGCTTGCTG	Tb927.10.7930_rev	GGGGACCACTTTGTACAAGAAAGCTGGGTCTACGCTACCTCAATGAGCGAC
5_known	G4	Tb927.4.2290_for	GGGGACAAGTTTGTACAAAAAAGCAGGCTATGATTGAGGGAAGCAGAAACTCAAG	Tb927.4.2290_rev	GGGGACCACTTTGTACAAGAAAGCTGGGTCTAAGATGATTGCCATAAGCGTAAATGC
5_known	G5	Tb927.11.16760_for	GGGGACAAGTTTGTACAAAAAAGCAGGCTATGCAAACCAAGCAGAAGGCC	Tb927.11.16760_rev	GGGGACCACTTTGTACAAGAAAGCTGGGTTTAACGGCGTCCCTCCTCC
5_known	G6	Tb927.6.5030_for	GGGGACAAGTTTGTACAAAAAAGCAGGCTATGTCCAAAGGTTACACGGTATATTCC	Tb927.6.5030_rev	GGGGACCACTTTGTACAAGAAAGCTGGGTCTAACAACTGTGGACCCACAGGG
5_known	G7	Tb927.2.5750_for	GGGGACAAGTTTGTACAAAAAAGCAGGCTATGGAGGAAACAAAGTTATGCCCATT	Tb927.2.5750_rev	GGGGACCACTTTGTACAAGAAAGCTGGGTTCACGCATCCCTGTTGGTTCG
5_known	G8	Tb927.11.9900_for	GGGGACAAGTTTGTACAAAAAAGCAGGCTATGTTGCGCCGTTCTTTCTTGC	Tb927.11.9900_rev	GGGGACCACTTTGTACAAGAAAGCTGGGTTCACTTCCGCCCGAATCCA
5_known	G9	Tb927.8.1640_for	GGGGACAAGTTTGTACAAAAAAGCAGGCTATGATGCTACCGGCGTGTGT	Tb927.8.1640_rev	GGGGACCACTTTGTACAAGAAAGCTGGGTTTAACAGGCAGCATAAACTATAGCGAA
5_known	G10	Tb927.8.2960_for	GGGGACAAGTTTGTACAAAAAAGCAGGCTATGGGCGGGAACCGACGG	Tb927.8.2960_rev	GGGGACCACTTTGTACAAGAAAGCTGGGTTCACCATGGGCTTTCATCGC
5_known	G11	Tb927.11.8940_for	GGGGACAAGTTTGTACAAAAAAGCAGGCTATGTCTACGGGAAAGATAATTGGTGATC	Tb927.11.8940_rev	GGGGACCACTTTGTACAAGAAAGCTGGGTCTAGTTCTTCTCCAGCCAACGGA
5_known	G14	Tb927.11.1930_for	GGGGACAAGTTTGTACAAAAAAGCAGGCTATGTCCTTTTTCCAGTTGGATTGGG	Tb927.11.1930_rev	GGGGACCACTTTGTACAAGAAAGCTGGGTCTAAGCAGAACGTACGTAACACAGA
5_known	G16	Tb927.11.8350_for	GGGGACAAGTTTGTACAAAAAAGCAGGCTATGAGCACATCTAATGACGTGCTG	Tb927.11.8350_rev	GGGGACCACTTTGTACAAGAAAGCTGGGTCTAAACATTTACCTCGAACTCCTTCTTCC
5_known	G17	Tb927.10.1620_for	GGGGACAAGTTTGTACAAAAAAGCAGGCTATGCAGGAACTTATCGCTGTCCG	Tb927.10.1620_rev	GGGGACCACTTTGTACAAGAAAGCTGGGTTCAACTCCTGCTGGATGGTATGG
5_known	G18	Tb927.10.8720_for	GGGGACAAGTTTGTACAAAAAAGCAGGCTATGACGGATGATGTCTTGAGACAAC	Tb927.10.8720_rev	GGGGACCACTTTGTACAAGAAAGCTGGGTTCATGCGCACCATGAAAGCC
5_known	G19	Tb927.11.5050_for	GGGGACAAGTTTGTACAAAAAAGCAGGCTATGTTACGACGGTCGTCCCT	Tb927.11.5050_rev	GGGGACCACTTTGTACAAGAAAGCTGGGTCTACCTGAGCTGTTGGAAGAAGTCA
5_known	G20	Tb927.9.10770_for	GGGGACAAGTTTGTACAAAAAAGCAGGCTATGGCTGCATTTGCTGCTGC	Tb927.9.10770_rev	GGGGACCACTTTGTACAAGAAAGCTGGGTCTACATGCCAATGTGACGGTTGA
5_known	G21	Tb927.7.4000_for	GGGGACAAGTTTGTACAAAAAAGCAGGCTATGGTGTTAAAATTGTTGCTGGAGC	Tb927.7.4000_rev	GGGGACCACTTTGTACAAGAAAGCTGGGTTTACGGTACAACCGCTAAGGAATCA
5_known	G22	Tb927.8.3150_for	GGGGACAAGTTTGTACAAAAAAGCAGGCTATGCAGCAGCAACAGCCC	Tb927.8.3150_rev	GGGGACCACTTTGTACAAGAAAGCTGGGTTTAGGCAGCTTCAGCCTCACC
5_known	G23	Tb927.7.210_for	GGGGACAAGTTTGTACAAAAAAGCAGGCTATGTTTCGCCTCCTGTGCCG	Tb927.7.210_rev	GGGGACCACTTTGTACAAGAAAGCTGGGTTCACATCCAAAAGACGCGACG
5_known	G24	Tb927.11.13810_for	GGGGACAAGTTTGTACAAAAAAGCAGGCTATGGATATATTTAGTGCTCTTTCCACGG	Tb927.11.13810_rev	GGGGACCACTTTGTACAAGAAAGCTGGGTCTACTGCACCTCTTCCACGC
5_known	H1	Tb927.4.260_for	GGGGACAAGTTTGTACAAAAAAGCAGGCTATGAGGAGGCCACGGGATG	Tb927.4.260_rev	GGGGACCACTTTGTACAAGAAAGCTGGGTTTAATTCATCATCATATTTCTGATCATCAG
5_known	H2	Tb927.9.12790_for	GGGGACAAGTTTGTACAAAAAAGCAGGCTATGATGATATATCCTCGCATGAGAGTGG	Tb927.9.12790_rev	GGGGACCACTTTGTACAAGAAAGCTGGGTCTAAGCACAACTATCTTCACCCCAC
5_known	H5	Tb927.11.790_for	GGGGACAAGTTTGTACAAAAAAGCAGGCTATGCCCTTGAATTCATCTCACCG	Tb927.11.790_rev	GGGGACCACTTTGTACAAGAAAGCTGGGTTTACGCCACGAGCGATAGTTTC
5_known	H6	Tb927.10.4050_for	GGGGACAAGTTTGTACAAAAAAGCAGGCTATGCCTACTTACGTGGAGGCG	Tb927.10.4050_rev	GGGGACCACTTTGTACAAGAAAGCTGGGTTTACTCCTCGCCCTGCGG
5_known	H7	Tb927.4.280_for	GGGGACAAGTTTGTACAAAAAAGCAGGCTATGAGGAGGCCACGGGATG	Tb927.4.280_rev	GGGGACCACTTTGTACAAGAAAGCTGGGTTTAATTCATCATCATATTTCTGATCATAAG
5_known	H8	Tb927.8.1830_for	GGGGACAAGTTTGTACAAAAAAGCAGGCTATGACGTCCCAGGCGGTG	Tb927.8.1830_rev	GGGGACCACTTTGTACAAGAAAGCTGGGTCTAAATAATCCAACCACTGGCTACCAC
5_known	H9	Tb927.10.2490_for	GGGGACAAGTTTGTACAAAAAAGCAGGCTATGGACGGTGATCTTTCCCAACT	Tb927.10.2490_rev	GGGGACCACTTTGTACAAGAAAGCTGGGTTTACAAATGATGAAGCTTCCGCTGC
5_known	H10	Tb927.10.4780_for	GGGGACAAGTTTGTACAAAAAAGCAGGCTATGAGTCGACTCTATATATTTTACAAACTG	Tb927.10.4780_rev	GGGGACCACTTTGTACAAGAAAGCTGGGTTCAGTAGCCCCCCTGGTCC
5_known	H11	Tb927.10.310_for	GGGGACAAGTTTGTACAAAAAAGCAGGCTATGTGGACTGTGCAGGAGGAC	Tb927.10.310_rev	GGGGACCACTTTGTACAAGAAAGCTGGGTTCAGCCGGAGAGCGGTTG
5_known	H12	Tb927.6.1880_for	GGGGACAAGTTTGTACAAAAAAGCAGGCTATGTCGGACCCCGCTCAA	Tb927.6.1880_rev	GGGGACCACTTTGTACAAGAAAGCTGGGTCTACGGTGTCACACGCTGC
5_known	H13	Tb927.11.6780_for	GGGGACAAGTTTGTACAAAAAAGCAGGCTATGGAAGAACGCGTTTCTTGTGT	Tb927.11.6780_rev	GGGGACCACTTTGTACAAGAAAGCTGGGTCTAACAAAGTGTTGCGGGGTCG
5_known	H14	Tb927.11.4900_for	GGGGACAAGTTTGTACAAAAAAGCAGGCTATGCGAATCAAGGTAGACGTTGAG	Tb927.11.4900_rev	GGGGACCACTTTGTACAAGAAAGCTGGGTTTACGCAGCATACGCTTCAGTC
5_known	H15	Tb927.6.2850_for	GGGGACAAGTTTGTACAAAAAAGCAGGCTATGTCCCACTTTCAGCCTTCGC	Tb927.6.2850_rev	GGGGACCACTTTGTACAAGAAAGCTGGGTTTAGTCGCTGCAAAAGTCCGC
5_known	H16	Tb927.9.12360_for	GGGGACAAGTTTGTACAAAAAAGCAGGCTATGGAGGGCACTGGCGGT	Tb927.9.12360_rev	GGGGACCACTTTGTACAAGAAAGCTGGGTTTATGCTGACTGATGCGGTGGG

5_known	H17	Tb927.4.4590_for	GGGGACAAGTTTGTACAAAAAAGCAGGCTATGGTGCCGTCTGAACCCC	Tb927.4.4590_rev	GGGGACCACTTTGTACAAGAAAGCTGGGTTCAAGGCCCCGGCTTCGA
5_known	H18	Tb927.9.4300_for	GGGGACAAGTTTGTACAAAAAAGCAGGCTATGCCTGCGAAGCGACGG	Tb927.9.4300_rev	GGGGACCACTTTGTACAAGAAAGCTGGGTTCACACAGTTGACTGACTTTCCG
5_known	H19	Tb927.10.3210_for	GGGGACAAGTTTGTACAAAAAAGCAGGCTATGCTTCGCCGTACGTTGC	Tb927.10.3210_rev	GGGGACCACTTTGTACAAGAAAGCTGGGTTCACAGAGTCACGTAGTCGGG
5_known	H20	Tb927.11.7750_for	GGGGACAAGTTTGTACAAAAAAGCAGGCTATGACAGTGATTATGTTCCCTCGTTATATC	Tb927.11.7750_rev	GGGGACCACTTTGTACAAGAAAGCTGGGTTTATAATAGGGAAAAGCAAGAAACGAAGAG
5_known	H21	Tb927.10.6400_for	GGGGACAAGTTTGTACAAAAAAGCAGGCTATGTTCCGCTGTGTCGTCCG	Tb927.10.6400_rev	GGGGACCACTTTGTACAAGAAAGCTGGGTTCAATACATGCCGTCCATACCTCC
5_known	H23	Tb927.10.9850_for	GGGGACAAGTTTGTACAAAAAAGCAGGCTATGCATTCAGTCCACGAATCGC	Tb927.10.9850_rev	GGGGACCACTTTGTACAAGAAAGCTGGGTTTACAAAGCAGGAAGGACGTACTGT
5_known	H24	Tb927.10.15570_for	GGGGACAAGTTTGTACAAAAAAGCAGGCTATGAATGGAAGGTATGCTCGGCA	Tb927.10.15570_rev	GGGGACCACTTTGTACAAGAAAGCTGGGTCTATATTTCACGAGAAGTTACGGCCC
5_known	I1	Tb927.4.4220_for	GGGGACAAGTTTGTACAAAAAAGCAGGCTATGCGCCCCGAGTTCCCG	Tb927.4.4220_rev	GGGGACCACTTTGTACAAGAAAGCTGGGTTCAACAACCCGAGCGTTTGG
5_known	I2	Tb927.11.11500_for	GGGGACAAGTTTGTACAAAAAAGCAGGCTATGAACGATCTGTCCGCAGTCG	Tb927.11.11500_rev	GGGGACCACTTTGTACAAGAAAGCTGGGTTTAGGGCCACGCAAACTTCTTAC
5_known	I3	Tb927.10.15970_for	GGGGACAAGTTTGTACAAAAAAGCAGGCTATGTGTACGTGCACCTGCTG	Tb927.10.15970_rev	GGGGACCACTTTGTACAAGAAAGCTGGGTCTAGGTGGATCGTCCGTTGGT
5_known	I4	Tb927.11.5440_for	GGGGACAAGTTTGTACAAAAAAGCAGGCTATGACGCATCAATCAACTGATGTGT	Tb927.11.5440_rev	GGGGACCACTTTGTACAAGAAAGCTGGGTTTACTCGTCAGGGAGAATGCCTG
5_known	I5	Tb927.11.7710_for	GGGGACAAGTTTGTACAAAAAAGCAGGCTATGACAGTGATTATGTTCCCTCGTTATATC	Tb927.11.7710_rev	GGGGACCACTTTGTACAAGAAAGCTGGGTTTATAATAGGGGAAAACAAGAAACGAAGAG
5_known	I6	Tb927.8.1240_for	GGGGACAAGTTTGTACAAAAAAGCAGGCTATGCTTCGTCGAACAAGTTGTTGT	Tb927.8.1240_rev	GGGGACCACTTTGTACAAGAAAGCTGGGTTCACATTTGTGAGTTATAGTTGGGACC
5_known	I7	Tb927.10.1540_for	GGGGACAAGTTTGTACAAAAAAGCAGGCTATGCCTCCCGCTGACAGG	Tb927.10.1540_rev	GGGGACCACTTTGTACAAGAAAGCTGGGTCTACGCTTTTTGCCGACGTTTC
5_known	I8	Tb927.11.6270_for	GGGGACAAGTTTGTACAAAAAAGCAGGCTATGCGCAGCCTACTGGAAGG	Tb927.11.6270_rev	GGGGACCACTTTGTACAAGAAAGCTGGGTTCAAAAAAACAGTGTCACAGCGGC
5_known	I9	Tb927.10.5510_for	GGGGACAAGTTTGTACAAAAAAGCAGGCTATGAGGGCTGCCGAGGGT	Tb927.10.5510_rev	GGGGACCACTTTGTACAAGAAAGCTGGGTTTATTTGTTGCTTGGAATTGTTGCAACG
5_known	I10	Tb927.3.4500_for	GGGGACAAGTTTGTACAAAAAAGCAGGCTATGAGTCTCTGCGAAAACTGTGAG	Tb927.3.4500_rev	GGGGACCACTTTGTACAAGAAAGCTGGGTCTAGACGAGTTTAGCATAGAAGTCATTACC
5_known	I11	Tb927.11.2470_for	GGGGACAAGTTTGTACAAAAAAGCAGGCTATGCCTGCAACCCTCAAGC	Tb927.11.2470_rev	GGGGACCACTTTGTACAAGAAAGCTGGGTTTATGCTGTCCCAAAGAGTGAGGC
5_known	I12	Tb927.10.5970_for	GGGGACAAGTTTGTACAAAAAAGCAGGCTATGTCGACCACTTCTTTCGGGT	Tb927.10.5970_rev	GGGGACCACTTTGTACAAGAAAGCTGGGTTCACCTACTTCTCTTGCGCCC
5_known	I13	Tb927.11.15600_for	GGGGACAAGTTTGTACAAAAAAGCAGGCTATGGTTGTGGAGCCTCCTGC	Tb927.11.15600_rev	GGGGACCACTTTGTACAAGAAAGCTGGGTTCACTGTCCCTCACTTAGACTCCC
5_known	I14	Tb927.3.1130_for	GGGGACAAGTTTGTACAAAAAAGCAGGCTATGTCTGAGGCTCTGATTGACGA	Tb927.3.1130_rev	GGGGACCACTTTGTACAAGAAAGCTGGGTTTAACGTGGGTTGCGCAGC
5_known	I15	Tb927.11.5300_for	GGGGACAAGTTTGTACAAAAAAGCAGGCTATGAATAATAGCCGCATTTGCGTTG	Tb927.11.5300_rev	GGGGACCACTTTGTACAAGAAAGCTGGGTTTAATTGCGTCTCTTCATGGAAGAGTT
5_known	I16	Tb927.5.1580_for	GGGGACAAGTTTGTACAAAAAAGCAGGCTATGGCGAGAAACCGGAAACATG	Tb927.5.1580_rev	GGGGACCACTTTGTACAAGAAAGCTGGGTTCACAGAGGGTACACGGGC
5_known	I17	Tb927.9.9290_for	GGGGACAAGTTTGTACAAAAAAGCAGGCTATGACAATCGCTGCACAGGG	Tb927.9.9290_rev	GGGGACCACTTTGTACAAGAAAGCTGGGTTTAAGCGCTTGAGGCGTGTAC
5_known	I18	Tb927.3.3000_for	GGGGACAAGTTTGTACAAAAAAGCAGGCTATGCTGTCTACAAGTGGGAGGG	Tb927.3.3000_rev	GGGGACCACTTTGTACAAGAAAGCTGGGTTCACTTCTTCTTCAATTTGCTCTCCTT
5_known	I19	Tb927.3.2820_for	GGGGACAAGTTTGTACAAAAAAGCAGGCTATGTTAAATGTTCGGGGTTTGTTAAGC	Tb927.3.2820_rev	GGGGACCACTTTGTACAAGAAAGCTGGGTTTAGCTCCCCAGCCGACC
5_known	I20	Tb927.11.3870_for	GGGGACAAGTTTGTACAAAAAAGCAGGCTATGAGTAAAAAGGGTAACATTCGCCTT	Tb927.11.3870_rev	GGGGACCACTTTGTACAAGAAAGCTGGGTTTATGACGACGCTCCGGAGC
5_known	I21	Tb927.10.6630_for	GGGGACAAGTTTGTACAAAAAAGCAGGCTATGGAGGAGACGTACAGTCCCT	Tb927.10.6630_rev	GGGGACCACTTTGTACAAGAAAGCTGGGTCTATCTCGCGTATTTCTTGCGGG
5_known	I22	Tb927.9.11270_for	GGGGACAAGTTTGTACAAAAAAGCAGGCTATGATTCAGCCACAATTAATTTTACTCCG	Tb927.9.11270_rev	GGGGACCACTTTGTACAAGAAAGCTGGGTTCAGCGGCCGGCACGACC
5_known	I23	Tb927.10.11100_for	GGGGACAAGTTTGTACAAAAAAGCAGGCTATGTACGTACCGAGTCGTGTTGT	Tb927.10.11100_rev	GGGGACCACTTTGTACAAGAAAGCTGGGTCTAAATCACCTCTGCTGCATGTTTCT
5_known	I24	Tb927.11.16570_for	GGGGACAAGTTTGTACAAAAAAGCAGGCTATGAGATGCTGTGTCCGAGCC	Tb927.11.16570_rev	GGGGACCACTTTGTACAAGAAAGCTGGGTCTACGATCCAAAGGAACGTCGC
5_known	J1	Tb927.10.13510_for	GGGGACAAGTTTGTACAAAAAAGCAGGCTATGAGCACTGCTAGCAACACC	Tb927.10.13510_rev	GGGGACCACTTTGTACAAGAAAGCTGGGTCTAGTACTGCGGCTGGTTTTGC
5_known	J2	Tb927.1.730_for	GGGGACAAGTTTGTACAAAAAAGCAGGCTATGTGGCGGCTGCTTCGT	Tb927.1.730_rev	GGGGACCACTTTGTACAAGAAAGCTGGGTTCACTTCAGTTTGGAGTGTTTACCAC
5_known	J3	Tb927.10.10870_for	GGGGACAAGTTTGTACAAAAAAGCAGGCTATGCACAATGTCCCGTTACTCG	Tb927.10.10870_rev	GGGGACCACTTTGTACAAGAAAGCTGGGTTTAACGAGGTGATGAAAAGTGCCTC
5_known	J6	Tb927.11.5450_for	GGGGACAAGTTTGTACAAAAAAGCAGGCTATGTTGGGTCGTTCGTTTAAACTTTG	Tb927.11.5450_rev	GGGGACCACTTTGTACAAGAAAGCTGGGTTTACTCCAGCTTCTTCGACAGGT
5_known	J7	Tb927.11.8620_for	GGGGACAAGTTTGTACAAAAAAGCAGGCTATGTGTGGGGGAGAAGAGAATGG	Tb927.11.8620_rev	GGGGACCACTTTGTACAAGAAAGCTGGGTCTAAATTATGGTAACTTCCCTCTCAACAGT
5_known	J11	Tb927.4.300_for	GGGGACAAGTTTGTACAAAAAAGCAGGCTATGTCAAGAGCCAATAGCCCCG	Tb927.4.300_rev	GGGGACCACTTTGTACAAGAAAGCTGGGTTTAATTCATCATCATATTTCTGATCATCAG
5_known	J12	Tb927.11.8490_for	GGGGACAAGTTTGTACAAAAAAGCAGGCTATGTGTGGGGGAGAAGAGAATGG	Tb927.11.8490_rev	GGGGACCACTTTGTACAAGAAAGCTGGGTTCACTCAATTATGGTAACTTCCCTCTCA
5_known	J13	Tb927.10.4430_for	GGGGACAAGTTTGTACAAAAAAGCAGGCTATGTCGTCGGATGAGGAAACCA	Tb927.10.4430_rev	GGGGACCACTTTGTACAAGAAAGCTGGGTCTATGTTCCTTTCTTGCTTTCCGGA
5_known	J15	Tb927.11.10640_for	GGGGACAAGTTTGTACAAAAAAGCAGGCTATGTTTCTCATCAGCTGGAATGTGG	Tb927.11.10640_rev	GGGGACCACTTTGTACAAGAAAGCTGGGTTTATTCACTTCTTGGCCGCTTTTGG
5_known	J16	Tb927.5.3050_for	GGGGACAAGTTTGTACAAAAAAGCAGGCTATGGCGGCTAAAGGAAAGTCAAC	Tb927.5.3050_rev	GGGGACCACTTTGTACAAGAAAGCTGGGTCTATTTATACTCAAAAGAAACACGAACTAG
5_known	J17	Tb927.11.15010_for	GGGGACAAGTTTGTACAAAAAAGCAGGCTATGGGAAACGGTGTTGTGAGAGT	Tb927.11.15010_rev	GGGGACCACTTTGTACAAGAAAGCTGGGTCTACCCCATGTCAAGAGTAAACATGAG
5_known	J18	Tb927.1.1380_for	GGGGACAAGTTTGTACAAAAAAGCAGGCTATGTCAGAGCACTTTAAAAATGTCAAGTC	Tb927.1.1380_rev	GGGGACCACTTTGTACAAGAAAGCTGGGTTTAATTCTCTGTGGCGGCAAAACG
5_known	J19	Tb927.11.6750_for	GGGGACAAGTTTGTACAAAAAAGCAGGCTATGTTCCGTCTTGTAAGACCCCG	Tb927.11.6750_rev	GGGGACCACTTTGTACAAGAAAGCTGGGTTCACACGAACCACCCCTTGC
5_known	J20	Tb927.8.5140_for	GGGGACAAGTTTGTACAAAAAAGCAGGCTATGTCGTTATCTAACGTTGTTGCAAATAAC	Tb927.8.5140_rev	GGGGACCACTTTGTACAAGAAAGCTGGGTTCACGCCCCTTCCCCTTC
5_known	J21	Tb927.11.3070_for	GGGGACAAGTTTGTACAAAAAAGCAGGCTATGCGGAAAGCCGCTAAACG	Tb927.11.3070_rev	GGGGACCACTTTGTACAAGAAAGCTGGGTTCACTTCATCAGTGACCGCCA
5_known	J22	Tb927.7.1640_for	GGGGACAAGTTTGTACAAAAAAGCAGGCTATGAGACGCTGCATCCCTGC	Tb927.7.1640_rev	GGGGACCACTTTGTACAAGAAAGCTGGGTTTAGATGTAGTCAGGGTTGGAATCCTT
5_known	J23	Tb927.10.5780_for	GGGGACAAGTTTGTACAAAAAAGCAGGCTATGACAAGTCGTGAGGGTATTCACC	Tb927.10.5780_rev	GGGGACCACTTTGTACAAGAAAGCTGGGTTTACTTTGATAGCATTGGGGCGAAA
5_known	J24	Tb927.9.5210_for	GGGGACAAGTTTGTACAAAAAAGCAGGCTATGACAACAACGGTTGATTCCGC	Tb927.9.5210_rev	GGGGACCACTTTGTACAAGAAAGCTGGGTTTACTGTTTCTTTCCCTTCACCCCC
5_known	K1	Tb927.10.3110_for	GGGGACAAGTTTGTACAAAAAAGCAGGCTATGCAAGCGATAGGAACTTCGC	Tb927.10.3110_rev	GGGGACCACTTTGTACAAGAAAGCTGGGTCTACAAAGAATCACGCGGACGG
5_known	K2	Tb927.7.3790_for	GGGGACAAGTTTGTACAAAAAAGCAGGCTATGGATCATATTGATCTTGCTGAAGAGC	Tb927.7.3790_rev	GGGGACCACTTTGTACAAGAAAGCTGGGTTTAGTCCAATAGCACGTCAATAGCC
5_known	K3	Tb927.10.16150_for	GGGGACAAGTTTGTACAAAAAAGCAGGCTATGGATCCCCTGTCGCGTG	Tb927.10.16150_rev	GGGGACCACTTTGTACAAGAAAGCTGGGTTCAGGCTGTCACATCACTGAACG
5_known	K4	Tb927.11.6220_for	GGGGACAAGTTTGTACAAAAAAGCAGGCTATGCCAACGCGGGAGGGT	Tb927.11.6220_rev	GGGGACCACTTTGTACAAGAAAGCTGGGTCTAAAGTCGCTCTAGTTCCTTTGCC
5_known	K5	Tb927.3.3400_for	GGGGACAAGTTTGTACAAAAAAGCAGGCTATGCATGACTCTGTGGGAACGG	Tb927.3.3400_rev	GGGGACCACTTTGTACAAGAAAGCTGGGTTTAAACACATTCAGATAGGATAAACCCGTC
5_known	K6	Tb927.9.2490_for	GGGGACAAGTTTGTACAAAAAAGCAGGCTATGACGGGAATGAACAGATCGTG	Tb927.9.2490_rev	GGGGACCACTTTGTACAAGAAAGCTGGGTCTATCCATCAACGTCCTCCCCT
5_known	K7	Tb927.2.1180_for	GGGGACAAGTTTGTACAAAAAAGCAGGCTATGAATCAACAGGTTCCTATTGAAGGAAG	Tb927.2.1180_rev	GGGGACCACTTTGTACAAGAAAGCTGGGTTTAACTCATCATCATATTTCGAATCATCAG
5_known	K8	Tb927.4.3840_for	GGGGACAAGTTTGTACAAAAAAGCAGGCTATGTCCAAGCCGGATCGAAAGG	Tb927.4.3840_rev	GGGGACCACTTTGTACAAGAAAGCTGGGTTCATCCACTTTTCCCTTTACTCTTTGG
5_known	K9	Tb927.3.5250_for	GGGGACAAGTTTGTACAAAAAAGCAGGCTATGGGGCGTGGAAAGGGG	Tb927.3.5250_rev	GGGGACCACTTTGTACAAGAAAGCTGGGTCTATTCTGTGGTTAGCAGCGCC
5_known	K10	Tb927.8.5500_for	GGGGACAAGTTTGTACAAAAAAGCAGGCTATGGCTCATGGAGCTTCTCCG	Tb927.8.5500_rev	GGGGACCACTTTGTACAAGAAAGCTGGGTCTATGTATGATGATTCTGGATGACTTTAGC
5_known	K11	Tb927.5.4480_for	GGGGACAAGTTTGTACAAAAAAGCAGGCTATGGCTCCGAAGAGGGGAAG	Tb927.5.4480_rev	GGGGACCACTTTGTACAAGAAAGCTGGGTCTACATCTCATATGGTATAAAGTGAACCTC
5_known	K12	Tb927.11.3270_for	GGGGACAAGTTTGTACAAAAAAGCAGGCTATGGAGGCGGCCATTATTGGA	Tb927.11.3270_rev	GGGGACCACTTTGTACAAGAAAGCTGGGTTCACAAACCAATTGGCTTCCTTTTCC
5_known	K13	Tb927.9.12900_for	GGGGACAAGTTTGTACAAAAAAGCAGGCTATGTATCGACACGGTCTGGAATCA	Tb927.9.12900_rev	GGGGACCACTTTGTACAAGAAAGCTGGGTTCACTTTCGCTCATGTAGCTTGC
5_known	K14	Tb927.10.10990_for	GGGGACAAGTTTGTACAAAAAAGCAGGCTATGAGACATTGGGCGCAGGC	Tb927.10.10990_rev	GGGGACCACTTTGTACAAGAAAGCTGGGTCTACCGCAACTCGTCTTCAAGG
5_known	K15	Tb927.10.12380_for	GGGGACAAGTTTGTACAAAAAAGCAGGCTATGGAAGATGCGAAATACCGTACG	Tb927.10.12380_rev	GGGGACCACTTTGTACAAGAAAGCTGGGTTCAGTCTACGATGATGCCGATAAGC
5_known	K16	Tb927.5.3710_for	GGGGACAAGTTTGTACAAAAAAGCAGGCTATGGCTGCGGAGATAACTGTTCT	Tb927.5.3710_rev	GGGGACCACTTTGTACAAGAAAGCTGGGTTCATTTACAACCATTACCTTTATGCATTTC
5_known	K17	Tb927.11.10750_for	GGGGACAAGTTTGTACAAAAAAGCAGGCTATGTCACGCTACATCCCACCA	Tb927.11.10750_rev	GGGGACCACTTTGTACAAGAAAGCTGGGTTCACGCGTACTCCAGTGCG
5_known	K18	Tb927.4.1140_for	GGGGACAAGTTTGTACAAAAAAGCAGGCTATGTCGAATGCTTTGTTTTGTCTTCAC	Tb927.4.1140_rev	GGGGACCACTTTGTACAAGAAAGCTGGGTCTAGAGTATACCATTCTGGAGAGTTTGGA
5_known	K19	Tb927.4.5040_for	GGGGACAAGTTTGTACAAAAAAGCAGGCTATGATTCGGCTGTGCCGGG	Tb927.4.5040_rev	GGGGACCACTTTGTACAAGAAAGCTGGGTCTAGCGTCGCTCAATATCCTCG
5_known	K20	Tb927.11.9890_for	GGGGACAAGTTTGTACAAAAAAGCAGGCTATGATCGACACGCTTTCGATAGTTAG	Tb927.11.9890_rev	GGGGACCACTTTGTACAAGAAAGCTGGGTCTACGCCATTAAAGCGCTGACA
5_known	K21	Tb927.3.690_for	GGGGACAAGTTTGTACAAAAAAGCAGGCTATGGAGAACTACCAACTTCTCAATCAGT	Tb927.3.690_rev	GGGGACCACTTTGTACAAGAAAGCTGGGTCTAACTGTTGGAACCTTGGTTGTGC
5_known	K22	Tb927.7.6900_for	GGGGACAAGTTTGTACAAAAAAGCAGGCTATGTTCTACTCAACCTACGTGCTGA	Tb927.7.6900_rev	GGGGACCACTTTGTACAAGAAAGCTGGGTTCACACAGTGAGTTGCACCTCC
5_known	K23	Tb927.7.7420_for	GGGGACAAGTTTGTACAAAAAAGCAGGCTATGCGTCGCTTTGGTTCCAA	Tb927.7.7420_rev	GGGGACCACTTTGTACAAGAAAGCTGGGTCTACACTGCCCGCTTACAAGAG
5_known	K24	Tb927.7.250_for	GGGGACAAGTTTGTACAAAAAAGCAGGCTATGCAGAAACAGCGTATCACTTTCG	Tb927.7.250_rev	GGGGACCACTTTGTACAAGAAAGCTGGGTTTACACCCTTGCTTCCATGATTACAC
5_known	L1	Tb927.3.680_for	GGGGACAAGTTTGTACAAAAAAGCAGGCTATGGCTGTAATAACGAATACCTCAAGTC	Tb927.3.680_rev	GGGGACCACTTTGTACAAGAAAGCTGGGTTTAATAACGAGGTCGCGGATGGC
5_known	L3	Tb927.8.1600_for	GGGGACAAGTTTGTACAAAAAAGCAGGCTATGTCGGCTGTGGAGGAGC	Tb927.8.1600_rev	GGGGACCACTTTGTACAAGAAAGCTGGGTTCAAAGAAGTGGCACCCCTTGT
5_known	L4	Tb927.1.1470_for	GGGGACAAGTTTGTACAAAAAAGCAGGCTATGGGTGGTATTCCTTCACGCG	Tb927.1.1470_rev	GGGGACCACTTTGTACAAGAAAGCTGGGTTCACTGACCCCAGAAGCCC
5_known	L5	Tb927.7.5970_for	GGGGACAAGTTTGTACAAAAAAGCAGGCTATGAGCGCACCCGTCGAC	Tb927.7.5970_rev	GGGGACCACTTTGTACAAGAAAGCTGGGTCTATTCTGTGTGGGACAGTGACTCA
5_known	L6	Tb927.7.460_for	GGGGACAAGTTTGTACAAAAAAGCAGGCTATGTCTTTTGTACCACCTCCAGCA	Tb927.7.460_rev	GGGGACCACTTTGTACAAGAAAGCTGGGTTCAGCACATGAAAGTCTTTGCGC
5_known	L7	Tb927.11.4480_for	GGGGACAAGTTTGTACAAAAAAGCAGGCTATGCCAGTAAATCCTGAACCACAG	Tb927.11.4480_rev	GGGGACCACTTTGTACAAGAAAGCTGGGTTTACATTTCTTCTTCCTCTTCCTCAGCT
5_known	L8	Tb927.9.8400_for	GGGGACAAGTTTGTACAAAAAAGCAGGCTATGATTGGAAATGGGCGAGGATTG	Tb927.9.8400_rev	GGGGACCACTTTGTACAAGAAAGCTGGGTTTAGCAACGCCTTGACTGACC
5_known	L10	Tb927.11.14580_for	GGGGACAAGTTTGTACAAAAAAGCAGGCTATGTCAGCTACTGATGTCGCATCT	Tb927.11.14580_rev	GGGGACCACTTTGTACAAGAAAGCTGGGTTCACACATTTCTGTAAAAGGGAACTTTCT
5_known	L11	Tb927.8.2850_for	GGGGACAAGTTTGTACAAAAAAGCAGGCTATGCAGGTGACACGGGACA	Tb927.8.2850_rev	GGGGACCACTTTGTACAAGAAAGCTGGGTTCACCTTCTTAGTGCTTTGCAAACC
5_known	L12	Tb927.5.2900_for	GGGGACAAGTTTGTACAAAAAAGCAGGCTATGTCTGTGAGGGAACGGAATAAAAAT	Tb927.5.2900_rev	GGGGACCACTTTGTACAAGAAAGCTGGGTTCACTTGATGATATTGGACTCCGCA
5_known	L13	Tb927.3.1340_for	GGGGACAAGTTTGTACAAAAAAGCAGGCTATGCCTCCCAGACGTTCCTT	Tb927.3.1340_rev	GGGGACCACTTTGTACAAGAAAGCTGGGTTTACTCCTTTTCTTCCACCTGGGA
5_known	L15	Tb927.11.12150_for	GGGGACAAGTTTGTACAAAAAAGCAGGCTATGGCGTTTCTCGTACAAGTAGC	Tb927.11.12150_rev	GGGGACCACTTTGTACAAGAAAGCTGGGTTTAATCTCGCGGATAGGACCTCC
5_known	L16	Tb927.11.5650_for	GGGGACAAGTTTGTACAAAAAAGCAGGCTATGATGACCAGCAGTCCGAGT	Tb927.11.5650_rev	GGGGACCACTTTGTACAAGAAAGCTGGGTCTAATAGTCATCGGAACTGTAGCTCTCA
5_known	L17	Tb927.10.8210_for	GGGGACAAGTTTGTACAAAAAAGCAGGCTATGTACCGCAGGTTCTTTCGC	Tb927.10.8210_rev	GGGGACCACTTTGTACAAGAAAGCTGGGTCTATGTGATCACAGCTAGAGTCCCT
5_known	L18	Tb927.10.15650_for	GGGGACAAGTTTGTACAAAAAAGCAGGCTATGTTTGTTACCTCGCAACTAACACA	Tb927.10.15650_rev	GGGGACCACTTTGTACAAGAAAGCTGGGTTTATATGTCACGGGCGATGTTCAC
5_known	L19	Tb927.8.2910_for	GGGGACAAGTTTGTACAAAAAAGCAGGCTATGCCGTGGGTTCGTGCT	Tb927.8.2910_rev	GGGGACCACTTTGTACAAGAAAGCTGGGTTTATCGAAACACAGACTTCGACAGC
5_known	L20	Tb927.10.13960_for	GGGGACAAGTTTGTACAAAAAAGCAGGCTATGGCGCAGGTCGGCGAG	Tb927.10.13960_rev	GGGGACCACTTTGTACAAGAAAGCTGGGTTCAGCACCAGTACCTCACCAC
5_known	L21	Tb927.10.5560_for	GGGGACAAGTTTGTACAAAAAAGCAGGCTATGAAGCGAGCACCTTGTTTGT	Tb927.10.5560_rev	GGGGACCACTTTGTACAAGAAAGCTGGGTTCACTTCGTCACCTTGACCCA
5_known	L22	Tb927.3.4310_for	GGGGACAAGTTTGTACAAAAAAGCAGGCTATGGCCGCAGTTGACGATG	Tb927.3.4310_rev	GGGGACCACTTTGTACAAGAAAGCTGGGTCTATTCGAGGCGTGCCGG
5_known	M1	Tb927.10.6780_for	GGGGACAAGTTTGTACAAAAAAGCAGGCTATGTCGACCGCGCCTCCC	Tb927.10.6780_rev	GGGGACCACTTTGTACAAGAAAGCTGGGTTCATGCGTGCGCTGCTAG
5_known	M2	Tb927.1.1240_for	GGGGACAAGTTTGTACAAAAAAGCAGGCTATGGAAGGCAGTGCAACTGC	Tb927.1.1240_rev	GGGGACCACTTTGTACAAGAAAGCTGGGTTTACGCCTGTTTCAACCGGC
5_known	M5	Tb927.5.1020_for	GGGGACAAGTTTGTACAAAAAAGCAGGCTATGCAACGAACGAAAAGTTTGCTT	Tb927.5.1020_rev	GGGGACCACTTTGTACAAGAAAGCTGGGTTTAGTTCCTCTTCTTTTCAACTGCAGAA
5_known	M6	Tb927.9.15810_for	GGGGACAAGTTTGTACAAAAAAGCAGGCTATGCCAAGGGCAAACAGACC	Tb927.9.15810_rev	GGGGACCACTTTGTACAAGAAAGCTGGGTTTAATTCATCATCATATTTCTAATCATCAC
5_known	M7	Tb927.7.5950_for	GGGGACAAGTTTGTACAAAAAAGCAGGCTATGAGCGCACCCGTCGAC	Tb927.7.5950_rev	GGGGACCACTTTGTACAAGAAAGCTGGGTTTAAATTACACCTATCGGTTCCGTAGAAAC
5_known	M8	Tb927.8.4110_for	GGGGACAAGTTTGTACAAAAAAGCAGGCTATGGGTGGCAGGACTGAATCA	Tb927.8.4110_rev	GGGGACCACTTTGTACAAGAAAGCTGGGTCTACTCCAGCGCCGGCAG
5_known	M10	Tb927.9.12960_for	GGGGACAAGTTTGTACAAAAAAGCAGGCTATGCAACAAAAATCGGACGCCG	Tb927.9.12960_rev	GGGGACCACTTTGTACAAGAAAGCTGGGTTCACCCCTTAATACCTGTGAAAATTCC
5_known	M11	Tb927.10.9720_for	GGGGACAAGTTTGTACAAAAAAGCAGGCTATGTATCACTACGAGTACGATGACGA	Tb927.10.9720_rev	GGGGACCACTTTGTACAAGAAAGCTGGGTTCAACCGACAGTGGCGGC
5_known	M12	Tb927.10.2410_for	GGGGACAAGTTTGTACAAAAAAGCAGGCTATGACCCAACTGTTAGGAACCGC	Tb927.10.2410_rev	GGGGACCACTTTGTACAAGAAAGCTGGGTTCACATTTGACGAGGGTTCACG
5_known	M13	Tb927.3.3390_for	GGGGACAAGTTTGTACAAAAAAGCAGGCTATGCATGACTCTGTGGGAACGG	Tb927.3.3390_rev	GGGGACCACTTTGTACAAGAAAGCTGGGTTCACTCCATGCCGAAGGCG
5_known	M14	Tb927.8.1650_for	GGGGACAAGTTTGTACAAAAAAGCAGGCTATGAACTGCGCGGCGGAC	Tb927.8.1650_rev	GGGGACCACTTTGTACAAGAAAGCTGGGTTTAGTTGGGAAGTTCCGCCGT
5_known	M15	Tb927.4.2190_for	GGGGACAAGTTTGTACAAAAAAGCAGGCTATGGAAACTAAAGAGAAATCACCAATGTTG	Tb927.4.2190_rev	GGGGACCACTTTGTACAAGAAAGCTGGGTCTACACATTTGCAAATACCGATTGCA
5_known	M16	Tb927.8.1670_for	GGGGACAAGTTTGTACAAAAAAGCAGGCTATGGGGATGGATCACTACACCG	Tb927.8.1670_rev	GGGGACCACTTTGTACAAGAAAGCTGGGTTCACTGCACAGCAGGTGCG
5_known	M17	Tb927.11.13500_for	GGGGACAAGTTTGTACAAAAAAGCAGGCTATGATCGAGGTGCAACTTAACAGTT	Tb927.11.13500_rev	GGGGACCACTTTGTACAAGAAAGCTGGGTCTACGCACGACCGACGGG
5_known	M18	Tb927.3.3940_for	GGGGACAAGTTTGTACAAAAAAGCAGGCTATGGAAGTTCCTTTTGAAGAAGGGAC	Tb927.3.3940_rev	GGGGACCACTTTGTACAAGAAAGCTGGGTTTACAAAAACGGCACAAATGAGCC
5_known	M19	Tb927.10.13740_for	GGGGACAAGTTTGTACAAAAAAGCAGGCTATGGAAAAGGCAAATGAACTGATGC	Tb927.10.13740_rev	GGGGACCACTTTGTACAAGAAAGCTGGGTTTAGTGGCGCAGAAGCTTAAGTTC
5_known	M20	Tb927.11.15040_for	GGGGACAAGTTTGTACAAAAAAGCAGGCTATGCTGAGGCGCTCCTTCG	Tb927.11.15040_rev	GGGGACCACTTTGTACAAGAAAGCTGGGTCTAAATACCCTTCATTTGAAACTTCATCGG
5_known	M21	Tb927.9.6710_for	GGGGACAAGTTTGTACAAAAAAGCAGGCTATGTGGCGGCGCTCTCGG	Tb927.9.6710_rev	GGGGACCACTTTGTACAAGAAAGCTGGGTCTACGTATATACCCTGCCAATGTGATG
5_known	M22	Tb927.6.4210_for	GGGGACAAGTTTGTACAAAAAAGCAGGCTATGGAAGGTGTATCTGCCTTACTGG	Tb927.6.4210_rev	GGGGACCACTTTGTACAAGAAAGCTGGGTTCAAGAATCCTTCTTGCGTGGAGC
5_known	M23	Tb927.10.5320_for	GGGGACAAGTTTGTACAAAAAAGCAGGCTATGCGGAACTCGCTCATTCAG	Tb927.10.5320_rev	GGGGACCACTTTGTACAAGAAAGCTGGGTTCAGCCGGCCGATTGTGC
5_known	M24	Tb927.7.5930_for	GGGGACAAGTTTGTACAAAAAAGCAGGCTATGAGCGCACCCGTCGAC	Tb927.7.5930_rev	GGGGACCACTTTGTACAAGAAAGCTGGGTTCATTGCGGAGCAGCCTCA
5_known	N1	Tb927.10.1720_for	GGGGACAAGTTTGTACAAAAAAGCAGGCTATGAACATCTACAGTTGGGAGAGCT	Tb927.10.1720_rev	GGGGACCACTTTGTACAAGAAAGCTGGGTTTAATTTTGTGGTGTGACTTCCTTCAATG
5_known	N2	Tb927.7.3580_for	GGGGACAAGTTTGTACAAAAAAGCAGGCTATGGATAGGTTTACTAAGGTCCGTAATATT	Tb927.7.3580_rev	GGGGACCACTTTGTACAAGAAAGCTGGGTTCAAGTAGCATTCCGCCTTGC
5_known	N3	Tb927.8.2920_for	GGGGACAAGTTTGTACAAAAAAGCAGGCTATGAAAGGCGCTCAGTTTTTGAAGA	Tb927.8.2920_rev	GGGGACCACTTTGTACAAGAAAGCTGGGTTTATCGAAACACAGACTTCGACAGC
5_known	N4	Tb927.9.10670_for	GGGGACAAGTTTGTACAAAAAAGCAGGCTATGCGTCGGGAGCAGTTAGG	Tb927.9.10670_rev	GGGGACCACTTTGTACAAGAAAGCTGGGTCTATGAATTGTCTTCTATGAATCCAGCGT
5_known	N6	Tb927.3.2280_for	GGGGACAAGTTTGTACAAAAAAGCAGGCTATGAACCCTTTATGCGAAATCATTAAGC	Tb927.3.2280_rev	GGGGACCACTTTGTACAAGAAAGCTGGGTCTACTCTTCCAAAGGATAGGGCAGT
5_known	N7	Tb927.11.11670_for	GGGGACAAGTTTGTACAAAAAAGCAGGCTATGGACTTCAAACGACAGCTGG	Tb927.11.11670_rev	GGGGACCACTTTGTACAAGAAAGCTGGGTCTACTCGCCGCTTCTTTTTGAGC
5_known	N8	Tb927.7.5980_for	GGGGACAAGTTTGTACAAAAAAGCAGGCTATGAGCGCACCCGTCGAC	Tb927.7.5980_rev	GGGGACCACTTTGTACAAGAAAGCTGGGTTCACTTGAGTTGATGCGCAGG
5_known	N9	Tb927.9.3800_for	GGGGACAAGTTTGTACAAAAAAGCAGGCTATGACCTGCGTAAAAGAGTTGTTGC	Tb927.9.3800_rev	GGGGACCACTTTGTACAAGAAAGCTGGGTCTATGTGCTAGCACCAGCCAC
5_known	N10	Tb927.10.5590_for	GGGGACAAGTTTGTACAAAAAAGCAGGCTATGTCCGGTCAGGGAAATTTGC	Tb927.10.5590_rev	GGGGACCACTTTGTACAAGAAAGCTGGGTTCAAACCTTCCTTTCCGGCAAAC
5_known	N12	Tb927.7.5960_for	GGGGACAAGTTTGTACAAAAAAGCAGGCTATGAGCGCACCCGTCGAC	Tb927.7.5960_rev	GGGGACCACTTTGTACAAGAAAGCTGGGTTCACTTGAGTTGATGCACAGGTTC
5_known	N13	Tb927.11.7450_for	GGGGACAAGTTTGTACAAAAAAGCAGGCTATGAGTAGGGATCGTGGTGGTATAG	Tb927.11.7450_rev	GGGGACCACTTTGTACAAGAAAGCTGGGTCTAAAAACGCCGCCTGCCT
5_known	N14	Tb927.10.4300_for	GGGGACAAGTTTGTACAAAAAAGCAGGCTATGGCCAGTCAAGGAGAAACGG	Tb927.10.4300_rev	GGGGACCACTTTGTACAAGAAAGCTGGGTTCACGGCAGCGAATTGAAACC
5_known	N15	Tb927.7.5380_for	GGGGACAAGTTTGTACAAAAAAGCAGGCTATGAGCTTATTTTCTTTTACGCCAACTG	Tb927.7.5380_rev	GGGGACCACTTTGTACAAGAAAGCTGGGTTTACGTAAGTGCAGCAAATGTCCC
5_known	N16	Tb927.11.360_for	GGGGACAAGTTTGTACAAAAAAGCAGGCTATGAAGACTATTGCCCTCATCTCCG	Tb927.11.360_rev	GGGGACCACTTTGTACAAGAAAGCTGGGTTCAAGTACCTTCCCGCTCATCTG
5_known	N17	Tb927.8.4990_for	GGGGACAAGTTTGTACAAAAAAGCAGGCTATGAGCGGAAAGGAAGTTGAAGG	Tb927.8.4990_rev	GGGGACCACTTTGTACAAGAAAGCTGGGTCTACTGAGTGATCTGCGGCATC
5_known	N21	Tb927.10.2230_for	GGGGACAAGTTTGTACAAAAAAGCAGGCTATGCTCTTGCGGCGGAGC	Tb927.10.2230_rev	GGGGACCACTTTGTACAAGAAAGCTGGGTTCACAGCTGTGATGGATGGAGC
5_known	N23	Tb927.11.4980_for	GGGGACAAGTTTGTACAAAAAAGCAGGCTATGAGTGGGTCACCGAGTGAC	Tb927.11.4980_rev	GGGGACCACTTTGTACAAGAAAGCTGGGTTTACAACTTGCGCCGCTTCA

5_known	N24	Tb927.7.7450_for	GGGGACAAGTTTGTACAAAAAAGCAGGCTATGGGGAAGCCTGGTAAAAAGGC	Tb927.7.7450_rev	GGGGACCACTTTGTACAAGAAAGCTGGGTTCAGTCCTGAGCTGCAAGAAGG
5_known	O1	Tb927.11.3650_for	GGGGACAAGTTTGTACAAAAAAGCAGGCTATGGCCGCGGCTCCAAGT	Tb927.11.3650_rev	GGGGACCACTTTGTACAAGAAAGCTGGGTTTATTGCTTGAGGTATATAATGTCTGAACC
5_known	O2	Tb927.10.15530_for	GGGGACAAGTTTGTACAAAAAAGCAGGCTATGGCCAAACCGAAGTACCTAAAAA	Tb927.10.15530_rev	GGGGACCACTTTGTACAAGAAAGCTGGGTTCACTTCCGGTTGGCGTAGT
5_known	O3	Tb927.8.980_for	GGGGACAAGTTTGTACAAAAAAGCAGGCTATGGTGCTGCAGGCTGCG	Tb927.8.980_rev	GGGGACCACTTTGTACAAGAAAGCTGGGTCTACGCTCCACCGCAGAAAC
5_known	O4	Tb927.11.14300_for	GGGGACAAGTTTGTACAAAAAAGCAGGCTATGACGGATGTGACGTACAGTTACT	Tb927.11.14300_rev	GGGGACCACTTTGTACAAGAAAGCTGGGTTCAATCCGAATCAACATCTATTCCCCC
5_known	O5	Tb927.4.3420_for	GGGGACAAGTTTGTACAAAAAAGCAGGCTATGTTGGCGCCTAAAGGGGG	Tb927.4.3420_rev	GGGGACCACTTTGTACAAGAAAGCTGGGTCTAAAATTTTGCACTATCTCCCCTATCCC
5_known	O6	Tb927.8.3800_for	GGGGACAAGTTTGTACAAAAAAGCAGGCTATGTTGCACTCAGACCACCCC	Tb927.8.3800_rev	GGGGACCACTTTGTACAAGAAAGCTGGGTTCACACACAGTGCTCCTCTAAGC
5_known	O7	Tb927.11.7380_for	GGGGACAAGTTTGTACAAAAAAGCAGGCTATGTCTCGTATTCCTGAGAAGATTCTCT	Tb927.11.7380_rev	GGGGACCACTTTGTACAAGAAAGCTGGGTTTATTGGCCGTGAAATGATTCCAGC
5_known	O8	Tb927.2.4020_for	GGGGACAAGTTTGTACAAAAAAGCAGGCTATGAGTAATGAAAAATATGACAGGCAGCT	Tb927.2.4020_rev	GGGGACCACTTTGTACAAGAAAGCTGGGTTTATTGAGCCTCTGCAACCCAGA
5_known	O9	Tb927.10.6260_for	GGGGACAAGTTTGTACAAAAAAGCAGGCTATGGGTGTGGATGACGTAGGC	Tb927.10.6260_rev	GGGGACCACTTTGTACAAGAAAGCTGGGTCTACCACTCACCACTGATGTTCTCC
5_known	O10	Tb927.11.11530_for	GGGGACAAGTTTGTACAAAAAAGCAGGCTATGGCGTCGTTAGCAGCAAA	Tb927.11.11530_rev	GGGGACCACTTTGTACAAGAAAGCTGGGTTCAGCTCTGGTGCTGATTGTGC
5_known	O11	Tb927.3.2470_for	GGGGACAAGTTTGTACAAAAAAGCAGGCTATGGGTAAAACTAACACAAAAAAGCAGG	Tb927.3.2470_rev	GGGGACCACTTTGTACAAGAAAGCTGGGTTTATTTCTTGGGGAGAACCTTCTTTTTCT
5_known	O12	Tb927.3.1920_for	GGGGACAAGTTTGTACAAAAAAGCAGGCTATGACAAATAATAAAAAGAGTCAACAGGAG	Tb927.3.1920_rev	GGGGACCACTTTGTACAAGAAAGCTGGGTTCAACGCAGCTCATTCTCGAG
5_known	O13	Tb927.7.1370_for	GGGGACAAGTTTGTACAAAAAAGCAGGCTATGAGAAGTAGTGCTAATTTTTTTTTAGCG	Tb927.7.1370_rev	GGGGACCACTTTGTACAAGAAAGCTGGGTTCAAAAAATAACTTCTTCCTCCGTAGCC
5_known	O14	Tb927.7.5940_for	GGGGACAAGTTTGTACAAAAAAGCAGGCTATGAGCGCACCCGTCGAC	Tb927.7.5940_rev	GGGGACCACTTTGTACAAGAAAGCTGGGTTTACTTGCTTGAATCAGCAGCTTTCT
5_known	O15	Tb927.10.10350_for	GGGGACAAGTTTGTACAAAAAAGCAGGCTATGAGTACACAAGAATCTCATGGTTCTG	Tb927.10.10350_rev	GGGGACCACTTTGTACAAGAAAGCTGGGTTCAAAACGTAAGAAATGGATGTTTCAAAAG
5_known	O16	Tb927.8.6660_for	GGGGACAAGTTTGTACAAAAAAGCAGGCTATGACGACGATGCAGAGGGT	Tb927.8.6660_rev	GGGGACCACTTTGTACAAGAAAGCTGGGTCTAAGGTGCGAAGGCTGCC
5_known	O17	Tb927.11.3470_for	GGGGACAAGTTTGTACAAAAAAGCAGGCTATGGGCAAGCAGGGAAATGTTG	Tb927.11.3470_rev	GGGGACCACTTTGTACAAGAAAGCTGGGTTTAATCAAGCTTCACCGAAGTGTATATCTT
5_known	O18	Tb927.11.7630_for	GGGGACAAGTTTGTACAAAAAAGCAGGCTATGACAGTGATTATGTTCCCTCGTTATG	Tb927.11.7630_rev	GGGGACCACTTTGTACAAGAAAGCTGGGTTTATAATAGGGAAAAGCAAGAAACGAAGAG
5_known	O19	Tb927.11.7640_for	GGGGACAAGTTTGTACAAAAAAGCAGGCTATGACGCTGATTATGTTCCCCCG	Tb927.11.7640_rev	GGGGACCACTTTGTACAAGAAAGCTGGGTTCATAATAGGGGAAAGCAAGAAACGAAG
5_known	O20	Tb927.6.4590_for	GGGGACAAGTTTGTACAAAAAAGCAGGCTATGCTCCGGTTAGCTAACAAGTTTAC	Tb927.6.4590_rev	GGGGACCACTTTGTACAAGAAAGCTGGGTCTAGACAGCTTGTTCTTTGTTCTGACT
5_known	O21	Tb927.11.11780_for	GGGGACAAGTTTGTACAAAAAAGCAGGCTATGTTTCGTCGTAGCCTTTCCC	Tb927.11.11780_rev	GGGGACCACTTTGTACAAGAAAGCTGGGTTCACCTAAGATCCCATGTCGACTC
5_known	O22	Tb927.1.3830_for	GGGGACAAGTTTGTACAAAAAAGCAGGCTATGAGCAATTACTTGGATGATTTGCG	Tb927.1.3830_rev	GGGGACCACTTTGTACAAGAAAGCTGGGTTCAAAGGTGCGACAATTCATTAAACATATT
5_known	O23	Tb927.5.4070_for	GGGGACAAGTTTGTACAAAAAAGCAGGCTATGCTCTGGTGTAGGGCAACG	Tb927.5.4070_rev	GGGGACCACTTTGTACAAGAAAGCTGGGTCTAAGCGTCCTGCGACGG
5_known	O24	Tb927.11.6990_for	GGGGACAAGTTTGTACAAAAAAGCAGGCTATGAACACCTTCAAGGGTGATGGG	Tb927.11.6990_rev	GGGGACCACTTTGTACAAGAAAGCTGGGTTCATGCAATCGTGGGGTTCCA
5_known	P1	Tb927.11.470_for	GGGGACAAGTTTGTACAAAAAAGCAGGCTATGTCTAGTTGTTCGCATCCTACCT	Tb927.11.470_rev	GGGGACCACTTTGTACAAGAAAGCTGGGTCTAAGCGACCCATTCATCCTCCG
5_known	P2	Tb927.10.15910_for	GGGGACAAGTTTGTACAAAAAAGCAGGCTATGTCAGACGACGAGCACAAAC	Tb927.10.15910_rev	GGGGACCACTTTGTACAAGAAAGCTGGGTTCAACGAACTATACCGTACCTTTCCT
5_known	P3	Tb927.10.960_for	GGGGACAAGTTTGTACAAAAAAGCAGGCTATGGAGTTAATTGATCCCGTAGTCCC	Tb927.10.960_rev	GGGGACCACTTTGTACAAGAAAGCTGGGTTTACTTTCTCTTCAGTAGTTGCATTTCATC
5_known	P4	Tb927.8.6580_for	GGGGACAAGTTTGTACAAAAAAGCAGGCTATGCTACGGCGTTCTTTTAACCG	Tb927.8.6580_rev	GGGGACCACTTTGTACAAGAAAGCTGGGTCTAGTAGACGCGCTTCGCTG
5_known	P5	Tb927.4.1950_for	GGGGACAAGTTTGTACAAAAAAGCAGGCTATGGAGGAAAATGAAGGGATTGGAAC	Tb927.4.1950_rev	GGGGACCACTTTGTACAAGAAAGCTGGGTCTACACAGACCATGTATCCACCACA
5_known	P6	Tb927.9.14550_for	GGGGACAAGTTTGTACAAAAAAGCAGGCTATGGTGTGGAAGGGTCAAGCC	Tb927.9.14550_rev	GGGGACCACTTTGTACAAGAAAGCTGGGTTTATTTAGCTCTCCACCAATCAAATAATGA
5_known	P7	Tb927.10.13210_for	GGGGACAAGTTTGTACAAAAAAGCAGGCTATGTTTAGCTACGATGATCTTGTGTGG	Tb927.10.13210_rev	GGGGACCACTTTGTACAAGAAAGCTGGGTTCAGAGTAAGTTCGCTAACAAGGGC
5_known	P8	Tb927.4.1080_for	GGGGACAAGTTTGTACAAAAAAGCAGGCTATGTCGAGCGACCGCAAC	Tb927.4.1080_rev	GGGGACCACTTTGTACAAGAAAGCTGGGTCTACTGCACGAGGGAAGCGA
5_known	P9	Tb927.11.6630_for	GGGGACAAGTTTGTACAAAAAAGCAGGCTATGAAGTCGTTTTGCCGGTTGG	Tb927.11.6630_rev	GGGGACCACTTTGTACAAGAAAGCTGGGTTCACATACGAAAAACCCCAAACTTTGT
5_known	P10	Tb927.3.4490_for	GGGGACAAGTTTGTACAAAAAAGCAGGCTATGAATAAGAGCGCGGTGCG	Tb927.3.4490_rev	GGGGACCACTTTGTACAAGAAAGCTGGGTTCAAACATAGTCCCGGTTCATAACG
5_known	P11	Tb927.6.140_for	GGGGACAAGTTTGTACAAAAAAGCAGGCTATGGACAACGAAACTCAACTGCT	Tb927.6.140_rev	GGGGACCACTTTGTACAAGAAAGCTGGGTTTACGTTCGGCCCGCAGC
5_known	P12	Tb927.7.7280_for	GGGGACAAGTTTGTACAAAAAAGCAGGCTATGCCAAACAGTGATCACAGCTAC	Tb927.7.7280_rev	GGGGACCACTTTGTACAAGAAAGCTGGGTCTACGTTGTTTCAATCGTCTGCG
5_known	P13	Tb927.6.1500_for	GGGGACAAGTTTGTACAAAAAAGCAGGCTATGGATAAGAGAATGATTACTGATGCATTT	Tb927.6.1500_rev	GGGGACCACTTTGTACAAGAAAGCTGGGTTCAGAGGTGAGCCTGAAGAGC
5_known	P14	Tb927.10.4480_for	GGGGACAAGTTTGTACAAAAAAGCAGGCTATGGAGACGAATGGACCGAAGA	Tb927.10.4480_rev	GGGGACCACTTTGTACAAGAAAGCTGGGTCTATGTAAAAGCAACAGGGTGAGGC
5_known	P15	Tb927.8.6740_for	GGGGACAAGTTTGTACAAAAAAGCAGGCTATGCCGAACAGCCATAACGAAA	Tb927.8.6740_rev	GGGGACCACTTTGTACAAGAAAGCTGGGTCTAGCAGTAACTCCTCGTCAATATAAATTT
5_known	P16	Tb927.1.670_for	GGGGACAAGTTTGTACAAAAAAGCAGGCTATGATGCAAGTCAAACTGAAGCATTTT	Tb927.1.670_rev	GGGGACCACTTTGTACAAGAAAGCTGGGTTCATGCGGCAGCTCGACA
5_known	P17	Tb927.8.1720_for	GGGGACAAGTTTGTACAAAAAAGCAGGCTATGCTCTTTCTTCTCGCTTACGC	Tb927.8.1720_rev	GGGGACCACTTTGTACAAGAAAGCTGGGTCTACAAAAGGTCCTGCCCCATTT
5_known	P18	Tb927.11.7620_for	GGGGACAAGTTTGTACAAAAAAGCAGGCTATGACAGTGATTATGTTCCCTCGTTATG	Tb927.11.7620_rev	GGGGACCACTTTGTACAAGAAAGCTGGGTTTATAGAGAAAAAAAAGAAACGAAGAGTAG
5_known	P19	Tb927.7.3210_for	GGGGACAAGTTTGTACAAAAAAGCAGGCTATGGCGACCTCTCGCAAGC	Tb927.7.3210_rev	GGGGACCACTTTGTACAAGAAAGCTGGGTTTAATCAGAGTCCGCAACAGTTTTAATGG
5_known	P20	Tb927.3.2090_for	GGGGACAAGTTTGTACAAAAAAGCAGGCTATGTCGACCAACAGTGCCAAG	Tb927.3.2090_rev	GGGGACCACTTTGTACAAGAAAGCTGGGTTCAACAACCGCCCTCAAGC
5_known	P21	Tb927.8.1870_for	GGGGACAAGTTTGTACAAAAAAGCAGGCTATGCGGCGTTCTTTCTTTGTTG	Tb927.8.1870_rev	GGGGACCACTTTGTACAAGAAAGCTGGGTCTAATCCTCCACCTCAATGACGGA
5_known	P22	Tb927.1.1130_for	GGGGACAAGTTTGTACAAAAAAGCAGGCTATGGGTCGCTATACGCGCC	Tb927.1.1130_rev	GGGGACCACTTTGTACAAGAAAGCTGGGTTCATGTGAGAGCCGTGGCG
5_known	P23	Tb927.11.10760_for	GGGGACAAGTTTGTACAAAAAAGCAGGCTATGCAAGCCGACACATTTGCT	Tb927.11.10760_rev	GGGGACCACTTTGTACAAGAAAGCTGGGTTTACATGGTAACACACTGGCATCC
5_known	P24	Tb927.10.10810_for	GGGGACAAGTTTGTACAAAAAAGCAGGCTATGCATGTGGATGTGCCTCAC	Tb927.10.10810_rev	GGGGACCACTTTGTACAAGAAAGCTGGGTTCATGCAACGCGTTGCTCC
6_hypothetical	A1	Tb927.10.14740_for	GGGGACAAGTTTGTACAAAAAAGCAGGCTATGATACCTCGAGCAGCGCA	Tb927.10.14740_rev	GGGGACCACTTTGTACAAGAAAGCTGGGTCTATTCACCGTTCCGCCGTC
6_hypothetical	A3	Tb927.10.7900_for	GGGGACAAGTTTGTACAAAAAAGCAGGCTATGCTCGGTAACTCACAAGGCG	Tb927.10.7900_rev	GGGGACCACTTTGTACAAGAAAGCTGGGTCTACAAGTCAAACAGCATTTCCTCCT
6_hypothetical	A4	Tb927.11.2580_for	GGGGACAAGTTTGTACAAAAAAGCAGGCTATGACGTCGTTCAAGGACAATATTTCA	Tb927.11.2580_rev	GGGGACCACTTTGTACAAGAAAGCTGGGTTCATACACGCGTTAAGACGAGGC
6_hypothetical	A5	Tb927.9.5020_for	GGGGACAAGTTTGTACAAAAAAGCAGGCTATGAGGCGGTGCTGTTGTG	Tb927.9.5020_rev	GGGGACCACTTTGTACAAGAAAGCTGGGTTCATTCTTTCCCTTTTTTCTTGCTTGTAG
6_hypothetical	A6	Tb927.8.6960_for	GGGGACAAGTTTGTACAAAAAAGCAGGCTATGTACGCTGTGAAACCGTGG	Tb927.8.6960_rev	GGGGACCACTTTGTACAAGAAAGCTGGGTTTACTTTGGCAATTGAGAGAACGTGC
6_hypothetical	A7	Tb927.11.4840_for	GGGGACAAGTTTGTACAAAAAAGCAGGCTATGATTGTTAGGGCGAAGTCCCT	Tb927.11.4840_rev	GGGGACCACTTTGTACAAGAAAGCTGGGTCTACTGCTGGCGGCTGGA
6_hypothetical	A8	Tb927.6.1540_for	GGGGACAAGTTTGTACAAAAAAGCAGGCTATGGCAGCCAGTAGAAGTGATGA	Tb927.6.1540_rev	GGGGACCACTTTGTACAAGAAAGCTGGGTCTAAAAGCTGTAGTTAAGGAAGTAAAACTT
6_hypothetical	A9	Tb927.8.3850_for	GGGGACAAGTTTGTACAAAAAAGCAGGCTATGATAGTCAGAACGCCTCTTGCG	Tb927.8.3850_rev	GGGGACCACTTTGTACAAGAAAGCTGGGTTCAAGCGGCTACCGCGCC
6_hypothetical	A10	Tb927.9.14200_for	GGGGACAAGTTTGTACAAAAAAGCAGGCTATGTTCCGGCGGTTTATTCCATG	Tb927.9.14200_rev	GGGGACCACTTTGTACAAGAAAGCTGGGTCTATTCCCTGGGTTTTTGGACGTC
6_hypothetical	A11	Tb927.10.16110_for	GGGGACAAGTTTGTACAAAAAAGCAGGCTATGTCTTACTTGACAGAGGAGGAGG	Tb927.10.16110_rev	GGGGACCACTTTGTACAAGAAAGCTGGGTTCATTGTACGAGCGACAACTTGG
6_hypothetical	A12	Tb927.5.4150_for	GGGGACAAGTTTGTACAAAAAAGCAGGCTATGATAACACTAAAGACTGCTGTAAACACC	Tb927.5.4150_rev	GGGGACCACTTTGTACAAGAAAGCTGGGTCTAAAAAAGTTTCCTTGGTTTCTTATCCAC
6_hypothetical	A13	Tb927.2.4670_for	GGGGACAAGTTTGTACAAAAAAGCAGGCTATGGAGTCAAACAAGAGAGTAACTGGG	Tb927.2.4670_rev	GGGGACCACTTTGTACAAGAAAGCTGGGTCTACACGTCAGCGATGGCTTC
6_hypothetical	A14	Tb927.11.6800_for	GGGGACAAGTTTGTACAAAAAAGCAGGCTATGGCCGTTGCCACTTCG	Tb927.11.6800_rev	GGGGACCACTTTGTACAAGAAAGCTGGGTTCATGATCCGCGTGAAGTTGAAC
6_hypothetical	A15	Tb927.7.3060_for	GGGGACAAGTTTGTACAAAAAAGCAGGCTATGTTTTGTTTCTTCCAAAACCGCTG	Tb927.7.3060_rev	GGGGACCACTTTGTACAAGAAAGCTGGGTCTAAAGGGCGGCTTCAAGTCC
6_hypothetical	A16	Tb927.9.12000_for	GGGGACAAGTTTGTACAAAAAAGCAGGCTATGTCTTTCGGTAGGTTATTGAAGCAG	Tb927.9.12000_rev	GGGGACCACTTTGTACAAGAAAGCTGGGTTTATTTTGTTAGCAATCGCTCCACGA
6_hypothetical	A17	Tb927.6.2440_for	GGGGACAAGTTTGTACAAAAAAGCAGGCTATGCGCAAGAAGAGCGTGC	Tb927.6.2440_rev	GGGGACCACTTTGTACAAGAAAGCTGGGTCTACGCCGACGCATTCAACA
6_hypothetical	A18	Tb927.3.2700_for	GGGGACAAGTTTGTACAAAAAAGCAGGCTATGATCCTTTCAGAAGGCGTTCC	Tb927.3.2700_rev	GGGGACCACTTTGTACAAGAAAGCTGGGTCTAGGCGAATGCCTCCAAAATACC
6_hypothetical	A19	Tb927.3.4460_for	GGGGACAAGTTTGTACAAAAAAGCAGGCTATGATAGCTGAACAACAGAACCAGG	Tb927.3.4460_rev	GGGGACCACTTTGTACAAGAAAGCTGGGTTTACCAAAAGGGAACACTTTCATATCCC
6_hypothetical	A20	Tb927.2.2230_for	GGGGACAAGTTTGTACAAAAAAGCAGGCTATGCGGCGCGGTGGTGCG	Tb927.2.2230_rev	GGGGACCACTTTGTACAAGAAAGCTGGGTTTATTTACGTCGTCGGTTGTTGTGC
6_hypothetical	A21	Tb927.7.6720_for	GGGGACAAGTTTGTACAAAAAAGCAGGCTATGGGGTGTGGCGATTCGC	Tb927.7.6720_rev	GGGGACCACTTTGTACAAGAAAGCTGGGTTTAGCGACTAATGTGAACCACATGG
6_hypothetical	A22	Tb927.10.12020_for	GGGGACAAGTTTGTACAAAAAAGCAGGCTATGTCTGTGTGTCCTCCACCA	Tb927.10.12020_rev	GGGGACCACTTTGTACAAGAAAGCTGGGTTCAGGGTAAAAAGATGTTTCCCACG
6_hypothetical	A23	Tb927.10.11380_for	GGGGACAAGTTTGTACAAAAAAGCAGGCTATGGATTTGTCCGGCCTAGTGC	Tb927.10.11380_rev	GGGGACCACTTTGTACAAGAAAGCTGGGTCTACCTTAACATCTGAACATCCCACTC
6_hypothetical	A24	Tb927.4.2240_for	GGGGACAAGTTTGTACAAAAAAGCAGGCTATGCAGACCGGCCCATTCG	Tb927.4.2240_rev	GGGGACCACTTTGTACAAGAAAGCTGGGTTTAAAGAAGAGCCTCCGCTTCATAGG
6_hypothetical	B1	Tb927.2.5130_for	GGGGACAAGTTTGTACAAAAAAGCAGGCTATGGTGGTTACTTTGGTGGAACC	Tb927.2.5130_rev	GGGGACCACTTTGTACAAGAAAGCTGGGTTTACCATTCACGGAATCTCTTGGAAAC
6_hypothetical	B2	Tb927.3.1140_for	GGGGACAAGTTTGTACAAAAAAGCAGGCTATGTCTGGTGAGGCCGATTCT	Tb927.3.1140_rev	GGGGACCACTTTGTACAAGAAAGCTGGGTTCACGCAGAGTTATTTCCGTCCA
6_hypothetical	B3	Tb927.10.15420_for	GGGGACAAGTTTGTACAAAAAAGCAGGCTATGCCATCTCCAATTGTGCTCAC	Tb927.10.15420_rev	GGGGACCACTTTGTACAAGAAAGCTGGGTCTAACTCTCCTTTTTCTGGAAGGGAGG
6_hypothetical	B4	Tb927.3.3370_for	GGGGACAAGTTTGTACAAAAAAGCAGGCTATGTCTGAGGTGGTACCTACTCGT	Tb927.3.3370_rev	GGGGACCACTTTGTACAAGAAAGCTGGGTTCAGCTTCGCTTTGAGTCATCC
6_hypothetical	B5	Tb927.8.3810_for	GGGGACAAGTTTGTACAAAAAAGCAGGCTATGAGAGCAAGTATGAATTGCTCCG	Tb927.8.3810_rev	GGGGACCACTTTGTACAAGAAAGCTGGGTCTACGGGTCTCCGAATGTGCG
6_hypothetical	B6	Tb927.11.3160_for	GGGGACAAGTTTGTACAAAAAAGCAGGCTATGTCCCGCCTGTTCCTCG	Tb927.11.3160_rev	GGGGACCACTTTGTACAAGAAAGCTGGGTTTAAAAGTCGGGGGAACAACCAG
6_hypothetical	B7	Tb927.11.520_for	GGGGACAAGTTTGTACAAAAAAGCAGGCTATGAGCAATGGTGGTGTTACTGC	Tb927.11.520_rev	GGGGACCACTTTGTACAAGAAAGCTGGGTTCATTGGAGGCCACTCCTCAA
6_hypothetical	B8	Tb927.6.1920_for	GGGGACAAGTTTGTACAAAAAAGCAGGCTATGTCCTACCGGAGGCCTACC	Tb927.6.1920_rev	GGGGACCACTTTGTACAAGAAAGCTGGGTTCAGTCCGCCCGACTTGT
6_hypothetical	B9	Tb927.10.4750_for	GGGGACAAGTTTGTACAAAAAAGCAGGCTATGGCGGTGCGAGCTCTG	Tb927.10.4750_rev	GGGGACCACTTTGTACAAGAAAGCTGGGTTTATTCCCATTCATTAGATGGATCGTAACG
6_hypothetical	B10	Tb927.7.5120_for	GGGGACAAGTTTGTACAAAAAAGCAGGCTATGCAGAGATGGAGCCAGAGAC	Tb927.7.5120_rev	GGGGACCACTTTGTACAAGAAAGCTGGGTTTAGTGATTCCTATACACCTCCACAGC
6_hypothetical	B11	Tb927.6.3860_for	GGGGACAAGTTTGTACAAAAAAGCAGGCTATGGATGTGTGTGGTTTGTCGC	Tb927.6.3860_rev	GGGGACCACTTTGTACAAGAAAGCTGGGTCTAACAGCGAACAACAGCATTGG
6_hypothetical	B12	Tb927.10.14660_for	GGGGACAAGTTTGTACAAAAAAGCAGGCTATGGAGGGCCTGTCGCCC	Tb927.10.14660_rev	GGGGACCACTTTGTACAAGAAAGCTGGGTTCATCTGTCTTCAGTTCTCTGCTTGT
6_hypothetical	B13	Tb927.5.850_for	GGGGACAAGTTTGTACAAAAAAGCAGGCTATGAGCGCCCAGGTGCCC	Tb927.5.850_rev	GGGGACCACTTTGTACAAGAAAGCTGGGTTCAGTCAATGGAAATCGTGTGAAGC
6_hypothetical	B14	Tb927.7.3170_for	GGGGACAAGTTTGTACAAAAAAGCAGGCTATGATGAGCAGCGGGAACCA	Tb927.7.3170_rev	GGGGACCACTTTGTACAAGAAAGCTGGGTTCACGACAGAGGAAGTGCCA
6_hypothetical	B15	Tb927.11.7213_for	GGGGACAAGTTTGTACAAAAAAGCAGGCTATGATAGGCCTCCGGAAACGC	Tb927.11.7213_rev	GGGGACCACTTTGTACAAGAAAGCTGGGTTTACACAAGTCGTGTCAGACGC
6_hypothetical	B16	Tb927.7.2520_for	GGGGACAAGTTTGTACAAAAAAGCAGGCTATGGAGGAGAGTACTGAAAGATCACTT	Tb927.7.2520_rev	GGGGACCACTTTGTACAAGAAAGCTGGGTTCACGCCACTGCTTCCTCT
6_hypothetical	B17	Tb927.11.9840_for	GGGGACAAGTTTGTACAAAAAAGCAGGCTATGCTAGATTTTCTCGACACGATTAACA	Tb927.11.9840_rev	GGGGACCACTTTGTACAAGAAAGCTGGGTTCACTGACAGTCGGAATGGCT
6_hypothetical	B18	Tb927.8.6140_for	GGGGACAAGTTTGTACAAAAAAGCAGGCTATGGCTCTATGTAACAGTCCTTCACG	Tb927.8.6140_rev	GGGGACCACTTTGTACAAGAAAGCTGGGTTCATTCATGCTGAGAGTTGCTGTC
6_hypothetical	B19	Tb927.3.1450_for	GGGGACAAGTTTGTACAAAAAAGCAGGCTATGCCGTGTGCCACTAAAGG	Tb927.3.1450_rev	GGGGACCACTTTGTACAAGAAAGCTGGGTTTATCCACCCTTTTCCCTGATGAGG
6_hypothetical	B20	Tb927.10.4860_for	GGGGACAAGTTTGTACAAAAAAGCAGGCTATGAGCGGGATGCAGGCA	Tb927.10.4860_rev	GGGGACCACTTTGTACAAGAAAGCTGGGTTTATATGAGGCCGACAGTCTTCAAATATTG
6_hypothetical	B21	Tb927.7.2610_for	GGGGACAAGTTTGTACAAAAAAGCAGGCTATGGGTAGCGCTTCTCAGGT	Tb927.7.2610_rev	GGGGACCACTTTGTACAAGAAAGCTGGGTTTATCTCAAATCCATCTCTTCACCTGTG
6_hypothetical	B22	Tb927.11.12580_for	GGGGACAAGTTTGTACAAAAAAGCAGGCTATGGGTGCCAACAACATAAGGG	Tb927.11.12580_rev	GGGGACCACTTTGTACAAGAAAGCTGGGTCTAATCCGACTTATCGCTGCCTTC
6_hypothetical	B24	Tb927.5.410_for	GGGGACAAGTTTGTACAAAAAAGCAGGCTATGCATATGTATTTCATGCGTGTGAAG	Tb927.5.410_rev	GGGGACCACTTTGTACAAGAAAGCTGGGTTCACTCCCTCAGGTAACTTGGTG
6_hypothetical	C1	Tb927.5.309b_for	GGGGACAAGTTTGTACAAAAAAGCAGGCTATGGCACGTGTCACCTTTTGC	Tb927.5.309b_rev	GGGGACCACTTTGTACAAGAAAGCTGGGTCTACAGTTTGGGCTGCGCA
6_hypothetical	C2	Tb927.10.9950_for	GGGGACAAGTTTGTACAAAAAAGCAGGCTATGACGGAGCGTACCGACA	Tb927.10.9950_rev	GGGGACCACTTTGTACAAGAAAGCTGGGTTTAGCGAATGATCCAGTTGGCTAAC
6_hypothetical	C3	Tb927.8.6780_for	GGGGACAAGTTTGTACAAAAAAGCAGGCTATGTTGAGAGGGCGTGCCA	Tb927.8.6780_rev	GGGGACCACTTTGTACAAGAAAGCTGGGTCTAAAGATTAGATGACAACAGCGGCA
6_hypothetical	C4	Tb927.8.1900_for	GGGGACAAGTTTGTACAAAAAAGCAGGCTATGCACGAAGAGGACCTTATGAAGT	Tb927.8.1900_rev	GGGGACCACTTTGTACAAGAAAGCTGGGTCTACTTTTTAATCCCTGGAGCACAAGG
6_hypothetical	C5	Tb927.11.12440_for	GGGGACAAGTTTGTACAAAAAAGCAGGCTATGAGAGTAGAGGAGCAACATCAGT	Tb927.11.12440_rev	GGGGACCACTTTGTACAAGAAAGCTGGGTTCAAATTTCTTTGGGCTCAAACAGTTC
6_hypothetical	C6	Tb927.6.5000_for	GGGGACAAGTTTGTACAAAAAAGCAGGCTATGGAACAACAAACCATATCATTGCAC	Tb927.6.5000_rev	GGGGACCACTTTGTACAAGAAAGCTGGGTTCACAAAAATTTGTTGATATCGCCCAC
6_hypothetical	C7	Tb927.9.12710_for	GGGGACAAGTTTGTACAAAAAAGCAGGCTATGCTCGGTGCAGGCACG	Tb927.9.12710_rev	GGGGACCACTTTGTACAAGAAAGCTGGGTTTACGTTACTCTCAGCATCTCCACG
6_hypothetical	C8	Tb927.11.12940_for	GGGGACAAGTTTGTACAAAAAAGCAGGCTATGTCTCGGGGGCATATGGC	Tb927.11.12940_rev	GGGGACCACTTTGTACAAGAAAGCTGGGTCTCACACAGCCACGAGTGCC
6_hypothetical	C9	Tb927.3.4700_for	GGGGACAAGTTTGTACAAAAAAGCAGGCTATGTTGAGACAACTCCTCCACCA	Tb927.3.4700_rev	GGGGACCACTTTGTACAAGAAAGCTGGGTTCAGGACTTCCCTTTTCCGTGG
6_hypothetical	C10	Tb927.10.14450_for	GGGGACAAGTTTGTACAAAAAAGCAGGCTATGAGTACGGAGCCGCACA	Tb927.10.14450_rev	GGGGACCACTTTGTACAAGAAAGCTGGGTTCATCGAAACTTTTGTGACACCAATG
6_hypothetical	C11	Tb927.3.2500_for	GGGGACAAGTTTGTACAAAAAAGCAGGCTATGAGGAGTTACATCGTGTTTGTTGC	Tb927.3.2500_rev	GGGGACCACTTTGTACAAGAAAGCTGGGTTTAAAAAACTAGAAAAAATAACTTAAGTGG
6_hypothetical	C12	Tb927.11.3930_for	GGGGACAAGTTTGTACAAAAAAGCAGGCTATGGCGGGAACTTCTTTACCGT	Tb927.11.3930_rev	GGGGACCACTTTGTACAAGAAAGCTGGGTTTACGGCTCAAACGATGCTGC
6_hypothetical	C13	Tb927.7.620_for	GGGGACAAGTTTGTACAAAAAAGCAGGCTATGAGTGGTTCGGCCGGT	Tb927.7.620_rev	GGGGACCACTTTGTACAAGAAAGCTGGGTTCATCCTCCCATTGACGAAGTTGC
6_hypothetical	C14	Tb927.11.6450_for	GGGGACAAGTTTGTACAAAAAAGCAGGCTATGGGTTCGAGTGATGGTACTTCT	Tb927.11.6450_rev	GGGGACCACTTTGTACAAGAAAGCTGGGTTTACCAATCGTCCACAGCAATACC
6_hypothetical	C15	Tb927.10.520_for	GGGGACAAGTTTGTACAAAAAAGCAGGCTATGCAGGGCAGTTGGTCGG	Tb927.10.520_rev	GGGGACCACTTTGTACAAGAAAGCTGGGTCTAAGCTGTGTGTCGGCCC
6_hypothetical	C16	Tb927.10.6710_for	GGGGACAAGTTTGTACAAAAAAGCAGGCTATGGAAGCTGTTGTGTATGAGATCG	Tb927.10.6710_rev	GGGGACCACTTTGTACAAGAAAGCTGGGTTTAAGACCCGCAGTCTTCATACCA
6_hypothetical	C17	Tb927.6.2080_for	GGGGACAAGTTTGTACAAAAAAGCAGGCTATGTTTCGGTCGACCCGCC	Tb927.6.2080_rev	GGGGACCACTTTGTACAAGAAAGCTGGGTTCACGGGTCACCCGTGTAAC
6_hypothetical	C18	Tb927.11.8230_for	GGGGACAAGTTTGTACAAAAAAGCAGGCTATGGAGTTCTCTGGATCTACATTATCCG	Tb927.11.8230_rev	GGGGACCACTTTGTACAAGAAAGCTGGGTTTACTTCAACTGATCGGAAAACATTTCG
6_hypothetical	C19	Tb927.3.1100_for	GGGGACAAGTTTGTACAAAAAAGCAGGCTATGAGACGCGCTTGTGTACATT	Tb927.3.1100_rev	GGGGACCACTTTGTACAAGAAAGCTGGGTCTAAATGATGAACTCCTCTCCACGATC
6_hypothetical	C20	Tb927.3.1170_for	GGGGACAAGTTTGTACAAAAAAGCAGGCTATGTCTACCGATGGCAGTGAAGT	Tb927.3.1170_rev	GGGGACCACTTTGTACAAGAAAGCTGGGTTCATGCGTAGTTTCCTCTTGCAG
6_hypothetical	C21	Tb927.11.9050_for	GGGGACAAGTTTGTACAAAAAAGCAGGCTATGCCTAAGCGCCACCGT	Tb927.11.9050_rev	GGGGACCACTTTGTACAAGAAAGCTGGGTTCAGTAATGCAGCACACTGGC
6_hypothetical	C22	Tb927.6.4170_for	GGGGACAAGTTTGTACAAAAAAGCAGGCTATGCATCAGACGTTCGCATCG	Tb927.6.4170_rev	GGGGACCACTTTGTACAAGAAAGCTGGGTTCACGGCTCTCCTTCAGCA
6_hypothetical	C23	Tb927.4.3900_for	GGGGACAAGTTTGTACAAAAAAGCAGGCTATGGCGAGTAACTCCTCGGC	Tb927.4.3900_rev	GGGGACCACTTTGTACAAGAAAGCTGGGTTTATTTGGGCTTTTCACGTGATATTCTTTC
6_hypothetical	C24	Tb927.11.15790_for	GGGGACAAGTTTGTACAAAAAAGCAGGCTATGAAGGGCAAGGCAGTTAAAGAAC	Tb927.11.15790_rev	GGGGACCACTTTGTACAAGAAAGCTGGGTTCATTGTGTTACACTTGAGGCTGC
6_hypothetical	D1	Tb927.4.1280_for	GGGGACAAGTTTGTACAAAAAAGCAGGCTATGAGGCGCTTCATGTCCCT	Tb927.4.1280_rev	GGGGACCACTTTGTACAAGAAAGCTGGGTTTACCAACCGTCTTTAGCACGAG
6_hypothetical	D2	Tb927.8.5700_for	GGGGACAAGTTTGTACAAAAAAGCAGGCTATGATGTACCGGTGGAGCCC	Tb927.8.5700_rev	GGGGACCACTTTGTACAAGAAAGCTGGGTTCACGCTGCGCCTATTAATACC
6_hypothetical	D3	Tb927.11.1290_for	GGGGACAAGTTTGTACAAAAAAGCAGGCTATGAAATTGTCACAACGGTTCCTATCA	Tb927.11.1290_rev	GGGGACCACTTTGTACAAGAAAGCTGGGTCTAACGTGTTTGACGCACCAAAAG
6_hypothetical	D4	Tb927.6.4320_for	GGGGACAAGTTTGTACAAAAAAGCAGGCTATGGCGGACGTCGAGGCC	Tb927.6.4320_rev	GGGGACCACTTTGTACAAGAAAGCTGGGTTCAAGTGGACAAATAGTCCATAGCGT
6_hypothetical	D5	Tb927.1.4220_for	GGGGACAAGTTTGTACAAAAAAGCAGGCTATGCGAGCCGTCAAGAGGA	Tb927.1.4220_rev	GGGGACCACTTTGTACAAGAAAGCTGGGTCTATGTTTCTGCAGTCCCGCAT
6_hypothetical	D6	Tb927.9.9730_for	GGGGACAAGTTTGTACAAAAAAGCAGGCTATGCCCGTAACGAAGCCAAG	Tb927.9.9730_rev	GGGGACCACTTTGTACAAGAAAGCTGGGTTTAAGCCGCTGCACGCTT
6_hypothetical	D7	Tb927.11.10320_for	GGGGACAAGTTTGTACAAAAAAGCAGGCTATGATACATCTCCCAACATCAACTACAC	Tb927.11.10320_rev	GGGGACCACTTTGTACAAGAAAGCTGGGTCTAAACACCAGGTAACACACCCG
6_hypothetical	D8	Tb927.11.1840_for	GGGGACAAGTTTGTACAAAAAAGCAGGCTATGGAAACAGCAGAGTTAGCAAGAG	Tb927.11.1840_rev	GGGGACCACTTTGTACAAGAAAGCTGGGTCTACTGGCTTAGCTTTGAGCGG
6_hypothetical	D9	Tb927.9.2260_for	GGGGACAAGTTTGTACAAAAAAGCAGGCTATGGAAACAACACCAACGTTTTCTG	Tb927.9.2260_rev	GGGGACCACTTTGTACAAGAAAGCTGGGTTCATTCCCTACGTACGGGACTC
6_hypothetical	D10	Tb927.3.2240_for	GGGGACAAGTTTGTACAAAAAAGCAGGCTATGTTACTTAGGTTTGTCACTTTTGCCT	Tb927.3.2240_rev	GGGGACCACTTTGTACAAGAAAGCTGGGTTTAAACCCCAATAGAGATCTTAACTGGAGG
6_hypothetical	D11	Tb927.11.9870_for	GGGGACAAGTTTGTACAAAAAAGCAGGCTATGGAGTCATCGCTCGGTGC	Tb927.11.9870_rev	GGGGACCACTTTGTACAAGAAAGCTGGGTTCAAGACAGGGGTATGTCCTCG

6_hypothetical	D12	Tb927.11.6340_for	GGGGACAAGTTTGTACAAAAAAGCAGGCTATGTCATCCCTCTCTGGAGAGC	Tb927.11.6340_rev	GGGGACCACTTTGTACAAGAAAGCTGGGTTTACTCTTCTCGGTACCCGAACC
6_hypothetical	D13	Tb927.9.3100_for	GGGGACAAGTTTGTACAAAAAAGCAGGCTATGAGACGTACGATTGTCCAACTTTG	Tb927.9.3100_rev	GGGGACCACTTTGTACAAGAAAGCTGGGTTCACCGCTGGCTGGATGC
6_hypothetical	D14	Tb927.11.710_for	GGGGACAAGTTTGTACAAAAAAGCAGGCTATGTTTGGTCGGGGATTTGGTG	Tb927.11.710_rev	GGGGACCACTTTGTACAAGAAAGCTGGGTCTACACACCAATTACATTATCAATATGACT
6_hypothetical	D15	Tb927.5.660_for	GGGGACAAGTTTGTACAAAAAAGCAGGCTATGTTGGGGATGAGGAGTTACATCG	Tb927.5.660_rev	GGGGACCACTTTGTACAAGAAAGCTGGGTTTAAAAAACTAGAAAAAATAACTTAAGTGG
6_hypothetical	D16	Tb927.4.3760_for	GGGGACAAGTTTGTACAAAAAAGCAGGCTATGGTAAGAAACATGGGTGGAAATGC	Tb927.4.3760_rev	GGGGACCACTTTGTACAAGAAAGCTGGGTTCATCCTTCACTTTGAATTTCGTTTCTTTT
6_hypothetical	D17	Tb927.8.3320_for	GGGGACAAGTTTGTACAAAAAAGCAGGCTATGTGGAGGTTTGGTGCCTCC	Tb927.8.3320_rev	GGGGACCACTTTGTACAAGAAAGCTGGGTCTACTCTTCAATTTCATTCATACGCTTCTG
6_hypothetical	D18	Tb927.7.1480_for	GGGGACAAGTTTGTACAAAAAAGCAGGCTATGGTTGTAAATGAGGCTCCGTG	Tb927.7.1480_rev	GGGGACCACTTTGTACAAGAAAGCTGGGTTCACAGTCCCTCCACAAGAAGC
6_hypothetical	D19	Tb927.10.12320_for	GGGGACAAGTTTGTACAAAAAAGCAGGCTATGTTGAGTAGTGGAGAGCGCA	Tb927.10.12320_rev	GGGGACCACTTTGTACAAGAAAGCTGGGTTTAGTCCCCAAAGAGGGCTTGT
6_hypothetical	D20	Tb927.10.6340_for	GGGGACAAGTTTGTACAAAAAAGCAGGCTATGATGAGTCGAAGGCCGCC	Tb927.10.6340_rev	GGGGACCACTTTGTACAAGAAAGCTGGGTCTAAAAGCGCTTGGGCCGT
6_hypothetical	D21	Tb927.1.2770_for	GGGGACAAGTTTGTACAAAAAAGCAGGCTATGTCGGTCAGTTCCCCGC	Tb927.1.2770_rev	GGGGACCACTTTGTACAAGAAAGCTGGGTTCACATCAAGTGAAGCGCTCG
6_hypothetical	D22	Tb927.8.4140_for	GGGGACAAGTTTGTACAAAAAAGCAGGCTATGATTACAGAAGAACTCTTTCGATCCTTC	Tb927.8.4140_rev	GGGGACCACTTTGTACAAGAAAGCTGGGTCTAAACGTGCCGGTCGTCTAAA
6_hypothetical	D23	Tb927.11.6560_for	GGGGACAAGTTTGTACAAAAAAGCAGGCTATGCCCACTGCAGTTAGGCA	Tb927.11.6560_rev	GGGGACCACTTTGTACAAGAAAGCTGGGTCTAACTGGAGAACCACATTCGTCTC
6_hypothetical	D24	Tb927.7.1760_for	GGGGACAAGTTTGTACAAAAAAGCAGGCTATGGAGCATCTAATCGCTGCG	Tb927.7.1760_rev	GGGGACCACTTTGTACAAGAAAGCTGGGTCTAATCAAGCCATATATCAAGTGGTTCTTG
6_hypothetical	E1	Tb927.5.2030_for	GGGGACAAGTTTGTACAAAAAAGCAGGCTATGTTTCGCTGCACTGTGAGG	Tb927.5.2030_rev	GGGGACCACTTTGTACAAGAAAGCTGGGTTCAACCGCCGTCGTGGCC
6_hypothetical	E2	Tb927.8.7270_for	GGGGACAAGTTTGTACAAAAAAGCAGGCTATGAAGGGCCTCAGCCGC	Tb927.8.7270_rev	GGGGACCACTTTGTACAAGAAAGCTGGGTTCAAAACTGCTTCCCTCTGTTTATCTT
6_hypothetical	E3	Tb927.2.2910_for	GGGGACAAGTTTGTACAAAAAAGCAGGCTATGGTCTCCACGCGAAGAATAGC	Tb927.2.2910_rev	GGGGACCACTTTGTACAAGAAAGCTGGGTCAAACATCATTAATATTCCATAATGAAAC
6_hypothetical	E4	Tb927.6.2240_for	GGGGACAAGTTTGTACAAAAAAGCAGGCTATGTCTGGGCTATGGAGTTTTAGGG	Tb927.6.2240_rev	GGGGACCACTTTGTACAAGAAAGCTGGGTTTATGAATGTATGGGACCAAATCTTCCTG
6_hypothetical	E5	Tb927.9.12950_for	GGGGACAAGTTTGTACAAAAAAGCAGGCTATGGAAACGAAAGGTTCTTCTGTTCA	Tb927.9.12950_rev	GGGGACCACTTTGTACAAGAAAGCTGGGTTTAATCGTCAACACTAAGTCTTTCCCAC
6_hypothetical	E6	Tb927.4.5100_for	GGGGACAAGTTTGTACAAAAAAGCAGGCTATGAAAGGCCTCAGCCGCC	Tb927.4.5100_rev	GGGGACCACTTTGTACAAGAAAGCTGGGTTCAAAACTGCTTCCCTCTATTCATCTTTAT
6_hypothetical	E7	Tb927.9.9030_for	GGGGACAAGTTTGTACAAAAAAGCAGGCTATGAGTGAAGACGGCTCCGA	Tb927.9.9030_rev	GGGGACCACTTTGTACAAGAAAGCTGGGTTCAAGCCTTCACTCCGTCTTTTC
6_hypothetical	E8	Tb927.4.370_for	GGGGACAAGTTTGTACAAAAAAGCAGGCTATGGTGATAGATGGTGACTTAGTTGCC	Tb927.4.370_rev	GGGGACCACTTTGTACAAGAAAGCTGGGTTCACAAATGAATACGAATGGATGGTAACA
6_hypothetical	E9	Tb927.10.800_for	GGGGACAAGTTTGTACAAAAAAGCAGGCTATGCATCCGGAAATTGAAGCACT	Tb927.10.800_rev	GGGGACCACTTTGTACAAGAAAGCTGGGTTTAATCAATCTCGAATATATCTGGGTCATT
6_hypothetical	E10	Tb927.3.3820_for	GGGGACAAGTTTGTACAAAAAAGCAGGCTATGTACCAAAGCAGAACAATGGTATCTT	Tb927.3.3820_rev	GGGGACCACTTTGTACAAGAAAGCTGGGTTTATCCATCAAGTTTCCAACTCCATTCC
6_hypothetical	E11	Tb927.11.16500_for	GGGGACAAGTTTGTACAAAAAAGCAGGCTATGTGCCTTCCGATCATCCGC	Tb927.11.16500_rev	GGGGACCACTTTGTACAAGAAAGCTGGGTTTAGGGGTTTACGGTACCTTCCAG
6_hypothetical	E12	Tb927.2.5000_for	GGGGACAAGTTTGTACAAAAAAGCAGGCTATGGCAACAAGAGGTGTGGC	Tb927.2.5000_rev	GGGGACCACTTTGTACAAGAAAGCTGGGTTCACTTGTGTTGAAGCCTGAGAGC
6_hypothetical	E13	Tb927.9.1950_for	GGGGACAAGTTTGTACAAAAAAGCAGGCTATGTATCGTAGGCTATGTGCGAGA	Tb927.9.1950_rev	GGGGACCACTTTGTACAAGAAAGCTGGGTCTAGTGTAGACCGTACCCACTCTC
6_hypothetical	E14	Tb927.8.1460_for	GGGGACAAGTTTGTACAAAAAAGCAGGCTATGGCATCCCAAAATGCAGCA	Tb927.8.1460_rev	GGGGACCACTTTGTACAAGAAAGCTGGGTCTAACTGGAGCGCGAACCC
6_hypothetical	E15	Tb927.6.4560_for	GGGGACAAGTTTGTACAAAAAAGCAGGCTATGCGAAAGTTTTGTCATTTCATGGC	Tb927.6.4560_rev	GGGGACCACTTTGTACAAGAAAGCTGGGTTTACAAAGTGAGCCATTCCTCCTCG
6_hypothetical	E16	Tb927.7.4510_for	GGGGACAAGTTTGTACAAAAAAGCAGGCTATGTACGAGTATGATGACCAGCAGG	Tb927.7.4510_rev	GGGGACCACTTTGTACAAGAAAGCTGGGTTTAACTTGCCAGAGACTCTTTTGCTT
6_hypothetical	E17	Tb927.5.2770_for	GGGGACAAGTTTGTACAAAAAAGCAGGCTATGACGGGGGCGGACGAG	Tb927.5.2770_rev	GGGGACCACTTTGTACAAGAAAGCTGGGTTTACCACTGGCGTCCATTTCG
6_hypothetical	E18	Tb927.10.10000_for	GGGGACAAGTTTGTACAAAAAAGCAGGCTATGCAACGCTGCACACCA	Tb927.10.10000_rev	GGGGACCACTTTGTACAAGAAAGCTGGGTTCAATAACTGCCGAGTACACGC
6_hypothetical	E19	Tb927.5.310_for	GGGGACAAGTTTGTACAAAAAAGCAGGCTATGGCACGTGTCACCTTTTGC	Tb927.5.310_rev	GGGGACCACTTTGTACAAGAAAGCTGGGTTTACATTTTCACGGAATGGTATGGATCC
6_hypothetical	E20	Tb927.4.2120_for	GGGGACAAGTTTGTACAAAAAAGCAGGCTATGGAATCACCAACGTGCGG	Tb927.4.2120_rev	GGGGACCACTTTGTACAAGAAAGCTGGGTCTACGAACCCCCTCCCCG
6_hypothetical	E21	Tb927.11.5660_for	GGGGACAAGTTTGTACAAAAAAGCAGGCTATGATGACGCAAAACGTGAAACAG	Tb927.11.5660_rev	GGGGACCACTTTGTACAAGAAAGCTGGGTTCACATCTTGCAGCCTTTTTGAAAC
6_hypothetical	E22	Tb927.3.4180_for	GGGGACAAGTTTGTACAAAAAAGCAGGCTATGCCGGCAGAGGGTCCA	Tb927.3.4180_rev	GGGGACCACTTTGTACAAGAAAGCTGGGTTTAAAGGCTTGCATTTCCATTGCG
6_hypothetical	E23	Tb927.11.10690_for	GGGGACAAGTTTGTACAAAAAAGCAGGCTATGAAGAGGCAAAGAACTGCATCC	Tb927.11.10690_rev	GGGGACCACTTTGTACAAGAAAGCTGGGTTCAGCTGCATTCAGCGAATCC
6_hypothetical	E24	Tb927.5.4280_for	GGGGACAAGTTTGTACAAAAAAGCAGGCTATGCCCCCCAAAAAGGGTAAAAA	Tb927.5.4280_rev	GGGGACCACTTTGTACAAGAAAGCTGGGTTTAGTATGCACGATACACAATATCCATGTC
6_hypothetical	F1	Tb927.3.1280_for	GGGGACAAGTTTGTACAAAAAAGCAGGCTATGGCTGATGCTTCTGCACTTG	Tb927.3.1280_rev	GGGGACCACTTTGTACAAGAAAGCTGGGTCTAACTTTCGATGAGCAACTGCAGT
6_hypothetical	F2	Tb927.7.2770_for	GGGGACAAGTTTGTACAAAAAAGCAGGCTATGTTTAGGAAACGTTACAGACCCC	Tb927.7.2770_rev	GGGGACCACTTTGTACAAGAAAGCTGGGTTCACACATGAGCAAGCCACTG
6_hypothetical	F3	Tb927.10.8130_for	GGGGACAAGTTTGTACAAAAAAGCAGGCTATGAGTGGTTGGTTTGGTTCGC	Tb927.10.8130_rev	GGGGACCACTTTGTACAAGAAAGCTGGGTCTATGGTTGCGTCCCAGCAG
6_hypothetical	F4	Tb927.11.16490_for	GGGGACAAGTTTGTACAAAAAAGCAGGCTATGCCAGCAAAACGCAAAGGC	Tb927.11.16490_rev	GGGGACCACTTTGTACAAGAAAGCTGGGTTCAGACATTGAAAATAGCAGCACCA
6_hypothetical	F5	Tb927.8.3960_for	GGGGACAAGTTTGTACAAAAAAGCAGGCTATGCGCTTTTTTGTTGTGGCAG	Tb927.8.3960_rev	GGGGACCACTTTGTACAAGAAAGCTGGGTCTATTCACGCTCCCTTTTGTCTCG
6_hypothetical	F6	Tb927.11.2560_for	GGGGACAAGTTTGTACAAAAAAGCAGGCTATGACAGATGAAAAAAAAGAGGAATGGATT	Tb927.11.2560_rev	GGGGACCACTTTGTACAAGAAAGCTGGGTTCAACAAAAAAAACTTTTTTGCAACTCCA
6_hypothetical	F7	Tb11.02.5430_for	GGGGACAAGTTTGTACAAAAAAGCAGGCTATGACACCACCAACGACTCGA	Tb11.02.5430_rev	GGGGACCACTTTGTACAAGAAAGCTGGGTTTACTCAGCACCCGCAGCG
6_hypothetical	F8	Tb927.10.11830_for	GGGGACAAGTTTGTACAAAAAAGCAGGCTATGCCTGCGCTCAGTTGG	Tb927.10.11830_rev	GGGGACCACTTTGTACAAGAAAGCTGGGTCTACACCAATCGTGGTTGCTGA
6_hypothetical	F9	Tb927.9.2816_for	GGGGACAAGTTTGTACAAAAAAGCAGGCTATGGAAATCCGCATGGTGCC	Tb927.9.2816_rev	GGGGACCACTTTGTACAAGAAAGCTGGGTTCACTCATCAGCTGCTGGAGT
6_hypothetical	F10	Tb927.10.11560_for	GGGGACAAGTTTGTACAAAAAAGCAGGCTATGGTTGGCGAAGATTCTAATTTAGGG	Tb927.10.11560_rev	GGGGACCACTTTGTACAAGAAAGCTGGGTTTAGAGACCAAGCCGCGACA
6_hypothetical	F11	Tb927.10.9920_for	GGGGACAAGTTTGTACAAAAAAGCAGGCTATGTCCTCACGAGCATGGGT	Tb927.10.9920_rev	GGGGACCACTTTGTACAAGAAAGCTGGGTTTAGAGCGCATAGTTTGTCACTCG
6_hypothetical	F12	Tb927.5.2540_for	GGGGACAAGTTTGTACAAAAAAGCAGGCTATGTCTGATTCTTCTCTGGTGTTCCT	Tb927.5.2540_rev	GGGGACCACTTTGTACAAGAAAGCTGGGTTCACTCCTCATCCATAATGTCCAGC
6_hypothetical	F13	Tb927.1.2700_for	GGGGACAAGTTTGTACAAAAAAGCAGGCTATGGAACAGTGCTGCGGGG	Tb927.1.2700_rev	GGGGACCACTTTGTACAAGAAAGCTGGGTTTACCACGGACACGACACCA
6_hypothetical	F14	Tb927.3.1300_for	GGGGACAAGTTTGTACAAAAAAGCAGGCTATGTGGTTCACAAACATTACGAAGGA	Tb927.3.1300_rev	GGGGACCACTTTGTACAAGAAAGCTGGGTCTACTTTACATTTGTCCAACCATCATTGTC
6_hypothetical	F15	Tb927.9.2802_for	GGGGACAAGTTTGTACAAAAAAGCAGGCTATGGAAATCCGCATGGTGCC	Tb927.9.2802_rev	GGGGACCACTTTGTACAAGAAAGCTGGGTTCACTCATCAGCTGCTGGAGT
6_hypothetical	F16	Tb927.2.4680_for	GGGGACAAGTTTGTACAAAAAAGCAGGCTATGCCCTCGGGATTCTTCCAG	Tb927.2.4680_rev	GGGGACCACTTTGTACAAGAAAGCTGGGTTCACCTCCTCTCTGTTATTTGTAGGTAA
6_hypothetical	F17	Tb927.11.10300_for	GGGGACAAGTTTGTACAAAAAAGCAGGCTATGAGCCACCCACAGCAGC	Tb927.11.10300_rev	GGGGACCACTTTGTACAAGAAAGCTGGGTTCATGATTCCGCCCTGAAGCG
6_hypothetical	F18	Tb927.2.4380_for	GGGGACAAGTTTGTACAAAAAAGCAGGCTATGAAGAGATTTGGAATTTGGACCCG	Tb927.2.4380_rev	GGGGACCACTTTGTACAAGAAAGCTGGGTTCAGTTTTGATCAATGCCAGCGC
6_hypothetical	F19	Tb927.5.3630_for	GGGGACAAGTTTGTACAAAAAAGCAGGCTATGGAAAAAGTAACGCCAACTTCCC	Tb927.5.3630_rev	GGGGACCACTTTGTACAAGAAAGCTGGGTTCAATATTTCTGGAGGGTACTCCACG
6_hypothetical	F20	Tb927.11.16040_for	GGGGACAAGTTTGTACAAAAAAGCAGGCTATGGACTCGGACGAAGGGG	Tb927.11.16040_rev	GGGGACCACTTTGTACAAGAAAGCTGGGTTCATAAACTGCCTACACCACCCA
6_hypothetical	F21	Tb927.4.840_for	GGGGACAAGTTTGTACAAAAAAGCAGGCTATGAGATGTCTTTTCGCTTCAGCA	Tb927.4.840_rev	GGGGACCACTTTGTACAAGAAAGCTGGGTTCACCACGGCGCACTCAT
6_hypothetical	F22	Tb927.7.7330_for	GGGGACAAGTTTGTACAAAAAAGCAGGCTATGTCTTCAGGCGGAAATGTTGG	Tb927.7.7330_rev	GGGGACCACTTTGTACAAGAAAGCTGGGTTCAAATATGCCGATAGTCGTAGTCCT
6_hypothetical	F23	Tb927.10.2050_for	GGGGACAAGTTTGTACAAAAAAGCAGGCTATGTTCCGAGGTAGTGGGGC	Tb927.10.2050_rev	GGGGACCACTTTGTACAAGAAAGCTGGGTTCAACAAAGTGAGACAACGAAAGATATGG
6_hypothetical	F24	Tb927.8.4800_for	GGGGACAAGTTTGTACAAAAAAGCAGGCTATGACAACGCTAGGTGTAGGAGAC	Tb927.8.4800_rev	GGGGACCACTTTGTACAAGAAAGCTGGGTTCACGTCGTAGCGGTGTCG
6_hypothetical	G1	Tb927.9.12740_for	GGGGACAAGTTTGTACAAAAAAGCAGGCTATGGGTTCAAAGTATGACCGAGTTAAG	Tb927.9.12740_rev	GGGGACCACTTTGTACAAGAAAGCTGGGTTCACGTATCGTTAGTAGGCGACA
6_hypothetical	G2	Tb927.3.5170_for	GGGGACAAGTTTGTACAAAAAAGCAGGCTATGGGGAGGGAGGTTTCTTGG	Tb927.3.5170_rev	GGGGACCACTTTGTACAAGAAAGCTGGGTCTATAGACCGAGTAACCCTCTCACAA
6_hypothetical	G3	Tb927.3.4690_for	GGGGACAAGTTTGTACAAAAAAGCAGGCTATGAAAATTGCGGTTAACCCGTACA	Tb927.3.4690_rev	GGGGACCACTTTGTACAAGAAAGCTGGGTCTAATGTTCCCCTGATTCCTTGTAGTG
6_hypothetical	G4	Tb927.10.9200_for	GGGGACAAGTTTGTACAAAAAAGCAGGCTATGACGCACTATGTTGATAGTGTATTGTAC	Tb927.10.9200_rev	GGGGACCACTTTGTACAAGAAAGCTGGGTTTAGGATCCGTCCGTTTCCCG
6_hypothetical	G5	Tb927.10.6130_for	GGGGACAAGTTTGTACAAAAAAGCAGGCTATGAACAACGGTGGTCCTTACAATG	Tb927.10.6130_rev	GGGGACCACTTTGTACAAGAAAGCTGGGTTTACCGGTACGAGTAACTACGTAACC
6_hypothetical	G6	Tb927.6.4610_for	GGGGACAAGTTTGTACAAAAAAGCAGGCTATGAACTCCGGCACTTGCATTG	Tb927.6.4610_rev	GGGGACCACTTTGTACAAGAAAGCTGGGTTTACTTCTTCATAGTGCTCGCACC
6_hypothetical	G7	Tb927.6.1810_for	GGGGACAAGTTTGTACAAAAAAGCAGGCTATGAATACGATACGTTACCTAGTGTCGG	Tb927.6.1810_rev	GGGGACCACTTTGTACAAGAAAGCTGGGTTCAATCTTTTCTCTCTAACAGCAAATCATT
6_hypothetical	G8	Tb927.7.4750_for	GGGGACAAGTTTGTACAAAAAAGCAGGCTATGTACCATTGTAAAACCAGTGATACCAT	Tb927.7.4750_rev	GGGGACCACTTTGTACAAGAAAGCTGGGTTTATGCTCGCTTGCAGGGTTT
6_hypothetical	G9	Tb927.1.3070_for	GGGGACAAGTTTGTACAAAAAAGCAGGCTATGTCTGCGTGGGCCTCT	Tb927.1.3070_rev	GGGGACCACTTTGTACAAGAAAGCTGGGTTCATGGTGCGGAAGATGTGTG
6_hypothetical	G10	Tb927.8.2720_for	GGGGACAAGTTTGTACAAAAAAGCAGGCTATGAAAACGGTAATCAGGGACCCG	Tb927.8.2720_rev	GGGGACCACTTTGTACAAGAAAGCTGGGTTTACGCATTCAGTATTTCGGCAGC
6_hypothetical	G11	Tb927.11.14570_for	GGGGACAAGTTTGTACAAAAAAGCAGGCTATGAGTTATAGCTGGCGCGGC	Tb927.11.14570_rev	GGGGACCACTTTGTACAAGAAAGCTGGGTCTACTCGTCAATGATATTTGCAAAGCC
6_hypothetical	G12	Tb927.11.9260_for	GGGGACAAGTTTGTACAAAAAAGCAGGCTATGGGATGTCAACAAAGTGGAGTG	Tb927.11.9260_rev	GGGGACCACTTTGTACAAGAAAGCTGGGTTCACGCCTGCGCCTTATGA
6_hypothetical	G13	Tb927.8.6520_for	GGGGACAAGTTTGTACAAAAAAGCAGGCTATGGAAGAAAAGATGACGAAGATTGACA	Tb927.8.6520_rev	GGGGACCACTTTGTACAAGAAAGCTGGGTTTACCACCACACAAGCCTCTGA
6_hypothetical	G14	Tb927.11.12380_for	GGGGACAAGTTTGTACAAAAAAGCAGGCTATGTCCACCAACTACGCACTGG	Tb927.11.12380_rev	GGGGACCACTTTGTACAAGAAAGCTGGGTTTAATTCTTATTCTCTGCCATTTTTTCCTG
6_hypothetical	G15	Tb927.8.5420_for	GGGGACAAGTTTGTACAAAAAAGCAGGCTATGGCAATACTTAGCCGTGTCATTG	Tb927.8.5420_rev	GGGGACCACTTTGTACAAGAAAGCTGGGTTTACAACTCCGAGAGTGATGATCGA
6_hypothetical	G16	Tb927.11.7920_for	GGGGACAAGTTTGTACAAAAAAGCAGGCTATGATACGGCAATGTGTTAAAACCATCC	Tb927.11.7920_rev	GGGGACCACTTTGTACAAGAAAGCTGGGTTCACGGGATAGTATCAGGATGTTCG
6_hypothetical	G17	Tb927.9.2100_for	GGGGACAAGTTTGTACAAAAAAGCAGGCTATGGAAACCCCCGATAACGTTG	Tb927.9.2100_rev	GGGGACCACTTTGTACAAGAAAGCTGGGTTTACCGGGCATCCTCTTCCC
6_hypothetical	G18	Tb927.11.15580_for	GGGGACAAGTTTGTACAAAAAAGCAGGCTATGATACGGAAGACCTTGGATGTGG	Tb927.11.15580_rev	GGGGACCACTTTGTACAAGAAAGCTGGGTTTAAGCATTAGACCTCCGTGATAGGT
6_hypothetical	G19	Tb927.9.4420_for	GGGGACAAGTTTGTACAAAAAAGCAGGCTATGTCCAAGTCTAGTCGTAAAATCACTTG	Tb927.9.4420_rev	GGGGACCACTTTGTACAAGAAAGCTGGGTTTAAATGCGGGCTCCGTCATC
6_hypothetical	G20	Tb927.11.1240_for	GGGGACAAGTTTGTACAAAAAAGCAGGCTATGAGTCACACAATGACAAATGCTTCT	Tb927.11.1240_rev	GGGGACCACTTTGTACAAGAAAGCTGGGTCTAAAACAAAAGTTGGCTGACAAGGC
6_hypothetical	G21	Tb927.9.13040_for	GGGGACAAGTTTGTACAAAAAAGCAGGCTATGAACTTCGTTCCTCTTTATAGATCAATT	Tb927.9.13040_rev	GGGGACCACTTTGTACAAGAAAGCTGGGTCTATTCGTCGTTAAACAAAAGAATGTGCC
6_hypothetical	G22	Tb927.7.2120_for	GGGGACAAGTTTGTACAAAAAAGCAGGCTATGCATCAAACGTCTGCAACTGT	Tb927.7.2120_rev	GGGGACCACTTTGTACAAGAAAGCTGGGTTCACACCACGCCATCAACCT
6_hypothetical	G23	Tb927.11.6530_for	GGGGACAAGTTTGTACAAAAAAGCAGGCTATGTTCGGTGGGTCTAAATCGGA	Tb927.11.6530_rev	GGGGACCACTTTGTACAAGAAAGCTGGGTTCATTGATGTGCGCGGTGATT
6_hypothetical	G24	Tb927.11.10090_for	GGGGACAAGTTTGTACAAAAAAGCAGGCTATGTCATCACCCATGTGTTCAGTC	Tb927.11.10090_rev	GGGGACCACTTTGTACAAGAAAGCTGGGTCTAAGATAGGTACCCCATTTCTACGGC
6_hypothetical	H1	Tb927.11.5470_for	GGGGACAAGTTTGTACAAAAAAGCAGGCTATGTCTTTTTTTGAAGATAGCGCAGAC	Tb927.11.5470_rev	GGGGACCACTTTGTACAAGAAAGCTGGGTCTACCGCTGAGTAACCATCATGC
6_hypothetical	H2	Tb927.10.15890_for	GGGGACAAGTTTGTACAAAAAAGCAGGCTATGCGGTCATCTTCCGCCG	Tb927.10.15890_rev	GGGGACCACTTTGTACAAGAAAGCTGGGTCTATGACTTCGCACTTCTGGCAA
6_hypothetical	H3	Tb927.8.6320_for	GGGGACAAGTTTGTACAAAAAAGCAGGCTATGATGTCATCCTCAGCTTTTACGG	Tb927.8.6320_rev	GGGGACCACTTTGTACAAGAAAGCTGGGTTTATTTGTGGCCATCAGAGTCGATAAC
6_hypothetical	H4	Tb927.4.3350_for	GGGGACAAGTTTGTACAAAAAAGCAGGCTATGCTTGGTGACGACCGC	Tb927.4.3350_rev	GGGGACCACTTTGTACAAGAAAGCTGGGTCTACGCAGCTTCCCTCGTATCG
6_hypothetical	H5	Tb927.11.4890_for	GGGGACAAGTTTGTACAAAAAAGCAGGCTATGAACTTTCCGGATAGTAGGGTACTT	Tb927.11.4890_rev	GGGGACCACTTTGTACAAGAAAGCTGGGTTCACGGTGCACGATCAGAGG
6_hypothetical	H6	Tb927.11.8030_for	GGGGACAAGTTTGTACAAAAAAGCAGGCTATGGAAGAAGGAACGTATGCGGG	Tb927.11.8030_rev	GGGGACCACTTTGTACAAGAAAGCTGGGTTTATTTGATAGCATTACGTGCGTGAAGT
6_hypothetical	H7	Tb927.9.12530_for	GGGGACAAGTTTGTACAAAAAAGCAGGCTATGTTTAAGTGCCGCGTGGC	Tb927.9.12530_rev	GGGGACCACTTTGTACAAGAAAGCTGGGTTCATAACTTCATGTGTTCAAGCGCG
6_hypothetical	H8	Tb927.5.3780_for	GGGGACAAGTTTGTACAAAAAAGCAGGCTATGAGTGTATCTCGGGCACAAGT	Tb927.5.3780_rev	GGGGACCACTTTGTACAAGAAAGCTGGGTTTAATTCCACCCCAAAAGTAATCTTACCTC
6_hypothetical	H9	Tb927.9.15230_for	GGGGACAAGTTTGTACAAAAAAGCAGGCTATGGAGTGCTCTGTGCACTCG	Tb927.9.15230_rev	GGGGACCACTTTGTACAAGAAAGCTGGGTTCAGAAGAACACCGCACGGG
6_hypothetical	H10	Tb927.10.9180_for	GGGGACAAGTTTGTACAAAAAAGCAGGCTATGTCAGCGTCAAGTGATTTGGAT	Tb927.10.9180_rev	GGGGACCACTTTGTACAAGAAAGCTGGGTTTACATCAGGCACGGTGCAGC
6_hypothetical	H11	Tb927.4.320_for	GGGGACAAGTTTGTACAAAAAAGCAGGCTATGAGGAAGTCGGCATTGAAGTTG	Tb927.4.320_rev	GGGGACCACTTTGTACAAGAAAGCTGGGTTCAAGGAAGCCCGTTGCG
6_hypothetical	H12	Tb927.9.12100_for	GGGGACAAGTTTGTACAAAAAAGCAGGCTATGTCTGTTTCTAGTCAAATAGGCATAAGG	Tb927.9.12100_rev	GGGGACCACTTTGTACAAGAAAGCTGGGTTTAATCTCTTGTTGCGTCCGTACG
6_hypothetical	H13	Tb927.11.6150_for	GGGGACAAGTTTGTACAAAAAAGCAGGCTATGTCGGCCCACCGCTTT	Tb927.11.6150_rev	GGGGACCACTTTGTACAAGAAAGCTGGGTTTAGTCTTTCACCTTTCTCCCCCTT
6_hypothetical	H14	Tb927.10.8990_for	GGGGACAAGTTTGTACAAAAAAGCAGGCTATGCCAATAAAGGGTTCGAGGAAG	Tb927.10.8990_rev	GGGGACCACTTTGTACAAGAAAGCTGGGTTCACTGGGGTTGCTCCCG
6_hypothetical	H15	Tb927.6.4800_for	GGGGACAAGTTTGTACAAAAAAGCAGGCTATGACTAGTATCGAGATGCAGGTGG	Tb927.6.4800_rev	GGGGACCACTTTGTACAAGAAAGCTGGGTTTATATAAATAGCCTTGTCGAGACCTGTGG
6_hypothetical	H16	Tb927.2.3000_for	GGGGACAAGTTTGTACAAAAAAGCAGGCTATGAATTACACTGCGGCCCG	Tb927.2.3000_rev	GGGGACCACTTTGTACAAGAAAGCTGGGTCTATACATCCAACTGAGTGTCCTGAGA
6_hypothetical	H17	Tb927.8.6770_for	GGGGACAAGTTTGTACAAAAAAGCAGGCTATGCTTACGTCCCTTTCGTGGG	Tb927.8.6770_rev	GGGGACCACTTTGTACAAGAAAGCTGGGTTTAGCAAAACACTCGCTGCACA
6_hypothetical	H18	Tb927.11.7780_for	GGGGACAAGTTTGTACAAAAAAGCAGGCTATGGAGTCGCCGATCAACCC	Tb927.11.7780_rev	GGGGACCACTTTGTACAAGAAAGCTGGGTTTACATGCCGCCACCCATCA
6_hypothetical	H19	Tb927.11.10850_for	GGGGACAAGTTTGTACAAAAAAGCAGGCTATGCTAAACCTGGAGTCGCTTAAC	Tb927.11.10850_rev	GGGGACCACTTTGTACAAGAAAGCTGGGTTCATAGCCCACGTCGTTCCC
6_hypothetical	H20	Tb927.8.4210_for	GGGGACAAGTTTGTACAAAAAAGCAGGCTATGCGGCTTCGTTCCTTCTTG	Tb927.8.4210_rev	GGGGACCACTTTGTACAAGAAAGCTGGGTCAACTTTTCCATATACCACACGTATTATC
6_hypothetical	H21	Tb927.3.950_for	GGGGACAAGTTTGTACAAAAAAGCAGGCTATGCGTCGTACTTTTTCGCTTTTC	Tb927.3.950_rev	GGGGACCACTTTGTACAAGAAAGCTGGGTTTAAGGTGCTGCTGGCATTAGATC
6_hypothetical	H22	Tb927.5.3020_for	GGGGACAAGTTTGTACAAAAAAGCAGGCTATGCTGAACAACAAGAAAGAAAACACG	Tb927.5.3020_rev	GGGGACCACTTTGTACAAGAAAGCTGGGTCTACTCGAGTTCCTTTGTTGTCGAA
6_hypothetical	H23	Tb927.11.11580_for	GGGGACAAGTTTGTACAAAAAAGCAGGCTATGGACTTTGACACGTCAACTACG	Tb927.11.11580_rev	GGGGACCACTTTGTACAAGAAAGCTGGGTTCATGAACGAATAAGCTCCAGTAGGT
6_hypothetical	H24	Tb927.10.14490_for	GGGGACAAGTTTGTACAAAAAAGCAGGCTATGCTCCGTGAGCAGTGCA	Tb927.10.14490_rev	GGGGACCACTTTGTACAAGAAAGCTGGGTCTAATTAGCTGCTGCGGGAAGG
6_hypothetical	I1	Tb927.2.5970_for	GGGGACAAGTTTGTACAAAAAAGCAGGCTATGTCGCTTTCATGTGGACCAC	Tb927.2.5970_rev	GGGGACCACTTTGTACAAGAAAGCTGGGTTCAAACATGATCTGCACGGTCAC
6_hypothetical	I2	Tb927.8.4490_for	GGGGACAAGTTTGTACAAAAAAGCAGGCTATGCAAGATAATGTTGCGGGCT	Tb927.8.4490_rev	GGGGACCACTTTGTACAAGAAAGCTGGGTTTACTTTGGGGCGTCGCG
6_hypothetical	I3	Tb927.11.13350_for	GGGGACAAGTTTGTACAAAAAAGCAGGCTATGGCGGCACACGATGAGG	Tb927.11.13350_rev	GGGGACCACTTTGTACAAGAAAGCTGGGTCTAACGATTCTTAACTTTGGACTCCAGTAT
6_hypothetical	I4	Tb927.11.8390_for	GGGGACAAGTTTGTACAAAAAAGCAGGCTATGGTGTTCATCCTCTTGGCATG	Tb927.11.8390_rev	GGGGACCACTTTGTACAAGAAAGCTGGGTTCAGTCATGAATGTCGTACCCATATTTG
6_hypothetical	I5	Tb927.7.390_for	GGGGACAAGTTTGTACAAAAAAGCAGGCTATGCATGATTTGGTCGCTGTTGT	Tb927.7.390_rev	GGGGACCACTTTGTACAAGAAAGCTGGGTTTATTCTTTCGACTGTCCCTGTTCAG
6_hypothetical	I6	Tb927.8.2030_for	GGGGACAAGTTTGTACAAAAAAGCAGGCTATGGCCGATTCAAATGCTGTCG	Tb927.8.2030_rev	GGGGACCACTTTGTACAAGAAAGCTGGGTCTACTTACCCAACTTACTTACAAGTGCA
6_hypothetical	I7	Tb927.5.620_for	GGGGACAAGTTTGTACAAAAAAGCAGGCTATGTCATACAACGCCCTTTGCA	Tb927.5.620_rev	GGGGACCACTTTGTACAAGAAAGCTGGGTTCAGATTACCTTGTCTCCCGGTAG
6_hypothetical	I8	Tb927.7.3610_for	GGGGACAAGTTTGTACAAAAAAGCAGGCTATGTCCTCTAGGAGCGGCG	Tb927.7.3610_rev	GGGGACCACTTTGTACAAGAAAGCTGGGTTCACGGGACGTTGGTACTAATCA
6_hypothetical	I9	Tb927.8.6290_for	GGGGACAAGTTTGTACAAAAAAGCAGGCTATGGAAGACGCGGTAGAGGC	Tb927.8.6290_rev	GGGGACCACTTTGTACAAGAAAGCTGGGTTTAGCAATCTTTTGAAACAACACTTGACTC
6_hypothetical	I10	Tb927.4.1450_for	GGGGACAAGTTTGTACAAAAAAGCAGGCTATGATGATAGGTGTCCCGCCTG	Tb927.4.1450_rev	GGGGACCACTTTGTACAAGAAAGCTGGGTCACATAAGTTTATTAAAGCAAGTACTTTC
6_hypothetical	I11	Tb927.4.1740_for	GGGGACAAGTTTGTACAAAAAAGCAGGCTATGCCGTTGTTGTATGCCTCAA	Tb927.4.1740_rev	GGGGACCACTTTGTACAAGAAAGCTGGGTTTAGGAGAAGTCCCATCGTTTCTTTAAC
6_hypothetical	I12	Tb927.6.4380_for	GGGGACAAGTTTGTACAAAAAAGCAGGCTATGGAGTGTGAATTCATGTGTGACG	Tb927.6.4380_rev	GGGGACCACTTTGTACAAGAAAGCTGGGTTCAATCAACCTTCCCCTTTTCGG
6_hypothetical	I13	Tb927.9.12980_for	GGGGACAAGTTTGTACAAAAAAGCAGGCTATGTCCGTCGTTGAAATGGAGAAG	Tb927.9.12980_rev	GGGGACCACTTTGTACAAGAAAGCTGGGTTTATTCATAAAGTTGAATGATACGCCTTTG
6_hypothetical	I14	Tb927.9.13580_for	GGGGACAAGTTTGTACAAAAAAGCAGGCTATGACCAAACATGGTGGCAGC	Tb927.9.13580_rev	GGGGACCACTTTGTACAAGAAAGCTGGGTTTAATAGAAGGGTTTGAACCGCGC
6_hypothetical	I15	Tb927.9.2814_for	GGGGACAAGTTTGTACAAAAAAGCAGGCTATGGAAATCCGCATGGTGCC	Tb927.9.2814_rev	GGGGACCACTTTGTACAAGAAAGCTGGGTTCACTCATCAGCTGCTGGAGT
6_hypothetical	I17	Tb927.5.3470_for	GGGGACAAGTTTGTACAAAAAAGCAGGCTATGCAAATGAGAGTAGAGTCTCCACA	Tb927.5.3470_rev	GGGGACCACTTTGTACAAGAAAGCTGGGTTTACTGGTCTTCCGAAAGGGACC
6_hypothetical	I18	Tb927.8.2890_for	GGGGACAAGTTTGTACAAAAAAGCAGGCTATGATGTCGAGTTTATTAGCGGACAC	Tb927.8.2890_rev	GGGGACCACTTTGTACAAGAAAGCTGGGTCTACTGCAAGGTTAGTGCTGACAA
6_hypothetical	I19	Tb927.10.1300_for	GGGGACAAGTTTGTACAAAAAAGCAGGCTATGCCAGCTGTCTGTCAAAGTC	Tb927.10.1300_rev	GGGGACCACTTTGTACAAGAAAGCTGGGTTCAAGAGAAGTCGACGGGAGG
6_hypothetical	I20	Tb927.10.15000_for	GGGGACAAGTTTGTACAAAAAAGCAGGCTATGATAGGCGGTCAGCACAAGA	Tb927.10.15000_rev	GGGGACCACTTTGTACAAGAAAGCTGGGTTCATCGGCGTTTCGGGATGA
6_hypothetical	I21	Tb927.11.2170_for	GGGGACAAGTTTGTACAAAAAAGCAGGCTATGATGGAATTAATCGGCTGCCC	Tb927.11.2170_rev	GGGGACCACTTTGTACAAGAAAGCTGGGTTTACCACACGCCATAGAACAAATCC
6_hypothetical	I22	Tb927.4.4750_for	GGGGACAAGTTTGTACAAAAAAGCAGGCTATGCAAGCATCGAGGTCAAAGG	Tb927.4.4750_rev	GGGGACCACTTTGTACAAGAAAGCTGGGTTTACGATCTTGCTGCTGTTTTCCT

6_hypothetical	I23	Tb927.11.7030_for	GGGGACAAGTTTGTACAAAAAAGCAGGCTATGGATGCACTCTGGCCAGT	Tb927.11.7030_rev	GGGGACCACTTTGTACAAGAAAGCTGGGTCTAGCAGTGAACAAATGTTGGCAC
6_hypothetical	I24	Tb927.9.10050_for	GGGGACAAGTTTGTACAAAAAAGCAGGCTATGCGGAAATTGGTTCGAAGCT	Tb927.9.10050_rev	GGGGACCACTTTGTACAAGAAAGCTGGGTCACGAGCGGACCCTTGT
6_hypothetical	J1	Tb927.3.4780_for	GGGGACAAGTTTGTACAAAAAAGCAGGCTATGCGCACCCGACTACTTGT	Tb927.3.4780_rev	GGGGACCACTTTGTACAAGAAAGCTGGGTCAGAGTGGCGGCATGGT
6_hypothetical	J2	Tb927.5.3550_for	GGGGACAAGTTTGTACAAAAAAGCAGGCTATGTCCAGGAGGTGGTCTTGC	Tb927.5.3550_rev	GGGGACCACTTTGTACAAGAAAGCTGGGTCTAAAGGTATGCTTGCAGCAACTCG
6_hypothetical	J4	Tb927.11.9090_for	GGGGACAAGTTTGTACAAAAAAGCAGGCTATGTTGCTACCTTGCACACCG	Tb927.11.9090_rev	GGGGACCACTTTGTACAAGAAAGCTGGGTCTAACAAATCGACCTCAGTCGAATCA
6_hypothetical	J5	Tb927.7.4410_for	GGGGACAAGTTTGTACAAAAAAGCAGGCTATGATAGAGTTCGCCAGCCCG	Tb927.7.4410_rev	GGGGACCACTTTGTACAAGAAAGCTGGGTCTACATGTTCTCTCTTAAAAAATTCCCTGA
6_hypothetical	J6	Tb927.6.3930_for	GGGGACAAGTTTGTACAAAAAAGCAGGCTATGTCCAGAACGCTTTTTCGTCT	Tb927.6.3930_rev	GGGGACCACTTTGTACAAGAAAGCTGGGTCTATTCATTGTACTCTTTCTGCCTGACG
6_hypothetical	J7	Tb927.4.3820_for	GGGGACAAGTTTGTACAAAAAAGCAGGCTATGAATGATTTACGTCAACGGGCC	Tb927.4.3820_rev	GGGGACCACTTTGTACAAGAAAGCTGGGTCAATACCCTTCATCATCGGGCG
6_hypothetical	J8	Tb927.1.4230_for	GGGGACAAGTTTGTACAAAAAAGCAGGCTATGCTTAAGGCCTGTTTGCCG	Tb927.1.4230_rev	GGGGACCACTTTGTACAAGAAAGCTGGGTTTATTTGGGAGGTTCCTTGGCAC
6_hypothetical	J9	Tb927.5.2750_for	GGGGACAAGTTTGTACAAAAAAGCAGGCTATGACTTCCTTAAAGGAGAAGGTACTGT	Tb927.5.2750_rev	GGGGACCACTTTGTACAAGAAAGCTGGGTCAATACAAACAAATACCTTCAGTTGGTTC
6_hypothetical	J10	Tb927.11.14500_for	GGGGACAAGTTTGTACAAAAAAGCAGGCTATGTCAAGCGGTTGCGCA	Tb927.11.14500_rev	GGGGACCACTTTGTACAAGAAAGCTGGGTTTAACCAATCTGCTTTATCCACGAGATC
6_hypothetical	J11	Tb927.7.6250_for	GGGGACAAGTTTGTACAAAAAAGCAGGCTATGAGTGGGACAAGTTCTTCACTTG	Tb927.7.6250_rev	GGGGACCACTTTGTACAAGAAAGCTGGGTCATACAGCGATCGAACACGAAAC
6_hypothetical	J12	Tb927.10.11510_for	GGGGACAAGTTTGTACAAAAAAGCAGGCTATGTCTGTTGCGTCTTCGACC	Tb927.10.11510_rev	GGGGACCACTTTGTACAAGAAAGCTGGGTCTAAAGGAGAAATGCCATGCCATTATCG
6_hypothetical	J13	Tb927.4.580_for	GGGGACAAGTTTGTACAAAAAAGCAGGCTATGGTAAATGGTGTGGTGCCCT	Tb927.4.580_rev	GGGGACCACTTTGTACAAGAAAGCTGGGTCTAATGCGTCATGCGACGTGA
6_hypothetical	J14	Tb927.3.3520_for	GGGGACAAGTTTGTACAAAAAAGCAGGCTATGGTCTACCCCTTTTCCAAGCA	Tb927.3.3520_rev	GGGGACCACTTTGTACAAGAAAGCTGGGTTTAACCCCCGGAACTTTCAACCT
6_hypothetical	J15	Tb927.3.1530_for	GGGGACAAGTTTGTACAAAAAAGCAGGCTATGCCGTGTGCCACTAAAGG	Tb927.3.1530_rev	GGGGACCACTTTGTACAAGAAAGCTGGGTCACTCATATGGAGCAATCGGATCA
6_hypothetical	J16	Tb927.11.4390_for	GGGGACAAGTTTGTACAAAAAAGCAGGCTATGACGTCCGTGCAGTATGC	Tb927.11.4390_rev	GGGGACCACTTTGTACAAGAAAGCTGGGTCTAGGTGTATTGTAACACGGTAGCAG
6_hypothetical	J17	Tb927.9.2812_for	GGGGACAAGTTTGTACAAAAAAGCAGGCTATGGAAATCCGCATGGTGCC	Tb927.9.2812_rev	GGGGACCACTTTGTACAAGAAAGCTGGGTCACTCATCAGCTGCTGGAGT
6_hypothetical	J18	Tb927.11.2720_for	GGGGACAAGTTTGTACAAAAAAGCAGGCTATGAGGGGAATGTGGGCGA	Tb927.11.2720_rev	GGGGACCACTTTGTACAAGAAAGCTGGGTCAACGGCAGTAGAGGTACCC
6_hypothetical	J19	Tb927.10.11240_for	GGGGACAAGTTTGTACAAAAAAGCAGGCTATGAGTGCTGAGGATGCACTTG	Tb927.10.11240_rev	GGGGACCACTTTGTACAAGAAAGCTGGGTTTACTTATCCTCCCTTTTGGCCAGT
6_hypothetical	J20	Tb927.9.3080_for	GGGGACAAGTTTGTACAAAAAAGCAGGCTATGCCACAGCCGTCAGCG	Tb927.9.3080_rev	GGGGACCACTTTGTACAAGAAAGCTGGGTCAAACGATTTTGTCAATCGGCG
6_hypothetical	J21	Tb927.8.2670_for	GGGGACAAGTTTGTACAAAAAAGCAGGCTATGCTCAGAAGAGCGGTACCC	Tb927.8.2670_rev	GGGGACCACTTTGTACAAGAAAGCTGGGTCACAACGAAGAGGGAAGCTCG
6_hypothetical	J22	Tb927.9.2860_for	GGGGACAAGTTTGTACAAAAAAGCAGGCTATGGAAATCCGCATGGTGCC	Tb927.9.2860_rev	GGGGACCACTTTGTACAAGAAAGCTGGGTCACTCATCAGCTGCTGGAGT
6_hypothetical	J23	Tb927.6.1170_for	GGGGACAAGTTTGTACAAAAAAGCAGGCTATGCACTTTATTGGCATTGTTGGC	Tb927.6.1170_rev	GGGGACCACTTTGTACAAGAAAGCTGGGTTCAATTCTCCAATCCCGCTGC
6_hypothetical	J24	Tb927.2.3160_for	GGGGACAAGTTTGTACAAAAAAGCAGGCTATGTCCAGTCGGTGCTCCTC	Tb927.2.3160_rev	GGGGACCACTTTGTACAAGAAAGCTGGGTCACCTCGCAATCCAAGAAGGA
6_hypothetical	K1	Tb927.10.14020_for	GGGGACAAGTTTGTACAAAAAAGCAGGCTATGGCAGAAGCGGACTGTTCC	Tb927.10.14020_rev	GGGGACCACTTTGTACAAGAAAGCTGGGTCATTCATACTGTTCAGTGGGTGTTAAG
6_hypothetical	K2	Tb927.7.5620_for	GGGGACAAGTTTGTACAAAAAAGCAGGCTATGGTGAGGCTTCGCTCGG	Tb927.7.5620_rev	GGGGACCACTTTGTACAAGAAAGCTGGGTTTACTCCCTTCGAATACATTTGGGC
6_hypothetical	K3	Tb927.7.3080_for	GGGGACAAGTTTGTACAAAAAAGCAGGCTATGGCAGAGGGAGTACCGGC	Tb927.7.3080_rev	GGGGACCACTTTGTACAAGAAAGCTGGGTCTACCGGCCTTCACCCTCG
6_hypothetical	K4	Tb927.4.500_for	GGGGACAAGTTTGTACAAAAAAGCAGGCTATGAAGAGGTATTTTCTCATTGATGAATCT	Tb927.4.500_rev	GGGGACCACTTTGTACAAGAAAGCTGGGTTTAAGCTGGAACTTTACCGCGC
6_hypothetical	K5	Tb927.11.9760_for	GGGGACAAGTTTGTACAAAAAAGCAGGCTATGAGCTCACCGTACCAACGA	Tb927.11.9760_rev	GGGGACCACTTTGTACAAGAAAGCTGGGTTTATATCGCCACCGCTTCCTCT
6_hypothetical	K6	Tb927.11.12920_for	GGGGACAAGTTTGTACAAAAAAGCAGGCTATGCCCCATTCGGGATACCC	Tb927.11.12920_rev	GGGGACCACTTTGTACAAGAAAGCTGGGTCTACAAGTCGTTCATGCTATGCGG
6_hypothetical	K7	Tb927.4.1590.1_for	GGGGACAAGTTTGTACAAAAAAGCAGGCTATGCTGTCACCCTTGTTACTATGGA	Tb927.4.1590.1_rev	GGGGACCACTTTGTACAAGAAAGCTGGGTCTACTTGCCGTACTCTGTGGAGA
6_hypothetical	K8	Tb927.7.2800_for	GGGGACAAGTTTGTACAAAAAAGCAGGCTATGAGTTTTGATGGCCTTCGGG	Tb927.7.2800_rev	GGGGACCACTTTGTACAAGAAAGCTGGGTTTATCCCGCTGTTTCCTTGTTCG
6_hypothetical	K9	Tb927.2.4340_for	GGGGACAAGTTTGTACAAAAAAGCAGGCTATGCAAAATCCTACGATTTATCGAAATTCC	Tb927.2.4340_rev	GGGGACCACTTTGTACAAGAAAGCTGGGTCATCTTGAAAGCAGGGATCGC
6_hypothetical	K10	Tb927.8.6820_for	GGGGACAAGTTTGTACAAAAAAGCAGGCTATGTGGAAATGTTACCCTCTTCGC	Tb927.8.6820_rev	GGGGACCACTTTGTACAAGAAAGCTGGGTCTAAACGACCGATCCTTGTGGC
6_hypothetical	K11	Tb927.11.5870_for	GGGGACAAGTTTGTACAAAAAAGCAGGCTATGAGCACATCCCTCACGTCG	Tb927.11.5870_rev	GGGGACCACTTTGTACAAGAAAGCTGGGTTTACGATTTCTTCCCAAACAGAGAAAGA
6_hypothetical	K12	Tb927.11.10890_for	GGGGACAAGTTTGTACAAAAAAGCAGGCTATGGATACGAGCTCGCCGA	Tb927.11.10890_rev	GGGGACCACTTTGTACAAGAAAGCTGGGTCATGGGGGCGCTATGATAGC
6_hypothetical	K13	Tb927.9.4440_for	GGGGACAAGTTTGTACAAAAAAGCAGGCTATGTCACTCAGGTCCACAATTCAGA	Tb927.9.4440_rev	GGGGACCACTTTGTACAAGAAAGCTGGGTCAGCAAAAGGTGCTTTCCTCAA
6_hypothetical	K14	Tb927.10.13800_for	GGGGACAAGTTTGTACAAAAAAGCAGGCTATGTCTGAGGCTAGCGACCG	Tb927.10.13800_rev	GGGGACCACTTTGTACAAGAAAGCTGGGTCTACTGAGAACAATCGGCGTTGG
6_hypothetical	K15	Tb927.11.10880_for	GGGGACAAGTTTGTACAAAAAAGCAGGCTATGCTTCGCCGTACACTTTCG	Tb927.11.10880_rev	GGGGACCACTTTGTACAAGAAAGCTGGGTCTACCAGCGGAAGTCGTTAAATATATTTGT
6_hypothetical	K16	Tb927.4.740_for	GGGGACAAGTTTGTACAAAAAAGCAGGCTATGTTGTTGCTCAGGGCAACG	Tb927.4.740_rev	GGGGACCACTTTGTACAAGAAAGCTGGGTCAAAAAATAACTTCGAGCGTTATGCC
6_hypothetical	K17	Tb927.10.5200_for	GGGGACAAGTTTGTACAAAAAAGCAGGCTATGCAACACATTAACCATAGATTCAGTTAC	Tb927.10.5200_rev	GGGGACCACTTTGTACAAGAAAGCTGGGTCTACGTCACGCATTCTACATCGG
6_hypothetical	K18	Tb927.7.2460_for	GGGGACAAGTTTGTACAAAAAAGCAGGCTATGTCCACACACCGTGAGAGT	Tb927.7.2460_rev	GGGGACCACTTTGTACAAGAAAGCTGGGTCATAGCTGAACACCACAAAGCAC
6_hypothetical	K19	Tb927.5.2860_for	GGGGACAAGTTTGTACAAAAAAGCAGGCTATGAAGGAGTTTCGACGTCGAATG	Tb927.5.2860_rev	GGGGACCACTTTGTACAAGAAAGCTGGGTTTACTTGCGTATCTGGCACTCAGC
6_hypothetical	K20	Tb927.11.10860_for	GGGGACAAGTTTGTACAAAAAAGCAGGCTATGTCGTGGGCTGCACTTG	Tb927.11.10860_rev	GGGGACCACTTTGTACAAGAAAGCTGGGTCACTTCCGTCGCGCCGC
6_hypothetical	K21	Tb927.3.870_for	GGGGACAAGTTTGTACAAAAAAGCAGGCTATGCAAACTCCGCTACGTCC	Tb927.3.870_rev	GGGGACCACTTTGTACAAGAAAGCTGGGTTTACTCTGTGAATAATATCTGTTGGGCAC
6_hypothetical	K22	Tb927.5.4400_for	GGGGACAAGTTTGTACAAAAAAGCAGGCTATGGGACGGAGGAAGGAGAAGA	Tb927.5.4400_rev	GGGGACCACTTTGTACAAGAAAGCTGGGTCTAAGTGCTTGCAGCCTTCTTTCC
6_hypothetical	K23	Tb927.10.5810_for	GGGGACAAGTTTGTACAAAAAAGCAGGCTATGGACGAGAGAGGCATCTTAGAAT	Tb927.10.5810_rev	GGGGACCACTTTGTACAAGAAAGCTGGGTTTATTTCTTTGTTTCATCAAGAATGTCGTT
6_hypothetical	K24	Tb927.3.3120_for	GGGGACAAGTTTGTACAAAAAAGCAGGCTATGGCGGAGGCCGCCCGC	Tb927.3.3120_rev	GGGGACCACTTTGTACAAGAAAGCTGGGTTTACACTGCAGCGTCAAACTCC
6_hypothetical	L1	Tb927.10.9590_for	GGGGACAAGTTTGTACAAAAAAGCAGGCTATGTCGTCGTCGCGGGCC	Tb927.10.9590_rev	GGGGACCACTTTGTACAAGAAAGCTGGGTTTATAGCGTGTCACGCGAAAGAG
6_hypothetical	L2	Tb927.11.14060_for	GGGGACAAGTTTGTACAAAAAAGCAGGCTATGGAACTTGATGAAGAGACGAATGC	Tb927.11.14060_rev	GGGGACCACTTTGTACAAGAAAGCTGGGTCTACCGCAAAAGAAGTTCCCACG
6_hypothetical	L3	Tb927.7.6370_for	GGGGACAAGTTTGTACAAAAAAGCAGGCTATGGCTGCCCGGGCTTTA	Tb927.7.6370_rev	GGGGACCACTTTGTACAAGAAAGCTGGGTTTAGTGTTGCCCCTGACACTCC
6_hypothetical	L4	Tb927.10.480_for	GGGGACAAGTTTGTACAAAAAAGCAGGCTATGGTTGATGTTAATGGAACGACAATTTT	Tb927.10.480_rev	GGGGACCACTTTGTACAAGAAAGCTGGGTTTACTCTTCCATTTCAGCCACCATTC
6_hypothetical	L5	Tb927.10.1850_for	GGGGACAAGTTTGTACAAAAAAGCAGGCTATGGGTCAGTGGCTTTGCTTTC	Tb927.10.1850_rev	GGGGACCACTTTGTACAAGAAAGCTGGGTTTAGCCTGGGCTGGCCCC
6_hypothetical	L6	Tb927.10.11290_for	GGGGACAAGTTTGTACAAAAAAGCAGGCTATGCAACGGTATGGTTCCCTGA	Tb927.10.11290_rev	GGGGACCACTTTGTACAAGAAAGCTGGGTCTAGCTGACAAATAACAGTGCAGTCT
6_hypothetical	L7	Tb927.10.12230_for	GGGGACAAGTTTGTACAAAAAAGCAGGCTATGGACTCGGTTTGCCCGG	Tb927.10.12230_rev	GGGGACCACTTTGTACAAGAAAGCTGGGTCTACATTTCACTTTTTGGAAAGTAGAAGCT
6_hypothetical	L8	Tb927.8.2570_for	GGGGACAAGTTTGTACAAAAAAGCAGGCTATGGCAAGGTTTCAATCTTCTGTGG	Tb927.8.2570_rev	GGGGACCACTTTGTACAAGAAAGCTGGGTCTACGATTTGCACTTTTCGGTGTC
6_hypothetical	L9	Tb927.2.2880_for	GGGGACAAGTTTGTACAAAAAAGCAGGCTATGGCGGCGATGAGCGAG	Tb927.2.2880_rev	GGGGACCACTTTGTACAAGAAAGCTGGGTCTACCGCGCCTTTCGCCG
6_hypothetical	L10	Tb927.4.3000_for	GGGGACAAGTTTGTACAAAAAAGCAGGCTATGGACAGCCAAGCACGGG	Tb927.4.3000_rev	GGGGACCACTTTGTACAAGAAAGCTGGGTCTACGAAGAGCCGCTTTGCA
6_hypothetical	L11	Tb927.8.1960_for	GGGGACAAGTTTGTACAAAAAAGCAGGCTATGGTGGTGGAGTACCTTGGTC	Tb927.8.1960_rev	GGGGACCACTTTGTACAAGAAAGCTGGGTTTACTTTAGTTCATCCCATCTCGTAACCA
6_hypothetical	L13	Tb927.3.2720_for	GGGGACAAGTTTGTACAAAAAAGCAGGCTATGCAAAAGTCAGTTCCTTCATATGTTG	Tb927.3.2720_rev	GGGGACCACTTTGTACAAGAAAGCTGGGTTCATGAAAGGAAATAAACCTTCATTTCCTT
6_hypothetical	L14	Tb927.11.8330_for	GGGGACAAGTTTGTACAAAAAAGCAGGCTATGCGTGTAGGAGGATATATGGGTAC	Tb927.11.8330_rev	GGGGACCACTTTGTACAAGAAAGCTGGGTTTACAGAGTGGATTCCAGGATTCGG
6_hypothetical	L17	Tb927.11.8320_for	GGGGACAAGTTTGTACAAAAAAGCAGGCTATGATGATGCGAGTGGTAGGTAAGG	Tb927.11.8320_rev	GGGGACCACTTTGTACAAGAAAGCTGGGTTCAAAGGGTTCGAATCACTATAGGAGA
6_hypothetical	L18	Tb927.11.3580_for	GGGGACAAGTTTGTACAAAAAAGCAGGCTATGACACACAACAGGAGCGGG	Tb927.11.3580_rev	GGGGACCACTTTGTACAAGAAAGCTGGGTCTAGCCCCCCTCCTTTTTTTGC
6_hypothetical	L19	Tb927.10.6300_for	GGGGACAAGTTTGTACAAAAAAGCAGGCTATGTTTCGGTTGACTGCTGCTT	Tb927.10.6300_rev	GGGGACCACTTTGTACAAGAAAGCTGGGTTCAGTGTAACTGGTAGTTACGTTTTATGTG
6_hypothetical	L20	Tb927.10.330_for	GGGGACAAGTTTGTACAAAAAAGCAGGCTATGGAAAAGGCCCGTCAATGG	Tb927.10.330_rev	GGGGACCACTTTGTACAAGAAAGCTGGGTTTAATGTGACATTGTTCTACACCCACT
6_hypothetical	L21	Tb927.6.3100_for	GGGGACAAGTTTGTACAAAAAAGCAGGCTATGCCCCGAGGATCAAGCA	Tb927.6.3100_rev	GGGGACCACTTTGTACAAGAAAGCTGGGTCTATTCGTGTTGAAAGTGTTGATTCGC
6_hypothetical	L22	Tb927.10.6100_for	GGGGACAAGTTTGTACAAAAAAGCAGGCTATGCAGCCGGTAAGGACGT	Tb927.10.6100_rev	GGGGACCACTTTGTACAAGAAAGCTGGGTCTAGTACATAATGGTGCGGCATAAGT
6_hypothetical	L23	Tb927.11.2010_for	GGGGACAAGTTTGTACAAAAAAGCAGGCTATGGGTCTTTTAACGAGGTATTATGAGAAA	Tb927.11.2010_rev	GGGGACCACTTTGTACAAGAAAGCTGGGTTCAGTTTCCGGCCAGCCC
6_hypothetical	L24	Tb927.4.3340_for	GGGGACAAGTTTGTACAAAAAAGCAGGCTATGAACTTGCCTTTCCCTACTCACA	Tb927.4.3340_rev	GGGGACCACTTTGTACAAGAAAGCTGGGTTTAGCGTGCACATCCCTCTTTG
6_hypothetical	M1	Tb927.11.920_for	GGGGACAAGTTTGTACAAAAAAGCAGGCTATGCGTACAGGGCGCCTG	Tb927.11.920_rev	GGGGACCACTTTGTACAAGAAAGCTGGGTCTACCATACGCTGGGGGCG
6_hypothetical	M2	Tb927.8.8320_for	GGGGACAAGTTTGTACAAAAAAGCAGGCTATGAATGTACTTTCTGACATTGCTCACT	Tb927.8.8320_rev	GGGGACCACTTTGTACAAGAAAGCTGGGTCACTTGAGGGATATCATGCACGC
6_hypothetical	M3	Tb927.6.4520_for	GGGGACAAGTTTGTACAAAAAAGCAGGCTATGGACGCTGGAGATGCCC	Tb927.6.4520_rev	GGGGACCACTTTGTACAAGAAAGCTGGGTCTATTTCGTAATCCCAAATTTCTTCTTTTC
6_hypothetical	M4	Tb927.7.3400_for	GGGGACAAGTTTGTACAAAAAAGCAGGCTATGGCTTTGGGATCAGGAATCGG	Tb927.7.3400_rev	GGGGACCACTTTGTACAAGAAAGCTGGGTCATGTTGGTGCATCCGCC
6_hypothetical	M5	Tb927.7.180_for	GGGGACAAGTTTGTACAAAAAAGCAGGCTATGTATGCTATTAAAATTTTTGTTATTTCG	Tb927.7.180_rev	GGGGACCACTTTGTACAAGAAAGCTGGGTCATTCCACTGGAAATAGTTGCTTCAG
6_hypothetical	M6	Tb927.11.5580_for	GGGGACAAGTTTGTACAAAAAAGCAGGCTATGGTAAGAAAGGAGGAAGAAAAAGTTTCA	Tb927.11.5580_rev	GGGGACCACTTTGTACAAGAAAGCTGGGTCTATTTTCTCCAGCGAGTCCATATACC
6_hypothetical	M7	Tb927.1.3410_for	GGGGACAAGTTTGTACAAAAAAGCAGGCTATGAAGCGCAGGGTATTGCG	Tb927.1.3410_rev	GGGGACCACTTTGTACAAGAAAGCTGGGTCTCACTCTTTTCGCTCTTTCTTCCTC
6_hypothetical	M8	Tb927.11.4750_for	GGGGACAAGTTTGTACAAAAAAGCAGGCTATGAATCGCATACTGTTGCACTTG	Tb927.11.4750_rev	GGGGACCACTTTGTACAAGAAAGCTGGGTTTAGACGACAAAGGTAAGAAACACAAACA
6_hypothetical	M9	Tb927.10.13100_for	GGGGACAAGTTTGTACAAAAAAGCAGGCTATGAAAGACGATGAGTTGGAAAGAAAAATT	Tb927.10.13100_rev	GGGGACCACTTTGTACAAGAAAGCTGGGTCTAGTGAGCCAATAGTGACATCTGTTT
6_hypothetical	M10	Tb927.3.1940_for	GGGGACAAGTTTGTACAAAAAAGCAGGCTATGGAGCGGACACGGATTAACT	Tb927.3.1940_rev	GGGGACCACTTTGTACAAGAAAGCTGGGTCTACAGCTTTATTCCGGTCTTCTTGT
6_hypothetical	M11	Tb927.9.11540_for	GGGGACAAGTTTGTACAAAAAAGCAGGCTATGGCTGACGTTAGCGCCG	Tb927.9.11540_rev	GGGGACCACTTTGTACAAGAAAGCTGGGTTTATCGCATTGGGCGGGTTC
6_hypothetical	M12	Tb927.9.13110_for	GGGGACAAGTTTGTACAAAAAAGCAGGCTATGGAGAATTCGTGTCCAGGGG	Tb927.9.13110_rev	GGGGACCACTTTGTACAAGAAAGCTGGGTTTATTTCCCTTCTTTGCTTCGAAAACC
6_hypothetical	M13	Tb927.4.3690_for	GGGGACAAGTTTGTACAAAAAAGCAGGCTATGCTGCGGAGGACTGTAACA	Tb927.4.3690_rev	GGGGACCACTTTGTACAAGAAAGCTGGGTTTACCGCCTAATCATCTCCTCCTTC
6_hypothetical	M14	Tb927.6.3600_for	GGGGACAAGTTTGTACAAAAAAGCAGGCTATGTGGTCCAAGGTGTTTACACC	Tb927.6.3600_rev	GGGGACCACTTTGTACAAGAAAGCTGGGTCTACGATGCTAGTTCTAGCAAGAAAGC
6_hypothetical	M15	Tb927.6.2630_for	GGGGACAAGTTTGTACAAAAAAGCAGGCTATGTGGTTCAGCCGCTTTTCG	Tb927.6.2630_rev	GGGGACCACTTTGTACAAGAAAGCTGGGTTTATTTTTTAAAGGAAGTACGGTCGCGC
6_hypothetical	M16	Tb927.11.13980_for	GGGGACAAGTTTGTACAAAAAAGCAGGCTATGACGGAAATGACGAAGGATGTTT	Tb927.11.13980_rev	GGGGACCACTTTGTACAAGAAAGCTGGGTTTACTCTATACGTTTCTCCCACGAATAAAA
6_hypothetical	M17	Tb927.10.12880_for	GGGGACAAGTTTGTACAAAAAAGCAGGCTATGCCGACTCAGAGCCCC	Tb927.10.12880_rev	GGGGACCACTTTGTACAAGAAAGCTGGGTCTAGGGCGGAAAAAGTTTCTGGC
6_hypothetical	M18	Tb927.9.2811_for	GGGGACAAGTTTGTACAAAAAAGCAGGCTATGGAAATCCGCATGGTGCC	Tb927.9.2811_rev	GGGGACCACTTTGTACAAGAAAGCTGGGTCACTCATCAGCTGCTGGAGT
6_hypothetical	M19	Tb927.9.11880_for	GGGGACAAGTTTGTACAAAAAAGCAGGCTATGATGCGCAGTTCTTCTTTTTGC	Tb927.9.11880_rev	GGGGACCACTTTGTACAAGAAAGCTGGGTTTAAAGATCCTCACCGTCAGATTTATCG
6_hypothetical	M20	Tb927.11.4630_for	GGGGACAAGTTTGTACAAAAAAGCAGGCTATGCAACGGCACCAGTTATATGTT	Tb927.11.4630_rev	GGGGACCACTTTGTACAAGAAAGCTGGGTTTACTCCACATCCAGCGCTCT
6_hypothetical	M21	Tb927.7.1200_for	GGGGACAAGTTTGTACAAAAAAGCAGGCTATGTCTGTCACTTGCTGCGC	Tb927.7.1200_rev	GGGGACCACTTTGTACAAGAAAGCTGGGTTCATTTCGATCCCTCTTTTGGAGC
6_hypothetical	M22	Tb927.10.7980_for	GGGGACAAGTTTGTACAAAAAAGCAGGCTATGGCAGCCCAAACACCAC	Tb927.10.7980_rev	GGGGACCACTTTGTACAAGAAAGCTGGGTTCATCGTGGAATGAGGTCGTAGA
6_hypothetical	M23	Tb927.11.6440_for	GGGGACAAGTTTGTACAAAAAAGCAGGCTATGCCTCCAAAAAACAATCCGAAAC	Tb927.11.6440_rev	GGGGACCACTTTGTACAAGAAAGCTGGGTCTACCACCTATTAGGGAGAGCGG
6_hypothetical	M24	Tb927.3.2270_for	GGGGACAAGTTTGTACAAAAAAGCAGGCTATGTCCGCCATAGAACAGGCT	Tb927.3.2270_rev	GGGGACCACTTTGTACAAGAAAGCTGGGTTTAACTTGTTACCCACCGGAAAGC
6_hypothetical	N1	Tb927.11.13170_for	GGGGACAAGTTTGTACAAAAAAGCAGGCTATGAACCGCAGAGTGCCGA	Tb927.11.13170_rev	GGGGACCACTTTGTACAAGAAAGCTGGGTCATTGCACCGGCGGGTT
6_hypothetical	N2	Tb927.7.6340_for	GGGGACAAGTTTGTACAAAAAAGCAGGCTATGTTCCATTCCAAAACCGCGC	Tb927.7.6340_rev	GGGGACCACTTTGTACAAGAAAGCTGGGTTTAATTCGTCCGTTCATGATAAGAAACAGC
6_hypothetical	N3	Tb927.1.560_for	GGGGACAAGTTTGTACAAAAAAGCAGGCTATGTTGTTCGTCGCTTCCAGC	Tb927.1.560_rev	GGGGACCACTTTGTACAAGAAAGCTGGGTCACTTGCAGCCCTCAATTTCC
6_hypothetical	N4	Tb927.3.2390_for	GGGGACAAGTTTGTACAAAAAAGCAGGCTATGAAAATATTACGCGATGTTTACAGGC	Tb927.3.2390_rev	GGGGACCACTTTGTACAAGAAAGCTGGGTCATTCCCCAACAAGGCTGTAATAATT
6_hypothetical	N5	Tb927.6.1830_for	GGGGACAAGTTTGTACAAAAAAGCAGGCTATGGAGTTTGAGACGGAGCGC	Tb927.6.1830_rev	GGGGACCACTTTGTACAAGAAAGCTGGGTTTATTGTTGCCTATTCATCTCAAAAACATC
6_hypothetical	N6	Tb927.11.4710_for	GGGGACAAGTTTGTACAAAAAAGCAGGCTATGCCACGTCAGACTACTTTGTC	Tb927.11.4710_rev	GGGGACCACTTTGTACAAGAAAGCTGGGTTCATGTCTTCAGAGCCGTGAATCT
6_hypothetical	N7	Tb927.8.3110_for	GGGGACAAGTTTGTACAAAAAAGCAGGCTATGCAGAAGCTCTTATCGCCAAG	Tb927.8.3110_rev	GGGGACCACTTTGTACAAGAAAGCTGGGTCTACCGCTTGGCAAAGCGA
6_hypothetical	N8	Tb927.7.7070_for	GGGGACAAGTTTGTACAAAAAAGCAGGCTATGCCCTCGAACAATAGCAGC	Tb927.7.7070_rev	GGGGACCACTTTGTACAAGAAAGCTGGGTTTAAGATCCGCGGACGGTGT
6_hypothetical	N9	Tb927.1.4470_for	GGGGACAAGTTTGTACAAAAAAGCAGGCTATGATAAAAAACGCGTCGCCAC	Tb927.1.4470_rev	GGGGACCACTTTGTACAAGAAAGCTGGGTCTATGCATTTGATGAGGCTGCAGT
6_hypothetical	N10	Tb927.11.4850_for	GGGGACAAGTTTGTACAAAAAAGCAGGCTATGCTTCGGCCGAGATCGG	Tb927.11.4850_rev	GGGGACCACTTTGTACAAGAAAGCTGGGTCTACCGAAGCCCAGTGTGTTTT
6_hypothetical	N11	Tb927.10.6560_for	GGGGACAAGTTTGTACAAAAAAGCAGGCTATGTCCGGGCCTGGGGTT	Tb927.10.6560_rev	GGGGACCACTTTGTACAAGAAAGCTGGGTCTAATCACTTATTATGAATGTCTCCTTTAC
6_hypothetical	N12	Tb927.8.5020_for	GGGGACAAGTTTGTACAAAAAAGCAGGCTATGGAAATGGATAGGTTGACACGC	Tb927.8.5020_rev	GGGGACCACTTTGTACAAGAAAGCTGGGTCTATTTGTTCTTCCGCTTCCTTTTTCG
6_hypothetical	N13	Tb927.10.5990_for	GGGGACAAGTTTGTACAAAAAAGCAGGCTATGTCATCTTCCCGTGTCACATCC	Tb927.10.5990_rev	GGGGACCACTTTGTACAAGAAAGCTGGGTCTACCGATGCTGGGAATATAAGGAGC
6_hypothetical	N14	Tb927.3.4420_for	GGGGACAAGTTTGTACAAAAAAGCAGGCTATGAGTATCTTTTACAACTTCCTCCCCG	Tb927.3.4420_rev	GGGGACCACTTTGTACAAGAAAGCTGGGTCCACATTTGCTCGCATTTTCCAG
6_hypothetical	N15	Tb927.8.2750_for	GGGGACAAGTTTGTACAAAAAAGCAGGCTATGCGGTGTTCAGCGCGC	Tb927.8.2750_rev	GGGGACCACTTTGTACAAGAAAGCTGGGTCTACCCCAACTCATCACCCGC
6_hypothetical	N16	Tb927.4.3360_for	GGGGACAAGTTTGTACAAAAAAGCAGGCTATGAAGGTAGCTGCGGTTGGA	Tb927.4.3360_rev	GGGGACCACTTTGTACAAGAAAGCTGGGTCACATCATAACCCCCCACTGAC
6_hypothetical	N17	Tb927.11.12980_for	GGGGACAAGTTTGTACAAAAAAGCAGGCTATGCTCCCCTCGCTCATCC	Tb927.11.12980_rev	GGGGACCACTTTGTACAAGAAAGCTGGGTTTACTTCTTACCTGCTCTCTTCCGC
6_hypothetical	N18	Tb927.8.6370_for	GGGGACAAGTTTGTACAAAAAAGCAGGCTATGAAAGAAAAAAAAGAGGTGAATTCAAGC	Tb927.8.6370_rev	GGGGACCACTTTGTACAAGAAAGCTGGGTTTATTTTATGAGTAACTCATCGGGGACG
6_hypothetical	N19	Tb927.6.4200_for	GGGGACAAGTTTGTACAAAAAAGCAGGCTATGCCGATATGCCCTGGACT	Tb927.6.4200_rev	GGGGACCACTTTGTACAAGAAAGCTGGGTTCATGCTGCAACCGTTTCTCTC
6_hypothetical	N20	Tb927.11.12970_for	GGGGACAAGTTTGTACAAAAAAGCAGGCTATGGGGGACGGAGAGCAGG	Tb927.11.12970_rev	GGGGACCACTTTGTACAAGAAAGCTGGGTTCATTTGTTTGTCACCGCAGCC
6_hypothetical	N21	Tb927.4.5070_for	GGGGACAAGTTTGTACAAAAAAGCAGGCTATGTCTACTCATGAAGTCGACGTACT	Tb927.4.5070_rev	GGGGACCACTTTGTACAAGAAAGCTGGGTTTACACATTCATTCCCCAAGGCAAA
6_hypothetical	N22	Tb927.11.8810_for	GGGGACAAGTTTGTACAAAAAAGCAGGCTATGATAAAACGAAGACAACACACAGATGT	Tb927.11.8810_rev	GGGGACCACTTTGTACAAGAAAGCTGGGTCTAACTAAGAGTACTTCCAGAGTCGATTTC
6_hypothetical	N23	Tb927.11.2220_for	GGGGACAAGTTTGTACAAAAAAGCAGGCTATGCAGCGGACGCTTCGC	Tb927.11.2220_rev	GGGGACCACTTTGTACAAGAAAGCTGGGTTCAGACTCGTCGCTCTGGG
6_hypothetical	N24	Tb927.6.2430_for	GGGGACAAGTTTGTACAAAAAAGCAGGCTATGGCTACAGAAGCGGAGGAA	Tb927.6.2430_rev	GGGGACCACTTTGTACAAGAAAGCTGGGTTCAAAACACTGTAAATGTGACGTACAAC
6_hypothetical	O1	Tb927.8.3610_for	GGGGACAAGTTTGTACAAAAAAGCAGGCTATGTCGTGGAGGGCTCTTTCG	Tb927.8.3610_rev	GGGGACCACTTTGTACAAGAAAGCTGGGTCTAAAGGTAAACCACGGCCTCG
6_hypothetical	O2	Tb927.10.8680_for	GGGGACAAGTTTGTACAAAAAAGCAGGCTATGACTTTGAGCAATGCTCTGGA	Tb927.10.8680_rev	GGGGACCACTTTGTACAAGAAAGCTGGGTTTACGGTTGCCGCTGCAAC
6_hypothetical	O3	Tb927.11.11260_for	GGGGACAAGTTTGTACAAAAAAGCAGGCTATGGCGCTTTCATTAGAGGACAG	Tb927.11.11260_rev	GGGGACCACTTTGTACAAGAAAGCTGGGTCTAGCTGGTTGCAGTGAGATGTG
6_hypothetical	O4	Tb927.8.7960_for	GGGGACAAGTTTGTACAAAAAAGCAGGCTATGACAAATGAACCATCTGAAGCAGC	Tb927.8.7960_rev	GGGGACCACTTTGTACAAGAAAGCTGGGTTTACCCAACATCATCGCCATCATC
6_hypothetical	O5	Tb927.5.2190_for	GGGGACAAGTTTGTACAAAAAAGCAGGCTATGATGGATATTACTGCGTTGTGCG	Tb927.5.2190_rev	GGGGACCACTTTGTACAAGAAAGCTGGGTCTACTGGTGCACTGCGTCATAG
6_hypothetical	O6	Tb927.10.4920_for	GGGGACAAGTTTGTACAAAAAAGCAGGCTATGGCCTTTCCTCATCGTGAAC	Tb927.10.4920_rev	GGGGACCACTTTGTACAAGAAAGCTGGGTCTAGTTGATATCTGTTTTGTTCCGCTTCC
6_hypothetical	O7	Tb927.4.2570_for	GGGGACAAGTTTGTACAAAAAAGCAGGCTATGACTAACAATCATGTAGAAAATAATTGC	Tb927.4.2570_rev	GGGGACCACTTTGTACAAGAAAGCTGGGTCTATGAGAGGTCTGTTTTCTTGTGAGC
6_hypothetical	O10	Tb927.10.5220_for	GGGGACAAGTTTGTACAAAAAAGCAGGCTATGCGTAGGGGACCACAATGG	Tb927.10.5220_rev	GGGGACCACTTTGTACAAGAAAGCTGGGTCTAAAGTGTGGGTTGCACCACA
6_hypothetical	O11	Tb927.10.3180_for	GGGGACAAGTTTGTACAAAAAAGCAGGCTATGACAAAACAGCAGATGTGCCT	Tb927.10.3180_rev	GGGGACCACTTTGTACAAGAAAGCTGGGTTTATTGAAAATGAGACGACGCACCG
6_hypothetical	O12	Tb927.11.5620_for	GGGGACAAGTTTGTACAAAAAAGCAGGCTATGAACGTTTCACTTGGTACGGC	Tb927.11.5620_rev	GGGGACCACTTTGTACAAGAAAGCTGGGTCTATCGCATCACATTGAAGCTACCG
6_hypothetical	O13	Tb927.4.3070_for	GGGGACAAGTTTGTACAAAAAAGCAGGCTATGCTGCGCCATACATGCA	Tb927.4.3070_rev	GGGGACCACTTTGTACAAGAAAGCTGGGTTTATTTTGTGTGCGAGTACAAATCCCC
6_hypothetical	O14	Tb927.7.7340_for	GGGGACAAGTTTGTACAAAAAAGCAGGCTATGGCACTGGTGGCGTCC	Tb927.7.7340_rev	GGGGACCACTTTGTACAAGAAAGCTGGGTTCAGATCAACGCCATGCGTTC

6_hypothetical	O15	Tb927.2.2920_for	GGGGACAAGTTTGTACAAAAAAGCAGGCTATGGCTGCACATATATCGGAACAC	Tb927.2.2920_rev	GGGGACCACTTTGTACAAGAAAGCTGGGTTCATGCACGTTCATCATTCCTTCTAC
6_hypothetical	O16	Tb927.11.1230_for	GGGGACAAGTTTGTACAAAAAAGCAGGCTATGGTGACCACACAACCTAACCA	Tb927.11.1230_rev	GGGGACCACTTTGTACAAGAAAGCTGGGTTTATGAAACCTTGTTAGAACCTACGGTG
6_hypothetical	O17	Tb927.10.9910_for	GGGGACAAGTTTGTACAAAAAAGCAGGCTATGCAGCATCAACTTAGAGAAGAGC	Tb927.10.9910_rev	GGGGACCACTTTGTACAAGAAAGCTGGGTTTAGTTCTCCACTGCCTCCATCC
6_hypothetical	O18	Tb927.10.15460_for	GGGGACAAGTTTGTACAAAAAAGCAGGCTATGAGTTCATGGGAATCGTGTGAGG	Tb927.10.15460_rev	GGGGACCACTTTGTACAAGAAAGCTGGGTTTAAAAGACAAATATATTCCAATGGAACGT
6_hypothetical	O19	Tb927.7.3460_for	GGGGACAAGTTTGTACAAAAAAGCAGGCTATGCTTTTCCGGAGTGTTTCATGT	Tb927.7.3460_rev	GGGGACCACTTTGTACAAGAAAGCTGGGTTCATAACCTCTCCATCGATCTCGATG
6_hypothetical	O20	Tb927.10.14030_for	GGGGACAAGTTTGTACAAAAAAGCAGGCTATGCTCAGCCTCACTTTTCTTCTTT	Tb927.10.14030_rev	GGGGACCACTTTGTACAAGAAAGCTGGGTCTACCAGAAAAACGCTTGTTTTTCTAAATC
6_hypothetical	O21	Tb927.6.3830_for	GGGGACAAGTTTGTACAAAAAAGCAGGCTATGCCACTGGAAAACAATAGCAACG	Tb927.6.3830_rev	GGGGACCACTTTGTACAAGAAAGCTGGGTTTACCGCACCTCAAATATTCCCCA
6_hypothetical	O22	Tb927.4.1550_for	GGGGACAAGTTTGTACAAAAAAGCAGGCTATGGAGCTGCGTTGCTTTTTAGC	Tb927.4.1550_rev	GGGGACCACTTTGTACAAGAAAGCTGGGTTCAGTGGCGGTAGTACACAACC
6_hypothetical	O23	Tb927.5.920_for	GGGGACAAGTTTGTACAAAAAAGCAGGCTATGCTTCGGTTTCTCCACCGC	Tb927.5.920_rev	GGGGACCACTTTGTACAAGAAAGCTGGGTCTAACGTGAACGATTGTTGTCGTCC
6_hypothetical	O24	Tb927.11.1190_for	GGGGACAAGTTTGTACAAAAAAGCAGGCTATGAGCACCTCAATTGAGGGAGA	Tb927.11.1190_rev	GGGGACCACTTTGTACAAGAAAGCTGGGTTCAGTCAACCTTCGGATCCCC
6_hypothetical	P1	Tb927.11.1760_for	GGGGACAAGTTTGTACAAAAAAGCAGGCTATGCTGGAACTGGACGGACG	Tb927.11.1760_rev	GGGGACCACTTTGTACAAGAAAGCTGGGTTCACCCCCTCACGCAGTG
6_hypothetical	P2	Tb927.4.2490_for	GGGGACAAGTTTGTACAAAAAAGCAGGCTATGAGCAACAATGTGGAGTTGTTGC	Tb927.4.2490_rev	GGGGACCACTTTGTACAAGAAAGCTGGGTTCAACCTTCACGCGCAAGTG
6_hypothetical	P3	Tb927.4.3440_for	GGGGACAAGTTTGTACAAAAAAGCAGGCTATGCACGCTCATGCAGCTG	Tb927.4.3440_rev	GGGGACCACTTTGTACAAGAAAGCTGGGTTCAGTCGCTAACGAACTCCAGT
6_hypothetical	P4	Tb927.3.4150_for	GGGGACAAGTTTGTACAAAAAAGCAGGCTATGAGCGACGTCTGCGCG	Tb927.3.4150_rev	GGGGACCACTTTGTACAAGAAAGCTGGGTTCAGCCCAGCAAATAACGCTT
6_hypothetical	P5	Tb927.11.3860_for	GGGGACAAGTTTGTACAAAAAAGCAGGCTATGAGGTGGCCACGCGGG	Tb927.11.3860_rev	GGGGACCACTTTGTACAAGAAAGCTGGGTTTATTTGCTTGAAGCCGTCGGG
6_hypothetical	P6	Tb927.11.9880_for	GGGGACAAGTTTGTACAAAAAAGCAGGCTATGCGCTACGAGAGTGTCTTACT	Tb927.11.9880_rev	GGGGACCACTTTGTACAAGAAAGCTGGGTTTAGTCAAATCCATTGAAGCCTGTTAAGA
6_hypothetical	P7	Tb927.10.12150_for	GGGGACAAGTTTGTACAAAAAAGCAGGCTATGAGGGATGTACTAGCGAGCTT	Tb927.10.12150_rev	GGGGACCACTTTGTACAAGAAAGCTGGGTTTACGAGCGAAATAATCCCCGC
6_hypothetical	P8	Tb927.10.15310_for	GGGGACAAGTTTGTACAAAAAAGCAGGCTATGTATCCAAAAAATAAGCCCCTGCC	Tb927.10.15310_rev	GGGGACCACTTTGTACAAGAAAGCTGGGTTTACAAAGGGGTTGTAGTCTCTTCCT
6_hypothetical	P9	Tb927.5.2620_for	GGGGACAAGTTTGTACAAAAAAGCAGGCTATGCACGTACTTGGCAGAGATCC	Tb927.5.2620_rev	GGGGACCACTTTGTACAAGAAAGCTGGGTTTACAGCTGTTCAAGAGAAACAGCA
6_hypothetical	P10	Tb927.9.2815_for	GGGGACAAGTTTGTACAAAAAAGCAGGCTATGGAAATCCGCATGGTGCC	Tb927.9.2815_rev	GGGGACCACTTTGTACAAGAAAGCTGGGTTCACTCATCAACTGCTGGAGTTTC
6_hypothetical	P11	Tb927.2.5890_for	GGGGACAAGTTTGTACAAAAAAGCAGGCTATGTGGCGGATCACGCGG	Tb927.2.5890_rev	GGGGACCACTTTGTACAAGAAAGCTGGGTTCATTGACCACACGGCAGGC
6_hypothetical	P12	Tb927.11.16940_for	GGGGACAAGTTTGTACAAAAAAGCAGGCTATGCCAACACGCTCCTCAGT	Tb927.11.16940_rev	GGGGACCACTTTGTACAAGAAAGCTGGGTCTATCTCCCGGAACCACTGAGAC
6_hypothetical	P13	Tb927.10.7220_for	GGGGACAAGTTTGTACAAAAAAGCAGGCTATGCTCAGTGACAAGGAGGTGA	Tb927.10.7220_rev	GGGGACCACTTTGTACAAGAAAGCTGGGTTCACCTGTGAAGAAAGTCGATAATAAGC
6_hypothetical	P14	Tb927.9.6450_for	GGGGACAAGTTTGTACAAAAAAGCAGGCTATGGTGTCCCGGGTATTCAGTG	Tb927.9.6450_rev	GGGGACCACTTTGTACAAGAAAGCTGGGTTCACTCTCCGATAGAGTACATGAACG
6_hypothetical	P15	Tb927.3.3980_for	GGGGACAAGTTTGTACAAAAAAGCAGGCTATGAAGTGCTGGTCTCCGCA	Tb927.3.3980_rev	GGGGACCACTTTGTACAAGAAAGCTGGGTTTACCCAATGGTGGTGTATGGACT
6_hypothetical	P16	Tb927.10.11660_for	GGGGACAAGTTTGTACAAAAAAGCAGGCTATGCTGCCGGCTCCGGTA	Tb927.10.11660_rev	GGGGACCACTTTGTACAAGAAAGCTGGGTCTAGTACAACTCATCAAAGTCATCCAATGC
6_hypothetical	P17	Tb927.7.2640_for	GGGGACAAGTTTGTACAAAAAAGCAGGCTATGAGCACCACTGCACTGTCT	Tb927.7.2640_rev	GGGGACCACTTTGTACAAGAAAGCTGGGTCTATTTGTCGGCCTCCCGC
6_hypothetical	P18	Tb927.10.3400_for	GGGGACAAGTTTGTACAAAAAAGCAGGCTATGGAGGCATCCGTGACAACA	Tb927.10.3400_rev	GGGGACCACTTTGTACAAGAAAGCTGGGTTCACCAACGGAGCGCAAG
6_hypothetical	P19	Tb927.10.11840_for	GGGGACAAGTTTGTACAAAAAAGCAGGCTATGTACACTACGACAGCCAAACAG	Tb927.10.11840_rev	GGGGACCACTTTGTACAAGAAAGCTGGGTTTAAACGGTATTAGTTGTGTTAACCGGA
6_hypothetical	P20	Tb927.4.3850_for	GGGGACAAGTTTGTACAAAAAAGCAGGCTATGCAAGAAGAAGCTATTTCCTCCG	Tb927.4.3850_rev	GGGGACCACTTTGTACAAGAAAGCTGGGTTTAATTAAGAAGAGAGTCCCCTTTGAAACA
6_hypothetical	P21	Tb927.11.14230_for	GGGGACAAGTTTGTACAAAAAAGCAGGCTATGAATGATAGGGAGTTGCGGGC	Tb927.11.14230_rev	GGGGACCACTTTGTACAAGAAAGCTGGGTCTACGACATAATGGCATGAAGCTGT
6_hypothetical	P22	Tb927.11.5110_for	GGGGACAAGTTTGTACAAAAAAGCAGGCTATGGCACTGAATGTCGTGAGC	Tb927.11.5110_rev	GGGGACCACTTTGTACAAGAAAGCTGGGTTCAAAAGTAACCGAGCATGTTCACC
6_hypothetical	P23	Tb927.7.1530_for	GGGGACAAGTTTGTACAAAAAAGCAGGCTATGGAGACTCACAACACCGAGC	Tb927.7.1530_rev	GGGGACCACTTTGTACAAGAAAGCTGGGTTTAAGTACCATCACATAAGCTGGGACA
6_hypothetical	P24	Tb927.6.5070_for	GGGGACAAGTTTGTACAAAAAAGCAGGCTATGCGAGCAAACCCAATTGAGC	Tb927.6.5070_rev	GGGGACCACTTTGTACAAGAAAGCTGGGTCTAATACGCCCTCCACAGTGGA
6_known	A1	Tb927.11.7950_for	GGGGACAAGTTTGTACAAAAAAGCAGGCTATGGCCCTTGAAAAGATCGACTG	Tb927.11.7950_rev	GGGGACCACTTTGTACAAGAAAGCTGGGTTCATTCACCGGAACAGGAGATGT
6_known	A2	Tb927.9.10710_for	GGGGACAAGTTTGTACAAAAAAGCAGGCTATGAATCGCCACAGGAAGTTTTACA	Tb927.9.10710_rev	GGGGACCACTTTGTACAAGAAAGCTGGGTTCAAAGCATGATCGACTCCACCA
6_known	A3	Tb927.10.10770_for	GGGGACAAGTTTGTACAAAAAAGCAGGCTATGCCGACGGAGACGTTATCA	Tb927.10.10770_rev	GGGGACCACTTTGTACAAGAAAGCTGGGTTCAGTGCACTGTGTTAACGTACG
6_known	A4	Tb927.3.2330_for	GGGGACAAGTTTGTACAAAAAAGCAGGCTATGAAGTCTCGACAACCGACGA	Tb927.3.2330_rev	GGGGACCACTTTGTACAAGAAAGCTGGGTCTAACAAATGCTTTGGTTACCCTCGG
6_known	A5	Tb927.10.14550_for	GGGGACAAGTTTGTACAAAAAAGCAGGCTATGCACGGCATGAATTTCGGC	Tb927.10.14550_rev	GGGGACCACTTTGTACAAGAAAGCTGGGTTTACATGGTGGGGCCTCCTC
6_known	A6	Tb927.7.1510_for	GGGGACAAGTTTGTACAAAAAAGCAGGCTATGTCTAAGCGTAAGAATTTTAACACGC	Tb927.7.1510_rev	GGGGACCACTTTGTACAAGAAAGCTGGGTTCACTCGCTTCCGCCGCC
6_known	A7	Tb927.11.4130_for	GGGGACAAGTTTGTACAAAAAAGCAGGCTATGCACATCAACGTCTCCGG	Tb927.11.4130_rev	GGGGACCACTTTGTACAAGAAAGCTGGGTTCACCTCCTCTCCAGTTGCTTC
6_known	A8	Tb927.11.7760_for	GGGGACAAGTTTGTACAAAAAAGCAGGCTATGGCAGTGATTATGTTCCCTCGT	Tb927.11.7760_rev	GGGGACCACTTTGTACAAGAAAGCTGGGTTTATAATAGGGAAAAGCAAGAAACGAAGAG
6_known	A9	Tb927.10.13000_for	GGGGACAAGTTTGTACAAAAAAGCAGGCTATGTTGGAAGCGCTCCGAAAG	Tb927.10.13000_rev	GGGGACCACTTTGTACAAGAAAGCTGGGTCTAGGTTCCCTGTCCAGTGTTACC
6_known	A10	Tb927.2.1820_for	GGGGACAAGTTTGTACAAAAAAGCAGGCTATGGAGTTTAGTACGGAACCTCAGG	Tb927.2.1820_rev	GGGGACCACTTTGTACAAGAAAGCTGGGTCTACATCAGTTCCGGTTTCCCCT
6_known	A11	Tb927.3.3900_for	GGGGACAAGTTTGTACAAAAAAGCAGGCTATGAAGCGCATCCCCGTAAAG	Tb927.3.3900_rev	GGGGACCACTTTGTACAAGAAAGCTGGGTCTATTTTAGTTTAGTCCCTGCCTTAAGCA
6_known	A12	Tb927.10.16040_for	GGGGACAAGTTTGTACAAAAAAGCAGGCTATGAAGCATGACTTTCCAAAGCGT	Tb927.10.16040_rev	GGGGACCACTTTGTACAAGAAAGCTGGGTTCAGCTGAAGAGGCCAACCG
6_known	A13	Tb927.3.1360_for	GGGGACAAGTTTGTACAAAAAAGCAGGCTATGTGCTCTGTTGCACCGTT	Tb927.3.1360_rev	GGGGACCACTTTGTACAAGAAAGCTGGGTTTACTTAAGGATACGACTTAAAAGTGCACG
6_known	A14	Tb927.8.4820_for	GGGGACAAGTTTGTACAAAAAAGCAGGCTATGCACGTCTACACCATCGACC	Tb927.8.4820_rev	GGGGACCACTTTGTACAAGAAAGCTGGGTTCAAGTCAAGGTCCCCTTCCC
6_known	A15	Tb927.3.1570_for	GGGGACAAGTTTGTACAAAAAAGCAGGCTATGAGTGGCCATTGGGAGGG	Tb927.3.1570_rev	GGGGACCACTTTGTACAAGAAAGCTGGGTTCACTCGTACACAATCTTTACGTAGTCA
6_known	A16	Tb927.11.11720_for	GGGGACAAGTTTGTACAAAAAAGCAGGCTATGAATAGGTGCAATCCTATCTCTCTCA	Tb927.11.11720_rev	GGGGACCACTTTGTACAAGAAAGCTGGGTCTACCTCAGCTTCAGCTCCCG
6_known	A17	Tb927.3.3670_for	GGGGACAAGTTTGTACAAAAAAGCAGGCTATGCGAGGAAGAGGGAGAGGT	Tb927.3.3670_rev	GGGGACCACTTTGTACAAGAAAGCTGGGTCTAAGAAGATTTCTTCCCCTTCCCCT
6_known	A18	Tb927.4.4310_for	GGGGACAAGTTTGTACAAAAAAGCAGGCTATGCACCGGAAGACCATCGT	Tb927.4.4310_rev	GGGGACCACTTTGTACAAGAAAGCTGGGTTTAGTACCTCCTACACGGCCTTTT
6_known	A19	Tb927.9.13510_for	GGGGACAAGTTTGTACAAAAAAGCAGGCTATGAAGCTCATTACACGGAACAATTTTG	Tb927.9.13510_rev	GGGGACCACTTTGTACAAGAAAGCTGGGTCTACTTCTCAGTCAAATGTTTGATCCGA
6_known	A20	Tb927.11.2360_for	GGGGACAAGTTTGTACAAAAAAGCAGGCTATGCCAACCCTCGCTGTTG	Tb927.11.2360_rev	GGGGACCACTTTGTACAAGAAAGCTGGGTCTACTGGGCAAACTGAACATTCAGC
6_known	A21	Tb927.11.9640_for	GGGGACAAGTTTGTACAAAAAAGCAGGCTATGAGCACCGGGCTGCCC	Tb927.11.9640_rev	GGGGACCACTTTGTACAAGAAAGCTGGGTTCACGTTGCGGCTGCCTG
6_known	A22	Tb927.4.2380_for	GGGGACAAGTTTGTACAAAAAAGCAGGCTATGTCGTCACACGGCAAATTAGT	Tb927.4.2380_rev	GGGGACCACTTTGTACAAGAAAGCTGGGTCTAAGGAAACAATTGTCCCTGCTGC
6_known	A23	Tb927.7.5040_for	GGGGACAAGTTTGTACAAAAAAGCAGGCTATGGAGCAAACCAGCAAACCC	Tb927.7.5040_rev	GGGGACCACTTTGTACAAGAAAGCTGGGTCTACACAGCGGAGTACCGCG
6_known	A24	Tb927.10.5880_for	GGGGACAAGTTTGTACAAAAAAGCAGGCTATGCTCCAGAGCAAAGTTGTGG	Tb927.10.5880_rev	GGGGACCACTTTGTACAAGAAAGCTGGGTCTAGCTTTCAGTTGGCGCTTGC
6_known	B1	Tb927.4.1990_for	GGGGACAAGTTTGTACAAAAAAGCAGGCTATGCGCCGTTTGTCAGTTACT	Tb927.4.1990_rev	GGGGACCACTTTGTACAAGAAAGCTGGGTTCACAACCTCCGCAACAGC
6_known	B2	Tb927.2.4370_for	GGGGACAAGTTTGTACAAAAAAGCAGGCTATGACGAAGTCGGCACTTGC	Tb927.2.4370_rev	GGGGACCACTTTGTACAAGAAAGCTGGGTCTACATTTGAATACGTACGGGACCAA
6_known	B3	Tb927.9.1780_for	GGGGACAAGTTTGTACAAAAAAGCAGGCTATGCCACCGCAGCGGAAG	Tb927.9.1780_rev	GGGGACCACTTTGTACAAGAAAGCTGGGTTTATGATATTCCTTCACCAAGAACAGTGAG
6_known	B4	Tb927.10.3490_for	GGGGACAAGTTTGTACAAAAAAGCAGGCTATGCGTATGATAGACAGTGAAGATGTG	Tb927.10.3490_rev	GGGGACCACTTTGTACAAGAAAGCTGGGTCTACGCTAAGTGCAAATGGCGG
6_known	B5	Tb927.8.1940_for	GGGGACAAGTTTGTACAAAAAAGCAGGCTATGGCGATGCTTCTCAACAATCTATATG	Tb927.8.1940_rev	GGGGACCACTTTGTACAAGAAAGCTGGGTTTAGTCGGCCTTTCCATAACGATATATCC
6_known	B6	Tb927.5.1140_for	GGGGACAAGTTTGTACAAAAAAGCAGGCTATGGCATTACGACCGTTCCCG	Tb927.5.1140_rev	GGGGACCACTTTGTACAAGAAAGCTGGGTTCAGGAAAGAAGCCCCTTCTCTG
6_known	B7	Tb927.6.1770_for	GGGGACAAGTTTGTACAAAAAAGCAGGCTATGGCAAAACCACTCAGCGG	Tb927.6.1770_rev	GGGGACCACTTTGTACAAGAAAGCTGGGTTTACGCTTTCAGTTTCTCAAGATGGT
6_known	B8	Tb927.11.1820_for	GGGGACAAGTTTGTACAAAAAAGCAGGCTATGTCCTCCGCAGTGGGAG	Tb927.11.1820_rev	GGGGACCACTTTGTACAAGAAAGCTGGGTTCACGTAGCCGACATCAAATAAAGATC
6_known	B9	Tb927.11.16880_for	GGGGACAAGTTTGTACAAAAAAGCAGGCTATGCTCTTTGAACCTTCGGGGG	Tb927.11.16880_rev	GGGGACCACTTTGTACAAGAAAGCTGGGTTCATTTCTTACCGCGCGGC
6_known	B10	Tb927.11.7560_for	GGGGACAAGTTTGTACAAAAAAGCAGGCTATGGCAGCGAAGGAGTACGA	Tb927.11.7560_rev	GGGGACCACTTTGTACAAGAAAGCTGGGTTTACAGCGTCTTAAACTCCAAGTCAC
6_known	B11	Tb927.11.14590_for	GGGGACAAGTTTGTACAAAAAAGCAGGCTATGTCACTAAACCCTAACGCTCCT	Tb927.11.14590_rev	GGGGACCACTTTGTACAAGAAAGCTGGGTTTACCCAGAAGCTTCATTCTGCAC
6_known	B12	Tb927.11.8380_for	GGGGACAAGTTTGTACAAAAAAGCAGGCTATGCTATTAGTGGTGTCGGCCA	Tb927.11.8380_rev	GGGGACCACTTTGTACAAGAAAGCTGGGTTCACGCCGTCCAGACATCC
6_known	B13	Tb927.8.2550_for	GGGGACAAGTTTGTACAAAAAAGCAGGCTATGCTCCGCATCACCTTACCA	Tb927.8.2550_rev	GGGGACCACTTTGTACAAGAAAGCTGGGTTTACGCTCCTTGTGGAGGGATG
6_known	B14	Tb927.10.3140_for	GGGGACAAGTTTGTACAAAAAAGCAGGCTATGTACACCGTGGCTCCTGC	Tb927.10.3140_rev	GGGGACCACTTTGTACAAGAAAGCTGGGTTCATTCAAGACACGCGCTCTC
6_known	B15	Tb927.10.12640_for	GGGGACAAGTTTGTACAAAAAAGCAGGCTATGAACAATGACCTTGAGCGATCAA	Tb927.10.12640_rev	GGGGACCACTTTGTACAAGAAAGCTGGGTTCAACCCTGTGGTTTCCTCAGC
6_known	B16	Tb11.02.5420_for	GGGGACAAGTTTGTACAAAAAAGCAGGCTATGATTTTCCTCGTGATATCCACCG	Tb11.02.5420_rev	GGGGACCACTTTGTACAAGAAAGCTGGGTTCACGCCGTCCAGACATCC
6_known	B17	Tb927.10.1630_for	GGGGACAAGTTTGTACAAAAAAGCAGGCTATGCCACCTAAAAACAGGCAAGAT	Tb927.10.1630_rev	GGGGACCACTTTGTACAAGAAAGCTGGGTCTACTCGTTGCTTTCATCATCTTCGT
6_known	B18	Tb927.3.4560_for	GGGGACAAGTTTGTACAAAAAAGCAGGCTATGCCGGAAGGTGAACAGGC	Tb927.3.4560_rev	GGGGACCACTTTGTACAAGAAAGCTGGGTTCACGCCAATTTCTGGACAAGAG
6_known	B19	Tb927.5.4420_for	GGGGACAAGTTTGTACAAAAAAGCAGGCTATGTCTTCCAGAGCTGAGGAAGG	Tb927.5.4420_rev	GGGGACCACTTTGTACAAGAAAGCTGGGTCTAATAACGCCTTTGAGTCGACCG
6_known	B20	Tb927.8.8140_for	GGGGACAAGTTTGTACAAAAAAGCAGGCTATGCGTGTGGAGGACCCG	Tb927.8.8140_rev	GGGGACCACTTTGTACAAGAAAGCTGGGTTCAGCAACCTGAACGTTTAGACC
6_known	B21	Tb927.6.3570_for	GGGGACAAGTTTGTACAAAAAAGCAGGCTATGATAAAACTGTGCCGATCGCT	Tb927.6.3570_rev	GGGGACCACTTTGTACAAGAAAGCTGGGTTCAAGCCTCCAATGCCCACA
6_known	B22	Tb927.11.16090_for	GGGGACAAGTTTGTACAAAAAAGCAGGCTATGCCTCCGAGCCAGCTG	Tb927.11.16090_rev	GGGGACCACTTTGTACAAGAAAGCTGGGTTTACTTTCTGCGGAGTATAGGCGG
6_known	B23	Tb927.6.1510_for	GGGGACAAGTTTGTACAAAAAAGCAGGCTATGCGCCGTTGGGGAGCG	Tb927.6.1510_rev	GGGGACCACTTTGTACAAGAAAGCTGGGTTCAGGAACGTGGTCTACCACAC
6_known	B24	Tb927.4.1090_for	GGGGACAAGTTTGTACAAAAAAGCAGGCTATGGGCTACAAGTTTCACTTTTACCG	Tb927.4.1090_rev	GGGGACCACTTTGTACAAGAAAGCTGGGTTTAGTCAGCTTTTATGGAGTTGTAAATAGT
6_known	C1	Tb927.6.1760_for	GGGGACAAGTTTGTACAAAAAAGCAGGCTATGGCTTTCCGTACCTCAACTATTTTC	Tb927.6.1760_rev	GGGGACCACTTTGTACAAGAAAGCTGGGTTTACGACGAGCCGCTCCC
6_known	C2	Tb927.5.4380_for	GGGGACAAGTTTGTACAAAAAAGCAGGCTATGGATCCACCTAATGTACCTCCC	Tb927.5.4380_rev	GGGGACCACTTTGTACAAGAAAGCTGGGTTTAAATGCGCTTAAGACGCGGT
6_known	C3	Tb927.11.12480_for	GGGGACAAGTTTGTACAAAAAAGCAGGCTATGTCATCGTCAGAAGCGGTGA	Tb927.11.12480_rev	GGGGACCACTTTGTACAAGAAAGCTGGGTTCACCGCGGCCCGTTTTC
6_known	C4	Tb927.10.6540_for	GGGGACAAGTTTGTACAAAAAAGCAGGCTATGGGATTCTTCTCGTTTAAGAGCAAC	Tb927.10.6540_rev	GGGGACCACTTTGTACAAGAAAGCTGGGTTTAACTACAAAATTCCTTTGGTGGGACC
6_known	C5	Tb927.7.3650_for	GGGGACAAGTTTGTACAAAAAAGCAGGCTATGTCCAATTCGAGTGGCAAAACA	Tb927.7.3650_rev	GGGGACCACTTTGTACAAGAAAGCTGGGTTCAAATATTGGGGAAAGAAACACCCA
6_known	C6	Tb927.4.880_for	GGGGACAAGTTTGTACAAAAAAGCAGGCTATGAGTCACCATATCCAACTGCCT	Tb927.4.880_rev	GGGGACCACTTTGTACAAGAAAGCTGGGTTCATGTAGCCGCTGAAAAAGTCAT
6_known	C7	Tb927.7.5650_for	GGGGACAAGTTTGTACAAAAAAGCAGGCTATGGCTGAAAAGTACAGGTGGTG	Tb927.7.5650_rev	GGGGACCACTTTGTACAAGAAAGCTGGGTTTATACTTGACACCTCCCGTCGAT
6_known	C8	Tb927.7.2580_for	GGGGACAAGTTTGTACAAAAAAGCAGGCTATGCGTCACGGAGCATCCC	Tb927.7.2580_rev	GGGGACCACTTTGTACAAGAAAGCTGGGTCTATGCATCTATCGGCGAAGCG
6_known	C9	Tb927.4.2560_for	GGGGACAAGTTTGTACAAAAAAGCAGGCTATGACCTTCAATGGAAGGTTTCGTG	Tb927.4.2560_rev	GGGGACCACTTTGTACAAGAAAGCTGGGTCTAGTCTATCTCGGAGGAGAGATCGG
6_known	C10	Tb927.6.2030_for	GGGGACAAGTTTGTACAAAAAAGCAGGCTATGTCCCTCTTGGAAAATTGCCAC	Tb927.6.2030_rev	GGGGACCACTTTGTACAAGAAAGCTGGGTTCAGCGGCTGACAATGGGC
6_known	C11	Tb927.11.13220_for	GGGGACAAGTTTGTACAAAAAAGCAGGCTATGACTTACAAACATATGGCAAACCACC	Tb927.11.13220_rev	GGGGACCACTTTGTACAAGAAAGCTGGGTTCACTGGCCTTCCATTAAAAGACAAT
6_known	C12	Tb927.7.710_for	GGGGACAAGTTTGTACAAAAAAGCAGGCTATGCCTGCTCCGGCGATC	Tb927.7.710_rev	GGGGACCACTTTGTACAAGAAAGCTGGGTTTAGTCAACATCATCGGGGACTGG
6_known	C13	Tb927.10.14770_for	GGGGACAAGTTTGTACAAAAAAGCAGGCTATGGAATCCACTGACACAGAAGTGA	Tb927.10.14770_rev	GGGGACCACTTTGTACAAGAAAGCTGGGTCTACACCTTCTTGACTTGGGACG
6_known	C14	Tb927.10.9080_for	GGGGACAAGTTTGTACAAAAAAGCAGGCTATGAGTTTCGCCCCTACGCG	Tb927.10.9080_rev	GGGGACCACTTTGTACAAGAAAGCTGGGTTTAAGTACTACCGCTCTCCTGCAC
6_known	C15	Tb927.11.3130_for	GGGGACAAGTTTGTACAAAAAAGCAGGCTATGAGTTCTGTACTTGACGGGCT	Tb927.11.3130_rev	GGGGACCACTTTGTACAAGAAAGCTGGGTTCAAATGGTTACCCTGGGCTGC
6_known	C16	Tb927.4.5200_for	GGGGACAAGTTTGTACAAAAAAGCAGGCTATGAGTGTGCTGGGATCAGGC	Tb927.4.5200_rev	GGGGACCACTTTGTACAAGAAAGCTGGGTTCAGGAGGAAGCGACGCG
6_known	C17	Tb927.9.1970_for	GGGGACAAGTTTGTACAAAAAAGCAGGCTATGATGAGCAACCATGCAAAGCT	Tb927.9.1970_rev	GGGGACCACTTTGTACAAGAAAGCTGGGTTTATTCACTCAGTACCTTAACAAACTCATT
6_known	C18	Tb927.11.14380_for	GGGGACAAGTTTGTACAAAAAAGCAGGCTATGTTTGCCTGTCGTCGGC	Tb927.11.14380_rev	GGGGACCACTTTGTACAAGAAAGCTGGGTTTACCACCGTCTTTTGCGTCG
6_known	C19	Tb927.11.1030_for	GGGGACAAGTTTGTACAAAAAAGCAGGCTATGAGCGGATATGGCGAATCTATGC	Tb927.11.1030_rev	GGGGACCACTTTGTACAAGAAAGCTGGGTTCACCGCGTTCTCCCCCC
6_known	C20	Tb927.7.7180_for	GGGGACAAGTTTGTACAAAAAAGCAGGCTATGGCGAAGCGAAGCCGC	Tb927.7.7180_rev	GGGGACCACTTTGTACAAGAAAGCTGGGTTTACCAACCGTGACGATTCTTAGC
6_known	C21	Tb927.3.4190_for	GGGGACAAGTTTGTACAAAAAAGCAGGCTATGACTCGCTTCTCGTGTGTTG	Tb927.3.4190_rev	GGGGACCACTTTGTACAAGAAAGCTGGGTTCAGTCCACCTTGATGCTTCCA
6_known	C22	Tb927.7.7110_for	GGGGACAAGTTTGTACAAAAAAGCAGGCTATGGTGAAGCGACTCGGCT	Tb927.7.7110_rev	GGGGACCACTTTGTACAAGAAAGCTGGGTTTACCAACCGTGACGATTCTTAGC
6_known	C23	Tb927.8.3680_for	GGGGACAAGTTTGTACAAAAAAGCAGGCTATGGGAGACAATCCACTGTCTGT	Tb927.8.3680_rev	GGGGACCACTTTGTACAAGAAAGCTGGGTTTAATCACGACTTATAGCGAAACGTAACA
6_known	C24	Tb927.10.7360_for	GGGGACAAGTTTGTACAAAAAAGCAGGCTATGGAGGGCAATGGCAGCT	Tb927.10.7360_rev	GGGGACCACTTTGTACAAGAAAGCTGGGTTCACTGAAGATACTTCGCACGC
6_known	D1	Tb927.9.6310_for	GGGGACAAGTTTGTACAAAAAAGCAGGCTATGCCGGATTCACCTGAAACTG	Tb927.9.6310_rev	GGGGACCACTTTGTACAAGAAAGCTGGGTTCACTTCCCCTCTTCCGTTTCA
6_known	D2	Tb927.11.190_for	GGGGACAAGTTTGTACAAAAAAGCAGGCTATGGCGATGCGACGTTTGT	Tb927.11.190_rev	GGGGACCACTTTGTACAAGAAAGCTGGGTTCATTTCCTTTTGGTAGCGTTGCG
6_known	D3	Tb927.4.5180_for	GGGGACAAGTTTGTACAAAAAAGCAGGCTATGAAAAACATTGAGGTTAACCCCCA	Tb927.4.5180_rev	GGGGACCACTTTGTACAAGAAAGCTGGGTTCAGGAGAGATTGCTCAACTTCTCC
6_known	D4	Tb927.8.5570_for	GGGGACAAGTTTGTACAAAAAAGCAGGCTATGATCGCCGACGCGGTG	Tb927.8.5570_rev	GGGGACCACTTTGTACAAGAAAGCTGGGTTCATCTAACTATATAAATTGACAACCCGTA
6_known	D5	Tb927.9.1670_for	GGGGACAAGTTTGTACAAAAAAGCAGGCTATGAGCACACGTCTGTGCG	Tb927.9.1670_rev	GGGGACCACTTTGTACAAGAAAGCTGGGTCTATCTCACGTCATATGACGACCCT
6_known	D6	Tb927.8.7220_for	GGGGACAAGTTTGTACAAAAAAGCAGGCTATGAAAAACAACGACGCTAACCATATG	Tb927.8.7220_rev	GGGGACCACTTTGTACAAGAAAGCTGGGTTCAGGGGATGTTACTTAACTTCTCCTC
6_known	D7	Tb927.11.15470_for	GGGGACAAGTTTGTACAAAAAAGCAGGCTATGCGGCAGTGCATATCGAG	Tb927.11.15470_rev	GGGGACCACTTTGTACAAGAAAGCTGGGTTCAACAGGATGCCAGGTTGC
6_known	D8	Tb927.8.7520_for	GGGGACAAGTTTGTACAAAAAAGCAGGCTATGCACCGTTGGCGTCCA	Tb927.8.7520_rev	GGGGACCACTTTGTACAAGAAAGCTGGGTTCACGTACAGTACAGCGCAGC
6_known	D9	Tb927.1.1540_for	GGGGACAAGTTTGTACAAAAAAGCAGGCTATGACAGAAAGAGAGCTTGTCACG	Tb927.1.1540_rev	GGGGACCACTTTGTACAAGAAAGCTGGGTTCAGTTCATGCTAATATCATCACCGTTATC
6_known	D10	Tb927.9.6880_for	GGGGACAAGTTTGTACAAAAAAGCAGGCTATGGAAGGAAAAAATGAGAGAGACCAATAC	Tb927.9.6880_rev	GGGGACCACTTTGTACAAGAAAGCTGGGTCTACTTTCTTCCACGGTCCATACCC
6_known	D11	Tb927.1.2820_for	GGGGACAAGTTTGTACAAAAAAGCAGGCTATGGGTCGACAGGAAGATGGTT	Tb927.1.2820_rev	GGGGACCACTTTGTACAAGAAAGCTGGGTTCAGGACCTCTTGGGCGC
6_known	D13	Tb927.11.8730_for	GGGGACAAGTTTGTACAAAAAAGCAGGCTATGCGCAAGGGGGGAAATG	Tb927.11.8730_rev	GGGGACCACTTTGTACAAGAAAGCTGGGTCTAGTAACGCACTGCGCTCG
6_known	D15	Tb927.4.670_for	GGGGACAAGTTTGTACAAAAAAGCAGGCTATGGATTTTGTGGAAGTGCTTCCTG	Tb927.4.670_rev	GGGGACCACTTTGTACAAGAAAGCTGGGTTCAACGAGGTGAGACACTGCG
6_known	D16	Tb927.7.1610_for	GGGGACAAGTTTGTACAAAAAAGCAGGCTATGCGCCCAGCTCCGATG	Tb927.7.1610_rev	GGGGACCACTTTGTACAAGAAAGCTGGGTTCAAAGTTTGTTGAAGTCCTCTGTGC
6_known	D17	Tb927.11.7460_for	GGGGACAAGTTTGTACAAAAAAGCAGGCTATGTCGAGGATGTGGCTGACC	Tb927.11.7460_rev	GGGGACCACTTTGTACAAGAAAGCTGGGTTTACAGATCGTCCATGGGTTGTGG
6_known	D19	Tb927.5.3170_for	GGGGACAAGTTTGTACAAAAAAGCAGGCTATGCGCACAGGAAAGGGTCG	Tb927.5.3170_rev	GGGGACCACTTTGTACAAGAAAGCTGGGTTTACTTTTCAAAGAGCGTGGTGAGAG
6_known	D20	Tb927.10.11940_for	GGGGACAAGTTTGTACAAAAAAGCAGGCTATGACGCTATGGAGTGCGACG	Tb927.10.11940_rev	GGGGACCACTTTGTACAAGAAAGCTGGGTTTACCGTATGGAATAAAACTTGTTTTCCTT
6_known	D21	Tb927.10.14980_for	GGGGACAAGTTTGTACAAAAAAGCAGGCTATGACGGATGCCCCAAGGG	Tb927.10.14980_rev	GGGGACCACTTTGTACAAGAAAGCTGGGTTCAAAGCTCCTCAATTTCATAGACGAAA
6_known	D22	Tb927.5.1100_for	GGGGACAAGTTTGTACAAAAAAGCAGGCTATGGACTGCGGCGCCGGT	Tb927.5.1100_rev	GGGGACCACTTTGTACAAGAAAGCTGGGTTCACAGTAACATAGACTGCAGGCC
6_known	D23	Tb927.4.1820_for	GGGGACAAGTTTGTACAAAAAAGCAGGCTATGACAGAAGTTAATGATGTTGCCTCTG	Tb927.4.1820_rev	GGGGACCACTTTGTACAAGAAAGCTGGGTCTACTGCTGAAGCTGACTGTGG
6_known	D24	Tb927.10.5250_for	GGGGACAAGTTTGTACAAAAAAGCAGGCTATGTCTGGCACTAATCACCTCCG	Tb927.10.5250_rev	GGGGACCACTTTGTACAAGAAAGCTGGGTTCATCTCTGTTTCTGCGTGGC
6_known	E1	Tb927.11.3120_for	GGGGACAAGTTTGTACAAAAAAGCAGGCTATGTCGACAATCTACAATTTCAAAACGG	Tb927.11.3120_rev	GGGGACCACTTTGTACAAGAAAGCTGGGTTCAGCGTCTGTCGCGTCT
6_known	E2	Tb927.11.5000_for	GGGGACAAGTTTGTACAAAAAAGCAGGCTATGGATACCAATGCACGCCG	Tb927.11.5000_rev	GGGGACCACTTTGTACAAGAAAGCTGGGTTCACGCCTGCCCCAGGAG
6_known	E3	Tb927.6.3800_for	GGGGACAAGTTTGTACAAAAAAGCAGGCTATGTTGGCCCGTCGTGTATGC	Tb927.6.3800_rev	GGGGACCACTTTGTACAAGAAAGCTGGGTTTACTCCTTCTTTTCACCAGATGCC

6_known	E4	Tb927.9.9580_for	GGGGACAAGTTTGTACAAAAAAGCAGGCTATGGCCGAACCCGTGCTT	Tb927.9.9580_rev	GGGGACCACTTTGTACAAGAAAGCTGGGTTCACGCTGTCTTCTTTTCCTGC
6_known	E5	Tb927.11.16790_for	GGGGACAAGTTTGTACAAAAAAGCAGGCTATGGATGCGTACGAAACACTTGG	Tb927.11.16790_rev	GGGGACCACTTTGTACAAGAAAGCTGGGTTCATCGTTTTGTTGCACCATCTCG
6_known	E6	Tb927.11.960_for	GGGGACAAGTTTGTACAAAAAAGCAGGCTATGATTAGGGTGCCCTCCCG	Tb927.11.960_rev	GGGGACCACTTTGTACAAGAAAGCTGGGTTCATACAGCTCCCGAGTCGG
6_known	E7	Tb927.10.7620_for	GGGGACAAGTTTGTACAAAAAAGCAGGCTATGCACCGGCGCTGTCCC	Tb927.10.7620_rev	GGGGACCACTTTGTACAAGAAAGCTGGGTTTACGTTATGGAAACGGGGCG
6_known	E9	Tb927.6.3750_for	GGGGACAAGTTTGTACAAAAAAGCAGGCTATGTTGGCCCGCCGTGTA	Tb927.6.3750_rev	GGGGACCACTTTGTACAAGAAAGCTGGGTTTACTCCTTCTTTTCACCAGATGCC
6_known	E10	Tb927.11.11480_for	GGGGACAAGTTTGTACAAAAAAGCAGGCTATGCCTGACAGCGAGGGT	Tb927.11.11480_rev	GGGGACCACTTTGTACAAGAAAGCTGGGTCTACGAAACGGGCACGAACTC
6_known	E11	Tb927.1.1960_for	GGGGACAAGTTTGTACAAAAAAGCAGGCTATGATAGTACTTCCCACTTTTGATTTCCC	Tb927.1.1960_rev	GGGGACCACTTTGTACAAGAAAGCTGGGTCTACTTGCGCTTATTCATAGCACAGA
6_known	E12	Tb927.7.2100_for	GGGGACAAGTTTGTACAAAAAAGCAGGCTATGTCAGTTCAACAGGAGCCGG	Tb927.7.2100_rev	GGGGACCACTTTGTACAAGAAAGCTGGGTTTATTCCCATTCCGTCGTCCCC
6_known	E13	Tb927.3.1610_for	GGGGACAAGTTTGTACAAAAAAGCAGGCTATGCAACCAACGCGAAGATGC	Tb927.3.1610_rev	GGGGACCACTTTGTACAAGAAAGCTGGGTCTAAACAGGTGAAGTAAAAAAAGGATGACT
6_known	E14	Tb927.8.1690_for	GGGGACAAGTTTGTACAAAAAAGCAGGCTATGAATGAATATCATATCATAGAAAAGATG	Tb927.8.1690_rev	GGGGACCACTTTGTACAAGAAAGCTGGGTTCATGCTGGTCCCGCCAC
6_known	E16	Tb927.7.5560_for	GGGGACAAGTTTGTACAAAAAAGCAGGCTATGTGCGGGATATTCGGTTACATTC	Tb927.7.5560_rev	GGGGACCACTTTGTACAAGAAAGCTGGGTTCATTGAACCGTTACACTCTTCGC
6_known	E17	Tb927.3.4720_for	GGGGACAAGTTTGTACAAAAAAGCAGGCTATGGAGCGACTCATCTCGGTC	Tb927.3.4720_rev	GGGGACCACTTTGTACAAGAAAGCTGGGTCTAAATAAGAGCAAACTCTCGAACACTGT
6_known	E19	Tb927.5.1760_for	GGGGACAAGTTTGTACAAAAAAGCAGGCTATGCGGAGTTATGTATTTGGCCAA	Tb927.5.1760_rev	GGGGACCACTTTGTACAAGAAAGCTGGGTCTAGAATGGGCACACGTATGAAGC
6_known	E20	Tb927.9.11070_for	GGGGACAAGTTTGTACAAAAAAGCAGGCTATGGTGATCCGACCTGCAACC	Tb927.9.11070_rev	GGGGACCACTTTGTACAAGAAAGCTGGGTTTACAACACAATGGGATGATCCGC
6_known	E21	Tb927.5.1830_for	GGGGACAAGTTTGTACAAAAAAGCAGGCTATGATGTGTACCGCAACCGTTATG	Tb927.5.1830_rev	GGGGACCACTTTGTACAAGAAAGCTGGGTCTAGTTCTGAGGATCTATAAGACCTTCAGC
6_known	E22	Tb927.11.11330_for	GGGGACAAGTTTGTACAAAAAAGCAGGCTATGACATACGAAGGCGCCATTG	Tb927.11.11330_rev	GGGGACCACTTTGTACAAGAAAGCTGGGTTTAGTCAACCTCCTCGACTTTAGGC
6_known	E23	Tb927.11.1660_for	GGGGACAAGTTTGTACAAAAAAGCAGGCTATGTCTGACCAGATGCGGCG	Tb927.11.1660_rev	GGGGACCACTTTGTACAAGAAAGCTGGGTTCAGCAAGGGCCCTTCGC
6_known	E24	Tb927.10.2210_for	GGGGACAAGTTTGTACAAAAAAGCAGGCTATGGGGGGAAGTTATTCCACGG	Tb927.10.2210_rev	GGGGACCACTTTGTACAAGAAAGCTGGGTTTACGCAACTCGCTCGTACAG
6_known	F1	Tb927.1.1420_for	GGGGACAAGTTTGTACAAAAAAGCAGGCTATGGGTGCCTTTCCGTCTCA	Tb927.1.1420_rev	GGGGACCACTTTGTACAAGAAAGCTGGGTCTACTGTTGAGCTACTTGCCCCG
6_known	F2	Tb927.1.1500_for	GGGGACAAGTTTGTACAAAAAAGCAGGCTATGGGAGGTGCGCCTTCT	Tb927.1.1500_rev	GGGGACCACTTTGTACAAGAAAGCTGGGTCTACGACAGCGAGCTTGGAATG
6_known	F3	Tb927.11.4490_for	GGGGACAAGTTTGTACAAAAAAGCAGGCTATGAGGCGTTTGCTCGGC	Tb927.11.4490_rev	GGGGACCACTTTGTACAAGAAAGCTGGGTTCAAAACAGATCATCATAGTCGTCCAC
6_known	F4	Tb927.11.540_for	GGGGACAAGTTTGTACAAAAAAGCAGGCTATGGTCCGCCACGCTCAT	Tb927.11.540_rev	GGGGACCACTTTGTACAAGAAAGCTGGGTTCACACCTCCGCCCCTGC
6_known	F5	Tb927.10.9050_for	GGGGACAAGTTTGTACAAAAAAGCAGGCTATGACTGTAGTCGAGTCTCCTTTCA	Tb927.10.9050_rev	GGGGACCACTTTGTACAAGAAAGCTGGGTTCATCCAGCGGATGGCTCA
6_known	F6	Tb927.8.6650_for	GGGGACAAGTTTGTACAAAAAAGCAGGCTATGACAGACCACCAAGTCATCTGC	Tb927.8.6650_rev	GGGGACCACTTTGTACAAGAAAGCTGGGTTCAACCTACACGCATGGTGGG
6_known	F7	Tb927.8.3310_for	GGGGACAAGTTTGTACAAAAAAGCAGGCTATGTCGCTAAAGCGCCCTCG	Tb927.8.3310_rev	GGGGACCACTTTGTACAAGAAAGCTGGGTTCATTTACCGAACATAAATCCCTTCAGC
6_known	F8	Tb927.4.2310_for	GGGGACAAGTTTGTACAAAAAAGCAGGCTATGTCGGAGGAAATAATTACAAAGCTTCG	Tb927.4.2310_rev	GGGGACCACTTTGTACAAGAAAGCTGGGTTTAGGGTTGGCATCGACCCA
6_known	F9	Tb927.1.2600_for	GGGGACAAGTTTGTACAAAAAAGCAGGCTATGGAAGTACGCGATGTGAATACAC	Tb927.1.2600_rev	GGGGACCACTTTGTACAAGAAAGCTGGGTCTAACATTCTCCGTCATCACAATCAACC
6_known	F10	Tb927.7.2250_for	GGGGACAAGTTTGTACAAAAAAGCAGGCTATGCGCCTTTCCTCTGTTTGC	Tb927.7.2250_rev	GGGGACCACTTTGTACAAGAAAGCTGGGTCTAATCCTCGAGCATATCGTAGCAATC
6_known	F11	Tb927.3.1590_for	GGGGACAAGTTTGTACAAAAAAGCAGGCTATGTACCATCGTGGCTACGGC	Tb927.3.1590_rev	GGGGACCACTTTGTACAAGAAAGCTGGGTTCATCTTTTCGCAGTAACGGTCC
6_known	F12	Tb927.10.7700_for	GGGGACAAGTTTGTACAAAAAAGCAGGCTATGCGCTGCTTTCGAAAAAAGG	Tb927.10.7700_rev	GGGGACCACTTTGTACAAGAAAGCTGGGTTTATAGTTTGTGTCGCGCCGC
6_known	F13	Tb927.8.6170_for	GGGGACAAGTTTGTACAAAAAAGCAGGCTATGTCGTTTAATGATAACCTGGCCAC	Tb927.8.6170_rev	GGGGACCACTTTGTACAAGAAAGCTGGGTTCACAAATGGGACCGCTTCG
6_known	F14	Tb927.11.9110_for	GGGGACAAGTTTGTACAAAAAAGCAGGCTATGCAAAGATTGGCAGGGAAGC	Tb927.11.9110_rev	GGGGACCACTTTGTACAAGAAAGCTGGGTTTAGAAGTTACCGACACGCTTCG
6_known	F15	Tb927.1.4780_for	GGGGACAAGTTTGTACAAAAAAGCAGGCTATGGCGGCCTTCTTCGGT	Tb927.1.4780_rev	GGGGACCACTTTGTACAAGAAAGCTGGGTTCATGACGCGCTGAAAGAGG
6_known	F16	Tb927.6.1940_for	GGGGACAAGTTTGTACAAAAAAGCAGGCTATGATTGTACCTCCTCCCTGGGG	Tb927.6.1940_rev	GGGGACCACTTTGTACAAGAAAGCTGGGTTCAAGCAACTGGCGCCGC
6_known	F17	Tb927.5.3940_for	GGGGACAAGTTTGTACAAAAAAGCAGGCTATGTCGTCTTCAGACATCGCG	Tb927.5.3940_rev	GGGGACCACTTTGTACAAGAAAGCTGGGTTCAGTTAAGCAGGAGGGCGT
6_known	F18	Tb927.8.3060_for	GGGGACAAGTTTGTACAAAAAAGCAGGCTATGCTCAAGAGAGTTATTGCGAGG	Tb927.8.3060_rev	GGGGACCACTTTGTACAAGAAAGCTGGGTTCAATTGCCAGACCTTTTCTTTCCT
6_known	F19	Tb927.6.160_for	GGGGACAAGTTTGTACAAAAAAGCAGGCTATGAATCAGAACGGTGGTAGTGTGG	Tb927.6.160_rev	GGGGACCACTTTGTACAAGAAAGCTGGGTCTAATTGTTAACTCCGTTGTTATTGTTACC
6_known	F20	Tb927.3.3590_for	GGGGACAAGTTTGTACAAAAAAGCAGGCTATGGTAACTGCGATGGCAAACA	Tb927.3.3590_rev	GGGGACCACTTTGTACAAGAAAGCTGGGTCTATGCAGCAGCCTTCCGTAG
6_known	F21	Tb927.6.4710_for	GGGGACAAGTTTGTACAAAAAAGCAGGCTATGACGCACGTTGGTGCTG	Tb927.6.4710_rev	GGGGACCACTTTGTACAAGAAAGCTGGGTCTACATCCCAACACGGCGC
6_known	F22	Tb927.8.2520_for	GGGGACAAGTTTGTACAAAAAAGCAGGCTATGCGTGACACAGGTCGC	Tb927.8.2520_rev	GGGGACCACTTTGTACAAGAAAGCTGGGTTCACCCCACTGTTTGCTTCCT
6_known	F23	Tb927.11.5340_for	GGGGACAAGTTTGTACAAAAAAGCAGGCTATGAGGGTGATGAGACACCTCTC	Tb927.11.5340_rev	GGGGACCACTTTGTACAAGAAAGCTGGGTTCACTCAAACAGCACCTTGCG
6_known	F24	Tb927.6.1140_for	GGGGACAAGTTTGTACAAAAAAGCAGGCTATGTGGCTCTTTCTCGGGGC	Tb927.6.1140_rev	GGGGACCACTTTGTACAAGAAAGCTGGGTTTACTTTGGGTTTTGTTTGCTCAGC
6_known	G1	Tb927.2.240_for	GGGGACAAGTTTGTACAAAAAAGCAGGCTATGAATCAGAACGGCGGTAGTGT	Tb927.2.240_rev	GGGGACCACTTTGTACAAGAAAGCTGGGTTTACCTTCGGCCCACAGCA
6_known	G3	Tb927.9.5490_for	GGGGACAAGTTTGTACAAAAAAGCAGGCTATGAGTGGATCGTTGAACGTGG	Tb927.9.5490_rev	GGGGACCACTTTGTACAAGAAAGCTGGGTCTACTCCAGAAGTCCTCGTCGG
6_known	G4	Tb927.10.9820_for	GGGGACAAGTTTGTACAAAAAAGCAGGCTATGTTGCGGCGTGTCACC	Tb927.10.9820_rev	GGGGACCACTTTGTACAAGAAAGCTGGGTTCACACCCATATGTCGATTTCATCG
6_known	G5	Tb927.1.420_for	GGGGACAAGTTTGTACAAAAAAGCAGGCTATGAATCAGAATGGCGGTAGTGTG	Tb927.1.420_rev	GGGGACCACTTTGTACAAGAAAGCTGGGTTTACCTTCGGCCCACAGCA
6_known	G6	Tb927.11.7410_for	GGGGACAAGTTTGTACAAAAAAGCAGGCTATGCTTCACCATAAATTAGATACAGTGAGC	Tb927.11.7410_rev	GGGGACCACTTTGTACAAGAAAGCTGGGTTTAGAAGGGAAGCATCGATCGGT
6_known	G7	Tb927.4.250_for	GGGGACAAGTTTGTACAAAAAAGCAGGCTATGAATCAGAACGGCGGTAGTGT	Tb927.4.250_rev	GGGGACCACTTTGTACAAGAAAGCTGGGTTTACCTTCGGCCCGCAGC
6_known	G8	Tb927.4.3390_for	GGGGACAAGTTTGTACAAAAAAGCAGGCTATGTGGCGGCGGACATGG	Tb927.4.3390_rev	GGGGACCACTTTGTACAAGAAAGCTGGGTCTATTGCCAGCAGCGAGCC
6_known	G10	Tb927.7.4390_for	GGGGACAAGTTTGTACAAAAAAGCAGGCTATGCTCACCTTACGCGTATGTG	Tb927.7.4390_rev	GGGGACCACTTTGTACAAGAAAGCTGGGTTCAGCAAGAGCCCTCACTGG
6_known	G11	Tb927.11.11290_for	GGGGACAAGTTTGTACAAAAAAGCAGGCTATGACATACGAAGGCGCCATTG	Tb927.11.11290_rev	GGGGACCACTTTGTACAAGAAAGCTGGGTTTACTCTGTGTTTGCGTTGATAGCC
6_known	G12	Tb927.10.15520_for	GGGGACAAGTTTGTACAAAAAAGCAGGCTATGAGTGATGTGAGTAAGTTCGTGAGG	Tb927.10.15520_rev	GGGGACCACTTTGTACAAGAAAGCTGGGTCTACGACGACGGGTCTGCT
6_known	G13	Tb927.3.3340_for	GGGGACAAGTTTGTACAAAAAAGCAGGCTATGGGCTGTGGAATCGCAAC	Tb927.3.3340_rev	GGGGACCACTTTGTACAAGAAAGCTGGGTCTACTCACACCATGCAGTGCG
6_known	G14	Tb927.10.11360_for	GGGGACAAGTTTGTACAAAAAAGCAGGCTATGTCCTCCAAATTAGGGACCGC	Tb927.10.11360_rev	GGGGACCACTTTGTACAAGAAAGCTGGGTTTACAGGCGGAGGCTGGG
6_known	G15	Tb927.10.14270_for	GGGGACAAGTTTGTACAAAAAAGCAGGCTATGCGACGTAGTGGATTCGTCG	Tb927.10.14270_rev	GGGGACCACTTTGTACAAGAAAGCTGGGTCTACTCGGGTGGTGAAGGTACG
6_known	G16	Tb927.11.14730_for	GGGGACAAGTTTGTACAAAAAAGCAGGCTATGCAGGGAGAACCGCGT	Tb927.11.14730_rev	GGGGACCACTTTGTACAAGAAAGCTGGGTCTAGCTGGTATCAGTGACTATTGGACG
6_known	G17	Tb927.8.590_for	GGGGACAAGTTTGTACAAAAAAGCAGGCTATGACACGGGTGTCCACGT	Tb927.8.590_rev	GGGGACCACTTTGTACAAGAAAGCTGGGTCTAAAGCTTCGACTGGAGTGGAAG
6_known	G18	Tb927.8.6970_for	GGGGACAAGTTTGTACAAAAAAGCAGGCTATGCTCCGATACAACGTGTTCTAC	Tb927.8.6970_rev	GGGGACCACTTTGTACAAGAAAGCTGGGTTTAAACTGCAGCAGAGGCAATATGG
6_known	G19	Tb927.4.200_for	GGGGACAAGTTTGTACAAAAAAGCAGGCTATGAATCAGAACGGCGGTAGTGT	Tb927.4.200_rev	GGGGACCACTTTGTACAAGAAAGCTGGGTCTATGCATTGTTACCACCTCTATTGTTAAC
6_known	G20	Tb927.8.5770_for	GGGGACAAGTTTGTACAAAAAAGCAGGCTATGCTACACGCTAGTGGTAATGGC	Tb927.8.5770_rev	GGGGACCACTTTGTACAAGAAAGCTGGGTTCACTGGCCTCGGGGCCG
6_known	G21	Tb927.1.1370_for	GGGGACAAGTTTGTACAAAAAAGCAGGCTATGCAGTATCGTGAGTATCGCGT	Tb927.1.1370_rev	GGGGACCACTTTGTACAAGAAAGCTGGGTTCACTCTTCCGCCTCACCATC
6_known	G22	Tb927.10.12370_for	GGGGACAAGTTTGTACAAAAAAGCAGGCTATGGGTCTTCTAACCACTGGCG	Tb927.10.12370_rev	GGGGACCACTTTGTACAAGAAAGCTGGGTTCACCCTTCGCGTTGTCTTTTAC
6_known	G23	Tb927.10.1210_for	GGGGACAAGTTTGTACAAAAAAGCAGGCTATGGACGACGACGTGGGG	Tb927.10.1210_rev	GGGGACCACTTTGTACAAGAAAGCTGGGTTCACACTGACCCCAGCTCTTC
6_known	G24	Tb927.6.2690_for	GGGGACAAGTTTGTACAAAAAAGCAGGCTATGTCGAAAACAATTTGCAAATGTTGTG	Tb927.6.2690_rev	GGGGACCACTTTGTACAAGAAAGCTGGGTTTAATGGTAAAGCTGGTCGTAGAAGAGC
6_known	H1	Tb927.9.15470_for	GGGGACAAGTTTGTACAAAAAAGCAGGCTATGGCGGATATGAAGAAGGTAACAGT	Tb927.9.15470_rev	GGGGACCACTTTGTACAAGAAAGCTGGGTTCATGCGGTTCCGACATGTTC
6_known	H2	Tb927.11.11410_for	GGGGACAAGTTTGTACAAAAAAGCAGGCTATGAGAATGCAAATACTCATTTTACTGTTA	Tb927.11.11410_rev	GGGGACCACTTTGTACAAGAAAGCTGGGTTCAAAAAGTGACAACAAAGATCATAGCAG
6_known	H3	Tb927.7.580_for	GGGGACAAGTTTGTACAAAAAAGCAGGCTATGGCACCATTTAGCGGGAAAC	Tb927.7.580_rev	GGGGACCACTTTGTACAAGAAAGCTGGGTTTACACTGAACTGGCCAATGGC
6_known	H4	Tb927.1.2110_for	GGGGACAAGTTTGTACAAAAAAGCAGGCTATGAGCTCGCGTAACGGC	Tb927.1.2110_rev	GGGGACCACTTTGTACAAGAAAGCTGGGTTTAGGACAGCTCAATAGAGTTACCAAGA
6_known	H5	Tb927.4.4050_for	GGGGACAAGTTTGTACAAAAAAGCAGGCTATGACAGTGTTTGAGCTATGCAAGG	Tb927.4.4050_rev	GGGGACCACTTTGTACAAGAAAGCTGGGTTCACTGTTTCCCGTCAGATACATCA
6_known	H6	Tb927.11.6740_for	GGGGACAAGTTTGTACAAAAAAGCAGGCTATGGGCAAGAAGGGAACGCT	Tb927.11.6740_rev	GGGGACCACTTTGTACAAGAAAGCTGGGTTCATGTGGATTCACTTATCTGCACAA
6_known	H7	Tb927.5.1650_for	GGGGACAAGTTTGTACAAAAAAGCAGGCTATGAGCGGCAGTGCAACTAC	Tb927.5.1650_rev	GGGGACCACTTTGTACAAGAAAGCTGGGTTCATCGCTTCATCGGTTCTAAAAAGG
6_known	H8	Tb927.11.1070_for	GGGGACAAGTTTGTACAAAAAAGCAGGCTATGACAACGCATTGGGTTCGT	Tb927.11.1070_rev	GGGGACCACTTTGTACAAGAAAGCTGGGTTTAGAAACCCCTGTGCAAATCAACT
6_known	H9	Tb927.10.10880_for	GGGGACAAGTTTGTACAAAAAAGCAGGCTATGTCTTCGCTTAGTACTGCTGGA	Tb927.10.10880_rev	GGGGACCACTTTGTACAAGAAAGCTGGGTTTAACCATCGTTAGCCTTCTCCTCC
6_known	H10	Tb927.11.2710_for	GGGGACAAGTTTGTACAAAAAAGCAGGCTATGGCTGATACACTTTCACCGTG	Tb927.11.2710_rev	GGGGACCACTTTGTACAAGAAAGCTGGGTTCAGCGCCCGCCAGCGAG
6_known	H11	Tb927.10.7240_for	GGGGACAAGTTTGTACAAAAAAGCAGGCTATGGAAGAAGTCAATGCGTATTCACC	Tb927.10.7240_rev	GGGGACCACTTTGTACAAGAAAGCTGGGTCTAACGAGGAAATCGGAGCGC
6_known	H12	Tb927.1.70_for	GGGGACAAGTTTGTACAAAAAAGCAGGCTATGAATCAGAATGGTGGTTGTGTGG	Tb927.1.70_rev	GGGGACCACTTTGTACAAGAAAGCTGGGTTCACCTTCGCTTTGCTTTCATCG
6_known	H13	Tb927.7.190_for	GGGGACAAGTTTGTACAAAAAAGCAGGCTATGACGTCGAGACTGTTTGCG	Tb927.7.190_rev	GGGGACCACTTTGTACAAGAAAGCTGGGTTTATACACCAAGGCTTTTGAAAAAAGCG
6_known	H14	Tb927.2.2190_for	GGGGACAAGTTTGTACAAAAAAGCAGGCTATGGGCAAAGAAACGCGTGA	Tb927.2.2190_rev	GGGGACCACTTTGTACAAGAAAGCTGGGTTTACTTGCACATATGGTCAAAAGAATTGG
6_known	H15	Tb927.11.9920_for	GGGGACAAGTTTGTACAAAAAAGCAGGCTATGCAGATCTTCGTGAAAACTCTTACC	Tb927.11.9920_rev	GGGGACCACTTTGTACAAGAAAGCTGGGTCTACCAACCACCTCGAAGACGA
6_known	H16	Tb927.7.1970_for	GGGGACAAGTTTGTACAAAAAAGCAGGCTATGATTCAAAACAACGGCAATATGAGAAA	Tb927.7.1970_rev	GGGGACCACTTTGTACAAGAAAGCTGGGTTCATTTCCGTGTGAATGCTGGTG
6_known	H17	Tb927.4.1920_for	GGGGACAAGTTTGTACAAAAAAGCAGGCTATGAAGCGAGTGCCGTGC	Tb927.4.1920_rev	GGGGACCACTTTGTACAAGAAAGCTGGGTTTAGCCAGTGCCAGCAGCG
6_known	H18	Tb927.9.12880_for	GGGGACAAGTTTGTACAAAAAAGCAGGCTATGGATGACGGAACTGGACACC	Tb927.9.12880_rev	GGGGACCACTTTGTACAAGAAAGCTGGGTTCATATGGGACCCATAGCCCTTG
6_known	H19	Tb927.7.3780_for	GGGGACAAGTTTGTACAAAAAAGCAGGCTATGTCACGAACAGACAAGTCGTTC	Tb927.7.3780_rev	GGGGACCACTTTGTACAAGAAAGCTGGGTTCAAAAACCGAGTCCCAGCG
6_known	H21	Tb927.7.2030_for	GGGGACAAGTTTGTACAAAAAAGCAGGCTATGATTCAAAACAACGGCAATATGGG	Tb927.7.2030_rev	GGGGACCACTTTGTACAAGAAAGCTGGGTTCATTTCCGTGTGAATGCTGGTG
6_known	H22	Tb927.10.7080_for	GGGGACAAGTTTGTACAAAAAAGCAGGCTATGAGGCGCTTCGCGAGG	Tb927.10.7080_rev	GGGGACCACTTTGTACAAGAAAGCTGGGTTCAAAGAGGCCTAAAGTTCTTGTTCAG
6_known	H23	Tb927.7.6970_for	GGGGACAAGTTTGTACAAAAAAGCAGGCTATGACCAAAAAGAGTGGTCATGAGG	Tb927.7.6970_rev	GGGGACCACTTTGTACAAGAAAGCTGGGTCTATGAAGACGTTTTAGGCGCCA
6_known	H24	Tb927.10.6090_for	GGGGACAAGTTTGTACAAAAAAGCAGGCTATGCGCCGTGTCAGTCTGC	Tb927.10.6090_rev	GGGGACCACTTTGTACAAGAAAGCTGGGTTTAAACAGCGGCCGTGATGTC
6_known	I1	Tb927.3.5490_for	GGGGACAAGTTTGTACAAAAAAGCAGGCTATGACATCCCGACCCTTGACG	Tb927.3.5490_rev	GGGGACCACTTTGTACAAGAAAGCTGGGTTTACTCACGCATATGCAAATATAAATGCTT
6_known	I2	Tb927.10.9130_for	GGGGACAAGTTTGTACAAAAAAGCAGGCTATGTTGAATGAACGGCGGCC	Tb927.10.9130_rev	GGGGACCACTTTGTACAAGAAAGCTGGGTTTACAACATCATACGTGAGAAAGACGAT
6_known	I3	Tb927.10.6230_for	GGGGACAAGTTTGTACAAAAAAGCAGGCTATGTTAACTGTAACGGAGGATAAGATCTCC	Tb927.10.6230_rev	GGGGACCACTTTGTACAAGAAAGCTGGGTTTAGGCAGCGTAAATACCGTTTTTGA
6_known	I4	Tb927.1.1550_for	GGGGACAAGTTTGTACAAAAAAGCAGGCTATGACAGACGGCGTTACATTACATG	Tb927.1.1550_rev	GGGGACCACTTTGTACAAGAAAGCTGGGTTTAAGAAGCCCACGATGGGGG
6_known	I5	Tb927.7.1950_for	GGGGACAAGTTTGTACAAAAAAGCAGGCTATGATTCAAAACAACGGCAATATGAGAAA	Tb927.7.1950_rev	GGGGACCACTTTGTACAAGAAAGCTGGGTCTACTCATGACCTCGTTTCCGTG
6_known	I6	Tb927.11.4660_for	GGGGACAAGTTTGTACAAAAAAGCAGGCTATGACCTCACTCTGTCCAATTACGA	Tb927.11.4660_rev	GGGGACCACTTTGTACAAGAAAGCTGGGTTCACACGCTTTCAAGTTCATGAAGC
6_known	I7	Tb927.9.13250_for	GGGGACAAGTTTGTACAAAAAAGCAGGCTATGTTTCGTCGGTTCTGCACC	Tb927.9.13250_rev	GGGGACCACTTTGTACAAGAAAGCTGGGTTCAGTACACGTCCTTCAAGTATCTACC
6_known	I8	Tb927.7.4090_for	GGGGACAAGTTTGTACAAAAAAGCAGGCTATGCAACAACAACAGCAGCAGC	Tb927.7.4090_rev	GGGGACCACTTTGTACAAGAAAGCTGGGTCTAAGAGGAACCAACGCCCAAAT
6_known	I11	Tb927.11.7010_for	GGGGACAAGTTTGTACAAAAAAGCAGGCTATGACTCAGGAGGAATGCACGG	Tb927.11.7010_rev	GGGGACCACTTTGTACAAGAAAGCTGGGTTCACTCCTCGTCTCCTTTACAAGG
6_known	I12	Tb927.9.3650_for	GGGGACAAGTTTGTACAAAAAAGCAGGCTATGGCGAAGTGGGAATTAAAGCTG	Tb927.9.3650_rev	GGGGACCACTTTGTACAAGAAAGCTGGGTCTAAATCCCGTTTTGCTCGAGACG
6_known	I13	Tb927.7.2000_for	GGGGACAAGTTTGTACAAAAAAGCAGGCTATGATTCAAAACAACGGCAATATGGG	Tb927.7.2000_rev	GGGGACCACTTTGTACAAGAAAGCTGGGTCTACTCATGACCTCGTTTCCGTG
6_known	I14	Tb927.4.1700_for	GGGGACAAGTTTGTACAAAAAAGCAGGCTATGAACAGAACGCTACTCGGTTTAG	Tb927.4.1700_rev	GGGGACCACTTTGTACAAGAAAGCTGGGTTCACATGCAATGGCATGTACAGG
6_known	I15	Tb927.11.1990_for	GGGGACAAGTTTGTACAAAAAAGCAGGCTATGGCGAAATATATCGCAGGGC	Tb927.11.1990_rev	GGGGACCACTTTGTACAAGAAAGCTGGGTTCAAATCTTCTCGGCGACGTC
6_known	I16	Tb927.7.1000_for	GGGGACAAGTTTGTACAAAAAAGCAGGCTATGACACGTTGCCGGCTC	Tb927.7.1000_rev	GGGGACCACTTTGTACAAGAAAGCTGGGTTCAAGCTTCTCCACCGTTGC
6_known	I17	Tb927.7.1980_for	GGGGACAAGTTTGTACAAAAAAGCAGGCTATGATTCAAAACAACGGCAATATGGG	Tb927.7.1980_rev	GGGGACCACTTTGTACAAGAAAGCTGGGTCTACTCATGACCTCGTTTCCGTG
6_known	I18	Tb927.1.1730_for	GGGGACAAGTTTGTACAAAAAAGCAGGCTATGTTGCGCTACACCAGCTG	Tb927.1.1730_rev	GGGGACCACTTTGTACAAGAAAGCTGGGTTTAAGCTGGAGCTGCTACTTTATCTTTAG
6_known	I19	Tb927.10.8040_for	GGGGACAAGTTTGTACAAAAAAGCAGGCTATGGAAGCGGTTCTTCGCAAG	Tb927.10.8040_rev	GGGGACCACTTTGTACAAGAAAGCTGGGTTCATGAAAACAAATCATCCAGAGTTGACG
6_known	I20	Tb927.4.1330_for	GGGGACAAGTTTGTACAAAAAAGCAGGCTATGGGTAAGGCACAGAAGCCG	Tb927.4.1330_rev	GGGGACCACTTTGTACAAGAAAGCTGGGTTTATTTTGCTATGATGTTGACAAGCGG
6_known	I21	Tb927.10.2700_for	GGGGACAAGTTTGTACAAAAAAGCAGGCTATGCTTGCCAAGAAGAGGGC	Tb927.10.2700_rev	GGGGACCACTTTGTACAAGAAAGCTGGGTTTACTTATCCTCTTCGCCAGCAGT
6_known	I22	Tb927.10.13680_for	GGGGACAAGTTTGTACAAAAAAGCAGGCTATGCCGGCGTTGCAATGG	Tb927.10.13680_rev	GGGGACCACTTTGTACAAGAAAGCTGGGTCTACACAACGCCCATGCTACC
6_known	I23	Tb927.5.2570_for	GGGGACAAGTTTGTACAAAAAAGCAGGCTATGGAGGACGATGGCTTGATGT	Tb927.5.2570_rev	GGGGACCACTTTGTACAAGAAAGCTGGGTCTAATTTAGCGGGCGCCGG
6_known	I24	Tb927.7.3620_for	GGGGACAAGTTTGTACAAAAAAGCAGGCTATGACAACCAACACCGCCG	Tb927.7.3620_rev	GGGGACCACTTTGTACAAGAAAGCTGGGTTTACTTACTCTTAGAAAGCGACTTCTCCG
6_known	J1	Tb927.11.10560_for	GGGGACAAGTTTGTACAAAAAAGCAGGCTATGCTGTTCAAACCTCGTGGTG	Tb927.11.10560_rev	GGGGACCACTTTGTACAAGAAAGCTGGGTTCACGCATATATAACTGAGAGTTCTTCCA
6_known	J2	Tb927.11.11860_for	GGGGACAAGTTTGTACAAAAAAGCAGGCTATGAGAGAAAAGGAAGCACATAAGGG	Tb927.11.11860_rev	GGGGACCACTTTGTACAAGAAAGCTGGGTTCAAAGTTCCGGGCTGATACGA
6_known	J3	Tb927.11.6160_for	GGGGACAAGTTTGTACAAAAAAGCAGGCTATGTCAGGTTGGGCACAACCA	Tb927.11.6160_rev	GGGGACCACTTTGTACAAGAAAGCTGGGTTTAAGCTTTACCTGCGTTCGCC
6_known	J4	Tb927.10.3100_for	GGGGACAAGTTTGTACAAAAAAGCAGGCTATGGAGGGAAAGAGCGTAGTAGG	Tb927.10.3100_rev	GGGGACCACTTTGTACAAGAAAGCTGGGTTCAATTTTTGATATCCGCAAGCCGG
6_known	J5	Tb927.10.8020_for	GGGGACAAGTTTGTACAAAAAAGCAGGCTATGCGCCTCGCTTACCCC	Tb927.10.8020_rev	GGGGACCACTTTGTACAAGAAAGCTGGGTTCAGTCTGTCCACTGGGCG
6_known	J6	Tb927.9.4190_for	GGGGACAAGTTTGTACAAAAAAGCAGGCTATGGGAATGTACGTCACTAAGCTTCT	Tb927.9.4190_rev	GGGGACCACTTTGTACAAGAAAGCTGGGTTTACTCATACTTGAAAAGTTGTTCAATGAC
6_known	J7	Tb927.6.4780_for	GGGGACAAGTTTGTACAAAAAAGCAGGCTATGGTAAGTGGACCTACAGCTACTG	Tb927.6.4780_rev	GGGGACCACTTTGTACAAGAAAGCTGGGTTTACTCCATATCGCCCACACCC
6_known	J8	Tb927.11.11490_for	GGGGACAAGTTTGTACAAAAAAGCAGGCTATGAGGAATGCGATGGCGGA	Tb927.11.11490_rev	GGGGACCACTTTGTACAAGAAAGCTGGGTTTACTGGCGGTAGTGATACCAATGG
6_known	J9	Tb927.10.3770_for	GGGGACAAGTTTGTACAAAAAAGCAGGCTATGATGAACACCGCCACTTTTTCT	Tb927.10.3770_rev	GGGGACCACTTTGTACAAGAAAGCTGGGTTCACCTTCCATCGCCTTTATTATCAC
6_known	J10	Tb927.9.10960_for	GGGGACAAGTTTGTACAAAAAAGCAGGCTATGAGCGGTGTCAGCAGCA	Tb927.9.10960_rev	GGGGACCACTTTGTACAAGAAAGCTGGGTTCATTTCACTGCCCGGCG
6_known	J11	Tb927.8.1020_for	GGGGACAAGTTTGTACAAAAAAGCAGGCTATGACGGAGGCTCCTAAGCC	Tb927.8.1020_rev	GGGGACCACTTTGTACAAGAAAGCTGGGTTCATTCCACCGGTGCAGCT
6_known	J12	Tb927.9.4210_for	GGGGACAAGTTTGTACAAAAAAGCAGGCTATGGGGGGATGTGTGATTTCCG	Tb927.9.4210_rev	GGGGACCACTTTGTACAAGAAAGCTGGGTTCAGCACTCCTCCACGAAAAGT
6_known	J14	Tb927.2.5280_for	GGGGACAAGTTTGTACAAAAAAGCAGGCTATGCGCGTTGTATACCAGCG	Tb927.2.5280_rev	GGGGACCACTTTGTACAAGAAAGCTGGGTTTACATAAATAGGGAATATAAGTGCAGAAC
6_known	J15	Tb927.10.3260_for	GGGGACAAGTTTGTACAAAAAAGCAGGCTATGGGTGGCTGCGTTGCA	Tb927.10.3260_rev	GGGGACCACTTTGTACAAGAAAGCTGGGTTCAAACATCATTGCGGAATATCTCTTCG
6_known	J16	Tb927.4.1630_for	GGGGACAAGTTTGTACAAAAAAGCAGGCTATGTCTGGCTCTCTTCCACAAACA	Tb927.4.1630_rev	GGGGACCACTTTGTACAAGAAAGCTGGGTTCACGATTTGTTTTTCTTTCCGAGC
6_known	J17	Tb927.11.16110_for	GGGGACAAGTTTGTACAAAAAAGCAGGCTATGCGACTTCGTGATGTTTTCATTG	Tb927.11.16110_rev	GGGGACCACTTTGTACAAGAAAGCTGGGTTCAGTCCGTTGCACGGCC
6_known	J18	Tb927.3.3160_for	GGGGACAAGTTTGTACAAAAAAGCAGGCTATGGAGTGGATGTATGGCCCAA	Tb927.3.3160_rev	GGGGACCACTTTGTACAAGAAAGCTGGGTTTACTTCAGGTCTTGTAACAGGTGC
6_known	J19	Tb927.10.15610_for	GGGGACAAGTTTGTACAAAAAAGCAGGCTATGGACCCCTCGAGAGCG	Tb927.10.15610_rev	GGGGACCACTTTGTACAAGAAAGCTGGGTTTACGGTTTGAGGCATAACCGC
6_known	J20	Tb927.10.10890_for	GGGGACAAGTTTGTACAAAAAAGCAGGCTATGACGGAGACATTCGCATTCC	Tb927.10.10890_rev	GGGGACCACTTTGTACAAGAAAGCTGGGTTCAGTCCACTTCCTCCATACCCG
6_known	K6	Tb927.11.14960_for	GGGGACAAGTTTGTACAAAAAAGCAGGCTATGCCAAAAATGCGTTTAGACTTCTTAAA	Tb927.11.14960_rev	GGGGACCACTTTGTACAAGAAAGCTGGGTTCATTCGGCCGTTTTGAAAGGC
6_known	K7	Tb927.10.4910_for	GGGGACAAGTTTGTACAAAAAAGCAGGCTATGCTGGCCAAGGACGTTG	Tb927.10.4910_rev	GGGGACCACTTTGTACAAGAAAGCTGGGTTTACCTTGTAAAGAAAATGTCGTGCAAAA

6_known	K8	Tb927.11.16210_for	GGGGACAAGTTTGTACAAAAAAGCAGGCTATGGGTAGTTATGAATACCCAGACTACG	Tb927.11.16210_rev	GGGGACCACTTTGTACAAGAAAGCTGGGTTTACCTTTTCGCCATGAACTCTTTGC
6_known	K9	Tb927.4.2220_for	GGGGACAAGTTTGTACAAAAAAGCAGGCTATGGAGACGACATGCAAGGAGG	Tb927.4.2220_rev	GGGGACCACTTTGTACAAGAAAGCTGGGTTCACCAAAATCCACTACTGCCTTG
6_known	K10	Tb927.3.580_for	GGGGACAAGTTTGTACAAAAAAGCAGGCTATGGTGAAGATTTCCTGGGTGACG	Tb927.3.580_rev	GGGGACCACTTTGTACAAGAAAGCTGGGTCTACATATATTCATCATCCATCCTTTTAAC
6_known	K11	Tb927.7.2680_for	GGGGACAAGTTTGTACAAAAAAGCAGGCTATGTTCCTTCCCGAACAATCGAAC	Tb927.7.2680_rev	GGGGACCACTTTGTACAAGAAAGCTGGGTTCAAAAGGGTACGTGTAGGGGAA
6_known	K12	Tb927.9.4200_for	GGGGACAAGTTTGTACAAAAAAGCAGGCTATGAACACCGCTTCTGCGC	Tb927.9.4200_rev	GGGGACCACTTTGTACAAGAAAGCTGGGTTCAGCACTCCTCCACGAAAAGT
6_known	K13	Tb927.10.7490_for	GGGGACAAGTTTGTACAAAAAAGCAGGCTATGTTGGACGCTGCGCTG	Tb927.10.7490_rev	GGGGACCACTTTGTACAAGAAAGCTGGGTTCAAGCTTCGTAGACGTAGATGTCG
6_known	K14	Tb927.10.8930_for	GGGGACAAGTTTGTACAAAAAAGCAGGCTATGGACTGTGTGTATAACTCAGTGGG	Tb927.10.8930_rev	GGGGACCACTTTGTACAAGAAAGCTGGGTTTACTGGCGAGCTAATGCAAGC
6_known	K15	Tb927.10.5670_for	GGGGACAAGTTTGTACAAAAAAGCAGGCTATGGCTACAACCTTGCCTTTACAG	Tb927.10.5670_rev	GGGGACCACTTTGTACAAGAAAGCTGGGTTTAATTCAGTTTTTCCAAAGCGGTAGTAAG
6_known	K16	Tb927.10.1950_for	GGGGACAAGTTTGTACAAAAAAGCAGGCTATGCGCCGAGTTTTTAGGAAAAGT	Tb927.10.1950_rev	GGGGACCACTTTGTACAAGAAAGCTGGGTTCAAACGTGGACAGTTATTCCCTTAC
6_known	K17	Tb927.10.10410_for	GGGGACAAGTTTGTACAAAAAAGCAGGCTATGTCGATGAATGGCGTTCCAC	Tb927.10.10410_rev	GGGGACCACTTTGTACAAGAAAGCTGGGTCTAAACACCGCGCAGTTGGT
6_known	K18	Tb927.11.8950_for	GGGGACAAGTTTGTACAAAAAAGCAGGCTATGCATTCGAAAGCATCCTCTGTT	Tb927.11.8950_rev	GGGGACCACTTTGTACAAGAAAGCTGGGTTCAATATATGGATTGGACGGAACCTTTAC
6_known	K19	Tb927.11.5970_for	GGGGACAAGTTTGTACAAAAAAGCAGGCTATGGGAGGGTGTACGTCACG	Tb927.11.5970_rev	GGGGACCACTTTGTACAAGAAAGCTGGGTCTACTGATAAGATACTTGAACCATTAAAAA
6_known	K20	Tb927.11.12850_for	GGGGACAAGTTTGTACAAAAAAGCAGGCTATGCAAACTGAACGTGGTCCAA	Tb927.11.12850_rev	GGGGACCACTTTGTACAAGAAAGCTGGGTCTACTTCCGCAGCAGCTGC
6_known	K21	Tb927.2.5030_for	GGGGACAAGTTTGTACAAAAAAGCAGGCTATGTCCGACGAACTAGGTGTAAAGG	Tb927.2.5030_rev	GGGGACCACTTTGTACAAGAAAGCTGGGTTTACTCTATGTCCATCTCGTACCACTC
6_known	K22	Tb927.1.3230_for	GGGGACAAGTTTGTACAAAAAAGCAGGCTATGGCATACAGTGGTGCAGGG	Tb927.1.3230_rev	GGGGACCACTTTGTACAAGAAAGCTGGGTCTACAGTGGAAGGACCTTTGTAAAACA
6_known	K23	Tb927.10.12890_for	GGGGACAAGTTTGTACAAAAAAGCAGGCTATGTCAGCAAGTAATTGTCGAGGC	Tb927.10.12890_rev	GGGGACCACTTTGTACAAGAAAGCTGGGTTCAATAGCTTCTCCCAAATAAAACCCAC
6_known	K24	Tb927.7.7240_for	GGGGACAAGTTTGTACAAAAAAGCAGGCTATGGGTATTGGACAGAGCCGT	Tb927.7.7240_rev	GGGGACCACTTTGTACAAGAAAGCTGGGTTTACTCCTTGAGGCCCCGG
6_known	L1	Tb927.7.2470_for	GGGGACAAGTTTGTACAAAAAAGCAGGCTATGGATGTTGGTAGTAAAGTCTTCGTG	Tb927.7.2470_rev	GGGGACCACTTTGTACAAGAAAGCTGGGTTCACGTTGTGTTGCCTTCCAA
6_known	L2	Tb927.11.15490_for	GGGGACAAGTTTGTACAAAAAAGCAGGCTATGTGGAACTTTTTAATAGACATAAAGAAC	Tb927.11.15490_rev	GGGGACCACTTTGTACAAGAAAGCTGGGTCTACCAGTCATCCCACTCCTCC
6_known	L3	Tb927.11.16930_for	GGGGACAAGTTTGTACAAAAAAGCAGGCTATGCGGCGCGCTGCGTTC	Tb927.11.16930_rev	GGGGACCACTTTGTACAAGAAAGCTGGGTCTACTCCTCAGCACGATGTGC
6_known	L4	Tb927.11.3280_for	GGGGACAAGTTTGTACAAAAAAGCAGGCTATGGAGCGACAGCTTCGCG	Tb927.11.3280_rev	GGGGACCACTTTGTACAAGAAAGCTGGGTTTACCGGCGTTGCCGAGA
6_known	L5	Tb927.1.1010_for	GGGGACAAGTTTGTACAAAAAAGCAGGCTATGGACGGCAGTGAGTGCT	Tb927.1.1010_rev	GGGGACCACTTTGTACAAGAAAGCTGGGTCTATCCTTCCAGAAGAAGCTTTACCG
6_known	L6	Tb927.4.3300_for	GGGGACAAGTTTGTACAAAAAAGCAGGCTATGTTTAGGATACCCCGATCAGCC	Tb927.4.3300_rev	GGGGACCACTTTGTACAAGAAAGCTGGGTTTAGCTCTCCAGATAAGTGCGGAA
6_known	L7	Tb927.5.1150_for	GGGGACAAGTTTGTACAAAAAAGCAGGCTATGGGGTGCGGAGAGAAAAATC	Tb927.5.1150_rev	GGGGACCACTTTGTACAAGAAAGCTGGGTCTAAGACGAATCATCATCATCATCATCATC
6_known	L8	Tb927.6.4970_for	GGGGACAAGTTTGTACAAAAAAGCAGGCTATGCCCCGACCCGACAGA	Tb927.6.4970_rev	GGGGACCACTTTGTACAAGAAAGCTGGGTTTAGTCATTAGTGCAGCTGCTGTC
6_known	L9	Tb927.10.3170_for	GGGGACAAGTTTGTACAAAAAAGCAGGCTATGGATCTTGCACAGGCAAAGAC	Tb927.10.3170_rev	GGGGACCACTTTGTACAAGAAAGCTGGGTTCAGCGCATCTGCTTCATAACAA
6_known	L10	Tb927.7.5080_for	GGGGACAAGTTTGTACAAAAAAGCAGGCTATGCTTCGGCACGCGTTG	Tb927.7.5080_rev	GGGGACCACTTTGTACAAGAAAGCTGGGTTCACATGTTCGCGGGGAACT
6_known	L11	Tb927.10.7690_for	GGGGACAAGTTTGTACAAAAAAGCAGGCTATGACGGACCGGACCATTTATGA	Tb927.10.7690_rev	GGGGACCACTTTGTACAAGAAAGCTGGGTTTAAAACATAATGAGCCGTTTTATTTCTTT
6_known	L12	Tb927.7.7170_for	GGGGACAAGTTTGTACAAAAAAGCAGGCTATGAATCCACACGCCTGTGGT	Tb927.7.7170_rev	GGGGACCACTTTGTACAAGAAAGCTGGGTTTATCGGTAGTCCCTGTAGTGATCAG
6_known	L13	Tb927.11.3890_for	GGGGACAAGTTTGTACAAAAAAGCAGGCTATGCGCTCCGGTTTGTTCG	Tb927.11.3890_rev	GGGGACCACTTTGTACAAGAAAGCTGGGTTCATGGAGCATCGACAGCGG
6_known	L14	Tb927.1.1560_for	GGGGACAAGTTTGTACAAAAAAGCAGGCTATGGGGAAAAGTTTTGTCGTACGG	Tb927.1.1560_rev	GGGGACCACTTTGTACAAGAAAGCTGGGTCTACTCTGACCTTCCCGCAATGG
6_known	L15	Tb927.11.15460_for	GGGGACAAGTTTGTACAAAAAAGCAGGCTATGGGCATCAAGGGTCGTAGG	Tb927.11.15460_rev	GGGGACCACTTTGTACAAGAAAGCTGGGTTTAAGACTTCGGAAAAGCCATTCCAA
6_known	L16	Tb927.9.6560_for	GGGGACAAGTTTGTACAAAAAAGCAGGCTATGGGCAGTGATAGTAAATCTAGTAAGTCC	Tb927.9.6560_rev	GGGGACCACTTTGTACAAGAAAGCTGGGTTTACTTGAAGAGACTGGCGAAGGG
6_known	L17	Tb927.7.5540_for	GGGGACAAGTTTGTACAAAAAAGCAGGCTATGCCGCTAGGGATTGTTGC	Tb927.7.5540_rev	GGGGACCACTTTGTACAAGAAAGCTGGGTTCAAATCTTGTAACCGATTTCATACCCC
6_known	L18	Tb927.5.2060_for	GGGGACAAGTTTGTACAAAAAAGCAGGCTATGACGCGCCTACACTTCAAG	Tb927.5.2060_rev	GGGGACCACTTTGTACAAGAAAGCTGGGTTTAGCTGACCTGTTTGTTGCGG
6_known	L19	Tb927.11.7530_for	GGGGACAAGTTTGTACAAAAAAGCAGGCTATGTTTACTCTTGTCGGGCGC	Tb927.11.7530_rev	GGGGACCACTTTGTACAAGAAAGCTGGGTTTAAACAAGGTTAAACATGGATCTGAGCG
6_known	L20	Tb927.2.5020_for	GGGGACAAGTTTGTACAAAAAAGCAGGCTATGATGTGCAAGGGTCTATTTTCAAAATTG	Tb927.2.5020_rev	GGGGACCACTTTGTACAAGAAAGCTGGGTTCATTTCTCTTGTGCAAAGGACTGT
6_known	L21	Tb927.11.11160_for	GGGGACAAGTTTGTACAAAAAAGCAGGCTATGAAGTTCGGAAAGCGGCTT	Tb927.11.11160_rev	GGGGACCACTTTGTACAAGAAAGCTGGGTCTACGGTTTGCCTTCAAACACAAC
6_known	L22	Tb927.3.4570_for	GGGGACAAGTTTGTACAAAAAAGCAGGCTATGATGGTAACCAACACATGCATAATAATG	Tb927.3.4570_rev	GGGGACCACTTTGTACAAGAAAGCTGGGTTTACGCAACGGCCGTCTTTG
6_known	L23	Tb927.10.5310_for	GGGGACAAGTTTGTACAAAAAAGCAGGCTATGAGTCAGCGGTTCGGACC	Tb927.10.5310_rev	GGGGACCACTTTGTACAAGAAAGCTGGGTTCAAGTGCATACGGTGAACTTACC
6_known	L24	Tb927.10.11450_for	GGGGACAAGTTTGTACAAAAAAGCAGGCTATGAATATTTTTTCCTTTGGGAGGGAAAGC	Tb927.10.11450_rev	GGGGACCACTTTGTACAAGAAAGCTGGGTTCAGGAACACGACACTTCCCG
6_known	M1	Tb927.3.4840_for	GGGGACAAGTTTGTACAAAAAAGCAGGCTATGCCGCCTCCCTTTTCTATAACT	Tb927.3.4840_rev	GGGGACCACTTTGTACAAGAAAGCTGGGTCTAATTAGAAACCCTTTTTCTCTTGTAAAA
6_known	M2	Tb927.11.16140_for	GGGGACAAGTTTGTACAAAAAAGCAGGCTATGGCGGACGGGCCGACG	Tb927.11.16140_rev	GGGGACCACTTTGTACAAGAAAGCTGGGTTCACTCCAGTGAAAAGTGAACAGTG
6_known	M3	Tb927.10.2520_for	GGGGACAAGTTTGTACAAAAAAGCAGGCTATGGGTCCGAAAACTCAGGAGG	Tb927.10.2520_rev	GGGGACCACTTTGTACAAGAAAGCTGGGTTCACCACTGTTGGACGATATCTTCA
6_known	M4	Tb927.1.2120_for	GGGGACAAGTTTGTACAAAAAAGCAGGCTATGGGGTGTGGTGCGTCT	Tb927.1.2120_rev	GGGGACCACTTTGTACAAGAAAGCTGGGTTTACTTTTTTTTATTTTCCACATACCAGTC
6_known	M5	Tb927.6.640_for	GGGGACAAGTTTGTACAAAAAAGCAGGCTATGACTTCAAGCACTTTACTGGAAAAGT	Tb927.6.640_rev	GGGGACCACTTTGTACAAGAAAGCTGGGTTTAGGCATTTTGGGGACTCACGG
6_known	M6	Tb927.6.4770_for	GGGGACAAGTTTGTACAAAAAAGCAGGCTATGTACCCCCGACACGATGAT	Tb927.6.4770_rev	GGGGACCACTTTGTACAAGAAAGCTGGGTTCAATAATATGTTTCTCGCACTCCCG
6_known	M7	Tb927.1.5030_for	GGGGACAAGTTTGTACAAAAAAGCAGGCTATGGGTGGTGTGGTCGGC	Tb927.1.5030_rev	GGGGACCACTTTGTACAAGAAAGCTGGGTTTACATTTGTTTAACTACAATACCGCGTTT
6_known	M8	Tb927.6.2900_for	GGGGACAAGTTTGTACAAAAAAGCAGGCTATGGAAGGGGAACTTGCCTTCG	Tb927.6.2900_rev	GGGGACCACTTTGTACAAGAAAGCTGGGTTTAGCGGCTGCGTTTATTTCCC
6_known	M9	Tb927.9.12510_for	GGGGACAAGTTTGTACAAAAAAGCAGGCTATGAGTTTATCCCGCACAGAAAACA	Tb927.9.12510_rev	GGGGACCACTTTGTACAAGAAAGCTGGGTTTAGAAGCCTCCGTCATCCACA
6_known	M10	Tb927.11.2230_for	GGGGACAAGTTTGTACAAAAAAGCAGGCTATGGCAACATCAGAAAGTTCAGGTG	Tb927.11.2230_rev	GGGGACCACTTTGTACAAGAAAGCTGGGTTTACAAATTTGGTGAGGCATCAGCA
6_known	M11	Tb927.1.1620_for	GGGGACAAGTTTGTACAAAAAAGCAGGCTATGCTAAGTCCCTGGAATGATTGTG	Tb927.1.1620_rev	GGGGACCACTTTGTACAAGAAAGCTGGGTTCAGCCACGTTCTCTCTTTGTTTG
6_known	M12	Tb927.8.8050_for	GGGGACAAGTTTGTACAAAAAAGCAGGCTATGCATCGAAAAAGCATCGTGTCG	Tb927.8.8050_rev	GGGGACCACTTTGTACAAGAAAGCTGGGTTCAGAACCGCTTCGGTGTCC
6_known	M13	Tb927.10.8290_for	GGGGACAAGTTTGTACAAAAAAGCAGGCTATGGCGGATTTCTTTAACGTAAGTGA	Tb927.10.8290_rev	GGGGACCACTTTGTACAAGAAAGCTGGGTTTACTGCCCTCCCCTAGCAC
6_known	M14	Tb927.5.760_for	GGGGACAAGTTTGTACAAAAAAGCAGGCTATGAGTGGAAGCGCTGATATTCTCG	Tb927.5.760_rev	GGGGACCACTTTGTACAAGAAAGCTGGGTCTAACTGCTGCTGCGCTTTGC
6_known	M15	Tb927.10.6680_for	GGGGACAAGTTTGTACAAAAAAGCAGGCTATGCAGCCACTACAGGCAAC	Tb927.10.6680_rev	GGGGACCACTTTGTACAAGAAAGCTGGGTTTAAGGAATGCGCTCCGCC
6_known	M16	Tb927.10.3900_for	GGGGACAAGTTTGTACAAAAAAGCAGGCTATGACCTCAACGACCGAATTCAC	Tb927.10.3900_rev	GGGGACCACTTTGTACAAGAAAGCTGGGTTTATATAAGTACGCTTTCGCCCCTGG
6_known	M17	Tb927.10.8270_for	GGGGACAAGTTTGTACAAAAAAGCAGGCTATGGCGGATTTCTTTAACGTAAGTGA	Tb927.10.8270_rev	GGGGACCACTTTGTACAAGAAAGCTGGGTTTACTGCCCTCCTCTAGCACG
6_known	M18	Tb927.6.1160_for	GGGGACAAGTTTGTACAAAAAAGCAGGCTATGTCTCGTATTGCGTCCGTTG	Tb927.6.1160_rev	GGGGACCACTTTGTACAAGAAAGCTGGGTTTAGTCTATGGTTTGTATGCCGTAATGG
6_known	M19	Tb927.8.1060_for	GGGGACAAGTTTGTACAAAAAAGCAGGCTATGTTGACCTTAGTCCGGCGC	Tb927.8.1060_rev	GGGGACCACTTTGTACAAGAAAGCTGGGTTTAGGATATGTTGAGGAGTCGAAGAACA
6_known	M20	Tb927.1.1340_for	GGGGACAAGTTTGTACAAAAAAGCAGGCTATGGCATGCAGTACCAAAAGGG	Tb927.1.1340_rev	GGGGACCACTTTGTACAAGAAAGCTGGGTCTACTCGCCCAGCTGCTGA
6_known	M21	Tb927.4.2390_for	GGGGACAAGTTTGTACAAAAAAGCAGGCTATGGAAGGTACAAATAGCGGTAATGAAG	Tb927.4.2390_rev	GGGGACCACTTTGTACAAGAAAGCTGGGTTTATCCCTTTTGTGTTGTGCCCC
6_known	M22	Tb927.8.5970_for	GGGGACAAGTTTGTACAAAAAAGCAGGCTATGCTTGAGGAAGACCTTGAGGC	Tb927.8.5970_rev	GGGGACCACTTTGTACAAGAAAGCTGGGTTCAGGCGGCATACCTGTACA
6_known	M23	Tb927.9.7210_for	GGGGACAAGTTTGTACAAAAAAGCAGGCTATGACCCCTCGGCGCGTC	Tb927.9.7210_rev	GGGGACCACTTTGTACAAGAAAGCTGGGTTCACGAGTCCAGAGATGGAAGAAG
6_known	M24	Tb927.1.4180_for	GGGGACAAGTTTGTACAAAAAAGCAGGCTATGGAGACATCGATCGGCTTAACG	Tb927.1.4180_rev	GGGGACCACTTTGTACAAGAAAGCTGGGTCTATTTCTTCTTGCTTCCCTTGCCT
6_known	N1	Tb927.10.7740_for	GGGGACAAGTTTGTACAAAAAAGCAGGCTATGGACCAATACGGAATACCACAGC	Tb927.10.7740_rev	GGGGACCACTTTGTACAAGAAAGCTGGGTCTACTGGGAAACTGCGAGCC
6_known	N2	Tb927.9.2220_for	GGGGACAAGTTTGTACAAAAAAGCAGGCTATGGCAGATATCCTTTTAAATGCCGC	Tb927.9.2220_rev	GGGGACCACTTTGTACAAGAAAGCTGGGTTCACGCCTTCGTGTATAAGGCC
6_known	N3	Tb927.3.5030_for	GGGGACAAGTTTGTACAAAAAAGCAGGCTATGGCGCACTATGATGAGTGGC	Tb927.3.5030_rev	GGGGACCACTTTGTACAAGAAAGCTGGGTCTAAATGTCGCCTTGCAATTGAGATATC
6_known	N4	Tb927.8.3830_for	GGGGACAAGTTTGTACAAAAAAGCAGGCTATGCGACACTGGACATTTCACTG	Tb927.8.3830_rev	GGGGACCACTTTGTACAAGAAAGCTGGGTTTATTTACGGGTCGCTGGTCGC
6_known	N5	Tb927.6.2020_for	GGGGACAAGTTTGTACAAAAAAGCAGGCTATGAGTCTTCAGACAAACTGCTACTCC	Tb927.6.2020_rev	GGGGACCACTTTGTACAAGAAAGCTGGGTTCACTTTTCGCCAGTACGAATGAG
6_known	N6	Tb927.8.1090_for	GGGGACAAGTTTGTACAAAAAAGCAGGCTATGAAAGATCGGTCGGCCGC	Tb927.8.1090_rev	GGGGACCACTTTGTACAAGAAAGCTGGGTTTAACGTCGGGTCAATGACCGA
6_known	N7	Tb927.9.11760_for	GGGGACAAGTTTGTACAAAAAAGCAGGCTATGGGTGTCCATGGACTGTGG	Tb927.9.11760_rev	GGGGACCACTTTGTACAAGAAAGCTGGGTTCACGGATCACCCCTGGC
6_known	N8	Tb927.10.13030_for	GGGGACAAGTTTGTACAAAAAAGCAGGCTATGGAAATAATTAGTGTGGCTGAGGAG	Tb927.10.13030_rev	GGGGACCACTTTGTACAAGAAAGCTGGGTTTACTCCCATTCTAGAGGAACAACACA
6_known	N9	Tb927.10.5010_for	GGGGACAAGTTTGTACAAAAAAGCAGGCTATGAGGAGGTTTTGTGATGTCGTC	Tb927.10.5010_rev	GGGGACCACTTTGTACAAGAAAGCTGGGTTTACTGTCCACTCAACCTGTACAGC
6_known	N10	Tb927.7.4020_for	GGGGACAAGTTTGTACAAAAAAGCAGGCTATGTATACCAGTGTTAGAAAGCCTCCG	Tb927.7.4020_rev	GGGGACCACTTTGTACAAGAAAGCTGGGTTTACCACAGAGAGCGACTCCG
6_known	N11	Tb927.3.2020_for	GGGGACAAGTTTGTACAAAAAAGCAGGCTATGAGCAACATTAAGGTGGCAGTT	Tb927.3.2020_rev	GGGGACCACTTTGTACAAGAAAGCTGGGTCTATTTTTCACTGGGCGCTGAGG
6_known	N12	Tb927.7.6940_for	GGGGACAAGTTTGTACAAAAAAGCAGGCTATGAAAAGCAACTGGAGAGACAAGAA	Tb927.7.6940_rev	GGGGACCACTTTGTACAAGAAAGCTGGGTTCAACTTGCATCCATCCATCCCT
6_known	N13	Tb927.11.2530_for	GGGGACAAGTTTGTACAAAAAAGCAGGCTATGCGGCGGTGTTCCTTTG	Tb927.11.2530_rev	GGGGACCACTTTGTACAAGAAAGCTGGGTTCACAAACTTACCTTCCCTACAGAAGG
6_known	N14	Tb927.8.2870_for	GGGGACAAGTTTGTACAAAAAAGCAGGCTATGGCGGATCACAGTGTCAAAG	Tb927.8.2870_rev	GGGGACCACTTTGTACAAGAAAGCTGGGTTCAAAGAAGGGCGAGAGTCTCG
6_known	N15	Tb927.8.7340_for	GGGGACAAGTTTGTACAAAAAAGCAGGCTATGGCATCCTACATGTTGTTGCTAG	Tb927.8.7340_rev	GGGGACCACTTTGTACAAGAAAGCTGGGTCTATGCTGACAGTAACTGGAGCAG
6_known	N17	Tb927.3.4940_for	GGGGACAAGTTTGTACAAAAAAGCAGGCTATGACCTCACTCGTCCTAAATCACT	Tb927.3.4940_rev	GGGGACCACTTTGTACAAGAAAGCTGGGTTTATTTCTTTCGGTAGACGGACAGC
6_known	N18	Tb927.8.4500_for	GGGGACAAGTTTGTACAAAAAAGCAGGCTATGGAGCACCGCGCCGCA	Tb927.8.4500_rev	GGGGACCACTTTGTACAAGAAAGCTGGGTTTATTTAGATTTCTCAAACGCAGCAAAGG
6_known	N19	Tb927.8.2630_for	GGGGACAAGTTTGTACAAAAAAGCAGGCTATGCGGACAAATGAAGAAAGAGATGA	Tb927.8.2630_rev	GGGGACCACTTTGTACAAGAAAGCTGGGTCTACTTTCTCTTCACCATCGCCTTC
6_known	N20	Tb927.11.14270_for	GGGGACAAGTTTGTACAAAAAAGCAGGCTATGGTCTCTCTCCGGATCAGGA	Tb927.11.14270_rev	GGGGACCACTTTGTACAAGAAAGCTGGGTTCAACACAAGTGGGGAAATTTTTCATC
6_known	N21	Tb927.10.15260_for	GGGGACAAGTTTGTACAAAAAAGCAGGCTATGAAGAAATCCACAGTACTTGATGCG	Tb927.10.15260_rev	GGGGACCACTTTGTACAAGAAAGCTGGGTCTAAAATACAACAGGACTAACATCCTCTTC
6_known	N22	Tb927.11.2040_for	GGGGACAAGTTTGTACAAAAAAGCAGGCTATGAAGCTGAGGGCTGACTCC	Tb927.11.2040_rev	GGGGACCACTTTGTACAAGAAAGCTGGGTTTATTTGATCAACCACTTGATCACCGA
6_known	N23	Tb927.11.11800_for	GGGGACAAGTTTGTACAAAAAAGCAGGCTATGCGGCGCTTAGTGCGT	Tb927.11.11800_rev	GGGGACCACTTTGTACAAGAAAGCTGGGTTCAGTCCGTCAAACCAACAAGTAAC
6_known	N24	Tb927.3.3640_for	GGGGACAAGTTTGTACAAAAAAGCAGGCTATGATTAGCTCCCATGATATTGAATTAGTT	Tb927.3.3640_rev	GGGGACCACTTTGTACAAGAAAGCTGGGTTTAACAACGAAAAATACGCCGAAGC
6_known	O1	Tb927.9.14240_for	GGGGACAAGTTTGTACAAAAAAGCAGGCTATGAGGTGCACGGCATCCG	Tb927.9.14240_rev	GGGGACCACTTTGTACAAGAAAGCTGGGTCTATAGAAGCCTCATCAGCTTCTCACG
6_known	O2	Tb927.2.4390_for	GGGGACAAGTTTGTACAAAAAAGCAGGCTATGGCCGAGAGGGCATCC	Tb927.2.4390_rev	GGGGACCACTTTGTACAAGAAAGCTGGGTCTAATAGTTATCTGGCATGCCAGAACTAC
6_known	O3	Tb927.5.1560_for	GGGGACAAGTTTGTACAAAAAAGCAGGCTATGCCACCGACCCCGTTG	Tb927.5.1560_rev	GGGGACCACTTTGTACAAGAAAGCTGGGTTTAATGCCTCGACATGCGCC
6_known	O4	Tb927.11.2650_for	GGGGACAAGTTTGTACAAAAAAGCAGGCTATGATGCGTCGTGTTTGTCAGA	Tb927.11.2650_rev	GGGGACCACTTTGTACAAGAAAGCTGGGTCTACTTATCGGCAGTGGGCTCC
6_known	O5	Tb927.11.12930_for	GGGGACAAGTTTGTACAAAAAAGCAGGCTATGGGACGACGCAACATAGTTTT	Tb927.11.12930_rev	GGGGACCACTTTGTACAAGAAAGCTGGGTTTATTTAGGTGGCCCTCGAGGTG
6_known	O6	Tb927.11.15850_for	GGGGACAAGTTTGTACAAAAAAGCAGGCTATGCGGCACACAATACCTTTTTC	Tb927.11.15850_rev	GGGGACCACTTTGTACAAGAAAGCTGGGTTCAGTTATTGTCATCAAGTATATCCTTCGC
6_known	O7	Tb927.10.11180_for	GGGGACAAGTTTGTACAAAAAAGCAGGCTATGGAGCGCTTGAAGTATTTGATGC	Tb927.10.11180_rev	GGGGACCACTTTGTACAAGAAAGCTGGGTCTACTGATTGTCCACATCATCATCCC
6_known	O8	Tb927.9.10640_for	GGGGACAAGTTTGTACAAAAAAGCAGGCTATGTCTGTAGGGGATTTTTTTGGAAATATC	Tb927.9.10640_rev	GGGGACCACTTTGTACAAGAAAGCTGGGTTCATTTCAGCAAAGGGTAGTTTGTGT
6_known	O9	Tb927.4.4230_for	GGGGACAAGTTTGTACAAAAAAGCAGGCTATGGGAAGGCGGATGCGG	Tb927.4.4230_rev	GGGGACCACTTTGTACAAGAAAGCTGGGTTTAGCGATCCACGCCAAACG
6_known	O10	Tb927.6.2550_for	GGGGACAAGTTTGTACAAAAAAGCAGGCTATGAGCGCACCACCACCG	Tb927.6.2550_rev	GGGGACCACTTTGTACAAGAAAGCTGGGTTTACCGACGCCTCTTCTTTGTG
6_known	O11	Tb927.10.11730_for	GGGGACAAGTTTGTACAAAAAAGCAGGCTATGGATAGCTCGCTTTGCTTCG	Tb927.10.11730_rev	GGGGACCACTTTGTACAAGAAAGCTGGGTTCACGGTTCCCCACTCGTG
6_known	O12	Tb927.7.4400_for	GGGGACAAGTTTGTACAAAAAAGCAGGCTATGGGGGAAGAGGAGAATTTACGT	Tb927.7.4400_rev	GGGGACCACTTTGTACAAGAAAGCTGGGTTTATGTGAGCATGTCAAGTACAGTTTTCA
6_known	O13	Tb927.2.4720_for	GGGGACAAGTTTGTACAAAAAAGCAGGCTATGACTGTTTTATCGTTTTTTAGTTTTTTA	Tb927.2.4720_rev	GGGGACCACTTTGTACAAGAAAGCTGGGTTCAACATCGTGGACCAAGGGC
6_known	O14	Tb927.3.2950_for	GGGGACAAGTTTGTACAAAAAAGCAGGCTATGGTTGTAGGTGCTGTGAAAGAG	Tb927.3.2950_rev	GGGGACCACTTTGTACAAGAAAGCTGGGTTTATCGTCTTGAAGATCTCTGAGGAGAT
6_known	O15	Tb927.9.2470_for	GGGGACAAGTTTGTACAAAAAAGCAGGCTATGACGGGAATGAACAGATCGTG	Tb927.9.2470_rev	GGGGACCACTTTGTACAAGAAAGCTGGGTTTAAGATGCCTGTTGCACAGATGC
6_known	O16	Tb927.1.760_for	GGGGACAAGTTTGTACAAAAAAGCAGGCTATGTTCGCCCCACCGGAG	Tb927.1.760_rev	GGGGACCACTTTGTACAAGAAAGCTGGGTCTACTGTCCAATACGATCTTCAGCG
6_known	O17	Tb927.2.4330_for	GGGGACAAGTTTGTACAAAAAAGCAGGCTATGACGCAATCAGCTCAATCAACT	Tb927.2.4330_rev	GGGGACCACTTTGTACAAGAAAGCTGGGTTCAATCATCTTCATCACAGGGTGC
6_known	O18	Tb927.2.2470_for	GGGGACAAGTTTGTACAAAAAAGCAGGCTATGAGACGCCTCACCCGC	Tb927.2.2470_rev	GGGGACCACTTTGTACAAGAAAGCTGGGTTTACCCTACAACTTTCACATAGCCGA
6_known	O19	Tb927.11.8010_for	GGGGACAAGTTTGTACAAAAAAGCAGGCTATGTTGTTTTGTCGGCATTTATTGTTCA	Tb927.11.8010_rev	GGGGACCACTTTGTACAAGAAAGCTGGGTTTATCTTGAACTTTTACTGCCACTGGC
6_known	O20	Tb927.10.460_for	GGGGACAAGTTTGTACAAAAAAGCAGGCTATGGAGAATTACACCCAACTTCGTG	Tb927.10.460_rev	GGGGACCACTTTGTACAAGAAAGCTGGGTCTACCGCAGCATTTGACGATTAAATG
6_known	O21	Tb927.11.5270_for	GGGGACAAGTTTGTACAAAAAAGCAGGCTATGGATATCGTTAATAACAATGGTTCTTTT	Tb927.11.5270_rev	GGGGACCACTTTGTACAAGAAAGCTGGGTTTACGAGAATTTTCCGTGGGCG
6_known	O22	Tb927.11.5220_for	GGGGACAAGTTTGTACAAAAAAGCAGGCTATGCAGTTGCATTTATCTTCAATCAGC	Tb927.11.5220_rev	GGGGACCACTTTGTACAAGAAAGCTGGGTTCACAGCTCCGGAAGCGG
6_known	O23	Tb927.10.2640_for	GGGGACAAGTTTGTACAAAAAAGCAGGCTATGAACTTTGGCCACACCATGG	Tb927.10.2640_rev	GGGGACCACTTTGTACAAGAAAGCTGGGTCTAGTCACCCGGAAGTACCAACA
6_known	O24	Tb927.7.6310_for	GGGGACAAGTTTGTACAAAAAAGCAGGCTATGCACGCAACCGCTGAG	Tb927.7.6310_rev	GGGGACCACTTTGTACAAGAAAGCTGGGTCTAAATATCACGGTTTTGTATGAGCTCGC
6_known	P1	Tb927.8.8350_for	GGGGACAAGTTTGTACAAAAAAGCAGGCTATGCAGAAAAGTATACAAGATGAAGTGCG	Tb927.8.8350_rev	GGGGACCACTTTGTACAAGAAAGCTGGGTCTAGTTTTGTTGCTGTTGTATTTGACGT
6_known	P2	Tb927.10.2960_for	GGGGACAAGTTTGTACAAAAAAGCAGGCTATGAGGTTATGGAATAGATTCTCTCGTCT	Tb927.10.2960_rev	GGGGACCACTTTGTACAAGAAAGCTGGGTTCACAATGCAGTGGCACCC
6_known	P3	Tb927.9.11930_for	GGGGACAAGTTTGTACAAAAAAGCAGGCTATGGGGCAGCAGAAAAAAGATGC	Tb927.9.11930_rev	GGGGACCACTTTGTACAAGAAAGCTGGGTTCAGTGAGAGGAAAGATGTCCATGT
6_known	P4	Tb927.11.12310_for	GGGGACAAGTTTGTACAAAAAAGCAGGCTATGGGTATGGCAACACGTTCG	Tb927.11.12310_rev	GGGGACCACTTTGTACAAGAAAGCTGGGTTTACGCAGCGCTTGGATAAGG
6_known	P5	Tb927.4.2720_for	GGGGACAAGTTTGTACAAAAAAGCAGGCTATGGAAAGACTGTACCGTAGCCA	Tb927.4.2720_rev	GGGGACCACTTTGTACAAGAAAGCTGGGTTTATTTATTACTCTTCATACCATAAAGTGC
6_known	P6	Tb927.10.9230_for	GGGGACAAGTTTGTACAAAAAAGCAGGCTATGGAAACCTCATGTGCCTCTGC	Tb927.10.9230_rev	GGGGACCACTTTGTACAAGAAAGCTGGGTTCAAAACTGATCCATGATTTTGTGAAAATG
6_known	P7	Tb927.7.1240_for	GGGGACAAGTTTGTACAAAAAAGCAGGCTATGAATGCCAACGGGGTACCC	Tb927.7.1240_rev	GGGGACCACTTTGTACAAGAAAGCTGGGTCTACTTCCCAGGCAAACTCAGGC
6_known	P8	Tb927.11.5570_for	GGGGACAAGTTTGTACAAAAAAGCAGGCTATGGAATCGGATGCGGGTGT	Tb927.11.5570_rev	GGGGACCACTTTGTACAAGAAAGCTGGGTTTAACGTAACCGATGAATTTGGGTGC
6_known	P9	Tb927.4.1340_for	GGGGACAAGTTTGTACAAAAAAGCAGGCTATGTTTTCACACGCGGCGT	Tb927.4.1340_rev	GGGGACCACTTTGTACAAGAAAGCTGGGTCTATGCGCCCTCGTCAATGG
6_known	P10	Tb927.7.6850_for	GGGGACAAGTTTGTACAAAAAAGCAGGCTATGGAGGAACTCCACCAACAAATG	Tb927.7.6850_rev	GGGGACCACTTTGTACAAGAAAGCTGGGTTCAAATCGCCAACACATACATTAAGATCA
6_known	P11	Tb927.3.3580_for	GGGGACAAGTTTGTACAAAAAAGCAGGCTATGATCCAATCGGGTATGTTCTTCG	Tb927.3.3580_rev	GGGGACCACTTTGTACAAGAAAGCTGGGTTTACAAGTCTCCAGCATTGGCTCC
6_known	P12	Tb927.9.9600_for	GGGGACAAGTTTGTACAAAAAAGCAGGCTATGAATGGTGTTGCACCAATAACGG	Tb927.9.9600_rev	GGGGACCACTTTGTACAAGAAAGCTGGGTTCATGCATTAAACGACAAACTACGC
6_known	P13	Tb927.4.1840_for	GGGGACAAGTTTGTACAAAAAAGCAGGCTATGATGGTAGAAGCAAAGGTGCAG	Tb927.4.1840_rev	GGGGACCACTTTGTACAAGAAAGCTGGGTTCAAGAGAATGAAGTACGATTGGCC
6_known	P14	Tb927.10.1500_for	GGGGACAAGTTTGTACAAAAAAGCAGGCTATGGCTCTAAAGCTGCTTTCAGAAA	Tb927.10.1500_rev	GGGGACCACTTTGTACAAGAAAGCTGGGTTTATGTACTCTTTGTATTCTCTGTTGAGCG
6_known	P15	Tb927.7.680_for	GGGGACAAGTTTGTACAAAAAAGCAGGCTATGCGGCGGCATGTTTCT	Tb927.7.680_rev	GGGGACCACTTTGTACAAGAAAGCTGGGTTTAAAAAGAACCCGGTTTACTTGTAGAGG
6_known	P16	Tb927.2.4510_for	GGGGACAAGTTTGTACAAAAAAGCAGGCTATGGCTGAGTTGTTAAGAAAGAAAATTGAG	Tb927.2.4510_rev	GGGGACCACTTTGTACAAGAAAGCTGGGTTTAGCGGCGCCGCACTTC
6_known	P17	Tb927.5.440_for	GGGGACAAGTTTGTACAAAAAAGCAGGCTATGAGAGTATGGATGAAAGATGACTTCCC	Tb927.5.440_rev	GGGGACCACTTTGTACAAGAAAGCTGGGTTCATTTCCTTACATCCAGTGTGTCATC
6_known	P18	Tb927.11.11920_for	GGGGACAAGTTTGTACAAAAAAGCAGGCTATGTTGCGGCTCGTGCTG	Tb927.11.11920_rev	GGGGACCACTTTGTACAAGAAAGCTGGGTCTACCGTTTCTCAGGGCGTTG

6_known	P19	Tb927.7.3880_for	GGGGACAAGTTTGTACAAAAAAGCAGGCTATGGAACCCGCTGATGGGT	Tb927.7.3880_rev	GGGGACCACTTTGTACAAGAAAGCTGGGTTCAATCAACCTGCGCCAAAAAGG
6_known	P20	Tb927.8.1410_for	GGGGACAAGTTTGTACAAAAAAGCAGGCTATGGCACGTAATAAGGCTCACAAG	Tb927.8.1410_rev	GGGGACCACTTTGTACAAGAAAGCTGGGTCTACTCTTCGGCAACAACTTTCGG
6_known	P21	Tb927.11.16270_for	GGGGACAAGTTTGTACAAAAAAGCAGGCTATGCAGTGCCTGTTCTTGGAG	Tb927.11.16270_rev	GGGGACCACTTTGTACAAGAAAGCTGGGTCTAACTCTCATGATAGACAACGTCATCG
6_known	P22	Tb927.11.12830_for	GGGGACAAGTTTGTACAAAAAAGCAGGCTATGTGGAGCAAAGCGGAGC	Tb927.11.12830_rev	GGGGACCACTTTGTACAAGAAAGCTGGGTTCACCCAATTGAACTCTTACGATGC
6_known	P23	Tb927.8.1550_for	GGGGACAAGTTTGTACAAAAAAGCAGGCTATGTCGGCCACTTATCCAGAGC	Tb927.8.1550_rev	GGGGACCACTTTGTACAAGAAAGCTGGGTTTATGCGCACTCCTCTACAGCA
6_known	P24	Tb927.7.990_for	GGGGACAAGTTTGTACAAAAAAGCAGGCTATGTCTGACAGTGGTGACGAATACG	Tb927.7.990_rev	GGGGACCACTTTGTACAAGAAAGCTGGGTCTACGACTCAACCAAATGTTCCCTT
7_hypothetical	A1	Tb927.5.3930_for	GGGGACAAGTTTGTACAAAAAAGCAGGCTATGACGGGAAAAAATCACTCGGTC	Tb927.5.3930_rev	GGGGACCACTTTGTACAAGAAAGCTGGGTTTACTTCGTGTTAATAAACGCTAGTGTAAT
7_hypothetical	A2	Tb927.6.2860_for	GGGGACAAGTTTGTACAAAAAAGCAGGCTATGCAACAGCGTTCCATCAGT	Tb927.6.2860_rev	GGGGACCACTTTGTACAAGAAAGCTGGGTTCAGTTAAACCTATAAACCATAGCTTTGCC
7_hypothetical	A3	Tb927.7.4610_for	GGGGACAAGTTTGTACAAAAAAGCAGGCTATGGAAGCTGCTGCCGAGC	Tb927.7.4610_rev	GGGGACCACTTTGTACAAGAAAGCTGGGTTCACGTTTCGTCGTTGCTTTGC
7_hypothetical	A4	Tb927.10.5870_for	GGGGACAAGTTTGTACAAAAAAGCAGGCTATGATTTTTCAAGACACGAGCTCCT	Tb927.10.5870_rev	GGGGACCACTTTGTACAAGAAAGCTGGGTTCAACGACGATGACGAAGCATG
7_hypothetical	A5	Tb927.7.2960_for	GGGGACAAGTTTGTACAAAAAAGCAGGCTATGGCGGGCATCAGGTTTG	Tb927.7.2960_rev	GGGGACCACTTTGTACAAGAAAGCTGGGTTCACCTGAGCTGTTGCATGC
7_hypothetical	A6	Tb927.6.3810_for	GGGGACAAGTTTGTACAAAAAAGCAGGCTATGTTTGGTCCTGTAGACGATGC	Tb927.6.3810_rev	GGGGACCACTTTGTACAAGAAAGCTGGGTTCACAACGCTCGAATAAGGAAGATTT
7_hypothetical	A7	Tb927.10.13230_for	GGGGACAAGTTTGTACAAAAAAGCAGGCTATGATGAGCTCTCGCTCCGT	Tb927.10.13230_rev	GGGGACCACTTTGTACAAGAAAGCTGGGTTCAGAGAGAAATTACGTCGACTGTTTG
7_hypothetical	A8	Tb927.10.13640_for	GGGGACAAGTTTGTACAAAAAAGCAGGCTATGGCCGAAACCTTTCAGTTGC	Tb927.10.13640_rev	GGGGACCACTTTGTACAAGAAAGCTGGGTTTAACCATCCTCGTGACCCAGC
7_hypothetical	A9	Tb927.9.2310_for	GGGGACAAGTTTGTACAAAAAAGCAGGCTATGGGTGATTTGTTAGAACTGACACC	Tb927.9.2310_rev	GGGGACCACTTTGTACAAGAAAGCTGGGTTCACCAATACTTATGTGACTCTTTCATTCT
7_hypothetical	A10	Tb927.11.3670_for	GGGGACAAGTTTGTACAAAAAAGCAGGCTATGCTCGTATGTTGTCGAAGCAG	Tb927.11.3670_rev	GGGGACCACTTTGTACAAGAAAGCTGGGTCTATGCAGTTCGCGATACAGCA
7_hypothetical	A11	Tb927.4.3670_for	GGGGACAAGTTTGTACAAAAAAGCAGGCTATGGAGAAAGAATTCACGAAGGCAC	Tb927.4.3670_rev	GGGGACCACTTTGTACAAGAAAGCTGGGTTTACACAAACAGCAGCGCTCC
7_hypothetical	A12	Tb927.10.8060_for	GGGGACAAGTTTGTACAAAAAAGCAGGCTATGGTTCTCCCCTCACCACATT	Tb927.10.8060_rev	GGGGACCACTTTGTACAAGAAAGCTGGGTTTATTTCTCACTTCCATTGACCTGACG
7_hypothetical	A13	Tb927.11.10770_for	GGGGACAAGTTTGTACAAAAAAGCAGGCTATGGTGCTCCTCTCTGTTCTGG	Tb927.11.10770_rev	GGGGACCACTTTGTACAAGAAAGCTGGGTCTACTTTGGTGCCTGTCGCG
7_hypothetical	A14	Tb927.7.4810_for	GGGGACAAGTTTGTACAAAAAAGCAGGCTATGAGAAATGGAATTATCTGTCGATACTTG	Tb927.7.4810_rev	GGGGACCACTTTGTACAAGAAAGCTGGGTTTATGGAGTATTGTAGAAGGGAAGTGAGT
7_hypothetical	A15	Tb927.9.10840_for	GGGGACAAGTTTGTACAAAAAAGCAGGCTATGCACCGGTGCGCTCTG	Tb927.9.10840_rev	GGGGACCACTTTGTACAAGAAAGCTGGGTTCATGTGAAGGACACGGGTCT
7_hypothetical	A16	Tb927.8.5370_for	GGGGACAAGTTTGTACAAAAAAGCAGGCTATGTCCCTCTCACCACAGTGG	Tb927.8.5370_rev	GGGGACCACTTTGTACAAGAAAGCTGGGTTCAGGCACTCGGCGCCAA
7_hypothetical	A17	Tb927.9.5880_for	GGGGACAAGTTTGTACAAAAAAGCAGGCTATGTATGGCATTAGTGAAGATGGTGG	Tb927.9.5880_rev	GGGGACCACTTTGTACAAGAAAGCTGGGTCTACAGACGGCGCTTCTTTTTCT
7_hypothetical	A18	Tb927.10.7040_for	GGGGACAAGTTTGTACAAAAAAGCAGGCTATGGTGGGTTGCAGAAGGTTG	Tb927.10.7040_rev	GGGGACCACTTTGTACAAGAAAGCTGGGTTCATCTTCTCCCCGATGTCTCAAC
7_hypothetical	A19	Tb927.10.9960_for	GGGGACAAGTTTGTACAAAAAAGCAGGCTATGACGGCGGGACTCGTT	Tb927.10.9960_rev	GGGGACCACTTTGTACAAGAAAGCTGGGTTCACGAGGACGACGCTGC
7_hypothetical	A20	Tb927.5.2920_for	GGGGACAAGTTTGTACAAAAAAGCAGGCTATGTCCAGAAGTGTTCCGTGGC	Tb927.5.2920_rev	GGGGACCACTTTGTACAAGAAAGCTGGGTTCAGACAGTACCATAAAAGAATTGTTCGT
7_hypothetical	A21	Tb927.8.970_for	GGGGACAAGTTTGTACAAAAAAGCAGGCTATGACGCGATTCACCTGTTTCA	Tb927.8.970_rev	GGGGACCACTTTGTACAAGAAAGCTGGGTCTACAAAAGACCAGTTCCTAGCAAACA
7_hypothetical	A22	Tb927.4.1110_for	GGGGACAAGTTTGTACAAAAAAGCAGGCTATGCTGGATGCCCTTCCACT	Tb927.4.1110_rev	GGGGACCACTTTGTACAAGAAAGCTGGGTCTACGAGGCTGAGGAGCATGC
7_hypothetical	A23	Tb927.7.4960_for	GGGGACAAGTTTGTACAAAAAAGCAGGCTATGGGTGAAAATGACAACGCTCA	Tb927.7.4960_rev	GGGGACCACTTTGTACAAGAAAGCTGGGTTCACACCGGATGATGTGTAGCT
7_hypothetical	A24	Tb927.11.6000_for	GGGGACAAGTTTGTACAAAAAAGCAGGCTATGCGCCGCGTCTCTTTC	Tb927.11.6000_rev	GGGGACCACTTTGTACAAGAAAGCTGGGTCTAACTGAGCATTGACCACGATACTT
7_hypothetical	B1	Tb927.7.2130_for	GGGGACAAGTTTGTACAAAAAAGCAGGCTATGGCGCGGGTAACGCTA	Tb927.7.2130_rev	GGGGACCACTTTGTACAAGAAAGCTGGGTTTAAGAGAGGTTGCCATACATTACTGGA
7_hypothetical	B2	Tb927.10.14190_for	GGGGACAAGTTTGTACAAAAAAGCAGGCTATGATTCATCCGAAGCTTATGCCTTATC	Tb927.10.14190_rev	GGGGACCACTTTGTACAAGAAAGCTGGGTTCAACAATGCCGTCGCTCG
7_hypothetical	B3	Tb927.9.9700_for	GGGGACAAGTTTGTACAAAAAAGCAGGCTATGAGACTCCGGCGCATCG	Tb927.9.9700_rev	GGGGACCACTTTGTACAAGAAAGCTGGGTCTAGTGATGGTTGTTCCGGTAAAAACG
7_hypothetical	B4	Tb927.11.3720_for	GGGGACAAGTTTGTACAAAAAAGCAGGCTATGATCCGTTGGGACCATGGG	Tb927.11.3720_rev	GGGGACCACTTTGTACAAGAAAGCTGGGTCTAATGATCGCCGCCACGG
7_hypothetical	B5	Tb927.11.10360_for	GGGGACAAGTTTGTACAAAAAAGCAGGCTATGGCCACGACTGGGTATACTC	Tb927.11.10360_rev	GGGGACCACTTTGTACAAGAAAGCTGGGTTCATTCCTCCCACAGCTTTCTCA
7_hypothetical	B6	Tb927.4.1050_for	GGGGACAAGTTTGTACAAAAAAGCAGGCTATGCGCTTCCTCGGTGCA	Tb927.4.1050_rev	GGGGACCACTTTGTACAAGAAAGCTGGGTCTAACAATGAAACTCAGGTGCGCT
7_hypothetical	B7	Tb927.11.2610_for	GGGGACAAGTTTGTACAAAAAAGCAGGCTATGGCAGCCGCTGTTACAATT	Tb927.11.2610_rev	GGGGACCACTTTGTACAAGAAAGCTGGGTCTACGACGTGCGTTCGACTT
7_hypothetical	B8	Tb927.11.3840_for	GGGGACAAGTTTGTACAAAAAAGCAGGCTATGGCCCGTCGGCAACGT	Tb927.11.3840_rev	GGGGACCACTTTGTACAAGAAAGCTGGGTTCAGGAGAAAAGTGGAAATTGGTGAC
7_hypothetical	B9	Tb927.4.640_for	GGGGACAAGTTTGTACAAAAAAGCAGGCTATGGACGCTGTTGTAAATAGTGAAGT	Tb927.4.640_rev	GGGGACCACTTTGTACAAGAAAGCTGGGTTCAAAACCTTCTCTTGACCCACCC
7_hypothetical	B10	Tb927.9.13300_for	GGGGACAAGTTTGTACAAAAAAGCAGGCTATGCCGCCGGCAGGTGGA	Tb927.9.13300_rev	GGGGACCACTTTGTACAAGAAAGCTGGGTTTAGTAGTCAACCTTCACATAAGCCGA
7_hypothetical	B11	Tb927.5.1690_for	GGGGACAAGTTTGTACAAAAAAGCAGGCTATGGAGAGCTACAAGGATGTGATTCT	Tb927.5.1690_rev	GGGGACCACTTTGTACAAGAAAGCTGGGTTCATATGGCAGGGCAACGATACA
7_hypothetical	B12	Tb927.7.590_for	GGGGACAAGTTTGTACAAAAAAGCAGGCTATGTTGTCTGCTCCACGACGC	Tb927.7.590_rev	GGGGACCACTTTGTACAAGAAAGCTGGGTTCAGTACAGAAGAGAGGAGAGGCG
7_hypothetical	B13	Tb927.11.170_for	GGGGACAAGTTTGTACAAAAAAGCAGGCTATGAGATGGACTGCTGTGTTATGTAAAG	Tb927.11.170_rev	GGGGACCACTTTGTACAAGAAAGCTGGGTCTATCGCACTCCATAACTTCGAAGG
7_hypothetical	B14	Tb927.6.880_for	GGGGACAAGTTTGTACAAAAAAGCAGGCTATGGTGTACATTCTTGCCCATGATG	Tb927.6.880_rev	GGGGACCACTTTGTACAAGAAAGCTGGGTCTACGCATTTTTCTTAGCATTTAGCGC
7_hypothetical	B15	Tb927.9.1560_for	GGGGACAAGTTTGTACAAAAAAGCAGGCTATGGACGTCGTGGTTCTTGC	Tb927.9.1560_rev	GGGGACCACTTTGTACAAGAAAGCTGGGTCTAATCGTCATCGTCTGAAGAAGTTTCT
7_hypothetical	B16	Tb927.11.1940_for	GGGGACAAGTTTGTACAAAAAAGCAGGCTATGGGCTCTGAATGCTCAAGGC	Tb927.11.1940_rev	GGGGACCACTTTGTACAAGAAAGCTGGGTTTACTCTTGTAGAAGCGATACTAGTCTTTT
7_hypothetical	B17	Tb927.11.6060_for	GGGGACAAGTTTGTACAAAAAAGCAGGCTATGGAAGGGGGGTTAAGAAAGCG	Tb927.11.6060_rev	GGGGACCACTTTGTACAAGAAAGCTGGGTTTAGCCCATCAAAAAGGTGGGAAAC
7_hypothetical	B18	Tb927.10.15770_for	GGGGACAAGTTTGTACAAAAAAGCAGGCTATGATCCAGTCGTCTAAAGAGCGA	Tb927.10.15770_rev	GGGGACCACTTTGTACAAGAAAGCTGGGTCTACACGGCGTCGTGACC
7_hypothetical	B19	Tb927.11.6480_for	GGGGACAAGTTTGTACAAAAAAGCAGGCTATGGAGATTGCTATAGTTGCTGCAC	Tb927.11.6480_rev	GGGGACCACTTTGTACAAGAAAGCTGGGTTCACCTGCCAGCCGCACG
7_hypothetical	B20	Tb927.6.2870_for	GGGGACAAGTTTGTACAAAAAAGCAGGCTATGTATCGTTTTTCAATCAGTGGTAGTGC	Tb927.6.2870_rev	GGGGACCACTTTGTACAAGAAAGCTGGGTTTATACACAGCGCTGTTGTAACTCC
7_hypothetical	B21	Tb927.11.8710_for	GGGGACAAGTTTGTACAAAAAAGCAGGCTATGTGGCGTCACAGCTGTATTC	Tb927.11.8710_rev	GGGGACCACTTTGTACAAGAAAGCTGGGTTTACGGTTCAATGGTGGCGTG
7_hypothetical	B22	Tb927.9.1710_for	GGGGACAAGTTTGTACAAAAAAGCAGGCTATGACGGGGTACAACTTGAAAACTATAG	Tb927.9.1710_rev	GGGGACCACTTTGTACAAGAAAGCTGGGTCTAAGAAGTTACGTCAGCGCAGT
7_hypothetical	B23	Tb927.8.910_for	GGGGACAAGTTTGTACAAAAAAGCAGGCTATGACTGAATTCAATTACGATACGATGTTT	Tb927.8.910_rev	GGGGACCACTTTGTACAAGAAAGCTGGGTTTAATTGAAGCGACATCCCATGGG
7_hypothetical	B24	Tb927.11.4860_for	GGGGACAAGTTTGTACAAAAAAGCAGGCTATGGCCGTGGCCTACGTT	Tb927.11.4860_rev	GGGGACCACTTTGTACAAGAAAGCTGGGTTTACTGCGGTGGGAAGAAGTGT
7_hypothetical	C1	Tb927.10.14520_for	GGGGACAAGTTTGTACAAAAAAGCAGGCTATGAGCGCGGACACTATTTGTC	Tb927.10.14520_rev	GGGGACCACTTTGTACAAGAAAGCTGGGTTTACAAATGGTGAGCGCTTGTGC
7_hypothetical	C2	Tb927.7.660_for	GGGGACAAGTTTGTACAAAAAAGCAGGCTATGCCAAATGAAAAGGCGCAGA	Tb927.7.660_rev	GGGGACCACTTTGTACAAGAAAGCTGGGTTTACAATAGAAGACCAAACTCGCGC
7_hypothetical	C3	Tb927.8.4250_for	GGGGACAAGTTTGTACAAAAAAGCAGGCTATGTGGTTCAACCACCGCATC	Tb927.8.4250_rev	GGGGACCACTTTGTACAAGAAAGCTGGGTTTACAAAACGAACTTGTACGGCTTTC
7_hypothetical	C4	Tb927.5.2470_for	GGGGACAAGTTTGTACAAAAAAGCAGGCTATGGTAAACAATGAAGGGTATTCGCA	Tb927.5.2470_rev	GGGGACCACTTTGTACAAGAAAGCTGGGTTTATGAGGGAGTGATATCTGCAGGTG
7_hypothetical	C5	Tb927.5.1780_for	GGGGACAAGTTTGTACAAAAAAGCAGGCTATGTCATCGGTGGGAATCGAGT	Tb927.5.1780_rev	GGGGACCACTTTGTACAAGAAAGCTGGGTCTAACGAGGAAGCGATGCGAG
7_hypothetical	C6	Tb927.10.7250_for	GGGGACAAGTTTGTACAAAAAAGCAGGCTATGCCGGCCACGTTTAGC	Tb927.10.7250_rev	GGGGACCACTTTGTACAAGAAAGCTGGGTTTACCCACTGGGTGGGGC
7_hypothetical	C7	Tb927.10.5900_for	GGGGACAAGTTTGTACAAAAAAGCAGGCTATGTCTTTTTACCGTACAAAAAAGGTTATA	Tb927.10.5900_rev	GGGGACCACTTTGTACAAGAAAGCTGGGTTCACATAGCTACAGGGGCCTC
7_hypothetical	C8	Tb927.3.2160_for	GGGGACAAGTTTGTACAAAAAAGCAGGCTATGACGGGAGAGCCTGTTCG	Tb927.3.2160_rev	GGGGACCACTTTGTACAAGAAAGCTGGGTTCAGGGTCCCAAATTATCGGGG
7_hypothetical	C9	Tb927.4.1430_for	GGGGACAAGTTTGTACAAAAAAGCAGGCTATGACCTTGCCTGAATTGGCG	Tb927.4.1430_rev	GGGGACCACTTTGTACAAGAAAGCTGGGTTCATCCTTGGCTAGCTGGGTAAG
7_hypothetical	C10	Tb927.1.4800_for	GGGGACAAGTTTGTACAAAAAAGCAGGCTATGCGCTCGCGCGAGGTG	Tb927.1.4800_rev	GGGGACCACTTTGTACAAGAAAGCTGGGTTTATGCACACCGCCTTCCCC
7_hypothetical	C11	Tb927.10.9680_for	GGGGACAAGTTTGTACAAAAAAGCAGGCTATGAGTGTTTGCCTTGGCACG	Tb927.10.9680_rev	GGGGACCACTTTGTACAAGAAAGCTGGGTTTAACACATTCCTTTCACGTACTCCTG
7_hypothetical	C12	Tb927.4.3140_for	GGGGACAAGTTTGTACAAAAAAGCAGGCTATGAGCGCGCGCATGCAG	Tb927.4.3140_rev	GGGGACCACTTTGTACAAGAAAGCTGGGTTCACTCCTCTCCTACCCCTTCC
7_hypothetical	C13	Tb927.9.10400_for	GGGGACAAGTTTGTACAAAAAAGCAGGCTATGTCTGTGTTACGTCTTCCAAAAGAT	Tb927.9.10400_rev	GGGGACCACTTTGTACAAGAAAGCTGGGTTTAATCGCTGTCATCGAACCTTGTG
7_hypothetical	C14	Tb927.9.6650_for	GGGGACAAGTTTGTACAAAAAAGCAGGCTATGAAGACTGTAATTTCCACTGCTTTTGC	Tb927.9.6650_rev	GGGGACCACTTTGTACAAGAAAGCTGGGTTTACCCCTTGGAAGTCAACTCCTT
7_hypothetical	C15	Tb927.9.14290_for	GGGGACAAGTTTGTACAAAAAAGCAGGCTATGACCGCCTCAGCGAGG	Tb927.9.14290_rev	GGGGACCACTTTGTACAAGAAAGCTGGGTCTAAAACATTGCACAGCATGGCG
7_hypothetical	C16	Tb927.6.1130_for	GGGGACAAGTTTGTACAAAAAAGCAGGCTATGGGGCGGAGGTCCGAT	Tb927.6.1130_rev	GGGGACCACTTTGTACAAGAAAGCTGGGTCTAAGCGGGGGTGATTGTTTCTG
7_hypothetical	C17	Tb927.10.950_for	GGGGACAAGTTTGTACAAAAAAGCAGGCTATGCTGCGTGGCGTGACA	Tb927.10.950_rev	GGGGACCACTTTGTACAAGAAAGCTGGGTCTAACTGGACAGCATTAACGGAACC
7_hypothetical	C18	Tb927.6.1200_for	GGGGACAAGTTTGTACAAAAAAGCAGGCTATGCGGCGATGCGCTACA	Tb927.6.1200_rev	GGGGACCACTTTGTACAAGAAAGCTGGGTTTACTTCTCACTTAATAATTCCAACCAGTT
7_hypothetical	C19	Tb927.10.10200_for	GGGGACAAGTTTGTACAAAAAAGCAGGCTATGGCCATCACGGTGCAGC	Tb927.10.10200_rev	GGGGACCACTTTGTACAAGAAAGCTGGGTCTACTTCGGCCGTTCACCTCC
7_hypothetical	C20	Tb927.6.4940_for	GGGGACAAGTTTGTACAAAAAAGCAGGCTATGAGGCCACGATGTGGCT	Tb927.6.4940_rev	GGGGACCACTTTGTACAAGAAAGCTGGGTTTACACAACACCGTCCACGC
7_hypothetical	C21	Tb927.3.5260_for	GGGGACAAGTTTGTACAAAAAAGCAGGCTATGTTAAGTTTTTTGATTGACTGCAGCG	Tb927.3.5260_rev	GGGGACCACTTTGTACAAGAAAGCTGGGTTTATGTCACATGAGCCTTCCCCC
7_hypothetical	C22	Tb927.7.5140_for	GGGGACAAGTTTGTACAAAAAAGCAGGCTATGGGCTCCACGGAGGATG	Tb927.7.5140_rev	GGGGACCACTTTGTACAAGAAAGCTGGGTTTATACAATCCCTCCAGTGCTGTTCT
7_hypothetical	C23	Tb927.10.4260_for	GGGGACAAGTTTGTACAAAAAAGCAGGCTATGGATTGGGTATTTTTTCTCATGGCG	Tb927.10.4260_rev	GGGGACCACTTTGTACAAGAAAGCTGGGTTTACCACCGACTGACTCTAGCG
7_hypothetical	C24	Tb927.10.5470_for	GGGGACAAGTTTGTACAAAAAAGCAGGCTATGTTCCTTGCAGATGGTAGACGA	Tb927.10.5470_rev	GGGGACCACTTTGTACAAGAAAGCTGGGTTTATGGTCTTTTGTCCTCCCACAAC
7_hypothetical	D1	Tb927.11.13200_for	GGGGACAAGTTTGTACAAAAAAGCAGGCTATGGCCCGTGGCGGTGTG	Tb927.11.13200_rev	GGGGACCACTTTGTACAAGAAAGCTGGGTTCACTCATCATCAGAGCTTTGGGG
7_hypothetical	D2	Tb927.9.6370_for	GGGGACAAGTTTGTACAAAAAAGCAGGCTATGAGCAGCACAGACAATGATATCG	Tb927.9.6370_rev	GGGGACCACTTTGTACAAGAAAGCTGGGTTTACCGCAAAACCTTGCCGC
7_hypothetical	D3	Tb927.2.1850_for	GGGGACAAGTTTGTACAAAAAAGCAGGCTATGGCAGAGGGGGGTTTTAATATGC	Tb927.2.1850_rev	GGGGACCACTTTGTACAAGAAAGCTGGGTTCATGATGGGAGGAGACTCACAAC
7_hypothetical	D4	Tb927.8.950_for	GGGGACAAGTTTGTACAAAAAAGCAGGCTATGACACGATTCACCTGTTTCACA	Tb927.8.950_rev	GGGGACCACTTTGTACAAGAAAGCTGGGTCTACAAAAGACCAACTAATGTGAAAGTAAG
7_hypothetical	D5	Tb927.8.930_for	GGGGACAAGTTTGTACAAAAAAGCAGGCTATGACGCGATTCACCTGTTTCA	Tb927.8.930_rev	GGGGACCACTTTGTACAAGAAAGCTGGGTCTACAAAAGACCAACTAATGTGAAAGTAAG
7_hypothetical	D6	Tb927.8.2840_for	GGGGACAAGTTTGTACAAAAAAGCAGGCTATGGACATCCTGCGGAGGAC	Tb927.8.2840_rev	GGGGACCACTTTGTACAAGAAAGCTGGGTCTACGTGGCCAACCTCGTCG
7_hypothetical	D7	Tb927.4.610_for	GGGGACAAGTTTGTACAAAAAAGCAGGCTATGTCCGAAAACCCCGCTCA	Tb927.4.610_rev	GGGGACCACTTTGTACAAGAAAGCTGGGTTCACTGAGAACCCTCCTCCGG
7_hypothetical	D8	Tb927.8.5610_for	GGGGACAAGTTTGTACAAAAAAGCAGGCTATGCATGCGTATCATTTCCAGCA	Tb927.8.5610_rev	GGGGACCACTTTGTACAAGAAAGCTGGGTTCAAGAAAGGAGTACTAAGATAGGGTACG
7_hypothetical	D9	Tb927.11.4830_for	GGGGACAAGTTTGTACAAAAAAGCAGGCTATGTTCACTGTATCTTCATCGATGCG	Tb927.11.4830_rev	GGGGACCACTTTGTACAAGAAAGCTGGGTTCAAGCTGTATTTACACTTTGCTGTACT
7_hypothetical	D10	Tb927.4.3030_for	GGGGACAAGTTTGTACAAAAAAGCAGGCTATGACAGATGGAAACCCTGGCG	Tb927.4.3030_rev	GGGGACCACTTTGTACAAGAAAGCTGGGTTCAGCGCGTCACCGCTTC
7_hypothetical	D11	Tb927.9.7170_for	GGGGACAAGTTTGTACAAAAAAGCAGGCTATGCAGCGTAATTTAACGAAATTGAGC	Tb927.9.7170_rev	GGGGACCACTTTGTACAAGAAAGCTGGGTCTAAATGTCAGTAACGCCCACGT
7_hypothetical	D12	Tb927.10.9060_for	GGGGACAAGTTTGTACAAAAAAGCAGGCTATGTCCTCCACGACACTAGTGG	Tb927.10.9060_rev	GGGGACCACTTTGTACAAGAAAGCTGGGTTCACGCAGCAACGTCCTCG
7_hypothetical	D13	Tb927.10.11210_for	GGGGACAAGTTTGTACAAAAAAGCAGGCTATGATCTCGTGTGTTGCTGGTC	Tb927.10.11210_rev	GGGGACCACTTTGTACAAGAAAGCTGGGTTCAAGTGCGTGTTTTTTTCATGGC
7_hypothetical	D14	Tb927.10.4850_for	GGGGACAAGTTTGTACAAAAAAGCAGGCTATGATCCGGCGTTATTCACTAGC	Tb927.10.4850_rev	GGGGACCACTTTGTACAAGAAAGCTGGGTTCACTGTAAACCGTATGCCTTCTTTAC
7_hypothetical	D15	Tb927.11.1570_for	GGGGACAAGTTTGTACAAAAAAGCAGGCTATGAATAATGGAAGTGCTATGGAGGAGG	Tb927.11.1570_rev	GGGGACCACTTTGTACAAGAAAGCTGGGTTCAGGTCTTTCCGAAAGTGCG
7_hypothetical	D16	Tb927.5.1290_for	GGGGACAAGTTTGTACAAAAAAGCAGGCTATGTCGTGCGACACTGTCAC	Tb927.5.1290_rev	GGGGACCACTTTGTACAAGAAAGCTGGGTTCACCTGCGGTGAGAGTGAA
7_hypothetical	D17	Tb927.8.5940_for	GGGGACAAGTTTGTACAAAAAAGCAGGCTATGGACAAAGCAAGGCCGTTT	Tb927.8.5940_rev	GGGGACCACTTTGTACAAGAAAGCTGGGTTCAGCTACCTCCGTGAGGGG
7_hypothetical	D18	Tb927.7.3710_for	GGGGACAAGTTTGTACAAAAAAGCAGGCTATGCACGACGGTGCGGGG	Tb927.7.3710_rev	GGGGACCACTTTGTACAAGAAAGCTGGGTTTACGAAGGTACATCCAAAAACAACCT
7_hypothetical	D19	Tb927.11.14800_for	GGGGACAAGTTTGTACAAAAAAGCAGGCTATGCCCTCAGAATCTTTGGCCG	Tb927.11.14800_rev	GGGGACCACTTTGTACAAGAAAGCTGGGTCTATCGCTGACGCAGATTGCA
7_hypothetical	D20	Tb927.11.6820_for	GGGGACAAGTTTGTACAAAAAAGCAGGCTATGACTTTTGTTGTTATGTGTGACGGC	Tb927.11.6820_rev	GGGGACCACTTTGTACAAGAAAGCTGGGTTCATAGAATTGACGTCTCCTTACTCGG
7_hypothetical	D21	Tb927.11.2750_for	GGGGACAAGTTTGTACAAAAAAGCAGGCTATGTTTCCCTTTGATGAAAGGTCACC	Tb927.11.2750_rev	GGGGACCACTTTGTACAAGAAAGCTGGGTTCACATTACAGTACATCGAGGGCA
7_hypothetical	D22	Tb927.11.10260_for	GGGGACAAGTTTGTACAAAAAAGCAGGCTATGTGTGACGAGTTCTTTGGAGAC	Tb927.11.10260_rev	GGGGACCACTTTGTACAAGAAAGCTGGGTTTATAAATCGCGGCATGTAACTCTAATCG
7_hypothetical	D23	Tb927.11.9250_for	GGGGACAAGTTTGTACAAAAAAGCAGGCTATGCTGCCTCGCACAGCA	Tb927.11.9250_rev	GGGGACCACTTTGTACAAGAAAGCTGGGTCTACGGTTTTGATAGAGCTCCATTAGC
7_hypothetical	D24	Tb927.10.2180_for	GGGGACAAGTTTGTACAAAAAAGCAGGCTATGTCTTGCGGTGGCGCA	Tb927.10.2180_rev	GGGGACCACTTTGTACAAGAAAGCTGGGTTCATTCGACTACCAACGCGC
7_hypothetical	E1	Tb927.3.1830_for	GGGGACAAGTTTGTACAAAAAAGCAGGCTATGGAGCAGCCGAGCGCT	Tb927.3.1830_rev	GGGGACCACTTTGTACAAGAAAGCTGGGTTTACTCCAATTGTCGCACATGAACG
7_hypothetical	E2	Tb927.3.2360_for	GGGGACAAGTTTGTACAAAAAAGCAGGCTATGATTCACTTCGTCTTACTATGCTATCTC	Tb927.3.2360_rev	GGGGACCACTTTGTACAAGAAAGCTGGGTCTAGGTTGAACAAAAAAAATAAGCGGTAAG
7_hypothetical	E3	Tb927.11.4320_for	GGGGACAAGTTTGTACAAAAAAGCAGGCTATGGAGAAAATTGTTCGCACTGTATACA	Tb927.11.4320_rev	GGGGACCACTTTGTACAAGAAAGCTGGGTCTAAGCATCTGGTGGTGCATGC
7_hypothetical	E4	Tb927.9.11140_for	GGGGACAAGTTTGTACAAAAAAGCAGGCTATGAGTTATTTGGTCGAAGAATTGCCT	Tb927.9.11140_rev	GGGGACCACTTTGTACAAGAAAGCTGGGTCTACATATTATCGGACTGGTTGATTGTGC
7_hypothetical	E5	Tb927.5.2210_for	GGGGACAAGTTTGTACAAAAAAGCAGGCTATGGCATCAGACAATGAGTCCACT	Tb927.5.2210_rev	GGGGACCACTTTGTACAAGAAAGCTGGGTTCATCGTCTTCGCCGGGA
7_hypothetical	E6	Tb927.9.8670_for	GGGGACAAGTTTGTACAAAAAAGCAGGCTATGGTTGTACGTACTTTAACTGGCAA	Tb927.9.8670_rev	GGGGACCACTTTGTACAAGAAAGCTGGGTTCAAACCCGGTCCCAGATAACT
7_hypothetical	E8	Tb927.10.9020_for	GGGGACAAGTTTGTACAAAAAAGCAGGCTATGATGGGTGATGCTGGTAGTTTCT	Tb927.10.9020_rev	GGGGACCACTTTGTACAAGAAAGCTGGGTCTACGAGTCTGAAAGATTTCCTTCTTGT
7_hypothetical	E10	Tb927.11.15050_for	GGGGACAAGTTTGTACAAAAAAGCAGGCTATGATAGGAGAAGCTGTTCGTCTGT	Tb927.11.15050_rev	GGGGACCACTTTGTACAAGAAAGCTGGGTCTAAACTGCGGTGACGGAACC
7_hypothetical	E11	Tb927.8.1980_for	GGGGACAAGTTTGTACAAAAAAGCAGGCTATGCAAAATAAGTCTTGGGCCCTG	Tb927.8.1980_rev	GGGGACCACTTTGTACAAGAAAGCTGGGTTTATACGTCATTTACTATTAACTCCATTAT
7_hypothetical	E12	Tb927.8.1450_for	GGGGACAAGTTTGTACAAAAAAGCAGGCTATGATCCGCTGGACTGTAAATCTCG	Tb927.8.1450_rev	GGGGACCACTTTGTACAAGAAAGCTGGGTTCACTCCGAACCGTCAGAGTG
7_hypothetical	E13	Tb927.8.2760_for	GGGGACAAGTTTGTACAAAAAAGCAGGCTATGACGAGCAAGCATGTGGTG	Tb927.8.2760_rev	GGGGACCACTTTGTACAAGAAAGCTGGGTTTAAACAATGGCGCTGAAGTCAGT
7_hypothetical	E14	Tb927.10.410_for	GGGGACAAGTTTGTACAAAAAAGCAGGCTATGACTTCCGTGGGGTTCCC	Tb927.10.410_rev	GGGGACCACTTTGTACAAGAAAGCTGGGTCTACATTCGCACAGAGTGACTGC
7_hypothetical	E15	Tb927.5.2270_for	GGGGACAAGTTTGTACAAAAAAGCAGGCTATGAACACCGCGTCAACTGTT	Tb927.5.2270_rev	GGGGACCACTTTGTACAAGAAAGCTGGGTCTACCGAACACTGACAATCTTTGATAATTT
7_hypothetical	E16	Tb927.11.8930_for	GGGGACAAGTTTGTACAAAAAAGCAGGCTATGGATTTCCGCCTCCCCC	Tb927.11.8930_rev	GGGGACCACTTTGTACAAGAAAGCTGGGTTTAAAGCTCCTGTGACCGCTGT
7_hypothetical	E17	Tb927.6.2840_for	GGGGACAAGTTTGTACAAAAAAGCAGGCTATGGTCAAACTTGACGTCTCACTC	Tb927.6.2840_rev	GGGGACCACTTTGTACAAGAAAGCTGGGTTTAGTCAAAGAGTCCTTCATTTTCCGC
7_hypothetical	E18	Tb927.8.5760_for	GGGGACAAGTTTGTACAAAAAAGCAGGCTATGAACACACCACTGCATGCC	Tb927.8.5760_rev	GGGGACCACTTTGTACAAGAAAGCTGGGTTCACCCCCCACCGTACCG
7_hypothetical	E19	Tb927.3.1390_for	GGGGACAAGTTTGTACAAAAAAGCAGGCTATGTTGAACTGGTGGCTCATAGTACT	Tb927.3.1390_rev	GGGGACCACTTTGTACAAGAAAGCTGGGTTTATGTTGAATAGTTTGGCTTCTTGGGT
7_hypothetical	E20	Tb927.3.1930_for	GGGGACAAGTTTGTACAAAAAAGCAGGCTATGCTTTTTGACCTTGCACCGC	Tb927.3.1930_rev	GGGGACCACTTTGTACAAGAAAGCTGGGTTCACGCCCATGTCTTAATGGC
7_hypothetical	E21	Tb927.5.2740_for	GGGGACAAGTTTGTACAAAAAAGCAGGCTATGGATGAACTATCGGATATTTATGACTCC	Tb927.5.2740_rev	GGGGACCACTTTGTACAAGAAAGCTGGGTTTATTTCGGATATGCCCCATCTGC
7_hypothetical	E22	Tb927.10.3910_for	GGGGACAAGTTTGTACAAAAAAGCAGGCTATGAAGCGATCAGATGACGTCAGT	Tb927.10.3910_rev	GGGGACCACTTTGTACAAGAAAGCTGGGTTCACAATAAGTGACTGCTGAAATTCTTTCG
7_hypothetical	E23	Tb927.9.6360_for	GGGGACAAGTTTGTACAAAAAAGCAGGCTATGAGCAGCACAGACAATGATATCG	Tb927.9.6360_rev	GGGGACCACTTTGTACAAGAAAGCTGGGTTCAAGTTCGTGATTTCGCTTTTGC
7_hypothetical	E24	Tb927.6.2570_for	GGGGACAAGTTTGTACAAAAAAGCAGGCTATGCTCAGTGGAACTACTTTTGGAG	Tb927.6.2570_rev	GGGGACCACTTTGTACAAGAAAGCTGGGTTCAAATGACCGACCGCCAAC
7_hypothetical	F2	Tb927.5.4100_for	GGGGACAAGTTTGTACAAAAAAGCAGGCTATGCAGAGGCCCCTGACG	Tb927.5.4100_rev	GGGGACCACTTTGTACAAGAAAGCTGGGTTTAAAGGCTCAGTTTGAAAAAGCGAAC
7_hypothetical	F3	Tb927.5.2870_for	GGGGACAAGTTTGTACAAAAAAGCAGGCTATGCCGCACAAGGGGTGC	Tb927.5.2870_rev	GGGGACCACTTTGTACAAGAAAGCTGGGTTTAAACCCCCCAAACGTGTTCC
7_hypothetical	F4	Tb927.10.3740_for	GGGGACAAGTTTGTACAAAAAAGCAGGCTATGCCGCGGTTACATATTTGGAT	Tb927.10.3740_rev	GGGGACCACTTTGTACAAGAAAGCTGGGTTCACAACACGTTTGTAGGGGTATG
7_hypothetical	F5	Tb927.11.14090_for	GGGGACAAGTTTGTACAAAAAAGCAGGCTATGCAAGGCGCATACGGAG	Tb927.11.14090_rev	GGGGACCACTTTGTACAAGAAAGCTGGGTTTATGCTGAACCATTTTCCCCAGC
7_hypothetical	F6	Tb927.11.7650_for	GGGGACAAGTTTGTACAAAAAAGCAGGCTATGGAGCGCGAGCAACTG	Tb927.11.7650_rev	GGGGACCACTTTGTACAAGAAAGCTGGGTTCACGTGCTAGTCACTATAAGGGAC
7_hypothetical	F7	Tb927.6.3170_for	GGGGACAAGTTTGTACAAAAAAGCAGGCTATGACGTCCAGTCGTGCGG	Tb927.6.3170_rev	GGGGACCACTTTGTACAAGAAAGCTGGGTTTACTCCTTCTTCACCGATATAGTCGAG

7_hypothetical	F8	Tb927.10.1180_for	GGGGACAAGTTTGTACAAAAAAGCAGGCTATGCCCCTGACCTGTCTTGG	Tb927.10.1180_rev	GGGGACCACTTTGTACAAGAAAGCTGGGTTTAAAAACAAAAGGATCCGTCATCCTGG
7_hypothetical	F9	Tb927.4.3660_for	GGGGACAAGTTTGTACAAAAAAGCAGGCTATGAGATGTTTCGGGGCCTGC	Tb927.4.3660_rev	GGGGACCACTTTGTACAAGAAAGCTGGGTTCACGGAGTGGTGACGACG
7_hypothetical	F10	Tb927.3.1990_for	GGGGACAAGTTTGTACAAAAAAGCAGGCTATGGCTAGTGTTGGGCCTGT	Tb927.3.1990_rev	GGGGACCACTTTGTACAAGAAAGCTGGGTTTACAAGATGCCATTACTCATCCACTC
7_hypothetical	F11	Tb927.10.2310_for	GGGGACAAGTTTGTACAAAAAAGCAGGCTATGAACCGCAACGCGGCG	Tb927.10.2310_rev	GGGGACCACTTTGTACAAGAAAGCTGGGTTTAGTCCTCCATATCAATCTCTGGGATTT
7_hypothetical	F12	Tb927.4.1940_for	GGGGACAAGTTTGTACAAAAAAGCAGGCTATGGCGGACAACTCGTCTGC	Tb927.4.1940_rev	GGGGACCACTTTGTACAAGAAAGCTGGGTTCAGGTCTTCACGGGTACCCA
7_hypothetical	F13	Tb927.10.15470_for	GGGGACAAGTTTGTACAAAAAAGCAGGCTATGCGGGTCGATGTAGAAGCC	Tb927.10.15470_rev	GGGGACCACTTTGTACAAGAAAGCTGGGTTCATTGGACCATGTATACTCTCATATCCTT
7_hypothetical	F14	Tb927.10.10310_for	GGGGACAAGTTTGTACAAAAAAGCAGGCTATGCTGCGATCCTGTCGAAC	Tb927.10.10310_rev	GGGGACCACTTTGTACAAGAAAGCTGGGTCTACACACAGCGGCCAGAAC
7_hypothetical	F15	Tb927.8.1530_for	GGGGACAAGTTTGTACAAAAAAGCAGGCTATGCTGTTGAAGCTTGGCGC	Tb927.8.1530_rev	GGGGACCACTTTGTACAAGAAAGCTGGGTTTAATCTGCGGCGGCACAC
7_hypothetical	F16	Tb927.10.15450_for	GGGGACAAGTTTGTACAAAAAAGCAGGCTATGTACGTGGGTCCTTGGGAG	Tb927.10.15450_rev	GGGGACCACTTTGTACAAGAAAGCTGGGTTCAAACAATGCTATCAGGGTCCAAC
7_hypothetical	F17	Tb927.9.9300_for	GGGGACAAGTTTGTACAAAAAAGCAGGCTATGGCAACGGCACACTGTAC	Tb927.9.9300_rev	GGGGACCACTTTGTACAAGAAAGCTGGGTCTATCTCGTTCCTGGCCTAGTCTG
7_hypothetical	F18	Tb927.3.670_for	GGGGACAAGTTTGTACAAAAAAGCAGGCTATGCTATCAACGCCCTGTCCT	Tb927.3.670_rev	GGGGACCACTTTGTACAAGAAAGCTGGGTTTAGGTGATGGGAACACCACAACT
7_hypothetical	F19	Tb927.7.3510_for	GGGGACAAGTTTGTACAAAAAAGCAGGCTATGTGGCATTCGTCCCTCCG	Tb927.7.3510_rev	GGGGACCACTTTGTACAAGAAAGCTGGGTTCAGGAGCCATAACGCATGGA
7_hypothetical	F20	Tb927.5.1230_for	GGGGACAAGTTTGTACAAAAAAGCAGGCTATGGCTCACGTTGCAATCCG	Tb927.5.1230_rev	GGGGACCACTTTGTACAAGAAAGCTGGGTCTAGATTGGTGGGAATCGTTGCC
7_hypothetical	F21	Tb927.3.3460_for	GGGGACAAGTTTGTACAAAAAAGCAGGCTATGTTTTGCGTAAGTGCCGCA	Tb927.3.3460_rev	GGGGACCACTTTGTACAAGAAAGCTGGGTCTAAGCATCAGCAGAAGTAATCCTTTTAAG
7_hypothetical	F22	Tb927.10.11070_for	GGGGACAAGTTTGTACAAAAAAGCAGGCTATGGGCTCTCCGCCGCCT	Tb927.10.11070_rev	GGGGACCACTTTGTACAAGAAAGCTGGGTCTATTCGCTTGCATCGCATTTTTCC
7_hypothetical	F23	Tb927.8.4580_for	GGGGACAAGTTTGTACAAAAAAGCAGGCTATGATTGGTATGATGGCGCGTAG	Tb927.8.4580_rev	GGGGACCACTTTGTACAAGAAAGCTGGGTCTATCGCCGGGCCACCTT
7_hypothetical	F24	Tb927.9.2700_for	GGGGACAAGTTTGTACAAAAAAGCAGGCTATGCTTCGGTGGACCGGA	Tb927.9.2700_rev	GGGGACCACTTTGTACAAGAAAGCTGGGTCTACCGTGACCGCTGACGA
7_hypothetical	G1	Tb927.10.6620_for	GGGGACAAGTTTGTACAAAAAAGCAGGCTATGCCTCACTGCCCGCGG	Tb927.10.6620_rev	GGGGACCACTTTGTACAAGAAAGCTGGGTTTAAGGGCATGCCGAAGCAG
7_hypothetical	G2	Tb927.4.4670_for	GGGGACAAGTTTGTACAAAAAAGCAGGCTATGACAACTTCCCCATCCACGC	Tb927.4.4670_rev	GGGGACCACTTTGTACAAGAAAGCTGGGTTCAGCTGAAGGCAGCGGA
7_hypothetical	G3	Tb927.11.5720_for	GGGGACAAGTTTGTACAAAAAAGCAGGCTATGATGAATCGTAACGAGAACTCTGG	Tb927.11.5720_rev	GGGGACCACTTTGTACAAGAAAGCTGGGTTTAGACATTTCCCGTGCCTCGC
7_hypothetical	G4	Tb927.11.9650_for	GGGGACAAGTTTGTACAAAAAAGCAGGCTATGCCCTCAGGTACAGACACTC	Tb927.11.9650_rev	GGGGACCACTTTGTACAAGAAAGCTGGGTTCAAGAAAAGTTCTTCCTCAACTGCC
7_hypothetical	G5	Tb927.10.2610_for	GGGGACAAGTTTGTACAAAAAAGCAGGCTATGACGGAGTTGGTTCCCGA	Tb927.10.2610_rev	GGGGACCACTTTGTACAAGAAAGCTGGGTTTACGCGCATCGAACACTGC
7_hypothetical	G6	Tb927.10.11690_for	GGGGACAAGTTTGTACAAAAAAGCAGGCTATGCAGGCCACTTCTAAATCTACTGG	Tb927.10.11690_rev	GGGGACCACTTTGTACAAGAAAGCTGGGTCTAGCGCTTCCACGACGTC
7_hypothetical	G7	Tb927.10.11910_for	GGGGACAAGTTTGTACAAAAAAGCAGGCTATGGAAGGGGCCGCTGGA	Tb927.10.11910_rev	GGGGACCACTTTGTACAAGAAAGCTGGGTCTAGCACCTCTCGCTTTCACCA
7_hypothetical	G8	Tb927.10.15930_for	GGGGACAAGTTTGTACAAAAAAGCAGGCTATGTTATGGAGCACACCGCCA	Tb927.10.15930_rev	GGGGACCACTTTGTACAAGAAAGCTGGGTTCAAGAACCCGACGCACGT
7_hypothetical	G9	Tb927.9.8330_for	GGGGACAAGTTTGTACAAAAAAGCAGGCTATGTCTCCTTTTGAGCAACAGCG	Tb927.9.8330_rev	GGGGACCACTTTGTACAAGAAAGCTGGGTTCATCTCTTCCGAGCCCCG
7_hypothetical	G10	Tb927.3.5110_for	GGGGACAAGTTTGTACAAAAAAGCAGGCTATGTCGATACAAGGGGACCCAA	Tb927.3.5110_rev	GGGGACCACTTTGTACAAGAAAGCTGGGTTTACCTCACACGCGCCTTTG
7_hypothetical	G11	Tb927.8.6300_for	GGGGACAAGTTTGTACAAAAAAGCAGGCTATGATAGCTGCAACTCAGGTGC	Tb927.8.6300_rev	GGGGACCACTTTGTACAAGAAAGCTGGGTTCATCCACCGAGTATCGGCG
7_hypothetical	G12	Tb927.7.2450_for	GGGGACAAGTTTGTACAAAAAAGCAGGCTATGAGTGGTGATGACGATGACGT	Tb927.7.2450_rev	GGGGACCACTTTGTACAAGAAAGCTGGGTTTAATTTCGTTGACTTACATTTGTAGCGG
7_hypothetical	G13	Tb927.10.11190_for	GGGGACAAGTTTGTACAAAAAAGCAGGCTATGCGTCACGCTGTCACAAA	Tb927.10.11190_rev	GGGGACCACTTTGTACAAGAAAGCTGGGTTTACCCCTTAACGCACAACAACTG
7_hypothetical	G14	Tb927.4.2800_for	GGGGACAAGTTTGTACAAAAAAGCAGGCTATGACCGTGAGCGCAAACG	Tb927.4.2800_rev	GGGGACCACTTTGTACAAGAAAGCTGGGTTTAATTCGATGTCTTCGTTCCTTTACTCTT
7_hypothetical	G15	Tb927.2.6100_for	GGGGACAAGTTTGTACAAAAAAGCAGGCTATGCTGCGGTCCTTGTTGTTAC	Tb927.2.6100_rev	GGGGACCACTTTGTACAAGAAAGCTGGGTTTAAAGTCGAAGAAGTCCGGTATTTGTT
7_hypothetical	G16	Tb927.10.6360_for	GGGGACAAGTTTGTACAAAAAAGCAGGCTATGAGCAGTGAGACTGTTGCG	Tb927.10.6360_rev	GGGGACCACTTTGTACAAGAAAGCTGGGTTTAAACCGGCTCCCCTCTGTG
7_hypothetical	G17	Tb927.4.3970_for	GGGGACAAGTTTGTACAAAAAAGCAGGCTATGTCGGCAGGTTCGGAACC	Tb927.4.3970_rev	GGGGACCACTTTGTACAAGAAAGCTGGGTTTAATTGCTAAAGAACTCAATGATATTTTG
7_hypothetical	G18	Tb927.11.1060_for	GGGGACAAGTTTGTACAAAAAAGCAGGCTATGTATTCTTCAGACAGGGTTCGGA	Tb927.11.1060_rev	GGGGACCACTTTGTACAAGAAAGCTGGGTCTACGATCGGCCCACATCGT
7_hypothetical	G19	Tb927.3.5480_for	GGGGACAAGTTTGTACAAAAAAGCAGGCTATGTTTAGTGCTAAGAGAAAGGATGTGG	Tb927.3.5480_rev	GGGGACCACTTTGTACAAGAAAGCTGGGTTTAAACAACTGGATCGGACCCGT
7_hypothetical	G20	Tb927.7.6690_for	GGGGACAAGTTTGTACAAAAAAGCAGGCTATGAGCCTCTCCAGAAGCCC	Tb927.7.6690_rev	GGGGACCACTTTGTACAAGAAAGCTGGGTTCATGCGGAGCGAAGTGTCC
7_hypothetical	G21	Tb927.9.10690_for	GGGGACAAGTTTGTACAAAAAAGCAGGCTATGAACCTCTTTGGGCTTCGTTC	Tb927.9.10690_rev	GGGGACCACTTTGTACAAGAAAGCTGGGTTCATAGACTGAGGTCACCGTCG
7_hypothetical	G22	Tb927.10.3670_for	GGGGACAAGTTTGTACAAAAAAGCAGGCTATGAATGAAGATGATATTCTCTTTGCTCAA	Tb927.10.3670_rev	GGGGACCACTTTGTACAAGAAAGCTGGGTTCAGAACGGAAGCTGCAAAGAC
7_hypothetical	G23	Tb927.10.3920_for	GGGGACAAGTTTGTACAAAAAAGCAGGCTATGGTCGGTGGGTTCACTGA	Tb927.10.3920_rev	GGGGACCACTTTGTACAAGAAAGCTGGGTCTACCTTCCGACTAACCACGTCA
7_hypothetical	G24	Tb927.8.2950_for	GGGGACAAGTTTGTACAAAAAAGCAGGCTATGTTGTCTCGTTCATCTCAGGATCC	Tb927.8.2950_rev	GGGGACCACTTTGTACAAGAAAGCTGGGTCTACATGTCCGTGAGATCCGTTTC
7_hypothetical	H1	Tb927.9.9950_for	GGGGACAAGTTTGTACAAAAAAGCAGGCTATGGAGGAAGTCTCCGGTTCC	Tb927.9.9950_rev	GGGGACCACTTTGTACAAGAAAGCTGGGTTTACACACAACCAAAGTCACTTTTGTTT
7_hypothetical	H2	Tb927.10.6720_for	GGGGACAAGTTTGTACAAAAAAGCAGGCTATGATGCAAGGATTTCCCTCCTTAGG	Tb927.10.6720_rev	GGGGACCACTTTGTACAAGAAAGCTGGGTTTACACACGTGGAGGTGCTGC
7_hypothetical	H3	Tb927.10.8880_for	GGGGACAAGTTTGTACAAAAAAGCAGGCTATGTACTCGGATAGCGAGAGCAT	Tb927.10.8880_rev	GGGGACCACTTTGTACAAGAAAGCTGGGTTTAAAGATACCTCTGCACCACGC
7_hypothetical	H4	Tb927.7.5240_for	GGGGACAAGTTTGTACAAAAAAGCAGGCTATGGCCAACTTTAATAAAGTCTCTAGGGC	Tb927.7.5240_rev	GGGGACCACTTTGTACAAGAAAGCTGGGTTTAGCGACTATTTCGCCCCTCC
7_hypothetical	H5	Tb927.10.6730_for	GGGGACAAGTTTGTACAAAAAAGCAGGCTATGAACAAGCAATACGGAACAGAAACA	Tb927.10.6730_rev	GGGGACCACTTTGTACAAGAAAGCTGGGTTTACACACGTGGAGGTGCTGC
7_hypothetical	H6	Tb927.11.10200_for	GGGGACAAGTTTGTACAAAAAAGCAGGCTATGAAGTCCAAGGATGTGGAGGAT	Tb927.11.10200_rev	GGGGACCACTTTGTACAAGAAAGCTGGGTCTAGAAAACATTTGTAGCGTAACCTGTG
7_hypothetical	H7	Tb927.10.6740_for	GGGGACAAGTTTGTACAAAAAAGCAGGCTATGAACAAGCAACACGGAACAGA	Tb927.10.6740_rev	GGGGACCACTTTGTACAAGAAAGCTGGGTTTACACACGTGGAGGTGCTGC
7_hypothetical	H8	Tb927.10.11640_for	GGGGACAAGTTTGTACAAAAAAGCAGGCTATGCATGACCAACGAAAGGTACG	Tb927.10.11640_rev	GGGGACCACTTTGTACAAGAAAGCTGGGTTTACGCTGTGGTAACCTCAGAAAGG
7_hypothetical	H9	Tb927.6.1740_for	GGGGACAAGTTTGTACAAAAAAGCAGGCTATGAAACGGTACTACTTACCTGCGG	Tb927.6.1740_rev	GGGGACCACTTTGTACAAGAAAGCTGGGTTCAGCCCAAGTGAGTTGCGC
7_hypothetical	H10	Tb927.3.3510_for	GGGGACAAGTTTGTACAAAAAAGCAGGCTATGCTGCCCTCAGCGCAA	Tb927.3.3510_rev	GGGGACCACTTTGTACAAGAAAGCTGGGTTCACATCAGCATGTAAACAAGAACTGC
7_hypothetical	H11	Tb927.6.2470_for	GGGGACAAGTTTGTACAAAAAAGCAGGCTATGTTTCGTCTAGACGGCTGCC	Tb927.6.2470_rev	GGGGACCACTTTGTACAAGAAAGCTGGGTCTAGCACGCTGCTCGCAT
7_hypothetical	H12	Tb927.4.730_for	GGGGACAAGTTTGTACAAAAAAGCAGGCTATGGCGACGGTGGCTAATGA	Tb927.4.730_rev	GGGGACCACTTTGTACAAGAAAGCTGGGTTCAGCGGGGATCGAAGGTG
7_hypothetical	H13	Tb927.6.1100_for	GGGGACAAGTTTGTACAAAAAAGCAGGCTATGTGCGCGCCTTCTTGTG	Tb927.6.1100_rev	GGGGACCACTTTGTACAAGAAAGCTGGGTCTACTCGTGCGCCTCTTCATAGT
7_hypothetical	H14	Tb927.3.4470_for	GGGGACAAGTTTGTACAAAAAAGCAGGCTATGTTGGCACTATCACAGGGGT	Tb927.3.4470_rev	GGGGACCACTTTGTACAAGAAAGCTGGGTCTACCGCTGCTCGCGATTT
7_hypothetical	H15	Tb927.5.2460_for	GGGGACAAGTTTGTACAAAAAAGCAGGCTATGCCACTCCTTCTCCGTTTGT	Tb927.5.2460_rev	GGGGACCACTTTGTACAAGAAAGCTGGGTTCACAATTCGTCAAGTTCCTGTAAAAGG
7_hypothetical	H16	Tb927.6.3180_for	GGGGACAAGTTTGTACAAAAAAGCAGGCTATGGTCTCCAACGACCCCC	Tb927.6.3180_rev	GGGGACCACTTTGTACAAGAAAGCTGGGTTCACAGGCACGAACGTCGC
7_hypothetical	H17	Tb927.11.12720_for	GGGGACAAGTTTGTACAAAAAAGCAGGCTATGTGTGGTGATTCCTTTGATACTTCTC	Tb927.11.12720_rev	GGGGACCACTTTGTACAAGAAAGCTGGGTTTACATCCGTGGGGAGGAGC
7_hypothetical	H18	Tb927.3.1020_for	GGGGACAAGTTTGTACAAAAAAGCAGGCTATGTTTTCGGGTTCTGAAGGCAG	Tb927.3.1020_rev	GGGGACCACTTTGTACAAGAAAGCTGGGTTCACTTAGTTTCGTATTGCTCGGTG
7_hypothetical	H19	Tb927.11.11000_for	GGGGACAAGTTTGTACAAAAAAGCAGGCTATGACTCTTGAACACAATGATGAGTTCG	Tb927.11.11000_rev	GGGGACCACTTTGTACAAGAAAGCTGGGTTCAGTTAGAGAAGTCACGCTCATGC
7_hypothetical	H20	Tb927.7.6620_for	GGGGACAAGTTTGTACAAAAAAGCAGGCTATGACGGAAGCGGCTCCA	Tb927.7.6620_rev	GGGGACCACTTTGTACAAGAAAGCTGGGTTTATTTCCCGCCCTCTCCATTCC
7_hypothetical	H21	Tb927.5.560_for	GGGGACAAGTTTGTACAAAAAAGCAGGCTATGAGGAAATATCATCTATCCCCATTGGT	Tb927.5.560_rev	GGGGACCACTTTGTACAAGAAAGCTGGGTTTACCATTTATTCCCCGCACCCG
7_hypothetical	H22	Tb927.7.950_for	GGGGACAAGTTTGTACAAAAAAGCAGGCTATGAATCCGAAGCTGTTCTCGAC	Tb927.7.950_rev	GGGGACCACTTTGTACAAGAAAGCTGGGTCTACCCCCTCAACTTCCCCAA
7_hypothetical	H23	Tb927.7.1060_for	GGGGACAAGTTTGTACAAAAAAGCAGGCTATGTTTCGCGTTGTGTCGTTTG	Tb927.7.1060_rev	GGGGACCACTTTGTACAAGAAAGCTGGGTCTACGATGAGTCTGGGTTGCTGT
7_hypothetical	H24	Tb927.9.12380_for	GGGGACAAGTTTGTACAAAAAAGCAGGCTATGTATGCTGATGAAGAGGAGAGTCG	Tb927.9.12380_rev	GGGGACCACTTTGTACAAGAAAGCTGGGTTTAACCCGCCAAAGCTACCGA
7_hypothetical	I1	Tb927.8.7970_for	GGGGACAAGTTTGTACAAAAAAGCAGGCTATGGAAGACGCAACGAAAGAAACA	Tb927.8.7970_rev	GGGGACCACTTTGTACAAGAAAGCTGGGTTCATTCAATCATCTCCCCGGATGC
7_hypothetical	I2	Tb927.9.7040_for	GGGGACAAGTTTGTACAAAAAAGCAGGCTATGCACCGTACCAGTTTACGC	Tb927.9.7040_rev	GGGGACCACTTTGTACAAGAAAGCTGGGTTTAGACACGCCGAGATCGGC
7_hypothetical	I3	Tb927.11.1850_for	GGGGACAAGTTTGTACAAAAAAGCAGGCTATGGGCGCTGCGCGGAGT	Tb927.11.1850_rev	GGGGACCACTTTGTACAAGAAAGCTGGGTCTAGCCTGATTTAGGTGGATCCTCA
7_hypothetical	I4	Tb927.10.1700_for	GGGGACAAGTTTGTACAAAAAAGCAGGCTATGTCGGTAGTGGCGTCCTT	Tb927.10.1700_rev	GGGGACCACTTTGTACAAGAAAGCTGGGTTTAATGCTGCGGGCTCTTTCC
7_hypothetical	I5	Tb927.9.2940_for	GGGGACAAGTTTGTACAAAAAAGCAGGCTATGATCGACAAATCGCCCCG	Tb927.9.2940_rev	GGGGACCACTTTGTACAAGAAAGCTGGGTTCAACGATGTTGGGGAATGAGGT
7_hypothetical	I6	Tb927.11.2150_for	GGGGACAAGTTTGTACAAAAAAGCAGGCTATGCAGGACCTCAACTGGGC	Tb927.11.2150_rev	GGGGACCACTTTGTACAAGAAAGCTGGGTTTACTGTGGTCTGGGTGTCGT
7_hypothetical	I7	Tb927.9.4660_for	GGGGACAAGTTTGTACAAAAAAGCAGGCTATGATGTCAAAAGCGTCGTATATGATGC	Tb927.9.4660_rev	GGGGACCACTTTGTACAAGAAAGCTGGGTCTAAGATAGTGGAGGCATACCCCAC
7_hypothetical	I8	Tb927.10.5640_for	GGGGACAAGTTTGTACAAAAAAGCAGGCTATGGAAGACGATGCTGATGCCT	Tb927.10.5640_rev	GGGGACCACTTTGTACAAGAAAGCTGGGTTTACAGCGGAACCAAACGATTACC
7_hypothetical	I9	Tb927.10.2820_for	GGGGACAAGTTTGTACAAAAAAGCAGGCTATGCCAGCCACCAGGGAA	Tb927.10.2820_rev	GGGGACCACTTTGTACAAGAAAGCTGGGTTTACAGCTTCAGGAGACGTGTTTC
7_hypothetical	I10	Tb927.5.3660_for	GGGGACAAGTTTGTACAAAAAAGCAGGCTATGAACGCAGCAGTTGTGACA	Tb927.5.3660_rev	GGGGACCACTTTGTACAAGAAAGCTGGGTTCAAAACAAATTTTTCAGTCGAAGGATGA
7_hypothetical	I11	Tb927.4.1460_for	GGGGACAAGTTTGTACAAAAAAGCAGGCTATGCTTCGGTGTCGAGTAGCG	Tb927.4.1460_rev	GGGGACCACTTTGTACAAGAAAGCTGGGTCTACGCTCTCCCGTCTCTTGT
7_hypothetical	I12	Tb927.11.7250_for	GGGGACAAGTTTGTACAAAAAAGCAGGCTATGCGCCGTTTCTACTGCATT	Tb927.11.7250_rev	GGGGACCACTTTGTACAAGAAAGCTGGGTTTATTTGAGCCCCTCCAGGACA
7_hypothetical	I13	Tb927.3.4540_for	GGGGACAAGTTTGTACAAAAAAGCAGGCTATGAGTGATGATGCGTCTCAAAACT	Tb927.3.4540_rev	GGGGACCACTTTGTACAAGAAAGCTGGGTTTATGTCAGTGGTTTCTTCCATACTTTACT
7_hypothetical	I14	Tb927.9.1510_for	GGGGACAAGTTTGTACAAAAAAGCAGGCTATGCCGGCCAAACCCACT	Tb927.9.1510_rev	GGGGACCACTTTGTACAAGAAAGCTGGGTTTAGTAGTTGTTTCCATTTTCCTCTTTTGT
7_hypothetical	I15	Tb927.7.3070_for	GGGGACAAGTTTGTACAAAAAAGCAGGCTATGCTAAGGACAAATGGTAAGATTCTCAA	Tb927.7.3070_rev	GGGGACCACTTTGTACAAGAAAGCTGGGTTCACTTACTACTTACAGGCTCCTTGC
7_hypothetical	I16	Tb927.10.3950_for	GGGGACAAGTTTGTACAAAAAAGCAGGCTATGGCACTTTCTTTTCCCTCTTCATC	Tb927.10.3950_rev	GGGGACCACTTTGTACAAGAAAGCTGGGTTTAAAAAGCCATGAAAAACTCGTGGTC
7_hypothetical	I17	Tb927.9.8240_for	GGGGACAAGTTTGTACAAAAAAGCAGGCTATGTCCGGGACGCCGACA	Tb927.9.8240_rev	GGGGACCACTTTGTACAAGAAAGCTGGGTCTACGTCCACACTAATAAATCACGCA
7_hypothetical	I18	Tb927.11.11310_for	GGGGACAAGTTTGTACAAAAAAGCAGGCTATGAACTCTGAAGAATGCACGAAGC	Tb927.11.11310_rev	GGGGACCACTTTGTACAAGAAAGCTGGGTTTAAAATCGTGTATGCGCATCGTG
7_hypothetical	I19	Tb927.6.1250_for	GGGGACAAGTTTGTACAAAAAAGCAGGCTATGCGCTCCAAGACCCTAAAGA	Tb927.6.1250_rev	GGGGACCACTTTGTACAAGAAAGCTGGGTCTACATTACCCCCACGGCACC
7_hypothetical	I20	Tb927.7.5340_for	GGGGACAAGTTTGTACAAAAAAGCAGGCTATGGGAGGAGGTTCATCCGTTG	Tb927.7.5340_rev	GGGGACCACTTTGTACAAGAAAGCTGGGTTTAGTTCAATTGGTCATCAAACATCATGAA
7_hypothetical	I21	Tb927.10.3610_for	GGGGACAAGTTTGTACAAAAAAGCAGGCTATGGCGGTTGGTGCAGGC	Tb927.10.3610_rev	GGGGACCACTTTGTACAAGAAAGCTGGGTTTAGTTCGTTTCGTTAAGGAGGCG
7_hypothetical	I22	Tb927.11.10840_for	GGGGACAAGTTTGTACAAAAAAGCAGGCTATGAATCCGCCGACTACTCAGG	Tb927.11.10840_rev	GGGGACCACTTTGTACAAGAAAGCTGGGTTCAATCTCTTCCTCTTTTCTTATTCTCTTC
7_hypothetical	I23	Tb927.3.4950_for	GGGGACAAGTTTGTACAAAAAAGCAGGCTATGCGGCGTGCATTGTACC	Tb927.3.4950_rev	GGGGACCACTTTGTACAAGAAAGCTGGGTTTATGGAGGTCCAACAGGGCG
7_hypothetical	I24	Tb927.7.550_for	GGGGACAAGTTTGTACAAAAAAGCAGGCTATGGGTGGCGGTGGAAGC	Tb927.7.550_rev	GGGGACCACTTTGTACAAGAAAGCTGGGTTTACATTCTGTCAATGTAATCATAATTCCA
7_hypothetical	J1	Tb927.10.6180_for	GGGGACAAGTTTGTACAAAAAAGCAGGCTATGAACCCGTTTGTTTTTTCCCTTTAC	Tb927.10.6180_rev	GGGGACCACTTTGTACAAGAAAGCTGGGTTCAGTTGTTCGTTCCGTATCCAC
7_hypothetical	J2	Tb927.6.4230_for	GGGGACAAGTTTGTACAAAAAAGCAGGCTATGAGCCGCTGTAACGTTGC	Tb927.6.4230_rev	GGGGACCACTTTGTACAAGAAAGCTGGGTTCACGACCGGTACCTTTCTAAGG
7_hypothetical	J3	Tb927.11.14740_for	GGGGACAAGTTTGTACAAAAAAGCAGGCTATGGCCCAACCCAGGAAGAA	Tb927.11.14740_rev	GGGGACCACTTTGTACAAGAAAGCTGGGTTCAGCTAATAACCTCAACAGAAGAACATG
7_hypothetical	J4	Tb927.2.5010_for	GGGGACAAGTTTGTACAAAAAAGCAGGCTATGGACGCCGTACACGAGG	Tb927.2.5010_rev	GGGGACCACTTTGTACAAGAAAGCTGGGTTTATACTACATGTGCGCTCTGGTCA
7_hypothetical	J5	Tb927.5.2350_for	GGGGACAAGTTTGTACAAAAAAGCAGGCTATGGAAAGAGGGGGAAAAATTGCG	Tb927.5.2350_rev	GGGGACCACTTTGTACAAGAAAGCTGGGTCTAATCTTCAGAAGCCATATGTTTGATGGA
7_hypothetical	J6	Tb927.7.4100_for	GGGGACAAGTTTGTACAAAAAAGCAGGCTATGGCAACTGAGGTGAAGCCA	Tb927.7.4100_rev	GGGGACCACTTTGTACAAGAAAGCTGGGTCTATTGCCTTCTCTTCATGTCCAAAAGA
7_hypothetical	J7	Tb927.11.15980_for	GGGGACAAGTTTGTACAAAAAAGCAGGCTATGTCCCAACGTCACGCCG	Tb927.11.15980_rev	GGGGACCACTTTGTACAAGAAAGCTGGGTTCACATCACCGAACAGCATTTAGAC
7_hypothetical	J8	Tb927.6.3880_for	GGGGACAAGTTTGTACAAAAAAGCAGGCTATGACAATACACGAGTCGTGGC	Tb927.6.3880_rev	GGGGACCACTTTGTACAAGAAAGCTGGGTTCATTTTGAACCATATATCTCTTTTAATTT
7_hypothetical	J9	Tb927.8.1800_for	GGGGACAAGTTTGTACAAAAAAGCAGGCTATGCAAGCGCGGCTGCTG	Tb927.8.1800_rev	GGGGACCACTTTGTACAAGAAAGCTGGGTCTAACGCCGTAACTTCCCCAC
7_hypothetical	J10	Tb927.4.4900_for	GGGGACAAGTTTGTACAAAAAAGCAGGCTATGGCGCAAGTCACCCAAAG	Tb927.4.4900_rev	GGGGACCACTTTGTACAAGAAAGCTGGGTTTATACCCCGTAGCAGAAACCGC
7_hypothetical	J11	Tb927.11.12290_for	GGGGACAAGTTTGTACAAAAAAGCAGGCTATGATTGAACTCCTCCTTGAGGTTGC	Tb927.11.12290_rev	GGGGACCACTTTGTACAAGAAAGCTGGGTTTAGCAACAGAGAGGCAGAGATGC
7_hypothetical	J13	Tb927.7.3910_for	GGGGACAAGTTTGTACAAAAAAGCAGGCTATGTTTTTAAAGAAGACAATGTCGGGTG	Tb927.7.3910_rev	GGGGACCACTTTGTACAAGAAAGCTGGGTTTACTTAAACCCCTTTCCCTCCTGC
7_hypothetical	J14	Tb927.9.7480_for	GGGGACAAGTTTGTACAAAAAAGCAGGCTATGAACCAACCGACAACCACAC	Tb927.9.7480_rev	GGGGACCACTTTGTACAAGAAAGCTGGGTCTACTTGGCACGTAGGTGCTG
7_hypothetical	J15	Tb927.4.4520_for	GGGGACAAGTTTGTACAAAAAAGCAGGCTATGCTCTTCCATCCCACTGTATGC	Tb927.4.4520_rev	GGGGACCACTTTGTACAAGAAAGCTGGGTTCAATTGTCATCAAAGCTACCAAATGGC
7_hypothetical	J16	Tb927.8.7820_for	GGGGACAAGTTTGTACAAAAAAGCAGGCTATGCTTTTTCACACGACACCATGC	Tb927.8.7820_rev	GGGGACCACTTTGTACAAGAAAGCTGGGTTCATTTCCCATCAAATCCACCAAAGA
7_hypothetical	J17	Tb927.11.13540_for	GGGGACAAGTTTGTACAAAAAAGCAGGCTATGTTTTCGAGGACTGAAGTTGATGC	Tb927.11.13540_rev	GGGGACCACTTTGTACAAGAAAGCTGGGTCTACTGTGTGAAGATACTAAACACCTCC
7_hypothetical	J18	Tb927.10.6750_for	GGGGACAAGTTTGTACAAAAAAGCAGGCTATGACGCAAAAGGGGTACGTC	Tb927.10.6750_rev	GGGGACCACTTTGTACAAGAAAGCTGGGTTTACACACGTGGAGGTGCTGC
7_hypothetical	J19	Tb927.10.11400_for	GGGGACAAGTTTGTACAAAAAAGCAGGCTATGAGTGACAGTGACACTGATTCGT	Tb927.10.11400_rev	GGGGACCACTTTGTACAAGAAAGCTGGGTCTACAATCTCAGCTCATCTCCGTCG
7_hypothetical	J20	Tb927.6.1910_for	GGGGACAAGTTTGTACAAAAAAGCAGGCTATGACGAAGGAGGAGGTTTTATCCT	Tb927.6.1910_rev	GGGGACCACTTTGTACAAGAAAGCTGGGTTTAGGGCGTCTTTTCCAAACGC
7_hypothetical	J21	Tb927.10.2280_for	GGGGACAAGTTTGTACAAAAAAGCAGGCTATGGAATCGCTTATAAAAAACATACAGAGT	Tb927.10.2280_rev	GGGGACCACTTTGTACAAGAAAGCTGGGTTCATATCCCTTTACTACCCCGTTTACC
7_hypothetical	J22	Tb927.11.4180_for	GGGGACAAGTTTGTACAAAAAAGCAGGCTATGATGAGCGGGAGTAGGGAATC	Tb927.11.4180_rev	GGGGACCACTTTGTACAAGAAAGCTGGGTTCAGCCAGTGTGTGCATCATTG
7_hypothetical	J23	Tb927.9.13320_for	GGGGACAAGTTTGTACAAAAAAGCAGGCTATGAGTGCCCCCCAACCC	Tb927.9.13320_rev	GGGGACCACTTTGTACAAGAAAGCTGGGTTCAACCGTCCAATGAAACAACAGG
7_hypothetical	J24	Tb927.7.4120_for	GGGGACAAGTTTGTACAAAAAAGCAGGCTATGGATCTGGATTCTTTCCTCGACA	Tb927.7.4120_rev	GGGGACCACTTTGTACAAGAAAGCTGGGTTTAGCTTGGTGGTCTAAAGGTGCT
7_hypothetical	K1	Tb927.11.14980_for	GGGGACAAGTTTGTACAAAAAAGCAGGCTATGGTAATGCGTCGATGTAGTTACAAC	Tb927.11.14980_rev	GGGGACCACTTTGTACAAGAAAGCTGGGTCTACCGTCTGTACGTTATGGGAACA
7_hypothetical	K2	Tb927.9.6270_for	GGGGACAAGTTTGTACAAAAAAGCAGGCTATGTTTTGTTGCACCTTCACTCAAGC	Tb927.9.6270_rev	GGGGACCACTTTGTACAAGAAAGCTGGGTTCATATTTGGTGCTTCTCACCGG
7_hypothetical	K3	Tb927.10.3720_for	GGGGACAAGTTTGTACAAAAAAGCAGGCTATGCGAGGGGATGTGCCA	Tb927.10.3720_rev	GGGGACCACTTTGTACAAGAAAGCTGGGTTCACCATCTATTCCGCTTACGGG
7_hypothetical	K4	Tb927.4.1540_for	GGGGACAAGTTTGTACAAAAAAGCAGGCTATGCTTCGTGTATTCTCCGGC	Tb927.4.1540_rev	GGGGACCACTTTGTACAAGAAAGCTGGGTTCAATTCTTTCCCCTCCTTCGCA
7_hypothetical	K5	Tb927.4.1120_for	GGGGACAAGTTTGTACAAAAAAGCAGGCTATGGAGAGGTGTTGCACGCT	Tb927.4.1120_rev	GGGGACCACTTTGTACAAGAAAGCTGGGTTCAATTGTAAACCTTTTGTGCGCG
7_hypothetical	K6	Tb927.10.7830_for	GGGGACAAGTTTGTACAAAAAAGCAGGCTATGCAGCTTTGTTATAGTTGTCGACA	Tb927.10.7830_rev	GGGGACCACTTTGTACAAGAAAGCTGGGTTCAAACCTCCTTTTCTGTAATGAAGATTGA
7_hypothetical	K7	Tb927.4.350_for	GGGGACAAGTTTGTACAAAAAAGCAGGCTATGAAAAGCGCAAATGTACCCACA	Tb927.4.350_rev	GGGGACCACTTTGTACAAGAAAGCTGGGTTTAATAACCTTTACGTCTTCGTTTACCCA
7_hypothetical	K8	Tb927.10.6700_for	GGGGACAAGTTTGTACAAAAAAGCAGGCTATGGCTGGTGCGGAACAAC	Tb927.10.6700_rev	GGGGACCACTTTGTACAAGAAAGCTGGGTCTACCGGTGATACAACGTCGC
7_hypothetical	K9	Tb927.5.1860_for	GGGGACAAGTTTGTACAAAAAAGCAGGCTATGCAATTCTCGACCTACATGACG	Tb927.5.1860_rev	GGGGACCACTTTGTACAAGAAAGCTGGGTCTATTCAGCTTCACAGAAAAGAGTACAAAC
7_hypothetical	K10	Tb927.8.7550_for	GGGGACAAGTTTGTACAAAAAAGCAGGCTATGCCCCAAACGGTTCAGAAG	Tb927.8.7550_rev	GGGGACCACTTTGTACAAGAAAGCTGGGTCTACAGCACAATCCACCGTCG
7_hypothetical	K11	Tb927.11.7660_for	GGGGACAAGTTTGTACAAAAAAGCAGGCTATGTTTGATGAGGACGCGACG	Tb927.11.7660_rev	GGGGACCACTTTGTACAAGAAAGCTGGGTCTATTTCCCCCCTTTACGCGATTT
7_hypothetical	K12	Tb927.8.6670_for	GGGGACAAGTTTGTACAAAAAAGCAGGCTATGGCTGCCGCCCCGGCG	Tb927.8.6670_rev	GGGGACCACTTTGTACAAGAAAGCTGGGTTCATTGTCCCATAAAGAGAGACATCATTG
7_hypothetical	K13	Tb927.4.530_for	GGGGACAAGTTTGTACAAAAAAGCAGGCTATGTCTCCTCTAGATTTACTGCAGCA	Tb927.4.530_rev	GGGGACCACTTTGTACAAGAAAGCTGGGTTCAGCGGTTGGTATCAGCACT
7_hypothetical	K14	Tb927.11.5240_for	GGGGACAAGTTTGTACAAAAAAGCAGGCTATGCAAGGGACCGCATGTG	Tb927.11.5240_rev	GGGGACCACTTTGTACAAGAAAGCTGGGTTTATTGAAAGGCAGGTAATGAGGGGT
7_hypothetical	K15	Tb927.8.3000_for	GGGGACAAGTTTGTACAAAAAAGCAGGCTATGTGCGCAATGGAATATAGCGA	Tb927.8.3000_rev	GGGGACCACTTTGTACAAGAAAGCTGGGTCTAACCCGACACATATTGTGGAGC
7_hypothetical	K16	Tb927.3.1220_for	GGGGACAAGTTTGTACAAAAAAGCAGGCTATGGACACCCGGTTTCACTGT	Tb927.3.1220_rev	GGGGACCACTTTGTACAAGAAAGCTGGGTTCAGTGGTTAAGCCACTCGTCG
7_hypothetical	K17	Tb927.9.12040_for	GGGGACAAGTTTGTACAAAAAAGCAGGCTATGTATCCTAGTCTTTTTAAGAAGCGACC	Tb927.9.12040_rev	GGGGACCACTTTGTACAAGAAAGCTGGGTTTACTGTTTCATTTTTGTTGTTACCCAAAC
7_hypothetical	K18	Tb927.4.4110_for	GGGGACAAGTTTGTACAAAAAAGCAGGCTATGGCAAACACGGCTCTGC	Tb927.4.4110_rev	GGGGACCACTTTGTACAAGAAAGCTGGGTTCACGTAGGGGGTTCCACATC

7_hypothetical	K19	Tb927.8.2130_for	GGGGACAAGTTTGTACAAAAAAGCAGGCTATGTGGACTGTTTTTGCTCCAGATC	Tb927.8.2130_rev	GGGGACCACTTTGTACAAGAAAGCTGGGTTCACTTTTGGACGGGTTGCAG
7_hypothetical	K20	Tb927.4.2340_for	GGGGACAAGTTTGTACAAAAAAGCAGGCTATGTGGACTTGATCTTCCCCGAAGA	Tb927.4.2340_rev	GGGGACCACTTTGTACAAGAAAGCTGGGTCTACAGAAGTCCAACCTCTTGAGAAGC
7_hypothetical	K21	Tb927.11.7300_for	GGGGACAAGTTTGTACAAAAAAGCAGGCTATGTGGAAAGACAATCTGATGAAGATATGCTC	Tb927.11.7300_rev	GGGGACCACTTTGTACAAGAAAGCTGGGTCTAGGGCTCACGATGCAAATATACC
7_hypothetical	K22	Tb927.11.15710_for	GGGGACAAGTTTGTACAAAAAAGCAGGCTATGTCCTTCAGGATTCCCAATAATTTACTG	Tb927.11.15710_rev	GGGGACCACTTTGTACAAGAAAGCTGGGTTCACGCACAGATAAACTGCCAC
7_hypothetical	K23	Tb927.7.4310_for	GGGGACAAGTTTGTACAAAAAAGCAGGCTATGCTCTGGAAGTTATTCACAAGTAGC	Tb927.7.4310_rev	GGGGACCACTTTGTACAAGAAAGCTGGGTTTAACAGTGAGAAAAGTCAACAATATCGGT
7_hypothetical	K24	Tb927.6.1590_for	GGGGACAAGTTTGTACAAAAAAGCAGGCTATGCTGCGTTTATCGTTGATTCGC	Tb927.6.1590_rev	GGGGACCACTTTGTACAAGAAAGCTGGGTTTATTGAGGTGGCGCCCTAAGT
7_hypothetical	L1	Tb927.3.5390_for	GGGGACAAGTTTGTACAAAAAAGCAGGCTATGACTTCTCCAAAACGGCCC	Tb927.3.5390_rev	GGGGACCACTTTGTACAAGAAAGCTGGGTTTACATGGGAGAACAGGAGCTGT
7_hypothetical	L2	Tb927.11.3710_for	GGGGACAAGTTTGTACAAAAAAGCAGGCTATGGTAGGGCAAACCCAATCGA	Tb927.11.3710_rev	GGGGACCACTTTGTACAAGAAAGCTGGGTTTACACGTGCAACTCCATTGCT
7_hypothetical	L3	Tb927.5.1500_for	GGGGACAAGTTTGTACAAAAAAGCAGGCTATGTCAGACGCCGGCTGC	Tb927.5.1500_rev	GGGGACCACTTTGTACAAGAAAGCTGGGTCTACGCACTGGTTGGCGAC
7_hypothetical	L4	Tb927.2.5080_for	GGGGACAAGTTTGTACAAAAAAGCAGGCTATGAATTTGAAAGAGTTGTTAACTTTTTTA	Tb927.2.5080_rev	GGGGACCACTTTGTACAAGAAAGCTGGGTCTACAATTTTAACTTAGAAATGAGCGAAAC
7_hypothetical	L5	Tb927.7.5060_for	GGGGACAAGTTTGTACAAAAAAGCAGGCTATGACCGGTGGGGAGTTGG	Tb927.7.5060_rev	GGGGACCACTTTGTACAAGAAAGCTGGGTCTAACTGCAACCACCACCGC
7_hypothetical	L6	Tb927.9.9270_for	GGGGACAAGTTTGTACAAAAAAGCAGGCTATGACCTCACCCATCGCAGT	Tb927.9.9270_rev	GGGGACCACTTTGTACAAGAAAGCTGGGTTTAATCAGTCTGCAAAACGTAATCAACAG
7_hypothetical	L7	Tb927.4.540_for	GGGGACAAGTTTGTACAAAAAAGCAGGCTATGTCTTTTCGTCACTTCAGCCG	Tb927.4.540_rev	GGGGACCACTTTGTACAAGAAAGCTGGGTTTAATCATCGTCCGGATCCACAAAAT
7_hypothetical	L8	Tb927.10.4020_for	GGGGACAAGTTTGTACAAAAAAGCAGGCTATGCCCTATTTTTTTAAACCACTGGGA	Tb927.10.4020_rev	GGGGACCACTTTGTACAAGAAAGCTGGGTCTCACCATAGACGCCACCATATCG
7_hypothetical	L9	Tb927.7.5350_for	GGGGACAAGTTTGTACAAAAAAGCAGGCTATGTTGCGCGAAAGGAGTTGC	Tb927.7.5350_rev	GGGGACCACTTTGTACAAGAAAGCTGGGTTTACAGAAAGCATTTAAGTTCAAGGGAGC
7_hypothetical	L10	Tb927.10.1840_for	GGGGACAAGTTTGTACAAAAAAGCAGGCTATGGAGGACACGGCGGCT	Tb927.10.1840_rev	GGGGACCACTTTGTACAAGAAAGCTGGGTTTAAGTCATCATCGCAATATCTGTCAAGA
7_hypothetical	L11	Tb927.10.3970_for	GGGGACAAGTTTGTACAAAAAAGCAGGCTATGGTAGGTGCTTCTTCCAAGCG	Tb927.10.3970_rev	GGGGACCACTTTGTACAAGAAAGCTGGGTCTAAAATTCCTCATCAAAACTAAGTCCTTT
7_hypothetical	L12	Tb927.11.14220_for	GGGGACAAGTTTGTACAAAAAAGCAGGCTATGGACAGCATCACTGCAAACG	Tb927.11.14220_rev	GGGGACCACTTTGTACAAGAAAGCTGGGTCTATTGTTGCGGCTGCTGCA
7_hypothetical	L13	Tb927.8.6800_for	GGGGACAAGTTTGTACAAAAAAGCAGGCTATGTCGTGGCGACTATTCCTTGC	Tb927.8.6800_rev	GGGGACCACTTTGTACAAGAAAGCTGGGTCTCAATCAGATATCTCCTCTGCCATCC
7_hypothetical	L14	Tb927.3.5380_for	GGGGACAAGTTTGTACAAAAAAGCAGGCTATGTAACGGACGTTACGAGCAC	Tb927.3.5380_rev	GGGGACCACTTTGTACAAGAAAGCTGGGTTTACCTCAACCCGTTTGCATGAC
7_hypothetical	L15	Tb927.10.15740_for	GGGGACAAGTTTGTACAAAAAAGCAGGCTATGTCTGGAGAAAACTTGAATAAATCGATG	Tb927.10.15740_rev	GGGGACCACTTTGTACAAGAAAGCTGGGTCTCACGCCGTGGTCCATGC
7_hypothetical	L16	Tb927.9.13060_for	GGGGACAAGTTTGTACAAAAAAGCAGGCTATGCCTCCGAGAAGTGCTGG	Tb927.9.13060_rev	GGGGACCACTTTGTACAAGAAAGCTGGGTCTCATTCGCCTTCTTGCGGTG
7_hypothetical	L18	Tb927.11.660_for	GGGGACAAGTTTGTACAAAAAAGCAGGCTATGAGCTTACAATGCGAAGGTGATG	Tb927.11.660_rev	GGGGACCACTTTGTACAAGAAAGCTGGGTCTACATGAGAGCACAGCTCTTCATC
7_hypothetical	L19	Tb927.3.3350_for	GGGGACAAGTTTGTACAAAAAAGCAGGCTATGGGAGATTGTTTTGACGAAGGC	Tb927.3.3350_rev	GGGGACCACTTTGTACAAGAAAGCTGGGTCTAGCGGCAGGCAGAACCA
7_hypothetical	L20	Tb927.8.5720_for	GGGGACAAGTTTGTACAAAAAAGCAGGCTATGGAGAGGCAGCAACCATTG	Tb927.8.5720_rev	GGGGACCACTTTGTACAAGAAAGCTGGGTCTCAGCCCTCGCGCGCTCG
7_hypothetical	L21	Tb927.10.3750_for	GGGGACAAGTTTGTACAAAAAAGCAGGCTATGTCCAGTACCGGTAAGTTTGTGG	Tb927.10.3750_rev	GGGGACCACTTTGTACAAGAAAGCTGGGTTTAAAGATTTCTGTTGATGATGGACCGC
7_hypothetical	L22	Tb927.11.15660_for	GGGGACAAGTTTGTACAAAAAAGCAGGCTATGATTCATGCGGTTGTTATGGCG	Tb927.11.15660_rev	GGGGACCACTTTGTACAAGAAAGCTGGGTCTAGGTCTCCGGCCTTTCAACC
7_hypothetical	L23	Tb927.11.13460_for	GGGGACAAGTTTGTACAAAAAAGCAGGCTATGCCGCCCAAGCGTTCC	Tb927.11.13460_rev	GGGGACCACTTTGTACAAGAAAGCTGGGTCTATTTCAATTTTGAAGCTGTGCGTTTG
7_hypothetical	L24	Tb927.11.560_for	GGGGACAAGTTTGTACAAAAAAGCAGGCTATGCCACCAAAGAAAGATGTGGC	Tb927.11.560_rev	GGGGACCACTTTGTACAAGAAAGCTGGGTCTAGTCAGATGACATGATCGAGCG
7_hypothetical	M1	Tb927.9.14130_for	GGGGACAAGTTTGTACAAAAAAGCAGGCTATGCAATGCAATATGAAAACTTGGATAATT	Tb927.9.14130_rev	GGGGACCACTTTGTACAAGAAAGCTGGGTTTAACTTGGTTCAACGTATGACCGC
7_hypothetical	M2	Tb927.5.780_for	GGGGACAAGTTTGTACAAAAAAGCAGGCTATGTACGTTTGTGAGAACGTTGATGC	Tb927.5.780_rev	GGGGACCACTTTGTACAAGAAAGCTGGGTTTAGGAGCGTCGCCTGACTT
7_hypothetical	M3	Tb927.10.9360_for	GGGGACAAGTTTGTACAAAAAAGCAGGCTATGGCCAAAAGTTTCCTCAAGTGG	Tb927.10.9360_rev	GGGGACCACTTTGTACAAGAAAGCTGGGTCTACCAGCGGAATTGTTCAGCA
7_hypothetical	M4	Tb927.10.5530_for	GGGGACAAGTTTGTACAAAAAAGCAGGCTATGAAAGCCCGGCGACGT	Tb927.10.5530_rev	GGGGACCACTTTGTACAAGAAAGCTGGGTCTACGTTGCCAGCAGGCG
7_hypothetical	M5	Tb927.5.2130_for	GGGGACAAGTTTGTACAAAAAAGCAGGCTATGTCGAGTCTGGTGCATCACT	Tb927.5.2130_rev	GGGGACCACTTTGTACAAGAAAGCTGGGTTCACACATCCCCTACGGCAT
7_hypothetical	M6	Tb927.8.4920_for	GGGGACAAGTTTGTACAAAAAAGCAGGCTATGTTCCAACTGACCTTGCGC	Tb927.8.4920_rev	GGGGACCACTTTGTACAAGAAAGCTGGGTTTAAGTGTGCGAGCACCGGG
7_hypothetical	M7	Tb927.4.4030_for	GGGGACAAGTTTGTACAAAAAAGCAGGCTATGGATGCCACAACTCAGGAAGG	Tb927.4.4030_rev	GGGGACCACTTTGTACAAGAAAGCTGGGTTTACCGAAAGGCGACAATACAAATGT
7_hypothetical	M8	Tb927.10.15040_for	GGGGACAAGTTTGTACAAAAAAGCAGGCTATGGGGGGAGGCGCTTGC	Tb927.10.15040_rev	GGGGACCACTTTGTACAAGAAAGCTGGGTCTACATCTCTTCCGCGGCG
7_hypothetical	M9	Tb927.10.13440_for	GGGGACAAGTTTGTACAAAAAAGCAGGCTATGGGTGTAGAAATCCCACTACCC	Tb927.10.13440_rev	GGGGACCACTTTGTACAAGAAAGCTGGGTCTCATTTGTGGGTTTGCAAGCGC
7_hypothetical	M10	Tb927.6.2010_for	GGGGACAAGTTTGTACAAAAAAGCAGGCTATGCTCCGTCGCCTCGCC	Tb927.6.2010_rev	GGGGACCACTTTGTACAAGAAAGCTGGGTCTAGTACAATTGGGTGCCATGCA
7_hypothetical	M11	Tb927.11.8050_for	GGGGACAAGTTTGTACAAAAAAGCAGGCTATGATGAAGGGCAAACGCCTG	Tb927.11.8050_rev	GGGGACCACTTTGTACAAGAAAGCTGGGTCTACTGAAACCGAACGCTTTGCG
7_hypothetical	M12	Tb927.11.7670_for	GGGGACAAGTTTGTACAAAAAAGCAGGCTATGCCACCAGCGTACCGA	Tb927.11.7670_rev	GGGGACCACTTTGTACAAGAAAGCTGGGTTCAATTGTCAAGGTAGGACAAGAGC
7_hypothetical	M13	Tb927.2.5930_for	GGGGACAAGTTTGTACAAAAAAGCAGGCTATGCGGCGCTTTACACTTATAACG	Tb927.2.5930_rev	GGGGACCACTTTGTACAAGAAAGCTGGGTTCATTCCTGATCCGAGGAGTGATG
7_hypothetical	M14	Tb927.3.990_for	GGGGACAAGTTTGTACAAAAAAGCAGGCTATGGATCGATCACCTTCGCAAC	Tb927.3.990_rev	GGGGACCACTTTGTACAAGAAAGCTGGGTCTCAAAGTTGACGCGCCGC
7_hypothetical	M15	Tb927.11.7110_for	GGGGACAAGTTTGTACAAAAAAGCAGGCTATGAGTGTGAAAGAGCAGCTTCG	Tb927.11.7110_rev	GGGGACCACTTTGTACAAGAAAGCTGGGTTTACAAGACAGCACTTGATATTGTCGA
7_hypothetical	M16	Tb927.11.1550_for	GGGGACAAGTTTGTACAAAAAAGCAGGCTATGGGCACCAAGGGGCTAC	Tb927.11.1550_rev	GGGGACCACTTTGTACAAGAAAGCTGGGTCTCATTTTACACCTCCATCGCAGAC
7_hypothetical	M17	Tb927.9.12500_for	GGGGACAAGTTTGTACAAAAAAGCAGGCTATGCAAGCCGCTTGTACCAG	Tb927.9.12500_rev	GGGGACCACTTTGTACAAGAAAGCTGGGTCTCATGCTGTTGGAACCTCCAAGT
7_hypothetical	M18	Tb927.8.5990_for	GGGGACAAGTTTGTACAAAAAAGCAGGCTATGCCAGTCCGAAAGAAGCAAC	Tb927.8.5990_rev	GGGGACCACTTTGTACAAGAAAGCTGGGTTTAGTGGACCCAAATTAGAACTTTCTTGT
7_hypothetical	M19	Tb927.9.14050_for	GGGGACAAGTTTGTACAAAAAAGCAGGCTATGAGTGTGCCATCCGCAATC	Tb927.9.14050_rev	GGGGACCACTTTGTACAAGAAAGCTGGGTTTACACTGGGCTTCTTAACTCGTCC
7_hypothetical	M20	Tb927.10.7270_for	GGGGACAAGTTTGTACAAAAAAGCAGGCTATGTTTTTCGTCTTTCCGTTTCCTACG	Tb927.10.7270_rev	GGGGACCACTTTGTACAAGAAAGCTGGGTCTATAGTGCGTCAGGAGAATCGATG
7_hypothetical	M21	Tb927.10.2510_for	GGGGACAAGTTTGTACAAAAAAGCAGGCTATGGACCTACCCATCGAACTGC	Tb927.10.2510_rev	GGGGACCACTTTGTACAAGAAAGCTGGGTTTATGGTCCAAACTCCACCCCA
7_hypothetical	M22	Tb927.11.13080_for	GGGGACAAGTTTGTACAAAAAAGCAGGCTATGGAAGGTTTTTGCTACACTGCT	Tb927.11.13080_rev	GGGGACCACTTTGTACAAGAAAGCTGGGTCTCAGAACAGTAGGGCGGCTAG
7_hypothetical	M23	Tb927.11.12630_for	GGGGACAAGTTTGTACAAAAAAGCAGGCTATGACCGTTCCGGTTAGGCA	Tb927.11.12630_rev	GGGGACCACTTTGTACAAGAAAGCTGGGTTTATAACGCGGCACGCTTGC
7_hypothetical	M24	Tb927.8.1770_for	GGGGACAAGTTTGTACAAAAAAGCAGGCTATGCATAGTGTAGAGGTGATGGGG	Tb927.8.1770_rev	GGGGACCACTTTGTACAAGAAAGCTGGGTCTCAGGACTGCTGTGTTGCGT
7_hypothetical	N1	Tb927.11.14410_for	GGGGACAAGTTTGTACAAAAAAGCAGGCTATGAACGGCGTAGAAATAAACGAAAGG	Tb927.11.14410_rev	GGGGACCACTTTGTACAAGAAAGCTGGGTCTACGATTTCTTTGAAATGTCAAATCTGCG
7_hypothetical	N2	Tb927.10.12470_for	GGGGACAAGTTTGTACAAAAAAGCAGGCTATGAGTTTACCGGAGCTTGATTCG	Tb927.10.12470_rev	GGGGACCACTTTGTACAAGAAAGCTGGGTTTACGGTTTCGTGTGCATGTTGA
7_hypothetical	N3	Tb927.4.4890_for	GGGGACAAGTTTGTACAAAAAAGCAGGCTATGGGCCACCAACTGTGTGA	Tb927.4.4890_rev	GGGGACCACTTTGTACAAGAAAGCTGGGTCTAACGAAAGACAAACTTGTCAGGTCT
7_hypothetical	N4	Tb927.4.4720_for	GGGGACAAGTTTGTACAAAAAAGCAGGCTATGGTCCTCCGCAGAAGTCTGT	Tb927.4.4720_rev	GGGGACCACTTTGTACAAGAAAGCTGGGTCTCACCGACCTGTGGCGGC
7_hypothetical	N5	Tb927.5.3450_for	GGGGACAAGTTTGTACAAAAAAGCAGGCTATGGAGTCGAATGTGTCCGATACT	Tb927.5.3450_rev	GGGGACCACTTTGTACAAGAAAGCTGGGTCTATCTCCGTTTCTTCTTCTGCACTAC
7_hypothetical	N6	Tb927.7.6240_for	GGGGACAAGTTTGTACAAAAAAGCAGGCTATGCATCCAACTTCGTTCCCG	Tb927.7.6240_rev	GGGGACCACTTTGTACAAGAAAGCTGGGTCTCACTCCTCTTGCTCATCTATGATCC
7_hypothetical	N7	Tb927.7.4500_for	GGGGACAAGTTTGTACAAAAAAGCAGGCTATGATGCCTTCAGGTGACGGC	Tb927.7.4500_rev	GGGGACCACTTTGTACAAGAAAGCTGGGTCTCACTCATCAGCGGTGTATGCC
7_hypothetical	N8	Tb927.11.2340_for	GGGGACAAGTTTGTACAAAAAAGCAGGCTATGAGCCGTCCGTATACCAAAGG	Tb927.11.2340_rev	GGGGACCACTTTGTACAAGAAAGCTGGGTTTACCTCCAAGTAAGTTTGCCATGAC
7_hypothetical	N9	Tb927.11.5150_for	GGGGACAAGTTTGTACAAAAAAGCAGGCTATGGATAAGGCATTGTGTACCTCTCT	Tb927.11.5150_rev	GGGGACCACTTTGTACAAGAAAGCTGGGTTTATGTCACTGTTAATGGACAGGAAGAC
7_hypothetical	N10	Tb927.5.3420_for	GGGGACAAGTTTGTACAAAAAAGCAGGCTATGGCTTGGCGCACAATTCG	Tb927.5.3420_rev	GGGGACCACTTTGTACAAGAAAGCTGGGTCTAACCGCTAAGTTGCAGCTGC
7_hypothetical	N11	Tb927.2.6080_for	GGGGACAAGTTTGTACAAAAAAGCAGGCTATGCTAACGTTTCCCGGATTTGT	Tb927.2.6080_rev	GGGGACCACTTTGTACAAGAAAGCTGGGTCTACGCATATTCATCATTGGCTTCAGC
7_hypothetical	N12	Tb927.11.10500_for	GGGGACAAGTTTGTACAAAAAAGCAGGCTATGACTAGCAGGAAGCGGCC	Tb927.11.10500_rev	GGGGACCACTTTGTACAAGAAAGCTGGGTTTACAGGAGCTGCACTCGGC
7_hypothetical	N13	Tb927.9.10500_for	GGGGACAAGTTTGTACAAAAAAGCAGGCTATGTTCTCCGCCTCTTGGGG	Tb927.9.10500_rev	GGGGACCACTTTGTACAAGAAAGCTGGGTTTAACTGAAGCCATAATCTTCATGGAGATC
7_hypothetical	N14	Tb927.6.2930_for	GGGGACAAGTTTGTACAAAAAAGCAGGCTATGGACACTTACGCAGCGTTG	Tb927.6.2930_rev	GGGGACCACTTTGTACAAGAAAGCTGGGTCTAGAAGACAGATCGCCTACTTCCT
7_hypothetical	N15	Tb927.5.2150_for	GGGGACAAGTTTGTACAAAAAAGCAGGCTATGCTGGAGAGGCGTGCG	Tb927.5.2150_rev	GGGGACCACTTTGTACAAGAAAGCTGGGTCTACCTGGCCGCGAGGCG
7_hypothetical	N16	Tb927.11.4420_for	GGGGACAAGTTTGTACAAAAAAGCAGGCTATGGATCGTCCAACTAAGGAAGCA	Tb927.11.4420_rev	GGGGACCACTTTGTACAAGAAAGCTGGGTCTCACCGAAAGTGGATCTCCTTTTTC
7_hypothetical	N17	Tb927.10.8730_for	GGGGACAAGTTTGTACAAAAAAGCAGGCTATGCTTCGCCGCATTTGGA	Tb927.10.8730_rev	GGGGACCACTTTGTACAAGAAAGCTGGGTCTACCATGCATTCGCATTTAAGTGAAC
7_hypothetical	N18	Tb927.8.7290_for	GGGGACAAGTTTGTACAAAAAAGCAGGCTATGCTCCGTCGGTTGCAC	Tb927.8.7290_rev	GGGGACCACTTTGTACAAGAAAGCTGGGTCTAACGCATGCTCCGCAGAG
7_hypothetical	N19	Tb927.11.6600_for	GGGGACAAGTTTGTACAAAAAAGCAGGCTATGGATCCCAGAAACCCAGCC	Tb927.11.6600_rev	GGGGACCACTTTGTACAAGAAAGCTGGGTCTCAAGCACTTATGCCACACCTCC
7_hypothetical	N20	Tb927.6.4830_for	GGGGACAAGTTTGTACAAAAAAGCAGGCTATGCCGTACCCATTGGGGT	Tb927.6.4830_rev	GGGGACCACTTTGTACAAGAAAGCTGGGTCTACTCAAGCGTTTCGATGTTGTCA
7_hypothetical	N21	Tb927.11.3440_for	GGGGACAAGTTTGTACAAAAAAGCAGGCTATGGTGTATGAGTTTGTGCTTGAGC	Tb927.11.3440_rev	GGGGACCACTTTGTACAAGAAAGCTGGGTCTAAGACGGATGCATGTCCTGTC
7_hypothetical	N22	Tb927.2.5950_for	GGGGACAAGTTTGTACAAAAAAGCAGGCTATGAATCGCGACAAGGGTAAGTTC	Tb927.2.5950_rev	GGGGACCACTTTGTACAAGAAAGCTGGGTCTCATTTGAACGTTTTGCGGTCG
7_hypothetical	N23	Tb927.1.4380_for	GGGGACAAGTTTGTACAAAAAAGCAGGCTATGTTGAGTCTGCTGCTTCTACTGC	Tb927.1.4380_rev	GGGGACCACTTTGTACAAGAAAGCTGGGTCTCACCTATGTGGTCGACGTTCG
7_hypothetical	N24	Tb927.6.3350_for	GGGGACAAGTTTGTACAAAAAAGCAGGCTATGCTCTTGGGATGCCGTCG	Tb927.6.3350_rev	GGGGACCACTTTGTACAAGAAAGCTGGGTTTAATTAAAGTTATTCAGTTCGTCCAACAA
7_hypothetical	O1	Tb927.9.14120_for	GGGGACAAGTTTGTACAAAAAAGCAGGCTATGGAGCATTTTGCCCCATGTG	Tb927.9.14120_rev	GGGGACCACTTTGTACAAGAAAGCTGGGTCTACCGGTTCCTATGCTTACGTCG
7_hypothetical	O2	Tb927.3.2680_for	GGGGACAAGTTTGTACAAAAAAGCAGGCTATGGCGAGTCCGGAAGTCA	Tb927.3.2680_rev	GGGGACCACTTTGTACAAGAAAGCTGGGTCTCAACTCCGAAGGGATGTGTCA
7_hypothetical	O3	Tb927.3.5080_for	GGGGACAAGTTTGTACAAAAAAGCAGGCTATGCCTGTAGTGTCGACTGTCG	Tb927.3.5080_rev	GGGGACCACTTTGTACAAGAAAGCTGGGTTCAATTACTTAAATCAGGAGTTGTGGCA
7_hypothetical	O4	Tb927.9.3670_for	GGGGACAAGTTTGTACAAAAAAGCAGGCTATGCTTCGCATCAGCACCC	Tb927.9.3670_rev	GGGGACCACTTTGTACAAGAAAGCTGGGTCTCAATCCTTTATAGATATTTCCCCCACGT
7_hypothetical	O5	Tb927.5.4080_for	GGGGACAAGTTTGTACAAAAAAGCAGGCTATGTTCTGTTGCTTTTGGGGACTT	Tb927.5.4080_rev	GGGGACCACTTTGTACAAGAAAGCTGGGTCTCACACATTCGTGTGGGGGC
7_hypothetical	O6	Tb927.8.5560_for	GGGGACAAGTTTGTACAAAAAAGCAGGCTATGGCTTTTGCAACCTCCCC	Tb927.8.5560_rev	GGGGACCACTTTGTACAAGAAAGCTGGGTCTCACTTTTCCATCCACTTCAAATCAGT
7_hypothetical	O7	Tb927.7.4300_for	GGGGACAAGTTTGTACAAAAAAGCAGGCTATGCCGCAGATCTTTAGTGATGAATC	Tb927.7.4300_rev	GGGGACCACTTTGTACAAGAAAGCTGGGTCTCACACATCTCTGCGATTGTGC
7_hypothetical	O8	Tb927.10.620_for	GGGGACAAGTTTGTACAAAAAAGCAGGCTATGGAGTTTCCGCAAGTCACATG	Tb927.10.620_rev	GGGGACCACTTTGTACAAGAAAGCTGGGTTTAGATAACAAAATGTGATTGCCACTCCG
7_hypothetical	O9	Tb927.7.790_for	GGGGACAAGTTTGTACAAAAAAGCAGGCTATGCTGGTCTCCTTGGCGC	Tb927.7.790_rev	GGGGACCACTTTGTACAAGAAAGCTGGGTCTATTCTTGCTGAAAGTCAGCCGC
7_hypothetical	O10	Tb927.4.1560_for	GGGGACAAGTTTGTACAAAAAAGCAGGCTATGAGTGACTTAAATTTTGAGCGACCT	Tb927.4.1560_rev	GGGGACCACTTTGTACAAGAAAGCTGGGTCTCAAGGAACCAGCGCATCAGT
7_hypothetical	O11	Tb927.10.13520_for	GGGGACAAGTTTGTACAAAAAAGCAGGCTATGAATGTCAACTTGGACGATCTCTTT	Tb927.10.13520_rev	GGGGACCACTTTGTACAAGAAAGCTGGGTCTACTTACGAGCCTCGAAGCAAG
7_hypothetical	O12	Tb927.9.2760_for	GGGGACAAGTTTGTACAAAAAAGCAGGCTATGGACCATCGCAATACCCATGG	Tb927.9.2760_rev	GGGGACCACTTTGTACAAGAAAGCTGGGTTTACTCTGCAGCGTACAATACATCC
7_hypothetical	O13	Tb927.10.12670_for	GGGGACAAGTTTGTACAAAAAAGCAGGCTATGATGATAAGTGTAAGGAAAACGGTGAA	Tb927.10.12670_rev	GGGGACCACTTTGTACAAGAAAGCTGGGTCTATATGGACGGACGGACGACC
7_hypothetical	O14	Tb927.10.5790_for	GGGGACAAGTTTGTACAAAAAAGCAGGCTATGGCCACAATTCTTACAACTAGCA	Tb927.10.5790_rev	GGGGACCACTTTGTACAAGAAAGCTGGGTCTCACAAAACTTTTGAGTGTATATCACCG
7_hypothetical	O15	Tb927.11.12280_for	GGGGACAAGTTTGTACAAAAAAGCAGGCTATGAATGCCAAATCTTCTTGTCTCGT	Tb927.11.12280_rev	GGGGACCACTTTGTACAAGAAAGCTGGGTCTCACTGCTTTACCCCACAACCT
7_hypothetical	O16	Tb927.9.8030_for	GGGGACAAGTTTGTACAAAAAAGCAGGCTATGAAGAGGAAAGGCATGACCAAC	Tb927.9.8030_rev	GGGGACCACTTTGTACAAGAAAGCTGGGTCTCACTGTGTTGACGGGGGAT
7_hypothetical	O17	Tb927.9.1350_for	GGGGACAAGTTTGTACAAAAAAGCAGGCTATGGTATTCAGAGATGCCTCCCAA	Tb927.9.1350_rev	GGGGACCACTTTGTACAAGAAAGCTGGGTCTACGCACTGGGGTGCATG
7_hypothetical	O18	Tb11.02.5440_for	GGGGACAAGTTTGTACAAAAAAGCAGGCTATGGAGTGTGATAAACCATCAGGGA	Tb11.02.5440_rev	GGGGACCACTTTGTACAAGAAAGCTGGGTCTACGCTGCAAAGCGCATCC
7_hypothetical	O19	Tb927.10.9210_for	GGGGACAAGTTTGTACAAAAAAGCAGGCTATGCTTCGCACTAGCATTATTGTGC	Tb927.10.9210_rev	GGGGACCACTTTGTACAAGAAAGCTGGGTCTCACTTACTTCTCGATACCTTTATCAAGTC
7_hypothetical	O20	Tb927.7.6790_for	GGGGACAAGTTTGTACAAAAAAGCAGGCTATGCTGGTGGAATGTTCGGAAAT	Tb927.7.6790_rev	GGGGACCACTTTGTACAAGAAAGCTGGGTTTATTGGGGTTGTAAAACATTACCACCT
7_hypothetical	O21	Tb927.5.3460_for	GGGGACAAGTTTGTACAAAAAAGCAGGCTATGAATAGCGTATCGTGCCAGATC	Tb927.5.3460_rev	GGGGACCACTTTGTACAAGAAAGCTGGGTTCACAAAACCTGCTCCACTGGC
7_hypothetical	O22	Tb927.7.2650_for	GGGGACAAGTTTGTACAAAAAAGCAGGCTATGAGCACCACTGCACTGTCT	Tb927.7.2650_rev	GGGGACCACTTTGTACAAGAAAGCTGGGTCTCAGTAGTTGGCTTCACGAGCA
7_hypothetical	O23	Tb927.5.1530_for	GGGGACAAGTTTGTACAAAAAAGCAGGCTATGCAGTCCTGTGCTGTGATTG	Tb927.5.1530_rev	GGGGACCACTTTGTACAAGAAAGCTGGGTCTAAACGGTATTGCTGAAAAGGATGTG
7_hypothetical	O24	Tb927.4.030_for	GGGGACAAGTTTGTACAAAAAAGCAGGCTATGATAATCTCTAAGGAGGAGCTGAGTG	Tb927.7.4030_rev	GGGGACCACTTTGTACAAGAAAGCTGGGTTTAAGACTTATTGGAAACAGAGTGCCC
7_hypothetical	P1	Tb927.5.3070_for	GGGGACAAGTTTGTACAAAAAAGCAGGCTATGGCTAAGAAAAAGAAGTCCAAACGG	Tb927.5.3070_rev	GGGGACCACTTTGTACAAGAAAGCTGGGTTTACCTCTTCCGCCCCTCAG
7_hypothetical	P2	Tb927.11.4190_for	GGGGACAAGTTTGTACAAAAAAGCAGGCTATGGGAAGAGGGTACAGTAACAGTC	Tb927.11.4190_rev	GGGGACCACTTTGTACAAGAAAGCTGGGTCTATCCTTCATCCTCCTCGTTACCG
7_hypothetical	P3	Tb927.7.200_for	GGGGACAAGTTTGTACAAAAAAGCAGGCTATGCCGGTAGGCTGGTCC	Tb927.7.200_rev	GGGGACCACTTTGTACAAGAAAGCTGGGTCTAAACATTGCGAATTAGTACGCATACG
7_hypothetical	P4	Tb927.4.4100_for	GGGGACAAGTTTGTACAAAAAAGCAGGCTATGTCAAGTGAGCAAGGTGGC	Tb927.4.4100_rev	GGGGACCACTTTGTACAAGAAAGCTGGGTTTAAGTACCCTGTTCTGAAGGAGTTATGC
7_hypothetical	P5	Tb927.7.4760_for	GGGGACAAGTTTGTACAAAAAAGCAGGCTATGCGACTCTCATATGCGGATCA	Tb927.7.4760_rev	GGGGACCACTTTGTACAAGAAAGCTGGGTCTACAAAACGTCCAACAGTCCTGA
7_hypothetical	P6	Tb927.10.3600_for	GGGGACAAGTTTGTACAAAAAAGCAGGCTATGAGCACCAACGGCGGC	Tb927.10.3600_rev	GGGGACCACTTTGTACAAGAAAGCTGGGTCTCACTTCGCTAAGTGATACATCATTCG
7_hypothetical	P7	Tb927.1.4040_for	GGGGACAAGTTTGTACAAAAAAGCAGGCTATGTCTGATGATGCTGCGGAC	Tb927.1.4040_rev	GGGGACCACTTTGTACAAGAAAGCTGGGTCTCAATCCACTAAAATGGTAACACGCC
7_hypothetical	P8	Tb927.11.14940_for	GGGGACAAGTTTGTACAAAAAAGCAGGCTATGTTTTTCAATCAACTCTTGTTACGTTTG	Tb927.11.14940_rev	GGGGACCACTTTGTACAAGAAAGCTGGGTCTACAGGGTCGTGATATTTGGATGC
7_hypothetical	P9	Tb927.10.7550_for	GGGGACAAGTTTGTACAAAAAAGCAGGCTATGCCGGCAAAGAAGGGTG	Tb927.10.7550_rev	GGGGACCACTTTGTACAAGAAAGCTGGGTCTATCCCTGCAGTGGCGCA
7_hypothetical	P11	Tb927.3.1270_for	GGGGACAAGTTTGTACAAAAAAGCAGGCTATGGTTGAACCGCACTTATTTTGTG	Tb927.3.1270_rev	GGGGACCACTTTGTACAAGAAAGCTGGGTTCAGAACTGAAGAAAATGCTCACAATGC
7_hypothetical	P12	Tb927.9.8050_for	GGGGACAAGTTTGTACAAAAAAGCAGGCTATGTCAAAAGACGGTGTATCACCC	Tb927.9.8050_rev	GGGGACCACTTTGTACAAGAAAGCTGGGTTTAACACACCAAAAGTTCGCAAATCAC
7_hypothetical	P13	Tb927.10.1340_for	GGGGACAAGTTTGTACAAAAAAGCAGGCTATGCGGCGATGGCTTAATATCG	Tb927.10.1340_rev	GGGGACCACTTTGTACAAGAAAGCTGGGTTCATTTCGCTGGTGCCACTTT
7_hypothetical	P14	Tb927.10.3300_for	GGGGACAAGTTTGTACAAAAAAGCAGGCTATGACAGAGGTAGCCGTTGATTGT	Tb927.10.3300_rev	GGGGACCACTTTGTACAAGAAAGCTGGGTCTCACTCTTTTATCAACTGTGTTGCTCT
7_hypothetical	P15	Tb927.10.11860_for	GGGGACAAGTTTGTACAAAAAAGCAGGCTATGAGAAGCGCGCAGTTCC	Tb927.10.11860_rev	GGGGACCACTTTGTACAAGAAAGCTGGGTTTATTCAACCGGTGGCGGTCC
7_hypothetical	P16	Tb927.10.600_for	GGGGACAAGTTTGTACAAAAAAGCAGGCTATGTTGTACACTAGGCGGTTGATG	Tb927.10.600_rev	GGGGACCACTTTGTACAAGAAAGCTGGGTCTAATGAACACCATACGTCACGTTCT
7_hypothetical	P17	Tb927.7.970_for	GGGGACAAGTTTGTACAAAAAAGCAGGCTATGGATCTCGATGACGAGTTCATTACA	Tb927.7.970_rev	GGGGACCACTTTGTACAAGAAAGCTGGGTTTACACCTCAGGCCGTTTCTTTTT
7_hypothetical	P18	Tb927.8.8000_for	GGGGACAAGTTTGTACAAAAAAGCAGGCTATGAAAAGCAACCGCAAGAAGGA	Tb927.8.8000_rev	GGGGACCACTTTGTACAAGAAAGCTGGGTCTCACATATTCACGTAGGAAACAGAAGG
7_hypothetical	P19	Tb927.11.15740_for	GGGGACAAGTTTGTACAAAAAAGCAGGCTATGAAAGGTGGTCCCTTGACTAGC	Tb927.11.15740_rev	GGGGACCACTTTGTACAAGAAAGCTGGGTCTACTGGGGCACACCGGT
7_hypothetical	P20	Tb927.4.2650_for	GGGGACAAGTTTGTACAAAAAAGCAGGCTATGATTCGGGATGCAGAGTTTTGG	Tb927.4.2650_rev	GGGGACCACTTTGTACAAGAAAGCTGGGTCTATAACACCACACAACACCTTTCCACA
7_hypothetical	P21	Tb927.9.15270_for	GGGGACAAGTTTGTACAAAAAAGCAGGCTATGGTTCGGTTTCAGCCATGG	Tb927.9.15270_rev	GGGGACCACTTTGTACAAGAAAGCTGGGTTTAATGAGGTTCCCACAATCCTCTCA
7_hypothetical	P22	Tb927.7.490_for	GGGGACAAGTTTGTACAAAAAAGCAGGCTATGACAAAATTAGAAGCAAATGCAGCG	Tb927.7.490_rev	GGGGACCACTTTGTACAAGAAAGCTGGGTTTAATACGTGTGACCGTTGAGCAC
7_hypothetical	P23	Tb927.11.8000_for	GGGGACAAGTTTGTACAAAAAAGCAGGCTATGTTCGCTGCTGATAGCCATTTAGGTG	Tb927.11.8000_rev	GGGGACCACTTTGTACAAGAAAGCTGGGTCTAAGCTACATCTTCTTCCGTTAATGCA
7_hypothetical	P24	Tb927.9.15450_for	GGGGACAAGTTTGTACAAAAAAGCAGGCTATGCCAGCAAAAAGGCGACT	Tb927.9.15450_rev	GGGGACCACTTTGTACAAGAAAGCTGGGTTCAAAAGGCGGTCCGCTC
7_known	A1	Tb927.8.1780_for	GGGGACAAGTTTGTACAAAAAAGCAGGCTATGGACCATAAAACTGCTACGGTAGA	Tb927.8.1780_rev	GGGGACCACTTTGTACAAGAAAGCTGGGTTCAGCGCTTTTGAAGGTTTGAGC
7_known	A2	Tb927.11.4430_for	GGGGACAAGTTTGTACAAAAAAGCAGGCTATGGATTAATCCCACACATAACGAGAAC	Tb927.11.4430_rev	GGGGACCACTTTGTACAAGAAAGCTGGGTTTATAATAAAAATGTTTCCTGGTGAAAGTC
7_known	A3	Tb927.9.2240_for	GGGGACAAGTTTGTACAAAAAAGCAGGCTATGGGGTGCAACACTTCCACC	Tb927.9.2240_rev	GGGGACCACTTTGTACAAGAAAGCTGGGTTCAATTGACAACTCCTGTGCATTCT
7_known	A4	Tb927.4.3410_for	GGGGACAAGTTTGTACAAAAAAGCAGGCTATGGTTTCTGAAGCGGGGAGG	Tb927.4.3410_rev	GGGGACCACTTTGTACAAGAAAGCTGGGTTTACCTGGGTCAGCTAATGCATATGA
7_known	A5	Tb927.10.5770_for	GGGGACAAGTTTGTACAAAAAAGCAGGCTATGTCAGCTAAGGCGCAACAA	Tb927.10.5770_rev	GGGGACCACTTTGTACAAGAAAGCTGGGTCTCAACTATAGAGATCGTCATCTCCTGC
7_known	A6	Tb927.8.2610_for	GGGGACAAGTTTGTACAAAAAAGCAGGCTATGATGACGACCGCAACGC	Tb927.8.2610_rev	GGGGACCACTTTGTACAAGAAAGCTGGGTCTAGTGCACAGTAAAGTGCCGC

7_known	A7	Tb927.11.6790_for	GGGGACAAGTTTGTACAAAAAAGCAGGCTATGGTCAAGAAGCGTGAGCCC	Tb927.11.6790_rev	GGGGACCACTTTGTACAAGAAAGCTGGGTTTATTCCGTCCATGCTGATACAACAC
7_known	A8	Tb927.11.11340_for	GGGGACAAGTTTGTACAAAAAAGCAGGCTATGTCTGACGGGGTGCAGT	Tb927.11.11340_rev	GGGGACCACTTTGTACAAGAAAGCTGGGTTCAAAACTCTGTGTTGTCGCCG
7_known	A9	Tb927.10.14990_for	GGGGACAAGTTTGTACAAAAAAGCAGGCTATGCGGTTGAATATTACGATTCCCAA	Tb927.10.14990_rev	GGGGACCACTTTGTACAAGAAAGCTGGGTCTACTGGTAGGGCTTGGCACC
7_known	A10	Tb927.5.1300_for	GGGGACAAGTTTGTACAAAAAAGCAGGCTATGTCAAGTGAAGCTGCTAGTGGC	Tb927.5.1300_rev	GGGGACCACTTTGTACAAGAAAGCTGGGTTTAGTCCAGTTCCTTCAGCACTTCA
7_known	A11	Tb927.10.9770_for	GGGGACAAGTTTGTACAAAAAAGCAGGCTATGACGACCCACTTGTGGGC	Tb927.10.9770_rev	GGGGACCACTTTGTACAAGAAAGCTGGGTTCAACGCTTTCGCCTGTCC
7_known	A12	Tb927.10.640_for	GGGGACAAGTTTGTACAAAAAAGCAGGCTATGAAGCCATGTGTTTCCGCTG	Tb927.10.640_rev	GGGGACCACTTTGTACAAGAAAGCTGGGTTCACGTCAGCAGAGAAATGTGG
7_known	A13	Tb927.9.5680_for	GGGGACAAGTTTGTACAAAAAAGCAGGCTATGGTGAATGGTCTCCCACCG	Tb927.9.5680_rev	GGGGACCACTTTGTACAAGAAAGCTGGGTTCACAATATCACGGCTGGGGG
7_known	A14	Tb927.6.950_for	GGGGACAAGTTTGTACAAAAAAGCAGGCTATGAAGGAAAGTGACGGTTTGTTATTAGC	Tb927.6.950_rev	GGGGACCACTTTGTACAAGAAAGCTGGGTCTATGATACTTCGGTGCTTTTTAAGCTCT
7_known	A15	Tb927.7.3280_for	GGGGACAAGTTTGTACAAAAAAGCAGGCTATGCAGTCGGGACTTTGCCA	Tb927.7.3280_rev	GGGGACCACTTTGTACAAGAAAGCTGGGTTCATATGGTGGTCTTCACTTCGGA
7_known	A16	Tb927.8.6350_for	GGGGACAAGTTTGTACAAAAAAGCAGGCTATGACTGCTCAAACCCCCTCA	Tb927.8.6350_rev	GGGGACCACTTTGTACAAGAAAGCTGGGTTCAACTCCCTCGCCTTGCC
7_known	A17	Tb927.4.3910_for	GGGGACAAGTTTGTACAAAAAAGCAGGCTATGCAGGACGAACAGCATGAG	Tb927.4.3910_rev	GGGGACCACTTTGTACAAGAAAGCTGGGTTTAATTCGGTTGCTGCTGCACC
7_known	A18	Tb927.9.14330_for	GGGGACAAGTTTGTACAAAAAAGCAGGCTATGATTACCCTTTTGGACGAGGGA	Tb927.9.14330_rev	GGGGACCACTTTGTACAAGAAAGCTGGGTTCACGCACGGCGGGACTT
7_known	A19	Tb927.2.280_for	GGGGACAAGTTTGTACAAAAAAGCAGGCTATGTGGAATTCAATGCGGCGT	Tb927.2.280_rev	GGGGACCACTTTGTACAAGAAAGCTGGGTTCAGGCAGCGGGGCCACC
7_known	A21	Tb927.2.380_for	GGGGACAAGTTTGTACAAAAAAGCAGGCTATGTGGAATTCAATAAGGCGTTTCTATACT	Tb927.2.380_rev	GGGGACCACTTTGTACAAGAAAGCTGGGTTCAGGCAGCGGGGCCACC
7_known	A22	Tb927.3.4280_for	GGGGACAAGTTTGTACAAAAAAGCAGGCTATGGACGAAGGTTTGGATGATATTGC	Tb927.3.4280_rev	GGGGACCACTTTGTACAAGAAAGCTGGGTTCAAACACACTCACTGGTCATCAC
7_known	A23	Tb927.11.12240_for	GGGGACAAGTTTGTACAAAAAAGCAGGCTATGTTGTCATCTTCGTCGCCG	Tb927.11.12240_rev	GGGGACCACTTTGTACAAGAAAGCTGGGTTCATACGAGTGCGTCTTGTTCAC
7_known	A24	Tb927.9.6150_for	GGGGACAAGTTTGTACAAAAAAGCAGGCTATGAACTCCGGTAGCCCAAGT	Tb927.9.6150_rev	GGGGACCACTTTGTACAAGAAAGCTGGGTTCATGCCTTACGGAAATTTAAGTGGAG
7_known	B1	Tb927.4.3940_for	GGGGACAAGTTTGTACAAAAAAGCAGGCTATGACGAGCAGCGGTGGT	Tb927.4.3940_rev	GGGGACCACTTTGTACAAGAAAGCTGGGTTTACTCACCCAAATCGAACTCTTTCAG
7_known	B2	Tb927.11.12220_for	GGGGACAAGTTTGTACAAAAAAGCAGGCTATGCCCTTCAGCAAAGCATGG	Tb927.11.12220_rev	GGGGACCACTTTGTACAAGAAAGCTGGGTTCAGAAGGTGTCGCTTCCGG
7_known	B3	Tb927.6.2230_for	GGGGACAAGTTTGTACAAAAAAGCAGGCTATGTTGCGGCCATTCGTCG	Tb927.6.2230_rev	GGGGACCACTTTGTACAAGAAAGCTGGGTTTACCTAGTTTGGTGGGAAAGTACCT
7_known	B4	Tb927.6.3790_for	GGGGACAAGTTTGTACAAAAAAGCAGGCTATGGGCGCCAAAGCAAGG	Tb927.6.3790_rev	GGGGACCACTTTGTACAAGAAAGCTGGGTTTACTTCATCGACACACTTTGCCC
7_known	B5	Tb927.5.3430_for	GGGGACAAGTTTGTACAAAAAAGCAGGCTATGCACGTTAATGTCGGACATATTGT	Tb927.5.3430_rev	GGGGACCACTTTGTACAAGAAAGCTGGGTCTAATCAATTTCTACAACCTCGTCACTATC
7_known	B6	Tb927.9.3460_for	GGGGACAAGTTTGTACAAAAAAGCAGGCTATGGGAAGGAAGGATGACGCC	Tb927.9.3460_rev	GGGGACCACTTTGTACAAGAAAGCTGGGTTTACTTACGCCGCGGTGC
7_known	B7	Tb927.10.910_for	GGGGACAAGTTTGTACAAAAAAGCAGGCTATGGTAGTACTGCGGCCAAAGA	Tb927.10.910_rev	GGGGACCACTTTGTACAAGAAAGCTGGGTTCAATCATCGTCCACCATTAGATTGTT
7_known	B8	Tb927.11.2950_for	GGGGACAAGTTTGTACAAAAAAGCAGGCTATGCTGACAACATTTGAGCAGCA	Tb927.11.2950_rev	GGGGACCACTTTGTACAAGAAAGCTGGGTTCACCATTTCGGTTGTGATGTCAC
7_known	B9	Tb927.8.3220_for	GGGGACAAGTTTGTACAAAAAAGCAGGCTATGGGGATTAAAGGGTTATGGGGAG	Tb927.8.3220_rev	GGGGACCACTTTGTACAAGAAAGCTGGGTCTACCGTCGCGTATAAGATAACAGC
7_known	B10	Tb927.5.1090_for	GGGGACAAGTTTGTACAAAAAAGCAGGCTATGGCCACGGCTGTTGTTG	Tb927.5.1090_rev	GGGGACCACTTTGTACAAGAAAGCTGGGTTTAGAAGGACGGATCATATTTATCGGCC
7_known	B11	Tb927.11.1740_for	GGGGACAAGTTTGTACAAAAAAGCAGGCTATGGACTTACAACAGGGTGACGG	Tb927.11.1740_rev	GGGGACCACTTTGTACAAGAAAGCTGGGTCTATATTCCCGGGAGGTCAATTTCATC
7_known	B12	Tb927.11.11570_for	GGGGACAAGTTTGTACAAAAAAGCAGGCTATGGACATATCAGCCACGCGC	Tb927.11.11570_rev	GGGGACCACTTTGTACAAGAAAGCTGGGTTCAAAACATTCCCTCCTCTTCTTCTTC
7_known	B13	Tb927.5.890_for	GGGGACAAGTTTGTACAAAAAAGCAGGCTATGACGAAAGGTGGGAAAGTAGCT	Tb927.5.890_rev	GGGGACCACTTTGTACAAGAAAGCTGGGTTCATTCGTAATGGAACCGCTTCG
7_known	B14	Tb927.4.760_for	GGGGACAAGTTTGTACAAAAAAGCAGGCTATGAACAGCCAAAAGCTTCGTGA	Tb927.4.760_rev	GGGGACCACTTTGTACAAGAAAGCTGGGTCTAAACCTCTTGAGACACCTGCAG
7_known	B15	Tb927.10.6220_for	GGGGACAAGTTTGTACAAAAAAGCAGGCTATGGGGATTGCCGGTTTTTACC	Tb927.10.6220_rev	GGGGACCACTTTGTACAAGAAAGCTGGGTTCAGCGCCCAATGACTCTGT
7_known	B16	Tb927.8.5980_for	GGGGACAAGTTTGTACAAAAAAGCAGGCTATGAAGTTGTTCATTGAAGACGTGCC	Tb927.8.5980_rev	GGGGACCACTTTGTACAAGAAAGCTGGGTTCACTCTCGGGACCGCTTT
7_known	B17	Tb927.4.2730_for	GGGGACAAGTTTGTACAAAAAAGCAGGCTATGACCACTCTATCTTTAGATTCAACAGC	Tb927.4.2730_rev	GGGGACCACTTTGTACAAGAAAGCTGGGTTCATAACTCACCCCTCATACCTATTTCTT
7_known	B18	Tb927.2.4130_for	GGGGACAAGTTTGTACAAAAAAGCAGGCTATGCGTCGCTTGGAAACCAT	Tb927.2.4130_rev	GGGGACCACTTTGTACAAGAAAGCTGGGTTTACGGCCACGTGGAACGA
7_known	B19	Tb927.11.1110_for	GGGGACAAGTTTGTACAAAAAAGCAGGCTATGCAGCGGTCGTTGCGC	Tb927.11.1110_rev	GGGGACCACTTTGTACAAGAAAGCTGGGTTCATCTACGCTGCGGTGTCG
7_known	B20	Tb927.11.11430_for	GGGGACAAGTTTGTACAAAAAAGCAGGCTATGGAGCAGACCAACCATGACG	Tb927.11.11430_rev	GGGGACCACTTTGTACAAGAAAGCTGGGTTTATGTAACGTACGAAAACGTGTCTGT
7_known	B21	Tb927.2.3620_for	GGGGACAAGTTTGTACAAAAAAGCAGGCTATGGCTGACGGATCACATGACA	Tb927.2.3620_rev	GGGGACCACTTTGTACAAGAAAGCTGGGTCTACGTTTCTTTATGTTTCAACCTCTTCG
7_known	B22	Tb927.7.5450_for	GGGGACAAGTTTGTACAAAAAAGCAGGCTATGATATCTGGTTTTGCAGATTTCTTTAAC	Tb927.7.5450_rev	GGGGACCACTTTGTACAAGAAAGCTGGGTTCACACAGTGTCACCTTGTTGATAG
7_known	B23	Tb927.10.1900_for	GGGGACAAGTTTGTACAAAAAAGCAGGCTATGTTTCGGCAAACAGTTACATCACT	Tb927.10.1900_rev	GGGGACCACTTTGTACAAGAAAGCTGGGTTCACCCACCGCCCGCAAC
7_known	B24	Tb927.10.12980_for	GGGGACAAGTTTGTACAAAAAAGCAGGCTATGTGGAAACCGAAACAGCGC	Tb927.10.12980_rev	GGGGACCACTTTGTACAAGAAAGCTGGGTTCAAGGATCACGCACGTCTGG
7_known	C1	Tb927.10.13630_for	GGGGACAAGTTTGTACAAAAAAGCAGGCTATGATGCTATCGCTTGTGCTATCG	Tb927.10.13630_rev	GGGGACCACTTTGTACAAGAAAGCTGGGTCTATCTCTTCAGCACAATGGTCCAG
7_known	C2	Tb927.3.620_for	GGGGACAAGTTTGTACAAAAAAGCAGGCTATGTCACTAGCGGGTTCCGA	Tb927.3.620_rev	GGGGACCACTTTGTACAAGAAAGCTGGGTTTAGGCATCGAATTCAGTAAATTGCTGG
7_known	C3	Tb927.11.2270_for	GGGGACAAGTTTGTACAAAAAAGCAGGCTATGTCTACGGAAGCAGAGGATGAT	Tb927.11.2270_rev	GGGGACCACTTTGTACAAGAAAGCTGGGTTCAAATTGGTGAGGCCCGTG
7_known	C4	Tb927.7.7120_for	GGGGACAAGTTTGTACAAAAAAGCAGGCTATGAATGGGATTCATTTAAATTCGACTATT	Tb927.7.7120_rev	GGGGACCACTTTGTACAAGAAAGCTGGGTTCATAAGGATGTCTCGCAACTCTCG
7_known	C5	Tb927.10.12710_for	GGGGACAAGTTTGTACAAAAAAGCAGGCTATGTCAGTCTTCGGTGTGGACT	Tb927.10.12710_rev	GGGGACCACTTTGTACAAGAAAGCTGGGTCTAGTCAAGCTGATCATCCGACG
7_known	C6	Tb927.5.940_for	GGGGACAAGTTTGTACAAAAAAGCAGGCTATGTTTTCCGGTCCTGCTGAAG	Tb927.5.940_rev	GGGGACCACTTTGTACAAGAAAGCTGGGTTCAAAGAGTGACTATGTTTGATTTGTTATG
7_known	C7	Tb927.2.3030_for	GGGGACAAGTTTGTACAAAAAAGCAGGCTATGCGAAAGCTGGGTTCGG	Tb927.2.3030_rev	GGGGACCACTTTGTACAAGAAAGCTGGGTTCATTCTGTGAGTTGCGGACCC
7_known	C8	Tb927.10.15880_for	GGGGACAAGTTTGTACAAAAAAGCAGGCTATGTATTTAAAACCATATAACAATCCCTAC	Tb927.10.15880_rev	GGGGACCACTTTGTACAAGAAAGCTGGGTTTAGCAACGCCCCTCCTGT
7_known	C9	Tb927.2.3930_for	GGGGACAAGTTTGTACAAAAAAGCAGGCTATGCTTAGCAACAGGACCCTCA	Tb927.2.3930_rev	GGGGACCACTTTGTACAAGAAAGCTGGGTTCAAATGAACTGAACCCATTCATCTTCG
7_known	C10	Tb927.5.2790_for	GGGGACAAGTTTGTACAAAAAAGCAGGCTATGCTTTTGTTCCGCCCCAC	Tb927.5.2790_rev	GGGGACCACTTTGTACAAGAAAGCTGGGTTCATTTTGATCTTCGTTTTTTATCTGCTCC
7_known	C11	Tb927.9.1340_for	GGGGACAAGTTTGTACAAAAAAGCAGGCTATGAGCATCAGTGAGTCGGACA	Tb927.9.1340_rev	GGGGACCACTTTGTACAAGAAAGCTGGGTCTACTGTGGCTGCTTTACTTCCTC
7_known	C12	Tb927.5.4310_for	GGGGACAAGTTTGTACAAAAAAGCAGGCTATGCAATCGTTGGGGAGGAGC	Tb927.5.4310_rev	GGGGACCACTTTGTACAAGAAAGCTGGGTTTAGTAACCTTGAACAACCTGCCTG
7_known	C13	Tb927.3.1440_for	GGGGACAAGTTTGTACAAAAAAGCAGGCTATGGGTCGCCACCATGATGT	Tb927.3.1440_rev	GGGGACCACTTTGTACAAGAAAGCTGGGTCTACTTACTTCCGAAAGTTTTGCTCCA
7_known	C14	Tb927.3.2610_for	GGGGACAAGTTTGTACAAAAAAGCAGGCTATGCGCCTTTGTGGTGATGAA	Tb927.3.2610_rev	GGGGACCACTTTGTACAAGAAAGCTGGGTTTAGTTTGAGGTACCAGGAGCTAGAAG
7_known	C15	Tb927.3.1490_for	GGGGACAAGTTTGTACAAAAAAGCAGGCTATGGCAACACAGTTAAATAGTGATACGG	Tb927.3.1490_rev	GGGGACCACTTTGTACAAGAAAGCTGGGTTCAGATTGTTTTGACTCTAACACCCC
7_known	C16	Tb927.7.7480_for	GGGGACAAGTTTGTACAAAAAAGCAGGCTATGGAACGCCTGCAGGTGC	Tb927.7.7480_rev	GGGGACCACTTTGTACAAGAAAGCTGGGTTCAAATATTAGTAGGAACCACGTCCCG
7_known	C17	Tb927.5.640_for	GGGGACAAGTTTGTACAAAAAAGCAGGCTATGAAGCGCCTGCCTGTACG	Tb927.5.640_rev	GGGGACCACTTTGTACAAGAAAGCTGGGTTCAGATTATAGTAGAATCCCCGTCCCA
7_known	C18	Tb927.11.8980_for	GGGGACAAGTTTGTACAAAAAAGCAGGCTATGAAAGGGTTCCGGTGTGC	Tb927.11.8980_rev	GGGGACCACTTTGTACAAGAAAGCTGGGTTTAACTGTCGTATATACCTAAGTTCTTCAT
7_known	C19	Tb927.1.1690_for	GGGGACAAGTTTGTACAAAAAAGCAGGCTATGGGGCTACATTGGCCCC	Tb927.1.1690_rev	GGGGACCACTTTGTACAAGAAAGCTGGGTTCACGCACCAACCGAGATGC
7_known	C20	Tb927.11.230_for	GGGGACAAGTTTGTACAAAAAAGCAGGCTATGCTGCGCGCTTCGGCT	Tb927.11.230_rev	GGGGACCACTTTGTACAAGAAAGCTGGGTTCACAGAACTTGAGAAAAGTGCTTGT
7_known	C21	Tb927.11.12430_for	GGGGACAAGTTTGTACAAAAAAGCAGGCTATGCCGAGGCCACTCCGA	Tb927.11.12430_rev	GGGGACCACTTTGTACAAGAAAGCTGGGTTTAAGGTTGGAGGAATACAACCCGC
7_known	C22	Tb927.11.14790_for	GGGGACAAGTTTGTACAAAAAAGCAGGCTATGCTCCTCAACTCCACCAGG	Tb927.11.14790_rev	GGGGACCACTTTGTACAAGAAAGCTGGGTTCACTTGTGGTGTTTCCACGTC
7_known	C23	Tb927.10.14890_for	GGGGACAAGTTTGTACAAAAAAGCAGGCTATGTCTGTGGAACAACCCGAGC	Tb927.10.14890_rev	GGGGACCACTTTGTACAAGAAAGCTGGGTTTATGGATTCTTCTTGATCTGGCCAAG
7_known	C24	Tb927.7.3490_for	GGGGACAAGTTTGTACAAAAAAGCAGGCTATGTACGTGGAGAAGAGCCCG	Tb927.7.3490_rev	GGGGACCACTTTGTACAAGAAAGCTGGGTTTACTTGTATAATCCCCGCACGAAATT
7_known	D1	Tb927.9.7700_for	GGGGACAAGTTTGTACAAAAAAGCAGGCTATGTGGGTCTTTAGCGACCGA	Tb927.9.7700_rev	GGGGACCACTTTGTACAAGAAAGCTGGGTTTAGGCACTAGGCACAGTGGAG
7_known	D2	Tb927.5.910_for	GGGGACAAGTTTGTACAAAAAAGCAGGCTATGACGAAAGGTGGGAAAGTAGCT	Tb927.5.910_rev	GGGGACCACTTTGTACAAGAAAGCTGGGTCTAACCCATCTGACGCATATACGC
7_known	D4	Tb927.7.2970_for	GGGGACAAGTTTGTACAAAAAAGCAGGCTATGCCCGTCTCCCTTACTTCAG	Tb927.7.2970_rev	GGGGACCACTTTGTACAAGAAAGCTGGGTTTAATTCTCGGATTCGCTGGTTTCA
7_known	D5	Tb927.8.7980_for	GGGGACAAGTTTGTACAAAAAAGCAGGCTATGATTAAATTGTCACCACTGCTTCTAAAA	Tb927.8.7980_rev	GGGGACCACTTTGTACAAGAAAGCTGGGTTTACTTCACCAGAAGCCCTCCC
7_known	D6	Tb927.4.4380_for	GGGGACAAGTTTGTACAAAAAAGCAGGCTATGATTAAAATGTCACCGCTGCTTCT	Tb927.4.4380_rev	GGGGACCACTTTGTACAAGAAAGCTGGGTTTACTTCACCAGAAGCCCTCCC
7_known	D7	Tb927.10.7310_for	GGGGACAAGTTTGTACAAAAAAGCAGGCTATGTTCACATCGGTGCCTGTAGC	Tb927.10.7310_rev	GGGGACCACTTTGTACAAGAAAGCTGGGTCTAGTTTTTCTGACAGTTGTACGCCT
7_known	D8	Tb927.5.4270_for	GGGGACAAGTTTGTACAAAAAAGCAGGCTATGGATGAAAGGCAGATGGAGGA	Tb927.5.4270_rev	GGGGACCACTTTGTACAAGAAAGCTGGGTTTATTTACTTTTTTGTGGGGTGGGATTATT
7_known	D9	Tb927.5.295b_for	GGGGACAAGTTTGTACAAAAAAGCAGGCTATGTTTTCCAAACGCTGTTTTGCA	Tb927.5.295b_rev	GGGGACCACTTTGTACAAGAAAGCTGGGTCTATGCAATGTTACCACCCCTATTGT
7_known	D10	Tb927.2.100_for	GGGGACAAGTTTGTACAAAAAAGCAGGCTATGTCAAGGGCAAATAGTCCCGC	Tb927.2.100_rev	GGGGACCACTTTGTACAAGAAAGCTGGGTCTATGCATTGTTACCACCTCTATTGTTAAC
7_known	D11	Tb927.5.2970_for	GGGGACAAGTTTGTACAAAAAAGCAGGCTATGTTGACGCTCCGTGAACTAGA	Tb927.5.2970_rev	GGGGACCACTTTGTACAAGAAAGCTGGGTTTAAAAGACTGTGGGCGTCGG
7_known	D12	Tb927.2.3780_for	GGGGACAAGTTTGTACAAAAAAGCAGGCTATGCCGCCTAAAGGACCAAAG	Tb927.2.3780_rev	GGGGACCACTTTGTACAAGAAAGCTGGGTCTATCGAGGCGGCACCTTCA
7_known	D13	Tb927.1.180_for	GGGGACAAGTTTGTACAAAAAAGCAGGCTATGTCAAGAGCAAATAGTCCCGC	Tb927.1.180_rev	GGGGACCACTTTGTACAAGAAAGCTGGGTCTATGCATTGTTACCACCTCTATTGTTAAC
7_known	D14	Tb927.1.220_for	GGGGACAAGTTTGTACAAAAAAGCAGGCTATGTCAAGAGCAAATAGTCCCGC	Tb927.1.220_rev	GGGGACCACTTTGTACAAGAAAGCTGGGTCTATGCATTGTTACCACCTCTATTGTTAAC
7_known	D15	Tb927.11.4990_for	GGGGACAAGTTTGTACAAAAAAGCAGGCTATGGATTTAAACACAGTCGACGAGAG	Tb927.11.4990_rev	GGGGACCACTTTGTACAAGAAAGCTGGGTTCAATCAAGGTTAACCACAGGTTTACC
7_known	D16	Tb927.2.370_for	GGGGACAAGTTTGTACAAAAAAGCAGGCTATGTCAAGAGCAAATAGTCCCGC	Tb927.2.370_rev	GGGGACCACTTTGTACAAGAAAGCTGGGTCTATGCATTGTTACCACCTCTATTGTTAAC
7_known	D17	Tb927.10.15540_for	GGGGACAAGTTTGTACAAAAAAGCAGGCTATGCAGTCTGTGAGGTTCAGCG	Tb927.10.15540_rev	GGGGACCACTTTGTACAAGAAAGCTGGGTCTAATTAGCACTATTGTCGTTGCTGTCC
7_known	D18	Tb927.9.4500_for	GGGGACAAGTTTGTACAAAAAAGCAGGCTATGTCCATGCTCTGTTTGGCAC	Tb927.9.4500_rev	GGGGACCACTTTGTACAAGAAAGCTGGGTTCACTCACCGAGCTTTAGGTCC
7_known	D19	Tb927.11.12250_for	GGGGACAAGTTTGTACAAAAAAGCAGGCTATGAACAGGCATGCATTTGTGGA	Tb927.11.12250_rev	GGGGACCACTTTGTACAAGAAAGCTGGGTTCACGAAAGCCTACCGGCG
7_known	D20	Tb927.2.1380_for	GGGGACAAGTTTGTACAAAAAAGCAGGCTATGGGAATAACCCTAGCCAACAAAAA	Tb927.2.1380_rev	GGGGACCACTTTGTACAAGAAAGCTGGGTCTAACATTGAGAGCAGGGGACAGA
7_known	D21	Tb927.2.510_for	GGGGACAAGTTTGTACAAAAAAGCAGGCTATGTCAAGAGCAAATAGTCCCGC	Tb927.2.510_rev	GGGGACCACTTTGTACAAGAAAGCTGGGTTCACCTCCCCTGTGCCTTTT
7_known	D22	Tb927.11.7840_for	GGGGACAAGTTTGTACAAAAAAGCAGGCTATGTTGGAAACGGTGAAGCTTGT	Tb927.11.7840_rev	GGGGACCACTTTGTACAAGAAAGCTGGGTCTACGAACCACAATTCAGGCATCC
7_known	D23	Tb927.11.5070_for	GGGGACAAGTTTGTACAAAAAAGCAGGCTATGACCTCACGCTCTGATGCT	Tb927.11.5070_rev	GGGGACCACTTTGTACAAGAAAGCTGGGTTCATGCCAGTAACGCAGCC
7_known	D24	Tb927.5.4460_for	GGGGACAAGTTTGTACAAAAAAGCAGGCTATGAGTGATATCATACGAATTAAACGTCAT	Tb927.5.4460_rev	GGGGACCACTTTGTACAAGAAAGCTGGGTTTATGACTTTGTCTCGGTTGTTTGGC
7_known	E1	Tb927.10.3890_for	GGGGACAAGTTTGTACAAAAAAGCAGGCTATGCTGCAGTCCGATGTGTCT	Tb927.10.3890_rev	GGGGACCACTTTGTACAAGAAAGCTGGGTCTACATTGTAGCATTGCACAAAACACC
7_known	E2	Tb927.11.13640_for	GGGGACAAGTTTGTACAAAAAAGCAGGCTATGCGAAGGCAGGTGAAGAAG	Tb927.11.13640_rev	GGGGACCACTTTGTACAAGAAAGCTGGGTCTATTTCTCCCGTCTGCGGACG
7_known	E3	Tb927.7.1210_for	GGGGACAAGTTTGTACAAAAAAGCAGGCTATGCACGAACCTCGTGAAGTAGT	Tb927.7.1210_rev	GGGGACCACTTTGTACAAGAAAGCTGGGTCTAACGGGCACTTCGCCTG
7_known	E4	Tb927.8.7840_for	GGGGACAAGTTTGTACAAAAAAGCAGGCTATGACGACGAAGTCAGCCGT	Tb927.8.7840_rev	GGGGACCACTTTGTACAAGAAAGCTGGGTTCACACATTTTGGGGAATGGGAAG
7_known	E5	Tb927.6.5100_for	GGGGACAAGTTTGTACAAAAAAGCAGGCTATGTTTAACGTAAGAACGCCGATGT	Tb927.6.5100_rev	GGGGACCACTTTGTACAAGAAAGCTGGGTTTAAAAGCACTCCACAATATTTGCACAG
7_known	E6	Tb927.2.1210_for	GGGGACAAGTTTGTACAAAAAAGCAGGCTATGTCAAGGGCAAATAGTCCCGC	Tb927.2.1210_rev	GGGGACCACTTTGTACAAGAAAGCTGGGTTCACCCTCCTTGCGCTCC
7_known	E7	Tb927.11.9170_for	GGGGACAAGTTTGTACAAAAAAGCAGGCTATGGTCCAAACAGTACTTATGGTTGC	Tb927.11.9170_rev	GGGGACCACTTTGTACAAGAAAGCTGGGTCTAGTTTCGGTAGCCTCTTCCTCT
7_known	E8	Tb927.5.2140_for	GGGGACAAGTTTGTACAAAAAAGCAGGCTATGTTCAGTGAGCATGCTAGTGATG	Tb927.5.2140_rev	GGGGACCACTTTGTACAAGAAAGCTGGGTTCAAGACTCACCAGGCAAGAACA
7_known	E9	Tb927.4.2630_for	GGGGACAAGTTTGTACAAAAAAGCAGGCTATGAAAGGTGTAGGTACGTGGGA	Tb927.4.2630_rev	GGGGACCACTTTGTACAAGAAAGCTGGGTTCACCTTTTTCGTTTACCTTTCTGCT
7_known	E10	Tb927.11.12600_for	GGGGACAAGTTTGTACAAAAAAGCAGGCTATGGAGCACGAACAAAGGGTTT	Tb927.11.12600_rev	GGGGACCACTTTGTACAAGAAAGCTGGGTTCACTCTTGCTGCCCTTCACT
7_known	E11	Tb927.10.11760_for	GGGGACAAGTTTGTACAAAAAAGCAGGCTATGAGTTCAACCAAAGAGCCGC	Tb927.10.11760_rev	GGGGACCACTTTGTACAAGAAAGCTGGGTTCACTCGGCATCGAAGTGCT
7_known	E12	Tb927.7.6460_for	GGGGACAAGTTTGTACAAAAAAGCAGGCTATGACCCTCACAGGCAACATCA	Tb927.7.6460_rev	GGGGACCACTTTGTACAAGAAAGCTGGGTCTAACCAGTAAGAGGTACCTGATGTTTTT
7_known	E13	Tb927.2.560_for	GGGGACAAGTTTGTACAAAAAAGCAGGCTATGTCAAGAGCAAATAGTCCCGC	Tb927.2.560_rev	GGGGACCACTTTGTACAAGAAAGCTGGGTTCACCCTCCTTGCGCTCC
7_known	E14	Tb927.11.5550_for	GGGGACAAGTTTGTACAAAAAAGCAGGCTATGAAAAATCTTTCAATCTTCTCCGCAAG	Tb927.11.5550_rev	GGGGACCACTTTGTACAAGAAAGCTGGGTTTAAGGAACATCAAGTTTCTCGAGTGAA
7_known	E15	Tb927.6.3430_for	GGGGACAAGTTTGTACAAAAAAGCAGGCTATGGAGGATTACACTATTTACCGTCCA	Tb927.6.3430_rev	GGGGACCACTTTGTACAAGAAAGCTGGGTTCATACCTCACGGTTTTCAATCCAG
7_known	E16	Tb927.10.4570_for	GGGGACAAGTTTGTACAAAAAAGCAGGCTATGGTCAACTTCACCGTTGATGAG	Tb927.10.4570_rev	GGGGACCACTTTGTACAAGAAAGCTGGGTCTATAGCTTGTCCAGGAAGGTGTCG
7_known	E17	Tb927.10.1460_for	GGGGACAAGTTTGTACAAAAAAGCAGGCTATGAGATCTTGCTGTAGCTGTGGA	Tb927.10.1460_rev	GGGGACCACTTTGTACAAGAAAGCTGGGTCTACACTCTGACGAATAGGTTTCGG
7_known	E19	Tb927.11.10810_for	GGGGACAAGTTTGTACAAAAAAGCAGGCTATGAACGTCGCGTGGGGG	Tb927.11.10810_rev	GGGGACCACTTTGTACAAGAAAGCTGGGTTTATTTATGCGATGCAGGAATTCCGG
7_known	E20	Tb927.10.12660_for	GGGGACAAGTTTGTACAAAAAAGCAGGCTATGTCTGGTTGGGACGAACCC	Tb927.10.12660_rev	GGGGACCACTTTGTACAAGAAAGCTGGGTCTACAGCGTTGGCATGCAGG
7_known	E21	Tb927.11.10900_for	GGGGACAAGTTTGTACAAAAAAGCAGGCTATGGAAAACGGAAAGAATGACGAATTTAC	Tb927.11.10900_rev	GGGGACCACTTTGTACAAGAAAGCTGGGTTCACGACTTAATGCTCCTCGAAGC
7_known	E22	Tb927.11.12350_for	GGGGACAAGTTTGTACAAAAAAGCAGGCTATGAAGCGAATATTTAGGAGTTCGGTT	Tb927.11.12350_rev	GGGGACCACTTTGTACAAGAAAGCTGGGTCTACTCGTCGGAGGCACTTAGC
7_known	E23	Tb927.7.960_for	GGGGACAAGTTTGTACAAAAAAGCAGGCTATGACCAGAAATGCTGATGCACA	Tb927.7.960_rev	GGGGACCACTTTGTACAAGAAAGCTGGGTTCAGTTGTTGGTGGCATCATACAG
7_known	E24	Tb927.10.6940_for	GGGGACAAGTTTGTACAAAAAAGCAGGCTATGCCGACCTTGCCTTTGTC	Tb927.10.6940_rev	GGGGACCACTTTGTACAAGAAAGCTGGGTTTAAAGCACCTCCATAAGTGTCTTTGC
7_known	F2	Tb927.4.3950_for	GGGGACAAGTTTGTACAAAAAAGCAGGCTATGGGTTGTGGTGGATCAAAGGT	Tb927.4.3950_rev	GGGGACCACTTTGTACAAGAAAGCTGGGTTTATTCATCACTGTCAGTTTCATCACCC
7_known	F3	Tb927.2.6050_for	GGGGACAAGTTTGTACAAAAAAGCAGGCTATGTCGTCAAAAATGATGTCACCGC	Tb927.2.6050_rev	GGGGACCACTTTGTACAAGAAAGCTGGGTTTACTGCTCACCCCATTCGTCG
7_known	F4	Tb927.11.370_for	GGGGACAAGTTTGTACAAAAAAGCAGGCTATGACCGACGTTGACGGTGG	Tb927.11.370_rev	GGGGACCACTTTGTACAAGAAAGCTGGGTTTATTGTGGCGCTTTCGAAGTGG
7_known	F5	Tb927.9.7540_for	GGGGACAAGTTTGTACAAAAAAGCAGGCTATGATGCAGGCATCAGCAGATG	Tb927.9.7540_rev	GGGGACCACTTTGTACAAGAAAGCTGGGTTTAGGAAAAAGAGGAACCAACTAACTCTTC
7_known	F6	Tb927.1.120_for	GGGGACAAGTTTGTACAAAAAAGCAGGCTATGAATCAACAATTTCCTATTGAAGGAAGG	Tb927.1.120_rev	GGGGACCACTTTGTACAAGAAAGCTGGGTTCATCTCCTTCCACGTCGTCTC
7_known	F7	Tb927.8.4170_for	GGGGACAAGTTTGTACAAAAAAGCAGGCTATGTCTCGCGTACGTATTTTGAACC	Tb927.8.4170_rev	GGGGACCACTTTGTACAAGAAAGCTGGGTTTACGATTCGTCCAGCTTGGC
7_known	F9	Tb927.11.9270_for	GGGGACAAGTTTGTACAAAAAAGCAGGCTATGCTATCCAAGTTATTCCGGTATGACA	Tb927.11.9270_rev	GGGGACCACTTTGTACAAGAAAGCTGGGTTTAGCAGGAACCCGTCCGT
7_known	F10	Tb927.2.340_for	GGGGACAAGTTTGTACAAAAAAGCAGGCTATGAATCAACAGGTTCCTATTGAAGGAAG	Tb927.2.340_rev	GGGGACCACTTTGTACAAGAAAGCTGGGTTTACGCGTCTTCACCTCCTTCC
7_known	F11	Tb927.11.7770_for	GGGGACAAGTTTGTACAAAAAAGCAGGCTATGAGTGACGGGGATGAGGG	Tb927.11.7770_rev	GGGGACCACTTTGTACAAGAAAGCTGGGTCTACCAAGGTATTTCCCTAAAATCCTTGAA
7_known	F13	Tb927.11.2880_for	GGGGACAAGTTTGTACAAAAAAGCAGGCTATGAGTATTGAGGTTCATGTACGCATT	Tb927.11.2880_rev	GGGGACCACTTTGTACAAGAAAGCTGGGTTCAACGACGGCGGGAGCG
7_known	F14	Tb927.10.6310_for	GGGGACAAGTTTGTACAAAAAAGCAGGCTATGAATGATTATTTAGCGAATGAGCTGGT	Tb927.10.6310_rev	GGGGACCACTTTGTACAAGAAAGCTGGGTTTACTGCTGCACATGACCAGTC
7_known	F15	Tb927.10.1990_for	GGGGACAAGTTTGTACAAAAAAGCAGGCTATGGTGGACAAGGAGAATCAGGT	Tb927.10.1990_rev	GGGGACCACTTTGTACAAGAAAGCTGGGTTCATGGCAACGCGTCGTT
7_known	F16	Tb927.8.6880_for	GGGGACAAGTTTGTACAAAAAAGCAGGCTATGCGTCACACTCGTTTTTTTCAG	Tb927.8.6880_rev	GGGGACCACTTTGTACAAGAAAGCTGGGTTTATGTGGAAGGTGTTAGTAGAAGTTGTC
7_known	F17	Tb927.2.4060_for	GGGGACAAGTTTGTACAAAAAAGCAGGCTATGAAAAATGGCTCCTCGAGTGG	Tb927.2.4060_rev	GGGGACCACTTTGTACAAGAAAGCTGGGTTTATGTGGTCGCCTTTCGAACTAC
7_known	F18	Tb927.3.4220_for	GGGGACAAGTTTGTACAAAAAAGCAGGCTATGGACGCACCGCTGCAG	Tb927.3.4220_rev	GGGGACCACTTTGTACAAGAAAGCTGGGTCTACTCAAAATCCTCATCGTCGTCG
7_known	F19	Tb927.10.2900_for	GGGGACAAGTTTGTACAAAAAAGCAGGCTATGAGCTCCTTGACGGAATTGTTAAC	Tb927.10.2900_rev	GGGGACCACTTTGTACAAGAAAGCTGGGTTTACACCACCTTTGAAAGTTGTTGAAGT
7_known	F20	Tb927.10.12440_for	GGGGACAAGTTTGTACAAAAAAGCAGGCTATGGGACGAAGGACACATGTGG	Tb927.10.12440_rev	GGGGACCACTTTGTACAAGAAAGCTGGGTCTACATTATTACACAGTAATGGCAACCCT
7_known	F21	Tb927.8.2810_for	GGGGACAAGTTTGTACAAAAAAGCAGGCTATGGGTGTCCCATTACTTCTCACT	Tb927.8.2810_rev	GGGGACCACTTTGTACAAGAAAGCTGGGTTTAGGTCCCCAGGGTGGCA
7_known	F22	Tb927.11.11640_for	GGGGACAAGTTTGTACAAAAAAGCAGGCTATGGACCTCGTGCCAACAGG	Tb927.11.11640_rev	GGGGACCACTTTGTACAAGAAAGCTGGGTCTACTGCTTCGTAATATCTGGGTTATAGTT

7_known	F23	Tb927.2.5980_for	GGGGACAAGTTTGTACAAAAAAGCAGGCTATGGCACATTCAGATCGCCAAT	Tb927.2.5980_rev	GGGGACCACTTTGTACAAGAAAGCTGGGTTTATTCCCATTCGTCACTGGTAAGC
7_known	F24	Tb927.7.1030_for	GGGGACAAGTTTGTACAAAAAAGCAGGCTATGCACTGTTGTAAGGTTTTTTCGTTT	Tb927.7.1030_rev	GGGGACCACTTTGTACAAGAAAGCTGGGTCTAGTCCATCGCGAACAGTTTGG
7_known	G1	Tb927.3.4750_for	GGGGACAAGTTTGTACAAAAAAGCAGGCTATGCCAACTCTCCCCTCAGATG	Tb927.3.4750_rev	GGGGACCACTTTGTACAAGAAAGCTGGGTTCACCGCGGAAAGAACAGGT
7_known	G3	Tb927.11.3570_for	GGGGACAAGTTTGTACAAAAAAGCAGGCTATGTCCACGATCGAACGGGA	Tb927.11.3570_rev	GGGGACCACTTTGTACAAGAAAGCTGGGTCTATAACGCTGCAACCGAAAGAAAC
7_known	G4	Tb927.2.1860_for	GGGGACAAGTTTGTACAAAAAAGCAGGCTATGCTGCTGCCCACTCTCG	Tb927.2.1860_rev	GGGGACCACTTTGTACAAGAAAGCTGGGTTCACACCAGCTGTAAAGGCGG
7_known	G5	Tb927.11.1510_for	GGGGACAAGTTTGTACAAAAAAGCAGGCTATGGGAACTACAATGTCAGGAAACC	Tb927.11.1510_rev	GGGGACCACTTTGTACAAGAAAGCTGGGTCTAGCTCGCGAAGCTTAGGC
7_known	G6	Tb927.1.3300_for	GGGGACAAGTTTGTACAAAAAAGCAGGCTATGTCGTTGGGTACGCCATGT	Tb927.1.3300_rev	GGGGACCACTTTGTACAAGAAAGCTGGGTCTATGCACTATTTAACTTGTGTGCACTATA
7_known	G7	Tb927.10.2040_for	GGGGACAAGTTTGTACAAAAAAGCAGGCTATGCGGGCTAACGACATTGTG	Tb927.10.2040_rev	GGGGACCACTTTGTACAAGAAAGCTGGGTTCATCCCACATTTGTCTGTGCC
7_known	G8	Tb927.4.4210_for	GGGGACAAGTTTGTACAAAAAAGCAGGCTATGCGCCGCTACTTGCTC	Tb927.4.4210_rev	GGGGACCACTTTGTACAAGAAAGCTGGGTTCACACGCCCTTCTTACGGT
7_known	G9	Tb927.10.3500_for	GGGGACAAGTTTGTACAAAAAAGCAGGCTATGGGGCGTGATAGTCGCG	Tb927.10.3500_rev	GGGGACCACTTTGTACAAGAAAGCTGGGTTTAACCGTCAATACCTGCGATACCG
7_known	G10	Tb927.10.9780_for	GGGGACAAGTTTGTACAAAAAAGCAGGCTATGTCACTGAAGAAGAAGACGCTAAAG	Tb927.10.9780_rev	GGGGACCACTTTGTACAAGAAAGCTGGGTCTACTCCTTCTCTGTATTTTTTCGGGC
7_known	G11	Tb927.11.11900_for	GGGGACAAGTTTGTACAAAAAAGCAGGCTATGCAACACGACCGTGACC	Tb927.11.11900_rev	GGGGACCACTTTGTACAAGAAAGCTGGGTCTACGACAGCAGCGCACC
7_known	G12	Tb927.9.4820_for	GGGGACAAGTTTGTACAAAAAAGCAGGCTATGTCGAAATCACGCGCGG	Tb927.9.4820_rev	GGGGACCACTTTGTACAAGAAAGCTGGGTTCAACTAGCCGGTAGAAGGTAGAAA
7_known	G13	Tb927.11.6370_for	GGGGACAAGTTTGTACAAAAAAGCAGGCTATGCTGTTTGTGACGCCGAC	Tb927.11.6370_rev	GGGGACCACTTTGTACAAGAAAGCTGGGTTTAAACCTGAGGAGCCATCAGTGC
7_known	G14	Tb927.10.5280_for	GGGGACAAGTTTGTACAAAAAAGCAGGCTATGGACAGGGAAGCCATGCG	Tb927.10.5280_rev	GGGGACCACTTTGTACAAGAAAGCTGGGTTCAAACGTGTTTCCGCTTGTGC
7_known	G15	Tb927.10.7060_for	GGGGACAAGTTTGTACAAAAAAGCAGGCTATGATGTCCATCCCAAACCTCACG	Tb927.10.7060_rev	GGGGACCACTTTGTACAAGAAAGCTGGGTTCACTGCACAAACAACCGCT
7_known	G16	Tb927.8.1100_for	GGGGACAAGTTTGTACAAAAAAGCAGGCTATGAGTGAGGACAGCGCAGC	Tb927.8.1100_rev	GGGGACCACTTTGTACAAGAAAGCTGGGTTTAGCGGGTGACCAAGAAAGGA
7_known	G17	Tb927.1.1350_for	GGGGACAAGTTTGTACAAAAAAGCAGGCTATGCAAACATCGCGACCCTC	Tb927.1.1350_rev	GGGGACCACTTTGTACAAGAAAGCTGGGTTTACGCAAAGGCGTGTTCCA
7_known	G18	Tb927.5.1870_for	GGGGACAAGTTTGTACAAAAAAGCAGGCTATGCTTTCATGTTTGCGTCAGATCC	Tb927.5.1870_rev	GGGGACCACTTTGTACAAGAAAGCTGGGTTCACCGTTTGATGGTTGCCTC
7_known	G19	Tb927.8.6840_for	GGGGACAAGTTTGTACAAAAAAGCAGGCTATGAGGGGTATTGAGCGGCT	Tb927.8.6840_rev	GGGGACCACTTTGTACAAGAAAGCTGGGTTCAGCAACGCTCGAAGACCT
7_known	G20	Tb927.8.8330_for	GGGGACAAGTTTGTACAAAAAAGCAGGCTATGGGTTGTGGTGGATCAAAGC	Tb927.8.8330_rev	GGGGACCACTTTGTACAAGAAAGCTGGGTCTAGTCAGTGTCGCTATCATCCGA
7_known	G21	Tb927.11.1680_for	GGGGACAAGTTTGTACAAAAAAGCAGGCTATGATAGTCAAGACCACAGTTAGCAAAA	Tb927.11.1680_rev	GGGGACCACTTTGTACAAGAAAGCTGGGTTCAGTACATTTCCTCCCCGTGAA
7_known	G22	Tb927.3.2170_for	GGGGACAAGTTTGTACAAAAAAGCAGGCTATGAATCTCGAAACGACTGAGCAC	Tb927.3.2170_rev	GGGGACCACTTTGTACAAGAAAGCTGGGTCTACGCCCCCTTCAATGAAAATTTC
7_known	G23	Tb927.7.6290_for	GGGGACAAGTTTGTACAAAAAAGCAGGCTATGCGGTCACAGGTGCGT	Tb927.7.6290_rev	GGGGACCACTTTGTACAAGAAAGCTGGGTTCACCGCGTGCTGGGAAT
7_known	G24	Tb927.10.3340_for	GGGGACAAGTTTGTACAAAAAAGCAGGCTATGTCCTCACAGGACAGTGAAAGC	Tb927.10.3340_rev	GGGGACCACTTTGTACAAGAAAGCTGGGTTCACATGATAATGCAGCAGAAGTTAGG
7_known	H1	Tb927.7.1070_for	GGGGACAAGTTTGTACAAAAAAGCAGGCTATGGCATTGGCTCAGTCATGTC	Tb927.7.1070_rev	GGGGACCACTTTGTACAAGAAAGCTGGGTTTACGGTAACTTCAATGAAAACGTTATACT
7_known	H2	Tb927.11.8290_for	GGGGACAAGTTTGTACAAAAAAGCAGGCTATGGATGAAACACCGAATGCTGT	Tb927.11.8290_rev	GGGGACCACTTTGTACAAGAAAGCTGGGTTCAAACGAGACTTGTGGGTATTGC
7_known	H3	Tb927.4.4160_for	GGGGACAAGTTTGTACAAAAAAGCAGGCTATGCGATGTCTTAGTCTTCTCCCC	Tb927.4.4160_rev	GGGGACCACTTTGTACAAGAAAGCTGGGTCTAATAAAGCACCTTCGCTAACATTTCC
7_known	H4	Tb927.3.1770_for	GGGGACAAGTTTGTACAAAAAAGCAGGCTATGTACACAACCGCAAATAGGTCAT	Tb927.3.1770_rev	GGGGACCACTTTGTACAAGAAAGCTGGGTTCACACAGGGGTTGTGTTCGA
7_known	H5	Tb927.10.14000_for	GGGGACAAGTTTGTACAAAAAAGCAGGCTATGCTCAGCACGATGAAGTTACTG	Tb927.10.14000_rev	GGGGACCACTTTGTACAAGAAAGCTGGGTCTACAAATTACCCTTGATTTTCTCGCG
7_known	H6	Tb927.10.11440_for	GGGGACAAGTTTGTACAAAAAAGCAGGCTATGACATTGTCTGTCGACACCG	Tb927.10.11440_rev	GGGGACCACTTTGTACAAGAAAGCTGGGTTTATTTCCTGGGAAGACGGTGGT
7_known	H7	Tb927.7.6680_for	GGGGACAAGTTTGTACAAAAAAGCAGGCTATGCCTGTCGTGAAGGGCG	Tb927.7.6680_rev	GGGGACCACTTTGTACAAGAAAGCTGGGTTCACTCATCGTCTGGTGGCA
7_known	H8	Tb927.11.9310_for	GGGGACAAGTTTGTACAAAAAAGCAGGCTATGAGGGTGGGGTGGACAAC	Tb927.11.9310_rev	GGGGACCACTTTGTACAAGAAAGCTGGGTTCACCACGTCACGTTCCACG
7_known	H9	Tb927.7.4110_for	GGGGACAAGTTTGTACAAAAAAGCAGGCTATGTTTGATAAGGTAACAGCAATGAGCG	Tb927.7.4110_rev	GGGGACCACTTTGTACAAGAAAGCTGGGTTCACTTCACTGTAGGAGATACTGGATTAC
7_known	H10	Tb927.3.3220_for	GGGGACAAGTTTGTACAAAAAAGCAGGCTATGCAATATATATCGGAACCCGTTCAAC	Tb927.3.3220_rev	GGGGACCACTTTGTACAAGAAAGCTGGGTTCATATATTCCCATCATTAGCGGTGTCA
7_known	H11	Tb927.10.10850_for	GGGGACAAGTTTGTACAAAAAAGCAGGCTATGTCTGACTGGGAACGTGGA	Tb927.10.10850_rev	GGGGACCACTTTGTACAAGAAAGCTGGGTTTATAGATAATGCATTGTTGTCCGCAAGT
7_known	H12	Tb927.3.5420_for	GGGGACAAGTTTGTACAAAAAAGCAGGCTATGAACTTCGCCGTACCTCCA	Tb927.3.5420_rev	GGGGACCACTTTGTACAAGAAAGCTGGGTTCACTTAGCGATCAGTGAAGACCT
7_known	H13	Tb927.7.4940_for	GGGGACAAGTTTGTACAAAAAAGCAGGCTATGACGGCACTCTATCCGTGG	Tb927.7.4940_rev	GGGGACCACTTTGTACAAGAAAGCTGGGTCTATTTTCTCCCGTTTTCCGAGGC
7_known	H14	Tb927.7.3980_for	GGGGACAAGTTTGTACAAAAAAGCAGGCTATGACCATCACTGTGGATCTATTTGGT	Tb927.7.3980_rev	GGGGACCACTTTGTACAAGAAAGCTGGGTCTATGGCAGTAACTTCTCCAAGTTCTTG
7_known	H15	Tb927.2.4550_for	GGGGACAAGTTTGTACAAAAAAGCAGGCTATGGGTACCAAATCTAAAAAGAAGGCTAAG	Tb927.2.4550_rev	GGGGACCACTTTGTACAAGAAAGCTGGGTTCACTTACGCCTCTTCGCACG
7_known	H16	Tb927.8.8170_for	GGGGACAAGTTTGTACAAAAAAGCAGGCTATGCGACGGCTAAGCCTTCT	Tb927.8.8170_rev	GGGGACCACTTTGTACAAGAAAGCTGGGTCTAACAAAATACCTTCGCTAACATCTTCTG
7_known	H17	Tb927.4.2080_for	GGGGACAAGTTTGTACAAAAAAGCAGGCTATGCAGCAGGTTGATTTTGGTCA	Tb927.4.2080_rev	GGGGACCACTTTGTACAAGAAAGCTGGGTCTAATGGGTATTCATAGCCATTAGAACATC
7_known	H18	Tb927.7.5230_for	GGGGACAAGTTTGTACAAAAAAGCAGGCTATGATGCTCTATCAGAGAAGGGGC	Tb927.7.5230_rev	GGGGACCACTTTGTACAAGAAAGCTGGGTCTAATCCATCGATCGGGGAAAATGT
7_known	H19	Tb927.10.3570_for	GGGGACAAGTTTGTACAAAAAAGCAGGCTATGTTGCGCCGCAGTCGC	Tb927.10.3570_rev	GGGGACCACTTTGTACAAGAAAGCTGGGTCTAAACCACCTGAAACTCAACAAGGA
7_known	H20	Tb927.3.3070_for	GGGGACAAGTTTGTACAAAAAAGCAGGCTATGGTGTGCTACGACGGTTCG	Tb927.3.3070_rev	GGGGACCACTTTGTACAAGAAAGCTGGGTTCAAGGACCCACCGACGC
7_known	H21	Tb927.4.1250_for	GGGGACAAGTTTGTACAAAAAAGCAGGCTATGCAGCACAGCTCCTTTGAAG	Tb927.4.1250_rev	GGGGACCACTTTGTACAAGAAAGCTGGGTTCACCGTTGGGTCAATTTCCTTC
7_known	H22	Tb927.3.5520_for	GGGGACAAGTTTGTACAAAAAAGCAGGCTATGTCGGACAACAGAAAAGCAGT	Tb927.3.5520_rev	GGGGACCACTTTGTACAAGAAAGCTGGGTCTACTCCATATTTGGTTTCTCTTCCACAA
7_known	H23	Tb927.10.12500_for	GGGGACAAGTTTGTACAAAAAAGCAGGCTATGGGGGATACTGGACCAAAGG	Tb927.10.12500_rev	GGGGACCACTTTGTACAAGAAAGCTGGGTTTAAATAGGTTCCTTAGACGACTTAGCATT
7_known	H24	Tb927.11.2380_for	GGGGACAAGTTTGTACAAAAAAGCAGGCTATGTCTCTTAGCGCCTTACGGT	Tb927.11.2380_rev	GGGGACCACTTTGTACAAGAAAGCTGGGTCTATTTTGTTACTCGGGGCAACCG
7_known	I1	Tb927.10.13220_for	GGGGACAAGTTTGTACAAAAAAGCAGGCTATGCAGGCACATCGGTCGA	Tb927.10.13220_rev	GGGGACCACTTTGTACAAGAAAGCTGGGTCTATTCCAACGATAACCCTTCTTCAGATAC
7_known	I2	Tb927.6.3580_for	GGGGACAAGTTTGTACAAAAAAGCAGGCTATGCAAAATAGTTATGAAGAGATTCTTTTC	Tb927.6.3580_rev	GGGGACCACTTTGTACAAGAAAGCTGGGTCTACGTTTCTTCCTTTTCGTCCAAGT
7_known	I3	Tb927.11.6280_for	GGGGACAAGTTTGTACAAAAAAGCAGGCTATGGTGGCTAAAAAGTGGGTTTACTAC	Tb927.11.6280_rev	GGGGACCACTTTGTACAAGAAAGCTGGGTTTAAAGCTTGGCCGCGAATCC
7_known	I4	Tb927.10.13020_for	GGGGACAAGTTTGTACAAAAAAGCAGGCTATGTCACGTCCAAGAAGGTTTGTG	Tb927.10.13020_rev	GGGGACCACTTTGTACAAGAAAGCTGGGTTCATCCGAATAATTTGGCCAATCTTACG
7_known	I5	Tb927.11.12180_for	GGGGACAAGTTTGTACAAAAAAGCAGGCTATGGAAAATATTCGCGTGGTGGT	Tb927.11.12180_rev	GGGGACCACTTTGTACAAGAAAGCTGGGTTCATGAAGTTTGTCTAAAAGAATCACTAAT
7_known	I6	Tb927.11.9520_for	GGGGACAAGTTTGTACAAAAAAGCAGGCTATGACAATATGGCTGAATGTTGCGG	Tb927.11.9520_rev	GGGGACCACTTTGTACAAGAAAGCTGGGTTCAGTCCAACCACTGAAAAAATTGGC
7_known	I7	Tb927.8.5710_for	GGGGACAAGTTTGTACAAAAAAGCAGGCTATGCACATTATTGAAGTGAACCACGG	Tb927.8.5710_rev	GGGGACCACTTTGTACAAGAAAGCTGGGTTTAGACGACCAATGGTCGGACA
7_known	I8	Tb927.11.10650_for	GGGGACAAGTTTGTACAAAAAAGCAGGCTATGAACCGCGCTATCATTGCA	Tb927.11.10650_rev	GGGGACCACTTTGTACAAGAAAGCTGGGTTCACTCCGCGGGACCACT
7_known	I9	Tb927.11.16690_for	GGGGACAAGTTTGTACAAAAAAGCAGGCTATGTCCAACAAGTTGTTGCCATCG	Tb927.11.16690_rev	GGGGACCACTTTGTACAAGAAAGCTGGGTCTAGGCCCTCGGCACGGC
7_known	I10	Tb927.10.12510_for	GGGGACAAGTTTGTACAAAAAAGCAGGCTATGGGGGATACTGGACCAAAGG	Tb927.10.12510_rev	GGGGACCACTTTGTACAAGAAAGCTGGGTTTATAGCGTAGGTTCTTTAGGTGCACC
7_known	I11	Tb927.10.12310_for	GGGGACAAGTTTGTACAAAAAAGCAGGCTATGGTCCGCCTGACCGTG	Tb927.10.12310_rev	GGGGACCACTTTGTACAAGAAAGCTGGGTTCATTTCATGTTTTGTCGATGGGCC
7_known	I12	Tb927.1.4050_for	GGGGACAAGTTTGTACAAAAAAGCAGGCTATGGGTTGCTCGACCTCCAA	Tb927.1.4050_rev	GGGGACCACTTTGTACAAGAAAGCTGGGTTCAATCTCTCACAAAGGAGGAAAAGC
7_known	I13	Tb927.5.490_for	GGGGACAAGTTTGTACAAAAAAGCAGGCTATGTTATTTTTTTGTTGGTTTGCTTGTTCC	Tb927.5.490_rev	GGGGACCACTTTGTACAAGAAAGCTGGGTTTATTCCTTTTCATTTTGCGGTGCTG
7_known	I14	Tb927.9.5100_for	GGGGACAAGTTTGTACAAAAAAGCAGGCTATGACACACAACGGTGGTCG	Tb927.9.5100_rev	GGGGACCACTTTGTACAAGAAAGCTGGGTCTAAGACGAAGCCCCTGTACTCC
7_known	I15	Tb927.8.3560_for	GGGGACAAGTTTGTACAAAAAAGCAGGCTATGCTCTCCCGTCTAAGCGC	Tb927.8.3560_rev	GGGGACCACTTTGTACAAGAAAGCTGGGTTTATTCTTCCACTTCCCCTTCATACTCC
7_known	I16	Tb927.3.5650_for	GGGGACAAGTTTGTACAAAAAAGCAGGCTATGATAGAATATGCGTGTGGTTGGC	Tb927.3.5650_rev	GGGGACCACTTTGTACAAGAAAGCTGGGTCTACAAATGAAATTCGCTTCTCGAGATATT
7_known	I17	Tb927.11.6890_for	GGGGACAAGTTTGTACAAAAAAGCAGGCTATGTTGCGTCGGCTCCTCC	Tb927.11.6890_rev	GGGGACCACTTTGTACAAGAAAGCTGGGTCTATTCCAACGACTCACCAGAGGA
7_known	I18	Tb927.9.5040_for	GGGGACAAGTTTGTACAAAAAAGCAGGCTATGTTCATGAACAAGCCCTTTGGC	Tb927.9.5040_rev	GGGGACCACTTTGTACAAGAAAGCTGGGTTCAACGAGTACTGCTGTTGTTGC
7_known	I19	Tb927.10.2270_for	GGGGACAAGTTTGTACAAAAAAGCAGGCTATGTACGGCCATGTTGTGGTG	Tb927.10.2270_rev	GGGGACCACTTTGTACAAGAAAGCTGGGTCTACTCATTATCAGCGGCCTCGC
7_known	I20	Tb927.8.8180_for	GGGGACAAGTTTGTACAAAAAAGCAGGCTATGTACCGTCTTTATCGTCGGACT	Tb927.8.8180_rev	GGGGACCACTTTGTACAAGAAAGCTGGGTCTAAACTGCTGTGGCCAGTTTATTCT
7_known	I22	Tb927.9.5240_for	GGGGACAAGTTTGTACAAAAAAGCAGGCTATGAGCAAACGCCGCCGT	Tb927.9.5240_rev	GGGGACCACTTTGTACAAGAAAGCTGGGTCTAAGAATTGTTTTTGACGAAGCGCC
7_known	I23	Tb927.9.8540_for	GGGGACAAGTTTGTACAAAAAAGCAGGCTATGTATACCAGCATGTCCATCCAAGG	Tb927.9.8540_rev	GGGGACCACTTTGTACAAGAAAGCTGGGTTTAAGGACAAGAGTTGATCGCAGTG
7_known	I24	Tb927.3.5100_for	GGGGACAAGTTTGTACAAAAAAGCAGGCTATGATTCTTGGCCCTGGTGGG	Tb927.3.5100_rev	GGGGACCACTTTGTACAAGAAAGCTGGGTCTAGGTAGTCAGCGGAATTCGGT
7_known	J1	Tb927.8.4260_for	GGGGACAAGTTTGTACAAAAAAGCAGGCTATGAGTGAAATCAGACGACCACAGG	Tb927.8.4260_rev	GGGGACCACTTTGTACAAGAAAGCTGGGTCTAAGCGGTAAAGGAAGGAGTAACAG
7_known	J2	Tb927.10.13270_for	GGGGACAAGTTTGTACAAAAAAGCAGGCTATGGAGACAAACTTTCAGCTGGC	Tb927.10.13270_rev	GGGGACCACTTTGTACAAGAAAGCTGGGTTCATTCAGTAACTTCCGCGGC
7_known	J3	Tb927.10.3360_for	GGGGACAAGTTTGTACAAAAAAGCAGGCTATGCCGCGGTTGTTGTTACC	Tb927.10.3360_rev	GGGGACCACTTTGTACAAGAAAGCTGGGTCTATTCAACAAAATGCCACGATGTGG
7_known	J4	Tb927.10.7630_for	GGGGACAAGTTTGTACAAAAAAGCAGGCTATGAGTTTTGCGGGAGGTTATTCC	Tb927.10.7630_rev	GGGGACCACTTTGTACAAGAAAGCTGGGTTCAACAAATGAGTCCATATGAAGATATCAC
7_known	J5	Tb927.5.2300_for	GGGGACAAGTTTGTACAAAAAAGCAGGCTATGCAACGTGCCCAGATTCTC	Tb927.5.2300_rev	GGGGACCACTTTGTACAAGAAAGCTGGGTTTAACTTCTAATTGCACGACGGGG
7_known	J6	Tb927.1.4420_for	GGGGACAAGTTTGTACAAAAAAGCAGGCTATGACGACAACTCAAGGTGAAGTAATC	Tb927.1.4420_rev	GGGGACCACTTTGTACAAGAAAGCTGGGTTCACAATGTTACGGTCTCATCCGC
7_known	J7	Tb927.11.7218_for	GGGGACAAGTTTGTACAAAAAAGCAGGCTATGACATCTAGCGTAACCGGACC	Tb927.11.7218_rev	GGGGACCACTTTGTACAAGAAAGCTGGGTCTATTTTTTCTTCCCCTTGTTCTTTCCTTT
7_known	J8	Tb927.3.1290_for	GGGGACAAGTTTGTACAAAAAAGCAGGCTATGTCATCACGGACGCTGTATG	Tb927.3.1290_rev	GGGGACCACTTTGTACAAGAAAGCTGGGTCTACGAAATATATATATATTCATTTGTATT
7_known	J9	Tb927.11.7860_for	GGGGACAAGTTTGTACAAAAAAGCAGGCTATGCCTCCTCACCGCAAGC	Tb927.11.7860_rev	GGGGACCACTTTGTACAAGAAAGCTGGGTTTACACCAGGGAGTGCTCTATTTGA
7_known	J10	Tb927.5.3600_for	GGGGACAAGTTTGTACAAAAAAGCAGGCTATGCCTAAACGTGAAAGGGAAGAC	Tb927.5.3600_rev	GGGGACCACTTTGTACAAGAAAGCTGGGTCTAAAGCTGTGCCCCAACCG
7_known	J11	Tb927.11.860_for	GGGGACAAGTTTGTACAAAAAAGCAGGCTATGCACGCTGGCACTCCA	Tb927.11.860_rev	GGGGACCACTTTGTACAAGAAAGCTGGGTCTAGGCCCTTGTTGCACCTTC
7_known	J12	Tb927.11.460_for	GGGGACAAGTTTGTACAAAAAAGCAGGCTATGGAAGGAAAGAACTGCTTCCG	Tb927.11.460_rev	GGGGACCACTTTGTACAAGAAAGCTGGGTTCAAGTCTTCTCACGGCGCT
7_known	J13	Tb927.3.2140_for	GGGGACAAGTTTGTACAAAAAAGCAGGCTATGAAGTTTGAGAACCCCGTTGC	Tb927.3.2140_rev	GGGGACCACTTTGTACAAGAAAGCTGGGTCTAGTGAGATTCACTGAAACTGCCG
7_known	J14	Tb927.3.1230_for	GGGGACAAGTTTGTACAAAAAAGCAGGCTATGATTTTCGGGGAAGATATTGGACC	Tb927.3.1230_rev	GGGGACCACTTTGTACAAGAAAGCTGGGTTCACTGAGTGGTAATATTCAACGCATC
7_known	J15	Tb927.8.6210_for	GGGGACAAGTTTGTACAAAAAAGCAGGCTATGGCCACGAACGAGGACA	Tb927.8.6210_rev	GGGGACCACTTTGTACAAGAAAGCTGGGTTTAGGCCCGCCGCGCCTG
7_known	J16	Tb927.10.7440_for	GGGGACAAGTTTGTACAAAAAAGCAGGCTATGCAGGATGACATACCCGTGG	Tb927.10.7440_rev	GGGGACCACTTTGTACAAGAAAGCTGGGTCTACAGGTACAGCGACGACGC
7_known	J17	Tb927.10.11020_for	GGGGACAAGTTTGTACAAAAAAGCAGGCTATGAGTGACGACCGCGACC	Tb927.10.11020_rev	GGGGACCACTTTGTACAAGAAAGCTGGGTTCACACCTCCACCAATGACGA
7_known	J18	Tb927.11.7211_for	GGGGACAAGTTTGTACAAAAAAGCAGGCTATGGAGCAGCTACTTCGAAGGG	Tb927.11.7211_rev	GGGGACCACTTTGTACAAGAAAGCTGGGTCTAGACGGTGGGGCACGC
7_known	J19	Tb927.10.5450_for	GGGGACAAGTTTGTACAAAAAAGCAGGCTATGTCACAGAAAGAGGAGAATAGCGT	Tb927.10.5450_rev	GGGGACCACTTTGTACAAGAAAGCTGGGTTCACAACTCGAGCCTTAGCTTGT
7_known	J20	Tb927.4.2950_for	GGGGACAAGTTTGTACAAAAAAGCAGGCTATGTCGTTACATGGTCATCCGTACC	Tb927.4.2950_rev	GGGGACCACTTTGTACAAGAAAGCTGGGTTCAACTCGGCACCCGCAG
7_known	J21	Tb927.6.4740_for	GGGGACAAGTTTGTACAAAAAAGCAGGCTATGGATCCAATCAACCCGAATGATC	Tb927.6.4740_rev	GGGGACCACTTTGTACAAGAAAGCTGGGTTCACTGCAATTGCGCCATTACC
7_known	J22	Tb927.11.1260_for	GGGGACAAGTTTGTACAAAAAAGCAGGCTATGGAGGAGAACAACGCAACTACA	Tb927.11.1260_rev	GGGGACCACTTTGTACAAGAAAGCTGGGTCTAAATGATATCAATGAACTGTTCCCGTTT
7_known	J23	Tb927.3.3090_for	GGGGACAAGTTTGTACAAAAAAGCAGGCTATGTCGTCTGTATTTATTGACGGCG	Tb927.3.3090_rev	GGGGACCACTTTGTACAAGAAAGCTGGGTTCAGGAAACCCTTGGTCGCT
7_known	J24	Tb927.11.9330_for	GGGGACAAGTTTGTACAAAAAAGCAGGCTATGACAGAGGACGAGGAACCATATG	Tb927.11.9330_rev	GGGGACCACTTTGTACAAGAAAGCTGGGTTCACTGCGGAAACCCGTCC
7_known	K1	Tb927.6.4600_for	GGGGACAAGTTTGTACAAAAAAGCAGGCTATGACTAAGAAGAATGTTATTCGCTTTACT	Tb927.6.4600_rev	GGGGACCACTTTGTACAAGAAAGCTGGGTTTACTCGACAGTGACAGCGCA
7_known	K2	Tb927.6.700_for	GGGGACAAGTTTGTACAAAAAAGCAGGCTATGACAAAAGCGGTGACTGAATGG	Tb927.6.700_rev	GGGGACCACTTTGTACAAGAAAGCTGGGTTCAGTTCAAAAGCACTGCCATCA
7_known	K3	Tb927.7.7160_for	GGGGACAAGTTTGTACAAAAAAGCAGGCTATGGAAAGAGAAGCAACAAAACGGG	Tb927.7.7160_rev	GGGGACCACTTTGTACAAGAAAGCTGGGTCTAATGCGGCGAGGCGCA
7_known	K4	Tb927.11.5260_for	GGGGACAAGTTTGTACAAAAAAGCAGGCTATGAATTGCAACTGTGGCACACC	Tb927.11.5260_rev	GGGGACCACTTTGTACAAGAAAGCTGGGTTCACTGAGTCTCACCGGTTTCA
7_known	K5	Tb927.8.3660_for	GGGGACAAGTTTGTACAAAAAAGCAGGCTATGCAAGGACACGTACCACAGC	Tb927.8.3660_rev	GGGGACCACTTTGTACAAGAAAGCTGGGTCTACTTTTTCGACTCTGGCGTAACA
7_known	K6	Tb927.1.1100_for	GGGGACAAGTTTGTACAAAAAAGCAGGCTATGCCGAAGTCTTCCTCAGCC	Tb927.1.1100_rev	GGGGACCACTTTGTACAAGAAAGCTGGGTTTAGTCCAACACCCCATTACCATGA
7_known	K7	Tb927.7.1910_for	GGGGACAAGTTTGTACAAAAAAGCAGGCTATGTATCGCAGTGCGTTTCGC	Tb927.7.1910_rev	GGGGACCACTTTGTACAAGAAAGCTGGGTCTATTTGAATGAGTCCAACGACATACAGT
7_known	K8	Tb11.02.5380_for	GGGGACAAGTTTGTACAAAAAAGCAGGCTATGTTTACGAAAAAACGACTGGCTG	Tb11.02.5380_rev	GGGGACCACTTTGTACAAGAAAGCTGGGTTTACTGTTGCCCCTCCTCCAG
7_known	K9	Tb927.9.6430_for	GGGGACAAGTTTGTACAAAAAAGCAGGCTATGAAGTCATCTGATCATGACGAAGAGG	Tb927.9.6430_rev	GGGGACCACTTTGTACAAGAAAGCTGGGTTCAGGAACTTACTGAGGAAACGGG
7_known	K10	Tb927.3.1210_for	GGGGACAAGTTTGTACAAAAAAGCAGGCTATGTCGTACCCACACAACTTTGAC	Tb927.3.1210_rev	GGGGACCACTTTGTACAAGAAAGCTGGGTTCATAGCGCCAACTCGCCC
7_known	K11	Tb927.10.5180_for	GGGGACAAGTTTGTACAAAAAAGCAGGCTATGAGTGTAAGGCACCTGTTTGG	Tb927.10.5180_rev	GGGGACCACTTTGTACAAGAAAGCTGGGTTCAACGATTGCCGATCACCTG
7_known	K12	Tb927.11.15430_for	GGGGACAAGTTTGTACAAAAAAGCAGGCTATGAACTTTGGTTATGATGAAGAGGGG	Tb927.11.15430_rev	GGGGACCACTTTGTACAAGAAAGCTGGGTTCACATCTGGAACTCTGCGAGA
7_known	K13	Tb927.6.4480_for	GGGGACAAGTTTGTACAAAAAAGCAGGCTATGAAACAACTTGCTCCCCAGATG	Tb927.6.4480_rev	GGGGACCACTTTGTACAAGAAAGCTGGGTTTAGAGTAAACTCTTCATCTTTGTTAAACC
7_known	K14	Tb927.7.3950_for	GGGGACAAGTTTGTACAAAAAAGCAGGCTATGGTAAGTAAGTACCACCGCTTGC	Tb927.7.3950_rev	GGGGACCACTTTGTACAAGAAAGCTGGGTTCAACGAGGGCTCTTGAATGGA
7_known	K15	Tb927.11.9220_for	GGGGACAAGTTTGTACAAAAAAGCAGGCTATGGCCGTCGGACTCTCATC	Tb927.11.9220_rev	GGGGACCACTTTGTACAAGAAAGCTGGGTCTAGGGCCATGTAAACGGTTCTG
7_known	K16	Tb927.11.6810_for	GGGGACAAGTTTGTACAAAAAAGCAGGCTATGAGGAACATGATGGAGCCAGT	Tb927.11.6810_rev	GGGGACCACTTTGTACAAGAAAGCTGGGTCTACTCATCCAAATGGAATGAATCTTCTCT
7_known	K17	Tb927.5.3920_for	GGGGACAAGTTTGTACAAAAAAGCAGGCTATGATGTCACGGACCGTTGAGC	Tb927.5.3920_rev	GGGGACCACTTTGTACAAGAAAGCTGGGTTCAATCAGCCACACGACCCG
7_known	K18	Tb927.1.2570_for	GGGGACAAGTTTGTACAAAAAAGCAGGCTATGGAGGCCAAAGCTGAGGG	Tb927.1.2570_rev	GGGGACCACTTTGTACAAGAAAGCTGGGTTTATGACACTATACGTCGATTCAGTATATT
7_known	K19	Tb927.11.330_for	GGGGACAAGTTTGTACAAAAAAGCAGGCTATGTTCCCTGGGTCAAAAAGGTCG	Tb927.11.330_rev	GGGGACCACTTTGTACAAGAAAGCTGGGTTCAACTGGACTGGGATTCTCCTTC
7_known	K20	Tb927.11.750_for	GGGGACAAGTTTGTACAAAAAAGCAGGCTATGACAAGCAAACATAGTGCTGTATTAAC	Tb927.11.750_rev	GGGGACCACTTTGTACAAGAAAGCTGGGTCTACCTCGTGAACAGAAACTGAAGATC
7_known	K21	Tb927.10.11680_for	GGGGACAAGTTTGTACAAAAAAGCAGGCTATGAAGGGGCAGTCTTCACAGT	Tb927.10.11680_rev	GGGGACCACTTTGTACAAGAAAGCTGGGTTTAGAAAGTAGCATCGGGTTTCCAAG
7_known	K22	Tb927.5.2400_for	GGGGACAAGTTTGTACAAAAAAGCAGGCTATGCATCACGGTCGCAAAGT	Tb927.5.2400_rev	GGGGACCACTTTGTACAAGAAAGCTGGGTTCACTCGCTTCCCCGTGG
7_known	K23	Tb927.11.6400_for	GGGGACAAGTTTGTACAAAAAAGCAGGCTATGGGCGGAGAAGATAACGTCG	Tb927.11.6400_rev	GGGGACCACTTTGTACAAGAAAGCTGGGTTCAGGTGGAGACGTTGAGTCC
7_known	K24	Tb927.10.1380_for	GGGGACAAGTTTGTACAAAAAAGCAGGCTATGACAAGCCGAGCCCCT	Tb927.10.1380_rev	GGGGACCACTTTGTACAAGAAAGCTGGGTTTATGACATAGCCATCCATAGTTGTAGCC
7_known	L1	Tb927.6.920_for	GGGGACAAGTTTGTACAAAAAAGCAGGCTATGCATCGCTTTGTGTTGGTGC	Tb927.6.920_rev	GGGGACCACTTTGTACAAGAAAGCTGGGTTCATGAGGTATGGGGGAAGTATGAG
7_known	L2	Tb927.3.3180_for	GGGGACAAGTTTGTACAAAAAAGCAGGCTATGCGTGAGCGGCCGCGT	Tb927.3.3180_rev	GGGGACCACTTTGTACAAGAAAGCTGGGTTTAAGCATTCCCAGAAGAGGCACC
7_known	L3	Tb927.8.700_for	GGGGACAAGTTTGTACAAAAAAGCAGGCTATGTGGGATGGGCCGCTAC	Tb927.8.700_rev	GGGGACCACTTTGTACAAGAAAGCTGGGTTCAGTTCACCCTTCGGGCG
7_known	L4	Tb927.9.5900_for	GGGGACAAGTTTGTACAAAAAAGCAGGCTATGATGCGCCGTGCTTCTT	Tb927.9.5900_rev	GGGGACCACTTTGTACAAGAAAGCTGGGTTTAAGAGATGTGCTTGGCCTGC
7_known	L5	Tb927.10.6410_for	GGGGACAAGTTTGTACAAAAAAGCAGGCTATGCCTGAGGTTTCGCTTGAATG	Tb927.10.6410_rev	GGGGACCACTTTGTACAAGAAAGCTGGGTCTACCGATGCGAGAACTGTGTC
7_known	L6	Tb927.10.2570_for	GGGGACAAGTTTGTACAAAAAAGCAGGCTATGCTCACACTTGGGATGCATAC	Tb927.10.2570_rev	GGGGACCACTTTGTACAAGAAAGCTGGGTTTACACACCCGTGCAGCTCA
7_known	L7	Tb927.9.5460_for	GGGGACAAGTTTGTACAAAAAAGCAGGCTATGGACTCACCTGAAGCATGCC	Tb927.9.5460_rev	GGGGACCACTTTGTACAAGAAAGCTGGGTCTAAACCCGCCTCACAGCG
7_known	L8	Tb927.6.2460_for	GGGGACAAGTTTGTACAAAAAAGCAGGCTATGATTGAGGTAAGCAGTTTGGAGC	Tb927.6.2460_rev	GGGGACCACTTTGTACAAGAAAGCTGGGTTCATTCACGGTGCGCCTTC
7_known	L9	Tb927.11.17000_for	GGGGACAAGTTTGTACAAAAAAGCAGGCTATGAGTGCGTCTGCGATAAGGC	Tb927.11.17000_rev	GGGGACCACTTTGTACAAGAAAGCTGGGTCTATGCGCCGTCAGTCCG
7_known	L10	Tb927.4.2140_for	GGGGACAAGTTTGTACAAAAAAGCAGGCTATGGCAGATACCTTGCAACTTCTG	Tb927.4.2140_rev	GGGGACCACTTTGTACAAGAAAGCTGGGTTCACTGCGTCAGCGAAAAGTG

7_known	L11	Tb927.11.15990_for	GGGGACAAGTTTGTACAAAAAAGCAGGCTATGGCGGAGGTGCGGCGT	Tb927.11.15990_rev	GGGGACCACTTTGTACAAGAAAGCTGGGTTCATTTCCTCACCCTGTCATGATACT
7_known	L12	Tb927.2.3180_for	GGGGACAAGTTTGTACAAAAAAGCAGGCTATGTTCCAACGCCGGTTAGTG	Tb927.2.3180_rev	GGGGACCACTTTGTACAAGAAAGCTGGGTCTAAACATCCGCACCGGCA
7_known	L13	Tb927.10.5820_for	GGGGACAAGTTTGTACAAAAAAGCAGGCTATGTGGCGCCCACCAATCC	Tb927.10.5820_rev	GGGGACCACTTTGTACAAGAAAGCTGGGTCTATTTGTCGCATTTGGCAATGCC
7_known	L14	Tb927.9.2350_for	GGGGACAAGTTTGTACAAAAAAGCAGGCTATGGAGGAGTATCTGACTGTCAACAT	Tb927.9.2350_rev	GGGGACCACTTTGTACAAGAAAGCTGGGTTCACCACCTACGGTGGGC
7_known	L15	Tb927.10.2990_for	GGGGACAAGTTTGTACAAAAAAGCAGGCTATGTATGCAATGCCCGGTAATAGTG	Tb927.10.2990_rev	GGGGACCACTTTGTACAAGAAAGCTGGGTCTATACACTTACTTCGTATCGACCGC
7_known	L16	Tb927.11.5740_for	GGGGACAAGTTTGTACAAAAAAGCAGGCTATGCGTTTTTTTAGCGGGTGGT	Tb927.11.5740_rev	GGGGACCACTTTGTACAAGAAAGCTGGGTTCACTCTTTCTTGGAAGAGTCTTCAGG
7_known	L17	Tb927.11.9980_for	GGGGACAAGTTTGTACAAAAAAGCAGGCTATGATGCGAAGGCTCAGTCCT	Tb927.11.9980_rev	GGGGACCACTTTGTACAAGAAAGCTGGGTTTATGAAAATACGCATTCGCAAATGTACC
7_known	L18	Tb927.2.6130_for	GGGGACAAGTTTGTACAAAAAAGCAGGCTATGCACGCGAGAATAAATATACGTAACG	Tb927.2.6130_rev	GGGGACCACTTTGTACAAGAAAGCTGGGTTTATTTCACAAAGTGGGGCAATTGC
7_known	L19	Tb927.11.1450_for	GGGGACAAGTTTGTACAAAAAAGCAGGCTATGATGCGCCGTGCCTTT	Tb927.11.1450_rev	GGGGACCACTTTGTACAAGAAAGCTGGGTTTACGCCAGCGCCTGTCT
7_known	L20	Tb927.4.2500_for	GGGGACAAGTTTGTACAAAAAAGCAGGCTATGCCGGATGCCTATGTTTTTGC	Tb927.4.2500_rev	GGGGACCACTTTGTACAAGAAAGCTGGGTTCAGTTGGCTGACGGCAAAC
7_known	L21	Tb927.10.3800_for	GGGGACAAGTTTGTACAAAAAAGCAGGCTATGACCACAGAGCTTAACAGTTTTCG	Tb927.10.3800_rev	GGGGACCACTTTGTACAAGAAAGCTGGGTTCACTGATCAGAGCCTACACAAGA
7_known	L22	Tb927.11.15690_for	GGGGACAAGTTTGTACAAAAAAGCAGGCTATGAGCGCTGCGGCACCT	Tb927.11.15690_rev	GGGGACCACTTTGTACAAGAAAGCTGGGTTTACAATTTGATCATTTCCACGACTTGC
7_known	L23	Tb927.6.3310_for	GGGGACAAGTTTGTACAAAAAAGCAGGCTATGGTTGTGAGTCTTTCCGGTTATTC	Tb927.6.3310_rev	GGGGACCACTTTGTACAAGAAAGCTGGGTTTAAACACTCGACAACACTCTAACAGAA
7_known	L24	Tb927.8.1510_for	GGGGACAAGTTTGTACAAAAAAGCAGGCTATGTATGGGATGAACAGAGGAGGAG	Tb927.8.1510_rev	GGGGACCACTTTGTACAAGAAAGCTGGGTCTAATAAGAACCACCCGTCGGTTG
7_known	M1	Tb927.5.3400_for	GGGGACAAGTTTGTACAAAAAAGCAGGCTATGCTACCCGAGAACCTTCCG	Tb927.5.3400_rev	GGGGACCACTTTGTACAAGAAAGCTGGGTCTAGTCCTTCTTCTTTTCCTGGGCC
7_known	M2	Tb927.10.14460_for	GGGGACAAGTTTGTACAAAAAAGCAGGCTATGGAAGCGACTACAGATCCACC	Tb927.10.14460_rev	GGGGACCACTTTGTACAAGAAAGCTGGGTTCATATCCCACTGGGTTTGCTTCC
7_known	M3	Tb927.9.3120_for	GGGGACAAGTTTGTACAAAAAAGCAGGCTATGTTGCCTCACCCCATTTTAGC	Tb927.9.3120_rev	GGGGACCACTTTGTACAAGAAAGCTGGGTCTACGAATGGGGTTGTGTGGTG
7_known	M4	Tb927.8.4840_for	GGGGACAAGTTTGTACAAAAAAGCAGGCTATGGTAAATAGTTCCGCTTTGGTTAGTG	Tb927.8.4840_rev	GGGGACCACTTTGTACAAGAAAGCTGGGTTCAATAGGGTCCCAAAGTCGATGC
7_known	M5	Tb927.7.7300_for	GGGGACAAGTTTGTACAAAAAAGCAGGCTATGAACGCATGCAACAATAATACTGG	Tb927.7.7300_rev	GGGGACCACTTTGTACAAGAAAGCTGGGTTTAAGTTGTGATGGTGCCATGCG
7_known	M6	Tb927.3.2710_for	GGGGACAAGTTTGTACAAAAAAGCAGGCTATGAATCCACAAAATCACGTTTGTTGC	Tb927.3.2710_rev	GGGGACCACTTTGTACAAGAAAGCTGGGTTCATTCGCTCGGCTGCAGT
7_known	M7	Tb927.6.2980_for	GGGGACAAGTTTGTACAAAAAAGCAGGCTATGGCCAGCGAGTGCTCC	Tb927.6.2980_rev	GGGGACCACTTTGTACAAGAAAGCTGGGTTCACACCTCAGAAAAAAAGGAGAGC
7_known	M8	Tb927.6.2960_for	GGGGACAAGTTTGTACAAAAAAGCAGGCTATGTCTAAGCTTTACAAGGCTCAGAC	Tb927.6.2960_rev	GGGGACCACTTTGTACAAGAAAGCTGGGTCTACGACAATGGTATCGCGCTG
7_known	M9	Tb927.9.2140_for	GGGGACAAGTTTGTACAAAAAAGCAGGCTATGGCACGGGATAGCGGT	Tb927.9.2140_rev	GGGGACCACTTTGTACAAGAAAGCTGGGTTTACTCCGACATTATCTTTTCGTACAGAAA
7_known	M10	Tb927.2.1800_for	GGGGACAAGTTTGTACAAAAAAGCAGGCTATGACTGATTCACCTGATCTCTCTCG	Tb927.2.1800_rev	GGGGACCACTTTGTACAAGAAAGCTGGGTTCACCAATCGTCCACTACACCG
7_known	M11	Tb927.7.5600_for	GGGGACAAGTTTGTACAAAAAAGCAGGCTATGGGGTGTAACCTTTCCAGCT	Tb927.7.5600_rev	GGGGACCACTTTGTACAAGAAAGCTGGGTCTAATAAATACCCTTCTGACTATCTGCACA
7_known	M12	Tb927.8.7260_for	GGGGACAAGTTTGTACAAAAAAGCAGGCTATGACCGCTCGACGACGC	Tb927.8.7260_rev	GGGGACCACTTTGTACAAGAAAGCTGGGTTCAGTGCGGTTTTCTAAACACAAAC
7_known	M13	Tb927.5.4300_for	GGGGACAAGTTTGTACAAAAAAGCAGGCTATGTCAGCCTCCTCAGAGCATAG	Tb927.5.4300_rev	GGGGACCACTTTGTACAAGAAAGCTGGGTCTATGTGCTAGACTGCTCAGCAGC
7_known	M14	Tb927.9.9450_for	GGGGACAAGTTTGTACAAAAAAGCAGGCTATGTATTCTAGCGAAAAGGAGAAGGAAG	Tb927.9.9450_rev	GGGGACCACTTTGTACAAGAAAGCTGGGTTCACGAACGACCCGGATGC
7_known	M15	Tb927.5.2820_for	GGGGACAAGTTTGTACAAAAAAGCAGGCTATGAATGCCTTGGATGAGCTTAACG	Tb927.5.2820_rev	GGGGACCACTTTGTACAAGAAAGCTGGGTTCACTGTGCCGTAGCTCCA
7_known	M16	Tb927.8.1860_for	GGGGACAAGTTTGTACAAAAAAGCAGGCTATGATTCGACGTTGTGCTGTTCC	Tb927.8.1860_rev	GGGGACCACTTTGTACAAGAAAGCTGGGTCTACTCTTTCCGCTCCTCGGG
7_known	M17	Tb927.9.4560_for	GGGGACAAGTTTGTACAAAAAAGCAGGCTATGTATTTCCCGCGTCACCAAC	Tb927.9.4560_rev	GGGGACCACTTTGTACAAGAAAGCTGGGTTTAGAACAACCGAATACCCGCTAAATC
7_known	M18	Tb927.6.820_for	GGGGACAAGTTTGTACAAAAAAGCAGGCTATGACAACCATGGAGGAAGAAAACAT	Tb927.6.820_rev	GGGGACCACTTTGTACAAGAAAGCTGGGTTCATCCCTTCCTGCCGCG
7_known	M19	Tb927.11.14340_for	GGGGACAAGTTTGTACAAAAAAGCAGGCTATGGAAGATATTCTCGACTTCAGCAAG	Tb927.11.14340_rev	GGGGACCACTTTGTACAAGAAAGCTGGGTTCAGGGATAAAAAGAAGGTGTTGGTG
7_known	M20	Tb927.11.5430_for	GGGGACAAGTTTGTACAAAAAAGCAGGCTATGCCAGTTTTTCGTAATTCCACTGG	Tb927.11.5430_rev	GGGGACCACTTTGTACAAGAAAGCTGGGTCTAATCGTCACCAGAGGGATAATCATCA
7_known	M21	Tb927.2.4200_for	GGGGACAAGTTTGTACAAAAAAGCAGGCTATGTCACCCTCGAAGGGTGAA	Tb927.2.4200_rev	GGGGACCACTTTGTACAAGAAAGCTGGGTTTACTTTCTTTTTCCATGCGCTTCAG
7_known	M22	Tb927.9.15460_for	GGGGACAAGTTTGTACAAAAAAGCAGGCTATGACGGGCTCAGGTGGC	Tb927.9.15460_rev	GGGGACCACTTTGTACAAGAAAGCTGGGTTTACATCTTTCCTCCTTTCCCCCC
7_known	M23	Tb927.7.7260_for	GGGGACAAGTTTGTACAAAAAAGCAGGCTATGGGGAAGTCGGAGGGTATTC	Tb927.7.7260_rev	GGGGACCACTTTGTACAAGAAAGCTGGGTCTATTGAGGGGGGTCCAGCTG
7_known	M24	Tb927.2.4280_for	GGGGACAAGTTTGTACAAAAAAGCAGGCTATGGGTGCTGCCTTATGCAC	Tb927.2.4280_rev	GGGGACCACTTTGTACAAGAAAGCTGGGTTTAATCAAGTGCTAGCAACAGTTCATCC
7_known	N1	Tb927.11.5730_for	GGGGACAAGTTTGTACAAAAAAGCAGGCTATGCTTGACCGCCGGCGG	Tb927.11.5730_rev	GGGGACCACTTTGTACAAGAAAGCTGGGTTCAGATGTTGTTTCGCCGTCTTG
7_known	N2	Tb927.11.6900_for	GGGGACAAGTTTGTACAAAAAAGCAGGCTATGTCTTCAAAGACGGTAGTTTGGAG	Tb927.11.6900_rev	GGGGACCACTTTGTACAAGAAAGCTGGGTTTACGGTGTTGCTTCCTTCGC
7_known	N3	Tb927.7.2080_for	GGGGACAAGTTTGTACAAAAAAGCAGGCTATGGAATCGACGAGTTCACTTTCG	Tb927.7.2080_rev	GGGGACCACTTTGTACAAGAAAGCTGGGTTCAAATCTTGACGAACAAGAAGGTTCT
7_known	N4	Tb927.9.3400_for	GGGGACAAGTTTGTACAAAAAAGCAGGCTATGGCCACATCTCTTGTGCAG	Tb927.9.3400_rev	GGGGACCACTTTGTACAAGAAAGCTGGGTTTAGTTTCCAGAAGCTGATCCATAGAAGA
7_known	N5	Tb927.11.12540_for	GGGGACAAGTTTGTACAAAAAAGCAGGCTATGTCGGCGGTTGCAACC	Tb927.11.12540_rev	GGGGACCACTTTGTACAAGAAAGCTGGGTTCAGGCGATCCTCCGGTG
7_known	N6	Tb927.8.2640_for	GGGGACAAGTTTGTACAAAAAAGCAGGCTATGACTAGTGAAGAGCAAAGACGGC	Tb927.8.2640_rev	GGGGACCACTTTGTACAAGAAAGCTGGGTTTAAAAATCCTTGTACCTGTAATAAATTAG
7_known	N7	Tb927.11.10370_for	GGGGACAAGTTTGTACAAAAAAGCAGGCTATGCCCGCATACACTGGCC	Tb927.11.10370_rev	GGGGACCACTTTGTACAAGAAAGCTGGGTTCATAACCTCAAATCGAATGCAGCG
7_known	N8	Tb927.10.3550_for	GGGGACAAGTTTGTACAAAAAAGCAGGCTATGTATGCGGCTGGTCGCT	Tb927.10.3550_rev	GGGGACCACTTTGTACAAGAAAGCTGGGTTCACTTCCGCCGCTCGAT
7_known	N9	Tb927.11.6720_for	GGGGACAAGTTTGTACAAAAAAGCAGGCTATGTTGAACGCAAATGCCAGGT	Tb927.11.6720_rev	GGGGACCACTTTGTACAAGAAAGCTGGGTTTAATCCCTGTTAAGGTCAACGTTTTGT
7_known	N10	Tb927.8.3350_for	GGGGACAAGTTTGTACAAAAAAGCAGGCTATGCGCAAGACGGTGGTG	Tb927.8.3350_rev	GGGGACCACTTTGTACAAGAAAGCTGGGTTCAGTAGGTTGAAGATTGGAGGGTT
7_known	N11	Tb927.9.10920_for	GGGGACAAGTTTGTACAAAAAAGCAGGCTATGATTGGCACAGTAGAACAAATATTGTAC	Tb927.9.10920_rev	GGGGACCACTTTGTACAAGAAAGCTGGGTTTACACGCCACCGTAGAGCA
7_known	N12	Tb927.11.16310_for	GGGGACAAGTTTGTACAAAAAAGCAGGCTATGACCACGGAGCAAAAAGTCG	Tb927.11.16310_rev	GGGGACCACTTTGTACAAGAAAGCTGGGTCTACGTAACGTTGTTAAAAAGGCTACG
7_known	N13	Tb927.10.7280_for	GGGGACAAGTTTGTACAAAAAAGCAGGCTATGAGCGATGAGTTATGTGTGCTG	Tb927.10.7280_rev	GGGGACCACTTTGTACAAGAAAGCTGGGTTCACCTGCGACGCCTCTG
7_known	N14	Tb927.8.7020_for	GGGGACAAGTTTGTACAAAAAAGCAGGCTATGACGCGGGAGGAAGTCC	Tb927.8.7020_rev	GGGGACCACTTTGTACAAGAAAGCTGGGTCTAGAAGGTCCTGCAACGAATGAC
7_known	N15	Tb927.11.5690_for	GGGGACAAGTTTGTACAAAAAAGCAGGCTATGGATGGCGATGAATTATTCGTTTTAAC	Tb927.11.5690_rev	GGGGACCACTTTGTACAAGAAAGCTGGGTCTAGACAGTATCGTCATCAACGTATATGG
7_known	N16	Tb927.3.2040_for	GGGGACAAGTTTGTACAAAAAAGCAGGCTATGAACACTTCCGCGTGTTTTACA	Tb927.3.2040_rev	GGGGACCACTTTGTACAAGAAAGCTGGGTTCATGACTGCATCGCCTTCAC
7_known	N17	Tb927.11.3730_for	GGGGACAAGTTTGTACAAAAAAGCAGGCTATGTCGACTGTACGACGTGATAGA	Tb927.11.3730_rev	GGGGACCACTTTGTACAAGAAAGCTGGGTTCATGCCTTCTTGTCCGGAAAGG
7_known	N18	Tb927.11.9780_for	GGGGACAAGTTTGTACAAAAAAGCAGGCTATGGAACGGTTGATGGAGGGA	Tb927.11.9780_rev	GGGGACCACTTTGTACAAGAAAGCTGGGTCTATGAAACACTTTGTGAAGATGAGACAAA
7_known	N19	Tb927.7.4650_for	GGGGACAAGTTTGTACAAAAAAGCAGGCTATGCCTATGTTTATGGATGGGGCG	Tb927.7.4650_rev	GGGGACCACTTTGTACAAGAAAGCTGGGTTTATATCCACCGCTCGCTCTTTACA
7_known	N20	Tb927.8.6490_for	GGGGACAAGTTTGTACAAAAAAGCAGGCTATGGGTTGTGGTGCCTCCA	Tb927.8.6490_rev	GGGGACCACTTTGTACAAGAAAGCTGGGTTCACGCGTAGAGCCCCCG
7_known	N21	Tb927.4.1510_for	GGGGACAAGTTTGTACAAAAAAGCAGGCTATGCCAGAAGTAGAGAACGATAGCT	Tb927.4.1510_rev	GGGGACCACTTTGTACAAGAAAGCTGGGTTCACTTCATCCGCGCGTTG
7_known	N22	Tb927.8.1200_for	GGGGACAAGTTTGTACAAAAAAGCAGGCTATGCATCCACTTGAGTCACCGC	Tb927.8.1200_rev	GGGGACCACTTTGTACAAGAAAGCTGGGTTCATACCATCCTTAATGTGTCATAAAGTTG
7_known	N23	Tb927.1.1670_for	GGGGACAAGTTTGTACAAAAAAGCAGGCTATGTTGACAGGTGACATCTTGTTGC	Tb927.1.1670_rev	GGGGACCACTTTGTACAAGAAAGCTGGGTTCAGTAGGATGAACACGACTCGT
7_known	N24	Tb927.10.9570_for	GGGGACAAGTTTGTACAAAAAAGCAGGCTATGGAGAAAACACAACCGTTCAACG	Tb927.10.9570_rev	GGGGACCACTTTGTACAAGAAAGCTGGGTCTACTCTTTCACAAGAGCCCGAAC
7_known	O1	Tb927.10.14300_for	GGGGACAAGTTTGTACAAAAAAGCAGGCTATGAGTTCGATGGAGGAATTACACG	Tb927.10.14300_rev	GGGGACCACTTTGTACAAGAAAGCTGGGTTTACACATCCCCTGAATGCGTG
7_known	O2	Tb927.11.15100_for	GGGGACAAGTTTGTACAAAAAAGCAGGCTATGGCCGTTGTCCACAAGAAC	Tb927.11.15100_rev	GGGGACCACTTTGTACAAGAAAGCTGGGTTTACGCATCACCCTCACCAGT
7_known	O3	Tb927.6.2370_for	GGGGACAAGTTTGTACAAAAAAGCAGGCTATGCATGGAAATTTTGTATTTACCGGC	Tb927.6.2370_rev	GGGGACCACTTTGTACAAGAAAGCTGGGTTCAACTGAGACCGAAGTCATCCG
7_known	O4	Tb927.6.2880_for	GGGGACAAGTTTGTACAAAAAAGCAGGCTATGAACGTGCGTGTTGCTACA	Tb927.6.2880_rev	GGGGACCACTTTGTACAAGAAAGCTGGGTCTAACTCCGCCGAGTGATACCG
7_known	O5	Tb11.02.5390_for	GGGGACAAGTTTGTACAAAAAAGCAGGCTATGAATGGGAGGCTGTACTGTTTG	Tb11.02.5390_rev	GGGGACCACTTTGTACAAGAAAGCTGGGTTCAGCGTCTCAATAGAAGCTGAAGA
7_known	O6	Tb927.5.2090_for	GGGGACAAGTTTGTACAAAAAAGCAGGCTATGGGGAAGAAAACCAACAGCG	Tb927.5.2090_rev	GGGGACCACTTTGTACAAGAAAGCTGGGTTTAGAAATCAGCTCTTCGGACGTTC
7_known	O7	Tb927.11.4470_for	GGGGACAAGTTTGTACAAAAAAGCAGGCTATGGACAACTATACCATAGTCGAACTTATA	Tb927.11.4470_rev	GGGGACCACTTTGTACAAGAAAGCTGGGTTCACGAGGACGAAATCCTTTGC
7_known	O8	Tb927.11.13920_for	GGGGACAAGTTTGTACAAAAAAGCAGGCTATGTCTAAGAAACACCAAGGGGCG	Tb927.11.13920_rev	GGGGACCACTTTGTACAAGAAAGCTGGGTTCAGAGACGCCGCGTCGC
7_known	O9	Tb927.6.2770_for	GGGGACAAGTTTGTACAAAAAAGCAGGCTATGAGTATAGCGCGGTTGGAGC	Tb927.6.2770_rev	GGGGACCACTTTGTACAAGAAAGCTGGGTCTATGCGCTCTGCATTTCAACCT
7_known	O10	Tb927.4.3890_for	GGGGACAAGTTTGTACAAAAAAGCAGGCTATGGGCAAGAAGACCAGGGAT	Tb927.4.3890_rev	GGGGACCACTTTGTACAAGAAAGCTGGGTTCAGCCACGCTTTGGTAGGG
7_known	O11	Tb927.10.15020_for	GGGGACAAGTTTGTACAAAAAAGCAGGCTATGGACGCTGACGAGGGC	Tb927.10.15020_rev	GGGGACCACTTTGTACAAGAAAGCTGGGTCTAACGTTGTGAGTATACCTGTAAATATTT
7_known	O12	Tb927.10.15390_for	GGGGACAAGTTTGTACAAAAAAGCAGGCTATGTGTGACCGTCTAAATACAATGTCTATC	Tb927.10.15390_rev	GGGGACCACTTTGTACAAGAAAGCTGGGTTCAGCGTGGCCGTGAAGA
7_known	O13	Tb927.11.1100_for	GGGGACAAGTTTGTACAAAAAAGCAGGCTATGATGAATTGTAGGACTACGGAGCT	Tb927.11.1100_rev	GGGGACCACTTTGTACAAGAAAGCTGGGTTCAGTTTAGCAGCTCATGTGAATGC
7_known	O14	Tb927.6.1110_for	GGGGACAAGTTTGTACAAAAAAGCAGGCTATGGTTAGTGCGGGCAGTGA	Tb927.6.1110_rev	GGGGACCACTTTGTACAAGAAAGCTGGGTCTAATATGCAGTTTTAGCTGATCCACGC
7_known	O15	Tb927.11.11850_for	GGGGACAAGTTTGTACAAAAAAGCAGGCTATGGCCGATGAAGACGGTTCT	Tb927.11.11850_rev	GGGGACCACTTTGTACAAGAAAGCTGGGTTTACAGTAGCACCTCCAACTCGT
7_known	O16	Tb927.11.13060_for	GGGGACAAGTTTGTACAAAAAAGCAGGCTATGGTGAAACAGCGCATGACG	Tb927.11.13060_rev	GGGGACCACTTTGTACAAGAAAGCTGGGTCTATTTGCGAAGGCCAGTGCT
7_known	O17	Tb927.10.11620_for	GGGGACAAGTTTGTACAAAAAAGCAGGCTATGCGTGGGGAGAGCAGT	Tb927.10.11620_rev	GGGGACCACTTTGTACAAGAAAGCTGGGTTTAAACGTATGCAGGGGCAGTTT
7_known	O18	Tb927.8.1160_for	GGGGACAAGTTTGTACAAAAAAGCAGGCTATGATTTCCCCAAAAGACACTTCGAA	Tb927.8.1160_rev	GGGGACCACTTTGTACAAGAAAGCTGGGTTCATCTAATACGTTCCTCGCACCA
7_known	O19	Tb927.10.2220_for	GGGGACAAGTTTGTACAAAAAAGCAGGCTATGATGCGGAGGACTCTTCGT	Tb927.10.2220_rev	GGGGACCACTTTGTACAAGAAAGCTGGGTTTAAAAGAACCACAGTCTTTCATTCAAAGG
7_known	O20	Tb927.10.14610_for	GGGGACAAGTTTGTACAAAAAAGCAGGCTATGTCACGATCATGGTGTACATTTTCC	Tb927.10.14610_rev	GGGGACCACTTTGTACAAGAAAGCTGGGTCTACACCGTTGCGACTACCTG
7_known	O21	Tb927.8.1180_for	GGGGACAAGTTTGTACAAAAAAGCAGGCTATGATTTCCCCAAAAGACACTTCGAA	Tb927.8.1180_rev	GGGGACCACTTTGTACAAGAAAGCTGGGTTCATACCATCCTTAATGTGTCATAAAGTTG
7_known	O22	Tb927.2.690_for	GGGGACAAGTTTGTACAAAAAAGCAGGCTATGTCAGGAAACCAAGGAAGAAAGC	Tb927.2.690_rev	GGGGACCACTTTGTACAAGAAAGCTGGGTTTAACGGATATATACCCTCCTCTTTCTCAG
7_known	O23	Tb927.6.4390_for	GGGGACAAGTTTGTACAAAAAAGCAGGCTATGTCGGATGCCGATGTGAAAG	Tb927.6.4390_rev	GGGGACCACTTTGTACAAGAAAGCTGGGTTCAGTTGGAGCGCAGGCG
7_known	O24	Tb927.11.4640_for	GGGGACAAGTTTGTACAAAAAAGCAGGCTATGAATACGATCGATGCTGCTCTTG	Tb927.11.4640_rev	GGGGACCACTTTGTACAAGAAAGCTGGGTTTACAACACCTCCGCCTTCAGT
7_known	P1	Tb927.11.15330_for	GGGGACAAGTTTGTACAAAAAAGCAGGCTATGACCCAGTCGGCGGAA	Tb927.11.15330_rev	GGGGACCACTTTGTACAAGAAAGCTGGGTTCATTTTGTTTGATTTCGTGCATCCA
7_known	P2	Tb927.9.14650_for	GGGGACAAGTTTGTACAAAAAAGCAGGCTATGAGTAACAACGGTTACTGCAACA	Tb927.9.14650_rev	GGGGACCACTTTGTACAAGAAAGCTGGGTTCATGGATATCCTCCTGCGGG
7_known	P3	Tb927.5.1490_for	GGGGACAAGTTTGTACAAAAAAGCAGGCTATGAACTGCTCCGCGCCA	Tb927.5.1490_rev	GGGGACCACTTTGTACAAGAAAGCTGGGTTTAATTTCCCCCAAGGGACCGA
7_known	P4	Tb927.1.1050_for	GGGGACAAGTTTGTACAAAAAAGCAGGCTATGAATGTCACTAACCCCTCCACT	Tb927.1.1050_rev	GGGGACCACTTTGTACAAGAAAGCTGGGTTCACTCACGGAGAGGATCTGGC
7_known	P5	Tb927.7.4080_for	GGGGACAAGTTTGTACAAAAAAGCAGGCTATGGCAGATGAACTCTCCCAGT	Tb927.7.4080_rev	GGGGACCACTTTGTACAAGAAAGCTGGGTTTACGATCCCTCCACCCTGGG
7_known	P6	Tb927.5.3610_for	GGGGACAAGTTTGTACAAAAAAGCAGGCTATGACGGATGCAACTAAGATGGC	Tb927.5.3610_rev	GGGGACCACTTTGTACAAGAAAGCTGGGTCTACGACGTGGCCGTCATC
7_known	P7	Tb927.11.3350_for	GGGGACAAGTTTGTACAAAAAAGCAGGCTATGGCGGAAGACGCTGCA	Tb927.11.3350_rev	GGGGACCACTTTGTACAAGAAAGCTGGGTCTACCCATGAAGCGTATCCCCG
7_known	P8	Tb927.1.540_for	GGGGACAAGTTTGTACAAAAAAGCAGGCTATGACCCCTAGGGCCCGT	Tb927.1.540_rev	GGGGACCACTTTGTACAAGAAAGCTGGGTTCATTGCGATCCCATGAAATCAAGC
7_known	P9	Tb927.10.12490_for	GGGGACAAGTTTGTACAAAAAAGCAGGCTATGATGTCTTCTTCATCCTCAGCTCA	Tb927.10.12490_rev	GGGGACCACTTTGTACAAGAAAGCTGGGTTCAGAAGAAAAGAGCGGGCCT
7_known	P10	Tb927.5.930_for	GGGGACAAGTTTGTACAAAAAAGCAGGCTATGGTAGACGGGCGATCTTCTG	Tb927.5.930_rev	GGGGACCACTTTGTACAAGAAAGCTGGGTTCAAATTTTGGATGAGCCGCTCG
7_known	P11	Tb927.10.9190_for	GGGGACAAGTTTGTACAAAAAAGCAGGCTATGAATCGATGCTACTCCCTCCTT	Tb927.10.9190_rev	GGGGACCACTTTGTACAAGAAAGCTGGGTTTACGATTCACCAGCCGACGG
7_known	P12	Tb927.3.2790_for	GGGGACAAGTTTGTACAAAAAAGCAGGCTATGGTTCGCGACGATACGC	Tb927.3.2790_rev	GGGGACCACTTTGTACAAGAAAGCTGGGTTCAACGGCGCACACGCCA
7_known	P13	Tb927.1.2100_for	GGGGACAAGTTTGTACAAAAAAGCAGGCTATGTCGGAGTTCGAACTTATGAGTG	Tb927.1.2100_rev	GGGGACCACTTTGTACAAGAAAGCTGGGTTCAGCACACGGAGGAGTCC
7_known	P14	Tb927.10.7180_for	GGGGACAAGTTTGTACAAAAAAGCAGGCTATGTCCGCCAAATACTACATTCCTTT	Tb927.10.7180_rev	GGGGACCACTTTGTACAAGAAAGCTGGGTTTACGGTTTCGCTTCATCTGAGG
7_known	P15	Tb927.7.1190_for	GGGGACAAGTTTGTACAAAAAAGCAGGCTATGCGTCCATTAGTTGAGCGTT	Tb927.7.1190_rev	GGGGACCACTTTGTACAAGAAAGCTGGGTCTACTTCTCTCTATTCTGGTTGTTGCG
7_known	P16	Tb927.10.15300_for	GGGGACAAGTTTGTACAAAAAAGCAGGCTATGTTACTGGATTTCGTCCTAAAGTACG	Tb927.10.15300_rev	GGGGACCACTTTGTACAAGAAAGCTGGGTCTAACGTCCCTTTTGTAGCCACG
7_known	P17	Tb927.8.2380_for	GGGGACAAGTTTGTACAAAAAAGCAGGCTATGCACTCGGAATTGAAAGCCTATG	Tb927.8.2380_rev	GGGGACCACTTTGTACAAGAAAGCTGGGTTCAACGTGCGACAGTAGTGAATTG
7_known	P18	Tb927.1.3120_for	GGGGACAAGTTTGTACAAAAAAGCAGGCTATGAGGCGCACTCAGGTTCG	Tb927.1.3120_rev	GGGGACCACTTTGTACAAGAAAGCTGGGTTCACATCGCTCCACCCGG
7_known	P19	Tb927.2.1810_for	GGGGACAAGTTTGTACAAAAAAGCAGGCTATGGAGGCACCAGCAGGAC	Tb927.2.1810_rev	GGGGACCACTTTGTACAAGAAAGCTGGGTTTATTCCGGAAACTTCCGCTCCA
7_known	P20	Tb927.9.14470_for	GGGGACAAGTTTGTACAAAAAAGCAGGCTATGCTTCAGGTTCTAGGTATTGTGAAG	Tb927.9.14470_rev	GGGGACCACTTTGTACAAGAAAGCTGGGTTTAATTGTGGATAACAACCTTATCCTCTTT
7_known	P21	Tb927.8.5210_for	GGGGACAAGTTTGTACAAAAAAGCAGGCTATGCCCTTGTCTGGGGGC	Tb927.8.5210_rev	GGGGACCACTTTGTACAAGAAAGCTGGGTTCACACAACGTACTCGAGTAATTGTG
7_known	P22	Tb927.10.11080_for	GGGGACAAGTTTGTACAAAAAAGCAGGCTATGTATAATAATTCCCCAGGCGAGGG	Tb927.10.11080_rev	GGGGACCACTTTGTACAAGAAAGCTGGGTTCACCTCAGGCTGCGCAA
7_known	P23	Tb927.10.1270_for	GGGGACAAGTTTGTACAAAAAAGCAGGCTATGCGCAACAATTTCCCGATTCC	Tb927.10.1270_rev	GGGGACCACTTTGTACAAGAAAGCTGGGTTCACTCAGTAGAGGAACCGCATG
7_known	P24	Tb927.4.3380_for	GGGGACAAGTTTGTACAAAAAAGCAGGCTATGGCGCATCTGGACCTTAATAAAC	Tb927.4.3380_rev	GGGGACCACTTTGTACAAGAAAGCTGGGTTTACCGCTTTGACCGTTGCTC
8_hypothetical	A1	Tb927.11.400_for	GGGGACAAGTTTGTACAAAAAAGCAGGCTATGAATATTTATGAATGGCAGTCAATTGAC	Tb927.11.400_rev	GGGGACCACTTTGTACAAGAAAGCTGGGTCTACACATTAACACCCGTACTAATGGG
8_hypothetical	A2	Tb927.6.910_for	GGGGACAAGTTTGTACAAAAAAGCAGGCTATGGTACTTGGTGGTGTAGTTGGG	Tb927.6.910_rev	GGGGACCACTTTGTACAAGAAAGCTGGGTCTAGTCGACTGGTGAGGCCTC
8_hypothetical	A3	Tb927.7.2240_for	GGGGACAAGTTTGTACAAAAAAGCAGGCTATGCCGGTTAAGAAGAAGGTTGC	Tb927.7.2240_rev	GGGGACCACTTTGTACAAGAAAGCTGGGTTTAATACTTCCAACCACGACTTCTTCC
8_hypothetical	A4	Tb927.11.13560_for	GGGGACAAGTTTGTACAAAAAAGCAGGCTATGCGATGTTGTCGGACAATGC	Tb927.11.13560_rev	GGGGACCACTTTGTACAAGAAAGCTGGGTTTACGCGTTTTGGGGTGGAAA
8_hypothetical	A5	Tb927.11.12450.1_for	GGGGACAAGTTTGTACAAAAAAGCAGGCTATGGCTGAGATGACGGAGGC	Tb927.11.12450.1_rev	GGGGACCACTTTGTACAAGAAAGCTGGGTTTAGGGGGCCGCAAGTATTTTTC
8_hypothetical	A6	Tb927.11.6840_for	GGGGACAAGTTTGTACAAAAAAGCAGGCTATGGACACATCTGAGGAGGCAA	Tb927.11.6840_rev	GGGGACCACTTTGTACAAGAAAGCTGGGTTCACAACGAAAACGACCTAACGAC
8_hypothetical	A7	Tb927.8.6470_for	GGGGACAAGTTTGTACAAAAAAGCAGGCTATGCCTGGGTGTCTTGAGGT	Tb927.8.6470_rev	GGGGACCACTTTGTACAAGAAAGCTGGGTTTATGTTGGTTCACATTCAGTTACGCA
8_hypothetical	A8	Tb927.10.3350_for	GGGGACAAGTTTGTACAAAAAAGCAGGCTATGTGCCAAACTTTAACTACGGACG	Tb927.10.3350_rev	GGGGACCACTTTGTACAAGAAAGCTGGGTTCATCTGACTCTCTCGTGTGAGG
8_hypothetical	A9	Tb927.5.2340_for	GGGGACAAGTTTGTACAAAAAAGCAGGCTATGTTGGAGGGACTATGCGTGC	Tb927.5.2340_rev	GGGGACCACTTTGTACAAGAAAGCTGGGTTCACCTCATAAACTTTCTCCGCCA
8_hypothetical	A10	Tb927.11.1080_for	GGGGACAAGTTTGTACAAAAAAGCAGGCTATGACGAACAGAGAAAACACGTGG	Tb927.11.1080_rev	GGGGACCACTTTGTACAAGAAAGCTGGGTTTAGTCACCCTCAAAGGAAGATAATGACC
8_hypothetical	A11	Tb927.11.13070_for	GGGGACAAGTTTGTACAAAAAAGCAGGCTATGGATGAGCGGTCATTTGAGTTG	Tb927.11.13070_rev	GGGGACCACTTTGTACAAGAAAGCTGGGTTCATACCCCCTCGACCGGC
8_hypothetical	A12	Tb927.8.2590_for	GGGGACAAGTTTGTACAAAAAAGCAGGCTATGGCGGTGTCGGGATCG	Tb927.8.2590_rev	GGGGACCACTTTGTACAAGAAAGCTGGGTTTAATCTTCTTCCAGCAACCGGTTAAG
8_hypothetical	A13	Tb927.10.7010_for	GGGGACAAGTTTGTACAAAAAAGCAGGCTATGAACATTTCTGATATCGTGGGAGAGG	Tb927.10.7010_rev	GGGGACCACTTTGTACAAGAAAGCTGGGTTCACACGATCACTTGGGCTTCT
8_hypothetical	A14	Tb927.3.4710_for	GGGGACAAGTTTGTACAAAAAAGCAGGCTATGAGCATGCAACACACCCTC	Tb927.3.4710_rev	GGGGACCACTTTGTACAAGAAAGCTGGGTTTAGATCCTGGCGGTATCGCA
8_hypothetical	A15	Tb927.10.5830_for	GGGGACAAGTTTGTACAAAAAAGCAGGCTATGTATTTACATTCCATGAAGCGCTACA	Tb927.10.5830_rev	GGGGACCACTTTGTACAAGAAAGCTGGGTTCAAATTTTGACGGGCTTCCGC
8_hypothetical	A16	Tb927.10.8860_for	GGGGACAAGTTTGTACAAAAAAGCAGGCTATGACCACAATTTTAGAAAATGACAGAGC	Tb927.10.8860_rev	GGGGACCACTTTGTACAAGAAAGCTGGGTTTAGTCGTTCTGTGGGACGTACG
8_hypothetical	A17	Tb927.3.1580_for	GGGGACAAGTTTGTACAAAAAAGCAGGCTATGGAAACGGTCCCTGAGTCG	Tb927.3.1580_rev	GGGGACCACTTTGTACAAGAAAGCTGGGTTCACATGAAAGATGTAGCCAAGCTC
8_hypothetical	A18	Tb927.11.14700_for	GGGGACAAGTTTGTACAAAAAAGCAGGCTATGTATTTCAAGCCATCTCTGACGC	Tb927.11.14700_rev	GGGGACCACTTTGTACAAGAAAGCTGGGTTTACTTTTCGATAGTGTCCTTTTTTTCAGT
8_hypothetical	A19	Tb927.8.6700_for	GGGGACAAGTTTGTACAAAAAAGCAGGCTATGTCCTCCGTTACCACTGGC	Tb927.8.6700_rev	GGGGACCACTTTGTACAAGAAAGCTGGGTTTAAGAAGCCTCCGGCGAGG
8_hypothetical	A20	Tb927.10.9380_for	GGGGACAAGTTTGTACAAAAAAGCAGGCTATGCCAGTGGATGCGCCC	Tb927.10.9380_rev	GGGGACCACTTTGTACAAGAAAGCTGGGTCTATGACTTTGTTTCCCTTGGTGGC

8_hypothetical	A21	Tb927.4.490_for	GGGGACAAGTTTGTACAAAAAAGCAGGCTATGTCGAAACGCAAGCGGT	Tb927.4.490_rev	GGGGACCACTTTGTACAAGAAAGCTGGGTCTAACTATCCGACGATATTTCTATGCCG
8_hypothetical	A22	Tb927.6.3190_for	GGGGACAAGTTTGTACAAAAAAGCAGGCTATGGAAAGCACCATCATGTCTATCG	Tb927.6.3190_rev	GGGGACCACTTTGTACAAGAAAGCTGGGTTTATTCATTTTCTTCTTCGTCAGAATTATC
8_hypothetical	A23	Tb927.11.4050_for	GGGGACAAGTTTGTACAAAAAAGCAGGCTATGACCGTCTGCTTCAAATGCA	Tb927.11.4050_rev	GGGGACCACTTTGTACAAGAAAGCTGGGTTTAACTGTCTTTCTCACCATCATCTGTC
8_hypothetical	A24	Tb927.7.6300_for	GGGGACAAGTTTGTACAAAAAAGCAGGCTATGGGTTGCGGTTCGAGTGT	Tb927.7.6300_rev	GGGGACCACTTTGTACAAGAAAGCTGGGTCTACATGAGTTCTTGGATATATATATTCCC
8_hypothetical	B1	Tb927.10.6580_for	GGGGACAAGTTTGTACAAAAAAGCAGGCTATGAGCTTAAGACTTGGTCAGCCG	Tb927.10.6580_rev	GGGGACCACTTTGTACAAGAAAGCTGGGTCTACGCCCCGAGTGCTACA
8_hypothetical	B2	Tb927.11.3700_for	GGGGACAAGTTTGTACAAAAAAGCAGGCTATGACTGACAGCGAGTCGGA	Tb927.11.3700_rev	GGGGACCACTTTGTACAAGAAAGCTGGGTTCACTTCCCGCGTTCCACC
8_hypothetical	B3	Tb927.10.2470_for	GGGGACAAGTTTGTACAAAAAAGCAGGCTATGCCACAAAATATGGAAGTACGTGA	Tb927.10.2470_rev	GGGGACCACTTTGTACAAGAAAGCTGGGTCTAGTCCCCCTTTTCCCTCGTG
8_hypothetical	B4	Tb927.3.760_for	GGGGACAAGTTTGTACAAAAAAGCAGGCTATGTCAGTTTTAAGCGAATGGAGGC	Tb927.3.760_rev	GGGGACCACTTTGTACAAGAAAGCTGGGTTTAAGAGGCTCGGAGAATGACAGC
8_hypothetical	B5	Tb927.11.11380_for	GGGGACAAGTTTGTACAAAAAAGCAGGCTATGCTGCGCCGCGGTGCG	Tb927.11.11380_rev	GGGGACCACTTTGTACAAGAAAGCTGGGTTTAGCTCTTTTTTTTATCGTCGTCTTCATT
8_hypothetical	B6	Tb927.11.6540_for	GGGGACAAGTTTGTACAAAAAAGCAGGCTATGCAACTTCGACATGAATTGAAGTTTG	Tb927.11.6540_rev	GGGGACCACTTTGTACAAGAAAGCTGGGTTCAGTTAGTCACTGCAGGCTCA
8_hypothetical	B7	Tb927.11.2000_for	GGGGACAAGTTTGTACAAAAAAGCAGGCTATGGCGCAGAGCTGGTTCA	Tb927.11.2000_rev	GGGGACCACTTTGTACAAGAAAGCTGGGTTCAGAAGCTGAACGCAAAGCC
8_hypothetical	B8	Tb927.8.4940_for	GGGGACAAGTTTGTACAAAAAAGCAGGCTATGGGATCCTGCTGCGGAG	Tb927.8.4940_rev	GGGGACCACTTTGTACAAGAAAGCTGGGTTCAAATTCCCCCCGTCCTGC
8_hypothetical	B9	Tb927.3.3950_for	GGGGACAAGTTTGTACAAAAAAGCAGGCTATGCCTCCAAAACGCTCAAAAGG	Tb927.3.3950_rev	GGGGACCACTTTGTACAAGAAAGCTGGGTTTACAAGAAGGACAATGTCTTCCTCG
8_hypothetical	B10	Tb927.9.15480_for	GGGGACAAGTTTGTACAAAAAAGCAGGCTATGAAAGTTCCAATAGGCATCTTGCG	Tb927.9.15480_rev	GGGGACCACTTTGTACAAGAAAGCTGGGTTCAGACGAAGAGAACAGATGTGAGA
8_hypothetical	B11	Tb927.6.4190_for	GGGGACAAGTTTGTACAAAAAAGCAGGCTATGAAGTATATGGCAAAGGGGGCG	Tb927.6.4190_rev	GGGGACCACTTTGTACAAGAAAGCTGGGTTTATGACAAGTGGGGCTGAAGATGC
8_hypothetical	B12	Tb927.9.4050_for	GGGGACAAGTTTGTACAAAAAAGCAGGCTATGAGTGTCACCGGCTTCGG	Tb927.9.4050_rev	GGGGACCACTTTGTACAAGAAAGCTGGGTTCACTCCAAATCCCTCATCAAACTCT
8_hypothetical	B13	Tb927.7.1700_for	GGGGACAAGTTTGTACAAAAAAGCAGGCTATGAGGTACAGTGTTGTCAGAACGC	Tb927.7.1700_rev	GGGGACCACTTTGTACAAGAAAGCTGGGTTTACGGAAATAGTACAGTTCGGCG
8_hypothetical	B14	Tb927.11.9440_for	GGGGACAAGTTTGTACAAAAAAGCAGGCTATGAACGAATTAAAGAACCCGATGCG	Tb927.11.9440_rev	GGGGACCACTTTGTACAAGAAAGCTGGGTTTACATATTTAATCTAATGTCCTTGATGTC
8_hypothetical	B15	Tb927.11.9540_for	GGGGACAAGTTTGTACAAAAAAGCAGGCTATGAAAACGTACAGTGAGCTGCTG	Tb927.11.9540_rev	GGGGACCACTTTGTACAAGAAAGCTGGGTTCACTTTGACAACTCAACGAGCC
8_hypothetical	B16	Tb927.9.6660_for	GGGGACAAGTTTGTACAAAAAAGCAGGCTATGGCAGTTGATCGTATCGGAAAC	Tb927.9.6660_rev	GGGGACCACTTTGTACAAGAAAGCTGGGTTCAACTGGACTCAAACTCCTGCT
8_hypothetical	B17	Tb927.7.1400_for	GGGGACAAGTTTGTACAAAAAAGCAGGCTATGAACAATGCTAATGACACTTTAGATTAC	Tb927.7.1400_rev	GGGGACCACTTTGTACAAGAAAGCTGGGTTCACGCACCCTCCCGTCG
8_hypothetical	B18	Tb927.8.820_for	GGGGACAAGTTTGTACAAAAAAGCAGGCTATGTCATTCCACAAGTTATTTTCTTCAAAT	Tb927.8.820_rev	GGGGACCACTTTGTACAAGAAAGCTGGGTCTATTCACTCGTCGACCGCCA
8_hypothetical	B19	Tb927.11.11790_for	GGGGACAAGTTTGTACAAAAAAGCAGGCTATGGCGGCAACAGGTTCAC	Tb927.11.11790_rev	GGGGACCACTTTGTACAAGAAAGCTGGGTTTACGCGGGCTTGCTATCGC
8_hypothetical	B20	Tb927.9.3630_for	GGGGACAAGTTTGTACAAAAAAGCAGGCTATGGCCGCGTCGCAGGTA	Tb927.9.3630_rev	GGGGACCACTTTGTACAAGAAAGCTGGGTTCACTCATCGACCTCGTCTTCC
8_hypothetical	B21	Tb927.11.7214_for	GGGGACAAGTTTGTACAAAAAAGCAGGCTATGAAGAGATACCGTTCCTCTGTTGT	Tb927.11.7214_rev	GGGGACCACTTTGTACAAGAAAGCTGGGTTCAAAAACGATAACACGAAAGGCAAG
8_hypothetical	B22	Tb927.7.5400_for	GGGGACAAGTTTGTACAAAAAAGCAGGCTATGTATACAACAACAGACCTCATACAGAC	Tb927.7.5400_rev	GGGGACCACTTTGTACAAGAAAGCTGGGTCTACGTGTTAAACAAACCACCAGACG
8_hypothetical	B23	Tb927.11.12490_for	GGGGACAAGTTTGTACAAAAAAGCAGGCTATGAGCTCCAGGATGTCACAGAA	Tb927.11.12490_rev	GGGGACCACTTTGTACAAGAAAGCTGGGTCTAACGTGTGCGAACACGCT
8_hypothetical	B24	Tb927.10.15430_for	GGGGACAAGTTTGTACAAAAAAGCAGGCTATGAAGAAGAATAACTCCAAGGGTAACTCC	Tb927.10.15430_rev	GGGGACCACTTTGTACAAGAAAGCTGGGTTTAACCGAACAAACTTACAATGGTATTCCT
8_hypothetical	C1	Tb927.3.2010_for	GGGGACAAGTTTGTACAAAAAAGCAGGCTATGGATACCCTCGCATTCCGC	Tb927.3.2010_rev	GGGGACCACTTTGTACAAGAAAGCTGGGTTTACCGACTAGGCAAATCCGCG
8_hypothetical	C2	Tb927.11.8700_for	GGGGACAAGTTTGTACAAAAAAGCAGGCTATGGTTTCGTTTATTTACAGCTGCG	Tb927.11.8700_rev	GGGGACCACTTTGTACAAGAAAGCTGGGTTTATGTGACGATGCGAATTCCCG
8_hypothetical	C3	Tb927.11.12400_for	GGGGACAAGTTTGTACAAAAAAGCAGGCTATGATCCATGCCACCGGTGA	Tb927.11.12400_rev	GGGGACCACTTTGTACAAGAAAGCTGGGTTCACTTTCCGTACACCACAGTGC
8_hypothetical	C4	Tb927.10.10070_for	GGGGACAAGTTTGTACAAAAAAGCAGGCTATGGATCTTGACAACGTGGTTCTTG	Tb927.10.10070_rev	GGGGACCACTTTGTACAAGAAAGCTGGGTTCAGGAATTAAGATCGCCAAAGAAACC
8_hypothetical	C5	Tb927.10.14390_for	GGGGACAAGTTTGTACAAAAAAGCAGGCTATGTCGAGCTTGGCAGGAGT	Tb927.10.14390_rev	GGGGACCACTTTGTACAAGAAAGCTGGGTTTAATGATCTTTCTTGTGCTTTTTATGACG
8_hypothetical	C6	Tb927.11.14530_for	GGGGACAAGTTTGTACAAAAAAGCAGGCTATGCCGGTTACGACACACTTTT	Tb927.11.14530_rev	GGGGACCACTTTGTACAAGAAAGCTGGGTCTAATCAATTATGAAGACGACACCGGG
8_hypothetical	C7	Tb927.5.3480_for	GGGGACAAGTTTGTACAAAAAAGCAGGCTATGAGTTCTACAAAGGATGGATCTCAAATT	Tb927.5.3480_rev	GGGGACCACTTTGTACAAGAAAGCTGGGTCTAAGGTTTCTTAACCGCAAGTTGTACC
8_hypothetical	C8	Tb927.10.3320_for	GGGGACAAGTTTGTACAAAAAAGCAGGCTATGCGAGGGTGTGCAAAAGG	Tb927.10.3320_rev	GGGGACCACTTTGTACAAGAAAGCTGGGTTCAAGTACCTTTCGTGCTATTGTCG
8_hypothetical	C9	Tb927.3.3650_for	GGGGACAAGTTTGTACAAAAAAGCAGGCTATGGAGCCTCTGTGGGACTC	Tb927.3.3650_rev	GGGGACCACTTTGTACAAGAAAGCTGGGTCTAACGACCCTTTCTTGACCTCCC
8_hypothetical	C10	Tb927.11.12190_for	GGGGACAAGTTTGTACAAAAAAGCAGGCTATGTCAGGTTGCGACGGGA	Tb927.11.12190_rev	GGGGACCACTTTGTACAAGAAAGCTGGGTCTACTCTTTCCGCTTTTCTTGGTAGC
8_hypothetical	C11	Tb927.10.10330_for	GGGGACAAGTTTGTACAAAAAAGCAGGCTATGACAGCTCAGAGAGACCACC	Tb927.10.10330_rev	GGGGACCACTTTGTACAAGAAAGCTGGGTCTACTCAGGCTCTTCCATGGATATTCT
8_hypothetical	C12	Tb927.11.5880_for	GGGGACAAGTTTGTACAAAAAAGCAGGCTATGATGCACTTGTCGTTACAACTATGT	Tb927.11.5880_rev	GGGGACCACTTTGTACAAGAAAGCTGGGTTCACCCTGAGGGAAGTAAATGTTTTTT
8_hypothetical	C13	Tb927.11.8920_for	GGGGACAAGTTTGTACAAAAAAGCAGGCTATGGATGAGGACATGTGTGTTGC	Tb927.11.8920_rev	GGGGACCACTTTGTACAAGAAAGCTGGGTTTAAAAATTCTGTGACAGGAAATTTTCATA
8_hypothetical	C14	Tb927.1.3050_for	GGGGACAAGTTTGTACAAAAAAGCAGGCTATGGAGGGGACGGAGTGC	Tb927.1.3050_rev	GGGGACCACTTTGTACAAGAAAGCTGGGTTTATGAGCTATTACTTCTGGAAAGGACGG
8_hypothetical	C15	Tb927.8.7770_for	GGGGACAAGTTTGTACAAAAAAGCAGGCTATGCAGTTTTGTGGCGGGC	Tb927.8.7770_rev	GGGGACCACTTTGTACAAGAAAGCTGGGTTCACCAGTCATGCACAACATAATGT
8_hypothetical	C16	Tb927.11.7340_for	GGGGACAAGTTTGTACAAAAAAGCAGGCTATGCAAAGTTCCTCTGAGACGATG	Tb927.11.7340_rev	GGGGACCACTTTGTACAAGAAAGCTGGGTTCAAAACCGTGAACCCCTGC
8_hypothetical	C17	Tb927.10.3590_for	GGGGACAAGTTTGTACAAAAAAGCAGGCTATGTTCCGCCGGACAAGTCG	Tb927.10.3590_rev	GGGGACCACTTTGTACAAGAAAGCTGGGTCTATCCATGCGGCATGTTGTCC
8_hypothetical	C18	Tb927.11.13180_for	GGGGACAAGTTTGTACAAAAAAGCAGGCTATGCCTTCAAAGCGGAAAACTATCG	Tb927.11.13180_rev	GGGGACCACTTTGTACAAGAAAGCTGGGTTTAGATGAACTCGAGGGACTCCAC
8_hypothetical	C19	Tb927.11.9300_for	GGGGACAAGTTTGTACAAAAAAGCAGGCTATGGGAAACAGGTTTGAAGGGAGT	Tb927.11.9300_rev	GGGGACCACTTTGTACAAGAAAGCTGGGTCTAAGAAAGCAGTCTCGCACCG
8_hypothetical	C20	Tb927.4.1970_for	GGGGACAAGTTTGTACAAAAAAGCAGGCTATGGGTGGGCACCACGGA	Tb927.4.1970_rev	GGGGACCACTTTGTACAAGAAAGCTGGGTTCACAGTGAAATGTTGGAAAGCATTG
8_hypothetical	C21	Tb927.7.7150_for	GGGGACAAGTTTGTACAAAAAAGCAGGCTATGGAATTGTTGGGGAACGCAA	Tb927.7.7150_rev	GGGGACCACTTTGTACAAGAAAGCTGGGTTCATATGCTGTGCACAATCCTCC
8_hypothetical	C22	Tb927.3.4140_for	GGGGACAAGTTTGTACAAAAAAGCAGGCTATGGTACGGCGGCGGCCT	Tb927.3.4140_rev	GGGGACCACTTTGTACAAGAAAGCTGGGTTTACTGTCTGGTGAGTTCACAAACC
8_hypothetical	C23	Tb927.6.3640_for	GGGGACAAGTTTGTACAAAAAAGCAGGCTATGACGGAGGTTCCGGGG	Tb927.6.3640_rev	GGGGACCACTTTGTACAAGAAAGCTGGGTTCAGCACGACACAATCAAGGATTC
8_hypothetical	C24	Tb927.4.1650_for	GGGGACAAGTTTGTACAAAAAAGCAGGCTATGCCGGAGAATCCCCAACG	Tb927.4.1650_rev	GGGGACCACTTTGTACAAGAAAGCTGGGTTCACCGTGATGTGATTGCTGC
8_hypothetical	D1	Tb927.9.11480_for	GGGGACAAGTTTGTACAAAAAAGCAGGCTATGACAGGTGATGTTCGGCCG	Tb927.9.11480_rev	GGGGACCACTTTGTACAAGAAAGCTGGGTTTACGACTGACGCGCCACA
8_hypothetical	D2	Tb927.8.5300_for	GGGGACAAGTTTGTACAAAAAAGCAGGCTATGACCGCTGTTGGGGGC	Tb927.8.5300_rev	GGGGACCACTTTGTACAAGAAAGCTGGGTTTAGGACACTATTGCCGGCACC
8_hypothetical	D3	Tb927.7.3150_for	GGGGACAAGTTTGTACAAAAAAGCAGGCTATGGTGAAAACGGTGTCGTCG	Tb927.7.3150_rev	GGGGACCACTTTGTACAAGAAAGCTGGGTTCACATTACCGTACAACAGGAGGA
8_hypothetical	D4	Tb927.2.4560_for	GGGGACAAGTTTGTACAAAAAAGCAGGCTATGTTAACAGGTCGACAAAAGGTTGT	Tb927.2.4560_rev	GGGGACCACTTTGTACAAGAAAGCTGGGTTTACGCAAGCATGCTTGGCG
8_hypothetical	D5	Tb927.2.2990_for	GGGGACAAGTTTGTACAAAAAAGCAGGCTATGTCACGGCGTGCTGAGG	Tb927.2.2990_rev	GGGGACCACTTTGTACAAGAAAGCTGGGTTTAGTAAAAGTCCAAAACTAGCATCATGTC
8_hypothetical	D6	Tb927.11.1210_for	GGGGACAAGTTTGTACAAAAAAGCAGGCTATGAGCAACAAATACCTTTTGGATGAGA	Tb927.11.1210_rev	GGGGACCACTTTGTACAAGAAAGCTGGGTTCACGCCACCACAGAGCA
8_hypothetical	D7	Tb927.11.11350_for	GGGGACAAGTTTGTACAAAAAAGCAGGCTATGCAATCAACGTCTGCAGGT	Tb927.11.11350_rev	GGGGACCACTTTGTACAAGAAAGCTGGGTTTACTTTTCACCGTACCATTTAGCAAGT
8_hypothetical	D8	Tb927.3.4370_for	GGGGACAAGTTTGTACAAAAAAGCAGGCTATGCAGACGGTATGGAATGTTCC	Tb927.3.4370_rev	GGGGACCACTTTGTACAAGAAAGCTGGGTCTACGCACTCCCAAGAAAAATTAATGC
8_hypothetical	D9	Tb927.3.5040_for	GGGGACAAGTTTGTACAAAAAAGCAGGCTATGACACAACCTGCCCACCT	Tb927.3.5040_rev	GGGGACCACTTTGTACAAGAAAGCTGGGTTCACATGTACGCCACTTCATCTTC
8_hypothetical	D10	Tb927.8.7560_for	GGGGACAAGTTTGTACAAAAAAGCAGGCTATGATACCGAATGGCGGGGA	Tb927.8.7560_rev	GGGGACCACTTTGTACAAGAAAGCTGGGTCTAAAAGACTAGTTTGTCCGGTCTGC
8_hypothetical	D11	Tb927.6.3460_for	GGGGACAAGTTTGTACAAAAAAGCAGGCTATGGTACTAAATGTGATTCTTCTTGGTACG	Tb927.6.3460_rev	GGGGACCACTTTGTACAAGAAAGCTGGGTTCACAATGACGAATCCTGAGAAAGC
8_hypothetical	D12	Tb927.11.16250_for	GGGGACAAGTTTGTACAAAAAAGCAGGCTATGCAGCGGGTTCCGCCG	Tb927.11.16250_rev	GGGGACCACTTTGTACAAGAAAGCTGGGTCTAATTTTCCTCCGATTTGACAGCGG
8_hypothetical	D13	Tb927.11.16370_for	GGGGACAAGTTTGTACAAAAAAGCAGGCTATGTTAACGCCCGTGTTCGTT	Tb927.11.16370_rev	GGGGACCACTTTGTACAAGAAAGCTGGGTTCACGAAATTGGGAGGCCTAAGT
8_hypothetical	D14	Tb927.6.1220_for	GGGGACAAGTTTGTACAAAAAAGCAGGCTATGGAATGCGCGTCGCAG	Tb927.6.1220_rev	GGGGACCACTTTGTACAAGAAAGCTGGGTTTACCACAGTGTGGAGGTCCTG
8_hypothetical	D15	Tb927.5.3370_for	GGGGACAAGTTTGTACAAAAAAGCAGGCTATGGACACTACAGGGAATTCTCCAG	Tb927.5.3370_rev	GGGGACCACTTTGTACAAGAAAGCTGGGTTTACCTTTGTAATGATGGCGACTTCG
8_hypothetical	D16	Tb927.11.9210_for	GGGGACAAGTTTGTACAAAAAAGCAGGCTATGGATGTTTATCGACAAGCAAGTGA	Tb927.11.9210_rev	GGGGACCACTTTGTACAAGAAAGCTGGGTTCACCGCTTCTTCTTCGACCT
8_hypothetical	D17	Tb927.9.9280_for	GGGGACAAGTTTGTACAAAAAAGCAGGCTATGAGCAACAACCCATACCTTGC	Tb927.9.9280_rev	GGGGACCACTTTGTACAAGAAAGCTGGGTTCAGGGGCATCCCCCACT
8_hypothetical	D18	Tb927.3.5000_for	GGGGACAAGTTTGTACAAAAAAGCAGGCTATGTTCATTTATGTCCGCTGCTTACT	Tb927.3.5000_rev	GGGGACCACTTTGTACAAGAAAGCTGGGTCTAGAGCATACGTCGTCTCACCT
8_hypothetical	D19	Tb927.9.9220_for	GGGGACAAGTTTGTACAAAAAAGCAGGCTATGTCTTCAGATTGCGCTGGAC	Tb927.9.9220_rev	GGGGACCACTTTGTACAAGAAAGCTGGGTCTACCTATTTATGGCAGCTGTGCA
8_hypothetical	D20	Tb927.6.5080_for	GGGGACAAGTTTGTACAAAAAAGCAGGCTATGCATTGTAGGACAGCATCTGATG	Tb927.6.5080_rev	GGGGACCACTTTGTACAAGAAAGCTGGGTTTACCCATCATTAGTCTTTTGTTTAAGTTG
8_hypothetical	D21	Tb927.9.3130_for	GGGGACAAGTTTGTACAAAAAAGCAGGCTATGGATCTTGGTAGTGACGAGGATC	Tb927.9.3130_rev	GGGGACCACTTTGTACAAGAAAGCTGGGTCTATTGCTTTTCTGTTCCCCCCC
8_hypothetical	D22	Tb927.8.3940_for	GGGGACAAGTTTGTACAAAAAAGCAGGCTATGGTGAAGGTCGAACCCCC	Tb927.8.3940_rev	GGGGACCACTTTGTACAAGAAAGCTGGGTCTACTTCTGCACAAATCGGAGTATCC
8_hypothetical	D23	Tb927.11.4350_for	GGGGACAAGTTTGTACAAAAAAGCAGGCTATGCAGTCTAGAGTATCCCCGGG	Tb927.11.4350_rev	GGGGACCACTTTGTACAAGAAAGCTGGGTTCAGTTAGCTGTGACTGGCTCC
8_hypothetical	D24	Tb927.10.10740_for	GGGGACAAGTTTGTACAAAAAAGCAGGCTATGGGACTCAGGGACCTGGA	Tb927.10.10740_rev	GGGGACCACTTTGTACAAGAAAGCTGGGTTTACACAACAAGGTCGTTCGTTGA
8_hypothetical	E1	Tb927.8.3050_for	GGGGACAAGTTTGTACAAAAAAGCAGGCTATGACCACAGCAACCGTCAAG	Tb927.8.3050_rev	GGGGACCACTTTGTACAAGAAAGCTGGGTTCACTGTATGGCAAAGCCACG
8_hypothetical	E2	Tb927.11.14540_for	GGGGACAAGTTTGTACAAAAAAGCAGGCTATGGATACCGCGTACGCGA	Tb927.11.14540_rev	GGGGACCACTTTGTACAAGAAAGCTGGGTTTACAAGGGACAAAGTATTGTTTTTTTCTT
8_hypothetical	E3	Tb927.9.11970_for	GGGGACAAGTTTGTACAAAAAAGCAGGCTATGGCGATGCATCGGCTATG	Tb927.9.11970_rev	GGGGACCACTTTGTACAAGAAAGCTGGGTCTAGCAGAGTTTCATGGTTTTGTTTGG
8_hypothetical	E4	Tb927.11.10430_for	GGGGACAAGTTTGTACAAAAAAGCAGGCTATGCGAAACGATAAGTCGATGCC	Tb927.11.10430_rev	GGGGACCACTTTGTACAAGAAAGCTGGGTTCAGGTCTTGCGCTTATGCTTG
8_hypothetical	E5	Tb927.11.15140_for	GGGGACAAGTTTGTACAAAAAAGCAGGCTATGTTCCAACGCCTTGACGAG	Tb927.11.15140_rev	GGGGACCACTTTGTACAAGAAAGCTGGGTTCATTTTCGGGGTGTCACAAGATAAG
8_hypothetical	E6	Tb927.11.2030_for	GGGGACAAGTTTGTACAAAAAAGCAGGCTATGTCTTCATCTTTCTTGGTTCCTTTCA	Tb927.11.2030_rev	GGGGACCACTTTGTACAAGAAAGCTGGGTTCAACGGTTCAAGTAATATGGGTGATTC
8_hypothetical	E7	Tb927.11.1360_for	GGGGACAAGTTTGTACAAAAAAGCAGGCTATGACGATGTCGGACACAAGTG	Tb927.11.1360_rev	GGGGACCACTTTGTACAAGAAAGCTGGGTTTACAACCGTATTGGTGGGTTCCC
8_hypothetical	E8	Tb927.9.1880_for	GGGGACAAGTTTGTACAAAAAAGCAGGCTATGGCTAAACGCACAGTCACAG	Tb927.9.1880_rev	GGGGACCACTTTGTACAAGAAAGCTGGGTCTACAGATACGTAATACCTGGCGTTATG
8_hypothetical	E9	Tb927.7.760_for	GGGGACAAGTTTGTACAAAAAAGCAGGCTATGCCTCCGAAGAAAAGCGAG	Tb927.7.760_rev	GGGGACCACTTTGTACAAGAAAGCTGGGTTTAGTTGTCTTCACTCTCAAGGGGA
8_hypothetical	E10	Tb927.11.1040_for	GGGGACAAGTTTGTACAAAAAAGCAGGCTATGCTATTTTATCTGCATATCAATTTATTT	Tb927.11.1040_rev	GGGGACCACTTTGTACAAGAAAGCTGGGTTCACTCCTTCAAACGGTACTTTCTACC
8_hypothetical	E11	Tb927.7.5110_for	GGGGACAAGTTTGTACAAAAAAGCAGGCTATGGTGGTGGGTGGTGATCG	Tb927.7.5110_rev	GGGGACCACTTTGTACAAGAAAGCTGGGTTCACTTCACTATCTTGGGCATCAGC
8_hypothetical	E12	Tb927.10.10840_for	GGGGACAAGTTTGTACAAAAAAGCAGGCTATGTCCTTGTGGCTAACATTGTTTTCG	Tb927.10.10840_rev	GGGGACCACTTTGTACAAGAAAGCTGGGTTTACAAATGTATGATATTGTGGGGTAAAAG
8_hypothetical	E13	Tb927.4.2790_for	GGGGACAAGTTTGTACAAAAAAGCAGGCTATGATACACAGAAGTTTGCGTTCCA	Tb927.4.2790_rev	GGGGACCACTTTGTACAAGAAAGCTGGGTTCAGTCCACTTCACCGGTGG
8_hypothetical	E14	Tb927.11.1830_for	GGGGACAAGTTTGTACAAAAAAGCAGGCTATGTATAATGCCCTGAGGTCAGCA	Tb927.11.1830_rev	GGGGACCACTTTGTACAAGAAAGCTGGGTCTACGAAGCTTTCGCATCCCTAG
8_hypothetical	E15	Tb927.8.7470_for	GGGGACAAGTTTGTACAAAAAAGCAGGCTATGCAAAGCGGCAGTGAGAA	Tb927.8.7470_rev	GGGGACCACTTTGTACAAGAAAGCTGGGTCTACAGCCGCAACACATCCAT
8_hypothetical	E16	Tb927.6.1660_for	GGGGACAAGTTTGTACAAAAAAGCAGGCTATGTCGAGCAATCGCATGTTTGC	Tb927.6.1660_rev	GGGGACCACTTTGTACAAGAAAGCTGGGTTCACCAAATCCATTTCTCTTCAGAGAGG
8_hypothetical	E17	Tb927.11.1700_for	GGGGACAAGTTTGTACAAAAAAGCAGGCTATGTGGCGCCGCATCTCC	Tb927.11.1700_rev	GGGGACCACTTTGTACAAGAAAGCTGGGTTTAGTTATGTTCATCATCATCATCGTCGT
8_hypothetical	E18	Tb927.11.16670_for	GGGGACAAGTTTGTACAAAAAAGCAGGCTATGAGTACGGGCGGGGTG	Tb927.11.16670_rev	GGGGACCACTTTGTACAAGAAAGCTGGGTTCATCCAACCGTCTGCCCA
8_hypothetical	E20	Tb927.11.3010_for	GGGGACAAGTTTGTACAAAAAAGCAGGCTATGCGCTCCGTGTTGAACG	Tb927.11.3010_rev	GGGGACCACTTTGTACAAGAAAGCTGGGTCTAGCGTACACATAACCAAGGCTG
8_hypothetical	E21	Tb927.3.5400_for	GGGGACAAGTTTGTACAAAAAAGCAGGCTATGCCCACCCACAGAGATCC	Tb927.3.5400_rev	GGGGACCACTTTGTACAAGAAAGCTGGGTCTATTTCTTTTTCTTCGCCTTTCCATTTTT
8_hypothetical	E22	Tb927.4.2370_for	GGGGACAAGTTTGTACAAAAAAGCAGGCTATGGCGAAAGGTAATGAGGAGTGT	Tb927.4.2370_rev	GGGGACCACTTTGTACAAGAAAGCTGGGTTTATCGGAGTACGGCCGACTC
8_hypothetical	E23	Tb927.6.2390_for	GGGGACAAGTTTGTACAAAAAAGCAGGCTATGTCCTCTGTGTCAAGTGATCACA	Tb927.6.2390_rev	GGGGACCACTTTGTACAAGAAAGCTGGGTTCACACGCCTCGAACAGTCA
8_hypothetical	E24	Tb927.11.11610_for	GGGGACAAGTTTGTACAAAAAAGCAGGCTATGTTTAAACGCACCCGAGGTG	Tb927.11.11610_rev	GGGGACCACTTTGTACAAGAAAGCTGGGTCTACCGTCGATAATTGCTGTTGGT
8_hypothetical	F1	Tb927.8.6220_for	GGGGACAAGTTTGTACAAAAAAGCAGGCTATGAAACGGGGTAGTGATGGATGT	Tb927.8.6220_rev	GGGGACCACTTTGTACAAGAAAGCTGGGTTCACCGTCCGGCCGCCGT
8_hypothetical	F2	Tb927.10.14900_for	GGGGACAAGTTTGTACAAAAAAGCAGGCTATGGAGTACCCAGCGGAGTTC	Tb927.10.14900_rev	GGGGACCACTTTGTACAAGAAAGCTGGGTTCACCCCTTGCGGCGAAT
8_hypothetical	F3	Tb927.11.8780_for	GGGGACAAGTTTGTACAAAAAAGCAGGCTATGGCGCTTTTATCACTGGACG	Tb927.11.8780_rev	GGGGACCACTTTGTACAAGAAAGCTGGGTTCATAACCGCAACACCAACCC
8_hypothetical	F4	Tb927.11.8060_for	GGGGACAAGTTTGTACAAAAAAGCAGGCTATGCAACCTATAGAACGTGCTGAG	Tb927.11.8060_rev	GGGGACCACTTTGTACAAGAAAGCTGGGTCTACAGCGTTTCCAGGCCAC
8_hypothetical	F5	Tb927.10.2720_for	GGGGACAAGTTTGTACAAAAAAGCAGGCTATGAGTTGCGCTGGATCCACA	Tb927.10.2720_rev	GGGGACCACTTTGTACAAGAAAGCTGGGTTTACGGCAACTCAATCTTACACGC
8_hypothetical	F6	Tb927.11.2570_for	GGGGACAAGTTTGTACAAAAAAGCAGGCTATGGTGGCGACAGGAACCT	Tb927.11.2570_rev	GGGGACCACTTTGTACAAGAAAGCTGGGTTTAGTAATCCTTCAAAACGTGTAGCATCA
8_hypothetical	F7	Tb927.5.1260_for	GGGGACAAGTTTGTACAAAAAAGCAGGCTATGGTGTCTTGTTTCGTGCGG	Tb927.5.1260_rev	GGGGACCACTTTGTACAAGAAAGCTGGGTTCAGTACAATTCTTGATACTCCAACTTGTG
8_hypothetical	F8	Tb927.11.10590_for	GGGGACAAGTTTGTACAAAAAAGCAGGCTATGACTTATCGTGTACTGGGCTCA	Tb927.11.10590_rev	GGGGACCACTTTGTACAAGAAAGCTGGGTTCAGACAACAATCGTCGAAGTGTT
8_hypothetical	F9	Tb927.11.15110_for	GGGGACAAGTTTGTACAAAAAAGCAGGCTATGATGCGTTTGGTTGCCTGC	Tb927.11.15110_rev	GGGGACCACTTTGTACAAGAAAGCTGGGTTTAATTAAGGGGGTTTAAATGTCGCATACA
8_hypothetical	F10	Tb927.10.10120_for	GGGGACAAGTTTGTACAAAAAAGCAGGCTATGCGTCGTTGCAAACTGTGG	Tb927.10.10120_rev	GGGGACCACTTTGTACAAGAAAGCTGGGTCTACATGGTGTCGCCCGAGA
8_hypothetical	F11	Tb927.7.4580_for	GGGGACAAGTTTGTACAAAAAAGCAGGCTATGCCGGACCCTCCGTTG	Tb927.7.4580_rev	GGGGACCACTTTGTACAAGAAAGCTGGGTCTAGTGCTGACGCCCTCTCA
8_hypothetical	F12	Tb927.11.5630_for	GGGGACAAGTTTGTACAAAAAAGCAGGCTATGACTGAGGTCTTCCGTGTGC	Tb927.11.5630_rev	GGGGACCACTTTGTACAAGAAAGCTGGGTCTACATAACTTTTGCGGCCAGCC
8_hypothetical	F13	Tb927.10.5400_for	GGGGACAAGTTTGTACAAAAAAGCAGGCTATGACACCCGGAGCAATGTACT	Tb927.10.5400_rev	GGGGACCACTTTGTACAAGAAAGCTGGGTCTACTGCACTTTTCTCTTATTGGCATTG
8_hypothetical	F14	Tb927.8.8110_for	GGGGACAAGTTTGTACAAAAAAGCAGGCTATGCGGACCAGAACAACAATGAC	Tb927.8.8110_rev	GGGGACCACTTTGTACAAGAAAGCTGGGTTCACTCTCGCAAATAGAAACTAAGCAC
8_hypothetical	F15	Tb927.10.2680_for	GGGGACAAGTTTGTACAAAAAAGCAGGCTATGTACCTTCCCAAACAACCGGT	Tb927.10.2680_rev	GGGGACCACTTTGTACAAGAAAGCTGGGTTTACAACATAGTTCCATCATCGCCAA
8_hypothetical	F16	Tb927.2.4970_for	GGGGACAAGTTTGTACAAAAAAGCAGGCTATGACGTGTTCGGTGGTTCCA	Tb927.2.4970_rev	GGGGACCACTTTGTACAAGAAAGCTGGGTTCACACTCCTTGATCATTTGGAGACG
8_hypothetical	F17	Tb927.5.3840_for	GGGGACAAGTTTGTACAAAAAAGCAGGCTATGCATGGAGCTTCAGTCGGA	Tb927.5.3840_rev	GGGGACCACTTTGTACAAGAAAGCTGGGTTTACAGTTCCCATATTTCTTCCTCTTCG
8_hypothetical	F18	Tb927.9.7580_for	GGGGACAAGTTTGTACAAAAAAGCAGGCTATGCCCTTTGATAATGTGACTGCTT	Tb927.9.7580_rev	GGGGACCACTTTGTACAAGAAAGCTGGGTTTACATAAGTATTCCAGATACATCGCTGGA
8_hypothetical	F19	Tb927.11.2370_for	GGGGACAAGTTTGTACAAAAAAGCAGGCTATGCCCAACCCATACAGGAAAAAC	Tb927.11.2370_rev	GGGGACCACTTTGTACAAGAAAGCTGGGTTCACTGCGGGACTGCCGC
8_hypothetical	F20	Tb927.2.2550_for	GGGGACAAGTTTGTACAAAAAAGCAGGCTATGTGTGGCCTTTTGCGCT	Tb927.2.2550_rev	GGGGACCACTTTGTACAAGAAAGCTGGGTTCACCAAAAAACGCGGAGCA
8_hypothetical	F21	Tb927.7.3770_for	GGGGACAAGTTTGTACAAAAAAGCAGGCTATGAGAAGGAACTCTTTTATTCGGACC	Tb927.7.3770_rev	GGGGACCACTTTGTACAAGAAAGCTGGGTCTAAAGGTAGCACTGAGAAGCTTGAA
8_hypothetical	F22	Tb927.7.5660_for	GGGGACAAGTTTGTACAAAAAAGCAGGCTATGCAAGCAAAGGGAGATGGG	Tb927.7.5660_rev	GGGGACCACTTTGTACAAGAAAGCTGGGTTTACTCTCCTCTAGCCGTTGCG
8_hypothetical	F23	Tb927.9.8060_for	GGGGACAAGTTTGTACAAAAAAGCAGGCTATGTACAAGGAGACCACTATGACACC	Tb927.9.8060_rev	GGGGACCACTTTGTACAAGAAAGCTGGGTTTACCAATCGTCATCATCGTCGGT
8_hypothetical	F24	Tb927.5.1250_for	GGGGACAAGTTTGTACAAAAAAGCAGGCTATGACCGAAAGCGTTGGAGC	Tb927.5.1250_rev	GGGGACCACTTTGTACAAGAAAGCTGGGTCTACGATGTGTTAATAGTGTTCCACCA
8_hypothetical	G1	Tb927.8.5130_for	GGGGACAAGTTTGTACAAAAAAGCAGGCTATGCAGCATCACTTCCACCTCA	Tb927.8.5130_rev	GGGGACCACTTTGTACAAGAAAGCTGGGTTCAGTAGGCTGAAACAACAAAAAAGAAC
8_hypothetical	G2	Tb927.5.3730_for	GGGGACAAGTTTGTACAAAAAAGCAGGCTATGGCGGCCCTTGTGGAA	Tb927.5.3730_rev	GGGGACCACTTTGTACAAGAAAGCTGGGTCTAATGGAGTCGGACATTCACTTCTTC
8_hypothetical	G3	Tb927.8.560_for	GGGGACAAGTTTGTACAAAAAAGCAGGCTATGGTACTGGCATCCATTCGTGT	Tb927.8.560_rev	GGGGACCACTTTGTACAAGAAAGCTGGGTCTACCGGCCTTTCTTCCGTG
8_hypothetical	G4	Tb927.4.1850_for	GGGGACAAGTTTGTACAAAAAAGCAGGCTATGGACTTTGATCTACTGTTGTTCCTAAAG	Tb927.4.1850_rev	GGGGACCACTTTGTACAAGAAAGCTGGGTTTACTTACGCCAACCCCAACTCC
8_hypothetical	G5	Tb927.11.6240_for	GGGGACAAGTTTGTACAAAAAAGCAGGCTATGCGTCCGAGGTTGTACCA	Tb927.11.6240_rev	GGGGACCACTTTGTACAAGAAAGCTGGGTTCAACAGCACCTTACTTCTTTTTTCCC
8_hypothetical	G6	Tb927.8.1470_for	GGGGACAAGTTTGTACAAAAAAGCAGGCTATGGAAAGGGGTATGTGCCTCG	Tb927.8.1470_rev	GGGGACCACTTTGTACAAGAAAGCTGGGTTCAAAGAATGGAAGCGACGCC
8_hypothetical	G7	Tb927.9.3580_for	GGGGACAAGTTTGTACAAAAAAGCAGGCTATGCATGTGGCCTCTCCCG	Tb927.9.3580_rev	GGGGACCACTTTGTACAAGAAAGCTGGGTCTAGTCAGAATCACTGTCACTGTCAC

8_hypothetical	G8	Tb927.6.610_for	GGGGACAAGTTTGTACAAAAAAGCAGGCTATGTTAATACGCCGCCTCCGC	Tb927.6.610_rev	GGGGACCACTTTGTACAAGAAAGCTGGGTTCAGAATGTGCCATCTGGATTGAC
8_hypothetical	G9	Tb927.11.9200_for	GGGGACAAGTTTGTACAAAAAAGCAGGCTATGGTGAAGTTGAAGGATGTTTCCC	Tb927.11.9200_rev	GGGGACCACTTTGTACAAGAAAGCTGGGTTTAATCGACATGAGTCCATAATTTAACAAA
8_hypothetical	G10	Tb927.3.5240_for	GGGGACAAGTTTGTACAAAAAAGCAGGCTATGCTTTCTCAGAATGTGGCCAA	Tb927.3.5240_rev	GGGGACCACTTTGTACAAGAAAGCTGGGTCTACCGTGACCCACCACCT
8_hypothetical	G11	Tb927.8.4670_for	GGGGACAAGTTTGTACAAAAAAGCAGGCTATGCCTCCTCGGAGGGCT	Tb927.8.4670_rev	GGGGACCACTTTGTACAAGAAAGCTGGGTTCAAAAGTTGTTGCTTAATGGGTTATCG
8_hypothetical	G12	Tb927.11.4000_for	GGGGACAAGTTTGTACAAAAAAGCAGGCTATGTTGGCGTCATGGGCCG	Tb927.11.4000_rev	GGGGACCACTTTGTACAAGAAAGCTGGGTCTAATATAGTTTCTTAGACAAAGTCCAGTG
8_hypothetical	G13	Tb927.10.4800_for	GGGGACAAGTTTGTACAAAAAAGCAGGCTATGGGTTCGTCGGGGTATGAC	Tb927.10.4800_rev	GGGGACCACTTTGTACAAGAAAGCTGGGTCTACGCAGAAGAACATTGACTTGTATCC
8_hypothetical	G14	Tb927.10.6250_for	GGGGACAAGTTTGTACAAAAAAGCAGGCTATGGCTCACAGTGGGGCC	Tb927.10.6250_rev	GGGGACCACTTTGTACAAGAAAGCTGGGTTCAGAAGAACACACTCCCCATACC
8_hypothetical	G15	Tb927.6.1980_for	GGGGACAAGTTTGTACAAAAAAGCAGGCTATGACTATTCCCGCCGTATACTCC	Tb927.6.1980_rev	GGGGACCACTTTGTACAAGAAAGCTGGGTTCACGACCGCCTCGCTCC
8_hypothetical	G16	Tb927.11.9820_for	GGGGACAAGTTTGTACAAAAAAGCAGGCTATGATCCGCGTGTATGTTGCTG	Tb927.11.9820_rev	GGGGACCACTTTGTACAAGAAAGCTGGGTTCATTTAAAGGGGTGAAAAACTGAATCGT
8_hypothetical	G17	Tb927.3.4080_for	GGGGACAAGTTTGTACAAAAAAGCAGGCTATGGTTTACCTTGTCGATGACCTTG	Tb927.3.4080_rev	GGGGACCACTTTGTACAAGAAAGCTGGGTTTAATTTTCTTTTTCTGAATCCTCTGTAAC
8_hypothetical	G18	Tb927.4.570_for	GGGGACAAGTTTGTACAAAAAAGCAGGCTATGTTCCAATTAACGACAGGATTCTGC	Tb927.4.570_rev	GGGGACCACTTTGTACAAGAAAGCTGGGTTTACAAACCTCCCTCACTCATTATTTGC
8_hypothetical	G19	Tb927.6.1560_for	GGGGACAAGTTTGTACAAAAAAGCAGGCTATGCCAGAGGAACTTAGGCAGT	Tb927.6.1560_rev	GGGGACCACTTTGTACAAGAAAGCTGGGTTCACACGGCTTGCTTTACTTTCT
8_hypothetical	G20	Tb927.11.7680_for	GGGGACAAGTTTGTACAAAAAAGCAGGCTATGCACAAGAGAGTCGGTTATACTGA	Tb927.11.7680_rev	GGGGACCACTTTGTACAAGAAAGCTGGGTTCAGAGATCATTCTGGGTTGATTGACC
8_hypothetical	G21	Tb927.8.3230_for	GGGGACAAGTTTGTACAAAAAAGCAGGCTATGAATCGAAAAAAAAGGTCCGTTGC	Tb927.8.3230_rev	GGGGACCACTTTGTACAAGAAAGCTGGGTTCAGCTTCCTCGTAACCTAAACGT
8_hypothetical	G22	Tb927.10.2600_for	GGGGACAAGTTTGTACAAAAAAGCAGGCTATGGTCGGTGAGGAGGTCG	Tb927.10.2600_rev	GGGGACCACTTTGTACAAGAAAGCTGGGTTCAAGTTTCTCGTGGAATTTTCTGACT
8_hypothetical	G23	Tb927.4.2910_for	GGGGACAAGTTTGTACAAAAAAGCAGGCTATGAGGCGTTGTGTTAGAGCAG	Tb927.4.2910_rev	GGGGACCACTTTGTACAAGAAAGCTGGGTTCACACTTCTCCTCCATCTGCA
8_hypothetical	G24	Tb927.10.8830_for	GGGGACAAGTTTGTACAAAAAAGCAGGCTATGGTATTCGACAAAGAGTTGAGGAATG	Tb927.10.8830_rev	GGGGACCACTTTGTACAAGAAAGCTGGGTCTAATTTCGTCGCACATTTTCAGAAGG
8_hypothetical	H1	Tb927.3.4100_for	GGGGACAAGTTTGTACAAAAAAGCAGGCTATGGTTTACCTTGTCGATGACCTTG	Tb927.3.4100_rev	GGGGACCACTTTGTACAAGAAAGCTGGGTTTATTGCTTCTTTCCTGAATGTCCACC
8_hypothetical	H2	Tb927.9.9010_for	GGGGACAAGTTTGTACAAAAAAGCAGGCTATGAAGACGAAGGACGGTAGTTGC	Tb927.9.9010_rev	GGGGACCACTTTGTACAAGAAAGCTGGGTCTAGGCGTCGCGGCGCGT
8_hypothetical	H3	Tb927.10.8850_for	GGGGACAAGTTTGTACAAAAAAGCAGGCTATGCCCGTCCATGCCCAT	Tb927.10.8850_rev	GGGGACCACTTTGTACAAGAAAGCTGGGTCTACTCATGAAGATCCGCCACAAA
8_hypothetical	H4	Tb927.9.10490_for	GGGGACAAGTTTGTACAAAAAAGCAGGCTATGACCGACACGCAAAAGGC	Tb927.9.10490_rev	GGGGACCACTTTGTACAAGAAAGCTGGGTCTAATCCAGAGCAAACAAATAGCGATTG
8_hypothetical	H5	Tb927.11.11700_for	GGGGACAAGTTTGTACAAAAAAGCAGGCTATGCAAAACCAGGCTCCAGTG	Tb927.11.11700_rev	GGGGACCACTTTGTACAAGAAAGCTGGGTTCAAACACGGATGACTGAATCTTTCTT
8_hypothetical	H6	Tb927.5.4340_for	GGGGACAAGTTTGTACAAAAAAGCAGGCTATGGTGACGCTGCGCTATTTAC	Tb927.5.4340_rev	GGGGACCACTTTGTACAAGAAAGCTGGGTCTATAACTCTGAGTGCAGTCGCAAC
8_hypothetical	H7	Tb927.5.680_for	GGGGACAAGTTTGTACAAAAAAGCAGGCTATGTGGCGAAGGCCACCG	Tb927.5.680_rev	GGGGACCACTTTGTACAAGAAAGCTGGGTCTACTGGCTGTAACGCATCCG
8_hypothetical	H8	Tb927.9.12850_for	GGGGACAAGTTTGTACAAAAAAGCAGGCTATGCACTGGCCGCACGGA	Tb927.9.12850_rev	GGGGACCACTTTGTACAAGAAAGCTGGGTTCACCTGTCGTAATTCTCCGGT
8_hypothetical	H9	Tb927.9.12830_for	GGGGACAAGTTTGTACAAAAAAGCAGGCTATGCAGAAAAATGCACCCAAGTTTT	Tb927.9.12830_rev	GGGGACCACTTTGTACAAGAAAGCTGGGTCTAACCAAAGGGGCCATGTATTTCC
8_hypothetical	H10	Tb927.4.2060_for	GGGGACAAGTTTGTACAAAAAAGCAGGCTATGGAGGCTTCTGTTGGAGTGG	Tb927.4.2060_rev	GGGGACCACTTTGTACAAGAAAGCTGGGTTTACGGAGCAGAAGGCAACCT
8_hypothetical	H11	Tb927.10.8800_for	GGGGACAAGTTTGTACAAAAAAGCAGGCTATGGCTTCGTTATCACCCCTGG	Tb927.10.8800_rev	GGGGACCACTTTGTACAAGAAAGCTGGGTTTAATCCGTGAGAAATGTGTTTACTTCAGG
8_hypothetical	H12	Tb927.5.3500_for	GGGGACAAGTTTGTACAAAAAAGCAGGCTATGGAGAGCGTGTCGCTTATCC	Tb927.5.3500_rev	GGGGACCACTTTGTACAAGAAAGCTGGGTCTAAACGCAGTCATGTAGGAAATAAACAC
8_hypothetical	H13	Tb927.8.1740_for	GGGGACAAGTTTGTACAAAAAAGCAGGCTATGCGCCGAGGTTTCTTTCTT	Tb927.8.1740_rev	GGGGACCACTTTGTACAAGAAAGCTGGGTCTAGTGTGTGGGAGTCTTCGCC
8_hypothetical	H14	Tb927.10.13090_for	GGGGACAAGTTTGTACAAAAAAGCAGGCTATGTTAAGGCGAGGCGTTTACAG	Tb927.10.13090_rev	GGGGACCACTTTGTACAAGAAAGCTGGGTTTACGCCTTCTTTGCCTTTCTGT
8_hypothetical	H15	Tb927.2.3730_for	GGGGACAAGTTTGTACAAAAAAGCAGGCTATGAGTTACACTGAAGGAGATGGGAA	Tb927.2.3730_rev	GGGGACCACTTTGTACAAGAAAGCTGGGTTCATCCGTCTGTTTTGACACTATTCG
8_hypothetical	H16	Tb927.10.2240_for	GGGGACAAGTTTGTACAAAAAAGCAGGCTATGTCCATTACACAAAGTGCACTCG	Tb927.10.2240_rev	GGGGACCACTTTGTACAAGAAAGCTGGGTCTAGGGATAGAACCGTGCCACC
8_hypothetical	H17	Tb927.7.3190_for	GGGGACAAGTTTGTACAAAAAAGCAGGCTATGCAGTGGAAAGAGGTATTAGATAGCG	Tb927.7.3190_rev	GGGGACCACTTTGTACAAGAAAGCTGGGTTCAATCATCTTCCAAAGAGTTTCCGTC
8_hypothetical	H18	Tb927.11.12170_for	GGGGACAAGTTTGTACAAAAAAGCAGGCTATGTCCGTGGCCTCTGAGG	Tb927.11.12170_rev	GGGGACCACTTTGTACAAGAAAGCTGGGTTCACGAACCCAACTGTGACGA
8_hypothetical	H19	Tb927.3.5220_for	GGGGACAAGTTTGTACAAAAAAGCAGGCTATGTCCTCCGCTAATTCCATCCA	Tb927.3.5220_rev	GGGGACCACTTTGTACAAGAAAGCTGGGTTTAAAGCACCGGCACTGCG
8_hypothetical	H20	Tb927.11.7870_for	GGGGACAAGTTTGTACAAAAAAGCAGGCTATGACCAAATGGAGGTGGAAAACAC	Tb927.11.7870_rev	GGGGACCACTTTGTACAAGAAAGCTGGGTCTAAACACCAATGTCACAGGCAGT
8_hypothetical	H21	Tb927.4.4370_for	GGGGACAAGTTTGTACAAAAAAGCAGGCTATGCTCATTTGCCCCACCG	Tb927.4.4370_rev	GGGGACCACTTTGTACAAGAAAGCTGGGTTCACAACTCCGCACCCTCA
8_hypothetical	H22	Tb927.9.11520_for	GGGGACAAGTTTGTACAAAAAAGCAGGCTATGGATGCTGATCTTAACGGTTTGC	Tb927.9.11520_rev	GGGGACCACTTTGTACAAGAAAGCTGGGTTCAATCGACCAACTCGCCACC
8_hypothetical	H23	Tb927.8.3520_for	GGGGACAAGTTTGTACAAAAAAGCAGGCTATGAACTTCTCTGGTGGAAACAAGG	Tb927.8.3520_rev	GGGGACCACTTTGTACAAGAAAGCTGGGTTTACTTCGATTCGGGTTGATTTTGAAATAC
8_hypothetical	H24	Tb927.10.9930_for	GGGGACAAGTTTGTACAAAAAAGCAGGCTATGCGTATTGTAATCGATGTCGATGC	Tb927.10.9930_rev	GGGGACCACTTTGTACAAGAAAGCTGGGTCTAATTGTCGTCGGCGATACCAC
8_hypothetical	I1	Tb927.3.1910_for	GGGGACAAGTTTGTACAAAAAAGCAGGCTATGTCCATGACTGAGAGCAGCA	Tb927.3.1910_rev	GGGGACCACTTTGTACAAGAAAGCTGGGTCTACTGACGCTGCTGGCG
8_hypothetical	I2	Tb927.11.16580_for	GGGGACAAGTTTGTACAAAAAAGCAGGCTATGCTCAACCCGGGTGATCA	Tb927.11.16580_rev	GGGGACCACTTTGTACAAGAAAGCTGGGTTCACAGCACCACGTACAAAAACC
8_hypothetical	I3	Tb927.9.12160_for	GGGGACAAGTTTGTACAAAAAAGCAGGCTATGCGCCGCTTTTGTGGG	Tb927.9.12160_rev	GGGGACCACTTTGTACAAGAAAGCTGGGTTCAGTTGCCCCCCCTCTCC
8_hypothetical	I4	Tb927.10.10300_for	GGGGACAAGTTTGTACAAAAAAGCAGGCTATGTCATGGATGAGCGCGC	Tb927.10.10300_rev	GGGGACCACTTTGTACAAGAAAGCTGGGTTTAAGCCGGAAGAAAGTGTATGGC
8_hypothetical	I5	Tb927.7.3340_for	GGGGACAAGTTTGTACAAAAAAGCAGGCTATGAGGTACTTCCCACTCGGTG	Tb927.7.3340_rev	GGGGACCACTTTGTACAAGAAAGCTGGGTTCACATCTCGGATGGATGGTGC
8_hypothetical	I6	Tb927.6.730_for	GGGGACAAGTTTGTACAAAAAAGCAGGCTATGAATGCTGCATCCCCTCCT	Tb927.6.730_rev	GGGGACCACTTTGTACAAGAAAGCTGGGTTCAAACCATTGGAAGCTCCAGGA
8_hypothetical	I7	Tb927.3.2210_for	GGGGACAAGTTTGTACAAAAAAGCAGGCTATGGACCACACTGCGTTCAC	Tb927.3.2210_rev	GGGGACCACTTTGTACAAGAAAGCTGGGTTCAAGCAGTACGGTTGTACACAC
8_hypothetical	I8	Tb927.8.6710_for	GGGGACAAGTTTGTACAAAAAAGCAGGCTATGTCCTCCGTTACCACTGGC	Tb927.8.6710_rev	GGGGACCACTTTGTACAAGAAAGCTGGGTTCATTCTCGAACGTATTCCTTATTCTCCC
8_hypothetical	I9	Tb927.8.6730_for	GGGGACAAGTTTGTACAAAAAAGCAGGCTATGTCCTCCGTTACCACTGGC	Tb927.8.6730_rev	GGGGACCACTTTGTACAAGAAAGCTGGGTTTATTTCCTTGCAGGGCTTTTGTCA
8_hypothetical	I10	Tb927.8.5080_for	GGGGACAAGTTTGTACAAAAAAGCAGGCTATGCTTACTCTGGCTATCATATGTGTCA	Tb927.8.5080_rev	GGGGACCACTTTGTACAAGAAAGCTGGGTTTACTCGAGAATAGCCCAGCCAG
8_hypothetical	I11	Tb927.3.4070_for	GGGGACAAGTTTGTACAAAAAAGCAGGCTATGGTTTACCTTGTCGATGACCTTG	Tb927.3.4070_rev	GGGGACCACTTTGTACAAGAAAGCTGGGTCTACTGCTGCGCTGCTCC
8_hypothetical	I12	Tb927.10.3810_for	GGGGACAAGTTTGTACAAAAAAGCAGGCTATGTGGAAGCAAGATATTGGGCAG	Tb927.10.3810_rev	GGGGACCACTTTGTACAAGAAAGCTGGGTTCAGAGTGTTTTTGCCCTGTATTTGT
8_hypothetical	I13	Tb927.3.1850_for	GGGGACAAGTTTGTACAAAAAAGCAGGCTATGAGGGGGAACTTAATTACCGGG	Tb927.3.1850_rev	GGGGACCACTTTGTACAAGAAAGCTGGGTCTAAGAACTCACATCAAGATTAAAGAACGG
8_hypothetical	I14	Tb927.11.5210_for	GGGGACAAGTTTGTACAAAAAAGCAGGCTATGGCCGAACTACGGGAAGG	Tb927.11.5210_rev	GGGGACCACTTTGTACAAGAAAGCTGGGTTTAGTCGGCATTGGCTGCAG
8_hypothetical	I15	Tb927.10.12600_for	GGGGACAAGTTTGTACAAAAAAGCAGGCTATGTCCGTATATGGGAACGGCG	Tb927.10.12600_rev	GGGGACCACTTTGTACAAGAAAGCTGGGTCTACGCCACATTATTCCAGCAACT
8_hypothetical	I16	Tb927.7.900_for	GGGGACAAGTTTGTACAAAAAAGCAGGCTATGTTTTTGTCGTTGGCACTACTGG	Tb927.7.900_rev	GGGGACCACTTTGTACAAGAAAGCTGGGTTCAACTGCTTCCACTGCTAGCA
8_hypothetical	I17	Tb927.3.4110_for	GGGGACAAGTTTGTACAAAAAAGCAGGCTATGACGTATATTATCGATGACCTTGCG	Tb927.3.4110_rev	GGGGACCACTTTGTACAAGAAAGCTGGGTCTACTGCTGCGCTGCTCC
8_hypothetical	I19	Tb927.5.830_for	GGGGACAAGTTTGTACAAAAAAGCAGGCTATGCTGTACAAAGACCTCCTGCA	Tb927.5.830_rev	GGGGACCACTTTGTACAAGAAAGCTGGGTCTACTCACCCCTCAATCGGACC
8_hypothetical	I20	Tb927.11.14550_for	GGGGACAAGTTTGTACAAAAAAGCAGGCTATGGAAAATTTGGTGGACGTTGGA	Tb927.11.14550_rev	GGGGACCACTTTGTACAAGAAAGCTGGGTCTATGCGTGGGGACACCTTAAAAT
8_hypothetical	I21	Tb927.6.580_for	GGGGACAAGTTTGTACAAAAAAGCAGGCTATGATAAAACTCGTGTCGGGTGTC	Tb927.6.580_rev	GGGGACCACTTTGTACAAGAAAGCTGGGTTCAAAGCACATCTTCACTCAGCAA
8_hypothetical	I22	Tb927.11.9680_for	GGGGACAAGTTTGTACAAAAAAGCAGGCTATGGTGACGCTGGGGAAGAA	Tb927.11.9680_rev	GGGGACCACTTTGTACAAGAAAGCTGGGTTTATTGAGAAAACCCATACTTGCTCATAAT
8_hypothetical	I23	Tb927.10.9070_for	GGGGACAAGTTTGTACAAAAAAGCAGGCTATGACCGCGAGGTTCACTGC	Tb927.10.9070_rev	GGGGACCACTTTGTACAAGAAAGCTGGGTCTATTGAGAGACAATGTAGTCAAGAAGGTT
8_hypothetical	I24	Tb927.7.3370_for	GGGGACAAGTTTGTACAAAAAAGCAGGCTATGCAGGGGGGATTTGGTCG	Tb927.7.3370_rev	GGGGACCACTTTGTACAAGAAAGCTGGGTTCAGCGGTCACCAACCATCA
8_hypothetical	J1	Tb927.2.4460_for	GGGGACAAGTTTGTACAAAAAAGCAGGCTATGGCGCTACAGCGACGC	Tb927.2.4460_rev	GGGGACCACTTTGTACAAGAAAGCTGGGTTTACATGATGGAGTAAAGTTCTTCAGCAG
8_hypothetical	J2	Tb927.3.4660_for	GGGGACAAGTTTGTACAAAAAAGCAGGCTATGTCAGTTCATCAACTGCAACTTGA	Tb927.3.4660_rev	GGGGACCACTTTGTACAAGAAAGCTGGGTCTAGTTGTTTGACTTCCATAACCGTATGC
8_hypothetical	J3	Tb927.10.6520_for	GGGGACAAGTTTGTACAAAAAAGCAGGCTATGGACGGTGGCTTCCCTT	Tb927.10.6520_rev	GGGGACCACTTTGTACAAGAAAGCTGGGTTTAGCGCAGAGGCATGGCC
8_hypothetical	J4	Tb927.11.10180_for	GGGGACAAGTTTGTACAAAAAAGCAGGCTATGCACCTACTCGGTGATATTGTAGC	Tb927.11.10180_rev	GGGGACCACTTTGTACAAGAAAGCTGGGTCTACAGTGTAACCGAGACATTTTCGT
8_hypothetical	J5	Tb927.4.2590_for	GGGGACAAGTTTGTACAAAAAAGCAGGCTATGAGGGCGGAGCCGAAT	Tb927.4.2590_rev	GGGGACCACTTTGTACAAGAAAGCTGGGTTCATAAGCTTTCCCTGCCGCG
8_hypothetical	J6	Tb927.10.11600_for	GGGGACAAGTTTGTACAAAAAAGCAGGCTATGGAGTATTCGTCAGATGCAGTGT	Tb927.10.11600_rev	GGGGACCACTTTGTACAAGAAAGCTGGGTCTATTGATAACGGTGATGGGTAAGAGC
8_hypothetical	J7	Tb927.10.13450_for	GGGGACAAGTTTGTACAAAAAAGCAGGCTATGCTCGACGAGAATCAACGAG	Tb927.10.13450_rev	GGGGACCACTTTGTACAAGAAAGCTGGGTTCACTGCTCGTCTTCACTGTCC
8_hypothetical	J8	Tb927.1.4370_for	GGGGACAAGTTTGTACAAAAAAGCAGGCTATGGATACTCTCTTTTATTTTCCTGTTGGA	Tb927.1.4370_rev	GGGGACCACTTTGTACAAGAAAGCTGGGTCTAATGATCACCCGCCTCCTGA
8_hypothetical	J9	Tb927.3.1010_for	GGGGACAAGTTTGTACAAAAAAGCAGGCTATGTTGGTCGAGGAACAGACTGG	Tb927.3.1010_rev	GGGGACCACTTTGTACAAGAAAGCTGGGTTTAAGCAGTCGATTCCTTCTCTGAAGC
8_hypothetical	J10	Tb927.7.2410_for	GGGGACAAGTTTGTACAAAAAAGCAGGCTATGAGGCAGTTAGGAAGTGCAGT	Tb927.7.2410_rev	GGGGACCACTTTGTACAAGAAAGCTGGGTTTATTTGACCCTCACCTCATTACCCA
8_hypothetical	J11	Tb927.9.1700_for	GGGGACAAGTTTGTACAAAAAAGCAGGCTATGGAACTACTTGAGACGGTGGA	Tb927.9.1700_rev	GGGGACCACTTTGTACAAGAAAGCTGGGTTTACAATTGGGTTTGTCCAATTGATTGG
8_hypothetical	J12	Tb927.10.5600_for	GGGGACAAGTTTGTACAAAAAAGCAGGCTATGCATCACACGGGGATGC	Tb927.10.5600_rev	GGGGACCACTTTGTACAAGAAAGCTGGGTCTAAGCGCCCCGCTGCAA
8_hypothetical	J13	Tb927.4.3470_for	GGGGACAAGTTTGTACAAAAAAGCAGGCTATGTCTCTTCGTGACGATATCTTGGC	Tb927.4.3470_rev	GGGGACCACTTTGTACAAGAAAGCTGGGTCTATGCACTCCCATGCATGCTC
8_hypothetical	J14	Tb927.8.1500_for	GGGGACAAGTTTGTACAAAAAAGCAGGCTATGGCAATGCAGTTATTTACCTTTGGA	Tb927.8.1500_rev	GGGGACCACTTTGTACAAGAAAGCTGGGTTCAGTCATCGCAGATTCCCTTGA
8_hypothetical	J15	Tb927.10.10020_for	GGGGACAAGTTTGTACAAAAAAGCAGGCTATGAATTCTGCTGCGAGTGAAGAG	Tb927.10.10020_rev	GGGGACCACTTTGTACAAGAAAGCTGGGTTTACGACCGGGCGTTCCG
8_hypothetical	J16	Tb927.9.10150_for	GGGGACAAGTTTGTACAAAAAAGCAGGCTATGGATGCGCCATCGCTG	Tb927.9.10150_rev	GGGGACCACTTTGTACAAGAAAGCTGGGTTCAGCACATACTTGGGGTACTATCC
8_hypothetical	J17	Tb927.9.11120_for	GGGGACAAGTTTGTACAAAAAAGCAGGCTATGCTTCGACGGGCTTACATC	Tb927.9.11120_rev	GGGGACCACTTTGTACAAGAAAGCTGGGTTCACAACTCGGGAATCGCCT
8_hypothetical	J18	Tb927.11.16050_for	GGGGACAAGTTTGTACAAAAAAGCAGGCTATGCCAGTCAAACCTGTATACGAGT	Tb927.11.16050_rev	GGGGACCACTTTGTACAAGAAAGCTGGGTTCAAGTGGCAATCGCTGGTG
8_hypothetical	J19	Tb927.9.2030_for	GGGGACAAGTTTGTACAAAAAAGCAGGCTATGATGGAAAATACCCCGCTATTGG	Tb927.9.2030_rev	GGGGACCACTTTGTACAAGAAAGCTGGGTTCACAGGGATAAAATGCCCTTGAGT
8_hypothetical	J20	Tb927.6.1890_for	GGGGACAAGTTTGTACAAAAAAGCAGGCTATGCGGCGGTGCTTATGG	Tb927.6.1890_rev	GGGGACCACTTTGTACAAGAAAGCTGGGTTTATCGACGACGATGGTGCGC
8_hypothetical	J21	Tb927.11.14990_for	GGGGACAAGTTTGTACAAAAAAGCAGGCTATGGGCGGCGGTACTAGAG	Tb927.11.14990_rev	GGGGACCACTTTGTACAAGAAAGCTGGGTTCAAAATAATTCGTCCAGTATTGAGTCCC
8_hypothetical	J22	Tb927.10.1330_for	GGGGACAAGTTTGTACAAAAAAGCAGGCTATGAACTTGTCCAATCGGGTTGTG	Tb927.10.1330_rev	GGGGACCACTTTGTACAAGAAAGCTGGGTCTAGTTTGTCGGTTCCGCCTG
8_hypothetical	J23	Tb927.10.810_for	GGGGACAAGTTTGTACAAAAAAGCAGGCTATGGGGGCGGAAAAAAAAATAAATCG	Tb927.10.810_rev	GGGGACCACTTTGTACAAGAAAGCTGGGTTTAATTATCACGCTTCTTTTTCGCATCAT
8_hypothetical	J24	Tb927.6.660_for	GGGGACAAGTTTGTACAAAAAAGCAGGCTATGGATCTCCATTCCCCCTCCG	Tb927.6.660_rev	GGGGACCACTTTGTACAAGAAAGCTGGGTTTAAGTGCGGACCCAACCTCT
8_hypothetical	K1	Tb927.6.4450_for	GGGGACAAGTTTGTACAAAAAAGCAGGCTATGATTCGGCGCCTCAATGTG	Tb927.6.4450_rev	GGGGACCACTTTGTACAAGAAAGCTGGGTCTAACCTGTAGAACCGCTTGTTGAT
8_hypothetical	K2	Tb927.4.1490_for	GGGGACAAGTTTGTACAAAAAAGCAGGCTATGTCAGACGGCGGTATCTCC	Tb927.4.1490_rev	GGGGACCACTTTGTACAAGAAAGCTGGGTTCACGCGTGTTTCCATCTGC
8_hypothetical	K3	Tb927.8.3020_for	GGGGACAAGTTTGTACAAAAAAGCAGGCTATGGAGACGAAGCGTAGTTTCGA	Tb927.8.3020_rev	GGGGACCACTTTGTACAAGAAAGCTGGGTTCAACCACGGTGGCGCCC
8_hypothetical	K5	Tb927.10.9330_for	GGGGACAAGTTTGTACAAAAAAGCAGGCTATGGAACACGAAATTCATAAGGGAATGG	Tb927.10.9330_rev	GGGGACCACTTTGTACAAGAAAGCTGGGTTTACATTCTTCCCGGTACATTTTGAGG
8_hypothetical	K6	Tb927.8.6640_for	GGGGACAAGTTTGTACAAAAAAGCAGGCTATGCCACTCGACGTCCATGA	Tb927.8.6640_rev	GGGGACCACTTTGTACAAGAAAGCTGGGTTCAAAGGGACGAGGCCCTC
8_hypothetical	K7	Tb927.9.8350_for	GGGGACAAGTTTGTACAAAAAAGCAGGCTATGGGGGCTGGAGCGTCG	Tb927.9.8350_rev	GGGGACCACTTTGTACAAGAAAGCTGGGTCTACTCGGGTTTCCCCGCT
8_hypothetical	K8	Tb927.6.2580_for	GGGGACAAGTTTGTACAAAAAAGCAGGCTATGCTGTTCTGGTGTCGTCATG	Tb927.6.2580_rev	GGGGACCACTTTGTACAAGAAAGCTGGGTTTATGCACCACTACCAGTATAGTAACATCC
8_hypothetical	K9	Tb927.11.6670_for	GGGGACAAGTTTGTACAAAAAAGCAGGCTATGACACAAAAAAATGTGCTGTGTCA	Tb927.11.6670_rev	GGGGACCACTTTGTACAAGAAAGCTGGGTCTACAGACCATCTGCCGTCATG
8_hypothetical	K10	Tb927.4.3400_for	GGGGACAAGTTTGTACAAAAAAGCAGGCTATGGATATTCGGGAGATTATTGATCTTGTG	Tb927.4.3400_rev	GGGGACCACTTTGTACAAGAAAGCTGGGTCTATCGGCGAGGGCGCTT
8_hypothetical	K11	Tb927.11.10490_for	GGGGACAAGTTTGTACAAAAAAGCAGGCTATGTTTAGTGAGTCTTTGTACTCGTTTCA	Tb927.11.10490_rev	GGGGACCACTTTGTACAAGAAAGCTGGGTTCAAAGTTTCCATTTGTAGTACTCAAGCT
8_hypothetical	K12	Tb927.11.14640_for	GGGGACAAGTTTGTACAAAAAAGCAGGCTATGCCGTTGGGCTTGGATG	Tb927.11.14640_rev	GGGGACCACTTTGTACAAGAAAGCTGGGTTTAGGGAGTCCAAAAAACATTACCGG
8_hypothetical	K13	Tb927.7.2320_for	GGGGACAAGTTTGTACAAAAAAGCAGGCTATGAGCATCGTCAATCAGCTCAC	Tb927.7.2320_rev	GGGGACCACTTTGTACAAGAAAGCTGGGTTCACTTGACACGACTGCAACC
8_hypothetical	K14	Tb927.8.6270_for	GGGGACAAGTTTGTACAAAAAAGCAGGCTATGCGGGGAATATCAGCTCTGT	Tb927.8.6270_rev	GGGGACCACTTTGTACAAGAAAGCTGGGTCTACGCATCCGTCATGACGG
8_hypothetical	K15	Tb927.7.1770_for	GGGGACAAGTTTGTACAAAAAAGCAGGCTATGGATCGTATATACAGCCAGCCG	Tb927.7.1770_rev	GGGGACCACTTTGTACAAGAAAGCTGGGTCTATGTTTCATCATAACAACGTGCTACAC
8_hypothetical	K16	Tb927.10.15330_for	GGGGACAAGTTTGTACAAAAAAGCAGGCTATGACACGCATGCGAGTGG	Tb927.10.15330_rev	GGGGACCACTTTGTACAAGAAAGCTGGGTTTACGCTGCAGCCAAGGAAG
8_hypothetical	K17	Tb927.10.980_for	GGGGACAAGTTTGTACAAAAAAGCAGGCTATGGCCGCCGTGGAGCCA	Tb927.10.980_rev	GGGGACCACTTTGTACAAGAAAGCTGGGTTCATCCGTCATCTGGATGCTGG
8_hypothetical	K18	Tb927.9.2650_for	GGGGACAAGTTTGTACAAAAAAGCAGGCTATGACTAGCCAGCTGAATGCG	Tb927.9.2650_rev	GGGGACCACTTTGTACAAGAAAGCTGGGTTCACTTAGTCTTTCTCCTCCGCC
8_hypothetical	K19	Tb927.9.13130_for	GGGGACAAGTTTGTACAAAAAAGCAGGCTATGACGAAAGATAAAGTTAACTTTAGGTAC	Tb927.9.13130_rev	GGGGACCACTTTGTACAAGAAAGCTGGGTTTACTTCAGAAGCCTGAATATCGCTC
8_hypothetical	K20	Tb927.11.16620_for	GGGGACAAGTTTGTACAAAAAAGCAGGCTATGCTCACGCGGAAGCTG	Tb927.11.16620_rev	GGGGACCACTTTGTACAAGAAAGCTGGGTTTAAATTTCCACCGCAGCATAACCA
8_hypothetical	K21	Tb927.8.5680_for	GGGGACAAGTTTGTACAAAAAAGCAGGCTATGTTACGGCATACAGTGAAATTGTGC	Tb927.8.5680_rev	GGGGACCACTTTGTACAAGAAAGCTGGGTCTAAACGACACGGCCCTTATTTCT
8_hypothetical	K22	Tb927.10.13890_for	GGGGACAAGTTTGTACAAAAAAGCAGGCTATGCGTCCACGAATGGGTTC	Tb927.10.13890_rev	GGGGACCACTTTGTACAAGAAAGCTGGGTCTAAGTGTGTTGCCTTATGGGTGAT
8_hypothetical	K23	Tb927.11.6980_for	GGGGACAAGTTTGTACAAAAAAGCAGGCTATGCTTCGTAGAGTCCTCTCCG	Tb927.11.6980_rev	GGGGACCACTTTGTACAAGAAAGCTGGGTTCACACTGAAGCGTGCGC
8_hypothetical	K24	Tb927.8.3480_for	GGGGACAAGTTTGTACAAAAAAGCAGGCTATGACAGAAGGGATGGACCGC	Tb927.8.3480_rev	GGGGACCACTTTGTACAAGAAAGCTGGGTTTATTTTTGTACACTGCCCCCAGC
8_hypothetical	L1	Tb927.3.4430_for	GGGGACAAGTTTGTACAAAAAAGCAGGCTATGCAACGGTTAGTTAGTGCACTC	Tb927.3.4430_rev	GGGGACCACTTTGTACAAGAAAGCTGGGTCTAGCGCTGAACACGTTCCC
8_hypothetical	L2	Tb927.7.5690_for	GGGGACAAGTTTGTACAAAAAAGCAGGCTATGCCGACAACGGCGGAT	Tb927.7.5690_rev	GGGGACCACTTTGTACAAGAAAGCTGGGTTCAGGAGGAAAACTGTATAACGCCT
8_hypothetical	L3	Tb927.5.3590_for	GGGGACAAGTTTGTACAAAAAAGCAGGCTATGCCACCGAACCGTTGGG	Tb927.5.3590_rev	GGGGACCACTTTGTACAAGAAAGCTGGGTTTAGTTCACCTCCATAAGTTGCTTCC
8_hypothetical	L4	Tb927.7.730_for	GGGGACAAGTTTGTACAAAAAAGCAGGCTATGACGGAAAATTCACTCTCCACG	Tb927.7.730_rev	GGGGACCACTTTGTACAAGAAAGCTGGGTTCAGTTTCTCGGTGGGATAAATATATCACC
8_hypothetical	L5	Tb927.7.3100_for	GGGGACAAGTTTGTACAAAAAAGCAGGCTATGACGGGGCCAACGCGT	Tb927.7.3100_rev	GGGGACCACTTTGTACAAGAAAGCTGGGTTTAGCTGCTACGCCACTTATGTTC
8_hypothetical	L6	Tb927.3.3230_for	GGGGACAAGTTTGTACAAAAAAGCAGGCTATGAGCCGAAATCACAACAGAAGG	Tb927.3.3230_rev	GGGGACCACTTTGTACAAGAAAGCTGGGTTCAACTCTCTTCCGCGCAAC
8_hypothetical	L7	Tb927.1.3840_for	GGGGACAAGTTTGTACAAAAAAGCAGGCTATGGCTCACGCCACCAGT	Tb927.1.3840_rev	GGGGACCACTTTGTACAAGAAAGCTGGGTCTATGGTGACGCTATACCTGCTGA
8_hypothetical	L8	Tb927.11.6570_for	GGGGACAAGTTTGTACAAAAAAGCAGGCTATGAGTGTCACTGAAGGAGATGAACG	Tb927.11.6570_rev	GGGGACCACTTTGTACAAGAAAGCTGGGTTCACTCAGTCCCCCTGCG
8_hypothetical	L9	Tb927.11.7580_for	GGGGACAAGTTTGTACAAAAAAGCAGGCTATGAGAAGGCCTCTGTCGTTTATTG	Tb927.11.7580_rev	GGGGACCACTTTGTACAAGAAAGCTGGGTTTAAATTGAAAAGGCAACAGCTTCCTC
8_hypothetical	L10	Tb927.11.16120_for	GGGGACAAGTTTGTACAAAAAAGCAGGCTATGCGTGGTGTATTTTACCTTACCG	Tb927.11.16120_rev	GGGGACCACTTTGTACAAGAAAGCTGGGTTTAGAGTGTGGTGCCCTGGG
8_hypothetical	L11	Tb927.5.1900_for	GGGGACAAGTTTGTACAAAAAAGCAGGCTATGCACGAAGAGGACCCCG	Tb927.5.1900_rev	GGGGACCACTTTGTACAAGAAAGCTGGGTTCATATGGTTTCATCCTTCACTCGC
8_hypothetical	L12	Tb927.5.1950_for	GGGGACAAGTTTGTACAAAAAAGCAGGCTATGGAGGAAGTGCAACCGCT	Tb927.5.1950_rev	GGGGACCACTTTGTACAAGAAAGCTGGGTTTAGCCGATTTCACTCATAGCAATATCTAA
8_hypothetical	L13	Tb927.11.5130_for	GGGGACAAGTTTGTACAAAAAAGCAGGCTATGGTGGAACTTACAGTGTACGAAGG	Tb927.11.5130_rev	GGGGACCACTTTGTACAAGAAAGCTGGGTTCACGACAATGACCACATTTCTTTTTG
8_hypothetical	L14	Tb927.10.6210_for	GGGGACAAGTTTGTACAAAAAAGCAGGCTATGTCGGTGTCGCCTGAACC	Tb927.10.6210_rev	GGGGACCACTTTGTACAAGAAAGCTGGGTTTAGGTACATGTCGTGGAGTGGC
8_hypothetical	L15	Tb927.11.7200_for	GGGGACAAGTTTGTACAAAAAAGCAGGCTATGGCCGCTGAGGATCAAGG	Tb927.11.7200_rev	GGGGACCACTTTGTACAAGAAAGCTGGGTTTACTTCAATGGGGGACACTGTGG
8_hypothetical	L16	Tb927.10.2030_for	GGGGACAAGTTTGTACAAAAAAGCAGGCTATGCGGAGAGCCTTGTGGC	Tb927.10.2030_rev	GGGGACCACTTTGTACAAGAAAGCTGGGTTCAAATGACGATGTCATTTCGGGC
8_hypothetical	L17	Tb927.10.6320_for	GGGGACAAGTTTGTACAAAAAAGCAGGCTATGGCAGCCATGCAGCGC	Tb927.10.6320_rev	GGGGACCACTTTGTACAAGAAAGCTGGGTCTACTCACCAGCGGGATCTGA
8_hypothetical	L18	Tb927.10.1120_for	GGGGACAAGTTTGTACAAAAAAGCAGGCTATGCTTTTTGCCCAGCGCA	Tb927.10.1120_rev	GGGGACCACTTTGTACAAGAAAGCTGGGTCTATGCATTGTTACCGGCGAGT
8_hypothetical	L19	Tb927.9.1410_for	GGGGACAAGTTTGTACAAAAAAGCAGGCTATGTCCAGCAATAGCAGTGACG	Tb927.9.1410_rev	GGGGACCACTTTGTACAAGAAAGCTGGGTTCACTCCTCCCATAATCCACCAC

8_hypothetical	L20	Tb927.11.780_for	GGGGACAAGTTTGTACAAAAAAGCAGGCTATGGGGTTTAGCGACGGAGC	Tb927.11.780_rev	GGGGACCACTTTGTACAAGAAAGCTGGGTCTATGATTTTAATGTGTGACGATACCGC
8_hypothetical	L21	Tb927.11.1200_for	GGGGACAAGTTTGTACAAAAAAGCAGGCTATGGGAGACAGTGTGATACCCC	Tb927.11.1200_rev	GGGGACCACTTTGTACAAGAAAGCTGGGTTCACGCCTCTGCTTCCCA
8_hypothetical	L22	Tb927.4.1410_for	GGGGACAAGTTTGTACAAAAAAGCAGGCTATGTTTTCCTTCCTTACTTCGGGAGG	Tb927.4.1410_rev	GGGGACCACTTTGTACAAGAAAGCTGGGTTCATTCGCGCCCCCGCTG
8_hypothetical	L23	Tb927.9.2821_for	GGGGACAAGTTTGTACAAAAAAGCAGGCTATGGAAATCCGCATGGTGCC	Tb927.9.2821_rev	GGGGACCACTTTGTACAAGAAAGCTGGGTTCACTCATCAGCTGCTGGAGT
8_hypothetical	L24	Tb927.8.6720_for	GGGGACAAGTTTGTACAAAAAAGCAGGCTATGTCCTCCGTTACCACTGGC	Tb927.8.6720_rev	GGGGACCACTTTGTACAAGAAAGCTGGGTTTACTGTTCTTCTTTCTTCCCATACCCT
8_hypothetical	M1	Tb927.11.16610_for	GGGGACAAGTTTGTACAAAAAAGCAGGCTATGAGCTCTCAGCACCGCG	Tb927.11.16610_rev	GGGGACCACTTTGTACAAGAAAGCTGGGTTTACGCATCCACTACGAGCTTCG
8_hypothetical	M2	Tb927.11.5990_for	GGGGACAAGTTTGTACAAAAAAGCAGGCTATGAAGGTAAGTTCAAGGGGCCC	Tb927.11.5990_rev	GGGGACCACTTTGTACAAGAAAGCTGGGTCTACAATAAGCCATAGAGGTCGTGAAAA
8_hypothetical	M3	Tb927.8.1560_for	GGGGACAAGTTTGTACAAAAAAGCAGGCTATGGAGTTTGAGCAGCTGGAGA	Tb927.8.1560_rev	GGGGACCACTTTGTACAAGAAAGCTGGGTCTATATTTTTTTCCTAACGTCAGAAACATT
8_hypothetical	M4	Tb927.4.4300_for	GGGGACAAGTTTGTACAAAAAAGCAGGCTATGAGGAAGAGGGATGCGCT	Tb927.4.4300_rev	GGGGACCACTTTGTACAAGAAAGCTGGGTTCAGCGTTGAGTTCTCAGAACTGC
8_hypothetical	M5	Tb927.10.3430_for	GGGGACAAGTTTGTACAAAAAAGCAGGCTATGGTAATGGCTTGCGGTGC	Tb927.10.3430_rev	GGGGACCACTTTGTACAAGAAAGCTGGGTCTATGAGTACTGGTAGCCTTTGGCC
8_hypothetical	M6	Tb927.8.5290_for	GGGGACAAGTTTGTACAAAAAAGCAGGCTATGTTCCTTTATCTTATCATCGATGGAGAC	Tb927.8.5290_rev	GGGGACCACTTTGTACAAGAAAGCTGGGTTCAAGGACGTGTCCAAGTTGTCT
8_hypothetical	M7	Tb927.8.4270_for	GGGGACAAGTTTGTACAAAAAAGCAGGCTATGGAGGGGCGGTTATATGACTT	Tb927.8.4270_rev	GGGGACCACTTTGTACAAGAAAGCTGGGTTTACGTGGAGGATCCCCAAGC
8_hypothetical	M8	Tb927.5.1600_for	GGGGACAAGTTTGTACAAAAAAGCAGGCTATGAGTGAACCATTGATGGCTAGGT	Tb927.5.1600_rev	GGGGACCACTTTGTACAAGAAAGCTGGGTCTATTCTTCATTTTCCTCACCATCGGA
8_hypothetical	M9	Tb927.11.14170_for	GGGGACAAGTTTGTACAAAAAAGCAGGCTATGCTTCTCGTAGCGCTGTTTATC	Tb927.11.14170_rev	GGGGACCACTTTGTACAAGAAAGCTGGGTCTAAATCGATAGAAGCTCGGCAGC
8_hypothetical	M10	Tb927.10.13240_for	GGGGACAAGTTTGTACAAAAAAGCAGGCTATGAAGGCGCCATCCCTCC	Tb927.10.13240_rev	GGGGACCACTTTGTACAAGAAAGCTGGGTTCACATCTTTGACGTGTAGTTGTGAT
8_hypothetical	M11	Tb927.3.4800_for	GGGGACAAGTTTGTACAAAAAAGCAGGCTATGTCTACGAAGGAGGAAGTTGCG	Tb927.3.4800_rev	GGGGACCACTTTGTACAAGAAAGCTGGGTCTACAAAGGCTTTTCGTTGTTTGTGT
8_hypothetical	M12	Tb927.3.4040_for	GGGGACAAGTTTGTACAAAAAAGCAGGCTATGCAGACGCCATTGACTGATG	Tb927.3.4040_rev	GGGGACCACTTTGTACAAGAAAGCTGGGTCTAAATGAAGAAGCATGGAATAGCTACCT
8_hypothetical	M13	Tb927.9.9160_for	GGGGACAAGTTTGTACAAAAAAGCAGGCTATGGCAAAGGACTCGGAGAAGA	Tb927.9.9160_rev	GGGGACCACTTTGTACAAGAAAGCTGGGTTCACAACACACCCAAGAAAGACAA
8_hypothetical	M14	Tb927.11.8760_for	GGGGACAAGTTTGTACAAAAAAGCAGGCTATGGAAGAGGTCAGTAACGTGGT	Tb927.11.8760_rev	GGGGACCACTTTGTACAAGAAAGCTGGGTTCACAACATTATATCATCTACGGCAGC
8_hypothetical	M15	Tb927.11.9370_for	GGGGACAAGTTTGTACAAAAAAGCAGGCTATGGAGAATGTGGTTATGGAAGCG	Tb927.11.9370_rev	GGGGACCACTTTGTACAAGAAAGCTGGGTTCACCATAAGTCAACTGACTCTCGT
8_hypothetical	M16	Tb927.11.12260_for	GGGGACAAGTTTGTACAAAAAAGCAGGCTATGCAGGAAGCACAGCAGAC	Tb927.11.12260_rev	GGGGACCACTTTGTACAAGAAAGCTGGGTTTAAGCTGTTCTCTCCGTGCTGA
8_hypothetical	M17	Tb927.3.3740_for	GGGGACAAGTTTGTACAAAAAAGCAGGCTATGAGAAACATCTGGGTTTCTGTTCC	Tb927.3.3740_rev	GGGGACCACTTTGTACAAGAAAGCTGGGTTTAACGAGGCTCAAAGAAATTACTCTTCA
8_hypothetical	M18	Tb927.11.2620_for	GGGGACAAGTTTGTACAAAAAAGCAGGCTATGCTTCAAAACTGTTGGTTACTTGC	Tb927.11.2620_rev	GGGGACCACTTTGTACAAGAAAGCTGGGTCTATAAGTGAGCCATACTCGGGTTCA
8_hypothetical	M19	Tb927.11.15320_for	GGGGACAAGTTTGTACAAAAAAGCAGGCTATGATCGCATCTTCTCCGGCC	Tb927.11.15320_rev	GGGGACCACTTTGTACAAGAAAGCTGGGTCTAGCTGTTTCCCTTTGGATTGAGG
8_hypothetical	M20	Tb927.11.12050_for	GGGGACAAGTTTGTACAAAAAAGCAGGCTATGGATATAGGCAACTTTGACTCACG	Tb927.11.12050_rev	GGGGACCACTTTGTACAAGAAAGCTGGGTCTACGCTTTTTTCTTCGCGAGGT
8_hypothetical	M21	Tb927.11.15250_for	GGGGACAAGTTTGTACAAAAAAGCAGGCTATGCACCTTCCACCGTTACCC	Tb927.11.15250_rev	GGGGACCACTTTGTACAAGAAAGCTGGGTTCATGACAAAGACACCCTCCCTT
8_hypothetical	M22	Tb927.10.8570_for	GGGGACAAGTTTGTACAAAAAAGCAGGCTATGCCACTTTTTAAACCATCGGAAATAC	Tb927.10.8570_rev	GGGGACCACTTTGTACAAGAAAGCTGGGTTCAATCTCGTATAACCTCAAACGTTCC
8_hypothetical	M23	Tb927.9.6010_for	GGGGACAAGTTTGTACAAAAAAGCAGGCTATGCTTCTAAAGTTCACGGCAACT	Tb927.9.6010_rev	GGGGACCACTTTGTACAAGAAAGCTGGGTTTAGTAACTCTGTGTTTGGGGGACA
8_hypothetical	M24	Tb927.8.5490_for	GGGGACAAGTTTGTACAAAAAAGCAGGCTATGAAAATCAAAAAGAAGGACTTGCGC	Tb927.8.5490_rev	GGGGACCACTTTGTACAAGAAAGCTGGGTCTATTCATCACCGTATAAATCCTCCACC
8_hypothetical	N1	Tb927.11.11460_for	GGGGACAAGTTTGTACAAAAAAGCAGGCTATGTCTAGTGACGCCACTGCT	Tb927.11.11460_rev	GGGGACCACTTTGTACAAGAAAGCTGGGTTCAGAACTGGAGAGGTTGTGAGG
8_hypothetical	N2	Tb927.7.6920_for	GGGGACAAGTTTGTACAAAAAAGCAGGCTATGCGAATGCCTGATACGAGGC	Tb927.7.6920_rev	GGGGACCACTTTGTACAAGAAAGCTGGGTTCATTGTTTCTCCTTCACCTCAGTATTTC
8_hypothetical	N3	Tb927.6.4750_for	GGGGACAAGTTTGTACAAAAAAGCAGGCTATGTTCGCCTACATCTGTGCCA	Tb927.6.4750_rev	GGGGACCACTTTGTACAAGAAAGCTGGGTTTATTCGCTGGTTGCATTGAGTGA
8_hypothetical	N4	Tb927.11.15620_for	GGGGACAAGTTTGTACAAAAAAGCAGGCTATGAAGCACTGTTCTGCTTGCG	Tb927.11.15620_rev	GGGGACCACTTTGTACAAGAAAGCTGGGTTTATACTGGAGAGTTGCGCGGA
8_hypothetical	N5	Tb927.3.3560_for	GGGGACAAGTTTGTACAAAAAAGCAGGCTATGGCGACAGTTAAGAAACTCTTGG	Tb927.3.3560_rev	GGGGACCACTTTGTACAAGAAAGCTGGGTCTAAAAGTCATAAATATCCACTTCATTCTC
8_hypothetical	N6	Tb927.7.5850_for	GGGGACAAGTTTGTACAAAAAAGCAGGCTATGTTGCGTACATCCCGCG	Tb927.7.5850_rev	GGGGACCACTTTGTACAAGAAAGCTGGGTTTATTCCGAATCGTCGAGCAAAAGC
8_hypothetical	N7	Tb927.6.3010_for	GGGGACAAGTTTGTACAAAAAAGCAGGCTATGACGCACGGTAGTAATAATGATTCTAC	Tb927.6.3010_rev	GGGGACCACTTTGTACAAGAAAGCTGGGTCTATTCTGCTCTTTCGTCATAATTAGCAGG
8_hypothetical	N8	Tb927.9.11960_for	GGGGACAAGTTTGTACAAAAAAGCAGGCTATGAAGCATATGTTGCCGATAAGCA	Tb927.9.11960_rev	GGGGACCACTTTGTACAAGAAAGCTGGGTCTATGATATATTGCTTTCTTGGAGTTGGGT
8_hypothetical	N9	Tb927.11.15340_for	GGGGACAAGTTTGTACAAAAAAGCAGGCTATGCCACGGGATCCGTTTAATG	Tb927.11.15340_rev	GGGGACCACTTTGTACAAGAAAGCTGGGTTCACCTTCGCATCGACCAGA
8_hypothetical	N10	Tb927.8.4200_for	GGGGACAAGTTTGTACAAAAAAGCAGGCTATGCTAGATCTATTTGCATCGTTGGTT	Tb927.8.4200_rev	GGGGACCACTTTGTACAAGAAAGCTGGGTTCACCCCTTATGTTGCGTTACCA
8_hypothetical	N11	Tb927.6.3900_for	GGGGACAAGTTTGTACAAAAAAGCAGGCTATGCAGGGTGTAAGGCGAGA	Tb927.6.3900_rev	GGGGACCACTTTGTACAAGAAAGCTGGGTCTACGAAGCACTCCTCGCAC
8_hypothetical	N12	Tb927.1.1850_for	GGGGACAAGTTTGTACAAAAAAGCAGGCTATGAATCGTGCTCGAAAGCCAG	Tb927.1.1850_rev	GGGGACCACTTTGTACAAGAAAGCTGGGTTTAAAAGTAATATGGGTTCACACTAGAGCA
8_hypothetical	N13	Tb927.8.5410_for	GGGGACAAGTTTGTACAAAAAAGCAGGCTATGGCTGCCATTTTCCCCAAA	Tb927.8.5410_rev	GGGGACCACTTTGTACAAGAAAGCTGGGTTCAATTAGGTTTGCTGAAAACAGTACTCA
8_hypothetical	N14	Tb927.11.2700_for	GGGGACAAGTTTGTACAAAAAAGCAGGCTATGACATGCAACGTTGGGACG	Tb927.11.2700_rev	GGGGACCACTTTGTACAAGAAAGCTGGGTTTACTGTTGAATTATTGCAACACGGC
8_hypothetical	N15	Tb927.5.1720_for	GGGGACAAGTTTGTACAAAAAAGCAGGCTATGAGGGGGTCTTTGCCTCTT	Tb927.5.1720_rev	GGGGACCACTTTGTACAAGAAAGCTGGGTTTATTCACATGTTCCCCTCGATACGG
8_hypothetical	N16	Tb927.11.9750_for	GGGGACAAGTTTGTACAAAAAAGCAGGCTATGCGCTGCTCTTTGGTTCA	Tb927.11.9750_rev	GGGGACCACTTTGTACAAGAAAGCTGGGTCTAAACCATGATGGGGTTCGTCC
8_hypothetical	N17	Tb927.11.4210_for	GGGGACAAGTTTGTACAAAAAAGCAGGCTATGTCCGTACACAGTGCCATCT	Tb927.11.4210_rev	GGGGACCACTTTGTACAAGAAAGCTGGGTTCACGCCACGGCACTGGC
8_hypothetical	N18	Tb927.7.4380_for	GGGGACAAGTTTGTACAAAAAAGCAGGCTATGTCGGGGGGCACCTCC	Tb927.7.4380_rev	GGGGACCACTTTGTACAAGAAAGCTGGGTTCAGTTAAGTTCATTTTCAACCCGTTTAAG
8_hypothetical	N19	Tb927.7.780_for	GGGGACAAGTTTGTACAAAAAAGCAGGCTATGGAGGCAACACGGCTCC	Tb927.7.780_rev	GGGGACCACTTTGTACAAGAAAGCTGGGTTTACTCTACAACCCAAGGTGGCG
8_hypothetical	N20	Tb927.5.1790_for	GGGGACAAGTTTGTACAAAAAAGCAGGCTATGTTCCGTCGTGCGATTCC	Tb927.5.1790_rev	GGGGACCACTTTGTACAAGAAAGCTGGGTTCATGCCTCCTGTAGCCAGAG
8_hypothetical	N21	Tb927.1.4720_for	GGGGACAAGTTTGTACAAAAAAGCAGGCTATGGAGGAGTTGCGTAAACGC	Tb927.1.4720_rev	GGGGACCACTTTGTACAAGAAAGCTGGGTCTAAAGGTAATAAACCTGCGCCAATTTC
8_hypothetical	N22	Tb927.4.2890_for	GGGGACAAGTTTGTACAAAAAAGCAGGCTATGAATTCTGCCTCACAGGATATCGC	Tb927.4.2890_rev	GGGGACCACTTTGTACAAGAAAGCTGGGTTCAGGTAACGAAGATGCGCTTTATT
8_hypothetical	N23	Tb927.10.770_for	GGGGACAAGTTTGTACAAAAAAGCAGGCTATGCATCGCCAGCGCCGC	Tb927.10.770_rev	GGGGACCACTTTGTACAAGAAAGCTGGGTCTATGAGGAGGCAGCGGCC
8_hypothetical	N24	Tb927.11.8860_for	GGGGACAAGTTTGTACAAAAAAGCAGGCTATGTCTGGACCGGTTTCCCA	Tb927.11.8860_rev	GGGGACCACTTTGTACAAGAAAGCTGGGTTCATGGAGACCTGTAGGCTGC
8_hypothetical	O1	Tb927.5.1920_for	GGGGACAAGTTTGTACAAAAAAGCAGGCTATGCACGCGGGAGGAACA	Tb927.5.1920_rev	GGGGACCACTTTGTACAAGAAAGCTGGGTTCACGCCGCATCGTTTCG
8_hypothetical	O2	Tb927.11.2100_for	GGGGACAAGTTTGTACAAAAAAGCAGGCTATGTTTCAAATTGATGTCACCGACG	Tb927.11.2100_rev	GGGGACCACTTTGTACAAGAAAGCTGGGTTTATGGCAGTAGAGGAGTTATACGTCG
8_hypothetical	O3	Tb927.9.13010_for	GGGGACAAGTTTGTACAAAAAAGCAGGCTATGTGGTGGAGTGTTCGCCT	Tb927.9.13010_rev	GGGGACCACTTTGTACAAGAAAGCTGGGTTTACTTTCCTGCGCCTAAGGGT
8_hypothetical	O4	Tb927.10.11630_for	GGGGACAAGTTTGTACAAAAAAGCAGGCTATGAGTGCCAAGTTACAACCCATC	Tb927.10.11630_rev	GGGGACCACTTTGTACAAGAAAGCTGGGTTTAAACTACGATCAAATCTCCCGCG
8_hypothetical	O5	Tb927.11.16650_for	GGGGACAAGTTTGTACAAAAAAGCAGGCTATGAGCGCCCGGGAAAGT	Tb927.11.16650_rev	GGGGACCACTTTGTACAAGAAAGCTGGGTTCATTCAACACCCGCAATCGC
8_hypothetical	O6	Tb927.5.2070_for	GGGGACAAGTTTGTACAAAAAAGCAGGCTATGAAATCTGTGTTTGATATTGCCAGAAG	Tb927.5.2070_rev	GGGGACCACTTTGTACAAGAAAGCTGGGTCTACACTACCGGCACCCCA
8_hypothetical	O7	Tb927.8.4510_for	GGGGACAAGTTTGTACAAAAAAGCAGGCTATGTTCAAAAAAACGAGAAAGGCACA	Tb927.8.4510_rev	GGGGACCACTTTGTACAAGAAAGCTGGGTTCATTGCTTAAAGAGAAACATAATGACGGA
8_hypothetical	O8	Tb927.7.3040_for	GGGGACAAGTTTGTACAAAAAAGCAGGCTATGATTGACCCATTTCGCTCTTCTT	Tb927.7.3040_rev	GGGGACCACTTTGTACAAGAAAGCTGGGTTCAGTCTCTCACGGAATACACACT
8_hypothetical	O9	Tb927.8.7480_for	GGGGACAAGTTTGTACAAAAAAGCAGGCTATGAAGTCTCGTGGTTTTACTCGAC	Tb927.8.7480_rev	GGGGACCACTTTGTACAAGAAAGCTGGGTCTAATATTGCACCACATGGCGCA
8_hypothetical	O10	Tb927.8.4000_for	GGGGACAAGTTTGTACAAAAAAGCAGGCTATGATGGGAAGAATTCGTGTTTGCC	Tb927.8.4000_rev	GGGGACCACTTTGTACAAGAAAGCTGGGTTCACGCTTTCTCCTCTTGCGA
8_hypothetical	O11	Tb927.10.3850_for	GGGGACAAGTTTGTACAAAAAAGCAGGCTATGACAGAAGTGAAGGGGCACC	Tb927.10.3850_rev	GGGGACCACTTTGTACAAGAAAGCTGGGTTCAGTCACTCATAGGAAATTGAGCGC
8_hypothetical	O12	Tb927.10.11480_for	GGGGACAAGTTTGTACAAAAAAGCAGGCTATGAGATGCGCACGTTTCTATCTT	Tb927.10.11480_rev	GGGGACCACTTTGTACAAGAAAGCTGGGTCTACAGCCTTGGCTTTTCCAACG
8_hypothetical	O13	Tb927.10.11810_for	GGGGACAAGTTTGTACAAAAAAGCAGGCTATGTCACAGGCAGGTGTGAGT	Tb927.10.11810_rev	GGGGACCACTTTGTACAAGAAAGCTGGGTTCATAACTTCGATACCACATACGCCG
8_hypothetical	O14	Tb927.10.9160_for	GGGGACAAGTTTGTACAAAAAAGCAGGCTATGACTGCGACTTCCGGCT	Tb927.10.9160_rev	GGGGACCACTTTGTACAAGAAAGCTGGGTCTACCTTCGACGCCGCTG
8_hypothetical	O15	Tb927.7.6320_for	GGGGACAAGTTTGTACAAAAAAGCAGGCTATGATGCGACGGACGGGT	Tb927.7.6320_rev	GGGGACCACTTTGTACAAGAAAGCTGGGTTTAAGTTGTTGGGAAATCATCACCACG
8_hypothetical	O16	Tb927.8.770_for	GGGGACAAGTTTGTACAAAAAAGCAGGCTATGAATGAGGGGAAGGGTACGTC	Tb927.8.770_rev	GGGGACCACTTTGTACAAGAAAGCTGGGTTCAGGTTTTCCACGTTATTGTGGAA
8_hypothetical	O17	Tb927.7.5070_for	GGGGACAAGTTTGTACAAAAAAGCAGGCTATGGCACAAGGACGAGTTATTGAG	Tb927.7.5070_rev	GGGGACCACTTTGTACAAGAAAGCTGGGTTTAGCACGTGAGTTCCTTGCG
8_hypothetical	O18	Tb927.11.13550_for	GGGGACAAGTTTGTACAAAAAAGCAGGCTATGACCCACCAAACCCATGCG	Tb927.11.13550_rev	GGGGACCACTTTGTACAAGAAAGCTGGGTTCACCAAAAACAAACGTACGCAGC
8_hypothetical	O19	Tb927.7.6280_for	GGGGACAAGTTTGTACAAAAAAGCAGGCTATGGACGTCACGCCTGTGG	Tb927.7.6280_rev	GGGGACCACTTTGTACAAGAAAGCTGGGTTTAATACAGGAAGGTCTTCTGTACCGT
8_hypothetical	O20	Tb927.11.4150_for	GGGGACAAGTTTGTACAAAAAAGCAGGCTATGCGCGTGCTAGCTGTG	Tb927.11.4150_rev	GGGGACCACTTTGTACAAGAAAGCTGGGTTCAAATCTCACCCGTTTCACCATC
8_hypothetical	O21	Tb927.10.450_for	GGGGACAAGTTTGTACAAAAAAGCAGGCTATGCCCGAGGCGTATATTGAGG	Tb927.10.450_rev	GGGGACCACTTTGTACAAGAAAGCTGGGTCTAACGGGTAAGGGCATCGGG
8_hypothetical	O22	Tb927.9.8180_for	GGGGACAAGTTTGTACAAAAAAGCAGGCTATGGGGAGTGATCTTTCCATTGTGT	Tb927.9.8180_rev	GGGGACCACTTTGTACAAGAAAGCTGGGTTTATTTTACACGCTTTGGTGGCTCC
8_hypothetical	O23	Tb927.11.2180_for	GGGGACAAGTTTGTACAAAAAAGCAGGCTATGCCGTCTGCTAGTTCCGG	Tb927.11.2180_rev	GGGGACCACTTTGTACAAGAAAGCTGGGTTCAAGTTTGATTCCTCAGCGCG
8_hypothetical	O24	Tb927.8.3930_for	GGGGACAAGTTTGTACAAAAAAGCAGGCTATGAACTTCTTATCCCAGATTTACACCG	Tb927.8.3930_rev	GGGGACCACTTTGTACAAGAAAGCTGGGTCTAATAGACGGCGGTGCCG
8_hypothetical	P1	Tb927.11.2890_for	GGGGACAAGTTTGTACAAAAAAGCAGGCTATGCTTTTTCGTGAAACGGCAAG	Tb927.11.2890_rev	GGGGACCACTTTGTACAAGAAAGCTGGGTTCACTGCAGTGGCGGAGG
8_hypothetical	P2	Tb927.11.6040_for	GGGGACAAGTTTGTACAAAAAAGCAGGCTATGCATTTGGAGCAAGCTGGAG	Tb927.11.6040_rev	GGGGACCACTTTGTACAAGAAAGCTGGGTTTAATAAATGAACCCACCCCTCTTTTTGA
8_hypothetical	P3	Tb927.10.9140_for	GGGGACAAGTTTGTACAAAAAAGCAGGCTATGGGTTCTTTTACTTCCTGTGACCA	Tb927.10.9140_rev	GGGGACCACTTTGTACAAGAAAGCTGGGTCTACGATGACGATGACGGAGTGG
8_hypothetical	P4	Tb927.11.14860_for	GGGGACAAGTTTGTACAAAAAAGCAGGCTATGTTTGCGGACCGCGAA	Tb927.11.14860_rev	GGGGACCACTTTGTACAAGAAAGCTGGGTTTAGTTTGCGCACTGTTGGCG
8_hypothetical	P5	Tb927.9.10820_for	GGGGACAAGTTTGTACAAAAAAGCAGGCTATGAGCGATAAGGTAAATGTCCATAAAAAT	Tb927.9.10820_rev	GGGGACCACTTTGTACAAGAAAGCTGGGTTTATATCCCGCTCATCGCCAAAC
8_hypothetical	P6	Tb927.10.930_for	GGGGACAAGTTTGTACAAAAAAGCAGGCTATGCAGAACGTTATCCTTGCCC	Tb927.10.930_rev	GGGGACCACTTTGTACAAGAAAGCTGGGTTTATTCCTGCGGCTGCTGC
8_hypothetical	P7	Tb927.3.5070_for	GGGGACAAGTTTGTACAAAAAAGCAGGCTATGTCCTCTCTCCCTTACCGC	Tb927.3.5070_rev	GGGGACCACTTTGTACAAGAAAGCTGGGTTCACTCAACATATTTCCCCCCCC
8_hypothetical	P8	Tb927.8.2600_for	GGGGACAAGTTTGTACAAAAAAGCAGGCTATGGGCATGAAGCTGCAGC	Tb927.8.2600_rev	GGGGACCACTTTGTACAAGAAAGCTGGGTCTACCCCATCAAACGCCTGAGG
8_hypothetical	P9	Tb927.11.4400_for	GGGGACAAGTTTGTACAAAAAAGCAGGCTATGCTTGGTTCATGGCTGAACG	Tb927.11.4400_rev	GGGGACCACTTTGTACAAGAAAGCTGGGTTCAACTCTGGGATTTCGTCTGTTG
8_hypothetical	P10	Tb927.11.2420_for	GGGGACAAGTTTGTACAAAAAAGCAGGCTATGATGAAAGACTTTGAAGCATTCTCGA	Tb927.11.2420_rev	GGGGACCACTTTGTACAAGAAAGCTGGGTTCACAGTGGTGTCATGTTCGTTG
8_hypothetical	P11	Tb927.11.10330_for	GGGGACAAGTTTGTACAAAAAAGCAGGCTATGTTCTTCTGGGGTTCGTACAAC	Tb927.11.10330_rev	GGGGACCACTTTGTACAAGAAAGCTGGGTTCATTTTCCGACGACATAGTTTCCAG
8_hypothetical	P12	Tb927.2.2410_for	GGGGACAAGTTTGTACAAAAAAGCAGGCTATGGTAGTGCACCGTCGCA	Tb927.2.2410_rev	GGGGACCACTTTGTACAAGAAAGCTGGGTTCACCTTCCCGCGAGAAGT
8_hypothetical	P13	Tb927.10.1930_for	GGGGACAAGTTTGTACAAAAAAGCAGGCTATGCGCCCTGCAGCCACT	Tb927.10.1930_rev	GGGGACCACTTTGTACAAGAAAGCTGGGTCTATCCCATCTCCTTACGCCCG
8_hypothetical	P14	Tb927.8.4310_for	GGGGACAAGTTTGTACAAAAAAGCAGGCTATGGAAAGGCGTATTGAAGAGGATG	Tb927.8.4310_rev	GGGGACCACTTTGTACAAGAAAGCTGGGTTTAATTGCGATAGCAACCGCGC
8_hypothetical	P15	Tb927.11.280_for	GGGGACAAGTTTGTACAAAAAAGCAGGCTATGAGTTTGACACTGGGAGAATTAAGTG	Tb927.11.280_rev	GGGGACCACTTTGTACAAGAAAGCTGGGTTCACTGAGTAGCAGAAATGTAGTTTCC
8_hypothetical	P16	Tb927.11.14890_for	GGGGACAAGTTTGTACAAAAAAGCAGGCTATGCAACAATCAACGCGATGGA	Tb927.11.14890_rev	GGGGACCACTTTGTACAAGAAAGCTGGGTTCACGCAGCGGCGGCGAT
8_hypothetical	P17	Tb927.5.1980_for	GGGGACAAGTTTGTACAAAAAAGCAGGCTATGATACCGAGGCATTATGTAGCAATG	Tb927.5.1980_rev	GGGGACCACTTTGTACAAGAAAGCTGGGTTTAACCCCTCAGTTCCTCGGG
8_hypothetical	P18	Tb927.7.3660_for	GGGGACAAGTTTGTACAAAAAAGCAGGCTATGTCGACACTGGAGGGCTG	Tb927.7.3660_rev	GGGGACCACTTTGTACAAGAAAGCTGGGTTCAATACGGGAAGATATCTGAAAAGACG
8_hypothetical	P19	Tb927.10.12430_for	GGGGACAAGTTTGTACAAAAAAGCAGGCTATGGCAAAGGAAAAAAAATCGTTCAAGA	Tb927.10.12430_rev	GGGGACCACTTTGTACAAGAAAGCTGGGTCTAGGAGTTGTGGTCGCTCCT
8_hypothetical	P20	Tb927.11.10960_for	GGGGACAAGTTTGTACAAAAAAGCAGGCTATGCGCAGGTGCCTTATTTTGC	Tb927.11.10960_rev	GGGGACCACTTTGTACAAGAAAGCTGGGTTTACGCAGTTGGCTGGCCG
8_hypothetical	P21	Tb927.11.5120_for	GGGGACAAGTTTGTACAAAAAAGCAGGCTATGCAGTTAGCTGAGGAACTAAAACAA	Tb927.11.5120_rev	GGGGACCACTTTGTACAAGAAAGCTGGGTTTACACCTTCTCGACTGCCTTTTTAAT
8_hypothetical	P22	Tb927.11.5410_for	GGGGACAAGTTTGTACAAAAAAGCAGGCTATGATCGAAAATGTTACCGCAGTGT	Tb927.11.5410_rev	GGGGACCACTTTGTACAAGAAAGCTGGGTCTATTCAGCCGGTATTGTAGTAGCAC
8_hypothetical	P23	Tb927.10.13690_for	GGGGACAAGTTTGTACAAAAAAGCAGGCTATGACATTTATTTCCTTCCATAGTTTGGCG	Tb927.10.13690_rev	GGGGACCACTTTGTACAAGAAAGCTGGGTCTACGCAACTAGCTCCGTATCCA
8_hypothetical	P24	Tb927.6.3520_for	GGGGACAAGTTTGTACAAAAAAGCAGGCTATGAACCAAAAAGAGGATCACGAGG	Tb927.6.3520_rev	GGGGACCACTTTGTACAAGAAAGCTGGGTTCAGTCCTCTGCACGGGG
9_hypothetical	A1	Tb927.10.13390_for	GGGGACAAGTTTGTACAAAAAAGCAGGCTATGACATTTATTTCCTTCCATAGTTTGGCG	Tb927.10.13390_rev	GGGGACCACTTTGTACAAGAAAGCTGGGTTTACGCAACTAGCTCCGTATCCA
9_hypothetical	A2	Tb927.10.1350_for	GGGGACAAGTTTGTACAAAAAAGCAGGCTATGGGAGCCGCGTGCACC	Tb927.10.1350_rev	GGGGACCACTTTGTACAAGAAAGCTGGGTTCACGAAACCCTAGTGACAAATCG
9_hypothetical	A3	Tb927.10.7880_for	GGGGACAAGTTTGTACAAAAAAGCAGGCTATGGAGACGGGAGGCGAC	Tb927.10.7880_rev	GGGGACCACTTTGTACAAGAAAGCTGGGTTCAGGTATGGGCACGGGTTT
9_hypothetical	A4	Tb927.7.7030_for	GGGGACAAGTTTGTACAAAAAAGCAGGCTATGCCCCACGAGCCGTCG	Tb927.7.7030_rev	GGGGACCACTTTGTACAAGAAAGCTGGGTCTACCGCCGCGTTGGCCG
9_hypothetical	A5	Tb927.8.3590_for	GGGGACAAGTTTGTACAAAAAAGCAGGCTATGAGTTCTGTGGGTGTGAGCG	Tb927.8.3590_rev	GGGGACCACTTTGTACAAGAAAGCTGGGTCTATGACGGGACAGGAGAGGC
9_hypothetical	A6	Tb927.11.10470_for	GGGGACAAGTTTGTACAAAAAAGCAGGCTATGAAGACTCATCGTGCCGC	Tb927.11.10470_rev	GGGGACCACTTTGTACAAGAAAGCTGGGTCTACTCAGTTAATGAACCTTCTGTGTAGTA
9_hypothetical	A7	Tb927.9.11830_for	GGGGACAAGTTTGTACAAAAAAGCAGGCTATGCTTACTTCATCCTTCATCAGCG	Tb927.9.11830_rev	GGGGACCACTTTGTACAAGAAAGCTGGGTCTAGTCTAGCGCTAAGCGAGCG
9_hypothetical	A8	Tb927.11.7180_for	GGGGACAAGTTTGTACAAAAAAGCAGGCTATGACAACGAAGGAAGCAGTGG	Tb927.11.7180_rev	GGGGACCACTTTGTACAAGAAAGCTGGGTTCACACCCCGTCATGAGAGG
9_hypothetical	A9	Tb927.8.2680_for	GGGGACAAGTTTGTACAAAAAAGCAGGCTATGCGGTGGATCCCTGCA	Tb927.8.2680_rev	GGGGACCACTTTGTACAAGAAAGCTGGGTTTACATGTCTCTCGGCCGCT
9_hypothetical	A10	Tb927.7.3670_for	GGGGACAAGTTTGTACAAAAAAGCAGGCTATGCAGGAAGACGTAAAACACCTT	Tb927.7.3670_rev	GGGGACCACTTTGTACAAGAAAGCTGGGTCTACGGTGAATTTGCCGAGCA
9_hypothetical	A11	Tb927.4.2430_for	GGGGACAAGTTTGTACAAAAAAGCAGGCTATGGCACGGCACCCCAAC	Tb927.4.2430_rev	GGGGACCACTTTGTACAAGAAAGCTGGGTCTATTTGTACGACAAATCGGCAATCAA
9_hypothetical	A12	Tb927.3.3170_for	GGGGACAAGTTTGTACAAAAAAGCAGGCTATGCATACAGCGGCATCGAAG	Tb927.3.3170_rev	GGGGACCACTTTGTACAAGAAAGCTGGGTCTAGACGGCGGCACGAGG
9_hypothetical	A13	Tb927.11.990_for	GGGGACAAGTTTGTACAAAAAAGCAGGCTATGTTCTTCACACATGGGCCG	Tb927.11.990_rev	GGGGACCACTTTGTACAAGAAAGCTGGGTTTATGTCCCGCATCCCTCTCTC
9_hypothetical	A14	Tb927.3.2870_for	GGGGACAAGTTTGTACAAAAAAGCAGGCTATGAATGGACGAATCACCGGC	Tb927.3.2870_rev	GGGGACCACTTTGTACAAGAAAGCTGGGTCTAAGGAATCTGCGTCTCAGCTTT
9_hypothetical	A15	Tb927.10.6900_for	GGGGACAAGTTTGTACAAAAAAGCAGGCTATGGGAGGTCCAAGTGAGGGG	Tb927.10.6900_rev	GGGGACCACTTTGTACAAGAAAGCTGGGTTTAGTCTGCCTGCGACCCTC
9_hypothetical	A16	Tb927.11.16990_for	GGGGACAAGTTTGTACAAAAAAGCAGGCTATGTGCATATGTACACGTCTCTTGTG	Tb927.11.16990_rev	GGGGACCACTTTGTACAAGAAAGCTGGGTTTACGCAGCCCAGAAACCGG
9_hypothetical	A17	Tb927.9.14170_for	GGGGACAAGTTTGTACAAAAAAGCAGGCTATGGATGGGCGGATTAAAGGCC	Tb927.9.14170_rev	GGGGACCACTTTGTACAAGAAAGCTGGGTTTACACAGCACTCACTGAGGGA
9_hypothetical	A18	Tb927.10.2460_for	GGGGACAAGTTTGTACAAAAAAGCAGGCTATGTTCCTCAGAAGTGATCAGCATCG	Tb927.10.2460_rev	GGGGACCACTTTGTACAAGAAAGCTGGGTCTAGAGGAAATGATATAACAGAACGATCGT
9_hypothetical	A19	Tb927.9.4450_for	GGGGACAAGTTTGTACAAAAAAGCAGGCTATGGAAAACGCAAGGAATCTGGAG	Tb927.9.4450_rev	GGGGACCACTTTGTACAAGAAAGCTGGGTTTAATTTTGATTACTAGTATCATTAGGACG
9_hypothetical	A20	Tb927.11.5310_for	GGGGACAAGTTTGTACAAAAAAGCAGGCTATGCCTCTCTGCTGTTGTAAAGATG	Tb927.11.5310_rev	GGGGACCACTTTGTACAAGAAAGCTGGGTTTAGAATGGCGAAGAGTAAGTTCTCATATG
9_hypothetical	A21	Tb927.10.630_for	GGGGACAAGTTTGTACAAAAAAGCAGGCTATGTCGTACAAATATAGCAGTAGAGAATAT	Tb927.10.630_rev	GGGGACCACTTTGTACAAGAAAGCTGGGTTCATTTTTTCTCTTCACGACTAAATAACAT
9_hypothetical	A22	Tb927.9.13140_for	GGGGACAAGTTTGTACAAAAAAGCAGGCTATGCACAAACCGGTGACGG	Tb927.9.13140_rev	GGGGACCACTTTGTACAAGAAAGCTGGGTCTATGCTCCCTCATTCTCATCCGG
9_hypothetical	A23	Tb927.11.2630_for	GGGGACAAGTTTGTACAAAAAAGCAGGCTATGTACCACGATGCTCCGCC	Tb927.11.2630_rev	GGGGACCACTTTGTACAAGAAAGCTGGGTTCATTGTTCCTTTTCTGTTGCATATCCA
9_hypothetical	A24	Tb927.9.8200_for	GGGGACAAGTTTGTACAAAAAAGCAGGCTATGCACCGAAAGCAGGCATG	Tb927.9.8200_rev	GGGGACCACTTTGTACAAGAAAGCTGGGTTCAACGCACCCACTTTGGAAG
9_hypothetical	B1	Tb927.11.14930_for	GGGGACAAGTTTGTACAAAAAAGCAGGCTATGGATAGGGAAAAACTAGCGTGGG	Tb927.11.14930_rev	GGGGACCACTTTGTACAAGAAAGCTGGGTTTACACATACACGTTAAACGGTGCT
9_hypothetical	B2	Tb927.11.2970_for	GGGGACAAGTTTGTACAAAAAAGCAGGCTATGAGACCGCGCCCTGTT	Tb927.11.2970_rev	GGGGACCACTTTGTACAAGAAAGCTGGGTTCATAAAAGACCACTGAGGTTAAGGAGT
9_hypothetical	B3	Tb927.11.6770_for	GGGGACAAGTTTGTACAAAAAAGCAGGCTATGCTGTGCCACTTCTTTGCA	Tb927.11.6770_rev	GGGGACCACTTTGTACAAGAAAGCTGGGTCTAGTCGTCTTCGTCTCCATCCT
9_hypothetical	B4	Tb927.9.14300_for	GGGGACAAGTTTGTACAAAAAAGCAGGCTATGGATGTGTGTGAGGGATTGAGG	Tb927.9.14300_rev	GGGGACCACTTTGTACAAGAAAGCTGGGTTCATAGAAGCTCACGGTGTCTGG
9_hypothetical	B5	Tb927.10.15190_for	GGGGACAAGTTTGTACAAAAAAGCAGGCTATGGAAGGTGCTGCAAGGACG	Tb927.10.15190_rev	GGGGACCACTTTGTACAAGAAAGCTGGGTTTACTGTAATTCGTGATGGGTTAAACCC

9_hypothetical	B6	Tb927.9.11690_for	GGGGACAAGTTTGTACAAAAAAGCAGGCTATGAGTGGTTCCCATTGGATGCT	Tb927.9.11690_rev	GGGGACCACTTTGTACAAGAAAGCTGGGTTTATGGAGAACGAACTGATGAGGTGA
9_hypothetical	B7	Tb927.11.13400_for	GGGGACAAGTTTGTACAAAAAAGCAGGCTATGAGTCAAAACCGGCAGCTG	Tb927.11.13400_rev	GGGGACCACTTTGTACAAGAAAGCTGGGTTCACAAGGCGGGAGCCTC
9_hypothetical	B8	Tb927.5.530_for	GGGGACAAGTTTGTACAAAAAAGCAGGCTATGAGGACGTTCTTTTCGCACG	Tb927.5.530_rev	GGGGACCACTTTGTACAAGAAAGCTGGGTTCAGACGCTAGTTTTGTTGTTGTTGC
9_hypothetical	B9	Tb927.10.10150_for	GGGGACAAGTTTGTACAAAAAAGCAGGCTATGACAGACGCCGAATCTGACG	Tb927.10.10150_rev	GGGGACCACTTTGTACAAGAAAGCTGGGTTCACCAGGGGCCCCGCGT
9_hypothetical	B10	Tb927.11.2780_for	GGGGACAAGTTTGTACAAAAAAGCAGGCTATGCTAGCAGGAAAGATTAGCGGA	Tb927.11.2780_rev	GGGGACCACTTTGTACAAGAAAGCTGGGTCTATTTTACTCGATCACTCCTCTTCTTCG
9_hypothetical	B11	Tb927.7.5310_for	GGGGACAAGTTTGTACAAAAAAGCAGGCTATGATTCACGGCAATGCTGCG	Tb927.7.5310_rev	GGGGACCACTTTGTACAAGAAAGCTGGGTTTATGTGTACTGCTGGTAGATATGAGCC
9_hypothetical	B12	Tb927.4.4140_for	GGGGACAAGTTTGTACAAAAAAGCAGGCTATGACTGCAGTTAAGGAGCCTCG	Tb927.4.4140_rev	GGGGACCACTTTGTACAAGAAAGCTGGGTTCATAAGTTCCACGTCAGCCCG
9_hypothetical	B13	Tb927.11.8300_for	GGGGACAAGTTTGTACAAAAAAGCAGGCTATGTCGTCAAAGAAGAAGGGCTCG	Tb927.11.8300_rev	GGGGACCACTTTGTACAAGAAAGCTGGGTCTATTTCAGCAGCGTTAACAACACC
9_hypothetical	B15	Tb927.7.2720_for	GGGGACAAGTTTGTACAAAAAAGCAGGCTATGTTTCACAAGAAATATGTCGTGAAAGG	Tb927.7.2720_rev	GGGGACCACTTTGTACAAGAAAGCTGGGTTCATGCACAGCGTTCAGCTATT
9_hypothetical	B16	Tb927.10.4320_for	GGGGACAAGTTTGTACAAAAAAGCAGGCTATGAAACCGCGAGACCTATGGG	Tb927.10.4320_rev	GGGGACCACTTTGTACAAGAAAGCTGGGTCTACGCCTGCATAGAGTAAAGTCTCT
9_hypothetical	B17	Tb927.5.1640_for	GGGGACAAGTTTGTACAAAAAAGCAGGCTATGCGCCCCAACTCGATGC	Tb927.5.1640_rev	GGGGACCACTTTGTACAAGAAAGCTGGGTTCAGTAGGTTGTGGACCGCC
9_hypothetical	B18	Tb927.3.710_for	GGGGACAAGTTTGTACAAAAAAGCAGGCTATGCTCTTATATTTTATTGTTGTTGCCGTA	Tb927.3.710_rev	GGGGACCACTTTGTACAAGAAAGCTGGGTCTACACGATGTCGGCGTTACG
9_hypothetical	B19	Tb927.7.700_for	GGGGACAAGTTTGTACAAAAAAGCAGGCTATGCAGGTATCGCTCCATAACAAG	Tb927.7.700_rev	GGGGACCACTTTGTACAAGAAAGCTGGGTTCATTTAGACGACACTCTCCGAAGC
9_hypothetical	B20	Tb927.7.5630_for	GGGGACAAGTTTGTACAAAAAAGCAGGCTATGCGGGCATCTGCAGCT	Tb927.7.5630_rev	GGGGACCACTTTGTACAAGAAAGCTGGGTTTACTCAGCCTTTGCACTAGGGA
9_hypothetical	B21	Tb927.9.6510_for	GGGGACAAGTTTGTACAAAAAAGCAGGCTATGTCAGTGAGCGGTGTGTTC	Tb927.9.6510_rev	GGGGACCACTTTGTACAAGAAAGCTGGGTCTAAAACTCCGGAGTCGCGC
9_hypothetical	B22	Tb927.7.5890_for	GGGGACAAGTTTGTACAAAAAAGCAGGCTATGAATGAGGATAAAAAAGCACCCTTGA	Tb927.7.5890_rev	GGGGACCACTTTGTACAAGAAAGCTGGGTTCACACTCTCCTTTCCATGTCCTT
9_hypothetical	B23	Tb927.11.3020_for	GGGGACAAGTTTGTACAAAAAAGCAGGCTATGCATGGGAGGAATAATGCGG	Tb927.11.3020_rev	GGGGACCACTTTGTACAAGAAAGCTGGGTCTAGAAGAGGGAACATATTCGCTTTTTCA
9_hypothetical	B24	Tb927.3.2800_for	GGGGACAAGTTTGTACAAAAAAGCAGGCTATGATTGGCAGGTTATTGGGGTTTAG	Tb927.3.2800_rev	GGGGACCACTTTGTACAAGAAAGCTGGGTCTATGAAAAGCAAACGACGTTCAGC
9_hypothetical	C1	Tb927.8.4400_for	GGGGACAAGTTTGTACAAAAAAGCAGGCTATGACTAGGTTGCCTTCAGCGG	Tb927.8.4400_rev	GGGGACCACTTTGTACAAGAAAGCTGGGTTCACCGCTGGCGTTCCTC
9_hypothetical	C2	Tb927.11.9280_for	GGGGACAAGTTTGTACAAAAAAGCAGGCTATGCTCTCCAGTGCGCTGA	Tb927.11.9280_rev	GGGGACCACTTTGTACAAGAAAGCTGGGTTCAACCTACGCGCATAACCTTATC
9_hypothetical	C3	Tb927.11.16680_for	GGGGACAAGTTTGTACAAAAAAGCAGGCTATGCTTCAGGCCTGTGGTGT	Tb927.11.16680_rev	GGGGACCACTTTGTACAAGAAAGCTGGGTTCACCACCAAGTGTCACAGCC
9_hypothetical	C4	Tb927.10.14560_for	GGGGACAAGTTTGTACAAAAAAGCAGGCTATGAGCGATTCAAAGAAAAAGGCCG	Tb927.10.14560_rev	GGGGACCACTTTGTACAAGAAAGCTGGGTTTATCTCCGTTTAACCTTCTTTCCGC
9_hypothetical	C5	Tb927.4.4940_for	GGGGACAAGTTTGTACAAAAAAGCAGGCTATGAAGTCCCGTGGTTTCACCC	Tb927.4.4940_rev	GGGGACCACTTTGTACAAGAAAGCTGGGTCTAGCATAGCGCAACGTGGC
9_hypothetical	C6	Tb927.8.1700_for	GGGGACAAGTTTGTACAAAAAAGCAGGCTATGCCGTTGAAGTTTGTTCACCA	Tb927.8.1700_rev	GGGGACCACTTTGTACAAGAAAGCTGGGTCTAGACGGCGTCAAACCCAAG
9_hypothetical	C7	Tb927.6.2400_for	GGGGACAAGTTTGTACAAAAAAGCAGGCTATGGAAGAACTGCAGTGCGC	Tb927.6.2400_rev	GGGGACCACTTTGTACAAGAAAGCTGGGTCTACCGGTGCCCTGCTTTC
9_hypothetical	C8	Tb927.7.2220_for	GGGGACAAGTTTGTACAAAAAAGCAGGCTATGCGCGGCTTCCGCTCA	Tb927.7.2220_rev	GGGGACCACTTTGTACAAGAAAGCTGGGTCTACTGTTCCCTCGACCCACC
9_hypothetical	C9	Tb927.10.2690_for	GGGGACAAGTTTGTACAAAAAAGCAGGCTATGATGAACTTAGGAGAAACTCTTGACAC	Tb927.10.2690_rev	GGGGACCACTTTGTACAAGAAAGCTGGGTTCACCACACCCTCGACTTGC
9_hypothetical	C10	Tb927.9.6410_for	GGGGACAAGTTTGTACAAAAAAGCAGGCTATGATTCTCCGGGGACGGTT	Tb927.9.6410_rev	GGGGACCACTTTGTACAAGAAAGCTGGGTTCAAGAACCATCAATTTCCGCGC
9_hypothetical	C11	Tb927.6.3720_for	GGGGACAAGTTTGTACAAAAAAGCAGGCTATGTCCCACACTGGGTGTCC	Tb927.6.3720_rev	GGGGACCACTTTGTACAAGAAAGCTGGGTTCACAAATGTGGAGCAACATCCC
9_hypothetical	C12	Tb927.7.2950_for	GGGGACAAGTTTGTACAAAAAAGCAGGCTATGTTTACATCTTTTTTAATAGTTTTAAAT	Tb927.7.2950_rev	GGGGACCACTTTGTACAAGAAAGCTGGGTTCACTGCACGTTGGCAGAAG
9_hypothetical	C13	Tb927.4.2760_for	GGGGACAAGTTTGTACAAAAAAGCAGGCTATGGAAGAGGGTGATCATATGGAGTT	Tb927.4.2760_rev	GGGGACCACTTTGTACAAGAAAGCTGGGTCTAAATGATGTGACTGACCACTGGC
9_hypothetical	C14	Tb927.10.3730_for	GGGGACAAGTTTGTACAAAAAAGCAGGCTATGCGTCCGTATTTCCCAGGT	Tb927.10.3730_rev	GGGGACCACTTTGTACAAGAAAGCTGGGTTCACCGTGCTGGTCGCGC
9_hypothetical	C15	Tb927.9.10190_for	GGGGACAAGTTTGTACAAAAAAGCAGGCTATGGAAGGAGACGTCATCCCTTC	Tb927.9.10190_rev	GGGGACCACTTTGTACAAGAAAGCTGGGTTTAGTCAGCCGCATCATACACCA
9_hypothetical	C16	Tb927.7.1560_for	GGGGACAAGTTTGTACAAAAAAGCAGGCTATGACTCTGGGCGCTGCT	Tb927.7.1560_rev	GGGGACCACTTTGTACAAGAAAGCTGGGTTCACTCCACCTGCTCATTACCC
9_hypothetical	C17	Tb927.4.3310_for	GGGGACAAGTTTGTACAAAAAAGCAGGCTATGAGTGGTGGTGTGTCCCC	Tb927.4.3310_rev	GGGGACCACTTTGTACAAGAAAGCTGGGTTCACAACGCCCTCTGGCA
9_hypothetical	C18	Tb927.3.1880_for	GGGGACAAGTTTGTACAAAAAAGCAGGCTATGGGACCTTTAGTTGTTGTATCGC	Tb927.3.1880_rev	GGGGACCACTTTGTACAAGAAAGCTGGGTTCAGTAGACACAAACACAGTTACATCC
9_hypothetical	C19	Tb927.3.4550_for	GGGGACAAGTTTGTACAAAAAAGCAGGCTATGTTAAACCGCTGTTACATACCACG	Tb927.3.4550_rev	GGGGACCACTTTGTACAAGAAAGCTGGGTTCAAGACGGTACAAAGAGTCGCT
9_hypothetical	C20	Tb927.9.2620_for	GGGGACAAGTTTGTACAAAAAAGCAGGCTATGTACAAGAAGGCCCTTCATTCCT	Tb927.9.2620_rev	GGGGACCACTTTGTACAAGAAAGCTGGGTTTAAAATCCAGGTTTTATCCCCCTGGT
9_hypothetical	C21	Tb927.3.4120_for	GGGGACAAGTTTGTACAAAAAAGCAGGCTATGAGTAGCGAGGGGCCCC	Tb927.3.4120_rev	GGGGACCACTTTGTACAAGAAAGCTGGGTCTAGCGACGCAGTAAGAAGGC
9_hypothetical	C22	Tb927.11.8410_for	GGGGACAAGTTTGTACAAAAAAGCAGGCTATGTCACAACTGCCGTTAATTCATACG	Tb927.11.8410_rev	GGGGACCACTTTGTACAAGAAAGCTGGGTTCAAATAGGTGAAACGGGGCG
9_hypothetical	C23	Tb927.7.5470_for	GGGGACAAGTTTGTACAAAAAAGCAGGCTATGTCCCGCCCCTGTGGC	Tb927.7.5470_rev	GGGGACCACTTTGTACAAGAAAGCTGGGTCTACGTTGCATGCGCTAGTCT
9_hypothetical	C24	Tb927.8.4860_for	GGGGACAAGTTTGTACAAAAAAGCAGGCTATGCTGTCGCACGCCGTC	Tb927.8.4860_rev	GGGGACCACTTTGTACAAGAAAGCTGGGTTTATACCAATTGCTCCCCAGGCG
9_hypothetical	D1	Tb927.10.13790_for	GGGGACAAGTTTGTACAAAAAAGCAGGCTATGGCAACCATAGCGGAGTTG	Tb927.10.13790_rev	GGGGACCACTTTGTACAAGAAAGCTGGGTTTACGCGTTGCGAACACCTT
9_hypothetical	D2	Tb927.2.2360_for	GGGGACAAGTTTGTACAAAAAAGCAGGCTATGTTTAGTGGGAATCCATATATAACCCCT	Tb927.2.2360_rev	GGGGACCACTTTGTACAAGAAAGCTGGGTTTACGTGTATATTTGCATCGCTTGCT
9_hypothetical	D3	Tb927.9.12340_for	GGGGACAAGTTTGTACAAAAAAGCAGGCTATGGCAACCACGAACCCCC	Tb927.9.12340_rev	GGGGACCACTTTGTACAAGAAAGCTGGGTCTACGCCTCACTAGCCATACGA
9_hypothetical	D4	Tb927.10.14870_for	GGGGACAAGTTTGTACAAAAAAGCAGGCTATGCCTAAAGTAAAGAGTGGGGCT	Tb927.10.14870_rev	GGGGACCACTTTGTACAAGAAAGCTGGGTTCAACAAGATCGATCATCCTCTCCC
9_hypothetical	D5	Tb927.4.2850_for	GGGGACAAGTTTGTACAAAAAAGCAGGCTATGCAAAGCACGAAAGGAACGA	Tb927.4.2850_rev	GGGGACCACTTTGTACAAGAAAGCTGGGTTCAGCCTCTCCACACGGAG
9_hypothetical	D6	Tb927.11.15190_for	GGGGACAAGTTTGTACAAAAAAGCAGGCTATGTCTTTCCAGAAGTCTCCACTTCT	Tb927.11.15190_rev	GGGGACCACTTTGTACAAGAAAGCTGGGTCTAGGCACGGAAGAACTCGTCA
9_hypothetical	D7	Tb927.11.11450_for	GGGGACAAGTTTGTACAAAAAAGCAGGCTATGCAGGCATGGTACCAATTCG	Tb927.11.11450_rev	GGGGACCACTTTGTACAAGAAAGCTGGGTTCATTGGCCACGAAGTCGC
9_hypothetical	D8	Tb927.10.4010_for	GGGGACAAGTTTGTACAAAAAAGCAGGCTATGTCATCAGCACTTCAGGCG	Tb927.10.4010_rev	GGGGACCACTTTGTACAAGAAAGCTGGGTTTATCCAGCAAATGCAGCCGG
9_hypothetical	D9	Tb927.3.1740_for	GGGGACAAGTTTGTACAAAAAAGCAGGCTATGGCTAAGGGTAAAGGTAAAAAGAAGG	Tb927.3.1740_rev	GGGGACCACTTTGTACAAGAAAGCTGGGTTTACTTAACCAACTCAGGGAGAACTGG
9_hypothetical	D10	Tb927.11.16630_for	GGGGACAAGTTTGTACAAAAAAGCAGGCTATGGCGTGTTCTCTCCGTCA	Tb927.11.16630_rev	GGGGACCACTTTGTACAAGAAAGCTGGGTCTAAATATTTGCTGCAGAACCACCTGC
9_hypothetical	D11	Tb927.11.3920_for	GGGGACAAGTTTGTACAAAAAAGCAGGCTATGGGTGAATATCCAGACAACGC	Tb927.11.3920_rev	GGGGACCACTTTGTACAAGAAAGCTGGGTTTACATTCCCTCTTTTTTCCCCTTCG
9_hypothetical	D12	Tb927.10.15670_for	GGGGACAAGTTTGTACAAAAAAGCAGGCTATGTTCTCACAGCTACGAGTTCAC	Tb927.10.15670_rev	GGGGACCACTTTGTACAAGAAAGCTGGGTTTAAAACACTGTCCGTAGTGGTAATATCAT
9_hypothetical	D13	Tb927.10.11090_for	GGGGACAAGTTTGTACAAAAAAGCAGGCTATGAGGGTTACTGGGAAATGTTTTGT	Tb927.10.11090_rev	GGGGACCACTTTGTACAAGAAAGCTGGGTCTAGTTTTCTTCACTGTCTGTGTTGGC
9_hypothetical	D14	Tb927.8.1210_for	GGGGACAAGTTTGTACAAAAAAGCAGGCTATGTCTGCGCCTCTTGCAC	Tb927.8.1210_rev	GGGGACCACTTTGTACAAGAAAGCTGGGTTTAGGCAACGCCGCACTTAC
9_hypothetical	D15	Tb927.4.660_for	GGGGACAAGTTTGTACAAAAAAGCAGGCTATGCCACCTAAAACTGTGAAAGTGA	Tb927.4.660_rev	GGGGACCACTTTGTACAAGAAAGCTGGGTTTAACCCCAAGTAAGTGGGCGA
9_hypothetical	D16	Tb927.5.700_for	GGGGACAAGTTTGTACAAAAAAGCAGGCTATGGGTGGTGGAGCAGCG	Tb927.5.700_rev	GGGGACCACTTTGTACAAGAAAGCTGGGTCTACAGCCCACATGAGTACTCCA
9_hypothetical	D17	Tb927.10.3480_for	GGGGACAAGTTTGTACAAAAAAGCAGGCTATGATCAAAGGGAGTGATCCGCT	Tb927.10.3480_rev	GGGGACCACTTTGTACAAGAAAGCTGGGTTCATTGCCTTCGTCGTTGTTCTT
9_hypothetical	D18	Tb927.10.530_for	GGGGACAAGTTTGTACAAAAAAGCAGGCTATGGGCAATGAACCTGATGAAGC	Tb927.10.530_rev	GGGGACCACTTTGTACAAGAAAGCTGGGTTTAACCTCTCCTATGTGCGGCC
9_hypothetical	D19	Tb927.9.15335_for	GGGGACAAGTTTGTACAAAAAAGCAGGCTATGCCGGTACGACCACCC	Tb927.9.15335_rev	GGGGACCACTTTGTACAAGAAAGCTGGGTTTAGGCCTCCACTTGCACGG
9_hypothetical	D20	Tb927.11.3450_for	GGGGACAAGTTTGTACAAAAAAGCAGGCTATGTGGACCAGGCGTAATGTGT	Tb927.11.3450_rev	GGGGACCACTTTGTACAAGAAAGCTGGGTTCACTTTCCCCACTTGTCAATACCG
9_hypothetical	D21	Tb927.11.5370_for	GGGGACAAGTTTGTACAAAAAAGCAGGCTATGTTAAGAACGAAAAGCACAACCG	Tb927.11.5370_rev	GGGGACCACTTTGTACAAGAAAGCTGGGTCTACTCCATTGCCGTATCCCAGT
9_hypothetical	D22	Tb927.11.690_for	GGGGACAAGTTTGTACAAAAAAGCAGGCTATGGAGGCCGCTGTATACGAG	Tb927.11.690_rev	GGGGACCACTTTGTACAAGAAAGCTGGGTTCAAACAACAGAAGGGAAGAAAAAAAAATC
9_hypothetical	D23	Tb927.10.4100_for	GGGGACAAGTTTGTACAAAAAAGCAGGCTATGCAAACGCCGGTATCTATCAC	Tb927.10.4100_rev	GGGGACCACTTTGTACAAGAAAGCTGGGTCTACACCCCAGGTGAACGCT
9_hypothetical	D24	Tb927.8.6250_for	GGGGACAAGTTTGTACAAAAAAGCAGGCTATGCGCTCCAATGGCGCA	Tb927.8.6250_rev	GGGGACCACTTTGTACAAGAAAGCTGGGTTCACACCTTACTCTTTTCCTTTCGC
9_hypothetical	E1	Tb927.8.3300_for	GGGGACAAGTTTGTACAAAAAAGCAGGCTATGAGGCGGTTATTTGTAACCACTG	Tb927.8.3300_rev	GGGGACCACTTTGTACAAGAAAGCTGGGTTTATCCGCTGAGTCTCGATTGAGC
9_hypothetical	E2	Tb927.11.310_for	GGGGACAAGTTTGTACAAAAAAGCAGGCTATGCTGGTGGCAGTTCGAGA	Tb927.11.310_rev	GGGGACCACTTTGTACAAGAAAGCTGGGTCTAACTGGAAGACTCCACACGGG
9_hypothetical	E3	Tb927.7.3530_for	GGGGACAAGTTTGTACAAAAAAGCAGGCTATGGGGGAGTCCTTCGTCCC	Tb927.7.3530_rev	GGGGACCACTTTGTACAAGAAAGCTGGGTTTACAAAATGTCGGCATCCCCC
9_hypothetical	E4	Tb927.10.12860_for	GGGGACAAGTTTGTACAAAAAAGCAGGCTATGCCACTAGAAACAGTTAATTTTATGAAT	Tb927.10.12860_rev	GGGGACCACTTTGTACAAGAAAGCTGGGTCTAATCTATGGTAAAAATATGTTTGTTGCT
9_hypothetical	E5	Tb927.11.13840_for	GGGGACAAGTTTGTACAAAAAAGCAGGCTATGTCCTTTATCGGCCGCCG	Tb927.11.13840_rev	GGGGACCACTTTGTACAAGAAAGCTGGGTCTACGCCTCGTCTTTTGAGGAAA
9_hypothetical	E6	Tb927.5.2500_for	GGGGACAAGTTTGTACAAAAAAGCAGGCTATGTCTCGACGTGTATTGTGGGA	Tb927.5.2500_rev	GGGGACCACTTTGTACAAGAAAGCTGGGTTCATACACTTTCCCCTTCCTCTTTGC
9_hypothetical	E7	Tb927.3.5590_for	GGGGACAAGTTTGTACAAAAAAGCAGGCTATGTCAGTATGGCAAAAATGGAAGCT	Tb927.3.5590_rev	GGGGACCACTTTGTACAAGAAAGCTGGGTTTACGCCTTTTGTCCTCGCAAC
9_hypothetical	E8	Tb927.10.15170_for	GGGGACAAGTTTGTACAAAAAAGCAGGCTATGTCCTCCCTGACATCCGTC	Tb927.10.15170_rev	GGGGACCACTTTGTACAAGAAAGCTGGGTCTACTGGTTTTCCTCCCTCTGTGG
9_hypothetical	E9	Tb927.7.3820_for	GGGGACAAGTTTGTACAAAAAAGCAGGCTATGGATTTATCGGAGAAGCGACG	Tb927.7.3820_rev	GGGGACCACTTTGTACAAGAAAGCTGGGTCTATACCGAGGCCGGAGGC
9_hypothetical	E10	Tb927.7.7210_for	GGGGACAAGTTTGTACAAAAAAGCAGGCTATGTCATCCCTGCCAAGTGGT	Tb927.7.7210_rev	GGGGACCACTTTGTACAAGAAAGCTGGGTTCACAGTCCGTATATACGGGACTTG
9_hypothetical	E11	Tb927.7.4530_for	GGGGACAAGTTTGTACAAAAAAGCAGGCTATGCCCCGGATCCACATATTCG	Tb927.7.4530_rev	GGGGACCACTTTGTACAAGAAAGCTGGGTTCATCCATTCTTCTCGTTCGTTTTACC
9_hypothetical	E12	Tb927.11.7130_for	GGGGACAAGTTTGTACAAAAAAGCAGGCTATGCAACGGTATCGTGGGAAGC	Tb927.11.7130_rev	GGGGACCACTTTGTACAAGAAAGCTGGGTCTAGCCTGTACAGCAGCACCG
9_hypothetical	E13	Tb927.9.8510_for	GGGGACAAGTTTGTACAAAAAAGCAGGCTATGAAAGATCTGCCTTCACCCGA	Tb927.9.8510_rev	GGGGACCACTTTGTACAAGAAAGCTGGGTTCATCCTTCACTGTTCAGCAGGG
9_hypothetical	E14	Tb927.7.4700_for	GGGGACAAGTTTGTACAAAAAAGCAGGCTATGAGCAAGGAGAAGCTACGCT	Tb927.7.4700_rev	GGGGACCACTTTGTACAAGAAAGCTGGGTTTAATCCTCATTCACGGACCACAGT
9_hypothetical	E15	Tb927.1.4280_for	GGGGACAAGTTTGTACAAAAAAGCAGGCTATGGAGAAGGTAGCAACAACAGC	Tb927.1.4280_rev	GGGGACCACTTTGTACAAGAAAGCTGGGTTCACATCACTGGTTCAACAATCCTAAC
9_hypothetical	E16	Tb927.11.2680_for	GGGGACAAGTTTGTACAAAAAAGCAGGCTATGTTCTGCGAAGATGGCTCCC	Tb927.11.2680_rev	GGGGACCACTTTGTACAAGAAAGCTGGGTTTACCCAGCCAATGCATGCTG
9_hypothetical	E17	Tb927.5.1770_for	GGGGACAAGTTTGTACAAAAAAGCAGGCTATGGACAACCAAGGTGATGCTG	Tb927.5.1770_rev	GGGGACCACTTTGTACAAGAAAGCTGGGTTCAGTGGTTTCCATCCGATCGG
9_hypothetical	E18	Tb927.3.980_for	GGGGACAAGTTTGTACAAAAAAGCAGGCTATGAATAGCGAAGAAGGCCGGC	Tb927.3.980_rev	GGGGACCACTTTGTACAAGAAAGCTGGGTTTATGACTCCCTACTAGGGAACTCACC
9_hypothetical	E19	Tb927.6.1750_for	GGGGACAAGTTTGTACAAAAAAGCAGGCTATGAGTGGAGCAACTGAGAAACTTTT	Tb927.6.1750_rev	GGGGACCACTTTGTACAAGAAAGCTGGGTCTACGAAGAACCTCCAAAGAGTTCC
9_hypothetical	E20	Tb927.7.2790_for	GGGGACAAGTTTGTACAAAAAAGCAGGCTATGGACGGGGATGATGGCA	Tb927.7.2790_rev	GGGGACCACTTTGTACAAGAAAGCTGGGTTCACAGCACGTTTGACGGC
9_hypothetical	E21	Tb927.10.12180_for	GGGGACAAGTTTGTACAAAAAAGCAGGCTATGGGAAGAAATAAGTCCCATGCG	Tb927.10.12180_rev	GGGGACCACTTTGTACAAGAAAGCTGGGTTCAAAGCTTGAAAAACTTAATACTCAGCTT
9_hypothetical	E22	Tb927.7.2490_for	GGGGACAAGTTTGTACAAAAAAGCAGGCTATGCTTCAAAAGGCAGGCCG	Tb927.7.2490_rev	GGGGACCACTTTGTACAAGAAAGCTGGGTTCAAACAGCGCTGTTGCCG
9_hypothetical	E23	Tb927.9.3870_for	GGGGACAAGTTTGTACAAAAAAGCAGGCTATGAAAAAGAGGAGGCCAATACCCA	Tb927.9.3870_rev	GGGGACCACTTTGTACAAGAAAGCTGGGTCTACGACATATCATTCGCAGTCGC
9_hypothetical	E24	Tb927.10.15640_for	GGGGACAAGTTTGTACAAAAAAGCAGGCTATGAGCTCTACATTTATTATATCCGCTTCT	Tb927.10.15640_rev	GGGGACCACTTTGTACAAGAAAGCTGGGTCTATTGGTTTTTCTTCCTTTCCACATAGAG
9_hypothetical	F1	Tb927.9.1980_for	GGGGACAAGTTTGTACAAAAAAGCAGGCTATGGTGGTTGTGGACAATGCTC	Tb927.9.1980_rev	GGGGACCACTTTGTACAAGAAAGCTGGGTCTACCGACTCGTCATGTTGTATGAC
9_hypothetical	F2	Tb927.11.15730_for	GGGGACAAGTTTGTACAAAAAAGCAGGCTATGGACAGCAGGTGGGCG	Tb927.11.15730_rev	GGGGACCACTTTGTACAAGAAAGCTGGGTTTACACCACACTCGACCGCC
9_hypothetical	F3	Tb927.8.3980_for	GGGGACAAGTTTGTACAAAAAAGCAGGCTATGGATAGTGTCGCGGAGCC	Tb927.8.3980_rev	GGGGACCACTTTGTACAAGAAAGCTGGGTCTAGCGTTTGCTTCGACAATCGC
9_hypothetical	F4	Tb927.8.8010_for	GGGGACAAGTTTGTACAAAAAAGCAGGCTATGATGAGTGCCAATAACTGCACC	Tb927.8.8010_rev	GGGGACCACTTTGTACAAGAAAGCTGGGTTCAAGTTGGGTTGGCAGCAG
9_hypothetical	F5	Tb927.9.9190_for	GGGGACAAGTTTGTACAAAAAAGCAGGCTATGACGACTATATCCGCTTTAGAAAAACA	Tb927.9.9190_rev	GGGGACCACTTTGTACAAGAAAGCTGGGTCTAAAACAGTTTCGATAGCAACTCAGACC
9_hypothetical	F6	Tb927.10.1980_for	GGGGACAAGTTTGTACAAAAAAGCAGGCTATGACCGATAATAAGGTTCTCTACGTTGG	Tb927.10.1980_rev	GGGGACCACTTTGTACAAGAAAGCTGGGTTTACGCTGCTACTGCAGTCCT
9_hypothetical	F7	Tb927.4.2300_for	GGGGACAAGTTTGTACAAAAAAGCAGGCTATGTCTGCGGTTTCTTCTCCTAAGC	Tb927.4.2300_rev	GGGGACCACTTTGTACAAGAAAGCTGGGTTTATACATCATCGTCCCATCCCCAA
9_hypothetical	F8	Tb927.3.5120_for	GGGGACAAGTTTGTACAAAAAAGCAGGCTATGTGGAAAACTAAGGGGCGTG	Tb927.3.5120_rev	GGGGACCACTTTGTACAAGAAAGCTGGGTTTATGAATTTTGCGGCGTCTCCA
9_hypothetical	F9	Tb927.7.5300_for	GGGGACAAGTTTGTACAAAAAAGCAGGCTATGAATGTCGATGGTACTGTCCAAC	Tb927.7.5300_rev	GGGGACCACTTTGTACAAGAAAGCTGGGTTTACATTGTTTCAGCCAACATCAAAGC
9_hypothetical	F10	Tb927.11.2790_for	GGGGACAAGTTTGTACAAAAAAGCAGGCTATGTACTACCGAACGCAGCAGA	Tb927.11.2790_rev	GGGGACCACTTTGTACAAGAAAGCTGGGTTCACTCTTTCGTCTCTTTTTCATTTGGA
9_hypothetical	F11	Tb927.10.11880_for	GGGGACAAGTTTGTACAAAAAAGCAGGCTATGACAATAAGCAGCCCGTGC	Tb927.10.11880_rev	GGGGACCACTTTGTACAAGAAAGCTGGGTCTACTCCCAGGTTGGGATTAGCG
9_hypothetical	F12	Tb927.6.2090_for	GGGGACAAGTTTGTACAAAAAAGCAGGCTATGGGTTTGTGCAATTCTAAAAGTTTTGA	Tb927.6.2090_rev	GGGGACCACTTTGTACAAGAAAGCTGGGTTTAGGCCGCCTTGTTCCAGG
9_hypothetical	F13	Tb927.8.1950_for	GGGGACAAGTTTGTACAAAAAAGCAGGCTATGCTCTCGTGGAATCCCGA	Tb927.8.1950_rev	GGGGACCACTTTGTACAAGAAAGCTGGGTTTAAGCTGGGATGAGGCGCT
9_hypothetical	F14	Tb927.5.2310_for	GGGGACAAGTTTGTACAAAAAAGCAGGCTATGCGCGTGCGAACAAAGC	Tb927.5.2310_rev	GGGGACCACTTTGTACAAGAAAGCTGGGTCTACGGGCTATCAACTCTGTTCTCC
9_hypothetical	F15	Tb927.6.3990_for	GGGGACAAGTTTGTACAAAAAAGCAGGCTATGTCCGACCATGTGGTATTTGGA	Tb927.6.3990_rev	GGGGACCACTTTGTACAAGAAAGCTGGGTTCACTCATCATTTACCTCCCCTTCC
9_hypothetical	F16	Tb927.4.2680_for	GGGGACAAGTTTGTACAAAAAAGCAGGCTATGCAACCACCTTCACCAACTG	Tb927.4.2680_rev	GGGGACCACTTTGTACAAGAAAGCTGGGTTTAGGATTGCTTGGGGTTGTAGAGC
9_hypothetical	F17	Tb927.11.3480_for	GGGGACAAGTTTGTACAAAAAAGCAGGCTATGCAGCAGACCGAAAAGCG	Tb927.11.3480_rev	GGGGACCACTTTGTACAAGAAAGCTGGGTTCACTGTCCAATGACATTGCCAC
9_hypothetical	F18	Tb927.11.14820_for	GGGGACAAGTTTGTACAAAAAAGCAGGCTATGGCTGCTCTGCGTAGTGT	Tb927.11.14820_rev	GGGGACCACTTTGTACAAGAAAGCTGGGTTTACTCTTTACGCAGCCTTTTATAGTCTC
9_hypothetical	F19	Tb927.10.7810_for	GGGGACAAGTTTGTACAAAAAAGCAGGCTATGAAGACACTACAAAGCTCCTCCC	Tb927.10.7810_rev	GGGGACCACTTTGTACAAGAAAGCTGGGTCTACTCCCTTTTTGCTTTCTTACTCGG
9_hypothetical	F20	Tb927.1.2710_for	GGGGACAAGTTTGTACAAAAAAGCAGGCTATGGACTCGCTACGCTCGC	Tb927.1.2710_rev	GGGGACCACTTTGTACAAGAAAGCTGGGTTTAATTTTTTTCTTTTGTTGGATCTACAGA
9_hypothetical	F21	Tb927.10.13380_for	GGGGACAAGTTTGTACAAAAAAGCAGGCTATGAAAGGAGTATTTTGTGAAAGCGGT	Tb927.10.13380_rev	GGGGACCACTTTGTACAAGAAAGCTGGGTTCACACGAGGCTGCGTAATCG
9_hypothetical	F22	Tb927.8.2710_for	GGGGACAAGTTTGTACAAAAAAGCAGGCTATGGCCACTGTCACGGAGG	Tb927.8.2710_rev	GGGGACCACTTTGTACAAGAAAGCTGGGTTTAGGAAGTCCCCCCCCACG
9_hypothetical	F23	Tb927.11.6580_for	GGGGACAAGTTTGTACAAAAAAGCAGGCTATGGAAGAAATCATCGAGTTGTGTGC	Tb927.11.6580_rev	GGGGACCACTTTGTACAAGAAAGCTGGGTTCAGCGGGTATGCTGAACAATAC
9_hypothetical	F24	Tb927.7.910_for	GGGGACAAGTTTGTACAAAAAAGCAGGCTATGAGAAGCTGCTCGCGTCA	Tb927.7.910_rev	GGGGACCACTTTGTACAAGAAAGCTGGGTTTAAACTTGGCCACTTCCGCC
9_hypothetical	G1	Tb927.2.5200_for	GGGGACAAGTTTGTACAAAAAAGCAGGCTATGGATGAGCAAAACAGTGAGAGC	Tb927.2.5200_rev	GGGGACCACTTTGTACAAGAAAGCTGGGTCTAATTGTCGGAATCGCTATCGCT
9_hypothetical	G2	Tb927.10.14860_for	GGGGACAAGTTTGTACAAAAAAGCAGGCTATGCTTCGTCGAGCATGCC	Tb927.10.14860_rev	GGGGACCACTTTGTACAAGAAAGCTGGGTCTAAATCTGCGAGACAACCACATTTC
9_hypothetical	G3	Tb927.10.590_for	GGGGACAAGTTTGTACAAAAAAGCAGGCTATGTCCAGAAACCCCCCTGATAG	Tb927.10.590_rev	GGGGACCACTTTGTACAAGAAAGCTGGGTTTACTCATCATAAGGTAAATACTGCAGTAA
9_hypothetical	G4	Tb927.11.340_for	GGGGACAAGTTTGTACAAAAAAGCAGGCTATGACTATGTGGTTTCCTGCCGA	Tb927.11.340_rev	GGGGACCACTTTGTACAAGAAAGCTGGGTCTAAGCATCTTCTATATCATATGGTGTTTT
9_hypothetical	G5	Tb927.10.6110_for	GGGGACAAGTTTGTACAAAAAAGCAGGCTATGAATCGGGGTAAGGGGGG	Tb927.10.6110_rev	GGGGACCACTTTGTACAAGAAAGCTGGGTCTATACCCTACCAGATTCACGCAGT
9_hypothetical	G6	Tb927.10.1320_for	GGGGACAAGTTTGTACAAAAAAGCAGGCTATGGCCTTCCTCCTGAGGGC	Tb927.10.1320_rev	GGGGACCACTTTGTACAAGAAAGCTGGGTCTACGACAGGCGAGAGGCG
9_hypothetical	G7	Tb927.8.8130_for	GGGGACAAGTTTGTACAAAAAAGCAGGCTATGGTAACGCAGCAGCGAATG	Tb927.8.8130_rev	GGGGACCACTTTGTACAAGAAAGCTGGGTTTAATCCCATGGAGAAAAGAAGGAGAAAC
9_hypothetical	G8	Tb927.1.4340_for	GGGGACAAGTTTGTACAAAAAAGCAGGCTATGAGTAGTACTGAAAGCAGCGGT	Tb927.1.4340_rev	GGGGACCACTTTGTACAAGAAAGCTGGGTCTAACCTCTCACTACGGCACTACT
9_hypothetical	G9	Tb927.5.4060_for	GGGGACAAGTTTGTACAAAAAAGCAGGCTATGTTACAAGGTGTTGATCACTTTCTTGC	Tb927.5.4060_rev	GGGGACCACTTTGTACAAGAAAGCTGGGTCTATACTGCAGGTGTTTTCCTCAAACA
9_hypothetical	G10	Tb927.10.1190_for	GGGGACAAGTTTGTACAAAAAAGCAGGCTATGGCTTCCCACAGTAGAGGATG	Tb927.10.1190_rev	GGGGACCACTTTGTACAAGAAAGCTGGGTTTATTTCTTCCGGGTCCGACTCT
9_hypothetical	G11	Tb927.11.1170_for	GGGGACAAGTTTGTACAAAAAAGCAGGCTATGAAGTTTTGGGTGAAGGCAATAACG	Tb927.11.1170_rev	GGGGACCACTTTGTACAAGAAAGCTGGGTTCACGGTTCTGTCGTTAGTGGT
9_hypothetical	G12	Tb927.10.14570_for	GGGGACAAGTTTGTACAAAAAAGCAGGCTATGGAGAATTCCAGTTCGTTCGAC	Tb927.10.14570_rev	GGGGACCACTTTGTACAAGAAAGCTGGGTCTACTCTACATCCAAGCCCCATCC
9_hypothetical	G13	Tb927.7.7320_for	GGGGACAAGTTTGTACAAAAAAGCAGGCTATGTACCCACAGCAACAAATGAGC	Tb927.7.7320_rev	GGGGACCACTTTGTACAAGAAAGCTGGGTCTACACTTCATTCAGCATCCTATCGATG
9_hypothetical	G14	Tb927.10.7370_for	GGGGACAAGTTTGTACAAAAAAGCAGGCTATGGCAGTTGACTGTTTGGCG	Tb927.10.7370_rev	GGGGACCACTTTGTACAAGAAAGCTGGGTTCAAACTTCAGCGACGACAGC
9_hypothetical	G15	Tb927.6.3710_for	GGGGACAAGTTTGTACAAAAAAGCAGGCTATGCAGGAGCCCGAAGTAGC	Tb927.6.3710_rev	GGGGACCACTTTGTACAAGAAAGCTGGGTTTAAGACACGCGGGTCCGG
9_hypothetical	G16	Tb927.4.1480_for	GGGGACAAGTTTGTACAAAAAAGCAGGCTATGGCAGGTGGAGCTGCA	Tb927.4.1480_rev	GGGGACCACTTTGTACAAGAAAGCTGGGTTTATTCTGAGTCACTACTCAGGTCAATCA

9_hypothetical	G17	Tb927.9.9860_for	GGGGACAAGTTTGTACAAAAAAGCAGGCTATGAGAGTGCTTGCAAATGAAATCAGG	Tb927.9.9860_rev	GGGGACCACTTTGTACAAGAAAGCTGGGTTCACAAGTCTCCCTTGACAGCA
9_hypothetical	G18	Tb927.11.13320_for	GGGGACAAGTTTGTACAAAAAAGCAGGCTATGCGAATTGTCCGCATTCAGT	Tb927.11.13320_rev	GGGGACCACTTTGTACAAGAAAGCTGGGTTCATCTGCCGTGCGTTAAACG
9_hypothetical	G19	Tb927.6.2380_for	GGGGACAAGTTTGTACAAAAAAGCAGGCTATGCTTAGCATCACACGGTGC	Tb927.6.2380_rev	GGGGACCACTTTGTACAAGAAAGCTGGGTTCACAATTCACCAAACTGTTCCTGG
9_hypothetical	G20	Tb927.9.6580_for	GGGGACAAGTTTGTACAAAAAAGCAGGCTATGGTTCGTGTGAAGGAAAAGGC	Tb927.9.6580_rev	GGGGACCACTTTGTACAAGAAAGCTGGGTTCACGCACAAACACCGGG
9_hypothetical	G21	Tb927.10.2760_for	GGGGACAAGTTTGTACAAAAAAGCAGGCTATGGAAGGGCGCCACGGC	Tb927.10.2760_rev	GGGGACCACTTTGTACAAGAAAGCTGGGTTTAATATTGACGCTCCAACTCCAGTAGT
9_hypothetical	G22	Tb927.8.1300_for	GGGGACAAGTTTGTACAAAAAAGCAGGCTATGAGTCGAAGAAATTCGGCTTCC	Tb927.8.1300_rev	GGGGACCACTTTGTACAAGAAAGCTGGGTTTACGATGAAGGCCCTGTGGG
9_hypothetical	G23	Tb927.11.15120_for	GGGGACAAGTTTGTACAAAAAAGCAGGCTATGAGCTCATCAGAGTCAGGGAG	Tb927.11.15120_rev	GGGGACCACTTTGTACAAGAAAGCTGGGTCTAAAAATCTAGCCAGCTGGCACC
9_hypothetical	G24	Tb927.10.250_for	GGGGACAAGTTTGTACAAAAAAGCAGGCTATGTCAGAACTGACGGCCGG	Tb927.10.250_rev	GGGGACCACTTTGTACAAGAAAGCTGGGTTCACATTTTGTGATAAAGCAGCTTTCC
9_hypothetical	H1	Tb927.7.5200_for	GGGGACAAGTTTGTACAAAAAAGCAGGCTATGGAAGATGACATTATACAAGGTCGC	Tb927.7.5200_rev	GGGGACCACTTTGTACAAGAAAGCTGGGTCTATGCAACCGGCTGGTCTAAC
9_hypothetical	H2	Tb927.11.7570_for	GGGGACAAGTTTGTACAAAAAAGCAGGCTATGCCGAAGGAGGAGCAGT	Tb927.11.7570_rev	GGGGACCACTTTGTACAAGAAAGCTGGGTTTACACACCACAGATGAGTTGAGTAGC
9_hypothetical	H3	Tb927.5.770_for	GGGGACAAGTTTGTACAAAAAAGCAGGCTATGATGCATCGCACTGCAGC	Tb927.5.770_rev	GGGGACCACTTTGTACAAGAAAGCTGGGTTTATGTGTACTTGTGATATACGTCATTCCC
9_hypothetical	H4	Tb927.10.11420_for	GGGGACAAGTTTGTACAAAAAAGCAGGCTATGCGAGGCCGATTCTTGTC	Tb927.10.11420_rev	GGGGACCACTTTGTACAAGAAAGCTGGGTTTAAATATGAAGCAGCTTCGCAGCC
9_hypothetical	H5	Tb927.9.10550_for	GGGGACAAGTTTGTACAAAAAAGCAGGCTATGAGTCGTAGCGTAGACACAAGT	Tb927.9.10550_rev	GGGGACCACTTTGTACAAGAAAGCTGGGTTTAGAAGTATGAGCTCCTCACATTGTCA
9_hypothetical	H6	Tb927.11.13110_for	GGGGACAAGTTTGTACAAAAAAGCAGGCTATGGGGTCTCCAGTGAAAGACAC	Tb927.11.13110_rev	GGGGACCACTTTGTACAAGAAAGCTGGGTTCATATGAAGTACGCGGCAGACA
9_hypothetical	H7	Tb927.7.6750_for	GGGGACAAGTTTGTACAAAAAAGCAGGCTATGAGCTCCTACTGTGATATTTGCCG	Tb927.7.6750_rev	GGGGACCACTTTGTACAAGAAAGCTGGGTTCACAACCCCGGACATGCC
9_hypothetical	H8	Tb927.9.2270_for	GGGGACAAGTTTGTACAAAAAAGCAGGCTATGTTACGGCGCTCCTTCATG	Tb927.9.2270_rev	GGGGACCACTTTGTACAAGAAAGCTGGGTTCAGTGCAGAGCAGCCGC
9_hypothetical	H9	Tb927.2.4140_for	GGGGACAAGTTTGTACAAAAAAGCAGGCTATGAAGCGAGAGCGCTCATTTT	Tb927.2.4140_rev	GGGGACCACTTTGTACAAGAAAGCTGGGTTTAAAAGCGTTTCATTTGTCCATGACC
9_hypothetical	H10	Tb927.11.3970_for	GGGGACAAGTTTGTACAAAAAAGCAGGCTATGGCAGCTAACCGAAGTATATTTCTTT	Tb927.11.3970_rev	GGGGACCACTTTGTACAAGAAAGCTGGGTCTATTTTCGTTCCCTCTTTTTCGTTGC
9_hypothetical	H11	Tb927.3.2660_for	GGGGACAAGTTTGTACAAAAAAGCAGGCTATGGGATTTGTCGACCGTGGT	Tb927.3.2660_rev	GGGGACCACTTTGTACAAGAAAGCTGGGTCTACTCAGAAGGGGTTTCACTTACGC
9_hypothetical	H12	Tb927.10.7890_for	GGGGACAAGTTTGTACAAAAAAGCAGGCTATGTTGCGTCATGGTTCCCAC	Tb927.10.7890_rev	GGGGACCACTTTGTACAAGAAAGCTGGGTCTATATGGCCACATTCAGGTACCCA
9_hypothetical	H13	Tb927.11.3960_for	GGGGACAAGTTTGTACAAAAAAGCAGGCTATGAATGATCGGCCACATTTTTATGAAG	Tb927.11.3960_rev	GGGGACCACTTTGTACAAGAAAGCTGGGTTCACAATGAGATAAGGTAAGAGTCATTGAG
9_hypothetical	H14	Tb927.7.5250_for	GGGGACAAGTTTGTACAAAAAAGCAGGCTATGGGCCATTGTTGTGCCAC	Tb927.7.5250_rev	GGGGACCACTTTGTACAAGAAAGCTGGGTTTATGATTGACAGTGTGCCTTTCCG
9_hypothetical	H15	Tb927.6.630_for	GGGGACAAGTTTGTACAAAAAAGCAGGCTATGATGGAGTTGTTACAACTACCCAC	Tb927.6.630_rev	GGGGACCACTTTGTACAAGAAAGCTGGGTTTATCGGCAGCGCAGCGG
9_hypothetical	H16	Tb927.9.1730_for	GGGGACAAGTTTGTACAAAAAAGCAGGCTATGGCACGAAGTGAGGGGAA	Tb927.9.1730_rev	GGGGACCACTTTGTACAAGAAAGCTGGGTTTACGCATCCTCTCGAAGGCT
9_hypothetical	H17	Tb927.10.6160_for	GGGGACAAGTTTGTACAAAAAAGCAGGCTATGTGCACCTCTTTTGACGCC	Tb927.10.6160_rev	GGGGACCACTTTGTACAAGAAAGCTGGGTTCATTCACGTCGTTCCTGCG
9_hypothetical	H18	Tb927.1.4520_for	GGGGACAAGTTTGTACAAAAAAGCAGGCTATGTACAGCGGGGATCGTGC	Tb927.1.4520_rev	GGGGACCACTTTGTACAAGAAAGCTGGGTTCACGACTTCTCTACTTCCGCC
9_hypothetical	H19	Tb927.5.3870_for	GGGGACAAGTTTGTACAAAAAAGCAGGCTATGAGGCGCTGCGGGAAT	Tb927.5.3870_rev	GGGGACCACTTTGTACAAGAAAGCTGGGTTTAAGGATACGTCGTAACCAAACTCCC
9_hypothetical	H20	Tb927.11.210_for	GGGGACAAGTTTGTACAAAAAAGCAGGCTATGGATGTTGATAGTGACAGGCGC	Tb927.11.210_rev	GGGGACCACTTTGTACAAGAAAGCTGGGTCTAGCCCCCAGATCTCTCAGGT
9_hypothetical	H21	Tb927.10.13610_for	GGGGACAAGTTTGTACAAAAAAGCAGGCTATGCTGTCGTCCCGTAGCA	Tb927.10.13610_rev	GGGGACCACTTTGTACAAGAAAGCTGGGTCTAAAACCGCCTTCGCGGA
9_hypothetical	H22	Tb927.11.14850_for	GGGGACAAGTTTGTACAAAAAAGCAGGCTATGTCTGATCAGGACGGCAACC	Tb927.11.14850_rev	GGGGACCACTTTGTACAAGAAAGCTGGGTTTAGCGCTCAAACAGGAAGACG
9_hypothetical	H23	Tb927.9.3860_for	GGGGACAAGTTTGTACAAAAAAGCAGGCTATGGGAGGGACGTCTACGAAG	Tb927.9.3860_rev	GGGGACCACTTTGTACAAGAAAGCTGGGTTTAGTGAGGGAATATCCCCAACGT
9_hypothetical	H24	Tb927.11.8240_for	GGGGACAAGTTTGTACAAAAAAGCAGGCTATGTCTGATGTTCCGGAGCTTCC	Tb927.11.8240_rev	GGGGACCACTTTGTACAAGAAAGCTGGGTCTACACAAAATATGTTGTAGTCAATCGATC
9_hypothetical	I1	Tb927.10.11570_for	GGGGACAAGTTTGTACAAAAAAGCAGGCTATGTCGTCGCGCTTAACGC	Tb927.10.11570_rev	GGGGACCACTTTGTACAAGAAAGCTGGGTCTAACCGGCAATATTACGATCTTCAGA
9_hypothetical	I2	Tb927.7.4640_for	GGGGACAAGTTTGTACAAAAAAGCAGGCTATGCGATTGGGGATTCAACAGAAT	Tb927.7.4640_rev	GGGGACCACTTTGTACAAGAAAGCTGGGTTTACATGGCGCGATGCACG
9_hypothetical	I3	Tb927.9.1530_for	GGGGACAAGTTTGTACAAAAAAGCAGGCTATGCCACCACGTGTAACTGAGA	Tb927.9.1530_rev	GGGGACCACTTTGTACAAGAAAGCTGGGTTCACCACACTGCCCGATCC
9_hypothetical	I4	Tb927.7.3570_for	GGGGACAAGTTTGTACAAAAAAGCAGGCTATGGAGGACGAGTGGAACACC	Tb927.7.3570_rev	GGGGACCACTTTGTACAAGAAAGCTGGGTCTAGTTTATCCCAAGTCGCTTCGC
9_hypothetical	I5	Tb927.11.12870_for	GGGGACAAGTTTGTACAAAAAAGCAGGCTATGGAGAGTAAAACGTGGTTCGGC	Tb927.11.12870_rev	GGGGACCACTTTGTACAAGAAAGCTGGGTCTAAACAATGCCTGCAATGCGC
9_hypothetical	I6	Tb927.5.1540_for	GGGGACAAGTTTGTACAAAAAAGCAGGCTATGAGTAAGAAGCCGAATCCGCA	Tb927.5.1540_rev	GGGGACCACTTTGTACAAGAAAGCTGGGTCTATACATTGGGGAATTCTATGTCACCTTC
9_hypothetical	I7	Tb927.7.5730_for	GGGGACAAGTTTGTACAAAAAAGCAGGCTATGATGGAGGGAGCAACGCA	Tb927.7.5730_rev	GGGGACCACTTTGTACAAGAAAGCTGGGTTCAACTCCTTTTCCGTTTTTGTCTAGA
9_hypothetical	I8	Tb927.5.2320_for	GGGGACAAGTTTGTACAAAAAAGCAGGCTATGTTGTGCGCGTTGGATGA	Tb927.5.2320_rev	GGGGACCACTTTGTACAAGAAAGCTGGGTTCAAGCGGTTGTCCTCTTCCT
9_hypothetical	I9	Tb927.8.3010_for	GGGGACAAGTTTGTACAAAAAAGCAGGCTATGAAATATAGTGGTGACACTCCTGTGG	Tb927.8.3010_rev	GGGGACCACTTTGTACAAGAAAGCTGGGTTTACGTGGACTTCATATAGCTCAGCC
9_hypothetical	I10	Tb927.9.15070_for	GGGGACAAGTTTGTACAAAAAAGCAGGCTATGCCGTGGCAGTGGCTC	Tb927.9.15070_rev	GGGGACCACTTTGTACAAGAAAGCTGGGTTTAACTAGGGTTATTGGCTAAATACTCCGC
9_hypothetical	I11	Tb927.7.1100_for	GGGGACAAGTTTGTACAAAAAAGCAGGCTATGAATACCACCGGGGAGCC	Tb927.7.1100_rev	GGGGACCACTTTGTACAAGAAAGCTGGGTCTATAAAGGTTCAGGTGCTACACCAC
9_hypothetical	I12	Tb927.2.3750_for	GGGGACAAGTTTGTACAAAAAAGCAGGCTATGAGTCTTCGCAGATTCTACAATGAG	Tb927.2.3750_rev	GGGGACCACTTTGTACAAGAAAGCTGGGTTCAAGTGAAATAAGTCGAATCGTCTCG
9_hypothetical	I13	Tb927.6.1120_for	GGGGACAAGTTTGTACAAAAAAGCAGGCTATGAGTGATGTTCTCGCTATACTCGA	Tb927.6.1120_rev	GGGGACCACTTTGTACAAGAAAGCTGGGTTCAGCTAAGCGCAAGCAAGG
9_hypothetical	I14	Tb927.10.13660_for	GGGGACAAGTTTGTACAAAAAAGCAGGCTATGGTATTTGGCGTAAGGCGATG	Tb927.10.13660_rev	GGGGACCACTTTGTACAAGAAAGCTGGGTCTACTTGGGAGGGTTTAGAAGCTTCA
9_hypothetical	I15	Tb927.5.3880_for	GGGGACAAGTTTGTACAAAAAAGCAGGCTATGGTCGGCAGCGCATCG	Tb927.5.3880_rev	GGGGACCACTTTGTACAAGAAAGCTGGGTTCATCTCCCTCCCGTATCCGC
9_hypothetical	I16	Tb927.7.6730_for	GGGGACAAGTTTGTACAAAAAAGCAGGCTATGGAGTTTGAGGCCGGGC	Tb927.7.6730_rev	GGGGACCACTTTGTACAAGAAAGCTGGGTCTATGGTGCGTACGTGCTCC
9_hypothetical	I17	Tb927.10.8080_for	GGGGACAAGTTTGTACAAAAAAGCAGGCTATGAGTGAGTTGGAGCTGAGTGA	Tb927.10.8080_rev	GGGGACCACTTTGTACAAGAAAGCTGGGTCTACGCATCCTCAAGTACAAGCC
9_hypothetical	I18	Tb927.11.930_for	GGGGACAAGTTTGTACAAAAAAGCAGGCTATGCCTCACAAGGAGGCTGA	Tb927.11.930_rev	GGGGACCACTTTGTACAAGAAAGCTGGGTCTACACCTCCTCTGCTTCTTCAACA
9_hypothetical	I19	Tb927.7.4920_for	GGGGACAAGTTTGTACAAAAAAGCAGGCTATGTGGATCGTGTTTATGAATGGTCA	Tb927.7.4920_rev	GGGGACCACTTTGTACAAGAAAGCTGGGTCTACAGGAATGCTTCCAGGTCGA
9_hypothetical	I20	Tb927.9.5380_for	GGGGACAAGTTTGTACAAAAAAGCAGGCTATGATGACAAAAACCTATGGGTCGAAA	Tb927.9.5380_rev	GGGGACCACTTTGTACAAGAAAGCTGGGTTCACTTCGCCATAAACAGAAGGTATTG
9_hypothetical	I21	Tb927.4.1150_for	GGGGACAAGTTTGTACAAAAAAGCAGGCTATGACATGTAGGAGTGAGGAAACTAACT	Tb927.4.1150_rev	GGGGACCACTTTGTACAAGAAAGCTGGGTTTACTCCAACGTCCATCCCTTCC
9_hypothetical	I22	Tb927.8.3840_for	GGGGACAAGTTTGTACAAAAAAGCAGGCTATGCCCGAGGCGCATATTG	Tb927.8.3840_rev	GGGGACCACTTTGTACAAGAAAGCTGGGTCTAACTACCAAAGTTGAAAGTAAGCGTG
9_hypothetical	I23	Tb927.11.16460_for	GGGGACAAGTTTGTACAAAAAAGCAGGCTATGCGCTGGACCACTCTGT	Tb927.11.16460_rev	GGGGACCACTTTGTACAAGAAAGCTGGGTTTAATAGAAGGAAAACCGGGGCTCT
9_hypothetical	I24	Tb927.11.15310_for	GGGGACAAGTTTGTACAAAAAAGCAGGCTATGGACCCGACCTTACGCA	Tb927.11.15310_rev	GGGGACCACTTTGTACAAGAAAGCTGGGTTCAACTATCAGCCGCTTCCGT
9_hypothetical	J1	Tb927.10.11550_for	GGGGACAAGTTTGTACAAAAAAGCAGGCTATGGACAATCAGCGCAATACAGAA	Tb927.10.11550_rev	GGGGACCACTTTGTACAAGAAAGCTGGGTCTACATCAACGTAAACTCATCCTCTTCG
9_hypothetical	J2	Tb927.5.3900_for	GGGGACAAGTTTGTACAAAAAAGCAGGCTATGGGCAAGGGTCGCGAC	Tb927.5.3900_rev	GGGGACCACTTTGTACAAGAAAGCTGGGTTCAACTATTTTCATCTCCTTCACCTTTTTC
9_hypothetical	J3	Tb927.10.6590_for	GGGGACAAGTTTGTACAAAAAAGCAGGCTATGCCCTCCTCTCAGTGGGT	Tb927.10.6590_rev	GGGGACCACTTTGTACAAGAAAGCTGGGTTCAGGGCTTCAACCTACTTATCGC
9_hypothetical	J4	Tb927.7.3010_for	GGGGACAAGTTTGTACAAAAAAGCAGGCTATGAAGTTCCACCTTCAAAGCGT	Tb927.7.3010_rev	GGGGACCACTTTGTACAAGAAAGCTGGGTTCATTGAGCAACCAGCCAACC
9_hypothetical	J5	Tb927.3.2130_for	GGGGACAAGTTTGTACAAAAAAGCAGGCTATGCATCACGCTCGTGGGC	Tb927.3.2130_rev	GGGGACCACTTTGTACAAGAAAGCTGGGTTCACTCACAGGACAGACGAAAAATG
9_hypothetical	J6	Tb927.8.5050_for	GGGGACAAGTTTGTACAAAAAAGCAGGCTATGGGTGCCTGTGTTTCAGATG	Tb927.8.5050_rev	GGGGACCACTTTGTACAAGAAAGCTGGGTTTATTCAACAGGTCTCGAGATTGTATCCA
9_hypothetical	J7	Tb927.7.5320_for	GGGGACAAGTTTGTACAAAAAAGCAGGCTATGGAAACACAGGTACCTCAGAATGT	Tb927.7.5320_rev	GGGGACCACTTTGTACAAGAAAGCTGGGTCTACCATCGTCTAATGAACTTAATTTGCAT
9_hypothetical	J8	Tb927.7.4990_for	GGGGACAAGTTTGTACAAAAAAGCAGGCTATGTCTGGTTCTTTCCGAAAAATATGTGA	Tb927.7.4990_rev	GGGGACCACTTTGTACAAGAAAGCTGGGTTCACCGCTCGGCTAGAAGC
9_hypothetical	J9	Tb927.10.7130_for	GGGGACAAGTTTGTACAAAAAAGCAGGCTATGGCTTCCGACTTGCAGAAG	Tb927.10.7130_rev	GGGGACCACTTTGTACAAGAAAGCTGGGTTTAGCGCAGCAACTTCCTCAC
9_hypothetical	J10	Tb927.7.5290_for	GGGGACAAGTTTGTACAAAAAAGCAGGCTATGTCTCACAGCCAGAACAGCA	Tb927.7.5290_rev	GGGGACCACTTTGTACAAGAAAGCTGGGTTCAGTCATCATTCTGCACCAGC
9_hypothetical	J11	Tb927.7.1650_for	GGGGACAAGTTTGTACAAAAAAGCAGGCTATGGGCAAAAGACGGAGGTCC	Tb927.7.1650_rev	GGGGACCACTTTGTACAAGAAAGCTGGGTCTATCCTTCGCCGTCACTCTTG
9_hypothetical	J12	Tb927.10.9100_for	GGGGACAAGTTTGTACAAAAAAGCAGGCTATGGCAAAAACACTTAGCGATGC	Tb927.10.9100_rev	GGGGACCACTTTGTACAAGAAAGCTGGGTTCATTCCAATCTGCCTTTCCTCG
9_hypothetical	J13	Tb927.10.1960_for	GGGGACAAGTTTGTACAAAAAAGCAGGCTATGTCGAACGGGTATAAGCGC	Tb927.10.1960_rev	GGGGACCACTTTGTACAAGAAAGCTGGGTTCAAAATGCTACAGAAGACATACGTTGC
9_hypothetical	J14	Tb927.9.2070_for	GGGGACAAGTTTGTACAAAAAAGCAGGCTATGAGTCGACAGACGAGGCG	Tb927.9.2070_rev	GGGGACCACTTTGTACAAGAAAGCTGGGTTCATGAGGGTTTGGGCATTCG
9_hypothetical	J15	Tb927.8.6860_for	GGGGACAAGTTTGTACAAAAAAGCAGGCTATGGGTACATCTGGTTCTACGATAGATG	Tb927.8.6860_rev	GGGGACCACTTTGTACAAGAAAGCTGGGTCTATCGAAACCGGAGCATTTTGATGG
9_hypothetical	J16	Tb927.2.2400_for	GGGGACAAGTTTGTACAAAAAAGCAGGCTATGCATTATGCCTCGCGTGG	Tb927.2.2400_rev	GGGGACCACTTTGTACAAGAAAGCTGGGTTTAAATCTTACCTTTGCCACACCATCG
9_hypothetical	J17	Tb927.10.2930_for	GGGGACAAGTTTGTACAAAAAAGCAGGCTATGGGGAATTGCTTGTGCTGC	Tb927.10.2930_rev	GGGGACCACTTTGTACAAGAAAGCTGGGTTTACATCAAACTTTCGTCACCATCTTCT
9_hypothetical	J18	Tb927.8.880_for	GGGGACAAGTTTGTACAAAAAAGCAGGCTATGACTATCAACATCTTTGCTGAACTGG	Tb927.8.880_rev	GGGGACCACTTTGTACAAGAAAGCTGGGTTTACATAACATGGCAGCATTTCGTTTT
9_hypothetical	J19	Tb927.11.5590_for	GGGGACAAGTTTGTACAAAAAAGCAGGCTATGAAAATATTGCACACACGCGC	Tb927.11.5590_rev	GGGGACCACTTTGTACAAGAAAGCTGGGTCTAGCCACGGTAAAAATCAACAACAAC
9_hypothetical	J20	Tb927.8.4100_for	GGGGACAAGTTTGTACAAAAAAGCAGGCTATGCCGTTGTGGAAACAAACCA	Tb927.8.4100_rev	GGGGACCACTTTGTACAAGAAAGCTGGGTTCACTGCTCGACCCTTTCTTTGT
9_hypothetical	J22	Tb927.6.2920_for	GGGGACAAGTTTGTACAAAAAAGCAGGCTATGCTGCATGAAATTTACGCTCCA	Tb927.6.2920_rev	GGGGACCACTTTGTACAAGAAAGCTGGGTTTAATCGCTAAACTCCTCAATTACCTCTC
9_hypothetical	J23	Tb927.11.9620.1_for	GGGGACAAGTTTGTACAAAAAAGCAGGCTATGCTGTCTGTTGTGCGCG	Tb927.11.9620.1_rev	GGGGACCACTTTGTACAAGAAAGCTGGGTTCACCAACCCAAACCCTTAAGAAATC
9_hypothetical	J24	Tb927.5.2680_for	GGGGACAAGTTTGTACAAAAAAGCAGGCTATGGTCGATGAAAAATTACCGACGT	Tb927.5.2680_rev	GGGGACCACTTTGTACAAGAAAGCTGGGTTTAATTCTCTTCTTCTGCGGCTGG
9_hypothetical	K1	Tb927.11.1430_for	GGGGACAAGTTTGTACAAAAAAGCAGGCTATGGCTTACCAGCAGTCACGG	Tb927.11.1430_rev	GGGGACCACTTTGTACAAGAAAGCTGGGTTTACTTGGATTCACCTGCTTTGATATCC
9_hypothetical	K2	Tb927.11.5560_for	GGGGACAAGTTTGTACAAAAAAGCAGGCTATGATGCCAGTATTCGGCCCA	Tb927.11.5560_rev	GGGGACCACTTTGTACAAGAAAGCTGGGTTTATTCAACGAAAGCGCGAATTGC
9_hypothetical	K3	Tb927.5.2360_for	GGGGACAAGTTTGTACAAAAAAGCAGGCTATGGAGAGAAATACTCCTCGTTACCA	Tb927.5.2360_rev	GGGGACCACTTTGTACAAGAAAGCTGGGTTTAACGCACATATGTCTTCGGAGC
9_hypothetical	K4	Tb927.3.850_for	GGGGACAAGTTTGTACAAAAAAGCAGGCTATGACGTTGGCGAGCGAAG	Tb927.3.850_rev	GGGGACCACTTTGTACAAGAAAGCTGGGTTCACGCACGTTCCCCTACG
9_hypothetical	K5	Tb927.4.3530_for	GGGGACAAGTTTGTACAAAAAAGCAGGCTATGAGGAGTCGGTTCTTTACACACG	Tb927.4.3530_rev	GGGGACCACTTTGTACAAGAAAGCTGGGTCTAACTAACCCAGTTGTTGGTGCG
9_hypothetical	K6	Tb927.11.13700_for	GGGGACAAGTTTGTACAAAAAAGCAGGCTATGGCATCAACCGATGAAGTAGTG	Tb927.11.13700_rev	GGGGACCACTTTGTACAAGAAAGCTGGGTCTACTGGGAAAGGTGTGACAAAGTG
9_hypothetical	K7	Tb927.7.4050_for	GGGGACAAGTTTGTACAAAAAAGCAGGCTATGATCCACAGCACCGCGA	Tb927.7.4050_rev	GGGGACCACTTTGTACAAGAAAGCTGGGTTCAAGTATTACCCTGGCGAATGGT
9_hypothetical	K8	Tb927.11.12620_for	GGGGACAAGTTTGTACAAAAAAGCAGGCTATGAGAGAACGAAACGCTTCCG	Tb927.11.12620_rev	GGGGACCACTTTGTACAAGAAAGCTGGGTTCACTCCCCCACTTCCCTTCT
9_hypothetical	K9	Tb927.11.14450_for	GGGGACAAGTTTGTACAAAAAAGCAGGCTATGACTATTTTGCGGCGTGTGA	Tb927.11.14450_rev	GGGGACCACTTTGTACAAGAAAGCTGGGTTCACATAGCTGAGCTGTTGGATCT
9_hypothetical	K10	Tb927.6.2650_for	GGGGACAAGTTTGTACAAAAAAGCAGGCTATGGACGTGTTTTCAAGACATGGG	Tb927.6.2650_rev	GGGGACCACTTTGTACAAGAAAGCTGGGTTCACGAAATGCCACCCTTCAAA
9_hypothetical	K11	Tb927.11.840.1_for	GGGGACAAGTTTGTACAAAAAAGCAGGCTATGGCAAAACATCCCGGTGAG	Tb927.11.840.1_rev	GGGGACCACTTTGTACAAGAAAGCTGGGTTTACTTTGCAGCAGCTGGAACA
9_hypothetical	K12	Tb927.9.14030_for	GGGGACAAGTTTGTACAAAAAAGCAGGCTATGACATCGCTTCCGTCGC	Tb927.9.14030_rev	GGGGACCACTTTGTACAAGAAAGCTGGGTCTATGGACCGTCAGCAAGGTGT
9_hypothetical	K13	Tb927.7.6480_for	GGGGACAAGTTTGTACAAAAAAGCAGGCTATGAATCAAAGCCCGTTTGATCACC	Tb927.7.6480_rev	GGGGACCACTTTGTACAAGAAAGCTGGGTCTACCTTGTTTCCTGGAAGTGACAG
9_hypothetical	K14	Tb927.2.3390_for	GGGGACAAGTTTGTACAAAAAAGCAGGCTATGCACGTGGGTGATGGAAC	Tb927.2.3390_rev	GGGGACCACTTTGTACAAGAAAGCTGGGTTCACATGAGCGCAGACAAGGC
9_hypothetical	K15	Tb927.10.3880_for	GGGGACAAGTTTGTACAAAAAAGCAGGCTATGTTTTGGGCTATCACGGGTTC	Tb927.10.3880_rev	GGGGACCACTTTGTACAAGAAAGCTGGGTCTACGCCAAATCATCACACAATAGAAAG
9_hypothetical	K16	Tb927.6.2710_for	GGGGACAAGTTTGTACAAAAAAGCAGGCTATGTCAGTTACCGTTGAAGGTGC	Tb927.6.2710_rev	GGGGACCACTTTGTACAAGAAAGCTGGGTTCATGCTGAAAGCAACAAACAATTGA
9_hypothetical	K17	Tb927.7.6090_for	GGGGACAAGTTTGTACAAAAAAGCAGGCTATGTTGCAAGCGGAAGTAAACTCG	Tb927.7.6090_rev	GGGGACCACTTTGTACAAGAAAGCTGGGTCTACTTCCCTTGTAGGCGCTCT
9_hypothetical	K18	Tb927.2.1700_for	GGGGACAAGTTTGTACAAAAAAGCAGGCTATGCTTGTCGGGGTGCTTG	Tb927.2.1700_rev	GGGGACCACTTTGTACAAGAAAGCTGGGTTCATTCCTTGTTAGGGAACCCTTCA
9_hypothetical	K19	Tb927.3.5140_for	GGGGACAAGTTTGTACAAAAAAGCAGGCTATGGATTTGAAGGAGAACCGTAGAAGT	Tb927.3.5140_rev	GGGGACCACTTTGTACAAGAAAGCTGGGTTCATTCCTGAGCTGGGCCTG
9_hypothetical	K20	Tb927.11.8270_for	GGGGACAAGTTTGTACAAAAAAGCAGGCTATGACGTCACCTCACCTCCTTAC	Tb927.11.8270_rev	GGGGACCACTTTGTACAAGAAAGCTGGGTTCAAAACGTTGCTACACCCGC
9_hypothetical	K21	Tb927.11.9290_for	GGGGACAAGTTTGTACAAAAAAGCAGGCTATGCTACAAAAGGAGGTATTGGGC	Tb927.11.9290_rev	GGGGACCACTTTGTACAAGAAAGCTGGGTTTACGTCCCCTCAGGCTGTTC
9_hypothetical	K22	Tb927.6.1720_for	GGGGACAAGTTTGTACAAAAAAGCAGGCTATGCAGTACCTTTACGATGATACGGT	Tb927.6.1720_rev	GGGGACCACTTTGTACAAGAAAGCTGGGTTTAATGTGCAGCGTTCATTAGGCG
9_hypothetical	K23	Tb927.2.5480_for	GGGGACAAGTTTGTACAAAAAAGCAGGCTATGTCCACGGTAAAGGCAGCC	Tb927.2.5480_rev	GGGGACCACTTTGTACAAGAAAGCTGGGTTTAAACAATGAGGTATCCCTTGGGC
9_hypothetical	K24	Tb927.10.4650_for	GGGGACAAGTTTGTACAAAAAAGCAGGCTATGAGCGCCAGTGTGTCTCC	Tb927.10.4650_rev	GGGGACCACTTTGTACAAGAAAGCTGGGTTCAACCTCTGACCGCGAGC
9_hypothetical	L1	Tb927.2.5650_for	GGGGACAAGTTTGTACAAAAAAGCAGGCTATGTCCACGGTACAGGCAGC	Tb927.2.5650_rev	GGGGACCACTTTGTACAAGAAAGCTGGGTTTAAACAATGAGGTATCCCTTGGGC
9_hypothetical	L2	Tb927.10.8820_for	GGGGACAAGTTTGTACAAAAAAGCAGGCTATGAACATGATATCCCTCAATTTCACCC	Tb927.10.8820_rev	GGGGACCACTTTGTACAAGAAAGCTGGGTCTATCGGCCCGGGATTGGT
9_hypothetical	L3	Tb927.11.2860_for	GGGGACAAGTTTGTACAAAAAAGCAGGCTATGGAACCCGGAACGGAGG	Tb927.11.2860_rev	GGGGACCACTTTGTACAAGAAAGCTGGGTCTACACGAAAGTCCACACCCG
9_hypothetical	L4	Tb927.11.15520_for	GGGGACAAGTTTGTACAAAAAAGCAGGCTATGCCGTGGATTCACCTTCAGT	Tb927.11.15520_rev	GGGGACCACTTTGTACAAGAAAGCTGGGTTTAGGAAGTGGCATAGCGCCA
9_hypothetical	L5	Tb927.11.14760_for	GGGGACAAGTTTGTACAAAAAAGCAGGCTATGGTGCGACTACCACCAATAGT	Tb927.11.14760_rev	GGGGACCACTTTGTACAAGAAAGCTGGGTTTAGATTGGTTGCTCAGTAAGGTAGAATG
9_hypothetical	L6	Tb927.6.1430_for	GGGGACAAGTTTGTACAAAAAAGCAGGCTATGGATCCCCAGTATTCGCAACC	Tb927.6.1430_rev	GGGGACCACTTTGTACAAGAAAGCTGGGTTCACGACAATCTCTCGAGGATGT
9_hypothetical	L7	Tb927.11.9120_for	GGGGACAAGTTTGTACAAAAAAGCAGGCTATGCTGCCTTGCCCAAGC	Tb927.11.9120_rev	GGGGACCACTTTGTACAAGAAAGCTGGGTTCACTGATTATCACCATCCTCTTCGT
9_hypothetical	L8	Tb927.11.5330_for	GGGGACAAGTTTGTACAAAAAAGCAGGCTATGAGTTGGCTTCAGGCTCTCG	Tb927.11.5330_rev	GGGGACCACTTTGTACAAGAAAGCTGGGTTCATGAAACTTCACAATTCCCGGTT
9_hypothetical	L9	Tb927.10.11040_for	GGGGACAAGTTTGTACAAAAAAGCAGGCTATGGTAGAGGGTCACAGCGAC	Tb927.10.11040_rev	GGGGACCACTTTGTACAAGAAAGCTGGGTTCACTTTGCATCTGCCGGC
9_hypothetical	L10	Tb927.7.450_for	GGGGACAAGTTTGTACAAAAAAGCAGGCTATGCAGGACGTACCAAATCCAAAC	Tb927.7.450_rev	GGGGACCACTTTGTACAAGAAAGCTGGGTTTATTCTTTCGACTGTCCCTGTTCAG
9_hypothetical	L11	Tb927.9.11500_for	GGGGACAAGTTTGTACAAAAAAGCAGGCTATGGAAGAAGATGAGGCGTTTGC	Tb927.9.11500_rev	GGGGACCACTTTGTACAAGAAAGCTGGGTTCAACCCTTGGGCAACGTATACA
9_hypothetical	L12	Tb927.2.2530_for	GGGGACAAGTTTGTACAAAAAAGCAGGCTATGTCAGGGCAACCACACTACG	Tb927.2.2530_rev	GGGGACCACTTTGTACAAGAAAGCTGGGTTCACATAGGGTCCGAAACACCG
9_hypothetical	L13	Tb927.11.10390_for	GGGGACAAGTTTGTACAAAAAAGCAGGCTATGAGTGGGAAGGGCTTTGTATCA	Tb927.11.10390_rev	GGGGACCACTTTGTACAAGAAAGCTGGGTCTAGTAAAACAACTTCTCACGCCGA
9_hypothetical	L14	Tb927.7.5330_for	GGGGACAAGTTTGTACAAAAAAGCAGGCTATGCCCATTTCGCCCTTGG	Tb927.7.5330_rev	GGGGACCACTTTGTACAAGAAAGCTGGGTTCAGCGAAAGCGTACCACCA
9_hypothetical	L15	Tb927.9.7820_for	GGGGACAAGTTTGTACAAAAAAGCAGGCTATGAAGTTGTGTGAACTTAGTCCAATAGC	Tb927.9.7820_rev	GGGGACCACTTTGTACAAGAAAGCTGGGTTCACTCGCTCTTCTCCCTATCTCG
9_hypothetical	L16	Tb927.3.1860_for	GGGGACAAGTTTGTACAAAAAAGCAGGCTATGTACAGAATAGCAATTCGACGTTTTG	Tb927.3.1860_rev	GGGGACCACTTTGTACAAGAAAGCTGGGTTTATTTTTTACCCCCGTGCCCTTT
9_hypothetical	L17	Tb927.11.9950_for	GGGGACAAGTTTGTACAAAAAAGCAGGCTATGGACCCCCTTTTACTTTCCAGG	Tb927.11.9950_rev	GGGGACCACTTTGTACAAGAAAGCTGGGTCTACAAGTCATGTCGGCAAAGATAAAC
9_hypothetical	L18	Tb927.7.560_for	GGGGACAAGTTTGTACAAAAAAGCAGGCTATGTTTGTACTGCACCGGGGC	Tb927.7.560_rev	GGGGACCACTTTGTACAAGAAAGCTGGGTCTAACGTAAAGTCGATCCCACTGC
9_hypothetical	L19	Tb927.3.1200_for	GGGGACAAGTTTGTACAAAAAAGCAGGCTATGAGTTTGCATGTCGGTAACGG	Tb927.3.1200_rev	GGGGACCACTTTGTACAAGAAAGCTGGGTCTACATTCCGGCAAGGCGAAG
9_hypothetical	L20	Tb927.10.3020_for	GGGGACAAGTTTGTACAAAAAAGCAGGCTATGTCGGGTCGGCGTGTC	Tb927.10.3020_rev	GGGGACCACTTTGTACAAGAAAGCTGGGTCTACTCACTGACCCCCTCACAC
9_hypothetical	L21	Tb927.3.4350_for	GGGGACAAGTTTGTACAAAAAAGCAGGCTATGCTTGTTACAACAACAGCGATATCT	Tb927.3.4350_rev	GGGGACCACTTTGTACAAGAAAGCTGGGTTCACGAAGAATCCTTCGGGCG
9_hypothetical	L22	Tb927.11.16640_for	GGGGACAAGTTTGTACAAAAAAGCAGGCTATGCAGGCGGAGGTGGAC	Tb927.11.16640_rev	GGGGACCACTTTGTACAAGAAAGCTGGGTTCATACGGACACTGCCGACT
9_hypothetical	L23	Tb927.10.9660_for	GGGGACAAGTTTGTACAAAAAAGCAGGCTATGAAGCGGAACCGTAACCAC	Tb927.10.9660_rev	GGGGACCACTTTGTACAAGAAAGCTGGGTTCATGCCTTTTTCTTCACAAACAACC
9_hypothetical	L24	Tb927.6.860_for	GGGGACAAGTTTGTACAAAAAAGCAGGCTATGCACATGTACAACCCTGTGG	Tb927.6.860_rev	GGGGACCACTTTGTACAAGAAAGCTGGGTTCATGAACCGCCATTCTGTTGG
9_hypothetical	M1	Tb927.8.850_for	GGGGACAAGTTTGTACAAAAAAGCAGGCTATGTTTGGGTCTTCGCTAAGCG	Tb927.8.850_rev	GGGGACCACTTTGTACAAGAAAGCTGGGTCTACACGCTCACAGCGCTG
9_hypothetical	M2	Tb927.1.4410_for	GGGGACAAGTTTGTACAAAAAAGCAGGCTATGCAAAAAACCCCTCGATTAACGA	Tb927.1.4410_rev	GGGGACCACTTTGTACAAGAAAGCTGGGTTCACATGATGCCGTAGTCGTCC
9_hypothetical	M3	Tb927.5.2950_for	GGGGACAAGTTTGTACAAAAAAGCAGGCTATGAATTCGACGAGTACATCAAGTTACG	Tb927.5.2950_rev	GGGGACCACTTTGTACAAGAAAGCTGGGTCTACGACAGTTGCTGAAGTGTGC

9_hypothetical	M4	Tb927.10.15660_for	GGGGACAAGTTTGTACAAAAAAGCAGGCTATGATTCGTTACACTCAATACCTTATCACG	Tb927.10.15660_rev	GGGGACCACTTTGTACAAGAAAGCTGGGTTCAGTTATGCTGTGTGTCGGTTG
9_hypothetical	M5	Tb927.10.2860_for	GGGGACAAGTTTGTACAAAAAAGCAGGCTATGTCCACTCCGAAGTGCACG	Tb927.10.2860_rev	GGGGACCACTTTGTACAAGAAAGCTGGGTTCAAGCCTTCCGAAGTGCCT
9_hypothetical	M6	Tb927.11.1220_for	GGGGACAAGTTTGTACAAAAAAGCAGGCTATGTCGGATTTCGTCGATATTAATGTGA	Tb927.11.1220_rev	GGGGACCACTTTGTACAAGAAAGCTGGGTTTACCGTTGCCTACTTCTGTATTCG
9_hypothetical	M7	Tb927.10.15780_for	GGGGACAAGTTTGTACAAAAAAGCAGGCTATGTCTCTGGGATGTGATCAACACA	Tb927.10.15780_rev	GGGGACCACTTTGTACAAGAAAGCTGGGTTCAACAACCATCAGGCGTTAGC
9_hypothetical	M8	Tb927.10.11720_for	GGGGACAAGTTTGTACAAAAAAGCAGGCTATGCTAACTCGAAGAGCCGCA	Tb927.10.11720_rev	GGGGACCACTTTGTACAAGAAAGCTGGGTCTATGTCCTCCTCCTTTTAAGCTCCT
9_hypothetical	M9	Tb927.9.13470_for	GGGGACAAGTTTGTACAAAAAAGCAGGCTATGGATGTGCATTCCCCGTTAC	Tb927.9.13470_rev	GGGGACCACTTTGTACAAGAAAGCTGGGTTTAGCCTATGCGAGCCAAAAATGG
9_hypothetical	M10	Tb927.3.2630_for	GGGGACAAGTTTGTACAAAAAAGCAGGCTATGCGGGTGTTTAGCTCCCC	Tb927.3.2630_rev	GGGGACCACTTTGTACAAGAAAGCTGGGTTCACAGTGCAGCTGCCTGT
9_hypothetical	M11	Tb927.11.16470_for	GGGGACAAGTTTGTACAAAAAAGCAGGCTATGACCGAAACTGATGGGGGG	Tb927.11.16470_rev	GGGGACCACTTTGTACAAGAAAGCTGGGTTCAAGCCAATTCACGTTTGTGACC
9_hypothetical	M12	Tb927.10.12130_for	GGGGACAAGTTTGTACAAAAAAGCAGGCTATGTCTGCGACATCCCCGA	Tb927.10.12130_rev	GGGGACCACTTTGTACAAGAAAGCTGGGTTTACTCTTGCATTACCTCGACGACA
9_hypothetical	M13	Tb927.11.12560_for	GGGGACAAGTTTGTACAAAAAAGCAGGCTATGGACGTGGAGGAATCAGAGC	Tb927.11.12560_rev	GGGGACCACTTTGTACAAGAAAGCTGGGTCTAGCAACCGTTGTCGGCC
9_hypothetical	M14	Tb927.10.2260_for	GGGGACAAGTTTGTACAAAAAAGCAGGCTATGAGTTCACGGGAGCCAACA	Tb927.10.2260_rev	GGGGACCACTTTGTACAAGAAAGCTGGGTCTAAATTTGATCGTCGCCGTCATG
9_hypothetical	M15	Tb927.10.3130_for	GGGGACAAGTTTGTACAAAAAAGCAGGCTATGAATGTTCTCATTGGAAAACGTGAGC	Tb927.10.3130_rev	GGGGACCACTTTGTACAAGAAAGCTGGGTTTACCGTCCTATCACTCCAGGC
9_hypothetical	M16	Tb927.3.3150_for	GGGGACAAGTTTGTACAAAAAAGCAGGCTATGTCAGCATTTGAAGATAATCCGTTTG	Tb927.3.3150_rev	GGGGACCACTTTGTACAAGAAAGCTGGGTCTACCAACTGCCAAACGGACC
9_hypothetical	M17	Tb927.10.3000_for	GGGGACAAGTTTGTACAAAAAAGCAGGCTATGAACAGTTTCGTGTCTCATTCCTC	Tb927.10.3000_rev	GGGGACCACTTTGTACAAGAAAGCTGGGTTCATATGGAGTCATCACTGTCATCTCC
9_hypothetical	M18	Tb927.11.12660_for	GGGGACAAGTTTGTACAAAAAAGCAGGCTATGAAAACCGAGGTGGAGACCA	Tb927.11.12660_rev	GGGGACCACTTTGTACAAGAAAGCTGGGTTCAACAGCAAGTGAGGGATGGC
9_hypothetical	M19	Tb927.3.1350_for	GGGGACAAGTTTGTACAAAAAAGCAGGCTATGCAACGACGTAAGCGCT	Tb927.3.1350_rev	GGGGACCACTTTGTACAAGAAAGCTGGGTTCAGTTACCGAAGCGTCGCA
9_hypothetical	M20	Tb927.11.4950_for	GGGGACAAGTTTGTACAAAAAAGCAGGCTATGGAATACAAATCTGTTTTTGAACTTCGA	Tb927.11.4950_rev	GGGGACCACTTTGTACAAGAAAGCTGGGTCTAATGCCGTTTCTGCCGGG
9_hypothetical	M21	Tb927.7.7270_for	GGGGACAAGTTTGTACAAAAAAGCAGGCTATGCATGTCGGTGCGGAAC	Tb927.7.7270_rev	GGGGACCACTTTGTACAAGAAAGCTGGGTTTAAGACTTCAGGAAGCTTCGTTGG
9_hypothetical	M22	Tb927.11.2120_for	GGGGACAAGTTTGTACAAAAAAGCAGGCTATGACTCGAGAAGAGGAGGTATTGG	Tb927.11.2120_rev	GGGGACCACTTTGTACAAGAAAGCTGGGTTTACCGCCTTCTGCTCACAAAG
9_hypothetical	M23	Tb927.3.1660_for	GGGGACAAGTTTGTACAAAAAAGCAGGCTATGACAATGTCGCCTTCTACTCGA	Tb927.3.1660_rev	GGGGACCACTTTGTACAAGAAAGCTGGGTCTAAGACTTCCCTACAACACATTGGAG
9_hypothetical	M24	Tb927.1.4250_for	GGGGACAAGTTTGTACAAAAAAGCAGGCTATGGTAGGAGGATATTTACGTATGCCT	Tb927.1.4250_rev	GGGGACCACTTTGTACAAGAAAGCTGGGTTTATCGGCTTAGTTTCCGCACATAAG
9_hypothetical	N1	Tb927.8.3710_for	GGGGACAAGTTTGTACAAAAAAGCAGGCTATGGGACGCCGAAAGTGTTC	Tb927.8.3710_rev	GGGGACCACTTTGTACAAGAAAGCTGGGTTTACTGTTGCTCGCATGCCG
9_hypothetical	N2	Tb927.4.2780_for	GGGGACAAGTTTGTACAAAAAAGCAGGCTATGACACATTGTGACCCATCGAAC	Tb927.4.2780_rev	GGGGACCACTTTGTACAAGAAAGCTGGGTTCAACGCCCATGAAAGGGGT
9_hypothetical	N3	Tb927.1.3450_for	GGGGACAAGTTTGTACAAAAAAGCAGGCTATGGCCTCCTTCCTGCCATC	Tb927.1.3450_rev	GGGGACCACTTTGTACAAGAAAGCTGGGTCTACCACAGCCGCACTCTTG
9_hypothetical	N4	Tb927.10.5920_for	GGGGACAAGTTTGTACAAAAAAGCAGGCTATGGGTGTGCCAGACTTTCTAAAG	Tb927.10.5920_rev	GGGGACCACTTTGTACAAGAAAGCTGGGTTCAGGAAAGTTTTTTCCGTTTTACTTTTTT
9_hypothetical	N5	Tb927.10.12140_for	GGGGACAAGTTTGTACAAAAAAGCAGGCTATGCCCGGGAGTTTGAATTCC	Tb927.10.12140_rev	GGGGACCACTTTGTACAAGAAAGCTGGGTTCAGTACTTTCCTTGTGAACTGATGTAC
9_hypothetical	N6	Tb927.10.12250_for	GGGGACAAGTTTGTACAAAAAAGCAGGCTATGGGGTCACCAACCCCAA	Tb927.10.12250_rev	GGGGACCACTTTGTACAAGAAAGCTGGGTTTATTTACCGGCATTTGTTGCACCC
9_hypothetical	N7	Tb927.11.15800_for	GGGGACAAGTTTGTACAAAAAAGCAGGCTATGTCAAAGCCAACTCGGAAGTC	Tb927.11.15800_rev	GGGGACCACTTTGTACAAGAAAGCTGGGTCTACGATTTGGAAGAAACTGTCGTCC
9_hypothetical	N8	Tb927.6.4760_for	GGGGACAAGTTTGTACAAAAAAGCAGGCTATGTCGGAGCCGACTACACC	Tb927.6.4760_rev	GGGGACCACTTTGTACAAGAAAGCTGGGTCTAAGCGGACGCCTCCTGC
9_hypothetical	N9	Tb927.2.4470_for	GGGGACAAGTTTGTACAAAAAAGCAGGCTATGTCACATTCCAGTAGAGATGATGGG	Tb927.2.4470_rev	GGGGACCACTTTGTACAAGAAAGCTGGGTTCATTTCACTGCTTCCCGTAACG
9_hypothetical	N10	Tb927.8.3170_for	GGGGACAAGTTTGTACAAAAAAGCAGGCTATGCAAGATAAGGCGGTGCAA	Tb927.8.3170_rev	GGGGACCACTTTGTACAAGAAAGCTGGGTTTACAGAGTTTTGCAATACTTAGCCTTAAC
9_hypothetical	N11	Tb927.11.810_for	GGGGACAAGTTTGTACAAAAAAGCAGGCTATGGAGCCGATGGAGCAACC	Tb927.11.810_rev	GGGGACCACTTTGTACAAGAAAGCTGGGTCTACTGCTCCCAATTAAAGCGGC
9_hypothetical	N12	Tb927.6.4100_for	GGGGACAAGTTTGTACAAAAAAGCAGGCTATGTCGTCACTAAAAGGGGCGC	Tb927.6.4100_rev	GGGGACCACTTTGTACAAGAAAGCTGGGTTCAAGTGTGAGAAATGCGTTGCA
9_hypothetical	N13	Tb927.5.3240_for	GGGGACAAGTTTGTACAAAAAAGCAGGCTATGGCGCAACACTCAGAAGC	Tb927.5.3240_rev	GGGGACCACTTTGTACAAGAAAGCTGGGTTTACTGGTCGTACACCGGTGAG
9_hypothetical	N14	Tb927.11.3490_for	GGGGACAAGTTTGTACAAAAAAGCAGGCTATGGACACCCTTACGTACGTGT	Tb927.11.3490_rev	GGGGACCACTTTGTACAAGAAAGCTGGGTTCATGTCACCAGGAAGTGAATGAGT
9_hypothetical	N15	Tb927.10.5910_for	GGGGACAAGTTTGTACAAAAAAGCAGGCTATGGGGATTCAAACGATGTCAGAAG	Tb927.10.5910_rev	GGGGACCACTTTGTACAAGAAAGCTGGGTCTATACAACCAACGAGTCCATGTACC
9_hypothetical	N16	Tb927.11.6260_for	GGGGACAAGTTTGTACAAAAAAGCAGGCTATGGCGGATGGAGCTGAAGG	Tb927.11.6260_rev	GGGGACCACTTTGTACAAGAAAGCTGGGTCTATGCGAGGTTAGTTGGAGCG
9_hypothetical	N17	Tb927.6.4550_for	GGGGACAAGTTTGTACAAAAAAGCAGGCTATGGCAGCCCAACACACATG	Tb927.6.4550_rev	GGGGACCACTTTGTACAAGAAAGCTGGGTCTACAGGGACTTCCCACAAGTGA
9_hypothetical	N18	Tb927.1.790_for	GGGGACAAGTTTGTACAAAAAAGCAGGCTATGCGGCTTCATTCGCGT	Tb927.1.790_rev	GGGGACCACTTTGTACAAGAAAGCTGGGTTCACGCCCTTCGACGGAA
9_hypothetical	N19	Tb927.11.10120_for	GGGGACAAGTTTGTACAAAAAAGCAGGCTATGTCCCTCAACCGCAAGTATAGC	Tb927.11.10120_rev	GGGGACCACTTTGTACAAGAAAGCTGGGTTTATCCCTGTAGTGACTTTTTTCTAGCAAT
9_hypothetical	N20	Tb927.2.1760_for	GGGGACAAGTTTGTACAAAAAAGCAGGCTATGAGTATTCTGTGGCTTACTTGTATCG	Tb927.2.1760_rev	GGGGACCACTTTGTACAAGAAAGCTGGGTTCACTCACGGTCAGATGAGTGAG
9_hypothetical	N21	Tb927.10.16090_for	GGGGACAAGTTTGTACAAAAAAGCAGGCTATGCGTAGATCGGGCAGAGG	Tb927.10.16090_rev	GGGGACCACTTTGTACAAGAAAGCTGGGTCTACTTGGCCGCGTGCCC
9_hypothetical	N22	Tb927.8.1250_for	GGGGACAAGTTTGTACAAAAAAGCAGGCTATGCGACGCCCATTGCGT	Tb927.8.1250_rev	GGGGACCACTTTGTACAAGAAAGCTGGGTTCATGCACCGTGATGCCTTAGT
9_hypothetical	N23	Tb927.5.3650_for	GGGGACAAGTTTGTACAAAAAAGCAGGCTATGCCGAGCGACCACCAG	Tb927.5.3650_rev	GGGGACCACTTTGTACAAGAAAGCTGGGTTCACAACAAATTCCACGTTCGCT
9_hypothetical	N24	Tb927.11.12530_for	GGGGACAAGTTTGTACAAAAAAGCAGGCTATGAGCACATCTTTAGTAACGCTTGC	Tb927.11.12530_rev	GGGGACCACTTTGTACAAGAAAGCTGGGTTTATTGCCGGCGTTGCGC
9_hypothetical	O1	Tb927.7.2050_for	GGGGACAAGTTTGTACAAAAAAGCAGGCTATGAACTGTTCAACTGCAGGCG	Tb927.7.2050_rev	GGGGACCACTTTGTACAAGAAAGCTGGGTCTAACGAGTTTCCCTCTCCCTCC
9_hypothetical	O2	Tb927.9.11150_for	GGGGACAAGTTTGTACAAAAAAGCAGGCTATGGGTGTAGATAAAAAAATAAGTTCTCTA	Tb927.9.11150_rev	GGGGACCACTTTGTACAAGAAAGCTGGGTTCATATGGTAAAAAATAGAAACACCATTAA
9_hypothetical	O3	Tb927.7.3270_for	GGGGACAAGTTTGTACAAAAAAGCAGGCTATGCATCGTCGCCTTCGAATT	Tb927.7.3270_rev	GGGGACCACTTTGTACAAGAAAGCTGGGTTCAACTCTCAACAAGAAAAACCATTTTTGC
9_hypothetical	O4	Tb927.10.3470_for	GGGGACAAGTTTGTACAAAAAAGCAGGCTATGTTGTCACCGATTGTGGACATG	Tb927.10.3470_rev	GGGGACCACTTTGTACAAGAAAGCTGGGTTCAAGCAAGAGGACCTTCCAACA
9_hypothetical	O5	Tb927.9.2817_for	GGGGACAAGTTTGTACAAAAAAGCAGGCTATGGAAATCCGCATGGTGCC	Tb927.9.2817_rev	GGGGACCACTTTGTACAAGAAAGCTGGGTTCACTCATCAGCTGCTGGAGT
9_hypothetical	O6	Tb927.10.2590_for	GGGGACAAGTTTGTACAAAAAAGCAGGCTATGCGGATCGCAACGAGTC	Tb927.10.2590_rev	GGGGACCACTTTGTACAAGAAAGCTGGGTTCAGTCAAAGTCCTTCAATTCAAATCGT
9_hypothetical	O7	Tb927.6.3450_for	GGGGACAAGTTTGTACAAAAAAGCAGGCTATGTATAAGGAAACGGGAGCTACGC	Tb927.6.3450_rev	GGGGACCACTTTGTACAAGAAAGCTGGGTTTACCGTTGCCCCATCAACTG
9_hypothetical	O8	Tb927.3.3300_for	GGGGACAAGTTTGTACAAAAAAGCAGGCTATGGCCACCCTCAACAACTTAAC	Tb927.3.3300_rev	GGGGACCACTTTGTACAAGAAAGCTGGGTCTAAGAACGCTGACGGTGCG
9_hypothetical	O9	Tb927.10.1530_for	GGGGACAAGTTTGTACAAAAAAGCAGGCTATGAGTGGCGTTTCTGGTGTTG	Tb927.10.1530_rev	GGGGACCACTTTGTACAAGAAAGCTGGGTCTACAAACACGGTTGAGCTTTACTAATG
9_hypothetical	O10	Tb927.9.1440_for	GGGGACAAGTTTGTACAAAAAAGCAGGCTATGTCAGCTGCATGTCCGTTG	Tb927.9.1440_rev	GGGGACCACTTTGTACAAGAAAGCTGGGTTCACGCCTTTACAGAACCATGAAA
9_hypothetical	O11	Tb927.5.4040_for	GGGGACAAGTTTGTACAAAAAAGCAGGCTATGGTTAGACGTAGTCATGTGGTTGC	Tb927.5.4040_rev	GGGGACCACTTTGTACAAGAAAGCTGGGTTTATGGTGCGTTCCACTCAAGC
9_hypothetical	O12	Tb927.5.1320_for	GGGGACAAGTTTGTACAAAAAAGCAGGCTATGCCGAATTCGGCACCG	Tb927.5.1320_rev	GGGGACCACTTTGTACAAGAAAGCTGGGTCTACGTTGCGTATAGATACGGATGTATC
9_hypothetical	O13	Tb927.4.590_for	GGGGACAAGTTTGTACAAAAAAGCAGGCTATGCGGCCCACTTTATACTTTCTG	Tb927.4.590_rev	GGGGACCACTTTGTACAAGAAAGCTGGGTTTATCGCCACAGTTGTCTCACAC
9_hypothetical	O14	Tb927.5.4570_for	GGGGACAAGTTTGTACAAAAAAGCAGGCTATGTGTTTTATATTTGGTGTTGAGATGAGT	Tb927.5.4570_rev	GGGGACCACTTTGTACAAGAAAGCTGGGTTTACGGTGCGGTGGTTCCC
9_hypothetical	O16	Tb927.6.5090_for	GGGGACAAGTTTGTACAAAAAAGCAGGCTATGGACCATGAGGAAGTTCACGC	Tb927.6.5090_rev	GGGGACCACTTTGTACAAGAAAGCTGGGTTTAGTGCAGCATCCTTCCGTGA
9_hypothetical	O17	Tb927.11.13340_for	GGGGACAAGTTTGTACAAAAAAGCAGGCTATGCCTCTAATTTTCTTGCAGACGC	Tb927.11.13340_rev	GGGGACCACTTTGTACAAGAAAGCTGGGTCTATTCTTCTTCATTTACAGGCCACTCA
9_hypothetical	O18	Tb927.7.6950_for	GGGGACAAGTTTGTACAAAAAAGCAGGCTATGCAGCCAACGGGGCGC	Tb927.7.6950_rev	GGGGACCACTTTGTACAAGAAAGCTGGGTCTATCGCGTGTGCCCAATGA
9_hypothetical	O19	Tb927.5.1340_for	GGGGACAAGTTTGTACAAAAAAGCAGGCTATGGGAACAACATCCACAGATACCC	Tb927.5.1340_rev	GGGGACCACTTTGTACAAGAAAGCTGGGTTCACGTAAGTGCTGGCACCG
9_hypothetical	O20	Tb927.11.450_for	GGGGACAAGTTTGTACAAAAAAGCAGGCTATGGACGCATATCAATCTTTAAGCGG	Tb927.11.450_rev	GGGGACCACTTTGTACAAGAAAGCTGGGTCTATTTACTGGTGACGGGATAAGGC
9_hypothetical	O21	Tb927.9.10140_for	GGGGACAAGTTTGTACAAAAAAGCAGGCTATGCAGAAAGCTAAGAACCAAAGCT	Tb927.9.10140_rev	GGGGACCACTTTGTACAAGAAAGCTGGGTCTAATGGGGTGGCTCCGCG
9_hypothetical	O22	Tb927.8.8160_for	GGGGACAAGTTTGTACAAAAAAGCAGGCTATGAGCGATTCGGAGTCCATTCA	Tb927.8.8160_rev	GGGGACCACTTTGTACAAGAAAGCTGGGTCTATACGGGGTTTTTGAGCAGGG
9_hypothetical	O23	Tb927.10.10100_for	GGGGACAAGTTTGTACAAAAAAGCAGGCTATGAATAGCAGTGGTCTTGTTTTGGA	Tb927.10.10100_rev	GGGGACCACTTTGTACAAGAAAGCTGGGTCTATTTTTCGGAATTCATTTTCTCGTTACC
9_hypothetical	O24	Tb927.8.7780_for	GGGGACAAGTTTGTACAAAAAAGCAGGCTATGGGAGCACTGCCGAGC	Tb927.8.7780_rev	GGGGACCACTTTGTACAAGAAAGCTGGGTCTATTCCCTGTAAAATATATTTGACTGCCC
9_hypothetical	P1	Tb927.11.14440_for	GGGGACAAGTTTGTACAAAAAAGCAGGCTATGATGAGTGCAAAACTTGGGATCA	Tb927.11.14440_rev	GGGGACCACTTTGTACAAGAAAGCTGGGTCTAAAGAAGACCCTTTAGATCCGTTGTT
9_hypothetical	P2	Tb927.11.14970_for	GGGGACAAGTTTGTACAAAAAAGCAGGCTATGCTTAAGTTGCCTACAATTGCTCA	Tb927.11.14970_rev	GGGGACCACTTTGTACAAGAAAGCTGGGTTTACTCCATCTCCCTTCGCTTAAGA
9_hypothetical	P3	Tb927.4.4550_for	GGGGACAAGTTTGTACAAAAAAGCAGGCTATGGGAGCACTTCCGAGCA	Tb927.4.4550_rev	GGGGACCACTTTGTACAAGAAAGCTGGGTTTACTCTAATATTAAAGACGGCGCGC
9_hypothetical	P4	Tb927.7.6910_for	GGGGACAAGTTTGTACAAAAAAGCAGGCTATGTCGGGAAGTGACGCAGA	Tb927.7.6910_rev	GGGGACCACTTTGTACAAGAAAGCTGGGTTTATTCCTTGGTCGCCACAGC
9_hypothetical	P5	Tb927.7.670_for	GGGGACAAGTTTGTACAAAAAAGCAGGCTATGGAAGATTTTGACTTTCTAGACGATGC	Tb927.7.670_rev	GGGGACCACTTTGTACAAGAAAGCTGGGTTCATGCCAAGCCTTTGGAGAAAC
9_hypothetical	P6	Tb927.11.10550_for	GGGGACAAGTTTGTACAAAAAAGCAGGCTATGATGGATGCGCAAACGAGT	Tb927.11.10550_rev	GGGGACCACTTTGTACAAGAAAGCTGGGTTCACGCCATGAGCCCTTCG
9_hypothetical	P7	Tb927.10.1560_for	GGGGACAAGTTTGTACAAAAAAGCAGGCTATGGCACTCAGTTTATCGGCG	Tb927.10.1560_rev	GGGGACCACTTTGTACAAGAAAGCTGGGTCTAGTTGGAAGCCCCTCCAGG
9_hypothetical	P8	Tb927.2.4990_for	GGGGACAAGTTTGTACAAAAAAGCAGGCTATGACACAGGAACATGGTAGTGAGG	Tb927.2.4990_rev	GGGGACCACTTTGTACAAGAAAGCTGGGTTCAAAACGGTCCGGAGGGC
9_hypothetical	P9	Tb927.6.3030_for	GGGGACAAGTTTGTACAAAAAAGCAGGCTATGAGCTGGAAATATTCGAATCAGGAG	Tb927.6.3030_rev	GGGGACCACTTTGTACAAGAAAGCTGGGTTCAACAGGTTGCTACACTGCG
9_hypothetical	P10	Tb927.10.2870_for	GGGGACAAGTTTGTACAAAAAAGCAGGCTATGGATTGGGCCCTCTTTAGCC	Tb927.10.2870_rev	GGGGACCACTTTGTACAAGAAAGCTGGGTTCATTCACTTTGGTACTGTAATTTCGCT
9_hypothetical	P11	Tb927.7.1410_for	GGGGACAAGTTTGTACAAAAAAGCAGGCTATGCTTCGACCGCACTGC	Tb927.7.1410_rev	GGGGACCACTTTGTACAAGAAAGCTGGGTTTAACGGCACTGAAGAACTATATCCATG
9_hypothetical	P12	Tb927.3.1180_for	GGGGACAAGTTTGTACAAAAAAGCAGGCTATGCCGGGCTACCCCACG	Tb927.3.1180_rev	GGGGACCACTTTGTACAAGAAAGCTGGGTTTAAAGAATCAGAAAGTAATCCTCCTGTGC
9_hypothetical	P13	Tb927.11.1630_for	GGGGACAAGTTTGTACAAAAAAGCAGGCTATGTTACGACGCTTTGCCCTC	Tb927.11.1630_rev	GGGGACCACTTTGTACAAGAAAGCTGGGTCTACTCTGCCTCAACGGTTGTTT
9_hypothetical	P14	Tb927.9.8610_for	GGGGACAAGTTTGTACAAAAAAGCAGGCTATGTCAACTCGGCAGATCCGG	Tb927.9.8610_rev	GGGGACCACTTTGTACAAGAAAGCTGGGTTCACTGCGAACCGCGCGT
9_hypothetical	P15	Tb927.4.4180_for	GGGGACAAGTTTGTACAAAAAAGCAGGCTATGAGCGACACGGAATCTATTCAGT	Tb927.4.4180_rev	GGGGACCACTTTGTACAAGAAAGCTGGGTCTACACGGGGTGTTTCAGTAGAGA
9_hypothetical	P16	Tb927.8.6980_for	GGGGACAAGTTTGTACAAAAAAGCAGGCTATGGAGGTTGACTACTCCGTGG	Tb927.8.6980_rev	GGGGACCACTTTGTACAAGAAAGCTGGGTCTAGCAACATTTCTTACTGTTAACCTTGG
9_hypothetical	P17	Tb927.11.5800_for	GGGGACAAGTTTGTACAAAAAAGCAGGCTATGCCTTTGTTTTCGTATGAGTGTCA	Tb927.11.5800_rev	GGGGACCACTTTGTACAAGAAAGCTGGGTTTATGACACCCCCAACTCTTCGC
9_hypothetical	P18	Tb927.5.3270_for	GGGGACAAGTTTGTACAAAAAAGCAGGCTATGGGCGACGGCAGCCAT	Tb927.5.3270_rev	GGGGACCACTTTGTACAAGAAAGCTGGGTTCAGCTGCTAGAGCTTTCATCCC
9_hypothetical	P19	Tb927.10.3090_for	GGGGACAAGTTTGTACAAAAAAGCAGGCTATGAGGTCGTTGGAGGTGCA	Tb927.10.3090_rev	GGGGACCACTTTGTACAAGAAAGCTGGGTTCACTCTTTCCTTGACTCTGCTAATTG
9_hypothetical	P20	Tb927.8.2620_for	GGGGACAAGTTTGTACAAAAAAGCAGGCTATGGCTCACCCGGTGCCA	Tb927.8.2620_rev	GGGGACCACTTTGTACAAGAAAGCTGGGTCTACTTCCTCTTCTTCGGCACAATAG
9_hypothetical	P21	Tb927.2.4160_for	GGGGACAAGTTTGTACAAAAAAGCAGGCTATGAGCTCCACACCGAATAATGAC	Tb927.2.4160_rev	GGGGACCACTTTGTACAAGAAAGCTGGGTCTACGATGCAGAGGGACTTGTACG
9_hypothetical	P22	Tb927.4.5340_for	GGGGACAAGTTTGTACAAAAAAGCAGGCTATGGAGGTCGAAGATTTCATGGC	Tb927.4.5340_rev	GGGGACCACTTTGTACAAGAAAGCTGGGTCTATTGGCAACATTTGTTGCTATTGACC
9_hypothetical	P23	Tb927.9.14260_for	GGGGACAAGTTTGTACAAAAAAGCAGGCTATGCGGGTAACATTGGAGGAGA	Tb927.9.14260_rev	GGGGACCACTTTGTACAAGAAAGCTGGGTCTACTGCACGTCCTTGTTGCA
9_hypothetical	P24	Tb927.11.830_for	GGGGACAAGTTTGTACAAAAAAGCAGGCTATGGGCCACGCTTTTTGCC	Tb927.11.830_rev	GGGGACCACTTTGTACAAGAAAGCTGGGTTCACACACTCCTGCCTTCACG
10_hypothetical	A1	Tb927.7.7130_for	GGGGACAAGTTTGTACAAAAAAGCAGGCTATGGAGCTGCTATCCGTTACGC	Tb927.7.7130_rev	GGGGACCACTTTGTACAAGAAAGCTGGGTTCAGCAGCCGGAAGTAATCACC
10_hypothetical	A2	Tb927.8.6870_for	GGGGACAAGTTTGTACAAAAAAGCAGGCTATGGCAAGCAGCGTACCAG	Tb927.8.6870_rev	GGGGACCACTTTGTACAAGAAAGCTGGGTTCAATACACTTGTTCCTCCAACCTAAATT
10_hypothetical	A3	Tb927.11.14470_for	GGGGACAAGTTTGTACAAAAAAGCAGGCTATGGGATCCCTATTCAAGCTACGT	Tb927.11.14470_rev	GGGGACCACTTTGTACAAGAAAGCTGGGTTCACTTCAGGAAGGAGGTCAATGC
10_hypothetical	A4	Tb927.4.3700_for	GGGGACAAGTTTGTACAAAAAAGCAGGCTATGCGTCGATCACTGACTTTATGG	Tb927.4.3700_rev	GGGGACCACTTTGTACAAGAAAGCTGGGTTTAACCTACTTCAAAGGACCGCCA
10_hypothetical	A5	Tb927.11.2740_for	GGGGACAAGTTTGTACAAAAAAGCAGGCTATGGCTGTTGTGGAGAAGTTCCT	Tb927.11.2740_rev	GGGGACCACTTTGTACAAGAAAGCTGGGTTCACTTTTTCCCACCTACGGAAGG
10_hypothetical	A6	Tb927.11.16960_for	GGGGACAAGTTTGTACAAAAAAGCAGGCTATGTGGACAGGGAGCATCAGT	Tb927.11.16960_rev	GGGGACCACTTTGTACAAGAAAGCTGGGTTCACTCAAGGGAAAAACTTTCGGTG
10_hypothetical	A7	Tb927.7.1230_for	GGGGACAAGTTTGTACAAAAAAGCAGGCTATGAGGCCCACACCAGACC	Tb927.7.1230_rev	GGGGACCACTTTGTACAAGAAAGCTGGGTCTATACAAGGCAGATTTCTTCCTTACTACA
10_hypothetical	A8	Tb927.7.3300_for	GGGGACAAGTTTGTACAAAAAAGCAGGCTATGGAATGCGTATCACAGTCGC	Tb927.7.3300_rev	GGGGACCACTTTGTACAAGAAAGCTGGGTTCAAATTTCAGCGTCTTCGAGCA
10_hypothetical	A9	Tb927.10.2130_for	GGGGACAAGTTTGTACAAAAAAGCAGGCTATGAAAACAAACAACTTGGAGCGTC	Tb927.10.2130_rev	GGGGACCACTTTGTACAAGAAAGCTGGGTTTAGGCTTCGGCAGATACCGA
10_hypothetical	A10	Tb927.7.4460_for	GGGGACAAGTTTGTACAAAAAAGCAGGCTATGAACTGCGAACACGTTGCG	Tb927.7.4460_rev	GGGGACCACTTTGTACAAGAAAGCTGGGTCTACACGCCGGCAAAGACC
10_hypothetical	A11	Tb927.11.5920_for	GGGGACAAGTTTGTACAAAAAAGCAGGCTATGTTGTCACGGAGCGCC	Tb927.11.5920_rev	GGGGACCACTTTGTACAAGAAAGCTGGGTTCATGGCGTTTGTGCTGCT
10_hypothetical	A12	Tb927.7.6630_for	GGGGACAAGTTTGTACAAAAAAGCAGGCTATGGACAGTGATACCATTTATTGTGATGAA	Tb927.7.6630_rev	GGGGACCACTTTGTACAAGAAAGCTGGGTTCACCACAACTCAGCGAGGC
10_hypothetical	A13	Tb927.10.11110_for	GGGGACAAGTTTGTACAAAAAAGCAGGCTATGAATGTTGCAGAGTTGCTGAAAAAC	Tb927.10.11110_rev	GGGGACCACTTTGTACAAGAAAGCTGGGTTTAGCTTCTGGCAGTGAGTCTGA
10_hypothetical	A14	Tb927.5.4470_for	GGGGACAAGTTTGTACAAAAAAGCAGGCTATGTCCGACGAGGCACCA	Tb927.5.4470_rev	GGGGACCACTTTGTACAAGAAAGCTGGGTTCAGTTGCCTCCACTCCCAA
10_hypothetical	A15	Tb927.11.1400_for	GGGGACAAGTTTGTACAAAAAAGCAGGCTATGCTGAAGCAATGTCTGCTTCA	Tb927.11.1400_rev	GGGGACCACTTTGTACAAGAAAGCTGGGTTTAGTCGTCAACATTCACCATAATCGATAC
10_hypothetical	A16	Tb927.10.7200_for	GGGGACAAGTTTGTACAAAAAAGCAGGCTATGCCAGAAATTAATTTGGATTGCAAGG	Tb927.10.7200_rev	GGGGACCACTTTGTACAAGAAAGCTGGGTCTACGAAGCCTGCTGCTGTTC
10_hypothetical	A17	Tb927.10.10190_for	GGGGACAAGTTTGTACAAAAAAGCAGGCTATGGCTGATCCCATCGCTGT	Tb927.10.10190_rev	GGGGACCACTTTGTACAAGAAAGCTGGGTTCAAAGAACTGAGCAAGCATGCT
10_hypothetical	A18	Tb927.6.3820_for	GGGGACAAGTTTGTACAAAAAAGCAGGCTATGGAATCCTCCGTTTATTCTGGTACA	Tb927.6.3820_rev	GGGGACCACTTTGTACAAGAAAGCTGGGTCTATAGCGTACATCCCTCCGGTT
10_hypothetical	A19	Tb927.8.2250_for	GGGGACAAGTTTGTACAAAAAAGCAGGCTATGGACGCGCAAGTTGGAG	Tb927.8.2250_rev	GGGGACCACTTTGTACAAGAAAGCTGGGTTCACCGCACAAACTGAAACACA
10_hypothetical	A20	Tb927.4.1980_for	GGGGACAAGTTTGTACAAAAAAGCAGGCTATGAAGCTATGGCAACACCTGC	Tb927.4.1980_rev	GGGGACCACTTTGTACAAGAAAGCTGGGTTTACTCACTTATGAGCCGATCGCT
10_hypothetical	A21	Tb927.7.750_for	GGGGACAAGTTTGTACAAAAAAGCAGGCTATGTTTGAGGGGGCTGGCG	Tb927.7.750_rev	GGGGACCACTTTGTACAAGAAAGCTGGGTCTACTCCGAATAGAAAGAACTTGGTTGC
10_hypothetical	A22	Tb927.7.3740_for	GGGGACAAGTTTGTACAAAAAAGCAGGCTATGTCGTACGTTGTTGGCGATG	Tb927.7.3740_rev	GGGGACCACTTTGTACAAGAAAGCTGGGTCTATGCACTACTCGGTTTAACATCTTTGT
10_hypothetical	A23	Tb927.11.16800_for	GGGGACAAGTTTGTACAAAAAAGCAGGCTATGAAGGGTAAATATATGCATAGCACTGTG	Tb927.11.16800_rev	GGGGACCACTTTGTACAAGAAAGCTGGGTCTATACGTTAGCCTCCAAAGTGTTCTC
10_hypothetical	A24	Tb927.10.12520_for	GGGGACAAGTTTGTACAAAAAAGCAGGCTATGCGCCGATATTCGACTAGGA	Tb927.10.12520_rev	GGGGACCACTTTGTACAAGAAAGCTGGGTTTATGTTTTTTTTCTAAAGAATGATTTCTT
10_hypothetical	B1	Tb927.11.3510_for	GGGGACAAGTTTGTACAAAAAAGCAGGCTATGTTTGGTGGTTTGGATTCGTCG	Tb927.11.3510_rev	GGGGACCACTTTGTACAAGAAAGCTGGGTTCAATCATCTCCGTCCGTTACTCT
10_hypothetical	B2	Tb927.10.14880_for	GGGGACAAGTTTGTACAAAAAAGCAGGCTATGGCAGAACATGCTTTTGCGT	Tb927.10.14880_rev	GGGGACCACTTTGTACAAGAAAGCTGGGTCTACTCGGTAATGGGCCCTGA
10_hypothetical	B3	Tb927.7.4040_for	GGGGACAAGTTTGTACAAAAAAGCAGGCTATGATGGAAAAGCAGTGGATAGTTTCTT	Tb927.7.4040_rev	GGGGACCACTTTGTACAAGAAAGCTGGGTTCATATTTTCATTGAAGAAGCGTCACTCG
10_hypothetical	B4	Tb927.10.13150_for	GGGGACAAGTTTGTACAAAAAAGCAGGCTATGGACGGCTTCACCTCCAA	Tb927.10.13150_rev	GGGGACCACTTTGTACAAGAAAGCTGGGTTCAAAGTTGAGAAGTACCGAATCTGG
10_hypothetical	B5	Tb927.7.4890_for	GGGGACAAGTTTGTACAAAAAAGCAGGCTATGAGTACTGGCCCTTCAACCA	Tb927.7.4890_rev	GGGGACCACTTTGTACAAGAAAGCTGGGTCTATGCCGTCACTCCGGTTG
10_hypothetical	B6	Tb927.6.2520_for	GGGGACAAGTTTGTACAAAAAAGCAGGCTATGGCGGAAATGCTGAACGAA	Tb927.6.2520_rev	GGGGACCACTTTGTACAAGAAAGCTGGGTTCACCTCGTCCCACAGTACAAC
10_hypothetical	B7	Tb927.10.14510_for	GGGGACAAGTTTGTACAAAAAAGCAGGCTATGGCTGCTTTCAGCGGG	Tb927.10.14510_rev	GGGGACCACTTTGTACAAGAAAGCTGGGTTTAGTCCTCCCCCTTGTCACG
10_hypothetical	B8	Tb927.10.9350_for	GGGGACAAGTTTGTACAAAAAAGCAGGCTATGTTAACCTCCCACCGCATTTC	Tb927.10.9350_rev	GGGGACCACTTTGTACAAGAAAGCTGGGTCTATCCAATTGAGGCAAGTACTTCTGT
10_hypothetical	B9	Tb927.11.10540_for	GGGGACAAGTTTGTACAAAAAAGCAGGCTATGCGAGTTAAGCGGAAGAACG	Tb927.11.10540_rev	GGGGACCACTTTGTACAAGAAAGCTGGGTTTAACTGATACGAGGCAACACATAGTG
10_hypothetical	B10	Tb927.7.4340_for	GGGGACAAGTTTGTACAAAAAAGCAGGCTATGGGAAAGCCGGGCGGC	Tb927.7.4340_rev	GGGGACCACTTTGTACAAGAAAGCTGGGTCTAGTTTGTTACGAAGGAATACGCCT
10_hypothetical	B11	Tb927.3.4060_for	GGGGACAAGTTTGTACAAAAAAGCAGGCTATGTGTGGCATCGGTTGCG	Tb927.3.4060_rev	GGGGACCACTTTGTACAAGAAAGCTGGGTTCAGGGAGAGCTGCAGCAT
10_hypothetical	B12	Tb927.4.4130_for	GGGGACAAGTTTGTACAAAAAAGCAGGCTATGAGTCTAGAGGTCGTGGATGC	Tb927.4.4130_rev	GGGGACCACTTTGTACAAGAAAGCTGGGTCTACTCAATTACAAGACGGCTGGC
10_hypothetical	B13	Tb927.8.8200_for	GGGGACAAGTTTGTACAAAAAAGCAGGCTATGAATCTTGAGGTCGTGGATGC	Tb927.8.8200_rev	GGGGACCACTTTGTACAAGAAAGCTGGGTCTACTCAACTACAAAACGACTGGCC
10_hypothetical	B14	Tb927.11.17020_for	GGGGACAAGTTTGTACAAAAAAGCAGGCTATGAATTTAAACGAAGTCGCTTCGGG	Tb927.11.17020_rev	GGGGACCACTTTGTACAAGAAAGCTGGGTCTATGAAGATGATGTCTTCATGAGATGAAG

10_hypothetical	B15	Tb927.6.410_for	GGGGACAAGTTTGTACAAAAAAGCAGGCTATGACACAACCAGATATATTCATGGGTG	Tb927.6.410_rev	GGGGACCACTTTGTACAAGAAAGCTGGGTTCAGCATCTGTAACGAACTACAACATT
10_hypothetical	B16	Tb927.4.5000_for	GGGGACAAGTTTGTACAAAAAAGCAGGCTATGTCAGCCCAGTTGCGGC	Tb927.4.5000_rev	GGGGACCACTTTGTACAAGAAAGCTGGGTTTACATGATGCAGCACGGTCC
10_hypothetical	B17	Tb927.11.12320_for	GGGGACAAGTTTGTACAAAAAAGCAGGCTATGATGCGGTTCACTAGGGATGG	Tb927.11.12320_rev	GGGGACCACTTTGTACAAGAAAGCTGGGTTTATATTCGCCCCTCCACTACATGT
10_hypothetical	B18	Tb927.8.7420_for	GGGGACAAGTTTGTACAAAAAAGCAGGCTATGTCAGCCCAGCTGCAGC	Tb927.8.7420_rev	GGGGACCACTTTGTACAAGAAAGCTGGGTTTACATGATGCAGCACGGTCC
10_hypothetical	B19	Tb927.3.5510_for	GGGGACAAGTTTGTACAAAAAAGCAGGCTATGCCACCACCGGCTCTAC	Tb927.3.5510_rev	GGGGACCACTTTGTACAAGAAAGCTGGGTTTACTTCATTTCAGATACTTGCACAGAACG
10_hypothetical	B20	Tb927.11.1420_for	GGGGACAAGTTTGTACAAAAAAGCAGGCTATGCCGGAAAAACCTGGTTCAC	Tb927.11.1420_rev	GGGGACCACTTTGTACAAGAAAGCTGGGTCTAACCAAATAACACACGACGGGT
10_hypothetical	B21	Tb927.4.4880_for	GGGGACAAGTTTGTACAAAAAAGCAGGCTATGATTCCGAACCGCTCCATGT	Tb927.4.4880_rev	GGGGACCACTTTGTACAAGAAAGCTGGGTCTAGGGCAACTGTTGCTGCTC
10_hypothetical	B22	Tb927.5.2660_for	GGGGACAAGTTTGTACAAAAAAGCAGGCTATGACGGAAACAGCGTTTCGA	Tb927.5.2660_rev	GGGGACCACTTTGTACAAGAAAGCTGGGTTTATGCCTTCCATCCACGGCG
10_hypothetical	B23	Tb927.10.14380_for	GGGGACAAGTTTGTACAAAAAAGCAGGCTATGCACGCGGTAAGCATTTCA	Tb927.10.14380_rev	GGGGACCACTTTGTACAAGAAAGCTGGGTTTACAAATCCACCATAACGCTCGC
10_hypothetical	B24	Tb927.5.4110_for	GGGGACAAGTTTGTACAAAAAAGCAGGCTATGGTAGGTTTGATCCCGTATGACA	Tb927.5.4110_rev	GGGGACCACTTTGTACAAGAAAGCTGGGTCTATACTTGTCGTTCCTGTGCCTTT
10_hypothetical	C1	Tb927.3.4580_for	GGGGACAAGTTTGTACAAAAAAGCAGGCTATGCTCTCTCAATTTGATGACAACCC	Tb927.3.4580_rev	GGGGACCACTTTGTACAAGAAAGCTGGGTTTAGGGTACAACTTCATCTCCATCCC
10_hypothetical	C2	Tb927.5.1180_for	GGGGACAAGTTTGTACAAAAAAGCAGGCTATGCAGTGGCCGTCATGGG	Tb927.5.1180_rev	GGGGACCACTTTGTACAAGAAAGCTGGGTCTACGCTGAACGGCCACAC
10_hypothetical	C3	Tb927.6.1950_for	GGGGACAAGTTTGTACAAAAAAGCAGGCTATGGCTGGCAGAGTTTCAGATCG	Tb927.6.1950_rev	GGGGACCACTTTGTACAAGAAAGCTGGGTCTAGCGCCTACGGGAGGC
10_hypothetical	C4	Tb927.11.2280_for	GGGGACAAGTTTGTACAAAAAAGCAGGCTATGACTACATTTGAGCACCTACCTGA	Tb927.11.2280_rev	GGGGACCACTTTGTACAAGAAAGCTGGGTCTAAGATAAGGGATTATGAGGTGCATAGTA
10_hypothetical	C5	Tb927.6.1070_for	GGGGACAAGTTTGTACAAAAAAGCAGGCTATGTCCCGAGCACTTGAAAGGC	Tb927.6.1070_rev	GGGGACCACTTTGTACAAGAAAGCTGGGTCTACCCTGATGCGATATTTGCCG
10_hypothetical	C6	Tb927.10.4200_for	GGGGACAAGTTTGTACAAAAAAGCAGGCTATGTCATCCAAGCAGGAGAGAATAGC	Tb927.10.4200_rev	GGGGACCACTTTGTACAAGAAAGCTGGGTTCAAGAATCTGTTGCAGGACGC
10_hypothetical	C7	Tb927.10.15840_for	GGGGACAAGTTTGTACAAAAAAGCAGGCTATGGATTTCCGAACTATCCGGCA	Tb927.10.15840_rev	GGGGACCACTTTGTACAAGAAAGCTGGGTTTAAAGCGATAGTGCATGTATCAAGTCA
10_hypothetical	C8	Tb927.10.14480_for	GGGGACAAGTTTGTACAAAAAAGCAGGCTATGGCCATTAAACCAAAGCGTATTGA	Tb927.10.14480_rev	GGGGACCACTTTGTACAAGAAAGCTGGGTTTACAACTTCTTACGCGTGAGACTC
10_hypothetical	C9	Tb927.1.3560_for	GGGGACAAGTTTGTACAAAAAAGCAGGCTATGTCCGTAGTTCTAGATGTTGTGACG	Tb927.1.3560_rev	GGGGACCACTTTGTACAAGAAAGCTGGGTTTAATGAGCCGATTTACCCACGTAG
10_hypothetical	C10	Tb927.11.390_for	GGGGACAAGTTTGTACAAAAAAGCAGGCTATGGGTAGTTTACCCTGCGACA	Tb927.11.390_rev	GGGGACCACTTTGTACAAGAAAGCTGGGTCTAAGCAATGGTTGCAGTATCGCC
10_hypothetical	C11	Tb927.9.12310_for	GGGGACAAGTTTGTACAAAAAAGCAGGCTATGGGATCTAGTGATGATGAGTGGC	Tb927.9.12310_rev	GGGGACCACTTTGTACAAGAAAGCTGGGTTTAGGGTGATTGCGTCGCTG
10_hypothetical	C12	Tb927.3.1900_for	GGGGACAAGTTTGTACAAAAAAGCAGGCTATGTCGAAGTCGCTCCCCC	Tb927.3.1900_rev	GGGGACCACTTTGTACAAGAAAGCTGGGTTTATGCTTCATCTTCTATCTCATCTTCACC
10_hypothetical	C13	Tb927.5.2020_for	GGGGACAAGTTTGTACAAAAAAGCAGGCTATGCTGATGGAAACGAAATTTCGC	Tb927.5.2020_rev	GGGGACCACTTTGTACAAGAAAGCTGGGTTTAACCTTCGCGGCGCCG
10_hypothetical	C14	Tb927.10.8120_for	GGGGACAAGTTTGTACAAAAAAGCAGGCTATGGAGGTTCTTGTTGAAAGGTGTC	Tb927.10.8120_rev	GGGGACCACTTTGTACAAGAAAGCTGGGTCTAAACAACAACAACAACTCCTCGC
10_hypothetical	C15	Tb927.10.9890_for	GGGGACAAGTTTGTACAAAAAAGCAGGCTATGAGTGTCTCCTTTTTTGTTCAGGAG	Tb927.10.9890_rev	GGGGACCACTTTGTACAAGAAAGCTGGGTTTACAGCCTCGCGTGCGG
10_hypothetical	C16	Tb927.3.2750_for	GGGGACAAGTTTGTACAAAAAAGCAGGCTATGCAGCGTCAGTGTGACG	Tb927.3.2750_rev	GGGGACCACTTTGTACAAGAAAGCTGGGTTCAACCGCGTTTTGTGTTTCG
10_hypothetical	C17	Tb927.10.11250_for	GGGGACAAGTTTGTACAAAAAAGCAGGCTATGTCCGACGTACCATACGATGT	Tb927.10.11250_rev	GGGGACCACTTTGTACAAGAAAGCTGGGTTCAGTGCAGAGATGATGCTATATCAGA
10_hypothetical	C18	Tb927.8.7580_for	GGGGACAAGTTTGTACAAAAAAGCAGGCTATGGAACATCTGAAAATTTTTCCGCG	Tb927.8.7580_rev	GGGGACCACTTTGTACAAGAAAGCTGGGTCTAGGGCAACTGCCGCTG
10_hypothetical	C19	Tb927.11.2820_for	GGGGACAAGTTTGTACAAAAAAGCAGGCTATGTGGCAGCCACGTCACG	Tb927.11.2820_rev	GGGGACCACTTTGTACAAGAAAGCTGGGTTCAGCGAAACTGGCACGG
10_hypothetical	C20	Tb927.2.2380_for	GGGGACAAGTTTGTACAAAAAAGCAGGCTATGCGCTCAGAAACACGGC	Tb927.2.2380_rev	GGGGACCACTTTGTACAAGAAAGCTGGGTCTACAATCCACTGCACACTTCTCC
10_hypothetical	C21	Tb927.11.1610_for	GGGGACAAGTTTGTACAAAAAAGCAGGCTATGCCGGTCCCACATCAGT	Tb927.11.1610_rev	GGGGACCACTTTGTACAAGAAAGCTGGGTTTACGTGTCTAAAACGCTGGCG
10_hypothetical	C22	Tb927.8.7090_for	GGGGACAAGTTTGTACAAAAAAGCAGGCTATGGATGATGGACCTGACCCTC	Tb927.8.7090_rev	GGGGACCACTTTGTACAAGAAAGCTGGGTTCATTCAACGGCAGTTGCAGAT
10_hypothetical	C23	Tb927.10.15160_for	GGGGACAAGTTTGTACAAAAAAGCAGGCTATGCTTGCCGTGAAGTACCTTC	Tb927.10.15160_rev	GGGGACCACTTTGTACAAGAAAGCTGGGTCTATGCGATGGGGATGAAGCC
10_hypothetical	C24	Tb927.8.5750_for	GGGGACAAGTTTGTACAAAAAAGCAGGCTATGCTGTCCTCTGCGCTCG	Tb927.8.5750_rev	GGGGACCACTTTGTACAAGAAAGCTGGGTTTAGAGCAGAGCCGCCTTCA
10_hypothetical	D1	Tb927.2.5440_for	GGGGACAAGTTTGTACAAAAAAGCAGGCTATGAGTTTCCGAAGACAAAGTTTTAGGG	Tb927.2.5440_rev	GGGGACCACTTTGTACAAGAAAGCTGGGTCTAACGGGGCTGGTTGGC
10_hypothetical	D2	Tb927.6.2800_for	GGGGACAAGTTTGTACAAAAAAGCAGGCTATGTCAGCCCGAAACAAAATGGG	Tb927.6.2800_rev	GGGGACCACTTTGTACAAGAAAGCTGGGTTTATGAATATCGGTGGCGGAACTG
10_hypothetical	D3	Tb927.8.610_for	GGGGACAAGTTTGTACAAAAAAGCAGGCTATGGGTCCCAAAAAGGCTGGT	Tb927.8.610_rev	GGGGACCACTTTGTACAAGAAAGCTGGGTTCAAAACTGTGTGCCAACCCC
10_hypothetical	D4	Tb927.11.16850_for	GGGGACAAGTTTGTACAAAAAAGCAGGCTATGTTTACAGACAGCATTCTTATGAGCC	Tb927.11.16850_rev	GGGGACCACTTTGTACAAGAAAGCTGGGTTCACTTCCCTACCTGGCGATG
10_hypothetical	D5	Tb927.9.4350_for	GGGGACAAGTTTGTACAAAAAAGCAGGCTATGACCGAATCTGAGAAAGCCCG	Tb927.9.4350_rev	GGGGACCACTTTGTACAAGAAAGCTGGGTTCATCCCCCACTCCATTTCACC
10_hypothetical	D6	Tb927.11.10580_for	GGGGACAAGTTTGTACAAAAAAGCAGGCTATGTCTCAATTTGCGACCCTGTC	Tb927.11.10580_rev	GGGGACCACTTTGTACAAGAAAGCTGGGTCTACTCGTCAGTTTCGTCAATATCCG
10_hypothetical	D7	Tb927.10.13470_for	GGGGACAAGTTTGTACAAAAAAGCAGGCTATGCAGCCCAACACGATGC	Tb927.10.13470_rev	GGGGACCACTTTGTACAAGAAAGCTGGGTCTACATGTTGTTAAGAATTTTCCACATTGG
10_hypothetical	D8	Tb927.4.5330_for	GGGGACAAGTTTGTACAAAAAAGCAGGCTATGAAGGGCCCGCTTTCCA	Tb927.4.5330_rev	GGGGACCACTTTGTACAAGAAAGCTGGGTTCACATCATCGATACCAGAGCACG
10_hypothetical	D9	Tb927.9.13570_for	GGGGACAAGTTTGTACAAAAAAGCAGGCTATGACTGAAGAGAAGCAGCAGAAGC	Tb927.9.13570_rev	GGGGACCACTTTGTACAAGAAAGCTGGGTCTAATTGGGATGCCGTCTACCCA
10_hypothetical	D10	Tb927.8.1070_for	GGGGACAAGTTTGTACAAAAAAGCAGGCTATGCACAGTGTTACTGTCCTTTCTC	Tb927.8.1070_rev	GGGGACCACTTTGTACAAGAAAGCTGGGTTCAGCACTTTCTTTCCACACCAC
10_hypothetical	D11	Tb927.10.8610_for	GGGGACAAGTTTGTACAAAAAAGCAGGCTATGCGCCGTTGCCTCTTC	Tb927.10.8610_rev	GGGGACCACTTTGTACAAGAAAGCTGGGTTCATTTCAACATGTTCCCCCTCG
10_hypothetical	D12	Tb927.8.6330_for	GGGGACAAGTTTGTACAAAAAAGCAGGCTATGTCGAAGGCCCCTGAGC	Tb927.8.6330_rev	GGGGACCACTTTGTACAAGAAAGCTGGGTTCAAGACCCATCAGCCTCTAGC
10_hypothetical	D13	Tb927.11.9400_for	GGGGACAAGTTTGTACAAAAAAGCAGGCTATGCAACAGCAAAACCGCAC	Tb927.11.9400_rev	GGGGACCACTTTGTACAAGAAAGCTGGGTTCAATCCCTGCTTCCTTCACTGC
10_hypothetical	D14	Tb927.4.5320_for	GGGGACAAGTTTGTACAAAAAAGCAGGCTATGGATAATGGCCCTCATCTCTTCA	Tb927.4.5320_rev	GGGGACCACTTTGTACAAGAAAGCTGGGTTCACTCAACAGCAGTTGCGG
10_hypothetical	D15	Tb927.11.4340_for	GGGGACAAGTTTGTACAAAAAAGCAGGCTATGAACTTTGACGATCTCTTCGCG	Tb927.11.4340_rev	GGGGACCACTTTGTACAAGAAAGCTGGGTCTACGGGTCAAAGGCTTGTGC
10_hypothetical	D16	Tb927.8.1540_for	GGGGACAAGTTTGTACAAAAAAGCAGGCTATGTCAACTCCATCTGTGGTCGG	Tb927.8.1540_rev	GGGGACCACTTTGTACAAGAAAGCTGGGTCTACTTTGAGTCGGAAACGCTGG
10_hypothetical	D17	Tb927.7.1580_for	GGGGACAAGTTTGTACAAAAAAGCAGGCTATGATTGCTGATGCGGAATGGAG	Tb927.7.1580_rev	GGGGACCACTTTGTACAAGAAAGCTGGGTCTATCCCTCCTCATCATCATCTTTGC
10_hypothetical	D18	Tb927.11.16910_for	GGGGACAAGTTTGTACAAAAAAGCAGGCTATGGTGAAAGTTTTATGTCTGCACGG	Tb927.11.16910_rev	GGGGACCACTTTGTACAAGAAAGCTGGGTTCACACCTTTGGCCCCACA
10_hypothetical	D19	Tb927.5.3850_for	GGGGACAAGTTTGTACAAAAAAGCAGGCTATGGGGATAATAGGTCTGCGCA	Tb927.5.3850_rev	GGGGACCACTTTGTACAAGAAAGCTGGGTTCATCTTTGATGGTCCTGTACCTTCC
10_hypothetical	D20	Tb927.11.8370_for	GGGGACAAGTTTGTACAAAAAAGCAGGCTATGTCTGATTCGCACCACAGC	Tb927.11.8370_rev	GGGGACCACTTTGTACAAGAAAGCTGGGTTCACCTTTTCTTGTACTGGCTGTG
10_hypothetical	D21	Tb927.2.4000_for	GGGGACAAGTTTGTACAAAAAAGCAGGCTATGTCACAACGAGTGCCACG	Tb927.2.4000_rev	GGGGACCACTTTGTACAAGAAAGCTGGGTCTAACGCCCTTTTTGTGAAAGAAAGC
10_hypothetical	D22	Tb927.4.3170_for	GGGGACAAGTTTGTACAAAAAAGCAGGCTATGCAGGGAGACACTAAAACTATGGG	Tb927.4.3170_rev	GGGGACCACTTTGTACAAGAAAGCTGGGTTTACAAAACTGAGCCCATGGAGGG
10_hypothetical	D23	Tb927.7.3230_for	GGGGACAAGTTTGTACAAAAAAGCAGGCTATGGATGGACAGCGTGCAAC	Tb927.7.3230_rev	GGGGACCACTTTGTACAAGAAAGCTGGGTTCAGTTGCTTGCATGACAAAGGC
10_hypothetical	D24	Tb927.11.5100_for	GGGGACAAGTTTGTACAAAAAAGCAGGCTATGATATCAGAGAGCCAGATGTTTAGCC	Tb927.11.5100_rev	GGGGACCACTTTGTACAAGAAAGCTGGGTTTACAGAGTTAACGTTAGAGATGTATCAGT
10_hypothetical	E1	Tb927.7.6610_for	GGGGACAAGTTTGTACAAAAAAGCAGGCTATGTTCTCCGTTGAGTTTGTAAGTGG	Tb927.7.6610_rev	GGGGACCACTTTGTACAAGAAAGCTGGGTTTACTTCACTTTTTGTGGACGATGCG
10_hypothetical	E2	Tb927.9.15330_for	GGGGACAAGTTTGTACAAAAAAGCAGGCTATGGCGCGAGCAACTGCT	Tb927.9.15330_rev	GGGGACCACTTTGTACAAGAAAGCTGGGTTTAGTATTTATTCAACATGAGAAGTAGATG
10_hypothetical	E3	Tb927.11.10630_for	GGGGACAAGTTTGTACAAAAAAGCAGGCTATGAAAGTTATTACACATGAGCAAGTAGCG	Tb927.11.10630_rev	GGGGACCACTTTGTACAAGAAAGCTGGGTCTAAAAGCGCCTCTTTATTTCGTTGC
10_hypothetical	E4	Tb927.8.2860_for	GGGGACAAGTTTGTACAAAAAAGCAGGCTATGTCTGTTAGCGCCCCAACG	Tb927.8.2860_rev	GGGGACCACTTTGTACAAGAAAGCTGGGTCTAAGCAAATGGGTCAAACTCCGC
10_hypothetical	E5	Tb927.7.5210_for	GGGGACAAGTTTGTACAAAAAAGCAGGCTATGAGCCTGGTACAGAACCAAGT	Tb927.7.5210_rev	GGGGACCACTTTGTACAAGAAAGCTGGGTTCAGTGACTCTCTCCGGTGGC
10_hypothetical	E6	Tb927.6.1290_for	GGGGACAAGTTTGTACAAAAAAGCAGGCTATGGATCTCTTCCCCAAGCATTTG	Tb927.6.1290_rev	GGGGACCACTTTGTACAAGAAAGCTGGGTTTAAAGTGACTCTATAAAGAGCAGAACTTC
10_hypothetical	E7	Tb927.8.6360_for	GGGGACAAGTTTGTACAAAAAAGCAGGCTATGTTTTACTCAGGGTGGCAGTTTC	Tb927.8.6360_rev	GGGGACCACTTTGTACAAGAAAGCTGGGTCTAGGCAAAAACCGCCCTATAAACG
10_hypothetical	E8	Tb927.3.5450_for	GGGGACAAGTTTGTACAAAAAAGCAGGCTATGAGTGATTACGGAGATTCTTTTGATAGC	Tb927.3.5450_rev	GGGGACCACTTTGTACAAGAAAGCTGGGTTTACACATTCCTTGCCTCTTCAACAC
10_hypothetical	E9	Tb927.8.7760_for	GGGGACAAGTTTGTACAAAAAAGCAGGCTATGGGTGCTGCTCCAAGCA	Tb927.8.7760_rev	GGGGACCACTTTGTACAAGAAAGCTGGGTTTATCTATCTGCGCAATATCTCCAGTCC
10_hypothetical	E10	Tb927.9.8040_for	GGGGACAAGTTTGTACAAAAAAGCAGGCTATGCAGGTTTTATTGAAACTTCGGC	Tb927.9.8040_rev	GGGGACCACTTTGTACAAGAAAGCTGGGTTCAATGGCATATGAGAAACGACTCC
10_hypothetical	E11	Tb927.4.4570_for	GGGGACAAGTTTGTACAAAAAAGCAGGCTATGGGTGTTGCACCCAGTCG	Tb927.4.4570_rev	GGGGACCACTTTGTACAAGAAAGCTGGGTTTAGGACTTCCTCCAGTCCCAAATAA
10_hypothetical	E12	Tb927.10.12720_for	GGGGACAAGTTTGTACAAAAAAGCAGGCTATGGAGACGCACCTCGGC	Tb927.10.12720_rev	GGGGACCACTTTGTACAAGAAAGCTGGGTTTAGCGCCATGGTCGAGGC
10_hypothetical	E13	Tb927.10.12340_for	GGGGACAAGTTTGTACAAAAAAGCAGGCTATGAGAAGATGGCGCCCTGT	Tb927.10.12340_rev	GGGGACCACTTTGTACAAGAAAGCTGGGTTTAGATGTCCTCCTTAACAGTGCAGT
10_hypothetical	E14	Tb927.6.4640_for	GGGGACAAGTTTGTACAAAAAAGCAGGCTATGCCCAAGAAAACTCGCGT	Tb927.6.4640_rev	GGGGACCACTTTGTACAAGAAAGCTGGGTTCAAAAACGCATCGGTGGTCC
10_hypothetical	E15	Tb927.10.5890_for	GGGGACAAGTTTGTACAAAAAAGCAGGCTATGCAAAGGGTAGGTAGTGAAAGGG	Tb927.10.5890_rev	GGGGACCACTTTGTACAAGAAAGCTGGGTTCAATGATTCTTTGACCTTCTCCCCC
10_hypothetical	E16	Tb927.7.5430_for	GGGGACAAGTTTGTACAAAAAAGCAGGCTATGTCTGCCTCTCGATCTCCC	Tb927.7.5430_rev	GGGGACCACTTTGTACAAGAAAGCTGGGTCTACCTTCTCCCACCCCGC
10_hypothetical	E18	Tb927.1.3910_for	GGGGACAAGTTTGTACAAAAAAGCAGGCTATGCATCAGCAAGACCCCACC	Tb927.1.3910_rev	GGGGACCACTTTGTACAAGAAAGCTGGGTTCAAGCTCGTTCTCCCTGACG
10_hypothetical	E19	Tb927.9.13520_for	GGGGACAAGTTTGTACAAAAAAGCAGGCTATGGGCAGCGTGGGACAG	Tb927.9.13520_rev	GGGGACCACTTTGTACAAGAAAGCTGGGTTTACTGGATACCATAGACATGATAGAGACG
10_hypothetical	E20	Tb927.4.1160_for	GGGGACAAGTTTGTACAAAAAAGCAGGCTATGCGTCATCTCCACACCGC	Tb927.4.1160_rev	GGGGACCACTTTGTACAAGAAAGCTGGGTTCACGACCATTTTTAAGGAAAGGGT
10_hypothetical	E21	Tb927.4.1320_for	GGGGACAAGTTTGTACAAAAAAGCAGGCTATGCGCCTTTCCCGCTATG	Tb927.4.1320_rev	GGGGACCACTTTGTACAAGAAAGCTGGGTCTATCTCGAGGCGGATAGAAGCA
10_hypothetical	E22	Tb927.10.8810_for	GGGGACAAGTTTGTACAAAAAAGCAGGCTATGATCCCCGTGGCAGGC	Tb927.10.8810_rev	GGGGACCACTTTGTACAAGAAAGCTGGGTTTAGTTGAGAGTTGAAGGTGTTGTAAAGT
10_hypothetical	E23	Tb927.11.3360_for	GGGGACAAGTTTGTACAAAAAAGCAGGCTATGTCTTCGTTGACATGTGAGGAGC	Tb927.11.3360_rev	GGGGACCACTTTGTACAAGAAAGCTGGGTTTATTCCTTCTTCTTGGGTCTCTTCTTAGC
10_hypothetical	E24	Tb927.10.420_for	GGGGACAAGTTTGTACAAAAAAGCAGGCTATGAACGAAAGTATTATTCAAGTGCCACT	Tb927.10.420_rev	GGGGACCACTTTGTACAAGAAAGCTGGGTTCAATCAGCATCTTCAGCAACTCTG
10_hypothetical	F1	Tb927.8.4370_for	GGGGACAAGTTTGTACAAAAAAGCAGGCTATGGCTGACAGTAACAACAGCG	Tb927.8.4370_rev	GGGGACCACTTTGTACAAGAAAGCTGGGTTCACTTTGGTGCACTCTGGATCA
10_hypothetical	F2	Tb927.11.16840.1_for	GGGGACAAGTTTGTACAAAAAAGCAGGCTATGGCCTCGCTACTTCAGCA	Tb927.11.16840.1_rev	GGGGACCACTTTGTACAAGAAAGCTGGGTCTAAGCAACTACCTCCTCACTCCT
10_hypothetical	F3	Tb927.1.3270_for	GGGGACAAGTTTGTACAAAAAAGCAGGCTATGAGTGCATTTGCGCTGGC	Tb927.1.3270_rev	GGGGACCACTTTGTACAAGAAAGCTGGGTTTAATTTCCCTTCCTTCCGCCTGC
10_hypothetical	F4	Tb927.7.7220_for	GGGGACAAGTTTGTACAAAAAAGCAGGCTATGAGCACCAGTTCGTCTATATTCTTTG	Tb927.7.7220_rev	GGGGACCACTTTGTACAAGAAAGCTGGGTCTATTGTTCGTGAGAGGCAGGC
10_hypothetical	F5	Tb927.1.3940_for	GGGGACAAGTTTGTACAAAAAAGCAGGCTATGGCAACACCTCGGCAGG	Tb927.1.3940_rev	GGGGACCACTTTGTACAAGAAAGCTGGGTTCAAAGAATAGACATGGTAGCAAAGCG
10_hypothetical	F6	Tb927.8.5650_for	GGGGACAAGTTTGTACAAAAAAGCAGGCTATGGATTTTGTTTTCCCAGTATTTGTTCC	Tb927.8.5650_rev	GGGGACCACTTTGTACAAGAAAGCTGGGTTCACTCCGACGTTAGAGCTTGA
10_hypothetical	F7	Tb927.8.3950_for	GGGGACAAGTTTGTACAAAAAAGCAGGCTATGGGGAGCGTTGATCATCCG	Tb927.8.3950_rev	GGGGACCACTTTGTACAAGAAAGCTGGGTTTATACCTTCGTAATACCCGACACAGT
10_hypothetical	F8	Tb927.5.2520_for	GGGGACAAGTTTGTACAAAAAAGCAGGCTATGGTGCCATCCATCCCTCAA	Tb927.5.2520_rev	GGGGACCACTTTGTACAAGAAAGCTGGGTTCAGTCGGCCATAATGCGAGT
10_hypothetical	F9	Tb927.5.1070_for	GGGGACAAGTTTGTACAAAAAAGCAGGCTATGTCTGCAATCAAAGAACTTGTCGA	Tb927.5.1070_rev	GGGGACCACTTTGTACAAGAAAGCTGGGTCTAATGAGGGCCTCGCAAATCTC
10_hypothetical	F10	Tb927.3.4270_for	GGGGACAAGTTTGTACAAAAAAGCAGGCTATGGGGCATCCGAGCGAC	Tb927.3.4270_rev	GGGGACCACTTTGTACAAGAAAGCTGGGTCTACATGAGGCTGGCAAGATCCT
10_hypothetical	F11	Tb927.10.2380_for	GGGGACAAGTTTGTACAAAAAAGCAGGCTATGACTACAGAGGTCAGTAATGACGG	Tb927.10.2380_rev	GGGGACCACTTTGTACAAGAAAGCTGGGTTCACGACTTTCCAAGGGCGG
10_hypothetical	F12	Tb927.11.6910_for	GGGGACAAGTTTGTACAAAAAAGCAGGCTATGACTATCCTACACCACCCGG	Tb927.11.6910_rev	GGGGACCACTTTGTACAAGAAAGCTGGGTTCAAACGGCAGGTGTTGCTT
10_hypothetical	F13	Tb927.1.830_for	GGGGACAAGTTTGTACAAAAAAGCAGGCTATGGAACCGGTGGACGGG	Tb927.1.830_rev	GGGGACCACTTTGTACAAGAAAGCTGGGTTTAATTCCCTTTAAGCACCCGTCTC
10_hypothetical	F14	Tb927.11.16920_for	GGGGACAAGTTTGTACAAAAAAGCAGGCTATGCATCACATACCCATCTCGTCC	Tb927.11.16920_rev	GGGGACCACTTTGTACAAGAAAGCTGGGTTTACGAGCCCACACCTAACCA
10_hypothetical	F15	Tb927.11.12510_for	GGGGACAAGTTTGTACAAAAAAGCAGGCTATGACGAGTGTTAAGGAAACAAAGGAG	Tb927.11.12510_rev	GGGGACCACTTTGTACAAGAAAGCTGGGTTTAATCTACATTTTTTCTTTGCGCCGC
10_hypothetical	F16	Tb927.11.5750_for	GGGGACAAGTTTGTACAAAAAAGCAGGCTATGGTGCGGTCCCAGTCTC	Tb927.11.5750_rev	GGGGACCACTTTGTACAAGAAAGCTGGGTTCAAATGATTTCCCCGCTATTTCCA
10_hypothetical	F17	Tb927.4.2900_for	GGGGACAAGTTTGTACAAAAAAGCAGGCTATGACTGAAAGTGACATGTATCGGGG	Tb927.4.2900_rev	GGGGACCACTTTGTACAAGAAAGCTGGGTTCAATCTGGCAGCTGACTGTCC
10_hypothetical	F18	Tb927.11.10720_for	GGGGACAAGTTTGTACAAAAAAGCAGGCTATGGTGTACAACAAAGTGCTGCG	Tb927.11.10720_rev	GGGGACCACTTTGTACAAGAAAGCTGGGTTCACCACACAATCTCCCCAGC
10_hypothetical	F19	Tb927.10.14430_for	GGGGACAAGTTTGTACAAAAAAGCAGGCTATGTGCACCACTAGGTGTCTTCA	Tb927.10.14430_rev	GGGGACCACTTTGTACAAGAAAGCTGGGTTTAACATGTCTCTTTTTCCTTCGCACC
10_hypothetical	F20	Tb927.3.2430_for	GGGGACAAGTTTGTACAAAAAAGCAGGCTATGAAACGATGCAACAGTGAGTCT	Tb927.3.2430_rev	GGGGACCACTTTGTACAAGAAAGCTGGGTTTATAAAATAGATGCAATAAATGCCGCAGC
10_hypothetical	F21	Tb927.7.2630_for	GGGGACAAGTTTGTACAAAAAAGCAGGCTATGCCATGGCTAACTCCACTACG	Tb927.7.2630_rev	GGGGACCACTTTGTACAAGAAAGCTGGGTCTACCGACCCTTGACTCGAGG
10_hypothetical	F22	Tb927.11.4500_for	GGGGACAAGTTTGTACAAAAAAGCAGGCTATGAGTCTCTCCAATCAACTTCCGC	Tb927.11.4500_rev	GGGGACCACTTTGTACAAGAAAGCTGGGTTTATTCCAGGAGTGTGGAGAAAAGC
10_hypothetical	F23	Tb927.9.7760_for	GGGGACAAGTTTGTACAAAAAAGCAGGCTATGCAGGTTTGCCCATTAGCAC	Tb927.9.7760_rev	GGGGACCACTTTGTACAAGAAAGCTGGGTTTACTGAAAAGGAGGCGGTAGCT
10_hypothetical	F24	Tb927.6.2350_for	GGGGACAAGTTTGTACAAAAAAGCAGGCTATGCTGTGCAGCGGCCGC	Tb927.6.2350_rev	GGGGACCACTTTGTACAAGAAAGCTGGGTTCACATCCCCGCACCCGT
10_hypothetical	G2	Tb927.11.14190_for	GGGGACAAGTTTGTACAAAAAAGCAGGCTATGTCTTTTCTTGTGTATGCCGTTGA	Tb927.11.14190_rev	GGGGACCACTTTGTACAAGAAAGCTGGGTTCAGTTATCCTCGTCGCTTTCGT
10_hypothetical	G3	Tb927.5.2710_for	GGGGACAAGTTTGTACAAAAAAGCAGGCTATGAGCTCTTCACAAGGAGAAATGC	Tb927.5.2710_rev	GGGGACCACTTTGTACAAGAAAGCTGGGTTCACTCGTATTTGACCGCCTCA
10_hypothetical	G4	Tb927.11.2600_for	GGGGACAAGTTTGTACAAAAAAGCAGGCTATGCTTATTACTGAAAATCTATTTGCCTCA	Tb927.11.2600_rev	GGGGACCACTTTGTACAAGAAAGCTGGGTTCACTTAAATACCACTCCACTACCACC
10_hypothetical	G5	Tb927.5.4520_for	GGGGACAAGTTTGTACAAAAAAGCAGGCTATGGAGTCTCCTGCGAAAGGT	Tb927.5.4520_rev	GGGGACCACTTTGTACAAGAAAGCTGGGTCTAATTCGATCTTTGCCTCTTTGCAC
10_hypothetical	G6	Tb927.11.5160_for	GGGGACAAGTTTGTACAAAAAAGCAGGCTATGAAGGAGTTAAACGCCATTCTTCA	Tb927.11.5160_rev	GGGGACCACTTTGTACAAGAAAGCTGGGTTCAGCTTGGACCTAGGCCC
10_hypothetical	G7	Tb927.10.13200_for	GGGGACAAGTTTGTACAAAAAAGCAGGCTATGACACTGACTCTTCTTAGGAGGG	Tb927.10.13200_rev	GGGGACCACTTTGTACAAGAAAGCTGGGTTCAGACGCGCGACATCGA
10_hypothetical	G8	Tb927.5.2600_for	GGGGACAAGTTTGTACAAAAAAGCAGGCTATGTGCCACCGTCTCGTCC	Tb927.5.2600_rev	GGGGACCACTTTGTACAAGAAAGCTGGGTTTATTGCGCCACGGGAAAACG
10_hypothetical	G9	Tb927.10.12910_for	GGGGACAAGTTTGTACAAAAAAGCAGGCTATGTTCCCCCCGGTAGCAC	Tb927.10.12910_rev	GGGGACCACTTTGTACAAGAAAGCTGGGTTCAATCAAGGAAATCGTAGACTTCATTGT
10_hypothetical	G10	Tb927.11.3300_for	GGGGACAAGTTTGTACAAAAAAGCAGGCTATGGCAGAGGTGAAGCTTCCG	Tb927.11.3300_rev	GGGGACCACTTTGTACAAGAAAGCTGGGTTCAACTTTCGTCAGGGAAAATGGTT
10_hypothetical	G11	Tb927.11.7690_for	GGGGACAAGTTTGTACAAAAAAGCAGGCTATGCGGCAAATCCATCTAAGTAGTAAC	Tb927.11.7690_rev	GGGGACCACTTTGTACAAGAAAGCTGGGTCTAAACAAAAGTCCTTTTAGCCAATTCCTG
10_hypothetical	G12	Tb927.4.3120_for	GGGGACAAGTTTGTACAAAAAAGCAGGCTATGGAAATTGAAGGGCATGAGACC	Tb927.4.3120_rev	GGGGACCACTTTGTACAAGAAAGCTGGGTTCACGATATCATGCTAAATAGTTCACTGC
10_hypothetical	G13	Tb927.10.12590_for	GGGGACAAGTTTGTACAAAAAAGCAGGCTATGGGTGACGACACGACCA	Tb927.10.12590_rev	GGGGACCACTTTGTACAAGAAAGCTGGGTCTACAACCAGAGTTCGTTCCTATCTGG
10_hypothetical	G14	Tb927.7.480_for	GGGGACAAGTTTGTACAAAAAAGCAGGCTATGCGACCCTCCAGGCGT	Tb927.7.480_rev	GGGGACCACTTTGTACAAGAAAGCTGGGTTCAACTGCCAATGCGGCTC
10_hypothetical	G15	Tb927.4.1390_for	GGGGACAAGTTTGTACAAAAAAGCAGGCTATGATGTCACCGTCCCTCGC	Tb927.4.1390_rev	GGGGACCACTTTGTACAAGAAAGCTGGGTTCAAGGATTACCTTCGACCTCAGG
10_hypothetical	G16	Tb927.7.4330_for	GGGGACAAGTTTGTACAAAAAAGCAGGCTATGACGGCACTCGACTTTCAGT	Tb927.7.4330_rev	GGGGACCACTTTGTACAAGAAAGCTGGGTTCATACACTACTACCACCAGAACTCAA
10_hypothetical	G17	Tb927.5.2420_for	GGGGACAAGTTTGTACAAAAAAGCAGGCTATGAGACACTGCCATATTATTCGGTATG	Tb927.5.2420_rev	GGGGACCACTTTGTACAAGAAAGCTGGGTCTACAGGATGAAGTAATCCTTTTCCTTTGT
10_hypothetical	G18	Tb927.11.6660_for	GGGGACAAGTTTGTACAAAAAAGCAGGCTATGCTGCGGTACGTTAAAAGAATTG	Tb927.11.6660_rev	GGGGACCACTTTGTACAAGAAAGCTGGGTTCAGAACACAAATACACTTTGAGCATCG
10_hypothetical	G19	Tb927.11.14370_for	GGGGACAAGTTTGTACAAAAAAGCAGGCTATGTCCAAGGAGGCTCCACAT	Tb927.11.14370_rev	GGGGACCACTTTGTACAAGAAAGCTGGGTCTACTTTCCTCCCTTGTTGAAGCG
10_hypothetical	G20	Tb927.2.4780_for	GGGGACAAGTTTGTACAAAAAAGCAGGCTATGTCCATCACAGTTGACACTATGAGT	Tb927.2.4780_rev	GGGGACCACTTTGTACAAGAAAGCTGGGTTTACCTCCCGGGGCGTACA
10_hypothetical	G21	Tb927.11.10610_for	GGGGACAAGTTTGTACAAAAAAGCAGGCTATGCAATATATTATTCACGACCCACACA	Tb927.11.10610_rev	GGGGACCACTTTGTACAAGAAAGCTGGGTTTACTCAATGGGAGGAATGACCAATTC
10_hypothetical	G22	Tb927.8.8270_for	GGGGACAAGTTTGTACAAAAAAGCAGGCTATGTCTCACAAGCGGGTGGC	Tb927.8.8270_rev	GGGGACCACTTTGTACAAGAAAGCTGGGTTTATTCATCCCCCACGACCCTC
10_hypothetical	G23	Tb927.11.16330_for	GGGGACAAGTTTGTACAAAAAAGCAGGCTATGGAAAAGAGAACCAACACGTTCA	Tb927.11.16330_rev	GGGGACCACTTTGTACAAGAAAGCTGGGTTTACTGTGCTGTTTCTTTCTGCTGC
10_hypothetical	G24	Tb927.8.5400_for	GGGGACAAGTTTGTACAAAAAAGCAGGCTATGTCTTTTCACACCGACGTGTT	Tb927.8.5400_rev	GGGGACCACTTTGTACAAGAAAGCTGGGTTTACGTCGCTGATACCCCTAATCT
10_hypothetical	H1	Tb927.4.4060_for	GGGGACAAGTTTGTACAAAAAAGCAGGCTATGTCTCAAAAACATATCTTCCAGCGG	Tb927.4.4060_rev	GGGGACCACTTTGTACAAGAAAGCTGGGTCTAACCTCCACTTATCTGACTGCTTTC
10_hypothetical	H2	Tb927.7.4600_for	GGGGACAAGTTTGTACAAAAAAGCAGGCTATGGTGTCGCGAGCAAAGC	Tb927.7.4600_rev	GGGGACCACTTTGTACAAGAAAGCTGGGTTTACGGAACACAACCAGCGTC

10_hypothetical	H3	Tb927.10.13590_for	GGGGACAAGTTTGTACAAAAAAGCAGGCTATGATAGACACAAAGCAGTTGTGCT	Tb927.10.13590_rev	GGGGACCACTTTGTACAAGAAAGCTGGGTTCAGCAGCCTGTGATCCCTC
10_hypothetical	H4	Tb927.10.1310_for	GGGGACAAGTTTGTACAAAAAAGCAGGCTATGCAGACAGCCCCCCCT	Tb927.10.1310_rev	GGGGACCACTTTGTACAAGAAAGCTGGGTTCACCCCAAAAGACCTTCTGCC
10_hypothetical	H5	Tb927.3.5290_for	GGGGACAAGTTTGTACAAAAAAGCAGGCTATGGGTCGGGCAACAGAGG	Tb927.3.5290_rev	GGGGACCACTTTGTACAAGAAAGCTGGGTTTAACTTCTACACACCCACGATAGTCC
10_hypothetical	H6	Tb927.10.3310_for	GGGGACAAGTTTGTACAAAAAAGCAGGCTATGAGTACCGAGAACGAGGGC	Tb927.10.3310_rev	GGGGACCACTTTGTACAAGAAAGCTGGGTTCACCGAGTTAGCGCAAAACC
10_hypothetical	H7	Tb927.4.4480_for	GGGGACAAGTTTGTACAAAAAAGCAGGCTATGAGCACGCGGTGCGCT	Tb927.4.4480_rev	GGGGACCACTTTGTACAAGAAAGCTGGGTTCATCCCTCATGTTGCCGAAGG
10_hypothetical	H8	Tb927.8.3920_for	GGGGACAAGTTTGTACAAAAAAGCAGGCTATGTCTGTTGAGGAAGTTGATACATTACTT	Tb927.8.3920_rev	GGGGACCACTTTGTACAAGAAAGCTGGGTCTACTTTAATTTATACCACTGATGCCGGC
10_hypothetical	H9	Tb927.8.6260_for	GGGGACAAGTTTGTACAAAAAAGCAGGCTATGATAGAAGTAGTGAAATCAACCACAAAC	Tb927.8.6260_rev	GGGGACCACTTTGTACAAGAAAGCTGGGTTCAGCGGTATGGCCGGCG
10_hypothetical	H11	Tb927.11.13520_for	GGGGACAAGTTTGTACAAAAAAGCAGGCTATGGGCTCTGGGTTTGTTACTAGG	Tb927.11.13520_rev	GGGGACCACTTTGTACAAGAAAGCTGGGTCTATTGTTCAGGTTTGCCGGTATGA
10_hypothetical	H12	Tb927.7.2390_for	GGGGACAAGTTTGTACAAAAAAGCAGGCTATGTATCGGCCTCGTGGCG	Tb927.7.2390_rev	GGGGACCACTTTGTACAAGAAAGCTGGGTTTACTTTATAAGCTGCCGAAGAATAAGCG
10_hypothetical	H13	Tb927.10.2480_for	GGGGACAAGTTTGTACAAAAAAGCAGGCTATGCAGTCTTGTATGATGCATATGGAA	Tb927.10.2480_rev	GGGGACCACTTTGTACAAGAAAGCTGGGTTCAAGCCCACGTCCGCGT
10_hypothetical	H14	Tb927.4.4760_for	GGGGACAAGTTTGTACAAAAAAGCAGGCTATGAAAACGAGTCTCCCGCCT	Tb927.4.4760_rev	GGGGACCACTTTGTACAAGAAAGCTGGGTTTACGCTCTGTAAACAAACTGCTCC
10_hypothetical	H15	Tb927.7.5090_for	GGGGACAAGTTTGTACAAAAAAGCAGGCTATGCAACACACCGAGGCG	Tb927.7.5090_rev	GGGGACCACTTTGTACAAGAAAGCTGGGTCTAGTCTGCATTCACCATGGTCTC
10_hypothetical	H16	Tb927.11.15890_for	GGGGACAAGTTTGTACAAAAAAGCAGGCTATGACCTTTAGTAGCGCCAAACG	Tb927.11.15890_rev	GGGGACCACTTTGTACAAGAAAGCTGGGTTTACCTGATTTTACGAAGGGACTGTTC
10_hypothetical	H17	Tb927.11.6460_for	GGGGACAAGTTTGTACAAAAAAGCAGGCTATGTCTCTAGTAGGAGACGTACTTCAAC	Tb927.11.6460_rev	GGGGACCACTTTGTACAAGAAAGCTGGGTTTATTCAAAACGCGGATCATCAACCG
10_hypothetical	H18	Tb927.2.2950_for	GGGGACAAGTTTGTACAAAAAAGCAGGCTATGCGTGCTTCCAGTGCAA	Tb927.2.2950_rev	GGGGACCACTTTGTACAAGAAAGCTGGGTTCACGCGCCTCCACCATC
10_hypothetical	H19	Tb927.7.3130_for	GGGGACAAGTTTGTACAAAAAAGCAGGCTATGGACGTGGCAGCATTACG	Tb927.7.3130_rev	GGGGACCACTTTGTACAAGAAAGCTGGGTTCAGCCACCCTTACCCTTCTG
10_hypothetical	H20	Tb927.11.5640_for	GGGGACAAGTTTGTACAAAAAAGCAGGCTATGCAGCGCTCACCTCAATC	Tb927.11.5640_rev	GGGGACCACTTTGTACAAGAAAGCTGGGTTCAGTGCTGCGCTACCCT
10_hypothetical	H21	Tb927.10.9280_for	GGGGACAAGTTTGTACAAAAAAGCAGGCTATGGTATCCGCTTCGAAGTTGC	Tb927.10.9280_rev	GGGGACCACTTTGTACAAGAAAGCTGGGTCTAGTAGTAGAGGACCGTCCCGT
10_hypothetical	H22	Tb927.7.980_for	GGGGACAAGTTTGTACAAAAAAGCAGGCTATGAAAGTCGCAGGTTCAATTCTCG	Tb927.7.980_rev	GGGGACCACTTTGTACAAGAAAGCTGGGTTTAAAGACCCTGTGCATGTAGAATCTC
10_hypothetical	H23	Tb927.10.12950_for	GGGGACAAGTTTGTACAAAAAAGCAGGCTATGGGGGAGGCCATGGGC	Tb927.10.12950_rev	GGGGACCACTTTGTACAAGAAAGCTGGGTTCACAGGTAGCCATAGTGCCG
10_hypothetical	H24	Tb927.11.15940_for	GGGGACAAGTTTGTACAAAAAAGCAGGCTATGAGCCGGCCTGATAAGAGAC	Tb927.11.15940_rev	GGGGACCACTTTGTACAAGAAAGCTGGGTTCACTCCCACGGTGGCAG
10_hypothetical	I1	Tb927.11.10210_for	GGGGACAAGTTTGTACAAAAAAGCAGGCTATGAATCCATCTGGTCCCAGTGG	Tb927.11.10210_rev	GGGGACCACTTTGTACAAGAAAGCTGGGTCTACTCTGCCTTCCTAGCCTTATCC
10_hypothetical	I2	Tb927.5.670_for	GGGGACAAGTTTGTACAAAAAAGCAGGCTATGCAAGCAGCAAACATTTCGG	Tb927.5.670_rev	GGGGACCACTTTGTACAAGAAAGCTGGGTTTACTCCACTGCTGAGATGAGTCTC
10_hypothetical	I3	Tb927.11.16700_for	GGGGACAAGTTTGTACAAAAAAGCAGGCTATGTTAACTCGCGTGATTCATGACT	Tb927.11.16700_rev	GGGGACCACTTTGTACAAGAAAGCTGGGTTTATAGGTGGAACTCCTCTACGTCCT
10_hypothetical	I4	Tb927.3.2490_for	GGGGACAAGTTTGTACAAAAAAGCAGGCTATGGATGCGAAACTTGTGTTCTGT	Tb927.3.2490_rev	GGGGACCACTTTGTACAAGAAAGCTGGGTTCAGTACGGGAGCGGTATGAC
10_hypothetical	I5	Tb927.9.11890_for	GGGGACAAGTTTGTACAAAAAAGCAGGCTATGCTGACGAACTCTTCTGCCG	Tb927.9.11890_rev	GGGGACCACTTTGTACAAGAAAGCTGGGTTTACACCACGAGAGCACCGC
10_hypothetical	I6	Tb927.7.7050_for	GGGGACAAGTTTGTACAAAAAAGCAGGCTATGGGCGCACACTCAAAAAGG	Tb927.7.7050_rev	GGGGACCACTTTGTACAAGAAAGCTGGGTTCACCTCATGAACTGCTTTTTGTAGT
10_hypothetical	I7	Tb927.5.710_for	GGGGACAAGTTTGTACAAAAAAGCAGGCTATGGACAGCGTAGGGGATAAGG	Tb927.5.710_rev	GGGGACCACTTTGTACAAGAAAGCTGGGTTCAGTTCGGTCTCACCCCC
10_hypothetical	I8	Tb927.11.14180_for	GGGGACAAGTTTGTACAAAAAAGCAGGCTATGAAGACGGGTGCGTCCT	Tb927.11.14180_rev	GGGGACCACTTTGTACAAGAAAGCTGGGTTTACAGGTGAGTGGCATATGGCA
10_hypothetical	I9	Tb927.9.9310_for	GGGGACAAGTTTGTACAAAAAAGCAGGCTATGTTGCGAGGGTGGCATCC	Tb927.9.9310_rev	GGGGACCACTTTGTACAAGAAAGCTGGGTCTACGACCAAAGCTCCTCCACG
10_hypothetical	I10	Tb927.4.2920_for	GGGGACAAGTTTGTACAAAAAAGCAGGCTATGACTTGTACGCCGCCCG	Tb927.4.2920_rev	GGGGACCACTTTGTACAAGAAAGCTGGGTCTACAGTCGAATGTCCTTTTGGAGG
10_hypothetical	I11	Tb927.10.2060_for	GGGGACAAGTTTGTACAAAAAAGCAGGCTATGTTGTCGAACACGGGAGGT	Tb927.10.2060_rev	GGGGACCACTTTGTACAAGAAAGCTGGGTTCACGTTCCGCCCAACTCT
10_hypothetical	I12	Tb927.11.6830_for	GGGGACAAGTTTGTACAAAAAAGCAGGCTATGTTCCGTAAGAAGGCCCACT	Tb927.11.6830_rev	GGGGACCACTTTGTACAAGAAAGCTGGGTTCATAGCCGGTATTCGCGCG
10_hypothetical	I13	Tb927.7.1460_for	GGGGACAAGTTTGTACAAAAAAGCAGGCTATGGACGTGGTGTTGGAGGT	Tb927.7.1460_rev	GGGGACCACTTTGTACAAGAAAGCTGGGTCTAAGAAATCGTGCATACGTCAAACAAT
10_hypothetical	I14	Tb927.7.6390_for	GGGGACAAGTTTGTACAAAAAAGCAGGCTATGAAAGCGCACACTGTCGC	Tb927.7.6390_rev	GGGGACCACTTTGTACAAGAAAGCTGGGTCTAAATCGGCATTTGGGTGGGA
10_hypothetical	I15	Tb927.8.3540_for	GGGGACAAGTTTGTACAAAAAAGCAGGCTATGAGCTATACTAATCAGTGCGTGAGA	Tb927.8.3540_rev	GGGGACCACTTTGTACAAGAAAGCTGGGTTCATTTGCTTTCGATTGAGGCGT
10_hypothetical	I16	Tb927.4.1530_for	GGGGACAAGTTTGTACAAAAAAGCAGGCTATGTGGGGCGGTGTGAAGG	Tb927.4.1530_rev	GGGGACCACTTTGTACAAGAAAGCTGGGTTCACCTCTGTAAGTGTCTCTTGAACG
10_hypothetical	I17	Tb927.7.6810_for	GGGGACAAGTTTGTACAAAAAAGCAGGCTATGAAAAATGAAAGGGTAGTTAGTGCATGG	Tb927.7.6810_rev	GGGGACCACTTTGTACAAGAAAGCTGGGTTTATTCGTTTGCGGCAACCTGA
10_hypothetical	I18	Tb927.7.5050_for	GGGGACAAGTTTGTACAAAAAAGCAGGCTATGCACGACGGGTCGCAG	Tb927.7.5050_rev	GGGGACCACTTTGTACAAGAAAGCTGGGTTCATGACTGATCATGTGTGGGTATTCC
10_hypothetical	I19	Tb927.11.16230_for	GGGGACAAGTTTGTACAAAAAAGCAGGCTATGGAGGATGAGCAGAGTAGCC	Tb927.11.16230_rev	GGGGACCACTTTGTACAAGAAAGCTGGGTTTAACACAGTGCGGGGATATTCTCA
10_hypothetical	I20	Tb927.11.12640_for	GGGGACAAGTTTGTACAAAAAAGCAGGCTATGAGCTCTGCGGTTTCAGC	Tb927.11.12640_rev	GGGGACCACTTTGTACAAGAAAGCTGGGTTCATTCATTCAACTCCTTTTCCTCAGC
10_hypothetical	I21	Tb927.11.7590_for	GGGGACAAGTTTGTACAAAAAAGCAGGCTATGTGGCACTCTTTTCTGCGG	Tb927.11.7590_rev	GGGGACCACTTTGTACAAGAAAGCTGGGTTTACACTTCCACTTGGGCCCC
10_hypothetical	I22	Tb927.11.10480_for	GGGGACAAGTTTGTACAAAAAAGCAGGCTATGGAGAGCTTCTCCCCATTTCG	Tb927.11.10480_rev	GGGGACCACTTTGTACAAGAAAGCTGGGTTTAGAAATGTGACCCCGAAAAAGTACC
10_hypothetical	I23	Tb927.10.11130_for	GGGGACAAGTTTGTACAAAAAAGCAGGCTATGTCATGGGTGGAAGACAAGTCA	Tb927.10.11130_rev	GGGGACCACTTTGTACAAGAAAGCTGGGTCTACAGCCCCACACTGGCT
10_hypothetical	I24	Tb927.10.9670_for	GGGGACAAGTTTGTACAAAAAAGCAGGCTATGGAGCAGGCACAGCGG	Tb927.10.9670_rev	GGGGACCACTTTGTACAAGAAAGCTGGGTCTATGTCGTTGTGTATTCAACTCCAGA
10_hypothetical	J1	Tb927.2.2090_for	GGGGACAAGTTTGTACAAAAAAGCAGGCTATGACAATGAAACCCGAAATGGATGA	Tb927.2.2090_rev	GGGGACCACTTTGTACAAGAAAGCTGGGTTCAGCTCCCCGTGTGTGT
10_hypothetical	J2	Tb927.6.900_for	GGGGACAAGTTTGTACAAAAAAGCAGGCTATGGCAATGGACGAGGTGGA	Tb927.6.900_rev	GGGGACCACTTTGTACAAGAAAGCTGGGTCTATGCCTGGAAAATGTATCGGACA
10_hypothetical	J3	Tb927.10.7540_for	GGGGACAAGTTTGTACAAAAAAGCAGGCTATGACAGGTGACGTCTGCCC	Tb927.10.7540_rev	GGGGACCACTTTGTACAAGAAAGCTGGGTTTATACAGGAGGGTGCGCTCC
10_hypothetical	J4	Tb927.7.650_for	GGGGACAAGTTTGTACAAAAAAGCAGGCTATGACGGAGCGCTCGCTG	Tb927.7.650_rev	GGGGACCACTTTGTACAAGAAAGCTGGGTCTAACCATCCGCTTTAGCTCGC
10_hypothetical	J5	Tb927.9.6110_for	GGGGACAAGTTTGTACAAAAAAGCAGGCTATGATGCAACGTGATACTGAGGGG	Tb927.9.6110_rev	GGGGACCACTTTGTACAAGAAAGCTGGGTTTACCTGTTTGCTTTTCGTGAATCTTTG
10_hypothetical	J6	Tb927.5.1330_for	GGGGACAAGTTTGTACAAAAAAGCAGGCTATGCCTCTTCAACCATCGTACTTAATG	Tb927.5.1330_rev	GGGGACCACTTTGTACAAGAAAGCTGGGTTCATCGGGCGTTAACAAACTCC
10_hypothetical	J7	Tb927.9.9640_for	GGGGACAAGTTTGTACAAAAAAGCAGGCTATGAGCAACAAACAAGTGGACGG	Tb927.9.9640_rev	GGGGACCACTTTGTACAAGAAAGCTGGGTTCAGCGCGATTGAAACTTACTTGC
10_hypothetical	J8	Tb927.11.14240_for	GGGGACAAGTTTGTACAAAAAAGCAGGCTATGACTAGCTTAAATGGCAGCAAGAG	Tb927.11.14240_rev	GGGGACCACTTTGTACAAGAAAGCTGGGTTTACACATTTTCTGCAACATACTTGTCG
10_hypothetical	J9	Tb927.8.7850_for	GGGGACAAGTTTGTACAAAAAAGCAGGCTATGAATCGCTTCCAACGGAGTG	Tb927.8.7850_rev	GGGGACCACTTTGTACAAGAAAGCTGGGTCTAAAGTTCCTGCGAGTTCACATTATCA
10_hypothetical	J10	Tb927.1.3290_for	GGGGACAAGTTTGTACAAAAAAGCAGGCTATGGGATGTGCATGCATCCG	Tb927.1.3290_rev	GGGGACCACTTTGTACAAGAAAGCTGGGTTCAGACACTTGACGCACTAATTGAA
10_hypothetical	J11	Tb927.10.4490_for	GGGGACAAGTTTGTACAAAAAAGCAGGCTATGCCAAAAACAGCGGTGCT	Tb927.10.4490_rev	GGGGACCACTTTGTACAAGAAAGCTGGGTCTACATGATATAGACAATAACATTCATCTT
10_hypothetical	J12	Tb927.11.11140_for	GGGGACAAGTTTGTACAAAAAAGCAGGCTATGTCAGCTACGAGTTTTCATTTCGC	Tb927.11.11140_rev	GGGGACCACTTTGTACAAGAAAGCTGGGTTCACGCCTTTCTTGCTTTGAGAG
10_hypothetical	J13	Tb927.7.6740_for	GGGGACAAGTTTGTACAAAAAAGCAGGCTATGATGGACATAGATTGCGCGG	Tb927.7.6740_rev	GGGGACCACTTTGTACAAGAAAGCTGGGTTCAAAAGTCATTCAAGGATGCGGG
10_hypothetical	J14	Tb927.10.10680_for	GGGGACAAGTTTGTACAAAAAAGCAGGCTATGCTGAGGAAACCGTCAGGG	Tb927.10.10680_rev	GGGGACCACTTTGTACAAGAAAGCTGGGTTCACACAAGCGGAATGGAAAGC
10_hypothetical	J15	Tb927.7.3850_for	GGGGACAAGTTTGTACAAAAAAGCAGGCTATGCCATTTCCCCAAACACCAC	Tb927.7.3850_rev	GGGGACCACTTTGTACAAGAAAGCTGGGTCTACACTCGCCGAGGCGC
10_hypothetical	J16	Tb927.5.840_for	GGGGACAAGTTTGTACAAAAAAGCAGGCTATGCCTAAGAAGGATCTATTTTCCTCATCA	Tb927.5.840_rev	GGGGACCACTTTGTACAAGAAAGCTGGGTTTACTCGTGACGAGGCTTAGACG
10_hypothetical	J17	Tb927.9.12070_for	GGGGACAAGTTTGTACAAAAAAGCAGGCTATGGCTAGTGCCCCCCGC	Tb927.9.12070_rev	GGGGACCACTTTGTACAAGAAAGCTGGGTTTAATCATCGTCATCATCCTCAATGAGTG
10_hypothetical	J18	Tb927.7.4450_for	GGGGACAAGTTTGTACAAAAAAGCAGGCTATGCTCAGCCAAGATGCCAGA	Tb927.7.4450_rev	GGGGACCACTTTGTACAAGAAAGCTGGGTTTAAAAAAGTGATTCACCGCCGC
10_hypothetical	J19	Tb927.11.15610_for	GGGGACAAGTTTGTACAAAAAAGCAGGCTATGAAGCAGCAAACGCCGG	Tb927.11.15610_rev	GGGGACCACTTTGTACAAGAAAGCTGGGTCTATCCATCTATTTTCCTGTTAGCAAGCT
10_hypothetical	J20	Tb927.10.1150_for	GGGGACAAGTTTGTACAAAAAAGCAGGCTATGCTACCGATGGCATCCAACC	Tb927.10.1150_rev	GGGGACCACTTTGTACAAGAAAGCTGGGTTTACCTGCTCGCACGCAAC
10_hypothetical	J21	Tb927.4.2820_for	GGGGACAAGTTTGTACAAAAAAGCAGGCTATGAATCTCGTGTATGGTTCCCACC	Tb927.4.2820_rev	GGGGACCACTTTGTACAAGAAAGCTGGGTTCAAAGCATACGAACACTTACGCG
10_hypothetical	J22	Tb927.5.1890_for	GGGGACAAGTTTGTACAAAAAAGCAGGCTATGCGGAGTTCCGACTCACA	Tb927.5.1890_rev	GGGGACCACTTTGTACAAGAAAGCTGGGTTCACCACTTGTAGACTAGTGTCTCAA
10_hypothetical	J23	Tb927.11.7120_for	GGGGACAAGTTTGTACAAAAAAGCAGGCTATGGGGCGGATAGAGGACCT	Tb927.11.7120_rev	GGGGACCACTTTGTACAAGAAAGCTGGGTCTATAAAGTTCGCTTTAGCGCGGC
10_hypothetical	J24	Tb927.9.11770_for	GGGGACAAGTTTGTACAAAAAAGCAGGCTATGACAACCCTTAAAGGTGGTGGT	Tb927.9.11770_rev	GGGGACCACTTTGTACAAGAAAGCTGGGTTTAAGCGGTTTTCCTCATGTGATCC
10_hypothetical	K1	Tb927.11.11650_for	GGGGACAAGTTTGTACAAAAAAGCAGGCTATGGGGGAGCCAGGGGTT	Tb927.11.11650_rev	GGGGACCACTTTGTACAAGAAAGCTGGGTTCAGTCCCTTCCCAAGTGCC
10_hypothetical	K2	Tb927.8.7360_for	GGGGACAAGTTTGTACAAAAAAGCAGGCTATGCTTCTGTGCCGTCCCG	Tb927.8.7360_rev	GGGGACCACTTTGTACAAGAAAGCTGGGTTCAACGCTCAGTGTCGCTCA
10_hypothetical	K3	Tb927.5.3540_for	GGGGACAAGTTTGTACAAAAAAGCAGGCTATGAATGCCATCCCACTCGTCC	Tb927.5.3540_rev	GGGGACCACTTTGTACAAGAAAGCTGGGTTTAAATAAAGAACCGATCGTCTATAAGAGT
10_hypothetical	K4	Tb927.3.1550_for	GGGGACAAGTTTGTACAAAAAAGCAGGCTATGAGTTCAATGGAATTAGCGGATGT	Tb927.3.1550_rev	GGGGACCACTTTGTACAAGAAAGCTGGGTCTAAAGTACTTCCTCGGCCAGCG
10_hypothetical	K5	Tb927.8.4460_for	GGGGACAAGTTTGTACAAAAAAGCAGGCTATGCTAGAAACTCATTGTCAGTTCAAACA	Tb927.8.4460_rev	GGGGACCACTTTGTACAAGAAAGCTGGGTTCACGTCCTTTCCTTCACACTACT
10_hypothetical	K6	Tb927.10.7580_for	GGGGACAAGTTTGTACAAAAAAGCAGGCTATGAGTGTCGAAAGGATCGTGC	Tb927.10.7580_rev	GGGGACCACTTTGTACAAGAAAGCTGGGTTTACCGATCACCCTCCTGACC
10_hypothetical	K7	Tb927.3.3050_for	GGGGACAAGTTTGTACAAAAAAGCAGGCTATGGCGCCGGCAGTAATG	Tb927.3.3050_rev	GGGGACCACTTTGTACAAGAAAGCTGGGTTTATTCATCACACGCTTGTCGTAGC
10_hypothetical	K8	Tb927.10.12820_for	GGGGACAAGTTTGTACAAAAAAGCAGGCTATGGGAGCCAAACCAGAATCTTTG	Tb927.10.12820_rev	GGGGACCACTTTGTACAAGAAAGCTGGGTTTACTTGGTTACCTTTCGTTTGATGCG
10_hypothetical	K9	Tb927.4.2580_for	GGGGACAAGTTTGTACAAAAAAGCAGGCTATGCATAATCGTCTATACCACAGGGT	Tb927.4.2580_rev	GGGGACCACTTTGTACAAGAAAGCTGGGTTTACTCGTGCTGCAGTCGTTC
10_hypothetical	K10	Tb927.7.7290_for	GGGGACAAGTTTGTACAAAAAAGCAGGCTATGGAGCAATATCAATCCCAATCCCA	Tb927.7.7290_rev	GGGGACCACTTTGTACAAGAAAGCTGGGTCTACAATCGAAGGCGGTCAATATCC
10_hypothetical	K11	Tb927.11.3500_for	GGGGACAAGTTTGTACAAAAAAGCAGGCTATGAGCGTAGAAGAAGTGTCATGTC	Tb927.11.3500_rev	GGGGACCACTTTGTACAAGAAAGCTGGGTTTACAGTTCAAAACACGAATCATCGTG
10_hypothetical	K12	Tb927.7.6590_for	GGGGACAAGTTTGTACAAAAAAGCAGGCTATGAATCTAGCGAAGAAAATCATCGTGT	Tb927.7.6590_rev	GGGGACCACTTTGTACAAGAAAGCTGGGTTTATTGCACAGACACACCCTCG
10_hypothetical	K13	Tb927.9.4530_for	GGGGACAAGTTTGTACAAAAAAGCAGGCTATGAAGCGGGATGAACTGTTTGC	Tb927.9.4530_rev	GGGGACCACTTTGTACAAGAAAGCTGGGTCTATGCCTCCACTGGAGCTCC
10_hypothetical	K14	Tb927.7.2350_for	GGGGACAAGTTTGTACAAAAAAGCAGGCTATGTCCGATGACGCTAAAACCAC	Tb927.7.2350_rev	GGGGACCACTTTGTACAAGAAAGCTGGGTCTAATCTTCTCTTTCCGTTTTCGCATC
10_hypothetical	K15	Tb927.11.2310_for	GGGGACAAGTTTGTACAAAAAAGCAGGCTATGTATAAGCAGCACACGAACTTTGC	Tb927.11.2310_rev	GGGGACCACTTTGTACAAGAAAGCTGGGTCTAGGCATCGTAATCATCAAAAATAATGTC
10_hypothetical	K16	Tb927.9.6530_for	GGGGACAAGTTTGTACAAAAAAGCAGGCTATGGGGAACGCACAGGACA	Tb927.9.6530_rev	GGGGACCACTTTGTACAAGAAAGCTGGGTTTACTCCCGGATAATGGTGGCG
10_hypothetical	K17	Tb927.10.3780_for	GGGGACAAGTTTGTACAAAAAAGCAGGCTATGATATCTCGAAAGGACACCGGG	Tb927.10.3780_rev	GGGGACCACTTTGTACAAGAAAGCTGGGTCTAACTAGCCACAACATCAAAAAGCAAC
10_hypothetical	K18	Tb927.9.12770_for	GGGGACAAGTTTGTACAAAAAAGCAGGCTATGAAACTACTGCGACGAGGGA	Tb927.9.12770_rev	GGGGACCACTTTGTACAAGAAAGCTGGGTTTAATGTGCACGATCATCTGCACC
10_hypothetical	K19	Tb927.3.5620_for	GGGGACAAGTTTGTACAAAAAAGCAGGCTATGTCGCGTGTTACCCTTTCC	Tb927.3.5620_rev	GGGGACCACTTTGTACAAGAAAGCTGGGTCTAAAACCGGCGCGGTGG
10_hypothetical	K20	Tb927.10.16200_for	GGGGACAAGTTTGTACAAAAAAGCAGGCTATGCCGGCCACCTCAACG	Tb927.10.16200_rev	GGGGACCACTTTGTACAAGAAAGCTGGGTTCACAATTCGTAGTGCTCTCGTCG
10_hypothetical	K21	Tb927.10.9970_for	GGGGACAAGTTTGTACAAAAAAGCAGGCTATGATGGCCAACAAGCGGTT	Tb927.10.9970_rev	GGGGACCACTTTGTACAAGAAAGCTGGGTTCACGCTGATGCTCCCCC
10_hypothetical	K22	Tb927.3.3800_for	GGGGACAAGTTTGTACAAAAAAGCAGGCTATGGATTGCAAGTTGCAACATGTTC	Tb927.3.3800_rev	GGGGACCACTTTGTACAAGAAAGCTGGGTTTAACAAGTTCGCTTCATGTTACACCG
10_hypothetical	K23	Tb927.1.2990_for	GGGGACAAGTTTGTACAAAAAAGCAGGCTATGTTAACCCCGACACGGCG	Tb927.1.2990_rev	GGGGACCACTTTGTACAAGAAAGCTGGGTCTATTTATTTCTGTGACGACTTCGTACCT
10_hypothetical	K24	Tb927.8.780_for	GGGGACAAGTTTGTACAAAAAAGCAGGCTATGGGAGACTGCGTTGCTTCA	Tb927.8.780_rev	GGGGACCACTTTGTACAAGAAAGCTGGGTCTACACAACTGGCGCAACCG
10_hypothetical	L1	Tb927.11.12460_for	GGGGACAAGTTTGTACAAAAAAGCAGGCTATGTTTTACGCCTCGGAGGAAGG	Tb927.11.12460_rev	GGGGACCACTTTGTACAAGAAAGCTGGGTTCAAGCGACCACTCCCCTG
10_hypothetical	L2	Tb927.3.1670_for	GGGGACAAGTTTGTACAAAAAAGCAGGCTATGGCGTATTCCACTCGAGTAATACG	Tb927.3.1670_rev	GGGGACCACTTTGTACAAGAAAGCTGGGTCTACTGCAAACTCATTTCCTCTTCCC
10_hypothetical	L3	Tb927.1.4760_for	GGGGACAAGTTTGTACAAAAAAGCAGGCTATGTCGTCTTACGAGGCCTTTCG	Tb927.1.4760_rev	GGGGACCACTTTGTACAAGAAAGCTGGGTTTACAACACTTCCCGCAATAGGAC
10_hypothetical	L4	Tb927.7.3090_for	GGGGACAAGTTTGTACAAAAAAGCAGGCTATGTGGAATGAAGTTAGCGCTAGTG	Tb927.7.3090_rev	GGGGACCACTTTGTACAAGAAAGCTGGGTTTACGGCAGTGACGCATCCC
10_hypothetical	L5	Tb927.8.4900_for	GGGGACAAGTTTGTACAAAAAAGCAGGCTATGTCTTCGGAGGCGGTAAAAAG	Tb927.8.4900_rev	GGGGACCACTTTGTACAAGAAAGCTGGGTTTAAACTTCTAGACCGACAGCAATATCG
10_hypothetical	L6	Tb927.4.4710_for	GGGGACAAGTTTGTACAAAAAAGCAGGCTATGACTTCAAGTTGGGGCCCG	Tb927.4.4710_rev	GGGGACCACTTTGTACAAGAAAGCTGGGTCTATTGTCCATTTGCGCCAACCA
10_hypothetical	L7	Tb927.2.3020_for	GGGGACAAGTTTGTACAAAAAAGCAGGCTATGTCTGATGCGTCCGTTAATGAC	Tb927.2.3020_rev	GGGGACCACTTTGTACAAGAAAGCTGGGTTCACTTACCCTCCTCTCCCGG
10_hypothetical	L8	Tb927.8.1260_for	GGGGACAAGTTTGTACAAAAAAGCAGGCTATGCGACGAAGGCGACAAC	Tb927.8.1260_rev	GGGGACCACTTTGTACAAGAAAGCTGGGTTCAAAAGAGCGCCTCCACAGC
10_hypothetical	L9	Tb927.8.4350_for	GGGGACAAGTTTGTACAAAAAAGCAGGCTATGCTATATGATGTTGTGGAGTTGTTGG	Tb927.8.4350_rev	GGGGACCACTTTGTACAAGAAAGCTGGGTTCATGCACTGCAATTGGGTAACA
10_hypothetical	L10	Tb927.10.12920_for	GGGGACAAGTTTGTACAAAAAAGCAGGCTATGAACTGCGACAACCAAGAAGT	Tb927.10.12920_rev	GGGGACCACTTTGTACAAGAAAGCTGGGTCTATCTTCGGCATAGACGCACTG
10_hypothetical	L11	Tb927.11.2830_for	GGGGACAAGTTTGTACAAAAAAGCAGGCTATGGGAAAGATTCTCCTTTGTGTACAG	Tb927.11.2830_rev	GGGGACCACTTTGTACAAGAAAGCTGGGTTCATTTACCTGAAGAAACTTCTGACTGAG
10_hypothetical	L12	Tb927.5.2530_for	GGGGACAAGTTTGTACAAAAAAGCAGGCTATGCCACCGCCCACATCT	Tb927.5.2530_rev	GGGGACCACTTTGTACAAGAAAGCTGGGTTCAGCCGCGACGAGTTTTC
10_hypothetical	L13	Tb927.2.2210_for	GGGGACAAGTTTGTACAAAAAAGCAGGCTATGTGGAGGAGGCACTGCTG	Tb927.2.2210_rev	GGGGACCACTTTGTACAAGAAAGCTGGGTTTACGGCATGGCCACCGT
10_hypothetical	L14	Tb927.10.14330_for	GGGGACAAGTTTGTACAAAAAAGCAGGCTATGGTCTTACGCCGTGATGGC	Tb927.10.14330_rev	GGGGACCACTTTGTACAAGAAAGCTGGGTCTACACGCGTGTTGTTATCTCACC
10_hypothetical	L15	Tb927.7.860_for	GGGGACAAGTTTGTACAAAAAAGCAGGCTATGCCAGTGGAATGGCCCA	Tb927.7.860_rev	GGGGACCACTTTGTACAAGAAAGCTGGGTTTACCCAGCAATGCTGGGAACG
10_hypothetical	L16	Tb927.11.11280_for	GGGGACAAGTTTGTACAAAAAAGCAGGCTATGAATCCGTTTATTTTACCGCCATTAGA	Tb927.11.11280_rev	GGGGACCACTTTGTACAAGAAAGCTGGGTTTACTTCTCGTAGTATGCCTCGCG
10_hypothetical	L17	Tb927.11.5060_for	GGGGACAAGTTTGTACAAAAAAGCAGGCTATGTTCTTCAGGTTTCATAGCACAACA	Tb927.11.5060_rev	GGGGACCACTTTGTACAAGAAAGCTGGGTTTAACGGCGTCGGTTCCCT
10_hypothetical	L18	Tb927.9.2550_for	GGGGACAAGTTTGTACAAAAAAGCAGGCTATGCGTCGTGAGGAACGGC	Tb927.9.2550_rev	GGGGACCACTTTGTACAAGAAAGCTGGGTTTAAGCTGCTGTCCGAGAGGC
10_hypothetical	L19	Tb927.8.6100_for	GGGGACAAGTTTGTACAAAAAAGCAGGCTATGGAAAAGCTCCAACTGGAGC	Tb927.8.6100_rev	GGGGACCACTTTGTACAAGAAAGCTGGGTTCACTGCGAAGTATCCCAACGA
10_hypothetical	L20	Tb927.9.10530_for	GGGGACAAGTTTGTACAAAAAAGCAGGCTATGAGCCGACGTGGTGGC	Tb927.9.10530_rev	GGGGACCACTTTGTACAAGAAAGCTGGGTCTACCTCGAAGAATAAGAATAACCGCTG
10_hypothetical	L21	Tb927.11.15970_for	GGGGACAAGTTTGTACAAAAAAGCAGGCTATGGAGGCTCTCCTGCGG	Tb927.11.15970_rev	GGGGACCACTTTGTACAAGAAAGCTGGGTTCACACAAGATTCATGAGCGGC
10_hypothetical	L22	Tb927.10.13550_for	GGGGACAAGTTTGTACAAAAAAGCAGGCTATGGATTTCATTGCATTTAAGGATGGTG	Tb927.10.13550_rev	GGGGACCACTTTGTACAAGAAAGCTGGGTTTAGGCAGTTCCTGTGCACTGA
10_hypothetical	L23	Tb927.8.5220_for	GGGGACAAGTTTGTACAAAAAAGCAGGCTATGCTCAGAACTCGCTTGTCAC	Tb927.8.5220_rev	GGGGACCACTTTGTACAAGAAAGCTGGGTTCAAAAGGGTGAGTGAGGACTGG
10_hypothetical	L24	Tb927.10.4470_for	GGGGACAAGTTTGTACAAAAAAGCAGGCTATGGATTGCCACTTCCGTCAAC	Tb927.10.4470_rev	GGGGACCACTTTGTACAAGAAAGCTGGGTTTAAACGAGAAGAATGCCCCTTATTCC
10_hypothetical	M1	Tb927.11.240_for	GGGGACAAGTTTGTACAAAAAAGCAGGCTATGTGGCGTTTGTTATTTCTGCG	Tb927.11.240_rev	GGGGACCACTTTGTACAAGAAAGCTGGGTTTATTCATTACCCTTTCCTTCGTAAACGA
10_hypothetical	M2	Tb927.9.8820_for	GGGGACAAGTTTGTACAAAAAAGCAGGCTATGAAAGCTGCAAGAAGTAACGGA	Tb927.9.8820_rev	GGGGACCACTTTGTACAAGAAAGCTGGGTCTAAACCGCTGAATCTATCAAACCTTCT
10_hypothetical	M3	Tb927.11.7400_for	GGGGACAAGTTTGTACAAAAAAGCAGGCTATGGAAGACTCTAACGAAGTTGCG	Tb927.11.7400_rev	GGGGACCACTTTGTACAAGAAAGCTGGGTCTAGCTAGACCTTACCAATGTAAATTTATT
10_hypothetical	M4	Tb927.8.4660_for	GGGGACAAGTTTGTACAAAAAAGCAGGCTATGCTGGGGAGTGTTGATGATGT	Tb927.8.4660_rev	GGGGACCACTTTGTACAAGAAAGCTGGGTTCATGCCAAACAGCGGGTTAC
10_hypothetical	M5	Tb927.11.3410_for	GGGGACAAGTTTGTACAAAAAAGCAGGCTATGAACAAGGCGGGTGTGTATTC	Tb927.11.3410_rev	GGGGACCACTTTGTACAAGAAAGCTGGGTCTACTCACCGTAAAATCGGTTGAAAGT
10_hypothetical	M6	Tb927.11.3330_for	GGGGACAAGTTTGTACAAAAAAGCAGGCTATGTGCGGTGTGTCGGGG	Tb927.11.3330_rev	GGGGACCACTTTGTACAAGAAAGCTGGGTTTATCGACCGTTCGCAGAGCT
10_hypothetical	M7	Tb927.10.2920_for	GGGGACAAGTTTGTACAAAAAAGCAGGCTATGACCAACCCACCCCAAGAC	Tb927.10.2920_rev	GGGGACCACTTTGTACAAGAAAGCTGGGTCTACTTTTTGACATTCCGTGGTGAAAC
10_hypothetical	M8	Tb927.2.5900_for	GGGGACAAGTTTGTACAAAAAAGCAGGCTATGTGTTGGTGCACTACGCAA	Tb927.2.5900_rev	GGGGACCACTTTGTACAAGAAAGCTGGGTTCAACAGTCTGCAGCAAAAAGCA
10_hypothetical	M9	Tb927.2.2580_for	GGGGACAAGTTTGTACAAAAAAGCAGGCTATGCCTCGAAAGAGGAATCCGT	Tb927.2.2580_rev	GGGGACCACTTTGTACAAGAAAGCTGGGTCTACGATTGCGCGCTTGGT
10_hypothetical	M10	Tb927.2.3990_for	GGGGACAAGTTTGTACAAAAAAGCAGGCTATGTGGGACCACATTAATGGTGTTAC	Tb927.2.3990_rev	GGGGACCACTTTGTACAAGAAAGCTGGGTTCAAGTTGTCGAAGCACCAACC
10_hypothetical	M11	Tb927.1.4740_for	GGGGACAAGTTTGTACAAAAAAGCAGGCTATGACCGCAAACTACCACACG	Tb927.1.4740_rev	GGGGACCACTTTGTACAAGAAAGCTGGGTTCATACTTGCCACAACTCCACAAG
10_hypothetical	M12	Tb927.9.12800_for	GGGGACAAGTTTGTACAAAAAAGCAGGCTATGGACGGCTGCGTCAAC	Tb927.9.12800_rev	GGGGACCACTTTGTACAAGAAAGCTGGGTTTACATACTCTTCCACATGTGTGAACTC
10_hypothetical	M13	Tb927.3.3110_for	GGGGACAAGTTTGTACAAAAAAGCAGGCTATGCCGCCTAAAAAGAAGCGA	Tb927.3.3110_rev	GGGGACCACTTTGTACAAGAAAGCTGGGTTTACCGGTCCGTCCTGCC

10_hypothetical	M14	Tb927.7.5750_for	GGGGACAAGTTTGTACAAAAAAGCAGGCTATGGTTTCACGTGCACCGTC	Tb927.7.5750_rev	GGGGACCACTTTGTACAAGAAAGCTGGGTTTAGGATGTTTTCAGCACCCTCTTAAG
10_hypothetical	M15	Tb927.3.4970_for	GGGGACAAGTTTGTACAAAAAAGCAGGCTATGGGAACAATAGAAGGCACAACTG	Tb927.3.4970_rev	GGGGACCACTTTGTACAAGAAAGCTGGGTTCAGCGTGCCGTTTTTACTGT
10_hypothetical	M16	Tb927.7.1660_for	GGGGACAAGTTTGTACAAAAAAGCAGGCTATGGGGGGTAGAAACACGGG	Tb927.7.1660_rev	GGGGACCACTTTGTACAAGAAAGCTGGGTTCAACTCTCTCGTATTTCCTGCACT
10_hypothetical	M17	Tb927.5.4130_for	GGGGACAAGTTTGTACAAAAAAGCAGGCTATGCTGCACCCCAATACTCGT	Tb927.5.4130_rev	GGGGACCACTTTGTACAAGAAAGCTGGGTTCAACACTTCTGGTTGTTATCCCAC
10_hypothetical	M18	Tb927.10.2790_for	GGGGACAAGTTTGTACAAAAAAGCAGGCTATGGACAACTCTGAGGCTGTGA	Tb927.10.2790_rev	GGGGACCACTTTGTACAAGAAAGCTGGGTTTATGGGGTGGTGTCCAAAAGGA
10_hypothetical	M19	Tb927.11.16530_for	GGGGACAAGTTTGTACAAAAAAGCAGGCTATGATCCGTACCACTCATTTGATGTTG	Tb927.11.16530_rev	GGGGACCACTTTGTACAAGAAAGCTGGGTCTATGGCTTGTGTTTCCCGTGA
10_hypothetical	M20	Tb927.7.6650_for	GGGGACAAGTTTGTACAAAAAAGCAGGCTATGTCGTACAACGCGTCCAC	Tb927.7.6650_rev	GGGGACCACTTTGTACAAGAAAGCTGGGTTCACACCGCATTGACACCCC
10_hypothetical	M21	Tb927.11.15370_for	GGGGACAAGTTTGTACAAAAAAGCAGGCTATGAACCAAGAGCAGCTGAACC	Tb927.11.15370_rev	GGGGACCACTTTGTACAAGAAAGCTGGGTTCACATCGCTGCAAGTTGATTTTTT
10_hypothetical	M22	Tb927.7.1220_for	GGGGACAAGTTTGTACAAAAAAGCAGGCTATGGAGCATCAAAACGACCGC	Tb927.7.1220_rev	GGGGACCACTTTGTACAAGAAAGCTGGGTCTAGTAAATACACGATAGCATCAGCGAC
10_hypothetical	M23	Tb927.7.6580_for	GGGGACAAGTTTGTACAAAAAAGCAGGCTATGCATGTTCTGTGGTGTATTGTGC	Tb927.7.6580_rev	GGGGACCACTTTGTACAAGAAAGCTGGGTTTATTGCACAGACACACCCTCG
10_hypothetical	M24	Tb927.11.15070_for	GGGGACAAGTTTGTACAAAAAAGCAGGCTATGAGAAGAGCCTTAAGTGGGCT	Tb927.11.15070_rev	GGGGACCACTTTGTACAAGAAAGCTGGGTTTAAGATTTGTTATCATCTCCTGGACGAAG
10_hypothetical	N1	Tb927.4.3920_for	GGGGACAAGTTTGTACAAAAAAGCAGGCTATGTCCTCTTTTCTACAGGAACTTGGT	Tb927.4.3920_rev	GGGGACCACTTTGTACAAGAAAGCTGGGTCTACATCTCATGAACCGATTCCTCCA
10_hypothetical	N2	Tb927.6.3220_for	GGGGACAAGTTTGTACAAAAAAGCAGGCTATGCCAGTTCGACAGAAGTATCCA	Tb927.6.3220_rev	GGGGACCACTTTGTACAAGAAAGCTGGGTTCAATCACTAAGTAACTTTGTCTCATCGC
10_hypothetical	N3	Tb927.3.4600_for	GGGGACAAGTTTGTACAAAAAAGCAGGCTATGAATATCCTACAACTCTTATCGGTTGC	Tb927.3.4600_rev	GGGGACCACTTTGTACAAGAAAGCTGGGTCTACTGAGTTTGCGCTGCAGC
10_hypothetical	N4	Tb927.4.4560_for	GGGGACAAGTTTGTACAAAAAAGCAGGCTATGGTTGAGGAGCCAAGCGG	Tb927.4.4560_rev	GGGGACCACTTTGTACAAGAAAGCTGGGTTCAGTTATTGCACTTATCCAGCTCCT
10_hypothetical	N5	Tb927.2.5820_for	GGGGACAAGTTTGTACAAAAAAGCAGGCTATGGGGCGGCAGCGAGCA	Tb927.2.5820_rev	GGGGACCACTTTGTACAAGAAAGCTGGGTTTAAGCCACTGTCGCGTGC
10_hypothetical	N6	Tb927.11.2590_for	GGGGACAAGTTTGTACAAAAAAGCAGGCTATGTCAACATCCTTTTCCTCCAAAAAAG	Tb927.11.2590_rev	GGGGACCACTTTGTACAAGAAAGCTGGGTTCAGCTTAATCGTGGAACAGCC
10_hypothetical	N7	Tb927.5.2980_for	GGGGACAAGTTTGTACAAAAAAGCAGGCTATGCCAGCAGGTAGTGATGACG	Tb927.5.2980_rev	GGGGACCACTTTGTACAAGAAAGCTGGGTTTAGACAGCCCGCAGCAGC
10_hypothetical	N8	Tb927.8.4230_for	GGGGACAAGTTTGTACAAAAAAGCAGGCTATGCTTCGCCGTAATCTTGTTTCT	Tb927.8.4230_rev	GGGGACCACTTTGTACAAGAAAGCTGGGTTTAACGCCGTACTCTCTCGAACC
10_hypothetical	N9	Tb927.10.720_for	GGGGACAAGTTTGTACAAAAAAGCAGGCTATGCCAAAGAAGAGTGAACCGG	Tb927.10.720_rev	GGGGACCACTTTGTACAAGAAAGCTGGGTCTACTTACTAATTCGTTTCTGAACAGCAGC
10_hypothetical	N10	Tb927.4.4580_for	GGGGACAAGTTTGTACAAAAAAGCAGGCTATGGGAGCGGTTCCCAGTC	Tb927.4.4580_rev	GGGGACCACTTTGTACAAGAAAGCTGGGTTCAGTGCTGTAAACCGTGCC
10_hypothetical	N11	Tb927.8.3490_for	GGGGACAAGTTTGTACAAAAAAGCAGGCTATGAACTTCTTATCCCAGATTTACACCG	Tb927.8.3490_rev	GGGGACCACTTTGTACAAGAAAGCTGGGTCTACGGCGTCAAAACGCAGA
10_hypothetical	N12	Tb927.5.2800_for	GGGGACAAGTTTGTACAAAAAAGCAGGCTATGTCAGTAGATACCATTCAGCTTGTG	Tb927.5.2800_rev	GGGGACCACTTTGTACAAGAAAGCTGGGTTCATTCACGTAGTCGTTTTGTTGTGC
10_hypothetical	N13	Tb927.10.13880_for	GGGGACAAGTTTGTACAAAAAAGCAGGCTATGATGGATCAGATGAATATTGGGGTGC	Tb927.10.13880_rev	GGGGACCACTTTGTACAAGAAAGCTGGGTTTACCAAGCCGATAGAGTTTGAACC
10_hypothetical	N14	Tb927.10.10090_for	GGGGACAAGTTTGTACAAAAAAGCAGGCTATGTCCGTTGCGCTCGAC	Tb927.10.10090_rev	GGGGACCACTTTGTACAAGAAAGCTGGGTCTACCGCGAACAGCGTTGT
10_hypothetical	N15	Tb927.8.840_for	GGGGACAAGTTTGTACAAAAAAGCAGGCTATGCAGTGCGAGAGATTCAAACA	Tb927.8.840_rev	GGGGACCACTTTGTACAAGAAAGCTGGGTTCAACTCAATGTTATATTAATGCCGCGC
10_hypothetical	N16	Tb927.10.12870_for	GGGGACAAGTTTGTACAAAAAAGCAGGCTATGACCGAAGCAACCAATGAGG	Tb927.10.12870_rev	GGGGACCACTTTGTACAAGAAAGCTGGGTTTAGTGTTTTTGTTTTCTACGCTTCCG
10_hypothetical	N17	Tb927.9.2590_for	GGGGACAAGTTTGTACAAAAAAGCAGGCTATGCAGACTAGCAGTGTCTCGG	Tb927.9.2590_rev	GGGGACCACTTTGTACAAGAAAGCTGGGTTTAGGTTCCGAGCCGGCG
10_hypothetical	N18	Tb927.10.8600_for	GGGGACAAGTTTGTACAAAAAAGCAGGCTATGTCAACAGGTCACCCCGT	Tb927.10.8600_rev	GGGGACCACTTTGTACAAGAAAGCTGGGTCTACGCCTGACCAAAGAACCATG
10_hypothetical	N19	Tb927.7.5610_for	GGGGACAAGTTTGTACAAAAAAGCAGGCTATGAAGTCAGGAATATCTAGTCGGTCG	Tb927.7.5610_rev	GGGGACCACTTTGTACAAGAAAGCTGGGTTTACCGTGTAGAAACCCTATTGTTCAC
10_hypothetical	N20	Tb927.10.5170_for	GGGGACAAGTTTGTACAAAAAAGCAGGCTATGCCTACGGCCGATTCAAC	Tb927.10.5170_rev	GGGGACCACTTTGTACAAGAAAGCTGGGTTCAGCGCCGCTTGAATGG
10_hypothetical	N21	Tb927.11.11810_for	GGGGACAAGTTTGTACAAAAAAGCAGGCTATGAGCAGGGCACTTTTATCTAGGA	Tb927.11.11810_rev	GGGGACCACTTTGTACAAGAAAGCTGGGTTTAAGACGTGGCGCATCCAC
10_hypothetical	N22	Tb927.8.2580_for	GGGGACAAGTTTGTACAAAAAAGCAGGCTATGCCGAACCATGGAAAGAGTAAG	Tb927.8.2580_rev	GGGGACCACTTTGTACAAGAAAGCTGGGTTCACTTCACAACATCACGAAACTTGT
10_hypothetical	N23	Tb927.7.2330_for	GGGGACAAGTTTGTACAAAAAAGCAGGCTATGAGATCGCGAAATACGGATGC	Tb927.7.2330_rev	GGGGACCACTTTGTACAAGAAAGCTGGGTTCAGCGGTTACGGATAACCCA
10_hypothetical	N24	Tb927.9.12780_for	GGGGACAAGTTTGTACAAAAAAGCAGGCTATGTCCATTGCGGTAGAAGAGAGT	Tb927.9.12780_rev	GGGGACCACTTTGTACAAGAAAGCTGGGTCTATCGAAGGGCAGTACACATATCATC
10_hypothetical	O1	Tb927.4.1060_for	GGGGACAAGTTTGTACAAAAAAGCAGGCTATGCGGTGTTCCGCGCGC	Tb927.4.1060_rev	GGGGACCACTTTGTACAAGAAAGCTGGGTTTATTCTCCTTCACCGTTGGTAGGG
10_hypothetical	O2	Tb927.3.2780_for	GGGGACAAGTTTGTACAAAAAAGCAGGCTATGTATGTGTTCTCGCCAGCG	Tb927.3.2780_rev	GGGGACCACTTTGTACAAGAAAGCTGGGTTCACACCGCCGATGATGCG
10_hypothetical	O3	Tb927.10.11850_for	GGGGACAAGTTTGTACAAAAAAGCAGGCTATGATGATCCAGGAGCGTGATGC	Tb927.10.11850_rev	GGGGACCACTTTGTACAAGAAAGCTGGGTTCATCCCTCGTCTTTCCTTTTGAAC
10_hypothetical	O4	Tb927.8.5240_for	GGGGACAAGTTTGTACAAAAAAGCAGGCTATGGATCCGCACCCAACGC	Tb927.8.5240_rev	GGGGACCACTTTGTACAAGAAAGCTGGGTCTACCGCTGTTCTATGATGCCAC
10_hypothetical	O5	Tb927.8.7750_for	GGGGACAAGTTTGTACAAAAAAGCAGGCTATGGGGGCTGTGCCCAGC	Tb927.8.7750_rev	GGGGACCACTTTGTACAAGAAAGCTGGGTTCAGTGTTGCAGGCCACGC
10_hypothetical	O6	Tb927.7.4220_for	GGGGACAAGTTTGTACAAAAAAGCAGGCTATGGTTGGGAAGACGTATCTTCGG	Tb927.7.4220_rev	GGGGACCACTTTGTACAAGAAAGCTGGGTTTAATGCTTCTCTCCTCTTTGCTCTTC
10_hypothetical	O7	Tb927.8.1290_for	GGGGACAAGTTTGTACAAAAAAGCAGGCTATGAGCCCTTCCAGTTTGACATTG	Tb927.8.1290_rev	GGGGACCACTTTGTACAAGAAAGCTGGGTTCAGCCGTAGAGAAATACGACAACG
10_hypothetical	O8	Tb927.11.1160_for	GGGGACAAGTTTGTACAAAAAAGCAGGCTATGAAGGCGCTTAGTGACGATTC	Tb927.11.1160_rev	GGGGACCACTTTGTACAAGAAAGCTGGGTCTACAACACTAACCAAGAATCGTCCTC
10_hypothetical	O9	Tb927.6.2660_for	GGGGACAAGTTTGTACAAAAAAGCAGGCTATGGGTGAAACAGTGAGAAGGTGT	Tb927.6.2660_rev	GGGGACCACTTTGTACAAGAAAGCTGGGTCTAGGCATCCTGCATGAGAAACTG
10_hypothetical	O10	Tb927.11.3910_for	GGGGACAAGTTTGTACAAAAAAGCAGGCTATGCCTAGTTCCAAGTCGGACA	Tb927.11.3910_rev	GGGGACCACTTTGTACAAGAAAGCTGGGTTTACTTTTCTTCCTCCCCAGGCA
10_hypothetical	O11	Tb927.8.7540_for	GGGGACAAGTTTGTACAAAAAAGCAGGCTATGCTGGACACCAATGGGACT	Tb927.8.7540_rev	GGGGACCACTTTGTACAAGAAAGCTGGGTTCAAATATTTTTCGCACTCCGTCCA
10_hypothetical	O12	Tb927.11.16240_for	GGGGACAAGTTTGTACAAAAAAGCAGGCTATGTCATCACGAGAAGAACGCAG	Tb927.11.16240_rev	GGGGACCACTTTGTACAAGAAAGCTGGGTCTACGTGGGTCTGTCGATGAATG
10_hypothetical	O13	Tb927.9.8620_for	GGGGACAAGTTTGTACAAAAAAGCAGGCTATGGAGGGGTTCAAAACACCAAAC	Tb927.9.8620_rev	GGGGACCACTTTGTACAAGAAAGCTGGGTCTAATGCCCCCGAGTTAAACGG
10_hypothetical	O14	Tb927.6.3590_for	GGGGACAAGTTTGTACAAAAAAGCAGGCTATGAATTTTGCGACATCACAGCAAG	Tb927.6.3590_rev	GGGGACCACTTTGTACAAGAAAGCTGGGTCTAAATGATCTCATCGCAAAATTGGACG
10_hypothetical	O15	Tb927.11.2140_for	GGGGACAAGTTTGTACAAAAAAGCAGGCTATGAGCGACTCCATGGGTGA	Tb927.11.2140_rev	GGGGACCACTTTGTACAAGAAAGCTGGGTCTAAGAGCTGTATGCATGCCGG
10_hypothetical	O16	Tb927.10.14400_for	GGGGACAAGTTTGTACAAAAAAGCAGGCTATGAGCCCCTCTCAATTATCGCG	Tb927.10.14400_rev	GGGGACCACTTTGTACAAGAAAGCTGGGTTCAGTGGCTACAACGGAAAAAAGC
10_hypothetical	O17	Tb927.11.14650_for	GGGGACAAGTTTGTACAAAAAAGCAGGCTATGATGGAGTTGGCGGAAGGT	Tb927.11.14650_rev	GGGGACCACTTTGTACAAGAAAGCTGGGTTCATGTTGTCAACAACTGGGGG
10_hypothetical	O18	Tb927.10.13350_for	GGGGACAAGTTTGTACAAAAAAGCAGGCTATGAGTACTCCCCCGCATGC	Tb927.10.13350_rev	GGGGACCACTTTGTACAAGAAAGCTGGGTCTAGCCTGCGGGTGTCGA
10_hypothetical	O19	Tb927.10.12620_for	GGGGACAAGTTTGTACAAAAAAGCAGGCTATGTCCGTGAAAAGGAACACCC	Tb927.10.12620_rev	GGGGACCACTTTGTACAAGAAAGCTGGGTTTACATACCTCCTTGACCCAAGGG
10_hypothetical	O20	Tb927.8.7200_for	GGGGACAAGTTTGTACAAAAAAGCAGGCTATGGCTGCCGTGTGGCGG	Tb927.8.7200_rev	GGGGACCACTTTGTACAAGAAAGCTGGGTCTACCACAGCTCTACTGCCTCC
10_hypothetical	O21	Tb927.9.1940_for	GGGGACAAGTTTGTACAAAAAAGCAGGCTATGACGTTTAGCGCAAGGGAG	Tb927.9.1940_rev	GGGGACCACTTTGTACAAGAAAGCTGGGTTCATCCCATCAAACCAAGAAGCAAC
10_hypothetical	O22	Tb927.6.2210_for	GGGGACAAGTTTGTACAAAAAAGCAGGCTATGGAAGGTTTGTTTATTCGGGTTGT	Tb927.6.2210_rev	GGGGACCACTTTGTACAAGAAAGCTGGGTTTACTGGCACCTTCTACTATCACCAC
10_hypothetical	O23	Tb927.8.2880_for	GGGGACAAGTTTGTACAAAAAAGCAGGCTATGTATTCAAGGGCGCTGCC	Tb927.8.2880_rev	GGGGACCACTTTGTACAAGAAAGCTGGGTCTACTGACTGTAACAATGAATCATCCGC
10_hypothetical	O24	Tb927.10.14320_for	GGGGACAAGTTTGTACAAAAAAGCAGGCTATGTGCTCGCTTTCTCCGAAC	Tb927.10.14320_rev	GGGGACCACTTTGTACAAGAAAGCTGGGTCTAACGCTTTCGTGCACGGG
10_hypothetical	P1	Tb927.7.6760_for	GGGGACAAGTTTGTACAAAAAAGCAGGCTATGTACCAGTCCGGAGGCC	Tb927.7.6760_rev	GGGGACCACTTTGTACAAGAAAGCTGGGTTCACTCTGCAAATATGGCTCTGC
10_hypothetical	P2	Tb927.10.5260_for	GGGGACAAGTTTGTACAAAAAAGCAGGCTATGACTGGAGGCAATAGCGGG	Tb927.10.5260_rev	GGGGACCACTTTGTACAAGAAAGCTGGGTTCACTCTTTGGTCGAATCCTTCTTATCG
10_hypothetical	P3	Tb927.3.2770_for	GGGGACAAGTTTGTACAAAAAAGCAGGCTATGAAGAAAGCATGGGCTCAACTG	Tb927.3.2770_rev	GGGGACCACTTTGTACAAGAAAGCTGGGTTTAGCCTTCGCACCAAACTAAGATATC
10_hypothetical	P4	Tb927.11.1580_for	GGGGACAAGTTTGTACAAAAAAGCAGGCTATGCCAGCTTTTTCTTCACTCTCTAAG	Tb927.11.1580_rev	GGGGACCACTTTGTACAAGAAAGCTGGGTTCAAGTAGACTTCAAACAGCGCC
10_hypothetical	P5	Tb927.1.2980_for	GGGGACAAGTTTGTACAAAAAAGCAGGCTATGTTGTTGTCCATCCCGGTAAGA	Tb927.1.2980_rev	GGGGACCACTTTGTACAAGAAAGCTGGGTTTACGGAACAGTTAGTCTCAATCTGTCA
10_hypothetical	P6	Tb927.4.4530_for	GGGGACAAGTTTGTACAAAAAAGCAGGCTATGTCGGCATCAACTAGTTTCCAC	Tb927.4.4530_rev	GGGGACCACTTTGTACAAGAAAGCTGGGTTCATACAAACTTAAGGGCACCACC
10_hypothetical	P7	Tb927.11.4010_for	GGGGACAAGTTTGTACAAAAAAGCAGGCTATGGTCAACCAAACAGCGGG	Tb927.11.4010_rev	GGGGACCACTTTGTACAAGAAAGCTGGGTTTATTCGTTAGTCTCCGCAGCAGC
10_hypothetical	P8	Tb927.9.6160_for	GGGGACAAGTTTGTACAAAAAAGCAGGCTATGACCTCCCTTTTACCAGCGG	Tb927.9.6160_rev	GGGGACCACTTTGTACAAGAAAGCTGGGTTCATTTGGTTGTAGAGGTTATACCGTTC
10_hypothetical	P9	Tb927.7.5370_for	GGGGACAAGTTTGTACAAAAAAGCAGGCTATGTCAGCGAGGGCAGCA	Tb927.7.5370_rev	GGGGACCACTTTGTACAAGAAAGCTGGGTTTATTTTTTTTGTGTGGTGCTCCCCT
10_hypothetical	P10	Tb927.3.1320_for	GGGGACAAGTTTGTACAAAAAAGCAGGCTATGAAGTACTTTGCATGTCGTTTGGA	Tb927.3.1320_rev	GGGGACCACTTTGTACAAGAAAGCTGGGTCTATGAAACATGACCGTCCACCG
10_hypothetical	P11	Tb927.4.2410_for	GGGGACAAGTTTGTACAAAAAAGCAGGCTATGGGGTTTTGGCCGCGT	Tb927.4.2410_rev	GGGGACCACTTTGTACAAGAAAGCTGGGTTTAACTGCAGGGATCACTTGGCG
10_hypothetical	P12	Tb927.11.16340_for	GGGGACAAGTTTGTACAAAAAAGCAGGCTATGACCACCGCAGCTTCCG	Tb927.11.16340_rev	GGGGACCACTTTGTACAAGAAAGCTGGGTTTAGCAGCAACCGTATCGTTCAA
10_hypothetical	P13	Tb927.9.13330_for	GGGGACAAGTTTGTACAAAAAAGCAGGCTATGAGTTTTTTGGTTAAGACGTTGCTC	Tb927.9.13330_rev	GGGGACCACTTTGTACAAGAAAGCTGGGTTTAACGCAAATCTGTACTGGGGTC
10_hypothetical	P14	Tb927.10.7290_for	GGGGACAAGTTTGTACAAAAAAGCAGGCTATGGGGAACGGCCAAATTAATGC	Tb927.10.7290_rev	GGGGACCACTTTGTACAAGAAAGCTGGGTTCATCGTCTTCTATCCGGCGAC
10_hypothetical	P15	Tb927.3.920_for	GGGGACAAGTTTGTACAAAAAAGCAGGCTATGATTCACCGAGAGTTAGCAGATTC	Tb927.3.920_rev	GGGGACCACTTTGTACAAGAAAGCTGGGTCTATACTTTCAACTCTTCAATCATATATCG
10_hypothetical	P16	Tb927.11.1340_for	GGGGACAAGTTTGTACAAAAAAGCAGGCTATGAGTTCTGTGAGGCTTCTACTTGG	Tb927.11.1340_rev	GGGGACCACTTTGTACAAGAAAGCTGGGTTTATTCATCAGTGAAGTATAATGGATACCA
10_hypothetical	P17	Tb927.8.3180_for	GGGGACAAGTTTGTACAAAAAAGCAGGCTATGTCCGGGCGCCGGGGG	Tb927.8.3180_rev	GGGGACCACTTTGTACAAGAAAGCTGGGTTCAAGGAATAAAGGAACCCAACTGGT
10_hypothetical	P18	Tb927.10.13260_for	GGGGACAAGTTTGTACAAAAAAGCAGGCTATGCAAACAACTACCGCAGCA	Tb927.10.13260_rev	GGGGACCACTTTGTACAAGAAAGCTGGGTTCATTCGCCTTGAGGATACAACCA
10_hypothetical	P19	Tb927.11.12390_for	GGGGACAAGTTTGTACAAAAAAGCAGGCTATGGCTGACCGAGCAGCT	Tb927.11.12390_rev	GGGGACCACTTTGTACAAGAAAGCTGGGTCTAACCGGAGTCGCCACGT
10_hypothetical	P20	Tb927.3.3550_for	GGGGACAAGTTTGTACAAAAAAGCAGGCTATGTCACACTCTGCCTTTTTGCA	Tb927.3.3550_rev	GGGGACCACTTTGTACAAGAAAGCTGGGTTTAAAGACTGTAGCAACGGAAATCGG
10_hypothetical	P21	Tb927.10.11780_for	GGGGACAAGTTTGTACAAAAAAGCAGGCTATGTCCTCGCGTGAGGAGAG	Tb927.10.11780_rev	GGGGACCACTTTGTACAAGAAAGCTGGGTTCATTCCGATGTGGGGATCGT
10_hypothetical	P22	Tb927.10.6010_for	GGGGACAAGTTTGTACAAAAAAGCAGGCTATGCCCAGGAAGGTGAAGACT	Tb927.10.6010_rev	GGGGACCACTTTGTACAAGAAAGCTGGGTCTATTCGTATTCCATCGTTGCGATGA
10_hypothetical	P23	Tb927.7.3050_for	GGGGACAAGTTTGTACAAAAAAGCAGGCTATGTTTCGGGTCCGAAGGCT	Tb927.7.3050_rev	GGGGACCACTTTGTACAAGAAAGCTGGGTTCAACTCCATTCTGATTTCTGATCGG
10_hypothetical	P24	Tb927.10.6930_for	GGGGACAAGTTTGTACAAAAAAGCAGGCTATGGAGGGAGAGGAGTTGCTTT	Tb927.10.6930_rev	GGGGACCACTTTGTACAAGAAAGCTGGGTTCACGCTACATAGATATAAGTGTCCGC
hypothetical_las	A1	Tb927.5.1910_for	GGGGACAAGTTTGTACAAAAAAGCAGGCTATGGAGAATCTCTCTGATGAGGATAAATCC	Tb927.5.1910_rev	GGGGACCACTTTGTACAAGAAAGCTGGGTTCAGAAGGATTGGGCACAGCA
hypothetical_las	A2	Tb927.1.4450_for	GGGGACAAGTTTGTACAAAAAAGCAGGCTATGTACACGCTGTACCCCCG	Tb927.1.4450_rev	GGGGACCACTTTGTACAAGAAAGCTGGGTTTAGCAACCCATTCTGTTATGTGCT
hypothetical_las	A3	Tb927.4.4500_for	GGGGACAAGTTTGTACAAAAAAGCAGGCTATGAGTGAGTGCCCACGCC	Tb927.4.4500_rev	GGGGACCACTTTGTACAAGAAAGCTGGGTTCAGTATTCTAACTTTTTTGCCCACTGT
hypothetical_las	A4	Tb927.8.7830_for	GGGGACAAGTTTGTACAAAAAAGCAGGCTATGAGTGGGCGCCTGTCT	Tb927.8.7830_rev	GGGGACCACTTTGTACAAGAAAGCTGGGTTCAAAGTCCTTGGCATCTTAACTCTTC
hypothetical_las	A5	Tb927.10.14340_for	GGGGACAAGTTTGTACAAAAAAGCAGGCTATGAGTGCTCACACACCGGA	Tb927.10.14340_rev	GGGGACCACTTTGTACAAGAAAGCTGGGTCTATTTGGACTTAACGAGTGAGGCG
hypothetical_las	A6	Tb927.10.15550_for	GGGGACAAGTTTGTACAAAAAAGCAGGCTATGCTGACTGCACTGCATGTAAC	Tb927.10.15550_rev	GGGGACCACTTTGTACAAGAAAGCTGGGTTTACGGTTCTATCCCGTCACCAC
hypothetical_las	A7	Tb927.10.2650_for	GGGGACAAGTTTGTACAAAAAAGCAGGCTATGATATCTCAACCTACTGGAACCACA	Tb927.10.2650_rev	GGGGACCACTTTGTACAAGAAAGCTGGGTCTAGCAGTGCTTCAACAGTAGTAAACT
hypothetical_las	A8	Tb927.5.2440_for	GGGGACAAGTTTGTACAAAAAAGCAGGCTATGGCTGTCATCCCTAAGGTGG	Tb927.5.2440_rev	GGGGACCACTTTGTACAAGAAAGCTGGGTTCATGAGGCAAGTTGCTGTTCAT
hypothetical_las	A9	Tb927.7.5500_for	GGGGACAAGTTTGTACAAAAAAGCAGGCTATGCAAAGTAGTCTCTCTATCTCACCTC	Tb927.7.5500_rev	GGGGACCACTTTGTACAAGAAAGCTGGGTCTAGAACCCCAGCTCCTCACG
hypothetical_las	A10	Tb927.4.960_for	GGGGACAAGTTTGTACAAAAAAGCAGGCTATGAGCCAGTTGACTGCCATTTT	Tb927.4.960_rev	GGGGACCACTTTGTACAAGAAAGCTGGGTTTAGGTGGAGGTGGAAGTTATCCC
hypothetical_las	A11	Tb927.8.2430_for	GGGGACAAGTTTGTACAAAAAAGCAGGCTATGAGTGATTTTGACAACAAATGTCAACC	Tb927.8.2430_rev	GGGGACCACTTTGTACAAGAAAGCTGGGTTTACACTTCTTCATCAGGCTGTTCTG
hypothetical_las	A12	Tb927.10.14920_for	GGGGACAAGTTTGTACAAAAAAGCAGGCTATGACTCAACCGGTGGATGACA	Tb927.10.14920_rev	GGGGACCACTTTGTACAAGAAAGCTGGGTCTACACCCTCAATGGAAAAGTGAAA
hypothetical_las	A13	Tb927.2.4400_for	GGGGACAAGTTTGTACAAAAAAGCAGGCTATGATTCGGAGACGAGTATGCGA	Tb927.2.4400_rev	GGGGACCACTTTGTACAAGAAAGCTGGGTTCAAAGTTGCACCCATTTGGGG
hypothetical_las	A14	Tb927.11.10710_for	GGGGACAAGTTTGTACAAAAAAGCAGGCTATGCGGCCCCAGGCTGAT	Tb927.11.10710_rev	GGGGACCACTTTGTACAAGAAAGCTGGGTTCATGCAACGAAGCATGATTCCC
hypothetical_las	A15	Tb927.1.3860_for	GGGGACAAGTTTGTACAAAAAAGCAGGCTATGGCGTCAAACCAACAGGAC	Tb927.1.3860_rev	GGGGACCACTTTGTACAAGAAAGCTGGGTTCAAAACCGGGGCTGCAAC
hypothetical_las	A16	Tb927.10.4080_for	GGGGACAAGTTTGTACAAAAAAGCAGGCTATGCGGCGATTTGCACCT	Tb927.10.4080_rev	GGGGACCACTTTGTACAAGAAAGCTGGGTTCACATGCGTGCGGACTG
hypothetical_las	A17	Tb927.7.5190_for	GGGGACAAGTTTGTACAAAAAAGCAGGCTATGGAGCGACTATCTATTATCCACACA	Tb927.7.5190_rev	GGGGACCACTTTGTACAAGAAAGCTGGGTTCAATTTTCGCTGTTTACTAGAGTGGAT
hypothetical_las	A18	Tb927.8.5110_for	GGGGACAAGTTTGTACAAAAAAGCAGGCTATGAGGGGGTTGCTGTCTCAC	Tb927.8.5110_rev	GGGGACCACTTTGTACAAGAAAGCTGGGTTTACTGTAGTATCACAACAGGACTTGTTC
hypothetical_las	A19	Tb927.5.3280_for	GGGGACAAGTTTGTACAAAAAAGCAGGCTATGGCATCCTTGCAGTTGGG	Tb927.5.3280_rev	GGGGACCACTTTGTACAAGAAAGCTGGGTTTACTGCGCAGAAGTATATGGGAGT
hypothetical_las	A20	Tb927.5.3290_for	GGGGACAAGTTTGTACAAAAAAGCAGGCTATGCAAATCACAGGAGTGCGG	Tb927.5.3290_rev	GGGGACCACTTTGTACAAGAAAGCTGGGTTTACTTCTTTGGTGTTGCAACACTTTTT
hypothetical_las	A21	Tb927.8.5170_for	GGGGACAAGTTTGTACAAAAAAGCAGGCTATGAAGGAAGCCGAACAACCAC	Tb927.8.5170_rev	GGGGACCACTTTGTACAAGAAAGCTGGGTTTAATAGACATCTTTGTGATGTTTGATTAC
hypothetical_las	A22	Tb927.10.15280_for	GGGGACAAGTTTGTACAAAAAAGCAGGCTATGCAGCGGGAAGTGATACGT	Tb927.10.15280_rev	GGGGACCACTTTGTACAAGAAAGCTGGGTTTACATTGCACTGAGCCACCC
hypothetical_las	A23	Tb927.8.7800_for	GGGGACAAGTTTGTACAAAAAAGCAGGCTATGTCAACACCAATTGGTTCTCGC	Tb927.8.7800_rev	GGGGACCACTTTGTACAAGAAAGCTGGGTTTACCTGAACTCAATGGCACCAC
hypothetical_las	A24	Tb927.11.4230_for	GGGGACAAGTTTGTACAAAAAAGCAGGCTATGGTGGACGAAGAGACGCT	Tb927.11.4230_rev	GGGGACCACTTTGTACAAGAAAGCTGGGTTCACGAGCTACGTGCTGCA
hypothetical_las	B1	Tb927.10.8180_for	GGGGACAAGTTTGTACAAAAAAGCAGGCTATGAGCGGGTCGCGTCTG	Tb927.10.8180_rev	GGGGACCACTTTGTACAAGAAAGCTGGGTCTAAAGCAGCCGCTCCAAGG
hypothetical_las	B2	Tb927.8.3870_for	GGGGACAAGTTTGTACAAAAAAGCAGGCTATGCCAGTTACTGTCGAGGAAGT	Tb927.8.3870_rev	GGGGACCACTTTGTACAAGAAAGCTGGGTCTATTCACTGTCCTCAAACTGAAACGA
hypothetical_las	B3	Tb927.7.2560_for	GGGGACAAGTTTGTACAAAAAAGCAGGCTATGCAACGCCTGCTTCATGC	Tb927.7.2560_rev	GGGGACCACTTTGTACAAGAAAGCTGGGTTTATTCAATAAGAAAAAATGTGAAGGGCCC
hypothetical_las	B4	Tb927.2.2340_for	GGGGACAAGTTTGTACAAAAAAGCAGGCTATGGCCGCCGTAGTGCAG	Tb927.2.2340_rev	GGGGACCACTTTGTACAAGAAAGCTGGGTTTATGTTGACACCGTCATTCCCCC
hypothetical_las	B5	Tb927.8.5160_for	GGGGACAAGTTTGTACAAAAAAGCAGGCTATGGGTGTGTGCCAGTCAAATC	Tb927.8.5160_rev	GGGGACCACTTTGTACAAGAAAGCTGGGTTCAGCAAGTGAGCGTAACTCCG
hypothetical_las	B6	Tb927.11.10150_for	GGGGACAAGTTTGTACAAAAAAGCAGGCTATGGAGCGAAGGAGGTTGTTGC	Tb927.11.10150_rev	GGGGACCACTTTGTACAAGAAAGCTGGGTTCAGCTGCTATCGTCCCCATC
hypothetical_las	B7	Tb927.3.4030_for	GGGGACAAGTTTGTACAAAAAAGCAGGCTATGACCTTTAGCACGCTTGTGC	Tb927.3.4030_rev	GGGGACCACTTTGTACAAGAAAGCTGGGTCTATTCAAGTGGCGGTAGGTCAGT
hypothetical_las	B8	Tb927.11.16010_for	GGGGACAAGTTTGTACAAAAAAGCAGGCTATGAACCCCACTGATCTTCGTGA	Tb927.11.16010_rev	GGGGACCACTTTGTACAAGAAAGCTGGGTTTATCGCACATCACTAAAAAAGCCCA
hypothetical_las	B9	Tb927.10.15240_for	GGGGACAAGTTTGTACAAAAAAGCAGGCTATGCAGAGCGCGGGAAGC	Tb927.10.15240_rev	GGGGACCACTTTGTACAAGAAAGCTGGGTCTATCGATCGCGCGCACG
hypothetical_las	B10	Tb927.11.9080_for	GGGGACAAGTTTGTACAAAAAAGCAGGCTATGGGAGCCGACGACGAG	Tb927.11.9080_rev	GGGGACCACTTTGTACAAGAAAGCTGGGTTTACTTCCGTGACTTGTGCTGTG
hypothetical_las	B11	Tb927.11.2320_for	GGGGACAAGTTTGTACAAAAAAGCAGGCTATGAGCCAACAAAAGGGGAAGAAA	Tb927.11.2320_rev	GGGGACCACTTTGTACAAGAAAGCTGGGTTCAGCCTTTTCCTTTAGTAACGGGC
hypothetical_las	B12	Tb927.11.15400_for	GGGGACAAGTTTGTACAAAAAAGCAGGCTATGTTCGAAAATTTGATGCGATGGC	Tb927.11.15400_rev	GGGGACCACTTTGTACAAGAAAGCTGGGTTTAGAACCAGCTATTTTTTTGCTTCTGATT
hypothetical_las	B13	Tb927.10.1490_for	GGGGACAAGTTTGTACAAAAAAGCAGGCTATGAGGAAAGCTGGAGCAAATCG	Tb927.10.1490_rev	GGGGACCACTTTGTACAAGAAAGCTGGGTCTAGTGAGGAATCGGTTCTTGGTG
hypothetical_las	B14	Tb927.11.910_for	GGGGACAAGTTTGTACAAAAAAGCAGGCTATGGAGGTGTTCGATCTGGCG	Tb927.11.910_rev	GGGGACCACTTTGTACAAGAAAGCTGGGTTCACGTCACTTTGATTGCTTCTGG
hypothetical_las	B15	Tb927.10.10050_for	GGGGACAAGTTTGTACAAAAAAGCAGGCTATGTTGTTGGGGTTGAAAGATCTTTTTATT	Tb927.10.10050_rev	GGGGACCACTTTGTACAAGAAAGCTGGGTCTACTGATGAACTCCGGGGCT
hypothetical_las	B16	Tb927.11.890_for	GGGGACAAGTTTGTACAAAAAAGCAGGCTATGAAGAAACTCAGATCATTTCTTCCCTG	Tb927.11.890_rev	GGGGACCACTTTGTACAAGAAAGCTGGGTCTACTCTCTTGAAAGCTCACGCAA
hypothetical_las	B17	Tb927.10.3010_for	GGGGACAAGTTTGTACAAAAAAGCAGGCTATGCCATTACCATCCACGCTACC	Tb927.10.3010_rev	GGGGACCACTTTGTACAAGAAAGCTGGGTTTAGTCTTTCGGTTTAGACTGGGCA
hypothetical_las	B18	Tb927.9.11670_for	GGGGACAAGTTTGTACAAAAAAGCAGGCTATGCGGCCGCCGACGTTA	Tb927.9.11670_rev	GGGGACCACTTTGTACAAGAAAGCTGGGTCTAAGAGTGGACAGAAGTCACACG
hypothetical_las	B19	Tb927.3.5160_for	GGGGACAAGTTTGTACAAAAAAGCAGGCTATGAGTGACCGTCCCCGAC	Tb927.3.5160_rev	GGGGACCACTTTGTACAAGAAAGCTGGGTTTATTTGTCCGTCAGGTCCACAAC
hypothetical_las	B20	Tb927.10.13180_for	GGGGACAAGTTTGTACAAAAAAGCAGGCTATGCTCCCTACGCCATACCAT	Tb927.10.13180_rev	GGGGACCACTTTGTACAAGAAAGCTGGGTTTACTTCACTTTTCTCTTCGCTACTTTTTC
hypothetical_las	B21	Tb927.2.4050_for	GGGGACAAGTTTGTACAAAAAAGCAGGCTATGTCTTCGAGTCGTGGAAGTTTTC	Tb927.2.4050_rev	GGGGACCACTTTGTACAAGAAAGCTGGGTTCAATTCTTCTTCGGTGATATCGATGG
hypothetical_las	B22	Tb927.7.3550_for	GGGGACAAGTTTGTACAAAAAAGCAGGCTATGTCTATATTCGGTGAACCACTTCCC	Tb927.7.3550_rev	GGGGACCACTTTGTACAAGAAAGCTGGGTTTAGAAACCTCGCCCAACGGA
hypothetical_las	B23	Tb927.2.5860_for	GGGGACAAGTTTGTACAAAAAAGCAGGCTATGGTTGCCACGGAACAATCAA	Tb927.2.5860_rev	GGGGACCACTTTGTACAAGAAAGCTGGGTTCACGATGGCTCAACCTCACT

hypothetical_las	B24	Tb927.9.13050_for	GGGGACAAGTTTGTACAAAAAAGCAGGCTATGAAGGATCCTGTAGTTAGCGGT	Tb927.9.13050_rev	GGGGACCACTTTGTACAAGAAAGCTGGGTTCAGATGTGTTTCCACTGCAACC
hypothetical_las	C1	Tb927.8.4160_for	GGGGACAAGTTTGTACAAAAAAGCAGGCTATGGAAAACAGCACAGTAGCCTTC	Tb927.8.4160_rev	GGGGACCACTTTGTACAAGAAAGCTGGGTTTAAAAACAAAGCTTCAGTGCTTTCGC
hypothetical_las	C2	Tb927.11.8960_for	GGGGACAAGTTTGTACAAAAAAGCAGGCTATGTCCGCATTTGAGTTTCTTCGG	Tb927.11.8960_rev	GGGGACCACTTTGTACAAGAAAGCTGGGTTCACGCCATGTCCGGAAGA
hypothetical_las	C3	Tb927.11.14040_for	GGGGACAAGTTTGTACAAAAAAGCAGGCTATGCGTTTTCCTAGCGGCTC	Tb927.11.14040_rev	GGGGACCACTTTGTACAAGAAAGCTGGGTCTAACTTACATTTTCAAGGCAATACCGTAT
hypothetical_las	C4	Tb927.7.4150_for	GGGGACAAGTTTGTACAAAAAAGCAGGCTATGGTCGTTCATCTTACCCCACT	Tb927.7.4150_rev	GGGGACCACTTTGTACAAGAAAGCTGGGTTCATTTCGTCCGCCGAAATTTCT
hypothetical_las	C5	Tb927.10.13070_for	GGGGACAAGTTTGTACAAAAAAGCAGGCTATGACGGATAAGGAAGATGCCAGT	Tb927.10.13070_rev	GGGGACCACTTTGTACAAGAAAGCTGGGTTTAGCTGTGAAACCAGCGGAC
hypothetical_las	C6	Tb927.3.1310_for	GGGGACAAGTTTGTACAAAAAAGCAGGCTATGTTGCTTTGCAAGCAACCAG	Tb927.3.1310_rev	GGGGACCACTTTGTACAAGAAAGCTGGGTTCACACACCACAACGCTCATTT
hypothetical_las	C7	Tb927.2.4520_for	GGGGACAAGTTTGTACAAAAAAGCAGGCTATGAAGCGTGTTCTCGTGCTC	Tb927.2.4520_rev	GGGGACCACTTTGTACAAGAAAGCTGGGTTCAAATCTCCGTTCGCCGC
hypothetical_las	C8	Tb927.11.5910_for	GGGGACAAGTTTGTACAAAAAAGCAGGCTATGCTCACAACCTTCGATTTTGGT	Tb927.11.5910_rev	GGGGACCACTTTGTACAAGAAAGCTGGGTCTAGCCTTCATCTGCATCTGTACCT
hypothetical_las	C9	Tb927.8.4560_for	GGGGACAAGTTTGTACAAAAAAGCAGGCTATGACCTTGACAGGCCTAAGCA	Tb927.8.4560_rev	GGGGACCACTTTGTACAAGAAAGCTGGGTCTACCAGCCGGTGACACTTTC
hypothetical_las	C10	Tb927.7.7100_for	GGGGACAAGTTTGTACAAAAAAGCAGGCTATGTCGCTTGCTTACCTCAAAAGG	Tb927.7.7100_rev	GGGGACCACTTTGTACAAGAAAGCTGGGTTTAAGCAATATTTACACCCCGCCC
hypothetical_las	C11	Tb927.11.6430_for	GGGGACAAGTTTGTACAAAAAAGCAGGCTATGGAGTCCGTGCCGCGC	Tb927.11.6430_rev	GGGGACCACTTTGTACAAGAAAGCTGGGTTCAAAAAGACCACTGCGCAGT
hypothetical_las	C12	Tb927.6.1180_for	GGGGACAAGTTTGTACAAAAAAGCAGGCTATGACTGATTTCGGGCGCG	Tb927.6.1180_rev	GGGGACCACTTTGTACAAGAAAGCTGGGTTCATGCACCCTCCTCCTCCG
hypothetical_las	C13	Tb927.4.2640_for	GGGGACAAGTTTGTACAAAAAAGCAGGCTATGTACGGCGTCACCCATCC	Tb927.4.2640_rev	GGGGACCACTTTGTACAAGAAAGCTGGGTCTAGAAGCAAGAGCCGCGG
hypothetical_las	C14	Tb927.6.1730_for	GGGGACAAGTTTGTACAAAAAAGCAGGCTATGCAGCAGAAGGTACTGATTGC	Tb927.6.1730_rev	GGGGACCACTTTGTACAAGAAAGCTGGGTTCACCAATCACTATCACTGCTATCAAATG
hypothetical_las	C15	Tb927.9.12490_for	GGGGACAAGTTTGTACAAAAAAGCAGGCTATGCAAGACATGCGTATGTTTAATGTATG	Tb927.9.12490_rev	GGGGACCACTTTGTACAAGAAAGCTGGGTCTATGAACTGTCTTGAAGGAATGAGGC
hypothetical_las	C16	Tb927.9.12990_for	GGGGACAAGTTTGTACAAAAAAGCAGGCTATGAAACTCTTCACACCAGAGGAGA	Tb927.9.12990_rev	GGGGACCACTTTGTACAAGAAAGCTGGGTCTACAACACAGTCGCGCCTC
hypothetical_las	C17	Tb927.4.2990_for	GGGGACAAGTTTGTACAAAAAAGCAGGCTATGTCTCTTTCCCTGGGTTGTAGG	Tb927.4.2990_rev	GGGGACCACTTTGTACAAGAAAGCTGGGTTTATCGCTTGAGTAAGGGCAATTTATTCC
hypothetical_las	C18	Tb927.11.2510_for	GGGGACAAGTTTGTACAAAAAAGCAGGCTATGAAACCTCCTAGTGCGGCT	Tb927.11.2510_rev	GGGGACCACTTTGTACAAGAAAGCTGGGTTTATTGGCTTAGATTCACTTTTTGTTTTTT
hypothetical_las	C19	Tb927.9.10450_for	GGGGACAAGTTTGTACAAAAAAGCAGGCTATGGTGGGAAAAAGGGGTCGT	Tb927.9.10450_rev	GGGGACCACTTTGTACAAGAAAGCTGGGTCTACCAGAAACTCAGCAGTGCG
hypothetical_las	C20	Tb927.8.2820_for	GGGGACAAGTTTGTACAAAAAAGCAGGCTATGCCGCTATACAATATCACTATTCATCC	Tb927.8.2820_rev	GGGGACCACTTTGTACAAGAAAGCTGGGTCTATACTGGAAAACGGGCGGC
hypothetical_las	C21	Tb927.11.620_for	GGGGACAAGTTTGTACAAAAAAGCAGGCTATGAGGAACCGGCGGCGT	Tb927.11.620_rev	GGGGACCACTTTGTACAAGAAAGCTGGGTTCAGTGCACAACTAAAATCTCACCTC
hypothetical_las	C22	Tb927.8.790_for	GGGGACAAGTTTGTACAAAAAAGCAGGCTATGACAGGCACAACCGAAAACC	Tb927.8.790_rev	GGGGACCACTTTGTACAAGAAAGCTGGGTCTAAACGTATTTTGCGGGATCAGAAAAG
hypothetical_las	C23	Tb927.8.3080_for	GGGGACAAGTTTGTACAAAAAAGCAGGCTATGCTGAGAAACTTGCAAGCCC	Tb927.8.3080_rev	GGGGACCACTTTGTACAAGAAAGCTGGGTTCATAGAGGCGCTACATCCTCC
hypothetical_las	C24	Tb927.7.1150_for	GGGGACAAGTTTGTACAAAAAAGCAGGCTATGCGAGGATCCAATAGGGCG	Tb927.7.1150_rev	GGGGACCACTTTGTACAAGAAAGCTGGGTTTACCACTTCTTGCCCCCGT
hypothetical_las	D1	Tb927.5.1010_for	GGGGACAAGTTTGTACAAAAAAGCAGGCTATGGAAACTGCCGCAGAGC	Tb927.5.1010_rev	GGGGACCACTTTGTACAAGAAAGCTGGGTTCACCTTCGCATGTCCCCG
hypothetical_las	D2	Tb927.7.7250_for	GGGGACAAGTTTGTACAAAAAAGCAGGCTATGCCACCCAGACCTAGGC	Tb927.7.7250_rev	GGGGACCACTTTGTACAAGAAAGCTGGGTCTACGCTCTGCAATCCCCCC
hypothetical_las	D3	Tb927.7.7190_for	GGGGACAAGTTTGTACAAAAAAGCAGGCTATGGGAAAAAAGAAGGCGGAAAGG	Tb927.7.7190_rev	GGGGACCACTTTGTACAAGAAAGCTGGGTTTACACTCCGACACTGCCGG
hypothetical_las	D4	Tb927.11.6420_for	GGGGACAAGTTTGTACAAAAAAGCAGGCTATGTCTCTTGGGAAAGTATTGCGTT	Tb927.11.6420_rev	GGGGACCACTTTGTACAAGAAAGCTGGGTCTACTTCTCGTTCGCCTCATTGTC
hypothetical_las	D5	Tb927.10.8000_for	GGGGACAAGTTTGTACAAAAAAGCAGGCTATGTTGGATGCGGAGGACTTTG	Tb927.10.8000_rev	GGGGACCACTTTGTACAAGAAAGCTGGGTTCAATCTACATCGATGGCAAATGTCTT
hypothetical_las	D6	Tb927.3.4510_for	GGGGACAAGTTTGTACAAAAAAGCAGGCTATGGGTGAAGTAATGATTTTGCGAGA	Tb927.3.4510_rev	GGGGACCACTTTGTACAAGAAAGCTGGGTTTAGTCAATGTACGGGACGCGG
hypothetical_las	D7	Tb927.2.4810_for	GGGGACAAGTTTGTACAAAAAAGCAGGCTATGGAGGAGAGGAAAGGTCACG	Tb927.2.4810_rev	GGGGACCACTTTGTACAAGAAAGCTGGGTCTACACAAGTGGGGGAGCG
hypothetical_las	D8	Tb927.9.14190_for	GGGGACAAGTTTGTACAAAAAAGCAGGCTATGAGCGAGTGGGAAGTAGATGAG	Tb927.9.14190_rev	GGGGACCACTTTGTACAAGAAAGCTGGGTTCAGGAAGCAACCATGAGCTCA
hypothetical_las	D9	Tb927.4.2470_for	GGGGACAAGTTTGTACAAAAAAGCAGGCTATGGAGCTACTCAACGTGTTACCG	Tb927.4.2470_rev	GGGGACCACTTTGTACAAGAAAGCTGGGTTCATATTTGCTCAGTCTTTTGAATGGTGC
hypothetical_las	D10	Tb927.9.4630_for	GGGGACAAGTTTGTACAAAAAAGCAGGCTATGCAGAGTTTGGTCTCTTTGCG	Tb927.9.4630_rev	GGGGACCACTTTGTACAAGAAAGCTGGGTTCAGCAAACAAAAAGATCTTCCGCT
hypothetical_las	D11	Tb927.9.5620_for	GGGGACAAGTTTGTACAAAAAAGCAGGCTATGGAGGCGATTTACTTTACTACTAAGC	Tb927.9.5620_rev	GGGGACCACTTTGTACAAGAAAGCTGGGTCTAAATCGCGCAATTTGCTCCTTG
hypothetical_las	D12	Tb927.7.1310_for	GGGGACAAGTTTGTACAAAAAAGCAGGCTATGGAGTCAAAACGCTTTTCGGT	Tb927.7.1310_rev	GGGGACCACTTTGTACAAGAAAGCTGGGTTCACTCCTGTCGACCCTCATTT
hypothetical_las	D13	Tb927.10.870_for	GGGGACAAGTTTGTACAAAAAAGCAGGCTATGAGCGAGCCCAGGGAAG	Tb927.10.870_rev	GGGGACCACTTTGTACAAGAAAGCTGGGTTTAGAGGGCCTCTCGTTGCG
hypothetical_las	D14	Tb927.3.2070_for	GGGGACAAGTTTGTACAAAAAAGCAGGCTATGGGAATGAGACGAAGCCGG	Tb927.3.2070_rev	GGGGACCACTTTGTACAAGAAAGCTGGGTTTAGTCGAGCACATGCCGTG
hypothetical_las	D15	Tb927.10.5420_for	GGGGACAAGTTTGTACAAAAAAGCAGGCTATGCGTGTGACATGTGTAGTTGC	Tb927.10.5420_rev	GGGGACCACTTTGTACAAGAAAGCTGGGTTTATCGCCTGTTGCAACGCA
hypothetical_las	D16	Tb927.10.11470_for	GGGGACAAGTTTGTACAAAAAAGCAGGCTATGTTATGCCGACGTTTCCTCG	Tb927.10.11470_rev	GGGGACCACTTTGTACAAGAAAGCTGGGTCTACTCACGGTGGCATACCCC
hypothetical_las	D17	Tb927.10.5390_for	GGGGACAAGTTTGTACAAAAAAGCAGGCTATGATGCAAAATGGTTTCGTTCTAATCC	Tb927.10.5390_rev	GGGGACCACTTTGTACAAGAAAGCTGGGTTTACCAAAACCAGCGAAGCAAGT
hypothetical_las	D18	Tb927.9.3680_for	GGGGACAAGTTTGTACAAAAAAGCAGGCTATGGCCCAGTGCTTTGGAAGT	Tb927.9.3680_rev	GGGGACCACTTTGTACAAGAAAGCTGGGTTCATTTGTCCTTCGAGGGGATCC
hypothetical_las	D19	Tb927.8.4360_for	GGGGACAAGTTTGTACAAAAAAGCAGGCTATGACTTTCGTTGACCTATTTGACATGA	Tb927.8.4360_rev	GGGGACCACTTTGTACAAGAAAGCTGGGTCTACCACCGCAAGATTTCTTGTTCC
hypothetical_las	D20	Tb927.7.2150_for	GGGGACAAGTTTGTACAAAAAAGCAGGCTATGGAGCGGACGAACCGT	Tb927.7.2150_rev	GGGGACCACTTTGTACAAGAAAGCTGGGTCTACACGCAAACAGGGCGT
hypothetical_las	D21	Tb927.10.14230_for	GGGGACAAGTTTGTACAAAAAAGCAGGCTATGGATGATCCATGCAGCGC	Tb927.10.14230_rev	GGGGACCACTTTGTACAAGAAAGCTGGGTTCAGTCACTCGCGTAGCCTAA
hypothetical_las	D22	Tb927.9.2730_for	GGGGACAAGTTTGTACAAAAAAGCAGGCTATGCTAAACTACACTTACGTGGAGAAG	Tb927.9.2730_rev	GGGGACCACTTTGTACAAGAAAGCTGGGTTCATACGTAGCGCTCAGCAAAC
hypothetical_las	D23	Tb927.4.5120_for	GGGGACAAGTTTGTACAAAAAAGCAGGCTATGGCTGCTCGCCGCCAT	Tb927.4.5120_rev	GGGGACCACTTTGTACAAGAAAGCTGGGTTCATTTCCGGAACACGAACGTC
hypothetical_las	D24	Tb927.9.15290_for	GGGGACAAGTTTGTACAAAAAAGCAGGCTATGAATCCAAGTGCCCAGCCG	Tb927.9.15290_rev	GGGGACCACTTTGTACAAGAAAGCTGGGTTCACGAGATCGGCAGGCC
hypothetical_las	E1	Tb927.10.11340_for	GGGGACAAGTTTGTACAAAAAAGCAGGCTATGATGCGTCGTATGACTGCAAC	Tb927.10.11340_rev	GGGGACCACTTTGTACAAGAAAGCTGGGTTTAGTTGCGGACCTTTCTTGACTTC
hypothetical_las	E2	Tb927.7.4370_for	GGGGACAAGTTTGTACAAAAAAGCAGGCTATGGAGGGTGTTGCTTCATCCA	Tb927.7.4370_rev	GGGGACCACTTTGTACAAGAAAGCTGGGTCTAGTAACCTGCCAAGTCCCAGA
hypothetical_las	E3	Tb927.8.1280_for	GGGGACAAGTTTGTACAAAAAAGCAGGCTATGTCATATGAAAAGATACTATGTATGGAA	Tb927.8.1280_rev	GGGGACCACTTTGTACAAGAAAGCTGGGTCTAGAGAGTCCACGGCACAATATTTC
hypothetical_las	E4	Tb927.2.2370_for	GGGGACAAGTTTGTACAAAAAAGCAGGCTATGTCATCGGAGGAAGCCATTCA	Tb927.2.2370_rev	GGGGACCACTTTGTACAAGAAAGCTGGGTCTACCACAATGAGCCCATCCAC
hypothetical_las	E5	Tb927.5.2510_for	GGGGACAAGTTTGTACAAAAAAGCAGGCTATGAATCGCAAGGGGTATAAGGCT	Tb927.5.2510_rev	GGGGACCACTTTGTACAAGAAAGCTGGGTTTACATGTCGTTTGCGTTAATGTGATAC
hypothetical_las	E6	Tb927.7.3930_for	GGGGACAAGTTTGTACAAAAAAGCAGGCTATGATGTTTTTTTCTCTGTGCCGTAGA	Tb927.7.3930_rev	GGGGACCACTTTGTACAAGAAAGCTGGGTCTAATCGTCTTCACTACCGAGTAGCT
hypothetical_las	E7	Tb927.7.1570_for	GGGGACAAGTTTGTACAAAAAAGCAGGCTATGTTTCGTGTCTCCTTCCCATTG	Tb927.7.1570_rev	GGGGACCACTTTGTACAAGAAAGCTGGGTTTATTCTCCCCTTACCTCCGTGC
hypothetical_las	E8	Tb927.5.3260_for	GGGGACAAGTTTGTACAAAAAAGCAGGCTATGGATCCACTAAATGCAGTTGCG	Tb927.5.3260_rev	GGGGACCACTTTGTACAAGAAAGCTGGGTTTACAATGGCAGCAAGTGAGCA
hypothetical_las	E9	Tb927.11.4520_for	GGGGACAAGTTTGTACAAAAAAGCAGGCTATGTTCCGCGAGGCACTTG	Tb927.11.4520_rev	GGGGACCACTTTGTACAAGAAAGCTGGGTCTACAAGTTTACCACATTCCACGGC
hypothetical_las	E10	Tb927.5.500_for	GGGGACAAGTTTGTACAAAAAAGCAGGCTATGTTACCACCAATACGCCGC	Tb927.5.500_rev	GGGGACCACTTTGTACAAGAAAGCTGGGTCTATAGCTGAACTGAATACTTGTCGGTC
hypothetical_las	E11	Tb927.8.3200_for	GGGGACAAGTTTGTACAAAAAAGCAGGCTATGGTTACCACTGGTCTTAGTCCTG	Tb927.8.3200_rev	GGGGACCACTTTGTACAAGAAAGCTGGGTTCAGCCACGCTGTGGTTCC
hypothetical_las	E12	Tb927.10.1970_for	GGGGACAAGTTTGTACAAAAAAGCAGGCTATGGCCCAACCGGTGGAT	Tb927.10.1970_rev	GGGGACCACTTTGTACAAGAAAGCTGGGTTCAGGCCTCCCCCGGCGG
hypothetical_las	E13	Tb927.10.9650_for	GGGGACAAGTTTGTACAAAAAAGCAGGCTATGTTGAGCACCCTTTTCTTCGAT	Tb927.10.9650_rev	GGGGACCACTTTGTACAAGAAAGCTGGGTCTATAGCCCTGCTAACTTCTTCTGC
hypothetical_las	E14	Tb927.7.5390_for	GGGGACAAGTTTGTACAAAAAAGCAGGCTATGGAGAGAACGGAGGATGTAACA	Tb927.7.5390_rev	GGGGACCACTTTGTACAAGAAAGCTGGGTTCAACCAAACAATGTTTCTGATTTTGGC
hypothetical_las	E15	Tb927.3.5020_for	GGGGACAAGTTTGTACAAAAAAGCAGGCTATGGGAATACAAGGTGATTTGGCTC	Tb927.3.5020_rev	GGGGACCACTTTGTACAAGAAAGCTGGGTTTATTGGCGACCATCAGCTGAAG
hypothetical_las	E16	Tb927.3.4620_for	GGGGACAAGTTTGTACAAAAAAGCAGGCTATGCGCGACATGGAGGTGG	Tb927.3.4620_rev	GGGGACCACTTTGTACAAGAAAGCTGGGTCTAATCTGCGCTCATCATGACCTC
hypothetical_las	E17	Tb927.2.4950_for	GGGGACAAGTTTGTACAAAAAAGCAGGCTATGCTGAAGCCACCTTTTATCCG	Tb927.2.4950_rev	GGGGACCACTTTGTACAAGAAAGCTGGGTTCATTCATGCAGCGGCAACA
hypothetical_las	E18	Tb927.3.1330_for	GGGGACAAGTTTGTACAAAAAAGCAGGCTATGCGAGGAACTGTTTTGATCTTTGG	Tb927.3.1330_rev	GGGGACCACTTTGTACAAGAAAGCTGGGTTCACCAAATAGAATCAGTCGCATTAATGA
hypothetical_las	E19	Tb927.11.4280_for	GGGGACAAGTTTGTACAAAAAAGCAGGCTATGGCAGCACCCCTTCATTCCT	Tb927.11.4280_rev	GGGGACCACTTTGTACAAGAAAGCTGGGTTTACTCCATATCATGGCAAAACCCC
hypothetical_las	E20	Tb927.10.7610_for	GGGGACAAGTTTGTACAAAAAAGCAGGCTATGCGTCGCCTACAAATTCGG	Tb927.10.7610_rev	GGGGACCACTTTGTACAAGAAAGCTGGGTCTACTTTGTTTCGAATAGTTCCTTTGTAGA
hypothetical_las	E21	Tb927.11.2290_for	GGGGACAAGTTTGTACAAAAAAGCAGGCTATGACTACATGGCTCTTCAATGTAAGTG	Tb927.11.2290_rev	GGGGACCACTTTGTACAAGAAAGCTGGGTTTATGAAGAGTCACTTTTGGATAGGAACAT
hypothetical_las	E22	Tb927.8.7490_for	GGGGACAAGTTTGTACAAAAAAGCAGGCTATGGAAGCCTTCATCGAAGCG	Tb927.8.7490_rev	GGGGACCACTTTGTACAAGAAAGCTGGGTTTATAGACTACCGAGAATATGACCAAAGTA
hypothetical_las	E24	Tb927.9.10100_for	GGGGACAAGTTTGTACAAAAAAGCAGGCTATGGTAATGAAAGCCAATTTCTGCTTC	Tb927.9.10100_rev	GGGGACCACTTTGTACAAGAAAGCTGGGTTTACTCACCGTCACGAGCGA
hypothetical_las	F1	Tb927.8.6790_for	GGGGACAAGTTTGTACAAAAAAGCAGGCTATGGTGACGATGCGGATGGA	Tb927.8.6790_rev	GGGGACCACTTTGTACAAGAAAGCTGGGTTTAAACAGTCCTCCCCACTGTGG
hypothetical_las	F2	Tb927.11.6410_for	GGGGACAAGTTTGTACAAAAAAGCAGGCTATGAGCTCAAGCGTCCTCCA	Tb927.11.6410_rev	GGGGACCACTTTGTACAAGAAAGCTGGGTCTAGCGACGCTCAGATTCCTGC
hypothetical_las	F3	Tb927.7.5150_for	GGGGACAAGTTTGTACAAAAAAGCAGGCTATGCCGGCAAAAAGCCGG	Tb927.7.5150_rev	GGGGACCACTTTGTACAAGAAAGCTGGGTTCATGTTAGCGGCGCAAGG
hypothetical_las	F4	Tb927.7.6410_for	GGGGACAAGTTTGTACAAAAAAGCAGGCTATGTCTTCGGGGGCTGTACTG	Tb927.7.6410_rev	GGGGACCACTTTGTACAAGAAAGCTGGGTTCAAGCAGTGGAAGCCGCC
hypothetical_las	F5	Tb927.8.4960_for	GGGGACAAGTTTGTACAAAAAAGCAGGCTATGCTGCGGCATACGACG	Tb927.8.4960_rev	GGGGACCACTTTGTACAAGAAAGCTGGGTTTAATTGCAGTGATCAAGCAACAGAAG
hypothetical_las	F6	Tb927.4.5380_for	GGGGACAAGTTTGTACAAAAAAGCAGGCTATGGCAACCAACGCGCGC	Tb927.4.5380_rev	GGGGACCACTTTGTACAAGAAAGCTGGGTTCATCTGGCGGTCCTCCTCG
hypothetical_las	F8	Tb927.3.1800_for	GGGGACAAGTTTGTACAAAAAAGCAGGCTATGAAGCGGGAAAAGCGACAA	Tb927.3.1800_rev	GGGGACCACTTTGTACAAGAAAGCTGGGTTCATTGGCCTCTGTCACGTTTTC
hypothetical_las	F9	Tb927.8.2390_for	GGGGACAAGTTTGTACAAAAAAGCAGGCTATGGCAACGCTTCGGTTCAA	Tb927.8.2390_rev	GGGGACCACTTTGTACAAGAAAGCTGGGTTCACTTCGTCGCCTTCTTTTTTTTG
hypothetical_las	F10	Tb927.8.4320_for	GGGGACAAGTTTGTACAAAAAAGCAGGCTATGGAACGACTTGATGAACTCCAGC	Tb927.8.4320_rev	GGGGACCACTTTGTACAAGAAAGCTGGGTTCACGAGTCTTCTCTGTACGTACAA
hypothetical_las	F11	Tb927.4.2400_for	GGGGACAAGTTTGTACAAAAAAGCAGGCTATGGAGGGAGAGCGTGGC	Tb927.4.2400_rev	GGGGACCACTTTGTACAAGAAAGCTGGGTCTAACTACCAGACATGAGGCTGCC
hypothetical_las	F12	Tb927.7.2170_for	GGGGACAAGTTTGTACAAAAAAGCAGGCTATGTCTGGTTCCTTTGCTCAGC	Tb927.7.2170_rev	GGGGACCACTTTGTACAAGAAAGCTGGGTCTACTCATCCGCGACAGCCG
hypothetical_las	F13	Tb927.6.3560_for	GGGGACAAGTTTGTACAAAAAAGCAGGCTATGGATGATTCGGATCCCCCAC	Tb927.6.3560_rev	GGGGACCACTTTGTACAAGAAAGCTGGGTTCACCTTCTCTGGAGCTCTCTTTT
hypothetical_las	F14	Tb927.1.4480_for	GGGGACAAGTTTGTACAAAAAAGCAGGCTATGGAACATCCCCATTTGCAGG	Tb927.1.4480_rev	GGGGACCACTTTGTACAAGAAAGCTGGGTCTACAAGCCAACGTTGTCATGACT
hypothetical_las	F15	Tb927.8.3700_for	GGGGACAAGTTTGTACAAAAAAGCAGGCTATGAGCGAGTCAGTGGGTACG	Tb927.8.3700_rev	GGGGACCACTTTGTACAAGAAAGCTGGGTTCAGCGAGGTGACATTTTTTCCTT
hypothetical_las	F16	Tb927.10.12060_for	GGGGACAAGTTTGTACAAAAAAGCAGGCTATGCGGGGGAGCTGCAGC	Tb927.10.12060_rev	GGGGACCACTTTGTACAAGAAAGCTGGGTCTACCTACAAAGAACTGAAGCCACG
hypothetical_las	F17	Tb927.8.2220_for	GGGGACAAGTTTGTACAAAAAAGCAGGCTATGGATACAACCAATGGCGGTG	Tb927.8.2220_rev	GGGGACCACTTTGTACAAGAAAGCTGGGTTCAAGCACACTGAACCTTAGTTGC
known_last	A1	Tb927.9.12650_for	GGGGACAAGTTTGTACAAAAAAGCAGGCTATGAGTAAGATGGCTGGTGCTACG	Tb927.9.12650_rev	GGGGACCACTTTGTACAAGAAAGCTGGGTTTATTGTTGGATGCGCAAAAGTATTGT
known_last	A2	Tb927.7.6080_for	GGGGACAAGTTTGTACAAAAAAGCAGGCTATGCCTCACAGAGCAGTTTGG	Tb927.7.6080_rev	GGGGACCACTTTGTACAAGAAAGCTGGGTTCATCCGCTGTTCTTGCGC
known_last	A3	Tb927.1.1890_for	GGGGACAAGTTTGTACAAAAAAGCAGGCTATGCCGTCGCCCAAACTC	Tb927.1.1890_rev	GGGGACCACTTTGTACAAGAAAGCTGGGTCTAATCTATATGTAAAACAACAGTAGACTG
known_last	A4	Tb927.10.10340_for	GGGGACAAGTTTGTACAAAAAAGCAGGCTATGCGCGTGAAGAGCATTGT	Tb927.10.10340_rev	GGGGACCACTTTGTACAAGAAAGCTGGGTCTAACGACTTCTATTTGACTGAATACGAGT
known_last	A5	Tb927.10.9700_for	GGGGACAAGTTTGTACAAAAAAGCAGGCTATGAAGGACAGTTATCGCGATCCC	Tb927.10.9700_rev	GGGGACCACTTTGTACAAGAAAGCTGGGTCTACTGTCCATGATCGCCGTTC
known_last	A6	Tb927.11.16100_for	GGGGACAAGTTTGTACAAAAAAGCAGGCTATGCGGTCGGGGGGGTTC	Tb927.11.16100_rev	GGGGACCACTTTGTACAAGAAAGCTGGGTTCAGCCCTCAACTAACTGCTCG
known_last	A7	Tb927.4.3810_for	GGGGACAAGTTTGTACAAAAAAGCAGGCTATGATGGACGACGAACTACTTCTACA	Tb927.4.3810_rev	GGGGACCACTTTGTACAAGAAAGCTGGGTTTACAACGGCCCTGTGCCA
known_last	A8	Tb927.4.450_for	GGGGACAAGTTTGTACAAAAAAGCAGGCTATGCTGACGAGATTTGATGTTCGC	Tb927.4.450_rev	GGGGACCACTTTGTACAAGAAAGCTGGGTTCACGTACGATTTGCAAGTCCAG
known_last	A9	Tb927.11.10190_for	GGGGACAAGTTTGTACAAAAAAGCAGGCTATGGCACAAATGTTCCCCTCTG	Tb927.11.10190_rev	GGGGACCACTTTGTACAAGAAAGCTGGGTTCATGCAGAGCGCGTCGC
known_last	A10	Tb927.11.10280_for	GGGGACAAGTTTGTACAAAAAAGCAGGCTATGACCTCTCGCATTGAGAAGAGT	Tb927.11.10280_rev	GGGGACCACTTTGTACAAGAAAGCTGGGTTTATTGCTGCAGTTGTTGCTGTTG
known_last	A11	Tb927.11.14070_for	GGGGACAAGTTTGTACAAAAAAGCAGGCTATGACGAAAGAGGATCACGGTGA	Tb927.11.14070_rev	GGGGACCACTTTGTACAAGAAAGCTGGGTTCACAACAAAAATGCATATGAAAGTAAACT
known_last	A12	Tb927.6.3550_for	GGGGACAAGTTTGTACAAAAAAGCAGGCTATGCGAATCGTAGCTCCGTTG	Tb927.6.3550_rev	GGGGACCACTTTGTACAAGAAAGCTGGGTCTACACAGAGTTTGCGTTCCTTATCG
known_last	A13	Tb927.5.3510_for	GGGGACAAGTTTGTACAAAAAAGCAGGCTATGTATATCAAAAATATTCTTATTTCCGGA	Tb927.5.3510_rev	GGGGACCACTTTGTACAAGAAAGCTGGGTTCATTCAACATGCTCACGGACTC
known_last	A14	Tb927.11.10670_for	GGGGACAAGTTTGTACAAAAAAGCAGGCTATGTCGTCCCGCGATTTGAG	Tb927.11.10670_rev	GGGGACCACTTTGTACAAGAAAGCTGGGTTCATTCCAACTGGTCCGGTGG
known_last	A15	Tb927.7.2300_for	GGGGACAAGTTTGTACAAAAAAGCAGGCTATGTTCGCTTTCCTTCCTCCTGC	Tb927.7.2300_rev	GGGGACCACTTTGTACAAGAAAGCTGGGTTCATGACACGAGTCCAGCGG
known_last	A16	Tb927.8.4870_for	GGGGACAAGTTTGTACAAAAAAGCAGGCTATGCCCGCTCCCCATAATTCG	Tb927.8.4870_rev	GGGGACCACTTTGTACAAGAAAGCTGGGTTTACTCCTCCTTCTTCGTGGAGAG
known_last	A17	Tb927.7.7470_for	GGGGACAAGTTTGTACAAAAAAGCAGGCTATGAAAGCACCAGCCTTGCTG	Tb927.7.7470_rev	GGGGACCACTTTGTACAAGAAAGCTGGGTCTAAAAATCACCCAGCTCCCCCA
known_last	A18	Tb927.5.330_for	GGGGACAAGTTTGTACAAAAAAGCAGGCTATGACCTCACTGCTGTCGAAGC	Tb927.5.330_rev	GGGGACCACTTTGTACAAGAAAGCTGGGTTCAGGAAGCATAACGACCAACAAA
known_last	A19	Tb927.10.13040_for	GGGGACAAGTTTGTACAAAAAAGCAGGCTATGAAAGCACCAGCCTTGCTG	Tb927.10.13040_rev	GGGGACCACTTTGTACAAGAAAGCTGGGTTTAAAACTTATCAAAATCCGTGGTCCGA
known_last	A20	Tb927.11.10730_for	GGGGACAAGTTTGTACAAAAAAGCAGGCTATGTCAAGTTCGCGAAAACGC	Tb927.11.10730_rev	GGGGACCACTTTGTACAAGAAAGCTGGGTTTACGAGTTAAATGTTTCCGTAAATAGAGG
known_last	A21	Tb927.6.190_for	GGGGACAAGTTTGTACAAAAAAGCAGGCTATGTGTGTCCTGCAGTGGCG	Tb927.6.190_rev	GGGGACCACTTTGTACAAGAAAGCTGGGTTTATGCCTCATGTTCCGCAAAGT
known_last	A22	Tb927.11.13740_for	GGGGACAAGTTTGTACAAAAAAGCAGGCTATGACCACCACGAAGGCTTCG	Tb927.11.13740_rev	GGGGACCACTTTGTACAAGAAAGCTGGGTTCACCGCTGCGCTTCGGG
known_last	A23	Tb927.1.750_for	GGGGACAAGTTTGTACAAAAAAGCAGGCTATGGCGATGCGCAGCAAT	Tb927.1.750_rev	GGGGACCACTTTGTACAAGAAAGCTGGGTCTACCGTATCATTGGTAAGAAGCGC
known_last	A24	Tb927.2.2240_for	GGGGACAAGTTTGTACAAAAAAGCAGGCTATGTCGCAACTGAGCTCCCC	Tb927.2.2240_rev	GGGGACCACTTTGTACAAGAAAGCTGGGTCTACGCTGCGTAATGTTGCCT
known_last	B1	Tb927.9.5590_for	GGGGACAAGTTTGTACAAAAAAGCAGGCTATGGCGGAGGCACACAAGT	Tb927.9.5590_rev	GGGGACCACTTTGTACAAGAAAGCTGGGTCTAGAACAGCAGACGCGGC
known_last	B2	Tb927.11.6170_for	GGGGACAAGTTTGTACAAAAAAGCAGGCTATGCGCTTAAAGGCCACACC	Tb927.11.6170_rev	GGGGACCACTTTGTACAAGAAAGCTGGGTCTACTGCAGAAACTTGAGGTTATTAATATG
known_last	B3	Tb927.3.3870_for	GGGGACAAGTTTGTACAAAAAAGCAGGCTATGGAGAGGAGACAGGCGGG	Tb927.3.3870_rev	GGGGACCACTTTGTACAAGAAAGCTGGGTTTACATGGCATCCGGCTCGC
known_last	B4	Tb927.10.3650_for	GGGGACAAGTTTGTACAAAAAAGCAGGCTATGCTCTCAACGAAGCAACTTCTC	Tb927.10.3650_rev	GGGGACCACTTTGTACAAGAAAGCTGGGTTCACAGCGTTCCCGACTCG
known_last	B5	Tb927.4.3860_for	GGGGACAAGTTTGTACAAAAAAGCAGGCTATGATTTACCACAGTGAAACTGATGGT	Tb927.4.3860_rev	GGGGACCACTTTGTACAAGAAAGCTGGGTTTAATTCCCGTGAAGCTCACGC
known_last	B7	Tb927.4.3880_for	GGGGACAAGTTTGTACAAAAAAGCAGGCTATGATTTACCACAGTGAAACTGATGGT	Tb927.4.3880_rev	GGGGACCACTTTGTACAAGAAAGCTGGGTTTAATTCCCGTGAAGTTCACGCTC
known_last	B8	Tb927.11.4160_for	GGGGACAAGTTTGTACAAAAAAGCAGGCTATGGCAACACTGACGGTGAC	Tb927.11.4160_rev	GGGGACCACTTTGTACAAGAAAGCTGGGTTCAGTATCCACAAATAAGGGGTTCTCC
known_last	B9	Tb927.5.320_for	GGGGACAAGTTTGTACAAAAAAGCAGGCTATGACCTCACTGCTGTCGAAGC	Tb927.5.320_rev	GGGGACCACTTTGTACAAGAAAGCTGGGTTCAATTAGCATCATTTTCCGTCATTCCG
known_last	B10	Tb927.11.7210_for	GGGGACAAGTTTGTACAAAAAAGCAGGCTATGCACGTAGACGAGGGAAAGT	Tb927.11.7210_rev	GGGGACCACTTTGTACAAGAAAGCTGGGTTTACTTCTTTCTCCGTACGTTGTCCC
known_last	B11	Tb927.4.3750_for	GGGGACAAGTTTGTACAAAAAAGCAGGCTATGTTTTCTTTCACGCCTCGGG	Tb927.4.3750_rev	GGGGACCACTTTGTACAAGAAAGCTGGGTCTATGTGGTACGCGCCTCCC
known_last	B12	Tb927.11.1480_for	GGGGACAAGTTTGTACAAAAAAGCAGGCTATGCCGGCAGCTTTGTGC	Tb927.11.1480_rev	GGGGACCACTTTGTACAAGAAAGCTGGGTTCAAAGCATGGAAGAACATGTATCGA
known_last	B13	Tb927.11.150_for	GGGGACAAGTTTGTACAAAAAAGCAGGCTATGTGCTACAGGGACGTCATTACA	Tb927.11.150_rev	GGGGACCACTTTGTACAAGAAAGCTGGGTCTAACCTTCATCAAGTAAAATTTTGTTATC
known_last	B14	Tb927.10.5380_for	GGGGACAAGTTTGTACAAAAAAGCAGGCTATGCTTAACACTGTGAAATGGGCT	Tb927.10.5380_rev	GGGGACCACTTTGTACAAGAAAGCTGGGTCTACTCCTTTTTCTTCTCCATCGAGGC
known_last	B15	Tb927.10.2430_for	GGGGACAAGTTTGTACAAAAAAGCAGGCTATGCACGAATCGACAGTGGG	Tb927.10.2430_rev	GGGGACCACTTTGTACAAGAAAGCTGGGTTTAAGAGGAAGAACCGCGTGGC
known_last	B16	Tb927.4.3160_for	GGGGACAAGTTTGTACAAAAAAGCAGGCTATGAAACAAGGTAACGGGGCCA	Tb927.4.3160_rev	GGGGACCACTTTGTACAAGAAAGCTGGGTCTACATCCTTGATGCCCGCTTG
known_last	B17	Tb927.5.3670_for	GGGGACAAGTTTGTACAAAAAAGCAGGCTATGCCACAGACTGTTAGCAGC	Tb927.5.3670_rev	GGGGACCACTTTGTACAAGAAAGCTGGGTTTATGTCTGAGTAGGTGGCACAGA
known_last	B18	Tb927.6.770_for	GGGGACAAGTTTGTACAAAAAAGCAGGCTATGTGCCGGCAGGAAGGT	Tb927.6.770_rev	GGGGACCACTTTGTACAAGAAAGCTGGGTTTAGTATTCCTGAAGGTGCATGTCGT
known_last	B19	Tb927.4.4430_for	GGGGACAAGTTTGTACAAAAAAGCAGGCTATGGTTACCTTCATGCGTGAATTGT	Tb927.4.4430_rev	GGGGACCACTTTGTACAAGAAAGCTGGGTTTAGCTGGCAGCGCTAACATTT

known_last	B20	Tb927.7.7520_for	GGGGACAAGTTTGTACAAAAAAGCAGGCTATGGTTATCTTCATGCGTGAATTGTATGT	Tb927.7.7520_rev	GGGGACCACTTTGTACAAGAAAGCTGGGTTTAGCTGGCGGCGCTAGC
known_last	B24	Tb927.5.4550_for	GGGGACAAGTTTGTACAAAAAAGCAGGCTATGATTTCCATTCATCAGTCGATTCTCTT	Tb927.5.4550_rev	GGGGACCACTTTGTACAAGAAAGCTGGGTCTAAGCTTCAGGGCTCTGGGT
known_last	C4	Tb927.4.4410_for	GGGGACAAGTTTGTACAAAAAAGCAGGCTATGGTTACCTTCATGCGTGAATTGT	Tb927.4.4410_rev	GGGGACCACTTTGTACAAGAAAGCTGGGTTTAGCTGGCAGCGCTAACATTATC
known_last	C5	Tb927.11.5940_for	GGGGACAAGTTTGTACAAAAAAGCAGGCTATGAACGATTACCGTTTGTGTGGT	Tb927.11.5940_rev	GGGGACCACTTTGTACAAGAAAGCTGGGTTCACACCGACACAAAGGGCT
known_last	C6	Tb927.6.430_for	GGGGACAAGTTTGTACAAAAAAGCAGGCTATGAGTTGGCAGGAAGGTGGT	Tb927.6.430_rev	GGGGACCACTTTGTACAAGAAAGCTGGGTTTAAATAGAATAATTTTCACGGCAGTACAG
known_last	C7	Tb927.6.800_for	GGGGACAAGTTTGTACAAAAAAGCAGGCTATGTGCCGGCAGGAAGGT	Tb927.6.800_rev	GGGGACCACTTTGTACAAGAAAGCTGGGTTTAAAAAGAACACATTTCACGGTAGCAAG
known_last	C9	Tb927.3.3860_for	GGGGACAAGTTTGTACAAAAAAGCAGGCTATGCCTATCCCAACGGTAACCA	Tb927.3.3860_rev	GGGGACCACTTTGTACAAGAAAGCTGGGTTTACACGTTCGCAGCTTGGTG
known_last	C10	Tb927.1.4010_for	GGGGACAAGTTTGTACAAAAAAGCAGGCTATGTTTCACCTTCCCAGGGCG	Tb927.1.4010_rev	GGGGACCACTTTGTACAAGAAAGCTGGGTCTAACAACCCGGTACAGAACGC
known_last	C11	Tb927.5.1450_for	GGGGACAAGTTTGTACAAAAAAGCAGGCTATGAACGACAGGACGAGTTTACTCG	Tb927.5.1450_rev	GGGGACCACTTTGTACAAGAAAGCTGGGTTCATAACGTTACCGTAAAGCCATCAG
known_last	C12	Tb927.8.7940_for	GGGGACAAGTTTGTACAAAAAAGCAGGCTATGGCTTCCTTCATGCGTCTTG	Tb927.8.7940_rev	GGGGACCACTTTGTACAAGAAAGCTGGGTCTAATGAGAACACCTTCCAGCATCAT
known_last	C13	Tb927.6.740_for	GGGGACAAGTTTGTACAAAAAAGCAGGCTATGAACCAGAAGCGATGCGAAG	Tb927.6.740_rev	GGGGACCACTTTGTACAAGAAAGCTGGGTTCACTCCTGCGCAGTGTCC
known_last	C14	Tb927.5.3320_for	GGGGACAAGTTTGTACAAAAAAGCAGGCTATGCGCAATGTTTCTTCCCTCG	Tb927.5.3320_rev	GGGGACCACTTTGTACAAGAAAGCTGGGTTCATGCATCAACGGCAACAACG
known_last	C15	Tb927.11.8150_for	GGGGACAAGTTTGTACAAAAAAGCAGGCTATGAACAATTATCATATTTACGACGAAATT	Tb927.11.8150_rev	GGGGACCACTTTGTACAAGAAAGCTGGGTCTACGGACGTAAAAGTTCTTTAATACCCC
known_last	C16	Tb927.9.7150_for	GGGGACAAGTTTGTACAAAAAAGCAGGCTATGTCAGTCGGCAAGGTCGC	Tb927.9.7150_rev	GGGGACCACTTTGTACAAGAAAGCTGGGTTTAAGCACCCAACCCTACCGG
known_last	C17	Tb927.6.200_for	GGGGACAAGTTTGTACAAAAAAGCAGGCTATGTGCTGGCAGGAAGGTGG	Tb927.6.200_rev	GGGGACCACTTTGTACAAGAAAGCTGGGTCTAATGTAACGACGGAAGCGGAC
known_last	C18	Tb927.8.7870_for	GGGGACAAGTTTGTACAAAAAAGCAGGCTATGGCTTCCTTCATGCGTCTTG	Tb927.8.7870_rev	GGGGACCACTTTGTACAAGAAAGCTGGGTTCAATGCTTAGGGGAATATTTGCTGG
known_last	C20	Tb927.1.1910_for	GGGGACAAGTTTGTACAAAAAAGCAGGCTATGTTACCATCCTCTCAGGACCAAC	Tb927.1.1910_rev	GGGGACCACTTTGTACAAGAAAGCTGGGTTCAAACATCTCCGGTATCAGATTGACG
known_last	C21	Tb927.9.4900_for	GGGGACAAGTTTGTACAAAAAAGCAGGCTATGTCCGATCTCCTTGAGTTCTTTGT	Tb927.9.4900_rev	GGGGACCACTTTGTACAAGAAAGCTGGGTTTACAAGCCGTCAGCCGTAGT
known_last	C22	Tb927.11.10520_for	GGGGACAAGTTTGTACAAAAAAGCAGGCTATGTTCAATGTCTCACCAGCGAG	Tb927.11.10520_rev	GGGGACCACTTTGTACAAGAAAGCTGGGTTCATTTCCGGTAGACGTTGCG
known_last	C23	Tb927.5.3030_for	GGGGACAAGTTTGTACAAAAAAGCAGGCTATGCATCTCTACTTAACCAAGCGAG	Tb927.5.3030_rev	GGGGACCACTTTGTACAAGAAAGCTGGGTCTAGTGACGAAGAGGTGTGTGACA
known_last	C24	Tb927.8.650_for	GGGGACAAGTTTGTACAAAAAAGCAGGCTATGATAACAAAAAATTCCATAATCAAAGAG	Tb927.8.650_rev	GGGGACCACTTTGTACAAGAAAGCTGGGTTCAATCATACTCCGTCAGAAGTTTCAG
known_last	D1	Tb927.3.2690_for	GGGGACAAGTTTGTACAAAAAAGCAGGCTATGAATTTCTATGAACGAGTAGCGAGTG	Tb927.3.2690_rev	GGGGACCACTTTGTACAAGAAAGCTGGGTTTACGTGTTCCCAACACCCTCC
known_last	D2	Tb927.8.7920_for	GGGGACAAGTTTGTACAAAAAAGCAGGCTATGGCTTCCTTCATGCGTCTTG	Tb927.8.7920_rev	GGGGACCACTTTGTACAAGAAAGCTGGGTTTATTCTGGCAATAATGGACACATATTACA
known_last	D3	Tb927.8.7900_for	GGGGACAAGTTTGTACAAAAAAGCAGGCTATGGCTTCCTTCATGCGTCTTG	Tb927.8.7900_rev	GGGGACCACTTTGTACAAGAAAGCTGGGTTTATTCTGGCAATAATGGACACATATTGCA
known_last	D4	Tb927.8.7930_for	GGGGACAAGTTTGTACAAAAAAGCAGGCTATGGCTTCCTTCATGCGTCTTG	Tb927.8.7930_rev	GGGGACCACTTTGTACAAGAAAGCTGGGTTTATTCTGGCAATAACGGACACATATTACA
known_last	D5	Tb927.8.7890_for	GGGGACAAGTTTGTACAAAAAAGCAGGCTATGGCTTCCTTCATGCGTCTTG	Tb927.8.7890_rev	GGGGACCACTTTGTACAAGAAAGCTGGGTTTATTCTGGCAATAACGGACGCA
known_last	D6	Tb927.7.6070_for	GGGGACAAGTTTGTACAAAAAAGCAGGCTATGCCTCACAGAGCAGTTTGG	Tb927.7.6070_rev	GGGGACCACTTTGTACAAGAAAGCTGGGTCTAGAGGTCACCCTTCAGCACC
known_last	D7	Tb927.8.7590_for	GGGGACAAGTTTGTACAAAAAAGCAGGCTATGGGCTTTAGGGCTGGCT	Tb927.8.7590_rev	GGGGACCACTTTGTACAAGAAAGCTGGGTTTAAAATGGGCCCTTGCATTGGT
known_last	D9	Tb927.6.330_for	GGGGACAAGTTTGTACAAAAAAGCAGGCTATGAGTTGGCAGGAAGGTGGT	Tb927.6.330_rev	GGGGACCACTTTGTACAAGAAAGCTGGGTTTAACAGGTACTACCAACACTTTCCAGG
known_last	D10	Tb927.6.280_for	GGGGACAAGTTTGTACAAAAAAGCAGGCTATGTGCTGGCAGGAAGGTGG	Tb927.6.280_rev	GGGGACCACTTTGTACAAGAAAGCTGGGTTTAACAGGTACTACCAACACTTTCCAGG
known_last	D12	Tb927.7.6050_for	GGGGACAAGTTTGTACAAAAAAGCAGGCTATGCCTCACAGAGCAGTTTGG	Tb927.7.6050_rev	GGGGACCACTTTGTACAAGAAAGCTGGGTCTAGAGGTCACCCTTCAGCGA
known_last	D14	Tb927.6.5110_for	GGGGACAAGTTTGTACAAAAAAGCAGGCTATGCCGTTTTTGGCGTCGA	Tb927.6.5110_rev	GGGGACCACTTTGTACAAGAAAGCTGGGTCTAGTGAATTCTTTGGATGGTTTGTATAAA
known_last	D15	Tb927.7.6060_for	GGGGACAAGTTTGTACAAAAAAGCAGGCTATGCCTCACAGAGCAGTTTGG	Tb927.7.6060_rev	GGGGACCACTTTGTACAAGAAAGCTGGGTCTAGAGGTCACCCTTCATCGACG
known_last	D16	Tb927.11.6350_for	GGGGACAAGTTTGTACAAAAAAGCAGGCTATGGTTGTGACCCGCTCCC	Tb927.11.6350_rev	GGGGACCACTTTGTACAAGAAAGCTGGGTTCATCCGTTTGAGCTTACCACCT
known_last	D17	Tb927.4.4470_for	GGGGACAAGTTTGTACAAAAAAGCAGGCTATGCTTAAATTACTTCTACTGCCATTACTC	Tb927.4.4470_rev	GGGGACCACTTTGTACAAGAAAGCTGGGTTCAACTATTTTTTCCATTGTCCCCGA
known_last	D18	Tb927.6.310_for	GGGGACAAGTTTGTACAAAAAAGCAGGCTATGAGCTGGCAGGAAGGTGG	Tb927.6.310_rev	GGGGACCACTTTGTACAAGAAAGCTGGGTTCATGAAGGATATTGGCTGGCAC
known_last	D19	Tb927.10.3390_for	GGGGACAAGTTTGTACAAAAAAGCAGGCTATGAGCGGCCGCGGAGGA	Tb927.10.3390_rev	GGGGACCACTTTGTACAAGAAAGCTGGGTCTACTCTCCAACGACAATCACCTCA
known_last	D20	Tb927.6.300_for	GGGGACAAGTTTGTACAAAAAAGCAGGCTATGTGCTGGCAGGAAGGTGG	Tb927.6.300_rev	GGGGACCACTTTGTACAAGAAAGCTGGGTTCATGAAGGATATTGGCTGGCAC
known_last	D21	Tb927.11.5780_for	GGGGACAAGTTTGTACAAAAAAGCAGGCTATGCGGCGGTTGAGTCTGG	Tb927.11.5780_rev	GGGGACCACTTTGTACAAGAAAGCTGGGTCTAAGAGAAAAAATATGGGGAGTTTAGGAC
known_last	D22	Tb927.9.11850_for	GGGGACAAGTTTGTACAAAAAAGCAGGCTATGTCGTCGCGGATAGAGCG	Tb927.9.11850_rev	GGGGACCACTTTGTACAAGAAAGCTGGGTCTAATAACCATTTATGTGTTTATTGCCACC
known_last	D23	Tb927.10.8590_for	GGGGACAAGTTTGTACAAAAAAGCAGGCTATGCAAAGTGGTGTGGGTAACATT	Tb927.10.8590_rev	GGGGACCACTTTGTACAAGAAAGCTGGGTTCACGGAACTGGTGCCGG
known_last	D24	Tb927.5.650_for	GGGGACAAGTTTGTACAAAAAAGCAGGCTATGTTTCTCCTTTCCCGCACTG	Tb927.5.650_rev	GGGGACCACTTTGTACAAGAAAGCTGGGTTCACTCCCCATTCCTTGACGT
known_last	E1	Tb927.11.13360_for	GGGGACAAGTTTGTACAAAAAAGCAGGCTATGCCGAAACGAAGCCGTC	Tb927.11.13360_rev	GGGGACCACTTTGTACAAGAAAGCTGGGTTTAGGCGAACATTGAGTTGTGCG
known_last	E2	Tb927.4.2460_for	GGGGACAAGTTTGTACAAAAAAGCAGGCTATGGGTGCTTGCATAGCCG	Tb927.4.2460_rev	GGGGACCACTTTGTACAAGAAAGCTGGGTCTACACCCTTACATTGGGACTTTCTC
known_last	E3	Tb927.4.770_for	GGGGACAAGTTTGTACAAAAAAGCAGGCTATGTTTCTATCCCTTGATGTCACGC	Tb927.4.770_rev	GGGGACCACTTTGTACAAGAAAGCTGGGTTTACGACAAACTGAAGTTCCTTCTGC
known_last	E4	Tb927.4.4460_for	GGGGACAAGTTTGTACAAAAAAGCAGGCTATGGTTACCTTCATGCGTGAATTGT	Tb927.4.4460_rev	GGGGACCACTTTGTACAAGAAAGCTGGGTTCAACTATTTTTTCCATTGTCCCCGA
known_last	E5	Tb927.10.14410_for	GGGGACAAGTTTGTACAAAAAAGCAGGCTATGAACGTTGATTTATCGAACAAGTCTATC	Tb927.10.14410_rev	GGGGACCACTTTGTACAAGAAAGCTGGGTTTAAATGAAGACTGGGCGCTGAG
known_last	E6	Tb927.11.12110_for	GGGGACAAGTTTGTACAAAAAAGCAGGCTATGGGCCCCAACAGACAAAGG	Tb927.11.12110_rev	GGGGACCACTTTGTACAAGAAAGCTGGGTTCAACGCATGGGGGTCGT
known_last	E7	Tb927.9.4080_for	GGGGACAAGTTTGTACAAAAAAGCAGGCTATGGCATCGGAGGCAGCG	Tb927.9.4080_rev	GGGGACCACTTTGTACAAGAAAGCTGGGTTTATTCGCCCCTAACTGCCTCC
known_last	E8	Tb927.10.8170_for	GGGGACAAGTTTGTACAAAAAAGCAGGCTATGGAAGTACGAGATGATAACCCGA	Tb927.10.8170_rev	GGGGACCACTTTGTACAAGAAAGCTGGGTCTAGAAAGTAAACCTGGAACTGGCAC
known_last	E9	Tb927.9.13610_for	GGGGACAAGTTTGTACAAAAAAGCAGGCTATGCGGAAAGACGAATACCGG	Tb927.9.13610_rev	GGGGACCACTTTGTACAAGAAAGCTGGGTCTATTTGTAGCTCCTTGGGTGCTG
known_last	E10	Tb927.5.2410_for	GGGGACAAGTTTGTACAAAAAAGCAGGCTATGAGCAAAGCTGCGGTTTCA	Tb927.5.2410_rev	GGGGACCACTTTGTACAAGAAAGCTGGGTTTAAGTTCTAACAAGACTTTCCTGGCTAAG
known_last	E11	Tb927.8.4880_for	GGGGACAAGTTTGTACAAAAAAGCAGGCTATGGAGGATTGGGTTAGCTGCC	Tb927.8.4880_rev	GGGGACCACTTTGTACAAGAAAGCTGGGTTCATGGAGTATCGAACAACTTGTCAAG
known_last	E12	Tb927.9.5520_for	GGGGACAAGTTTGTACAAAAAAGCAGGCTATGAATCAGAACAATTTATCTTGCGAGGA	Tb927.9.5520_rev	GGGGACCACTTTGTACAAGAAAGCTGGGTTCACCTTCTTGAAATACGTAACGCC
known_last	E13	Tb927.7.3000_for	GGGGACAAGTTTGTACAAAAAAGCAGGCTATGACGTCTCAAACGTCGGC	Tb927.7.3000_rev	GGGGACCACTTTGTACAAGAAAGCTGGGTTCATCCCTCCCCGTCGATACA
known_last	E14	Tb927.8.6200_for	GGGGACAAGTTTGTACAAAAAAGCAGGCTATGGATGGTGAACAATTAGAAACAGATCC	Tb927.8.6200_rev	GGGGACCACTTTGTACAAGAAAGCTGGGTTCAGTACCCTGCCTCATGTACG
known_last	E15	Tb927.9.12400_for	GGGGACAAGTTTGTACAAAAAAGCAGGCTATGTGTGCCTTAAGGGATATCGCG	Tb927.9.12400_rev	GGGGACCACTTTGTACAAGAAAGCTGGGTTTAGTTTACCATAAAGTCATGGAACAAAAG
known_last	E16	Tb927.10.13330_for	GGGGACAAGTTTGTACAAAAAAGCAGGCTATGGATCAATCATGCAACGAAGCT	Tb927.10.13330_rev	GGGGACCACTTTGTACAAGAAAGCTGGGTCTATCTGCAACCGTCACTGCG
known_last	E17	Tb927.11.8210_for	GGGGACAAGTTTGTACAAAAAAGCAGGCTATGACGAGTATCGAACAGATTGAAATCA	Tb927.11.8210_rev	GGGGACCACTTTGTACAAGAAAGCTGGGTTCAACTCGCGAACAACTGATCG
known_last	E18	Tb927.11.260_for	GGGGACAAGTTTGTACAAAAAAGCAGGCTATGAGCATTCAAAAAAAGTTTGAGCTTACG	Tb927.11.260_rev	GGGGACCACTTTGTACAAGAAAGCTGGGTCTAAAGAAGATGAGTCTTCAAATCAGGATC
known_last	E19	Tb927.9.7720_for	GGGGACAAGTTTGTACAAAAAAGCAGGCTATGCCCTCCTTAATACGTACCAACT	Tb927.9.7720_rev	GGGGACCACTTTGTACAAGAAAGCTGGGTTTAGGGTCGCAGCAAACTGTG
known_last	E20	Tb927.11.14630_for	GGGGACAAGTTTGTACAAAAAAGCAGGCTATGCCAATCTTGTGCGATTGCC	Tb927.11.14630_rev	GGGGACCACTTTGTACAAGAAAGCTGGGTTTACCACCATCCTCTGTGTACCG
known_last	E21	Tb927.10.740_for	GGGGACAAGTTTGTACAAAAAAGCAGGCTATGAGCTGCGAAAGCAACGG	Tb927.10.740_rev	GGGGACCACTTTGTACAAGAAAGCTGGGTTTAGGCATCTTCCTCTACCTTCAAACG
known_last	E22	Tb927.11.9190_for	GGGGACAAGTTTGTACAAAAAAGCAGGCTATGGGAAATCAGCTCGCAGC	Tb927.11.9190_rev	GGGGACCACTTTGTACAAGAAAGCTGGGTTTACTCAGCGTTGAACCACAGTG
known_last	E23	Tb927.11.11880_for	GGGGACAAGTTTGTACAAAAAAGCAGGCTATGGGCACCGTGTCTCCG	Tb927.11.11880_rev	GGGGACCACTTTGTACAAGAAAGCTGGGTTTACTCGGCTGCGGCTACC
known_last	E24	Tb927.8.710_for	GGGGACAAGTTTGTACAAAAAAGCAGGCTATGATAACTCCAGAAACGGCAGGA	Tb927.8.710_rev	GGGGACCACTTTGTACAAGAAAGCTGGGTTTAACGACCATCGTCCATCAACAC
known_last	F1	Tb927.3.3850_for	GGGGACAAGTTTGTACAAAAAAGCAGGCTATGAATACAAACCGGCCGAACAC	Tb927.3.3850_rev	GGGGACCACTTTGTACAAGAAAGCTGGGTTCAGGAGGAGTTAAATGTGTCCACA
known_last	F2	Tb927.3.4230_for	GGGGACAAGTTTGTACAAAAAAGCAGGCTATGTCGTCCAGTCAAAACCATCTG	Tb927.3.4230_rev	GGGGACCACTTTGTACAAGAAAGCTGGGTTCATGGTAGTCTTTCCATCTCAATATATTC
known_last	F3	Tb927.1.370_for	GGGGACAAGTTTGTACAAAAAAGCAGGCTATGTCAGGAAACCAAGGAAGAAAGC	Tb927.1.370_rev	GGGGACCACTTTGTACAAGAAAGCTGGGTTTAACGGATATGCACCCTCTTTTTTCT
known_last	F4	Tb927.1.290_for	GGGGACAAGTTTGTACAAAAAAGCAGGCTATGTCAGGAAACCAAGGAAGAAAGC	Tb927.1.290_rev	GGGGACCACTTTGTACAAGAAAGCTGGGTTTAACGGATATGTACTCTCCTTTTTCTCAG
known_last	F5	Tb927.2.170_for	GGGGACAAGTTTGTACAAAAAAGCAGGCTATGTCAGGAAACCAAGGAAGAAAGC	Tb927.2.170_rev	GGGGACCACTTTGTACAAGAAAGCTGGGTTTAGGTATGTACCCTCCTTTTTCTCAGT
known_last	F6	Tb927.1.480_for	GGGGACAAGTTTGTACAAAAAAGCAGGCTATGTCAGGAAACCAAGGAAGAAAGC	Tb927.1.480_rev	GGGGACCACTTTGTACAAGAAAGCTGGGTTTAACGGATATGTACCCTTCTTTTTCTCAG
known_last	F7	Tb927.2.950_for	GGGGACAAGTTTGTACAAAAAAGCAGGCTATGTCAGGAAACCAAGGAAGAAAGC	Tb927.2.950_rev	GGGGACCACTTTGTACAAGAAAGCTGGGTTTAATATATATGTACCCTCCTTTTTCCCAG
known_last	F8	Tb927.9.4370_for	GGGGACAAGTTTGTACAAAAAAGCAGGCTATGCTGGTCGCCACAGCG	Tb927.9.4370_rev	GGGGACCACTTTGTACAAGAAAGCTGGGTTCAAGTAGGGACCATTTCCACAATG
known_last	F9	Tb927.11.4690_for	GGGGACAAGTTTGTACAAAAAAGCAGGCTATGCGGCTAAATAGCTGCTGG	Tb927.11.4690_rev	GGGGACCACTTTGTACAAGAAAGCTGGGTTTACACCGTAATTTCTACACTGTCAGTAAC
known_last	F10	Tb927.8.6070_for	GGGGACAAGTTTGTACAAAAAAGCAGGCTATGGCACAACTTGACCAGTTGC	Tb927.8.6070_rev	GGGGACCACTTTGTACAAGAAAGCTGGGTTCAATCGACACCTCCAAACATGAAC
known_last	F11	Tb927.11.15750_for	GGGGACAAGTTTGTACAAAAAAGCAGGCTATGTCGGATCCAACGCCCC	Tb927.11.15750_rev	GGGGACCACTTTGTACAAGAAAGCTGGGTTTAAGAATCCCTGCTCTCGTCGT
known_last	F12	Tb927.9.9740_for	GGGGACAAGTTTGTACAAAAAAGCAGGCTATGAGTGTGTCTCTGGCTCCC	Tb927.9.9740_rev	GGGGACCACTTTGTACAAGAAAGCTGGGTCTATCGGTGCAGAGGAGGTAACG
known_last	F13	Tb927.7.4900_for	GGGGACAAGTTTGTACAAAAAAGCAGGCTATGGGTGTTCCAAAATTCTTTCGATGG	Tb927.7.4900_rev	GGGGACCACTTTGTACAAGAAAGCTGGGTTCATCGTTTCCTGTCTCCTGATAAATG
known_last	F14	Tb927.8.5620_for	GGGGACAAGTTTGTACAAAAAAGCAGGCTATGCTTGATCTAGATAGCTGGGTGG	Tb927.8.5620_rev	GGGGACCACTTTGTACAAGAAAGCTGGGTCTACAGTGCCGCTTCACACAT
known_last	F15	Tb927.11.11540_for	GGGGACAAGTTTGTACAAAAAAGCAGGCTATGTCGGGTCGCACCGTT	Tb927.11.11540_rev	GGGGACCACTTTGTACAAGAAAGCTGGGTTCAAAATACGCAGGCATCAAACCC
known_last	F16	Tb927.5.3160_for	GGGGACAAGTTTGTACAAAAAAGCAGGCTATGACCACAACGCCAGGAGG	Tb927.5.3160_rev	GGGGACCACTTTGTACAAGAAAGCTGGGTTCAAACGTTTTTGGAAACTGTCAGTG
known_last	F17	Tb927.8.1130_for	GGGGACAAGTTTGTACAAAAAAGCAGGCTATGAGCCATGAATTCAGTGGCTC	Tb927.8.1130_rev	GGGGACCACTTTGTACAAGAAAGCTGGGTTTAACCAGAATGGTCACCGTCACC
known_last	F18	Tb927.11.11080_for	GGGGACAAGTTTGTACAAAAAAGCAGGCTATGTCTGAAATAGCTCACTCTGCG	Tb927.11.11080_rev	GGGGACCACTTTGTACAAGAAAGCTGGGTTCAATAGGGTTTAGTGAAAGCATTCTCC
known_last	F19	Tb927.4.3020_for	GGGGACAAGTTTGTACAAAAAAGCAGGCTATGTTCACACGCGCTCTCAG	Tb927.4.3020_rev	GGGGACCACTTTGTACAAGAAAGCTGGGTTCACACCTCGTGTAACTCCCAT
known_last	F20	Tb927.1.1880_for	GGGGACAAGTTTGTACAAAAAAGCAGGCTATGAATGAATCCCACATGTGTCATCA	Tb927.1.1880_rev	GGGGACCACTTTGTACAAGAAAGCTGGGTTCACTCTTTCATCATAATAGGCACGC
known_last	F21	Tb927.11.1460_for	GGGGACAAGTTTGTACAAAAAAGCAGGCTATGCGCAGTGCGCTTCCA	Tb927.11.1460_rev	GGGGACCACTTTGTACAAGAAAGCTGGGTTTAAGCCAAACTGCATGAATCCCC
known_last	F22	Tb927.11.14560_for	GGGGACAAGTTTGTACAAAAAAGCAGGCTATGCTGCATGCAACTGTGGC	Tb927.11.14560_rev	GGGGACCACTTTGTACAAGAAAGCTGGGTTCAAAACAAACCACATTCTTCATGCAC
known_last	F23	Tb927.11.11550_for	GGGGACAAGTTTGTACAAAAAAGCAGGCTATGTCGGGTCGCACCGTT	Tb927.11.11550_rev	GGGGACCACTTTGTACAAGAAAGCTGGGTTTAGTCACTGAAGTCAAGAGAGGACTC
known_last	F24	Tb927.8.4950_for	GGGGACAAGTTTGTACAAAAAAGCAGGCTATGGAGTACAAGCGATGCAACTC	Tb927.8.4950_rev	GGGGACCACTTTGTACAAGAAAGCTGGGTCTAGCAGGTCAGAGACTCCTCAG
known_last	G1	Tb927.11.14210_for	GGGGACAAGTTTGTACAAAAAAGCAGGCTATGTCACAAAGATCGAGGAAAGTTCC	Tb927.11.14210_rev	GGGGACCACTTTGTACAAGAAAGCTGGGTTTAGCTCGACATCTTCGAGCGT
known_last	G2	Tb927.11.11090_for	GGGGACAAGTTTGTACAAAAAAGCAGGCTATGTCCCTGGACCCCCGT	Tb927.11.11090_rev	GGGGACCACTTTGTACAAGAAAGCTGGGTCTACTGCGAAAAGACACTTTTAAAATCAGG
known_last	G3	Tb927.11.7290_for	GGGGACAAGTTTGTACAAAAAAGCAGGCTATGATCTCCTCCAGTCAAGGGGG	Tb927.11.7290_rev	GGGGACCACTTTGTACAAGAAAGCTGGGTTCAGGACACATGTTCCTCTGTGT
known_last	G4	Tb927.11.3780_for	GGGGACAAGTTTGTACAAAAAAGCAGGCTATGACATGCATACAGGAAAACATTACAGG	Tb927.11.3780_rev	GGGGACCACTTTGTACAAGAAAGCTGGGTCTACTCAGATGGTTCCACTGCCA
known_last	G5	Tb927.3.5440_for	GGGGACAAGTTTGTACAAAAAAGCAGGCTATGAAAGGAAATGACAACGACTGTAACG	Tb927.3.5440_rev	GGGGACCACTTTGTACAAGAAAGCTGGGTCTACTTGATGACGCGCACCG
known_last	G6	Tb927.11.5190_for	GGGGACAAGTTTGTACAAAAAAGCAGGCTATGGTGTATGAGAGTGAATCTTGCAG	Tb927.11.5190_rev	GGGGACCACTTTGTACAAGAAAGCTGGGTTTAACGGGCACCGTGTAGCC
