# Supplementary figures and images for: A Trypanosoma brucei ORFeome-Based Gain-of-Function Library Identifies Genes That Promote Survival during Melarsoprol Treatment
Source: mSphere. 2020 Oct 7;5(5):e00769-20. doi: 10.1128/mSphere.00769-20 (PMC7568655; doi:10.1128/mSphere.00769-20)

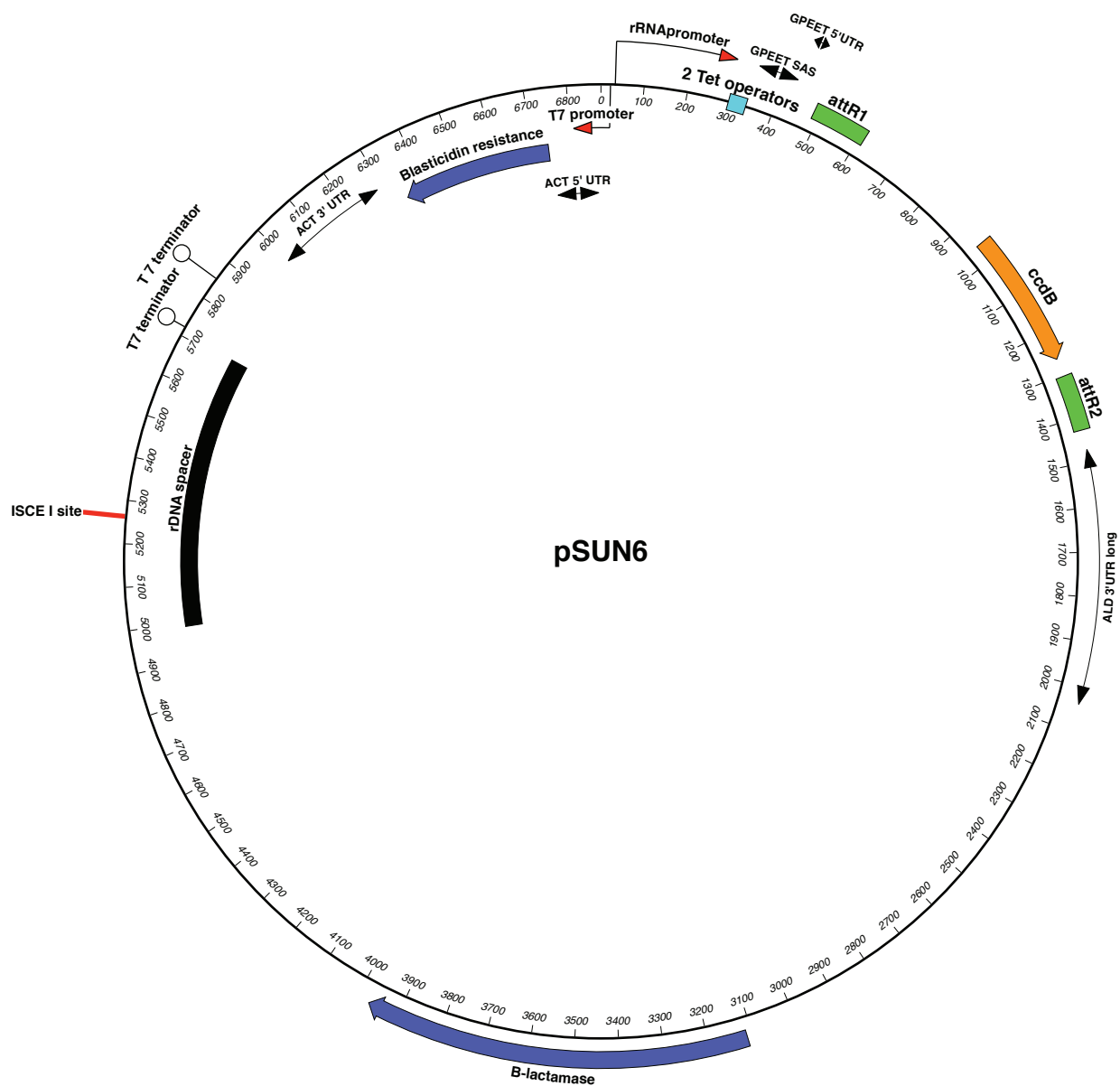

Supplement: FIG S1 [file mSphere.00769-20-sf001.pdf]

A)

## Library Prep

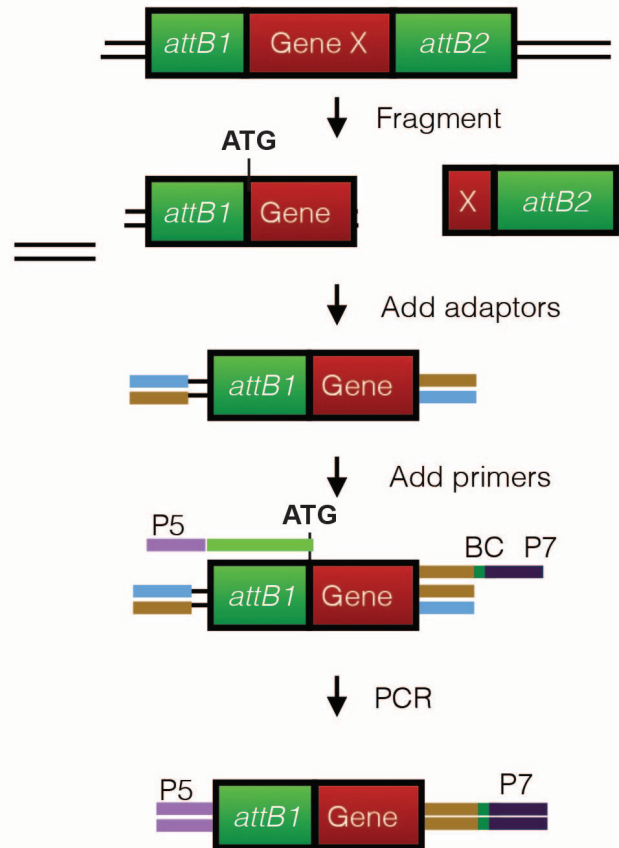

## Sequencing

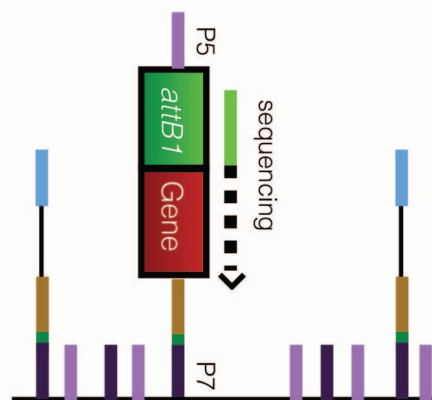

B)

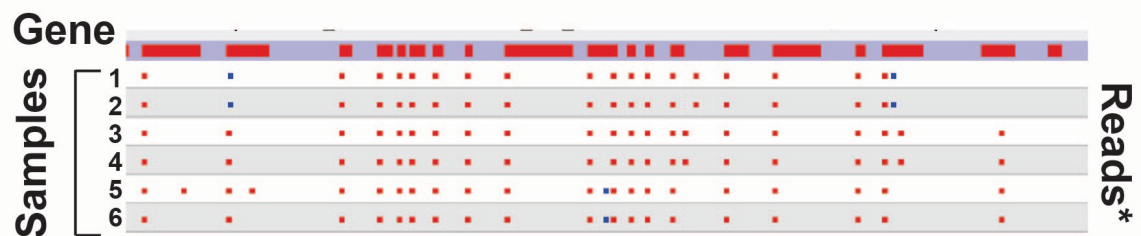

Supplement: FIG S3 [file mSphere.00769-20-sf003.pdf]
