# Supplementary material for: A Trypanosoma brucei ORFeome-Based Gain-of-Function Library Identifies Genes That Promote Survival during Melarsoprol Treatment
Source: mSphere. 2020 Oct 7;5(5):e00769-20. doi: 10.1128/mSphere.00769-20 (PMC7568655; doi:10.1128/mSphere.00769-20)
Supplement: TABLE S2 [file mSphere.00769-20-st002.pdf]

Plate name	Min length*	Max length*	Total	missing	#_MISS _PLATE	ORF# / MISS_ Plate
0_hypothetical	102	375	373	124		
0_known	147	591	315	51		
1_hypothetical	375	522	374	72		
1_known	522	666	365	64	1	311
2_hypothetical	594	849	372	111		
2_known	849	1056	363	77		
3_hypothetical	669	822	382	89	2	277
3_known	822	993	367	226		
4_hypothetical	993	1155	372	105	3	331
4_known	1056	1287	348	171		
5_hypothetical	1287	1524	365	104		
5_known	1158	1365	342	94	4	369
6_hypothetical	1524	1857	375	158		
6_known	1368	1635	362	173	5	331
7_hypothetical	1857	2337	378	106		
7_known	1635	1953	376	117		
8_hypothetical	1953	2508	381	147	6	370
9_hypothetical	2340	3504	381	195		
10_hypothetical	2508	3501	381	141	7	336
hypothetical_last	3504	4488	135	58		
known_last	3507	4497	138	67	8	125
NEG_PICKS_#1	102	1365	252	122		
NEG_PICKS_#2	1857	4497	177	79		
1_MISS	102	666	311			
2_MISS	594	822	277			
3_MISS	822	1155	331			
4_MISS	1056	1365	369			
5_MISS	1524	1635	331			
6_MISS	1857	2508	370			
7_MISS	2340	3501	336			
8_MISS	3504	4497	125			

21 Pools of original ORFs from oligo plates.
2 Pools of PCR NEG_PICKs_Redo PCR reactions

8 Pools of MISSING ORFs from original 21 PCR plates.
Were identified as "Missing" from first ORFeome assesments (pENTR & pDEST). Isolated from PCR plates to form new sets of ORFS for an additional 8 gateway clonings.

31 Total Pools for final pENTR followed by pDEST (pSUN6) transfections
