# Supplementary material for: A Trypanosoma brucei ORFeome-Based Gain-of-Function Library Identifies Genes That Promote Survival during Melarsoprol Treatment
Source: mSphere. 2020 Oct 7;5(5):e00769-20. doi: 10.1128/mSphere.00769-20 (PMC7568655; doi:10.1128/mSphere.00769-20)

Gain-of-Function Library (GoF_L2) - Distribution of Coverage for Targeted ORFs

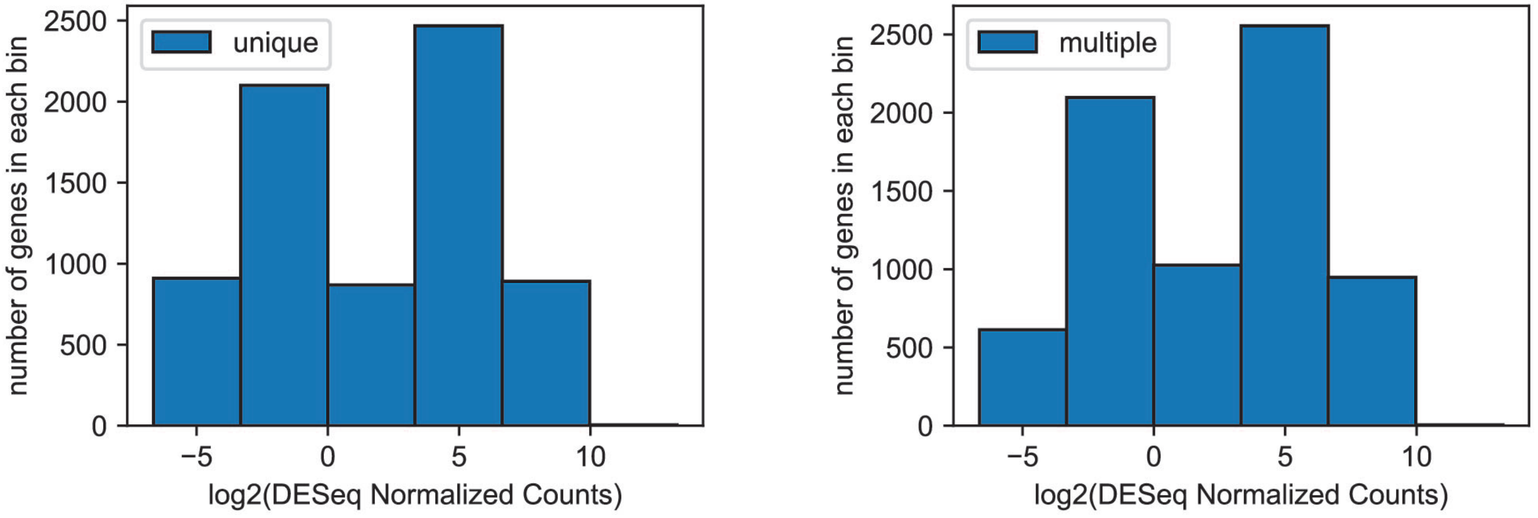

Gain-of-Function Library (GoF_L2) - ORF Coverage vs. ORF Length

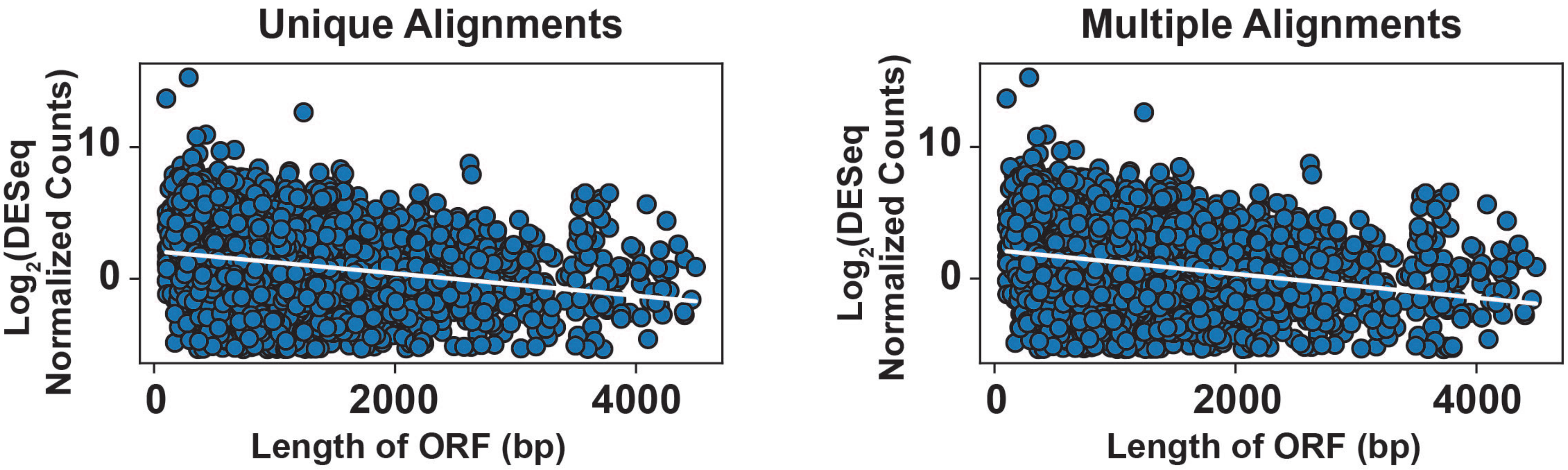

Supplement: FIG S4 [file mSphere.00769-20-sf004.pdf]
