# Supplementary material for: A Trypanosoma brucei ORFeome-Based Gain-of-Function Library Identifies Genes That Promote Survival during Melarsoprol Treatment
Source: mSphere. 2020 Oct 7;5(5):e00769-20. doi: 10.1128/mSphere.00769-20 (PMC7568655; doi:10.1128/mSphere.00769-20)
Supplement: TEXT S1 [file mSphere.00769-20-s0001.pdf]

Oligo design and PCR amplification of the ORFeome

In order to design oligos for the ORFeome, we used gene annotations generated from ribosome profiling data(1). We first filtered out genes that were either < 100 bp in size or > 4,500bp in size. We then filtered out any genes annotated as pseudogenes, hypothetical protein unlikely, VSG, ISG, ESAG, GRESAG, or ribosomal protein. Following this procedure, we used custom python scripts to design forward and reverse primers. If the first 30bp of the ORF had a Tm >55, this primer was used. Otherwise, a nucleotide was added one at a time until the primer had a Tm of greater than 55. A reverse primer was designed using the same procedure for the reverse complement of the last 30 nucleotides. Many genes in the *T. brucei* genome have identical beginnings and ends, and we did not wish to order duplicate sets of primers that were identical to each other. For this reason, only one primer pair was kept for duplicated or highly similar genes, and a record was made of any pair that was eliminated based on duplication. Finally, the *attB1* and *attB2* Gateway cloning sequences 5'-GGGGACAAGTTTGTACAAAAAAGCAGGCT and 5'-GGGGACCACTTTGTACAAGAAAGCTGGGT were added to the forward and reverse primers, respectively.

Each 384-well plate containing mixed forward and reverse primer pairs were diluted to 10 mM in primer dilution plates. PCR plates were filled with a master mix for KOD Hotstart (Novagen #71086) PCR reactions containing Lister427 gDNA according to manufacturer's specifications and primer pairs specific to each ORF were transferred into the associated well number for each plate by TECAN Freedom EVO 150 pipetting instrument. PCR reaction conditions followed manufacturers specifications for 25 µL reactions with annealing temperature of 55°C and elongation times ranging from 10-60 seconds depending on the anticipated lengths of products in each plate. Following PCR reaction completion SYBR Green I (Invitrogen S-7563) was added to each well at 1:10 PCR volume for a final concentration of 10x SYBR and relative fluorescence units (RFU) were measured on a BioTek Synergy H4 Hybrid Multi-Mode Microplate reader in comparison to a standard dilution of known DNA concentration. The resulting PCR reactions from all wells of each 384-well plate were then pooled prior to agarose gel separation and DNA extraction. PCR reactions called 'negative' based on SYBR (less than 10,000 RFU) were revisited by isolating primer pairs from original 384-well plates using a Perkin-Emer Janus Automated Workstation into two new PCR plates, which were then amplified using KOD Hotstart specifications and extracted from agarose gels as described. The resulting extracted DNAs from each PCR product pool were utilized in subsequent gateway cloning reactions.

Next-generation Illumina sequencing of the *T. brucei* ORFeome and Gain-of-Function libraries

The pENTR and pTrypLib ORF plasmid libraries were Illumina sequenced using tagmentation kits from Illumina (Nextera XT kit) according to the manufacturer's instructions. Sequencing was performed either on an Illumina HiSeq 2500 or an Illumina MiSeq. Reads were trimmed for quality using trim galore (http://www.bioinformatics.babraham.ac.uk/projects/trim_galore/) using this command: trim_galore --nextera --stringency 3. Reads were aligned to the Tb927v5.1 genome using bowtie requiring unique alignments(2): bowtie --best --strata -t -v 2 -a -m 1, or allowing multiple alignments: bowtie --best --strata -t -v 2 -a -m 10. RPKM values were calculated using SeqMonk from Babraham Bioinformatics (http://www.bioinformatics.babraham.ac.uk/projects/seqmonk).

To Illumina sequence the GoF *T. brucei* libraries, genomic DNA was isolated from 100-200 million transfected parasites and fragmented by sonication (Bioruptor). Sequencing libraries were prepared using the NEBNext Ultra II DNA library prep kit (NEB) according to the manufacturer's instructions with the following exception: a custom forward primer containing the *attB1* cloning site was used for 5' indexing and PCR enrichment (5'-AATGATACGGCGACCACCGAGATATATAACAAGTTTGTACAAAAAAGCAGGCTATG). A custom sequencing oligo containing attB1 site and ATG (5'-GGGACAAGTTTGTAC AAAAAAGCAGGCTATG) was loaded on to the Illumina sequencing platform to sequence only GoF library containing fragments. Sequencing reads were trimmed for quality and aligned to the genome using the same parameters described above. Raw counts were calculated for reads aligning exclusively to the first 100 bp of the ORF using SeqMonk from Babraham Bioinformatics (http://www.bioinformatics.babraham.ac.uk/projects/seqmonk). DESeq2 was used to calculate the normalized read counts between sequencing samples (3).

Bioinformatic analysis platforms for ORF identification and hit calling pipeline

In order to call which genes were overrepresented in the melarsoprol selected libraries, we aligned reads from each of the 12 samples (3 reps of each: GoF_L1, GoF_L2, MEL1, and MEL2) to the genome, and then calculated the number of reads that fell within the first 100bp of each of the ORFeome targeted ORFs. DESeq was then used to identify genes that were 'differentially expressed' in the melarsoprol survivor populations with a multiple testing corrected p value of less than 0.05(3). In this context, 'differential expression' simply refers to an ORF being overrepresented or

underrepresented in the melarsoprol selected samples. Using normalized read counts, we then calculated the fold change between each pair-wise comparison of melarsoprol selected and unselected replicates (3 total) for every statistically significant gene called by DESeq, provided that gene had a normalized read count of 5 or greater in the minimally propagated samples. If all 3 replicates showed a > 4-fold change for a particular gene, that gene was considered significantly overrepresented. Because we had two sets of replicates for melarsoprol untreated (GoF_L1 and GoF_L2) and treated (MEL1 and MEL2), we ran the pipeline described above for all 4 comparisons (GoF_L1 vs. MEL1, GoF_L1 vs. MEL2, GoF_L2 vs. MEL1, and GoF_L2 vs. MEL2). Only those genes that were called as hits in all 4 comparisons were reported in the final hit list. The entire analysis was performed for both uniquely aligned and multiply aligned reads and all comparisons are given in Data Set S3.

REFERENCES

1. Parsons M, Ramasamy G, Vasconcelos EJR, Jensen BC, Myler PJ. 2015. Advancing Trypanosoma brucei genome annotation through ribosome profiling and spliced leader mapping. Mol Biochem Parasit 202:1–10.

2. Langmead B, Trapnell C, Pop M, Salzberg SL. 2009. Ultrafast and memory-efficient alignment of short DNA sequences to the human genome. Genome Biol 10:R25.

3. Love MI, Huber W, Anders S. 2014. Moderated estimation of fold change and dispersion for RNA-seq data with DESeq2. Genome Biol 15:550.
